# *ElemNet*: Deep Learning the Chemistry of Materials From Only Elemental Composition

**DOI:** 10.1038/s41598-018-35934-y

**Published:** 2018-12-04

**Authors:** Dipendra Jha, Logan Ward, Arindam Paul, Wei-keng Liao, Alok Choudhary, Chris Wolverton, Ankit Agrawal

**Affiliations:** 1 Department of Electrical Engineering and Computer Science, Northwestern University, Evanston, USA; 2 Computation Institute, University of Chicago, Chicago, USA; 3 Department of Materials Science and Engineering, Northwestern University, Evanston, USA

## Abstract

Conventional machine learning approaches for predicting material properties from elemental compositions have emphasized the importance of leveraging domain knowledge when designing model inputs. Here, we demonstrate that by using a deep learning approach, we can bypass such manual feature engineering requiring domain knowledge and achieve much better results, even with only a few thousand training samples. We present the design and implementation of a deep neural network model referred to as *ElemNet*; it automatically captures the physical and chemical interactions and similarities between different elements using artificial intelligence which allows it to predict the materials properties with better accuracy and speed. The speed and best-in-class accuracy of *ElemNet* enable us to perform a fast and robust screening for new material candidates in a huge combinatorial space; where we predict hundreds of thousands of chemical systems that could contain yet-undiscovered compounds.

## Introduction

Materials scientists, condensed matter physicists and solid-state chemists rely on data generated by experiments and simulation-based models to discover new materials and understand their characteristics. For the major part of the history of materials science, experimental observations have been the primary means to know the various chemical and physical properties of materials^1–6^. Nevertheless, experimentation of all possible combinations of material composition and crystal structures is not feasible as that would be very expensive and time-consuming, and the composition space is practically infinite. Computational methods, such as Density Functional Theory (DFT)^7^, offer a less expensive means to predict many material properties and processes on the atomic level^8^. DFT calculations have offered opportunities for large-scale data collection such as the Open Quantum Materials Database (OQMD)^9,10^, the Automatic Flow of Materials Discovery Library (AFLOWLIB)^11^, the Materials Project^12^, and the Novel Materials Discovery (NoMaD)^13^; they contain DFT computed properties of ~10^4^–10^6^ of experimentally-observed and hypothetical materials. In the past few decades, such materials datasets have led to the new data-driven paradigm of materials informatics^14–19^. The availability of such large data resources has spurred the interest of researchers in applying advanced data-driven based machine learning (ML) techniques for accelerated discovery and design of new materials with select engineering properties^19–39^.

**Figure 1:**
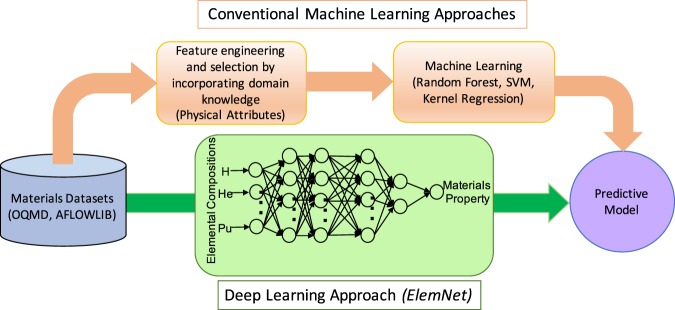
Comparison of deep learning approach with conventional ML approach for prediction of materials properties. The conventional ML approach for predictive modeling of materials properties involve representing the material composition in the model input format, manual feature engineering and selection by incorporating the required domain knowledge and human intuition by computing the important chemical and physical attributes of the constituent elements, and applying ML techniques to construct the predictive models. Our deep learning based predictive approach directly learns to predict properties of materials such as the formation enthalpy from their elemental compositions with better accuracy and speed than conventional ML approaches.

Conventionally, constructing an effective ML model requires first developing a suitable representation for the input data as shown in Fig. 1. As has been discussed in several recent works, the best representations are those that encode knowledge about the physics of the underlying problem. To that end, there have been many distinct approaches for encoding information regarding the composition^23,32^ or crystal structure^34,37,40,41^ of a material. For instance, Ward *et al*. developed a set of attributes based on the composition of a material that can be useful for problems including predicting formation enthalpies of crystalline materials and glass-forming ability of metal alloys^32^. Ghiringhelli *et al*.^42^ analyzed the tendency for materials to form different crystal structures using thousands of descriptors. Developing ML models based on intuitive representations is evidently successful given the large number and growing rate of ML models constructed over the past several years using this approach^18,19,43^. However, the prediction accuracy for these problems is limited by our ability to feature engineer the materials representation to incorporate all the domain knowledge required to make correct predictions. Given that one of the major use cases of ML is for problems where the physics driving behavior is yet to be understood^19^, this limit could be a significant impediment to the use of ML. A better approach would be to construct a system that can automatically learn the optimal representation.

Deep learning^44^ offers an alternative route for accelerating the creation of predictive models by reducing the need for designing physically-relevant features. It makes use of deep neural network (DNN) models composed of multiple processing layers (network architecture) to learn representations of data with multiple levels of abstraction^44^. DNN models can learn from input representations such as numerical encoding of texts, color pixels of images, etc., without any need to first compute application-specific descriptors^45–47^ thereby eliminating the manual step of feature engineering and representation required in conventional ML. Due to this powerful advantage, deep learning has gained significant attention in the field of computer science with breakthrough results in computer vision^48,49^, speech recognition^50,51^ and text processing^52^. Although deep learning models have enjoyed great success in the above applications, implementation of deep learning systems in materials science is in its early stages - mainly due to scarcity of big training datasets. Nevertheless, they have already shown some promise in materials science. Convolutional Neural Networks (CNN) have been used for building models from microstructural data and improving characterization methods^53–55^, and deep neural networks have been shown to be useful for predicting properties of crystal structures and molecules^56–58^.

Our goal in this work is to leverage the power and elegance of deep learning to directly learn the properties of materials from their elemental compositions, eliminating the limitations of current ML approaches that require manual feature engineering. We design a deep neural network model that we refer to as *ElemNet*, which takes only the elemental compositions as inputs and leverages artificial intelligence to automatically capture the essential chemistry to predict materials properties. Here, we evaluate the effectiveness of this approach by revisiting a commonly-studied challenge in materials informatics: predicting whether a crystal structure will be stable given its composition^23,32,59–61^. We adopt the approach of Meredig *et al*.^23^ and Ward *et al*.^32^, and train *ElemNet* on the DFT-computed formation enthalpies (the energy of forming a compound from its constituent elements) of 275, 759 compounds with unique elemental compositions from the OQMD. As demonstrated by Meredig *et al*., the formation energy predicted using this model can be compared to the formation energies of existing compounds in order to identify compositions where there is likely a yet-undiscovered compounds. In contrast to these previous papers which relied on physics-informed features to train a model, we approach this material prediction problem without using any domain knowledge about materials stability and rely purely on representation learning.

We find that *ElemNet* is able to automatically learn the chemical interactions and similarities between different elements which allows it to even predict the phase diagrams of chemical systems absent from the training dataset more accurately than conventional ML models based on physical attributes leveraging domain knowledge. We compared the performance of our deep learning model to a recent conventional ML approach that used engineered features^32^ on the OQMD; using a ten-fold cross validation, we find that *ElemNet* outperforms the conventional ML models both in terms of speed and accuracy for all training data size exceeding 4000 compounds. As deep learning frameworks support execution on Graphics Processing Units (GPUs), *ElemNet* can make predictions at two orders of magnitude faster than the physical attributes based ML models running on CPUs. The improved accuracy and higher speed of the model can allow us to perform combinatorial screening for new material candidates. As a case study, we perform a combinatorial screening in a huge composition space of around half a billion compounds, and find that our model successfully identifies compounds not in our training set. We believe *ElemNet* opens a new direction for more robust and faster identification of promising materials and thus, can play a crucial role in accelerating the materials discovery process.

## Results

### Dataset

We used the OQMD^10,62^ for training and testing our proposed deep learning model. OQMD is an extensive high-throughput DFT database, consisting of DFT computed crystallographic parameters and formation enthalpies of experimentally observed compounds taken from the Inorganic Crystal Structure Database (ICSD)^63^ and hypothetical structures created by decorating prototype structures from the ICSD with different compositions. OQMD is continually growing and, at the time of writing, contains 506,115 compounds at 275,778 unique compositions. We train our predictive models on the lowest formation enthalpy at each composition becauses they represent the most stable compounds, which causes our model to predict the energy of the ground-state structure given composition.

### Design

**Figure 2:**
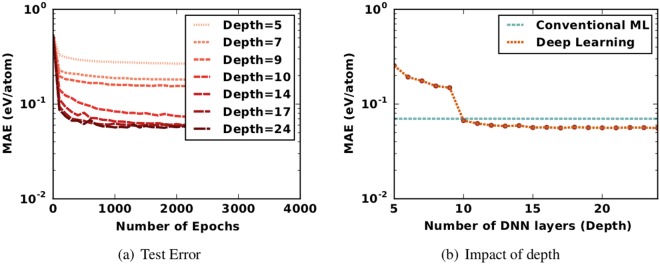
Performance of deep learning models of different depths in model architecture. The models are trained and tested on the lowest DFT-computed formation enthalpy of 256, 622 compounds. Here, we present the impact of depth of architecture for one sample split from our ten-fold cross validation. (**a**) Shows the mean absolute error (MAE) on the test dataset of 25, 662 compounds with unique compositions at different epochs for one split from the cross validation. The DNN models keep learning new features from the training dataset with the increase in the number of layers up to 17 layers, after which they begin to slowly overfit to the training data. (**b**) Shows the MAE for different depths of deep learning model architectures and also illustrates mean absolute error of the best performing conventional ML model trained using physical attributes computed on the same training and test sets. The deep learning model starts outperforming the best performing conventional ML model with an architecture depth of 10 layers, achieving the best performance at 17 layers, we refer to the best performing DNN model as *ElemNet*. The detailed architecture for *ElemNet* is available in the Method section.

We perform an extensive search for deep neural network (DNN) architectures and hyperparameters (details in Method section). Figure 2 illustrates the improvement in DNN learning capacity with the increase in the number of layers for different training epochs. From the test error plot, it is obvious that the learning capacity of DNN models improves with the increase in the depth of the network. The errors observed on training and test sets decrease rapidly up to 17 layers. After a certain depth, the improvement in learning of features by the DNN models starts plateauing. This plateauing effect can be a result of the features reaching the maximal extent of learning possible via our models. Figure 2(b) illustrates the overall comparison of the test errors of DNN models with different architecture depths. The best predictive model is a 17-layered DNN architecture (excluding four dropout layers) with tuned hyperparameters; we refer to this model as *ElemNet*. The model with 17 layers has the best accuracy of 0.050 ± 0.0007 eV/atom in 10-fold cross-validation, which is only 9% of the mean absolute deviation in the set (0.550 eV/atom). The detailed architecture of *ElemNet* is provided in the Method section. The results illustrate that deep neural networks can effectively learn the optimal feature representation from materials composition without any need for manual feature engineering using domain knowledge.

### Deep Learning vs Physical-attributes-based Conventional ML Approach

Our next step is to compare *ElemNet* against the current ML approach: conventional ML models that rely on the computation of physical attributes. We chose to compare *ElemNet* against the general-purpose approach of Ward *et al*., which uses 145 physical attributes that fall into four different categories - stoichiometric attributes, elemental property statistics, electronic structure attributes and ionic compound attributes^32^. As shown in Table 1, the models created using conventional ML are better with the physical attributes than with only the element fractions using the same training and test sets. We also find that deep learning surpasses all the conventional ML models–whether with physical attributes or not–in accuracy by at least 30%. This improvement in accuracy is quite fascinating as it is achieved without encoding any domain knowledge into the inputs of the function–a finding that shows carefully-developed features are not critical for success in ML if sufficient training data is available. While adding more domain knowledge is certainly expected to improve a ML model, for some problems, it may not be straightforward or even feasible to come up with appropriate physical attributes due to lack of understanding of the underlying phenomena. It is thus quite encouraging to find that this step of incorporating domain knowledge might not always be necessary to achieve excellent performance.

**Table 1:** Benchmarking our deep learning model–*ElemNet*–against conventional machine learning approaches.

Model	Input Type	MAE (eV/atom)	Training time (hour)	Prediction time (sec)
RandomForest	Physical Attributes	0.071 ± 0.0006	1.5	14.80
RandomForest	Elemental Compositions	0.157 ± 0.0012	1.5	2.87
***ElemNet***	**Elemental Compositions**	**0.050 **± **0.0007**	**7 (GPU)**	**9.28 (CPU) & 0.08 (GPU)**

We trained several conventional ML models such as Linear Regression, SGDRegression, ElasticNet, AdaBoost, Ridge, RBFSVM, DecisionTree, ExtraTrees, Bagging and Random Forest. Out of them, Random Forest performed the best with and without using physical attributes. Here, we show the results from our deep learning model and the best conventional ML model- Random Forest, in our study for both types of model inputs (without and without physical attributes), along with the type of input used, mean absolute error (MAE) on the test set, training time on the training set, and prediction time on the entire test set (25,662 entries). All the models are trained and tested using a ten-fold cross validation. All timings are on a single (logical) CPU core of an NVIDIA DIGITS DevBox with a Core i7-5930K 6 Core 3.5 GHz desktop processor with 64GB DDR4 RAM and 4 TITAN X GPUs with 12GB of memory per GPU, except the deep learning models.

### Impact of Training Data Size

Deep learning models have enjoyed great success in many applications, and typically these were applications where the training data is relatively abundant^44^. The perceived need for large datasets has discouraged many researchers in the scientific community having access to only small datasets from leveraging deep learning. To understand what the necessary dataset size is for deep learning to be effective for our application, we compared the effect of training dataset size on the accuracy of deep learning model and our best performing conventional ML model- Random Forest, with either the raw elemental compositions or the physical attributes as model inputs. We used different random subsets of the training dataset from the ten-fold cross validation with sizes ranging from 464 to 230,960 using a logarithmic spacing; the test set always contains 25,662 compounds. We used the same ten-fold training and test datasets for both *ElemNet* and Random Forest models (both with and without physical attributes) to ensure a fair comparison between the various approaches.

**Figure 3:**
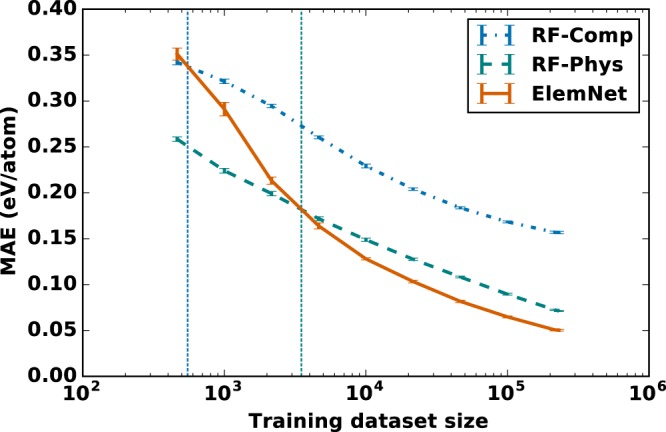
Impact of training dataset size on the prediction accuracy of *ElemNet* (DNN model) using elemental compositions only and the best conventional ML model, Random Forest, with either raw elemental compositions (RF-Comp) and physical attributes (RF-Phys). The training and test sets are created during the ten-fold cross validation from the OQMD; different random subsets of the training set with sizes ranging from 464 to 230, 960 are created using a logarithmic spacing for this analysis. Training dataset size has more impact on *ElemNet* (deep learning model) compared to Random Forest models, but *ElemNet* performs better than Random Forest for all size greater than 4*k*.

As illustrated in Fig. 3, our deep learning model achieves better accuracy than the best conventional ML approach based on physical attributes (manual feature engineering by incorporating domain knowledge) with only 2% of our training set. In general, *ElemNet* exhibits higher impact of training dataset size compared to the Random Forest models. The error curve has a steeper reduction in test error with the increase in training dataset size in the DNN model compared to Random Forest models. However, the important observation is that deep learning performs better than the Random Forest models even when the training dataset size is in ~10^3^–10^4^. It surpasses the accuracy of the Random Forest model with raw elemental compositions as input even at a training dataset size of 550, and the Random Forest model with physical attributes for all training dataset sizes exceeding 3500. Our results demonstrate that deep learning models can not only benefit more with an increase in dataset size compared to traditional ML models, but also deep learning can outperform them even at relatively smaller dataset size of around 4*k* samples. What the small training set requirement implies is that deep learning models such as *ElemNet* may be useful for building more accurate predictive models than conventional ML based models for many materials science datasets that are much smaller than the OQMD.

### Prediction Time Analysis

*ElemNet* predicts the formation enthalpy with better accuracy and speed. Table 1 shows the time taken by different predictive models to train on the training set and predict the formation enthalpy for the entire test set. All deep learning models are trained using GPUs and both the prediction time of deep learning using a single (logical) core of CPU as well as a GPU core are reported in Table 1. The prediction time of deep learning model is lower than the time required by the best conventional ML approach - Random Forest. Since deep neural networks mainly involve matrix multiplications, they are highly parallelizable compared to conventional ML methods such as Random Forest; hence, deep learning frameworks supports execution on GPUs. While running on GPUs, *ElemNet* can predict with two orders of magnitude faster than the current conventional ML models in practice. Our results illustrates that the proposed deep learning approach can predict with better accuracy as well as speed. It can, therefore, play a crucial role in accelerating the exploration of new composition spaces for materials discovery.

### Assessing Accuracy of Model

**Figure 4:**
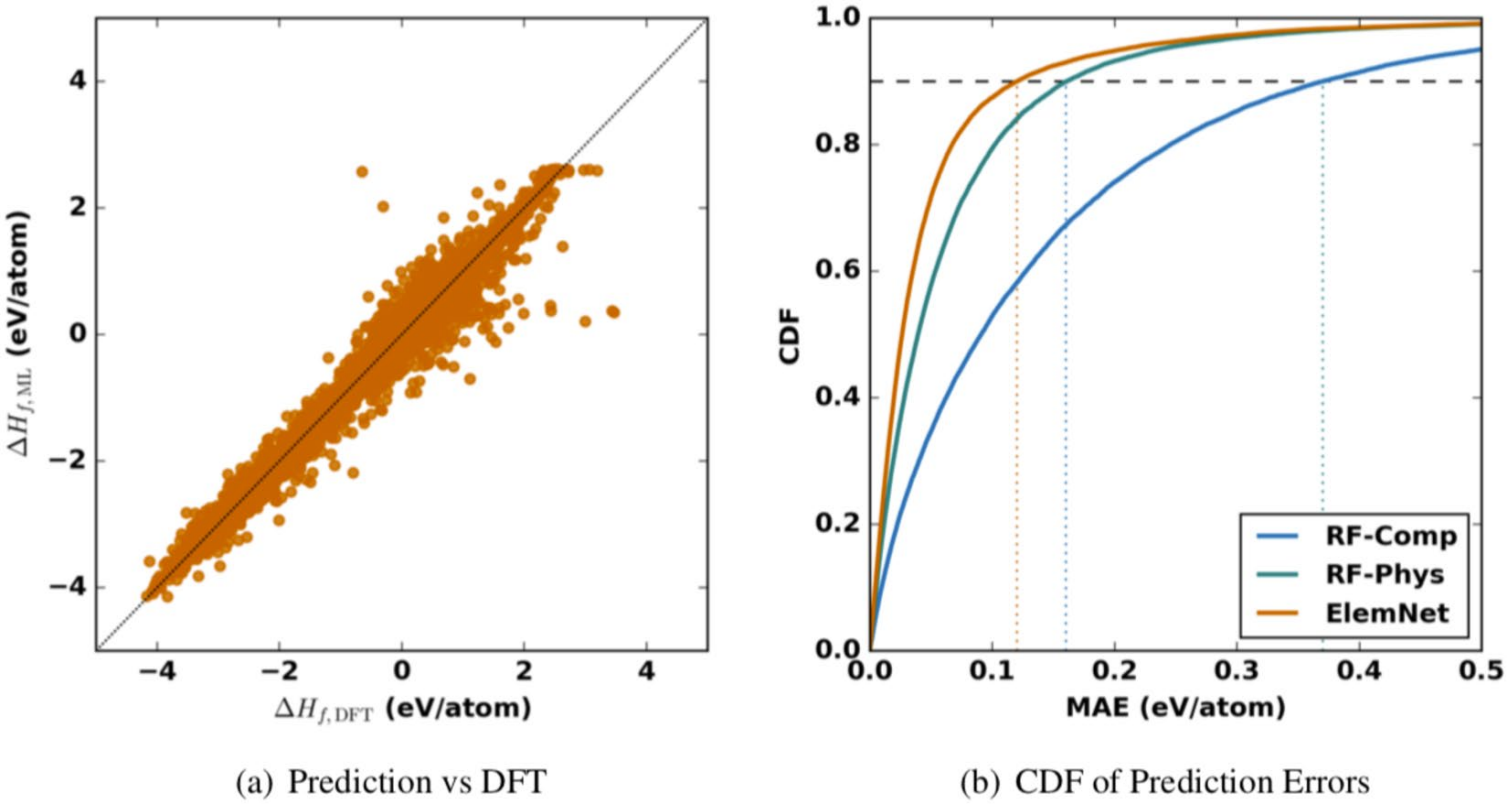
Error analysis of the predictions using *ElemNet* of a test set containing 25,662 compounds from our ten-fold cross validation. The left side shows that the predicted values are very close to the DFT-computed values. The right side illustrates the cumulative distribution function (CDF) of the prediction errors for *ElemNet* and Random Forest (the best performing conventional ML model) with elemental fractions (RF-Comp) and physical attributes (RF-Phys). Our error analysis demonstrates that the deep learning performs very well, achieving an MAE of 0.050 ± 0.000 eV/atom; predicting with an absolute error of less than 0.120 eV/atom for 90% of the compounds in our test set (right).

Our deep learning model achieves strong performance across a broad range of materials. As shown in Fig. 4b, *ElemNet* predicts the formation enthalpy of compounds in one of our test sets with a mean absolute error (MAE) of 0.055 eV/atom; predicting the formation enthalpy of 90% of compounds in our test set with an error of less than 0.120 eV/atom. To better understand how our model could be best used, we studied for which kinds of materials it performs the least accurately. The materials where our model has the largest errors typically have large, positive formation enthalpies (see the outliers in Fig. 4a), which suggests our model performs the worst at trying to predict the formation enthalpy of highly unstable compounds. Only 59% of our test set has a positive formation enthalpy yet 67% of the entries with the largest errors (99% percentile of absolute error) have positive formation enthalpies. These unstable compounds are arguably the least physically important part of the dataset, and therefore the inability of *ElemNet* to accurate predict these energies is not a significant drawback.

We also studied how *ElemNet* performs on different chemical classes of materials. The 25 entries with the highest errors include intermetallics (e.g., Cr_2_Ni_3_), metal/nonmetal compounds (e.g., Ho_2_C, Sm_3_AlN), and compounds with only non-metallic elements (e.g., BCl), so there does not seem to be a systematic problem with modeling a particular material class. To further understand if certain chemistries have larger errors, we first grouped entries in the test set by whether they contained certain elements and then computed the Spearman rank correlation coefficient for each group. The elements that exhibit the lowest correlation coefficients are Pu (0.66), Np (0.86), C (0.87), and N (0.87). The Pu and Np compounds are likely to have the lowest performance because they have the fewest number of training points among metallic elements. C and N both appear much less frequently in our training set than any metallic element because they are not included in the combinatorial searches for intermetallics, whose results constitute the bulk of the OQMD. Among these elements which appear less often in the OQMD (Br, C, Cl, F, H, I, N, P, S, Se, Xe), C and N have the highest number of compounds with positive formation enthalpies in the test set. Consequently, we conclude the poor performance on C- and N-containing compounds is also a result of the poor performance of the model on unstable material and not because of a systematic issue with modeling certain elements.

The types of compounds where *ElemNet* performs best also line up with our expectations. The elements with the highest correlation coefficients are lanthanides and alkali metal compounds. Lanthanides display a strong degree of chemical similarity (e.g., all form trivalent cations), and so we would expect the properties of lanthanide compounds to be relatively easy to predict if our model can recognize the similarity between these elements. Additionally, alkali metals are most often observed in single oxidation state (1+), which makes their chemistry somewhat simpler than most transition metals. In terms of the nonmetals, our model has the best performance on Se-, F-, and Cl-containing compounds, which have the highest fraction of compounds with negative formation enthalpies. In general, we find that *ElemNet* has strong predictive performance across many classes of materials and is most accurate for stable compounds that contain elements with fewer possible oxidation states.

### Learning Interaction between Elements

Due to the absence of domain knowledge in materials representation for *ElemNet*, one potential issue that might arise is that it may have difficulty generalizing trends learned from one materials system to systems not included in the training set. When presented with an entry from a system that was not included in a training set, the inputs to *ElemNet* would be in a previously-unobserved portion of feature space. In contrast, models that rely on physical features suffer from this problem less. For example, consider a case where a training set contains no entries with both Ti and O together, and a ML model is tasked with predicting the formation enthalpy of TiO_2_. A model trained on the features from Ward *et al*.^32^ would be provided with useful information such as “TiO_2_ is charge-balanced given the known oxidation states of Ti and O”, and that “Ti_2_O_3_ has a similar difference in electronegativities to Al_2_O_3_”. Without these physical features as guidance, the prediction task for *ElemNet* could potentially be more difficult.

To further test the predictive accuracy of *ElemNet* with respect to the above-described concern, we designed a holdout test where we withheld all training examples from several systems. We first analyzed the training set to determine that Ti-O is the binary chemical system with largest number of compositions in the training set and, similarly, that Na-Mn-O and Na-Fe-O are the two most common ternary chemical systems. Next, we created two separate training sets and test sets for two different holdout tests. For the first test, we withheld all entries that contain both Ti and O to use as a test set (561 entries) and used all other entries as a training set. For the second test, we withheld all entries from the Na-Fe-Mn-O quaternary phase diagram (i.e., any compound that contains exclusively Na, Mn, Fe, and O) - total of 96 entries. Each of these training/test splits provides a unique way for evaluating whether a ML model can accurately assess previously-unobserved combinations of elements.

We found that *ElemNet* outperformed both Random-Forest-based models (with and without physical features) in both of these cross-validation tests. The RF model without physical features achieves an MAE of 0.323 eV/atom on the Ti-O holdout test, and a MAE of 0.405 eV/atom on the Na-Fe-Mn-O holdout test. The performance of this model is quite poor when considering that the mean absolute deviation of the test sets are 0.478 and 0.792 eV/atom for the Ti-O and Na-Fe-Mn-O tests, respectively. The RF model using physical attributes is significantly better with MAE of 0.198 and 0.179 eV/atom for each test, which again illustrates the importance of physical features for conventional machine learning models. We found that *ElemNet* achieves markedly better performance on both tests (MAE of 0.138 and 0.122 eV/atom), demonstrating that *ElemNet* can infer the properties of unobserved chemical systems better than existing machine learning models.

**Figure 5:**
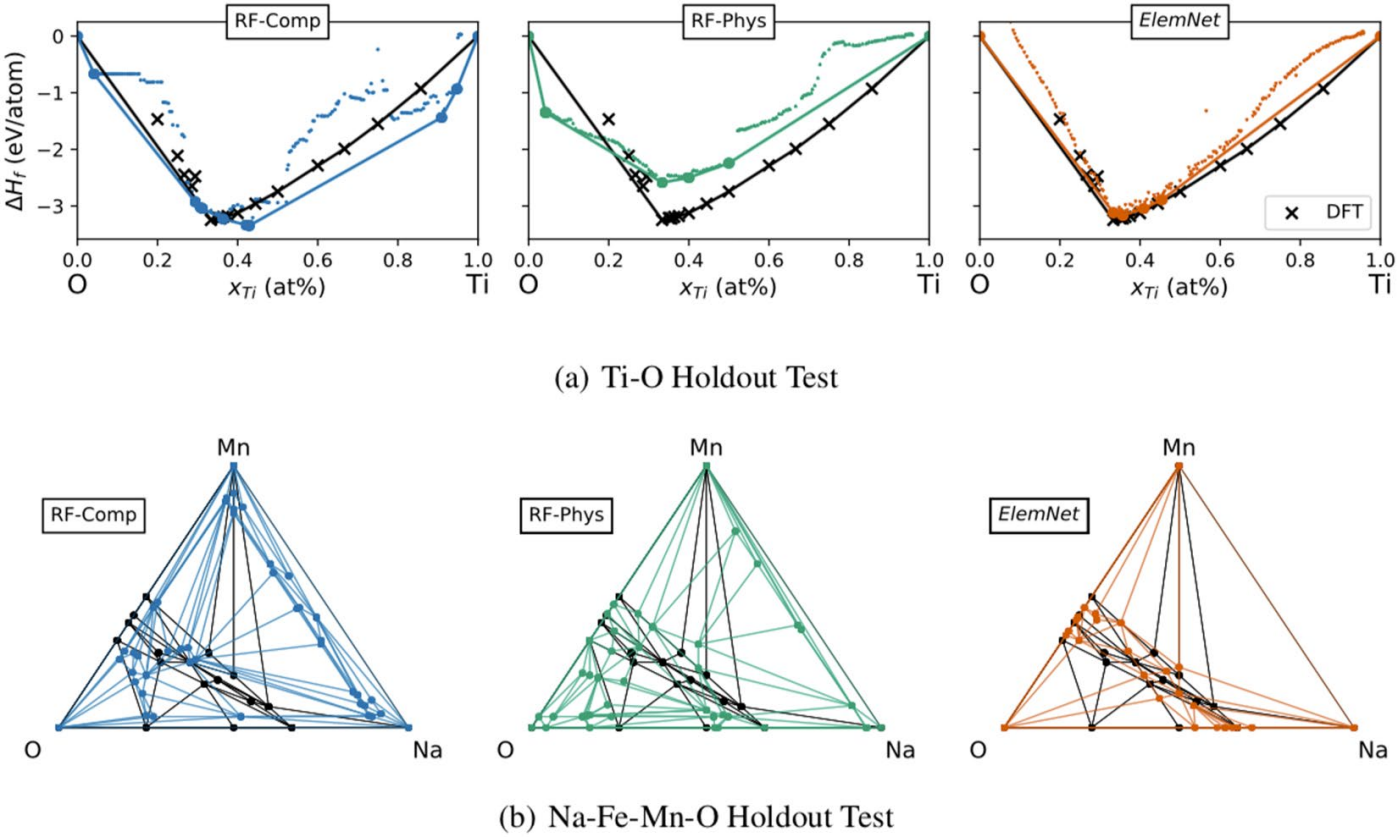
Predicted phase diagrams from the hold-out test. These charts show the convex hulls predicted for the (**a**) Ti-O binary and (**b**) Na-Mn-O from ML models that were trained without any data from each system in their training set. We compare the performance of a Random Forest model trained using only element fractions (RF-Comp), RF trained using physical features (RF-Phys) and a deep learning model (*ElemNet*). Each vertex on the convex hull corresponds to the composition of a stable compound. The black lines on each chart show the OQMD convex hull. We find that the deep learning model has the fewest predictions outside the regions where compounds are known to form, for both the Ti-O and Na-Mn-O phase diagrams.

*ElemNet* having quantitatively better accuracy on the test sets is promising, but it still does not effectively capture whether this network is better at discovering stable compounds. To test the discovering potential of each model, we emulated searching for stable compounds by using each model to evaluate a large number of candidate materials from each of the systems held out from the training set. These systems are composed of commonly-occurring elements, for these tests we assume that they are well studied and that there are no yet-undiscovered compounds that are not included in the OQMD. Figure 5 illustrates the formation enthalpies and convex hull predicted by each of the ML models, compared to the known DFT result. We find that *ElemNet* reproduces the Ti-O and Na-Mn-O phase diagrams the most accurately. All three models correctly identify that there should be a stable compound near TiO_2_, and all miss the Ti-rich stable compounds (e.g., Ti_2_O). This happens because the Ti-rich stable compounds have the Magneli phases which is specific to Ti-O system which are absent from training set; hence, they can not learn the specific behavior of Ti-rich compounds^64,65^. However, both Random Forest models predict spurious minima near pure O, while *ElemNet* makes no spurious predictions. *ElemNet* also has the fewest number of spurious predictions in the Na-Mn-O system, where it captures that ternary compounds are only known to form in the region bounded by Na_2_O, MnO_2_, and MnO. In contrast, the two RF-based models predict many stable compounds in Na- and O-rich regions where no compounds are known to exist. Consequently, we conclude that our deep learning model achieves not only better accuracy on these holdout tests but it can also predict the locations of unknown, stable phases with much higher fidelity than current best ML based predictive techniques.

### Chemistry Insights

*ElemNet* is evidently able to learn a useful representation of materials, given its strong prediction scores in the ten-fold cross validation and the hold-out tests. To understand how this network is performing so well, we studied the representation learned by the network. In deep neural networks, the inputs (known as activations) to each successive hidden layer become less related to the input data and more strongly related to the output. In our case, the activations for each layer are incrementally better representations of compositions for predicting formation enthalpy. We interrogated these representations by providing specific inputs to the network and measuring the activations of the network for several hidden layers. We can then understand the behavior of the network by comparing how the activations change for different materials.

**Figure 6:**
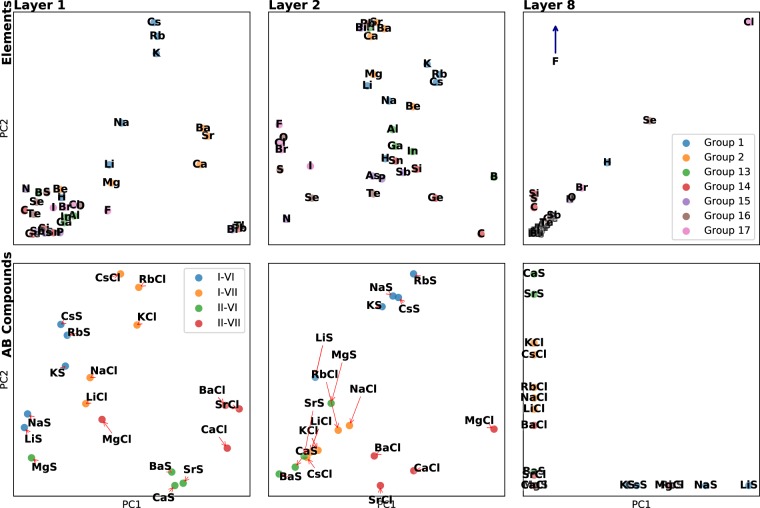
Visualization of the activations of different materials in *ElemNet*. Each frame shows a 2D projection (using PCA) of the activations of different materials in several layers of *ElemNet*, which shows which materials have similar representations. The upper row shows the activations of different elements, where each point is a different element and is colored by the group number. The second row shows the activations of AB compounds formed of group I and II metals combined with S (group VI) or Cl (group VII). We note that elements from the same group in the periodic table, such as alkali metals, are clustered together in the early layers of the network, and that later layers reflect properties related to combinations of elements (e.g., charge balance).

Specifically, we studied the activations of different main group elements and AB compounds that contain S or Cl paired with an Group I or Group II metal. Figure 6 shows the activations for each subset for the 1st, 2nd, and 8th layers of the network. As the hidden layers are composed of a large number of activations, we only considered the first two principal components of activations for this analysis. By projecting the activations down to a two-dimensional representation, we can view which compositions have similar representations and, with our knowledge of materials science, infer what kind of features the network is learning.

The 1st layer of the network exhibits clustering between elements based on their group number. The alkali and alkali earth metals, in particular, are easily identifiable and well-separated from the elements of other groups. Several groups of elements are also well-ordered by their period. The alkali metals group is ordered H, Li, Na, K, Rb, Cs from left to right and the halogens are ordered in a descending period. Elements groups are also separated where appropriate. Bi is clustered near Pb and Tl but not other chalcogens, which makes sense given that is the only metal in its group. B is also separated from the cluster containing Al, Ga, and In, which reflects that B is a metalloid unlike the other metallic elements in Group 13. Given the remarkably-clear periodic trends, it is worth emphasizing that no information about groups and periods of the periodic table was provided to *ElemNet*; all of these similarities are learned from the data.

The clustering of elements becomes less clear in later hidden layers in the network. Groups of elements are still clearly visible in Layer 2, although the ordering by period is less evident. By Layer 8, periodic trends are nearly unrecognizable in the activations of each element. One possible explanation is that each layer of the network is gradually learning more complex features in a way similar to networks built for image classification^44,48^. The early layers of the network are learning features based directly on the input values (i.e., presence of certain types of elements). Later layers in the network are learning more complex features of the compositions that have more to do with the interactions between elements than the types of elements present, which would explain why the similarity of elements becomes less visible in the activations.

To test our hypothesis that later layers in the model network capture features related to interactions between elements, we measured the activations AB compounds composed of alkali and alkaline earth metals combined with S or Cl. In the first layer, the compounds are clustered by similar groups and the distances between clusters are related to chemical similarity. The I-VII compounds (e.g., LiCl) are clustered together and closer to II-VII (for example, MgCl), which contain one element from the same group, than they are to II-VI compounds, which have no groups in common with I-VII compounds. Grouping based on similarity of element groups becomes less apparent in the second layer. I-VII compounds are now closer to II-VI compounds than any other group. We hypothesize that this change in the grouping is a result of both I-VII and II-VI compounds being charged balanced, which means they should have more negative formation enthalpies. The activations of the 8th layer show some of the I-VI and II-VI compounds together, though there are more violations of the rule (for example, BaS is far from CaS). The grouping based on charge balance is imperfect (Be-containing compounds from a separate cluster from the other group II compounds), but it is clear that the later layers are more related to interactions between elements than the presence of single elements. Overall, the activations for both single elements and binary compounds demonstrate the power of deep learning networks to learn essential domain knowledge without specially-designed inputs.

### Combinatorial Screening for New Materials Candidates

As our deep learning model can make robust and fast predictions, it can be used to perform combinatorial screening in huge composition space for discovery of new materials. As a case study, we conducted a combinatorial screening using our model in a huge composition space of around half a billion compounds to study if it can identify stable compounds which are not present in our training set. We first generate a list of about 450*M* hypothetical compounds of the form A_*w*_B_*x*_C_*y*_D_*z*_ where the elements (A-D) can be any of the 86 elements in the OQMD besides He, Ne and Ar, and *w*-*z* are positive integers where *w* + *x* + *y* + *z* ≤ 10. The order of the elements are not fixed based on electronegativity. The compositions are unique in the sense that the ratio of constituent elements, i.e., we take *AB* and *A*_2_*B*_2_ as one composition *AB* since they have same composition ratio. Since we are taking the combination, there is no duplicate counting. We then evaluate the Δ*H*_*f*_ of these compositions using *ElemNet*. As *ElemNet* is two orders of magnitude faster than the current best ML based predictive models^23,32^, it allows extremely fast scanning for the discovery of new materials compared to the models in practice–we scan the entire composition space of 450*M* within few days of GPU time.

We identified compositions where it could be possible to form a new compound by identifying the compositions where *ElemNet* predicted a formation enthalpy much lower than the OQMD convex hull. Specifically, we computed the difference between the Δ*H*_*f*_ predicted by *ElemNet* at each composition to the Δ*H*_*f*_ of the OQMD convex hull at that composition. Considering that 95% of the predictions on our test set had an error less than 0.2 eV/atom, we removed all predictions where this difference is smaller than 0.2 eV/atom to identify the predictions most likely to be correct. In total, we found 232 binary, 14,366 ternary, and 353,352 quaternary chemical systems out of the 4.3*M* compositions where the *ElemNet* Δ*H*_*f*_ is below the current OQMD hull by at least 0.2 eV/atom. The list of these binary and ternary compositions is available in its entirety in the Supplementary Material (we could not upload the quaternary compositions due to space limit for Supplementary Material).

Our first step for validating these predictions was to determine whether any compositions correspond to known compounds from the Inorganic Crystal Structure Database (ICSD) that are absent from the OQMD. These “missing” ICSD compounds are reasonable guesses for stable compounds, as many ICSD compounds are stable. We assembled a list of ICSD compounds not in the OQMD by first identifying all 92,756 unique compositions of compounds in the ICSD and then the 63,823 that are farther than 1% (measured using the *L*_2_ distance) of an entry in our training set. If we restrict the prediction to be within 1% of the ICSD composition, the 4.3*M* predicted compositions includes 29 ICSD binary compounds not in the OQMD, 179 ternary compounds, and 80 quaternary compounds. If we decrease the tolerance to 10%, our model identifies 108 of the missing ICSD binary compounds, 1,121 ternaries, and 1,087 quaternaries. The number of ICSD compounds we find with our *ElemNet* model is small compared to the number of ICSD compounds not in the OQMD, but this is not unexpected. For one, we apply a large threshold for the hull distance (0.2 eV/atom), such that the compounds we find must be very stable compared to compounds already in the OQMD. Finding some predictions from *ElemNet* that match up to ICSD entries shows *ElemNet* is at least identifying compounds that are reasonable to assume to be stable.

To further characterize the predictions of *ElemNet*, we analyzed how the predictions are distributed across composition space. Over 20% of the systems predicted to contain new stable compounds include lanthanides or actinides, which is unsurprising given that compounds of these elements have not been studied as extensively as other elements. We, therefore, exclude actinide and lanthanide compounds from further analysis, and identify predictions from systems with more commonly occurring elements for further study, as shown in Table 2. The predictions for compounds that include Li, K, or Na are particularly illustrative. We note that our model predicts KF_6_, NaF_8_, OF_9_ and SeF_9_ to be stable, which is unlikely given the known oxidation states and suggests *ElemNet* underestimates the enthalpy of F-containing compounds, especially at high F-fractions. The predictions for the ternary compounds are interesting as they reflect realistic oxidation states of each element despite the model having no information about oxidation states in the input. Additionally, KY_2_F_7_ and NaY_2_F_7_ are reasonable predictions given that they have already been synthesized experimentally^66^. NaY_2_F_7_ is indeed stable in the OQMD and KY_2_F_7_ is only unstable by 50 meV/atom. The prediction of quaternary fluorides with Na and Cs are also reasonable, given their similar stoichiometries to many known Elpasolite phases^67^. Overall, the predictions for Li-, K-, or Na-containing compounds illustrates that *ElemNet* is making reasonable predictions. The few numbers of predictions of new 3*d* metals oxides are in agreement with our expectations, given how extensively these materials have been studied. The only new binary oxide we predicted is Cu_2_O, which is a known compound and appears in this list because *ElemNet* overestimates its formation enthalpy. We also predict Zn_2_Cu_3_O_3_ to be stable, which is unlikely because ZnO-CuO is known to be phase separate^68^. These two unlikely predictions suggest that the formation enthalpies of Cu oxides may be generally overestimated by the models, which could be an effect of Cu_2_O being in the test set for *ElemNet* rather than the training set. The quaternary prediction, TiZnCrO_5_, is potentially interesting given that it is charged balanced and that there are already several known ABCO_5_ oxides^69,70^. Overall, these few subsets of compounds once again show that *ElemNet* is making reasonable predictions for new materials–an outstanding feat given how little knowledge of materials science was used to create it.

**Table 2:** Subset of Potential Stable Compounds Predicted using *ElemNet*. Out of the 450 M predictions, we determined the number of systems where *ElemNet* identifies at least one new potential stable compound.

Category	Binary		Ternary		Quaternary	
	Count	Examples	Count	Examples	Count	Examples
[Li,K,Na]-Containing	4	KF_6_ NaF_8_	707	***NaY***_**2**_ ***F***_**7**_ ***KY***_**2**_ ***F***_**7**_	18446	CsNa_2_CdF_4_ Na_2_CrPbF_5_
Chalco-/oxyhalides	5	OF_9_ SeF_9_	522	Y_2_OF_6_ Sc_2_OF_7_	17184	Sr_3_Cu_2_IO_4_ Zr_6_RhIO_2_
Metal Oxides	1	***Cu***_**2**_ ***O***	81	KTi_4_O_5_ ReAu_2_O_5_	501	YAlV_2_O_6_ Y_4_FeBi_2_O_3_
3*d* Metal Oxides	1	***Cu***_**2**_ ***O***	3	Zn_2_(CuO)_3_ Ti_5_CuO_2_	1	TiZnCrO_5_
Intermetallics	11	Nb_5_Sn_3_ Al _5_Ir_3_	123	HfAl_5_Ir_3_ YAl_4_Ir_3_	425	Sc_5_NiSn_3_Mo ZrAl_5_OsRh
Intermetallics HHI_p_ < 2500	0		0		1	NaMn_2_AlAu_6_

We list the number of binary, ternary, and quaternary systems for several categories of compounds along with the two most stable predictions. We validated some of the these compounds- *NaY*_2_*F*_7_ and *KY*_2_*F*_7_ using DFT computations by leveraging crystal structures of existing materials with similar stoi-chemistry; we found them to be stable using DFT, further literature search revealed that they have already been synthesized recently. Our model predicts *Cu*_2_*O* as the only new binary oxide which is a known compound but was not in our training set.

## Discussion

Conventional predictive ML modeling approaches require manual feature engineering of materials representation to incorporate domain knowledge in the model inputs. However, there is no consensus among researchers on how many and which physical attributes to include into the model inputs, such that they incorporate all the important domain knowledge required to make accurate predictions. Here, we demonstrated that the need to engineer features for materials can be bypassed by leveraging a deep learning approach. A deep learning model can learn the optimal materials representation required for the prediction task by automatically capturing the chemical interactions between different elements from the training dataset using artificial intelligence, without any need for manual feature engineering, domain knowledge or human intuition; which can allow it to make better prediction for chemical systems absent in the training set than the conventional ML models.

The general belief in scientific community is that deep learning techniques require big training datasets^44^ to perform well; however, we demonstrate that *ElemNet* can perform better than conventional ML models by leveraging only 2% of the OQMD dataset for training, which shows that deep learning can be used to build predictive models on relatively smaller materials and scientific datasets such as of size 4*K*. Our results provide a stimulus for researchers to use DNN based approaches for building predictive models on their datasets. Since the proposed deep learning approach yielded the highest accuracy to date, it provides a new direction for more robust and fast predictions to identify composition regions containing materials with strong-negative formation enthalpies for discovery. We scanned around 450 million candidate compositions for novel ternary and quaternary compounds, and predicted that new stable compounds could be found in about 368*k* different chemical systems. The entire list is made available in the Supplementary Material to facilitate further research and analysis for accelerating the process of new materials design and discovery. We have added *ElemNet* to our existing online formation enthalpy calculator^23,71^ publicly available at http://info.eecs.northwestern.edu/FEpredictor so that researchers can publicly access and evaluate its predictions. The model is also available at https://github.com/dipendra009/ElemNet with the trained weights and sample code to demonstrate how to load and use the model for making predictions and performing combinatorial screening for new materials discovery. We plan to keep refining the model by training on larger datasets as they become available in future which will help in further improvement in the prediction results.

### Data Cleaning Methods section heading is missing before this subsection

The data is composed of fixed size vectors containing raw elemental compositions in the compound as input and formation enthalpy in eV/atom as output labels. The input vector has non-zero values for all the elements present in the compound and zero values for others. As most compounds are composed of fewer than five elements, the input vector is very sparse. The composition ratio is normalized so that the elements of the input vector sum to one. Two stages of data cleaning are performed to remove single element compounds and outliers. First, all single-element materials are removed as their formation energy is zero, by definition. Next, data entries with formation energy values outside of ±5*σ* (*σ* is the standard deviation in the training data) are removed. Such outliers are discarded to prevent calculation errors undetected by strict value bounds. Further, the elements (attributes) that do not appear in the cleaned dataset are removed from the input attribute set. Out of 118 elements in the periodic table, 86 elements are present in our dataset. Our dataset contains 256,622 compounds after cleaning, out of which there are 16,339 binary compounds, 208,824 ternary compounds, and 31,459 compounds with between 4 and 7 constituent elements. The dataset (after cleaning) is randomly split into training and test sets using a ten-fold cross validation; each training set and test set contain 230,960 compounds and 25,662 compounds with unique compositions and their minimum formation enthalpies.

### Model Architecture Search

Our deep learning model is based on a deep neural network (DNN) composed of multiple consecutive layers of neurons. To find the best model for the formation enthalpy prediction, we carry out an extensive search for the best DNN model architecture as well as in the hyper-parameters space. We performed a systematic search through a large neural network architecture space, starting from a two-layered architecture and incrementally increasing the depth to improve the learning capacity of our model until a saturation point is reached. We explored with different combinations of the number of neurons units per layer. A dropout^72^ layer was added whenever the number of neurons between consecutive layers changed to avoid overfitting^73^. The test error started oscillating within small limits beyond 17-layered architecture. The architecture search was continued up to 24 layers DNN model where the test error remained same as the 17 layered network. We believe that the deep learning model already learned the necessary features it could find in the training dataset at this point, as increasing the depth did not improve the model performance any further. The best model architecture is shown in Table 3. We also experimented with different types of activation functions, and ReLU (rectified linear unit)^74^ was observed to perform the best.

**Table 3:** *ElemNet* Architecture.

Layer Types	No. of units	Activation	Layer Positions
Fully-connected Layer	1024	ReLU	First to 4th
Drop-out (0.8)	1024		After 4th
Fully-connected Layer	512	ReLU	5th to 7th
Drop-out (0.9)	512		After 7th
Fully-connected Layer	256	ReLU	8th to 10th
Drop-out (0.7)	256		After 10th
Fully-connected Layer	128	ReLU	11th to 13th
Drop-out (0.8)	128		After 13th
Fully-connected Layer	64	ReLU	14th to 15th
Fully-connected Layer	32	ReLU	16th
Fully-connected Layer	1	Linear	17th

Considering the Input as the 0th layer, types and positions of different types of fully connected and dropouts are shown below. Dropout layers are used to prevent overfitting and they are not counted as a separate layer. We used ReLU as the activation function.

### Model Hyperparameter Search

We performed an extensive search to tune the model hyperparameters as recommended by Bengio *et al*.^75^ We started with a small range of values for each hyperparameter based on our intuition, rather than performing a grid search that would have been infeasible due to time and computational resource constraints. The hyperparameter search space comprised of different candidate values of momentum^76^, learning rate^77^, optimization algorithms, dropouts^72^ and other hyperparameters. Learning rate was one of the most important DNN hyperparameters. Learning rates values from 0.1 to 1*e*^−6^ were tried, decreasing by a factor of 10. Dropouts^72^ are known to have a great impact on decreasing the overfitting^73^ of the model to training set^78^. A search for dropout values ranging from 0.5 to 0.9 (dropout value denotes the inputs retained, such as 0.7 means 30% input values are dropped and rest 70% are used) was carried for each of the four dropout layers used in our DNN models. Increasing dropout helped in improving prediction accuracy as it decreased overfitting the of model to the training dataset. For momentum, we experimented with values in the [0.9, 0.95, 0.99]; momentum value of 0.9 performed the best. Stochastic gradient descent (SGD) performed best among all optimization algorithms in our study. Similarly, we experimented with a range of values for other hyperparameters.

### Machine Learning Parameter Search

We performed a thorough grid search for parameters of all ML models used in this study. For instance, we experimented Random Forest regression with a number of different combinations of estimators in [50, 100, 150, 200], minimum samples splittings in^5,10,15,20^, maximum features in [0.25, 0.33] and maximum depths in^10,25^.

### Experimental Settings and Tools Used

The deep learning models are implemented using Python 2.7, Theano^79^ and TensorFlow^80^ framework. For other ML models, implementations available in Scikit-learn^81^ are used. All the models were trained and tested using NVIDIA DIGITS DevBox.

## Electronic supplementary material

Potential Stable Systems

## Data Availability

The OQMD dataset used for experiments in this work are openly available at http://www.oqmd.org.

## References

[1] Kubaschewski O, Slough W (1969). Recent progress in metallurgical thermochemistry. Progress in Materials Science.

[2] Kubaschewski, O., Alcock, C. B. & Spencer, P. Materials Thermochemistry. *Revised* (1993).

[3] Bracht H, Stolwijk N, Mehrer H (1995). Properties of intrinsic point defects in silicon determined by zinc diffusion experiments under nonequilibrium conditions. Physical Review B.

[4] Turns SR (1995). Understanding nox formation in nonpremixed flames: experiments and modeling. Progress in Energy and Combustion Science.

[5] Uberuaga BP, Leskovar M, Smith AP, Jónsson H, Olmstead M (2000). Diffusion of ge below the si (100) surface: Theory and experiment. Physical review letters.

[6] Van Vechten J, Thurmond C (1976). Comparison of theory with quenching experiments for the entropy and enthalpy of vacancy formation in si and ge. Physical Review B.

[7] Kohn W (1999). Nobel lecture: Electronic structure of matterwave functions and density functionals. Reviews of Modern Physics.

[8] Hafner J, Wolverton C, Ceder G (2006). Toward computational materials design: the impact of density functional theory on materials research. MRS bulletin.

[9] Saal JE, Kirklin S, Aykol M, Meredig B, Wolverton C (2013). Materials design and discovery with high-throughput density functional theory: the open quantum materials database (oqmd). Jom.

[10] Kirklin S (2015). The open quantum materials database (oqmd): assessing the accuracy of dft formation energies. npj Computational Materials.

[11] Curtarolo S (2012). AFLOWLIB.ORG: A distributed materials properties repository from high-throughput ab initio calculations. Computational Materials Science.

[12] Jain A (2013). Commentary: The materials project: A materials genome approach to accelerating materials innovation. Apl Materials.

[13] NoMaD, http://nomad-repository.eu/cms/.

[14] Agrawal A, Choudhary A (2016). Perspective: Materials informatics and big data: Realization of the “fourth paradigm” of science in materials science. APL Materials.

[15] Hey, T. *et al*. *The fourth paradigm: data-intensive scientific discovery*, vol. 1 (Microsoft research Redmond, WA, 2009).

[16] Rajan K (2015). Materials informatics: The materials “gene” and big data. Annual Review of Materials Research.

[17] Hill J (2016). Materials science with large-scale data and informatics: unlocking new opportunities. Mrs Bulletin.

[18] Ward L, Wolverton C (2017). Atomistic calculations and materials informatics: A review. Current Opinion in Solid State and Materials Science.

[19] Ramprasad R, Batra R, Pilania G, Mannodi-Kanakkithodi A, Kim C (2017). Machine learning in materials informatics: recent applications and prospects. npj Computational Materials.

[20] Pozun ZD (2012). Optimizing transition states via kernel-based machine learning. The Journal of chemical physics.

[21] Montavon, G. *et al*. Machine learning of molecular electronic properties in chemical compound space. *New Journal of Physics, Focus Issue, Novel Materials Discovery* To appear (2013).

[22] Agrawal A (2014). Exploration of data science techniques to predict fatigue strength of steel from composition and processing parameters. Integrating Materials and Manufacturing Innovation.

[23] Meredig B (2014). Combinatorial screening for new materials in unconstrained composition space with machine learning. Physical Review B.

[24] Kusne, A. G. *et al*. On-the-fly machine-learning for high-throughput experiments: search for rare-earth-free permanent magnets. *Scientific reports* **4** (2014). 10.1038/srep06367 PMC4163667 25220062

[25] Fernandez M, Boyd PG, Daff TD, Aghaji MZ, Woo TK (2014). Rapid and accurate machine learning recognition of high performing metal organic frameworks for co2 capture. The journal of physical chemistry letters.

[26] Kim C, Pilania G, Ramprasad R (2016). From organized high-throughput data to phenomenological theory using machine learning: the example of dielectric breakdown. Chemistry of Materials.

[27] Liu, R. *et al*. A predictive machine learning approach for microstructure optimization and materials design. *Scientific reports* **5** (2015). 10.1038/srep11551 PMC4477370 26100717

[28] Xue, D. *et al*. Accelerated search for materials with targeted properties by adaptive design. *Nature communications* **7** (2016). 10.1038/ncomms11241 PMC4835535 27079901

[29] Faber FA, Lindmaa A, Von Lilienfeld OA, Armiento R (2016). Machine learning energies of 2 million elpasolite (a b c 2 d 6) crystals. Physical review letters.

[30] Oliynyk AO (2016). High-throughput machine-learning-driven synthesis of full-heusler compounds. Chemistry of Materials.

[31] Raccuglia P (2016). Machine-learning-assisted materials discovery using failed experiments. Nature.

[32] Ward L, Agrawal A, Choudhary A, Wolverton C (2016). A general-purpose machine learning framework for predicting properties of inorganic materials. npj Computational Materials.

[33] Ward L (2017). Including crystal structure attributes in machine learning models of formation energies via voronoi tessellations. Physical Review B.

[34] Isayev O (2017). Universal fragment descriptors for predicting properties of inorganic crystals. Nature communications.

[35] Legrain F, Carrete J, van Roekeghem A, Curtarolo S, Mingo N (2017). How chemical composition alone can predict vibrational free energies and entropies of solids. Chemistry of Materials.

[36] Stanev V (2018). Machine learning modeling of superconducting critical temperature. npj Computational Materials.

[37] Seko A, Hayashi H, Nakayama K, Takahashi A, Tanaka I (2017). Representation of compounds for machine-learning prediction of physical properties. Physical Review B.

[38] De Jong M (2016). A statistical learning framework for materials science: application to elastic moduli of k-nary inorganic polycrystalline compounds. Scientific reports.

[39] Bucholz EW (2012). Data-driven model for estimation of friction coefficient via informatics methods. Tribology Letters.

[40] Schütt K (2014). How to represent crystal structures for machine learning: Towards fast prediction of electronic properties. Physical Review B.

[41] Faber F, Lindmaa A, von Lilienfeld OA, Armiento R (2015). Crystal structure representations for machine learning models of formation energies. International Journal of Quantum Chemistry.

[42] Ghiringhelli LM, Vybiral J, Levchenko SV, Draxl C, Scheffler M (2015). Big data of materials science: critical role of the descriptor. Physical review letters.

[43] Butler KT, Davies DW, Cartwright H, Isayev O, Walsh A (2018). Machine learning for molecular and materials science. Nature.

[44] LeCun Y, Bengio Y, Hinton G (2015). Deep learning. Nature.

[45] Lowe DG (2004). Distinctive image features from scale-invariant keypoints. International journal of computer vision.

[46] Winder, S. A. & Brown, M. Learning local image descriptors. In *Computer Vision and Pattern Recognition, 2007. CVPR'07. IEEE Conference on*, 1–8 (IEEE, 2007).

[47] Moreels P, Perona P (2007). Evaluation of features detectors and descriptors based on 3d objects. International Journal of Computer Vision.

[48] Krizhevsky, A., Sutskever, I. & Hinton, G. E. Imagenet classification with deep convolutional neural networks. In *Advances in neural information processing systems*, 1097–1105 (2012).

[49] Szegedy, C., Ioffe, S., Vanhoucke, V. & Alemi, A. A. Inception-v4, inception-resnet and the impact of residual connections on learning. In *AAAI*, vol. 4, 12 (2017).

[50] Deng, L. *et al*. Recent advances in deep learning for speech research at microsoft. In *Acoustics, Speech and Signal Processing (ICASSP), 2013 IEEE International Conference on*, 8604–8608 (IEEE, 2013).

[51] Mikolov, T., Deoras, A., Povey, D., Burget, L. & Černockỳ, J. Strategies for training large scale neural network language models. In *Automatic Speech Recognition and Understanding (ASRU), 2011 IEEE Workshop on*, 196–201 (IEEE, 2011).

[52] Sutskever, I., Vinyals, O. & Le, Q. V. Sequence to sequence learning with neural networks. In *Advances in neural information processing systems*, 3104–3112 (2014).

[53] Cecen A, Dai H, Yabansu YC, Kalidindi SR, Song L (2018). Material structure-property linkages using three-dimensional convolutional neural networks. Acta Materialia.

[54] Kondo R, Yamakawa S, Masuoka Y, Tajima S, Asahi R (2017). Microstructure recognition using convolutional neural networks for prediction of ionic conductivity in ceramics. Acta Materialia.

[55] Ling, J., Hutchinson, M., Antono, E. & Decost, B. Building Data-driven Models with Microstructural Images: Generalization and Interpretability 1–22. 1711.00404v1

[56] Wu Z (2018). Moleculenet: a benchmark for molecular machine learning. Chemical science.

[57] Schütt KT, Sauceda HE, Kindermans P-J, Tkatchenko A, Müller K-R (2018). Schnet–a deep learning architecture for molecules and materials. The Journal of Chemical Physics.

[58] Schütt KT, Arbabzadah F, Chmiela S, Müller KR, Tkatchenko A (2017). Quantum-chemical insights from deep tensor neural networks. Nature communications.

[59] Schmidt J (2017). Predicting the thermodynamic stability of solids combining density functional theory and machine learning. Chemistry of Materials.

[60] Deml AM, OHayre R, Wolverton C, Stevanovič V (2016). Predicting density functional theory total energies and enthalpies of formation of metal-nonmetal compounds by linear regression. Physical Review B.

[61] Seko A, Hayashi H, Kashima H, Tanaka I (2018). Matrix- and tensor-based recommender systems for the discovery of currently unknown inorganic compounds. Physical Review Materials.

[62] Open quantum materials database, http://oqmd.org/.

[63] Bergerhoff G, Hundt R, Sievers R, Brown I (1983). The inorganic crystal structure data base. Journal of chemical information and computer sciences.

[64] Andersson S, Collén B, Kuylenstierna U, Magnéli A (1957). Phase analysis studies on the titanium-oxygen system. Acta chem. scand.

[65] Walsh F, Wills R (2010). The continuing development of magnéli phase titanium sub-oxides and ebonex electrodes. Electrochimica Acta.

[66] Fedorov PP (1999). Systems of Alkali and Rare-Earth Metal Fluorides. Russ. J. Inorg. Chem..

[67] Peresypkina E, Blatov V (2003). Structure-forming components in crystals of ternary and quaternary 3d-metal complex fluorides. Acta Crystallographica Section B.

[68] Isherwood P (2017). Copper zinc oxide: Investigation into a p-type mixed metal oxide system. Vacuum.

[69] Benmokhtar S (2004). Synthesis, crystal structure and optical properties of BiMgVO5. Journal of Solid State Chemistry.

[70] Buisson G. (1970). Etude par rayons X et neutrons de la serie isomorphe ATiTO5 (A = Cr, Mn, Fe, T = Terres Rares). Journal of Physics and Chemistry of Solids.

[71] Agrawal, A., Meredig, B., Wolverton, C. & Choudhary, A. A formation energy predictor for crystalline materials using ensemble data mining. In *2015 IEEE International Conference on Data Mining Workshop (ICDMW) Demo* (IEEE, 2016).

[72] Tinto V (1975). Dropout from higher education: A theoretical synthesis of recent research. Review of educational research.

[73] Hawkins DM (2004). The problem of overfitting. Journal of chemical information and computer sciences.

[74] Nair, V. & Hinton, G. E. Rectified linear units improve restricted boltzmann machines. In *Proceedings of the 27th International Conference on Machine Learning (ICML-10)*, 807–814 (2010).

[75] Bengio, Y. Practical recommendations for gradient-based training of deep architectures. In *Neural Networks: Tricks of the Trade*, 437–478 (Springer, 2012).

[76] Sutskever I, Martens J, Dahl GE, Hinton GE (2013). On the importance of initialization and momentum in deep learning. ICML (3).

[77] Jacobs RA (1988). Increased rates of convergence through learning rate adaptation. Neural networks.

[78] Srivastava N, Hinton GE, Krizhevsky A, Sutskever I, Salakhutdinov R (2014). Dropout: a simple way to prevent neural networks from overfitting. Journal of Machine Learning Research.

[79] Bergstra, J. *et al*. Theano: A cpu and gpu math compiler in python. In *Proc. 9th Python in Science Conf*, 1–7 (2010).

[80] Abadi, M. *et al*. Tensorflow: Large-scale machine learning on heterogeneous distributed systems. *arXiv preprint arXiv:1603.04467* (2016).

[81] Pedregosa F (2011). Scikit-learn: Machine learning in Python. Journal of Machine Learning Research.

