# Supplementary material for: ElemNet: Deep Learning the Chemistry of Materials From Only Elemental Composition
Source: Sci Rep. 2018 Dec 4;8:17593. doi: 10.1038/s41598-018-35934-y (PMC6279928; doi:10.1038/s41598-018-35934-y)
Supplement: Supplementary file 1 — Potential Stable Systems [file 41598_2018_35934_MOESM1_ESM.pdf]

ElemNet: Deep Learning the Chemistry of Materials From Only Elemental Composition

Dipendra Jha, Logan Ward, Arindam Paul, Wei-keng Liao, Alok Choudhary, Chris Wolverton, and Ankit Agrawal

Supplementary Information

Potential Binary Stable Systems

system number_compounds most_stable_compound[composition, delta_e_predicted, stability_predicted]
AcAs 7 ['As0.4Ac0.6', '-1.127', '-0.217169455']
AcB 5 ['B0.9Ac0.1', '-0.286', '-0.286']
AcBi 8 ['Bi0.625Ac0.375', '-0.892', '-0.220809314063']
AcCl 1 ['Cl0.777778Ac0.222222', '-2.675', '-0.221363480142']
AcF 12 ['F0.625Ac0.375', '-3.312', '-0.420116056425']
AcGa 6 ['Ga0.8Ac0.2', '-0.38', '-0.2058094935']
AcI 3 ['I0.714286Ac0.285714', '-1.495', '-0.208267052549']
AcIr 2 ['Ir0.428571Ac0.571429', '-0.575', '-0.207853881071']
AcN 6 ['N0.428571Ac0.571429', '-0.71', '-0.203450161638']
AcO 4 ['O0.7Ac0.3', '-3.285', '-0.600176867603']
AcP 8 ['P0.625Ac0.375', '-1.137', '-0.215789540078']
AcPt 5 ['Pt0.5Ac0.5', '-1.25', '-0.24321190125']
AcS 1 ['S0.428571Ac0.571429', '-2.077', '-0.229626223634']
AcSb 5 ['Sb0.571429Ac0.428571', '-1.067', '-0.211612255357']
AcSe 7 ['Se0.5Ac0.5', '-1.87', '-0.23109862125']
AcSi 3 ['Si0.625Ac0.375', '-0.453', '-0.210862015313']
AcTe 7 ['Te0.714286Ac0.285714', '-1.082', '-0.228984476904']
AgF 7 ['F0.777778Ag0.222222', '-1.248', '-0.202938081815']
AlF 7 ['F0.777778Al0.222222', '-3.678', '-0.277011793911']
AlIr 4 ['Al0.555556Ir0.444444', '-1.108', '-0.224992043889']
AlRh 3 ['Al0.6Rh0.4', '-1.152', '-0.2187075015']
AsBr 4 ['As0.2Br0.8', '-0.757', '-0.21190409355']
AsCe 2 ['As0.571429Ce0.428571', '-1.494', '-0.220247741786']
AsF 4 ['F0.857143As0.142857', '-2.18', '-0.226418997046']
AsK 4 ['K0.571429As0.428571', '-0.691', '-0.264803383571']
AsLa 1 ['As0.6La0.4', '-1.477', '-0.211956029998']
AsNd 1 ['As0.444444Nd0.555556', '-1.595', '-0.249426537778']
AsPm 9 ['As0.625Pm0.375', '-1.057', '-0.247760332187']
AsRb 5 ['As0.3Rb0.7', '-0.51', '-0.211494555375']
AsRe 1 ['As0.625Re0.375', '-0.289', '-0.225044996429']
AuCe 6 ['Ce0.2Au0.8', '-0.516', '-0.2037920545']

AuF 3 ['F0.875Au0.125', '-1.01', '-0.25138904867']
AuLa 2 ['La0.375Au0.625', '-0.878', '-0.209906405']
AuPa 10 ['Au0.888889Pa0.111111', '-0.267', '-0.204788864444']
AuPm 14 ['Pm0.142857Au0.857143', '-0.428', '-0.2022159025']
AuPu 3 ['Au0.6Pu0.4', '-0.528', '-0.201708432']
BF 9 ['B0.3F0.7', '-3.076', '-0.282125462317']
BI 11 ['B0.1I0.9', '-0.283', '-0.208608065772']
BPa 11 ['B0.833333Pa0.166667', '-0.207', '-0.207']
BPm 8 ['B0.75Pm0.25', '-0.207', '-0.207']
BRb 1 ['B0.9Rb0.1', '-0.22', '-0.22']
BTa 2 ['B0.625Ta0.375', '-0.949', '-0.246639295625']
BaF 9 ['F0.714286Ba0.285714', '-3.812', '-0.268240346214']
BaSe 1 ['Se0.555556Ba0.444444', '-2.045', '-0.21233450111']
BaTe 1 ['Te0.6Ba0.4', '-1.667', '-0.234290190664']
BeF 10 ['Be0.285714F0.714286', '-3.21', '-0.228679629069']
BeOs 8 ['Be0.8Os0.2', '-0.254', '-0.238657066']
BeSm 1 ['Be0.9Sm0.1', '-0.218', '-0.218']
BeTc 5 ['Be0.857143Tc0.142857', '-0.201', '-0.201']
BiCe 3 ['Ce0.444444Bi0.555556', '-1.1', '-0.222151492222']
BiF 4 ['F0.857143Bi0.142857', '-1.975', '-0.219314437051']
BiLa 5 ['La0.444444Bi0.555556', '-1.181', '-0.2341409']
BiPm 4 ['Pm0.375Bi0.625', '-0.876', '-0.245062975312']
BiPr 4 ['Pr0.444444Bi0.555556', '-1.087', '-0.215587358889']
BiPu 2 ['Bi0.666667Pu0.333333', '-0.465', '-0.237375198333']
BrCr 4 ['Cr0.285714Br0.714286', '-0.934', '-0.205978435762']
BrDy 7 ['Br0.857143Dy0.142857', '-1.079', '-0.364333584787']
BrEr 15 ['Br0.9Er0.1', '-0.57', '-0.358770505258']
BrEu 1 ['Br0.625Eu0.375', '-2.507', '-0.234813301306']
BrF 2 ['F0.888889Br0.111111', '-1.099', '-0.259216856327']
BrHf 15 ['Br0.555556Hf0.444444', '-0.634', '-0.350657070037']
BrHo 12 ['Br0.857143Ho0.142857', '-1.02', '-0.317613594914']
BrLa 1 ['Br0.8La0.2', '-2.053', '-0.246432618552']
BrLu 15 ['Br0.555556Lu0.444444', '-1.119', '-0.255095074482']
BrMo 3 ['Br0.833333Mo0.166667', '-0.786', '-0.254706810459']
BrNp 5 ['Br0.888889Np0.111111', '-0.915', '-0.209525816']
BrPm 15 ['Br0.9Pm0.1', '-0.672', '-0.416304712008']
BrPt 5 ['Br0.8Pt0.2', '-0.264', '-0.264']
BrPu 3 ['Br0.777778Pu0.222222', '-1.811', '-0.233584772836']
BrRe 4 ['Br0.888889Re0.111111', '-0.258', '-0.258']
BrSb 2 ['Br0.857143Sb0.142857', '-0.681', '-0.205011909322']
BrSc 15 ['Sc0.1Br0.9', '-0.483', '-0.300898819758']
BrTa 5 ['Br0.9Ta0.1', '-0.616', '-0.248359756353']
BrTb 8 ['Br0.666667Tb0.333333', '-1.551', '-0.227744943978']
BrTe 8 ['Br0.888889Te0.111111', '-0.246', '-0.201022800468']

BrTm 12 ['Br0.857143Tm0.142857', '-0.992', '-0.205306225394']
BrV 2 ['V0.2Br0.8', '-0.818', '-0.204695091032']
BrY 13 ['Br0.875Y0.125', '-0.85', '-0.211126366595']
BrYb 4 ['Br0.625Yb0.375', '-2.498', '-0.322389592241']
BrZr 3 ['Br0.857143Zr0.142857', '-1.221', '-0.292068488966']
CF 3 ['C0.125F0.875', '-1.505', '-0.227532912']
CRe 1 ['C0.444444Re0.555556', '-0.26', '-0.235178529861']
CaF 6 ['F0.8Ca0.2', '-2.739', '-0.207355998348']
CaH 2 ['H0.555556Ca0.444444', '-0.695', '-0.21920021337']
CaS 1 ['S0.428571Ca0.571429', '-2.194', '-0.210982936849']
CdF 7 ['F0.777778Cd0.222222', '-1.858', '-0.22082053372']
CeF 5 ['F0.857143Ce0.142857', '-2.72', '-0.221741944063']
CeI 10 ['I0.888889Ce0.111111', '-0.525', '-0.203612406539']
CeOs 1 ['Ce0.666667Os0.333333', '-0.256', '-0.218622531111']
ClCs 1 ['Cl0.5Cs0.5', '-2.367', '-0.21313108219']
ClCu 1 ['Cl0.555556Cu0.444444', '-0.82', '-0.200774913545']
ClEr 15 ['Cl0.9Er0.1', '-0.674', '-0.409119921438']
ClEu 3 ['Cl0.6Eu0.4', '-2.756', '-0.216612510878']
ClF 5 ['F0.833333Cl0.166667', '-0.878', '-0.240250194498']
ClGd 1 ['Cl0.777778Gd0.222222', '-2.478', '-0.229786952364']
ClHo 15 ['Cl0.9Ho0.1', '-0.669', '-0.398246238104']
ClIn 2 ['Cl0.777778In0.222222', '-0.977', '-0.225571602865']
ClIr 1 ['Cl0.666667Ir0.333333', '-0.21', '-0.21']
ClLu 15 ['Cl0.9Lu0.1', '-0.665', '-0.416831698938']
ClNp 2 ['Cl0.857143Np0.142857', '-1.598', '-0.260647920536']
ClPm 11 ['Cl0.833333Pm0.166667', '-1.631', '-0.221521270107']
ClPt 3 ['Cl0.777778Pt0.222222', '-0.609', '-0.205959653799']
ClPu 3 ['Cl0.857143Pu0.142857', '-1.512', '-0.268904614023']
ClRh 9 ['Cl0.833333Rh0.166667', '-0.254', '-0.254']
ClS 3 ['S0.125Cl0.875', '-0.372', '-0.215901092781']
ClSm 5 ['Cl0.714286Sm0.285714', '-2.291', '-0.302726052059']
ClTa 1 ['Cl0.833333Ta0.166667', '-1.63', '-0.31100816125']
ClTh 1 ['Cl0.833333Th0.166667', '-2.276', '-0.23517425875']
ClXe 2 ['Cl0.6Xe0.4', '-0.207', '-0.207']
CoF 9 ['F0.666667Co0.333333', '-1.964', '-0.233241455396']
CrF 4 ['F0.857143Cr0.142857', '-2.316', '-0.236138288837']
CsF 14 ['F0.555556Cs0.444444', '-2.778', '-0.242044734369']
CsO 3 ['O0.333333Cs0.666667', '-1.311', '-0.20080594137']
CsSe 2 ['Se0.4Cs0.6', '-1.165', '-0.2017893095']
CuF 8 ['F0.75Cu0.25', '-1.627', '-0.329242594187']
CuHf 1 ['Cu0.5Hf0.5', '-0.329', '-0.208247785']
CuO 1 ['O0.333333Cu0.666667', '-0.886', '-0.201575631916']
DyF 7 ['F0.857143Dy0.142857', '-2.819', '-0.46006329228']
DyI 11 ['I0.9Dy0.1', '-0.457', '-0.218674180969']

DyN 2 ['N0.444444Dy0.555556', '-1.845', '-0.237387999102']
DyPt 2 ['Dy0.111111Pt0.888889', '-0.683', '-0.229685177036']
ErF 7 ['F0.777778Er0.222222', '-4.172', '-0.223798714471']
ErI 12 ['I0.625Er0.375', '-1.075', '-0.209282051678']
ErN 2 ['N0.428571Er0.571429', '-1.843', '-0.226116251997']
ErOs 1 ['Er0.666667Os0.333333', '-0.523', '-0.25974886']
EuF 5 ['F0.833333Eu0.166667', '-2.53', '-0.246811071955']
EuIr 1 ['Eu0.7Ir0.3', '-0.638', '-0.202826325857']
EuN 5 ['N0.444444Eu0.555556', '-0.859', '-0.344874970958']
EuP 6 ['P0.5Eu0.5', '-1.358', '-0.240197211774']
EuSe 4 ['Se0.444444Eu0.555556', '-2.257', '-0.295324982222']
EuSi 1 ['Si0.6Eu0.4', '-0.811', '-0.247471581']
EuSn 2 ['Sn0.333333Eu0.666667', '-0.795', '-0.233251086049']
FFe 7 ['F0.777778Fe0.222222', '-2.379', '-0.252187313911']
FGa 6 ['F0.8Ga0.2', '-2.572', '-0.27421910952']
FGd 7 ['F0.8Gd0.2', '-3.723', '-0.398740231024']
FGe 5 ['F0.833333Ge0.166667', '-2.411', '-0.262127398083']
FH 9 ['H0.375F0.625', '-1.421', '-0.20274185888']
FHf 4 ['F0.888889Hf0.111111', '-2.625', '-0.349529787611']
FHg 8 ['F0.75Hg0.25', '-1.454', '-0.212527651693']
FHo 6 ['F0.8Ho0.2', '-3.801', '-0.247143427856']
FI 2 ['F0.8I0.2', '-1.844', '-0.218253265708']
FIn 7 ['F0.777778In0.222222', '-2.75', '-0.281070977244']
FIr 3 ['F0.875Ir0.125', '-1.71', '-0.222406349712']
FK 15 ['F0.555556K0.444444', '-2.861', '-0.271649331493']
FKr 7 ['F0.777778Kr0.222222', '-0.479', '-0.271089659275']
FLa 6 ['F0.8La0.2', '-3.908', '-0.324613149856']
FLi 10 ['Li0.3F0.7', '-2.113', '-0.223073944258']
FLu 7 ['F0.777778Lu0.222222', '-4.12', '-0.523347188356']
FMg 7 ['F0.777778Mg0.222222', '-2.862', '-0.31321753168']
FMn 4 ['F0.857143Mn0.142857', '-2.069', '-0.286217596564']
FMo 4 ['F0.875Mo0.125', '-2.56', '-0.201358337525']
FN 6 ['N0.2F0.8', '-0.314', '-0.314']
FNa 14 ['F0.555556Na0.444444', '-2.854', '-0.21044783112']
FNb 4 ['F0.857143Nb0.142857', '-3.011', '-0.220900610617']
FNd 6 ['F0.833333Nd0.166667', '-3.181', '-0.253012047653']
FNi 7 ['F0.777778Ni0.222222', '-1.826', '-0.212329748356']
FNp 5 ['F0.888889Np0.111111', '-2.749', '-0.653145720339']
FO 7 ['O0.222222F0.777778', '-0.255', '-0.255']
FOs 3 ['F0.875Os0.125', '-1.944', '-0.233802501585']
FP 4 ['F0.857143P0.142857', '-2.707', '-0.261640615051']
FPa 7 ['F0.777778Pa0.222222', '-3.939', '-0.741332953911']
FPb 4 ['F0.833333Pb0.166667', '-1.99', '-0.217553495583']
FPd 5 ['F0.833333Pd0.166667', '-1.487', '-0.303492123917']

FPm 7 ['F0.777778Pm0.222222', '-3.954', '-0.247989586693']
FPr 6 ['F0.8Pr0.2', '-3.817', '-0.310068586024']
FPt 5 ['F0.857143Pt0.142857', '-1.533', '-0.22481486824']
FPu 7 ['F0.777778Pu0.222222', '-4.017', '-0.547741543911']
FRb 15 ['F0.625Rb0.375', '-2.404', '-0.260963759072']
FRh 7 ['F0.777778Rh0.222222', '-1.808', '-0.212997511689']
FRu 5 ['F0.833333Ru0.166667', '-2.008', '-0.41893120225']
FS 1 ['F0.9S0.1', '-1.641', '-0.218522612123']
FSb 6 ['F0.8Sb0.2', '-2.58', '-0.225108226104']
FSc 6 ['F0.8Sc0.2', '-3.863', '-0.44713311152']
FSe 4 ['F0.857143Se0.142857', '-1.747', '-0.342419581929']
FSi 5 ['F0.833333Si0.166667', '-3.022', '-0.234490001417']
FSm 5 ['F0.833333Sm0.166667', '-3.198', '-0.250022343353']
FSn 5 ['F0.833333Sn0.166667', '-2.436', '-0.219756717767']
FSr 5 ['F0.8Sr0.2', '-2.745', '-0.221407406346']
FTa 2 ['F0.9Ta0.1', '-2.365', '-0.283386386434']
FTb 3 ['F0.875Tb0.125', '-2.361', '-0.22071222943']
FTc 5 ['F0.833333Tc0.166667', '-2.386', '-0.523737951625']
FTe 2 ['F0.888889Te0.111111', '-1.979', '-0.221153715766']
FTh 5 ['F0.833333Th0.166667', '-4.103', '-0.386022305858']
FTi 7 ['F0.777778Ti0.222222', '-3.7', '-0.492554519742']
FTl 6 ['F0.8Tl0.2', '-1.872', '-0.22868317352']
FTm 16 ['F0.5Tm0.5', '-2.542', '-0.36285658043']
FU 1 ['F0.9U0.1', '-2.711', '-0.222897395523']
FV 5 ['F0.833333V0.166667', '-2.956', '-0.45808371975']
FW 1 ['F0.9W0.1', '-2.271', '-0.224404256024']
FXe 4 ['F0.857143Xe0.142857', '-0.763', '-0.224589603354']
FY 7 ['F0.8Y0.2', '-3.779', '-0.420508316184']
FYb 14 ['F0.444444Yb0.555556', '-2.634', '-0.211525853642']
FZn 7 ['F0.777778Zn0.222222', '-1.921', '-0.209590698167']
FZr 4 ['F0.833333Zr0.166667', '-3.532', '-0.231198673708']
GaPa 7 ['Ga0.625Pa0.375', '-0.401', '-0.208014351875']
GaPm 7 ['Ga0.666667Pm0.333333', '-0.58', '-0.201737114167']
GdI 11 ['I0.625Gd0.375', '-1.173', '-0.219403936444']
GdN 1 ['N0.444444Gd0.555556', '-1.745', '-0.218582207627']
GdO 2 ['O0.666667Gd0.333333', '-3.384', '-0.223476952892']
GdP 1 ['P0.428571Gd0.571429', '-1.705', '-0.220188290089']
GdPt 3 ['Gd0.125Pt0.875', '-0.709', '-0.220129387812']
GeHo 1 ['Ge0.428571Ho0.571429', '-1.052', '-0.208841014381']
GePa 9 ['Ge0.555556Pa0.444444', '-0.495', '-0.211227202222']
GePm 4 ['Ge0.444444Pm0.555556', '-0.774', '-0.229407192222']
GeSc 1 ['Sc0.6Ge0.4', '-1.052', '-0.210229152']
GeU 2 ['Ge0.625U0.375', '-0.47', '-0.202891']
HLi 1 ['H0.555556Li0.444444', '-0.584', '-0.220039585696']

HPm 7 ['H0.714286Pm0.285714', '-0.596', '-0.241929693083']
HfN 2 ['N0.4Hf0.6', '-1.691', '-0.207336819864']
HfPt 1 ['Hf0.166667Pt0.833333', '-0.944', '-0.2119386175']
HfRe 2 ['Hf0.6Re0.4', '-0.446', '-0.20138496']
HfS 3 ['S0.4Hf0.6', '-1.485', '-0.228913883398']
HfZn 3 ['Zn0.4Hf0.6', '-0.388', '-0.204905913']
HoI 11 ['I0.9Ho0.1', '-0.452', '-0.221230014053']
HoN 2 ['N0.428571Ho0.571429', '-1.814', '-0.229602513423']
HoOs 3 ['Ho0.666667Os0.333333', '-0.282', '-0.240860868889']
HoPt 5 ['Ho0.111111Pt0.888889', '-0.664', '-0.202292037409']
HoTe 3 ['Te0.625Ho0.375', '-1.357', '-0.200234669165']
ILu 15 ['I0.9Lu0.1', '-0.395', '-0.283882753257']
INd 5 ['I0.833333Nd0.166667', '-0.958', '-0.247809578355']
INp 2 ['I0.857143Np0.142857', '-0.778', '-0.202081277709']
IPa 15 ['I0.571429Pa0.428571', '-0.456', '-0.337132528959']
IPm 15 ['I0.555556Pm0.444444', '-1.053', '-0.257779113365']
IPu 2 ['I0.8Pu0.2', '-1.192', '-0.225653426544']
IRh 2 ['Rh0.285714I0.714286', '-0.211', '-0.211']
ISc 1 ['Sc0.25I0.75', '-1.15', '-0.226596255032']
ISm 7 ['I0.857143Sm0.142857', '-0.755', '-0.209483683602']
ITb 15 ['I0.9Tb0.1', '-0.413', '-0.328299501291']
ITm 7 ['I0.7Tm0.3', '-1.329', '-0.202190281539']
IXe 9 ['I0.375Xe0.625', '-0.204', '-0.204']
InPm 4 ['In0.8Pm0.2', '-0.411', '-0.2081324715']
IrNd 2 ['Nd0.555556Ir0.444444', '-0.708', '-0.219133931111']
IrPa 7 ['Ir0.444444Pa0.555556', '-0.773', '-0.218798813333']
IrSm 1 ['Sm0.333333Ir0.666667', '-0.647', '-0.219452480833']
IrTm 1 ['Tm0.571429Ir0.428571', '-1.025', '-0.235210472857']
KrP 2 ['P0.5Kr0.5', '-0.207', '-0.207']
LaN 4 ['N0.375La0.625', '-1.345', '-0.275166664247']
LaO 2 ['O0.666667La0.333333', '-3.327', '-0.2332182181']
LaPd 2 ['Pd0.6La0.4', '-1.05', '-0.2252179935']
LaSb 3 ['Sb0.625La0.375', '-1.16', '-0.203146780625']
LaSe 2 ['Se0.444444La0.555556', '-1.987', '-0.209314542222']
LiPt 2 ['Li0.375Pt0.625', '-0.721', '-0.221968615417']
LuN 1 ['N0.444444Lu0.555556', '-1.979', '-0.215840377627']
LuOs 2 ['Lu0.625Os0.375', '-0.423', '-0.20114569125']
LuTe 4 ['Te0.555556Lu0.444444', '-1.316', '-0.251870549813']
MoN 1 ['N0.4Mo0.6', '-0.613', '-0.236318214862']
NNp 4 ['N0.555556Np0.444444', '-1.339', '-0.261260255956']
NPa 8 ['N0.428571Pa0.571429', '-1.415', '-0.285920924629']
NPu 3 ['N0.6Pu0.4', '-1.307', '-0.211702385864']
NTi 5 ['N0.444444Ti0.555556', '-1.648', '-0.206847975546']
NTm 3 ['N0.4Tm0.6', '-1.75', '-0.206536901864']

NbRh 1 ['Nb0.7Rh0.3', '-0.502', '-0.20525105']
NbRu 1 ['Nb0.666667Ru0.333333', '-0.337', '-0.21685651']
NbSn 5 ['Nb0.666667Sn0.333333', '-0.454', '-0.258267673383']
NdPt 1 ['Nd0.6Pt0.4', '-1.13', '-0.210909055']
NpPt 9 ['Pt0.625Np0.375', '-0.541', '-0.200020017344']
OPa 4 ['O0.666667Pa0.333333', '-3.31', '-0.21119982108']
OPu 1 ['O0.7Pu0.3', '-3.162', '-0.22724688999']
OSm 2 ['O0.571429Sm0.428571', '-3.726', '-0.201208824019']
OTe 1 ['O0.714286Te0.285714', '-1.431', '-0.22329346413']
OU 1 ['O0.714286U0.285714', '-3.756', '-0.306209161684']
OsSc 4 ['Sc0.625Os0.375', '-0.449', '-0.209482371875']
OsTm 3 ['Tm0.666667Os0.333333', '-0.307', '-0.217406496667']
OsV 8 ['V0.714286Os0.285714', '-0.34', '-0.210473245714']
PPb 1 ['P0.5Pb0.5', '-0.235', '-0.235']
PPm 8 ['P0.6Pm0.4', '-1.211', '-0.257585395416']
PSr 1 ['P0.4Sr0.6', '-1.16', '-0.21891435308']
PTa 8 ['P0.4Ta0.6', '-0.974', '-0.213294630916']
PXe 3 ['P0.75Xe0.25', '-0.218', '-0.218']
PaPt 3 ['Pt0.857143Pa0.142857', '-0.754', '-0.202827655714']
PaRh 6 ['Rh0.6Pa0.4', '-0.938', '-0.218240498']
PaS 4 ['S0.75Pa0.25', '-1.225', '-0.203079604119']
PaSe 8 ['Se0.75Pa0.25', '-0.853', '-0.2509873225']
PaSi 7 ['Si0.6Pa0.4', '-0.45', '-0.203489202']
PdPm 13 ['Pd0.6Pm0.4', '-0.846', '-0.222699971']
PdV 1 ['V0.666667Pd0.333333', '-0.36', '-0.204337431667']
PdZn 2 ['Zn0.375Pd0.625', '-0.633', '-0.204188115']
PmS 5 ['S0.428571Pm0.571429', '-1.951', '-0.347843574349']
PmSb 11 ['Sb0.666667Pm0.333333', '-0.823', '-0.204526854167']
PmSe 6 ['Se0.7Pm0.3', '-1.22', '-0.24741187925']
PmSi 5 ['Si0.333333Pm0.666667', '-0.504', '-0.211573580833']
PmSn 6 ['Sn0.375Pm0.625', '-0.681', '-0.200948928993']
PmTe 4 ['Te0.555556Pm0.444444', '-1.501', '-0.340949068516']
PrPt 2 ['Pr0.555556Pt0.444444', '-1.208', '-0.213905538889']
PtPu 1 ['Pt0.444444Pu0.555556', '-0.917', '-0.201515871111']
PtRe 5 ['Re0.714286Pt0.285714', '-0.209', '-0.209']
PtSm 3 ['Sm0.142857Pt0.857143', '-0.725', '-0.204356066071']
PtSr 3 ['Sr0.625Pt0.375', '-0.757', '-0.2156358625']
PtTb 2 ['Tb0.142857Pt0.857143', '-0.693', '-0.214450533619']
PtTc 7 ['Tc0.833333Pt0.166667', '-0.222', '-0.206959760833']
PtTh 13 ['Pt0.6Th0.4', '-1.197', '-0.219936314']
PtTm 2 ['Tm0.111111Pt0.888889', '-0.681', '-0.204353752222']
PtW 3 ['W0.142857Pt0.857143', '-0.234', '-0.206502958571']
PtYb 1 ['Yb0.111111Pt0.888889', '-0.579', '-0.214482274443']
PtZr 1 ['Zr0.142857Pt0.857143', '-0.808', '-0.225127234286']

PuTe 5 ['Te0.5Pu0.5', '-1.371', '-0.26623655833']
PuTl 3 ['Tl0.6Pu0.4', '-0.237', '-0.212142938']
RbSb 3 ['Rb0.7Sb0.3', '-0.58', '-0.201491821875']
RbSe 1 ['Se0.4Rb0.6', '-1.27', '-0.254121775998']
RhTc 1 ['Tc0.7Rh0.3', '-0.221', '-0.2023898315']
RhY 1 ['Y0.6Rh0.4', '-0.877', '-0.202946982']
RuV 4 ['V0.666667Ru0.333333', '-0.364', '-0.213871236667']
STc 1 ['S0.571429Tc0.428571', '-0.901', '-0.209186500564']
SXe 1 ['S0.5Xe0.5', '-0.263', '-0.263']
SbTm 1 ['Sb0.375Tm0.625', '-1.066', '-0.205759240625']
ScSe 4 ['Sc0.375Se0.625', '-1.455', '-0.259491185625']
SeTc 4 ['Se0.7Tc0.3', '-0.216', '-0.216']
SeTm 3 ['Se0.625Tm0.375', '-1.758', '-0.209553996253']
SeY 5 ['Se0.555556Y0.444444', '-1.888', '-0.31831002']
SeYb 1 ['Se0.444444Yb0.555556', '-2.082', '-0.209858333333']
SeZr 4 ['Se0.428571Zr0.571429', '-1.265', '-0.215937565516']
TbTe 3 ['Te0.555556Tb0.444444', '-1.259', '-0.28744628163']
TeTm 4 ['Te0.625Tm0.375', '-1.299', '-0.202629491667']
TeY 4 ['Y0.444444Te0.555556', '-1.474', '-0.213010723147']

Potential Ternary Stable Systems

system number_compounds most_stable_compound[composition, delta_e_predicted, stability_predicted]
AcAgBr 3 ['Br0.75Ag0.125Ac0.125', '-1.52', '-0.208218889264']
AcAgF 30 ['F0.4Ag0.4Ac0.2', '-2.006', '-0.207927151347']
AcAgGa 3 ['Ga0.666667Ag0.166667Ac0.166667', '-0.359', '-0.201367792361']
AcAgI 29 ['Ag0.142857I0.714286Ac0.142857', '-1.07', '-0.204528007896']
AcAgN 15 ['N0.285714Ag0.285714Ac0.428571', '-0.62', '-0.204572279902']
AcAgO 8 ['O0.666667Ag0.222222Ac0.111111', '-1.396', '-0.24621140226']
AcAgP 4 ['P0.5Ag0.166667Ac0.333333', '-1.077', '-0.210362364952']
AcAgSe 26 ['Se0.333333Ag0.333333Ac0.333333', '-1.295', '-0.202399080833']
AcAgTe 12 ['Ag0.142857Te0.714286Ac0.142857', '-0.656', '-0.206051485119']
AcAlBr 3 ['Al0.111111Br0.777778Ac0.111111', '-1.853', '-0.216672424498']
AcAlF 28 ['F0.571429Al0.285714Ac0.142857', '-3.223', '-0.231878587757']
AcAlGa 1 ['Al0.111111Ga0.666667Ac0.222222', '-0.416', '-0.200015166481']
AcAlH 7 ['H0.666667Al0.111111Ac0.222222', '-0.588', '-0.206189357248']
AcAlI 21 ['Al0.125I0.75Ac0.125', '-1.268', '-0.20453036224']
AcAlN 18 ['N0.5Al0.333333Ac0.166667', '-1.436', '-0.232432701079']
AcAlO 8 ['O0.6Al0.3Ac0.1', '-3.718', '-0.31940487372']
AcAlP 10 ['Al0.2P0.5Ac0.3', '-1.233', '-0.20132147627']
AcAlPt 1 ['Al0.125Pt0.375Ac0.5', '-1.018', '-0.2013744325']
AcAlS 16 ['Al0.125S0.375Ac0.5', '-1.884', '-0.206013452243']
AcAlSe 29 ['Al0.333333Se0.333333Ac0.333333', '-1.297', '-0.204399080833']
AcAlTe 6 ['Al0.1Te0.7Ac0.2', '-0.923', '-0.206626318604']
AcAsBi 5 ['As0.5Bi0.1Ac0.4', '-1.127', '-0.217169455']
AcAsBr 42 ['As0.1Br0.6Ac0.3', '-2.314', '-0.220162769798']
AcAsC 13 ['C0.3As0.3Ac0.4', '-0.901', '-0.21862709125']
AcAsCd 1 ['As0.444444Cd0.111111Ac0.444444', '-1.214', '-0.203077172222']
AcAsCl 35 ['Cl0.3As0.2Ac0.5', '-1.762', '-0.202948293564']
AcAsCu 1 ['Cu0.1As0.4Ac0.5', '-1.169', '-0.24151476625']
AcAsF 72 ['F0.444444As0.111111Ac0.444444', '-2.764', '-0.226323954922']
AcAsH 22 ['H0.4As0.4Ac0.2', '-0.658', '-0.2030847275']
AcAsI 58 ['As0.166667I0.333333Ac0.5', '-1.267', '-0.20535815961']
AcAsIn 1 ['As0.4In0.1Ac0.5', '-1.213', '-0.21715164125']
AcAsLi 2 ['Li0.142857As0.428571Ac0.428571', '-1.222', '-0.218340387857']
AcAsN 30 ['N0.1As0.5Ac0.4', '-1.148', '-0.238169455']
AcAsNa 5 ['Na0.125As0.5Ac0.375', '-1.183', '-0.217513051612']
AcAsO 25 ['O0.3As0.2Ac0.5', '-2.451', '-0.206202639235']
AcAsP 10 ['P0.4As0.3Ac0.3', '-0.943', '-0.206031632062']
AcAsPb 3 ['As0.5Pb0.125Ac0.375', '-1.058', '-0.205033864062']
AcAsPm 1 ['As0.428571Pm0.428571Ac0.142857', '-1.15', '-0.208496963214']
AcAsRh 1 ['As0.4Rh0.1Ac0.5', '-1.24', '-0.22384438025']
AcAsS 27 ['S0.1As0.5Ac0.4', '-1.344', '-0.201434316765']

AcAsSb 1 ['As0.4Sb0.1Ac0.5', '-1.318', '-0.21488629875']
AcAsSe 59 ['As0.555556Se0.111111Ac0.333333', '-1.073', '-0.203338279722']
AcAsTe 8 ['As0.222222Te0.333333Ac0.444444', '-1.411', '-0.229260462776']
AcAuBi 8 ['Au0.5Bi0.1Ac0.4', '-0.875', '-0.2003748065']
AcAuBr 19 ['Br0.5Au0.333333Ac0.166667', '-1.756', '-0.200683671707']
AcAuCd 1 ['Cd0.125Au0.75Ac0.125', '-0.524', '-0.201534119688']
AcAuCl 3 ['Cl0.625Au0.25Ac0.125', '-1.769', '-0.20639994055']
AcAuEu 1 ['Eu0.25Au0.625Ac0.125', '-0.981', '-0.219759334063']
AcAuF 23 ['F0.555556Au0.111111Ac0.333333', '-2.95', '-0.288003524023']
AcAuGa 1 ['Ga0.7Au0.1Ac0.2', '-0.455', '-0.2109206225']
AcAuGe 6 ['Ge0.25Au0.375Ac0.375', '-0.8', '-0.215932764688']
AcAuH 21 ['H0.666667Au0.166667Ac0.166667', '-0.508', '-0.206500882709']
AcAuI 49 ['I0.285714Au0.571429Ac0.142857', '-0.815', '-0.2120624849']
AcAuLa 1 ['La0.222222Au0.666667Ac0.111111', '-0.856', '-0.243463114167']
AcAuN 18 ['N0.2Au0.4Ac0.4', '-0.838', '-0.214994949']
AcAuNp 1 ['Au0.8Ac0.1Np0.1', '-0.429', '-0.223662544375']
AcAuO 8 ['O0.666667Au0.222222Ac0.111111', '-1.406', '-0.21367576067']
AcAuP 26 ['P0.333333Au0.333333Ac0.333333', '-1.03', '-0.211146257847']
AcAuPa 2 ['Au0.8Ac0.1Pa0.1', '-0.461', '-0.20328279125']
AcAuPb 9 ['Au0.4Pb0.2Ac0.4', '-0.847', '-0.203498578333']
AcAuS 1 ['S0.428571Au0.142857Ac0.428571', '-2.091', '-0.214251828159']
AcAuSe 51 ['Se0.111111Au0.555556Ac0.333333', '-0.99', '-0.203059236389']
AcAuSn 9 ['Sn0.1Au0.6Ac0.3', '-0.771', '-0.203001315732']
AcAuTe 3 ['Te0.555556Au0.111111Ac0.333333', '-1.24', '-0.246941128982']
AcAuTl 3 ['Au0.5Tl0.166667Ac0.333333', '-0.773', '-0.215515854167']
AcBBr 3 ['B0.111111Br0.777778Ac0.111111', '-1.594', '-0.219020020332']
AcBC 2 ['B0.142857C0.571429Ac0.285714', '-0.233', '-0.220902322582']
AcBCl 2 ['B0.2Cl0.7Ac0.1', '-1.991', '-0.222441949819']
AcBCo 2 ['B0.777778Co0.111111Ac0.111111', '-0.322', '-0.230870331667']
AcBCr 1 ['B0.8Cr0.1Ac0.1', '-0.366', '-0.21541387']
AcBF 19 ['B0.166667F0.5Ac0.333333', '-2.783', '-0.2124364946']
AcBFe 2 ['B0.777778Fe0.111111Ac0.111111', '-0.351', '-0.267310833889']
AcBH 14 ['H0.625B0.25Ac0.125', '-0.461', '-0.201356323494']
AcBI 11 ['B0.1I0.6Ac0.3', '-1.333', '-0.227523202792']
AcBLi 2 ['Li0.111111B0.777778Ac0.111111', '-0.31', '-0.203350519722']
AcBMg 1 ['B0.8Mg0.1Ac0.1', '-0.348', '-0.239578864334']
AcBMn 1 ['B0.8Mn0.1Ac0.1', '-0.345', '-0.209893463621']
AcBMo 1 ['B0.8Mo0.1Ac0.1', '-0.373', '-0.242614528']
AcBN 8 ['B0.1N0.3Ac0.6', '-0.72', '-0.209121120397']
AcBNi 3 ['B0.75Ni0.125Ac0.125', '-0.268', '-0.206008367266']
AcBO 2 ['B0.111111O0.666667Ac0.222222', '-3.18', '-0.462653810947']
AcBP 5 ['B0.111111P0.333333Ac0.555556', '-1.023', '-0.204146257847']
AcBPa 2 ['B0.777778Ac0.111111Pa0.111111', '-0.275', '-0.275']
AcBPm 2 ['B0.777778Pm0.111111Ac0.111111', '-0.251', '-0.251']

AcBRu 1 ['B0.8Ru0.1Ac0.1', '-0.287', '-0.203876465']
AcBSe 4 ['B0.166667Se0.5Ac0.333333', '-1.347', '-0.201941123333']
AcBaCl 1 ['Cl0.75Ba0.125Ac0.125', '-2.704', '-0.272047842424']
AcBaF 9 ['F0.666667Ba0.222222Ac0.111111', '-3.98', '-0.3668879897']
AcBaN 9 ['N0.428571Ba0.285714Ac0.285714', '-0.742', '-0.20462298271']
AcBaO 8 ['O0.666667Ba0.222222Ac0.111111', '-2.623', '-0.216312075015']
AcBaP 1 ['P0.5Ba0.1Ac0.4', '-1.435', '-0.230677223988']
AcBaPt 4 ['Ba0.111111Pt0.444444Ac0.444444', '-1.098', '-0.203077245556']
AcBaSe 16 ['Se0.5Ba0.2Ac0.3', '-2.005', '-0.20074828675']
AcBaTe 13 ['Te0.571429Ba0.285714Ac0.142857', '-1.644', '-0.203581795593']
AcBeBr 1 ['Be0.125Br0.75Ac0.125', '-1.859', '-0.223547488176']
AcBeF 17 ['Be0.166667F0.5Ac0.333333', '-2.878', '-0.28204194849']
AcBeH 2 ['H0.7Be0.1Ac0.2', '-0.575', '-0.221009448856']
AcBeN 18 ['Be0.3N0.4Ac0.3', '-1.017', '-0.201942222986']
AcBeO 6 ['Be0.1O0.7Ac0.2', '-2.623', '-0.239875946147']
AcBeP 4 ['Be0.166667P0.5Ac0.333333', '-1.161', '-0.211851148958']
AcBeSe 3 ['Be0.1Se0.5Ac0.4', '-1.729', '-0.258159866']
AcBiBr 25 ['Br0.5Bi0.25Ac0.25', '-1.938', '-0.200467136498']
AcBiCl 2 ['Cl0.777778Bi0.111111Ac0.111111', '-1.971', '-0.208170333476']
AcBiF 50 ['F0.222222Bi0.444444Ac0.333333', '-1.733', '-0.218664746581']
AcBiH 2 ['H0.571429Bi0.142857Ac0.285714', '-0.715', '-0.204958141341']
AcBiI 48 ['I0.4Bi0.1Ac0.5', '-1.18', '-0.201798710782']
AcBiN 30 ['N0.5Bi0.2Ac0.3', '-0.64', '-0.203512331216']
AcBiO 6 ['O0.6Bi0.1Ac0.3', '-3.289', '-0.235669986095']
AcBiP 30 ['P0.555556Bi0.333333Ac0.111111', '-0.473', '-0.200048752616']
AcBiPt 3 ['Pt0.375Bi0.125Ac0.5', '-1.164', '-0.209975984375']
AcBiS 16 ['S0.3Bi0.3Ac0.4', '-1.715', '-0.203178514294']
AcBiSe 32 ['Se0.2Bi0.3Ac0.5', '-1.333', '-0.20000038875']
AcBiTe 6 ['Te0.555556Bi0.111111Ac0.333333', '-1.204', '-0.201747992868']
AcBrCd 1 ['Br0.75Cd0.125Ac0.125', '-1.778', '-0.202975484425']
AcBrCe 2 ['Br0.7Ce0.2Ac0.1', '-2.297', '-0.200020812058']
AcBrCl 1 ['Cl0.375Br0.25Ac0.375', '-2.478', '-0.320171293433']
AcBrCo 3 ['Co0.111111Br0.777778Ac0.111111', '-1.425', '-0.264672257267']
AcBrCr 3 ['Cr0.125Br0.75Ac0.125', '-1.797', '-0.312003319426']
AcBrDy 3 ['Br0.8Dy0.1Ac0.1', '-1.932', '-0.498543712375']
AcBrEr 6 ['Br0.666667Er0.222222Ac0.111111', '-1.923', '-0.4167235706']
AcBrEu 2 ['Br0.666667Eu0.222222Ac0.111111', '-2.51', '-0.202971090576']
AcBrF 47 ['F0.375Br0.25Ac0.375', '-2.911', '-0.205419206803']
AcBrFe 2 ['Fe0.142857Br0.714286Ac0.142857', '-1.855', '-0.24294158363']
AcBrGa 3 ['Ga0.2Br0.7Ac0.1', '-1.747', '-0.223788463308']
AcBrGd 2 ['Br0.8Gd0.1Ac0.1', '-1.998', '-0.207569109048']
AcBrGe 18 ['Ge0.1Br0.7Ac0.2', '-2.19', '-0.208008402994']
AcBrH 2 ['H0.3Br0.4Ac0.3', '-1.715', '-0.215018519085']
AcBrHf 6 ['Br0.666667Hf0.222222Ac0.111111', '-1.418', '-0.239450982823']

AcBrHg 2 ['Br0.666667Hg0.166667Ac0.166667', '-1.991', '-0.255528914643']
AcBrHo 5 ['Br0.714286Ho0.142857Ac0.142857', '-2.131', '-0.291540188153']
AcBrI 35 ['Br0.6I0.2Ac0.2', '-2.069', '-0.202620406048']
AcBrIn 1 ['Br0.777778In0.111111Ac0.111111', '-1.755', '-0.238327868111']
AcBrIr 14 ['Br0.5Ir0.2Ac0.3', '-1.87', '-0.200460434707']
AcBrK 1 ['K0.142857Br0.714286Ac0.142857', '-2.106', '-0.221999201117']
AcBrKr 5 ['Br0.777778Kr0.111111Ac0.111111', '-1.252', '-0.215122447804']
AcBrLa 1 ['Br0.8La0.1Ac0.1', '-2.076', '-0.2395265123']
AcBrLu 6 ['Br0.666667Lu0.222222Ac0.111111', '-1.878', '-0.409169985045']
AcBrMg 1 ['Mg0.111111Br0.777778Ac0.111111', '-1.835', '-0.225441719581']
AcBrMn 1 ['Mn0.125Br0.75Ac0.125', '-1.765', '-0.215670880204']
AcBrMo 3 ['Br0.75Mo0.125Ac0.125', '-1.802', '-0.237042861624']
AcBrN 34 ['N0.125Br0.375Ac0.5', '-1.532', '-0.217769050925']
AcBrNb 3 ['Br0.75Nb0.125Ac0.125', '-2.0', '-0.262905484036']
AcBrNi 6 ['Ni0.1Br0.8Ac0.1', '-1.318', '-0.209959220541']
AcBrNp 2 ['Br0.777778Ac0.111111Np0.111111', '-2.018', '-0.275648263804']
AcBrO 43 ['O0.2Br0.4Ac0.4', '-2.768', '-0.203097553523']
AcBrOs 4 ['Br0.5Os0.125Ac0.375', '-1.767', '-0.211683671707']
AcBrP 26 ['P0.166667Br0.666667Ac0.166667', '-1.888', '-0.204388446172']
AcBrPa 1 ['Br0.777778Ac0.111111Pa0.111111', '-2.193', '-0.219035062843']
AcBrPb 2 ['Br0.75Pb0.125Ac0.125', '-1.795', '-0.20959788474']
AcBrPd 1 ['Br0.666667Pd0.166667Ac0.166667', '-1.905', '-0.218606319221']
AcBrPm 6 ['Br0.666667Pm0.222222Ac0.111111', '-1.899', '-0.293910696711']
AcBrPr 1 ['Br0.8Pr0.1Ac0.1', '-2.006', '-0.214360294048']
AcBrPt 54 ['Br0.222222Pt0.222222Ac0.555556', '-1.34', '-0.201286921314']
AcBrPu 1 ['Br0.8Ac0.1Pu0.1', '-2.032', '-0.3889733508']
AcBrRb 2 ['Br0.75Rb0.125Ac0.125', '-1.85', '-0.201616780352']
AcBrRe 3 ['Br0.777778Re0.111111Ac0.111111', '-1.425', '-0.388122447804']
AcBrRh 21 ['Br0.428571Rh0.285714Ac0.285714', '-1.688', '-0.202978754677']
AcBrRu 5 ['Br0.75Ru0.125Ac0.125', '-1.65', '-0.214106055999']
AcBrS 21 ['S0.333333Br0.111111Ac0.555556', '-1.986', '-0.203527878761']
AcBrSb 32 ['Br0.5Sb0.166667Ac0.333333', '-2.089', '-0.211545077957']
AcBrSc 6 ['Sc0.222222Br0.666667Ac0.111111', '-1.855', '-0.413453158379']
AcBrSe 53 ['Se0.2Br0.6Ac0.2', '-2.074', '-0.207620406048']
AcBrSi 13 ['Si0.285714Br0.285714Ac0.428571', '-1.283', '-0.209761728832']
AcBrSm 1 ['Br0.777778Sm0.111111Ac0.111111', '-2.218', '-0.238087192276']
AcBrSn 3 ['Br0.8Sn0.1Ac0.1', '-1.618', '-0.210526946123']
AcBrTa 3 ['Br0.75Ta0.125Ac0.125', '-1.857', '-0.230962449222']
AcBrTb 4 ['Br0.7Tb0.2Ac0.1', '-1.931', '-0.203857169411']
AcBrTc 4 ['Br0.714286Tc0.142857Ac0.142857', '-1.811', '-0.248688856963']
AcBrTe 42 ['Br0.444444Te0.222222Ac0.333333', '-2.12', '-0.216478658275']
AcBrTi 1 ['Ti0.1Br0.8Ac0.1', '-2.01', '-0.346807097429']
AcBrTl 3 ['Br0.75Tl0.125Ac0.125', '-1.69', '-0.201361169895']
AcBrTm 5 ['Br0.714286Tm0.142857Ac0.142857', '-2.154', '-0.281177571487']

AcBrU 1 ['Br0.8Ac0.1U0.1', '-2.076', '-0.240522091318']
AcBrV 3 ['V0.125Br0.75Ac0.125', '-1.828', '-0.278197185675']
AcBrW 3 ['Br0.75W0.125Ac0.125', '-1.754', '-0.234474898265']
AcBrY 5 ['Br0.714286Y0.142857Ac0.142857', '-2.138', '-0.278360057201']
AcBrYb 1 ['Br0.666667Yb0.222222Ac0.111111', '-2.459', '-0.22064756413']
AcBrZn 4 ['Zn0.222222Br0.666667Ac0.111111', '-1.756', '-0.202295975334']
AcBrZr 2 ['Br0.777778Zr0.111111Ac0.111111', '-2.082', '-0.322620161444']
AcCCl 1 ['C0.111111Cl0.777778Ac0.111111', '-1.545', '-0.318181740071']
AcCF 21 ['C0.1F0.4Ac0.5', '-2.26', '-0.20354919568']
AcCGe 4 ['C0.444444Ge0.333333Ac0.222222', '-0.444', '-0.203567983889']
AcCH 32 ['H0.444444C0.333333Ac0.222222', '-0.581', '-0.202143084029']
AcCI 7 ['C0.2I0.5Ac0.3', '-1.151', '-0.209251734536']
AcCIr 4 ['C0.111111Ir0.333333Ac0.555556', '-0.502', '-0.2164419075']
AcCN 44 ['C0.142857N0.571429Ac0.285714', '-0.54', '-0.202300107759']
AcCNa 1 ['C0.5Na0.333333Ac0.166667', '-0.223', '-0.223']
AcCNi 13 ['C0.222222Ni0.222222Ac0.555556', '-0.285', '-0.224555971667']
AcCO 10 ['C0.222222O0.555556Ac0.222222', '-2.716', '-0.213822842843']
AcCP 1 ['C0.1P0.5Ac0.4', '-1.187', '-0.204375509416']
AcCPt 18 ['C0.375Pt0.25Ac0.375', '-0.722', '-0.218605950625']
AcCRh 7 ['C0.333333Rh0.222222Ac0.444444', '-0.449', '-0.212722056111']
AcCS 6 ['C0.1S0.4Ac0.5', '-1.941', '-0.216784475392']
AcCSb 3 ['C0.166667Sb0.333333Ac0.5', '-0.862', '-0.2177228125']
AcCSe 11 ['C0.142857Se0.428571Ac0.428571', '-1.622', '-0.217227389643']
AcCSi 2 ['C0.333333Si0.222222Ac0.444444', '-0.344', '-0.200510823889']
AcCTe 6 ['C0.3Te0.3Ac0.4', '-1.056', '-0.219892052748']
AcCXe 2 ['C0.444444Xe0.333333Ac0.222222', '-0.205', '-0.205']
AcCaF 9 ['F0.666667Ca0.222222Ac0.111111', '-3.911', '-0.241207718587']
AcCaN 9 ['N0.444444Ca0.333333Ac0.222222', '-1.009', '-0.252273389847']
AcCaO 7 ['O0.625Ca0.25Ac0.125', '-3.022', '-0.281374792885']
AcCaP 3 ['P0.5Ca0.1Ac0.4', '-1.438', '-0.209283890854']
AcCaPt 8 ['Ca0.25Pt0.375Ac0.375', '-1.039', '-0.231284673438']
AcCaSe 4 ['Ca0.142857Se0.571429Ac0.285714', '-1.852', '-0.286627068571']
AcCaTe 3 ['Ca0.142857Te0.571429Ac0.285714', '-1.525', '-0.23532604595']
AcCdF 34 ['F0.428571Cd0.142857Ac0.428571', '-2.499', '-0.208570920014']
AcCdGa 1 ['Ga0.7Cd0.1Ac0.2', '-0.382', '-0.2078094935']
AcCdI 5 ['Cd0.166667I0.666667Ac0.166667', '-1.277', '-0.214559372869']
AcCdN 23 ['N0.333333Cd0.333333Ac0.333333', '-0.627', '-0.233016792385']
AcCdO 6 ['O0.666667Cd0.166667Ac0.166667', '-2.2', '-0.296372748799']
AcCdP 18 ['P0.571429Cd0.142857Ac0.285714', '-1.035', '-0.206254315951']
AcCdPt 1 ['Cd0.1Pt0.4Ac0.5', '-1.082', '-0.21560726825']
AcCdSe 11 ['Se0.5Cd0.2Ac0.3', '-1.441', '-0.20381025075']
AcCdTe 2 ['Cd0.111111Te0.555556Ac0.333333', '-1.312', '-0.264463242313']
AcCeF 5 ['F0.714286Ce0.142857Ac0.142857', '-3.934', '-0.33407187032']
AcCeI 5 ['I0.714286Ce0.142857Ac0.142857', '-1.483', '-0.309471954864']

AcCeN 3 ['N0.5Ce0.125Ac0.375', '-1.0', '-0.228922637328']
AcCeO 6 ['O0.666667Ce0.222222Ac0.111111', '-3.263', '-0.312854667753']
AcCePt 1 ['Ce0.1Pt0.4Ac0.5', '-1.065', '-0.2451423']
AcCeSe 4 ['Se0.571429Ce0.142857Ac0.285714', '-1.904', '-0.259650712073']
AcCeTe 4 ['Te0.625Ce0.125Ac0.25', '-1.404', '-0.203073468855']
AcClCs 1 ['Cl0.714286Cs0.142857Ac0.142857', '-2.439', '-0.24627111786']
AcClCu 2 ['Cl0.555556Cu0.333333Ac0.111111', '-1.682', '-0.200411823267']
AcClDy 1 ['Cl0.8Dy0.1Ac0.1', '-2.316', '-0.202382537212']
AcClEr 6 ['Cl0.666667Er0.222222Ac0.111111', '-2.242', '-0.426559343267']
AcClF 43 ['F0.333333Cl0.333333Ac0.333333', '-3.143', '-0.202472736471']
AcClFe 1 ['Cl0.777778Fe0.111111Ac0.111111', '-1.885', '-0.228284005976']
AcClGe 12 ['Cl0.375Ge0.25Ac0.375', '-1.851', '-0.200343439455']
AcClH 9 ['H0.25Cl0.375Ac0.375', '-1.802', '-0.208722442347']
AcClHo 6 ['Cl0.666667Ho0.222222Ac0.111111', '-2.208', '-0.379506713636']
AcClIn 3 ['Cl0.75In0.125Ac0.125', '-2.049', '-0.246150984191']
AcClIr 4 ['Cl0.714286Ir0.142857Ac0.142857', '-1.828', '-0.250662237234']
AcClK 2 ['Cl0.7K0.2Ac0.1', '-2.163', '-0.20242436394']
AcClKr 1 ['Cl0.75Kr0.125Ac0.125', '-1.595', '-0.21482945758']
AcClLu 6 ['Cl0.666667Lu0.222222Ac0.111111', '-2.185', '-0.4066966266']
AcClMn 1 ['Cl0.777778Mn0.111111Ac0.111111', '-1.869', '-0.200614666178']
AcClN 31 ['N0.1Cl0.6Ac0.3', '-2.533', '-0.206532169844']
AcClNd 1 ['Cl0.777778Nd0.111111Ac0.111111', '-2.579', '-0.2118053082']
AcClNp 1 ['Cl0.8Ac0.1Np0.1', '-2.398', '-0.357717110439']
AcClO 22 ['O0.7Cl0.2Ac0.1', '-1.334', '-0.229629219736']
AcClP 8 ['P0.3Cl0.2Ac0.5', '-1.68', '-0.206940676105']
AcClPm 1 ['Cl0.777778Pm0.111111Ac0.111111', '-2.44', '-0.273529253476']
AcClPt 27 ['Cl0.125Pt0.375Ac0.5', '-1.418', '-0.202852078464']
AcClPu 2 ['Cl0.777778Ac0.111111Pu0.111111', '-2.477', '-0.2833297732']
AcClRb 1 ['Cl0.714286Rb0.142857Ac0.142857', '-2.598', '-0.411545149289']
AcClRh 9 ['Cl0.7Rh0.1Ac0.2', '-2.441', '-0.232727132128']
AcClRu 2 ['Cl0.8Ru0.1Ac0.1', '-1.646', '-0.221334865378']
AcClS 2 ['S0.3Cl0.4Ac0.3', '-2.684', '-0.250884182081']
AcClSb 10 ['Cl0.285714Sb0.285714Ac0.428571', '-1.809', '-0.205203902204']
AcClSe 31 ['Cl0.25Se0.25Ac0.5', '-1.946', '-0.206435615678']
AcClSi 8 ['Si0.1Cl0.7Ac0.2', '-2.6', '-0.200845713441']
AcClSm 3 ['Cl0.8Sm0.1Ac0.1', '-2.139', '-0.267320007628']
AcClTc 1 ['Cl0.8Tc0.1Ac0.1', '-1.818', '-0.238046132942']
AcClTe 32 ['Cl0.375Te0.25Ac0.375', '-2.294', '-0.21707283487']
AcClTl 1 ['Cl0.777778Tl0.111111Ac0.111111', '-1.828', '-0.246954696601']
AcClV 1 ['Cl0.8V0.1Ac0.1', '-2.074', '-0.268790854814']
AcClY 1 ['Cl0.777778Y0.111111Ac0.111111', '-2.61', '-0.248388344311']
AcClZr 1 ['Cl0.8Zr0.1Ac0.1', '-2.353', '-0.218001238314']
AcCoF 23 ['F0.428571Co0.142857Ac0.428571', '-2.412', '-0.208659852514']
AcCoGa 1 ['Co0.1Ga0.7Ac0.2', '-0.504', '-0.22297019425']

AcCoH 7 ['H0.571429Co0.142857Ac0.285714', '-0.693', '-0.205898250895']
AcCoI 24 ['Co0.1I0.6Ac0.3', '-1.312', '-0.206523202792']
AcCoN 53 ['N0.666667Co0.222222Ac0.111111', '-0.339', '-0.203391140757']
AcCoO 8 ['O0.625Co0.25Ac0.125', '-2.117', '-0.208780856855']
AcCoP 2 ['P0.4Co0.1Ac0.5', '-1.214', '-0.231375509416']
AcCoSe 12 ['Co0.222222Se0.444444Ac0.333333', '-1.363', '-0.202527250972']
AcCrF 10 ['F0.666667Cr0.222222Ac0.111111', '-2.993', '-0.213993414467']
AcCrN 31 ['N0.6Cr0.2Ac0.2', '-0.667', '-0.203704530362']
AcCrO 11 ['O0.6Cr0.1Ac0.3', '-3.567', '-0.208170311795']
AcCrP 1 ['P0.4Cr0.1Ac0.5', '-1.19', '-0.207375509416']
AcCrSe 7 ['Cr0.142857Se0.571429Ac0.285714', '-1.36', '-0.211589510714']
AcCrTe 4 ['Cr0.166667Te0.666667Ac0.166667', '-0.79', '-0.201610520528']
AcCsF 27 ['F0.5Cs0.125Ac0.375', '-2.855', '-0.218820716058']
AcCsI 4 ['I0.75Cs0.125Ac0.125', '-1.358', '-0.208446294114']
AcCsN 6 ['N0.5Cs0.125Ac0.375', '-0.702', '-0.208449769099']
AcCsO 10 ['O0.714286Cs0.142857Ac0.142857', '-2.002', '-0.221369674791']
AcCsP 1 ['P0.5Cs0.1Ac0.4', '-1.335', '-0.247787332353']
AcCsS 1 ['S0.5Cs0.375Ac0.125', '-1.677', '-0.20461596346']
AcCsSe 24 ['Se0.555556Cs0.333333Ac0.111111', '-1.234', '-0.202690642222']
AcCsTe 9 ['Te0.666667Cs0.166667Ac0.166667', '-1.064', '-0.250679336528']
AcCuF 27 ['F0.5Cu0.1Ac0.4', '-2.785', '-0.2026667021']
AcCuI 11 ['Cu0.3I0.5Ac0.2', '-1.106', '-0.205286936784']
AcCuN 44 ['N0.222222Cu0.444444Ac0.333333', '-0.489', '-0.206728207424']
AcCuO 5 ['O0.571429Cu0.142857Ac0.285714', '-3.07', '-0.278589004436']
AcCuP 14 ['P0.333333Cu0.555556Ac0.111111', '-0.568', '-0.201291754791']
AcCuSe 29 ['Cu0.125Se0.625Ac0.25', '-1.154', '-0.212766585208']
AcDyF 5 ['F0.714286Dy0.142857Ac0.142857', '-4.178', '-0.717393218537']
AcDyI 8 ['I0.666667Dy0.166667Ac0.166667', '-1.355', '-0.203158871943']
AcDyN 2 ['N0.5Dy0.125Ac0.375', '-1.153', '-0.257628016181']
AcDyO 8 ['O0.6Dy0.2Ac0.2', '-4.102', '-0.387101385265']
AcDyP 1 ['P0.5Dy0.1Ac0.4', '-1.533', '-0.202186190854']
AcDySe 4 ['Se0.625Dy0.25Ac0.125', '-1.835', '-0.217373185519']
AcDyTe 8 ['Te0.571429Dy0.142857Ac0.285714', '-1.517', '-0.206821237676']
AcErF 5 ['F0.714286Er0.142857Ac0.142857', '-4.013', '-0.373200528417']
AcErI 8 ['I0.666667Er0.111111Ac0.222222', '-1.427', '-0.215178833519']
AcErN 5 ['N0.5Er0.166667Ac0.333333', '-1.243', '-0.220228668162']
AcErO 8 ['O0.6Er0.1Ac0.3', '-3.883', '-0.21924224472']
AcErP 2 ['P0.5Er0.1Ac0.4', '-1.54', '-0.20977120452']
AcErSe 4 ['Se0.571429Er0.142857Ac0.285714', '-1.854', '-0.221745518214']
AcErTe 2 ['Te0.625Er0.125Ac0.25', '-1.374', '-0.217945826978']
AcEuF 8 ['F0.666667Eu0.166667Ac0.166667', '-3.904', '-0.335529319255']
AcEuI 1 ['I0.7Eu0.1Ac0.2', '-1.693', '-0.231784229297']
AcEuN 22 ['N0.5Eu0.3Ac0.2', '-0.787', '-0.203575680828']
AcEuO 7 ['O0.6Eu0.2Ac0.2', '-3.446', '-0.297523088559']

AcEuP 24 ['P0.555556Eu0.222222Ac0.222222', '-1.426', '-0.201002584024']
AcEuPt 4 ['Eu0.142857Pt0.428571Ac0.428571', '-1.168', '-0.241203658214']
AcEuSe 6 ['Se0.555556Eu0.222222Ac0.222222', '-1.948', '-0.203789906667']
AcEuTe 3 ['Te0.571429Eu0.142857Ac0.285714', '-1.548', '-0.210741668807']
AcFFe 15 ['F0.625Fe0.25Ac0.125', '-2.623', '-0.220238956793']
AcFGa 41 ['F0.3Ga0.4Ac0.3', '-1.838', '-0.20856664351']
AcFGd 5 ['F0.714286Gd0.142857Ac0.142857', '-4.07', '-0.593858662703']
AcFGe 70 ['F0.111111Ge0.333333Ac0.555556', '-1.134', '-0.2021156413']
AcFH 69 ['H0.1F0.4Ac0.5', '-2.344', '-0.202306389587']
AcFHf 5 ['F0.714286Hf0.142857Ac0.142857', '-3.913', '-0.25142593732']
AcFHg 26 ['F0.444444Hg0.333333Ac0.222222', '-2.218', '-0.202749747056']
AcFHo 5 ['F0.714286Ho0.142857Ac0.142857', '-3.972', '-0.331860946154']
AcFI 76 ['F0.111111I0.555556Ac0.333333', '-1.789', '-0.20177314995']
AcFIn 49 ['F0.333333In0.444444Ac0.222222', '-1.916', '-0.2022909964']
AcFIr 23 ['F0.4Ir0.1Ac0.5', '-2.344', '-0.20188176793']
AcFK 20 ['F0.5K0.3Ac0.2', '-3.017', '-0.240962896598']
AcFKr 24 ['F0.5Kr0.4Ac0.1', '-1.164', '-0.229101555059']
AcFLa 5 ['F0.714286La0.142857Ac0.142857', '-4.116', '-0.45476789044']
AcFLi 18 ['Li0.222222F0.555556Ac0.222222', '-3.315', '-0.201345769924']
AcFLu 5 ['F0.714286Lu0.142857Ac0.142857', '-4.159', '-0.745195975914']
AcFMg 13 ['F0.625Mg0.25Ac0.125', '-3.744', '-0.23397884679']
AcFMn 17 ['F0.5Mn0.2Ac0.3', '-2.726', '-0.215298988839']
AcFMo 9 ['F0.7Mo0.2Ac0.1', '-2.784', '-0.212896125357']
AcFN 64 ['N0.5F0.1Ac0.4', '-1.143', '-0.234904091305']
AcFNa 29 ['F0.444444Na0.333333Ac0.222222', '-2.769', '-0.215099538807']
AcFNb 6 ['F0.666667Nb0.111111Ac0.222222', '-3.308', '-0.237687869652']
AcFNd 5 ['F0.714286Nd0.142857Ac0.142857', '-3.981', '-0.369625967103']
AcFNi 20 ['F0.5Ni0.333333Ac0.166667', '-2.239', '-0.231802582518']
AcFNp 6 ['F0.666667Ac0.222222Np0.111111', '-3.583', '-0.218426731271']
AcFO 36 ['O0.5F0.166667Ac0.333333', '-3.749', '-0.200426930193']
AcFOs 17 ['F0.444444Os0.111111Ac0.444444', '-2.499', '-0.214054661867']
AcFP 61 ['F0.4P0.1Ac0.5', '-2.511', '-0.208893073034']
AcFPa 5 ['F0.714286Ac0.142857Pa0.142857', '-3.763', '-0.6056868252']
AcFPb 44 ['F0.3Pb0.1Ac0.6', '-1.839', '-0.20034025301']
AcFPd 26 ['F0.5Pd0.4Ac0.1', '-1.709', '-0.232321926811']
AcFPm 5 ['F0.714286Pm0.142857Ac0.142857', '-4.093', '-0.60889466056']
AcFPr 5 ['F0.714286Pr0.142857Ac0.142857', '-4.103', '-0.496378916274']
AcFPt 49 ['F0.285714Pt0.285714Ac0.428571', '-2.178', '-0.20929217251']
AcFPu 35 ['F0.5Ac0.375Pu0.125', '-2.797', '-0.2029012421']
AcFRb 23 ['F0.5Rb0.25Ac0.25', '-2.914', '-0.200027420015']
AcFRe 8 ['F0.6Re0.1Ac0.3', '-2.927', '-0.226612627556']
AcFRh 23 ['F0.444444Rh0.222222Ac0.333333', '-2.528', '-0.203675004552']
AcFRu 16 ['F0.444444Ru0.111111Ac0.444444', '-2.495', '-0.210054661867']
AcFS 59 ['F0.1S0.4Ac0.5', '-2.441', '-0.202671774312']

AcFSb 70 ['F0.6Sb0.3Ac0.1', '-2.535', '-0.20173111827']
AcFSc 7 ['F0.666667Sc0.111111Ac0.222222', '-3.837', '-0.225587169467']
AcFSe 83 ['F0.222222Se0.222222Ac0.555556', '-2.077', '-0.206126718156']
AcFSi 51 ['F0.2Si0.4Ac0.4', '-1.425', '-0.224587586507']
AcFSm 5 ['F0.714286Sm0.142857Ac0.142857', '-3.976', '-0.347491934846']
AcFSn 51 ['F0.166667Sn0.333333Ac0.5', '-1.496', '-0.202032013416']
AcFSr 9 ['F0.666667Sr0.222222Ac0.111111', '-3.877', '-0.216153727473']
AcFTa 5 ['F0.714286Ta0.142857Ac0.142857', '-3.587', '-0.403716312691']
AcFTb 5 ['F0.714286Tb0.142857Ac0.142857', '-3.924', '-0.376286759891']
AcFTc 9 ['F0.6Tc0.1Ac0.3', '-3.083', '-0.350483884256']
AcFTe 57 ['F0.125Te0.375Ac0.5', '-1.909', '-0.221224189585']
AcFTh 3 ['F0.75Ac0.125Th0.125', '-4.042', '-0.290305414869']
AcFTi 9 ['F0.666667Ti0.222222Ac0.111111', '-3.469', '-0.206561388007']
AcFTl 47 ['F0.5Tl0.1Ac0.4', '-2.837', '-0.2110256431']
AcFTm 16 ['F0.6Tm0.1Ac0.3', '-3.009', '-0.259664161226']
AcFU 4 ['F0.7Ac0.2U0.1', '-3.636', '-0.210025641551']
AcFV 8 ['F0.7V0.2Ac0.1', '-3.261', '-0.21551465369']
AcFW 6 ['F0.714286W0.142857Ac0.142857', '-3.09', '-0.282833472944']
AcFXe 22 ['F0.5Xe0.4Ac0.1', '-1.404', '-0.256023564934']
AcFY 5 ['F0.714286Y0.142857Ac0.142857', '-3.931', '-0.43040729496']
AcFYb 21 ['F0.5Yb0.375Ac0.125', '-2.887', '-0.24014361115']
AcFZn 27 ['F0.428571Zn0.285714Ac0.285714', '-2.404', '-0.200659852514']
AcFZr 5 ['F0.714286Zr0.142857Ac0.142857', '-3.788', '-0.210728931538']
AcFeH 5 ['H0.625Fe0.125Ac0.25', '-0.633', '-0.206452720197']
AcFeI 1 ['Fe0.1I0.7Ac0.2', '-1.33', '-0.233527996797']
AcFeN 25 ['N0.5Fe0.333333Ac0.166667', '-0.577', '-0.227419624411']
AcFeO 10 ['O0.571429Fe0.142857Ac0.285714', '-3.212', '-0.206925567505']
AcFeP 4 ['P0.444444Fe0.111111Ac0.444444', '-1.296', '-0.204195010462']
AcFeSe 8 ['Fe0.2Se0.5Ac0.3', '-1.302', '-0.20570196975']
AcGaGd 1 ['Ga0.75Gd0.125Ac0.125', '-0.563', '-0.20359393875']
AcGaHg 1 ['Ga0.7Hg0.1Ac0.2', '-0.415', '-0.22784830217']
AcGaI 4 ['Ga0.125I0.5Ac0.375', '-1.237', '-0.206900269098']
AcGaIr 1 ['Ga0.7Ir0.1Ac0.2', '-0.552', '-0.2120291715']
AcGaK 3 ['K0.142857Ga0.714286Ac0.142857', '-0.427', '-0.20649743631']
AcGaKr 2 ['Ga0.666667Kr0.111111Ac0.222222', '-0.41', '-0.216454992778']
AcGaLi 2 ['Li0.111111Ga0.666667Ac0.222222', '-0.493', '-0.227322407778']
AcGaLu 1 ['Ga0.7Lu0.1Ac0.2', '-0.582', '-0.2144569465']
AcGaMg 1 ['Mg0.1Ga0.7Ac0.2', '-0.438', '-0.221547541333']
AcGaMn 1 ['Mn0.1Ga0.7Ac0.2', '-0.439', '-0.218831097371']
AcGaN 30 ['N0.5Ga0.125Ac0.375', '-0.793', '-0.204951302015']
AcGaNa 8 ['Na0.111111Ga0.777778Ac0.111111', '-0.391', '-0.202460165183']
AcGaNd 2 ['Ga0.75Nd0.125Ac0.125', '-0.575', '-0.206509475313']
AcGaO 9 ['O0.714286Ga0.142857Ac0.142857', '-2.336', '-0.25480607355']
AcGaOs 1 ['Ga0.7Os0.1Ac0.2', '-0.48', '-0.20077333025']

AcGaP 8 ['P0.5Ga0.1Ac0.4', '-1.313', '-0.22354171802']
AcGaPa 1 ['Ga0.75Ac0.125Pa0.125', '-0.398', '-0.224802384063']
AcGaPb 2 ['Ga0.666667Pb0.111111Ac0.222222', '-0.447', '-0.225154028519']
AcGaPd 4 ['Ga0.714286Pd0.142857Ac0.142857', '-0.545', '-0.207565292589']
AcGaPm 6 ['Ga0.8Pm0.1Ac0.1', '-0.417', '-0.216425881']
AcGaPr 1 ['Ga0.7Pr0.1Ac0.2', '-0.595', '-0.21314647575']
AcGaPt 3 ['Ga0.1Pt0.4Ac0.5', '-1.104', '-0.21147426775']
AcGaRb 1 ['Ga0.777778Rb0.111111Ac0.111111', '-0.405', '-0.217949942824']
AcGaRh 1 ['Ga0.7Rh0.1Ac0.2', '-0.582', '-0.217120435']
AcGaS 5 ['S0.4Ga0.2Ac0.4', '-1.928', '-0.203784475392']
AcGaSc 2 ['Sc0.142857Ga0.714286Ac0.142857', '-0.613', '-0.206281883929']
AcGaSe 32 ['Ga0.125Se0.625Ac0.25', '-1.266', '-0.203494279688']
AcGaSm 2 ['Ga0.75Sm0.125Ac0.125', '-0.579', '-0.208897748125']
AcGaSr 1 ['Ga0.7Sr0.1Ac0.2', '-0.596', '-0.2142830855']
AcGaTe 15 ['Ga0.166667Te0.5Ac0.333333', '-1.262', '-0.211944786942']
AcGaTm 1 ['Ga0.7Tm0.1Ac0.2', '-0.58', '-0.204120488']
AcGdI 10 ['I0.625Gd0.25Ac0.125', '-1.35', '-0.253653958793']
AcGdN 3 ['N0.5Gd0.166667Ac0.333333', '-1.167', '-0.200610120245']
AcGdO 8 ['O0.6Gd0.2Ac0.2', '-3.994', '-0.30780408347']
AcGdSe 7 ['Se0.555556Gd0.111111Ac0.333333', '-1.844', '-0.208610046528']
AcGdTe 4 ['Te0.6Gd0.2Ac0.2', '-1.483', '-0.204068614749']
AcGeH 9 ['H0.571429Ge0.142857Ac0.285714', '-0.691', '-0.203898250895']
AcGeI 29 ['Ge0.333333I0.333333Ac0.333333', '-1.034', '-0.202211542244']
AcGeIr 1 ['Ge0.7Ir0.1Ac0.2', '-0.534', '-0.229304525833']
AcGeN 39 ['N0.5Ge0.375Ac0.125', '-0.472', '-0.21014393148']
AcGeO 8 ['O0.666667Ge0.222222Ac0.111111', '-2.463', '-0.209382202795']
AcGeP 6 ['P0.5Ge0.166667Ac0.333333', '-1.062', '-0.206952388298']
AcGePa 3 ['Ge0.714286Ac0.142857Pa0.142857', '-0.455', '-0.209223876071']
AcGePt 4 ['Ge0.571429Pt0.285714Ac0.142857', '-0.665', '-0.211982468929']
AcGeRb 1 ['Ge0.75Rb0.125Ac0.125', '-0.354', '-0.218756990938']
AcGeRh 2 ['Ge0.666667Rh0.166667Ac0.166667', '-0.571', '-0.205548351667']
AcGeS 19 ['S0.333333Ge0.166667Ac0.5', '-1.834', '-0.21682971741']
AcGeSe 47 ['Ge0.375Se0.5Ac0.125', '-0.779', '-0.202109711875']
AcGeTe 9 ['Ge0.125Te0.5Ac0.375', '-1.293', '-0.225915159893']
AcHI 21 ['H0.5I0.3Ac0.2', '-0.907', '-0.204293590333']
AcHIr 11 ['H0.7Ir0.2Ac0.1', '-0.388', '-0.217514387813']
AcHLi 1 ['H0.5Li0.4Ac0.1', '-0.617', '-0.200841108158']
AcHMn 5 ['H0.7Mn0.1Ac0.2', '-0.545', '-0.204028775626']
AcHN 70 ['H0.4N0.5Ac0.1', '-0.528', '-0.210196149463']
AcHO 5 ['H0.5O0.333333Ac0.166667', '-2.014', '-0.226483499967']
AcHOs 2 ['H0.666667Os0.111111Ac0.222222', '-0.583', '-0.204143084029']
AcHP 5 ['H0.444444P0.222222Ac0.333333', '-0.943', '-0.207669047246']
AcHPd 1 ['H0.666667Pd0.166667Ac0.166667', '-0.576', '-0.219684391598']
AcHPm 6 ['H0.7Pm0.1Ac0.2', '-0.69', '-0.200319246721']

AcHPt 52 ['H0.125Pt0.5Ac0.375', '-1.026', '-0.209912432227']
AcHRh 15 ['H0.5Rh0.333333Ac0.166667', '-0.49', '-0.205857313022']
AcHRu 3 ['H0.7Ru0.1Ac0.2', '-0.559', '-0.218028775626']
AcHS 26 ['H0.5S0.166667Ac0.333333', '-1.224', '-0.204520480027']
AcHSe 43 ['H0.5Se0.25Ac0.25', '-1.031', '-0.211549310625']
AcHSi 3 ['H0.555556Si0.111111Ac0.333333', '-0.729', '-0.219556561009']
AcHTe 12 ['H0.1Te0.5Ac0.4', '-1.339', '-0.210954214331']
AcHfI 2 ['I0.75Hf0.125Ac0.125', '-1.384', '-0.21610873599']
AcHfN 10 ['N0.555556Hf0.333333Ac0.111111', '-1.678', '-0.211038237867']
AcHfO 9 ['O0.7Hf0.2Ac0.1', '-3.413', '-0.214569570513']
AcHfS 8 ['S0.5Hf0.25Ac0.25', '-2.072', '-0.207691292369']
AcHfSe 14 ['Se0.6Hf0.2Ac0.2', '-1.69', '-0.2266266375']
AcHgI 9 ['I0.6Hg0.3Ac0.1', '-0.918', '-0.204636321802']
AcHgN 13 ['N0.285714Hg0.285714Ac0.428571', '-0.7', '-0.208660110983']
AcHgO 7 ['O0.7Hg0.1Ac0.2', '-2.131', '-0.250235539317']
AcHgP 11 ['P0.6Hg0.2Ac0.2', '-0.695', '-0.203687754708']
AcHgRh 1 ['Rh0.428571Hg0.142857Ac0.428571', '-0.699', '-0.233962178457']
AcHgSe 23 ['Se0.428571Hg0.428571Ac0.142857', '-0.777', '-0.204180038938']
AcHgTe 7 ['Te0.5Hg0.125Ac0.375', '-1.28', '-0.200541402398']
AcHoI 7 ['I0.666667Ho0.111111Ac0.222222', '-1.383', '-0.200287116711']
AcHoN 3 ['N0.5Ho0.2Ac0.3', '-1.301', '-0.207029619411']
AcHoO 8 ['O0.6Ho0.1Ac0.3', '-3.883', '-0.22778996864']
AcHoP 1 ['P0.5Ho0.1Ac0.4', '-1.539', '-0.208247088186']
AcHoSe 6 ['Se0.666667Ho0.111111Ac0.222222', '-1.544', '-0.203267656853']
AcHoTe 3 ['Te0.666667Ho0.111111Ac0.222222', '-1.231', '-0.206336352406']
AcIIn 9 ['In0.2I0.5Ac0.3', '-1.234', '-0.2072999252']
AcIIr 30 ['I0.4Ir0.1Ac0.5', '-1.114', '-0.209277636282']
AcIK 2 ['K0.142857I0.714286Ac0.142857', '-1.478', '-0.265166046826']
AcIKr 1 ['Kr0.1I0.7Ac0.2', '-1.313', '-0.24855840504']
AcILi 13 ['Li0.3I0.5Ac0.2', '-1.405', '-0.20231023379']
AcILu 9 ['I0.666667Lu0.111111Ac0.222222', '-1.395', '-0.212287116711']
AcIMg 14 ['Mg0.1I0.6Ac0.3', '-1.35', '-0.219635396877']
AcIN 44 ['N0.222222I0.555556Ac0.222222', '-1.232', '-0.202630018635']
AcINb 2 ['Nb0.111111I0.777778Ac0.111111', '-1.16', '-0.234714067276']
AcINd 3 ['I0.8Nd0.1Ac0.1', '-1.253', '-0.294664949533']
AcINi 12 ['Ni0.3I0.6Ac0.1', '-0.831', '-0.200363366291']
AcINp 4 ['I0.714286Ac0.142857Np0.142857', '-1.353', '-0.208738760168']
AcIO 16 ['O0.7I0.2Ac0.1', '-1.559', '-0.250181974443']
AcIOs 9 ['I0.5Os0.2Ac0.3', '-1.162', '-0.220251734536']
AcIP 48 ['P0.111111I0.555556Ac0.333333', '-1.477', '-0.203256460153']
AcIPa 6 ['I0.7Ac0.2Pa0.1', '-1.299', '-0.206822661797']
AcIPb 11 ['I0.4Pb0.2Ac0.4', '-1.154', '-0.224372181028']
AcIPd 36 ['Pd0.125I0.5Ac0.375', '-1.301', '-0.201338835972']
AcIPm 6 ['I0.666667Pm0.222222Ac0.111111', '-1.293', '-0.304033115038']

AcIPr 1 ['I0.75Pr0.125Ac0.125', '-1.588', '-0.251874652392']
AcIPt 72 ['I0.111111Pt0.555556Ac0.333333', '-1.114', '-0.20924567297']
AcIPu 9 ['I0.666667Ac0.166667Pu0.166667', '-1.396', '-0.240535733796']
AcIRb 2 ['Rb0.142857I0.714286Ac0.142857', '-1.502', '-0.250250471835']
AcIRe 2 ['I0.777778Re0.111111Ac0.111111', '-0.91', '-0.234857721064']
AcIRh 44 ['Rh0.111111I0.777778Ac0.111111', '-0.811', '-0.219643558356']
AcIRu 10 ['Ru0.142857I0.714286Ac0.142857', '-0.967', '-0.206684575029']
AcIS 35 ['S0.166667I0.5Ac0.333333', '-1.806', '-0.20054220228']
AcISb 54 ['Sb0.1I0.4Ac0.5', '-1.218', '-0.205661907782']
AcISc 3 ['Sc0.111111I0.777778Ac0.111111', '-1.247', '-0.245241893926']
AcISe 44 ['Se0.111111I0.444444Ac0.444444', '-1.439', '-0.210333322811']
AcISi 35 ['Si0.2I0.7Ac0.1', '-1.024', '-0.226508040882']
AcISm 3 ['I0.8Sm0.1Ac0.1', '-1.271', '-0.356917781042']
AcISn 9 ['Sn0.125I0.5Ac0.375', '-1.29', '-0.204851778866']
AcITb 7 ['I0.7Tb0.1Ac0.2', '-1.382', '-0.232857906331']
AcITe 58 ['Te0.4I0.1Ac0.5', '-1.527', '-0.207425669672']
AcITh 2 ['I0.714286Ac0.142857Th0.142857', '-1.524', '-0.250611237554']
AcITi 6 ['Ti0.111111I0.777778Ac0.111111', '-1.298', '-0.229474454078']
AcITl 20 ['I0.375Tl0.25Ac0.375', '-1.043', '-0.200959342525']
AcITm 4 ['I0.714286Tm0.142857Ac0.142857', '-1.503', '-0.206108518619']
AcIV 3 ['V0.1I0.8Ac0.1', '-0.926', '-0.223359502034']
AcIW 3 ['I0.75W0.125Ac0.125', '-0.881', '-0.21572400315']
AcIXe 8 ['I0.714286Xe0.142857Ac0.142857', '-0.962', '-0.201684575029']
AcIZn 4 ['Zn0.166667I0.666667Ac0.166667', '-1.284', '-0.248878814629']
AcIZr 2 ['Zr0.1I0.8Ac0.1', '-1.17', '-0.20213795404']
AcInN 41 ['N0.25In0.125Ac0.625', '-0.618', '-0.214990327102']
AcInO 7 ['O0.7In0.1Ac0.2', '-2.543', '-0.273886794853']
AcInP 4 ['P0.5In0.1Ac0.4', '-1.261', '-0.20817624152']
AcInPt 2 ['In0.1Pt0.4Ac0.5', '-1.096', '-0.20455170725']
AcInS 1 ['S0.444444In0.111111Ac0.444444', '-2.16', '-0.236463550621']
AcInSe 23 ['Se0.555556In0.333333Ac0.111111', '-0.958', '-0.206013427778']
AcInTe 15 ['In0.25Te0.625Ac0.125', '-0.771', '-0.208143548407']
AcIrN 34 ['N0.333333Ir0.333333Ac0.333333', '-0.597', '-0.203016792385']
AcIrO 10 ['O0.714286Ir0.142857Ac0.142857', '-1.996', '-0.261375108844']
AcIrPt 3 ['Ir0.142857Pt0.285714Ac0.571429', '-0.94', '-0.242310475357']
AcIrS 3 ['S0.444444Ir0.111111Ac0.444444', '-2.127', '-0.211204972658']
AcIrSe 23 ['Se0.375Ir0.125Ac0.5', '-1.54', '-0.20373968125']
AcKN 6 ['N0.444444K0.222222Ac0.333333', '-0.665', '-0.227226637532']
AcKO 9 ['O0.625K0.25Ac0.125', '-2.091', '-0.205677425385']
AcKS 3 ['S0.5K0.333333Ac0.166667', '-1.841', '-0.228272305216']
AcKSe 20 ['K0.375Se0.5Ac0.125', '-1.331', '-0.205884891563']
AcKTe 8 ['K0.166667Te0.5Ac0.333333', '-1.421', '-0.210295306387']
AcKrN 11 ['N0.3Kr0.2Ac0.5', '-0.58', '-0.225415113147']
AcKrO 7 ['O0.7Kr0.1Ac0.2', '-1.99', '-0.200117911735']

AcKrP 9 ['P0.375Kr0.125Ac0.5', '-1.122', '-0.200789540078']
AcKrSe 16 ['Se0.555556Kr0.222222Ac0.222222', '-1.045', '-0.211683472222']
AcLaN 3 ['N0.5La0.125Ac0.375', '-1.033', '-0.233157779516']
AcLaO 7 ['O0.714286La0.142857Ac0.142857', '-2.837', '-0.232606316139']
AcLaP 5 ['P0.5La0.166667Ac0.333333', '-1.586', '-0.200956186353']
AcLaPt 4 ['La0.142857Pt0.428571Ac0.428571', '-1.144', '-0.229439485714']
AcLaSe 1 ['Se0.625La0.125Ac0.25', '-1.836', '-0.313241440313']
AcLaTe 3 ['Te0.625La0.125Ac0.25', '-1.461', '-0.221890374478']
AcLiN 32 ['Li0.142857N0.285714Ac0.571429', '-0.564', '-0.201497898592']
AcLiO 6 ['Li0.166667O0.666667Ac0.166667', '-2.307', '-0.211070250245']
AcLiP 19 ['Li0.25P0.375Ac0.375', '-1.184', '-0.212887268619']
AcLiPt 2 ['Li0.166667Pt0.333333Ac0.5', '-0.882', '-0.210807934167']
AcLiS 15 ['Li0.166667S0.5Ac0.333333', '-2.258', '-0.217783010075']
AcLiSe 21 ['Li0.333333Se0.5Ac0.166667', '-1.438', '-0.203192696252']
AcLiTe 3 ['Li0.142857Te0.571429Ac0.285714', '-1.299', '-0.250745543094']
AcLuN 5 ['N0.5Lu0.166667Ac0.333333', '-1.261', '-0.205831933995']
AcLuO 9 ['O0.6Lu0.3Ac0.1', '-4.08', '-0.20270639147']
AcLuSe 15 ['Se0.6Lu0.2Ac0.2', '-1.841', '-0.225102512']
AcLuTe 3 ['Te0.6Lu0.1Ac0.3', '-1.405', '-0.249265197749']
AcMgN 16 ['N0.4Mg0.3Ac0.3', '-0.896', '-0.228501122988']
AcMgO 9 ['O0.571429Mg0.142857Ac0.285714', '-3.615', '-0.216327473187']
AcMgP 4 ['Mg0.1P0.5Ac0.4', '-1.33', '-0.205258784428']
AcMgPt 2 ['Mg0.125Pt0.375Ac0.5', '-1.011', '-0.234009988854']
AcMgS 16 ['Mg0.375S0.5Ac0.125', '-1.928', '-0.204451192367']
AcMgSe 16 ['Mg0.1Se0.4Ac0.5', '-1.532', '-0.203359747333']
AcMgTe 2 ['Mg0.111111Te0.555556Ac0.333333', '-1.35', '-0.210597090183']
AcMnN 30 ['N0.571429Mn0.285714Ac0.142857', '-0.549', '-0.206872328412']
AcMnO 6 ['O0.6Mn0.2Ac0.2', '-3.221', '-0.30265737553']
AcMnP 1 ['P0.4Mn0.1Ac0.5', '-1.23', '-0.247375509416']
AcMnSe 10 ['Mn0.2Se0.6Ac0.2', '-1.081', '-0.217183574741']
AcMnTe 1 ['Mn0.1Te0.6Ac0.3', '-1.097', '-0.210211769119']
AcMoN 11 ['N0.4Mo0.2Ac0.4', '-0.675', '-0.202220150862']
AcMoO 10 ['O0.625Mo0.25Ac0.125', '-2.865', '-0.332244747577']
AcMoSe 11 ['Se0.5Mo0.2Ac0.3', '-1.395', '-0.21019103175']
AcNNa 29 ['N0.375Na0.375Ac0.25', '-0.548', '-0.204679130669']
AcNNb 8 ['N0.5Nb0.2Ac0.3', '-1.038', '-0.212359874079']
AcNNd 1 ['N0.5Nd0.1Ac0.4', '-1.016', '-0.250440152828']
AcNNi 43 ['N0.285714Ni0.428571Ac0.285714', '-0.538', '-0.200300107759']
AcNNp 27 ['N0.375Ac0.5Np0.125', '-0.825', '-0.226398291276']
AcNO 45 ['N0.142857O0.714286Ac0.142857', '-1.71', '-0.214402694511']
AcNOs 24 ['N0.25Os0.25Ac0.5', '-0.512', '-0.216512594289']
AcNP 58 ['N0.625P0.25Ac0.125', '-0.699', '-0.202302474761']
AcNPa 24 ['N0.4Ac0.5Pa0.1', '-0.795', '-0.203427954195']
AcNPb 13 ['N0.444444Pb0.222222Ac0.333333', '-0.628', '-0.200451685374']

AcNPd 31 ['N0.142857Pd0.285714Ac0.571429', '-0.784', '-0.207308911737']
AcNPm 2 ['N0.5Pm0.125Ac0.375', '-1.059', '-0.230383869203']
AcNPr 4 ['N0.5Pr0.166667Ac0.333333', '-1.063', '-0.204667716912']
AcNPt 49 ['N0.125Pt0.5Ac0.375', '-1.021', '-0.212381260312']
AcNPu 27 ['N0.6Ac0.2Pu0.2', '-0.991', '-0.206961268363']
AcNRb 9 ['N0.428571Rb0.285714Ac0.285714', '-0.595', '-0.201780179376']
AcNRe 14 ['N0.5Re0.25Ac0.25', '-0.544', '-0.21945662176']
AcNRh 42 ['N0.222222Rh0.333333Ac0.444444', '-0.7', '-0.201066584368']
AcNRu 20 ['N0.222222Ru0.222222Ac0.555556', '-0.471', '-0.208344528257']
AcNS 21 ['N0.125S0.375Ac0.5', '-1.966', '-0.201804242825']
AcNSb 26 ['N0.111111Sb0.444444Ac0.444444', '-1.06', '-0.20096375']
AcNSc 10 ['N0.4Sc0.1Ac0.5', '-0.963', '-0.204296246113']
AcNSe 39 ['N0.3Se0.4Ac0.3', '-1.22', '-0.20518439825']
AcNSi 31 ['N0.5Si0.3Ac0.2', '-1.181', '-0.212646768581']
AcNSm 4 ['N0.4Sm0.1Ac0.5', '-0.879', '-0.207121104863']
AcNSn 20 ['N0.25Sn0.25Ac0.5', '-0.831', '-0.207677480701']
AcNSr 8 ['N0.444444Sr0.333333Ac0.222222', '-0.832', '-0.223411147903']
AcNTa 11 ['N0.571429Ta0.142857Ac0.285714', '-1.009', '-0.221526594106']
AcNTb 2 ['N0.5Tb0.125Ac0.375', '-1.095', '-0.292702363931']
AcNTc 21 ['N0.6Tc0.2Ac0.2', '-0.497', '-0.208276014897']
AcNTe 34 ['N0.5Te0.125Ac0.375', '-0.851', '-0.207134282934']
AcNTh 9 ['N0.5Ac0.2Th0.3', '-1.535', '-0.205345706578']
AcNTi 18 ['N0.444444Ti0.333333Ac0.222222', '-1.328', '-0.200653313585']
AcNTl 15 ['N0.285714Tl0.142857Ac0.571429', '-0.658', '-0.219291730259']
AcNTm 6 ['N0.4Tm0.1Ac0.5', '-0.943', '-0.202549338613']
AcNU 9 ['N0.5Ac0.25U0.25', '-1.326', '-0.204606348578']
AcNV 17 ['N0.571429V0.285714Ac0.142857', '-1.097', '-0.212203840925']
AcNW 14 ['N0.5W0.2Ac0.3', '-0.632', '-0.206280360078']
AcNXe 14 ['N0.333333Xe0.333333Ac0.333333', '-0.594', '-0.200016792385']
AcNY 2 ['N0.5Y0.1Ac0.4', '-1.105', '-0.273175601828']
AcNYb 9 ['N0.5Yb0.25Ac0.25', '-0.934', '-0.202101443397']
AcNZn 34 ['N0.375Zn0.5Ac0.125', '-0.387', '-0.205123999679']
AcNZr 6 ['N0.5Zr0.25Ac0.25', '-1.419', '-0.230928310454']
AcNaO 6 ['O0.571429Na0.142857Ac0.285714', '-3.18', '-0.242190231516']
AcNaP 10 ['Na0.125P0.5Ac0.375', '-1.268', '-0.204870211039']
AcNaPt 7 ['Na0.2Pt0.4Ac0.4', '-1.007', '-0.201569521']
AcNaS 10 ['Na0.2S0.4Ac0.4', '-1.931', '-0.206784475392']
AcNaSb 2 ['Na0.1Sb0.4Ac0.5', '-0.988', '-0.209608145831']
AcNaSe 25 ['Na0.111111Se0.333333Ac0.555556', '-1.309', '-0.207332111156']
AcNaTe 9 ['Na0.166667Te0.5Ac0.333333', '-1.402', '-0.209155011523']
AcNbO 10 ['O0.7Nb0.2Ac0.1', '-3.203', '-0.323204559426']
AcNbSe 11 ['Se0.6Nb0.3Ac0.1', '-1.216', '-0.200973474625']
AcNdO 6 ['O0.666667Nd0.222222Ac0.111111', '-3.369', '-0.292949820575']
AcNdP 2 ['P0.5Nd0.125Ac0.375', '-1.554', '-0.216139920833']

AcNdPt 1 ['Nd0.1Pt0.4Ac0.5', '-1.075', '-0.2411544045']
AcNdSe 5 ['Se0.571429Nd0.142857Ac0.285714', '-1.842', '-0.204917427144']
AcNdTe 3 ['Te0.6Nd0.1Ac0.3', '-1.477', '-0.243656781748']
AcNiO 8 ['O0.571429Ni0.142857Ac0.285714', '-3.171', '-0.259307575004']
AcNiP 1 ['P0.8Ni0.1Ac0.1', '-0.711', '-0.270720526166']
AcNiS 1 ['S0.4Ni0.1Ac0.5', '-1.97', '-0.218584662642']
AcNiSe 19 ['Ni0.375Se0.375Ac0.25', '-1.109', '-0.201261706875']
AcNpO 7 ['O0.7Ac0.1Np0.2', '-3.342', '-0.220813759867']
AcNpP 1 ['P0.5Ac0.4Np0.1', '-1.424', '-0.243710908645']
AcNpSe 8 ['Se0.6Ac0.1Np0.3', '-1.396', '-0.212758211126']
AcNpTe 2 ['Te0.666667Ac0.222222Np0.111111', '-1.074', '-0.218273974027']
AcOOs 10 ['O0.7Os0.2Ac0.1', '-2.018', '-0.212970311259']
AcOP 27 ['O0.25P0.25Ac0.5', '-2.309', '-0.203291286498']
AcOPa 9 ['O0.625Ac0.125Pa0.25', '-3.469', '-0.316736077418']
AcOPb 9 ['O0.714286Pb0.142857Ac0.142857', '-2.0', '-0.243842404561']
AcOPd 8 ['O0.714286Pd0.142857Ac0.142857', '-1.777', '-0.226012077836']
AcOPm 4 ['O0.7Pm0.1Ac0.2', '-2.919', '-0.20995867954']
AcOPr 4 ['O0.7Pr0.1Ac0.2', '-3.023', '-0.33910069429']
AcOPt 7 ['O0.7Pt0.1Ac0.2', '-2.351', '-0.338857565559']
AcOPu 6 ['O0.6Ac0.3Pu0.1', '-3.814', '-0.220952648728']
AcORb 6 ['O0.666667Rb0.222222Ac0.111111', '-1.936', '-0.216354384689']
AcORe 9 ['O0.7Re0.2Ac0.1', '-2.541', '-0.249394233759']
AcORh 9 ['O0.666667Rh0.222222Ac0.111111', '-1.922', '-0.235753703908']
AcORu 8 ['O0.714286Ru0.142857Ac0.142857', '-2.099', '-0.220466449461']
AcOS 38 ['O0.2S0.3Ac0.5', '-2.689', '-0.202583631034']
AcOSb 11 ['O0.125Sb0.375Ac0.5', '-1.695', '-0.224403960619']
AcOSc 9 ['O0.6Sc0.3Ac0.1', '-4.011', '-0.22627421172']
AcOSe 40 ['O0.142857Se0.571429Ac0.285714', '-1.8', '-0.203427910826']
AcOSi 10 ['O0.7Si0.2Ac0.1', '-2.948', '-0.208925387517']
AcOSm 8 ['O0.6Sm0.2Ac0.2', '-3.858', '-0.217602544345']
AcOSn 7 ['O0.666667Sn0.222222Ac0.111111', '-2.52', '-0.223297635715']
AcOSr 5 ['O0.6Sr0.1Ac0.3', '-3.528', '-0.22486343622']
AcOTa 9 ['O0.7Ta0.2Ac0.1', '-3.36', '-0.273894784426']
AcOTb 6 ['O0.625Tb0.25Ac0.125', '-3.557', '-0.213156488809']
AcOTc 11 ['O0.625Tc0.25Ac0.125', '-2.46', '-0.225375440463']
AcOTe 10 ['O0.125Te0.5Ac0.375', '-1.85', '-0.263759758951']
AcOTh 6 ['O0.7Ac0.1Th0.2', '-3.713', '-0.275910810267']
AcOTi 10 ['O0.714286Ti0.142857Ac0.142857', '-2.896', '-0.227809554732']
AcOTl 6 ['O0.625Tl0.25Ac0.125', '-1.941', '-0.229067945129']
AcOTm 9 ['O0.6Tm0.3Ac0.1', '-4.061', '-0.20917586672']
AcOU 8 ['O0.6Ac0.3U0.1', '-3.799', '-0.254834638968']
AcOV 12 ['O0.6V0.3Ac0.1', '-3.165', '-0.23920415889']
AcOW 9 ['O0.7W0.2Ac0.1', '-2.921', '-0.232079851883']
AcOXe 5 ['O0.571429Xe0.142857Ac0.285714', '-2.762', '-0.205025588193']

AcOY 9 ['O0.714286Y0.142857Ac0.142857', '-2.869', '-0.230443379621']
AcOYb 6 ['O0.6Yb0.2Ac0.2', '-3.431', '-0.237794184559']
AcOZn 8 ['O0.571429Zn0.142857Ac0.285714', '-3.231', '-0.202910077353']
AcOZr 9 ['O0.7Zr0.2Ac0.1', '-3.29', '-0.215269497513']
AcOsS 1 ['S0.4Os0.1Ac0.5', '-1.97', '-0.245784475392']
AcOsSe 15 ['Se0.625Os0.125Ac0.25', '-1.157', '-0.21951890625']
AcPPb 21 ['P0.5Pb0.4Ac0.1', '-0.452', '-0.206343877354']
AcPPm 19 ['P0.444444Pm0.111111Ac0.444444', '-1.302', '-0.210195010462']
AcPPr 4 ['P0.555556Pr0.111111Ac0.333333', '-1.432', '-0.215096435161']
AcPPt 15 ['P0.125Pt0.375Ac0.5', '-1.271', '-0.20883877263']
AcPPu 25 ['P0.625Ac0.125Pu0.25', '-1.156', '-0.203882421952']
AcPRb 1 ['P0.5Rb0.1Ac0.4', '-1.289', '-0.207996929187']
AcPRh 3 ['P0.714286Rh0.142857Ac0.142857', '-1.001', '-0.20933149988']
AcPRu 1 ['P0.8Ru0.1Ac0.1', '-0.778', '-0.210675600437']
AcPS 7 ['P0.3S0.3Ac0.4', '-1.86', '-0.215567067503']
AcPSb 16 ['P0.3Sb0.2Ac0.5', '-1.325', '-0.201465319562']
AcPSc 1 ['P0.5Sc0.1Ac0.4', '-1.51', '-0.20356072302']
AcPSe 45 ['P0.333333Se0.444444Ac0.222222', '-1.034', '-0.20320456716']
AcPSi 10 ['Si0.2P0.4Ac0.4', '-1.184', '-0.201375509416']
AcPSn 15 ['P0.333333Sn0.166667Ac0.5', '-1.238', '-0.200589515455']
AcPSr 8 ['P0.555556Sr0.111111Ac0.333333', '-1.38', '-0.241572819967']
AcPTb 1 ['P0.555556Tb0.111111Ac0.333333', '-1.426', '-0.293955778556']
AcPTe 67 ['P0.375Te0.5Ac0.125', '-0.583', '-0.201533715313']
AcPTi 1 ['P0.444444Ti0.111111Ac0.444444', '-1.352', '-0.201853209152']
AcPTl 20 ['P0.4Tl0.4Ac0.2', '-0.756', '-0.208569843516']
AcPTm 2 ['P0.555556Tm0.111111Ac0.333333', '-1.457', '-0.228343667383']
AcPXe 8 ['P0.75Xe0.125Ac0.125', '-0.512', '-0.204929846693']
AcPY 7 ['P0.555556Y0.222222Ac0.222222', '-1.562', '-0.209066122106']
AcPYb 5 ['P0.5Yb0.1Ac0.4', '-1.404', '-0.21978707452']
AcPZn 24 ['P0.333333Zn0.166667Ac0.5', '-1.115', '-0.210230543263']
AcPaS 2 ['S0.7Ac0.1Pa0.2', '-1.621', '-0.226903339693']
AcPaSe 15 ['Se0.571429Ac0.142857Pa0.285714', '-1.414', '-0.212764011071']
AcPaSi 4 ['Si0.75Ac0.125Pa0.125', '-0.382', '-0.224252714062']
AcPaTe 5 ['Te0.7Ac0.2Pa0.1', '-0.949', '-0.212492704167']
AcPbPt 6 ['Pt0.4Pb0.2Ac0.4', '-1.054', '-0.205733477']
AcPbS 2 ['S0.555556Pb0.222222Ac0.222222', '-1.79', '-0.204401839991']
AcPbSe 9 ['Se0.4Pb0.1Ac0.5', '-1.612', '-0.20455725325']
AcPbTe 5 ['Te0.5Pb0.125Ac0.375', '-1.365', '-0.217806074268']
AcPdPt 2 ['Pd0.1Pt0.3Ac0.6', '-0.973', '-0.226182741']
AcPdSe 35 ['Se0.625Pd0.125Ac0.25', '-1.193', '-0.212721659375']
AcPmPt 1 ['Pm0.1Pt0.4Ac0.5', '-1.021', '-0.2011089095']
AcPmS 6 ['S0.5Pm0.25Ac0.25', '-2.215', '-0.20219071549']
AcPmSe 15 ['Se0.714286Pm0.142857Ac0.142857', '-1.223', '-0.201677335714']
AcPmTe 16 ['Te0.7Pm0.2Ac0.1', '-1.029', '-0.201804053082']

AcPrPt 1 ['Pr0.1Pt0.4Ac0.5', '-1.058', '-0.230255887']
AcPrSe 2 ['Se0.6Pr0.1Ac0.3', '-1.753', '-0.24333476895']
AcPrTe 4 ['Te0.7Pr0.1Ac0.2', '-1.188', '-0.203568136418']
AcPtRh 3 ['Rh0.125Pt0.25Ac0.625', '-0.839', '-0.202699607187']
AcPtS 30 ['S0.428571Pt0.142857Ac0.428571', '-2.136', '-0.201030990064']
AcPtSe 53 ['Se0.6Pt0.3Ac0.1', '-0.83', '-0.205993622834']
AcPtSi 7 ['Si0.111111Pt0.333333Ac0.555556', '-0.943', '-0.200063346111']
AcPtSn 2 ['Sn0.111111Pt0.333333Ac0.555556', '-1.04', '-0.223103439239']
AcPtSr 2 ['Sr0.1Pt0.4Ac0.5', '-1.057', '-0.251569521']
AcPtTh 3 ['Pt0.7Ac0.1Th0.2', '-0.979', '-0.20346627225']
AcPtTl 3 ['Pt0.5Tl0.125Ac0.375', '-1.02', '-0.206860418437']
AcPtYb 2 ['Yb0.1Pt0.4Ac0.5', '-1.078', '-0.225784728']
AcPuS 13 ['S0.444444Ac0.333333Pu0.222222', '-2.074', '-0.210758763491']
AcPuSe 17 ['Se0.4Ac0.4Pu0.2', '-1.514', '-0.202878897']
AcPuTe 3 ['Te0.571429Ac0.285714Pu0.142857', '-1.395', '-0.202753575951']
AcRbSe 25 ['Se0.666667Rb0.166667Ac0.166667', '-1.171', '-0.206115532083']
AcRbTe 9 ['Rb0.166667Te0.666667Ac0.166667', '-1.01', '-0.210669882361']
AcReSe 3 ['Se0.571429Re0.142857Ac0.285714', '-1.315', '-0.209581953929']
AcRhS 1 ['S0.4Rh0.2Ac0.4', '-1.926', '-0.201784475392']
AcRhSe 30 ['Se0.428571Rh0.428571Ac0.142857', '-0.849', '-0.205743509286']
AcRuSe 18 ['Se0.625Ru0.125Ac0.25', '-1.22', '-0.201333585938']
AcSSb 28 ['S0.3Sb0.2Ac0.5', '-1.908', '-0.228272044044']
AcSSe 18 ['S0.333333Se0.333333Ac0.333333', '-1.916', '-0.200610542272']
AcSSi 27 ['Si0.2S0.5Ac0.3', '-2.009', '-0.214739324867']
AcSSn 7 ['S0.4Sn0.2Ac0.4', '-1.994', '-0.211074225689']
AcSTc 1 ['S0.555556Tc0.333333Ac0.111111', '-1.333', '-0.221559875548']
AcSTe 25 ['S0.166667Te0.333333Ac0.5', '-1.857', '-0.209568034467']
AcSTl 3 ['S0.5Tl0.125Ac0.375', '-2.191', '-0.210781789554']
AcSZr 2 ['S0.555556Zr0.333333Ac0.111111', '-1.971', '-0.219139303878']
AcSbSe 48 ['Se0.5Sb0.3Ac0.2', '-0.989', '-0.2011410075']
AcSbTe 9 ['Sb0.222222Te0.333333Ac0.444444', '-1.37', '-0.205207715553']
AcScSe 24 ['Sc0.25Se0.5Ac0.25', '-1.824', '-0.207543434375']
AcSeSi 40 ['Si0.333333Se0.5Ac0.166667', '-0.974', '-0.20129642625']
AcSeSm 3 ['Se0.555556Sm0.111111Ac0.333333', '-1.865', '-0.227689080556']
AcSeSn 23 ['Se0.375Sn0.25Ac0.375', '-1.434', '-0.204823965937']
AcSeSr 6 ['Se0.6Sr0.2Ac0.2', '-1.788', '-0.2404558615']
AcSeTa 9 ['Se0.666667Ta0.222222Ac0.111111', '-1.169', '-0.224794621944']
AcSeTb 5 ['Se0.571429Tb0.142857Ac0.285714', '-1.759', '-0.222041047189']
AcSeTc 15 ['Se0.625Tc0.125Ac0.25', '-1.142', '-0.20451890625']
AcSeTe 66 ['Se0.166667Te0.333333Ac0.5', '-1.678', '-0.202690710137']
AcSeTi 9 ['Ti0.142857Se0.428571Ac0.428571', '-1.611', '-0.206227389643']
AcSeTl 29 ['Se0.571429Tl0.285714Ac0.142857', '-0.908', '-0.207406640786']
AcSeTm 8 ['Se0.571429Tm0.142857Ac0.285714', '-1.785', '-0.200283462144']
AcSeU 4 ['Se0.666667Ac0.166667U0.166667', '-1.37', '-0.203388936417']

AcSeV 6 ['V0.1Se0.5Ac0.4', '-1.657', '-0.219736841']
AcSeW 1 ['Se0.6W0.1Ac0.3', '-1.44', '-0.25867462275']
AcSeXe 10 ['Se0.428571Xe0.285714Ac0.285714', '-1.193', '-0.211520962857']
AcSeY 16 ['Se0.714286Y0.142857Ac0.142857', '-1.267', '-0.204271328214']
AcSeYb 3 ['Se0.6Yb0.1Ac0.3', '-1.726', '-0.25847774875']
AcSeZn 24 ['Zn0.444444Se0.444444Ac0.111111', '-1.045', '-0.201669890278']
AcSeZr 22 ['Se0.5Zr0.4Ac0.1', '-1.454', '-0.218470588139']
AcSiTe 3 ['Si0.1Te0.5Ac0.4', '-1.336', '-0.207954214331']
AcSmTe 5 ['Te0.571429Sm0.142857Ac0.285714', '-1.553', '-0.23003384702']
AcSnTe 11 ['Sn0.166667Te0.5Ac0.333333', '-1.248', '-0.208347218023']
AcSrTe 4 ['Sr0.125Te0.625Ac0.25', '-1.384', '-0.21747940604']
AcTbTe 5 ['Te0.6Tb0.3Ac0.1', '-1.25', '-0.203033651849']
AcTeTh 4 ['Te0.7Ac0.2Th0.1', '-1.155', '-0.220137102882']
AcTeTl 11 ['Te0.555556Tl0.222222Ac0.222222', '-0.944', '-0.200355017592']
AcTeTm 3 ['Te0.6Tm0.1Ac0.3', '-1.384', '-0.212476634249']
AcTeU 4 ['Te0.7Ac0.2U0.1', '-1.048', '-0.252456758166']
AcTeY 5 ['Y0.166667Te0.666667Ac0.166667', '-1.278', '-0.209164939862']
AcTeYb 5 ['Te0.571429Yb0.142857Ac0.285714', '-1.514', '-0.218841574521']
AcTeZn 1 ['Zn0.111111Te0.555556Ac0.333333', '-1.319', '-0.270859341572']
AcTeZr 2 ['Zr0.1Te0.6Ac0.3', '-1.333', '-0.218768566748']
AgAlF 1 ['F0.8Al0.1Ag0.1', '-2.205', '-0.204277444077']
AgAlI 17 ['Al0.222222Ag0.111111I0.666667', '-0.914', '-0.206100193937']
AgAlIr 1 ['Al0.6Ag0.1Ir0.3', '-0.884', '-0.2032628795']
AgAlO 3 ['O0.444444Al0.111111Ag0.444444', '-1.33', '-0.227200190723']
AgAlTe 1 ['Al0.125Ag0.25Te0.625', '-0.396', '-0.202705848646']
AgAsBa 9 ['As0.428571Ag0.142857Ba0.428571', '-0.986', '-0.207259215102']
AgAsBr 10 ['As0.125Br0.625Ag0.25', '-0.812', '-0.22486665474']
AgAsCa 1 ['Ca0.444444As0.333333Ag0.222222', '-1.125', '-0.222829335919']
AgAsDy 1 ['As0.375Ag0.25Dy0.375', '-1.383', '-0.231277379378']
AgAsEu 3 ['As0.375Ag0.25Eu0.375', '-1.213', '-0.218083609168']
AgAsI 22 ['As0.111111Ag0.444444I0.444444', '-0.533', '-0.205846235588']
AgAsK 9 ['K0.5As0.4Ag0.1', '-0.664', '-0.22247565825']
AgAsLa 1 ['As0.444444Ag0.222222La0.333333', '-1.3', '-0.230167342917']
AgAsNa 4 ['Na0.4As0.4Ag0.2', '-0.573', '-0.212933400159']
AgAsNd 6 ['As0.4Ag0.1Nd0.5', '-1.473', '-0.20805964275']
AgAsO 4 ['O0.625As0.25Ag0.125', '-1.488', '-0.272448831012']
AgAsPm 5 ['As0.5Ag0.1Pm0.4', '-1.108', '-0.244811021']
AgAsPr 3 ['As0.444444Ag0.222222Pr0.333333', '-1.226', '-0.2116658175']
AgAsSm 1 ['As0.444444Ag0.222222Sm0.333333', '-1.318', '-0.292989240278']
AgAsSr 4 ['As0.4Sr0.5Ag0.1', '-1.26', '-0.226017078001']
AgAuPa 2 ['Ag0.1Au0.7Pa0.2', '-0.33', '-0.20991492025']
AgAuPm 16 ['Ag0.111111Pm0.111111Au0.777778', '-0.385', '-0.200384551111']
AgBBr 7 ['B0.2Br0.7Ag0.1', '-0.921', '-0.213836269059']
AgBF 3 ['B0.125F0.75Ag0.125', '-2.301', '-0.216434168691']

AgBI 23 ['B0.25Ag0.25I0.5', '-0.447', '-0.200982728994']
AgBaF 9 ['F0.555556Ag0.333333Ba0.111111', '-2.358', '-0.207755270203']
AgBaH 6 ['H0.6Ag0.3Ba0.1', '-0.361', '-0.213790778313']
AgBaI 1 ['Ag0.4I0.5Ba0.1', '-1.011', '-0.217033367785']
AgBaO 12 ['O0.428571Ag0.285714Ba0.285714', '-1.878', '-0.202867377479']
AgBeF 5 ['Be0.142857F0.714286Ag0.142857', '-2.367', '-0.204514295701']
AgBiBr 11 ['Br0.5Ag0.333333Bi0.166667', '-0.871', '-0.202548599627']
AgBiI 10 ['Ag0.2I0.6Bi0.2', '-0.707', '-0.220496515543']
AgBiO 4 ['O0.666667Ag0.166667Bi0.166667', '-0.951', '-0.214635546454']
AgBrCa 1 ['Ca0.3Br0.5Ag0.2', '-1.964', '-0.208935166107']
AgBrCe 19 ['Br0.4Ag0.3Ce0.3', '-1.442', '-0.207941350199']
AgBrCr 9 ['Cr0.2Br0.6Ag0.2', '-0.91', '-0.20322618205']
AgBrDy 26 ['Br0.5Ag0.4Dy0.1', '-1.006', '-0.209995424876']
AgBrEr 40 ['Br0.428571Ag0.142857Er0.428571', '-1.137', '-0.23173073682']
AgBrEu 5 ['Br0.555556Ag0.222222Eu0.222222', '-1.932', '-0.20669016816']
AgBrF 10 ['F0.4Br0.4Ag0.2', '-1.075', '-0.201364330805']
AgBrFe 5 ['Fe0.166667Br0.666667Ag0.166667', '-0.816', '-0.205327699639']
AgBrGa 1 ['Ga0.2Br0.7Ag0.1', '-1.213', '-0.260251287556']
AgBrGd 9 ['Br0.6Ag0.3Gd0.1', '-1.374', '-0.221020821549']
AgBrGe 1 ['Ge0.142857Br0.714286Ag0.142857', '-0.976', '-0.203327196488']
AgBrH 1 ['H0.5Br0.2Ag0.3', '-0.412', '-0.214841277017']
AgBrHf 37 ['Br0.4Ag0.1Hf0.5', '-0.512', '-0.22115434845']
AgBrHo 40 ['Br0.375Ag0.25Ho0.375', '-1.063', '-0.22283168097']
AgBrIr 4 ['Br0.777778Ag0.111111Ir0.111111', '-0.577', '-0.217265527403']
AgBrLa 20 ['Br0.555556Ag0.111111La0.333333', '-1.957', '-0.206755346713']
AgBrLu 41 ['Br0.444444Ag0.111111Lu0.444444', '-1.076', '-0.20749676876']
AgBrMo 14 ['Br0.625Mo0.25Ag0.125', '-0.99', '-0.201035328386']
AgBrNb 2 ['Br0.75Nb0.125Ag0.125', '-1.163', '-0.204766738498']
AgBrNd 13 ['Br0.666667Ag0.166667Nd0.166667', '-1.793', '-0.203998979641']
AgBrNp 9 ['Br0.625Ag0.25Np0.125', '-1.256', '-0.215893139271']
AgBrO 15 ['O0.4Br0.4Ag0.2', '-0.466', '-0.201049682839']
AgBrOs 6 ['Br0.666667Ag0.222222Os0.111111', '-0.475', '-0.216699796417']
AgBrP 13 ['P0.285714Br0.428571Ag0.285714', '-0.649', '-0.201832933178']
AgBrPm 36 ['Br0.375Ag0.375Pm0.25', '-1.029', '-0.266537578155']
AgBrPr 25 ['Br0.375Ag0.375Pr0.25', '-1.391', '-0.211301182218']
AgBrPt 17 ['Br0.5Ag0.25Pt0.25', '-0.492', '-0.201412270969']
AgBrPu 41 ['Br0.428571Ag0.142857Pu0.428571', '-1.223', '-0.208947353966']
AgBrRe 8 ['Br0.666667Ag0.222222Re0.111111', '-0.549', '-0.290699796417']
AgBrRu 5 ['Br0.7Ru0.1Ag0.2', '-0.649', '-0.20100445855']
AgBrSb 9 ['Br0.666667Ag0.166667Sb0.166667', '-0.928', '-0.208381625056']
AgBrSc 38 ['Sc0.375Br0.375Ag0.25', '-0.895', '-0.203063674219']
AgBrSe 8 ['Se0.4Br0.4Ag0.2', '-0.494', '-0.201379799534']
AgBrSi 10 ['Si0.125Br0.625Ag0.25', '-1.002', '-0.204156201304']
AgBrSm 18 ['Br0.555556Ag0.333333Sm0.111111', '-1.368', '-0.205899496712']

AgBrSn 2 ['Br0.625Ag0.25Sn0.125', '-0.933', '-0.200202902009']
AgBrSr 1 ['Br0.7Sr0.2Ag0.1', '-1.789', '-0.255515109419']
AgBrTa 10 ['Br0.6Ag0.3Ta0.1', '-0.935', '-0.248080698384']
AgBrTb 26 ['Br0.8Ag0.1Tb0.1', '-0.832', '-0.261284435696']
AgBrTc 5 ['Br0.625Tc0.125Ag0.25', '-0.753', '-0.205749090365']
AgBrTe 65 ['Br0.285714Ag0.142857Te0.571429', '-0.46', '-0.203516684786']
AgBrTh 4 ['Br0.666667Ag0.111111Th0.222222', '-1.903', '-0.215546524503']
AgBrTi 1 ['Ti0.125Br0.75Ag0.125', '-1.284', '-0.226202253491']
AgBrTm 36 ['Br0.4Ag0.3Tm0.3', '-1.112', '-0.216068934118']
AgBrU 9 ['Br0.777778Ag0.111111U0.111111', '-1.335', '-0.203307551868']
AgBrV 10 ['V0.125Br0.625Ag0.25', '-0.89', '-0.216096702864']
AgBrW 8 ['Br0.7Ag0.1W0.2', '-0.864', '-0.200560069684']
AgBrY 39 ['Br0.4Y0.4Ag0.2', '-1.122', '-0.201040680533']
AgBrYb 26 ['Br0.333333Ag0.222222Yb0.444444', '-1.573', '-0.208674720028']
AgBrZr 12 ['Br0.555556Zr0.333333Ag0.111111', '-1.443', '-0.202868491714']
AgCDy 3 ['C0.3Ag0.2Dy0.5', '-0.503', '-0.204002955979']
AgCLa 4 ['C0.285714Ag0.285714La0.428571', '-0.43', '-0.208407337143']
AgCPu 7 ['C0.142857Ag0.428571Pu0.428571', '-0.208', '-0.200747624524']
AgCaF 1 ['F0.8Ca0.1Ag0.1', '-2.069', '-0.332900135991']
AgCaH 3 ['H0.5Ca0.375Ag0.125', '-0.725', '-0.209466267346']
AgCaO 5 ['O0.6Ca0.3Ag0.1', '-2.22', '-0.22399439022']
AgCdF 3 ['F0.777778Ag0.111111Cd0.111111', '-1.565', '-0.223879307767']
AgCdO 1 ['O0.625Ag0.25Cd0.125', '-0.654', '-0.204268853764']
AgCeI 42 ['Ag0.333333I0.333333Ce0.333333', '-0.917', '-0.219959551411']
AgCeO 6 ['O0.666667Ag0.166667Ce0.166667', '-1.78', '-0.215893876225']
AgClCo 1 ['Cl0.571429Co0.142857Ag0.285714', '-0.839', '-0.213103958932']
AgClCu 8 ['Cl0.5Cu0.2Ag0.3', '-0.803', '-0.20302502194']
AgClEr 42 ['Cl0.333333Ag0.333333Er0.333333', '-1.119', '-0.236066404793']
AgClEu 4 ['Cl0.5Ag0.125Eu0.375', '-2.437', '-0.206628361565']
AgClF 2 ['F0.555556Cl0.222222Ag0.222222', '-1.278', '-0.200627618796']
AgClHo 46 ['Cl0.3Ag0.4Ho0.3', '-1.02', '-0.207738714312']
AgClIn 6 ['Cl0.5Ag0.166667In0.333333', '-1.175', '-0.209876235524']
AgClIr 10 ['Cl0.571429Ag0.285714Ir0.142857', '-0.657', '-0.223906299021']
AgClK 5 ['Cl0.571429K0.142857Ag0.285714', '-1.261', '-0.216164011789']
AgClLa 7 ['Cl0.444444Ag0.222222La0.333333', '-1.891', '-0.200613433616']
AgClLu 51 ['Cl0.4Ag0.5Lu0.1', '-0.821', '-0.202149646002']
AgClMo 1 ['Cl0.666667Mo0.166667Ag0.166667', '-1.179', '-0.220342060377']
AgClNd 5 ['Cl0.444444Ag0.222222Nd0.333333', '-1.849', '-0.217016433987']
AgClNp 4 ['Cl0.666667Ag0.222222Np0.111111', '-1.525', '-0.210257973057']
AgClP 6 ['P0.166667Cl0.5Ag0.333333', '-0.903', '-0.223716908613']
AgClPm 37 ['Cl0.375Ag0.375Pm0.25', '-1.362', '-0.23398208508']
AgClPr 15 ['Cl0.571429Ag0.285714Pr0.142857', '-1.851', '-0.20214823536']
AgClPt 4 ['Cl0.7Ag0.1Pt0.2', '-0.719', '-0.204680893076']
AgClPu 36 ['Cl0.4Ag0.4Pu0.2', '-1.364', '-0.203777639755']

AgClRh 13 ['Cl0.571429Rh0.142857Ag0.285714', '-0.648', '-0.214906299021']
AgClRu 1 ['Cl0.666667Ru0.111111Ag0.222222', '-0.906', '-0.213006342921']
AgClSm 21 ['Cl0.7Ag0.2Sm0.1', '-1.291', '-0.220290850879']
AgClTa 3 ['Cl0.75Ag0.125Ta0.125', '-1.381', '-0.202277626759']
AgClTb 2 ['Cl0.5Ag0.166667Tb0.333333', '-1.839', '-0.229284865608']
AgClU 3 ['Cl0.7Ag0.2U0.1', '-1.594', '-0.201379635564']
AgClXe 7 ['Cl0.555556Ag0.111111Xe0.333333', '-0.372', '-0.203574671841']
AgClZr 1 ['Cl0.714286Zr0.142857Ag0.142857', '-1.86', '-0.210824268483']
AgCoF 7 ['F0.625Co0.125Ag0.25', '-1.764', '-0.211361337']
AgCoO 3 ['O0.6Co0.2Ag0.2', '-1.043', '-0.303679458976']
AgCrI 10 ['Cr0.25Ag0.25I0.5', '-0.54', '-0.201872281911']
AgCrO 4 ['O0.625Cr0.25Ag0.125', '-1.875', '-0.212190926198']
AgCrTe 5 ['Cr0.1Ag0.2Te0.7', '-0.29', '-0.204250579958']
AgCsF 9 ['F0.666667Ag0.166667Cs0.166667', '-1.986', '-0.22475013585']
AgCsH 1 ['H0.555556Ag0.333333Cs0.111111', '-0.279', '-0.244209587535']
AgCsO 2 ['O0.375Ag0.5Cs0.125', '-0.733', '-0.223628193212']
AgCsTe 2 ['Ag0.2Te0.7Cs0.1', '-0.46', '-0.22958314215']
AgCuF 3 ['F0.75Cu0.125Ag0.125', '-1.45', '-0.213273968115']
AgDyF 4 ['F0.8Ag0.1Dy0.1', '-2.355', '-0.233466441413']
AgDyI 32 ['Ag0.285714I0.428571Dy0.285714', '-0.885', '-0.204069088483']
AgDyO 2 ['O0.625Ag0.25Dy0.125', '-1.584', '-0.240177657955']
AgDyS 1 ['S0.428571Ag0.142857Dy0.428571', '-2.131', '-0.298405676851']
AgErF 1 ['F0.777778Ag0.111111Er0.111111', '-2.7', '-0.203368398143']
AgErI 31 ['Ag0.222222I0.444444Er0.333333', '-0.99', '-0.200025824549']
AgEuF 1 ['F0.8Ag0.1Eu0.1', '-2.127', '-0.28680877999']
AgEuH 1 ['H0.6Ag0.3Eu0.1', '-0.4', '-0.207266868563']
AgEuI 13 ['Ag0.125I0.625Eu0.25', '-1.703', '-0.209731235042']
AgEuN 16 ['N0.2Ag0.3Eu0.5', '-0.714', '-0.208526782931']
AgEuO 3 ['O0.625Ag0.25Eu0.125', '-1.332', '-0.31353574973']
AgEuP 12 ['P0.4Ag0.2Eu0.4', '-1.258', '-0.202863020085']
AgEuSe 4 ['Se0.375Ag0.125Eu0.5', '-1.972', '-0.20262130625']
AgEuSi 2 ['Si0.4Ag0.2Eu0.4', '-0.773', '-0.200361532']
AgEuSn 1 ['Ag0.333333Sn0.333333Eu0.333333', '-0.787', '-0.212159384716']
AgFFe 2 ['F0.777778Fe0.111111Ag0.111111', '-1.871', '-0.285062697863']
AgFGa 3 ['F0.625Ga0.125Ag0.25', '-2.228', '-0.21279996179']
AgFGd 5 ['F0.666667Ag0.222222Gd0.111111', '-2.788', '-0.217261595858']
AgFH 17 ['H0.25F0.625Ag0.125', '-1.615', '-0.214980576941']
AgFHf 1 ['F0.777778Ag0.111111Hf0.111111', '-3.008', '-0.209998828519']
AgFHg 3 ['F0.75Ag0.125Hg0.125', '-1.439', '-0.230416496867']
AgFHo 1 ['F0.8Ag0.1Ho0.1', '-2.449', '-0.201793850745']
AgFI 1 ['F0.75Ag0.125I0.125', '-1.764', '-0.228136372846']
AgFIn 2 ['F0.777778Ag0.111111In0.111111', '-1.981', '-0.22400452953']
AgFIr 2 ['F0.75Ag0.125Ir0.125', '-1.808', '-0.222740566222']
AgFK 7 ['F0.571429K0.142857Ag0.285714', '-1.994', '-0.218213746926']

AgFKr 2 ['F0.777778Kr0.111111Ag0.111111', '-0.83', '-0.203513870545']
AgFLa 3 ['F0.75Ag0.125La0.125', '-3.034', '-0.206535889681']
AgFLi 5 ['Li0.2F0.7Ag0.1', '-2.005', '-0.274771432989']
AgFLu 23 ['F0.555556Ag0.111111Lu0.333333', '-3.281', '-0.204895004463']
AgFMg 2 ['F0.777778Mg0.111111Ag0.111111', '-2.091', '-0.294077806747']
AgFN 2 ['N0.111111F0.777778Ag0.111111', '-0.791', '-0.268469040907']
AgFNa 11 ['F0.5Na0.125Ag0.375', '-1.819', '-0.206870480012']
AgFNi 3 ['F0.777778Ni0.111111Ag0.111111', '-1.579', '-0.249633915085']
AgFNp 4 ['F0.7Ag0.2Np0.1', '-2.812', '-0.274196469065']
AgFO 8 ['O0.166667F0.666667Ag0.166667', '-1.014', '-0.230203561361']
AgFPa 10 ['F0.7Ag0.1Pa0.2', '-3.329', '-0.219464865856']
AgFPb 2 ['F0.8Ag0.1Pb0.1', '-1.758', '-0.224254234167']
AgFPd 2 ['F0.8Pd0.1Ag0.1', '-1.391', '-0.210617411167']
AgFPr 4 ['F0.428571Ag0.142857Pr0.428571', '-2.794', '-0.215418325017']
AgFPu 12 ['F0.5Ag0.2Pu0.3', '-2.806', '-0.204056157933']
AgFRb 10 ['F0.6Rb0.1Ag0.3', '-1.912', '-0.20576293377']
AgFRh 3 ['F0.777778Rh0.111111Ag0.111111', '-1.598', '-0.277967796752']
AgFRu 1 ['F0.8Ru0.1Ag0.1', '-1.81', '-0.209926545112']
AgFS 1 ['F0.555556S0.111111Ag0.333333', '-1.561', '-0.20134907365']
AgFSc 5 ['F0.777778Sc0.111111Ag0.111111', '-2.628', '-0.207765213974']
AgFSe 3 ['F0.375Se0.25Ag0.375', '-1.163', '-0.201445120078']
AgFSn 2 ['F0.8Ag0.1Sn0.1', '-2.015', '-0.214976167477']
AgFSr 6 ['F0.571429Sr0.142857Ag0.285714', '-2.67', '-0.20562016835']
AgFTc 2 ['F0.777778Tc0.111111Ag0.111111', '-2.033', '-0.268961008657']
AgFTh 2 ['F0.8Ag0.1Th0.1', '-2.926', '-0.225535520332']
AgFTi 4 ['F0.625Ti0.125Ag0.25', '-2.631', '-0.200476387196']
AgFTl 6 ['F0.5Ag0.125Tl0.375', '-1.94', '-0.201856335744']
AgFTm 53 ['F0.333333Ag0.166667Tm0.5', '-1.888', '-0.239796578625']
AgFV 4 ['F0.75V0.125Ag0.125', '-2.708', '-0.314790871591']
AgFXe 1 ['F0.714286Ag0.142857Xe0.142857', '-1.221', '-0.202139231155']
AgFY 7 ['F0.571429Y0.142857Ag0.285714', '-2.969', '-0.204742018113']
AgFYb 45 ['F0.3Ag0.2Yb0.5', '-2.023', '-0.220273206293']
AgFZn 3 ['F0.6Zn0.1Ag0.3', '-1.865', '-0.211696342271']
AgFeO 3 ['O0.6Fe0.2Ag0.2', '-1.226', '-0.267689638464']
AgGaO 3 ['O0.625Ga0.125Ag0.25', '-1.016', '-0.218797378708']
AgGaPa 4 ['Ga0.714286Ag0.142857Pa0.142857', '-0.299', '-0.214790127381']
AgGaPm 2 ['Ga0.666667Ag0.111111Pm0.222222', '-0.468', '-0.207509107963']
AgGaTe 2 ['Ga0.125Ag0.25Te0.625', '-0.371', '-0.213112743646']
AgGdI 34 ['Ag0.1I0.5Gd0.4', '-1.103', '-0.202548831033']
AgGdO 4 ['O0.625Ag0.125Gd0.25', '-2.655', '-0.214264208168']
AgGeO 4 ['O0.625Ge0.25Ag0.125', '-1.688', '-0.205206137263']
AgGePa 1 ['Ge0.6Ag0.1Pa0.3', '-0.397', '-0.2054533615']
AgGeTe 7 ['Ge0.222222Ag0.111111Te0.666667', '-0.257', '-0.207099130741']
AgHI 7 ['H0.5Ag0.2I0.3', '-0.366', '-0.205454382053']

AgHK 6 ['H0.571429K0.142857Ag0.285714', '-0.265', '-0.208465163259']
AgHO 5 ['H0.375O0.375Ag0.25', '-0.977', '-0.201132794322']
AgHPm 27 ['H0.666667Ag0.166667Pm0.166667', '-0.448', '-0.200150785158']
AgHPu 19 ['H0.5Ag0.25Pu0.25', '-0.458', '-0.203073068908']
AgHYb 5 ['H0.571429Ag0.285714Yb0.142857', '-0.468', '-0.20416228009']
AgHfO 4 ['O0.625Ag0.125Hf0.25', '-3.158', '-0.21248812226']
AgHfS 2 ['S0.444444Ag0.111111Hf0.444444', '-1.492', '-0.204369869329']
AgHfTe 1 ['Ag0.2Te0.7Hf0.1', '-0.497', '-0.20616513']
AgHoI 34 ['Ag0.5I0.375Ho0.125', '-0.627', '-0.200519523204']
AgHoN 2 ['N0.375Ag0.125Ho0.5', '-1.678', '-0.205804277891']
AgHoOs 3 ['Ag0.111111Ho0.555556Os0.333333', '-0.363', '-0.245551605463']
AgHoTe 3 ['Ag0.125Te0.5Ho0.375', '-1.309', '-0.231665075523']
AgIIn 10 ['Ag0.444444In0.111111I0.444444', '-0.584', '-0.208650106837']
AgILu 37 ['Ag0.375I0.375Lu0.25', '-0.661', '-0.23535866336']
AgIMg 1 ['Mg0.3Ag0.1I0.6', '-1.262', '-0.204965391791']
AgINb 4 ['Nb0.222222Ag0.111111I0.666667', '-0.734', '-0.21522394727']
AgINd 20 ['Ag0.142857I0.571429Nd0.285714', '-1.418', '-0.200530705751']
AgINp 7 ['Ag0.111111I0.666667Np0.222222', '-1.107', '-0.211126431991']
AgIP 3 ['P0.125Ag0.375I0.5', '-0.54', '-0.206025800053']
AgIPa 28 ['Ag0.125I0.5Pa0.375', '-0.413', '-0.216978966598']
AgIPm 42 ['Ag0.5I0.375Pm0.125', '-0.622', '-0.214320133152']
AgIPr 8 ['Ag0.444444I0.444444Pr0.111111', '-0.925', '-0.206006804338']
AgIPu 39 ['Ag0.428571I0.428571Pu0.142857', '-0.895', '-0.204752447531']
AgIRh 2 ['Rh0.222222Ag0.111111I0.666667', '-0.282', '-0.200211558897']
AgISc 8 ['Sc0.2Ag0.2I0.6', '-1.086', '-0.20005781004']
AgISe 2 ['Se0.222222Ag0.444444I0.333333', '-0.459', '-0.200877176691']
AgISi 7 ['Si0.1Ag0.3I0.6', '-0.621', '-0.201217837793']
AgISm 19 ['Ag0.444444I0.444444Sm0.111111', '-0.785', '-0.210094746697']
AgITa 4 ['Ag0.111111I0.666667Ta0.222222', '-0.706', '-0.214755107964']
AgITb 34 ['Ag0.4I0.4Tb0.2', '-0.541', '-0.211027257762']
AgITm 20 ['Ag0.222222I0.555556Tm0.222222', '-1.129', '-0.21253769337']
AgIW 2 ['Ag0.222222I0.666667W0.111111', '-0.378', '-0.214423117794']
AgIXe 6 ['Ag0.125I0.625Xe0.25', '-0.299', '-0.206988003759']
AgIYb 1 ['Ag0.222222I0.555556Yb0.222222', '-1.487', '-0.219650397817']
AgInP 1 ['P0.555556Ag0.222222In0.222222', '-0.454', '-0.202433841108']
AgInTe 1 ['Ag0.25In0.125Te0.625', '-0.392', '-0.230940381146']
AgIrO 6 ['O0.625Ag0.125Ir0.25', '-1.109', '-0.24460890476']
AgKrO 1 ['O0.6Kr0.1Ag0.3', '-0.386', '-0.217175584398']
AgLaN 3 ['N0.4Ag0.1La0.5', '-1.4', '-0.205865504114']
AgLaO 5 ['O0.666667Ag0.166667La0.166667', '-1.889', '-0.248317767049']
AgLaS 1 ['S0.333333Ag0.111111La0.555556', '-1.801', '-0.202205685604']
AgLaSb 3 ['Ag0.1Sb0.5La0.4', '-1.28', '-0.21297092625']
AgLaSi 2 ['Si0.4Ag0.2La0.4', '-0.886', '-0.284621124']
AgLuN 3 ['N0.4Ag0.1Lu0.5', '-1.921', '-0.263151452614']

AgLuO 2 ['O0.625Ag0.25Lu0.125', '-1.652', '-0.273302247458']
AgMgO 1 ['O0.625Mg0.125Ag0.25', '-1.171', '-0.28286703626']
AgMnO 1 ['O0.6Mn0.2Ag0.2', '-1.468', '-0.304560521033']
AgMoO 4 ['O0.666667Mo0.166667Ag0.166667', '-1.769', '-0.237386084582']
AgMoS 1 ['S0.571429Mo0.142857Ag0.285714', '-0.778', '-0.257921005424']
AgNNp 3 ['N0.5Ag0.166667Np0.333333', '-1.047', '-0.238695191967']
AgNPa 19 ['N0.4Ag0.1Pa0.5', '-1.234', '-0.24605580905']
AgNPu 6 ['N0.5Ag0.125Pu0.375', '-1.27', '-0.243158486747']
AgNTi 3 ['N0.444444Ti0.444444Ag0.111111', '-1.384', '-0.231078380437']
AgNW 3 ['N0.555556Ag0.111111W0.333333', '-0.341', '-0.219089418736']
AgNaO 3 ['O0.625Na0.125Ag0.25', '-0.777', '-0.203623171844']
AgNaP 3 ['Na0.5P0.333333Ag0.166667', '-0.665', '-0.217640182525']
AgNaS 1 ['Na0.375S0.375Ag0.25', '-1.043', '-0.215472859061']
AgNaSe 1 ['Na0.444444Se0.333333Ag0.222222', '-1.058', '-0.267816508513']
AgNbO 4 ['O0.666667Nb0.166667Ag0.166667', '-1.95', '-0.202163327631']
AgNbTe 1 ['Nb0.111111Ag0.222222Te0.666667', '-0.413', '-0.205975612037']
AgNdO 4 ['O0.625Ag0.125Nd0.25', '-2.618', '-0.209969707265']
AgNpO 1 ['O0.625Ag0.25Np0.125', '-1.788', '-0.255909739498']
AgNpSe 1 ['Se0.428571Ag0.428571Np0.142857', '-0.645', '-0.20341786256']
AgOOs 3 ['O0.625Ag0.25Os0.125', '-1.137', '-0.258409992578']
AgOP 3 ['O0.6P0.1Ag0.3', '-1.264', '-0.216743095504']
AgOPa 9 ['O0.571429Ag0.285714Pa0.142857', '-1.953', '-0.215696416639']
AgOPb 1 ['O0.625Ag0.25Pb0.125', '-0.772', '-0.213351396155']
AgOPd 1 ['O0.6Pd0.2Ag0.2', '-0.665', '-0.226653935198']
AgOPr 1 ['O0.625Ag0.25Pr0.125', '-1.673', '-0.414791465192']
AgOPt 2 ['O0.6Ag0.3Pt0.1', '-0.594', '-0.202915238222']
AgOPu 12 ['O0.4Ag0.1Pu0.5', '-2.677', '-0.210793634176']
AgORb 5 ['O0.4Rb0.3Ag0.3', '-1.086', '-0.208837677647']
AgORe 3 ['O0.6Ag0.2Re0.2', '-1.857', '-0.336384289472']
AgORh 1 ['O0.6Rh0.2Ag0.2', '-1.0', '-0.271479143974']
AgORu 5 ['O0.625Ru0.25Ag0.125', '-1.244', '-0.228073532263']
AgOSb 5 ['O0.666667Ag0.111111Sb0.222222', '-1.527', '-0.220932276404']
AgOSc 2 ['O0.6Sc0.1Ag0.3', '-1.391', '-0.270328153766']
AgOSi 3 ['O0.571429Si0.142857Ag0.285714', '-1.682', '-0.213561403071']
AgOSn 3 ['O0.6Ag0.2Sn0.2', '-1.538', '-0.2600686826']
AgOSr 1 ['O0.625Sr0.125Ag0.25', '-1.171', '-0.220556759277']
AgOTa 6 ['O0.666667Ag0.166667Ta0.166667', '-2.124', '-0.204238515132']
AgOTc 4 ['O0.6Tc0.1Ag0.3', '-1.161', '-0.25233006372']
AgOTe 2 ['O0.6Ag0.2Te0.2', '-1.222', '-0.246749926806']
AgOTh 2 ['O0.625Ag0.25Th0.125', '-2.0', '-0.270470395998']
AgOTi 4 ['O0.571429Ti0.142857Ag0.285714', '-1.818', '-0.277096426813']
AgOU 7 ['O0.7Ag0.1U0.2', '-2.796', '-0.238238193378']
AgOV 2 ['O0.6V0.2Ag0.2', '-1.923', '-0.21587562242']
AgOW 2 ['O0.625Ag0.25W0.125', '-1.659', '-0.303814080701']

AgOXe 1 ['O0.6Ag0.3Xe0.1', '-0.378', '-0.209175584398']
AgOZr 4 ['O0.625Zr0.25Ag0.125', '-2.999', '-0.20811303101']
AgOsTm 2 ['Ag0.142857Tm0.571429Os0.285714', '-0.38', '-0.202519084643']
AgPPm 16 ['P0.3Ag0.1Pm0.6', '-0.972', '-0.200211952562']
AgPPr 1 ['P0.4Ag0.1Pr0.5', '-1.633', '-0.265406591916']
AgPPu 33 ['P0.166667Ag0.333333Pu0.5', '-0.641', '-0.210968383507']
AgPSr 6 ['P0.5Sr0.3Ag0.2', '-1.042', '-0.20198205477']
AgPTa 9 ['P0.5Ag0.166667Ta0.333333', '-0.709', '-0.202080101467']
AgPYb 1 ['P0.5Ag0.25Yb0.25', '-0.829', '-0.223033435833']
AgPaS 9 ['S0.5Ag0.333333Pa0.166667', '-0.962', '-0.201536651244']
AgPaSe 16 ['Se0.5Ag0.4Pa0.1', '-0.488', '-0.201267929']
AgPaTe 3 ['Ag0.25Te0.625Pa0.125', '-0.389', '-0.206821117917']
AgPbPm 1 ['Ag0.4Pm0.4Pb0.2', '-0.491', '-0.2026743375']
AgPdPm 9 ['Pd0.571429Ag0.142857Pm0.285714', '-0.665', '-0.203336666786']
AgPmS 11 ['S0.3Ag0.3Pm0.4', '-1.419', '-0.205134589295']
AgPmSe 32 ['Se0.5Ag0.4Pm0.1', '-0.574', '-0.20387695975']
AgPmSi 1 ['Si0.333333Ag0.111111Pm0.555556', '-0.559', '-0.203543476389']
AgPmSn 6 ['Ag0.4Sn0.3Pm0.3', '-0.592', '-0.207959143194']
AgPmTe 3 ['Ag0.111111Te0.555556Pm0.333333', '-1.084', '-0.203082987313']
AgPrSe 1 ['Se0.428571Ag0.285714Pr0.285714', '-1.5', '-0.207499022478']
AgPrSi 2 ['Si0.4Ag0.2Pr0.4', '-0.842', '-0.2412433995']
AgPtTh 14 ['Ag0.111111Pt0.555556Th0.333333', '-1.025', '-0.202012759722']
AgPuS 28 ['S0.5Ag0.3Pu0.2', '-1.217', '-0.209806992492']
AgPuSe 3 ['Se0.4Ag0.1Pu0.5', '-1.447', '-0.21022586']
AgSSe 1 ['S0.25Se0.375Ag0.375', '-0.303', '-0.21391902956']
AgSSm 1 ['S0.5Ag0.125Sm0.375', '-2.181', '-0.235132903616']
AgSXe 1 ['S0.5Ag0.1Xe0.4', '-0.253', '-0.229245074549']
AgSZr 4 ['S0.428571Zr0.428571Ag0.142857', '-1.511', '-0.234366369706']
AgSbTe 1 ['Ag0.25Sb0.125Te0.625', '-0.272', '-0.207382530833']
AgScSe 3 ['Sc0.4Se0.5Ag0.1', '-1.601', '-0.204793375248']
AgSeTc 11 ['Se0.5Tc0.25Ag0.25', '-0.235', '-0.206295625']
AgSeY 3 ['Se0.5Y0.4Ag0.1', '-1.749', '-0.20014781']
AgSeZr 2 ['Se0.5Zr0.4Ag0.1', '-1.301', '-0.219845431917']
AgSiTe 5 ['Si0.1Ag0.3Te0.6', '-0.246', '-0.216627202']
AgSmSn 1 ['Ag0.333333Sn0.333333Sm0.333333', '-0.705', '-0.216810164846']
AgTaTe 5 ['Ag0.25Te0.625Ta0.125', '-0.391', '-0.202548178958']
AgTeU 5 ['Ag0.2Te0.7U0.1', '-0.433', '-0.204055471667']
AgTeZr 1 ['Zr0.1Ag0.2Te0.7', '-0.541', '-0.2190653005']
AlAsBr 2 ['Al0.111111As0.111111Br0.777778', '-1.16', '-0.257718917555']
AlAsI 7 ['Al0.222222As0.111111I0.666667', '-0.913', '-0.205100193937']
AlAsK 1 ['Al0.111111K0.555556As0.333333', '-0.71', '-0.237004682778']
AlAuCl 3 ['Al0.111111Cl0.666667Au0.222222', '-1.234', '-0.202939639312']
AlAuHo 2 ['Al0.5Ho0.125Au0.375', '-0.786', '-0.213317696083']
AlAuI 6 ['Al0.142857I0.571429Au0.285714', '-0.684', '-0.22587666647']

AlAuP 6 ['Al0.2P0.5Au0.3', '-0.497', '-0.202289844208']
AlAuPm 1 ['Al0.5Pm0.1Au0.4', '-0.704', '-0.2123752325']
AlAuTh 1 ['Al0.5Au0.4Th0.1', '-0.777', '-0.21631666675']
AlAuY 3 ['Al0.5Y0.125Au0.375', '-0.747', '-0.20032111375']
AlAuZr 2 ['Al0.5Zr0.1Au0.4', '-0.673', '-0.20205843']
AlBCl 1 ['B0.2Al0.1Cl0.7', '-1.582', '-0.208539479571']
AlBF 5 ['B0.2F0.7Al0.1', '-3.366', '-0.239055571441']
AlBI 10 ['B0.111111Al0.222222I0.666667', '-0.963', '-0.255100193937']
AlBN 7 ['B0.3N0.4Al0.3', '-1.386', '-0.205592919364']
AlBPa 8 ['B0.571429Al0.142857Pa0.285714', '-0.27', '-0.211010843036']
AlBSe 1 ['B0.1Al0.3Se0.6', '-0.836', '-0.20104182875']
AlBaBr 2 ['Al0.111111Br0.666667Ba0.222222', '-2.228', '-0.206767508389']
AlBaCl 2 ['Al0.111111Cl0.666667Ba0.222222', '-2.633', '-0.214522926436']
AlBaF 7 ['F0.5Al0.125Ba0.375', '-3.373', '-0.221816705938']
AlBaH 1 ['H0.5Al0.2Ba0.3', '-0.64', '-0.212671608783']
AlBaI 4 ['Al0.1I0.7Ba0.2', '-1.673', '-0.208169404798']
AlBaO 2 ['O0.6Al0.2Ba0.2', '-3.22', '-0.280963044969']
AlBaP 1 ['Al0.3P0.4Ba0.3', '-1.058', '-0.206577285821']
AlBaS 6 ['Al0.375S0.375Ba0.25', '-1.592', '-0.20419017766']
AlBaSe 10 ['Al0.2Se0.5Ba0.3', '-1.753', '-0.216987280168']
AlBeF 3 ['Be0.125F0.75Al0.125', '-3.457', '-0.239616471792']
AlBeN 1 ['Be0.285714N0.428571Al0.285714', '-1.596', '-0.319886652442']
AlBeOs 2 ['Be0.555556Al0.111111Os0.333333', '-0.331', '-0.21604252']
AlBePt 1 ['Be0.5Al0.125Pt0.375', '-0.926', '-0.211033115313']
AlBeRh 1 ['Be0.1Al0.5Rh0.4', '-1.109', '-0.20347226075']
AlBiF 2 ['F0.777778Al0.111111Bi0.111111', '-3.21', '-0.21873610262']
AlBiI 2 ['Al0.125I0.625Bi0.25', '-0.801', '-0.200096573899']
AlBrCa 1 ['Al0.166667Ca0.166667Br0.666667', '-1.94', '-0.207720281723']
AlBrCe 3 ['Al0.2Br0.7Ce0.1', '-1.815', '-0.231164404056']
AlBrCl 3 ['Al0.2Cl0.5Br0.3', '-1.584', '-0.222433486035']
AlBrCo 2 ['Al0.125Co0.125Br0.75', '-1.033', '-0.219737259426']
AlBrCr 4 ['Al0.142857Cr0.142857Br0.714286', '-1.357', '-0.222267759344']
AlBrDy 7 ['Al0.111111Br0.666667Dy0.222222', '-1.756', '-0.241433817414']
AlBrEr 26 ['Al0.222222Br0.666667Er0.111111', '-1.459', '-0.205874573069']
AlBrEu 7 ['Al0.222222Br0.666667Eu0.111111', '-1.81', '-0.202844698278']
AlBrGd 1 ['Al0.111111Br0.777778Gd0.111111', '-1.819', '-0.267059872276']
AlBrH 8 ['H0.5Al0.166667Br0.333333', '-0.806', '-0.204334681607']
AlBrHf 6 ['Al0.142857Br0.714286Hf0.142857', '-1.239', '-0.377204028261']
AlBrHo 17 ['Al0.222222Br0.555556Ho0.222222', '-1.346', '-0.207935873711']
AlBrIr 4 ['Al0.142857Br0.714286Ir0.142857', '-1.17', '-0.201634795058']
AlBrLa 4 ['Al0.2Br0.7La0.1', '-1.846', '-0.223376281308']
AlBrLu 31 ['Al0.111111Br0.444444Lu0.444444', '-1.143', '-0.216819465791']
AlBrMo 1 ['Al0.1Br0.8Mo0.1', '-1.157', '-0.298719065299']
AlBrNa 8 ['Na0.333333Al0.111111Br0.555556', '-1.816', '-0.219035005042']

AlBrNi 3 ['Al0.125Ni0.125Br0.75', '-1.106', '-0.213054995676']
AlBrNp 4 ['Al0.125Br0.75Np0.125', '-1.691', '-0.22296026678']
AlBrOs 3 ['Al0.111111Br0.777778Os0.111111', '-0.839', '-0.239549976693']
AlBrPa 2 ['Al0.111111Br0.777778Pa0.111111', '-1.741', '-0.202347069509']
AlBrPm 21 ['Al0.222222Br0.666667Pm0.111111', '-1.495', '-0.200155987081']
AlBrPr 4 ['Al0.1Br0.8Pr0.1', '-1.626', '-0.228045070048']
AlBrPt 10 ['Al0.2Br0.7Pt0.1', '-1.306', '-0.226989958048']
AlBrPu 13 ['Al0.2Br0.6Pu0.2', '-1.683', '-0.263326295552']
AlBrRb 2 ['Al0.111111Br0.666667Rb0.222222', '-1.686', '-0.229846023933']
AlBrRe 2 ['Al0.111111Br0.777778Re0.111111', '-0.836', '-0.236549976693']
AlBrRh 9 ['Al0.2Br0.7Rh0.1', '-1.371', '-0.202792855306']
AlBrRu 4 ['Al0.125Br0.75Ru0.125', '-1.163', '-0.219212025999']
AlBrSb 2 ['Al0.111111Br0.777778Sb0.111111', '-1.186', '-0.216337017277']
AlBrSc 20 ['Al0.222222Sc0.222222Br0.555556', '-1.292', '-0.214850630392']
AlBrSm 4 ['Al0.1Br0.8Sm0.1', '-1.59', '-0.201763249048']
AlBrTa 2 ['Al0.111111Br0.777778Ta0.111111', '-1.261', '-0.253060817086']
AlBrTb 12 ['Al0.2Br0.7Tb0.1', '-1.549', '-0.202593176449']
AlBrTc 2 ['Al0.111111Br0.777778Tc0.111111', '-1.075', '-0.208169971444']
AlBrTe 10 ['Al0.25Br0.125Te0.625', '-0.663', '-0.200159627024']
AlBrTh 2 ['Al0.125Br0.75Th0.125', '-1.91', '-0.216735151939']
AlBrTl 1 ['Al0.125Br0.625Tl0.25', '-1.449', '-0.201764911303']
AlBrTm 25 ['Al0.142857Br0.428571Tm0.428571', '-1.179', '-0.216238874321']
AlBrU 2 ['Al0.111111Br0.777778U0.111111', '-1.714', '-0.241145063954']
AlBrV 2 ['Al0.1V0.1Br0.8', '-1.11', '-0.26384252454']
AlBrY 36 ['Al0.333333Br0.444444Y0.222222', '-1.239', '-0.210452317536']
AlBrYb 9 ['Al0.1Br0.7Yb0.2', '-2.15', '-0.218104318058']
AlBrZr 2 ['Al0.1Br0.8Zr0.1', '-1.424', '-0.2342429213']
AlCCa 6 ['C0.428571Al0.285714Ca0.285714', '-0.351', '-0.20014900011']
AlCDy 9 ['C0.428571Al0.285714Dy0.285714', '-0.526', '-0.203325094381']
AlCEu 2 ['C0.4Al0.5Eu0.1', '-0.392', '-0.216347282667']
AlCGd 1 ['C0.333333Al0.222222Gd0.444444', '-0.572', '-0.229236736919']
AlCH 5 ['H0.555556C0.333333Al0.111111', '-0.234', '-0.214963513819']
AlCHf 2 ['C0.4Al0.2Hf0.4', '-0.982', '-0.220495832445']
AlCLa 5 ['C0.4Al0.3La0.3', '-0.499', '-0.2015699545']
AlCLu 12 ['C0.333333Al0.111111Lu0.555556', '-0.464', '-0.201611091151']
AlCMo 8 ['C0.375Al0.125Mo0.5', '-0.357', '-0.217332691563']
AlCNd 25 ['C0.333333Al0.5Nd0.166667', '-0.5', '-0.201029205833']
AlCNp 21 ['C0.285714Al0.571429Np0.142857', '-0.317', '-0.207381018571']
AlCPa 12 ['C0.444444Al0.111111Pa0.444444', '-0.617', '-0.20283669453']
AlCPm 13 ['C0.25Al0.25Pm0.5', '-0.399', '-0.21381204375']
AlCPr 12 ['C0.5Al0.125Pr0.375', '-0.409', '-0.201841336484']
AlCPu 1 ['C0.375Al0.5Pu0.125', '-0.376', '-0.200843822344']
AlCSr 1 ['C0.375Al0.5Sr0.125', '-0.351', '-0.2096555775']
AlCTc 2 ['C0.25Al0.5Tc0.25', '-0.455', '-0.20225595325']

AlCW 2 ['C0.333333Al0.444444W0.222222', '-0.33', '-0.203969654459']
AlCaF 3 ['F0.777778Al0.111111Ca0.111111', '-3.429', '-0.306503557706']
AlCaH 8 ['H0.428571Al0.142857Ca0.428571', '-0.668', '-0.221854066138']
AlCaIr 9 ['Al0.428571Ca0.142857Ir0.428571', '-1.043', '-0.228847197112']
AlCaN 2 ['N0.444444Al0.444444Ca0.111111', '-1.555', '-0.209256332059']
AlCaO 1 ['O0.6Al0.2Ca0.2', '-3.345', '-0.382656439218']
AlCaOs 1 ['Al0.625Ca0.125Os0.25', '-0.692', '-0.204587365048']
AlCaPd 3 ['Al0.5Ca0.125Pd0.375', '-1.015', '-0.242351834531']
AlCaRh 4 ['Al0.5Ca0.1Rh0.4', '-1.195', '-0.264439170038']
AlCaS 2 ['Al0.285714S0.428571Ca0.285714', '-1.837', '-0.208067452625']
AlCaSe 2 ['Al0.2Ca0.3Se0.5', '-1.723', '-0.214619190333']
AlCdF 3 ['F0.75Al0.125Cd0.125', '-3.038', '-0.204030684292']
AlCdO 1 ['O0.6Al0.2Cd0.2', '-2.45', '-0.322996373059']
AlCeCl 15 ['Al0.285714Cl0.428571Ce0.285714', '-1.893', '-0.211936703306']
AlCeF 6 ['F0.75Al0.125Ce0.125', '-4.307', '-0.20796833513']
AlCeH 3 ['H0.6Al0.2Ce0.2', '-0.608', '-0.208604562574']
AlCeI 26 ['Al0.125I0.5Ce0.375', '-1.241', '-0.203712134929']
AlCeIr 4 ['Al0.5Ce0.125Ir0.375', '-1.045', '-0.221026987813']
AlCeN 19 ['N0.222222Al0.333333Ce0.444444', '-1.039', '-0.210713664369']
AlCeO 8 ['O0.25Al0.375Ce0.375', '-1.94', '-0.204728963946']
AlCeOs 2 ['Al0.6Ce0.1Os0.3', '-0.71', '-0.204301613']
AlCePd 2 ['Al0.5Pd0.4Ce0.1', '-1.072', '-0.20662457725']
AlCePt 3 ['Al0.444444Ce0.111111Pt0.444444', '-1.263', '-0.204305115184']
AlCeRh 2 ['Al0.555556Rh0.333333Ce0.111111', '-1.131', '-0.241576225833']
AlCeS 18 ['Al0.285714S0.285714Ce0.428571', '-1.656', '-0.220598977066']
AlCeSe 24 ['Al0.3Se0.2Ce0.5', '-1.175', '-0.20825875125']
AlCeSi 1 ['Al0.3Si0.4Ce0.3', '-0.783', '-0.25019905125']
AlClCs 13 ['Al0.3Cl0.5Cs0.2', '-1.775', '-0.204413528692']
AlClEr 44 ['Al0.375Cl0.375Er0.25', '-1.392', '-0.256196652091']
AlClEu 6 ['Al0.1Cl0.5Eu0.4', '-2.38', '-0.201255273665']
AlClH 3 ['H0.5Al0.25Cl0.25', '-0.804', '-0.206488361255']
AlClHo 32 ['Al0.166667Cl0.333333Ho0.5', '-1.258', '-0.216838449166']
AlClI 4 ['Al0.25Cl0.5I0.25', '-1.657', '-0.209806065753']
AlClIn 1 ['Al0.125Cl0.75In0.125', '-1.568', '-0.259022896382']
AlClIr 4 ['Al0.1Cl0.7Ir0.2', '-0.914', '-0.204961095816']
AlClK 1 ['Al0.142857Cl0.714286K0.142857', '-1.849', '-0.224344992506']
AlClLa 1 ['Al0.2Cl0.4La0.4', '-1.799', '-0.203337312255']
AlClLi 1 ['Li0.1Al0.2Cl0.7', '-2.047', '-0.204740334818']
AlClLu 38 ['Al0.333333Cl0.222222Lu0.444444', '-1.004', '-0.202441963195']
AlClNa 3 ['Na0.375Al0.125Cl0.5', '-2.06', '-0.212829095098']
AlClNp 5 ['Al0.125Cl0.625Np0.25', '-1.899', '-0.216377635344']
AlClPb 1 ['Al0.166667Cl0.666667Pb0.166667', '-1.738', '-0.207905187924']
AlClPm 31 ['Al0.25Cl0.625Pm0.125', '-1.889', '-0.222648184928']
AlClPr 10 ['Al0.2Cl0.3Pr0.5', '-1.39', '-0.203438517564']

AlClPt 1 ['Al0.111111Cl0.777778Pt0.111111', '-1.19', '-0.20065882225']
AlClPu 15 ['Al0.222222Cl0.555556Pu0.222222', '-1.908', '-0.250115321881']
AlClRb 5 ['Al0.222222Cl0.666667Rb0.111111', '-1.999', '-0.212207257183']
AlClRh 9 ['Al0.1Cl0.8Rh0.1', '-0.985', '-0.275961095816']
AlClRu 1 ['Al0.125Cl0.75Ru0.125', '-1.488', '-0.201040493913']
AlClSm 9 ['Al0.142857Cl0.714286Sm0.142857', '-2.117', '-0.20350842402']
AlClTa 2 ['Al0.125Cl0.75Ta0.125', '-1.848', '-0.219768460473']
AlClTb 7 ['Al0.1Cl0.7Tb0.2', '-2.347', '-0.210115265301']
AlClTe 1 ['Al0.3Cl0.2Te0.5', '-0.984', '-0.246042817825']
AlClY 11 ['Al0.1Cl0.5Y0.4', '-2.009', '-0.202714713719']
AlClZr 1 ['Al0.2Cl0.7Zr0.1', '-1.966', '-0.200782086682']
AlCoF 5 ['F0.7Al0.1Co0.2', '-2.836', '-0.267100180497']
AlCoI 9 ['Al0.142857Co0.142857I0.714286', '-0.671', '-0.202315812551']
AlCoIr 5 ['Al0.555556Co0.111111Ir0.333333', '-1.014', '-0.200917023889']
AlCoN 40 ['N0.5Al0.125Co0.375', '-0.594', '-0.209309718643']
AlCoOs 1 ['Al0.571429Co0.142857Os0.285714', '-0.741', '-0.204330700714']
AlCoP 19 ['Al0.166667P0.5Co0.333333', '-0.927', '-0.20460178302']
AlCoRh 4 ['Al0.571429Co0.142857Rh0.285714', '-1.054', '-0.211906335357']
AlCoS 1 ['Al0.111111S0.555556Co0.333333', '-1.012', '-0.230037583672']
AlCrF 1 ['F0.8Al0.1Cr0.1', '-3.222', '-0.235652109446']
AlCrIr 6 ['Al0.5Cr0.125Ir0.375', '-0.982', '-0.223057241562']
AlCrRh 1 ['Al0.6Cr0.1Rh0.3', '-0.975', '-0.226730169375']
AlCsF 10 ['F0.555556Al0.111111Cs0.333333', '-3.275', '-0.208534724924']
AlCsH 4 ['H0.6Al0.2Cs0.2', '-0.295', '-0.227060549356']
AlCsI 3 ['Al0.142857I0.571429Cs0.285714', '-1.486', '-0.238537925282']
AlCsS 22 ['Al0.333333S0.555556Cs0.111111', '-1.41', '-0.20437340888']
AlCsSe 27 ['Al0.125Se0.5Cs0.375', '-1.111', '-0.207304663438']
AlCuF 3 ['F0.777778Al0.111111Cu0.111111', '-2.561', '-0.283724827706']
AlCuIr 1 ['Al0.6Cu0.1Ir0.3', '-0.936', '-0.21469037']
AlCuLu 1 ['Al0.1Cu0.3Lu0.6', '-0.473', '-0.207992425']
AlCuTe 5 ['Al0.25Cu0.125Te0.625', '-0.521', '-0.206178645']
AlDyF 4 ['F0.7Al0.1Dy0.2', '-4.082', '-0.260714089362']
AlDyI 10 ['Al0.2I0.7Dy0.1', '-1.03', '-0.202525723267']
AlDyIr 6 ['Al0.142857Dy0.428571Ir0.428571', '-1.032', '-0.205579749643']
AlDyN 1 ['N0.444444Al0.444444Dy0.111111', '-1.669', '-0.2248638467']
AlDyO 4 ['O0.625Al0.125Dy0.25', '-3.648', '-0.202584446669']
AlDyOs 5 ['Al0.555556Dy0.111111Os0.333333', '-0.74', '-0.233275286481']
AlDyPd 5 ['Al0.555556Pd0.333333Dy0.111111', '-1.037', '-0.255497699259']
AlDyPt 1 ['Al0.5Dy0.1Pt0.4', '-1.263', '-0.234192047334']
AlDyRh 3 ['Al0.6Rh0.3Dy0.1', '-1.05', '-0.207897877583']
AlDyS 15 ['Al0.142857S0.428571Dy0.428571', '-2.05', '-0.206707272565']
AlDySe 4 ['Al0.285714Se0.428571Dy0.285714', '-1.547', '-0.21150514095']
AlErF 3 ['F0.3Al0.3Er0.4', '-2.244', '-0.218917705637']
AlErH 1 ['H0.571429Al0.142857Er0.285714', '-0.842', '-0.233078724847']

AlErI 15 ['Al0.111111I0.555556Er0.333333', '-1.132', '-0.200209040551']
AlErIr 10 ['Al0.571429Er0.142857Ir0.285714', '-1.027', '-0.267310246428']
AlErN 35 ['N0.5Al0.2Er0.3', '-1.936', '-0.20023595933']
AlErO 4 ['O0.625Al0.125Er0.25', '-3.672', '-0.202754074191']
AlErOs 6 ['Al0.6Er0.2Os0.2', '-0.723', '-0.206827309125']
AlErPd 3 ['Al0.5Pd0.375Er0.125', '-1.048', '-0.215488769583']
AlErPt 2 ['Al0.5Er0.125Pt0.375', '-1.244', '-0.210207163646']
AlErRh 7 ['Al0.5Rh0.333333Er0.166667', '-1.109', '-0.225165202222']
AlErS 22 ['Al0.1S0.4Er0.5', '-1.961', '-0.200091259767']
AlErSe 10 ['Al0.142857Se0.428571Er0.428571', '-1.715', '-0.206751439643']
AlEuF 3 ['F0.777778Al0.111111Eu0.111111', '-3.524', '-0.301379944926']
AlEuI 6 ['Al0.2I0.6Eu0.2', '-1.546', '-0.212624643207']
AlEuIr 7 ['Al0.571429Eu0.142857Ir0.285714', '-0.945', '-0.234466168571']
AlEuN 20 ['N0.333333Al0.222222Eu0.444444', '-1.035', '-0.235418279331']
AlEuO 6 ['O0.625Al0.125Eu0.25', '-3.034', '-0.244488782059']
AlEuP 4 ['Al0.222222P0.333333Eu0.444444', '-1.086', '-0.203471825516']
AlEuPt 2 ['Al0.555556Eu0.111111Pt0.333333', '-1.134', '-0.225272341445']
AlEuRh 1 ['Al0.5Rh0.4Eu0.1', '-1.177', '-0.2366485346']
AlEuS 5 ['Al0.2S0.4Eu0.4', '-2.182', '-0.205732242392']
AlEuSe 17 ['Al0.166667Se0.5Eu0.333333', '-1.938', '-0.206694797778']
AlFFe 2 ['F0.777778Al0.111111Fe0.111111', '-3.118', '-0.354099553911']
AlFGa 1 ['F0.75Al0.125Ga0.125', '-3.61', '-0.260831077525']
AlFGd 3 ['F0.75Al0.125Gd0.125', '-4.246', '-0.255281778465']
AlFGe 1 ['F0.8Al0.1Ge0.1', '-3.085', '-0.26523174611']
AlFH 2 ['H0.111111F0.777778Al0.111111', '-2.364', '-0.302540521809']
AlFHf 1 ['F0.8Al0.1Hf0.1', '-3.811', '-0.23263211611']
AlFHg 2 ['F0.777778Al0.111111Hg0.111111', '-2.469', '-0.216740408819']
AlFHo 2 ['F0.777778Al0.111111Ho0.111111', '-4.074', '-0.399141134653']
AlFIn 2 ['F0.777778Al0.111111In0.111111', '-3.294', '-0.359041385578']
AlFK 6 ['F0.714286Al0.142857K0.142857', '-3.428', '-0.33669159014']
AlFKr 2 ['F0.777778Al0.111111Kr0.111111', '-2.03', '-0.225550726593']
AlFLa 7 ['F0.333333Al0.166667La0.5', '-2.333', '-0.205552513114']
AlFLi 3 ['Li0.142857F0.714286Al0.142857', '-3.351', '-0.254380630338']
AlFLu 17 ['F0.444444Al0.111111Lu0.444444', '-2.696', '-0.207918222793']
AlFMg 3 ['F0.75Mg0.125Al0.125', '-3.66', '-0.313253995645']
AlFN 10 ['N0.4F0.1Al0.5', '-1.927', '-0.208960934951']
AlFNa 9 ['F0.5Na0.375Al0.125', '-3.114', '-0.211999274076']
AlFNb 1 ['F0.777778Al0.111111Nb0.111111', '-3.669', '-0.205165682155']
AlFNd 2 ['F0.777778Al0.111111Nd0.111111', '-3.948', '-0.295513928724']
AlFNi 3 ['F0.75Al0.125Ni0.125', '-3.028', '-0.207254617525']
AlFNp 1 ['F0.8Al0.1Np0.1', '-4.078', '-0.661286455565']
AlFO 5 ['O0.222222F0.666667Al0.111111', '-1.946', '-0.245505896956']
AlFP 3 ['F0.8Al0.1P0.1', '-3.451', '-0.208803737796']
AlFPa 4 ['F0.714286Al0.142857Pa0.142857', '-3.832', '-0.275221704905']

AlFPb 1 ['F0.777778Al0.111111Pb0.111111', '-3.138', '-0.255874894011']
AlFPm 4 ['F0.7Al0.1Pm0.2', '-4.092', '-0.239193534694']
AlFPr 2 ['F0.777778Al0.111111Pr0.111111', '-4.053', '-0.404210666969']
AlFPu 7 ['F0.666667Al0.111111Pu0.222222', '-3.808', '-0.310912782985']
AlFRb 10 ['F0.555556Al0.222222Rb0.222222', '-3.254', '-0.213011941591']
AlFRe 2 ['F0.777778Al0.111111Re0.111111', '-3.052', '-0.205194141151']
AlFRh 2 ['F0.777778Al0.111111Rh0.111111', '-2.731', '-0.2330046528']
AlFRu 2 ['F0.777778Al0.111111Ru0.111111', '-2.999', '-0.239126698456']
AlFSb 1 ['F0.8Al0.1Sb0.1', '-3.201', '-0.493109420312']
AlFSc 2 ['F0.777778Al0.111111Sc0.111111', '-3.872', '-0.273802070022']
AlFSm 2 ['F0.777778Al0.111111Sm0.111111', '-3.923', '-0.257187459191']
AlFSn 2 ['F0.777778Al0.111111Sn0.111111', '-3.406', '-0.228010375467']
AlFSr 4 ['F0.571429Al0.142857Sr0.285714', '-3.807', '-0.201867723351']
AlFTa 1 ['F0.777778Al0.111111Ta0.111111', '-3.928', '-0.377182684897']
AlFTb 1 ['F0.8Al0.1Tb0.1', '-3.443', '-0.200325090804']
AlFTc 2 ['F0.777778Al0.111111Tc0.111111', '-3.151', '-0.208997864706']
AlFTe 1 ['F0.8Al0.1Te0.1', '-3.115', '-0.202020132862']
AlFTh 2 ['F0.714286Al0.142857Th0.142857', '-4.189', '-0.274235932288']
AlFTi 4 ['F0.714286Al0.142857Ti0.142857', '-3.802', '-0.235757346569']
AlFTl 2 ['F0.8Al0.1Tl0.1', '-2.61', '-0.25789689402']
AlFTm 40 ['F0.4Al0.1Tm0.5', '-2.254', '-0.207054250201']
AlFU 2 ['F0.777778Al0.111111U0.111111', '-4.003', '-0.209576724501']
AlFV 1 ['F0.8Al0.1V0.1', '-3.35', '-0.32080553911']
AlFY 4 ['F0.666667Al0.111111Y0.222222', '-3.973', '-0.233909062751']
AlFYb 28 ['F0.5Al0.3Yb0.2', '-2.901', '-0.211874891234']
AlFZn 2 ['F0.777778Al0.111111Zn0.111111', '-2.812', '-0.255801246039']
AlFeN 16 ['N0.333333Al0.222222Fe0.444444', '-0.931', '-0.203576356553']
AlFeP 8 ['Al0.333333P0.333333Fe0.333333', '-0.801', '-0.201753170347']
AlFeRh 2 ['Al0.6Fe0.1Rh0.3', '-0.984', '-0.2037967215']
AlGaPa 2 ['Al0.111111Ga0.666667Pa0.222222', '-0.358', '-0.223246610556']
AlGdI 14 ['Al0.222222I0.666667Gd0.111111', '-1.032', '-0.204794783658']
AlGdIr 5 ['Al0.6Gd0.1Ir0.3', '-1.105', '-0.2148973445']
AlGdN 2 ['N0.444444Al0.444444Gd0.111111', '-1.656', '-0.225099952164']
AlGdO 1 ['O0.625Al0.125Gd0.25', '-3.636', '-0.219895445441']
AlGdOs 4 ['Al0.666667Gd0.111111Os0.222222', '-0.756', '-0.207169671944']
AlGdPd 4 ['Al0.444444Pd0.444444Gd0.111111', '-1.103', '-0.207350922685']
AlGdPt 1 ['Al0.555556Gd0.111111Pt0.333333', '-1.146', '-0.212744374008']
AlGdRh 3 ['Al0.5Rh0.375Gd0.125', '-1.141', '-0.20907906214']
AlGdS 17 ['Al0.333333S0.555556Gd0.111111', '-1.624', '-0.200003509157']
AlGdSe 3 ['Al0.125Se0.5Gd0.375', '-1.863', '-0.213098486979']
AlGePa 1 ['Al0.1Ge0.6Pa0.3', '-0.418', '-0.21222967']
AlHI 15 ['H0.571429Al0.285714I0.142857', '-0.364', '-0.204711886976']
AlHK 1 ['H0.444444Al0.222222K0.333333', '-0.399', '-0.233475697733']
AlHMn 6 ['H0.4Al0.2Mn0.4', '-0.314', '-0.206940597241']

AlHNp 3 ['H0.5Al0.25Np0.25', '-0.38', '-0.202407862137']
AlHPm 18 ['H0.5Al0.3Pm0.2', '-0.539', '-0.209537303345']
AlHPr 8 ['H0.6Al0.1Pr0.3', '-0.764', '-0.203333269773']
AlHPu 9 ['H0.666667Al0.166667Pu0.166667', '-0.456', '-0.211627225488']
AlHRb 12 ['H0.6Al0.1Rb0.3', '-0.303', '-0.20379079419']
AlHSe 4 ['H0.4Al0.3Se0.3', '-0.635', '-0.207707021345']
AlHSm 5 ['H0.5Al0.25Sm0.25', '-0.731', '-0.200299679255']
AlHTb 2 ['H0.4Al0.3Tb0.3', '-0.601', '-0.210400217226']
AlHTm 3 ['H0.571429Al0.142857Tm0.285714', '-0.82', '-0.21457772653']
AlHYb 5 ['H0.5Al0.25Yb0.25', '-0.669', '-0.207283990158']
AlHfIr 8 ['Al0.571429Hf0.142857Ir0.285714', '-0.937', '-0.205947299643']
AlHfN 11 ['N0.375Al0.375Hf0.25', '-1.564', '-0.203260492997']
AlHfOs 2 ['Al0.571429Hf0.142857Os0.285714', '-0.769', '-0.211383473036']
AlHfPd 4 ['Al0.444444Pd0.444444Hf0.111111', '-1.048', '-0.201994938889']
AlHfPt 3 ['Al0.444444Hf0.111111Pt0.444444', '-1.259', '-0.210322491203']
AlHfRh 6 ['Al0.444444Rh0.444444Hf0.111111', '-1.22', '-0.204534108705']
AlHfS 17 ['Al0.2S0.5Hf0.3', '-1.612', '-0.201338938627']
AlHfSe 14 ['Al0.25Se0.375Hf0.375', '-1.172', '-0.204262428438']
AlHgO 1 ['O0.6Al0.2Hg0.2', '-2.094', '-0.279702241399']
AlHoI 8 ['Al0.125I0.625Ho0.25', '-1.094', '-0.237715052899']
AlHoIr 6 ['Al0.444444Ho0.111111Ir0.444444', '-1.144', '-0.251873948006']
AlHoO 3 ['O0.625Al0.25Ho0.125', '-3.543', '-0.275865956278']
AlHoOs 5 ['Al0.571429Ho0.142857Os0.285714', '-0.696', '-0.208426592381']
AlHoPd 4 ['Al0.571429Pd0.285714Ho0.142857', '-0.953', '-0.210472636667']
AlHoPt 3 ['Al0.375Ho0.25Pt0.375', '-1.248', '-0.207165216487']
AlHoRh 6 ['Al0.625Rh0.25Ho0.125', '-0.984', '-0.207094864271']
AlHoS 23 ['Al0.375S0.25Ho0.375', '-1.48', '-0.208310937499']
AlIK 3 ['Al0.111111K0.333333I0.555556', '-1.502', '-0.210156832172']
AlILa 1 ['Al0.166667I0.5La0.333333', '-1.544', '-0.244851053784']
AlILu 18 ['Al0.222222I0.666667Lu0.111111', '-0.943', '-0.207335744016']
AlIMg 1 ['Mg0.222222Al0.111111I0.666667', '-1.245', '-0.226044799306']
AlINd 11 ['Al0.285714I0.571429Nd0.142857', '-1.119', '-0.206879721706']
AlINi 6 ['Al0.166667Ni0.166667I0.666667', '-0.793', '-0.207399680881']
AlINp 11 ['Al0.1I0.7Np0.2', '-1.115', '-0.202528817883']
AlIO 4 ['O0.222222Al0.333333I0.444444', '-1.889', '-0.207783082058']
AlIPa 12 ['Al0.222222I0.666667Pa0.111111', '-0.95', '-0.242100193937']
AlIPd 9 ['Al0.2Pd0.3I0.5', '-0.901', '-0.227746474036']
AlIPm 22 ['Al0.2I0.7Pm0.1', '-1.039', '-0.22296547505']
AlIPr 3 ['Al0.166667I0.5Pr0.333333', '-1.403', '-0.202641034787']
AlIPt 21 ['Al0.285714I0.428571Pt0.285714', '-1.027', '-0.201984694495']
AlIPu 13 ['Al0.111111I0.555556Pu0.333333', '-1.154', '-0.200423950319']
AlIRb 13 ['Al0.25Rb0.125I0.625', '-1.16', '-0.2289397389']
AlIRe 3 ['Al0.125I0.75Re0.125', '-0.701', '-0.208547292137']
AlIRh 17 ['Al0.125Rh0.375I0.5', '-0.599', '-0.200806359089']

AlIRu 2 ['Al0.166667Ru0.166667I0.666667', '-0.733', '-0.202075145453']
AlIS 5 ['Al0.222222S0.333333I0.444444', '-0.9', '-0.200323701345']
AlISe 3 ['Al0.111111Se0.333333I0.555556', '-0.555', '-0.201050096968']
AlISi 2 ['Al0.142857Si0.142857I0.714286', '-0.846', '-0.201442152075']
AlISm 9 ['Al0.125I0.625Sm0.25', '-1.275', '-0.211930197854']
AlITb 28 ['Al0.25I0.625Tb0.125', '-0.893', '-0.210411651198']
AlITe 19 ['Al0.222222Te0.333333I0.444444', '-0.759', '-0.202427999785']
AlITm 7 ['Al0.1I0.8Tm0.1', '-0.927', '-0.232841847785']
AlIXe 7 ['Al0.142857I0.571429Xe0.285714', '-0.688', '-0.232921553245']
AlIYb 5 ['Al0.166667I0.666667Yb0.166667', '-1.47', '-0.226879226158']
AlIZn 1 ['Al0.111111Zn0.222222I0.666667', '-0.864', '-0.213877051159']
AlIrLa 4 ['Al0.6La0.1Ir0.3', '-0.97', '-0.21542806']
AlIrLi 4 ['Li0.166667Al0.5Ir0.333333', '-1.048', '-0.243905073333']
AlIrLu 11 ['Al0.25Lu0.375Ir0.375', '-1.054', '-0.202024011154']
AlIrMg 2 ['Mg0.111111Al0.555556Ir0.333333', '-0.945', '-0.230455915556']
AlIrMn 1 ['Al0.625Mn0.125Ir0.25', '-0.869', '-0.234806939526']
AlIrMo 1 ['Al0.555556Mo0.111111Ir0.333333', '-0.944', '-0.205687130423']
AlIrNa 2 ['Na0.1Al0.6Ir0.3', '-0.889', '-0.2082628795']
AlIrNb 4 ['Al0.6Nb0.1Ir0.3', '-0.947', '-0.206972108']
AlIrNd 9 ['Al0.5Nd0.166667Ir0.333333', '-0.966', '-0.204247533333']
AlIrNi 1 ['Al0.6Ni0.1Ir0.3', '-1.011', '-0.23200874225']
AlIrOs 9 ['Al0.571429Os0.142857Ir0.285714', '-0.992', '-0.210434304286']
AlIrPa 1 ['Al0.5Ir0.4Pa0.1', '-1.051', '-0.253920699333']
AlIrPd 1 ['Al0.6Pd0.1Ir0.3', '-1.104', '-0.2784646765']
AlIrPm 4 ['Al0.5Pm0.125Ir0.375', '-1.082', '-0.280113209687']
AlIrPr 6 ['Al0.571429Pr0.142857Ir0.285714', '-0.995', '-0.244084324643']
AlIrPu 1 ['Al0.6Ir0.3Pu0.1', '-0.943', '-0.2316985015']
AlIrRe 3 ['Al0.555556Re0.111111Ir0.333333', '-0.941', '-0.204665174722']
AlIrRh 5 ['Al0.571429Rh0.285714Ir0.142857', '-1.156', '-0.203283498929']
AlIrRu 4 ['Al0.555556Ru0.111111Ir0.333333', '-1.098', '-0.229699147778']
AlIrSc 9 ['Al0.625Sc0.125Ir0.25', '-0.992', '-0.201187290937']
AlIrSm 7 ['Al0.444444Sm0.111111Ir0.444444', '-1.059', '-0.205897289537']
AlIrSr 2 ['Al0.6Sr0.1Ir0.3', '-0.954', '-0.2686792606']
AlIrTb 7 ['Al0.571429Tb0.142857Ir0.285714', '-0.889', '-0.21842170719']
AlIrTc 4 ['Al0.555556Tc0.111111Ir0.333333', '-1.028', '-0.265430419722']
AlIrTh 4 ['Al0.6Ir0.3Th0.1', '-1.018', '-0.2365879675']
AlIrTi 7 ['Al0.428571Ti0.142857Ir0.428571', '-1.103', '-0.217499157019']
AlIrTm 7 ['Al0.625Tm0.125Ir0.25', '-0.929', '-0.220609599375']
AlIrV 6 ['Al0.5V0.166667Ir0.333333', '-0.955', '-0.236008049722']
AlIrY 10 ['Al0.5Y0.166667Ir0.333333', '-1.002', '-0.20470589391']
AlIrYb 7 ['Al0.625Yb0.125Ir0.25', '-0.926', '-0.274003128125']
AlIrZr 10 ['Al0.4Zr0.1Ir0.5', '-1.148', '-0.21947406875']
AlKS 9 ['Al0.166667S0.5K0.333333', '-1.367', '-0.202784248341']
AlKSe 2 ['Al0.333333K0.166667Se0.5', '-1.161', '-0.22414382514']

AlKTe 4 ['Al0.333333K0.111111Te0.555556', '-0.806', '-0.200991376759']
AlLaN 7 ['N0.428571Al0.142857La0.428571', '-1.504', '-0.20237450795']
AlLaO 3 ['O0.625Al0.125La0.25', '-3.587', '-0.201105238956']
AlLaPd 1 ['Al0.555556Pd0.333333La0.111111', '-1.05', '-0.2589845475']
AlLaPt 3 ['Al0.5La0.1Pt0.4', '-1.24', '-0.229342752643']
AlLaRh 2 ['Al0.555556Rh0.333333La0.111111', '-1.106', '-0.208831173889']
AlLaS 14 ['Al0.3S0.3La0.4', '-1.77', '-0.204736220294']
AlLaSe 22 ['Al0.111111Se0.333333La0.555556', '-1.632', '-0.207617896944']
AlLaSi 1 ['Al0.3Si0.4La0.3', '-0.799', '-0.2201370115']
AlLiN 18 ['Li0.111111N0.444444Al0.444444', '-1.556', '-0.213899073182']
AlLiO 2 ['Li0.3O0.4Al0.3', '-2.612', '-0.216702407652']
AlLiRh 2 ['Li0.1Al0.5Rh0.4', '-1.154', '-0.210116334']
AlLiSe 2 ['Li0.285714Al0.285714Se0.428571', '-1.226', '-0.207524957143']
AlLuN 15 ['N0.444444Al0.111111Lu0.444444', '-1.992', '-0.210105498738']
AlLuO 3 ['O0.6Al0.3Lu0.1', '-3.651', '-0.20757433272']
AlLuOs 3 ['Al0.625Lu0.125Os0.25', '-0.772', '-0.244792607187']
AlLuPd 2 ['Al0.5Pd0.375Lu0.125', '-1.065', '-0.236660465104']
AlLuRh 9 ['Al0.25Rh0.375Lu0.375', '-1.1', '-0.204670226458']
AlLuS 2 ['Al0.3S0.4Lu0.3', '-1.751', '-0.20124456156']
AlLuTe 1 ['Al0.1Te0.6Lu0.3', '-1.183', '-0.263373893175']
AlMgN 1 ['N0.428571Mg0.285714Al0.285714', '-1.461', '-0.290285866729']
AlMgO 1 ['O0.6Mg0.1Al0.3', '-3.288', '-0.210851051348']
AlMgRh 1 ['Mg0.1Al0.5Rh0.4', '-1.17', '-0.26447226075']
AlMnN 15 ['N0.25Al0.375Mn0.375', '-1.022', '-0.200156101941']
AlMnPt 2 ['Al0.5Mn0.1Pt0.4', '-1.122', '-0.2026254185']
AlMoN 1 ['N0.4Al0.1Mo0.5', '-0.811', '-0.226515952613']
AlMoO 1 ['O0.666667Al0.111111Mo0.222222', '-2.734', '-0.268982311777']
AlNNa 3 ['N0.444444Na0.222222Al0.333333', '-1.253', '-0.203905670558']
AlNNb 6 ['N0.444444Al0.222222Nb0.333333', '-1.479', '-0.228744502071']
AlNNp 28 ['N0.625Al0.125Np0.25', '-1.191', '-0.207305508308']
AlNPa 31 ['N0.333333Al0.333333Pa0.333333', '-1.285', '-0.224029395642']
AlNPm 1 ['N0.444444Al0.444444Pm0.111111', '-1.632', '-0.234410435543']
AlNPr 1 ['N0.428571Al0.428571Pr0.142857', '-1.553', '-0.211029064259']
AlNPu 5 ['N0.5Al0.1Pu0.4', '-1.601', '-0.20372967733']
AlNRe 2 ['N0.5Al0.2Re0.3', '-0.852', '-0.213187415898']
AlNRh 7 ['N0.125Al0.5Rh0.375', '-1.404', '-0.203631320582']
AlNRu 1 ['N0.222222Al0.444444Ru0.333333', '-1.166', '-0.20038826548']
AlNSc 17 ['N0.5Al0.4Sc0.1', '-1.833', '-0.22099029883']
AlNSi 3 ['N0.5Al0.4Si0.1', '-1.635', '-0.21456959858']
AlNSm 10 ['N0.428571Al0.142857Sm0.428571', '-1.629', '-0.20303605164']
AlNTa 1 ['N0.333333Al0.222222Ta0.444444', '-1.242', '-0.201200255275']
AlNTb 6 ['N0.444444Al0.111111Tb0.444444', '-1.542', '-0.22620996388']
AlNTc 20 ['N0.3Al0.5Tc0.2', '-1.264', '-0.207954570598']
AlNTh 22 ['N0.333333Al0.333333Th0.333333', '-1.442', '-0.21194122822']

AlNTi 20 ['N0.5Al0.4Ti0.1', '-1.67', '-0.202701801462']
AlNTm 4 ['N0.444444Al0.111111Tm0.444444', '-1.947', '-0.207358797627']
AlNU 13 ['N0.375Al0.25U0.375', '-1.33', '-0.203245155081']
AlNV 17 ['N0.333333Al0.444444V0.222222', '-1.283', '-0.201010868431']
AlNW 4 ['N0.555556Al0.222222W0.222222', '-0.954', '-0.201675815748']
AlNY 16 ['N0.444444Al0.111111Y0.444444', '-1.834', '-0.200061769664']
AlNYb 15 ['N0.4Al0.1Yb0.5', '-1.307', '-0.216341199986']
AlNZn 2 ['N0.428571Al0.142857Zn0.428571', '-0.674', '-0.24261041638']
AlNZr 11 ['N0.4Al0.3Zr0.3', '-1.653', '-0.208605583864']
AlNaRh 1 ['Na0.1Al0.6Rh0.3', '-0.96', '-0.21838348725']
AlNaS 1 ['Na0.166667Al0.333333S0.5', '-1.469', '-0.253005429864']
AlNbO 1 ['O0.625Al0.125Nb0.25', '-3.068', '-0.246294132155']
AlNbP 1 ['Al0.222222P0.333333Nb0.444444', '-1.123', '-0.231346823063']
AlNdO 5 ['O0.625Al0.125Nd0.25', '-3.57', '-0.203691798825']
AlNdOs 2 ['Al0.625Nd0.125Os0.25', '-0.786', '-0.26643265']
AlNdPd 1 ['Al0.5Pd0.4Nd0.1', '-1.094', '-0.2195600555']
AlNdPt 2 ['Al0.5Nd0.1Pt0.4', '-1.24', '-0.212041044749']
AlNdRh 4 ['Al0.6Rh0.3Nd0.1', '-1.103', '-0.2652952885']
AlNdS 7 ['Al0.166667S0.5Nd0.333333', '-2.085', '-0.218659243408']
AlNdSe 14 ['Al0.1Se0.5Nd0.4', '-1.892', '-0.219538971499']
AlNiP 2 ['Al0.2P0.3Ni0.5', '-0.834', '-0.206163427479']
AlNiRh 2 ['Al0.571429Ni0.142857Rh0.285714', '-1.068', '-0.200158323393']
AlNiS 1 ['Al0.333333S0.222222Ni0.444444', '-0.972', '-0.210292801978']
AlNiSe 15 ['Al0.125Ni0.5Se0.375', '-0.634', '-0.207939503542']
AlNiYb 1 ['Al0.555556Ni0.333333Yb0.111111', '-0.784', '-0.207303603333']
AlNpO 9 ['O0.4Al0.2Np0.4', '-2.577', '-0.213989279578']
AlNpP 2 ['Al0.142857P0.428571Np0.428571', '-1.074', '-0.226865996696']
AlNpS 10 ['Al0.25S0.375Np0.375', '-1.339', '-0.207621672715']
AlNpSe 18 ['Al0.111111Se0.444444Np0.444444', '-1.161', '-0.207360767776']
AlOPa 5 ['O0.666667Al0.222222Pa0.111111', '-3.124', '-0.277141036177']
AlOPm 1 ['O0.6Al0.3Pm0.1', '-3.613', '-0.21520417922']
AlOPu 3 ['O0.6Al0.3Pu0.1', '-3.597', '-0.203556996225']
AlORb 1 ['O0.428571Al0.285714Rb0.285714', '-2.592', '-0.255287260337']
AlOSm 2 ['O0.6Al0.3Sm0.1', '-3.626', '-0.22501783572']
AlOTb 2 ['O0.625Al0.25Tb0.125', '-3.379', '-0.203691743922']
AlOTe 1 ['O0.7Al0.1Te0.2', '-1.894', '-0.212880025967']
AlOTh 2 ['O0.666667Al0.166667Th0.166667', '-3.733', '-0.254099033862']
AlOTi 3 ['O0.285714Al0.142857Ti0.571429', '-1.996', '-0.226646408756']
AlOTm 1 ['O0.6Al0.3Tm0.1', '-3.665', '-0.22464174797']
AlOU 3 ['O0.666667Al0.111111U0.222222', '-3.761', '-0.239352290587']
AlOYb 1 ['O0.571429Al0.285714Yb0.142857', '-3.536', '-0.201435889496']
AlOsP 3 ['Al0.333333P0.333333Os0.333333', '-0.841', '-0.215500677013']
AlOsPa 1 ['Al0.666667Os0.222222Pa0.111111', '-0.674', '-0.219219208472']
AlOsPd 1 ['Al0.6Pd0.2Os0.2', '-0.904', '-0.201976447']

AlOsPm 3 ['Al0.666667Pm0.111111Os0.222222', '-0.769', '-0.253037595']
AlOsPr 3 ['Al0.666667Pr0.111111Os0.222222', '-0.753', '-0.214951808055']
AlOsPt 1 ['Al0.625Os0.25Pt0.125', '-0.921', '-0.237510126562']
AlOsRh 10 ['Al0.6Rh0.1Os0.3', '-0.859', '-0.217032538']
AlOsSc 5 ['Al0.625Sc0.125Os0.25', '-0.776', '-0.208710389844']
AlOsSm 5 ['Al0.555556Sm0.111111Os0.333333', '-0.692', '-0.200153447639']
AlOsTb 2 ['Al0.666667Tb0.111111Os0.222222', '-0.687', '-0.211436438926']
AlOsTm 4 ['Al0.111111Tm0.555556Os0.333333', '-0.381', '-0.200273539583']
AlOsY 3 ['Al0.571429Y0.142857Os0.285714', '-0.729', '-0.212016387024']
AlOsYb 2 ['Al0.625Yb0.125Os0.25', '-0.696', '-0.208697055625']
AlOsZr 3 ['Al0.6Zr0.1Os0.3', '-0.773', '-0.205515293571']
AlPPd 6 ['Al0.428571P0.142857Pd0.428571', '-1.055', '-0.204320012053']
AlPPm 4 ['Al0.1P0.5Pm0.4', '-1.311', '-0.21023031752']
AlPPt 6 ['Al0.5P0.166667Pt0.333333', '-1.151', '-0.20654273434']
AlPRh 2 ['Al0.4P0.2Rh0.4', '-1.196', '-0.200023939833']
AlPRu 16 ['Al0.444444P0.222222Ru0.333333', '-0.937', '-0.205864507453']
AlPSr 1 ['Al0.25P0.5Sr0.25', '-1.212', '-0.219838835431']
AlPTa 4 ['Al0.125P0.375Ta0.5', '-1.011', '-0.209777983515']
AlPTc 5 ['Al0.125P0.5Tc0.375', '-0.865', '-0.205078119387']
AlPY 2 ['Al0.125P0.5Y0.375', '-1.728', '-0.228340302395']
AlPaSe 8 ['Al0.142857Se0.571429Pa0.285714', '-1.216', '-0.2256244775']
AlPdPm 6 ['Al0.111111Pd0.777778Pm0.111111', '-0.688', '-0.208020351111']
AlPdPr 3 ['Al0.5Pd0.375Pr0.125', '-1.043', '-0.233040680208']
AlPdSc 2 ['Al0.555556Sc0.111111Pd0.333333', '-0.995', '-0.214995466975']
AlPdSm 3 ['Al0.6Pd0.3Sm0.1', '-0.938', '-0.20461686375']
AlPdTb 3 ['Al0.5Pd0.4Tb0.1', '-1.07', '-0.286997335908']
AlPdTh 1 ['Al0.5Pd0.4Th0.1', '-1.063', '-0.23555429525']
AlPdTm 2 ['Al0.375Pd0.5Tm0.125', '-1.128', '-0.206636260625']
AlPdY 3 ['Al0.4Y0.2Pd0.4', '-1.097', '-0.206551950667']
AlPdYb 3 ['Al0.555556Pd0.333333Yb0.111111', '-0.932', '-0.204679501833']
AlPdZr 2 ['Al0.5Zr0.1Pd0.4', '-1.067', '-0.223720694933']
AlPmPt 4 ['Al0.6Pm0.1Pt0.3', '-1.105', '-0.24581807775']
AlPmRh 1 ['Al0.6Rh0.3Pm0.1', '-1.062', '-0.22292459725']
AlPmS 23 ['Al0.375S0.375Pm0.25', '-1.592', '-0.216485971619']
AlPmSe 21 ['Al0.3Se0.5Pm0.2', '-1.303', '-0.231302472']
AlPmTe 3 ['Al0.222222Te0.666667Pm0.111111', '-0.745', '-0.201070878611']
AlPrPt 2 ['Al0.5Pr0.1Pt0.4', '-1.223', '-0.202168058751']
AlPrRh 1 ['Al0.5Rh0.4Pr0.1', '-1.262', '-0.30627405425']
AlPrS 22 ['Al0.375S0.5Pr0.125', '-1.539', '-0.200171147523']
AlPrSe 15 ['Al0.142857Se0.428571Pr0.428571', '-1.79', '-0.202103185356']
AlPrSi 2 ['Al0.2Si0.4Pr0.4', '-0.87', '-0.233797335667']
AlPtS 8 ['Al0.222222S0.444444Pt0.333333', '-1.084', '-0.20145149262']
AlPtSc 3 ['Al0.4Sc0.2Pt0.4', '-1.237', '-0.202017514143']
AlPtSm 2 ['Al0.5Sm0.1Pt0.4', '-1.259', '-0.245835983893']

AlPtTb 1 ['Al0.555556Tb0.111111Pt0.333333', '-1.066', '-0.204185314085']
AlPtTc 2 ['Al0.6Tc0.1Pt0.3', '-1.056', '-0.2055616535']
AlPtTh 4 ['Al0.1Pt0.7Th0.2', '-1.009', '-0.239154517']
AlPtTm 3 ['Al0.5Tm0.125Pt0.375', '-1.223', '-0.226638348929']
AlPtV 1 ['Al0.5V0.125Pt0.375', '-1.152', '-0.247133910417']
AlPtY 3 ['Al0.555556Y0.111111Pt0.333333', '-1.14', '-0.203904835119']
AlPtYb 4 ['Al0.5Yb0.166667Pt0.333333', '-1.114', '-0.200336588125']
AlPtZr 3 ['Al0.428571Zr0.142857Pt0.428571', '-1.265', '-0.21675131476']
AlPuS 13 ['Al0.3S0.3Pu0.4', '-1.472', '-0.205076568294']
AlPuSe 12 ['Al0.222222Se0.555556Pu0.222222', '-1.358', '-0.200563869444']
AlRbS 22 ['Al0.333333S0.555556Rb0.111111', '-1.418', '-0.209207657908']
AlRbSe 9 ['Al0.3Se0.4Rb0.3', '-1.034', '-0.201638076415']
AlReRh 1 ['Al0.6Rh0.3Re0.1', '-0.996', '-0.229830477875']
AlRhRu 3 ['Al0.555556Ru0.111111Rh0.333333', '-1.17', '-0.201113094444']
AlRhS 4 ['Al0.2S0.5Rh0.3', '-1.118', '-0.218286785301']
AlRhSc 7 ['Al0.571429Sc0.142857Rh0.285714', '-1.069', '-0.212284692441']
AlRhSi 1 ['Al0.5Si0.1Rh0.4', '-1.146', '-0.24047226075']
AlRhSm 5 ['Al0.6Rh0.3Sm0.1', '-1.042', '-0.201713489']
AlRhTb 2 ['Al0.5Rh0.4Tb0.1', '-1.136', '-0.212799546517']
AlRhTc 4 ['Al0.555556Tc0.111111Rh0.333333', '-1.065', '-0.218315508472']
AlRhTi 8 ['Al0.5Ti0.166667Rh0.333333', '-1.096', '-0.219187274369']
AlRhTm 4 ['Al0.5Rh0.375Tm0.125', '-1.167', '-0.20885966625']
AlRhV 1 ['Al0.5V0.1Rh0.4', '-1.121', '-0.201869379']
AlRhY 5 ['Al0.428571Y0.142857Rh0.428571', '-1.194', '-0.225770381905']
AlRhYb 5 ['Al0.444444Rh0.444444Yb0.111111', '-1.204', '-0.2287078']
AlRhZr 7 ['Al0.625Zr0.125Rh0.25', '-1.015', '-0.222419710625']
AlRuS 1 ['Al0.142857S0.571429Ru0.285714', '-1.052', '-0.217269596456']
AlSSc 3 ['Al0.142857S0.428571Sc0.428571', '-1.95', '-0.212422736611']
AlSSm 17 ['Al0.222222S0.333333Sm0.444444', '-1.854', '-0.235514886438']
AlSSr 2 ['Al0.285714S0.428571Sr0.285714', '-1.853', '-0.2150433281']
AlSTa 4 ['Al0.111111S0.666667Ta0.222222', '-1.396', '-0.206654762647']
AlSTb 10 ['Al0.125S0.5Tb0.375', '-1.905', '-0.200256012289']
AlSTc 6 ['Al0.142857S0.571429Tc0.285714', '-1.08', '-0.208397737527']
AlSTh 22 ['Al0.1S0.6Th0.3', '-2.177', '-0.212858733338']
AlSTm 17 ['Al0.1S0.5Tm0.4', '-2.186', '-0.212766777179']
AlSU 2 ['Al0.166667S0.5U0.333333', '-1.574', '-0.201013359242']
AlSXe 1 ['Al0.1S0.5Xe0.4', '-0.514', '-0.206546822273']
AlSY 24 ['Al0.428571S0.285714Y0.285714', '-1.446', '-0.202661275386']
AlSYb 3 ['Al0.222222S0.444444Yb0.333333', '-2.042', '-0.208385354047']
AlSZr 19 ['Al0.1S0.4Zr0.5', '-1.5', '-0.206966327892']
AlScSe 4 ['Al0.111111Sc0.333333Se0.555556', '-1.505', '-0.207155805278']
AlSeSm 6 ['Al0.1Se0.5Sm0.4', '-1.898', '-0.208769271']
AlSeSr 2 ['Al0.285714Se0.428571Sr0.285714', '-1.662', '-0.250069759763']
AlSeTb 9 ['Al0.25Se0.5Tb0.25', '-1.39', '-0.202189409665']

AlSeTc 6 ['Al0.2Se0.6Tc0.2', '-0.637', '-0.2136945525']
AlSeTh 16 ['Al0.375Se0.375Th0.25', '-1.28', '-0.201855558748']
AlSeTm 14 ['Al0.111111Se0.444444Tm0.444444', '-1.702', '-0.229667764444']
AlSeY 18 ['Al0.25Se0.625Y0.125', '-1.176', '-0.20539288375']
AlSeYb 1 ['Al0.222222Se0.444444Yb0.333333', '-1.781', '-0.22011395463']
AlSeZr 14 ['Al0.222222Se0.555556Zr0.222222', '-1.276', '-0.203230012778']
AsAuBr 15 ['As0.2Br0.7Au0.1', '-0.831', '-0.230282719309']
AsAuCl 5 ['Cl0.777778As0.111111Au0.111111', '-0.936', '-0.245258696185']
AsAuEu 1 ['As0.285714Eu0.428571Au0.285714', '-1.311', '-0.205984459426']
AsAuNa 3 ['Na0.5As0.375Au0.125', '-0.718', '-0.216381506762']
AsAuNd 2 ['As0.4Nd0.3Au0.3', '-1.151', '-0.23826413475']
AsAuO 2 ['O0.625As0.25Au0.125', '-1.457', '-0.269679246955']
AsAuPm 4 ['As0.4Pm0.5Au0.1', '-1.234', '-0.21276215275']
AsAuS 1 ['S0.444444As0.444444Au0.111111', '-0.449', '-0.201436359327']
AsAuSr 2 ['As0.333333Sr0.5Au0.166667', '-1.229', '-0.201680969793']
AsAuYb 2 ['As0.3Yb0.4Au0.3', '-1.122', '-0.206166919001']
AsBBr 1 ['B0.1As0.1Br0.8', '-0.868', '-0.29115986205']
AsBCl 13 ['B0.333333Cl0.444444As0.222222', '-0.991', '-0.215759135036']
AsBF 4 ['B0.2F0.7As0.1', '-2.924', '-0.212620790525']
AsBI 12 ['B0.2As0.2I0.6', '-0.557', '-0.278065696972']
AsBaBr 33 ['As0.2Br0.3Ba0.5', '-1.83', '-0.202942295984']
AsBaCl 25 ['Cl0.714286As0.142857Ba0.142857', '-1.963', '-0.202295460985']
AsBaF 11 ['F0.111111As0.333333Ba0.555556', '-1.709', '-0.202915950256']
AsBaH 29 ['H0.428571As0.142857Ba0.428571', '-0.88', '-0.214400379548']
AsBaI 16 ['As0.2I0.6Ba0.2', '-1.434', '-0.200833701707']
AsBaN 2 ['N0.166667As0.333333Ba0.5', '-1.075', '-0.201509893812']
AsBaO 23 ['O0.3As0.4Ba0.3', '-1.884', '-0.217782206412']
AsBaP 5 ['P0.111111As0.333333Ba0.555556', '-1.218', '-0.223886694798']
AsBaPb 2 ['As0.428571Ba0.428571Pb0.142857', '-1.0', '-0.211422680318']
AsBaPd 3 ['As0.4Pd0.3Ba0.3', '-1.124', '-0.233180263']
AsBaPm 1 ['As0.4Ba0.1Pm0.5', '-1.164', '-0.27054588305']
AsBaPt 2 ['As0.4Ba0.3Pt0.3', '-1.143', '-0.2047974305']
AsBaS 11 ['S0.142857As0.285714Ba0.571429', '-1.548', '-0.212070675752']
AsBaSe 20 ['As0.375Se0.25Ba0.375', '-1.544', '-0.20338013']
AsBeBr 2 ['Be0.1As0.1Br0.8', '-0.852', '-0.204279834292']
AsBeF 2 ['Be0.1F0.8As0.1', '-2.632', '-0.221031168106']
AsBeK 1 ['Be0.111111K0.555556As0.333333', '-0.685', '-0.256758796667']
AsBiBr 2 ['As0.111111Br0.777778Bi0.111111', '-0.979', '-0.230534673946']
AsBiNa 1 ['Na0.555556As0.333333Bi0.111111', '-0.724', '-0.278489081054']
AsBiPm 3 ['As0.333333Pm0.555556Bi0.111111', '-1.125', '-0.216646491111']
AsBrCa 32 ['Ca0.333333As0.111111Br0.555556', '-2.221', '-0.207660801518']
AsBrCd 3 ['As0.125Br0.75Cd0.125', '-0.968', '-0.218777789114']
AsBrCe 38 ['As0.4Br0.1Ce0.5', '-1.68', '-0.200217211259']
AsBrCl 21 ['Cl0.1As0.2Br0.7', '-0.786', '-0.201397449563']

AsBrCo 13 ['Co0.222222As0.222222Br0.555556', '-0.773', '-0.245510104213']
AsBrCr 14 ['Cr0.25As0.125Br0.625', '-0.982', '-0.231419484115']
AsBrCs 4 ['As0.166667Br0.666667Cs0.166667', '-1.326', '-0.217329926722']
AsBrCu 17 ['Cu0.1As0.2Br0.7', '-0.848', '-0.218716562809']
AsBrDy 35 ['As0.333333Br0.5Dy0.166667', '-1.186', '-0.200806986016']
AsBrEr 47 ['As0.444444Br0.111111Er0.444444', '-1.605', '-0.205417952648']
AsBrEu 46 ['As0.2Br0.7Eu0.1', '-1.389', '-0.20765366771']
AsBrF 5 ['F0.166667As0.166667Br0.666667', '-1.059', '-0.200475733803']
AsBrFe 8 ['Fe0.125As0.125Br0.75', '-0.892', '-0.216535035063']
AsBrGa 9 ['Ga0.166667As0.166667Br0.666667', '-1.066', '-0.202776677971']
AsBrGd 22 ['As0.2Br0.5Gd0.3', '-2.05', '-0.209956804373']
AsBrGe 1 ['Ge0.1As0.1Br0.8', '-0.939', '-0.224160445809']
AsBrH 21 ['H0.428571As0.285714Br0.285714', '-0.467', '-0.207430520738']
AsBrHf 36 ['As0.4Br0.2Hf0.4', '-1.107', '-0.21969210435']
AsBrHg 3 ['As0.222222Br0.666667Hg0.111111', '-0.828', '-0.203178396726']
AsBrHo 53 ['As0.5Br0.1Ho0.4', '-1.519', '-0.205691162922']
AsBrI 15 ['As0.1Br0.7I0.2', '-0.596', '-0.200830349806']
AsBrIn 2 ['As0.111111Br0.777778In0.111111', '-1.023', '-0.240374361168']
AsBrIr 10 ['As0.2Br0.6Ir0.2', '-0.761', '-0.207004561884']
AsBrK 16 ['K0.25As0.25Br0.5', '-1.397', '-0.205849800041']
AsBrKr 13 ['As0.2Br0.7Kr0.1', '-0.746', '-0.20090409355']
AsBrLa 23 ['As0.2Br0.6La0.2', '-2.009', '-0.202432618552']
AsBrLi 13 ['Li0.25As0.25Br0.5', '-1.289', '-0.208235193791']
AsBrLu 41 ['As0.4Br0.2Lu0.4', '-1.498', '-0.234452832267']
AsBrMg 3 ['Mg0.1As0.1Br0.8', '-1.044', '-0.256039391374']
AsBrMn 11 ['Mn0.222222As0.111111Br0.666667', '-1.104', '-0.221504851994']
AsBrMo 7 ['As0.2Br0.6Mo0.2', '-0.916', '-0.201708988717']
AsBrNa 7 ['Na0.5As0.166667Br0.333333', '-1.576', '-0.22864060398']
AsBrNb 5 ['As0.142857Br0.714286Nb0.142857', '-1.174', '-0.219703931843']
AsBrNd 18 ['As0.333333Br0.444444Nd0.222222', '-1.696', '-0.205335903372']
AsBrNi 21 ['Ni0.1As0.3Br0.6', '-0.812', '-0.207025329765']
AsBrNp 8 ['As0.1Br0.7Np0.2', '-1.633', '-0.272297151058']
AsBrO 18 ['O0.125As0.125Br0.75', '-0.642', '-0.200124884472']
AsBrOs 10 ['As0.111111Br0.666667Os0.222222', '-0.504', '-0.201168940861']
AsBrP 9 ['P0.2As0.3Br0.5', '-0.658', '-0.203753411292']
AsBrPa 4 ['As0.111111Br0.777778Pa0.111111', '-1.542', '-0.201093045621']
AsBrPb 1 ['As0.111111Br0.777778Pb0.111111', '-0.916', '-0.240800168381']
AsBrPd 8 ['As0.1Br0.8Pd0.1', '-0.644', '-0.214159223792']
AsBrPm 68 ['As0.6Br0.1Pm0.3', '-0.949', '-0.210758948008']
AsBrPr 37 ['As0.125Br0.5Pr0.375', '-2.025', '-0.20132158273']
AsBrPt 18 ['As0.285714Br0.571429Pt0.142857', '-0.81', '-0.216376735643']
AsBrPu 39 ['As0.3Br0.3Pu0.4', '-1.536', '-0.206485111276']
AsBrRb 9 ['As0.222222Br0.666667Rb0.111111', '-1.136', '-0.202929591722']
AsBrRe 8 ['As0.111111Br0.666667Re0.222222', '-0.548', '-0.245168940861']

AsBrRh 17 ['As0.2Br0.5Rh0.3', '-0.822', '-0.207766644848']
AsBrRu 14 ['As0.285714Br0.571429Ru0.142857', '-0.782', '-0.20490437481']
AsBrS 5 ['S0.375As0.125Br0.5', '-0.628', '-0.20023416062']
AsBrSb 3 ['As0.142857Br0.714286Sb0.142857', '-0.947', '-0.21144243006']
AsBrSc 45 ['Sc0.5As0.166667Br0.333333', '-1.289', '-0.223082412528']
AsBrSe 11 ['As0.111111Se0.333333Br0.555556', '-0.609', '-0.200100632547']
AsBrSi 8 ['Si0.1As0.2Br0.7', '-1.053', '-0.24075636931']
AsBrSm 33 ['As0.4Br0.1Sm0.5', '-1.712', '-0.205707514341']
AsBrSn 1 ['As0.1Br0.8Sn0.1', '-0.961', '-0.214168789874']
AsBrSr 14 ['As0.3Br0.4Sr0.3', '-1.87', '-0.209988787032']
AsBrTa 3 ['As0.125Br0.75Ta0.125', '-1.107', '-0.306764753911']
AsBrTb 54 ['As0.4Br0.1Tb0.5', '-1.404', '-0.200987082103']
AsBrTc 11 ['As0.25Br0.625Tc0.125', '-0.827', '-0.230622360573']
AsBrTe 4 ['As0.142857Br0.714286Te0.142857', '-0.68', '-0.232817953137']
AsBrTh 38 ['As0.444444Br0.333333Th0.222222', '-1.475', '-0.207629364333']
AsBrTi 4 ['Ti0.111111As0.222222Br0.666667', '-1.222', '-0.208998065469']
AsBrTl 3 ['As0.111111Br0.777778Tl0.111111', '-0.806', '-0.216811977408']
AsBrTm 54 ['As0.375Br0.5Tm0.125', '-1.041', '-0.211093196917']
AsBrU 13 ['As0.3Br0.4U0.3', '-1.365', '-0.213413622535']
AsBrV 15 ['V0.111111As0.222222Br0.666667', '-0.952', '-0.207500305055']
AsBrW 10 ['As0.1Br0.7W0.2', '-0.923', '-0.236757992642']
AsBrXe 9 ['As0.166667Br0.666667Xe0.166667', '-0.666', '-0.211753411292']
AsBrY 53 ['As0.333333Br0.166667Y0.5', '-1.623', '-0.209120451263']
AsBrYb 51 ['As0.222222Br0.666667Yb0.111111', '-1.378', '-0.200674887278']
AsBrZn 2 ['Zn0.111111As0.111111Br0.777778', '-0.855', '-0.207617959215']
AsBrZr 24 ['As0.333333Br0.333333Zr0.333333', '-1.47', '-0.210678379473']
AsCCa 1 ['C0.1Ca0.5As0.4', '-1.237', '-0.237553148337']
AsCHf 22 ['C0.333333As0.222222Hf0.444444', '-1.085', '-0.200406550833']
AsCLu 1 ['C0.3As0.2Lu0.5', '-1.007', '-0.263512530536']
AsCNb 1 ['C0.25As0.375Nb0.375', '-0.73', '-0.217738837812']
AsCNd 1 ['C0.1As0.4Nd0.5', '-1.473', '-0.23316502425']
AsCNp 9 ['C0.5As0.125Np0.375', '-0.372', '-0.201660474531']
AsCPa 4 ['C0.5As0.1Pa0.4', '-0.687', '-0.20224453325']
AsCPm 4 ['C0.111111As0.444444Pm0.444444', '-1.207', '-0.247901134444']
AsCSr 3 ['C0.125As0.375Sr0.5', '-1.247', '-0.253922494064']
AsCTa 2 ['C0.222222As0.333333Ta0.444444', '-0.745', '-0.206460788333']
AsCTc 2 ['C0.222222As0.333333Tc0.444444', '-0.39', '-0.232729310556']
AsCU 2 ['C0.428571As0.285714U0.285714', '-0.61', '-0.200949525714']
AsCZr 4 ['C0.428571As0.142857Zr0.428571', '-0.996', '-0.203779151786']
AsCaCl 11 ['Cl0.5Ca0.333333As0.166667', '-2.383', '-0.206241778128']
AsCaCu 1 ['Ca0.444444Cu0.222222As0.333333', '-1.126', '-0.213370811711']
AsCaF 4 ['F0.25Ca0.5As0.25', '-2.491', '-0.210624922304']
AsCaH 42 ['H0.4Ca0.5As0.1', '-0.843', '-0.201600244461']
AsCaN 8 ['N0.4Ca0.3As0.3', '-0.879', '-0.20650179728']

AsCaO 1 ['O0.625Ca0.125As0.25', '-2.33', '-0.268300097891']
AsCaPd 1 ['Ca0.444444As0.333333Pd0.222222', '-1.184', '-0.211404640002']
AsCaRh 1 ['Ca0.2As0.4Rh0.4', '-1.033', '-0.248978607779']
AsCaS 1 ['S0.25Ca0.5As0.25', '-1.934', '-0.22602905858']
AsCaSe 10 ['Ca0.555556As0.333333Se0.111111', '-1.521', '-0.201346463892']
AsCaSi 2 ['Si0.111111Ca0.444444As0.444444', '-1.189', '-0.209069354818']
AsCaSn 1 ['Ca0.428571As0.428571Sn0.142857', '-1.178', '-0.225569482023']
AsCdCl 32 ['Cl0.125As0.375Cd0.5', '-0.539', '-0.202751369792']
AsCdO 1 ['O0.6As0.2Cd0.2', '-1.731', '-0.302568896725']
AsCeCl 21 ['Cl0.5As0.2Ce0.3', '-2.321', '-0.200412943107']
AsCeF 8 ['F0.25As0.375Ce0.375', '-2.526', '-0.201823624974']
AsCeH 1 ['H0.333333As0.333333Ce0.333333', '-1.247', '-0.256303799167']
AsCeI 47 ['As0.142857I0.428571Ce0.428571', '-1.452', '-0.2009906736']
AsCeO 13 ['O0.2As0.4Ce0.4', '-2.177', '-0.21098320274']
AsCeS 1 ['S0.428571As0.142857Ce0.428571', '-2.223', '-0.208244596493']
AsCeSe 1 ['As0.3Se0.3Ce0.4', '-1.771', '-0.20855854796']
AsClCr 3 ['Cl0.555556Cr0.333333As0.111111', '-1.269', '-0.201571322252']
AsClCs 4 ['Cl0.75As0.125Cs0.125', '-1.296', '-0.206538654014']
AsClCu 1 ['Cl0.6Cu0.3As0.1', '-0.943', '-0.207992727128']
AsClDy 3 ['Cl0.666667As0.166667Dy0.166667', '-2.148', '-0.248271682366']
AsClEr 45 ['Cl0.4As0.4Er0.2', '-1.342', '-0.213280430086']
AsClEu 39 ['Cl0.5As0.25Eu0.25', '-2.318', '-0.201843759065']
AsClF 1 ['F0.7Cl0.1As0.2', '-2.384', '-0.223909826295']
AsClFe 9 ['Cl0.571429Fe0.285714As0.142857', '-1.051', '-0.2019964892']
AsClGa 6 ['Cl0.666667Ga0.222222As0.111111', '-1.425', '-0.207566347369']
AsClGd 24 ['Cl0.125As0.375Gd0.5', '-1.791', '-0.204176033777']
AsClGe 1 ['Cl0.625Ge0.25As0.125', '-1.168', '-0.215692326979']
AsClHg 11 ['Cl0.5As0.2Hg0.3', '-0.884', '-0.206225145367']
AsClHo 52 ['Cl0.166667As0.333333Ho0.5', '-1.677', '-0.20702746406']
AsClI 1 ['Cl0.333333As0.166667I0.5', '-0.725', '-0.218071281056']
AsClIn 17 ['Cl0.7As0.2In0.1', '-1.224', '-0.208006220301']
AsClIr 7 ['Cl0.666667As0.111111Ir0.222222', '-0.708', '-0.222979260767']
AsClK 11 ['Cl0.5K0.25As0.25', '-1.615', '-0.218561093024']
AsClLa 18 ['Cl0.7As0.1La0.2', '-2.478', '-0.200168632903']
AsClLi 1 ['Li0.2Cl0.7As0.1', '-1.439', '-0.202543503567']
AsClLu 46 ['Cl0.375As0.125Lu0.5', '-1.515', '-0.216481768518']
AsClNd 18 ['Cl0.2As0.4Nd0.4', '-1.897', '-0.203594044211']
AsClNi 2 ['Cl0.777778Ni0.111111As0.111111', '-0.98', '-0.216573845074']
AsClNp 7 ['Cl0.75As0.125Np0.125', '-1.713', '-0.216927026147']
AsClPa 14 ['Cl0.3As0.3Pa0.4', '-1.608', '-0.204065354813']
AsClPb 26 ['Cl0.8As0.1Pb0.1', '-1.072', '-0.203514513755']
AsClPm 56 ['Cl0.6As0.3Pm0.1', '-1.441', '-0.204244876878']
AsClPr 40 ['Cl0.4As0.4Pr0.2', '-1.775', '-0.200553377919']
AsClPt 11 ['Cl0.571429As0.142857Pt0.285714', '-0.899', '-0.210782909884']

AsClPu 40 ['Cl0.3As0.2Pu0.5', '-1.489', '-0.205714538816']
AsClRb 10 ['Cl0.571429As0.285714Rb0.142857', '-1.368', '-0.200214504646']
AsClRe 3 ['Cl0.666667As0.222222Re0.111111', '-1.104', '-0.234960255143']
AsClRh 14 ['Cl0.571429As0.142857Rh0.285714', '-0.842', '-0.250866749646']
AsClRu 7 ['Cl0.7As0.1Ru0.2', '-1.049', '-0.2305605139']
AsClSc 20 ['Cl0.5Sc0.333333As0.166667', '-2.254', '-0.203425873027']
AsClSi 2 ['Si0.2Cl0.7As0.1', '-1.552', '-0.209659581781']
AsClSm 42 ['Cl0.1As0.5Sm0.4', '-1.596', '-0.213416727063']
AsClSr 25 ['Cl0.25As0.375Sr0.375', '-1.795', '-0.202854214346']
AsClTa 2 ['Cl0.714286As0.142857Ta0.142857', '-1.53', '-0.213212764316']
AsClTb 41 ['Cl0.1As0.4Tb0.5', '-1.487', '-0.200965010585']
AsClTc 9 ['Cl0.714286As0.142857Tc0.142857', '-1.153', '-0.207956594987']
AsClTe 11 ['Cl0.571429As0.142857Te0.285714', '-0.919', '-0.201238882831']
AsClTh 10 ['Cl0.428571As0.285714Th0.285714', '-2.076', '-0.202779830089']
AsClTl 3 ['Cl0.666667As0.166667Tl0.166667', '-1.257', '-0.203849907921']
AsClTm 10 ['Cl0.1As0.4Tm0.5', '-1.75', '-0.212141495439']
AsClU 20 ['Cl0.75As0.125U0.125', '-1.802', '-0.207181303074']
AsClW 2 ['Cl0.666667As0.166667W0.166667', '-1.141', '-0.215409717917']
AsClXe 2 ['Cl0.7As0.1Xe0.2', '-0.663', '-0.226481334691']
AsClY 22 ['Cl0.2As0.4Y0.4', '-1.921', '-0.202940112544']
AsClYb 19 ['Cl0.571429As0.285714Yb0.142857', '-1.748', '-0.2059231356']
AsClZr 5 ['Cl0.714286As0.142857Zr0.142857', '-1.871', '-0.212116729316']
AsCoF 13 ['F0.6Co0.2As0.2', '-2.13', '-0.23022236552']
AsCoN 33 ['N0.166667Co0.333333As0.5', '-0.474', '-0.20632258125']
AsCoO 1 ['O0.625Co0.125As0.25', '-1.714', '-0.323963340458']
AsCoPm 2 ['Co0.125As0.375Pm0.5', '-1.024', '-0.208063825625']
AsCoS 6 ['S0.333333Co0.222222As0.444444', '-0.633', '-0.202075309218']
AsCrF 1 ['F0.777778Cr0.111111As0.111111', '-2.782', '-0.21312332234']
AsCrI 5 ['Cr0.142857As0.285714I0.571429', '-0.51', '-0.205967775755']
AsCsF 1 ['F0.8As0.1Cs0.1', '-2.352', '-0.206927334024']
AsCsH 9 ['H0.142857As0.285714Cs0.571429', '-0.502', '-0.204814483855']
AsCsI 1 ['As0.142857I0.714286Cs0.142857', '-0.912', '-0.213056592909']
AsCsK 6 ['K0.5As0.375Cs0.125', '-0.587', '-0.200293015833']
AsCsP 2 ['P0.125As0.375Cs0.5', '-0.681', '-0.235025262086']
AsCsPm 1 ['As0.4Cs0.1Pm0.5', '-1.076', '-0.212811021']
AsCsS 3 ['S0.428571As0.142857Cs0.428571', '-1.14', '-0.200051813991']
AsCsSe 7 ['As0.2Se0.3Cs0.5', '-1.035', '-0.21560102241']
AsCuDy 2 ['Cu0.3As0.4Dy0.3', '-1.154', '-0.209696572501']
AsCuEu 1 ['Cu0.222222As0.333333Eu0.444444', '-1.268', '-0.23392244457']
AsCuK 3 ['K0.555556Cu0.111111As0.333333', '-0.594', '-0.202019087778']
AsCuLi 1 ['Li0.6Cu0.1As0.3', '-0.804', '-0.205559953125']
AsCuNa 7 ['Na0.5Cu0.1As0.4', '-0.643', '-0.200141523949']
AsCuO 2 ['O0.2Cu0.7As0.1', '-0.669', '-0.204817598655']
AsCuPm 6 ['Cu0.166667As0.333333Pm0.5', '-0.989', '-0.214843282083']

AsCuSr 4 ['Cu0.25As0.375Sr0.375', '-1.102', '-0.218445925']
AsCuY 3 ['Cu0.3As0.4Y0.3', '-1.193', '-0.240198908']
AsCuYb 8 ['Cu0.333333As0.333333Yb0.333333', '-0.982', '-0.209354565001']
AsDyI 37 ['As0.428571I0.142857Dy0.428571', '-1.539', '-0.203210430211']
AsDyO 3 ['O0.666667As0.222222Dy0.111111', '-2.445', '-0.202996315343']
AsDyS 5 ['S0.333333As0.222222Dy0.444444', '-2.141', '-0.209367390426']
AsErH 11 ['H0.4As0.4Er0.2', '-0.824', '-0.216704277']
AsErI 40 ['As0.428571I0.142857Er0.428571', '-1.555', '-0.234308767763']
AsErN 3 ['N0.333333As0.333333Er0.333333', '-1.459', '-0.201423751553']
AsErO 3 ['O0.666667As0.166667Er0.166667', '-2.665', '-0.249851201916']
AsErS 1 ['S0.4As0.2Er0.4', '-2.142', '-0.240809896173']
AsEuF 4 ['F0.222222As0.333333Eu0.444444', '-2.538', '-0.206107887417']
AsEuH 23 ['H0.2As0.4Eu0.4', '-1.172', '-0.200280607336']
AsEuI 38 ['As0.166667I0.666667Eu0.166667', '-1.282', '-0.203011127823']
AsEuLi 12 ['Li0.125As0.375Eu0.5', '-1.357', '-0.201284561204']
AsEuMg 2 ['Mg0.25As0.375Eu0.375', '-1.202', '-0.214526835591']
AsEuN 30 ['N0.4As0.3Eu0.3', '-0.947', '-0.218210455502']
AsEuNi 1 ['Ni0.125As0.375Eu0.5', '-1.339', '-0.21452310191']
AsEuO 6 ['O0.1As0.3Eu0.6', '-1.892', '-0.218353297246']
AsEuP 1 ['P0.3As0.1Eu0.6', '-1.206', '-0.210414493896']
AsEuPd 2 ['As0.333333Pd0.222222Eu0.444444', '-1.351', '-0.207091139163']
AsEuPm 3 ['As0.428571Pm0.428571Eu0.142857', '-1.262', '-0.243634732427']
AsEuS 29 ['S0.444444As0.111111Eu0.444444', '-2.405', '-0.209146935991']
AsEuSe 28 ['As0.142857Se0.428571Eu0.428571', '-2.093', '-0.201384804286']
AsEuZn 4 ['Zn0.444444As0.444444Eu0.111111', '-0.601', '-0.208381137406']
AsFFe 23 ['F0.428571Fe0.285714As0.285714', '-1.713', '-0.215980559033']
AsFGd 1 ['F0.25As0.375Gd0.375', '-2.641', '-0.356155427614']
AsFH 7 ['H0.1F0.8As0.1', '-1.901', '-0.2086244603']
AsFHf 1 ['F0.8As0.1Hf0.1', '-3.409', '-0.202379049196']
AsFIr 1 ['F0.8As0.1Ir0.1', '-2.191', '-0.202220368192']
AsFK 5 ['F0.111111K0.555556As0.333333', '-1.181', '-0.202176075651']
AsFLa 19 ['F0.6As0.1La0.3', '-4.098', '-0.200528016356']
AsFLu 8 ['F0.571429As0.142857Lu0.285714', '-3.564', '-0.200859797638']
AsFMo 1 ['F0.777778As0.111111Mo0.111111', '-2.696', '-0.214464798822']
AsFN 7 ['N0.166667F0.5As0.333333', '-1.803', '-0.219851971267']
AsFNa 21 ['F0.555556Na0.333333As0.111111', '-2.888', '-0.201714527236']
AsFNd 2 ['F0.166667As0.333333Nd0.5', '-2.193', '-0.207823919218']
AsFNi 7 ['F0.7Ni0.2As0.1', '-2.305', '-0.200046119108']
AsFNp 2 ['F0.75As0.125Np0.125', '-3.372', '-0.222589921015']
AsFO 19 ['O0.375F0.25As0.375', '-1.896', '-0.200629120302']
AsFP 3 ['F0.777778P0.111111As0.111111', '-2.839', '-0.233432465603']
AsFPa 15 ['F0.428571As0.285714Pa0.285714', '-2.663', '-0.208277251086']
AsFPd 1 ['F0.75As0.125Pd0.125', '-2.193', '-0.231649114712']
AsFPm 31 ['F0.3As0.2Pm0.5', '-2.311', '-0.211700824512']

AsFPr 10 ['F0.111111As0.333333Pr0.555556', '-1.861', '-0.210238819171']
AsFPt 2 ['F0.777778As0.111111Pt0.111111', '-2.147', '-0.211903847063']
AsFPu 8 ['F0.625As0.125Pu0.25', '-3.56', '-0.221503803458']
AsFRe 2 ['F0.777778As0.111111Re0.111111', '-2.421', '-0.21925622504']
AsFRh 6 ['F0.666667As0.111111Rh0.222222', '-2.095', '-0.242066736689']
AsFRu 2 ['F0.777778As0.111111Ru0.111111', '-2.353', '-0.238188782344']
AsFSi 1 ['F0.8Si0.1As0.1', '-3.044', '-0.212796241196']
AsFSr 20 ['F0.555556As0.111111Sr0.333333', '-3.834', '-0.200080112258']
AsFTc 1 ['F0.8As0.1Tc0.1', '-2.483', '-0.206945011321']
AsFTe 4 ['F0.666667As0.166667Te0.166667', '-2.34', '-0.263931682523']
AsFTi 2 ['F0.777778Ti0.111111As0.111111', '-3.093', '-0.2018353047']
AsFTl 2 ['F0.555556As0.111111Tl0.333333', '-2.21', '-0.224884162516']
AsFTm 54 ['F0.25As0.25Tm0.5', '-2.044', '-0.203107861465']
AsFU 8 ['F0.428571As0.285714U0.285714', '-2.568', '-0.201702149419']
AsFV 3 ['F0.777778V0.111111As0.111111', '-2.921', '-0.200290460678']
AsFY 3 ['F0.444444As0.222222Y0.333333', '-3.294', '-0.230015132359']
AsFYb 61 ['F0.5As0.375Yb0.125', '-2.197', '-0.206205748142']
AsFeI 2 ['Fe0.2As0.3I0.5', '-0.487', '-0.210627588586']
AsFeO 3 ['O0.6Fe0.1As0.3', '-1.709', '-0.209685055219']
AsFePm 2 ['Fe0.125As0.375Pm0.5', '-1.036', '-0.226760332187']
AsFeS 2 ['S0.444444Fe0.111111As0.444444', '-0.578', '-0.208933425438']
AsGaK 2 ['K0.6Ga0.1As0.3', '-0.622', '-0.229678474205']
AsGdH 2 ['H0.25As0.375Gd0.375', '-1.367', '-0.210196140313']
AsGdI 50 ['As0.142857I0.428571Gd0.428571', '-1.384', '-0.2167634336']
AsGdO 3 ['O0.666667As0.222222Gd0.111111', '-2.298', '-0.252101401083']
AsGeK 2 ['K0.555556Ge0.111111As0.333333', '-0.598', '-0.22375617']
AsGePm 2 ['Ge0.1As0.3Pm0.6', '-0.986', '-0.216074884']
AsGeS 4 ['S0.5Ge0.2As0.3', '-0.574', '-0.206647387493']
AsGeSe 4 ['Ge0.25As0.375Se0.375', '-0.375', '-0.210992394219']
AsHHo 2 ['H0.4As0.3Ho0.3', '-1.126', '-0.209155360498']
AsHI 16 ['H0.222222As0.222222I0.555556', '-0.442', '-0.200637178281']
AsHK 34 ['H0.555556K0.222222As0.222222', '-0.408', '-0.202343955556']
AsHLa 2 ['H0.4As0.3La0.3', '-1.156', '-0.2137445995']
AsHLi 35 ['H0.2Li0.4As0.4', '-0.598', '-0.2032869105']
AsHNa 12 ['H0.125Na0.5As0.375', '-0.612', '-0.204390373194']
AsHNd 4 ['H0.285714As0.428571Nd0.285714', '-1.072', '-0.206988488571']
AsHO 7 ['H0.444444O0.333333As0.222222', '-1.261', '-0.204013421145']
AsHPm 37 ['H0.5As0.25Pm0.25', '-0.746', '-0.206506888125']
AsHRb 37 ['H0.1As0.4Rb0.5', '-0.579', '-0.200569907282']
AsHSr 21 ['H0.555556As0.111111Sr0.333333', '-0.803', '-0.201870453474']
AsHYb 25 ['H0.3As0.4Yb0.3', '-0.83', '-0.210181132252']
AsHfN 6 ['N0.2As0.3Hf0.5', '-1.553', '-0.207157622182']
AsHfO 3 ['O0.7As0.2Hf0.1', '-2.385', '-0.283398704887']
AsHfS 5 ['S0.428571As0.142857Hf0.428571', '-1.664', '-0.201946228279']

AsHgI 9 ['As0.2I0.5Hg0.3', '-0.514', '-0.213871116546']
AsHoI 30 ['As0.285714I0.571429Ho0.142857', '-0.882', '-0.241596937818']
AsHoO 2 ['O0.142857As0.428571Ho0.428571', '-2.145', '-0.20387042886']
AsIIn 2 ['As0.2In0.2I0.6', '-0.71', '-0.208429925043']
AsIK 7 ['K0.444444As0.333333I0.222222', '-1.131', '-0.205971121094']
AsILa 32 ['As0.222222I0.666667La0.111111', '-1.091', '-0.203041801715']
AsILi 4 ['Li0.5As0.166667I0.333333', '-1.259', '-0.213244770235']
AsILu 38 ['As0.333333I0.444444Lu0.222222', '-1.05', '-0.251160791413']
AsINa 7 ['Na0.4As0.3I0.3', '-1.142', '-0.202314076056']
AsINd 53 ['As0.25I0.25Nd0.5', '-1.504', '-0.214472111266']
AsINi 18 ['Ni0.1As0.2I0.7', '-0.506', '-0.204894065757']
AsINp 13 ['As0.285714I0.571429Np0.142857', '-0.839', '-0.201016552124']
AsIO 14 ['O0.2As0.2I0.6', '-0.779', '-0.210084389338']
AsIP 7 ['P0.2As0.3I0.5', '-0.422', '-0.204773460453']
AsIPa 37 ['As0.4I0.3Pa0.3', '-0.967', '-0.201144107772']
AsIPd 20 ['As0.2Pd0.1I0.7', '-0.546', '-0.212103820658']
AsIPm 67 ['As0.555556I0.111111Pm0.333333', '-0.989', '-0.221403286489']
AsIPr 33 ['As0.5I0.1Pr0.4', '-1.5', '-0.203892415257']
AsIPt 25 ['As0.222222I0.444444Pt0.333333', '-0.585', '-0.233943320865']
AsIPu 7 ['As0.142857I0.714286Pu0.142857', '-1.016', '-0.201622996362']
AsIRb 1 ['As0.3Rb0.6I0.1', '-0.815', '-0.215630377008']
AsIRe 12 ['As0.25I0.625Re0.125', '-0.496', '-0.220203158661']
AsIRh 19 ['As0.166667Rh0.333333I0.5', '-0.471', '-0.232237120834']
AsIRu 6 ['As0.111111Ru0.222222I0.666667', '-0.352', '-0.207182306968']
AsIS 13 ['S0.3As0.3I0.4', '-0.514', '-0.20219201471']
AsISc 7 ['Sc0.166667As0.166667I0.666667', '-0.962', '-0.201579810323']
AsISe 11 ['As0.111111Se0.111111I0.777778', '-0.347', '-0.202182306968']
AsISi 2 ['Si0.142857As0.142857I0.714286', '-0.651', '-0.223911168027']
AsISm 48 ['As0.333333I0.555556Sm0.111111', '-0.801', '-0.219808870225']
AsISr 12 ['As0.285714Sr0.428571I0.285714', '-1.611', '-0.208794386091']
AsITb 48 ['As0.5I0.1Tb0.4', '-1.22', '-0.207276108227']
AsITc 6 ['As0.285714Tc0.142857I0.571429', '-0.498', '-0.204806614962']
AsITl 3 ['As0.1I0.7Tl0.2', '-0.673', '-0.2043146893']
AsITm 39 ['As0.428571I0.142857Tm0.428571', '-1.549', '-0.202333267367']
AsIU 7 ['As0.2I0.7U0.1', '-0.817', '-0.2021322198']
AsIV 4 ['V0.25As0.125I0.625', '-0.695', '-0.203912675513']
AsIW 7 ['As0.2I0.7W0.1', '-0.466', '-0.205328152543']
AsIXe 21 ['As0.25I0.5Xe0.25', '-0.42', '-0.202773460453']
AsIY 2 ['As0.285714Y0.428571I0.285714', '-1.702', '-0.217300951688']
AsIYb 19 ['As0.25I0.25Yb0.5', '-1.572', '-0.216434216061']
AsIZr 9 ['As0.142857Zr0.142857I0.714286', '-0.948', '-0.201525908144']
AsInO 4 ['O0.666667As0.166667In0.166667', '-1.765', '-0.205098845562']
AsIrPm 1 ['As0.4Pm0.5Ir0.1', '-1.227', '-0.24287256275']
AsIrS 1 ['S0.444444As0.444444Ir0.111111', '-0.577', '-0.206070763537']

AsKLi 1 ['Li0.125K0.5As0.375', '-0.675', '-0.210405867188']
AsKMg 2 ['Mg0.1K0.5As0.4', '-0.71', '-0.203544368083']
AsKN 2 ['N0.444444K0.333333As0.222222', '-0.552', '-0.200307635576']
AsKNa 5 ['Na0.1K0.5As0.4', '-0.652', '-0.211599740915']
AsKO 27 ['O0.142857K0.571429As0.285714', '-0.966', '-0.202836272017']
AsKP 3 ['P0.142857K0.571429As0.285714', '-0.683', '-0.233433086756']
AsKPm 1 ['K0.1As0.4Pm0.5', '-1.133', '-0.269811021']
AsKRb 3 ['K0.5As0.333333Rb0.166667', '-0.572', '-0.204297422292']
AsKS 4 ['S0.111111K0.555556As0.333333', '-0.907', '-0.210909174']
AsKSe 8 ['K0.6As0.3Se0.1', '-0.839', '-0.2074514925']
AsKSi 2 ['Si0.1K0.6As0.3', '-0.593', '-0.219013733375']
AsKTl 1 ['K0.6As0.3Tl0.1', '-0.558', '-0.214100883833']
AsKrPm 1 ['As0.4Kr0.1Pm0.5', '-1.129', '-0.265811021']
AsLaO 12 ['O0.1As0.5La0.4', '-1.881', '-0.208040335285']
AsLaS 5 ['S0.285714As0.285714La0.428571', '-2.081', '-0.202130307661']
AsLaSe 10 ['As0.444444Se0.222222La0.333333', '-1.518', '-0.200807610832']
AsLaTe 2 ['As0.142857Te0.428571La0.428571', '-1.764', '-0.208697098214']
AsLiN 26 ['Li0.3N0.4As0.3', '-0.519', '-0.208735958571']
AsLiNa 2 ['Li0.6Na0.1As0.3', '-0.869', '-0.20293515904']
AsLiO 1 ['Li0.125O0.625As0.25', '-1.87', '-0.20713559945']
AsLiP 15 ['Li0.375P0.375As0.25', '-0.702', '-0.202577725435']
AsLiPm 8 ['Li0.25As0.375Pm0.375', '-1.073', '-0.203571289896']
AsLiRh 2 ['Li0.333333As0.333333Rh0.333333', '-0.804', '-0.210915567389']
AsLiSr 5 ['Li0.142857As0.428571Sr0.428571', '-1.232', '-0.20015207131']
AsLiTm 1 ['Li0.1As0.4Tm0.5', '-1.404', '-0.201887314']
AsLiY 3 ['Li0.555556As0.333333Y0.111111', '-1.105', '-0.224410628403']
AsLiYb 2 ['Li0.375As0.375Yb0.25', '-1.118', '-0.238950806993']
AsLuN 5 ['N0.285714As0.142857Lu0.571429', '-1.755', '-0.201097839903']
AsMgPm 3 ['Mg0.125As0.375Pm0.5', '-1.054', '-0.216949300729']
AsMgRu 5 ['Mg0.142857As0.428571Ru0.428571', '-0.612', '-0.262891646786']
AsMgSr 1 ['Mg0.125As0.375Sr0.5', '-1.248', '-0.232007311238']
AsNNa 28 ['N0.111111Na0.555556As0.333333', '-0.649', '-0.202652251796']
AsNNp 15 ['N0.4As0.3Np0.3', '-0.938', '-0.21052567277']
AsNP 2 ['N0.2P0.4As0.4', '-0.402', '-0.234502165256']
AsNPa 22 ['N0.5As0.25Pa0.25', '-0.815', '-0.218819260387']
AsNPm 3 ['N0.125As0.375Pm0.5', '-1.463', '-0.268375309958']
AsNPu 6 ['N0.428571As0.142857Pu0.428571', '-1.387', '-0.213466841997']
AsNSm 1 ['N0.142857As0.428571Sm0.428571', '-1.561', '-0.233878624594']
AsNTa 1 ['N0.25As0.125Ta0.625', '-1.092', '-0.211957855123']
AsNTb 2 ['N0.2As0.4Tb0.4', '-1.26', '-0.200854264064']
AsNTi 13 ['N0.4Ti0.3As0.3', '-0.979', '-0.200777906795']
AsNTm 7 ['N0.333333As0.222222Tm0.444444', '-1.822', '-0.201860560998']
AsNY 2 ['N0.142857As0.428571Y0.428571', '-1.617', '-0.215064487094']
AsNYb 24 ['N0.333333As0.333333Yb0.333333', '-0.889', '-0.200312369168']

AsNaO 21 ['O0.1Na0.6As0.3', '-0.959', '-0.204318062796']
AsNaP 3 ['Na0.5P0.166667As0.333333', '-0.702', '-0.212718728081']
AsNaPm 4 ['Na0.142857As0.428571Pm0.428571', '-1.189', '-0.251578173807']
AsNaRh 3 ['Na0.2As0.4Rh0.4', '-0.703', '-0.209013753163']
AsNaRu 1 ['Na0.142857As0.428571Ru0.428571', '-0.535', '-0.212778068053']
AsNaS 4 ['Na0.333333S0.333333As0.333333', '-1.011', '-0.210853469211']
AsNaSr 2 ['Na0.1As0.4Sr0.5', '-1.278', '-0.209049255666']
AsNbNi 2 ['Ni0.25As0.375Nb0.375', '-0.781', '-0.237494057589']
AsNdNi 1 ['Ni0.1As0.4Nd0.5', '-1.504', '-0.2279002595']
AsNdO 5 ['O0.125As0.375Nd0.5', '-2.095', '-0.205416618744']
AsNdS 10 ['S0.333333As0.333333Nd0.333333', '-1.785', '-0.204379463383']
AsNdSe 10 ['As0.444444Se0.111111Nd0.444444', '-1.703', '-0.207067480557']
AsNiPm 4 ['Ni0.142857As0.428571Pm0.428571', '-1.205', '-0.280154665357']
AsNiU 3 ['Ni0.3As0.4U0.3', '-0.817', '-0.2319168305']
AsNiYb 1 ['Ni0.4As0.4Yb0.2', '-0.883', '-0.230541354717']
AsNpS 2 ['S0.375As0.25Np0.375', '-1.515', '-0.234240722717']
AsNpSe 2 ['As0.333333Se0.5Np0.166667', '-0.752', '-0.219419816004']
AsOP 3 ['O0.666667P0.222222As0.111111', '-2.309', '-0.206437896866']
AsOPa 6 ['O0.7As0.2Pa0.1', '-2.283', '-0.403503343888']
AsOPb 1 ['O0.625As0.25Pb0.125', '-1.787', '-0.266726424452']
AsOPm 20 ['O0.3As0.3Pm0.4', '-2.481', '-0.21108704611']
AsOPr 10 ['O0.1As0.4Pr0.5', '-2.009', '-0.211393196703']
AsORb 24 ['O0.3As0.2Rb0.5', '-1.309', '-0.205088078954']
AsORe 4 ['O0.666667As0.111111Re0.222222', '-1.895', '-0.205426988302']
AsOSb 1 ['O0.666667As0.222222Sb0.111111', '-1.726', '-0.212350216544']
AsOSi 4 ['O0.7Si0.1As0.2', '-2.098', '-0.226076613389']
AsOSm 9 ['O0.111111As0.333333Sm0.555556', '-1.905', '-0.200139013837']
AsOSr 1 ['O0.2As0.4Sr0.4', '-1.857', '-0.215207611224']
AsOTa 1 ['O0.666667As0.222222Ta0.111111', '-2.368', '-0.284306271635']
AsOTb 7 ['O0.222222As0.333333Tb0.444444', '-2.241', '-0.204398260234']
AsOTc 4 ['O0.666667As0.166667Tc0.166667', '-1.762', '-0.2051635758']
AsOTe 2 ['O0.666667As0.222222Te0.111111', '-1.569', '-0.206391174323']
AsOTh 3 ['O0.25As0.375Th0.375', '-2.599', '-0.225164657437']
AsOTm 3 ['O0.7As0.2Tm0.1', '-2.153', '-0.220182196556']
AsOU 1 ['O0.7As0.2U0.1', '-2.362', '-0.2597772558']
AsOW 2 ['O0.625As0.25W0.125', '-2.026', '-0.200583129241']
AsOYb 1 ['O0.666667As0.222222Yb0.111111', '-2.119', '-0.257826644971']
AsOZr 1 ['O0.666667As0.222222Zr0.111111', '-2.378', '-0.237620248303']
AsPPm 17 ['P0.5As0.166667Pm0.333333', '-1.001', '-0.206487829513']
AsPRb 4 ['P0.125As0.375Rb0.5', '-0.663', '-0.210996138854']
AsPS 2 ['P0.3S0.5As0.2', '-0.57', '-0.208735787039']
AsPSr 2 ['P0.375As0.25Sr0.375', '-1.17', '-0.208939360453']
AsPTa 8 ['P0.5As0.125Ta0.375', '-0.713', '-0.206788211262']
AsPaS 10 ['S0.555556As0.222222Pa0.222222', '-1.237', '-0.204844494436']

AsPaSe 18 ['As0.333333Se0.555556Pa0.111111', '-0.565', '-0.203882281076']
AsPbPm 1 ['As0.4Pm0.5Pb0.1', '-1.174', '-0.22337528375']
AsPbS 3 ['S0.4As0.1Pb0.5', '-0.75', '-0.202164686394']
AsPdPm 8 ['As0.375Pd0.25Pm0.375', '-1.067', '-0.203927725156']
AsPdS 6 ['S0.5As0.333333Pd0.166667', '-0.579', '-0.201580390827']
AsPdY 4 ['As0.333333Y0.333333Pd0.333333', '-1.362', '-0.225551935417']
AsPmPt 2 ['As0.375Pm0.5Pt0.125', '-1.31', '-0.230987543125']
AsPmPu 4 ['As0.375Pm0.5Pu0.125', '-1.039', '-0.229760332187']
AsPmRh 6 ['As0.3Rh0.3Pm0.4', '-1.041', '-0.214720055167']
AsPmRu 1 ['As0.4Ru0.1Pm0.5', '-1.147', '-0.26182988975']
AsPmS 20 ['S0.222222As0.222222Pm0.555556', '-1.515', '-0.20418427244']
AsPmSb 3 ['As0.3Sb0.1Pm0.6', '-1.035', '-0.202066322']
AsPmSe 48 ['As0.3Se0.1Pm0.6', '-1.172', '-0.2004122255']
AsPmSi 3 ['Si0.1As0.4Pm0.5', '-1.159', '-0.20808309525']
AsPmSn 1 ['As0.444444Sn0.111111Pm0.444444', '-1.162', '-0.202901134444']
AsPmSr 2 ['As0.444444Sr0.111111Pm0.444444', '-1.282', '-0.278686552168']
AsPmTe 3 ['As0.1Te0.5Pm0.4', '-1.265', '-0.218398226997']
AsPmYb 3 ['As0.444444Pm0.444444Yb0.111111', '-1.274', '-0.265169628126']
AsPrSe 3 ['As0.2Se0.4Pr0.4', '-1.835', '-0.226633654498']
AsPtS 12 ['S0.5As0.166667Pt0.333333', '-0.728', '-0.200927057785']
AsPuTe 2 ['As0.1Te0.5Pu0.4', '-1.19', '-0.208153568833']
AsRbS 23 ['S0.1As0.4Rb0.5', '-0.84', '-0.210509812475']
AsRbSe 4 ['As0.333333Se0.222222Rb0.444444', '-0.929', '-0.21002468041']
AsReS 9 ['S0.5As0.333333Re0.166667', '-0.669', '-0.201778130285']
AsReSe 1 ['As0.333333Se0.5Re0.166667', '-0.472', '-0.242872980278']
AsRhS 2 ['S0.4As0.4Rh0.2', '-0.65', '-0.202703911124']
AsRhSe 4 ['As0.444444Se0.444444Rh0.111111', '-0.457', '-0.207340538682']
AsRuYb 4 ['As0.375Ru0.5Yb0.125', '-0.64', '-0.214899750313']
AsSSm 5 ['S0.4As0.2Sm0.4', '-2.147', '-0.216340631395']
AsSTc 10 ['S0.5As0.4Tc0.1', '-0.612', '-0.202759817743']
AsSTm 4 ['S0.375As0.25Tm0.375', '-1.988', '-0.203300893025']
AsSU 3 ['S0.555556As0.111111U0.333333', '-1.664', '-0.21358600981']
AsSXe 1 ['S0.5As0.2Xe0.3', '-0.353', '-0.203672538046']
AsSY 1 ['S0.5As0.1Y0.4', '-2.315', '-0.231441483825']
AsSZr 3 ['S0.375As0.125Zr0.5', '-1.624', '-0.21975689818']
AsScSe 10 ['Sc0.444444As0.333333Se0.222222', '-1.528', '-0.207665461111']
AsSeSm 1 ['As0.4Se0.2Sm0.4', '-1.698', '-0.249743243333']
AsSeSr 9 ['As0.166667Se0.333333Sr0.5', '-1.948', '-0.204811753333']
AsSeTb 4 ['As0.142857Se0.428571Tb0.428571', '-1.705', '-0.202905282284']
AsSeTc 31 ['As0.111111Se0.666667Tc0.222222', '-0.254', '-0.201576436852']
AsSeTm 1 ['As0.1Se0.5Tm0.4', '-1.743', '-0.203715025502']
AsSeY 23 ['As0.3Se0.4Y0.3', '-1.292', '-0.213215132417']
AsSeYb 11 ['As0.3Se0.2Yb0.5', '-1.664', '-0.201717382252']
AsSeZn 1 ['Zn0.333333As0.222222Se0.444444', '-0.71', '-0.209487828796']

AsSrZn 2 ['Zn0.5As0.4Sr0.1', '-0.572', '-0.244054221724']
AuBCl 6 ['B0.166667Cl0.666667Au0.166667', '-1.153', '-0.200853301674']
AuBF 4 ['B0.142857F0.714286Au0.142857', '-2.395', '-0.214237862943']
AuBI 18 ['B0.285714I0.571429Au0.142857', '-0.438', '-0.29630107766']
AuBaF 1 ['F0.8Ba0.1Au0.1', '-2.157', '-0.231944664983']
AuBaI 1 ['I0.5Ba0.125Au0.375', '-0.93', '-0.208249146597']
AuBaO 3 ['O0.625Ba0.125Au0.25', '-1.352', '-0.23136104581']
AuBeF 2 ['Be0.142857F0.714286Au0.142857', '-2.377', '-0.225083015826']
AuBiCe 12 ['Ce0.375Au0.5Bi0.125', '-0.839', '-0.201845175312']
AuBiCl 8 ['Cl0.555556Au0.111111Bi0.333333', '-1.11', '-0.201335369563']
AuBiEu 1 ['Eu0.4Au0.5Bi0.1', '-1.127', '-0.242222157']
AuBiI 4 ['I0.444444Au0.222222Bi0.333333', '-0.592', '-0.204887907995']
AuBiO 1 ['O0.625Au0.25Bi0.125', '-0.898', '-0.229662154945']
AuBiP 18 ['P0.571429Au0.285714Bi0.142857', '-0.229', '-0.204612215']
AuBiPa 4 ['Au0.6Bi0.1Pa0.3', '-0.388', '-0.2029584845']
AuBiPm 13 ['Pm0.25Au0.625Bi0.125', '-0.627', '-0.2105385175']
AuBrCe 25 ['Br0.444444Ce0.444444Au0.111111', '-1.673', '-0.206451818094']
AuBrCr 10 ['Cr0.142857Br0.571429Au0.285714', '-0.732', '-0.209071005762']
AuBrCu 2 ['Cu0.375Br0.5Au0.125', '-0.611', '-0.208791776292']
AuBrDy 41 ['Br0.3Dy0.2Au0.5', '-1.097', '-0.200500166068']
AuBrEr 51 ['Br0.375Er0.125Au0.5', '-0.704', '-0.210256909719']
AuBrEu 15 ['Br0.6Eu0.2Au0.2', '-1.784', '-0.218557764353']
AuBrF 12 ['F0.5Br0.333333Au0.166667', '-1.1', '-0.201465789631']
AuBrFe 8 ['Fe0.166667Br0.666667Au0.166667', '-0.763', '-0.223924345056']
AuBrGa 3 ['Ga0.2Br0.7Au0.1', '-1.121', '-0.211209274806']
AuBrGd 10 ['Br0.5Gd0.2Au0.3', '-1.744', '-0.216680196582']
AuBrGe 1 ['Ge0.222222Br0.666667Au0.111111', '-0.958', '-0.209649325473']
AuBrHf 46 ['Br0.285714Hf0.285714Au0.428571', '-0.622', '-0.211054770024']
AuBrHo 46 ['Br0.285714Ho0.428571Au0.285714', '-1.241', '-0.224225547166']
AuBrLa 14 ['Br0.5La0.3Au0.2', '-1.94', '-0.201489841793']
AuBrLu 54 ['Br0.25Lu0.25Au0.5', '-0.906', '-0.206229193563']
AuBrMo 16 ['Br0.571429Mo0.285714Au0.142857', '-0.966', '-0.205153748858']
AuBrNd 13 ['Br0.6Nd0.2Au0.2', '-1.912', '-0.202357498552']
AuBrNp 8 ['Br0.666667Au0.166667Np0.166667', '-1.365', '-0.214086433597']
AuBrOs 8 ['Br0.666667Os0.166667Au0.166667', '-0.294', '-0.201297709597']
AuBrPa 5 ['Br0.555556Au0.222222Pa0.222222', '-1.432', '-0.213982417132']
AuBrPm 70 ['Br0.222222Pm0.444444Au0.333333', '-1.137', '-0.217568541684']
AuBrPr 20 ['Br0.444444Pr0.222222Au0.333333', '-1.675', '-0.204346990452']
AuBrPt 5 ['Br0.625Pt0.25Au0.125', '-0.353', '-0.283473282198']
AuBrPu 47 ['Br0.2Au0.4Pu0.4', '-0.949', '-0.203865791851']
AuBrRe 9 ['Br0.666667Re0.222222Au0.111111', '-0.297', '-0.235198473065']
AuBrRu 8 ['Br0.7Ru0.1Au0.2', '-0.527', '-0.200231893292']
AuBrSb 5 ['Br0.7Sb0.2Au0.1', '-0.928', '-0.205995298809']
AuBrSc 49 ['Sc0.5Br0.375Au0.125', '-1.085', '-0.200599894719']

AuBrSe 2 ['Se0.222222Br0.666667Au0.111111', '-0.371', '-0.20313016475']
AuBrSi 6 ['Si0.142857Br0.714286Au0.142857', '-1.059', '-0.208547068991']
AuBrSm 25 ['Br0.7Sm0.1Au0.2', '-1.161', '-0.201025521541']
AuBrTa 15 ['Br0.555556Ta0.222222Au0.222222', '-1.066', '-0.228421171807']
AuBrTb 38 ['Br0.375Tb0.375Au0.25', '-1.354', '-0.233353325783']
AuBrTc 3 ['Br0.75Tc0.125Au0.125', '-0.596', '-0.225670776292']
AuBrTe 5 ['Br0.5Te0.25Au0.25', '-0.537', '-0.201961166592']
AuBrTh 14 ['Br0.5Au0.3Th0.2', '-1.609', '-0.202474537169']
AuBrTi 2 ['Ti0.142857Br0.714286Au0.142857', '-1.332', '-0.209679314519']
AuBrTm 32 ['Br0.5Tm0.1Au0.4', '-0.891', '-0.229071609293']
AuBrU 9 ['Br0.5Au0.333333U0.166667', '-1.223', '-0.205421092127']
AuBrV 9 ['V0.111111Br0.777778Au0.111111', '-0.655', '-0.252473523638']
AuBrW 3 ['Br0.666667W0.166667Au0.166667', '-0.801', '-0.204310763737']
AuBrXe 3 ['Br0.555556Xe0.333333Au0.111111', '-0.263', '-0.201198473065']
AuBrY 50 ['Br0.222222Y0.444444Au0.333333', '-1.217', '-0.216888223352']
AuBrYb 21 ['Br0.5Yb0.25Au0.25', '-1.951', '-0.210511673792']
AuBrZr 23 ['Br0.555556Zr0.111111Au0.333333', '-1.052', '-0.20589465977']
AuCLu 9 ['C0.125Lu0.625Au0.25', '-0.724', '-0.208466686473']
AuCNp 10 ['C0.6Au0.1Np0.3', '-0.204', '-0.200389731125']
AuCPa 11 ['C0.5Au0.1Pa0.4', '-0.564', '-0.200112697']
AuCTi 1 ['C0.2Ti0.6Au0.2', '-0.829', '-0.240979725828']
AuCaCl 2 ['Cl0.625Ca0.25Au0.125', '-2.288', '-0.207289778675']
AuCaF 1 ['F0.75Ca0.125Au0.125', '-2.524', '-0.254862371367']
AuCaH 3 ['H0.444444Ca0.444444Au0.111111', '-0.797', '-0.204427016474']
AuCaO 3 ['O0.625Ca0.125Au0.25', '-1.375', '-0.228493992684']
AuCaP 1 ['P0.8Ca0.1Au0.1', '-0.529', '-0.200715390312']
AuCaSn 3 ['Ca0.375Sn0.25Au0.375', '-0.901', '-0.202600913426']
AuCdCe 14 ['Cd0.142857Ce0.285714Au0.571429', '-0.718', '-0.201315843889']
AuCdI 5 ['Cd0.142857I0.428571Au0.428571', '-0.513', '-0.209259173801']
AuCdNd 1 ['Cd0.25Nd0.125Au0.625', '-0.551', '-0.202163633854']
AuCdO 2 ['O0.625Cd0.125Au0.25', '-0.737', '-0.252156757686']
AuCdP 4 ['P0.5Cd0.166667Au0.333333', '-0.4', '-0.209483605103']
AuCdPa 8 ['Cd0.142857Au0.714286Pa0.142857', '-0.366', '-0.205663515']
AuCdPm 17 ['Cd0.111111Pm0.111111Au0.777778', '-0.439', '-0.200895126944']
AuCeCl 4 ['Cl0.666667Ce0.166667Au0.166667', '-2.046', '-0.20007174542']
AuCeH 37 ['H0.6Ce0.3Au0.1', '-0.76', '-0.201106867039']
AuCeHg 4 ['Ce0.2Au0.7Hg0.1', '-0.523', '-0.2107920545']
AuCeI 57 ['I0.1Ce0.3Au0.6', '-0.763', '-0.20592484084']
AuCeLa 5 ['La0.222222Ce0.111111Au0.666667', '-0.808', '-0.201085084167']
AuCeNa 1 ['Na0.111111Ce0.222222Au0.666667', '-0.69', '-0.208042278239']
AuCeO 5 ['O0.666667Ce0.166667Au0.166667', '-1.8', '-0.201528796642']
AuCePa 7 ['Ce0.166667Au0.666667Pa0.166667', '-0.566', '-0.21251000875']
AuCePb 6 ['Ce0.25Au0.625Pb0.125', '-0.622', '-0.20381324875']
AuCePm 10 ['Ce0.1Pm0.3Au0.6', '-0.841', '-0.2107494225']

AuCeSe 1 ['Se0.285714Ce0.285714Au0.428571', '-1.265', '-0.208088200714']
AuCeSn 1 ['Sn0.125Ce0.375Au0.5', '-0.912', '-0.200302973831']
AuCeTl 3 ['Ce0.222222Au0.666667Tl0.111111', '-0.552', '-0.2005702375']
AuClCr 10 ['Cl0.666667Cr0.111111Au0.222222', '-1.077', '-0.219772480978']
AuClCu 5 ['Cl0.571429Cu0.285714Au0.142857', '-0.818', '-0.208390885361']
AuClDy 6 ['Cl0.666667Dy0.166667Au0.166667', '-2.03', '-0.22591194056']
AuClEr 50 ['Cl0.375Er0.5Au0.125', '-1.42', '-0.202071716642']
AuClEu 15 ['Cl0.3Eu0.5Au0.2', '-1.914', '-0.206366948172']
AuClF 15 ['F0.428571Cl0.285714Au0.285714', '-1.111', '-0.201112465465']
AuClFe 13 ['Cl0.75Fe0.125Au0.125', '-0.955', '-0.211522676411']
AuClGa 6 ['Cl0.555556Ga0.333333Au0.111111', '-1.284', '-0.203680306048']
AuClGd 11 ['Cl0.571429Gd0.285714Au0.142857', '-2.338', '-0.207338901312']
AuClGe 10 ['Cl0.6Ge0.2Au0.2', '-1.111', '-0.20635102']
AuClHo 52 ['Cl0.3Ho0.2Au0.5', '-0.944', '-0.209554313147']
AuClI 5 ['Cl0.333333I0.444444Au0.222222', '-0.481', '-0.202914384964']
AuClIn 18 ['Cl0.7In0.1Au0.2', '-0.906', '-0.201653652568']
AuClIr 9 ['Cl0.625Ir0.125Au0.25', '-0.656', '-0.213728110238']
AuClLa 7 ['Cl0.75La0.125Au0.125', '-1.847', '-0.244985498913']
AuClLi 7 ['Li0.2Cl0.5Au0.3', '-1.231', '-0.21214674844']
AuClLu 63 ['Cl0.1Lu0.6Au0.3', '-0.991', '-0.201625775438']
AuClMg 1 ['Mg0.25Cl0.625Au0.125', '-1.888', '-0.219985693885']
AuClMn 4 ['Cl0.5Mn0.166667Au0.333333', '-0.992', '-0.20802971114']
AuClMo 7 ['Cl0.666667Mo0.222222Au0.111111', '-1.209', '-0.205368712307']
AuClNb 3 ['Cl0.75Nb0.125Au0.125', '-1.46', '-0.22560863756']
AuClNd 32 ['Cl0.5Nd0.2Au0.3', '-1.98', '-0.203130996027']
AuClNi 3 ['Cl0.625Ni0.125Au0.25', '-0.802', '-0.215149631801']
AuClNp 29 ['Cl0.444444Au0.222222Np0.333333', '-1.394', '-0.204149664217']
AuClOs 1 ['Cl0.7Os0.1Au0.2', '-0.791', '-0.239024176317']
AuClPa 11 ['Cl0.75Au0.125Pa0.125', '-1.912', '-0.21992133172']
AuClPb 1 ['Cl0.666667Au0.222222Pb0.111111', '-0.997', '-0.208196451811']
AuClPm 43 ['Cl0.4Pm0.2Au0.4', '-1.436', '-0.203051103919']
AuClPr 8 ['Cl0.7Pr0.2Au0.1', '-2.348', '-0.213757091316']
AuClPt 4 ['Cl0.777778Pt0.111111Au0.111111', '-0.638', '-0.205508828915']
AuClPu 43 ['Cl0.5Au0.4Pu0.1', '-1.229', '-0.212889616192']
AuClRe 2 ['Cl0.666667Re0.111111Au0.222222', '-0.873', '-0.211762323013']
AuClRh 11 ['Cl0.6Rh0.2Au0.2', '-0.634', '-0.218252203629']
AuClRu 9 ['Cl0.571429Ru0.142857Au0.285714', '-0.764', '-0.201856417861']
AuClSb 2 ['Cl0.7Sb0.1Au0.2', '-0.988', '-0.242008294817']
AuClSc 5 ['Cl0.625Sc0.125Au0.25', '-1.608', '-0.23182512774']
AuClSe 1 ['Cl0.666667Se0.111111Au0.222222', '-0.752', '-0.237699549349']
AuClSi 8 ['Si0.142857Cl0.714286Au0.142857', '-1.4', '-0.205003597769']
AuClSm 25 ['Cl0.444444Sm0.333333Au0.222222', '-1.856', '-0.221302647504']
AuClSr 6 ['Cl0.666667Sr0.166667Au0.166667', '-1.817', '-0.206668808755']
AuClTa 9 ['Cl0.666667Ta0.111111Au0.222222', '-1.283', '-0.241512536807']

AuClTb 5 ['Cl0.625Tb0.125Au0.25', '-1.591', '-0.220733962155']
AuClTc 5 ['Cl0.666667Tc0.166667Au0.166667', '-1.008', '-0.214970944796']
AuClTe 20 ['Cl0.428571Te0.428571Au0.142857', '-0.724', '-0.206143365152']
AuClTh 5 ['Cl0.75Au0.125Th0.125', '-1.93', '-0.216951177033']
AuClTi 2 ['Cl0.7Ti0.1Au0.2', '-1.359', '-0.218454223189']
AuClU 4 ['Cl0.714286Au0.142857U0.142857', '-1.84', '-0.203572863126']
AuClV 2 ['Cl0.7V0.1Au0.2', '-1.121', '-0.201011868314']
AuClXe 9 ['Cl0.7Xe0.1Au0.2', '-0.622', '-0.206252203629']
AuClY 28 ['Cl0.5Y0.25Au0.25', '-2.143', '-0.202960239277']
AuClYb 1 ['Cl0.5Yb0.3Au0.2', '-2.454', '-0.268476952943']
AuClZr 8 ['Cl0.666667Zr0.222222Au0.111111', '-2.03', '-0.216101330972']
AuCrN 1 ['N0.333333Cr0.555556Au0.111111', '-0.632', '-0.204060484145']
AuCsF 5 ['F0.666667Cs0.222222Au0.111111', '-2.256', '-0.287080752473']
AuCsO 7 ['O0.666667Cs0.222222Au0.111111', '-1.021', '-0.212313879277']
AuCsSe 4 ['Se0.3Cs0.5Au0.2', '-1.037', '-0.200387389']
AuCuEu 2 ['Cu0.7Eu0.1Au0.2', '-0.428', '-0.201379365']
AuCuLa 1 ['Cu0.7La0.1Au0.2', '-0.396', '-0.2110811345']
AuCuO 3 ['O0.222222Cu0.666667Au0.111111', '-0.681', '-0.209691975722']
AuCuPm 1 ['Cu0.1Pm0.2Au0.7', '-0.533', '-0.2063478925']
AuCuPr 1 ['Cu0.7Pr0.1Au0.2', '-0.434', '-0.20508758975']
AuCuTh 1 ['Cu0.7Au0.2Th0.1', '-0.428', '-0.210869157']
AuDyF 1 ['F0.75Dy0.125Au0.125', '-2.854', '-0.211330681875']
AuDyI 49 ['I0.4Dy0.4Au0.2', '-1.131', '-0.203884374695']
AuDyP 10 ['P0.4Dy0.3Au0.3', '-1.274', '-0.204941181418']
AuErH 1 ['H0.555556Er0.222222Au0.222222', '-0.798', '-0.223362714955']
AuErI 41 ['I0.5Er0.4Au0.1', '-1.175', '-0.231992966474']
AuErP 3 ['P0.555556Er0.111111Au0.333333', '-0.602', '-0.21577299456']
AuErSe 2 ['Se0.3Er0.3Au0.4', '-1.45', '-0.207350213']
AuEuI 4 ['I0.5Eu0.3Au0.2', '-1.743', '-0.200003449783']
AuEuLa 1 ['La0.2Eu0.2Au0.6', '-1.005', '-0.22248444']
AuEuN 10 ['N0.2Eu0.5Au0.3', '-1.026', '-0.200070223931']
AuEuO 5 ['O0.625Eu0.25Au0.125', '-2.072', '-0.230933570809']
AuEuP 2 ['P0.333333Eu0.555556Au0.111111', '-1.219', '-0.222637714828']
AuEuSe 7 ['Se0.333333Eu0.555556Au0.111111', '-1.935', '-0.217512127852']
AuEuY 1 ['Y0.1Eu0.4Au0.5', '-1.181', '-0.211054504']
AuFFe 2 ['F0.777778Fe0.111111Au0.111111', '-1.915', '-0.257277812422']
AuFGd 3 ['F0.714286Gd0.142857Au0.142857', '-3.058', '-0.213302030623']
AuFH 1 ['H0.1F0.8Au0.1', '-1.199', '-0.267242401304']
AuFI 1 ['F0.777778I0.111111Au0.111111', '-1.658', '-0.209994847814']
AuFIn 1 ['F0.777778In0.111111Au0.111111', '-2.029', '-0.200219644089']
AuFK 2 ['F0.571429K0.285714Au0.142857', '-2.469', '-0.20961749716']
AuFLu 4 ['F0.8Lu0.1Au0.1', '-2.455', '-0.229617473696']
AuFN 1 ['N0.1F0.8Au0.1', '-0.839', '-0.232111238936']
AuFNa 6 ['F0.666667Na0.222222Au0.111111', '-2.19', '-0.205612745113']

AuFNp 3 ['F0.75Au0.125Np0.125', '-3.004', '-0.234715567787']
AuFO 2 ['O0.142857F0.571429Au0.285714', '-1.2', '-0.209318271475']
AuFPa 9 ['F0.625Au0.25Pa0.125', '-2.437', '-0.22686391898']
AuFPb 1 ['F0.8Au0.1Pb0.1', '-1.799', '-0.20064783727']
AuFPu 8 ['F0.666667Au0.166667Pu0.166667', '-3.121', '-0.244757246204']
AuFRb 6 ['F0.714286Rb0.142857Au0.142857', '-1.972', '-0.250203578354']
AuFRh 1 ['F0.777778Rh0.111111Au0.111111', '-1.627', '-0.235182911311']
AuFRu 2 ['F0.777778Ru0.111111Au0.111111', '-1.857', '-0.28330995806']
AuFSb 2 ['F0.777778Sb0.111111Au0.111111', '-2.142', '-0.279413448294']
AuFTc 2 ['F0.777778Tc0.111111Au0.111111', '-1.957', '-0.20118112431']
AuFTi 5 ['F0.7Ti0.1Au0.2', '-2.353', '-0.251332145733']
AuFTl 6 ['F0.777778Au0.111111Tl0.111111', '-1.709', '-0.201730362978']
AuFTm 41 ['F0.444444Tm0.111111Au0.444444', '-1.261', '-0.205729117974']
AuFV 1 ['F0.75V0.125Au0.125', '-2.505', '-0.220114422218']
AuFY 1 ['F0.8Y0.1Au0.1', '-2.531', '-0.244865397028']
AuFYb 32 ['F0.666667Yb0.111111Au0.222222', '-2.171', '-0.204617959052']
AuGaI 1 ['Ga0.333333I0.555556Au0.111111', '-0.732', '-0.209857163836']
AuGaP 6 ['P0.5Ga0.25Au0.25', '-0.475', '-0.20791552151']
AuGaPa 2 ['Ga0.1Au0.6Pa0.3', '-0.427', '-0.21239943925']
AuGaPm 6 ['Ga0.222222Pm0.333333Au0.444444', '-0.808', '-0.203516138056']
AuGdH 2 ['H0.5Gd0.2Au0.3', '-0.724', '-0.200047844178']
AuGdI 49 ['I0.3Gd0.1Au0.6', '-0.506', '-0.210246059625']
AuGdP 1 ['P0.5Gd0.125Au0.375', '-0.739', '-0.209521371301']
AuGdSe 1 ['Se0.3Gd0.3Au0.4', '-1.437', '-0.202499982792']
AuGeP 7 ['P0.444444Ge0.222222Au0.333333', '-0.32', '-0.223483014536']
AuGePa 2 ['Ge0.1Au0.6Pa0.3', '-0.377', '-0.2011710765']
AuGePm 14 ['Ge0.285714Pm0.285714Au0.428571', '-0.66', '-0.204370855']
AuGeSe 9 ['Ge0.3Se0.3Au0.4', '-0.334', '-0.20026804525']
AuGeTh 2 ['Ge0.375Au0.5Th0.125', '-0.479', '-0.20007542875']
AuHI 20 ['H0.555556I0.222222Au0.222222', '-0.231', '-0.201385724531']
AuHK 10 ['H0.285714K0.285714Au0.428571', '-0.455', '-0.206464998571']
AuHLu 8 ['H0.4Lu0.3Au0.3', '-0.932', '-0.224748851845']
AuHNd 3 ['H0.555556Nd0.222222Au0.222222', '-0.693', '-0.2124685407']
AuHNp 29 ['H0.571429Au0.142857Np0.285714', '-0.372', '-0.208563941014']
AuHPm 42 ['H0.7Pm0.2Au0.1', '-0.509', '-0.202241602845']
AuHPu 18 ['H0.333333Au0.5Pu0.166667', '-0.407', '-0.20024340705']
AuHSe 1 ['H0.375Se0.25Au0.375', '-0.29', '-0.2455221575']
AuHSm 5 ['H0.571429Sm0.142857Au0.285714', '-0.578', '-0.20435935568']
AuHY 9 ['H0.5Y0.25Au0.25', '-0.841', '-0.201812332103']
AuHfI 1 ['I0.7Hf0.2Au0.1', '-1.171', '-0.203637533615']
AuHfO 1 ['O0.6Hf0.2Au0.2', '-2.607', '-0.209751089804']
AuHfS 2 ['S0.428571Hf0.428571Au0.142857', '-1.541', '-0.213441826851']
AuHfSe 2 ['Se0.5Hf0.125Au0.375', '-0.752', '-0.202639150625']
AuHgO 2 ['O0.6Au0.3Hg0.1', '-0.503', '-0.201158696687']

AuHgP 6 ['P0.5Au0.3Hg0.2', '-0.201', '-0.201']
AuHgPa 4 ['Au0.6Hg0.1Pa0.3', '-0.369', '-0.201029934']
AuHgPm 5 ['Pm0.166667Au0.666667Hg0.166667', '-0.469', '-0.205585219583']
AuHgPu 1 ['Au0.5Hg0.166667Pu0.333333', '-0.478', '-0.20609036']
AuHoI 42 ['I0.285714Ho0.142857Au0.571429', '-0.632', '-0.205313940952']
AuHoP 1 ['P0.444444Ho0.222222Au0.333333', '-1.033', '-0.206200615554']
AuHoSe 1 ['Se0.4Ho0.2Au0.4', '-1.172', '-0.209385620835']
AuILa 4 ['I0.7La0.2Au0.1', '-1.549', '-0.209215669801']
AuILu 60 ['I0.111111Lu0.555556Au0.333333', '-0.925', '-0.200196477508']
AuIMg 5 ['Mg0.222222I0.555556Au0.222222', '-1.005', '-0.219643156057']
AuIMn 4 ['Mn0.142857I0.571429Au0.285714', '-0.448', '-0.200185573785']
AuINd 24 ['I0.375Nd0.125Au0.5', '-0.75', '-0.214692907838']
AuINi 4 ['Ni0.333333I0.555556Au0.111111', '-0.386', '-0.203748451428']
AuINp 30 ['I0.333333Au0.333333Np0.333333', '-0.658', '-0.202040396273']
AuIPa 58 ['I0.142857Au0.571429Pa0.285714', '-0.372', '-0.208983621796']
AuIPm 74 ['I0.111111Pm0.444444Au0.444444', '-0.928', '-0.202365217508']
AuIPr 11 ['I0.555556Pr0.111111Au0.333333', '-0.813', '-0.210768852818']
AuIPu 5 ['I0.666667Au0.222222Pu0.111111', '-0.744', '-0.200036056715']
AuIRe 1 ['I0.7Re0.2Au0.1', '-0.359', '-0.206054072133']
AuIRh 5 ['Rh0.2I0.7Au0.1', '-0.212', '-0.208802868886']
AuISc 17 ['Sc0.3I0.6Au0.1', '-1.317', '-0.208915506039']
AuISe 19 ['Se0.3I0.5Au0.2', '-0.237', '-0.201417726']
AuISi 3 ['Si0.125I0.625Au0.25', '-0.531', '-0.208939630982']
AuISm 33 ['I0.75Sm0.125Au0.125', '-0.7', '-0.218676809259']
AuITa 5 ['I0.6Ta0.1Au0.3', '-0.5', '-0.200990223293']
AuITb 57 ['I0.285714Tb0.428571Au0.285714', '-0.72', '-0.204007053021']
AuITe 2 ['Te0.142857I0.428571Au0.428571', '-0.34', '-0.201605565499']
AuITh 3 ['I0.666667Au0.166667Th0.166667', '-1.354', '-0.209652667133']
AuITm 26 ['I0.6Tm0.3Au0.1', '-1.334', '-0.207190281539']
AuIU 5 ['I0.714286Au0.142857U0.142857', '-0.901', '-0.205766746837']
AuIV 3 ['V0.111111I0.555556Au0.333333', '-0.407', '-0.210540041429']
AuIW 9 ['I0.571429W0.142857Au0.285714', '-0.21', '-0.200865339674']
AuIXe 27 ['I0.3Xe0.6Au0.1', '-0.204', '-0.200802868886']
AuIYb 7 ['I0.6Yb0.3Au0.1', '-1.804', '-0.203492432542']
AuIZr 7 ['Zr0.125I0.625Au0.25', '-0.767', '-0.217119887543']
AuInNa 1 ['Na0.142857In0.571429Au0.285714', '-0.548', '-0.251317109164']
AuInP 17 ['P0.6In0.1Au0.3', '-0.307', '-0.207685034312']
AuInPa 11 ['In0.142857Au0.571429Pa0.285714', '-0.438', '-0.211431759524']
AuInPm 15 ['In0.25Pm0.375Au0.375', '-0.807', '-0.200049287396']
AuInS 1 ['S0.3In0.3Au0.4', '-0.636', '-0.219537765379']
AuInTh 10 ['In0.333333Au0.5Th0.166667', '-0.686', '-0.201221841667']
AuKO 14 ['O0.4K0.3Au0.3', '-1.126', '-0.221927931815']
AuKP 6 ['P0.3K0.3Au0.4', '-0.625', '-0.201246577265']
AuKrP 6 ['P0.444444Kr0.222222Au0.333333', '-0.2', '-0.2']

AuLaO 1 ['O0.625La0.125Au0.25', '-1.734', '-0.304103128567']
AuLaP 2 ['P0.444444La0.222222Au0.333333', '-1.044', '-0.201993304073']
AuLaPa 1 ['La0.1Au0.8Pa0.1', '-0.463', '-0.201809392']
AuLaPm 4 ['La0.125Pm0.25Au0.625', '-0.856', '-0.204377096875']
AuLaSe 2 ['Se0.4La0.4Au0.2', '-1.833', '-0.219136808']
AuLuN 2 ['N0.3Lu0.6Au0.1', '-1.622', '-0.251465280398']
AuLuSe 1 ['Se0.222222Lu0.555556Au0.222222', '-1.312', '-0.206067217778']
AuMgP 1 ['Mg0.428571P0.285714Au0.285714', '-0.829', '-0.217286169726']
AuMnO 3 ['O0.6Mn0.2Au0.2', '-1.363', '-0.213713014281']
AuMoO 2 ['O0.666667Mo0.166667Au0.166667', '-1.769', '-0.248882805111']
AuNNp 4 ['N0.5Au0.166667Np0.333333', '-1.021', '-0.212695191967']
AuNPa 16 ['N0.375Au0.25Pa0.375', '-0.98', '-0.239041856788']
AuNPu 1 ['N0.555556Au0.111111Pu0.333333', '-1.166', '-0.25325198822']
AuNTi 1 ['N0.444444Ti0.444444Au0.111111', '-1.388', '-0.235078380437']
AuNaNp 9 ['Na0.125Au0.625Np0.25', '-0.363', '-0.202031822706']
AuNaO 6 ['O0.444444Na0.444444Au0.111111', '-1.451', '-0.211033720757']
AuNaPa 6 ['Na0.111111Au0.666667Pa0.222222', '-0.472', '-0.21251772435']
AuNaPm 5 ['Na0.111111Pm0.111111Au0.777778', '-0.512', '-0.20133014185']
AuNaPu 1 ['Na0.1Au0.6Pu0.3', '-0.569', '-0.202727319915']
AuNbO 4 ['O0.6Nb0.1Au0.3', '-1.363', '-0.206493633305']
AuNdO 2 ['O0.625Nd0.125Au0.25', '-1.685', '-0.331872503489']
AuNdPa 5 ['Nd0.1Au0.7Pa0.2', '-0.512', '-0.2011068875']
AuNdPm 2 ['Nd0.1Pm0.2Au0.7', '-0.724', '-0.208989195']
AuNdSe 17 ['Se0.428571Nd0.285714Au0.285714', '-1.485', '-0.204650302143']
AuNpO 1 ['O0.375Au0.125Np0.5', '-2.394', '-0.213977999644']
AuNpP 18 ['P0.8Au0.1Np0.1', '-0.418', '-0.207824077437']
AuNpPm 2 ['Pm0.111111Au0.777778Np0.111111', '-0.384', '-0.204378736528']
AuNpS 1 ['S0.5Au0.333333Np0.166667', '-1.02', '-0.218057097453']
AuNpSe 22 ['Se0.375Au0.375Np0.25', '-0.908', '-0.21393141073']
AuOOs 1 ['O0.625Os0.125Au0.25', '-1.138', '-0.288477989661']
AuOPa 8 ['O0.625Au0.125Pa0.25', '-2.815', '-0.344547435699']
AuOPr 2 ['O0.6Pr0.1Au0.3', '-1.339', '-0.231453795471']
AuOPu 2 ['O0.666667Au0.166667Pu0.166667', '-1.968', '-0.220382199497']
AuORb 1 ['O0.444444Rb0.333333Au0.222222', '-1.348', '-0.314380042943']
AuORe 6 ['O0.666667Re0.111111Au0.222222', '-1.219', '-0.217947619414']
AuORu 2 ['O0.625Ru0.125Au0.25', '-0.935', '-0.284312730076']
AuOSb 2 ['O0.6Sb0.2Au0.2', '-1.369', '-0.237049130137']
AuOSc 1 ['O0.625Sc0.125Au0.25', '-1.627', '-0.26139160263']
AuOSi 2 ['O0.666667Si0.166667Au0.166667', '-1.858', '-0.204022620322']
AuOTa 5 ['O0.714286Ta0.142857Au0.142857', '-1.87', '-0.204426100925']
AuOTc 2 ['O0.666667Tc0.166667Au0.166667', '-1.472', '-0.270510567468']
AuOTe 1 ['O0.666667Te0.222222Au0.111111', '-1.224', '-0.232584069411']
AuOTi 3 ['O0.6Ti0.2Au0.2', '-2.244', '-0.204655940048']
AuOU 4 ['O0.625Au0.25U0.125', '-1.906', '-0.223274537832']

AuOV 2 ['O0.625V0.125Au0.25', '-1.338', '-0.201251970251']
AuOW 2 ['O0.666667W0.111111Au0.222222', '-1.592', '-0.348724678858']
AuOY 1 ['O0.625Y0.125Au0.25', '-1.595', '-0.229140153255']
AuOYb 1 ['O0.625Yb0.125Au0.25', '-1.275', '-0.203480550442']
AuOsSc 3 ['Sc0.625Os0.25Au0.125', '-0.564', '-0.202800901875']
AuPPa 1 ['P0.8Au0.1Pa0.1', '-0.579', '-0.216987509709']
AuPPb 1 ['P0.555556Au0.333333Pb0.111111', '-0.368', '-0.235776823564']
AuPPm 50 ['P0.222222Pm0.222222Au0.555556', '-0.731', '-0.201325219676']
AuPPu 22 ['P0.666667Au0.166667Pu0.166667', '-0.632', '-0.201968383507']
AuPS 8 ['P0.2S0.4Au0.4', '-0.472', '-0.201102608659']
AuPSc 2 ['P0.5Sc0.1Au0.4', '-0.53', '-0.206185213604']
AuPSm 1 ['P0.4Sm0.4Au0.2', '-1.578', '-0.218078501416']
AuPSr 1 ['P0.375Sr0.5Au0.125', '-1.27', '-0.204408654762']
AuPTa 22 ['P0.333333Ta0.5Au0.166667', '-0.838', '-0.204078859097']
AuPTb 8 ['P0.333333Tb0.222222Au0.444444', '-0.827', '-0.200619041418']
AuPTe 19 ['P0.444444Te0.333333Au0.222222', '-0.249', '-0.207394233681']
AuPTi 5 ['P0.5Ti0.166667Au0.333333', '-0.701', '-0.201372688883']
AuPTm 1 ['P0.4Tm0.3Au0.3', '-1.271', '-0.210006434791']
AuPU 1 ['P0.8Au0.1U0.1', '-0.527', '-0.236273879458']
AuPW 3 ['P0.5W0.125Au0.375', '-0.408', '-0.20280681026']
AuPXe 27 ['P0.5Xe0.125Au0.375', '-0.2', '-0.2']
AuPY 5 ['P0.5Y0.125Au0.375', '-0.736', '-0.204712165676']
AuPYb 30 ['P0.5Yb0.166667Au0.333333', '-0.757', '-0.20732767802']
AuPaPm 9 ['Pm0.166667Au0.666667Pa0.166667', '-0.584', '-0.22726851625']
AuPaS 14 ['S0.333333Au0.555556Pa0.111111', '-0.741', '-0.200789237551']
AuPaSe 24 ['Se0.5Au0.125Pa0.375', '-1.128', '-0.2027420625']
AuPaSi 1 ['Si0.375Au0.5Pa0.125', '-0.279', '-0.201965375625']
AuPaSn 6 ['Sn0.111111Au0.666667Pa0.222222', '-0.376', '-0.200552702016']
AuPaTh 1 ['Au0.8Th0.1Pa0.1', '-0.501', '-0.227879135']
AuPaTl 1 ['Au0.7Tl0.1Pa0.2', '-0.319', '-0.20294111525']
AuPaZn 1 ['Zn0.1Au0.7Pa0.2', '-0.379', '-0.2074338995']
AuPbPm 5 ['Pm0.4Au0.5Pb0.1', '-0.849', '-0.200623141833']
AuPbS 10 ['S0.333333Au0.166667Pb0.5', '-0.694', '-0.210501596718']
AuPdPm 51 ['Pd0.1Pm0.4Au0.5', '-0.848', '-0.2013983825']
AuPmPu 4 ['Pm0.111111Au0.666667Pu0.222222', '-0.557', '-0.200117053056']
AuPmS 8 ['S0.375Pm0.375Au0.25', '-1.794', '-0.220924886306']
AuPmSb 8 ['Sb0.1Pm0.4Au0.5', '-0.864', '-0.2043114515']
AuPmSe 54 ['Se0.571429Pm0.142857Au0.285714', '-0.719', '-0.205030979643']
AuPmSn 17 ['Sn0.222222Pm0.444444Au0.333333', '-0.883', '-0.203864457922']
AuPmTe 1 ['Te0.5Pm0.4Au0.1', '-1.325', '-0.263177655269']
AuPmTh 1 ['Pm0.142857Au0.714286Th0.142857', '-0.745', '-0.209028983928']
AuPmTl 5 ['Pm0.375Au0.5Tl0.125', '-0.801', '-0.203218193125']
AuPrSe 4 ['Se0.5Pr0.375Au0.125', '-1.841', '-0.206040607185']
AuPtTh 15 ['Pt0.666667Au0.166667Th0.166667', '-0.623', '-0.200499721667']

AuPuS 8 ['S0.444444Au0.111111Pu0.444444', '-1.911', '-0.205420135991']
AuPuSe 15 ['Se0.3Au0.4Pu0.3', '-1.131', '-0.203419395']
AuPuTe 1 ['Te0.5Au0.1Pu0.4', '-1.253', '-0.312974736331']
AuSW 1 ['S0.6W0.2Au0.2', '-0.894', '-0.239513530491']
AuSXe 4 ['S0.428571Xe0.428571Au0.142857', '-0.241', '-0.204132605785']
AuSZr 8 ['S0.444444Zr0.333333Au0.222222', '-1.554', '-0.227248945991']
AuSbU 9 ['Sb0.375Au0.375U0.25', '-0.464', '-0.200667834063']
AuScSe 29 ['Sc0.142857Se0.428571Au0.428571', '-0.708', '-0.201736250714']
AuSeSm 1 ['Se0.375Sm0.375Au0.25', '-1.722', '-0.244363065938']
AuSeTc 5 ['Se0.5Tc0.333333Au0.166667', '-0.26', '-0.230348105']
AuSeTh 3 ['Se0.25Au0.5Th0.25', '-1.128', '-0.204314106249']
AuSeTm 15 ['Se0.444444Tm0.333333Au0.222222', '-1.509', '-0.204938415558']
AuSeU 4 ['Se0.2Au0.5U0.3', '-0.742', '-0.2092997175']
AuSeY 42 ['Se0.4Y0.1Au0.5', '-0.608', '-0.2014463435']
BBaBr 2 ['B0.1Br0.7Ba0.2', '-1.966', '-0.202268586809']
BBaC 1 ['B0.142857C0.571429Ba0.285714', '-0.286', '-0.217792351905']
BBaCl 13 ['B0.1Cl0.6Ba0.3', '-2.748', '-0.223724123625']
BBaF 7 ['B0.125F0.625Ba0.25', '-3.855', '-0.21348758013']
BBaH 9 ['H0.5B0.375Ba0.125', '-0.476', '-0.204949160196']
BBaI 11 ['B0.125I0.625Ba0.25', '-1.691', '-0.227158757646']
BBaIr 2 ['B0.428571Ba0.142857Ir0.428571', '-0.591', '-0.218246006667']
BBaN 7 ['B0.375N0.5Ba0.125', '-1.472', '-0.241778521158']
BBaNi 1 ['B0.444444Ni0.444444Ba0.111111', '-0.532', '-0.206236304445']
BBaP 2 ['B0.3P0.5Ba0.2', '-1.012', '-0.236479604386']
BBaPa 1 ['B0.8Ba0.1Pa0.1', '-0.516', '-0.229527878']
BBaRh 2 ['B0.375Rh0.5Ba0.125', '-0.537', '-0.223189144375']
BBeCl 1 ['Be0.166667B0.166667Cl0.666667', '-1.61', '-0.223708961256']
BBeF 9 ['Be0.166667B0.166667F0.666667', '-3.282', '-0.212480003774']
BBeI 11 ['Be0.2B0.2I0.6', '-0.669', '-0.20530357821']
BBeP 12 ['Be0.375B0.25P0.375', '-0.576', '-0.203897643421']
BBiCl 3 ['B0.111111Cl0.666667Bi0.222222', '-1.291', '-0.2013039132']
BBiF 4 ['B0.2F0.7Bi0.1', '-2.988', '-0.228448484359']
BBiI 9 ['B0.1I0.7Bi0.2', '-0.744', '-0.232699204134']
BBrCa 3 ['B0.125Ca0.25Br0.625', '-2.039', '-0.212967047243']
BBrCe 7 ['B0.166667Br0.666667Ce0.166667', '-1.82', '-0.210122839637']
BBrCl 14 ['B0.166667Cl0.5Br0.333333', '-1.04', '-0.209472979693']
BBrCo 5 ['B0.125Co0.25Br0.625', '-0.752', '-0.23275330474']
BBrCr 4 ['B0.142857Cr0.142857Br0.714286', '-1.02', '-0.221286096846']
BBrCu 6 ['B0.166667Cu0.166667Br0.666667', '-0.885', '-0.237533807556']
BBrDy 8 ['B0.2Br0.7Dy0.1', '-1.225', '-0.216836115051']
BBrEr 27 ['B0.285714Br0.571429Er0.142857', '-1.003', '-0.205799332712']
BBrEu 10 ['B0.2Br0.7Eu0.1', '-1.442', '-0.20774661521']
BBrF 5 ['B0.142857F0.714286Br0.142857', '-2.399', '-0.227989185928']
BBrGd 2 ['B0.111111Br0.777778Gd0.111111', '-1.617', '-0.32640746811']

BBrHf 14 ['B0.2Br0.7Hf0.1', '-0.892', '-0.219663471309']
BBrHo 22 ['B0.142857Br0.428571Ho0.428571', '-1.22', '-0.211656329771']
BBrI 13 ['B0.25Br0.25I0.5', '-0.582', '-0.204436622349']
BBrIr 9 ['B0.2Br0.7Ir0.1', '-0.881', '-0.203240319309']
BBrK 3 ['B0.142857K0.142857Br0.714286', '-1.191', '-0.205424361476']
BBrLa 10 ['B0.2Br0.4La0.4', '-1.581', '-0.207556655867']
BBrLi 11 ['Li0.285714B0.142857Br0.571429', '-1.469', '-0.203607620047']
BBrLu 28 ['B0.142857Br0.428571Lu0.428571', '-1.171', '-0.268300538046']
BBrMo 2 ['B0.111111Br0.777778Mo0.111111', '-0.956', '-0.263702112834']
BBrNb 1 ['B0.1Br0.8Nb0.1', '-1.197', '-0.224700567565']
BBrNd 4 ['B0.1Br0.8Nd0.1', '-1.385', '-0.225886564551']
BBrNi 17 ['B0.1Ni0.1Br0.8', '-0.688', '-0.208856832792']
BBrNp 2 ['B0.111111Br0.777778Np0.111111', '-1.48', '-0.436423388528']
BBrOs 3 ['B0.125Br0.75Os0.125', '-0.649', '-0.268634769094']
BBrPa 1 ['B0.6Br0.1Pa0.3', '-0.487', '-0.276155338384']
BBrPm 29 ['B0.3Br0.5Pm0.2', '-1.019', '-0.203317239291']
BBrPr 5 ['B0.2Br0.7Pr0.1', '-1.492', '-0.227827178058']
BBrPt 10 ['B0.2Br0.7Pt0.1', '-0.877', '-0.26841563055']
BBrPu 3 ['B0.125Br0.75Pu0.125', '-1.584', '-0.316338703814']
BBrRb 1 ['B0.142857Br0.714286Rb0.142857', '-1.242', '-0.256558623619']
BBrRe 3 ['B0.125Br0.75Re0.125', '-0.737', '-0.356634769094']
BBrRh 15 ['B0.1Br0.6Rh0.3', '-0.828', '-0.21496945405']
BBrRu 8 ['B0.1Br0.7Ru0.2', '-0.803', '-0.211340670975']
BBrSb 2 ['B0.111111Br0.777778Sb0.111111', '-0.913', '-0.204684613112']
BBrSc 24 ['B0.1Sc0.5Br0.4', '-1.062', '-0.207824803284']
BBrSm 6 ['B0.1Br0.7Sm0.2', '-2.007', '-0.208105811806']
BBrSr 3 ['B0.1Br0.7Sr0.2', '-1.931', '-0.209458016307']
BBrTa 2 ['B0.111111Br0.777778Ta0.111111', '-1.093', '-0.346408412921']
BBrTb 13 ['B0.111111Br0.555556Tb0.333333', '-1.492', '-0.217532432366']
BBrTc 3 ['B0.125Br0.75Tc0.125', '-0.899', '-0.217832263188']
BBrTe 3 ['B0.125Br0.75Te0.125', '-0.66', '-0.22903541962']
BBrTh 2 ['B0.111111Br0.777778Th0.111111', '-1.696', '-0.252406612011']
BBrTm 18 ['B0.2Br0.7Tm0.1', '-1.174', '-0.211790103443']
BBrV 4 ['B0.142857V0.142857Br0.714286', '-1.076', '-0.203221943987']
BBrW 1 ['B0.125Br0.75W0.125', '-0.961', '-0.227596913579']
BBrY 25 ['B0.3Br0.4Y0.3', '-1.195', '-0.215953447596']
BBrYb 7 ['B0.166667Br0.666667Yb0.166667', '-1.702', '-0.203572021723']
BBrZr 3 ['B0.125Br0.75Zr0.125', '-1.402', '-0.208819696939']
BCCa 6 ['B0.2C0.5Ca0.3', '-0.398', '-0.225906379']
BCCe 6 ['B0.142857C0.571429Ce0.285714', '-0.429', '-0.231543193929']
BCEr 2 ['B0.625C0.125Er0.25', '-0.796', '-0.219489607969']
BCEu 4 ['B0.222222C0.555556Eu0.222222', '-0.441', '-0.202363761018']
BCGd 1 ['B0.6C0.2Gd0.2', '-0.671', '-0.229734234143']
BCH 6 ['H0.666667B0.166667C0.166667', '-0.246', '-0.214975559152']

BCI 9 ['B0.3C0.1I0.6', '-0.36', '-0.202747757351']
BCIr 1 ['B0.4C0.3Ir0.3', '-0.261', '-0.200431886667']
BCLa 1 ['B0.666667C0.111111La0.222222', '-0.723', '-0.203678422236']
BCLi 2 ['Li0.444444B0.111111C0.444444', '-0.362', '-0.234516829167']
BCLu 1 ['B0.125C0.5Lu0.375', '-0.654', '-0.215023552813']
BCMg 3 ['B0.8C0.1Mg0.1', '-0.367', '-0.201747017209']
BCMo 1 ['B0.428571C0.142857Mo0.428571', '-0.741', '-0.312023161071']
BCNd 1 ['B0.6C0.2Nd0.2', '-0.691', '-0.201863365']
BCNp 18 ['B0.4C0.4Np0.2', '-0.449', '-0.20690780025']
BCOs 4 ['B0.571429C0.142857Os0.285714', '-0.388', '-0.2053456']
BCP 1 ['B0.4C0.1P0.5', '-0.634', '-0.205900764416']
BCPa 40 ['B0.555556C0.333333Pa0.111111', '-0.37', '-0.221873670321']
BCPm 26 ['B0.222222C0.555556Pm0.222222', '-0.223', '-0.204181390684']
BCPr 4 ['B0.1C0.5Pr0.4', '-0.414', '-0.214747852479']
BCPt 1 ['B0.4C0.4Pt0.2', '-0.304', '-0.202868945423']
BCPu 18 ['B0.285714C0.428571Pu0.285714', '-0.399', '-0.204686993571']
BCRe 3 ['B0.111111C0.333333Re0.555556', '-0.3', '-0.202606859167']
BCRh 14 ['B0.222222C0.333333Rh0.444444', '-0.385', '-0.200061837778']
BCSc 3 ['B0.6C0.2Sc0.2', '-0.721', '-0.200981348616']
BCSr 6 ['B0.285714C0.571429Sr0.142857', '-0.354', '-0.203617948571']
BCTa 21 ['B0.444444C0.222222Ta0.333333', '-0.802', '-0.202359764444']
BCTm 2 ['B0.555556C0.222222Tm0.222222', '-0.748', '-0.213727916389']
BCU 1 ['B0.375C0.375U0.25', '-0.594', '-0.206742779687']
BCV 7 ['B0.444444C0.111111V0.444444', '-0.943', '-0.201212222222']
BCW 44 ['B0.5C0.4W0.1', '-0.33', '-0.204765294173']
BCY 1 ['B0.625C0.125Y0.25', '-0.788', '-0.235294638125']
BCYb 1 ['B0.142857C0.571429Yb0.285714', '-0.382', '-0.203937521429']
BCaCl 4 ['B0.25Cl0.625Ca0.125', '-1.843', '-0.225357003365']
BCaF 8 ['B0.25F0.625Ca0.125', '-3.308', '-0.229003996638']
BCaH 3 ['H0.5B0.125Ca0.375', '-0.73', '-0.239207061486']
BCaI 9 ['B0.1Ca0.1I0.8', '-0.845', '-0.243577142287']
BCaN 15 ['B0.222222N0.444444Ca0.333333', '-1.426', '-0.200221875956']
BCaNi 1 ['B0.444444Ca0.111111Ni0.444444', '-0.598', '-0.225653315312']
BCaP 4 ['B0.4P0.5Ca0.1', '-0.906', '-0.231809145854']
BCaPa 1 ['B0.8Ca0.1Pa0.1', '-0.501', '-0.212087294']
BCaPu 2 ['B0.8Ca0.1Pu0.1', '-0.666', '-0.216572756']
BCaRh 1 ['B0.444444Ca0.111111Rh0.444444', '-0.741', '-0.212381587111']
BCaS 1 ['B0.2S0.5Ca0.3', '-1.811', '-0.222956118491']
BCaSe 3 ['B0.111111Ca0.555556Se0.333333', '-1.631', '-0.212960352847']
BCdF 3 ['B0.125F0.75Cd0.125', '-2.699', '-0.281368047887']
BCdI 12 ['B0.2Cd0.2I0.6', '-0.675', '-0.204431061987']
BCeCl 8 ['B0.1Cl0.7Ce0.2', '-2.439', '-0.203724304067']
BCeF 2 ['B0.111111F0.777778Ce0.111111', '-3.63', '-0.356493954422']
BCeH 14 ['H0.428571B0.428571Ce0.142857', '-0.587', '-0.203594209889']

BCeI 16 ['B0.222222I0.555556Ce0.222222', '-0.95', '-0.231627841456']
BCeN 9 ['B0.4N0.5Ce0.1', '-1.61', '-0.233813339078']
BCePt 1 ['B0.375Ce0.125Pt0.5', '-0.838', '-0.248611228259']
BCeRe 1 ['B0.571429Ce0.142857Re0.285714', '-0.598', '-0.209782337063']
BClCr 2 ['B0.166667Cl0.666667Cr0.166667', '-1.427', '-0.200790064869']
BClCs 11 ['B0.111111Cl0.666667Cs0.222222', '-1.731', '-0.220040245213']
BClCu 3 ['B0.111111Cl0.555556Cu0.333333', '-1.049', '-0.240545402991']
BClDy 1 ['B0.25Cl0.625Dy0.125', '-1.895', '-0.217885203782']
BClEr 38 ['B0.4Cl0.3Er0.3', '-1.2', '-0.225108528853']
BClEu 15 ['B0.285714Cl0.571429Eu0.142857', '-1.904', '-0.220180993576']
BClF 5 ['B0.25F0.625Cl0.125', '-2.906', '-0.203837407707']
BClFe 7 ['B0.166667Cl0.666667Fe0.166667', '-1.279', '-0.203177247566']
BClGd 7 ['B0.25Cl0.625Gd0.125', '-1.887', '-0.207116650552']
BClGe 1 ['B0.2Cl0.7Ge0.1', '-1.374', '-0.226592745632']
BClHg 4 ['B0.222222Cl0.666667Hg0.111111', '-1.312', '-0.204630639591']
BClHo 36 ['B0.333333Cl0.555556Ho0.111111', '-1.322', '-0.200717418839']
BClI 16 ['B0.222222Cl0.333333I0.444444', '-0.863', '-0.226657615097']
BClIr 5 ['B0.1Cl0.8Ir0.1', '-0.724', '-0.225683787816']
BClK 3 ['B0.166667Cl0.666667K0.166667', '-1.751', '-0.20677364459']
BClLa 4 ['B0.125Cl0.75La0.125', '-2.185', '-0.219932606415']
BClLi 4 ['Li0.111111B0.222222Cl0.666667', '-1.585', '-0.217782293479']
BClLu 48 ['B0.2Cl0.3Lu0.5', '-1.157', '-0.205788353314']
BClMg 12 ['B0.2Mg0.3Cl0.5', '-1.814', '-0.201246712067']
BClMn 1 ['B0.111111Cl0.666667Mn0.222222', '-1.472', '-0.212027957189']
BClNa 2 ['B0.142857Na0.285714Cl0.571429', '-1.85', '-0.203922766545']
BClNb 6 ['B0.111111Cl0.777778Nb0.111111', '-1.609', '-0.200805738407']
BClNd 1 ['B0.142857Cl0.714286Nd0.142857', '-2.149', '-0.208214861705']
BClNi 8 ['B0.25Cl0.625Ni0.125', '-1.291', '-0.208364047742']
BClNp 5 ['B0.1Cl0.7Np0.2', '-2.031', '-0.267622314819']
BClPa 17 ['B0.555556Cl0.111111Pa0.333333', '-0.493', '-0.208401971597']
BClPb 7 ['B0.2Cl0.6Pb0.2', '-1.387', '-0.218717259631']
BClPm 33 ['B0.25Cl0.5Pm0.25', '-1.65', '-0.207333459065']
BClPr 7 ['B0.1Cl0.7Pr0.2', '-2.445', '-0.217623494067']
BClPt 11 ['B0.1Cl0.8Pt0.1', '-0.889', '-0.209315632025']
BClPu 16 ['B0.1Cl0.6Pu0.3', '-2.01', '-0.207368153069']
BClRb 9 ['B0.142857Cl0.714286Rb0.142857', '-1.53', '-0.209002608934']
BClRh 11 ['B0.142857Cl0.571429Rh0.285714', '-0.924', '-0.21211969688']
BClRu 3 ['B0.2Cl0.7Ru0.1', '-1.325', '-0.221524675403']
BClSc 7 ['B0.111111Cl0.777778Sc0.111111', '-1.86', '-0.245208337369']
BClSi 1 ['B0.125Si0.125Cl0.75', '-1.598', '-0.228329396409']
BClSm 16 ['B0.142857Cl0.571429Sm0.285714', '-1.995', '-0.205760658559']
BClSr 2 ['B0.222222Cl0.666667Sr0.111111', '-1.885', '-0.235359202924']
BClTa 3 ['B0.125Cl0.75Ta0.125', '-1.676', '-0.263026758535']
BClTb 12 ['B0.2Cl0.4Tb0.4', '-1.596', '-0.211450400064']

BClTc 8 ['B0.1Cl0.8Tc0.1', '-1.179', '-0.204866354693']
BClTe 3 ['B0.2Cl0.7Te0.1', '-1.325', '-0.217002678799']
BClTh 4 ['B0.2Cl0.7Th0.1', '-1.952', '-0.229188343066']
BClTi 7 ['B0.1Cl0.7Ti0.2', '-1.923', '-0.220708199211']
BClTl 5 ['B0.2Cl0.7Tl0.1', '-1.442', '-0.20454540557']
BClTm 2 ['B0.1Cl0.8Tm0.1', '-1.728', '-0.222446332132']
BClU 5 ['B0.1Cl0.8U0.1', '-1.911', '-0.267184792004']
BClV 6 ['B0.2Cl0.7V0.1', '-1.487', '-0.200428315571']
BClW 6 ['B0.222222Cl0.666667W0.111111', '-1.314', '-0.206630639591']
BClXe 4 ['B0.166667Cl0.666667Xe0.166667', '-1.041', '-0.210472979693']
BClY 4 ['B0.111111Cl0.777778Y0.111111', '-1.916', '-0.227521924036']
BClYb 2 ['B0.2Cl0.7Yb0.1', '-1.861', '-0.21173109807']
BClZn 6 ['B0.125Cl0.625Zn0.25', '-1.46', '-0.214991203991']
BClZr 7 ['B0.1Cl0.7Zr0.2', '-2.084', '-0.205614512438']
BCoF 9 ['B0.125F0.625Co0.25', '-2.391', '-0.245247043443']
BCoH 3 ['H0.285714B0.285714Co0.428571', '-0.444', '-0.209666567143']
BCoI 18 ['B0.111111Co0.333333I0.555556', '-0.371', '-0.258283790318']
BCoP 7 ['B0.444444P0.333333Co0.222222', '-0.719', '-0.201021773014']
BCoPa 6 ['B0.714286Co0.142857Pa0.142857', '-0.362', '-0.244833283571']
BCoPm 7 ['B0.666667Co0.166667Pm0.166667', '-0.386', '-0.2493054975']
BCoSc 1 ['B0.666667Sc0.222222Co0.111111', '-0.932', '-0.223354282222']
BCrF 8 ['B0.166667F0.666667Cr0.166667', '-2.934', '-0.263611739051']
BCrI 16 ['B0.142857Cr0.285714I0.571429', '-0.596', '-0.216609991898']
BCrN 2 ['B0.142857N0.285714Cr0.571429', '-0.79', '-0.203806641569']
BCrPa 8 ['B0.777778Cr0.111111Pa0.111111', '-0.37', '-0.202682077778']
BCrPm 6 ['B0.714286Cr0.142857Pm0.142857', '-0.423', '-0.207876957143']
BCsF 8 ['B0.111111F0.666667Cs0.222222', '-3.08', '-0.263085655533']
BCsI 16 ['B0.222222I0.555556Cs0.222222', '-1.065', '-0.235725968374']
BCsP 1 ['B0.25P0.5Cs0.25', '-0.794', '-0.235166321949']
BCsPa 2 ['B0.777778Cs0.111111Pa0.111111', '-0.25', '-0.25']
BCsPm 1 ['B0.8Cs0.1Pm0.1', '-0.223', '-0.223']
BCsPu 1 ['B0.8Cs0.1Pu0.1', '-0.529', '-0.238657176875']
BCsS 1 ['B0.142857S0.571429Cs0.285714', '-1.201', '-0.220780265856']
BCsSe 4 ['B0.111111Se0.444444Cs0.444444', '-1.021', '-0.219515932222']
BCuF 11 ['B0.111111F0.666667Cu0.222222', '-2.45', '-0.254411949609']
BCuI 18 ['B0.111111Cu0.333333I0.555556', '-0.433', '-0.213885719392']
BCuPa 3 ['B0.625Cu0.125Pa0.25', '-0.204', '-0.204']
BDyF 5 ['B0.125F0.75Dy0.125', '-3.777', '-0.216211878415']
BDyH 13 ['H0.5B0.4Dy0.1', '-0.516', '-0.206126616957']
BDyI 18 ['B0.285714I0.571429Dy0.142857', '-0.668', '-0.20259791602']
BDyN 8 ['B0.333333N0.444444Dy0.222222', '-1.702', '-0.241081194698']
BDyPa 4 ['B0.666667Dy0.222222Pa0.111111', '-0.741', '-0.22110794963']
BDyRu 1 ['B0.5Ru0.333333Dy0.166667', '-0.766', '-0.214126325034']
BErF 2 ['B0.111111F0.777778Er0.111111', '-3.715', '-0.41048291072']

BErH 17 ['H0.428571B0.428571Er0.142857', '-0.612', '-0.200920111155']
BErI 20 ['B0.2I0.5Er0.3', '-0.965', '-0.207664436925']
BErN 19 ['B0.125N0.375Er0.5', '-1.74', '-0.208521983623']
BErOs 3 ['B0.6Er0.1Os0.3', '-0.655', '-0.2033034085']
BErPa 4 ['B0.666667Er0.222222Pa0.111111', '-0.75', '-0.215806194167']
BErPd 1 ['B0.142857Pd0.714286Er0.142857', '-0.853', '-0.215563957143']
BErPu 1 ['B0.666667Er0.166667Pu0.166667', '-0.719', '-0.200126346459']
BErRh 1 ['B0.375Rh0.5Er0.125', '-0.853', '-0.21320343375']
BErU 1 ['B0.666667Er0.222222U0.111111', '-0.825', '-0.215430023889']
BEuF 4 ['B0.2F0.7Eu0.1', '-3.618', '-0.2524619734']
BEuI 12 ['B0.2I0.6Eu0.2', '-1.38', '-0.209399962207']
BEuN 14 ['B0.4N0.5Eu0.1', '-1.555', '-0.222601241285']
BEuNi 4 ['B0.5Ni0.4Eu0.1', '-0.648', '-0.203890147618']
BEuP 18 ['B0.25P0.5Eu0.25', '-1.153', '-0.204477651659']
BEuPu 1 ['B0.714286Eu0.142857Pu0.142857', '-0.661', '-0.214685584524']
BEuRh 1 ['B0.375Rh0.5Eu0.125', '-0.995', '-0.367395086875']
BEuS 3 ['B0.166667S0.5Eu0.333333', '-2.064', '-0.250500254241']
BEuSe 12 ['B0.142857Se0.571429Eu0.285714', '-1.462', '-0.200923202857']
BFFe 9 ['B0.166667F0.666667Fe0.166667', '-2.837', '-0.293261147872']
BFGa 6 ['B0.2F0.7Ga0.1', '-3.064', '-0.286286914525']
BFGd 3 ['B0.125F0.75Gd0.125', '-3.82', '-0.24561914206']
BFGe 5 ['B0.142857F0.714286Ge0.142857', '-2.926', '-0.224184297941']
BFH 13 ['H0.25B0.125F0.625', '-2.511', '-0.20210940359']
BFHf 2 ['B0.111111F0.777778Hf0.111111', '-3.905', '-0.299113341096']
BFHg 3 ['B0.125F0.75Hg0.125', '-2.332', '-0.214545323516']
BFHo 2 ['B0.1F0.8Ho0.1', '-3.42', '-0.445696912064']
BFI 16 ['B0.25F0.25I0.5', '-1.323', '-0.201201108066']
BFIn 5 ['B0.142857F0.714286In0.142857', '-3.042', '-0.20212947321']
BFIr 3 ['B0.125F0.75Ir0.125', '-2.512', '-0.238690335495']
BFK 5 ['B0.111111F0.777778K0.111111', '-2.478', '-0.3100665565']
BFKr 2 ['B0.111111F0.777778Kr0.111111', '-1.775', '-0.340628383122']
BFLa 3 ['B0.142857F0.714286La0.142857', '-3.921', '-0.221081010027']
BFLi 6 ['Li0.222222B0.111111F0.666667', '-3.008', '-0.206299611962']
BFLu 5 ['B0.166667F0.666667Lu0.166667', '-3.57', '-0.207302168009']
BFMg 8 ['B0.111111F0.666667Mg0.222222', '-3.662', '-0.226273234003']
BFMn 8 ['B0.111111F0.666667Mn0.222222', '-2.892', '-0.227912426738']
BFMo 4 ['B0.142857F0.714286Mo0.142857', '-2.853', '-0.20743349187']
BFNa 11 ['B0.2F0.7Na0.1', '-3.326', '-0.209835078396']
BFNb 3 ['B0.111111F0.777778Nb0.111111', '-3.364', '-0.270243338684']
BFNd 3 ['B0.125F0.75Nd0.125', '-3.982', '-0.28929053341']
BFNi 10 ['B0.125F0.625Ni0.25', '-2.462', '-0.243365832888']
BFNp 3 ['B0.125F0.75Np0.125', '-3.666', '-0.310066996401']
BFO 10 ['B0.25O0.125F0.625', '-3.033', '-0.265248545332']
BFOs 1 ['B0.1F0.8Os0.1', '-2.54', '-0.202493532526']

BFP 4 ['B0.1F0.8P0.1', '-3.158', '-0.248873628672']
BFPa 8 ['B0.2F0.6Pa0.2', '-3.101', '-0.22309965852']
BFPb 4 ['B0.142857F0.714286Pb0.142857', '-3.058', '-0.2084400533']
BFPd 4 ['B0.125F0.75Pd0.125', '-2.462', '-0.21272044487']
BFPm 2 ['B0.111111F0.777778Pm0.111111', '-3.592', '-0.408578346831']
BFPr 4 ['B0.2F0.7Pr0.1', '-3.571', '-0.221034557193']
BFPt 3 ['B0.125F0.75Pt0.125', '-2.491', '-0.292881805967']
BFPu 8 ['B0.166667F0.666667Pu0.166667', '-3.557', '-0.289847934676']
BFRb 7 ['B0.222222F0.666667Rb0.111111', '-3.262', '-0.20277969799']
BFRe 2 ['B0.111111F0.777778Re0.111111', '-2.726', '-0.24927179768']
BFRh 6 ['B0.166667F0.666667Rh0.166667', '-2.596', '-0.201624708149']
BFRu 4 ['B0.142857F0.714286Ru0.142857', '-2.619', '-0.211459456156']
BFSb 2 ['B0.111111F0.777778Sb0.111111', '-2.845', '-0.206310345764']
BFSc 5 ['B0.2F0.7Sc0.1', '-3.538', '-0.233566819941']
BFSe 2 ['B0.2F0.7Se0.1', '-2.862', '-0.22144882311']
BFSi 2 ['B0.2F0.7Si0.1', '-3.112', '-0.242119198986']
BFSm 2 ['B0.111111F0.777778Sm0.111111', '-3.642', '-0.34626511572']
BFSn 2 ['B0.111111F0.777778Sn0.111111', '-3.109', '-0.301088031996']
BFSr 8 ['B0.166667F0.666667Sr0.166667', '-3.649', '-0.215589725439']
BFTa 3 ['B0.125F0.75Ta0.125', '-3.434', '-0.30699261369']
BFTb 2 ['B0.1F0.8Tb0.1', '-3.127', '-0.21739498168']
BFTc 3 ['B0.125F0.75Tc0.125', '-2.782', '-0.237759095459']
BFTe 1 ['B0.1F0.8Te0.1', '-2.836', '-0.256090023738']
BFTh 1 ['B0.1F0.8Th0.1', '-3.752', '-0.324438581651']
BFTi 5 ['B0.2F0.7Ti0.1', '-3.264', '-0.207688841148']
BFTl 5 ['B0.111111F0.666667Tl0.222222', '-2.809', '-0.220891971692']
BFTm 30 ['B0.222222F0.333333Tm0.444444', '-1.946', '-0.208619599194']
BFU 3 ['B0.125F0.75U0.125', '-3.601', '-0.202353126883']
BFV 7 ['B0.166667F0.666667V0.166667', '-3.01', '-0.208066694497']
BFW 2 ['B0.1F0.8W0.1', '-3.115', '-0.212128744823']
BFXe 1 ['B0.1F0.8Xe0.1', '-1.835', '-0.260737920483']
BFY 8 ['B0.166667F0.666667Y0.166667', '-3.679', '-0.215048706896']
BFYb 32 ['B0.3F0.4Yb0.3', '-2.234', '-0.203111241568']
BFZn 6 ['B0.125F0.625Zn0.25', '-2.692', '-0.232613765389']
BFZr 1 ['B0.1F0.8Zr0.1', '-3.635', '-0.457144402361']
BFeH 1 ['H0.444444B0.444444Fe0.111111', '-0.365', '-0.219261952192']
BFeI 14 ['B0.222222Fe0.222222I0.555556', '-0.44', '-0.202474444207']
BFeN 2 ['B0.3N0.4Fe0.3', '-1.107', '-0.232797272864']
BFeNb 1 ['B0.375Fe0.25Nb0.375', '-0.825', '-0.222706180833']
BFeP 15 ['B0.25P0.25Fe0.5', '-0.754', '-0.20109563901']
BFePa 9 ['B0.6Fe0.1Pa0.3', '-0.281', '-0.2056797505']
BFePm 8 ['B0.666667Fe0.222222Pm0.111111', '-0.384', '-0.216621667778']
BFeS 1 ['B0.3S0.5Fe0.2', '-0.791', '-0.201949173593']
BFeSm 1 ['B0.444444Fe0.444444Sm0.111111', '-0.585', '-0.203802780334']

BGaI 9 ['B0.25Ga0.125I0.625', '-0.597', '-0.224396920566']
BGdH 2 ['H0.5B0.4Gd0.1', '-0.503', '-0.204520065226']
BGdI 17 ['B0.222222I0.555556Gd0.222222', '-0.887', '-0.247548519718']
BGdN 4 ['B0.375N0.375Gd0.25', '-1.501', '-0.200572398444']
BGdNi 1 ['B0.444444Ni0.444444Gd0.111111', '-0.688', '-0.209083441688']
BGdPa 4 ['B0.714286Gd0.142857Pa0.142857', '-0.614', '-0.221730879796']
BGdPd 1 ['B0.142857Pd0.714286Gd0.142857', '-0.815', '-0.203652833214']
BGdRh 1 ['B0.375Rh0.5Gd0.125', '-0.837', '-0.208900659687']
BGdV 1 ['B0.666667V0.222222Gd0.111111', '-0.909', '-0.211298564445']
BGeI 9 ['B0.111111Ge0.222222I0.666667', '-0.604', '-0.219300282733']
BGeSe 1 ['B0.111111Ge0.333333Se0.555556', '-0.465', '-0.242927435278']
BHHo 5 ['H0.428571B0.428571Ho0.142857', '-0.614', '-0.201083755815']
BHI 55 ['H0.571429B0.285714I0.142857', '-0.283', '-0.203254639632']
BHLa 18 ['H0.571429B0.142857La0.285714', '-0.826', '-0.211819637085']
BHLu 3 ['H0.5B0.4Lu0.1', '-0.505', '-0.226171289297']
BHMg 1 ['H0.4B0.5Mg0.1', '-0.404', '-0.222728575001']
BHOs 1 ['H0.111111B0.555556Os0.333333', '-0.419', '-0.22256838589']
BHPa 26 ['H0.555556B0.111111Pa0.333333', '-0.475', '-0.206507790453']
BHPm 44 ['H0.333333B0.5Pm0.166667', '-0.37', '-0.204767190105']
BHPr 7 ['H0.625B0.25Pr0.125', '-0.51', '-0.202360782266']
BHRb 5 ['H0.6B0.2Rb0.2', '-0.432', '-0.213270656032']
BHS 1 ['H0.625B0.125S0.25', '-0.439', '-0.206915774661']
BHSc 2 ['H0.375B0.5Sc0.125', '-0.564', '-0.203037149353']
BHSm 6 ['H0.4B0.5Sm0.1', '-0.525', '-0.210081542081']
BHTm 2 ['H0.4B0.5Tm0.1', '-0.561', '-0.243538806364']
BHYb 16 ['H0.444444B0.222222Yb0.333333', '-0.742', '-0.211221626437']
BHfI 7 ['B0.166667I0.666667Hf0.166667', '-1.11', '-0.237224329633']
BHfN 4 ['B0.444444N0.444444Hf0.111111', '-1.634', '-0.228903502627']
BHfPa 1 ['B0.666667Hf0.111111Pa0.222222', '-0.551', '-0.209845108333']
BHfRh 1 ['B0.111111Rh0.666667Hf0.222222', '-0.938', '-0.211299903']
BHgI 13 ['B0.1I0.6Hg0.3', '-0.567', '-0.207378115178']
BHoI 15 ['B0.222222I0.555556Ho0.222222', '-0.806', '-0.204025069001']
BHoN 18 ['B0.222222N0.444444Ho0.333333', '-1.796', '-0.213860933125']
BHoNi 2 ['B0.428571Ni0.428571Ho0.142857', '-0.796', '-0.281881081131']
BHoO 1 ['B0.25O0.625Ho0.125', '-3.154', '-0.232969247527']
BHoOs 1 ['B0.555556Ho0.111111Os0.333333', '-0.648', '-0.202300051852']
BHoP 1 ['B0.125P0.5Ho0.375', '-1.722', '-0.282737409268']
BHoPa 4 ['B0.666667Ho0.166667Pa0.166667', '-0.706', '-0.211010642361']
BHoPd 1 ['B0.166667Pd0.666667Ho0.166667', '-0.892', '-0.213525423498']
BHoS 1 ['B0.1S0.5Ho0.4', '-2.255', '-0.245510894555']
BIIn 10 ['B0.111111In0.222222I0.666667', '-0.765', '-0.207699916715']
BIIr 18 ['B0.25I0.5Ir0.25', '-0.349', '-0.212395086008']
BIK 16 ['B0.222222K0.222222I0.555556', '-0.997', '-0.210424584764']
BIKr 14 ['B0.222222Kr0.222222I0.555556', '-0.387', '-0.249237158836']

BILa 10 ['B0.25I0.625La0.125', '-1.187', '-0.288973653066']
BILi 17 ['Li0.1B0.3I0.6', '-0.609', '-0.220626010211']
BILu 23 ['B0.25I0.5Lu0.25', '-0.69', '-0.264223027358']
BIMg 12 ['B0.125Mg0.25I0.625', '-1.187', '-0.275141187231']
BIMn 12 ['B0.2Mn0.2I0.6', '-0.611', '-0.218564034213']
BIMo 8 ['B0.111111Mo0.222222I0.666667', '-0.61', '-0.217312345707']
BIN 5 ['B0.3N0.1I0.6', '-0.633', '-0.209727176509']
BINa 18 ['B0.1Na0.4I0.5', '-1.37', '-0.212310323279']
BINb 8 ['B0.1Nb0.2I0.7', '-0.846', '-0.2488332543']
BINd 12 ['B0.125I0.625Nd0.25', '-1.4', '-0.303717728271']
BINi 26 ['B0.333333Ni0.111111I0.555556', '-0.398', '-0.205133485295']
BINp 8 ['B0.1I0.7Np0.2', '-1.101', '-0.269916477383']
BIO 8 ['B0.333333O0.166667I0.5', '-1.078', '-0.215000241868']
BIOs 11 ['B0.1I0.7Os0.2', '-0.308', '-0.233608065772']
BIP 20 ['B0.222222P0.333333I0.444444', '-0.568', '-0.209301632323']
BIPa 29 ['B0.25I0.375Pa0.375', '-0.322', '-0.21799096284']
BIPb 10 ['B0.111111I0.666667Pb0.222222', '-0.807', '-0.231318254122']
BIPd 18 ['B0.25Pd0.25I0.5', '-0.504', '-0.238657708161']
BIPm 24 ['B0.222222I0.444444Pm0.333333', '-0.826', '-0.202031766791']
BIPr 10 ['B0.25I0.625Pr0.125', '-1.011', '-0.262659051087']
BIPt 31 ['B0.25I0.375Pt0.375', '-0.444', '-0.210793280756']
BIPu 8 ['B0.166667I0.666667Pu0.166667', '-1.064', '-0.217382336438']
BIRb 17 ['B0.166667Rb0.333333I0.5', '-1.321', '-0.212643396844']
BIRe 11 ['B0.2I0.6Re0.2', '-0.445', '-0.210834466098']
BIRh 21 ['B0.166667Rh0.333333I0.5', '-0.397', '-0.258296378333']
BIRu 17 ['B0.1Ru0.3I0.6', '-0.275', '-0.200608065772']
BIS 11 ['B0.2S0.2I0.6', '-0.484', '-0.234473439878']
BISb 7 ['B0.166667Sb0.166667I0.666667', '-0.566', '-0.200591350604']
BISc 12 ['B0.125Sc0.25I0.625', '-1.2', '-0.245599615771']
BISe 24 ['B0.222222Se0.333333I0.444444', '-0.365', '-0.254789727069']
BISi 8 ['B0.166667Si0.166667I0.666667', '-0.676', '-0.250138542548']
BISm 14 ['B0.1I0.6Sm0.3', '-1.363', '-0.252413398039']
BISn 10 ['B0.25Sn0.125I0.625', '-0.564', '-0.20644922273']
BISr 9 ['B0.111111Sr0.222222I0.666667', '-1.487', '-0.214592397455']
BITa 8 ['B0.1I0.7Ta0.2', '-0.767', '-0.202517787112']
BITb 27 ['B0.2I0.4Tb0.4', '-0.664', '-0.244361958316']
BITc 12 ['B0.2Tc0.2I0.6', '-0.409', '-0.233355574025']
BITe 13 ['B0.142857Te0.285714I0.571429', '-0.415', '-0.232599960874']
BITh 6 ['B0.222222I0.666667Th0.111111', '-1.076', '-0.257996641623']
BITi 9 ['B0.1Ti0.2I0.7', '-1.086', '-0.254446168168']
BITl 15 ['B0.222222I0.555556Tl0.222222', '-0.622', '-0.200548744762']
BITm 13 ['B0.1I0.6Tm0.3', '-1.335', '-0.208099950802']
BIU 6 ['B0.222222I0.666667U0.111111', '-0.855', '-0.261526134122']
BIV 12 ['B0.125V0.25I0.625', '-0.728', '-0.24007856317']

BIW 12 ['B0.25I0.625W0.125', '-0.395', '-0.206954561447']
BIXe 23 ['B0.142857I0.428571Xe0.428571', '-0.391', '-0.284725808245']
BIY 8 ['B0.1Y0.2I0.7', '-1.585', '-0.309265617135']
BIYb 12 ['B0.125I0.625Yb0.25', '-1.565', '-0.200247054523']
BIZn 12 ['B0.125Zn0.25I0.625', '-0.73', '-0.254743792024']
BIZr 11 ['B0.125Zr0.25I0.625', '-1.256', '-0.224453375667']
BIrMg 7 ['B0.333333Mg0.222222Ir0.444444', '-0.57', '-0.248204025741']
BIrNa 1 ['B0.375Na0.125Ir0.5', '-0.271', '-0.21421739375']
BIrP 20 ['B0.222222P0.333333Ir0.444444', '-0.721', '-0.217973579544']
BIrPa 3 ['B0.625Ir0.125Pa0.25', '-0.381', '-0.22513091625']
BIrPm 4 ['B0.4Pm0.2Ir0.4', '-0.502', '-0.2146969985']
BIrS 2 ['B0.25S0.5Ir0.25', '-0.726', '-0.240564598722']
BIrSr 3 ['B0.375Sr0.125Ir0.5', '-0.48', '-0.232878965']
BKN 1 ['B0.428571N0.428571K0.142857', '-1.47', '-0.29361876414']
BKPa 2 ['B0.777778K0.111111Pa0.111111', '-0.276', '-0.254167792778']
BKrPa 2 ['B0.666667Kr0.111111Pa0.222222', '-0.202', '-0.202']
BKrPm 1 ['B0.8Kr0.1Pm0.1', '-0.209', '-0.209']
BKrS 2 ['B0.111111S0.555556Kr0.333333', '-0.375', '-0.208390052248']
BLaN 8 ['B0.1N0.4La0.5', '-1.428', '-0.203541828363']
BLaNi 2 ['B0.5Ni0.375La0.125', '-0.747', '-0.268456812448']
BLaO 1 ['B0.3O0.6La0.1', '-3.27', '-0.24328496347']
BLaPt 5 ['B0.222222La0.111111Pt0.666667', '-0.798', '-0.224094590915']
BLaRh 2 ['B0.375Rh0.5La0.125', '-0.803', '-0.202000595625']
BLiN 3 ['Li0.142857B0.428571N0.428571', '-1.418', '-0.227377494318']
BLiP 1 ['Li0.3B0.3P0.4', '-0.841', '-0.214386724916']
BLiPa 6 ['Li0.111111B0.777778Pa0.111111', '-0.339', '-0.232350519722']
BLiPm 2 ['Li0.111111B0.777778Pm0.111111', '-0.368', '-0.261350519722']
BLiPt 2 ['Li0.3B0.1Pt0.6', '-0.707', '-0.207612780084']
BLuN 28 ['B0.1N0.4Lu0.5', '-1.902', '-0.211802968114']
BLuNi 3 ['B0.5Ni0.4Lu0.1', '-0.691', '-0.23242871315']
BLuOs 2 ['B0.555556Lu0.111111Os0.333333', '-0.671', '-0.205609767222']
BLuPa 7 ['B0.75Lu0.125Pa0.125', '-0.571', '-0.209637522344']
BLuPu 2 ['B0.7Lu0.2Pu0.1', '-0.814', '-0.213691593313']
BLuRh 1 ['B0.375Rh0.5Lu0.125', '-0.84', '-0.20027818']
BMgN 17 ['B0.25N0.375Mg0.375', '-1.167', '-0.211334517138']
BMgO 1 ['B0.111111O0.444444Mg0.444444', '-2.859', '-0.235557627952']
BMgP 1 ['B0.142857Mg0.285714P0.571429', '-0.831', '-0.218345704225']
BMgPa 10 ['B0.666667Mg0.222222Pa0.111111', '-0.332', '-0.205432119352']
BMgPm 1 ['B0.8Mg0.1Pm0.1', '-0.415', '-0.306578864334']
BMgRh 3 ['B0.166667Mg0.166667Rh0.666667', '-0.539', '-0.219620448056']
BMgU 1 ['B0.8Mg0.1U0.1', '-0.53', '-0.203952650333']
BMnN 8 ['B0.375N0.375Mn0.25', '-1.238', '-0.208666418622']
BMnPa 4 ['B0.666667Mn0.166667Pa0.166667', '-0.448', '-0.222822439368']
BMnPm 7 ['B0.666667Mn0.166667Pm0.166667', '-0.481', '-0.255822439368']

BMoN 5 ['B0.333333N0.222222Mo0.444444', '-0.939', '-0.217808326869']
BMoP 7 ['B0.1P0.5Mo0.4', '-0.933', '-0.20098989552']
BMoPa 5 ['B0.666667Mo0.111111Pa0.222222', '-0.37', '-0.225127253333']
BMoPm 7 ['B0.777778Mo0.111111Pm0.111111', '-0.375', '-0.230127253333']
BNNa 10 ['B0.2N0.5Na0.3', '-0.918', '-0.201877655448']
BNNb 1 ['B0.444444N0.333333Nb0.222222', '-1.513', '-0.361841859609']
BNNd 7 ['B0.4N0.5Nd0.1', '-1.611', '-0.22026418183']
BNNp 17 ['B0.125N0.5Np0.375', '-1.476', '-0.22354589717']
BNP 1 ['B0.333333N0.444444P0.222222', '-1.217', '-0.208982463918']
BNPa 35 ['B0.2N0.4Pa0.4', '-1.375', '-0.218659373861']
BNPm 12 ['B0.1N0.4Pm0.5', '-1.46', '-0.226767928864']
BNPr 4 ['B0.428571N0.428571Pr0.142857', '-1.511', '-0.228008498017']
BNPu 18 ['B0.4N0.4Pu0.2', '-1.431', '-0.234430171764']
BNSc 24 ['B0.142857N0.428571Sc0.428571', '-1.992', '-0.202009122473']
BNSi 1 ['B0.3N0.5Si0.2', '-1.476', '-0.22745400033']
BNSm 18 ['B0.2N0.5Sm0.3', '-1.722', '-0.22114006508']
BNSr 17 ['B0.5N0.4Sr0.1', '-1.352', '-0.201410461864']
BNTa 1 ['B0.428571N0.142857Ta0.428571', '-1.175', '-0.212961494237']
BNTb 9 ['B0.333333N0.5Tb0.166667', '-1.597', '-0.20328144655']
BNTh 4 ['B0.4N0.4Th0.2', '-1.52', '-0.200855700564']
BNTi 7 ['B0.4N0.4Ti0.2', '-1.574', '-0.201397103106']
BNTm 12 ['B0.1N0.4Tm0.5', '-1.857', '-0.215853510864']
BNU 13 ['B0.166667N0.5U0.333333', '-1.612', '-0.210626308163']
BNY 17 ['B0.444444N0.333333Y0.222222', '-1.452', '-0.20900717472']
BNYb 36 ['B0.285714N0.285714Yb0.428571', '-1.094', '-0.202976015043']
BNZn 4 ['B0.4N0.5Zn0.1', '-1.327', '-0.229044179864']
BNaPm 1 ['B0.8Na0.1Pm0.1', '-0.278', '-0.215397676995']
BNbO 1 ['B0.111111O0.666667Nb0.222222', '-3.027', '-0.27155569622']
BNbPa 2 ['B0.666667Nb0.111111Pa0.222222', '-0.479', '-0.24731595']
BNbS 2 ['B0.1S0.6Nb0.3', '-1.301', '-0.200369336441']
BNdP 1 ['B0.4P0.5Nd0.1', '-0.977', '-0.21558106902']
BNdPa 4 ['B0.777778Nd0.111111Pa0.111111', '-0.582', '-0.212131479445']
BNdPu 1 ['B0.8Nd0.1Pu0.1', '-0.802', '-0.27086494275']
BNdRh 1 ['B0.333333Rh0.5Nd0.166667', '-0.877', '-0.2136944125']
BNiP 12 ['B0.222222P0.333333Ni0.444444', '-0.731', '-0.200297500939']
BNiPa 8 ['B0.714286Ni0.142857Pa0.142857', '-0.307', '-0.225807841667']
BNiPm 10 ['B0.5Ni0.375Pm0.125', '-0.398', '-0.205802547969']
BNiS 6 ['B0.125S0.5Ni0.375', '-0.741', '-0.202011697212']
BNiSm 1 ['B0.444444Ni0.444444Sm0.111111', '-0.67', '-0.204256385278']
BNiSr 1 ['B0.8Ni0.1Sr0.1', '-0.627', '-0.261604385812']
BNiTa 1 ['B0.428571Ni0.142857Ta0.428571', '-0.94', '-0.207654267857']
BNiTb 1 ['B0.428571Ni0.428571Tb0.142857', '-0.62', '-0.202817608372']
BNiTm 1 ['B0.444444Ni0.444444Tm0.111111', '-0.686', '-0.213482681795']
BOSc 2 ['B0.111111O0.333333Sc0.555556', '-2.457', '-0.202038514428']

BOsP 8 ['B0.375P0.25Os0.375', '-0.621', '-0.215510089202']
BOsPa 13 ['B0.555556Os0.333333Pa0.111111', '-0.422', '-0.223400833889']
BOsPm 8 ['B0.666667Pm0.166667Os0.166667', '-0.339', '-0.2324516']
BOsPu 1 ['B0.555556Os0.333333Pu0.111111', '-0.584', '-0.205031481111']
BOsSc 1 ['B0.111111Sc0.666667Os0.222222', '-0.538', '-0.256318654722']
BOsSe 1 ['B0.1Se0.6Os0.3', '-0.373', '-0.203506603125']
BOsY 1 ['B0.555556Y0.111111Os0.333333', '-0.678', '-0.245487694166']
BOsYb 6 ['B0.5Yb0.125Os0.375', '-0.581', '-0.237240716562']
BPPa 9 ['B0.2P0.3Pa0.5', '-1.076', '-0.202526174312']
BPPm 13 ['B0.142857P0.428571Pm0.428571', '-1.242', '-0.220484352231']
BPPt 3 ['B0.25P0.375Pt0.375', '-0.799', '-0.216433902828']
BPRu 11 ['B0.4P0.2Ru0.4', '-0.661', '-0.201634828541']
BPSc 1 ['B0.125P0.25Sc0.625', '-1.243', '-0.220624504638']
BPSr 5 ['B0.4P0.5Sr0.1', '-0.865', '-0.201629352686']
BPTa 7 ['B0.1P0.5Ta0.4', '-0.852', '-0.21080952352']
BPTb 4 ['B0.3P0.4Tb0.3', '-1.204', '-0.202646401618']
BPTe 6 ['B0.3P0.5Te0.2', '-0.53', '-0.208925573312']
BPV 2 ['B0.142857P0.428571V0.428571', '-1.071', '-0.207672615446']
BPW 7 ['B0.285714P0.285714W0.428571', '-0.66', '-0.214304453154']
BPXe 3 ['B0.428571P0.428571Xe0.142857', '-0.666', '-0.207322247589']
BPYb 6 ['B0.333333P0.444444Yb0.222222', '-0.938', '-0.215618258399']
BPaPm 10 ['B0.625Pm0.125Pa0.25', '-0.313', '-0.313']
BPaPr 3 ['B0.714286Pr0.142857Pa0.142857', '-0.646', '-0.209217717679']
BPaRb 2 ['B0.777778Rb0.111111Pa0.111111', '-0.238', '-0.238']
BPaRe 7 ['B0.625Re0.25Pa0.125', '-0.53', '-0.21489535125']
BPaRh 4 ['B0.666667Rh0.166667Pa0.166667', '-0.413', '-0.213066805']
BPaRu 7 ['B0.75Ru0.125Pa0.125', '-0.306', '-0.20209558125']
BPaSc 6 ['B0.7Sc0.2Pa0.1', '-0.716', '-0.2058571893']
BPaSe 11 ['B0.2Se0.6Pa0.2', '-0.69', '-0.208389858']
BPaSm 3 ['B0.714286Sm0.142857Pa0.142857', '-0.651', '-0.2142981775']
BPaSr 1 ['B0.8Sr0.1Pa0.1', '-0.58', '-0.264197692']
BPaTa 4 ['B0.666667Ta0.166667Pa0.166667', '-0.527', '-0.202499491666']
BPaTb 2 ['B0.7Tb0.1Pa0.2', '-0.52', '-0.276684225283']
BPaTc 10 ['B0.571429Tc0.142857Pa0.285714', '-0.39', '-0.200893927143']
BPaTh 1 ['B0.7Th0.1Pa0.2', '-0.549', '-0.207656977']
BPaTi 2 ['B0.7Ti0.1Pa0.2', '-0.566', '-0.24595002962']
BPaTm 5 ['B0.714286Tm0.142857Pa0.142857', '-0.643', '-0.222200458884']
BPaV 2 ['B0.625V0.125Pa0.25', '-0.488', '-0.2134664675']
BPaW 3 ['B0.7W0.1Pa0.2', '-0.329', '-0.22917041675']
BPaXe 1 ['B0.625Xe0.125Pa0.25', '-0.255', '-0.255']
BPaY 4 ['B0.714286Y0.142857Pa0.142857', '-0.63', '-0.204876407902']
BPaYb 2 ['B0.7Yb0.1Pa0.2', '-0.558', '-0.233016815']
BPaZr 2 ['B0.7Zr0.1Pa0.2', '-0.526', '-0.228494344']
BPdPm 7 ['B0.2Pd0.7Pm0.1', '-0.59', '-0.22533909675']

BPdSm 1 ['B0.142857Pd0.714286Sm0.142857', '-0.829', '-0.241933636071']
BPmPu 4 ['B0.7Pm0.2Pu0.1', '-0.493', '-0.212944576094']
BPmRb 1 ['B0.8Rb0.1Pm0.1', '-0.308', '-0.308']
BPmRe 3 ['B0.666667Pm0.166667Re0.166667', '-0.428', '-0.217930234167']
BPmRh 2 ['B0.166667Rh0.666667Pm0.166667', '-0.609', '-0.24268626875']
BPmRu 7 ['B0.666667Ru0.166667Pm0.166667', '-0.419', '-0.280460775']
BPmS 15 ['B0.142857S0.285714Pm0.571429', '-1.275', '-0.206229049566']
BPmSc 1 ['B0.777778Sc0.111111Pm0.111111', '-0.51', '-0.217434298889']
BPmSe 11 ['B0.2Se0.5Pm0.3', '-1.179', '-0.20641187925']
BPmW 2 ['B0.666667Pm0.222222W0.111111', '-0.332', '-0.221078240833']
BPmXe 2 ['B0.777778Xe0.111111Pm0.111111', '-0.221', '-0.221']
BPrRe 1 ['B0.6Pr0.1Re0.3', '-0.646', '-0.22909540125']
BPrRu 1 ['B0.7Ru0.1Pr0.2', '-0.749', '-0.218510422875']
BPtS 1 ['B0.25S0.5Pt0.25', '-0.756', '-0.208501139511']
BPtTh 7 ['B0.166667Pt0.666667Th0.166667', '-0.759', '-0.225680244167']
BPtY 1 ['B0.2Y0.2Pt0.6', '-1.138', '-0.287157691891']
BPuS 1 ['B0.1S0.5Pu0.4', '-1.901', '-0.208102834116']
BPuSr 2 ['B0.8Sr0.1Pu0.1', '-0.667', '-0.20727190175']
BPuYb 3 ['B0.777778Yb0.111111Pu0.111111', '-0.671', '-0.213572423611']
BReS 6 ['B0.142857S0.571429Re0.285714', '-0.89', '-0.204763780581']
BReSe 2 ['B0.142857Se0.571429Re0.285714', '-0.551', '-0.207615641607']
BReSr 1 ['B0.6Sr0.1Re0.3', '-0.607', '-0.2288744215']
BReU 1 ['B0.6Re0.3U0.1', '-0.695', '-0.2266719202']
BReYb 1 ['B0.6Yb0.1Re0.3', '-0.623', '-0.2448744215']
BRhSc 6 ['B0.111111Sc0.333333Rh0.555556', '-1.079', '-0.200170211667']
BRhSr 2 ['B0.4Sr0.1Rh0.5', '-0.775', '-0.2927148637']
BRhTh 1 ['B0.166667Rh0.666667Th0.166667', '-0.835', '-0.220648360833']
BSSm 2 ['B0.125S0.5Sm0.375', '-2.186', '-0.204826072601']
BSTc 1 ['B0.142857S0.571429Tc0.285714', '-0.986', '-0.255541587635']
BSW 1 ['B0.142857S0.571429W0.285714', '-1.105', '-0.243754117703']
BSXe 11 ['B0.125S0.625Xe0.25', '-0.389', '-0.201563808779']
BScSe 16 ['B0.1Sc0.5Se0.4', '-1.608', '-0.20702012225']
BSeTc 4 ['B0.111111Se0.555556Tc0.333333', '-0.323', '-0.203907708333']
BSeY 14 ['B0.25Se0.5Y0.25', '-1.097', '-0.21404938625']
BSeYb 3 ['B0.125Se0.375Yb0.5', '-1.875', '-0.227675638542']
BSeZn 2 ['B0.142857Zn0.285714Se0.571429', '-0.624', '-0.213317311429']
BTcTm 1 ['B0.666667Tc0.166667Tm0.166667', '-0.749', '-0.202764945']
BaBeF 6 ['Be0.2F0.7Ba0.1', '-3.647', '-0.26961844277']
BaBiBr 25 ['Br0.333333Ba0.333333Bi0.333333', '-1.76', '-0.221969632528']
BaBiF 7 ['F0.555556Ba0.333333Bi0.111111', '-3.765', '-0.212401110903']
BaBiH 15 ['H0.571429Ba0.285714Bi0.142857', '-0.668', '-0.203698047977']
BaBiI 15 ['I0.3Ba0.4Bi0.3', '-1.511', '-0.200034645145']
BaBiO 9 ['O0.222222Ba0.555556Bi0.222222', '-1.899', '-0.200228388508']
BaBiSe 17 ['Se0.166667Ba0.5Bi0.333333', '-1.459', '-0.201218735556']

BaBiTe 1 ['Te0.5Ba0.4Bi0.1', '-1.872', '-0.399477402664']
BaBrCl 2 ['Cl0.285714Br0.428571Ba0.285714', '-2.46', '-0.215507276631']
BaBrCr 5 ['Cr0.111111Br0.777778Ba0.111111', '-1.305', '-0.211080931426']
BaBrDy 6 ['Br0.8Ba0.1Dy0.1', '-1.576', '-0.346013895118']
BaBrEr 11 ['Br0.6Ba0.1Er0.3', '-1.654', '-0.290591901541']
BaBrF 15 ['F0.5Br0.375Ba0.125', '-2.179', '-0.225689191485']
BaBrGa 1 ['Ga0.111111Br0.555556Ba0.333333', '-2.421', '-0.315469919353']
BaBrGe 1 ['Ge0.166667Br0.666667Ba0.166667', '-1.857', '-0.255427299431']
BaBrHf 11 ['Br0.6Ba0.1Hf0.3', '-1.137', '-0.216023908042']
BaBrHo 10 ['Br0.625Ba0.125Ho0.25', '-1.838', '-0.21131831922']
BaBrI 7 ['Br0.5I0.25Ba0.25', '-2.047', '-0.222700964418']
BaBrIr 3 ['Br0.8Ba0.1Ir0.1', '-1.146', '-0.208754452042']
BaBrLu 11 ['Br0.6Ba0.1Lu0.3', '-1.627', '-0.314144561042']
BaBrMg 4 ['Mg0.2Br0.6Ba0.2', '-2.175', '-0.200148116133']
BaBrMo 1 ['Br0.8Mo0.1Ba0.1', '-1.254', '-0.205504472042']
BaBrNa 1 ['Na0.333333Br0.5Ba0.166667', '-2.047', '-0.225416637885']
BaBrNp 6 ['Br0.714286Ba0.142857Np0.142857', '-2.189', '-0.239505171667']
BaBrO 41 ['O0.5Br0.125Ba0.375', '-2.551', '-0.202540005507']
BaBrOs 3 ['Br0.75Ba0.125Os0.125', '-1.127', '-0.214850482209']
BaBrP 17 ['P0.285714Br0.285714Ba0.428571', '-1.878', '-0.202119735588']
BaBrPb 1 ['Br0.7Ba0.2Pb0.1', '-2.005', '-0.210428876302']
BaBrPm 11 ['Br0.6Ba0.1Pm0.3', '-1.739', '-0.242194521791']
BaBrPt 19 ['Br0.333333Ba0.444444Pt0.222222', '-1.756', '-0.201004767389']
BaBrPu 11 ['Br0.6Ba0.1Pu0.3', '-1.898', '-0.221831249468']
BaBrRe 5 ['Br0.7Ba0.1Re0.2', '-0.987', '-0.257280385767']
BaBrRh 6 ['Br0.6Rh0.3Ba0.1', '-1.289', '-0.202491974801']
BaBrS 9 ['S0.222222Br0.222222Ba0.555556', '-2.003', '-0.203447709959']
BaBrSb 28 ['Br0.428571Sb0.142857Ba0.428571', '-2.131', '-0.205581607001']
BaBrSc 11 ['Sc0.1Br0.8Ba0.1', '-1.377', '-0.465179205525']
BaBrSe 22 ['Se0.375Br0.375Ba0.25', '-1.831', '-0.201505335812']
BaBrSi 20 ['Si0.125Br0.75Ba0.125', '-1.794', '-0.207230885377']
BaBrSn 3 ['Br0.4Sn0.2Ba0.4', '-1.967', '-0.208865764164']
BaBrTa 3 ['Br0.75Ba0.125Ta0.125', '-1.587', '-0.21530017765']
BaBrTb 8 ['Br0.666667Ba0.111111Tb0.222222', '-1.907', '-0.214030391282']
BaBrTe 36 ['Br0.625Te0.125Ba0.25', '-2.092', '-0.217101614944']
BaBrTi 1 ['Ti0.222222Br0.666667Ba0.111111', '-1.872', '-0.200394343291']
BaBrTm 9 ['Br0.7Ba0.2Tm0.1', '-2.211', '-0.20087512931']
BaBrV 3 ['V0.142857Br0.714286Ba0.142857', '-1.713', '-0.232468473261']
BaBrY 11 ['Br0.6Y0.3Ba0.1', '-1.771', '-0.2206873843']
BaBrYb 2 ['Br0.625Ba0.125Yb0.25', '-2.462', '-0.244484237553']
BaBrZn 5 ['Zn0.2Br0.6Ba0.2', '-1.999', '-0.229464888052']
BaBrZr 4 ['Br0.666667Zr0.111111Ba0.222222', '-2.346', '-0.23456294839']
BaCCl 5 ['C0.166667Cl0.666667Ba0.166667', '-1.659', '-0.256624513125']
BaCF 2 ['C0.111111F0.777778Ba0.111111', '-2.714', '-0.200344945306']

BaCH 5 ['H0.571429C0.285714Ba0.142857', '-0.412', '-0.201701111876']
BaCIr 2 ['C0.5Ba0.2Ir0.3', '-0.217', '-0.217']
BaCNi 13 ['C0.1Ni0.4Ba0.5', '-0.235', '-0.202216006667']
BaCNp 10 ['C0.6Ba0.3Np0.1', '-0.202', '-0.202']
BaCRh 10 ['C0.555556Rh0.222222Ba0.222222', '-0.241', '-0.215674134444']
BaCXe 10 ['C0.375Xe0.5Ba0.125', '-0.208', '-0.208']
BaCaF 5 ['F0.7Ca0.1Ba0.2', '-3.957', '-0.210546241524']
BaCaO 2 ['O0.625Ca0.25Ba0.125', '-2.63', '-0.221760266322']
BaCdF 5 ['F0.7Cd0.1Ba0.2', '-3.494', '-0.276637482524']
BaCdO 5 ['O0.6Cd0.2Ba0.2', '-1.977', '-0.21145129847']
BaCeF 2 ['F0.777778Ba0.111111Ce0.111111', '-3.597', '-0.275781646688']
BaCeI 2 ['I0.714286Ba0.142857Ce0.142857', '-1.779', '-0.334661013257']
BaCeO 6 ['O0.666667Ba0.111111Ce0.222222', '-2.917', '-0.255079112148']
BaClCu 1 ['Cl0.555556Cu0.333333Ba0.111111', '-1.55', '-0.232928133543']
BaClEr 11 ['Cl0.6Ba0.1Er0.3', '-1.968', '-0.331934472189']
BaClF 9 ['F0.3Cl0.4Ba0.3', '-3.372', '-0.214857900078']
BaClGa 1 ['Cl0.5Ga0.166667Ba0.333333', '-2.44', '-0.218640041354']
BaClGe 2 ['Cl0.666667Ge0.222222Ba0.111111', '-1.806', '-0.200972653194']
BaClH 1 ['H0.222222Cl0.333333Ba0.444444', '-1.916', '-0.318007685975']
BaClHo 11 ['Cl0.6Ba0.1Ho0.3', '-1.985', '-0.331313422187']
BaClIn 6 ['Cl0.666667In0.222222Ba0.111111', '-1.897', '-0.210654611615']
BaClIr 9 ['Cl0.666667Ba0.222222Ir0.111111', '-2.071', '-0.2011660175']
BaClLu 11 ['Cl0.6Ba0.1Lu0.3', '-2.005', '-0.419069804689']
BaClMg 1 ['Mg0.125Cl0.625Ba0.25', '-2.73', '-0.232236882787']
BaClMo 1 ['Cl0.7Mo0.2Ba0.1', '-1.913', '-0.268657872374']
BaClNa 6 ['Na0.2Cl0.5Ba0.3', '-2.32', '-0.201199081329']
BaClNp 2 ['Cl0.8Ba0.1Np0.1', '-2.015', '-0.23742825225']
BaClO 15 ['O0.166667Cl0.5Ba0.333333', '-2.837', '-0.20382089954']
BaClP 5 ['P0.166667Cl0.666667Ba0.166667', '-2.067', '-0.211769118867']
BaClPa 2 ['Cl0.7Ba0.2Pa0.1', '-2.676', '-0.224734739063']
BaClPm 6 ['Cl0.8Ba0.1Pm0.1', '-1.89', '-0.202887469939']
BaClPt 12 ['Cl0.666667Ba0.166667Pt0.166667', '-1.936', '-0.231344253474']
BaClPu 3 ['Cl0.75Ba0.125Pu0.125', '-2.483', '-0.343509922114']
BaClRh 13 ['Cl0.666667Rh0.111111Ba0.222222', '-2.083', '-0.2131660175']
BaClRu 1 ['Cl0.75Ru0.125Ba0.125', '-1.655', '-0.202557508987']
BaClS 1 ['S0.111111Cl0.777778Ba0.111111', '-1.298', '-0.224328424555']
BaClSb 1 ['Cl0.2Sb0.3Ba0.5', '-1.78', '-0.210724170375']
BaClSe 11 ['Cl0.3Se0.4Ba0.3', '-2.093', '-0.203813131811']
BaClSi 15 ['Si0.1Cl0.6Ba0.3', '-2.733', '-0.208724123625']
BaClSm 5 ['Cl0.8Ba0.1Sm0.1', '-1.914', '-0.223684292896']
BaClTa 1 ['Cl0.8Ba0.1Ta0.1', '-1.879', '-0.246179604625']
BaClTb 1 ['Cl0.75Ba0.125Tb0.125', '-2.444', '-0.204381864029']
BaClTc 1 ['Cl0.777778Tc0.111111Ba0.111111', '-1.689', '-0.225396971947']
BaClTe 31 ['Cl0.2Te0.4Ba0.4', '-2.129', '-0.213042350873']

BaClTh 1 ['Cl0.777778Ba0.111111Th0.111111', '-2.51', '-0.214532514583']
BaClV 1 ['Cl0.666667V0.222222Ba0.111111', '-2.05', '-0.206577479583']
BaClXe 1 ['Cl0.6Xe0.3Ba0.1', '-1.057', '-0.215574707875']
BaClZn 1 ['Cl0.6Zn0.1Ba0.3', '-2.736', '-0.211724123625']
BaCoF 3 ['F0.75Co0.125Ba0.125', '-2.833', '-0.309053470129']
BaCoH 3 ['H0.7Co0.2Ba0.1', '-0.348', '-0.200790778313']
BaCoI 4 ['Co0.222222I0.666667Ba0.111111', '-0.86', '-0.20201569088']
BaCoN 35 ['N0.571429Co0.142857Ba0.285714', '-0.756', '-0.200102978375']
BaCoO 4 ['O0.6Co0.1Ba0.3', '-2.374', '-0.22483101428']
BaCoP 4 ['P0.555556Co0.333333Ba0.111111', '-1.021', '-0.210974905855']
BaCrF 1 ['F0.8Cr0.1Ba0.1', '-2.992', '-0.295780923361']
BaCrN 27 ['N0.375Cr0.5Ba0.125', '-0.793', '-0.22193731876']
BaCrO 4 ['O0.428571Cr0.142857Ba0.428571', '-2.503', '-0.201340893194']
BaCsF 10 ['F0.625Cs0.25Ba0.125', '-3.293', '-0.306168787419']
BaCsO 3 ['O0.7Cs0.2Ba0.1', '-1.528', '-0.219795590134']
BaCsSe 1 ['Se0.5Cs0.2Ba0.3', '-1.809', '-0.2169658405']
BaCuF 6 ['F0.625Cu0.25Ba0.125', '-2.758', '-0.229272194605']
BaCuO 3 ['O0.666667Cu0.111111Ba0.222222', '-1.814', '-0.222621797099']
BaDyF 3 ['F0.75Ba0.125Dy0.125', '-4.09', '-0.475535532214']
BaDyI 8 ['I0.666667Ba0.166667Dy0.166667', '-1.584', '-0.23156056622']
BaDyO 6 ['O0.6Ba0.3Dy0.1', '-2.987', '-0.264426938055']
BaDyTe 1 ['Te0.6Ba0.2Dy0.2', '-1.651', '-0.208914159665']
BaErF 3 ['F0.75Ba0.125Er0.125', '-4.014', '-0.218362137417']
BaErI 9 ['I0.8Ba0.1Er0.1', '-1.089', '-0.28500403921']
BaErO 6 ['O0.6Ba0.3Er0.1', '-2.953', '-0.21767938072']
BaEuF 4 ['F0.75Ba0.125Eu0.125', '-3.516', '-0.253213455435']
BaEuN 4 ['N0.428571Ba0.142857Eu0.428571', '-0.727', '-0.214025253424']
BaEuO 6 ['O0.6Ba0.2Eu0.2', '-2.836', '-0.20632708847']
BaEuP 3 ['P0.444444Ba0.111111Eu0.444444', '-1.253', '-0.229705411715']
BaEuSe 1 ['Se0.4Ba0.1Eu0.5', '-2.022', '-0.256492484']
BaFFe 2 ['F0.777778Fe0.111111Ba0.111111', '-2.809', '-0.319956033259']
BaFGa 3 ['F0.75Ga0.125Ba0.125', '-3.318', '-0.281888659605']
BaFGd 4 ['F0.7Ba0.1Gd0.2', '-4.236', '-0.225467647028']
BaFGe 3 ['F0.7Ge0.1Ba0.2', '-3.769', '-0.203459411987']
BaFH 16 ['H0.444444F0.222222Ba0.333333', '-1.907', '-0.201739642001']
BaFHf 2 ['F0.777778Ba0.111111Hf0.111111', '-4.038', '-0.384401033361']
BaFHg 5 ['F0.75Ba0.125Hg0.125', '-2.411', '-0.239868977315']
BaFHo 3 ['F0.75Ba0.125Ho0.125', '-4.038', '-0.266444793879']
BaFIn 3 ['F0.75In0.125Ba0.125', '-3.251', '-0.283821853251']
BaFK 10 ['F0.6K0.3Ba0.1', '-3.244', '-0.255872419933']
BaFKr 7 ['F0.666667Kr0.222222Ba0.111111', '-1.815', '-0.228960905025']
BaFLa 3 ['F0.75Ba0.125La0.125', '-4.006', '-0.215988370129']
BaFLi 12 ['Li0.3F0.6Ba0.1', '-3.335', '-0.204758065433']
BaFLu 4 ['F0.714286Ba0.142857Lu0.142857', '-4.384', '-0.299986222764']

BaFMg 13 ['F0.571429Mg0.142857Ba0.285714', '-3.754', '-0.210240346214']
BaFMn 1 ['F0.8Mn0.1Ba0.1', '-2.79', '-0.30173643877']
BaFMo 2 ['F0.777778Mo0.111111Ba0.111111', '-3.374', '-0.234534471417']
BaFN 5 ['N0.142857F0.714286Ba0.142857', '-1.979', '-0.207120173107']
BaFNa 22 ['F0.428571Na0.428571Ba0.142857', '-2.845', '-0.223406975967']
BaFNb 3 ['F0.777778Nb0.111111Ba0.111111', '-3.786', '-0.237793942897']
BaFNd 2 ['F0.777778Ba0.111111Nd0.111111', '-3.7', '-0.369879277519']
BaFNi 2 ['F0.777778Ni0.111111Ba0.111111', '-2.627', '-0.228459279467']
BaFNp 2 ['F0.777778Ba0.111111Np0.111111', '-4.1', '-0.626016966089']
BaFO 15 ['O0.166667F0.5Ba0.333333', '-3.832', '-0.20159443279']
BaFOs 1 ['F0.8Ba0.1Os0.1', '-2.903', '-0.294526122443']
BaFP 13 ['F0.714286P0.142857Ba0.142857', '-3.446', '-0.206904542138']
BaFPa 5 ['F0.714286Ba0.142857Pa0.142857', '-4.044', '-0.21647707205']
BaFPb 6 ['F0.666667Ba0.166667Pb0.166667', '-3.608', '-0.211944933915']
BaFPd 4 ['F0.75Pd0.125Ba0.125', '-2.659', '-0.220974244406']
BaFPm 3 ['F0.777778Ba0.111111Pm0.111111', '-3.561', '-0.329866039097']
BaFPr 3 ['F0.75Ba0.125Pr0.125', '-4.084', '-0.341773017734']
BaFPt 6 ['F0.75Ba0.125Pt0.125', '-2.729', '-0.243405562531']
BaFPu 5 ['F0.714286Ba0.142857Pu0.142857', '-4.255', '-0.252882594193']
BaFRb 11 ['F0.6Rb0.3Ba0.1', '-3.158', '-0.203255128433']
BaFRe 2 ['F0.777778Ba0.111111Re0.111111', '-3.083', '-0.271981550994']
BaFRh 2 ['F0.777778Rh0.111111Ba0.111111', '-2.69', '-0.253513169467']
BaFRu 3 ['F0.75Ru0.125Ba0.125', '-3.037', '-0.294803553156']
BaFSb 10 ['F0.75Sb0.125Ba0.125', '-3.377', '-0.203646892527']
BaFSc 3 ['F0.75Sc0.125Ba0.125', '-4.035', '-0.349688346169']
BaFSe 18 ['F0.666667Se0.111111Ba0.222222', '-3.518', '-0.215516773361']
BaFSi 14 ['F0.444444Si0.222222Ba0.333333', '-3.095', '-0.219049592889']
BaFSm 3 ['F0.75Ba0.125Sm0.125', '-4.075', '-0.313621908984']
BaFSn 2 ['F0.777778Sn0.111111Ba0.111111', '-3.281', '-0.301034054267']
BaFSr 3 ['F0.75Sr0.125Ba0.125', '-3.42', '-0.292359780435']
BaFTa 1 ['F0.8Ba0.1Ta0.1', '-3.596', '-0.274070507609']
BaFTb 1 ['F0.777778Ba0.111111Tb0.111111', '-3.483', '-0.202393227466']
BaFTc 2 ['F0.777778Tc0.111111Ba0.111111', '-3.17', '-0.5503632135']
BaFTe 2 ['F0.777778Te0.111111Ba0.111111', '-3.193', '-0.253428507251']
BaFTh 2 ['F0.777778Ba0.111111Th0.111111', '-4.26', '-0.403886116322']
BaFTi 2 ['F0.777778Ti0.111111Ba0.111111', '-3.82', '-0.352213633493']
BaFTl 9 ['F0.555556Ba0.111111Tl0.333333', '-2.863', '-0.20337675437']
BaFTm 2 ['F0.777778Ba0.111111Tm0.111111', '-3.853', '-0.478382407151']
BaFV 2 ['F0.777778V0.111111Ba0.111111', '-3.459', '-0.415593725583']
BaFXe 3 ['F0.666667Xe0.166667Ba0.166667', '-2.664', '-0.237942693915']
BaFY 3 ['F0.75Y0.125Ba0.125', '-4.1', '-0.450547849084']
BaFYb 12 ['F0.75Ba0.125Yb0.125', '-3.389', '-0.243597906794']
BaFZn 3 ['F0.75Zn0.125Ba0.125', '-2.78', '-0.262389092398']
BaFZr 2 ['F0.777778Zr0.111111Ba0.111111', '-3.951', '-0.311157183911']

BaFeH 2 ['H0.714286Fe0.142857Ba0.142857', '-0.414', '-0.203320255492']
BaFeN 1 ['N0.666667Fe0.166667Ba0.166667', '-0.624', '-0.201802677479']
BaFeO 6 ['O0.5Fe0.25Ba0.25', '-2.329', '-0.218221901493']
BaFeP 1 ['P0.777778Fe0.111111Ba0.111111', '-0.925', '-0.227573200577']
BaFeSi 1 ['Si0.5Fe0.4Ba0.1', '-0.712', '-0.216374282']
BaGaH 7 ['H0.375Ga0.25Ba0.375', '-0.69', '-0.216193282149']
BaGaN 7 ['N0.285714Ga0.285714Ba0.428571', '-0.731', '-0.203809135497']
BaGaO 3 ['O0.333333Ga0.111111Ba0.555556', '-2.085', '-0.207260209072']
BaGaPt 3 ['Ga0.5Ba0.125Pt0.375', '-0.95', '-0.219307264375']
BaGaS 6 ['S0.5Ga0.3Ba0.2', '-1.603', '-0.207522645994']
BaGaSe 9 ['Ga0.2Se0.4Ba0.4', '-1.844', '-0.202178228']
BaGaTe 2 ['Ga0.25Te0.625Ba0.125', '-0.866', '-0.216214268416']
BaGdI 8 ['I0.8Ba0.1Gd0.1', '-1.135', '-0.307569875148']
BaGdO 7 ['O0.666667Ba0.222222Gd0.111111', '-2.68', '-0.214183330571']
BaGeH 10 ['H0.5Ge0.1Ba0.4', '-0.706', '-0.207674004408']
BaGeIr 6 ['Ge0.555556Ba0.111111Ir0.333333', '-0.636', '-0.205163578889']
BaGeO 1 ['O0.666667Ge0.222222Ba0.111111', '-2.586', '-0.553679505899']
BaGePa 2 ['Ge0.666667Ba0.111111Pa0.222222', '-0.549', '-0.231295162917']
BaGePt 5 ['Ge0.5Ba0.166667Pt0.333333', '-0.859', '-0.210741915208']
BaGeRh 3 ['Ge0.555556Rh0.333333Ba0.111111', '-0.769', '-0.208305118889']
BaGeRu 2 ['Ge0.5Ru0.4Ba0.1', '-0.637', '-0.23122273175']
BaGeSe 3 ['Ge0.3Se0.4Ba0.3', '-1.515', '-0.218300424416']
BaGeTe 4 ['Ge0.1Te0.7Ba0.2', '-0.946', '-0.204151588083']
BaHIn 6 ['H0.555556In0.222222Ba0.222222', '-0.558', '-0.212174881077']
BaHIr 3 ['H0.555556Ba0.333333Ir0.111111', '-0.641', '-0.208390206657']
BaHMn 6 ['H0.6Mn0.2Ba0.2', '-0.497', '-0.202581556626']
BaHN 5 ['H0.5N0.333333Ba0.166667', '-0.682', '-0.200190114575']
BaHO 19 ['H0.5O0.25Ba0.25', '-1.605', '-0.20211113556']
BaHP 12 ['H0.6P0.1Ba0.3', '-0.726', '-0.203695122558']
BaHPb 3 ['H0.5Ba0.333333Pb0.166667', '-0.694', '-0.211233844463']
BaHPd 2 ['H0.6Pd0.3Ba0.1', '-0.431', '-0.215929370017']
BaHPm 4 ['H0.6Ba0.1Pm0.3', '-0.577', '-0.231511406439']
BaHRh 5 ['H0.714286Rh0.142857Ba0.142857', '-0.414', '-0.203701111876']
BaHS 1 ['H0.166667S0.333333Ba0.5', '-1.832', '-0.226196570588']
BaHSb 11 ['H0.2Sb0.4Ba0.4', '-1.0', '-0.219068354998']
BaHSe 13 ['H0.3Se0.3Ba0.4', '-1.589', '-0.210424449313']
BaHSi 11 ['H0.4Si0.3Ba0.3', '-0.605', '-0.202857947876']
BaHSn 10 ['H0.5Sn0.125Ba0.375', '-0.763', '-0.208292566176']
BaHfO 2 ['O0.6Ba0.3Hf0.1', '-3.198', '-0.21292729797']
BaHfS 3 ['S0.5Ba0.166667Hf0.333333', '-1.975', '-0.23119145707']
BaHfSe 4 ['Se0.5Ba0.166667Hf0.333333', '-1.69', '-0.215652196667']
BaHgO 5 ['O0.666667Ba0.166667Hg0.166667', '-1.472', '-0.261297639009']
BaHgP 1 ['P0.3Ba0.4Hg0.3', '-1.013', '-0.206997633227']
BaHoI 8 ['I0.666667Ba0.166667Ho0.166667', '-1.573', '-0.233153621359']

BaHoO 7 ['O0.666667Ba0.222222Ho0.111111', '-2.728', '-0.239542157076']
BaIIn 3 ['In0.125I0.625Ba0.25', '-1.783', '-0.208863631604']
BaILi 4 ['Li0.3I0.5Ba0.2', '-1.677', '-0.266336884784']
BaILu 10 ['I0.625Ba0.125Lu0.25', '-1.281', '-0.286784581597']
BaIMg 3 ['Mg0.3I0.6Ba0.1', '-1.487', '-0.209172419957']
BaIN 4 ['N0.2I0.3Ba0.5', '-1.331', '-0.205909265993']
BaINd 3 ['I0.75Ba0.125Nd0.125', '-1.453', '-0.20393488222']
BaIO 19 ['O0.333333I0.333333Ba0.333333', '-2.326', '-0.208568588341']
BaIPa 6 ['I0.7Ba0.2Pa0.1', '-1.465', '-0.290988574283']
BaIPm 9 ['I0.666667Ba0.222222Pm0.111111', '-1.743', '-0.270555131148']
BaIPt 19 ['I0.5Ba0.375Pt0.125', '-1.824', '-0.200582716908']
BaIRh 11 ['Rh0.125I0.375Ba0.5', '-1.295', '-0.206120748306']
BaIRu 1 ['Ru0.2I0.7Ba0.1', '-0.78', '-0.206862158763']
BaIS 1 ['S0.25I0.25Ba0.5', '-2.05', '-0.221230889949']
BaISb 21 ['Sb0.285714I0.142857Ba0.571429', '-1.366', '-0.202872794355']
BaISc 2 ['Sc0.142857I0.714286Ba0.142857', '-1.561', '-0.214572372537']
BaISe 1 ['Se0.4I0.2Ba0.4', '-2.019', '-0.206918652762']
BaISm 6 ['I0.8Ba0.1Sm0.1', '-1.164', '-0.209000737285']
BaITb 9 ['I0.666667Ba0.222222Tb0.111111', '-1.729', '-0.361248687574']
BaITe 1 ['Te0.5I0.125Ba0.375', '-1.688', '-0.210484310683']
BaITi 1 ['Ti0.166667I0.666667Ba0.166667', '-1.623', '-0.256604830597']
BaITm 4 ['I0.7Ba0.2Tm0.1', '-1.739', '-0.217121078039']
BaIZn 1 ['Zn0.142857I0.428571Ba0.428571', '-1.487', '-0.205825383064']
BaInIr 1 ['In0.555556Ba0.111111Ir0.333333', '-0.494', '-0.224653153472']
BaInN 3 ['N0.5In0.375Ba0.125', '-0.531', '-0.250246708578']
BaInO 6 ['O0.375In0.125Ba0.5', '-2.327', '-0.20622746342']
BaInPt 2 ['In0.5Ba0.125Pt0.375', '-0.783', '-0.210216041562']
BaInSe 9 ['Se0.428571In0.285714Ba0.285714', '-1.528', '-0.201255996071']
BaIrN 12 ['N0.428571Ba0.285714Ir0.285714', '-0.64', '-0.2028429439']
BaIrP 11 ['P0.8Ba0.1Ir0.1', '-0.946', '-0.200098810144']
BaIrSi 4 ['Si0.428571Ba0.142857Ir0.428571', '-1.001', '-0.20084789']
BaKO 2 ['O0.666667K0.166667Ba0.166667', '-1.841', '-0.24090196191']
BaKS 1 ['S0.444444K0.111111Ba0.444444', '-2.253', '-0.275494562658']
BaLaO 5 ['O0.6Ba0.2La0.2', '-3.262', '-0.227505280973']
BaLaTe 1 ['Te0.6Ba0.2La0.2', '-1.768', '-0.210350669498']
BaLiO 4 ['Li0.2O0.4Ba0.4', '-2.416', '-0.210962259649']
BaLiSe 1 ['Li0.375Se0.375Ba0.25', '-1.738', '-0.234824727501']
BaLuO 4 ['O0.625Ba0.125Lu0.25', '-3.543', '-0.272554715697']
BaMgO 2 ['O0.625Mg0.125Ba0.25', '-2.561', '-0.235666538928']
BaMgS 10 ['Mg0.444444S0.444444Ba0.111111', '-1.757', '-0.209529012938']
BaMgSe 12 ['Mg0.25Se0.5Ba0.25', '-1.863', '-0.20296257646']
BaMnN 20 ['N0.333333Mn0.555556Ba0.111111', '-0.633', '-0.204802066022']
BaMoN 20 ['N0.4Mo0.4Ba0.2', '-0.629', '-0.206714673613']
BaMoO 1 ['O0.6Mo0.2Ba0.2', '-2.84', '-0.250802035141']

BaMoSe 3 ['Se0.5Mo0.333333Ba0.166667', '-1.226', '-0.20612736']
BaNNa 5 ['N0.375Na0.125Ba0.5', '-0.671', '-0.212373915156']
BaNNp 25 ['N0.4Ba0.4Np0.2', '-0.965', '-0.200469140111']
BaNO 16 ['N0.25O0.25Ba0.5', '-1.826', '-0.204750566778']
BaNOs 13 ['N0.6Ba0.1Os0.3', '-0.448', '-0.201524824794']
BaNP 41 ['N0.222222P0.333333Ba0.444444', '-1.156', '-0.200823540165']
BaNPa 20 ['N0.555556Ba0.333333Pa0.111111', '-0.918', '-0.203832498759']
BaNPt 8 ['N0.555556Ba0.111111Pt0.333333', '-0.505', '-0.203551767019']
BaNPu 9 ['N0.5Ba0.166667Pu0.333333', '-1.305', '-0.206508685385']
BaNRb 1 ['N0.75Rb0.125Ba0.125', '-0.616', '-0.210832985523']
BaNRe 22 ['N0.555556Ba0.333333Re0.111111', '-0.757', '-0.200806781291']
BaNRh 15 ['N0.5Rh0.2Ba0.3', '-0.717', '-0.216426103162']
BaNRu 3 ['N0.5Ru0.333333Ba0.166667', '-0.554', '-0.20809706337']
BaNSe 1 ['N0.1Se0.4Ba0.5', '-2.048', '-0.294732246299']
BaNTc 19 ['N0.571429Tc0.142857Ba0.285714', '-0.766', '-0.210102978375']
BaNTe 1 ['N0.1Te0.5Ba0.4', '-1.642', '-0.209290190664']
BaNTi 6 ['N0.5Ti0.25Ba0.25', '-1.345', '-0.246933137318']
BaNU 16 ['N0.428571Ba0.285714U0.285714', '-1.29', '-0.209495310564']
BaNV 1 ['N0.571429V0.285714Ba0.142857', '-1.246', '-0.21726843566']
BaNW 26 ['N0.428571Ba0.285714W0.285714', '-0.638', '-0.2008429439']
BaNaO 14 ['O0.7Na0.2Ba0.1', '-1.504', '-0.202323066714']
BaNaP 2 ['Na0.125P0.375Ba0.5', '-1.186', '-0.200233916222']
BaNaSe 12 ['Na0.142857Se0.428571Ba0.428571', '-1.962', '-0.202905244286']
BaNbO 2 ['O0.6Nb0.2Ba0.2', '-3.224', '-0.20752074247']
BaNbSe 8 ['Se0.6Nb0.1Ba0.3', '-1.7', '-0.201013902833']
BaNdO 6 ['O0.6Ba0.3Nd0.1', '-2.91', '-0.24319643922']
BaNiO 2 ['O0.6Ni0.2Ba0.2', '-2.014', '-0.246316693182']
BaNiSe 1 ['Ni0.125Se0.5Ba0.375', '-1.828', '-0.21438531125']
BaNiSi 2 ['Si0.6Ni0.3Ba0.1', '-0.669', '-0.25493308775']
BaNpO 13 ['O0.555556Ba0.333333Np0.111111', '-3.281', '-0.204650589427']
BaNpS 6 ['S0.5Ba0.1Np0.4', '-1.852', '-0.200257727494']
BaNpSe 13 ['Se0.666667Ba0.222222Np0.111111', '-1.442', '-0.207858348656']
BaOOs 6 ['O0.666667Ba0.166667Os0.166667', '-1.977', '-0.221422432331']
BaOP 24 ['O0.2P0.3Ba0.5', '-1.97', '-0.20830955156']
BaOPa 12 ['O0.7Ba0.2Pa0.1', '-2.435', '-0.223202406558']
BaOPd 9 ['O0.444444Pd0.333333Ba0.222222', '-1.794', '-0.223778715162']
BaOPm 2 ['O0.6Ba0.3Pm0.1', '-2.931', '-0.25012104972']
BaOPr 5 ['O0.6Ba0.1Pr0.3', '-3.482', '-0.212007935096']
BaOPt 6 ['O0.6Ba0.3Pt0.1', '-2.286', '-0.267235431219']
BaOPu 7 ['O0.6Ba0.1Pu0.3', '-3.753', '-0.28635918149']
BaORb 2 ['O0.4Rb0.3Ba0.3', '-2.142', '-0.205570369848']
BaORe 1 ['O0.625Ba0.125Re0.25', '-2.333', '-0.234796676849']
BaORh 3 ['O0.666667Rh0.166667Ba0.166667', '-1.825', '-0.24686774108']
BaOS 2 ['O0.555556S0.111111Ba0.333333', '-2.845', '-0.244881041846']

BaOSb 17 ['O0.285714Sb0.142857Ba0.571429', '-2.138', '-0.207330761177']
BaOSc 7 ['O0.5Sc0.125Ba0.375', '-3.108', '-0.219184964559']
BaOSe 26 ['O0.571429Se0.142857Ba0.285714', '-2.507', '-0.206964129496']
BaOSi 11 ['O0.3Si0.2Ba0.5', '-2.072', '-0.274890803361']
BaOSm 5 ['O0.625Ba0.125Sm0.25', '-3.369', '-0.261431985541']
BaOSn 1 ['O0.6Sn0.2Ba0.2', '-2.684', '-0.2414133711']
BaOTb 7 ['O0.6Ba0.3Tb0.1', '-2.854', '-0.200972391252']
BaOTc 8 ['O0.7Tc0.2Ba0.1', '-2.09', '-0.201618834582']
BaOTe 2 ['O0.375Te0.25Ba0.375', '-2.402', '-0.223187103629']
BaOTh 5 ['O0.6Ba0.2Th0.2', '-3.827', '-0.20674900772']
BaOTi 2 ['O0.444444Ti0.111111Ba0.444444', '-2.732', '-0.237344357803']
BaOTl 12 ['O0.666667Ba0.222222Tl0.111111', '-1.912', '-0.239342835016']
BaOTm 10 ['O0.6Ba0.1Tm0.3', '-3.763', '-0.225389491846']
BaOU 1 ['O0.6Ba0.2U0.2', '-3.696', '-0.23591049047']
BaOY 5 ['O0.6Y0.1Ba0.3', '-2.922', '-0.206364038722']
BaOZn 2 ['O0.444444Zn0.111111Ba0.444444', '-2.61', '-0.217361190045']
BaOZr 1 ['O0.6Zr0.2Ba0.2', '-3.712', '-0.23504319047']
BaOsP 8 ['P0.571429Ba0.142857Os0.285714', '-1.006', '-0.212835450594']
BaPPm 6 ['P0.375Ba0.125Pm0.5', '-1.115', '-0.201638709765']
BaPPt 2 ['P0.428571Ba0.285714Pt0.285714', '-1.326', '-0.219919882285']
BaPRh 8 ['P0.625Rh0.25Ba0.125', '-1.108', '-0.204213837525']
BaPSe 3 ['P0.2Se0.3Ba0.5', '-1.879', '-0.204237100145']
BaPSn 2 ['P0.666667Sn0.166667Ba0.166667', '-0.866', '-0.226561537093']
BaPTa 1 ['P0.5Ba0.1Ta0.4', '-1.067', '-0.23180205602']
BaPTe 4 ['P0.166667Te0.5Ba0.333333', '-1.452', '-0.21544936538']
BaPaS 9 ['S0.571429Ba0.142857Pa0.285714', '-2.008', '-0.204464228419']
BaPaSe 15 ['Se0.7Ba0.2Pa0.1', '-1.323', '-0.246129688998']
BaPaSi 4 ['Si0.75Ba0.125Pa0.125', '-0.42', '-0.208310864688']
BaPdSb 1 ['Pd0.3Sb0.4Ba0.3', '-1.077', '-0.21066385535']
BaPdSe 2 ['Se0.4Pd0.1Ba0.5', '-1.953', '-0.2032503815']
BaPmS 14 ['S0.4Ba0.2Pm0.4', '-1.848', '-0.209982887892']
BaPmSe 14 ['Se0.5Ba0.125Pm0.375', '-1.932', '-0.203195545312']
BaPmTe 4 ['Te0.625Ba0.125Pm0.25', '-1.38', '-0.279749535622']
BaPrSe 1 ['Se0.5Ba0.1Pr0.4', '-2.13', '-0.24076305']
BaPtS 1 ['S0.4Ba0.3Pt0.3', '-1.713', '-0.263211042142']
BaPtSe 6 ['Se0.5Ba0.4Pt0.1', '-1.912', '-0.204706050583']
BaPtSi 4 ['Si0.5Ba0.125Pt0.375', '-0.908', '-0.206163851563']
BaPuS 3 ['S0.571429Ba0.142857Pu0.285714', '-2.156', '-0.209998668419']
BaPuSe 2 ['Se0.6Ba0.1Pu0.3', '-1.722', '-0.227808467998']
BaRbS 1 ['S0.5Rb0.1Ba0.4', '-2.206', '-0.213882148324']
BaRbSe 1 ['Se0.5Rb0.125Ba0.375', '-2.007', '-0.23254101875']
BaRhS 1 ['S0.444444Rh0.222222Ba0.333333', '-1.854', '-0.244848148492']
BaRhSe 2 ['Se0.555556Rh0.333333Ba0.111111', '-0.942', '-0.213717801667']
BaRhSi 6 ['Si0.666667Rh0.166667Ba0.166667', '-0.673', '-0.214782585']

BaRuSi 1 ['Si0.5Ru0.375Ba0.125', '-0.811', '-0.22630119125']
BaSSb 4 ['S0.3Sb0.2Ba0.5', '-1.927', '-0.201718007293']
BaSSe 33 ['S0.333333Se0.222222Ba0.444444', '-2.149', '-0.201390233103']
BaSSi 15 ['Si0.1S0.3Ba0.6', '-1.631', '-0.203112065044']
BaSTc 4 ['S0.5Tc0.4Ba0.1', '-1.19', '-0.260791826993']
BaSTe 2 ['S0.1Te0.5Ba0.4', '-1.724', '-0.204528919596']
BaSZr 4 ['S0.444444Zr0.444444Ba0.111111', '-1.757', '-0.224157433212']
BaSbSe 20 ['Se0.111111Sb0.444444Ba0.444444', '-1.334', '-0.203238088332']
BaScSe 10 ['Sc0.111111Se0.555556Ba0.333333', '-1.946', '-0.215171270554']
BaSeSi 21 ['Si0.111111Se0.333333Ba0.555556', '-1.696', '-0.224402118056']
BaSeSn 1 ['Se0.428571Sn0.142857Ba0.428571', '-1.977', '-0.217905244286']
BaSeTa 11 ['Se0.625Ba0.125Ta0.25', '-1.332', '-0.20576845875']
BaSeTb 1 ['Se0.625Ba0.125Tb0.25', '-1.776', '-0.242025904455']
BaSeTc 6 ['Se0.666667Tc0.222222Ba0.111111', '-0.7', '-0.23551931111']
BaSeTe 7 ['Se0.285714Te0.285714Ba0.428571', '-1.89', '-0.205588088094']
BaSeTh 1 ['Se0.6Ba0.1Th0.3', '-2.008', '-0.244077765998']
BaSeV 1 ['V0.1Se0.5Ba0.4', '-1.968', '-0.200036172']
BaSeY 1 ['Se0.6Y0.3Ba0.1', '-1.97', '-0.222937304001']
BaSeZr 9 ['Se0.625Zr0.25Ba0.125', '-1.715', '-0.20107390625']
BaSmTe 1 ['Te0.6Ba0.2Sm0.2', '-1.688', '-0.225317047499']
BaTeTh 1 ['Te0.625Ba0.25Th0.125', '-1.558', '-0.245093183809']
BaTeY 1 ['Y0.3Te0.6Ba0.1', '-1.653', '-0.211820431003']
BeBiF 2 ['Be0.111111F0.777778Bi0.111111', '-2.749', '-0.2240644179']
BeBrCe 1 ['Be0.125Br0.75Ce0.125', '-1.758', '-0.208415274426']
BeBrCr 2 ['Be0.125Cr0.125Br0.75', '-1.053', '-0.265525300042']
BeBrDy 11 ['Be0.222222Br0.555556Dy0.222222', '-1.538', '-0.202219419559']
BeBrEr 23 ['Be0.222222Br0.444444Er0.333333', '-1.147', '-0.20573823837']
BeBrEu 1 ['Be0.111111Br0.555556Eu0.333333', '-2.271', '-0.240011663127']
BeBrF 9 ['Be0.1F0.7Br0.2', '-2.05', '-0.200705950207']
BeBrGd 2 ['Be0.166667Br0.5Gd0.333333', '-1.681', '-0.219925194367']
BeBrHf 14 ['Be0.2Br0.6Hf0.2', '-1.109', '-0.231151256551']
BeBrHo 16 ['Be0.2Br0.5Ho0.3', '-1.253', '-0.209406539628']
BeBrIr 3 ['Be0.1Br0.8Ir0.1', '-0.796', '-0.213301853792']
BeBrLa 4 ['Be0.142857Br0.571429La0.285714', '-1.964', '-0.203913438828']
BeBrLu 23 ['Be0.1Br0.5Lu0.4', '-1.198', '-0.223951186681']
BeBrMo 1 ['Be0.1Br0.8Mo0.1', '-0.895', '-0.201051873792']
BeBrNp 5 ['Be0.1Br0.7Np0.2', '-1.66', '-0.202231116369']
BeBrOs 3 ['Be0.111111Br0.777778Os0.111111', '-0.689', '-0.27214198613']
BeBrPm 19 ['Be0.25Br0.5Pm0.25', '-1.398', '-0.289796514416']
BeBrPr 2 ['Be0.2Br0.7Pr0.1', '-1.82', '-0.211205666058']
BeBrPt 10 ['Be0.111111Br0.777778Pt0.111111', '-0.623', '-0.20614198613']
BeBrPu 19 ['Be0.25Br0.625Pu0.125', '-1.577', '-0.220182476961']
BeBrRe 3 ['Be0.125Br0.75Re0.125', '-0.722', '-0.253034734396']
BeBrSb 2 ['Be0.1Br0.8Sb0.1', '-0.92', '-0.211636124042']

BeBrSc 26 ['Be0.285714Sc0.285714Br0.428571', '-1.045', '-0.21010730851']
BeBrSi 1 ['Be0.166667Si0.166667Br0.666667', '-1.309', '-0.233966581308']
BeBrSm 7 ['Be0.111111Br0.777778Sm0.111111', '-1.561', '-0.201106730601']
BeBrTa 2 ['Be0.111111Br0.777778Ta0.111111', '-1.101', '-0.275652826523']
BeBrTb 10 ['Be0.222222Br0.666667Tb0.111111', '-1.499', '-0.224198953586']
BeBrTe 4 ['Be0.142857Br0.714286Te0.142857', '-0.861', '-0.267211868483']
BeBrTh 2 ['Be0.166667Br0.666667Th0.166667', '-1.863', '-0.204763559225']
BeBrTm 23 ['Be0.222222Br0.444444Tm0.333333', '-1.154', '-0.233344982197']
BeBrV 4 ['Be0.142857V0.142857Br0.714286', '-1.188', '-0.213964761476']
BeBrY 25 ['Be0.2Br0.4Y0.4', '-1.162', '-0.225379981699']
BeBrYb 8 ['Be0.166667Br0.5Yb0.333333', '-1.983', '-0.223521085459']
BeBrZr 5 ['Be0.125Br0.75Zr0.125', '-1.495', '-0.213219662241']
BeCCa 3 ['Be0.222222C0.555556Ca0.222222', '-0.286', '-0.208045935833']
BeCLu 2 ['Be0.2C0.4Lu0.4', '-0.452', '-0.207294438407']
BeCNp 10 ['Be0.111111C0.555556Np0.333333', '-0.252', '-0.213022967917']
BeCOs 2 ['Be0.571429C0.142857Os0.285714', '-0.333', '-0.210854868929']
BeCP 9 ['Be0.375C0.125P0.5', '-0.61', '-0.200129757239']
BeCPa 9 ['Be0.111111C0.444444Pa0.444444', '-0.625', '-0.209314291111']
BeCPm 1 ['Be0.166667C0.5Pm0.333333', '-0.265', '-0.206534451875']
BeCPt 6 ['Be0.5C0.2Pt0.3', '-0.75', '-0.23273909625']
BeCPu 1 ['Be0.2C0.5Pu0.3', '-0.306', '-0.215534690917']
BeCRe 3 ['Be0.125C0.375Re0.5', '-0.307', '-0.217246648203']
BeCRu 4 ['Be0.5C0.125Ru0.375', '-0.4', '-0.210832931667']
BeCSe 6 ['Be0.375C0.25Se0.375', '-0.799', '-0.20005363375']
BeCSr 1 ['Be0.1C0.6Sr0.3', '-0.244', '-0.208920671125']
BeCTm 1 ['Be0.166667C0.5Tm0.333333', '-0.475', '-0.212648921758']
BeCaF 7 ['Be0.222222F0.666667Ca0.111111', '-3.939', '-0.213726377247']
BeCdF 7 ['Be0.166667F0.666667Cd0.166667', '-3.193', '-0.22601185058']
BeCeF 3 ['Be0.125F0.75Ce0.125', '-3.74', '-0.249696538773']
BeCeI 9 ['Be0.222222I0.666667Ce0.111111', '-1.003', '-0.221499296571']
BeCeN 1 ['Be0.5N0.4Ce0.1', '-1.388', '-0.248702243736']
BeCeO 2 ['Be0.125O0.625Ce0.25', '-3.245', '-0.303210349402']
BeClEr 18 ['Be0.25Cl0.625Er0.125', '-1.791', '-0.210990363987']
BeClEu 2 ['Be0.111111Cl0.666667Eu0.222222', '-2.661', '-0.224901324587']
BeClF 3 ['Be0.125F0.75Cl0.125', '-1.985', '-0.202359983591']
BeClHo 14 ['Be0.142857Cl0.571429Ho0.285714', '-1.694', '-0.20675523012']
BeClIn 1 ['Be0.142857Cl0.714286In0.142857', '-1.418', '-0.221276294522']
BeClLu 15 ['Be0.1Cl0.5Lu0.4', '-1.485', '-0.233285276727']
BeClNp 2 ['Be0.111111Cl0.666667Np0.222222', '-2.001', '-0.223768257108']
BeClPa 1 ['Be0.166667Cl0.666667Pa0.166667', '-1.963', '-0.233070715097']
BeClPm 10 ['Be0.166667Cl0.5Pm0.333333', '-1.689', '-0.213145648023']
BeClPt 1 ['Be0.1Cl0.7Pt0.2', '-1.099', '-0.236699873295']
BeClPu 15 ['Be0.2Cl0.7Pu0.1', '-2.072', '-0.202705599568']
BeClRb 1 ['Be0.222222Cl0.666667Rb0.111111', '-1.784', '-0.200100453544']

BeClRh 8 ['Be0.2Cl0.7Rh0.1', '-1.273', '-0.273872369752']
BeClSi 1 ['Be0.1Si0.2Cl0.7', '-1.679', '-0.225029091439']
BeClSm 5 ['Be0.111111Cl0.666667Sm0.222222', '-2.161', '-0.2187108607']
BeCoF 9 ['Be0.222222F0.666667Co0.111111', '-3.136', '-0.240275752185']
BeCoN 21 ['Be0.166667N0.333333Co0.5', '-0.532', '-0.20195561301']
BeCoOs 2 ['Be0.7Co0.1Os0.2', '-0.331', '-0.2113295695']
BeCoP 26 ['Be0.222222P0.333333Co0.444444', '-0.771', '-0.201257186643']
BeCoPt 1 ['Be0.555556Co0.111111Pt0.333333', '-0.849', '-0.217731980185']
BeCrF 2 ['Be0.2F0.7Cr0.1', '-3.445', '-0.204784490108']
BeCrN 9 ['Be0.125N0.5Cr0.375', '-0.87', '-0.203440497811']
BeCsF 11 ['Be0.1F0.6Cs0.3', '-3.084', '-0.214798560226']
BeCuF 5 ['Be0.1F0.7Cu0.2', '-2.321', '-0.239331945524']
BeDyF 5 ['Be0.25F0.625Dy0.125', '-3.584', '-0.200906410747']
BeDyI 10 ['Be0.1I0.8Dy0.1', '-0.703', '-0.257623281483']
BeDyN 1 ['Be0.1N0.4Dy0.5', '-1.811', '-0.34441537841']
BeErF 3 ['Be0.125F0.75Er0.125', '-3.791', '-0.265809114607']
BeErI 10 ['Be0.1I0.8Er0.1', '-0.708', '-0.270090980962']
BeErN 7 ['Be0.285714N0.428571Er0.285714', '-1.716', '-0.216445124206']
BeEuF 4 ['Be0.142857F0.714286Eu0.142857', '-3.688', '-0.240320733353']
BeEuN 14 ['Be0.222222N0.333333Eu0.444444', '-0.857', '-0.214138384236']
BeEuO 1 ['Be0.2O0.6Eu0.2', '-2.926', '-0.368889606091']
BeEuP 6 ['Be0.111111P0.333333Eu0.555556', '-0.958', '-0.201531314259']
BeEuSe 4 ['Be0.25Se0.5Eu0.25', '-1.709', '-0.206260225']
BeFFe 5 ['Be0.1F0.7Fe0.2', '-2.893', '-0.234735142608']
BeFGa 4 ['Be0.142857F0.714286Ga0.142857', '-3.411', '-0.279067749906']
BeFGd 4 ['Be0.1F0.7Gd0.2', '-4.138', '-0.286653560163']
BeFGe 5 ['Be0.2F0.7Ge0.1', '-3.292', '-0.213465864984']
BeFH 11 ['H0.3Be0.1F0.6', '-2.243', '-0.224931357278']
BeFHf 1 ['Be0.1F0.8Hf0.1', '-3.412', '-0.320614679024']
BeFHg 6 ['Be0.166667F0.666667Hg0.166667', '-2.784', '-0.217248218085']
BeFHo 3 ['Be0.125F0.75Ho0.125', '-3.748', '-0.222511980127']
BeFI 1 ['Be0.125F0.75I0.125', '-2.544', '-0.223580628785']
BeFIn 4 ['Be0.142857F0.714286In0.142857', '-3.283', '-0.205171157049']
BeFK 11 ['Be0.1F0.6K0.3', '-3.105', '-0.219010172436']
BeFKr 2 ['Be0.111111F0.777778Kr0.111111', '-1.548', '-0.284642463164']
BeFLa 3 ['Be0.125F0.75La0.125', '-3.965', '-0.421055556377']
BeFLi 11 ['Li0.3Be0.1F0.6', '-3.217', '-0.255886134102']
BeFLu 9 ['Be0.222222F0.555556Lu0.222222', '-3.261', '-0.22132848551']
BeFMg 6 ['Be0.1F0.7Mg0.2', '-3.589', '-0.251633648686']
BeFMn 1 ['Be0.1F0.8Mn0.1', '-2.523', '-0.231590187769']
BeFN 5 ['Be0.2N0.1F0.7', '-2.315', '-0.228075740348']
BeFNa 14 ['Be0.2F0.5Na0.3', '-3.081', '-0.200309758676']
BeFNb 1 ['Be0.1F0.8Nb0.1', '-3.305', '-0.308468297606']
BeFNd 3 ['Be0.125F0.75Nd0.125', '-3.853', '-0.352681373458']

BeFNi 10 ['Be0.25F0.625Ni0.125', '-3.179', '-0.209386843044']
BeFNp 5 ['Be0.2F0.7Np0.1', '-3.809', '-0.236297901732']
BeFO 14 ['Be0.333333O0.111111F0.555556', '-3.431', '-0.202915214046']
BeFP 2 ['Be0.2F0.7P0.1', '-3.358', '-0.24402479867']
BeFPa 4 ['Be0.222222F0.666667Pa0.111111', '-3.591', '-0.20630625169']
BeFPb 6 ['Be0.125F0.75Pb0.125', '-2.845', '-0.211337459405']
BeFPd 2 ['Be0.111111F0.777778Pd0.111111', '-2.218', '-0.269592382804']
BeFPm 4 ['Be0.125F0.75Pm0.125', '-3.603', '-0.214041480233']
BeFPr 3 ['Be0.125F0.75Pr0.125', '-3.749', '-0.252840203982']
BeFPt 6 ['Be0.222222F0.666667Pt0.111111', '-2.976', '-0.241551005303']
BeFPu 13 ['Be0.125F0.625Pu0.25', '-3.497', '-0.229807662561']
BeFRb 10 ['Be0.222222F0.666667Rb0.111111', '-3.356', '-0.317806301591']
BeFRe 1 ['Be0.111111F0.777778Re0.111111', '-2.821', '-0.228707938771']
BeFRh 5 ['Be0.142857F0.714286Rh0.142857', '-2.754', '-0.237981072049']
BeFRu 4 ['Be0.142857F0.714286Ru0.142857', '-2.786', '-0.24983214562']
BeFSb 3 ['Be0.125F0.75Sb0.125', '-3.094', '-0.317864979033']
BeFSc 4 ['Be0.166667F0.666667Sc0.166667', '-3.943', '-0.221861952877']
BeFSe 1 ['Be0.1F0.8Se0.1', '-2.245', '-0.218331577524']
BeFSi 5 ['Be0.125F0.75Si0.125', '-3.625', '-0.23003983878']
BeFSm 3 ['Be0.1F0.8Sm0.1', '-3.026', '-0.213751276186']
BeFSn 3 ['Be0.2F0.7Sn0.1', '-3.374', '-0.206067105424']
BeFSr 7 ['Be0.2F0.7Sr0.1', '-3.577', '-0.206208315274']
BeFTa 2 ['Be0.2F0.7Ta0.1', '-3.545', '-0.209107572208']
BeFTb 2 ['Be0.1F0.8Tb0.1', '-2.964', '-0.208307653718']
BeFTc 2 ['Be0.111111F0.777778Tc0.111111', '-2.857', '-0.456089601277']
BeFTe 1 ['Be0.125F0.75Te0.125', '-3.015', '-0.231911471486']
BeFTh 2 ['Be0.1F0.8Th0.1', '-3.595', '-0.321351253689']
BeFTi 3 ['Be0.125F0.75Ti0.125', '-3.651', '-0.542484255073']
BeFTl 8 ['Be0.111111F0.666667Tl0.222222', '-2.746', '-0.205550415577']
BeFTm 25 ['Be0.111111F0.444444Tm0.444444', '-2.467', '-0.20879535261']
BeFU 3 ['Be0.111111F0.777778U0.111111', '-3.896', '-0.2271989479']
BeFV 4 ['Be0.111111F0.666667V0.222222', '-3.28', '-0.282766284282']
BeFXe 1 ['Be0.1F0.8Xe0.1', '-1.693', '-0.272650592522']
BeFY 11 ['Be0.222222F0.666667Y0.111111', '-3.835', '-0.22454691446']
BeFYb 22 ['Be0.1F0.7Yb0.2', '-3.821', '-0.20128772511']
BeFZn 5 ['Be0.142857F0.714286Zn0.142857', '-2.825', '-0.234148120499']
BeFZr 2 ['Be0.111111F0.777778Zr0.111111', '-3.613', '-0.253063415999']
BeFeN 10 ['Be0.222222N0.333333Fe0.444444', '-0.732', '-0.212156974329']
BeFeP 31 ['Be0.428571P0.285714Fe0.285714', '-0.647', '-0.206052469251']
BeGdI 12 ['Be0.2I0.6Gd0.2', '-1.029', '-0.204266532514']
BeGdO 2 ['Be0.2O0.6Gd0.2', '-3.346', '-0.263202240559']
BeHPm 22 ['H0.428571Be0.285714Pm0.285714', '-0.413', '-0.20055781585']
BeHPu 14 ['H0.571429Be0.142857Pu0.285714', '-0.51', '-0.203467351225']
BeHRh 1 ['H0.666667Be0.222222Rh0.111111', '-0.378', '-0.220503287222']

BeHY 2 ['H0.666667Be0.111111Y0.222222', '-0.74', '-0.202057201469']
BeHfN 17 ['Be0.428571N0.285714Hf0.285714', '-1.327', '-0.211190887608']
BeHfS 9 ['Be0.111111S0.555556Hf0.333333', '-1.74', '-0.217077564567']
BeHfSe 6 ['Be0.111111Se0.555556Hf0.333333', '-1.386', '-0.206434271471']
BeHoI 10 ['Be0.125I0.625Ho0.25', '-1.037', '-0.201261410774']
BeHoN 10 ['Be0.428571N0.428571Ho0.142857', '-1.557', '-0.202199143839']
BeHoSe 1 ['Be0.2Se0.5Ho0.3', '-1.706', '-0.206410642054']
BeILu 11 ['Be0.1I0.8Lu0.1', '-0.568', '-0.249831853772']
BeINd 4 ['Be0.125I0.75Nd0.125', '-0.995', '-0.203543559409']
BeINi 1 ['Be0.142857Ni0.285714I0.571429', '-0.611', '-0.221483632898']
BeIPa 10 ['Be0.125I0.625Pa0.25', '-0.533', '-0.204847017536']
BeIPm 11 ['Be0.1I0.8Pm0.1', '-0.658', '-0.272024401022']
BeIPt 5 ['Be0.142857I0.571429Pt0.285714', '-0.633', '-0.207724332184']
BeIRh 2 ['Be0.125Rh0.25I0.625', '-0.478', '-0.219186375643']
BeISe 4 ['Be0.142857Se0.285714I0.571429', '-0.5', '-0.204213000735']
BeITb 14 ['Be0.125I0.5Tb0.375', '-0.693', '-0.222435128869']
BeITm 2 ['Be0.111111I0.666667Tm0.222222', '-1.299', '-0.234269579489']
BeIXe 1 ['Be0.1I0.5Xe0.4', '-0.413', '-0.205949100514']
BeIrOs 5 ['Be0.666667Os0.166667Ir0.166667', '-0.455', '-0.201630983333']
BeIrP 3 ['Be0.375P0.25Ir0.375', '-0.837', '-0.203776885885']
BeIrSi 1 ['Be0.5Si0.1Ir0.4', '-0.742', '-0.2238172255']
BeIrTa 1 ['Be0.5Ta0.1Ir0.4', '-0.703', '-0.202277050167']
BeIrTi 1 ['Be0.5Ti0.1Ir0.4', '-0.785', '-0.201863070455']
BeIrZr 5 ['Be0.555556Zr0.111111Ir0.333333', '-0.7', '-0.20143926671']
BeKP 9 ['Be0.3P0.5K0.2', '-0.669', '-0.206163031145']
BeKSe 5 ['Be0.3K0.3Se0.4', '-1.105', '-0.2058861085']
BeKrOs 1 ['Be0.625Kr0.125Os0.25', '-0.22', '-0.2008213325']
BeKrP 14 ['Be0.2P0.5Kr0.3', '-0.495', '-0.202183134666']
BeLaN 4 ['Be0.2N0.4La0.4', '-1.373', '-0.205703236183']
BeLaO 2 ['Be0.2O0.6La0.2', '-3.405', '-0.362246999684']
BeLaSe 1 ['Be0.1Se0.4La0.5', '-1.839', '-0.224815495346']
BeLiOs 1 ['Li0.1Be0.6Os0.3', '-0.225', '-0.201985599']
BeLiP 2 ['Li0.4Be0.2P0.4', '-0.904', '-0.240728292666']
BeLiPt 1 ['Li0.125Be0.5Pt0.375', '-0.835', '-0.205546419063']
BeLuN 1 ['Be0.125N0.375Lu0.5', '-1.795', '-0.28344984718']
BeMgOs 1 ['Be0.6Mg0.1Os0.3', '-0.268', '-0.244985599']
BeMnN 5 ['Be0.285714N0.428571Mn0.285714', '-0.919', '-0.201437427816']
BeMnP 1 ['Be0.285714P0.428571Mn0.285714', '-0.763', '-0.218621702321']
BeMoN 4 ['Be0.125N0.375Mo0.5', '-0.767', '-0.251224593152']
BeMoP 1 ['Be0.2P0.4Mo0.4', '-0.846', '-0.20786367087']
BeNNb 7 ['Be0.3N0.4Nb0.3', '-1.3', '-0.200047616487']
BeNNd 8 ['Be0.444444N0.333333Nd0.222222', '-1.219', '-0.205039992821']
BeNNp 22 ['Be0.25N0.375Np0.375', '-1.233', '-0.245586284608']
BeNP 6 ['Be0.4N0.4P0.2', '-1.129', '-0.241101760194']

BeNPa 27 ['Be0.3N0.4Pa0.3', '-1.395', '-0.223565632985']
BeNPm 11 ['Be0.333333N0.444444Pm0.222222', '-1.533', '-0.204906791097']
BeNPt 21 ['Be0.571429N0.142857Pt0.285714', '-1.063', '-0.200909262896']
BeNPu 16 ['Be0.1N0.6Pu0.3', '-1.263', '-0.24863750525']
BeNRh 1 ['Be0.5N0.375Rh0.125', '-1.184', '-0.219553579258']
BeNSc 10 ['Be0.428571N0.428571Sc0.142857', '-1.613', '-0.209018972173']
BeNSm 5 ['Be0.285714N0.428571Sm0.285714', '-1.523', '-0.203111510299']
BeNTa 1 ['Be0.1N0.4Ta0.5', '-1.33', '-0.214117321293']
BeNTh 13 ['Be0.5N0.375Th0.125', '-1.359', '-0.202013702424']
BeNTi 19 ['Be0.5N0.375Ti0.125', '-1.38', '-0.203788619696']
BeNTm 1 ['Be0.1N0.4Tm0.5', '-1.77', '-0.206967338441']
BeNU 9 ['Be0.111111N0.555556U0.333333', '-1.668', '-0.228278291519']
BeNV 10 ['Be0.222222N0.444444V0.333333', '-1.401', '-0.209127650557']
BeNW 10 ['Be0.285714N0.428571W0.285714', '-0.842', '-0.206203529732']
BeNY 8 ['Be0.333333N0.333333Y0.333333', '-1.462', '-0.221866332142']
BeNYb 1 ['Be0.5N0.4Yb0.1', '-1.356', '-0.216989118902']
BeNZr 15 ['Be0.25N0.5Zr0.25', '-1.619', '-0.20589317139']
BeNdO 1 ['Be0.2O0.6Nd0.2', '-3.307', '-0.247011951474']
BeNdSe 1 ['Be0.125Se0.5Nd0.375', '-1.829', '-0.201309547702']
BeNiOs 3 ['Be0.625Ni0.125Os0.25', '-0.374', '-0.21279626125']
BeNiP 24 ['Be0.111111P0.444444Ni0.444444', '-0.762', '-0.20092291709']
BeNpO 2 ['Be0.111111O0.333333Np0.555556', '-2.152', '-0.203651644599']
BeNpOs 1 ['Be0.666667Os0.222222Np0.111111', '-0.273', '-0.228015001068']
BeNpP 8 ['Be0.222222P0.444444Np0.333333', '-0.968', '-0.216729203726']
BeNpS 1 ['Be0.166667S0.5Np0.333333', '-1.617', '-0.209992760077']
BeNpSe 20 ['Be0.1Se0.5Np0.4', '-1.243', '-0.202211728539']
BeOPa 3 ['Be0.1O0.7Pa0.2', '-2.806', '-0.35347792706']
BeOsP 3 ['Be0.222222P0.444444Os0.333333', '-0.675', '-0.20706844824']
BeOsRh 5 ['Be0.6Rh0.1Os0.3', '-0.396', '-0.212698866']
BeOsRu 2 ['Be0.666667Ru0.111111Os0.222222', '-0.376', '-0.232840916667']
BeOsSc 3 ['Be0.1Sc0.6Os0.3', '-0.424', '-0.20954951']
BeOsTc 11 ['Be0.666667Tc0.222222Os0.111111', '-0.237', '-0.228476147778']
BeOsTi 3 ['Be0.625Ti0.125Os0.25', '-0.432', '-0.212325521172']
BeOsV 2 ['Be0.666667V0.111111Os0.222222', '-0.343', '-0.220103896667']
BeOsXe 1 ['Be0.666667Xe0.111111Os0.222222', '-0.218', '-0.200952295556']
BePPm 8 ['Be0.142857P0.428571Pm0.428571', '-1.227', '-0.205484352231']
BePPt 4 ['Be0.375P0.25Pt0.375', '-0.947', '-0.213091656822']
BePRb 1 ['Be0.3P0.5Rb0.2', '-0.68', '-0.218564430645']
BePRh 34 ['Be0.5P0.2Rh0.3', '-0.747', '-0.200124084708']
BePRu 32 ['Be0.333333P0.166667Ru0.5', '-0.584', '-0.215044855035']
BePSr 1 ['Be0.142857P0.571429Sr0.285714', '-1.082', '-0.200641062676']
BePTa 23 ['Be0.333333P0.444444Ta0.222222', '-0.737', '-0.202175040393']
BePTb 1 ['Be0.222222P0.444444Tb0.333333', '-1.232', '-0.200039768423']
BePTe 3 ['Be0.125P0.5Te0.375', '-0.4', '-0.216989459166']

BePTm 2 ['Be0.285714P0.428571Tm0.285714', '-1.328', '-0.220398359821']
BePW 7 ['Be0.3P0.4W0.3', '-0.643', '-0.200194694458']
BePXe 10 ['Be0.2P0.4Xe0.4', '-0.498', '-0.205183134666']
BePY 10 ['Be0.1P0.5Y0.4', '-1.69', '-0.208659361083']
BePYb 1 ['Be0.142857P0.428571Yb0.428571', '-1.084', '-0.21712463188']
BePZn 1 ['Be0.3P0.4Zn0.3', '-0.517', '-0.206773318166']
BePaS 2 ['Be0.125S0.625Pa0.25', '-1.605', '-0.282076392366']
BePaSe 14 ['Be0.1Se0.7Pa0.2', '-0.845', '-0.203670827']
BePmS 11 ['Be0.166667S0.5Pm0.333333', '-2.045', '-0.210981295492']
BePmSe 23 ['Be0.25Se0.375Pm0.375', '-1.426', '-0.210264849062']
BePrSe 2 ['Be0.3Se0.5Pr0.2', '-1.461', '-0.216343918165']
BePtSc 1 ['Be0.5Sc0.1Pt0.4', '-0.998', '-0.2405824695']
BePtSe 2 ['Be0.428571Se0.428571Pt0.142857', '-0.93', '-0.200724598571']
BePtTh 2 ['Be0.1Pt0.8Th0.1', '-0.682', '-0.2886999965']
BePtTi 4 ['Be0.444444Ti0.111111Pt0.444444', '-0.944', '-0.20499762319']
BePtV 1 ['Be0.5V0.125Pt0.375', '-0.836', '-0.21424105125']
BePtZr 1 ['Be0.5Zr0.1Pt0.4', '-0.99', '-0.261444664']
BePuS 3 ['Be0.125S0.5Pu0.375', '-1.952', '-0.21191377799']
BeRbSe 6 ['Be0.4Se0.5Rb0.1', '-1.03', '-0.20292302']
BeRhSe 1 ['Be0.428571Se0.428571Rh0.142857', '-0.917', '-0.208929912024']
BeRhSi 1 ['Be0.5Si0.1Rh0.4', '-0.817', '-0.203932581333']
BeRhTa 1 ['Be0.375Rh0.5Ta0.125', '-0.786', '-0.219288639821']
BeSTc 1 ['Be0.125S0.5Tc0.375', '-0.986', '-0.230994179243']
BeSTh 4 ['Be0.1S0.5Th0.4', '-2.088', '-0.217607020106']
BeSZr 15 ['Be0.142857S0.571429Zr0.285714', '-1.722', '-0.222705123417']
BeScSe 3 ['Be0.1Sc0.3Se0.6', '-1.345', '-0.2288739175']
BeSeSm 2 ['Be0.125Se0.5Sm0.375', '-1.85', '-0.208531477813']
BeSeTc 12 ['Be0.3Se0.5Tc0.2', '-0.698', '-0.218842907']
BeSeTi 1 ['Be0.2Ti0.3Se0.5', '-1.241', '-0.203609771545']
BeSeTm 6 ['Be0.3Se0.5Tm0.2', '-1.349', '-0.207293401']
BeSeY 12 ['Be0.1Se0.5Y0.4', '-1.777', '-0.204559987']
BeSeZr 13 ['Be0.142857Se0.571429Zr0.285714', '-1.364', '-0.20087501449']
BiBrCa 13 ['Ca0.166667Br0.666667Bi0.166667', '-1.78', '-0.201536038114']
BiBrCe 40 ['Br0.444444Ce0.222222Bi0.333333', '-1.634', '-0.206957740591']
BiBrCl 1 ['Cl0.5Br0.375Bi0.125', '-0.866', '-0.203436701243']
BiBrCo 3 ['Co0.111111Br0.777778Bi0.111111', '-0.776', '-0.206915542547']
BiBrCr 11 ['Cr0.2Br0.6Bi0.2', '-1.003', '-0.200858319552']
BiBrCu 3 ['Cu0.2Br0.6Bi0.2', '-1.005', '-0.202858319552']
BiBrDy 42 ['Br0.2Dy0.5Bi0.3', '-1.229', '-0.201355978851']
BiBrEr 44 ['Br0.375Er0.25Bi0.375', '-1.127', '-0.20910072097']
BiBrEu 38 ['Br0.5Eu0.166667Bi0.333333', '-1.644', '-0.209349960572']
BiBrF 2 ['F0.625Br0.25Bi0.125', '-1.992', '-0.221076870342']
BiBrGa 8 ['Ga0.4Br0.5Bi0.1', '-0.94', '-0.202472825354']
BiBrGd 22 ['Br0.666667Gd0.111111Bi0.222222', '-1.62', '-0.221875628667']

BiBrH 33 ['H0.4Br0.5Bi0.1', '-0.762', '-0.203455865856']
BiBrHf 17 ['Br0.666667Hf0.111111Bi0.222222', '-1.102', '-0.210731466169']
BiBrHo 46 ['Br0.25Ho0.5Bi0.25', '-1.184', '-0.212437417731']
BiBrI 14 ['Br0.285714I0.428571Bi0.285714', '-0.933', '-0.203525282318']
BiBrIr 10 ['Br0.666667Ir0.111111Bi0.222222', '-1.104', '-0.212731466169']
BiBrLa 24 ['Br0.625La0.25Bi0.125', '-2.173', '-0.202390937033']
BiBrLu 48 ['Br0.3Lu0.5Bi0.2', '-1.086', '-0.202785475275']
BiBrMn 6 ['Mn0.222222Br0.555556Bi0.222222', '-1.032', '-0.202847469115']
BiBrMo 1 ['Br0.8Mo0.1Bi0.1', '-0.965', '-0.245153246051']
BiBrNa 21 ['Na0.333333Br0.444444Bi0.222222', '-1.546', '-0.200123598275']
BiBrNd 4 ['Br0.666667Nd0.111111Bi0.222222', '-1.597', '-0.201564343391']
BiBrNi 21 ['Ni0.3Br0.5Bi0.2', '-0.876', '-0.204564105377']
BiBrNp 3 ['Br0.75Bi0.125Np0.125', '-1.556', '-0.26100299272']
BiBrO 32 ['O0.5Br0.2Bi0.3', '-1.415', '-0.200565713003']
BiBrOs 4 ['Br0.714286Os0.142857Bi0.142857', '-0.807', '-0.234041656823']
BiBrPa 8 ['Br0.7Bi0.1Pa0.2', '-1.722', '-0.237234511311']
BiBrPd 3 ['Br0.6Pd0.2Bi0.2', '-1.008', '-0.205858319552']
BiBrPm 70 ['Br0.3Pm0.5Bi0.2', '-1.307', '-0.203414389524']
BiBrPr 34 ['Br0.222222Pr0.444444Bi0.333333', '-1.515', '-0.211849735019']
BiBrPt 24 ['Br0.5Pt0.125Bi0.375', '-0.965', '-0.208412908897']
BiBrPu 36 ['Br0.222222Bi0.333333Pu0.444444', '-0.982', '-0.228570122612']
BiBrRe 5 ['Br0.7Re0.2Bi0.1', '-0.622', '-0.220929159776']
BiBrRh 21 ['Br0.8Rh0.1Bi0.1', '-0.884', '-0.215337851551']
BiBrRu 11 ['Br0.571429Ru0.142857Bi0.285714', '-0.985', '-0.216366348764']
BiBrSb 2 ['Br0.777778Sb0.111111Bi0.111111', '-1.02', '-0.204152773668']
BiBrSc 40 ['Sc0.5Br0.25Bi0.25', '-1.006', '-0.209832966896']
BiBrSm 45 ['Br0.375Sm0.25Bi0.375', '-1.505', '-0.207381258467']
BiBrSr 7 ['Br0.3Sr0.4Bi0.3', '-1.701', '-0.208022781139']
BiBrTa 3 ['Br0.75Ta0.125Bi0.125', '-1.162', '-0.201111145162']
BiBrTb 39 ['Br0.285714Tb0.428571Bi0.285714', '-1.177', '-0.223832597132']
BiBrTc 5 ['Br0.7Tc0.1Bi0.2', '-1.093', '-0.210644317977']
BiBrTe 3 ['Br0.75Te0.125Bi0.125', '-0.809', '-0.257062100246']
BiBrTh 14 ['Br0.375Bi0.25Th0.375', '-1.415', '-0.200776744805']
BiBrTi 1 ['Ti0.1Br0.7Bi0.2', '-1.337', '-0.205926054181']
BiBrTl 2 ['Br0.555556Tl0.333333Bi0.111111', '-1.19', '-0.201915177916']
BiBrTm 43 ['Br0.2Tm0.4Bi0.4', '-1.114', '-0.208609583351']
BiBrU 2 ['Br0.75Bi0.125U0.125', '-1.58', '-0.263193772982']
BiBrV 4 ['V0.166667Br0.666667Bi0.166667', '-1.16', '-0.203278308944']
BiBrY 55 ['Br0.125Y0.5Bi0.375', '-1.173', '-0.204817890948']
BiBrYb 38 ['Br0.375Yb0.5Bi0.125', '-1.81', '-0.215742879928']
BiBrZr 4 ['Br0.75Zr0.125Bi0.125', '-1.53', '-0.215846377565']
BiCDy 4 ['C0.375Dy0.375Bi0.25', '-0.737', '-0.213626320313']
BiCLa 1 ['C0.333333La0.444444Bi0.222222', '-0.772', '-0.203969471389']
BiCLu 11 ['C0.125Lu0.5Bi0.375', '-0.834', '-0.206824060223']

BiCNp 4 ['C0.4Bi0.2Np0.4', '-0.209', '-0.20882810575']
BiCPa 7 ['C0.5Bi0.166667Pa0.333333', '-0.516', '-0.212760580833']
BiCPm 1 ['C0.5Pm0.375Bi0.125', '-0.419', '-0.203996282187']
BiCPu 7 ['C0.333333Bi0.222222Pu0.444444', '-0.381', '-0.212327922778']
BiCSc 3 ['C0.333333Sc0.5Bi0.166667', '-0.734', '-0.220316121458']
BiCSm 1 ['C0.444444Sm0.333333Bi0.222222', '-0.707', '-0.227322621111']
BiCTh 2 ['C0.333333Bi0.222222Th0.444444', '-0.635', '-0.216404154722']
BiCY 4 ['C0.166667Y0.5Bi0.333333', '-0.931', '-0.214482838472']
BiCZr 1 ['C0.428571Zr0.428571Bi0.142857', '-0.919', '-0.222992171071']
BiCaCl 1 ['Cl0.333333Ca0.444444Bi0.222222', '-1.966', '-0.221079700496']
BiCaF 2 ['F0.2Ca0.5Bi0.3', '-2.056', '-0.201314074733']
BiCaH 23 ['H0.333333Ca0.444444Bi0.222222', '-0.906', '-0.201930137058']
BiCaI 1 ['Ca0.4I0.3Bi0.3', '-1.42', '-0.233760355795']
BiCaO 6 ['O0.6Ca0.3Bi0.1', '-2.565', '-0.22673503772']
BiCaPd 4 ['Ca0.428571Pd0.285714Bi0.285714', '-0.985', '-0.211359723572']
BiCaSe 11 ['Ca0.5Se0.2Bi0.3', '-1.506', '-0.213716637227']
BiCdCl 1 ['Cl0.5Cd0.2Bi0.3', '-1.179', '-0.219897591775']
BiCdF 5 ['F0.5Cd0.3Bi0.2', '-2.046', '-0.204173100435']
BiCdI 5 ['Cd0.285714I0.428571Bi0.285714', '-0.656', '-0.204956090864']
BiCdO 3 ['O0.6Cd0.3Bi0.1', '-1.365', '-0.237750213309']
BiCdP 1 ['P0.375Cd0.375Bi0.25', '-0.369', '-0.202481749609']
BiCeCl 8 ['Cl0.4Ce0.3Bi0.3', '-1.928', '-0.219359948335']
BiCeF 8 ['F0.1Ce0.4Bi0.5', '-1.51', '-0.202848101365']
BiCeH 13 ['H0.555556Ce0.333333Bi0.111111', '-0.866', '-0.215768660731']
BiCeI 61 ['I0.5Ce0.4Bi0.1', '-1.311', '-0.208517065395']
BiCeO 15 ['O0.625Ce0.125Bi0.25', '-2.264', '-0.201933031558']
BiCeRh 5 ['Rh0.375Ce0.25Bi0.375', '-0.777', '-0.21711940375']
BiCeS 5 ['S0.428571Ce0.428571Bi0.142857', '-2.082', '-0.209663090779']
BiCeSe 29 ['Se0.222222Ce0.555556Bi0.222222', '-1.491', '-0.201995886111']
BiClCr 15 ['Cl0.5Cr0.2Bi0.3', '-1.121', '-0.200017755527']
BiClDy 1 ['Cl0.777778Dy0.111111Bi0.111111', '-1.885', '-0.227343005791']
BiClEr 50 ['Cl0.444444Er0.111111Bi0.444444', '-1.108', '-0.208180022873']
BiClEu 12 ['Cl0.25Eu0.5Bi0.25', '-1.903', '-0.206465241824']
BiClFe 1 ['Cl0.666667Fe0.111111Bi0.222222', '-1.275', '-0.202977186809']
BiClGa 2 ['Cl0.555556Ga0.333333Bi0.111111', '-1.293', '-0.241721985075']
BiClGd 4 ['Cl0.7Gd0.1Bi0.2', '-1.861', '-0.206090440649']
BiClH 16 ['H0.444444Cl0.222222Bi0.333333', '-0.562', '-0.20465906227']
BiClHo 68 ['Cl0.1Ho0.5Bi0.4', '-1.159', '-0.20252074277']
BiClIn 2 ['Cl0.75In0.125Bi0.125', '-1.232', '-0.206308694191']
BiClIr 2 ['Cl0.777778Ir0.111111Bi0.111111', '-0.737', '-0.200988593404']
BiClLa 13 ['Cl0.4La0.3Bi0.3', '-1.989', '-0.202277567255']
BiClLu 63 ['Cl0.1Lu0.6Bi0.3', '-0.913', '-0.214713648938']
BiClN 3 ['N0.166667Cl0.5Bi0.333333', '-1.013', '-0.208982890107']
BiClNd 6 ['Cl0.25Nd0.5Bi0.25', '-1.587', '-0.201318190263']

BiClNp 5 ['Cl0.7Bi0.2Np0.1', '-1.666', '-0.224150171525']
BiClO 6 ['O0.571429Cl0.142857Bi0.285714', '-1.412', '-0.206882221728']
BiClPa 11 ['Cl0.5Bi0.166667Pa0.333333', '-1.536', '-0.211308844021']
BiClPb 4 ['Cl0.6Pb0.2Bi0.2', '-1.361', '-0.203321223797']
BiClPd 1 ['Cl0.625Pd0.125Bi0.25', '-1.26', '-0.211204348258']
BiClPm 53 ['Cl0.222222Pm0.333333Bi0.444444', '-1.265', '-0.202361633844']
BiClPr 9 ['Cl0.1Pr0.4Bi0.5', '-1.267', '-0.204539387438']
BiClPt 19 ['Cl0.777778Pt0.111111Bi0.111111', '-0.957', '-0.219468420304']
BiClPu 25 ['Cl0.666667Bi0.166667Pu0.166667', '-1.919', '-0.200716346396']
BiClRh 16 ['Cl0.666667Rh0.111111Bi0.222222', '-1.277', '-0.204977186809']
BiClRu 9 ['Cl0.666667Ru0.166667Bi0.166667', '-1.19', '-0.207911389726']
BiClSm 39 ['Cl0.3Sm0.5Bi0.2', '-1.55', '-0.200211740482']
BiClTa 2 ['Cl0.777778Ta0.111111Bi0.111111', '-1.622', '-0.206660700904']
BiClTb 15 ['Cl0.666667Tb0.111111Bi0.222222', '-1.804', '-0.212133908236']
BiClTc 1 ['Cl0.8Tc0.1Bi0.1', '-1.183', '-0.224772300942']
BiClTe 1 ['Cl0.7Te0.1Bi0.2', '-1.326', '-0.247565387435']
BiClTh 6 ['Cl0.5Bi0.166667Th0.333333', '-1.92', '-0.20170301143']
BiClTm 2 ['Cl0.777778Tm0.111111Bi0.111111', '-1.896', '-0.240835864867']
BiClXe 1 ['Cl0.666667Xe0.222222Bi0.111111', '-0.744', '-0.207988593404']
BiClY 13 ['Cl0.333333Y0.555556Bi0.111111', '-1.578', '-0.208036758128']
BiClZr 4 ['Cl0.7Zr0.1Bi0.2', '-1.723', '-0.209727406314']
BiCoF 4 ['F0.777778Co0.111111Bi0.111111', '-2.366', '-0.209850933362']
BiCoN 9 ['N0.25Co0.5Bi0.25', '-0.234', '-0.224367472414']
BiCoO 3 ['O0.6Co0.1Bi0.3', '-1.65', '-0.220245578735']
BiCrO 3 ['O0.666667Cr0.111111Bi0.222222', '-1.765', '-0.20489023116']
BiCsF 1 ['F0.666667Cs0.222222Bi0.111111', '-2.915', '-0.263183344746']
BiCsH 8 ['H0.555556Cs0.222222Bi0.222222', '-0.368', '-0.218168172222']
BiCsO 8 ['O0.666667Cs0.111111Bi0.222222', '-1.436', '-0.201539214294']
BiCsSe 1 ['Se0.3Cs0.6Bi0.1', '-1.116', '-0.203890444893']
BiCuF 1 ['F0.75Cu0.125Bi0.125', '-2.302', '-0.201005278466']
BiCuO 3 ['O0.6Cu0.1Bi0.3', '-1.54', '-0.240772126927']
BiDyF 8 ['F0.333333Dy0.333333Bi0.333333', '-2.426', '-0.200621426959']
BiDyI 57 ['I0.2Dy0.3Bi0.5', '-0.891', '-0.201454641348']
BiDyO 1 ['O0.625Dy0.125Bi0.25', '-2.383', '-0.217916299321']
BiDyS 6 ['S0.428571Dy0.428571Bi0.142857', '-2.09', '-0.225948016034']
BiDySe 7 ['Se0.333333Dy0.444444Bi0.222222', '-1.64', '-0.210631753702']
BiErI 39 ['I0.6Er0.1Bi0.3', '-0.855', '-0.201872224043']
BiErO 1 ['O0.625Er0.125Bi0.25', '-2.44', '-0.260350406324']
BiErS 12 ['S0.333333Er0.5Bi0.166667', '-1.886', '-0.20986659616']
BiEuF 5 ['F0.285714Eu0.428571Bi0.285714', '-2.694', '-0.201280349532']
BiEuH 6 ['H0.5Eu0.333333Bi0.166667', '-0.875', '-0.213678563283']
BiEuI 30 ['I0.625Eu0.25Bi0.125', '-1.708', '-0.205388338687']
BiEuN 28 ['N0.1Eu0.6Bi0.3', '-1.152', '-0.226115604466']
BiEuO 2 ['O0.625Eu0.25Bi0.125', '-2.46', '-0.201391341434']

BiEuP 11 ['P0.4Eu0.4Bi0.2', '-1.311', '-0.201371439669']
BiEuS 6 ['S0.333333Eu0.5Bi0.166667', '-2.174', '-0.214618203243']
BiEuSe 15 ['Se0.444444Eu0.444444Bi0.111111', '-2.178', '-0.216324982222']
BiFGa 2 ['F0.7Ga0.2Bi0.1', '-2.878', '-0.215417197607']
BiFGd 9 ['F0.333333Gd0.444444Bi0.222222', '-2.481', '-0.200362625569']
BiFGe 1 ['F0.8Ge0.1Bi0.1', '-2.655', '-0.203983623948']
BiFH 27 ['H0.2F0.5Bi0.3', '-2.035', '-0.210990440433']
BiFHf 1 ['F0.8Hf0.1Bi0.1', '-3.418', '-0.208383993948']
BiFHo 1 ['F0.777778Ho0.111111Bi0.111111', '-3.536', '-0.270865443362']
BiFIn 1 ['F0.8In0.1Bi0.1', '-2.557', '-0.217002045696']
BiFK 6 ['F0.714286K0.142857Bi0.142857', '-2.736', '-0.203495278303']
BiFLa 4 ['F0.428571La0.285714Bi0.285714', '-3.066', '-0.202090396326']
BiFLi 2 ['Li0.222222F0.666667Bi0.111111', '-2.981', '-0.230071865023']
BiFLu 20 ['F0.25Lu0.375Bi0.375', '-2.001', '-0.2146404248']
BiFMg 1 ['F0.8Mg0.1Bi0.1', '-2.753', '-0.377067995192']
BiFN 2 ['N0.222222F0.444444Bi0.333333', '-1.843', '-0.221658169274']
BiFNa 7 ['F0.777778Na0.111111Bi0.111111', '-2.36', '-0.204346653262']
BiFNd 3 ['F0.222222Nd0.333333Bi0.444444', '-2.007', '-0.20247390229']
BiFNi 2 ['F0.8Ni0.1Bi0.1', '-2.193', '-0.237868492696']
BiFNp 1 ['F0.8Bi0.1Np0.1', '-3.354', '-0.306038333403']
BiFO 2 ['O0.1F0.8Bi0.1', '-1.543', '-0.314020105936']
BiFP 1 ['F0.714286P0.142857Bi0.142857', '-2.771', '-0.229419480678']
BiFPa 5 ['F0.7Bi0.1Pa0.2', '-3.484', '-0.241297746607']
BiFPm 11 ['F0.428571Pm0.285714Bi0.285714', '-2.832', '-0.209207772517']
BiFPr 12 ['F0.777778Pr0.111111Bi0.111111', '-3.453', '-0.213934975678']
BiFPt 1 ['F0.8Pt0.1Bi0.1', '-2.127', '-0.200649632569']
BiFPu 5 ['F0.7Bi0.1Pu0.2', '-3.764', '-0.276865477607']
BiFRb 1 ['F0.8Rb0.1Bi0.1', '-2.174', '-0.206303549024']
BiFRh 1 ['F0.8Rh0.1Bi0.1', '-2.154', '-0.207268986196']
BiFRu 1 ['F0.8Ru0.1Bi0.1', '-2.379', '-0.263865906448']
BiFSc 3 ['F0.75Sc0.125Bi0.125', '-3.705', '-0.202076025025']
BiFSi 1 ['F0.8Si0.1Bi0.1', '-3.044', '-0.209801185948']
BiFSm 7 ['F0.3Sm0.3Bi0.4', '-2.353', '-0.205486879512']
BiFSr 7 ['F0.777778Sr0.111111Bi0.111111', '-2.97', '-0.20247089901']
BiFTc 1 ['F0.8Tc0.1Bi0.1', '-2.481', '-0.201949956073']
BiFTh 1 ['F0.8Bi0.1Th0.1', '-3.661', '-0.269120568613']
BiFTi 3 ['F0.75Ti0.125Bi0.125', '-3.417', '-0.24480474768']
BiFTm 52 ['F0.555556Tm0.111111Bi0.333333', '-2.412', '-0.207098997704']
BiFY 5 ['F0.714286Y0.142857Bi0.142857', '-3.645', '-0.203786191808']
BiFYb 60 ['F0.111111Yb0.555556Bi0.333333', '-1.484', '-0.218795953112']
BiFZr 1 ['F0.8Zr0.1Bi0.1', '-3.445', '-0.302826389323']
BiGaI 11 ['Ga0.142857I0.571429Bi0.285714', '-0.686', '-0.203677079327']
BiGaO 3 ['O0.666667Ga0.111111Bi0.222222', '-1.648', '-0.207666421682']
BiGaSe 4 ['Ga0.4Se0.5Bi0.1', '-0.752', '-0.205264231375']

BiGdI 46 ['I0.333333Gd0.444444Bi0.222222', '-1.182', '-0.208863762522']
BiGdO 1 ['O0.625Gd0.125Bi0.25', '-2.375', '-0.227855485699']
BiGdRh 1 ['Rh0.333333Gd0.333333Bi0.333333', '-0.908', '-0.207357854167']
BiGdS 3 ['S0.4Gd0.4Bi0.2', '-1.954', '-0.204625052393']
BiHI 15 ['H0.5I0.333333Bi0.166667', '-0.482', '-0.211720286413']
BiHK 6 ['H0.555556K0.333333Bi0.111111', '-0.375', '-0.202325073519']
BiHLa 10 ['H0.4La0.3Bi0.3', '-0.864', '-0.203343739293']
BiHLi 3 ['H0.142857Li0.571429Bi0.285714', '-0.642', '-0.202253527545']
BiHMn 4 ['H0.375Mn0.375Bi0.25', '-0.215', '-0.215']
BiHO 10 ['H0.333333O0.444444Bi0.222222', '-1.476', '-0.20168366277']
BiHP 12 ['H0.4P0.4Bi0.2', '-0.203', '-0.203']
BiHPm 13 ['H0.7Pm0.2Bi0.1', '-0.517', '-0.200040597845']
BiHPu 15 ['H0.2Bi0.3Pu0.5', '-0.52', '-0.213166906063']
BiHRb 22 ['H0.444444Rb0.222222Bi0.333333', '-0.393', '-0.209397324097']
BiHSe 3 ['H0.3Se0.5Bi0.2', '-0.395', '-0.201248149']
BiHSr 12 ['H0.4Sr0.4Bi0.2', '-0.87', '-0.20872016028']
BiHYb 11 ['H0.5Yb0.2Bi0.3', '-0.574', '-0.204627192126']
BiHfO 6 ['O0.625Hf0.25Bi0.125', '-3.387', '-0.200549200385']
BiHfS 17 ['S0.3Hf0.6Bi0.1', '-1.218', '-0.2030211633']
BiHfSe 5 ['Se0.5Hf0.333333Bi0.166667', '-1.302', '-0.233694958333']
BiHgI 2 ['I0.625Hg0.125Bi0.25', '-0.717', '-0.210225537024']
BiHgO 5 ['O0.666667Hg0.166667Bi0.166667', '-0.998', '-0.203956267759']
BiHgP 7 ['P0.5Hg0.125Bi0.375', '-0.207', '-0.207']
BiHoI 34 ['I0.571429Ho0.142857Bi0.285714', '-0.902', '-0.213535118533']
BiHoO 2 ['O0.625Ho0.25Bi0.125', '-3.11', '-0.206735240647']
BiHoS 13 ['S0.4Ho0.5Bi0.1', '-2.075', '-0.208570267231']
BiHoSe 8 ['Se0.555556Ho0.333333Bi0.111111', '-1.813', '-0.202414623984']
BiHoTe 1 ['Te0.5Ho0.4Bi0.1', '-1.355', '-0.258156361499']
BiIIn 1 ['In0.25I0.5Bi0.25', '-0.696', '-0.210706684203']
BiILa 37 ['I0.5La0.166667Bi0.333333', '-1.316', '-0.20128924212']
BiILu 41 ['I0.111111Lu0.555556Bi0.333333', '-0.831', '-0.207405225841']
BiINa 1 ['Na0.5I0.375Bi0.125', '-1.373', '-0.219260554288']
BiINd 31 ['I0.375Nd0.25Bi0.375', '-1.125', '-0.204729086274']
BiINi 5 ['Ni0.285714I0.428571Bi0.285714', '-0.572', '-0.211706821459']
BiINp 2 ['I0.666667Bi0.166667Np0.166667', '-1.011', '-0.2039549672']
BiIO 4 ['O0.5I0.2Bi0.3', '-1.423', '-0.2060339302']
BiIPa 43 ['I0.25Bi0.375Pa0.375', '-0.481', '-0.204875070018']
BiIPm 65 ['I0.1Pm0.3Bi0.6', '-0.8', '-0.214166466174']
BiIPr 46 ['I0.666667Pr0.166667Bi0.166667', '-1.3', '-0.202824413799']
BiIPt 25 ['I0.666667Pt0.166667Bi0.166667', '-0.69', '-0.218107942548']
BiIPu 3 ['I0.555556Bi0.111111Pu0.333333', '-1.187', '-0.216359720319']
BiIRh 19 ['Rh0.166667I0.666667Bi0.166667', '-0.606', '-0.200580429619']
BiIRu 2 ['Ru0.166667I0.333333Bi0.5', '-0.502', '-0.209907955858']
BiISm 48 ['I0.571429Sm0.142857Bi0.285714', '-0.973', '-0.21248091123']

BiITb 46 ['I0.555556Tb0.111111Bi0.333333', '-0.789', '-0.211709357392']
BiITh 9 ['I0.5Bi0.166667Th0.333333', '-1.275', '-0.212047327015']
BiITm 26 ['I0.375Tm0.375Bi0.25', '-1.209', '-0.203991408774']
BiIYb 11 ['I0.428571Yb0.285714Bi0.285714', '-1.473', '-0.204524681816']
BiInO 3 ['O0.625In0.125Bi0.25', '-1.778', '-0.239786139058']
BiIrO 1 ['O0.6Ir0.2Bi0.2', '-1.45', '-0.208422233161']
BiIrS 1 ['S0.5Ir0.125Bi0.375', '-0.664', '-0.206855562994']
BiKO 5 ['O0.666667K0.111111Bi0.222222', '-1.446', '-0.203204652587']
BiKrP 4 ['P0.5Kr0.2Bi0.3', '-0.204', '-0.204']
BiLaN 7 ['N0.333333La0.5Bi0.166667', '-1.507', '-0.200964872387']
BiLaO 9 ['O0.7La0.1Bi0.2', '-1.906', '-0.206777731576']
BiLaPm 1 ['La0.3Pm0.1Bi0.6', '-1.022', '-0.21462023425']
BiLaS 1 ['S0.333333La0.444444Bi0.222222', '-2.008', '-0.217432134217']
BiLaSe 24 ['Se0.25La0.5Bi0.25', '-1.734', '-0.20144368625']
BiLiPu 1 ['Li0.125Bi0.375Pu0.5', '-0.527', '-0.207999036042']
BiLuN 12 ['N0.142857Lu0.571429Bi0.285714', '-1.2', '-0.204586264237']
BiLuO 5 ['O0.666667Lu0.166667Bi0.166667', '-2.494', '-0.200745902401']
BiLuS 2 ['S0.285714Lu0.428571Bi0.285714', '-1.628', '-0.201450721948']
BiLuSe 8 ['Se0.333333Lu0.555556Bi0.111111', '-1.433', '-0.208730352778']
BiMgO 3 ['O0.625Mg0.125Bi0.25', '-1.911', '-0.200492738928']
BiMnO 1 ['O0.6Mn0.2Bi0.2', '-1.991', '-0.240125931329']
BiMnS 3 ['S0.444444Mn0.111111Bi0.444444', '-0.642', '-0.203124275989']
BiMnSe 10 ['Mn0.428571Se0.285714Bi0.285714', '-0.413', '-0.205419536059']
BiMoO 2 ['O0.625Mo0.125Bi0.25', '-2.008', '-0.225508341951']
BiNNp 11 ['N0.6Bi0.2Np0.2', '-0.696', '-0.21101711518']
BiNO 15 ['N0.25O0.375Bi0.375', '-1.124', '-0.202732796231']
BiNPa 19 ['N0.428571Bi0.285714Pa0.285714', '-0.775', '-0.210460462314']
BiNPu 2 ['N0.6Bi0.1Pu0.3', '-1.075', '-0.253526789398']
BiNSc 1 ['N0.5Sc0.4Bi0.1', '-1.817', '-0.200524531864']
BiNTi 2 ['N0.444444Ti0.444444Bi0.111111', '-1.368', '-0.215078380437']
BiNY 3 ['N0.375Y0.5Bi0.125', '-1.782', '-0.207029496123']
BiNYb 16 ['N0.5Yb0.333333Bi0.166667', '-0.783', '-0.200457284108']
BiNaO 6 ['O0.625Na0.25Bi0.125', '-1.526', '-0.205994612611']
BiNaPu 1 ['Na0.142857Bi0.428571Pu0.428571', '-0.574', '-0.224096668688']
BiNbO 1 ['O0.625Nb0.125Bi0.25', '-2.28', '-0.225843089765']
BiNdO 1 ['O0.3Nd0.4Bi0.3', '-2.496', '-0.237016180988']
BiNdS 4 ['S0.444444Nd0.444444Bi0.111111', '-2.161', '-0.218290744603']
BiNdSe 10 ['Se0.4Nd0.4Bi0.2', '-1.718', '-0.221816969499']
BiNiO 5 ['O0.6Ni0.1Bi0.3', '-1.591', '-0.204631811952']
BiNpO 2 ['O0.3Bi0.2Np0.5', '-1.976', '-0.23542077434']
BiNpSe 3 ['Se0.333333Bi0.222222Np0.444444', '-0.911', '-0.202395450136']
BiOOs 1 ['O0.625Os0.125Bi0.25', '-1.582', '-0.22519753601']
BiOPa 8 ['O0.6Bi0.2Pa0.2', '-2.57', '-0.219377383971']
BiOPd 2 ['O0.625Pd0.125Bi0.25', '-1.366', '-0.239789570698']

BiOPr 2 ['O0.375Pr0.5Bi0.125', '-2.713', '-0.210539840867']
BiOPu 1 ['O0.666667Bi0.222222Pu0.111111', '-2.182', '-0.238290254973']
BiORb 2 ['O0.625Rb0.125Bi0.25', '-1.64', '-0.265521289538']
BiORh 1 ['O0.6Rh0.2Bi0.2', '-1.522', '-0.283741914658']
BiORu 2 ['O0.6Ru0.1Bi0.3', '-1.604', '-0.210168226376']
BiOS 4 ['O0.666667S0.222222Bi0.111111', '-1.578', '-0.213188570396']
BiOSb 1 ['O0.666667Sb0.111111Bi0.222222', '-1.857', '-0.325505868023']
BiOSc 4 ['O0.6Sc0.2Bi0.2', '-2.872', '-0.23129137572']
BiOSi 1 ['O0.666667Si0.111111Bi0.222222', '-2.099', '-0.202345487998']
BiOSm 2 ['O0.625Sm0.125Bi0.25', '-2.319', '-0.200479523746']
BiOSr 1 ['O0.625Sr0.125Bi0.25', '-2.104', '-0.309249400385']
BiOTb 1 ['O0.666667Tb0.111111Bi0.222222', '-2.081', '-0.235272648596']
BiOTc 3 ['O0.666667Tc0.111111Bi0.222222', '-1.826', '-0.301519770708']
BiOTh 2 ['O0.666667Bi0.222222Th0.111111', '-2.474', '-0.20493132594']
BiOTm 3 ['O0.625Tm0.125Bi0.25', '-2.403', '-0.212350127105']
BiOY 3 ['O0.666667Y0.111111Bi0.222222', '-2.131', '-0.216404084459']
BiOZn 1 ['O0.6Zn0.2Bi0.2', '-1.703', '-0.27235055097']
BiOZr 1 ['O0.625Zr0.125Bi0.25', '-2.549', '-0.26536438476']
BiPPb 13 ['P0.444444Pb0.222222Bi0.333333', '-0.224', '-0.224']
BiPPm 17 ['P0.333333Pm0.444444Bi0.222222', '-1.186', '-0.204543525902']
BiPPu 7 ['P0.555556Bi0.222222Pu0.222222', '-0.786', '-0.212624511342']
BiPS 22 ['P0.444444S0.333333Bi0.222222', '-0.521', '-0.205407537596']
BiPSe 14 ['P0.2Se0.4Bi0.4', '-0.463', '-0.204664198667']
BiPSr 5 ['P0.5Sr0.166667Bi0.333333', '-0.765', '-0.210508160103']
BiPTa 2 ['P0.428571Ta0.428571Bi0.142857', '-0.79', '-0.217679641874']
BiPTb 2 ['P0.5Tb0.166667Bi0.333333', '-0.671', '-0.201214281063']
BiPTe 9 ['P0.4Te0.2Bi0.4', '-0.288', '-0.20826212']
BiPTm 1 ['P0.5Tm0.4Bi0.1', '-1.661', '-0.253920817895']
BiPXe 10 ['P0.5Xe0.3Bi0.2', '-0.202', '-0.202']
BiPY 6 ['P0.333333Y0.555556Bi0.111111', '-1.593', '-0.203453697569']
BiPYb 13 ['P0.375Yb0.375Bi0.25', '-0.996', '-0.221833826796']
BiPaS 6 ['S0.5Bi0.125Pa0.375', '-1.661', '-0.203459462366']
BiPaSe 16 ['Se0.555556Bi0.222222Pa0.222222', '-0.966', '-0.215597785556']
BiPdPm 10 ['Pd0.571429Pm0.285714Bi0.142857', '-0.769', '-0.200508780952']
BiPdTm 1 ['Pd0.4Tm0.3Bi0.3', '-0.971', '-0.207183783357']
BiPmS 11 ['S0.375Pm0.375Bi0.25', '-1.798', '-0.212173630056']
BiPmSe 29 ['Se0.428571Pm0.285714Bi0.285714', '-1.233', '-0.214462813095']
BiPmTe 1 ['Te0.5Pm0.4Bi0.1', '-1.343', '-0.259141373664']
BiPrRh 1 ['Rh0.333333Pr0.333333Bi0.333333', '-0.907', '-0.219909591945']
BiPrS 6 ['S0.25Pr0.375Bi0.375', '-1.542', '-0.202456792642']
BiPrSe 12 ['Se0.222222Pr0.444444Bi0.333333', '-1.477', '-0.205718480277']
BiPtS 30 ['S0.2Pt0.4Bi0.4', '-0.588', '-0.209129565697']
BiPtTh 6 ['Pt0.666667Bi0.111111Th0.222222', '-0.828', '-0.221027919444']
BiPtU 1 ['Pt0.75Bi0.125U0.125', '-0.647', '-0.201487665625']

BiPtY 1 ['Y0.375Pt0.375Bi0.25', '-1.304', '-0.202285422188']
BiPuS 2 ['S0.5Bi0.1Pu0.4', '-1.889', '-0.204226852491']
BiPuSe 4 ['Se0.5Bi0.166667Pu0.333333', '-1.387', '-0.226225313332']
BiRhS 2 ['S0.428571Rh0.142857Bi0.428571', '-0.671', '-0.203612471841']
BiRuSe 12 ['Se0.3Ru0.3Bi0.4', '-0.402', '-0.208248149']
BiSSc 3 ['S0.333333Sc0.444444Bi0.222222', '-1.722', '-0.225403220327']
BiSSm 9 ['S0.444444Sm0.333333Bi0.222222', '-1.966', '-0.232831410715']
BiSTb 7 ['S0.3Tb0.4Bi0.3', '-1.486', '-0.262271915682']
BiSTc 8 ['S0.5Tc0.3Bi0.2', '-0.809', '-0.203663187994']
BiSTh 5 ['S0.3Bi0.3Th0.4', '-1.583', '-0.207096432295']
BiSTm 10 ['S0.333333Tm0.555556Bi0.111111', '-1.764', '-0.204982668104']
BiSU 1 ['S0.5Bi0.2U0.3', '-1.512', '-0.213964576496']
BiSXe 1 ['S0.5Xe0.4Bi0.1', '-0.334', '-0.202899507023']
BiSY 8 ['S0.285714Y0.428571Bi0.285714', '-1.711', '-0.200513690896']
BiSZr 15 ['S0.333333Zr0.5Bi0.166667', '-1.337', '-0.213457765049']
BiScSe 17 ['Sc0.5Se0.3Bi0.2', '-1.435', '-0.2058616825']
BiSeSm 13 ['Se0.5Sm0.4Bi0.1', '-1.915', '-0.207085781167']
BiSeSr 3 ['Se0.333333Sr0.444444Bi0.222222', '-1.811', '-0.231411188148']
BiSeTb 5 ['Se0.375Tb0.375Bi0.25', '-1.395', '-0.240889169498']
BiSeTc 6 ['Se0.5Tc0.333333Bi0.166667', '-0.387', '-0.225540124167']
BiSeTh 11 ['Se0.555556Bi0.111111Th0.333333', '-1.743', '-0.202861352219']
BiSeTm 10 ['Se0.375Tm0.375Bi0.25', '-1.475', '-0.23271967625']
BiSeU 1 ['Se0.375Bi0.25U0.375', '-0.954', '-0.210459523125']
BiSeV 1 ['V0.333333Se0.5Bi0.166667', '-0.811', '-0.214488667593']
BiSeY 28 ['Se0.1Y0.4Bi0.5', '-1.103', '-0.2012914865']
BiSeYb 9 ['Se0.333333Yb0.444444Bi0.222222', '-1.818', '-0.219051663333']
BiSeZr 15 ['Se0.4Zr0.3Bi0.3', '-1.094', '-0.204596614625']
BiTeTh 5 ['Te0.285714Bi0.285714Th0.428571', '-1.108', '-0.201653621405']
BrCCa 1 ['C0.3Ca0.4Br0.3', '-1.323', '-0.303453274527']
BrCCl 7 ['C0.166667Cl0.5Br0.333333', '-0.38', '-0.201669225552']
BrCDy 18 ['C0.2Br0.6Dy0.2', '-1.22', '-0.219467018702']
BrCEr 41 ['C0.428571Br0.285714Er0.285714', '-0.813', '-0.20948715788']
BrCEu 7 ['C0.111111Br0.666667Eu0.222222', '-1.816', '-0.20022279204']
BrCGd 1 ['C0.125Br0.5Gd0.375', '-1.833', '-0.232014466498']
BrCHf 33 ['C0.375Br0.125Hf0.5', '-0.996', '-0.20556452251']
BrCHo 34 ['C0.375Br0.375Ho0.25', '-0.917', '-0.200428870211']
BrCKr 1 ['C0.111111Br0.555556Kr0.333333', '-0.2', '-0.2']
BrCLu 46 ['C0.3Br0.2Lu0.5', '-0.747', '-0.203374678053']
BrCNd 1 ['C0.25Br0.375Nd0.375', '-1.348', '-0.20742628722']
BrCOs 1 ['C0.2Br0.6Os0.2', '-0.264', '-0.264']
BrCPa 4 ['C0.5Br0.1Pa0.4', '-0.752', '-0.200010991821']
BrCPm 48 ['C0.4Br0.2Pm0.4', '-0.726', '-0.214609424016']
BrCPr 8 ['C0.3Br0.4Pr0.3', '-1.531', '-0.223369717357']
BrCPt 10 ['C0.111111Br0.555556Pt0.333333', '-0.24', '-0.24']

BrCPu 4 ['C0.142857Br0.571429Pu0.285714', '-1.616', '-0.256677429811']
BrCRh 1 ['C0.1Br0.7Rh0.2', '-0.771', '-0.23581738355']
BrCSc 9 ['C0.222222Sc0.333333Br0.444444', '-1.555', '-0.208443374346']
BrCSe 1 ['C0.111111Se0.333333Br0.555556', '-0.37', '-0.210897537528']
BrCTb 21 ['C0.4Br0.3Tb0.3', '-0.908', '-0.201424192072']
BrCTm 29 ['C0.1Br0.8Tm0.1', '-0.775', '-0.224314357776']
BrCXe 2 ['C0.142857Br0.571429Xe0.285714', '-0.2', '-0.2']
BrCY 25 ['C0.285714Br0.428571Y0.285714', '-1.335', '-0.203098589498']
BrCaDy 5 ['Ca0.2Br0.7Dy0.1', '-2.142', '-0.282337875387']
BrCaEr 11 ['Ca0.1Br0.6Er0.3', '-1.598', '-0.284613698792']
BrCaF 2 ['F0.625Ca0.125Br0.25', '-2.41', '-0.223348558993']
BrCaGe 1 ['Ca0.4Ge0.3Br0.3', '-1.58', '-0.217183515527']
BrCaH 4 ['H0.4Ca0.5Br0.1', '-0.889', '-0.206575245135']
BrCaHf 11 ['Ca0.1Br0.6Hf0.3', '-1.1', '-0.229045705293']
BrCaHo 7 ['Ca0.2Br0.7Ho0.1', '-2.071', '-0.219933882476']
BrCaLu 10 ['Ca0.125Br0.625Lu0.25', '-1.649', '-0.313431208168']
BrCaNp 1 ['Ca0.111111Br0.777778Np0.111111', '-1.671', '-0.210306019353']
BrCaO 28 ['O0.625Ca0.25Br0.125', '-1.872', '-0.205979857271']
BrCaP 1 ['P0.25Ca0.5Br0.25', '-1.699', '-0.234148682367']
BrCaPm 11 ['Ca0.1Br0.6Pm0.3', '-1.678', '-0.231216319042']
BrCaPt 9 ['Ca0.2Br0.6Pt0.2', '-1.604', '-0.244604366036']
BrCaPu 4 ['Ca0.111111Br0.666667Pu0.222222', '-2.041', '-0.23417005191']
BrCaRe 3 ['Ca0.125Br0.75Re0.125', '-1.073', '-0.223377728772']
BrCaS 16 ['S0.142857Ca0.428571Br0.428571', '-2.322', '-0.204498990179']
BrCaSb 22 ['Ca0.5Br0.166667Sb0.333333', '-1.496', '-0.201212311927']
BrCaSc 12 ['Ca0.111111Sc0.333333Br0.555556', '-1.601', '-0.238776269215']
BrCaSe 1 ['Ca0.2Se0.3Br0.5', '-1.625', '-0.217873627294']
BrCaSi 9 ['Si0.222222Ca0.222222Br0.555556', '-1.874', '-0.213644940744']
BrCaTa 3 ['Ca0.125Br0.75Ta0.125', '-1.513', '-0.203827424214']
BrCaTb 6 ['Ca0.111111Br0.666667Tb0.222222', '-1.845', '-0.207610166005']
BrCaTe 7 ['Ca0.285714Br0.285714Te0.428571', '-1.646', '-0.200529860977']
BrCaTm 5 ['Ca0.166667Br0.666667Tm0.166667', '-1.991', '-0.228527133392']
BrCaY 10 ['Ca0.222222Br0.666667Y0.111111', '-2.16', '-0.240051336726']
BrCaYb 2 ['Ca0.111111Br0.555556Yb0.333333', '-2.137', '-0.203124081992']
BrCaZn 5 ['Ca0.222222Zn0.222222Br0.555556', '-1.883', '-0.200284915883']
BrCaZr 1 ['Ca0.111111Br0.777778Zr0.111111', '-1.681', '-0.203277916993']
BrCdCr 4 ['Cr0.125Br0.75Cd0.125', '-0.93', '-0.202953296291']
BrCdDy 22 ['Br0.571429Cd0.285714Dy0.142857', '-1.384', '-0.202433848381']
BrCdEr 32 ['Br0.5Cd0.333333Er0.166667', '-1.145', '-0.21877236229']
BrCdEu 2 ['Br0.555556Cd0.111111Eu0.333333', '-2.302', '-0.20998347005']
BrCdF 10 ['F0.5Br0.375Cd0.125', '-1.53', '-0.206170590234']
BrCdHf 18 ['Br0.5Cd0.1Hf0.4', '-0.765', '-0.246913706791']
BrCdHo 29 ['Br0.428571Cd0.285714Ho0.285714', '-1.095', '-0.20545280883']
BrCdLu 29 ['Br0.6Cd0.3Lu0.1', '-1.256', '-0.234452888048']

BrCdMo 1 ['Br0.8Mo0.1Cd0.1', '-0.846', '-0.200394270791']
BrCdNp 3 ['Br0.75Cd0.125Np0.125', '-1.479', '-0.276804273645']
BrCdO 7 ['O0.555556Br0.333333Cd0.111111', '-0.603', '-0.203766722867']
BrCdOs 3 ['Br0.75Cd0.125Os0.125', '-0.624', '-0.215462730645']
BrCdPa 1 ['Br0.75Cd0.125Pa0.125', '-1.715', '-0.229334336']
BrCdPm 27 ['Br0.444444Cd0.333333Pm0.222222', '-1.168', '-0.23664400948']
BrCdPr 1 ['Br0.555556Cd0.222222Pr0.222222', '-1.841', '-0.211670629396']
BrCdPt 10 ['Br0.666667Cd0.222222Pt0.111111', '-0.948', '-0.221711521147']
BrCdPu 31 ['Br0.3Cd0.4Pu0.3', '-0.931', '-0.210893055776']
BrCdRe 4 ['Br0.714286Cd0.142857Re0.142857', '-0.722', '-0.255100263594']
BrCdRh 1 ['Br0.6Rh0.2Cd0.2', '-1.047', '-0.214946163549']
BrCdSb 1 ['Br0.777778Cd0.111111Sb0.111111', '-0.939', '-0.205642801157']
BrCdSc 24 ['Sc0.4Br0.4Cd0.2', '-1.0', '-0.2158572817']
BrCdSi 5 ['Si0.142857Br0.714286Cd0.142857', '-1.274', '-0.228854894882']
BrCdSm 3 ['Br0.75Cd0.125Sm0.125', '-1.688', '-0.218548068175']
BrCdTa 10 ['Br0.666667Cd0.166667Ta0.166667', '-1.296', '-0.226083292793']
BrCdTb 15 ['Br0.555556Cd0.111111Tb0.333333', '-1.491', '-0.208102461215']
BrCdTe 3 ['Br0.75Cd0.125Te0.125', '-0.69', '-0.230863381171']
BrCdTh 1 ['Br0.6Cd0.2Th0.2', '-1.724', '-0.203699065303']
BrCdTm 28 ['Br0.4Cd0.2Tm0.4', '-1.083', '-0.200511640034']
BrCdU 4 ['Br0.75Cd0.125U0.125', '-1.592', '-0.200882203814']
BrCdV 5 ['V0.2Br0.7Cd0.1', '-1.202', '-0.261865275548']
BrCdY 35 ['Br0.5Y0.125Cd0.375', '-1.13', '-0.202898058433']
BrCdYb 8 ['Br0.5Cd0.166667Yb0.333333', '-2.022', '-0.211631536292']
BrCdZr 2 ['Br0.777778Zr0.111111Cd0.111111', '-1.293', '-0.207353474213']
BrCeCl 7 ['Cl0.25Br0.25Ce0.5', '-1.807', '-0.217477342367']
BrCeCo 4 ['Co0.166667Br0.666667Ce0.166667', '-1.746', '-0.212586410471']
BrCeCr 3 ['Cr0.111111Br0.777778Ce0.111111', '-1.569', '-0.325329871712']
BrCeCu 14 ['Cu0.3Br0.4Ce0.3', '-1.444', '-0.205166895051']
BrCeDy 3 ['Br0.75Ce0.125Dy0.125', '-2.133', '-0.427047426719']
BrCeEr 6 ['Br0.666667Ce0.111111Er0.222222', '-1.866', '-0.436050491711']
BrCeFe 5 ['Fe0.2Br0.7Ce0.1', '-1.471', '-0.216002243057']
BrCeGa 6 ['Ga0.1Br0.8Ce0.1', '-1.501', '-0.209419756548']
BrCeGd 1 ['Br0.777778Ce0.111111Gd0.111111', '-2.179', '-0.265959264498']
BrCeGe 25 ['Ge0.1Br0.5Ce0.4', '-1.841', '-0.21029887121']
BrCeH 18 ['H0.5Br0.1Ce0.4', '-0.941', '-0.204415144292']
BrCeHf 6 ['Br0.666667Ce0.111111Hf0.222222', '-1.368', '-0.265777903934']
BrCeHo 6 ['Br0.666667Ce0.111111Ho0.222222', '-1.804', '-0.208309668481']
BrCeI 38 ['Br0.25I0.375Ce0.375', '-1.659', '-0.204166692172']
BrCeIn 2 ['Br0.777778In0.111111Ce0.111111', '-1.651', '-0.210654789222']
BrCeIr 20 ['Br0.444444Ce0.444444Ir0.111111', '-1.667', '-0.201497612399']
BrCeLa 1 ['Br0.8La0.1Ce0.1', '-1.988', '-0.2202207413']
BrCeLu 6 ['Br0.666667Ce0.111111Lu0.222222', '-1.807', '-0.414496906156']
BrCeMo 4 ['Br0.7Mo0.2Ce0.1', '-1.647', '-0.249912056224']

BrCeN 7 ['N0.3Br0.3Ce0.4', '-1.857', '-0.205673422172']
BrCeNb 4 ['Br0.8Nb0.1Ce0.1', '-1.823', '-0.290497184314']
BrCeNi 17 ['Ni0.142857Br0.428571Ce0.428571', '-1.545', '-0.218850005465']
BrCeNp 2 ['Br0.777778Ce0.111111Np0.111111', '-1.918', '-0.251975184916']
BrCeO 52 ['O0.142857Br0.571429Ce0.285714', '-2.685', '-0.200156177717']
BrCeOs 18 ['Br0.222222Ce0.555556Os0.222222', '-0.873', '-0.20028611013']
BrCeP 16 ['P0.333333Br0.166667Ce0.5', '-1.764', '-0.200205494944']
BrCePd 32 ['Br0.625Pd0.25Ce0.125', '-1.487', '-0.209764511301']
BrCePm 6 ['Br0.666667Ce0.111111Pm0.222222', '-1.887', '-0.358237617822']
BrCePt 47 ['Br0.1Ce0.5Pt0.4', '-1.37', '-0.207351382184']
BrCePu 8 ['Br0.666667Ce0.111111Pu0.222222', '-1.963', '-0.213741755333']
BrCeRb 4 ['Br0.7Rb0.1Ce0.2', '-2.323', '-0.208492085306']
BrCeRe 4 ['Br0.714286Ce0.142857Re0.142857', '-1.509', '-0.274006331463']
BrCeRh 35 ['Br0.6Rh0.1Ce0.3', '-2.09', '-0.239159782298']
BrCeRu 12 ['Br0.444444Ru0.222222Ce0.333333', '-1.525', '-0.203192285983']
BrCeS 47 ['S0.333333Br0.111111Ce0.555556', '-1.967', '-0.20602257867']
BrCeSb 45 ['Br0.571429Sb0.285714Ce0.142857', '-1.594', '-0.20034363457']
BrCeSc 9 ['Sc0.166667Br0.666667Ce0.166667', '-1.969', '-0.224672086304']
BrCeSe 18 ['Se0.4Br0.1Ce0.5', '-1.976', '-0.205376133259']
BrCeSi 47 ['Si0.222222Br0.222222Ce0.555556', '-1.177', '-0.20009139363']
BrCeSm 1 ['Br0.8Ce0.1Sm0.1', '-1.918', '-0.204772702048']
BrCeSn 5 ['Br0.777778Sn0.111111Ce0.111111', '-1.693', '-0.205467972358']
BrCeTa 3 ['Br0.75Ce0.125Ta0.125', '-1.823', '-0.282830235472']
BrCeTb 3 ['Br0.75Ce0.125Tb0.125', '-2.118', '-0.469279949165']
BrCeTc 5 ['Br0.7Tc0.2Ce0.1', '-1.41', '-0.224648425724']
BrCeTe 43 ['Br0.428571Te0.142857Ce0.428571', '-1.867', '-0.203970348131']
BrCeTi 2 ['Ti0.142857Br0.714286Ce0.142857', '-1.99', '-0.201630990888']
BrCeTl 7 ['Br0.4Ce0.4Tl0.2', '-1.54', '-0.212541334034']
BrCeTm 5 ['Br0.714286Ce0.142857Tm0.142857', '-2.016', '-0.241312184344']
BrCeV 4 ['V0.2Br0.7Ce0.1', '-1.731', '-0.253199523056']
BrCeY 6 ['Br0.714286Y0.142857Ce0.142857', '-2.044', '-0.282494670059']
BrCeYb 1 ['Br0.666667Ce0.111111Yb0.222222', '-2.416', '-0.222349603946']
BrCeZn 14 ['Zn0.2Br0.7Ce0.1', '-1.685', '-0.20031266506']
BrCeZr 2 ['Br0.777778Zr0.111111Ce0.111111', '-2.011', '-0.327947082556']
BrClCo 16 ['Cl0.142857Co0.285714Br0.571429', '-0.579', '-0.201277063523']
BrClCs 7 ['Cl0.555556Br0.333333Cs0.111111', '-0.851', '-0.204490174734']
BrClCu 3 ['Cl0.5Cu0.375Br0.125', '-0.853', '-0.213766241888']
BrClDy 18 ['Cl0.555556Br0.222222Dy0.222222', '-2.258', '-0.203306801886']
BrClEr 90 ['Cl0.1Br0.4Er0.5', '-1.334', '-0.22420194247']
BrClEu 10 ['Cl0.333333Br0.166667Eu0.5', '-2.218', '-0.201312719725']
BrClF 49 ['F0.6Cl0.1Br0.3', '-1.204', '-0.20133554115']
BrClFe 1 ['Cl0.285714Fe0.285714Br0.428571', '-1.032', '-0.239436754502']
BrClGd 13 ['Cl0.111111Br0.666667Gd0.222222', '-2.163', '-0.200814318031']
BrClHf 40 ['Cl0.2Br0.4Hf0.4', '-0.967', '-0.214804388279']

BrClHo 88 ['Cl0.1Br0.4Ho0.5', '-1.342', '-0.202471601808']
BrClIn 39 ['Cl0.333333Br0.5In0.166667', '-0.956', '-0.200950800306']
BrClIr 51 ['Cl0.3Br0.6Ir0.1', '-0.515', '-0.200475601606']
BrClK 12 ['Cl0.3K0.2Br0.5', '-1.105', '-0.212894642986']
BrClKr 9 ['Cl0.428571Br0.428571Kr0.142857', '-0.356', '-0.203145050473']
BrClLi 32 ['Li0.142857Cl0.285714Br0.571429', '-0.838', '-0.215664185069']
BrClLu 88 ['Cl0.166667Br0.333333Lu0.5', '-1.275', '-0.213457470758']
BrClMn 15 ['Cl0.5Mn0.125Br0.375', '-0.788', '-0.202071654646']
BrClMo 17 ['Cl0.3Br0.5Mo0.2', '-1.039', '-0.201311685']
BrClNa 29 ['Na0.25Cl0.5Br0.25', '-1.317', '-0.202780543656']
BrClNd 2 ['Cl0.714286Br0.142857Nd0.142857', '-1.743', '-0.200374255688']
BrClNi 26 ['Cl0.4Ni0.1Br0.5', '-0.522', '-0.200102816097']
BrClNp 17 ['Cl0.7Br0.1Np0.2', '-1.998', '-0.201012011256']
BrClO 6 ['O0.2Cl0.3Br0.5', '-0.395', '-0.201951619654']
BrClOs 23 ['Cl0.6Br0.3Os0.1', '-0.606', '-0.201607286973']
BrClP 3 ['P0.125Cl0.5Br0.375', '-0.887', '-0.211927671364']
BrClPb 1 ['Cl0.5Br0.2Pb0.3', '-1.42', '-0.206265845701']
BrClPm 79 ['Cl0.6Br0.3Pm0.1', '-1.156', '-0.203314297395']
BrClPr 8 ['Cl0.571429Br0.285714Pr0.142857', '-1.727', '-0.20371184882']
BrClPt 47 ['Cl0.7Br0.2Pt0.1', '-0.489', '-0.20063337954']
BrClPu 29 ['Cl0.111111Br0.555556Pu0.333333', '-1.842', '-0.205203321739']
BrClRb 31 ['Cl0.3Br0.3Rb0.4', '-1.866', '-0.201337336572']
BrClRe 22 ['Cl0.333333Br0.555556Re0.111111', '-0.627', '-0.209001679053']
BrClRh 42 ['Cl0.6Br0.3Rh0.1', '-0.475', '-0.207408691775']
BrClRu 28 ['Cl0.6Br0.3Ru0.1', '-0.64', '-0.212073810204']
BrClS 12 ['S0.142857Cl0.571429Br0.285714', '-0.49', '-0.20182025374']
BrClSb 12 ['Cl0.2Br0.6Sb0.2', '-0.965', '-0.205162529116']
BrClSc 48 ['Cl0.1Sc0.1Br0.8', '-0.651', '-0.211267118444']
BrClSe 3 ['Cl0.333333Se0.222222Br0.444444', '-0.528', '-0.200384325847']
BrClSi 1 ['Si0.142857Cl0.428571Br0.428571', '-1.227', '-0.216188320816']
BrClSm 37 ['Cl0.571429Br0.142857Sm0.285714', '-2.228', '-0.201065017992']
BrClTa 17 ['Cl0.2Br0.6Ta0.2', '-1.149', '-0.201842082905']
BrClTb 40 ['Cl0.285714Br0.571429Tb0.142857', '-1.326', '-0.204562425716']
BrClTc 24 ['Cl0.5Br0.3Tc0.2', '-0.977', '-0.201746705053']
BrClTe 26 ['Cl0.1Br0.6Te0.3', '-0.425', '-0.202316534325']
BrClTi 12 ['Cl0.4Ti0.2Br0.4', '-1.856', '-0.20436653803']
BrClTl 8 ['Cl0.5Br0.4Tl0.1', '-0.633', '-0.201047653549']
BrClTm 33 ['Cl0.285714Br0.285714Tm0.428571', '-1.748', '-0.203337193419']
BrClV 29 ['Cl0.5V0.1Br0.4', '-0.938', '-0.20126113386']
BrClXe 48 ['Cl0.375Br0.5Xe0.125', '-0.34', '-0.206251919164']
BrClY 45 ['Cl0.142857Br0.714286Y0.142857', '-1.179', '-0.205899109699']
BrClYb 5 ['Cl0.1Br0.6Yb0.3', '-2.35', '-0.200140732981']
BrClZn 1 ['Cl0.111111Zn0.333333Br0.555556', '-1.312', '-0.220094314565']
BrClZr 14 ['Cl0.222222Br0.444444Zr0.333333', '-1.809', '-0.201753073233']

BrCoCr 4 ['Cr0.111111Co0.111111Br0.777778', '-0.621', '-0.214430312259']
BrCoDy 15 ['Co0.1Br0.6Dy0.3', '-1.726', '-0.225200528053']
BrCoEr 34 ['Co0.4Br0.5Er0.1', '-0.639', '-0.205560162291']
BrCoF 14 ['F0.666667Co0.222222Br0.111111', '-1.932', '-0.201241455396']
BrCoFe 2 ['Fe0.166667Co0.166667Br0.666667', '-0.739', '-0.200038992556']
BrCoGd 3 ['Co0.142857Br0.714286Gd0.142857', '-1.606', '-0.222648192201']
BrCoH 22 ['H0.6Co0.1Br0.3', '-0.454', '-0.21779005912']
BrCoHf 25 ['Co0.333333Br0.555556Hf0.111111', '-0.574', '-0.203771064213']
BrCoHo 22 ['Co0.285714Br0.571429Ho0.142857', '-1.039', '-0.257252758141']
BrCoI 21 ['Co0.25Br0.5I0.25', '-0.503', '-0.210841414435']
BrCoLa 4 ['Co0.1Br0.8La0.1', '-1.223', '-0.208611137793']
BrCoLu 29 ['Co0.2Br0.4Lu0.4', '-1.012', '-0.234485567034']
BrCoMo 9 ['Co0.2Br0.7Mo0.1', '-0.751', '-0.210013743309']
BrCoNa 3 ['Na0.2Co0.2Br0.6', '-1.145', '-0.204390669381']
BrCoNp 4 ['Co0.142857Br0.714286Np0.142857', '-1.368', '-0.302240089881']
BrCoO 11 ['O0.5Co0.25Br0.25', '-1.05', '-0.202008144643']
BrCoPm 30 ['Co0.142857Br0.428571Pm0.428571', '-1.304', '-0.208163051463']
BrCoPr 2 ['Co0.111111Br0.777778Pr0.111111', '-1.281', '-0.203716577267']
BrCoPt 10 ['Co0.25Br0.625Pt0.125', '-0.486', '-0.208237071292']
BrCoPu 13 ['Co0.222222Br0.666667Pu0.111111', '-1.182', '-0.208117100612']
BrCoRe 7 ['Co0.1Br0.7Re0.2', '-0.321', '-0.209894828517']
BrCoS 1 ['S0.111111Co0.333333Br0.555556', '-0.625', '-0.220109879434']
BrCoSb 8 ['Co0.142857Br0.714286Sb0.142857', '-0.844', '-0.209290235774']
BrCoSc 28 ['Sc0.428571Co0.142857Br0.428571', '-0.981', '-0.200566370393']
BrCoSe 33 ['Co0.333333Se0.166667Br0.5', '-0.556', '-0.202617812959']
BrCoSi 3 ['Si0.222222Co0.111111Br0.666667', '-1.179', '-0.202062831586']
BrCoSm 2 ['Co0.142857Br0.714286Sm0.142857', '-1.589', '-0.217804426487']
BrCoTa 6 ['Co0.222222Br0.666667Ta0.111111', '-0.85', '-0.215185491075']
BrCoTb 19 ['Co0.111111Br0.555556Tb0.333333', '-1.463', '-0.203638228295']
BrCoTe 17 ['Co0.3Br0.6Te0.1', '-0.554', '-0.2020598393']
BrCoTh 3 ['Co0.125Br0.75Th0.125', '-1.586', '-0.203441205065']
BrCoTm 24 ['Co0.2Br0.4Tm0.4', '-1.083', '-0.214140216533']
BrCoU 1 ['Co0.1Br0.8U0.1', '-1.243', '-0.229606716811']
BrCoV 3 ['V0.125Co0.125Br0.75', '-0.725', '-0.202802967541']
BrCoXe 4 ['Co0.1Br0.6Xe0.3', '-0.319', '-0.207894828517']
BrCoY 29 ['Co0.3Br0.5Y0.2', '-1.095', '-0.208414202293']
BrCoYb 2 ['Co0.166667Br0.666667Yb0.166667', '-1.579', '-0.233499163389']
BrCoZr 8 ['Co0.166667Br0.666667Zr0.166667', '-1.385', '-0.208658927557']
BrCrCs 1 ['Cr0.2Br0.7Cs0.1', '-1.316', '-0.2855951633']
BrCrCu 7 ['Cr0.1Cu0.2Br0.7', '-0.763', '-0.20140941755']
BrCrDy 8 ['Cr0.166667Br0.666667Dy0.166667', '-1.463', '-0.20454326978']
BrCrEr 15 ['Cr0.25Br0.625Er0.125', '-1.168', '-0.266944262864']
BrCrEu 3 ['Cr0.1Br0.7Eu0.2', '-1.933', '-0.223992965353']
BrCrF 6 ['F0.222222Cr0.222222Br0.555556', '-1.454', '-0.222217522069']

BrCrFe 6 ['Cr0.2Fe0.1Br0.7', '-0.979', '-0.201560886309']
BrCrGa 7 ['Cr0.111111Ga0.222222Br0.666667', '-1.181', '-0.231922943387']
BrCrGd 3 ['Cr0.1Br0.8Gd0.1', '-1.44', '-0.327951358541']
BrCrGe 8 ['Cr0.222222Ge0.111111Br0.666667', '-1.051', '-0.22784544325']
BrCrHf 11 ['Cr0.1Br0.6Hf0.3', '-0.771', '-0.324935974792']
BrCrHg 1 ['Cr0.2Br0.7Hg0.1', '-0.968', '-0.263771820554']
BrCrHo 14 ['Cr0.111111Br0.555556Ho0.333333', '-1.25', '-0.221046886991']
BrCrI 24 ['Cr0.2Br0.6I0.2', '-0.833', '-0.200763208065']
BrCrIn 5 ['Cr0.1Br0.7In0.2', '-1.195', '-0.20370972781']
BrCrIr 5 ['Cr0.142857Br0.714286Ir0.142857', '-0.915', '-0.254523598274']
BrCrK 7 ['K0.111111Cr0.111111Br0.777778', '-0.917', '-0.205424100305']
BrCrKr 7 ['Cr0.125Br0.625Kr0.25', '-0.52', '-0.201490565646']
BrCrLa 4 ['Cr0.142857Br0.714286La0.142857', '-1.855', '-0.200583945418']
BrCrLi 3 ['Li0.142857Cr0.142857Br0.714286', '-1.096', '-0.24419406825']
BrCrLu 12 ['Cr0.142857Br0.571429Lu0.285714', '-1.132', '-0.212621765762']
BrCrMg 5 ['Mg0.125Cr0.125Br0.75', '-1.166', '-0.203224746395']
BrCrMo 3 ['Cr0.125Br0.75Mo0.125', '-0.995', '-0.27802067349']
BrCrNa 6 ['Na0.125Cr0.125Br0.75', '-0.993', '-0.225491198363']
BrCrNb 1 ['Cr0.1Br0.8Nb0.1', '-1.224', '-0.301185204807']
BrCrNd 2 ['Cr0.111111Br0.777778Nd0.111111', '-1.441', '-0.208079113103']
BrCrNi 7 ['Cr0.1Ni0.1Br0.8', '-0.638', '-0.208341470034']
BrCrNp 3 ['Cr0.125Br0.75Np0.125', '-1.363', '-0.250832108646']
BrCrO 20 ['O0.428571Cr0.285714Br0.285714', '-1.975', '-0.202336846264']
BrCrOs 5 ['Cr0.2Br0.7Os0.1', '-0.815', '-0.305384905033']
BrCrP 2 ['P0.111111Cr0.111111Br0.777778', '-0.922', '-0.211229751014']
BrCrPa 1 ['Cr0.1Br0.8Pa0.1', '-1.489', '-0.209074829059']
BrCrPb 4 ['Cr0.2Br0.7Pb0.1', '-1.064', '-0.219253009801']
BrCrPd 6 ['Cr0.25Br0.625Pd0.125', '-0.949', '-0.213673116927']
BrCrPm 16 ['Cr0.222222Br0.555556Pm0.222222', '-1.203', '-0.210109003101']
BrCrPr 3 ['Cr0.1Br0.8Pr0.1', '-1.395', '-0.281742543541']
BrCrPt 14 ['Cr0.142857Br0.571429Pt0.285714', '-0.66', '-0.295989217881']
BrCrPu 4 ['Cr0.142857Br0.714286Pu0.142857', '-1.59', '-0.211936571847']
BrCrRb 8 ['Cr0.111111Br0.666667Rb0.222222', '-1.341', '-0.201176550036']
BrCrRe 5 ['Cr0.2Br0.7Re0.1', '-0.716', '-0.206384905033']
BrCrRh 10 ['Cr0.222222Br0.666667Rh0.111111', '-0.986', '-0.221545221722']
BrCrRu 7 ['Cr0.2Br0.7Ru0.1', '-0.98', '-0.254859546809']
BrCrS 11 ['S0.166667Cr0.166667Br0.666667', '-0.745', '-0.207739853123']
BrCrSb 14 ['Cr0.222222Br0.555556Sb0.222222', '-0.895', '-0.205356685787']
BrCrSc 16 ['Sc0.222222Cr0.222222Br0.555556', '-1.042', '-0.212651464769']
BrCrSe 29 ['Cr0.25Se0.375Br0.375', '-0.772', '-0.208884078469']
BrCrSi 6 ['Si0.142857Cr0.142857Br0.714286', '-1.179', '-0.209635652276']
BrCrSm 4 ['Cr0.142857Br0.714286Sm0.142857', '-1.785', '-0.208515317915']
BrCrSn 4 ['Cr0.125Br0.75Sn0.125', '-1.13', '-0.218636494519']
BrCrSr 1 ['Cr0.142857Br0.714286Sr0.142857', '-1.597', '-0.220667932904']

BrCrTa 3 ['Cr0.125Br0.75Ta0.125', '-1.088', '-0.309940261088']
BrCrTb 8 ['Cr0.166667Br0.666667Tb0.166667', '-1.403', '-0.316693226183']
BrCrTc 5 ['Cr0.2Br0.7Tc0.1', '-0.973', '-0.222742900309']
BrCrTe 17 ['Cr0.25Br0.125Te0.625', '-0.481', '-0.217116957448']
BrCrTh 3 ['Cr0.125Br0.75Th0.125', '-1.841', '-0.278813235065']
BrCrTi 2 ['Ti0.111111Cr0.111111Br0.777778', '-1.359', '-0.264765941024']
BrCrTl 5 ['Cr0.222222Br0.666667Tl0.111111', '-1.059', '-0.206404042139']
BrCrTm 12 ['Cr0.222222Br0.666667Tm0.111111', '-1.266', '-0.22944781839']
BrCrU 2 ['Cr0.111111Br0.777778U0.111111', '-1.503', '-0.217338156456']
BrCrV 3 ['V0.125Cr0.125Br0.75', '-0.983', '-0.281174997541']
BrCrW 3 ['Cr0.1Br0.8W0.1', '-0.969', '-0.205012396067']
BrCrXe 7 ['Cr0.222222Br0.666667Xe0.111111', '-0.772', '-0.205761005593']
BrCrY 13 ['Cr0.2Br0.6Y0.2', '-1.264', '-0.241802186552']
BrCrYb 3 ['Cr0.125Br0.75Yb0.125', '-1.405', '-0.216246402542']
BrCrZn 6 ['Cr0.111111Zn0.111111Br0.777778', '-0.828', '-0.20032952115']
BrCrZr 4 ['Cr0.125Br0.75Zr0.125', '-1.425', '-0.293675493491']
BrCsDy 11 ['Br0.6Cs0.3Dy0.1', '-2.042', '-0.301915514259']
BrCsEr 9 ['Br0.8Cs0.1Er0.1', '-1.345', '-0.286907235861']
BrCsF 27 ['F0.428571Br0.142857Cs0.428571', '-2.704', '-0.203410687881']
BrCsFe 1 ['Fe0.166667Br0.666667Cs0.166667', '-1.314', '-0.213203150889']
BrCsGa 7 ['Ga0.222222Br0.555556Cs0.222222', '-1.567', '-0.20275057081']
BrCsGd 1 ['Br0.714286Cs0.142857Gd0.142857', '-2.058', '-0.204522326307']
BrCsHf 18 ['Br0.571429Cs0.142857Hf0.285714', '-1.094', '-0.283003434154']
BrCsHg 3 ['Br0.5Cs0.333333Hg0.166667', '-1.712', '-0.211473766919']
BrCsHo 6 ['Br0.666667Cs0.166667Ho0.166667', '-2.179', '-0.259620407557']
BrCsI 1 ['Br0.333333I0.222222Cs0.444444', '-1.914', '-0.206348073932']
BrCsIr 8 ['Br0.7Cs0.2Ir0.1', '-1.304', '-0.216087511057']
BrCsK 1 ['K0.5Br0.4Cs0.1', '-1.765', '-0.215513622532']
BrCsLu 19 ['Br0.555556Cs0.111111Lu0.333333', '-1.421', '-0.283967664074']
BrCsMo 2 ['Br0.8Mo0.1Cs0.1', '-1.017', '-0.210491621799']
BrCsNb 2 ['Br0.75Nb0.125Cs0.125', '-1.537', '-0.211173326935']
BrCsNi 1 ['Ni0.2Br0.6Cs0.2', '-1.339', '-0.20398985355']
BrCsNp 3 ['Br0.8Cs0.1Np0.1', '-1.416', '-0.283159935039']
BrCsO 2 ['O0.3Br0.1Cs0.6', '-1.518', '-0.216661728992']
BrCsOs 4 ['Br0.714286Cs0.142857Os0.142857', '-0.9', '-0.203239336463']
BrCsP 14 ['P0.142857Br0.714286Cs0.142857', '-1.235', '-0.234196239964']
BrCsPa 7 ['Br0.777778Cs0.111111Pa0.111111', '-1.829', '-0.200876776592']
BrCsPm 19 ['Br0.555556Cs0.111111Pm0.333333', '-1.542', '-0.200578731572']
BrCsPt 6 ['Br0.714286Cs0.142857Pt0.142857', '-1.048', '-0.231459391107']
BrCsPu 7 ['Br0.75Cs0.125Pu0.125', '-1.7', '-0.203038354125']
BrCsRb 1 ['Br0.444444Rb0.111111Cs0.444444', '-1.97', '-0.224870707813']
BrCsRe 9 ['Br0.666667Cs0.222222Re0.111111', '-1.295', '-0.211150078942']
BrCsRh 1 ['Br0.714286Rh0.142857Cs0.142857', '-1.232', '-0.220879163095']
BrCsRu 1 ['Br0.714286Ru0.142857Cs0.142857', '-1.174', '-0.237259091666']

BrCsSb 3 ['Br0.8Sb0.1Cs0.1', '-1.026', '-0.205075872049']
BrCsSc 20 ['Sc0.4Br0.5Cs0.1', '-1.34', '-0.218941188292']
BrCsSe 11 ['Se0.333333Br0.444444Cs0.222222', '-1.18', '-0.201367045592']
BrCsSi 5 ['Si0.166667Br0.666667Cs0.166667', '-1.534', '-0.204956918599']
BrCsSm 2 ['Br0.714286Cs0.142857Sm0.142857', '-2.053', '-0.211678560593']
BrCsTa 6 ['Br0.714286Cs0.142857Ta0.142857', '-1.421', '-0.221676696428']
BrCsTb 4 ['Br0.75Cs0.125Tb0.125', '-1.573', '-0.202452880289']
BrCsTc 7 ['Br0.666667Tc0.166667Cs0.166667', '-1.263', '-0.207506507555']
BrCsTe 36 ['Br0.111111Te0.444444Cs0.444444', '-1.255', '-0.205115902694']
BrCsTh 3 ['Br0.7Cs0.1Th0.2', '-2.088', '-0.202933112561']
BrCsTm 17 ['Br0.571429Cs0.142857Tm0.285714', '-1.608', '-0.214676917904']
BrCsU 2 ['Br0.714286Cs0.142857U0.142857', '-1.886', '-0.202116411133']
BrCsV 7 ['V0.166667Br0.666667Cs0.166667', '-1.473', '-0.210936521929']
BrCsY 8 ['Br0.777778Y0.111111Cs0.111111', '-1.491', '-0.202974556706']
BrCsYb 1 ['Br0.6Cs0.1Yb0.3', '-2.505', '-0.34612657705']
BrCsZr 14 ['Br0.625Zr0.25Cs0.125', '-1.807', '-0.214047091615']
BrCuDy 24 ['Cu0.1Br0.8Dy0.1', '-0.865', '-0.211341991868']
BrCuEr 41 ['Cu0.142857Br0.428571Er0.428571', '-1.108', '-0.20273073682']
BrCuEu 1 ['Cu0.111111Br0.555556Eu0.333333', '-2.27', '-0.209772480918']
BrCuF 2 ['F0.375Cu0.25Br0.375', '-1.27', '-0.200812247214']
BrCuFe 6 ['Fe0.111111Cu0.333333Br0.555556', '-0.698', '-0.200429236991']
BrCuGa 7 ['Cu0.333333Ga0.111111Br0.555556', '-0.863', '-0.201378070045']
BrCuGd 5 ['Cu0.222222Br0.666667Gd0.111111', '-1.423', '-0.206533175333']
BrCuGe 1 ['Cu0.166667Ge0.166667Br0.666667', '-0.963', '-0.225847331723']
BrCuHf 28 ['Cu0.2Br0.5Hf0.3', '-0.611', '-0.229257962292']
BrCuHo 32 ['Cu0.4Br0.5Ho0.1', '-0.92', '-0.259954454957']
BrCuIr 1 ['Cu0.125Br0.75Ir0.125', '-0.672', '-0.22085318599']
BrCuLa 6 ['Cu0.125Br0.75La0.125', '-1.546', '-0.225155989741']
BrCuLu 38 ['Cu0.25Br0.375Lu0.375', '-0.972', '-0.238565483261']
BrCuMo 4 ['Cu0.125Br0.75Mo0.125', '-0.791', '-0.20079071099']
BrCuNd 1 ['Cu0.25Br0.625Nd0.125', '-1.543', '-0.264004609741']
BrCuNp 6 ['Cu0.2Br0.7Np0.1', '-1.16', '-0.218290199434']
BrCuO 19 ['O0.444444Cu0.222222Br0.333333', '-0.646', '-0.201823978881']
BrCuOs 5 ['Cu0.1Br0.8Os0.1', '-0.373', '-0.219608482517']
BrCuP 11 ['P0.111111Cu0.222222Br0.666667', '-0.738', '-0.201000956258']
BrCuPb 2 ['Cu0.2Br0.6Pb0.2', '-1.044', '-0.205361148053']
BrCuPm 36 ['Cu0.25Br0.375Pm0.375', '-1.19', '-0.23114267003']
BrCuPr 5 ['Cu0.142857Br0.714286Pr0.142857', '-1.677', '-0.231512247916']
BrCuPt 7 ['Cu0.1Br0.6Pt0.3', '-0.376', '-0.222608482517']
BrCuPu 28 ['Cu0.111111Br0.555556Pu0.333333', '-1.547', '-0.232487310696']
BrCuRe 6 ['Cu0.222222Br0.666667Re0.111111', '-0.591', '-0.250129961149']
BrCuSb 5 ['Cu0.166667Br0.666667Sb0.166667', '-0.897', '-0.201368009639']
BrCuSc 33 ['Sc0.111111Cu0.444444Br0.444444', '-0.69', '-0.207040252815']
BrCuSi 9 ['Si0.2Cu0.1Br0.7', '-1.181', '-0.22794814628']

BrCuSm 9 ['Cu0.125Br0.75Sm0.125', '-1.46', '-0.207345940676']
BrCuSn 5 ['Cu0.375Br0.5Sn0.125', '-0.807', '-0.214145928873']
BrCuTa 9 ['Cu0.333333Br0.555556Ta0.111111', '-0.905', '-0.247066304862']
BrCuTb 27 ['Cu0.444444Br0.444444Tb0.111111', '-0.842', '-0.201972213075']
BrCuTe 12 ['Cu0.2Br0.7Te0.1', '-0.559', '-0.211737485455']
BrCuTh 4 ['Cu0.142857Br0.714286Th0.142857', '-1.742', '-0.200386578276']
BrCuTm 30 ['Cu0.3Br0.4Tm0.3', '-1.06', '-0.208954764368']
BrCuU 7 ['Cu0.3Br0.6U0.1', '-1.193', '-0.206524357552']
BrCuV 9 ['V0.2Cu0.1Br0.7', '-0.995', '-0.228303573549']
BrCuW 4 ['Cu0.2Br0.7W0.1', '-0.805', '-0.203028139413']
BrCuY 43 ['Cu0.333333Br0.333333Y0.333333', '-1.0', '-0.225431483861']
BrCuYb 17 ['Cu0.142857Br0.571429Yb0.285714', '-2.195', '-0.205870484334']
BrCuZr 9 ['Cu0.333333Br0.555556Zr0.111111', '-1.119', '-0.209414311992']
BrDyEr 11 ['Br0.6Dy0.1Er0.3', '-1.521', '-0.387045025125']
BrDyEu 6 ['Br0.8Eu0.1Dy0.1', '-1.482', '-0.254633765769']
BrDyF 20 ['F0.333333Br0.5Dy0.166667', '-2.315', '-0.202345621413']
BrDyFe 11 ['Fe0.25Br0.625Dy0.125', '-1.214', '-0.222571084533']
BrDyGa 11 ['Ga0.25Br0.625Dy0.125', '-1.397', '-0.217131271609']
BrDyGd 3 ['Br0.75Gd0.125Dy0.125', '-2.098', '-0.401115519219']
BrDyGe 22 ['Ge0.285714Br0.428571Dy0.285714', '-1.407', '-0.200110631585']
BrDyH 43 ['H0.375Br0.25Dy0.375', '-1.217', '-0.202586307745']
BrDyHf 11 ['Br0.6Dy0.1Hf0.3', '-0.962', '-0.270477031626']
BrDyHg 20 ['Br0.8Dy0.1Hg0.1', '-0.898', '-0.203120424871']
BrDyHo 8 ['Br0.666667Dy0.166667Ho0.166667', '-1.686', '-0.261502396723']
BrDyI 81 ['Br0.8I0.1Dy0.1', '-0.788', '-0.226422660867']
BrDyIn 15 ['Br0.6In0.2Dy0.2', '-1.547', '-0.209096154219']
BrDyIr 26 ['Br0.571429Dy0.142857Ir0.285714', '-1.119', '-0.206689838383']
BrDyK 11 ['K0.3Br0.6Dy0.1', '-1.897', '-0.239901222625']
BrDyKr 15 ['Br0.555556Kr0.333333Dy0.111111', '-0.83', '-0.274148343723']
BrDyLa 3 ['Br0.75La0.125Dy0.125', '-2.187', '-0.432562273284']
BrDyLi 11 ['Li0.25Br0.625Dy0.125', '-1.699', '-0.220025374834']
BrDyLu 11 ['Br0.6Dy0.1Lu0.3', '-1.325', '-0.241597684626']
BrDyMg 6 ['Mg0.1Br0.8Dy0.1', '-1.311', '-0.29532085395']
BrDyMn 7 ['Mn0.111111Br0.666667Dy0.222222', '-1.656', '-0.203992799823']
BrDyMo 6 ['Br0.7Mo0.2Dy0.1', '-1.385', '-0.299661407726']
BrDyN 36 ['N0.375Br0.125Dy0.5', '-1.886', '-0.216910817587']
BrDyNa 12 ['Na0.1Br0.8Dy0.1', '-1.087', '-0.227534015525']
BrDyNb 6 ['Br0.666667Nb0.111111Dy0.222222', '-1.692', '-0.229269817605']
BrDyNd 3 ['Br0.75Nd0.125Dy0.125', '-2.01', '-0.316140323284']
BrDyNi 25 ['Ni0.333333Br0.555556Dy0.111111', '-1.05', '-0.202730039585']
BrDyNp 4 ['Br0.714286Dy0.142857Np0.142857', '-1.824', '-0.202295348216']
BrDyO 39 ['O0.5Br0.375Dy0.125', '-1.507', '-0.20207074001']
BrDyOs 16 ['Br0.666667Dy0.111111Os0.222222', '-0.761', '-0.205148343723']
BrDyP 32 ['P0.1Br0.5Dy0.4', '-1.801', '-0.202144454816']

BrDyPa 5 ['Br0.7Dy0.2Pa0.1', '-1.843', '-0.209933033853']
BrDyPb 15 ['Br0.6Dy0.2Pb0.2', '-1.568', '-0.23233512347']
BrDyPd 33 ['Br0.428571Pd0.142857Dy0.428571', '-1.545', '-0.207021129086']
BrDyPm 10 ['Br0.625Pm0.25Dy0.125', '-1.548', '-0.283428666709']
BrDyPr 3 ['Br0.75Pr0.125Dy0.125', '-2.096', '-0.397604500469']
BrDyPt 35 ['Br0.428571Dy0.428571Pt0.142857', '-1.653', '-0.201612075841']
BrDyPu 10 ['Br0.714286Dy0.142857Pu0.142857', '-1.966', '-0.237280938753']
BrDyRb 11 ['Br0.8Rb0.1Dy0.1', '-1.153', '-0.267216730609']
BrDyRe 8 ['Br0.666667Dy0.166667Re0.166667', '-1.053', '-0.219222515585']
BrDyRh 33 ['Br0.5Rh0.375Dy0.125', '-1.087', '-0.238674129835']
BrDyRu 16 ['Br0.6Ru0.1Dy0.3', '-1.702', '-0.201200528053']
BrDyS 50 ['S0.428571Br0.428571Dy0.142857', '-1.356', '-0.207677228436']
BrDySb 39 ['Br0.555556Sb0.333333Dy0.111111', '-1.142', '-0.215935384307']
BrDySc 14 ['Sc0.2Br0.6Dy0.2', '-1.565', '-0.200264658219']
BrDySe 61 ['Se0.375Br0.25Dy0.375', '-2.006', '-0.21210888502']
BrDySi 21 ['Si0.1Br0.5Dy0.4', '-1.639', '-0.20980677619']
BrDySm 3 ['Br0.75Sm0.125Dy0.125', '-2.05', '-0.363752224219']
BrDySn 11 ['Br0.6Sn0.1Dy0.3', '-1.732', '-0.228523411305']
BrDySr 6 ['Br0.8Sr0.1Dy0.1', '-1.438', '-0.229108609867']
BrDyTa 5 ['Br0.714286Dy0.142857Ta0.142857', '-1.531', '-0.29113323672']
BrDyTb 6 ['Br0.7Tb0.1Dy0.2', '-1.863', '-0.40798654601']
BrDyTc 12 ['Br0.555556Tc0.111111Dy0.333333', '-1.6', '-0.210370859308']
BrDyTe 58 ['Br0.3Te0.6Dy0.1', '-0.805', '-0.207477568002']
BrDyTh 3 ['Br0.75Dy0.125Th0.125', '-2.13', '-0.260989556108']
BrDyTi 4 ['Ti0.1Br0.7Dy0.2', '-1.839', '-0.257422911098']
BrDyTl 27 ['Br0.444444Dy0.444444Tl0.111111', '-1.401', '-0.200121122323']
BrDyTm 9 ['Br0.666667Dy0.222222Tm0.111111', '-1.736', '-0.204534573021']
BrDyU 3 ['Br0.75Dy0.125U0.125', '-1.834', '-0.226086359858']
BrDyV 6 ['V0.2Br0.7Dy0.1', '-1.401', '-0.287428600383']
BrDyW 5 ['Br0.7Dy0.2W0.1', '-1.578', '-0.29503673429']
BrDyXe 12 ['Br0.6Xe0.3Dy0.1', '-0.735', '-0.234733509351']
BrDyY 9 ['Br0.666667Y0.111111Dy0.222222', '-1.75', '-0.228787617466']
BrDyYb 7 ['Br0.8Dy0.1Yb0.1', '-1.443', '-0.246538178868']
BrDyZn 23 ['Zn0.111111Br0.555556Dy0.333333', '-1.684', '-0.228934037549']
BrDyZr 6 ['Br0.666667Zr0.111111Dy0.222222', '-1.828', '-0.226458778577']
BrErEu 11 ['Br0.6Eu0.1Er0.3', '-1.704', '-0.343211772192']
BrErF 32 ['F0.111111Br0.444444Er0.444444', '-1.738', '-0.219401844204']
BrErFe 25 ['Fe0.333333Br0.555556Er0.111111', '-0.902', '-0.233409240324']
BrErGa 53 ['Ga0.4Br0.2Er0.4', '-1.0', '-0.202008938739']
BrErGd 6 ['Br0.666667Gd0.111111Er0.222222', '-1.787', '-0.365111018378']
BrErGe 48 ['Ge0.4Br0.2Er0.4', '-1.09', '-0.213077697117']
BrErH 69 ['H0.285714Br0.285714Er0.428571', '-1.118', '-0.216993931542']
BrErHf 19 ['Br0.555556Er0.222222Hf0.222222', '-1.075', '-0.463929657814']
BrErHg 39 ['Br0.444444Er0.111111Hg0.444444', '-0.8', '-0.20499104727']

BrErHo 17 ['Br0.555556Ho0.111111Er0.333333', '-1.329', '-0.231088271619']
BrErI 82 ['Br0.4I0.1Er0.5', '-1.246', '-0.21639552539']
BrErIn 29 ['Br0.444444In0.111111Er0.444444', '-1.179', '-0.209139331008']
BrErIr 33 ['Br0.3Er0.4Ir0.3', '-1.14', '-0.211707467989']
BrErK 19 ['K0.111111Br0.555556Er0.333333', '-1.474', '-0.341445281702']
BrErKr 32 ['Br0.428571Kr0.428571Er0.142857', '-0.514', '-0.21224357894']
BrErLa 6 ['Br0.666667La0.111111Er0.222222', '-1.866', '-0.392952577547']
BrErLi 19 ['Li0.222222Br0.666667Er0.111111', '-1.389', '-0.395508106416']
BrErLu 19 ['Br0.555556Er0.111111Lu0.333333', '-1.201', '-0.318371867259']
BrErMg 11 ['Mg0.222222Br0.666667Er0.111111', '-1.759', '-0.378939104951']
BrErMn 20 ['Mn0.25Br0.625Er0.125', '-1.247', '-0.21727938442']
BrErMo 14 ['Br0.571429Mo0.142857Er0.285714', '-1.25', '-0.266064032309']
BrErN 48 ['N0.333333Br0.5Er0.166667', '-0.839', '-0.210211875777']
BrErNa 23 ['Na0.375Br0.5Er0.125', '-1.822', '-0.210965029725']
BrErNb 11 ['Br0.555556Nb0.111111Er0.333333', '-1.287', '-0.231874814352']
BrErNd 6 ['Br0.666667Nd0.111111Er0.222222', '-1.813', '-0.393799733102']
BrErNi 39 ['Ni0.428571Br0.428571Er0.142857', '-0.853', '-0.256944049679']
BrErNp 8 ['Br0.666667Er0.166667Np0.166667', '-1.643', '-0.232739566097']
BrErO 40 ['O0.4Br0.3Er0.3', '-2.872', '-0.203827561399']
BrErOs 27 ['Br0.3Er0.5Os0.2', '-0.923', '-0.210336173774']
BrErP 61 ['P0.285714Br0.571429Er0.142857', '-1.149', '-0.212553076887']
BrErPa 10 ['Br0.666667Er0.111111Pa0.222222', '-1.629', '-0.222941330196']
BrErPb 33 ['Br0.285714Er0.428571Pb0.285714', '-0.999', '-0.205616357849']
BrErPd 42 ['Br0.3Pd0.4Er0.3', '-1.143', '-0.242967063489']
BrErPm 19 ['Br0.555556Pm0.333333Er0.111111', '-1.334', '-0.246982934758']
BrErPr 6 ['Br0.666667Pr0.111111Er0.222222', '-1.864', '-0.4407678906']
BrErPt 61 ['Br0.166667Er0.5Pt0.333333', '-1.431', '-0.202753169597']
BrErPu 19 ['Br0.5Er0.3Pu0.2', '-1.398', '-0.214938579627']
BrErRb 19 ['Br0.555556Rb0.111111Er0.333333', '-1.43', '-0.297549707813']
BrErRe 21 ['Br0.571429Er0.142857Re0.285714', '-0.526', '-0.22424357894']
BrErRh 38 ['Br0.5Rh0.375Er0.125', '-1.006', '-0.290551064209']
BrErRu 26 ['Br0.6Ru0.3Er0.1', '-0.819', '-0.248561574883']
BrErS 67 ['S0.222222Br0.222222Er0.555556', '-1.625', '-0.223316955236']
BrErSb 46 ['Br0.3Sb0.2Er0.5', '-1.281', '-0.208781456774']
BrErSc 23 ['Sc0.375Br0.5Er0.125', '-1.149', '-0.202083705666']
BrErSe 71 ['Se0.571429Br0.142857Er0.285714', '-1.618', '-0.203298377512']
BrErSi 40 ['Si0.3Br0.2Er0.5', '-1.132', '-0.213249399516']
BrErSm 6 ['Br0.666667Sm0.111111Er0.222222', '-1.81', '-0.397565867267']
BrErSn 31 ['Br0.571429Sn0.285714Er0.142857', '-1.195', '-0.203183395136']
BrErSr 11 ['Br0.6Sr0.1Er0.3', '-1.634', '-0.29168661629']
BrErTa 10 ['Br0.7Er0.1Ta0.2', '-1.253', '-0.306490017965']
BrErTb 11 ['Br0.6Tb0.1Er0.3', '-1.326', '-0.237831043082']
BrErTc 27 ['Br0.4Tc0.2Er0.4', '-1.053', '-0.208082021032']
BrErTe 69 ['Br0.142857Te0.428571Er0.428571', '-1.499', '-0.204142846797']

BrErTh 5 ['Br0.714286Er0.142857Th0.142857', '-1.934', '-0.210898058276']
BrErTi 15 ['Ti0.25Br0.625Er0.125', '-1.456', '-0.223556285566']
BrErTl 37 ['Br0.428571Er0.142857Tl0.428571', '-1.15', '-0.214487548249']
BrErTm 16 ['Br0.555556Er0.333333Tm0.111111', '-1.338', '-0.214139569768']
BrErU 7 ['Br0.666667Er0.166667U0.166667', '-1.593', '-0.236086167557']
BrErV 14 ['V0.142857Br0.571429Er0.285714', '-1.283', '-0.241412222903']
BrErW 14 ['Br0.6Er0.2W0.2', '-1.091', '-0.254846956383']
BrErXe 34 ['Br0.4Xe0.4Er0.2', '-0.639', '-0.216541010516']
BrErY 19 ['Br0.555556Y0.222222Er0.222222', '-1.378', '-0.24796158699']
BrErYb 13 ['Br0.6Er0.1Yb0.3', '-2.339', '-0.250414008551']
BrErZn 36 ['Zn0.25Br0.375Er0.375', '-1.069', '-0.215782014897']
BrErZr 17 ['Br0.571429Zr0.142857Er0.285714', '-1.48', '-0.246695560761']
BrEuGa 2 ['Ga0.166667Br0.666667Eu0.166667', '-1.926', '-0.222482310933']
BrEuGe 12 ['Ge0.2Br0.7Eu0.1', '-1.504', '-0.208918446773']
BrEuHf 11 ['Br0.6Eu0.1Hf0.3', '-1.122', '-0.203643778693']
BrEuHo 12 ['Br0.625Eu0.25Ho0.125', '-2.293', '-0.203758567203']
BrEuI 4 ['Br0.3I0.3Eu0.4', '-2.134', '-0.202596323396']
BrEuIn 11 ['Br0.666667In0.111111Eu0.222222', '-2.161', '-0.20670323317']
BrEuIr 15 ['Br0.6Eu0.3Ir0.1', '-2.385', '-0.203700769254']
BrEuK 2 ['K0.111111Br0.555556Eu0.333333', '-2.251', '-0.206766389549']
BrEuLi 20 ['Li0.222222Br0.555556Eu0.222222', '-2.201', '-0.205826564549']
BrEuLu 11 ['Br0.6Eu0.1Lu0.3', '-1.586', '-0.275764431693']
BrEuMg 1 ['Mg0.1Br0.6Eu0.3', '-2.388', '-0.206700769254']
BrEuN 27 ['N0.125Br0.5Eu0.375', '-2.199', '-0.236652976627']
BrEuNa 9 ['Na0.2Br0.5Eu0.3', '-2.018', '-0.200250641045']
BrEuNi 11 ['Ni0.111111Br0.555556Eu0.333333', '-2.29', '-0.207564784703']
BrEuNp 5 ['Br0.7Eu0.2Np0.1', '-2.31', '-0.220873747236']
BrEuO 23 ['O0.222222Br0.555556Eu0.222222', '-1.89', '-0.205413100996']
BrEuOs 6 ['Br0.8Eu0.1Os0.1', '-0.968', '-0.240900256418']
BrEuP 17 ['P0.142857Br0.428571Eu0.428571', '-2.313', '-0.211422435403']
BrEuPb 4 ['Br0.625Eu0.125Pb0.25', '-1.755', '-0.217753016962']
BrEuPd 13 ['Br0.3Pd0.3Eu0.4', '-1.781', '-0.211354695377']
BrEuPm 11 ['Br0.6Pm0.3Eu0.1', '-1.72', '-0.225814392442']
BrEuPt 24 ['Br0.375Eu0.375Pt0.25', '-2.058', '-0.204361558075']
BrEuPu 13 ['Br0.6Eu0.2Pu0.2', '-2.142', '-0.214575944687']
BrEuRe 6 ['Br0.666667Eu0.222222Re0.111111', '-1.837', '-0.22122279204']
BrEuRh 12 ['Br0.5Rh0.2Eu0.3', '-2.114', '-0.213558163795']
BrEuRu 1 ['Br0.7Ru0.1Eu0.2', '-1.87', '-0.200275154611']
BrEuS 20 ['S0.2Br0.4Eu0.4', '-2.643', '-0.200666634032']
BrEuSb 42 ['Br0.111111Sb0.333333Eu0.555556', '-1.508', '-0.207980895927']
BrEuSc 13 ['Sc0.125Br0.625Eu0.25', '-2.273', '-0.227624165743']
BrEuSe 45 ['Se0.1Br0.4Eu0.5', '-2.103', '-0.207423633836']
BrEuSi 25 ['Si0.1Br0.4Eu0.5', '-1.81', '-0.200688531836']
BrEuSn 1 ['Br0.666667Sn0.111111Eu0.222222', '-2.24', '-0.286584287687']

BrEuTa 3 ['Br0.777778Eu0.111111Ta0.111111', '-1.498', '-0.281622236413']
BrEuTb 9 ['Br0.8Eu0.1Tb0.1', '-1.408', '-0.226419783726']
BrEuTc 6 ['Br0.666667Tc0.166667Eu0.166667', '-1.691', '-0.21178708878']
BrEuTe 44 ['Br0.5Te0.375Eu0.125', '-1.265', '-0.204327272101']
BrEuTl 24 ['Br0.4Eu0.4Tl0.2', '-1.881', '-0.201899127836']
BrEuTm 8 ['Br0.777778Eu0.111111Tm0.111111', '-1.625', '-0.205238460216']
BrEuV 3 ['V0.125Br0.75Eu0.125', '-1.595', '-0.302809752418']
BrEuY 11 ['Br0.6Y0.3Eu0.1', '-1.766', '-0.218307254951']
BrEuYb 8 ['Br0.666667Eu0.166667Yb0.166667', '-2.573', '-0.200841543225']
BrEuZn 17 ['Zn0.125Br0.5Eu0.375', '-2.118', '-0.205702477295']
BrEuZr 4 ['Br0.8Zr0.1Eu0.1', '-1.616', '-0.238648198694']
BrFFe 7 ['F0.625Fe0.125Br0.25', '-1.831', '-0.231751904092']
BrFGa 2 ['F0.6Ga0.1Br0.3', '-1.834', '-0.20161040278']
BrFGd 2 ['F0.714286Br0.142857Gd0.142857', '-3.059', '-0.224053353608']
BrFGe 9 ['F0.375Ge0.25Br0.375', '-1.89', '-0.205067774263']
BrFH 5 ['H0.2F0.5Br0.3', '-1.338', '-0.204763172756']
BrFHf 35 ['F0.428571Br0.285714Hf0.285714', '-2.525', '-0.216953439408']
BrFHo 26 ['F0.4Br0.4Ho0.2', '-2.91', '-0.212981962864']
BrFI 7 ['F0.625Br0.25I0.125', '-1.479', '-0.252941122537']
BrFIn 1 ['F0.7Br0.2In0.1', '-2.032', '-0.276316403787']
BrFIr 15 ['F0.571429Br0.285714Ir0.142857', '-1.466', '-0.233773440864']
BrFK 15 ['F0.375K0.25Br0.375', '-1.916', '-0.205083070034']
BrFLa 2 ['F0.444444Br0.333333La0.222222', '-3.541', '-0.217540340098']
BrFLi 26 ['Li0.125F0.375Br0.5', '-1.391', '-0.200614850124']
BrFLu 50 ['F0.3Br0.2Lu0.5', '-2.212', '-0.204749018277']
BrFMn 2 ['F0.777778Mn0.111111Br0.111111', '-2.133', '-0.209170184017']
BrFMo 6 ['F0.714286Br0.142857Mo0.142857', '-2.531', '-0.208775580089']
BrFN 1 ['N0.1F0.8Br0.1', '-0.964', '-0.208195170694']
BrFNa 16 ['F0.5Na0.375Br0.125', '-2.636', '-0.204044877516']
BrFNb 3 ['F0.777778Br0.111111Nb0.111111', '-2.739', '-0.210775177161']
BrFNd 1 ['F0.666667Br0.166667Nd0.166667', '-3.44', '-0.243401407664']
BrFNi 18 ['F0.4Ni0.3Br0.3', '-1.531', '-0.201083953865']
BrFNp 5 ['F0.7Br0.2Np0.1', '-2.608', '-0.238231996325']
BrFO 11 ['O0.3F0.4Br0.3', '-0.908', '-0.209200907467']
BrFOs 18 ['F0.5Br0.4Os0.1', '-1.343', '-0.20286833439']
BrFPa 25 ['F0.285714Br0.571429Pa0.142857', '-2.104', '-0.20671634742']
BrFPb 11 ['F0.555556Br0.222222Pb0.222222', '-2.16', '-0.209110327061']
BrFPd 6 ['F0.714286Br0.142857Pd0.142857', '-1.527', '-0.200305138926']
BrFPm 38 ['F0.5Br0.2Pm0.3', '-3.328', '-0.207565139364']
BrFPt 26 ['F0.5Br0.375Pt0.125', '-1.137', '-0.201800411062']
BrFPu 14 ['F0.555556Br0.333333Pu0.111111', '-2.308', '-0.21522325197']
BrFRb 41 ['F0.375Br0.25Rb0.375', '-2.345', '-0.201963759073']
BrFRe 28 ['F0.6Br0.3Re0.1', '-1.748', '-0.200479129664']
BrFRh 7 ['F0.625Br0.25Rh0.125', '-1.549', '-0.235003748481']

BrFRu 20 ['F0.444444Br0.444444Ru0.111111', '-1.269', '-0.2096208015']
BrFSb 4 ['F0.4Br0.4Sb0.2', '-1.869', '-0.222267415929']
BrFSc 40 ['F0.444444Sc0.333333Br0.222222', '-3.069', '-0.201503822901']
BrFSe 4 ['F0.8Se0.1Br0.1', '-1.803', '-0.200141716707']
BrFSn 2 ['F0.666667Br0.222222Sn0.111111', '-2.075', '-0.239356958526']
BrFSr 2 ['F0.6Br0.2Sr0.2', '-3.049', '-0.203074638359']
BrFTa 8 ['F0.125Br0.75Ta0.125', '-1.103', '-0.214956352962']
BrFTb 18 ['F0.166667Br0.5Tb0.333333', '-2.244', '-0.221995086331']
BrFTc 19 ['F0.2Br0.6Tc0.2', '-1.128', '-0.2083583784']
BrFTe 9 ['F0.125Br0.625Te0.25', '-0.676', '-0.217760921863']
BrFTi 13 ['F0.5Ti0.2Br0.3', '-2.87', '-0.221081521275']
BrFTl 4 ['F0.666667Br0.166667Tl0.166667', '-1.845', '-0.206958671278']
BrFTm 82 ['F0.2Br0.3Tm0.5', '-1.778', '-0.20640588045']
BrFU 2 ['F0.8Br0.1U0.1', '-3.015', '-0.204564627536']
BrFV 12 ['F0.7V0.2Br0.1', '-2.941', '-0.20171407111']
BrFXe 27 ['F0.3Br0.5Xe0.2', '-0.688', '-0.20450084802']
BrFY 41 ['F0.6Br0.2Y0.2', '-3.56', '-0.201508316184']
BrFYb 58 ['F0.2Br0.3Yb0.5', '-2.4', '-0.20029104695']
BrFZn 1 ['F0.555556Zn0.111111Br0.333333', '-1.602', '-0.209074069106']
BrFZr 2 ['F0.666667Br0.222222Zr0.111111', '-2.759', '-0.200318262487']
BrFeGd 1 ['Fe0.125Br0.75Gd0.125', '-1.617', '-0.210668609124']
BrFeH 1 ['H0.375Fe0.125Br0.5', '-0.649', '-0.208094514496']
BrFeHf 20 ['Fe0.222222Br0.555556Hf0.222222', '-0.723', '-0.202485978843']
BrFeHo 23 ['Fe0.285714Br0.571429Ho0.142857', '-1.047', '-0.205148527426']
BrFeI 11 ['Fe0.2Br0.3I0.5', '-0.535', '-0.20311516479']
BrFeIr 1 ['Fe0.111111Br0.777778Ir0.111111', '-0.738', '-0.209833386167']
BrFeLa 5 ['Fe0.2Br0.7La0.1', '-1.507', '-0.213214120309']
BrFeLu 24 ['Fe0.3Br0.5Lu0.2', '-0.883', '-0.201366141792']
BrFeMo 5 ['Fe0.111111Br0.666667Mo0.222222', '-1.035', '-0.226285033019']
BrFeNd 1 ['Fe0.125Br0.75Nd0.125', '-1.66', '-0.256693413189']
BrFeNi 6 ['Fe0.125Ni0.125Br0.75', '-0.759', '-0.20565624849']
BrFeNp 5 ['Fe0.1Br0.7Np0.2', '-1.585', '-0.217520921558']
BrFeO 28 ['O0.444444Fe0.444444Br0.111111', '-1.629', '-0.20285696406']
BrFeOs 1 ['Fe0.1Br0.8Os0.1', '-0.499', '-0.231175981275']
BrFeP 3 ['P0.1Fe0.2Br0.7', '-0.886', '-0.218900386163']
BrFePm 23 ['Fe0.25Br0.5Pm0.25', '-1.123', '-0.239697911916']
BrFePr 4 ['Fe0.2Br0.7Pr0.1', '-1.458', '-0.209047902057']
BrFePt 13 ['Fe0.1Br0.6Pt0.3', '-0.485', '-0.217175981275']
BrFePu 11 ['Fe0.2Br0.7Pu0.1', '-1.318', '-0.217660958809']
BrFeRe 3 ['Fe0.125Br0.75Re0.125', '-0.562', '-0.227219976594']
BrFeRh 6 ['Fe0.25Br0.625Rh0.125', '-0.806', '-0.205524453774']
BrFeRu 3 ['Fe0.1Br0.8Ru0.1', '-0.686', '-0.20265062305']
BrFeS 2 ['S0.142857Fe0.285714Br0.571429', '-0.889', '-0.216054446379']
BrFeSb 4 ['Fe0.166667Br0.666667Sb0.166667', '-0.92', '-0.201971315473']

BrFeSc 25 ['Sc0.333333Fe0.222222Br0.444444', '-0.917', '-0.201523235593']
BrFeSe 16 ['Fe0.125Se0.25Br0.625', '-0.695', '-0.201745610365']
BrFeSm 5 ['Fe0.142857Br0.714286Sm0.142857', '-1.709', '-0.217595965058']
BrFeTa 2 ['Fe0.111111Br0.777778Ta0.111111', '-0.996', '-0.289928597365']
BrFeTb 13 ['Fe0.2Br0.6Tb0.2', '-1.227', '-0.205698598818']
BrFeTc 3 ['Fe0.111111Br0.777778Tc0.111111', '-0.777', '-0.212037751723']
BrFeTh 3 ['Fe0.125Br0.75Th0.125', '-1.699', '-0.211258801315']
BrFeTm 24 ['Fe0.2Br0.4Tm0.4', '-1.064', '-0.234448107533']
BrFeV 2 ['V0.1Fe0.1Br0.8', '-0.807', '-0.232523526791']
BrFeY 28 ['Fe0.1Br0.4Y0.5', '-1.134', '-0.220483323282']
BrFeZr 4 ['Fe0.142857Br0.714286Zr0.142857', '-1.452', '-0.24413835399']
BrGaGd 4 ['Ga0.142857Br0.714286Gd0.142857', '-1.857', '-0.210925765951']
BrGaHf 12 ['Ga0.142857Br0.571429Hf0.285714', '-0.902', '-0.200804521832']
BrGaHo 30 ['Ga0.25Br0.375Ho0.375', '-1.156', '-0.206646449094']
BrGaI 15 ['Ga0.428571Br0.285714I0.285714', '-0.877', '-0.204901305461']
BrGaIr 6 ['Ga0.1Br0.7Ir0.2', '-0.935', '-0.231214079558']
BrGaK 1 ['K0.285714Ga0.142857Br0.571429', '-1.776', '-0.22439680465']
BrGaLa 3 ['Ga0.1Br0.8La0.1', '-1.598', '-0.2676316338']
BrGaLu 52 ['Ga0.571429Br0.142857Lu0.285714', '-0.839', '-0.20326836144']
BrGaNb 1 ['Ga0.125Br0.75Nb0.125', '-1.329', '-0.224536885911']
BrGaNd 2 ['Ga0.428571Br0.285714Nd0.285714', '-1.439', '-0.229270872644']
BrGaNi 5 ['Ni0.111111Ga0.222222Br0.666667', '-1.152', '-0.202922943387']
BrGaNp 5 ['Ga0.222222Br0.666667Np0.111111', '-1.445', '-0.247841032903']
BrGaO 3 ['O0.142857Ga0.285714Br0.571429', '-1.516', '-0.202303011824']
BrGaOs 3 ['Ga0.125Br0.75Os0.125', '-0.782', '-0.248144155655']
BrGaP 1 ['P0.142857Ga0.142857Br0.714286', '-1.051', '-0.220944362689']
BrGaPa 1 ['Ga0.111111Br0.777778Pa0.111111', '-1.709', '-0.242190562982']
BrGaPm 55 ['Ga0.666667Br0.111111Pm0.222222', '-0.624', '-0.207929815079']
BrGaPr 10 ['Ga0.4Br0.3Pr0.3', '-1.482', '-0.208224055524']
BrGaPt 13 ['Ga0.285714Br0.571429Pt0.142857', '-1.15', '-0.202416883697']
BrGaPu 38 ['Ga0.4Br0.5Pu0.1', '-1.212', '-0.207152277918']
BrGaRb 1 ['Ga0.3Br0.6Rb0.1', '-1.385', '-0.261956046613']
BrGaRe 3 ['Ga0.125Br0.75Re0.125', '-0.791', '-0.257144155655']
BrGaRh 18 ['Ga0.1Br0.8Rh0.1', '-0.895', '-0.200324016299']
BrGaRu 5 ['Ga0.1Br0.7Ru0.2', '-0.915', '-0.200548180224']
BrGaS 12 ['S0.333333Ga0.166667Br0.5', '-0.912', '-0.20019220754']
BrGaSb 5 ['Ga0.1Br0.8Sb0.1', '-0.963', '-0.202723661049']
BrGaSc 36 ['Sc0.222222Ga0.333333Br0.444444', '-1.084', '-0.205458958994']
BrGaSe 24 ['Ga0.4Se0.4Br0.2', '-0.92', '-0.202506392849']
BrGaSi 8 ['Si0.1Ga0.3Br0.6', '-1.103', '-0.217967390425']
BrGaSm 9 ['Ga0.571429Br0.142857Sm0.285714', '-1.099', '-0.202890714416']
BrGaTa 3 ['Ga0.125Br0.75Ta0.125', '-1.258', '-0.264593851097']
BrGaTb 30 ['Ga0.285714Br0.571429Tb0.142857', '-1.253', '-0.210482158423']
BrGaTc 2 ['Ga0.1Br0.8Tc0.1', '-0.907', '-0.239273319799']

BrGaTe 23 ['Ga0.333333Br0.444444Te0.222222', '-0.973', '-0.200321762793']
BrGaTh 2 ['Ga0.1Br0.8Th0.1', '-1.674', '-0.251973460059']
BrGaTl 1 ['Ga0.111111Br0.555556Tl0.333333', '-1.267', '-0.230685922821']
BrGaTm 43 ['Ga0.2Br0.3Tm0.5', '-1.065', '-0.202169042774']
BrGaU 1 ['Ga0.1Br0.8U0.1', '-1.538', '-0.208627212818']
BrGaV 3 ['V0.125Ga0.125Br0.75', '-1.226', '-0.30882858755']
BrGaW 2 ['Ga0.111111Br0.777778W0.111111', '-1.097', '-0.224668647518']
BrGaY 53 ['Ga0.555556Br0.222222Y0.222222', '-0.975', '-0.206545714602']
BrGaYb 12 ['Ga0.142857Br0.428571Yb0.428571', '-1.844', '-0.202166812536']
BrGaZn 3 ['Zn0.222222Ga0.111111Br0.666667', '-1.224', '-0.207108181309']
BrGaZr 3 ['Ga0.142857Br0.714286Zr0.142857', '-1.586', '-0.235624389168']
BrGdGe 3 ['Ge0.111111Br0.777778Gd0.111111', '-1.644', '-0.200074783397']
BrGdH 2 ['H0.5Br0.125Gd0.375', '-1.07', '-0.201738497876']
BrGdHf 6 ['Br0.666667Gd0.111111Hf0.222222', '-1.416', '-0.321838430601']
BrGdHo 5 ['Br0.714286Gd0.142857Ho0.142857', '-1.964', '-0.233038335296']
BrGdI 58 ['Br0.333333I0.166667Gd0.5', '-1.439', '-0.203962913788']
BrGdIr 8 ['Br0.4Gd0.4Ir0.2', '-1.71', '-0.204483898364']
BrGdKr 2 ['Br0.75Kr0.125Gd0.125', '-1.275', '-0.20344863253']
BrGdLa 2 ['Br0.8La0.1Gd0.1', '-1.988', '-0.2274752153']
BrGdLu 6 ['Br0.666667Gd0.111111Lu0.222222', '-1.696', '-0.311557432823']
BrGdMn 1 ['Mn0.1Br0.5Gd0.4', '-1.647', '-0.218264843373']
BrGdMo 1 ['Br0.8Mo0.1Gd0.1', '-1.432', '-0.255982992299']
BrGdN 2 ['N0.111111Br0.444444Gd0.444444', '-1.893', '-0.200842730181']
BrGdNa 7 ['Na0.2Br0.5Gd0.3', '-1.786', '-0.210359918372']
BrGdNb 3 ['Br0.777778Nb0.111111Gd0.111111', '-1.803', '-0.225791526968']
BrGdNi 6 ['Ni0.125Br0.75Gd0.125', '-1.512', '-0.221884904426']
BrGdNp 2 ['Br0.777778Gd0.111111Np0.111111', '-1.934', '-0.276035711582']
BrGdO 37 ['O0.333333Br0.5Gd0.166667', '-1.871', '-0.201914978499']
BrGdOs 15 ['Br0.222222Gd0.555556Os0.222222', '-0.929', '-0.205428455666']
BrGdP 16 ['P0.2Br0.4Gd0.4', '-2.046', '-0.210099743407']
BrGdPd 4 ['Br0.5Pd0.2Gd0.3', '-1.928', '-0.203276631373']
BrGdPm 6 ['Br0.666667Pm0.222222Gd0.111111', '-1.796', '-0.275298144489']
BrGdPr 2 ['Br0.777778Pr0.111111Gd0.111111', '-2.119', '-0.212676663387']
BrGdPt 16 ['Br0.444444Gd0.333333Pt0.222222', '-1.968', '-0.204967276425']
BrGdPu 4 ['Br0.6Gd0.1Pu0.3', '-1.779', '-0.2119220538']
BrGdRb 2 ['Br0.571429Rb0.285714Gd0.142857', '-2.119', '-0.201103399808']
BrGdRe 3 ['Br0.75Gd0.125Re0.125', '-1.386', '-0.31444863253']
BrGdRh 28 ['Br0.375Rh0.25Gd0.375', '-1.677', '-0.202864745655']
BrGdRu 4 ['Br0.7Ru0.2Gd0.1', '-1.373', '-0.228391761724']
BrGdS 37 ['S0.444444Br0.333333Gd0.222222', '-1.797', '-0.200287127746']
BrGdSb 26 ['Br0.555556Sb0.111111Gd0.333333', '-2.062', '-0.203312387544']
BrGdSc 7 ['Sc0.3Br0.6Gd0.1', '-1.735', '-0.331455365299']
BrGdSe 17 ['Se0.444444Br0.333333Gd0.222222', '-1.684', '-0.200541192526']
BrGdSi 7 ['Si0.1Br0.8Gd0.1', '-1.614', '-0.217063228559']

BrGdSm 2 ['Br0.777778Sm0.111111Gd0.111111', '-2.103', '-0.207474640053']
BrGdSn 2 ['Br0.8Sn0.1Gd0.1', '-1.539', '-0.207475649123']
BrGdTa 2 ['Br0.777778Gd0.111111Ta0.111111', '-1.671', '-0.310020735975']
BrGdTb 3 ['Br0.75Gd0.125Tb0.125', '-2.032', '-0.392348041665']
BrGdTc 3 ['Br0.75Tc0.125Gd0.125', '-1.621', '-0.248646126624']
BrGdTe 38 ['Br0.444444Te0.222222Gd0.333333', '-2.03', '-0.20022461522']
BrGdTl 2 ['Br0.666667Gd0.111111Tl0.222222', '-1.684', '-0.21482116186']
BrGdTm 6 ['Br0.666667Gd0.111111Tm0.222222', '-1.814', '-0.21409648811']
BrGdV 2 ['V0.1Br0.8Gd0.1', '-1.485', '-0.32110645154']
BrGdY 5 ['Br0.714286Y0.142857Gd0.142857', '-2.008', '-0.256858204344']
BrGdYb 2 ['Br0.625Gd0.125Yb0.25', '-2.302', '-0.204327884636']
BrGdZn 6 ['Zn0.3Br0.6Gd0.1', '-1.526', '-0.203615080801']
BrGdZr 2 ['Br0.777778Zr0.111111Gd0.111111', '-1.968', '-0.293007609222']
BrGeHf 15 ['Ge0.222222Br0.666667Hf0.111111', '-1.076', '-0.229663613111']
BrGeHo 46 ['Ge0.375Br0.25Ho0.375', '-1.139', '-0.201898436269']
BrGeIr 8 ['Ge0.2Br0.6Ir0.2', '-0.899', '-0.203866699925']
BrGeLa 11 ['Ge0.2Br0.3La0.5', '-1.501', '-0.201578753875']
BrGeLu 48 ['Ge0.333333Br0.555556Lu0.111111', '-1.01', '-0.209457633894']
BrGeMn 2 ['Mn0.125Ge0.25Br0.625', '-1.017', '-0.200613114971']
BrGeMo 1 ['Ge0.1Br0.8Mo0.1', '-0.985', '-0.223932485309']
BrGeNi 12 ['Ni0.2Ge0.1Br0.7', '-0.91', '-0.202675234459']
BrGeNp 3 ['Ge0.111111Br0.777778Np0.111111', '-1.4', '-0.203090703815']
BrGeO 3 ['O0.555556Ge0.222222Br0.222222', '-1.538', '-0.224366684252']
BrGeOs 2 ['Ge0.111111Br0.777778Os0.111111', '-0.713', '-0.221564887815']
BrGeP 2 ['P0.111111Ge0.111111Br0.777778', '-0.949', '-0.200974436746']
BrGePa 1 ['Ge0.6Br0.1Pa0.3', '-0.645', '-0.258570919759']
BrGePd 1 ['Ge0.222222Br0.666667Pd0.111111', '-1.052', '-0.224447678111']
BrGePm 57 ['Ge0.4Br0.1Pm0.5', '-0.957', '-0.211171185008']
BrGePr 11 ['Ge0.166667Br0.666667Pr0.166667', '-1.838', '-0.21456347995']
BrGePt 16 ['Ge0.142857Br0.571429Pt0.285714', '-0.853', '-0.221154855762']
BrGePu 20 ['Ge0.125Br0.5Pu0.375', '-1.558', '-0.262018483168']
BrGeRe 2 ['Ge0.111111Br0.777778Re0.111111', '-0.792', '-0.300564887815']
BrGeRh 10 ['Ge0.25Br0.625Rh0.125', '-1.078', '-0.273131607006']
BrGeRu 5 ['Ge0.25Br0.625Ru0.125', '-0.958', '-0.212449983255']
BrGeS 1 ['S0.125Ge0.25Br0.625', '-1.0', '-0.204407943431']
BrGeSc 48 ['Sc0.222222Ge0.333333Br0.444444', '-1.087', '-0.226952644482']
BrGeSe 10 ['Ge0.25Se0.125Br0.625', '-0.947', '-0.201092998099']
BrGeSm 17 ['Ge0.375Br0.25Sm0.375', '-1.46', '-0.216162418458']
BrGeSr 5 ['Ge0.166667Br0.666667Sr0.166667', '-1.768', '-0.201585157346']
BrGeTa 2 ['Ge0.111111Br0.777778Ta0.111111', '-1.142', '-0.242075728208']
BrGeTb 36 ['Ge0.142857Br0.428571Tb0.428571', '-1.374', '-0.215122867145']
BrGeTe 9 ['Ge0.2Br0.2Te0.6', '-0.45', '-0.201216069059']
BrGeTi 2 ['Ti0.125Ge0.125Br0.75', '-1.432', '-0.230466110371']
BrGeTm 42 ['Ge0.333333Br0.555556Tm0.111111', '-1.08', '-0.211211501853']

BrGeV 2 ['V0.111111Ge0.222222Br0.666667', '-1.056', '-0.201443925889']
BrGeY 58 ['Ge0.4Br0.4Y0.2', '-1.038', '-0.203522430126']
BrGeYb 37 ['Ge0.166667Br0.333333Yb0.5', '-1.7', '-0.202242958778']
BrGeZr 3 ['Ge0.125Br0.75Zr0.125', '-1.457', '-0.223237673423']
BrHHf 66 ['H0.333333Br0.222222Hf0.444444', '-0.599', '-0.214238411791']
BrHHo 51 ['H0.571429Br0.142857Ho0.285714', '-0.963', '-0.200238110866']
BrHIr 2 ['H0.428571Br0.428571Ir0.142857', '-0.541', '-0.203557227314']
BrHLa 3 ['H0.3Br0.3La0.4', '-1.432', '-0.222205985622']
BrHLu 63 ['H0.625Br0.125Lu0.25', '-0.819', '-0.209456791458']
BrHMn 6 ['H0.6Mn0.2Br0.2', '-0.509', '-0.202726501139']
BrHMo 11 ['H0.166667Br0.666667Mo0.166667', '-0.876', '-0.213479065525']
BrHNd 4 ['H0.444444Br0.222222Nd0.333333', '-1.256', '-0.203921344789']
BrHNi 21 ['H0.333333Ni0.166667Br0.5', '-0.63', '-0.207353950928']
BrHOs 22 ['H0.5Br0.333333Os0.166667', '-0.47', '-0.207544510133']
BrHP 1 ['H0.5P0.2Br0.3', '-0.567', '-0.2382507213']
BrHPa 2 ['H0.25Br0.5Pa0.25', '-1.397', '-0.221955197623']
BrHPd 2 ['H0.5Br0.3Pd0.2', '-0.468', '-0.204935392683']
BrHPm 77 ['H0.7Br0.2Pm0.1', '-0.545', '-0.210568065048']
BrHPr 23 ['H0.375Br0.375Pr0.25', '-1.523', '-0.200112298273']
BrHPt 10 ['H0.222222Br0.555556Pt0.222222', '-0.38', '-0.205029673422']
BrHPu 21 ['H0.333333Br0.333333Pu0.333333', '-1.161', '-0.202341099023']
BrHRe 16 ['H0.222222Br0.555556Re0.222222', '-0.379', '-0.204029673422']
BrHRh 7 ['H0.333333Br0.444444Rh0.222222', '-0.609', '-0.21256843226']
BrHRu 28 ['H0.285714Br0.571429Ru0.142857', '-0.641', '-0.210775905614']
BrHS 9 ['H0.625S0.25Br0.125', '-0.478', '-0.20098705483']
BrHSb 5 ['H0.4Br0.4Sb0.2', '-0.65', '-0.2057444487']
BrHSc 72 ['H0.166667Sc0.5Br0.333333', '-0.977', '-0.202181953414']
BrHSm 16 ['H0.5Br0.25Sm0.25', '-1.294', '-0.205326994067']
BrHTa 4 ['H0.111111Br0.777778Ta0.111111', '-0.718', '-0.222025677104']
BrHTb 28 ['H0.166667Br0.5Tb0.333333', '-1.491', '-0.2135555486']
BrHTc 22 ['H0.125Br0.625Tc0.25', '-0.702', '-0.200662490157']
BrHTe 48 ['H0.555556Br0.333333Te0.111111', '-0.472', '-0.208309892245']
BrHTi 1 ['H0.333333Ti0.166667Br0.5', '-1.268', '-0.2938361235']
BrHTm 66 ['H0.333333Br0.222222Tm0.444444', '-1.018', '-0.216936528179']
BrHU 2 ['H0.333333Br0.444444U0.222222', '-1.194', '-0.202517848061']
BrHV 2 ['H0.111111V0.222222Br0.666667', '-0.984', '-0.215064937858']
BrHW 13 ['H0.333333Br0.555556W0.111111', '-0.707', '-0.214714480059']
BrHY 58 ['H0.5Br0.125Y0.375', '-1.011', '-0.201393186105']
BrHZr 4 ['H0.333333Br0.555556Zr0.111111', '-1.1', '-0.202527387062']
BrHfHg 24 ['Br0.5Hf0.2Hg0.3', '-0.718', '-0.216656471552']
BrHfHo 17 ['Br0.555556Ho0.222222Hf0.222222', '-1.187', '-0.246886050137']
BrHfI 52 ['Br0.285714I0.571429Hf0.142857', '-0.95', '-0.201906568257']
BrHfIn 12 ['Br0.666667In0.222222Hf0.111111', '-1.187', '-0.227410840613']
BrHfIr 21 ['Br0.5Hf0.25Ir0.25', '-0.791', '-0.219479390042']

BrHfK 19 ['K0.333333Br0.555556Hf0.111111', '-1.63', '-0.273795060036']
BrHfKr 31 ['Br0.428571Kr0.285714Hf0.285714', '-0.409', '-0.226850973595']
BrHfLa 6 ['Br0.666667La0.111111Hf0.222222', '-1.424', '-0.27867998977']
BrHfLi 19 ['Li0.222222Br0.555556Hf0.222222', '-1.136', '-0.235536080036']
BrHfLu 26 ['Br0.5Lu0.1Hf0.4', '-0.655', '-0.205612754792']
BrHfMg 11 ['Mg0.222222Br0.666667Hf0.111111', '-1.474', '-0.257802811063']
BrHfMn 14 ['Mn0.1Br0.5Hf0.4', '-0.714', '-0.216470023414']
BrHfMo 15 ['Br0.625Mo0.25Hf0.125', '-1.02', '-0.274569331198']
BrHfN 16 ['N0.222222Br0.333333Hf0.444444', '-1.53', '-0.205562518009']
BrHfNa 21 ['Na0.1Br0.5Hf0.4', '-0.834', '-0.219791869208']
BrHfNb 6 ['Br0.666667Nb0.111111Hf0.222222', '-1.06', '-0.246933992276']
BrHfNd 6 ['Br0.666667Nd0.111111Hf0.222222', '-1.321', '-0.229527145325']
BrHfNi 34 ['Ni0.25Br0.375Hf0.375', '-0.718', '-0.266463602844']
BrHfNp 6 ['Br0.666667Hf0.222222Np0.111111', '-1.283', '-0.435854351019']
BrHfO 30 ['O0.4Br0.3Hf0.3', '-2.57', '-0.202758455404']
BrHfOs 20 ['Br0.625Hf0.125Os0.25', '-0.488', '-0.310802851875']
BrHfP 18 ['P0.2Br0.5Hf0.3', '-0.977', '-0.212358837972']
BrHfPa 2 ['Br0.777778Hf0.111111Pa0.111111', '-1.702', '-0.492144685889']
BrHfPb 14 ['Br0.6Hf0.2Pb0.2', '-1.102', '-0.304231891053']
BrHfPd 39 ['Br0.428571Pd0.428571Hf0.142857', '-0.84', '-0.223968803352']
BrHfPm 19 ['Br0.555556Pm0.222222Hf0.222222', '-0.977', '-0.267116783925']
BrHfPr 6 ['Br0.666667Pr0.111111Hf0.222222', '-1.392', '-0.296495302823']
BrHfPt 35 ['Br0.5Hf0.125Pt0.375', '-0.752', '-0.202953963125']
BrHfPu 11 ['Br0.625Hf0.125Pu0.25', '-1.684', '-0.205173224533']
BrHfRb 19 ['Br0.555556Rb0.111111Hf0.333333', '-0.977', '-0.336140826148']
BrHfRe 16 ['Br0.5Hf0.375Re0.125', '-0.497', '-0.22251843737']
BrHfRh 33 ['Br0.4Rh0.2Hf0.4', '-0.797', '-0.244774917534']
BrHfRu 21 ['Br0.5Ru0.25Hf0.25', '-0.782', '-0.229652436292']
BrHfS 67 ['S0.444444Br0.444444Hf0.111111', '-0.94', '-0.200087621568']
BrHfSb 20 ['Br0.555556Sb0.222222Hf0.222222', '-0.905', '-0.231191698102']
BrHfSc 27 ['Sc0.125Br0.5Hf0.375', '-0.707', '-0.240302927542']
BrHfSe 65 ['Se0.5Br0.4Hf0.1', '-0.753', '-0.205901378275']
BrHfSi 15 ['Si0.142857Br0.571429Hf0.285714', '-0.979', '-0.20800617505']
BrHfSm 5 ['Br0.714286Sm0.142857Hf0.142857', '-1.803', '-0.499451586832']
BrHfSn 17 ['Br0.625Sn0.25Hf0.125', '-1.068', '-0.20180798834']
BrHfSr 10 ['Br0.625Sr0.125Hf0.25', '-1.324', '-0.278838477541']
BrHfTa 10 ['Br0.7Hf0.1Ta0.2', '-1.013', '-0.213967353465']
BrHfTb 13 ['Br0.625Tb0.25Hf0.125', '-1.308', '-0.235868508931']
BrHfTc 17 ['Br0.5Tc0.2Hf0.3', '-0.696', '-0.231315425792']
BrHfTe 60 ['Br0.285714Te0.285714Hf0.428571', '-0.753', '-0.216468397643']
BrHfTh 4 ['Br0.7Hf0.2Th0.1', '-1.389', '-0.266553817052']
BrHfTi 7 ['Ti0.2Br0.7Hf0.1', '-1.57', '-0.258952786801']
BrHfTl 24 ['Br0.5Hf0.25Tl0.25', '-0.889', '-0.211373118791']
BrHfTm 12 ['Br0.625Tm0.25Hf0.125', '-1.374', '-0.213410568491']

BrHfU 4 ['Br0.7Hf0.2U0.1', '-1.249', '-0.219207569811']
BrHfV 11 ['V0.1Br0.6Hf0.3', '-0.847', '-0.349091067791']
BrHfW 12 ['Br0.6Hf0.2W0.2', '-0.767', '-0.202139431175']
BrHfXe 28 ['Br0.5Xe0.125Hf0.375', '-0.521', '-0.281929402844']
BrHfY 15 ['Br0.555556Y0.333333Hf0.111111', '-1.278', '-0.207851394213']
BrHfYb 13 ['Br0.625Yb0.25Hf0.125', '-2.129', '-0.30882147474']
BrHfZn 18 ['Zn0.111111Br0.555556Hf0.333333', '-0.759', '-0.201941820881']
BrHfZr 8 ['Br0.6Zr0.1Hf0.3', '-1.067', '-0.225491464551']
BrHgHo 36 ['Br0.4Ho0.2Hg0.4', '-0.925', '-0.200104012705']
BrHgLu 45 ['Br0.333333Lu0.222222Hg0.444444', '-0.774', '-0.220091067543']
BrHgNb 1 ['Br0.714286Nb0.142857Hg0.142857', '-1.16', '-0.202372305727']
BrHgNp 3 ['Br0.8Hg0.1Np0.1', '-1.032', '-0.20246014992']
BrHgOs 3 ['Br0.714286Os0.142857Hg0.142857', '-0.483', '-0.204981307886']
BrHgP 3 ['P0.1Br0.6Hg0.3', '-0.8', '-0.210313561234']
BrHgPm 40 ['Br0.375Pm0.375Hg0.25', '-1.191', '-0.23214267003']
BrHgPr 4 ['Br0.333333Pr0.333333Hg0.333333', '-1.356', '-0.20338578059']
BrHgPt 4 ['Br0.625Pt0.25Hg0.125', '-0.491', '-0.2477336444']
BrHgPu 32 ['Br0.7Hg0.2Pu0.1', '-1.305', '-0.205936978817']
BrHgRe 6 ['Br0.7Re0.2Hg0.1', '-0.404', '-0.20938691552']
BrHgRh 1 ['Br0.6Rh0.2Hg0.2', '-0.792', '-0.219025880557']
BrHgSb 2 ['Br0.625Sb0.125Hg0.25', '-0.906', '-0.219278285061']
BrHgSc 35 ['Sc0.4Br0.3Hg0.3', '-0.848', '-0.215792083285']
BrHgSm 1 ['Br0.666667Sm0.222222Hg0.111111', '-2.119', '-0.232929488942']
BrHgTa 8 ['Br0.625Ta0.125Hg0.25', '-1.006', '-0.204117367978']
BrHgTb 21 ['Br0.6Tb0.1Hg0.3', '-1.054', '-0.234618180495']
BrHgTe 9 ['Br0.7Te0.1Hg0.2', '-0.643', '-0.213294351461']
BrHgTm 27 ['Br0.6Tm0.1Hg0.3', '-1.081', '-0.206035795061']
BrHgV 1 ['V0.125Br0.75Hg0.125', '-0.865', '-0.238418076295']
BrHgY 39 ['Br0.8Y0.1Hg0.1', '-0.911', '-0.205288008796']
BrHgYb 5 ['Br0.555556Yb0.222222Hg0.222222', '-1.883', '-0.215796094218']
BrHoI 79 ['Br0.222222I0.333333Ho0.444444', '-1.201', '-0.205525233286']
BrHoIn 26 ['Br0.6In0.3Ho0.1', '-1.221', '-0.223273219715']
BrHoIr 28 ['Br0.571429Ho0.142857Ir0.285714', '-1.008', '-0.206791721712']
BrHoK 18 ['K0.333333Br0.555556Ho0.111111', '-1.92', '-0.240817379952']
BrHoKr 24 ['Br0.5Kr0.25Ho0.25', '-1.11', '-0.223919821707']
BrHoLa 5 ['Br0.714286La0.142857Ho0.142857', '-2.103', '-0.306263197084']
BrHoLi 18 ['Li0.333333Br0.555556Ho0.111111', '-1.734', '-0.201997904952']
BrHoLu 16 ['Br0.555556Ho0.333333Lu0.111111', '-1.313', '-0.220557780326']
BrHoMg 12 ['Mg0.1Br0.8Ho0.1', '-1.23', '-0.222916861039']
BrHoMn 14 ['Mn0.1Br0.5Ho0.4', '-1.231', '-0.209088614274']
BrHoMo 15 ['Br0.555556Mo0.111111Ho0.333333', '-1.246', '-0.217046886991']
BrHoN 45 ['N0.333333Br0.111111Ho0.555556', '-1.704', '-0.230364555913']
BrHoNa 19 ['Na0.1Br0.8Ho0.1', '-1.099', '-0.248130022614']
BrHoNb 11 ['Br0.555556Nb0.111111Ho0.333333', '-1.28', '-0.204994266579']

BrHoNd 4 ['Br0.7Nd0.1Ho0.2', '-1.839', '-0.275314606642']
BrHoNi 33 ['Ni0.333333Br0.555556Ho0.111111', '-0.982', '-0.241420593285']
BrHoNp 9 ['Br0.625Ho0.25Np0.125', '-1.564', '-0.227357395315']
BrHoO 58 ['O0.166667Br0.666667Ho0.166667', '-1.552', '-0.202700875492']
BrHoOs 28 ['Br0.333333Ho0.5Os0.166667', '-0.966', '-0.221451570864']
BrHoP 58 ['P0.25Br0.625Ho0.125', '-1.134', '-0.217732377708']
BrHoPa 7 ['Br0.666667Ho0.166667Pa0.166667', '-1.662', '-0.245787260129']
BrHoPb 26 ['Br0.375Ho0.375Pb0.25', '-1.095', '-0.200319761483']
BrHoPd 35 ['Br0.555556Pd0.333333Ho0.111111', '-0.997', '-0.223602210385']
BrHoPm 14 ['Br0.6Pm0.3Ho0.1', '-1.498', '-0.239243652464']
BrHoPr 5 ['Br0.714286Pr0.142857Ho0.142857', '-1.957', '-0.224311456724']
BrHoPt 59 ['Br0.142857Ho0.571429Pt0.285714', '-1.291', '-0.209522076324']
BrHoPu 30 ['Br0.4Ho0.3Pu0.3', '-1.191', '-0.244550863701']
BrHoRb 18 ['Br0.555556Rb0.222222Ho0.222222', '-1.778', '-0.286156346806']
BrHoRe 14 ['Br0.666667Ho0.166667Re0.166667', '-1.057', '-0.237549194067']
BrHoRh 47 ['Br0.3Rh0.2Ho0.5', '-1.222', '-0.208407028111']
BrHoRu 26 ['Br0.5Ru0.25Ho0.25', '-1.1', '-0.213919821707']
BrHoS 68 ['S0.5Br0.25Ho0.25', '-1.701', '-0.202462884934']
BrHoSb 56 ['Br0.125Sb0.375Ho0.5', '-1.335', '-0.22603102053']
BrHoSc 23 ['Sc0.25Br0.5Ho0.25', '-1.206', '-0.207762901711']
BrHoSe 75 ['Se0.333333Br0.111111Ho0.555556', '-1.595', '-0.205735651027']
BrHoSi 39 ['Si0.333333Br0.222222Ho0.444444', '-1.148', '-0.200362438814']
BrHoSm 5 ['Br0.714286Sm0.142857Ho0.142857', '-1.929', '-0.210194569581']
BrHoSn 34 ['Br0.571429Sn0.285714Ho0.142857', '-1.225', '-0.210884451389']
BrHoSr 10 ['Br0.777778Sr0.111111Ho0.111111', '-1.535', '-0.201338463284']
BrHoTa 13 ['Br0.5Ho0.4Ta0.1', '-1.241', '-0.214665259284']
BrHoTb 11 ['Br0.625Tb0.125Ho0.25', '-1.459', '-0.208379962824']
BrHoTc 24 ['Br0.4Tc0.2Ho0.4', '-1.099', '-0.230225363704']
BrHoTe 67 ['Br0.285714Te0.571429Ho0.142857', '-0.903', '-0.220346393802']
BrHoTh 4 ['Br0.7Ho0.2Th0.1', '-1.892', '-0.325432405144']
BrHoTi 9 ['Ti0.125Br0.625Ho0.25', '-1.543', '-0.27371071781']
BrHoTl 37 ['Br0.4Ho0.3Tl0.3', '-1.129', '-0.214851832535']
BrHoTm 15 ['Br0.6Ho0.1Tm0.3', '-1.421', '-0.243348371218']
BrHoU 5 ['Br0.714286Ho0.142857U0.142857', '-1.663', '-0.229774170659']
BrHoV 11 ['V0.222222Br0.666667Ho0.111111', '-1.28', '-0.204736688572']
BrHoW 11 ['Br0.571429Ho0.285714W0.142857', '-1.27', '-0.257336939094']
BrHoXe 22 ['Br0.5Xe0.125Ho0.375', '-1.187', '-0.200975763169']
BrHoY 19 ['Br0.555556Y0.111111Ho0.333333', '-1.372', '-0.238512066439']
BrHoYb 12 ['Br0.625Ho0.125Yb0.25', '-2.271', '-0.25901959995']
BrHoZn 31 ['Zn0.375Br0.5Ho0.125', '-1.173', '-0.276776160432']
BrHoZr 14 ['Br0.6Zr0.2Ho0.2', '-1.524', '-0.207904445886']
BrIIr 4 ['Br0.5I0.333333Ir0.166667', '-0.553', '-0.207123443792']
BrILa 20 ['Br0.333333I0.333333La0.333333', '-1.988', '-0.201218217717']
BrILu 81 ['Br0.1I0.8Lu0.1', '-0.53', '-0.335621391758']

BrIMo 23 ['Br0.571429Mo0.285714I0.142857', '-0.965', '-0.20188001863']
BrINa 1 ['Na0.444444Br0.333333I0.222222', '-1.722', '-0.209976030216']
BrINd 36 ['Br0.222222I0.333333Nd0.444444', '-1.544', '-0.200608651893']
BrINi 2 ['Ni0.285714Br0.428571I0.285714', '-0.628', '-0.201675613628']
BrINp 18 ['Br0.5I0.333333Np0.166667', '-1.262', '-0.203788724']
BrIO 2 ['O0.666667Br0.166667I0.166667', '-0.622', '-0.222968958919']
BrIOs 11 ['Br0.5I0.333333Os0.166667', '-0.411', '-0.206630505052']
BrIP 1 ['P0.111111Br0.333333I0.555556', '-0.552', '-0.241569701225']
BrIPa 48 ['Br0.625I0.125Pa0.25', '-1.545', '-0.201218605608']
BrIPm 89 ['Br0.375I0.125Pm0.5', '-1.435', '-0.252486795664']
BrIPr 43 ['Br0.166667I0.333333Pr0.5', '-1.406', '-0.205321837286']
BrIPt 36 ['Br0.142857I0.571429Pt0.285714', '-0.469', '-0.210483821124']
BrIPu 37 ['Br0.4I0.4Pu0.2', '-1.469', '-0.200435339216']
BrIRb 9 ['Br0.333333Rb0.333333I0.333333', '-1.524', '-0.202532830051']
BrIRe 34 ['Br0.5I0.25Re0.25', '-0.36', '-0.206722878789']
BrIRh 19 ['Br0.3Rh0.3I0.4', '-0.47', '-0.202408691775']
BrIRu 21 ['Br0.375Ru0.25I0.375', '-0.473', '-0.203593302219']
BrIS 9 ['S0.111111Br0.333333I0.555556', '-0.415', '-0.203699735987']
BrISb 20 ['Br0.777778Sb0.111111I0.111111', '-0.643', '-0.204663875601']
BrISc 73 ['Sc0.5Br0.375I0.125', '-1.117', '-0.203269637852']
BrISe 14 ['Se0.375Br0.375I0.25', '-0.413', '-0.200059455362']
BrISi 6 ['Si0.2Br0.4I0.4', '-1.001', '-0.205787448063']
BrISm 43 ['Br0.222222I0.444444Sm0.333333', '-1.668', '-0.216653087454']
BrISn 7 ['Br0.444444Sn0.222222I0.333333', '-0.987', '-0.205241941733']
BrISr 1 ['Br0.428571Sr0.428571I0.142857', '-2.144', '-0.234242279866']
BrITa 13 ['Br0.7I0.1Ta0.2', '-0.999', '-0.202408664222']
BrITb 82 ['Br0.3I0.2Tb0.5', '-1.076', '-0.205396000483']
BrITc 11 ['Br0.5Tc0.25I0.25', '-0.617', '-0.215929992125']
BrITe 53 ['Br0.142857Te0.142857I0.714286', '-0.471', '-0.205707774409']
BrITh 14 ['Br0.25I0.5Th0.25', '-1.693', '-0.200037433652']
BrITl 1 ['Br0.5I0.1Tl0.4', '-1.11', '-0.219494778548']
BrITm 72 ['Br0.375I0.125Tm0.5', '-1.206', '-0.202924861288']
BrIU 13 ['Br0.4I0.4U0.2', '-1.477', '-0.206403722064']
BrIV 27 ['V0.125Br0.375I0.5', '-0.661', '-0.201045871289']
BrIW 7 ['Br0.166667I0.666667W0.166667', '-0.382', '-0.209627477444']
BrIXe 15 ['Br0.4I0.3Xe0.3', '-0.384', '-0.200067454547']
BrIY 61 ['Br0.8Y0.1I0.1', '-0.783', '-0.210590244792']
BrIYb 4 ['Br0.444444I0.111111Yb0.444444', '-2.115', '-0.271508975323']
BrIZr 38 ['Br0.777778Zr0.111111I0.111111', '-0.996', '-0.205374548657']
BrInIr 6 ['Br0.666667In0.166667Ir0.166667', '-1.036', '-0.201015945057']
BrInLa 4 ['Br0.7In0.2La0.1', '-1.714', '-0.20138110331']
BrInLu 36 ['Br0.3In0.3Lu0.4', '-0.918', '-0.201802265775']
BrInMo 2 ['Br0.777778Mo0.111111In0.111111', '-1.052', '-0.218009960612']
BrInNb 3 ['Br0.7Nb0.2In0.1', '-1.302', '-0.200195299645']

BrInNi 1 ['Ni0.166667Br0.666667In0.166667', '-1.067', '-0.201598978391']
BrInNp 4 ['Br0.714286In0.142857Np0.142857', '-1.544', '-0.201722330596']
BrInOs 2 ['Br0.777778In0.111111Os0.111111', '-0.714', '-0.234205420307']
BrInPm 34 ['Br0.375In0.375Pm0.25', '-1.069', '-0.200875104094']
BrInPr 4 ['Br0.777778In0.111111Pr0.111111', '-1.663', '-0.229372188111']
BrInPt 20 ['Br0.555556In0.333333Pt0.111111', '-1.13', '-0.201606934955']
BrInPu 15 ['Br0.777778In0.111111Pu0.111111', '-1.469', '-0.200497806724']
BrInRb 2 ['Br0.6Rb0.2In0.2', '-1.582', '-0.20163123655']
BrInRe 3 ['Br0.75In0.125Re0.125', '-0.781', '-0.241231097845']
BrInRh 14 ['Br0.333333Rh0.222222In0.444444', '-0.926', '-0.207614534195']
BrInRu 5 ['Br0.714286Ru0.142857In0.142857', '-1.031', '-0.208859008752']
BrInSb 1 ['Br0.8In0.1Sb0.1', '-0.986', '-0.220993214801']
BrInSc 38 ['Sc0.3Br0.4In0.3', '-1.003', '-0.207177896534']
BrInSe 2 ['Se0.1Br0.7In0.2', '-1.147', '-0.23563901781']
BrInSi 1 ['Si0.166667Br0.666667In0.166667', '-1.17', '-0.212474263807']
BrInSm 5 ['Br0.4In0.2Sm0.4', '-1.54', '-0.203815675032']
BrInTa 3 ['Br0.75In0.125Ta0.125', '-1.22', '-0.220680793287']
BrInTb 21 ['Br0.5In0.1Tb0.4', '-1.406', '-0.201586289013']
BrInTc 1 ['Br0.8Tc0.1In0.1', '-0.907', '-0.234542873551']
BrInTe 3 ['Br0.75In0.125Te0.125', '-0.804', '-0.213631748371']
BrInTh 1 ['Br0.8In0.1Th0.1', '-1.651', '-0.224243013811']
BrInTi 1 ['Ti0.125Br0.75In0.125', '-1.498', '-0.204661614277']
BrInTl 1 ['Br0.6In0.2Tl0.2', '-1.243', '-0.21906760755']
BrInTm 31 ['Br0.333333In0.222222Tm0.444444', '-1.038', '-0.200544763639']
BrInV 3 ['V0.111111Br0.777778In0.111111', '-1.062', '-0.24148047088']
BrInY 50 ['Br0.3Y0.5In0.2', '-1.095', '-0.215119741274']
BrInYb 8 ['Br0.444444In0.111111Yb0.444444', '-1.877', '-0.203474891427']
BrInZr 2 ['Br0.8Zr0.1In0.1', '-1.419', '-0.336932820552']
BrIrK 3 ['K0.142857Br0.714286Ir0.142857', '-1.06', '-0.212661862905']
BrIrKr 4 ['Br0.666667Kr0.222222Ir0.111111', '-0.479', '-0.248415629195']
BrIrLa 8 ['Br0.666667La0.111111Ir0.222222', '-1.461', '-0.226767083946']
BrIrLi 2 ['Li0.111111Br0.777778Ir0.111111', '-0.821', '-0.211019401704']
BrIrLu 42 ['Br0.166667Lu0.5Ir0.333333', '-1.182', '-0.208903424597']
BrIrMg 1 ['Mg0.111111Br0.777778Ir0.111111', '-1.015', '-0.211734900971']
BrIrMn 2 ['Mn0.1Br0.8Ir0.1', '-0.717', '-0.203200567414']
BrIrMo 8 ['Br0.7Mo0.1Ir0.2', '-0.839', '-0.226355924021']
BrIrNa 5 ['Na0.1Br0.8Ir0.1', '-0.799', '-0.232274572449']
BrIrNb 4 ['Br0.75Nb0.125Ir0.125', '-1.165', '-0.243521801311']
BrIrNd 15 ['Br0.333333Nd0.444444Ir0.222222', '-1.397', '-0.202765575862']
BrIrNi 3 ['Ni0.125Br0.75Ir0.125', '-0.695', '-0.21702885474']
BrIrNp 8 ['Br0.7Ir0.2Np0.1', '-1.133', '-0.221371989434']
BrIrO 1 ['O0.1Br0.7Ir0.2', '-0.676', '-0.205062909574']
BrIrOs 3 ['Br0.75Os0.125Ir0.125', '-0.492', '-0.232592582844']
BrIrP 10 ['P0.111111Br0.666667Ir0.222222', '-0.866', '-0.281451398334']

BrIrPa 2 ['Br0.777778Ir0.111111Pa0.111111', '-1.508', '-0.21525750451']
BrIrPb 2 ['Br0.75Ir0.125Pb0.125', '-0.888', '-0.209677713804']
BrIrPm 36 ['Br0.285714Pm0.428571Ir0.285714', '-1.112', '-0.208672808237']
BrIrPr 17 ['Br0.333333Pr0.5Ir0.166667', '-1.344', '-0.21164802432']
BrIrPt 6 ['Br0.666667Ir0.166667Pt0.166667', '-0.556', '-0.210123443792']
BrIrPu 17 ['Br0.5Ir0.2Pu0.3', '-1.573', '-0.20160984915']
BrIrRb 7 ['Br0.666667Rb0.222222Ir0.111111', '-1.289', '-0.201711676435']
BrIrRe 4 ['Br0.714286Re0.142857Ir0.142857', '-0.541', '-0.244534380393']
BrIrRh 3 ['Br0.8Rh0.1Ir0.1', '-0.689', '-0.21388275805']
BrIrRu 2 ['Br0.75Ru0.125Ir0.125', '-0.729', '-0.200185885063']
BrIrS 16 ['S0.333333Br0.444444Ir0.222222', '-0.703', '-0.201548751755']
BrIrSb 11 ['Br0.666667Sb0.111111Ir0.222222', '-0.856', '-0.255202669778']
BrIrSc 16 ['Sc0.222222Br0.555556Ir0.222222', '-1.352', '-0.231205621063']
BrIrSe 17 ['Se0.2Br0.6Ir0.2', '-0.632', '-0.20199980835']
BrIrSi 13 ['Si0.125Br0.625Ir0.25', '-0.967', '-0.205911264117']
BrIrSm 11 ['Br0.428571Sm0.428571Ir0.142857', '-1.599', '-0.203291448963']
BrIrSn 8 ['Br0.666667Sn0.222222Ir0.111111', '-1.078', '-0.213380099089']
BrIrTa 7 ['Br0.666667Ta0.166667Ir0.166667', '-1.048', '-0.229562488071']
BrIrTb 27 ['Br0.6Tb0.1Ir0.3', '-0.915', '-0.236653696084']
BrIrTc 4 ['Br0.714286Tc0.142857Ir0.142857', '-0.753', '-0.211581961131']
BrIrTe 15 ['Br0.555556Te0.222222Ir0.222222', '-0.652', '-0.212240601297']
BrIrTh 6 ['Br0.666667Ir0.222222Th0.111111', '-1.475', '-0.215786125613']
BrIrTi 1 ['Ti0.125Br0.75Ir0.125', '-1.309', '-0.223557839902']
BrIrTl 6 ['Br0.777778Ir0.111111Tl0.111111', '-0.717', '-0.200058665741']
BrIrTm 31 ['Br0.333333Tm0.5Ir0.166667', '-1.201', '-0.210905749193']
BrIrU 1 ['Br0.75Ir0.125U0.125', '-1.492', '-0.277670721315']
BrIrV 5 ['V0.2Br0.7Ir0.1', '-1.022', '-0.201169157307']
BrIrW 1 ['Br0.7W0.2Ir0.1', '-0.893', '-0.224280504121']
BrIrXe 7 ['Br0.666667Xe0.166667Ir0.166667', '-0.585', '-0.239123443792']
BrIrY 51 ['Br0.2Y0.4Ir0.4', '-1.138', '-0.205068597516']
BrIrYb 17 ['Br0.666667Yb0.111111Ir0.222222', '-1.288', '-0.207003805056']
BrIrZr 9 ['Br0.666667Zr0.111111Ir0.222222', '-1.238', '-0.284913342835']
BrKLu 19 ['K0.111111Br0.555556Lu0.333333', '-1.338', '-0.26161490337']
BrKMo 2 ['K0.1Br0.8Mo0.1', '-0.957', '-0.249323183033']
BrKNb 2 ['K0.142857Br0.714286Nb0.142857', '-1.567', '-0.212918151436']
BrKNp 5 ['K0.142857Br0.714286Np0.142857', '-1.688', '-0.23008924594']
BrKO 17 ['O0.333333K0.444444Br0.222222', '-1.701', '-0.202315557501']
BrKOs 2 ['K0.111111Br0.777778Os0.111111', '-0.781', '-0.230734240305']
BrKP 5 ['P0.2K0.2Br0.6', '-1.283', '-0.203869934233']
BrKPm 19 ['K0.111111Br0.555556Pm0.333333', '-1.517', '-0.236225970869']
BrKPr 2 ['K0.125Br0.75Pr0.125', '-1.784', '-0.20949794926']
BrKPt 3 ['K0.125Br0.625Pt0.25', '-0.9', '-0.220009224718']
BrKPu 8 ['K0.1Br0.8Pu0.1', '-1.3', '-0.204552385534']
BrKRb 1 ['K0.111111Br0.555556Rb0.333333', '-1.95', '-0.236487668369']

BrKRe 5 ['K0.1Br0.7Re0.2', '-0.746', '-0.208276897027']
BrKRh 1 ['K0.142857Br0.714286Rh0.142857', '-1.163', '-0.229854185047']
BrKS 5 ['S0.125K0.25Br0.625', '-1.251', '-0.202537418591']
BrKSb 5 ['K0.2Br0.7Sb0.1', '-1.325', '-0.220586812041']
BrKSc 19 ['K0.1Sc0.1Br0.8', '-1.013', '-0.445288057516']
BrKSi 3 ['Si0.125K0.25Br0.625', '-1.682', '-0.212008396772']
BrKSm 1 ['K0.142857Br0.714286Sm0.142857', '-2.007', '-0.243653582546']
BrKTa 6 ['K0.222222Br0.666667Ta0.111111', '-1.506', '-0.24059803541']
BrKTb 10 ['K0.25Br0.625Tb0.125', '-1.825', '-0.29287250353']
BrKTc 9 ['K0.111111Br0.666667Tc0.222222', '-1.084', '-0.209910255426']
BrKTe 25 ['K0.5Br0.2Te0.3', '-1.481', '-0.202725921516']
BrKTh 2 ['K0.111111Br0.777778Th0.111111', '-1.754', '-0.220052636992']
BrKTm 15 ['K0.285714Br0.571429Tm0.142857', '-1.859', '-0.217560817904']
BrKV 8 ['K0.1V0.1Br0.8', '-0.944', '-0.251736783274']
BrKY 19 ['K0.333333Br0.555556Y0.111111', '-1.921', '-0.226121722546']
BrKYb 2 ['K0.142857Br0.571429Yb0.285714', '-2.288', '-0.245280345762']
BrKZr 6 ['K0.222222Br0.666667Zr0.111111', '-1.863', '-0.283584908658']
BrKrLa 6 ['Br0.666667Kr0.166667La0.166667', '-1.707', '-0.201527182127']
BrKrLu 36 ['Br0.444444Kr0.444444Lu0.111111', '-0.431', '-0.21502376862']
BrKrMo 11 ['Br0.555556Kr0.333333Mo0.111111', '-0.563', '-0.208804540306']
BrKrNb 1 ['Br0.7Kr0.2Nb0.1', '-0.88', '-0.21199275229']
BrKrNp 9 ['Br0.666667Kr0.166667Np0.166667', '-1.261', '-0.202788724']
BrKrO 15 ['O0.222222Br0.555556Kr0.222222', '-0.327', '-0.218211087065']
BrKrOs 9 ['Br0.666667Kr0.166667Os0.166667', '-0.243', '-0.243']
BrKrPm 35 ['Br0.4Kr0.3Pm0.3', '-0.975', '-0.207914136024']
BrKrPr 1 ['Br0.7Kr0.2Pr0.1', '-1.071', '-0.212550091024']
BrKrPt 11 ['Br0.75Kr0.125Pt0.125', '-0.249', '-0.249']
BrKrPu 8 ['Br0.777778Kr0.111111Pu0.111111', '-0.997', '-0.208292386418']
BrKrRe 12 ['Br0.571429Kr0.285714Re0.142857', '-0.226', '-0.226']
BrKrRu 4 ['Br0.714286Kr0.142857Ru0.142857', '-0.509', '-0.201106631107']
BrKrS 10 ['S0.285714Br0.571429Kr0.142857', '-0.396', '-0.203004169591']
BrKrSb 10 ['Br0.7Kr0.1Sb0.2', '-0.875', '-0.20861667305']
BrKrSc 40 ['Sc0.428571Br0.428571Kr0.142857', '-0.997', '-0.216566370393']
BrKrSm 3 ['Br0.714286Kr0.142857Sm0.142857', '-1.421', '-0.208526100034']
BrKrTa 11 ['Br0.571429Kr0.285714Ta0.142857', '-0.76', '-0.234799651933']
BrKrTb 17 ['Br0.6Kr0.1Tb0.3', '-1.421', '-0.23007044958']
BrKrTc 6 ['Br0.666667Kr0.166667Tc0.166667', '-0.606', '-0.204929992125']
BrKrTe 20 ['Br0.5Kr0.2Te0.3', '-0.351', '-0.229561561263']
BrKrTh 1 ['Br0.777778Kr0.111111Th0.111111', '-1.325', '-0.219509039483']
BrKrTm 27 ['Br0.5Kr0.1Tm0.4', '-1.208', '-0.215555605791']
BrKrU 2 ['Br0.75Kr0.125U0.125', '-1.342', '-0.214139860367']
BrKrV 13 ['V0.142857Br0.571429Kr0.285714', '-0.661', '-0.222925065023']
BrKrY 29 ['Br0.5Kr0.333333Y0.166667', '-1.085', '-0.233168488793']
BrKrZr 6 ['Br0.666667Kr0.166667Zr0.166667', '-1.292', '-0.20824657046']

BrLaLi 1 ['Li0.125Br0.75La0.125', '-1.776', '-0.220074630668']
BrLaLu 6 ['Br0.666667La0.111111Lu0.222222', '-1.822', '-0.386398991992']
BrLaMg 1 ['Mg0.1Br0.7La0.2', '-2.319', '-0.254726290852']
BrLaMn 1 ['Mn0.125Br0.75La0.125', '-1.749', '-0.237053513019']
BrLaMo 3 ['Br0.75Mo0.125La0.125', '-1.826', '-0.298425494439']
BrLaN 12 ['N0.428571Br0.142857La0.428571', '-1.717', '-0.200050091907']
BrLaNa 8 ['Na0.25Br0.5La0.25', '-1.881', '-0.221880640331']
BrLaNb 3 ['Br0.75Nb0.125La0.125', '-1.926', '-0.226288116851']
BrLaNd 1 ['Br0.777778La0.111111Nd0.111111', '-2.242', '-0.288550065058']
BrLaNi 11 ['Ni0.2Br0.5La0.3', '-1.845', '-0.215975194728']
BrLaNp 2 ['Br0.777778La0.111111Np0.111111', '-2.046', '-0.336877270751']
BrLaO 60 ['O0.4Br0.3La0.3', '-3.126', '-0.203427423516']
BrLaOs 6 ['Br0.5La0.375Os0.125', '-1.751', '-0.220900331667']
BrLaP 6 ['P0.444444Br0.111111La0.444444', '-1.94', '-0.206915876722']
BrLaPa 1 ['Br0.777778La0.111111Pa0.111111', '-2.156', '-0.21526406979']
BrLaPd 26 ['Br0.375Pd0.375La0.25', '-1.737', '-0.200126345345']
BrLaPm 6 ['Br0.666667La0.111111Pm0.222222', '-1.931', '-0.359139703658']
BrLaPr 1 ['Br0.8La0.1Pr0.1', '-1.999', '-0.2372664003']
BrLaPt 22 ['Br0.428571La0.428571Pt0.142857', '-1.85', '-0.20596875625']
BrLaPu 10 ['Br0.571429La0.142857Pu0.285714', '-1.83', '-0.201577178859']
BrLaRb 2 ['Br0.714286Rb0.142857La0.142857', '-2.048', '-0.206856472191']
BrLaRe 4 ['Br0.714286La0.142857Re0.142857', '-1.596', '-0.305594727537']
BrLaRh 27 ['Br0.5Rh0.1La0.4', '-1.887', '-0.200554904959']
BrLaRu 5 ['Br0.7Ru0.2La0.1', '-1.476', '-0.285349164976']
BrLaS 18 ['S0.333333Br0.5La0.166667', '-1.711', '-0.205527182127']
BrLaSb 26 ['Br0.5Sb0.25La0.25', '-1.941', '-0.206695145251']
BrLaSc 6 ['Sc0.222222Br0.666667La0.111111', '-1.877', '-0.468682165325']
BrLaSe 34 ['Se0.222222Br0.555556La0.222222', '-2.071', '-0.2020013419']
BrLaSi 20 ['Si0.333333Br0.333333La0.333333', '-1.625', '-0.202359986418']
BrLaSm 1 ['Br0.777778La0.111111Sm0.111111', '-2.175', '-0.228316199222']
BrLaSn 2 ['Br0.75Sn0.125La0.125', '-1.856', '-0.240546692333']
BrLaTa 3 ['Br0.75La0.125Ta0.125', '-1.883', '-0.294345082037']
BrLaTb 3 ['Br0.75La0.125Tb0.125', '-2.097', '-0.39979479573']
BrLaTc 5 ['Br0.7Tc0.2La0.1', '-1.453', '-0.228860302976']
BrLaTe 21 ['Br0.333333Te0.5La0.166667', '-1.446', '-0.208658558686']
BrLaTh 2 ['Br0.75La0.125Th0.125', '-2.28', '-0.218137388659']
BrLaTl 15 ['Br0.666667La0.166667Tl0.166667', '-2.053', '-0.202029526723']
BrLaTm 5 ['Br0.714286La0.142857Tm0.142857', '-2.09', '-0.259900580419']
BrLaV 3 ['V0.125Br0.75La0.125', '-1.858', '-0.34557981849']
BrLaW 3 ['Br0.75La0.125W0.125', '-1.684', '-0.20185753108']
BrLaY 6 ['Br0.666667Y0.222222La0.111111', '-1.873', '-0.208711918946']
BrLaYb 3 ['Br0.6La0.1Yb0.3', '-2.317', '-0.228414008551']
BrLaZn 10 ['Zn0.166667Br0.666667La0.166667', '-1.97', '-0.206113945892']
BrLaZr 2 ['Br0.777778Zr0.111111La0.111111', '-2.052', '-0.325849168391']

BrLiLu 19 ['Li0.111111Br0.555556Lu0.333333', '-1.305', '-0.27767507837']
BrLiMg 1 ['Li0.1Mg0.3Br0.6', '-1.844', '-0.213398525757']
BrLiMo 3 ['Li0.125Br0.75Mo0.125', '-1.121', '-0.295709351916']
BrLiNa 19 ['Li0.25Na0.25Br0.5', '-1.954', '-0.20235975358']
BrLiNd 1 ['Li0.3Br0.5Nd0.2', '-1.863', '-0.268749351958']
BrLiNp 3 ['Li0.125Br0.75Np0.125', '-1.43', '-0.209520787072']
BrLiO 51 ['Li0.142857O0.571429Br0.285714', '-0.806', '-0.210941362022']
BrLiOs 3 ['Li0.1Br0.8Os0.1', '-0.612', '-0.270543395258']
BrLiPm 19 ['Li0.222222Br0.555556Pm0.222222', '-1.695', '-0.367995793924']
BrLiPt 15 ['Li0.2Br0.7Pt0.1', '-0.885', '-0.202086790516']
BrLiPu 14 ['Li0.2Br0.7Pu0.1', '-1.629', '-0.236249938292']
BrLiRe 5 ['Li0.2Br0.7Re0.1', '-0.905', '-0.222086790516']
BrLiRu 1 ['Li0.125Br0.75Ru0.125', '-0.933', '-0.236772546291']
BrLiS 2 ['Li0.571429S0.285714Br0.142857', '-1.632', '-0.216279268081']
BrLiSb 3 ['Li0.1Br0.8Sb0.1', '-0.875', '-0.200351731783']
BrLiSc 24 ['Li0.25Sc0.25Br0.5', '-1.523', '-0.214105537541']
BrLiSe 14 ['Li0.25Se0.25Br0.5', '-1.175', '-0.202031641291']
BrLiSi 5 ['Li0.166667Si0.166667Br0.666667', '-1.497', '-0.253286061932']
BrLiSr 3 ['Li0.4Br0.5Sr0.1', '-1.953', '-0.22000528629']
BrLiTa 4 ['Li0.142857Br0.714286Ta0.142857', '-1.323', '-0.310004502302']
BrLiTb 10 ['Li0.222222Br0.666667Tb0.111111', '-1.477', '-0.213229242027']
BrLiTc 2 ['Li0.142857Br0.714286Tc0.142857', '-1.046', '-0.214430557905']
BrLiTe 26 ['Li0.428571Br0.285714Te0.285714', '-1.401', '-0.201809646927']
BrLiTh 2 ['Li0.125Br0.75Th0.125', '-1.878', '-0.207501913491']
BrLiTm 21 ['Li0.111111Br0.777778Tm0.111111', '-1.192', '-0.200730836704']
BrLiV 3 ['Li0.1V0.2Br0.7', '-1.163', '-0.20823848629']
BrLiW 1 ['Li0.125Br0.75W0.125', '-1.169', '-0.200183245193']
BrLiY 20 ['Li0.125Br0.5Y0.375', '-1.483', '-0.208613921915']
BrLiYb 11 ['Li0.375Br0.5Yb0.125', '-1.951', '-0.227114325041']
BrLiZr 5 ['Li0.142857Br0.571429Zr0.285714', '-1.686', '-0.237761570047']
BrLuMg 11 ['Mg0.1Br0.6Lu0.3', '-1.45', '-0.351451519874']
BrLuMn 23 ['Mn0.3Br0.5Lu0.2', '-1.058', '-0.209832535225']
BrLuMo 16 ['Br0.555556Mo0.111111Lu0.333333', '-1.2', '-0.256186652639']
BrLuN 43 ['N0.444444Br0.111111Lu0.444444', '-1.978', '-0.214840377627']
BrLuNa 19 ['Na0.111111Br0.555556Lu0.333333', '-1.425', '-0.377960757166']
BrLuNb 11 ['Br0.571429Nb0.142857Lu0.285714', '-1.264', '-0.257312286657']
BrLuNd 6 ['Br0.666667Nd0.111111Lu0.222222', '-1.711', '-0.329246147548']
BrLuNi 39 ['Ni0.4Br0.5Lu0.1', '-0.792', '-0.208973326792']
BrLuNp 7 ['Br0.7Lu0.1Np0.2', '-1.746', '-0.281767860558']
BrLuO 39 ['O0.285714Br0.428571Lu0.285714', '-2.935', '-0.202755887629']
BrLuOs 42 ['Br0.6Lu0.1Os0.3', '-0.4', '-0.205621391758']
BrLuP 43 ['P0.375Br0.375Lu0.25', '-1.357', '-0.201877547859']
BrLuPa 10 ['Br0.625Lu0.25Pa0.125', '-1.503', '-0.205803431617']
BrLuPb 30 ['Br0.6Lu0.1Pb0.3', '-1.266', '-0.212261559781']

BrLuPd 50 ['Br0.5Pd0.4Lu0.1', '-0.869', '-0.212439881935']
BrLuPm 19 ['Br0.555556Pm0.333333Lu0.111111', '-1.352', '-0.28370614198']
BrLuPr 6 ['Br0.666667Pr0.111111Lu0.222222', '-1.754', '-0.368214305045']
BrLuPt 59 ['Br0.444444Lu0.111111Pt0.444444', '-0.689', '-0.203462830556']
BrLuPu 12 ['Br0.571429Lu0.285714Pu0.142857', '-1.521', '-0.229263627906']
BrLuRb 19 ['Br0.555556Rb0.111111Lu0.333333', '-1.408', '-0.331719329481']
BrLuRe 20 ['Br0.5Lu0.3Re0.2', '-0.834', '-0.250864175275']
BrLuRh 46 ['Br0.2Rh0.4Lu0.4', '-1.141', '-0.200382438017']
BrLuRu 30 ['Br0.5Ru0.3Lu0.2', '-0.85', '-0.245717425292']
BrLuS 41 ['S0.166667Br0.333333Lu0.5', '-1.631', '-0.202317414361']
BrLuSb 50 ['Br0.5Sb0.375Lu0.125', '-1.043', '-0.232502317125']
BrLuSc 25 ['Sc0.4Br0.5Lu0.1', '-1.145', '-0.222216670792']
BrLuSe 64 ['Se0.5Br0.375Lu0.125', '-0.944', '-0.214302590183']
BrLuSi 34 ['Si0.166667Br0.333333Lu0.5', '-1.141', '-0.227647425028']
BrLuSm 6 ['Br0.666667Sm0.111111Lu0.222222', '-1.659', '-0.284012281712']
BrLuSn 39 ['Br0.4Sn0.1Lu0.5', '-1.122', '-0.206324132803']
BrLuSr 11 ['Br0.6Sr0.1Lu0.3', '-1.555', '-0.263239275791']
BrLuTa 12 ['Br0.571429Lu0.285714Ta0.142857', '-1.226', '-0.220460820967']
BrLuTb 11 ['Br0.625Tb0.125Lu0.25', '-1.363', '-0.308952888531']
BrLuTc 32 ['Br0.4Tc0.3Lu0.3', '-0.889', '-0.2256501737']
BrLuTe 69 ['Br0.2Te0.3Lu0.5', '-1.27', '-0.229917944015']
BrLuTh 5 ['Br0.714286Lu0.142857Th0.142857', '-1.907', '-0.207970753276']
BrLuTi 12 ['Ti0.2Br0.6Lu0.2', '-1.401', '-0.237517306711']
BrLuTl 46 ['Br0.444444Lu0.111111Tl0.444444', '-1.147', '-0.20103643573']
BrLuTm 13 ['Br0.6Tm0.2Lu0.2', '-1.352', '-0.207670977551']
BrLuU 6 ['Br0.666667Lu0.222222U0.111111', '-1.605', '-0.299642624502']
BrLuV 11 ['V0.222222Br0.666667Lu0.111111', '-1.257', '-0.359573869767']
BrLuW 17 ['Br0.5Lu0.4W0.1', '-1.083', '-0.2020620535']
BrLuXe 30 ['Br0.5Xe0.125Lu0.375', '-0.941', '-0.212080219094']
BrLuY 18 ['Br0.571429Y0.142857Lu0.285714', '-1.376', '-0.294120886477']
BrLuYb 13 ['Br0.6Yb0.2Lu0.2', '-2.03', '-0.248852122551']
BrLuZn 40 ['Zn0.3Br0.3Lu0.4', '-0.955', '-0.236604915525']
BrLuZr 12 ['Br0.666667Zr0.222222Lu0.111111', '-1.672', '-0.22699542728']
BrMgMo 1 ['Mg0.1Br0.8Mo0.1', '-1.098', '-0.263811430874']
BrMgNp 3 ['Mg0.125Br0.75Np0.125', '-1.702', '-0.264075723749']
BrMgO 12 ['O0.5Mg0.25Br0.25', '-1.75', '-0.204874435105']
BrMgOs 3 ['Mg0.125Br0.75Os0.125', '-0.911', '-0.266734180749']
BrMgPm 13 ['Mg0.166667Br0.5Pm0.333333', '-1.51', '-0.214694124484']
BrMgPt 5 ['Mg0.125Br0.75Pt0.125', '-0.848', '-0.203734180749']
BrMgPu 14 ['Mg0.222222Br0.555556Pu0.222222', '-1.609', '-0.200736005693']
BrMgRb 1 ['Mg0.3Br0.6Rb0.1', '-1.898', '-0.223951582756']
BrMgRe 4 ['Mg0.142857Br0.714286Re0.142857', '-1.018', '-0.28169620657']
BrMgRu 2 ['Mg0.111111Br0.777778Ru0.111111', '-1.02', '-0.207846651527']
BrMgS 1 ['Mg0.333333S0.166667Br0.5', '-1.767', '-0.215194704566']

BrMgSb 6 ['Mg0.1Br0.8Sb0.1', '-1.064', '-0.215395681124']
BrMgSc 13 ['Mg0.125Sc0.375Br0.5', '-1.306', '-0.206481230145']
BrMgSi 3 ['Mg0.1Si0.2Br0.7', '-1.415', '-0.224967747768']
BrMgTa 3 ['Mg0.125Br0.75Ta0.125', '-1.424', '-0.32018387619']
BrMgTb 9 ['Mg0.222222Br0.666667Tb0.111111', '-1.803', '-0.216553524879']
BrMgTc 3 ['Mg0.1Br0.8Tc0.1', '-0.958', '-0.201945339874']
BrMgTe 7 ['Mg0.1Br0.7Te0.2', '-0.806', '-0.209628385441']
BrMgTm 12 ['Mg0.2Br0.7Tm0.1', '-1.816', '-0.234489046974']
BrMgV 2 ['Mg0.142857V0.142857Br0.714286', '-1.38', '-0.205621271593']
BrMgY 22 ['Mg0.285714Br0.571429Y0.142857', '-1.726', '-0.25339241314']
BrMgYb 7 ['Mg0.1Br0.5Yb0.4', '-1.963', '-0.207166429501']
BrMgZr 3 ['Mg0.1Br0.8Zr0.1', '-1.397', '-0.231335286875']
BrMnNp 2 ['Mn0.111111Br0.777778Np0.111111', '-1.266', '-0.220221928377']
BrMnO 7 ['O0.571429Mn0.142857Br0.285714', '-1.036', '-0.204271241079']
BrMnOs 3 ['Mn0.125Br0.75Os0.125', '-0.591', '-0.208158126424']
BrMnPm 16 ['Mn0.125Br0.5Pm0.375', '-1.361', '-0.210721733242']
BrMnPt 14 ['Mn0.111111Br0.777778Pt0.111111', '-0.58', '-0.239696112377']
BrMnPu 9 ['Mn0.111111Br0.555556Pu0.333333', '-1.521', '-0.206487310696']
BrMnRe 3 ['Mn0.125Br0.75Re0.125', '-0.587', '-0.204158126424']
BrMnRh 3 ['Mn0.166667Br0.666667Rh0.166667', '-1.017', '-0.20922049276']
BrMnRu 4 ['Mn0.111111Br0.666667Ru0.222222', '-0.862', '-0.202399285377']
BrMnSb 2 ['Mn0.1Br0.8Sb0.1', '-0.85', '-0.210534837664']
BrMnSc 19 ['Sc0.3Mn0.2Br0.5', '-1.084', '-0.226609140235']
BrMnSe 1 ['Mn0.222222Se0.222222Br0.555556', '-0.942', '-0.208358070596']
BrMnTa 3 ['Mn0.125Br0.75Ta0.125', '-1.061', '-0.218607821865']
BrMnTb 8 ['Mn0.2Br0.7Tb0.1', '-1.272', '-0.204972529586']
BrMnTe 6 ['Mn0.125Br0.625Te0.25', '-0.699', '-0.214959427476']
BrMnTm 18 ['Mn0.111111Br0.444444Tm0.444444', '-1.111', '-0.200394827813']
BrMnY 16 ['Mn0.2Br0.5Y0.3', '-1.197', '-0.212674243413']
BrMnZr 3 ['Mn0.125Br0.75Zr0.125', '-1.431', '-0.235343054269']
BrMoN 3 ['N0.125Br0.625Mo0.25', '-0.967', '-0.235659872969']
BrMoNa 3 ['Na0.1Br0.8Mo0.1', '-0.945', '-0.267024592449']
BrMoNd 1 ['Br0.8Mo0.1Nd0.1', '-1.406', '-0.232402835551']
BrMoNi 4 ['Ni0.1Br0.7Mo0.2', '-0.973', '-0.213076915892']
BrMoNp 3 ['Br0.75Mo0.125Np0.125', '-1.488', '-0.295871650844']
BrMoO 2 ['O0.571429Br0.285714Mo0.142857', '-1.516', '-0.21007776381']
BrMoOs 5 ['Br0.7Mo0.2Os0.1', '-0.839', '-0.20144817255']
BrMoP 2 ['P0.111111Br0.777778Mo0.111111', '-1.024', '-0.300464594996']
BrMoPb 1 ['Br0.8Mo0.1Pb0.1', '-0.943', '-0.289092191043']
BrMoPd 4 ['Br0.714286Mo0.142857Pd0.142857', '-0.882', '-0.201901804703']
BrMoPm 15 ['Br0.555556Mo0.111111Pm0.333333', '-1.436', '-0.287797720138']
BrMoPr 3 ['Br0.75Mo0.125Pr0.125', '-1.704', '-0.232467721624']
BrMoPt 15 ['Br0.555556Mo0.111111Pt0.333333', '-0.608', '-0.253804540306']
BrMoPu 12 ['Br0.6Mo0.2Pu0.2', '-1.64', '-0.220326295552']

BrMoRb 3 ['Br0.75Rb0.125Mo0.125', '-1.111', '-0.21632961025']
BrMoRe 3 ['Br0.75Mo0.125Re0.125', '-0.647', '-0.248530107844']
BrMoRh 10 ['Br0.625Mo0.25Rh0.125', '-1.004', '-0.223859624418']
BrMoRu 6 ['Br0.7Mo0.1Ru0.2', '-0.868', '-0.242494881726']
BrMoS 30 ['S0.285714Br0.428571Mo0.285714', '-1.136', '-0.228398058533']
BrMoSb 13 ['Br0.5Mo0.3Sb0.2', '-0.876', '-0.206909679417']
BrMoSc 20 ['Sc0.3Br0.5Mo0.2', '-1.065', '-0.252400271375']
BrMoSe 28 ['Se0.444444Br0.333333Mo0.222222', '-0.793', '-0.203446853195']
BrMoSi 2 ['Si0.125Br0.75Mo0.125', '-1.215', '-0.207510168294']
BrMoSm 3 ['Br0.75Mo0.125Sm0.125', '-1.666', '-0.206615445374']
BrMoTa 1 ['Br0.8Mo0.1Ta0.1', '-0.961', '-0.274583842629']
BrMoTb 9 ['Br0.666667Mo0.222222Tb0.111111', '-1.303', '-0.270145674882']
BrMoTc 5 ['Br0.714286Mo0.142857Tc0.142857', '-0.934', '-0.209802581965']
BrMoTe 7 ['Br0.7Mo0.2Te0.1', '-0.881', '-0.202968692971']
BrMoTh 2 ['Br0.777778Mo0.111111Th0.111111', '-1.734', '-0.274313579789']
BrMoTi 1 ['Ti0.1Br0.8Mo0.1', '-1.251', '-0.20222098068']
BrMoTl 6 ['Br0.7Mo0.2Tl0.1', '-1.047', '-0.202149579308']
BrMoTm 12 ['Br0.625Mo0.25Tm0.125', '-1.242', '-0.220772712345']
BrMoV 2 ['V0.111111Br0.777778Mo0.111111', '-0.902', '-0.207079590879']
BrMoXe 9 ['Br0.6Mo0.1Xe0.3', '-0.555', '-0.236224086275']
BrMoY 17 ['Br0.555556Y0.222222Mo0.222222', '-1.215', '-0.237603255881']
BrMoYb 2 ['Br0.8Mo0.1Yb0.1', '-1.232', '-0.217028755792']
BrMoZn 1 ['Zn0.1Br0.8Mo0.1', '-0.874', '-0.245128202793']
BrMoZr 2 ['Br0.777778Zr0.111111Mo0.111111', '-1.408', '-0.331302253946']
BrNNp 19 ['N0.222222Br0.555556Np0.222222', '-1.489', '-0.223398047996']
BrNOs 4 ['N0.333333Br0.444444Os0.222222', '-0.211', '-0.211']
BrNPa 35 ['N0.125Br0.625Pa0.25', '-1.73', '-0.20161692294']
BrNPm 48 ['N0.1Br0.8Pm0.1', '-0.538', '-0.229691982216']
BrNPr 5 ['N0.142857Br0.428571Pr0.428571', '-1.86', '-0.220689468273']
BrNPt 2 ['N0.111111Br0.666667Pt0.222222', '-0.205', '-0.205']
BrNPu 27 ['N0.428571Br0.285714Pu0.285714', '-1.408', '-0.210394610198']
BrNRe 13 ['N0.2Br0.5Re0.3', '-0.243', '-0.208132832966']
BrNSc 47 ['N0.142857Sc0.571429Br0.285714', '-1.301', '-0.203398246404']
BrNSm 3 ['N0.2Br0.5Sm0.3', '-2.04', '-0.202388438995']
BrNTa 4 ['N0.166667Br0.666667Ta0.166667', '-0.824', '-0.211266260589']
BrNTb 31 ['N0.166667Br0.666667Tb0.166667', '-0.971', '-0.213532545513']
BrNTe 2 ['N0.111111Br0.666667Te0.222222', '-0.332', '-0.242045600935']
BrNTh 1 ['N0.285714Br0.285714Th0.428571', '-2.054', '-0.227022690637']
BrNTi 15 ['N0.444444Ti0.333333Br0.222222', '-1.522', '-0.220724396989']
BrNTm 40 ['N0.222222Br0.555556Tm0.222222', '-1.453', '-0.290298523832']
BrNV 3 ['N0.1V0.2Br0.7', '-0.881', '-0.267695091032']
BrNW 3 ['N0.2Br0.6W0.2', '-0.793', '-0.228139431175']
BrNY 38 ['N0.4Br0.3Y0.3', '-1.438', '-0.208811995208']
BrNYb 2 ['N0.1Br0.5Yb0.4', '-2.239', '-0.236664983257']

BrNZr 8 ['N0.1Br0.6Zr0.3', '-1.87', '-0.212462171018']
BrNaNd 1 ['Na0.25Br0.5Nd0.25', '-1.829', '-0.218650223165']
BrNaNp 4 ['Na0.2Br0.7Np0.1', '-1.556', '-0.202674246748']
BrNaO 17 ['O0.333333Na0.111111Br0.555556', '-0.745', '-0.200766553549']
BrNaOs 7 ['Na0.222222Br0.666667Os0.111111', '-1.045', '-0.246778902609']
BrNaP 1 ['Na0.2P0.2Br0.6', '-1.227', '-0.200692471065']
BrNaPm 19 ['Na0.222222Br0.555556Pm0.222222', '-1.77', '-0.403567151516']
BrNaPr 7 ['Na0.142857Br0.571429Pr0.285714', '-1.964', '-0.209560827499']
BrNaPt 22 ['Na0.1Br0.8Pt0.1', '-0.593', '-0.233800506174']
BrNaPu 12 ['Na0.2Br0.6Pu0.2', '-1.87', '-0.205151876049']
BrNaRe 9 ['Na0.2Br0.7Re0.1', '-0.942', '-0.223601012348']
BrNaRh 1 ['Na0.222222Br0.555556Rh0.222222', '-1.317', '-0.221455226804']
BrNaRu 2 ['Na0.125Br0.75Ru0.125', '-0.949', '-0.230593934936']
BrNaS 2 ['Na0.3S0.2Br0.5', '-1.416', '-0.203304437236']
BrNaSb 5 ['Na0.142857Br0.571429Sb0.285714', '-1.195', '-0.205869775285']
BrNaSc 18 ['Na0.111111Sc0.111111Br0.777778', '-1.038', '-0.210863309562']
BrNaSe 10 ['Na0.2Se0.3Br0.5', '-1.08', '-0.218408796123']
BrNaSi 1 ['Na0.4Si0.1Br0.5', '-1.785', '-0.21327810533']
BrNaSm 1 ['Na0.285714Br0.571429Sm0.142857', '-2.042', '-0.207399798615']
BrNaTa 4 ['Na0.142857Br0.714286Ta0.142857', '-1.316', '-0.277657517896']
BrNaTb 13 ['Na0.285714Br0.571429Tb0.142857', '-1.852', '-0.258606422202']
BrNaTc 2 ['Na0.111111Br0.777778Tc0.111111', '-0.89', '-0.223509446055']
BrNaTe 53 ['Na0.5Br0.1Te0.4', '-1.166', '-0.200064105168']
BrNaTm 23 ['Na0.166667Br0.5Tm0.333333', '-1.487', '-0.205380297817']
BrNaV 5 ['Na0.222222V0.111111Br0.666667', '-1.363', '-0.224053953182']
BrNaY 18 ['Na0.222222Br0.666667Y0.111111', '-1.821', '-0.206354691065']
BrNaYb 8 ['Na0.222222Br0.444444Yb0.333333', '-1.779', '-0.207228535406']
BrNaZr 3 ['Na0.222222Br0.666667Zr0.111111', '-1.73', '-0.209276616249']
BrNbNp 4 ['Br0.714286Nb0.142857Np0.142857', '-1.599', '-0.240641502204']
BrNbO 3 ['O0.6Br0.1Nb0.3', '-2.782', '-0.206948752418']
BrNbOs 1 ['Br0.8Nb0.1Os0.1', '-0.873', '-0.20499275229']
BrNbP 2 ['P0.111111Br0.777778Nb0.111111', '-1.199', '-0.285709424054']
BrNbPa 1 ['Br0.777778Nb0.111111Pa0.111111', '-1.722', '-0.231953548539']
BrNbPb 1 ['Br0.714286Nb0.142857Pb0.142857', '-1.339', '-0.208117555675']
BrNbPd 2 ['Br0.666667Nb0.166667Pd0.166667', '-1.152', '-0.204369464331']
BrNbPm 12 ['Br0.555556Nb0.111111Pm0.333333', '-1.409', '-0.205655503519']
BrNbPr 3 ['Br0.777778Nb0.111111Pr0.111111', '-1.855', '-0.27644839919']
BrNbPt 11 ['Br0.6Nb0.1Pt0.3', '-0.87', '-0.20199275229']
BrNbPu 7 ['Br0.7Nb0.1Pu0.2', '-1.802', '-0.224364204123']
BrNbRe 1 ['Br0.8Nb0.1Re0.1', '-0.915', '-0.24699275229']
BrNbRh 7 ['Br0.8Nb0.1Rh0.1', '-1.138', '-0.202401444065']
BrNbRu 3 ['Br0.714286Nb0.142857Ru0.142857', '-1.175', '-0.220703931843']
BrNbSb 8 ['Br0.666667Nb0.111111Sb0.222222', '-1.08', '-0.20061644086']
BrNbSc 17 ['Sc0.3Br0.5Nb0.2', '-1.082', '-0.219772276418']

BrNbSe 3 ['Se0.125Br0.75Nb0.125', '-1.108', '-0.213327516936']
BrNbSi 5 ['Si0.2Br0.7Nb0.1', '-1.255', '-0.213439153212']
BrNbSm 3 ['Br0.75Nb0.125Sm0.125', '-1.852', '-0.220478067786']
BrNbSn 2 ['Br0.75Nb0.125Sn0.125', '-1.309', '-0.226348517912']
BrNbTb 7 ['Br0.666667Nb0.111111Tb0.222222', '-1.573', '-0.258240509434']
BrNbTc 1 ['Br0.75Nb0.125Tc0.125', '-1.161', '-0.225723438394']
BrNbTe 2 ['Br0.777778Nb0.111111Te0.111111', '-1.038', '-0.250792525234']
BrNbTh 3 ['Br0.777778Nb0.111111Th0.111111', '-1.876', '-0.263302577488']
BrNbTm 10 ['Br0.7Nb0.2Tm0.1', '-1.429', '-0.246465992062']
BrNbY 12 ['Br0.571429Y0.285714Nb0.142857', '-1.349', '-0.251915683798']
BrNbYb 4 ['Br0.7Nb0.1Yb0.2', '-2.072', '-0.223123523239']
BrNbZn 6 ['Zn0.142857Br0.714286Nb0.142857', '-1.319', '-0.223883286747']
BrNbZr 1 ['Br0.8Zr0.1Nb0.1', '-1.532', '-0.213740694566']
BrNdNi 3 ['Ni0.166667Br0.666667Nd0.166667', '-1.782', '-0.211588763391']
BrNdNp 1 ['Br0.8Nd0.1Np0.1', '-1.747', '-0.257251983676']
BrNdO 22 ['O0.3Br0.5Nd0.2', '-2.123', '-0.206827381519']
BrNdOs 6 ['Br0.428571Nd0.428571Os0.142857', '-1.446', '-0.201332030394']
BrNdP 1 ['P0.142857Br0.714286Nd0.142857', '-1.647', '-0.205892112335']
BrNdPd 8 ['Br0.666667Pd0.166667Nd0.166667', '-1.759', '-0.203220562974']
BrNdPm 6 ['Br0.666667Nd0.111111Pm0.222222', '-1.871', '-0.352986859213']
BrNdPt 28 ['Br0.444444Nd0.333333Pt0.222222', '-1.926', '-0.203196360409']
BrNdPu 1 ['Br0.8Nd0.1Pu0.1', '-1.845', '-0.280341897052']
BrNdRb 5 ['Br0.714286Rb0.142857Nd0.142857', '-1.979', '-0.207088529334']
BrNdRe 3 ['Br0.8Nd0.1Re0.1', '-1.183', '-0.328178749276']
BrNdRh 25 ['Br0.428571Rh0.285714Nd0.285714', '-1.705', '-0.20198291468']
BrNdRu 2 ['Br0.8Ru0.1Nd0.1', '-1.281', '-0.210653391051']
BrNdS 3 ['S0.125Br0.375Nd0.5', '-1.83', '-0.218294470467']
BrNdSb 7 ['Br0.3Sb0.3Nd0.4', '-1.796', '-0.224764928276']
BrNdSc 7 ['Sc0.3Br0.6Nd0.1', '-1.608', '-0.206875208551']
BrNdSe 5 ['Se0.2Br0.6Nd0.2', '-1.915', '-0.205357498552']
BrNdSi 4 ['Si0.166667Br0.666667Nd0.166667', '-1.886', '-0.236424716516']
BrNdTa 2 ['Br0.777778Nd0.111111Ta0.111111', '-1.597', '-0.238709450699']
BrNdTb 3 ['Br0.75Nd0.125Tb0.125', '-1.962', '-0.32537284573']
BrNdTc 4 ['Br0.714286Tc0.142857Nd0.142857', '-1.685', '-0.234643923037']
BrNdTe 25 ['Br0.333333Te0.333333Nd0.333333', '-1.978', '-0.213490427251']
BrNdTm 4 ['Br0.75Nd0.125Tm0.125', '-2.005', '-0.248116383815']
BrNdV 3 ['V0.125Br0.75Nd0.125', '-1.678', '-0.22615786849']
BrNdY 5 ['Br0.7Y0.2Nd0.1', '-1.801', '-0.20906242331']
BrNdZr 2 ['Br0.777778Zr0.111111Nd0.111111', '-1.912', '-0.239696323947']
BrNiNp 6 ['Ni0.166667Br0.666667Np0.166667', '-1.459', '-0.255079571931']
BrNiO 4 ['O0.5Ni0.333333Br0.166667', '-1.093', '-0.217859936438']
BrNiOs 4 ['Ni0.142857Br0.714286Os0.142857', '-0.514', '-0.264212882167']
BrNiP 10 ['P0.166667Ni0.166667Br0.666667', '-0.826', '-0.206019563733']
BrNiPa 2 ['Ni0.1Br0.8Pa0.1', '-1.408', '-0.208031394059']

BrNiPb 1 ['Ni0.25Br0.625Pb0.125', '-0.947', '-0.200239538804']
BrNiPm 43 ['Ni0.428571Br0.428571Pm0.142857', '-0.845', '-0.229933899321']
BrNiPr 9 ['Ni0.1Br0.8Pr0.1', '-1.245', '-0.211699108541']
BrNiPt 9 ['Ni0.125Br0.625Pt0.25', '-0.419', '-0.200436271896']
BrNiPu 32 ['Ni0.2Br0.7Pu0.1', '-1.304', '-0.24446118281']
BrNiRe 8 ['Ni0.222222Br0.666667Re0.111111', '-0.603', '-0.214442261149']
BrNiRh 1 ['Ni0.125Br0.75Rh0.125', '-0.799', '-0.245947136615']
BrNiRu 1 ['Ni0.111111Br0.777778Ru0.111111', '-0.658', '-0.224248510325']
BrNiS 2 ['S0.142857Ni0.285714Br0.571429', '-0.74', '-0.204386406189']
BrNiSb 6 ['Ni0.142857Br0.714286Sb0.142857', '-0.928', '-0.202224791489']
BrNiSc 35 ['Sc0.333333Ni0.333333Br0.333333', '-0.9', '-0.209430827195']
BrNiSe 7 ['Ni0.125Se0.375Br0.5', '-0.538', '-0.200109425042']
BrNiSi 8 ['Si0.1Ni0.2Br0.7', '-1.011', '-0.20902784881']
BrNiSm 15 ['Ni0.222222Br0.666667Sm0.111111', '-1.467', '-0.232546440333']
BrNiSn 2 ['Ni0.25Br0.625Sn0.125', '-0.927', '-0.219311090447']
BrNiTa 3 ['Ni0.125Br0.75Ta0.125', '-1.023', '-0.344885967338']
BrNiTb 28 ['Ni0.375Br0.5Tb0.125', '-0.933', '-0.20325485821']
BrNiTc 3 ['Ni0.125Br0.75Tc0.125', '-0.771', '-0.25163376599']
BrNiTe 22 ['Ni0.4Br0.2Te0.4', '-0.527', '-0.203349247684']
BrNiTh 4 ['Ni0.1Br0.8Th0.1', '-1.401', '-0.231207153052']
BrNiTm 42 ['Ni0.333333Br0.222222Tm0.444444', '-0.963', '-0.204117656408']
BrNiU 8 ['Ni0.1Br0.7U0.2', '-1.621', '-0.210406214011']
BrNiV 4 ['V0.142857Ni0.142857Br0.714286', '-0.936', '-0.24813794719']
BrNiXe 6 ['Ni0.2Br0.6Xe0.2', '-0.56', '-0.210298035034']
BrNiY 34 ['Ni0.142857Br0.428571Y0.428571', '-1.206', '-0.200627645948']
BrNiYb 4 ['Ni0.125Br0.625Yb0.25', '-2.067', '-0.217229809741']
BrNiZr 7 ['Ni0.222222Br0.666667Zr0.111111', '-1.26', '-0.246079409502']
BrNpO 11 ['O0.5Br0.3Np0.2', '-2.477', '-0.201799793179']
BrNpOs 5 ['Br0.7Os0.2Np0.1', '-0.861', '-0.2260732344']
BrNpP 4 ['P0.142857Br0.714286Np0.142857', '-1.369', '-0.242027091084']
BrNpPb 3 ['Br0.75Pb0.125Np0.125', '-1.428', '-0.21542667396']
BrNpPd 7 ['Br0.7Pd0.2Np0.1', '-1.205', '-0.255487588434']
BrNpPm 7 ['Br0.7Pm0.1Np0.2', '-1.799', '-0.273451180808']
BrNpPr 2 ['Br0.777778Pr0.111111Np0.111111', '-1.949', '-0.289692583804']
BrNpPt 18 ['Br0.5Pt0.2Np0.3', '-1.38', '-0.200551396833']
BrNpPu 2 ['Br0.777778Np0.111111Pu0.111111', '-1.765', '-0.270818202418']
BrNpRb 6 ['Br0.714286Rb0.142857Np0.142857', '-1.705', '-0.247223508083']
BrNpRe 3 ['Br0.75Re0.125Np0.125', '-1.164', '-0.370341543']
BrNpRh 18 ['Br0.5Rh0.2Np0.3', '-1.384', '-0.23128070075']
BrNpRu 8 ['Br0.666667Ru0.166667Np0.166667', '-1.394', '-0.216052413875']
BrNpS 21 ['S0.2Br0.7Np0.1', '-0.972', '-0.201976153114']
BrNpSb 2 ['Br0.777778Sb0.111111Np0.111111', '-1.363', '-0.287312856584']
BrNpSc 12 ['Sc0.333333Br0.555556Np0.111111', '-1.314', '-0.203856526574']
BrNpSe 11 ['Se0.6Br0.1Np0.3', '-1.153', '-0.208925088245']

BrNpSi 2 ['Si0.125Br0.75Np0.125', '-1.526', '-0.226376845377']
BrNpSm 2 ['Br0.777778Sm0.111111Np0.111111', '-1.956', '-0.307490560471']
BrNpSn 3 ['Br0.75Sn0.125Np0.125', '-1.541', '-0.260992848738']
BrNpSr 3 ['Br0.75Sr0.125Np0.125', '-1.911', '-0.231560418645']
BrNpTa 2 ['Br0.777778Ta0.111111Np0.111111', '-1.466', '-0.352036656393']
BrNpTb 4 ['Br0.7Tb0.2Np0.1', '-1.688', '-0.259120200787']
BrNpTc 5 ['Br0.7Tc0.2Np0.1', '-1.167', '-0.2112172281']
BrNpTe 8 ['Br0.625Te0.25Np0.125', '-1.107', '-0.212142844052']
BrNpTi 2 ['Ti0.111111Br0.777778Np0.111111', '-1.753', '-0.236411254228']
BrNpTl 17 ['Br0.625Tl0.25Np0.125', '-1.515', '-0.203095059895']
BrNpTm 8 ['Br0.7Tm0.1Np0.2', '-1.694', '-0.219260305058']
BrNpV 3 ['V0.125Br0.75Np0.125', '-1.463', '-0.286025974895']
BrNpXe 5 ['Br0.7Xe0.2Np0.1', '-0.847', '-0.2120732344']
BrNpY 10 ['Br0.7Y0.1Np0.2', '-1.714', '-0.218129049558']
BrNpYb 3 ['Br0.75Yb0.125Np0.125', '-1.904', '-0.240097379896']
BrNpZn 4 ['Zn0.222222Br0.666667Np0.111111', '-1.436', '-0.21369934353']
BrNpZr 2 ['Br0.777778Zr0.111111Np0.111111', '-1.697', '-0.26902352964']
BrOOs 7 ['O0.666667Br0.111111Os0.222222', '-1.303', '-0.212815495526']
BrOP 10 ['O0.555556P0.111111Br0.333333', '-1.205', '-0.218931753475']
BrOPa 19 ['O0.571429Br0.142857Pa0.285714', '-2.915', '-0.227580805656']
BrOPb 25 ['O0.2Br0.4Pb0.4', '-1.407', '-0.20816249136']
BrOPm 56 ['O0.4Br0.1Pm0.5', '-2.909', '-0.202213426155']
BrOPr 44 ['O0.222222Br0.333333Pr0.444444', '-2.589', '-0.200534904667']
BrOPt 7 ['O0.1Br0.6Pt0.3', '-0.341', '-0.222603894912']
BrOPu 51 ['O0.222222Br0.333333Pu0.444444', '-2.521', '-0.200393645866']
BrORb 10 ['O0.222222Br0.555556Rb0.222222', '-1.156', '-0.202299492582']
BrORe 7 ['O0.111111Br0.666667Re0.222222', '-0.512', '-0.227330067542']
BrOS 9 ['O0.571429S0.142857Br0.285714', '-1.08', '-0.206541377519']
BrOSb 26 ['O0.555556Br0.333333Sb0.111111', '-0.895', '-0.201699223913']
BrOSc 47 ['O0.6Sc0.1Br0.3', '-1.256', '-0.20671216756']
BrOSe 10 ['O0.555556Se0.111111Br0.333333', '-0.613', '-0.200367980616']
BrOSm 12 ['O0.625Br0.25Sm0.125', '-1.438', '-0.200227560541']
BrOSn 2 ['O0.428571Br0.285714Sn0.285714', '-1.801', '-0.206414369652']
BrOSr 6 ['O0.4Br0.3Sr0.3', '-2.289', '-0.20949224216']
BrOTa 5 ['O0.166667Br0.666667Ta0.166667', '-1.351', '-0.252971699206']
BrOTb 47 ['O0.428571Br0.428571Tb0.142857', '-1.584', '-0.207572287655']
BrOTc 6 ['O0.166667Br0.666667Tc0.166667', '-0.857', '-0.23134185154']
BrOTe 28 ['O0.111111Br0.555556Te0.333333', '-0.544', '-0.201843423931']
BrOTh 18 ['O0.2Br0.6Th0.2', '-2.468', '-0.201984062735']
BrOTi 10 ['O0.6Ti0.2Br0.2', '-2.203', '-0.200457825544']
BrOTl 10 ['O0.285714Br0.142857Tl0.571429', '-1.058', '-0.201854268332']
BrOTm 34 ['O0.625Br0.25Tm0.125', '-1.513', '-0.203098163901']
BrOU 17 ['O0.333333Br0.333333U0.333333', '-2.78', '-0.200440820008']
BrOV 9 ['O0.142857V0.285714Br0.571429', '-1.553', '-0.200675857023']

BrOXe 25 ['O0.1Br0.5Xe0.4', '-0.249', '-0.200044989179']
BrOY 64 ['O0.6Br0.3Y0.1', '-1.252', '-0.20251100806']
BrOYb 8 ['O0.6Br0.2Yb0.2', '-1.674', '-0.205716004952']
BrOZr 16 ['O0.2Br0.6Zr0.2', '-1.943', '-0.202853213099']
BrOsP 3 ['P0.125Br0.75Os0.125', '-0.779', '-0.256864842537']
BrOsPa 1 ['Br0.8Os0.1Pa0.1', '-1.311', '-0.285882376542']
BrOsPb 2 ['Br0.777778Os0.111111Pb0.111111', '-0.577', '-0.20463122752']
BrOsPm 25 ['Br0.428571Pm0.428571Os0.142857', '-1.36', '-0.264163051463']
BrOsPr 9 ['Br0.444444Pr0.444444Os0.111111', '-1.52', '-0.222871137982']
BrOsPt 12 ['Br0.6Os0.2Pt0.2', '-0.216', '-0.216']
BrOsPu 13 ['Br0.571429Os0.142857Pu0.285714', '-1.61', '-0.253033810716']
BrOsRb 4 ['Br0.714286Rb0.142857Os0.142857', '-0.906', '-0.220264983013']
BrOsRe 2 ['Br0.777778Re0.111111Os0.111111', '-0.258', '-0.258']
BrOsRh 4 ['Br0.714286Rh0.142857Os0.142857', '-0.619', '-0.236726702536']
BrOsS 28 ['S0.444444Br0.444444Os0.111111', '-0.555', '-0.220658460735']
BrOsSb 5 ['Br0.7Sb0.1Os0.2', '-0.543', '-0.209808336525']
BrOsSc 41 ['Sc0.375Br0.375Os0.25', '-0.893', '-0.210120574094']
BrOsSe 4 ['Se0.444444Br0.444444Os0.111111', '-0.468', '-0.20135434675']
BrOsSi 2 ['Si0.111111Br0.777778Os0.111111', '-0.844', '-0.24433813615']
BrOsSm 3 ['Br0.75Sm0.125Os0.125', '-1.308', '-0.24708533753']
BrOsSn 3 ['Br0.75Sn0.125Os0.125', '-0.872', '-0.279145928873']
BrOsTa 3 ['Br0.75Ta0.125Os0.125', '-0.714', '-0.254449695442']
BrOsTb 16 ['Br0.571429Tb0.285714Os0.142857', '-1.385', '-0.250781380552']
BrOsTc 3 ['Br0.75Tc0.125Os0.125', '-0.504', '-0.203197494094']
BrOsTe 13 ['Br0.666667Te0.111111Os0.222222', '-0.246', '-0.201022800468']
BrOsTh 3 ['Br0.6Os0.1Th0.3', '-1.784', '-0.213083842053']
BrOsTi 2 ['Ti0.111111Br0.777778Os0.111111', '-1.023', '-0.211885438228']
BrOsTl 5 ['Br0.7Os0.1Tl0.2', '-0.722', '-0.206557465783']
BrOsTm 30 ['Br0.111111Tm0.555556Os0.333333', '-0.524', '-0.20675520362']
BrOsV 3 ['V0.125Br0.75Os0.125', '-0.726', '-0.342684431895']
BrOsXe 8 ['Br0.8Xe0.1Os0.1', '-0.219', '-0.219']
BrOsY 18 ['Br0.1Y0.6Os0.3', '-0.649', '-0.201694747914']
BrOsYb 6 ['Br0.666667Yb0.222222Os0.111111', '-1.774', '-0.226899265593']
BrOsZn 2 ['Zn0.111111Br0.777778Os0.111111', '-0.555', '-0.210449018353']
BrOsZr 4 ['Br0.714286Zr0.142857Os0.142857', '-1.15', '-0.221068488966']
BrPPa 3 ['P0.1Br0.8Pa0.1', '-1.477', '-0.22095097058']
BrPPb 1 ['P0.428571Br0.142857Pb0.428571', '-0.443', '-0.203620074834']
BrPPd 1 ['P0.142857Br0.571429Pd0.285714', '-0.826', '-0.241508313151']
BrPPm 81 ['P0.714286Br0.142857Pm0.142857', '-0.654', '-0.203527447905']
BrPPr 19 ['P0.1Br0.5Pr0.4', '-1.978', '-0.200807136146']
BrPPt 17 ['P0.2Br0.7Pt0.1', '-0.832', '-0.204672736961']
BrPPu 21 ['P0.222222Br0.444444Pu0.333333', '-1.736', '-0.206576941342']
BrPRb 1 ['P0.125Br0.625Rb0.25', '-1.456', '-0.203543795692']
BrPRe 3 ['P0.125Br0.75Re0.125', '-0.75', '-0.227864842537']

BrPRh 14 ['P0.777778Br0.111111Rh0.111111', '-0.638', '-0.209678930268']
BrPRu 15 ['P0.5Br0.1Ru0.4', '-0.913', '-0.214980699178']
BrPSb 6 ['P0.142857Br0.714286Sb0.142857', '-0.912', '-0.216077236977']
BrPSc 43 ['P0.5Sc0.2Br0.3', '-1.083', '-0.204439021246']
BrPSe 20 ['P0.222222Se0.444444Br0.333333', '-0.532', '-0.204150716494']
BrPSi 1 ['Si0.125P0.125Br0.75', '-1.105', '-0.237937564867']
BrPSm 12 ['P0.222222Br0.333333Sm0.444444', '-1.907', '-0.208452800813']
BrPSn 7 ['P0.1Br0.7Sn0.2', '-1.068', '-0.204637787689']
BrPSr 23 ['P0.3Br0.5Sr0.2', '-1.697', '-0.202773065711']
BrPTa 7 ['P0.5Br0.1Ta0.4', '-0.861', '-0.226506263809']
BrPTb 51 ['P0.5Br0.3Tb0.2', '-0.999', '-0.204325731314']
BrPTc 6 ['P0.166667Br0.666667Tc0.166667', '-0.847', '-0.213943272491']
BrPTe 2 ['P0.111111Br0.777778Te0.111111', '-0.796', '-0.268667966408']
BrPTh 4 ['P0.142857Br0.714286Th0.142857', '-1.749', '-0.217687143163']
BrPTi 1 ['P0.1Ti0.1Br0.8', '-1.244', '-0.206088353122']
BrPTm 58 ['P0.444444Br0.111111Tm0.444444', '-1.823', '-0.200642910148']
BrPU 2 ['P0.111111Br0.777778U0.111111', '-1.464', '-0.290397352947']
BrPV 2 ['P0.1V0.1Br0.8', '-0.971', '-0.246639419546']
BrPW 3 ['P0.125Br0.75W0.125', '-0.917', '-0.234885356287']
BrPXe 2 ['P0.666667Br0.166667Xe0.166667', '-0.329', '-0.200704774465']
BrPY 61 ['P0.428571Br0.142857Y0.428571', '-1.803', '-0.202254440815']
BrPYb 23 ['P0.166667Br0.5Yb0.333333', '-2.151', '-0.20852281907']
BrPZn 1 ['P0.125Zn0.25Br0.625', '-1.115', '-0.243538872144']
BrPZr 4 ['P0.1Br0.7Zr0.2', '-1.58', '-0.202518749231']
BrPaPm 8 ['Br0.7Pm0.1Pa0.2', '-1.732', '-0.211236742311']
BrPaPr 1 ['Br0.777778Pr0.111111Pa0.111111', '-2.134', '-0.243079382843']
BrPaPt 9 ['Br0.7Pt0.1Pa0.2', '-1.813', '-0.218182383227']
BrPaPu 6 ['Br0.7Pa0.2Pu0.1', '-1.754', '-0.200784501311']
BrPaRe 1 ['Br0.8Re0.1Pa0.1', '-1.319', '-0.293882376542']
BrPaRh 5 ['Br0.714286Rh0.142857Pa0.142857', '-1.68', '-0.215546252203']
BrPaRu 1 ['Br0.777778Ru0.111111Pa0.111111', '-1.532', '-0.23333200488']
BrPaS 47 ['S0.2Br0.4Pa0.4', '-1.743', '-0.202629126731']
BrPaSb 4 ['Br0.666667Sb0.166667Pa0.166667', '-1.625', '-0.205324924723']
BrPaSc 8 ['Sc0.3Br0.6Pa0.1', '-1.412', '-0.204418993052']
BrPaSe 31 ['Se0.5Br0.333333Pa0.166667', '-1.136', '-0.211783180028']
BrPaSi 1 ['Si0.1Br0.7Pa0.2', '-1.745', '-0.253680443811']
BrPaSn 2 ['Br0.75Sn0.125Pa0.125', '-1.781', '-0.309681311979']
BrPaTa 1 ['Br0.8Ta0.1Pa0.1', '-1.614', '-0.221242132895']
BrPaTb 4 ['Br0.75Tb0.125Pa0.125', '-1.827', '-0.214438535356']
BrPaTe 22 ['Br0.1Te0.6Pa0.3', '-0.78', '-0.221725002375']
BrPaTi 4 ['Ti0.125Br0.75Pa0.125', '-1.758', '-0.219573268915']
BrPaTm 8 ['Br0.7Tm0.1Pa0.2', '-1.697', '-0.221087368686']
BrPaY 12 ['Br0.571429Y0.285714Pa0.142857', '-1.495', '-0.246822626119']
BrPaZn 7 ['Zn0.166667Br0.666667Pa0.166667', '-1.617', '-0.211368922558']

BrPbPm 29 ['Br0.6Pm0.1Pb0.3', '-1.349', '-0.247556442232']
BrPbPt 14 ['Br0.666667Pt0.111111Pb0.222222', '-0.976', '-0.23126245504']
BrPbPu 33 ['Br0.25Pb0.375Pu0.375', '-0.899', '-0.218260848772']
BrPbRe 5 ['Br0.7Re0.1Pb0.2', '-0.881', '-0.210736209536']
BrPbRh 8 ['Br0.75Rh0.125Pb0.125', '-0.962', '-0.208595995679']
BrPbSc 22 ['Sc0.125Br0.625Pb0.25', '-1.28', '-0.214543786618']
BrPbSm 10 ['Br0.375Sm0.375Pb0.25', '-1.491', '-0.209422200405']
BrPbSr 1 ['Br0.333333Sr0.444444Pb0.222222', '-1.693', '-0.237788899888']
BrPbTa 4 ['Br0.714286Ta0.142857Pb0.142857', '-1.257', '-0.253039801602']
BrPbTb 17 ['Br0.5Tb0.333333Pb0.166667', '-1.39', '-0.21057453953']
BrPbTe 12 ['Br0.666667Te0.111111Pb0.222222', '-1.014', '-0.224285255508']
BrPbTm 26 ['Br0.571429Tm0.142857Pb0.285714', '-1.261', '-0.202400878571']
BrPbV 5 ['V0.1Br0.8Pb0.1', '-0.855', '-0.213215650284']
BrPbY 40 ['Br0.222222Y0.444444Pb0.333333', '-0.996', '-0.204525127904']
BrPbYb 12 ['Br0.666667Yb0.111111Pb0.222222', '-1.72', '-0.201712087837']
BrPbZn 1 ['Zn0.3Br0.6Pb0.1', '-1.185', '-0.229676337804']
BrPbZr 4 ['Br0.777778Zr0.111111Pb0.111111', '-1.31', '-0.21512894116']
BrPdPm 66 ['Br0.4Pd0.5Pm0.1', '-0.696', '-0.204365477533']
BrPdPr 12 ['Br0.571429Pd0.142857Pr0.285714', '-2.03', '-0.20915607695']
BrPdPt 1 ['Br0.7Pd0.1Pt0.2', '-0.392', '-0.234707177017']
BrPdPu 30 ['Br0.4Pd0.2Pu0.4', '-1.334', '-0.215058210201']
BrPdRe 5 ['Br0.7Pd0.1Re0.2', '-0.407', '-0.249707177017']
BrPdSc 42 ['Sc0.5Br0.375Pd0.125', '-1.116', '-0.206081389719']
BrPdSi 4 ['Si0.2Br0.7Pd0.1', '-1.23', '-0.224067432786']
BrPdSm 15 ['Br0.625Pd0.25Sm0.125', '-1.461', '-0.203469308801']
BrPdSr 2 ['Br0.625Sr0.25Pd0.125', '-2.101', '-0.231129736925']
BrPdTa 11 ['Br0.6Pd0.3Ta0.1', '-0.871', '-0.212239213731']
BrPdTb 35 ['Br0.8Pd0.1Tb0.1', '-0.815', '-0.203226704325']
BrPdTe 14 ['Br0.5Pd0.25Te0.25', '-0.676', '-0.228206913186']
BrPdTh 3 ['Br0.714286Pd0.142857Th0.142857', '-1.736', '-0.202302462919']
BrPdTm 43 ['Br0.777778Pd0.111111Tm0.111111', '-1.008', '-0.221357260881']
BrPdU 5 ['Br0.75Pd0.125U0.125', '-1.431', '-0.204831846003']
BrPdV 2 ['V0.2Br0.7Pd0.1', '-0.991', '-0.220402268049']
BrPdY 50 ['Br0.4Y0.5Pd0.1', '-1.29', '-0.203279490032']
BrPdYb 7 ['Br0.444444Pd0.222222Yb0.333333', '-2.035', '-0.236468166704']
BrPdZr 12 ['Br0.666667Zr0.222222Pd0.111111', '-1.652', '-0.20699542728']
BrPmPr 6 ['Br0.666667Pr0.111111Pm0.222222', '-1.873', '-0.350955016711']
BrPmPt 64 ['Br0.4Pm0.1Pt0.5', '-0.56', '-0.20514095875']
BrPmPu 15 ['Br0.555556Pm0.222222Pu0.222222', '-1.571', '-0.214080635324']
BrPmRb 19 ['Br0.555556Rb0.111111Pm0.333333', '-1.592', '-0.31133039698']
BrPmRe 20 ['Br0.5Pm0.3Re0.2', '-1.019', '-0.251914136024']
BrPmRh 45 ['Br0.333333Rh0.444444Pm0.222222', '-0.902', '-0.234680356972']
BrPmRu 28 ['Br0.4Ru0.2Pm0.4', '-1.241', '-0.218218848032']
BrPmS 75 ['S0.5Br0.25Pm0.25', '-1.66', '-0.200050295815']

BrPmSb 70 ['Br0.142857Sb0.285714Pm0.571429', '-1.15', '-0.254601177869']
BrPmSc 21 ['Sc0.3Br0.5Pm0.2', '-1.322', '-0.264305883291']
BrPmSe 84 ['Se0.625Br0.25Pm0.125', '-0.727', '-0.202428102833']
BrPmSi 49 ['Si0.375Br0.125Pm0.5', '-0.852', '-0.203401168447']
BrPmSm 6 ['Br0.666667Pm0.222222Sm0.111111', '-1.823', '-0.311752993378']
BrPmSn 40 ['Br0.555556Sn0.333333Pm0.111111', '-1.179', '-0.201649961745']
BrPmSr 10 ['Br0.666667Sr0.222222Pm0.111111', '-2.164', '-0.3051721256']
BrPmTa 9 ['Br0.6Pm0.3Ta0.1', '-1.421', '-0.286273892377']
BrPmTb 10 ['Br0.625Pm0.25Tb0.125', '-1.538', '-0.330661189155']
BrPmTc 26 ['Br0.428571Tc0.285714Pm0.285714', '-1.062', '-0.216850603487']
BrPmTe 78 ['Br0.111111Te0.666667Pm0.222222', '-0.873', '-0.203020135193']
BrPmTh 4 ['Br0.7Pm0.2Th0.1', '-1.913', '-0.406667559551']
BrPmTi 13 ['Ti0.111111Br0.555556Pm0.333333', '-1.518', '-0.23527933069']
BrPmTl 32 ['Br0.4Pm0.2Tl0.4', '-1.16', '-0.203858866532']
BrPmTm 14 ['Br0.6Pm0.2Tm0.2', '-1.489', '-0.22203761805']
BrPmU 6 ['Br0.714286Pm0.142857U0.142857', '-1.779', '-0.290771843633']
BrPmV 12 ['V0.2Br0.6Pm0.2', '-1.366', '-0.241304515048']
BrPmW 17 ['Br0.5Pm0.4W0.1', '-1.352', '-0.225795334499']
BrPmXe 29 ['Br0.444444Xe0.333333Pm0.222222', '-0.784', '-0.215788248907']
BrPmY 15 ['Br0.6Y0.2Pm0.2', '-1.471', '-0.22249309805']
BrPmYb 12 ['Br0.625Pm0.125Yb0.25', '-2.291', '-0.230892563802']
BrPmZn 31 ['Zn0.1Br0.5Pm0.4', '-1.463', '-0.277813607473']
BrPmZr 9 ['Br0.625Zr0.125Pm0.25', '-1.668', '-0.215946707865']
BrPrPt 32 ['Br0.555556Pr0.333333Pt0.111111', '-2.075', '-0.217387631158']
BrPrPu 7 ['Br0.666667Pr0.166667Pu0.166667', '-2.044', '-0.218896344916']
BrPrRb 9 ['Br0.7Rb0.2Pr0.1', '-1.833', '-0.20351653354']
BrPrRe 3 ['Br0.75Pr0.125Re0.125', '-1.412', '-0.33893761378']
BrPrRh 27 ['Br0.6Rh0.3Pr0.1', '-1.331', '-0.204958782799']
BrPrRu 3 ['Br0.8Ru0.1Pr0.1', '-1.289', '-0.215024732799']
BrPrS 37 ['S0.3Br0.1Pr0.6', '-1.793', '-0.202476744902']
BrPrSb 39 ['Br0.2Sb0.3Pr0.5', '-1.537', '-0.200981844667']
BrPrSc 6 ['Sc0.222222Br0.666667Pr0.111111', '-1.807', '-0.448497478379']
BrPrSe 37 ['Se0.285714Br0.142857Pr0.571429', '-1.678', '-0.207961815512']
BrPrSi 21 ['Si0.1Br0.6Pr0.3', '-2.069', '-0.203049260798']
BrPrSn 2 ['Br0.777778Sn0.111111Pr0.111111', '-1.705', '-0.224185371247']
BrPrTa 3 ['Br0.75Pr0.125Ta0.125', '-1.834', '-0.301387309222']
BrPrTb 3 ['Br0.777778Pr0.111111Tb0.111111', '-1.817', '-0.358188464813']
BrPrTc 4 ['Br0.714286Tc0.142857Pr0.142857', '-1.786', '-0.330460125534']
BrPrTe 36 ['Br0.222222Te0.333333Pr0.444444', '-1.807', '-0.201656534961']
BrPrTi 2 ['Ti0.1Br0.8Pr0.1', '-1.794', '-0.205546985429']
BrPrTl 23 ['Br0.6Pr0.3Tl0.1', '-2.001', '-0.203961624798']
BrPrTm 5 ['Br0.714286Pr0.142857Tm0.142857', '-2.006', '-0.239948840059']
BrPrV 3 ['V0.125Br0.75Pr0.125', '-1.813', '-0.356622045675']
BrPrW 1 ['Br0.777778Pr0.111111W0.111111', '-1.563', '-0.211373943629']

BrPrY 6 ['Br0.666667Y0.222222Pr0.111111', '-1.843', '-0.228527231999']
BrPrYb 3 ['Br0.625Pr0.125Yb0.25', '-2.318', '-0.21546670424']
BrPrZn 15 ['Zn0.111111Br0.666667Pr0.222222', '-2.108', '-0.200333535609']
BrPrZr 2 ['Br0.777778Zr0.111111Pr0.111111', '-2.019', '-0.342664481444']
BrPtPu 31 ['Br0.5Pt0.1Pu0.4', '-1.565', '-0.220954650627']
BrPtRb 18 ['Br0.5Rb0.25Pt0.25', '-1.167', '-0.203208053145']
BrPtRe 15 ['Br0.555556Re0.111111Pt0.333333', '-0.201', '-0.201']
BrPtRh 5 ['Br0.8Rh0.1Pt0.1', '-0.526', '-0.258408691775']
BrPtRu 9 ['Br0.625Ru0.125Pt0.25', '-0.486', '-0.216593302219']
BrPtS 42 ['S0.5Br0.2Pt0.3', '-0.67', '-0.20035662301']
BrPtSb 17 ['Br0.571429Sb0.285714Pt0.142857', '-0.928', '-0.205099027905']
BrPtSc 54 ['Sc0.5Br0.125Pt0.375', '-1.352', '-0.201612594073']
BrPtSe 29 ['Se0.444444Br0.333333Pt0.222222', '-0.477', '-0.220606751111']
BrPtSi 15 ['Si0.3Br0.6Pt0.1', '-1.164', '-0.220323860053']
BrPtSm 38 ['Br0.222222Sm0.444444Pt0.333333', '-1.63', '-0.202787442981']
BrPtSn 13 ['Br0.6Sn0.1Pt0.3', '-0.69', '-0.215716743098']
BrPtSr 18 ['Br0.75Sr0.125Pt0.125', '-1.098', '-0.212218875645']
BrPtTa 14 ['Br0.6Ta0.2Pt0.2', '-1.044', '-0.308719512707']
BrPtTb 40 ['Br0.3Tb0.3Pt0.4', '-1.298', '-0.208894288662']
BrPtTc 12 ['Br0.571429Tc0.142857Pt0.285714', '-0.562', '-0.218225707536']
BrPtTe 41 ['Br0.25Te0.375Pt0.375', '-0.461', '-0.203055035625']
BrPtTh 22 ['Br0.625Pt0.125Th0.25', '-1.992', '-0.208404035367']
BrPtTi 9 ['Ti0.1Br0.6Pt0.3', '-0.992', '-0.261996894405']
BrPtTl 15 ['Br0.555556Pt0.111111Tl0.333333', '-1.048', '-0.244954302824']
BrPtTm 52 ['Br0.2Tm0.5Pt0.3', '-1.388', '-0.200835923515']
BrPtU 15 ['Br0.555556Pt0.222222U0.222222', '-1.53', '-0.212057131437']
BrPtV 16 ['V0.111111Br0.555556Pt0.333333', '-0.573', '-0.232275050573']
BrPtW 10 ['Br0.666667W0.222222Pt0.111111', '-0.859', '-0.23137714575']
BrPtXe 26 ['Br0.444444Xe0.444444Pt0.111111', '-0.228', '-0.228']
BrPtY 62 ['Br0.333333Y0.222222Pt0.444444', '-1.206', '-0.202053025307']
BrPtYb 37 ['Br0.444444Yb0.333333Pt0.222222', '-2.123', '-0.211381540037']
BrPtZn 8 ['Zn0.2Br0.7Pt0.1', '-0.823', '-0.202808233036']
BrPtZr 13 ['Br0.571429Zr0.142857Pt0.285714', '-1.184', '-0.255068488966']
BrPuRb 13 ['Br0.8Rb0.1Pu0.1', '-1.316', '-0.220646369034']
BrPuRe 4 ['Br0.75Re0.125Pu0.125', '-1.368', '-0.48070393472']
BrPuRh 32 ['Br0.333333Rh0.333333Pu0.333333', '-1.269', '-0.210645755862']
BrPuRu 15 ['Br0.5Ru0.166667Pu0.333333', '-1.474', '-0.237331351293']
BrPuS 72 ['S0.4Br0.1Pu0.5', '-1.997', '-0.225365838317']
BrPuSb 35 ['Br0.3Sb0.4Pu0.3', '-1.178', '-0.201900178776']
BrPuSc 20 ['Sc0.375Br0.5Pu0.125', '-1.343', '-0.228077459418']
BrPuSe 50 ['Se0.5Br0.125Pu0.375', '-1.673', '-0.216459958238']
BrPuSi 20 ['Si0.25Br0.625Pu0.125', '-1.447', '-0.222394136304']
BrPuSm 3 ['Br0.6Sm0.1Pu0.3', '-1.784', '-0.2254314178']
BrPuSn 26 ['Br0.5Sn0.1Pu0.4', '-1.534', '-0.260323571941']

BrPuSr 2 ['Br0.6Sr0.1Pu0.3', '-1.86', '-0.204925964217']
BrPuTa 4 ['Br0.714286Ta0.142857Pu0.142857', '-1.765', '-0.300775627051']
BrPuTb 9 ['Br0.7Tb0.1Pu0.2', '-1.857', '-0.203591961519']
BrPuTc 19 ['Br0.5Tc0.25Pu0.25', '-1.416', '-0.232938579627']
BrPuTe 20 ['Br0.555556Te0.111111Pu0.333333', '-1.767', '-0.206984323659']
BrPuTh 12 ['Br0.555556Th0.222222Pu0.222222', '-1.582', '-0.200136299354']
BrPuTi 7 ['Ti0.25Br0.625Pu0.125', '-1.574', '-0.202500511717']
BrPuTl 43 ['Br0.111111Tl0.555556Pu0.333333', '-0.49', '-0.208684823621']
BrPuTm 21 ['Br0.555556Tm0.333333Pu0.111111', '-1.464', '-0.219989800324']
BrPuU 3 ['Br0.777778U0.111111Pu0.111111', '-1.888', '-0.225887473679']
BrPuV 4 ['V0.142857Br0.714286Pu0.142857', '-1.658', '-0.205872418989']
BrPuW 3 ['Br0.777778W0.111111Pu0.111111', '-1.468', '-0.281499562242']
BrPuXe 4 ['Br0.666667Xe0.222222Pu0.111111', '-0.989', '-0.200292386418']
BrPuY 25 ['Br0.5Y0.4Pu0.1', '-1.374', '-0.212128309292']
BrPuYb 19 ['Br0.555556Yb0.111111Pu0.333333', '-1.768', '-0.205742019214']
BrPuZn 29 ['Zn0.3Br0.5Pu0.2', '-1.462', '-0.23168523321']
BrPuZr 14 ['Br0.571429Zr0.285714Pu0.142857', '-1.545', '-0.200295670763']
BrRbRe 5 ['Br0.7Rb0.2Re0.1', '-1.295', '-0.281436750625']
BrRbRh 1 ['Br0.75Rb0.125Rh0.125', '-1.022', '-0.205614891291']
BrRbRu 2 ['Br0.714286Rb0.142857Ru0.142857', '-1.063', '-0.204368375762']
BrRbS 7 ['S0.25Br0.25Rb0.5', '-1.7', '-0.205300657975']
BrRbSb 4 ['Br0.714286Rb0.142857Sb0.142857', '-1.298', '-0.208122752909']
BrRbSc 19 ['Sc0.111111Br0.555556Rb0.333333', '-2.026', '-0.538609426147']
BrRbSe 27 ['Se0.111111Br0.666667Rb0.222222', '-1.114', '-0.204261893083']
BrRbSi 10 ['Si0.125Br0.75Rb0.125', '-1.358', '-0.201484429741']
BrRbSm 2 ['Br0.555556Rb0.333333Sm0.111111', '-2.123', '-0.209253900507']
BrRbTa 5 ['Br0.714286Rb0.142857Ta0.142857', '-1.341', '-0.265061396588']
BrRbTb 14 ['Br0.571429Rb0.285714Tb0.142857', '-1.871', '-0.202414179585']
BrRbTc 8 ['Br0.666667Rb0.111111Tc0.222222', '-1.074', '-0.200014681537']
BrRbTe 17 ['Br0.75Rb0.125Te0.125', '-0.94', '-0.200432231026']
BrRbTh 2 ['Br0.7Rb0.2Th0.1', '-1.98', '-0.214024578051']
BrRbTi 1 ['Ti0.142857Br0.571429Rb0.285714', '-1.864', '-0.200504864504']
BrRbTm 14 ['Br0.6Rb0.1Tm0.3', '-1.577', '-0.23102525155']
BrRbU 3 ['Br0.8Rb0.1U0.1', '-1.509', '-0.221195109552']
BrRbV 3 ['V0.2Br0.7Rb0.1', '-1.317', '-0.212128635632']
BrRbY 18 ['Br0.571429Rb0.142857Y0.285714', '-1.625', '-0.224868055761']
BrRbYb 4 ['Br0.5Rb0.333333Yb0.166667', '-2.122', '-0.242104978999']
BrRbZr 10 ['Br0.666667Rb0.166667Zr0.166667', '-1.953', '-0.22671860589']
BrReRh 6 ['Br0.7Rh0.1Re0.2', '-0.475', '-0.207408691775']
BrReRu 5 ['Br0.7Ru0.2Re0.1', '-0.641', '-0.20994928355']
BrReS 35 ['S0.5Br0.3Re0.2', '-0.761', '-0.202988692102']
BrReSb 5 ['Br0.7Sb0.1Re0.2', '-0.65', '-0.316808336525']
BrReSc 21 ['Sc0.444444Br0.444444Re0.111111', '-1.049', '-0.239661421148']
BrReSe 27 ['Se0.444444Br0.333333Re0.222222', '-0.571', '-0.206963557241']

BrReSi 2 ['Si0.111111Br0.777778Re0.111111', '-0.867', '-0.26733813615']
BrReSm 5 ['Br0.7Sm0.1Re0.2', '-1.071', '-0.222268270024']
BrReSn 3 ['Br0.75Sn0.125Re0.125', '-0.866', '-0.273145928873']
BrReSr 2 ['Br0.777778Sr0.111111Re0.111111', '-1.054', '-0.266639000573']
BrReTa 3 ['Br0.75Ta0.125Re0.125', '-0.681', '-0.221449695442']
BrReTb 10 ['Br0.625Tb0.25Re0.125', '-1.306', '-0.241678763127']
BrReTc 2 ['Br0.777778Tc0.111111Re0.111111', '-0.518', '-0.25061999475']
BrReTe 7 ['Br0.666667Te0.111111Re0.222222', '-0.416', '-0.207424606037']
BrReTh 2 ['Br0.777778Re0.111111Th0.111111', '-1.48', '-0.374509039483']
BrReTi 2 ['Ti0.111111Br0.777778Re0.111111', '-1.047', '-0.235885438228']
BrReTl 8 ['Br0.666667Re0.166667Tl0.166667', '-0.673', '-0.243464554819']
BrReTm 11 ['Br0.625Tm0.25Re0.125', '-1.373', '-0.212410568491']
BrReU 1 ['Br0.8Re0.1U0.1', '-1.161', '-0.258711888294']
BrReV 3 ['V0.125Br0.75Re0.125', '-0.639', '-0.255684431895']
BrReXe 10 ['Br0.6Xe0.3Re0.1', '-0.278', '-0.278']
BrReY 14 ['Br0.5Y0.4Re0.1', '-1.27', '-0.223389579291']
BrReYb 6 ['Br0.7Yb0.2Re0.1', '-1.602', '-0.209609339034']
BrReZn 5 ['Zn0.2Br0.7Re0.1', '-0.872', '-0.251808233036']
BrReZr 3 ['Br0.75Zr0.125Re0.125', '-1.141', '-0.328184927845']
BrRhRu 1 ['Br0.75Ru0.125Rh0.125', '-0.881', '-0.277104166938']
BrRhS 10 ['S0.25Br0.5Rh0.25', '-0.815', '-0.208236580983']
BrRhSb 17 ['Br0.5Rh0.25Sb0.25', '-0.878', '-0.207879657334']
BrRhSc 42 ['Sc0.25Br0.375Rh0.375', '-1.071', '-0.222591055969']
BrRhSi 15 ['Si0.1Br0.6Rh0.3', '-1.014', '-0.270464759351']
BrRhSm 24 ['Br0.8Rh0.1Sm0.1', '-1.339', '-0.222676961799']
BrRhSn 7 ['Br0.777778Rh0.111111Sn0.111111', '-1.031', '-0.206694927637']
BrRhSr 3 ['Br0.666667Sr0.166667Rh0.166667', '-1.707', '-0.228634825055']
BrRhTa 9 ['Br0.625Rh0.25Ta0.125', '-1.072', '-0.283634685659']
BrRhTb 35 ['Br0.444444Rh0.111111Tb0.444444', '-1.381', '-0.228432471558']
BrRhTc 7 ['Br0.666667Tc0.166667Rh0.166667', '-0.799', '-0.219324483667']
BrRhTe 17 ['Br0.6Rh0.3Te0.1', '-0.811', '-0.207738831467']
BrRhTh 12 ['Br0.666667Rh0.111111Th0.222222', '-2.02', '-0.22358790867']
BrRhTi 8 ['Ti0.125Br0.625Rh0.25', '-1.247', '-0.222999739579']
BrRhTm 49 ['Br0.166667Rh0.333333Tm0.5', '-1.198', '-0.211815207097']
BrRhU 14 ['Br0.571429Rh0.285714U0.142857', '-1.335', '-0.212050826479']
BrRhV 11 ['V0.125Br0.625Rh0.25', '-0.947', '-0.221971666842']
BrRhW 5 ['Br0.8Rh0.1W0.1', '-0.953', '-0.251811892671']
BrRhXe 7 ['Br0.571429Rh0.142857Xe0.285714', '-0.587', '-0.204726702536']
BrRhY 48 ['Br0.25Y0.375Rh0.375', '-1.163', '-0.201329559188']
BrRhYb 18 ['Br0.5Rh0.166667Yb0.333333', '-2.11', '-0.230007800459']
BrRhZr 20 ['Br0.5Zr0.25Rh0.25', '-1.529', '-0.21728993546']
BrRuS 23 ['S0.375Br0.375Ru0.25', '-0.803', '-0.204583510589']
BrRuSb 10 ['Br0.625Ru0.125Sb0.25', '-0.952', '-0.241908318177']
BrRuSc 28 ['Sc0.1Br0.6Ru0.3', '-0.747', '-0.202240614765']

BrRuSe 14 ['Se0.4Br0.5Ru0.1', '-0.539', '-0.205787298992']
BrRuSi 9 ['Si0.125Br0.75Ru0.125', '-1.058', '-0.203775937981']
BrRuSm 6 ['Br0.666667Ru0.222222Sm0.111111', '-1.383', '-0.200492124221']
BrRuSn 4 ['Br0.7Ru0.1Sn0.2', '-1.073', '-0.209637787689']
BrRuTa 5 ['Br0.7Ru0.2Ta0.1', '-0.962', '-0.259098088004']
BrRuTb 18 ['Br0.5Ru0.166667Tb0.333333', '-1.38', '-0.211328329836']
BrRuTc 3 ['Br0.777778Tc0.111111Ru0.111111', '-0.711', '-0.2041473745']
BrRuTe 12 ['Br0.6Ru0.2Te0.2', '-0.678', '-0.215296626696']
BrRuTh 5 ['Br0.7Ru0.2Th0.1', '-1.44', '-0.22953277731']
BrRuTi 2 ['Ti0.1Br0.8Ru0.1', '-1.152', '-0.20647153618']
BrRuTm 25 ['Br0.444444Ru0.222222Tm0.333333', '-1.175', '-0.209056900409']
BrRuU 2 ['Br0.714286Ru0.142857U0.142857', '-1.497', '-0.208016983277']
BrRuV 7 ['V0.2Br0.7Ru0.1', '-1.038', '-0.209169732807']
BrRuXe 7 ['Br0.666667Ru0.166667Xe0.166667', '-0.567', '-0.207791069625']
BrRuY 31 ['Br0.375Y0.5Ru0.125', '-1.122', '-0.207271659718']
BrRuYb 1 ['Br0.666667Ru0.111111Yb0.222222', '-1.929', '-0.222250852093']
BrRuZr 8 ['Br0.666667Zr0.111111Ru0.222222', '-1.249', '-0.28702509339']
BrSSb 13 ['S0.375Br0.5Sb0.125', '-0.708', '-0.207074744853']
BrSSc 62 ['S0.5Sc0.333333Br0.166667', '-1.953', '-0.209901689658']
BrSSe 16 ['S0.1Se0.3Br0.6', '-0.411', '-0.200259243132']
BrSSi 5 ['Si0.2S0.1Br0.7', '-1.213', '-0.203952920111']
BrSSm 39 ['S0.375Br0.125Sm0.5', '-2.182', '-0.200315422357']
BrSSn 8 ['S0.285714Br0.428571Sn0.285714', '-1.018', '-0.205597268718']
BrSTa 16 ['S0.3Br0.6Ta0.1', '-0.82', '-0.209342294884']
BrSTb 63 ['S0.5Br0.25Tb0.25', '-1.545', '-0.200125558308']
BrSTc 49 ['S0.4Br0.2Tc0.4', '-0.845', '-0.200302547245']
BrSTe 38 ['S0.444444Br0.444444Te0.111111', '-0.532', '-0.202836130105']
BrSTh 33 ['S0.4Br0.5Th0.1', '-1.263', '-0.200509594892']
BrSTm 67 ['S0.222222Br0.222222Tm0.555556', '-1.58', '-0.204168335236']
BrSU 31 ['S0.555556Br0.111111U0.333333', '-1.791', '-0.200794345376']
BrSV 29 ['S0.4V0.2Br0.4', '-1.034', '-0.215048173735']
BrSW 24 ['S0.3Br0.6W0.1', '-0.799', '-0.207927897597']
BrSXe 17 ['S0.222222Br0.555556Xe0.222222', '-0.353', '-0.202892131904']
BrSY 70 ['S0.4Br0.1Y0.5', '-2.154', '-0.21518984115']
BrSYb 7 ['S0.2Br0.4Yb0.4', '-2.563', '-0.20708640823']
BrSZr 32 ['S0.125Br0.75Zr0.125', '-1.106', '-0.208749252041']
BrSbSc 43 ['Sc0.444444Br0.222222Sb0.333333', '-1.134', '-0.212635291477']
BrSbSe 10 ['Se0.333333Br0.555556Sb0.111111', '-0.682', '-0.205718732269']
BrSbSi 1 ['Si0.1Br0.8Sb0.1', '-1.085', '-0.21211265906']
BrSbSm 34 ['Br0.6Sb0.3Sm0.1', '-1.384', '-0.202076606549']
BrSbSr 25 ['Br0.375Sr0.25Sb0.375', '-1.656', '-0.201056972218']
BrSbTa 3 ['Br0.75Sb0.125Ta0.125', '-1.155', '-0.278960116098']
BrSbTb 38 ['Br0.2Sb0.4Tb0.4', '-1.131', '-0.210062567088']
BrSbTc 9 ['Br0.625Tc0.125Sb0.25', '-0.906', '-0.211850701094']

BrSbTe 5 ['Br0.714286Sb0.142857Te0.142857', '-0.736', '-0.202184081352']
BrSbTh 10 ['Br0.7Sb0.2Th0.1', '-1.547', '-0.21886647206']
BrSbTi 4 ['Ti0.111111Br0.777778Sb0.111111', '-1.407', '-0.225672478811']
BrSbTl 3 ['Br0.7Sb0.1Tl0.2', '-1.049', '-0.200365802309']
BrSbTm 49 ['Br0.555556Sb0.333333Tm0.111111', '-1.072', '-0.213318424585']
BrSbU 11 ['Br0.5Sb0.166667U0.333333', '-1.299', '-0.216664440252']
BrSbV 10 ['V0.25Br0.625Sb0.125', '-1.119', '-0.213539004009']
BrSbW 2 ['Br0.75Sb0.125W0.125', '-0.97', '-0.200472565141']
BrSbXe 7 ['Br0.777778Sb0.111111Xe0.111111', '-0.584', '-0.213787040584']
BrSbY 60 ['Br0.125Y0.5Sb0.375', '-1.373', '-0.214446797198']
BrSbYb 55 ['Br0.285714Sb0.428571Yb0.285714', '-1.493', '-0.21986768931']
BrSbZn 4 ['Zn0.1Br0.8Sb0.1', '-0.846', '-0.202712453043']
BrSbZr 7 ['Br0.714286Zr0.142857Sb0.142857', '-1.451', '-0.20474309518']
BrScSe 77 ['Sc0.25Se0.625Br0.125', '-1.063', '-0.206330700323']
BrScSi 41 ['Si0.25Sc0.125Br0.625', '-1.23', '-0.207362650242']
BrScSm 7 ['Sc0.3Br0.6Sm0.1', '-1.6', '-0.204964729299']
BrScSn 37 ['Sc0.5Br0.4Sn0.1', '-1.089', '-0.214486872485']
BrScSr 11 ['Sc0.1Br0.8Sr0.1', '-1.25', '-0.359273920274']
BrScTa 18 ['Sc0.3Br0.5Ta0.2', '-1.134', '-0.272576250435']
BrScTb 15 ['Sc0.333333Br0.555556Tb0.111111', '-1.285', '-0.236911047187']
BrScTc 29 ['Sc0.2Br0.5Tc0.3', '-0.817', '-0.212155634792']
BrScTe 67 ['Sc0.555556Br0.333333Te0.111111', '-1.093', '-0.212275357343']
BrScTh 5 ['Sc0.3Br0.6Th0.1', '-1.571', '-0.211855775052']
BrScTi 17 ['Sc0.4Ti0.1Br0.5', '-1.164', '-0.218753531913']
BrScTl 45 ['Sc0.125Br0.5Tl0.375', '-1.215', '-0.210003800041']
BrScTm 23 ['Sc0.3Br0.5Tm0.2', '-1.175', '-0.218924131791']
BrScU 13 ['Sc0.1Br0.7U0.2', '-1.589', '-0.200016773811']
BrScV 17 ['Sc0.125V0.25Br0.625', '-1.235', '-0.240742388488']
BrScW 16 ['Sc0.333333Br0.5W0.166667', '-0.996', '-0.216623543306']
BrScXe 35 ['Sc0.111111Br0.444444Xe0.444444', '-0.441', '-0.238665355287']
BrScY 24 ['Sc0.1Br0.5Y0.4', '-1.303', '-0.21682914279']
BrScYb 15 ['Sc0.285714Br0.571429Yb0.142857', '-1.724', '-0.209146155763']
BrScZn 39 ['Sc0.428571Zn0.285714Br0.285714', '-0.881', '-0.205866891929']
BrScZr 17 ['Sc0.125Br0.625Zr0.25', '-1.594', '-0.230117280366']
BrSeSm 30 ['Se0.5Br0.2Sm0.3', '-1.905', '-0.203169259033']
BrSeSn 1 ['Se0.25Br0.625Sn0.125', '-0.92', '-0.267482505446']
BrSeSr 4 ['Se0.166667Br0.5Sr0.333333', '-2.324', '-0.207006902123']
BrSeTa 22 ['Se0.444444Br0.333333Ta0.222222', '-0.939', '-0.201179737285']
BrSeTb 62 ['Se0.444444Br0.111111Tb0.444444', '-1.707', '-0.202964239394']
BrSeTc 34 ['Se0.444444Br0.333333Tc0.222222', '-0.47', '-0.20261999475']
BrSeTe 22 ['Se0.2Br0.5Te0.3', '-0.418', '-0.20110008378']
BrSeTh 23 ['Se0.375Br0.25Th0.375', '-1.923', '-0.200447547209']
BrSeTi 1 ['Ti0.142857Se0.142857Br0.714286', '-1.354', '-0.242951650948']
BrSeTm 73 ['Se0.571429Br0.142857Tm0.285714', '-1.558', '-0.206623901515']

BrSeU 11 ['Se0.333333Br0.333333U0.333333', '-1.528', '-0.203578953084']
BrSeV 28 ['V0.285714Se0.285714Br0.428571', '-1.031', '-0.21760610382']
BrSeW 2 ['Se0.125Br0.75W0.125', '-0.838', '-0.201524929438']
BrSeXe 27 ['Se0.3Br0.6Xe0.1', '-0.351', '-0.207807783775']
BrSeY 78 ['Se0.4Br0.1Y0.5', '-1.84', '-0.201261598758']
BrSeYb 16 ['Se0.125Br0.625Yb0.25', '-2.003', '-0.202848250366']
BrSeZr 30 ['Se0.166667Br0.5Zr0.333333', '-1.666', '-0.204017763747']
BrSiSm 24 ['Si0.1Br0.7Sm0.2', '-2.034', '-0.201612620682']
BrSiSn 6 ['Si0.111111Br0.777778Sn0.111111', '-1.241', '-0.209028185695']
BrSiSr 2 ['Si0.2Br0.7Sr0.1', '-1.589', '-0.205755503685']
BrSiTa 2 ['Si0.111111Br0.777778Ta0.111111', '-1.304', '-0.295848976543']
BrSiTb 18 ['Si0.2Br0.5Tb0.3', '-1.569', '-0.222757544703']
BrSiTc 1 ['Si0.1Br0.8Tc0.1', '-1.023', '-0.24266231781']
BrSiTe 6 ['Si0.222222Br0.666667Te0.111111', '-1.103', '-0.203507204225']
BrSiTh 13 ['Si0.142857Br0.571429Th0.285714', '-1.829', '-0.208688371479']
BrSiTi 3 ['Si0.2Ti0.1Br0.7', '-1.346', '-0.211225136306']
BrSiTl 7 ['Si0.222222Br0.666667Tl0.111111', '-1.183', '-0.203090899919']
BrSiTm 40 ['Si0.3Br0.6Tm0.1', '-1.179', '-0.206110039803']
BrSiU 1 ['Si0.125Br0.75U0.125', '-1.525', '-0.205109674753']
BrSiV 2 ['Si0.1V0.1Br0.8', '-1.169', '-0.322651868051']
BrSiY 45 ['Si0.125Br0.375Y0.5', '-1.269', '-0.208335002217']
BrSiYb 25 ['Si0.25Br0.375Yb0.375', '-1.765', '-0.24689086847']
BrSiZn 12 ['Si0.142857Zn0.285714Br0.571429', '-1.088', '-0.20201176148']
BrSiZr 2 ['Si0.142857Br0.714286Zr0.142857', '-1.525', '-0.210571576491']
BrSmSn 13 ['Br0.333333Sn0.222222Sm0.444444', '-1.583', '-0.215445814522']
BrSmTa 3 ['Br0.75Sm0.125Ta0.125', '-1.787', '-0.266535032972']
BrSmTb 3 ['Br0.75Sm0.125Tb0.125', '-1.994', '-0.364984746665']
BrSmTc 5 ['Br0.7Tc0.2Sm0.1', '-1.393', '-0.223412263724']
BrSmTe 48 ['Br0.222222Te0.333333Sm0.444444', '-1.754', '-0.202066466034']
BrSmTi 1 ['Ti0.1Br0.8Sm0.1', '-1.783', '-0.204265164429']
BrSmTl 18 ['Br0.5Sm0.1Tl0.4', '-1.465', '-0.20167108354']
BrSmTm 6 ['Br0.666667Sm0.111111Tm0.222222', '-1.813', '-0.222551336999']
BrSmV 3 ['V0.125Br0.75Sm0.125', '-1.768', '-0.323769769425']
BrSmW 3 ['Br0.777778Sm0.111111W0.111111', '-1.547', '-0.206171920296']
BrSmY 6 ['Br0.666667Y0.222222Sm0.111111', '-1.842', '-0.238325208666']
BrSmYb 3 ['Br0.666667Sm0.166667Yb0.166667', '-2.346', '-0.242639193666']
BrSmZn 12 ['Zn0.333333Br0.555556Sm0.111111', '-1.5', '-0.212413762824']
BrSmZr 2 ['Br0.777778Zr0.111111Sm0.111111', '-2.008', '-0.342462458111']
BrSnSr 1 ['Br0.625Sr0.25Sn0.125', '-2.188', '-0.226516092591']
BrSnTa 3 ['Br0.75Sn0.125Ta0.125', '-1.288', '-0.271097416603']
BrSnTb 26 ['Br0.444444Sn0.222222Tb0.333333', '-1.32', '-0.201750399739']
BrSnTc 6 ['Br0.777778Tc0.111111Sn0.111111', '-1.013', '-0.218638598193']
BrSnTe 8 ['Br0.3Sn0.2Te0.5', '-0.696', '-0.206994833614']
BrSnTh 1 ['Br0.8Sn0.1Th0.1', '-1.713', '-0.243774878634']

BrSnTi 2 ['Ti0.142857Br0.714286Sn0.142857', '-1.466', '-0.201830340683']
BrSnTm 33 ['Br0.7Sn0.2Tm0.1', '-1.398', '-0.23956504994']
BrSnU 5 ['Br0.8Sn0.1U0.1', '-1.498', '-0.206632932884']
BrSnV 3 ['V0.111111Br0.777778Sn0.111111', '-1.077', '-0.209293654016']
BrSnY 40 ['Br0.444444Y0.222222Sn0.333333', '-1.194', '-0.211152787794']
BrSnYb 11 ['Br0.5Sn0.125Yb0.375', '-2.122', '-0.201422792936']
BrSnZn 7 ['Zn0.2Br0.6Sn0.2', '-1.169', '-0.244933579118']
BrSnZr 4 ['Br0.714286Zr0.142857Sn0.142857', '-1.607', '-0.243961840511']
BrSrTa 3 ['Br0.75Sr0.125Ta0.125', '-1.58', '-0.234668571087']
BrSrTb 5 ['Br0.7Sr0.2Tb0.1', '-2.105', '-0.23326972834']
BrSrTe 21 ['Br0.666667Sr0.111111Te0.222222', '-1.083', '-0.205684601509']
BrSrTm 4 ['Br0.75Sr0.125Tm0.125', '-1.841', '-0.266861822865']
BrSrV 1 ['V0.125Br0.75Sr0.125', '-1.499', '-0.22990330754']
BrSrY 9 ['Br0.625Sr0.125Y0.25', '-1.853', '-0.223999397864']
BrSrYb 3 ['Br0.555556Sr0.111111Yb0.333333', '-2.202', '-0.254313449768']
BrSrZn 4 ['Zn0.3Br0.6Sr0.1', '-1.529', '-0.200183333552']
BrTaTb 6 ['Br0.666667Tb0.222222Ta0.111111', '-1.479', '-0.209638564483']
BrTaTc 3 ['Br0.75Tc0.125Ta0.125', '-1.032', '-0.271647189536']
BrTaTe 1 ['Br0.3Te0.5Ta0.2', '-0.826', '-0.247176977664']
BrTaTh 1 ['Br0.8Ta0.1Th0.1', '-1.735', '-0.372417891888']
BrTaTi 1 ['Ti0.1Br0.8Ta0.1', '-1.385', '-0.287356650758']
BrTaTl 6 ['Br0.666667Ta0.111111Tl0.222222', '-1.21', '-0.24747197788']
BrTaTm 9 ['Br0.571429Tm0.285714Ta0.142857', '-1.3', '-0.220611705763']
BrTaU 1 ['Br0.8Ta0.1U0.1', '-1.492', '-0.222071644647']
BrTaV 3 ['V0.125Br0.75Ta0.125', '-1.052', '-0.209134127337']
BrTaXe 6 ['Br0.666667Xe0.222222Ta0.111111', '-0.673', '-0.264510840393']
BrTaY 16 ['Br0.5Y0.4Ta0.1', '-1.269', '-0.207370218612']
BrTaYb 3 ['Br0.75Yb0.125Ta0.125', '-1.655', '-0.325205532338']
BrTaZn 7 ['Zn0.142857Br0.714286Ta0.142857', '-1.203', '-0.234805532673']
BrTaZr 2 ['Br0.777778Zr0.111111Ta0.111111', '-1.339', '-0.208008554033']
BrTbTc 15 ['Br0.555556Tc0.222222Tb0.222222', '-1.237', '-0.245516586955']
BrTbTe 61 ['Br0.1Te0.5Tb0.4', '-1.243', '-0.201201474101']
BrTbTh 3 ['Br0.75Tb0.125Th0.125', '-2.023', '-0.28310202341']
BrTbTi 5 ['Ti0.142857Br0.714286Tb0.142857', '-1.74', '-0.290567655353']
BrTbTl 28 ['Br0.428571Tb0.285714Tl0.285714', '-1.21', '-0.203109000579']
BrTbTm 10 ['Br0.6Tb0.1Tm0.3', '-1.441', '-0.210306939384']
BrTbU 3 ['Br0.75Tb0.125U0.125', '-1.793', '-0.284838959983']
BrTbV 8 ['V0.166667Br0.666667Tb0.166667', '-1.392', '-0.219285047849']
BrTbW 7 ['Br0.7Tb0.1W0.2', '-1.11', '-0.21651445113']
BrTbXe 15 ['Br0.6Xe0.1Tb0.3', '-1.394', '-0.20307044958']
BrTbY 9 ['Br0.666667Y0.111111Tb0.222222', '-1.597', '-0.229732636799']
BrTbYb 7 ['Br0.7Tb0.1Yb0.2', '-2.067', '-0.220128866342']
BrTbZn 19 ['Zn0.222222Br0.555556Tb0.222222', '-1.354', '-0.20790459119']
BrTbZr 9 ['Br0.6Zr0.2Tb0.2', '-1.579', '-0.200619422818']

BrTcTe 8 ['Br0.666667Tc0.111111Te0.222222', '-0.582', '-0.224665595686']
BrTcTh 4 ['Br0.75Tc0.125Th0.125', '-1.658', '-0.213787665481']
BrTcTl 2 ['Br0.75Tc0.125Tl0.125', '-0.845', '-0.222045910209']
BrTcTm 22 ['Br0.5Tc0.1Tm0.4', '-1.229', '-0.236555605791']
BrTcU 3 ['Br0.75Tc0.125U0.125', '-1.469', '-0.240872358399']
BrTcV 3 ['V0.125Br0.75Tc0.125', '-0.988', '-0.303881925989']
BrTcXe 9 ['Br0.777778Tc0.111111Xe0.111111', '-0.487', '-0.21961999475']
BrTcY 30 ['Br0.333333Y0.444444Tc0.222222', '-1.014', '-0.202810899471']
BrTcYb 4 ['Br0.666667Tc0.111111Yb0.222222', '-1.926', '-0.20064592876']
BrTcZr 3 ['Br0.75Zr0.125Tc0.125', '-1.416', '-0.302382421939']
BrTeTh 40 ['Br0.142857Te0.571429Th0.285714', '-1.391', '-0.200737249122']
BrTeTi 2 ['Ti0.111111Br0.777778Te0.111111', '-1.063', '-0.206908238695']
BrTeTl 7 ['Br0.375Te0.375Tl0.25', '-0.825', '-0.201643337427']
BrTeTm 53 ['Br0.5Te0.4Tm0.1', '-0.916', '-0.20339643946']
BrTeU 7 ['Br0.4Te0.4U0.2', '-1.208', '-0.212572915535']
BrTeV 14 ['V0.222222Br0.555556Te0.222222', '-0.975', '-0.203595702082']
BrTeXe 23 ['Br0.555556Te0.333333Xe0.111111', '-0.335', '-0.200068401403']
BrTeY 73 ['Br0.4Y0.5Te0.1', '-1.375', '-0.209087939198']
BrTeYb 47 ['Br0.4Te0.4Yb0.2', '-1.597', '-0.204609339034']
BrTeZn 7 ['Zn0.222222Br0.666667Te0.111111', '-0.949', '-0.214920837174']
BrTeZr 14 ['Br0.3Zr0.2Te0.5', '-1.168', '-0.215395108109']
BrThTl 3 ['Br0.7Tl0.1Th0.2', '-1.974', '-0.216634886311']
BrThTm 5 ['Br0.714286Tm0.142857Th0.142857', '-1.926', '-0.211959959704']
BrThV 2 ['V0.111111Br0.777778Th0.111111', '-1.675', '-0.228784090057']
BrThW 2 ['Br0.8W0.1Th0.1', '-1.578', '-0.225044593777']
BrThY 6 ['Br0.666667Y0.222222Th0.111111', '-1.843', '-0.235248107834']
BrThZn 2 ['Zn0.111111Br0.777778Th0.111111', '-1.684', '-0.233958057837']
BrThZr 1 ['Br0.8Zr0.1Th0.1', '-1.85', '-0.204806077811']
BrTiTm 14 ['Ti0.2Br0.6Tm0.2', '-1.404', '-0.219502195711']
BrTiU 1 ['Ti0.142857Br0.714286U0.142857', '-1.639', '-0.224312081248']
BrTiV 1 ['Ti0.1V0.1Br0.8', '-1.238', '-0.201344439921']
BrTiY 13 ['Ti0.2Br0.6Y0.2', '-1.482', '-0.255239684711']
BrTiZn 5 ['Ti0.1Zn0.3Br0.6', '-1.247', '-0.200812067173']
BrTiZr 1 ['Ti0.1Br0.8Zr0.1', '-1.642', '-0.261744836681']
BrTlTm 36 ['Br0.5Tm0.125Tl0.375', '-1.238', '-0.204522570665']
BrTlU 1 ['Br0.714286Tl0.142857U0.142857', '-1.63', '-0.21090997899']
BrTlV 10 ['V0.125Br0.625Tl0.25', '-1.182', '-0.217409606457']
BrTlY 47 ['Br0.3Y0.5Tl0.2', '-1.034', '-0.205905032274']
BrTlYb 8 ['Br0.5Yb0.375Tl0.125', '-2.068', '-0.202964015667']
BrTlZn 5 ['Zn0.222222Br0.555556Tl0.222222', '-1.187', '-0.209509986992']
BrTlZr 10 ['Br0.666667Zr0.111111Tl0.222222', '-1.451', '-0.211808979917']
BrTmU 6 ['Br0.666667Tm0.222222U0.111111', '-1.558', '-0.229292501168']
BrTmV 14 ['V0.142857Br0.571429Tm0.285714', '-1.325', '-0.245611705763']
BrTmW 11 ['Br0.666667Tm0.166667W0.166667', '-1.312', '-0.221818073738']

BrTmXe 15 ['Br0.8Xe0.1Tm0.1', '-0.768', '-0.217314357776']
BrTmY 19 ['Br0.555556Y0.111111Tm0.333333', '-1.361', '-0.234804833657']
BrTmYb 12 ['Br0.6Tm0.3Yb0.1', '-1.705', '-0.22124643905']
BrTmZn 35 ['Zn0.1Br0.8Tm0.1', '-1.093', '-0.232218474294']
BrTmZr 13 ['Br0.571429Zr0.285714Tm0.142857', '-1.482', '-0.222416805763']
BrUY 8 ['Br0.6Y0.3U0.1', '-1.485', '-0.20402893755']
BrUZr 2 ['Br0.75Zr0.125U0.125', '-1.805', '-0.23853642194']
BrVXe 7 ['V0.2Br0.7Xe0.1', '-0.823', '-0.209695091032']
BrVY 14 ['V0.142857Br0.571429Y0.285714', '-1.316', '-0.232161010046']
BrVYb 3 ['V0.125Br0.75Yb0.125', '-1.471', '-0.217440268791']
BrVZn 6 ['V0.111111Zn0.222222Br0.666667', '-1.237', '-0.20717308728']
BrVZr 3 ['V0.125Br0.75Zr0.125', '-1.438', '-0.24186935974']
BrWY 14 ['Br0.7Y0.1W0.2', '-1.143', '-0.218207039143']
BrXeY 18 ['Br0.5Y0.4Xe0.1', '-1.255', '-0.208389579291']
BrXeZr 5 ['Br0.75Zr0.125Xe0.125', '-1.033', '-0.220184927845']
BrYYb 9 ['Br0.6Y0.3Yb0.1', '-1.738', '-0.22121166805']
BrYZn 38 ['Zn0.4Br0.5Y0.1', '-1.118', '-0.227862606366']
BrYZr 18 ['Br0.7Y0.1Zr0.2', '-1.728', '-0.20147846531']
BrYbZn 28 ['Zn0.1Br0.7Yb0.2', '-1.904', '-0.201513455552']
BrYbZr 4 ['Br0.625Zr0.125Yb0.25', '-2.231', '-0.20119145099']
BrZnZr 14 ['Zn0.2Br0.7Zr0.1', '-1.476', '-0.205556175312']
CCaCo 12 ['C0.5Ca0.25Co0.25', '-0.218', '-0.218']
CCaCr 2 ['C0.571429Ca0.285714Cr0.142857', '-0.226', '-0.201203198214']
CCaFe 1 ['C0.571429Ca0.285714Fe0.142857', '-0.222', '-0.222']
CCaGe 6 ['C0.4Ca0.3Ge0.3', '-0.623', '-0.2193771815']
CCaH 27 ['H0.6C0.1Ca0.3', '-0.721', '-0.20713623044']
CCaI 9 ['C0.222222Ca0.444444I0.333333', '-1.091', '-0.212615127525']
CCaIr 2 ['C0.571429Ca0.285714Ir0.142857', '-0.368', '-0.232892735714']
CCaLi 5 ['Li0.333333C0.555556Ca0.111111', '-0.226', '-0.218230222778']
CCaMn 2 ['C0.571429Ca0.285714Mn0.142857', '-0.231', '-0.213646158061']
CCaN 13 ['C0.5N0.125Ca0.375', '-0.531', '-0.215072654332']
CCaNb 1 ['C0.6Ca0.3Nb0.1', '-0.305', '-0.203350234042']
CCaNd 1 ['C0.6Ca0.3Nd0.1', '-0.269', '-0.225771710375']
CCaNp 9 ['C0.555556Ca0.111111Np0.333333', '-0.261', '-0.261']
CCaO 1 ['C0.1O0.6Ca0.3', '-2.809', '-0.25305680772']
CCaOs 5 ['C0.6Ca0.3Os0.1', '-0.209', '-0.209']
CCaP 24 ['C0.2P0.5Ca0.3', '-1.051', '-0.209069836804']
CCaRe 22 ['C0.375Ca0.125Re0.5', '-0.23', '-0.207660676875']
CCaRh 15 ['C0.428571Ca0.285714Rh0.285714', '-0.392', '-0.210921561428']
CCaRu 3 ['C0.5Ca0.3Ru0.2', '-0.217', '-0.217']
CCaSc 4 ['C0.625Ca0.25Sc0.125', '-0.342', '-0.234597066172']
CCaTc 5 ['C0.571429Ca0.285714Tc0.142857', '-0.204', '-0.204']
CCaW 15 ['C0.444444Ca0.222222W0.333333', '-0.286', '-0.2022275675']
CCaXe 16 ['C0.555556Ca0.333333Xe0.111111', '-0.205', '-0.205']

CCeCl 4 ['C0.142857Cl0.714286Ce0.142857', '-1.695', '-0.217021220091']
CCeCo 1 ['C0.5Co0.166667Ce0.333333', '-0.399', '-0.221013317083']
CCeF 1 ['C0.3F0.3Ce0.4', '-2.015', '-0.208786819344']
CCeFe 1 ['C0.5Fe0.166667Ce0.333333', '-0.315', '-0.2192790975']
CCeH 20 ['H0.6C0.3Ce0.1', '-0.396', '-0.201192687595']
CCeI 21 ['C0.2I0.4Ce0.4', '-1.014', '-0.204381414693']
CCeLi 3 ['Li0.375C0.5Ce0.125', '-0.24', '-0.204104661563']
CCeN 16 ['C0.285714N0.428571Ce0.285714', '-0.956', '-0.206637133474']
CCeNa 5 ['C0.571429Na0.285714Ce0.142857', '-0.264', '-0.222976756071']
CCeNi 10 ['C0.5Ni0.2Ce0.3', '-0.395', '-0.207659334875']
CCeNp 1 ['C0.625Ce0.25Np0.125', '-0.285', '-0.213209323125']
CCeO 2 ['C0.111111O0.333333Ce0.555556', '-2.209', '-0.231962294011']
CCeOs 2 ['C0.6Ce0.3Os0.1', '-0.295', '-0.20885118775']
CCePt 16 ['C0.555556Ce0.333333Pt0.111111', '-0.506', '-0.202425130833']
CCeRe 16 ['C0.625Ce0.125Re0.25', '-0.265', '-0.200431583047']
CCeRu 18 ['C0.428571Ru0.142857Ce0.428571', '-0.31', '-0.20158866352']
CCeSe 9 ['C0.3Se0.3Ce0.4', '-1.342', '-0.20352634']
CCeTc 3 ['C0.444444Tc0.222222Ce0.333333', '-0.291', '-0.205914753333']
CCeW 3 ['C0.571429Ce0.285714W0.142857', '-0.339', '-0.221051041071']
CCeXe 6 ['C0.444444Xe0.333333Ce0.222222', '-0.269', '-0.205186065']
CClCs 8 ['C0.111111Cl0.777778Cs0.111111', '-0.68', '-0.201362462709']
CClDy 10 ['C0.3Cl0.4Dy0.3', '-1.658', '-0.200168755836']
CClEr 43 ['C0.3Cl0.6Er0.1', '-0.489', '-0.224119921438']
CClEu 11 ['C0.166667Cl0.5Eu0.333333', '-2.368', '-0.221908899482']
CClGd 3 ['C0.125Cl0.75Gd0.125', '-1.543', '-0.278380160705']
CClHo 45 ['C0.1Cl0.4Ho0.5', '-1.353', '-0.203341553416']
CClIn 2 ['C0.1Cl0.8In0.1', '-0.575', '-0.236857221289']
CClK 5 ['C0.1Cl0.6K0.3', '-1.486', '-0.201341196814']
CClKr 4 ['C0.1Cl0.6Kr0.3', '-0.213', '-0.213']
CClLa 4 ['C0.125Cl0.75La0.125', '-1.55', '-0.207827871645']
CClLi 6 ['Li0.285714C0.142857Cl0.571429', '-1.345', '-0.202231669823']
CClLu 25 ['C0.3Cl0.5Lu0.2', '-1.067', '-0.203566309125']
CClMg 4 ['C0.125Mg0.125Cl0.75', '-0.994', '-0.2056002262']
CClNa 9 ['C0.125Na0.25Cl0.625', '-1.229', '-0.20394593088']
CClNd 14 ['C0.2Cl0.4Nd0.4', '-1.639', '-0.212910562255']
CClNp 1 ['C0.1Cl0.8Np0.1', '-1.185', '-0.248853544375']
CClPb 1 ['C0.1Cl0.5Pb0.4', '-1.404', '-0.358910084693']
CClPm 30 ['C0.111111Cl0.444444Pm0.444444', '-1.573', '-0.202128911947']
CClPr 6 ['C0.333333Cl0.333333Pr0.333333', '-1.522', '-0.210028536209']
CClPu 12 ['C0.333333Cl0.333333Pu0.333333', '-1.185', '-0.201225823684']
CClRb 13 ['C0.166667Cl0.5Rb0.333333', '-1.628', '-0.206726794793']
CClRh 11 ['C0.111111Cl0.555556Rh0.333333', '-0.202', '-0.202']
CClSc 4 ['C0.125Cl0.75Sc0.125', '-1.407', '-0.21325464477']
CClSm 22 ['C0.222222Cl0.333333Sm0.444444', '-1.321', '-0.202092821832']

CClSr 3 ['C0.142857Cl0.714286Sr0.142857', '-1.386', '-0.214206998396']
CClTb 4 ['C0.142857Cl0.714286Tb0.142857', '-1.579', '-0.22147254764']
CClTh 2 ['C0.111111Cl0.777778Th0.111111', '-1.571', '-0.210449505833']
CClTm 3 ['C0.1Cl0.8Tm0.1', '-1.224', '-0.216762544316']
CClXe 20 ['C0.25Cl0.5Xe0.25', '-0.207', '-0.207']
CClY 8 ['C0.111111Cl0.777778Y0.111111', '-1.338', '-0.20320660424']
CCoEu 11 ['C0.3Co0.5Eu0.2', '-0.254', '-0.20011725275']
CCoGd 1 ['C0.2Co0.6Gd0.2', '-0.412', '-0.227927631']
CCoMg 3 ['C0.333333Mg0.333333Co0.333333', '-0.259', '-0.217512303704']
CCoPm 13 ['C0.555556Co0.222222Pm0.222222', '-0.224', '-0.212095099444']
CCoSc 1 ['C0.3Sc0.6Co0.1', '-0.669', '-0.23080537875']
CCoSr 1 ['C0.571429Co0.142857Sr0.285714', '-0.207', '-0.207']
CCrGd 2 ['C0.444444Cr0.444444Gd0.111111', '-0.37', '-0.20474617875']
CCrLa 1 ['C0.5Cr0.2La0.3', '-0.373', '-0.224891211388']
CCrLi 1 ['Li0.4C0.5Cr0.1', '-0.229', '-0.21164223875']
CCrNp 10 ['C0.5Cr0.375Np0.125', '-0.269', '-0.203908395313']
CCrPm 11 ['C0.5Cr0.333333Pm0.166667', '-0.258', '-0.200140795833']
CCrRe 3 ['C0.444444Cr0.111111Re0.444444', '-0.243', '-0.2038564225']
CCrSc 1 ['C0.375Sc0.375Cr0.25', '-0.574', '-0.2043389725']
CCrTb 1 ['C0.444444Cr0.444444Tb0.111111', '-0.323', '-0.224942826843']
CCrTh 1 ['C0.5Cr0.166667Th0.333333', '-0.439', '-0.204780475714']
CCrW 19 ['C0.4Cr0.4W0.2', '-0.317', '-0.2033080388']
CCrYb 4 ['C0.5Cr0.3Yb0.2', '-0.321', '-0.2009630125']
CCsF 4 ['C0.1F0.7Cs0.2', '-2.44', '-0.26887723836']
CCsH 3 ['H0.555556C0.111111Cs0.333333', '-0.313', '-0.206324600784']
CCsN 5 ['C0.125N0.625Cs0.25', '-0.464', '-0.204919804525']
CCsO 2 ['C0.111111O0.666667Cs0.222222', '-1.532', '-0.239581019747']
CCsS 7 ['C0.222222S0.444444Cs0.333333', '-0.998', '-0.20189845766']
CCsSe 9 ['C0.2Se0.3Cs0.5', '-1.004', '-0.22112322475']
CCuDy 4 ['C0.25Cu0.25Dy0.5', '-0.487', '-0.223090194011']
CCuHf 1 ['C0.375Cu0.125Hf0.5', '-0.958', '-0.217066667813']
CCuPr 1 ['C0.5Cu0.166667Pr0.333333', '-0.418', '-0.276910495625']
CCuSc 2 ['C0.285714Sc0.571429Cu0.142857', '-0.618', '-0.201154786429']
CDyF 4 ['C0.3F0.3Dy0.4', '-2.017', '-0.201219007408']
CDyGe 2 ['C0.333333Ge0.166667Dy0.5', '-0.705', '-0.2015614725']
CDyH 14 ['H0.6C0.3Dy0.1', '-0.451', '-0.208845072512']
CDyI 40 ['C0.2I0.3Dy0.5', '-0.824', '-0.207367380126']
CDyIr 4 ['C0.25Dy0.5Ir0.25', '-0.79', '-0.217239874375']
CDyMo 1 ['C0.375Mo0.5Dy0.125', '-0.378', '-0.206767637604']
CDyN 22 ['C0.1N0.3Dy0.6', '-1.372', '-0.201722683311']
CDyNi 9 ['C0.4Ni0.2Dy0.4', '-0.543', '-0.203034020833']
CDyNp 3 ['C0.625Dy0.25Np0.125', '-0.375', '-0.207215191458']
CDyO 2 ['C0.333333O0.166667Dy0.5', '-1.49', '-0.220908571556']
CDyOs 24 ['C0.5Dy0.3Os0.2', '-0.402', '-0.20065822975']

CDyP 3 ['C0.25P0.25Dy0.5', '-1.213', '-0.205422289272']
CDyPb 8 ['C0.375Dy0.375Pb0.25', '-0.551', '-0.202672736523']
CDyPd 4 ['C0.285714Pd0.285714Dy0.428571', '-0.852', '-0.224164470357']
CDyPt 8 ['C0.333333Dy0.444444Pt0.222222', '-0.952', '-0.230623784816']
CDyRe 4 ['C0.571429Dy0.142857Re0.285714', '-0.369', '-0.212106483244']
CDyRh 13 ['C0.333333Rh0.111111Dy0.555556', '-0.645', '-0.234348941991']
CDyRu 26 ['C0.5Ru0.125Dy0.375', '-0.451', '-0.2011088']
CDyS 17 ['C0.333333S0.111111Dy0.555556', '-0.892', '-0.200413233767']
CDySe 7 ['C0.285714Se0.142857Dy0.571429', '-0.908', '-0.208223206459']
CDySi 1 ['C0.333333Si0.222222Dy0.444444', '-0.743', '-0.224195102593']
CDyTc 16 ['C0.555556Tc0.111111Dy0.333333', '-0.434', '-0.210286921944']
CDyXe 5 ['C0.4Xe0.3Dy0.3', '-0.39', '-0.200930387437']
CDyZn 1 ['C0.375Zn0.25Dy0.375', '-0.518', '-0.2029823425']
CErH 8 ['H0.333333C0.333333Er0.333333', '-0.692', '-0.213751539633']
CErI 23 ['C0.125I0.5Er0.375', '-1.087', '-0.221282051678']
CErN 17 ['C0.111111N0.444444Er0.444444', '-1.879', '-0.202231668738']
CErNp 6 ['C0.625Er0.25Np0.125', '-0.402', '-0.205240455542']
CErO 2 ['C0.1O0.6Er0.3', '-3.517', '-0.247959052221']
CErOs 1 ['C0.5Er0.2Os0.3', '-0.361', '-0.203592364433']
CErP 3 ['C0.1P0.5Er0.4', '-1.612', '-0.221582780416']
CErS 1 ['C0.2S0.4Er0.4', '-1.883', '-0.204888498392']
CErTc 5 ['C0.375Tc0.25Er0.375', '-0.438', '-0.204995276299']
CEuH 6 ['H0.571429C0.142857Eu0.285714', '-0.757', '-0.206333910181']
CEuI 6 ['C0.222222I0.333333Eu0.444444', '-1.175', '-0.200915674744']
CEuN 33 ['C0.166667N0.5Eu0.333333', '-0.586', '-0.200406228219']
CEuNp 4 ['C0.625Eu0.125Np0.25', '-0.325', '-0.212744276562']
CEuP 25 ['C0.2P0.5Eu0.3', '-1.141', '-0.201685943322']
CEuRe 1 ['C0.6Eu0.2Re0.2', '-0.322', '-0.2142345055']
CEuRu 1 ['C0.375Ru0.25Eu0.375', '-0.284', '-0.216646565937']
CEuS 2 ['C0.285714S0.285714Eu0.428571', '-1.671', '-0.208063270994']
CEuSe 11 ['C0.25Se0.25Eu0.5', '-1.371', '-0.222655513125']
CEuW 4 ['C0.555556Eu0.111111W0.333333', '-0.343', '-0.219314421389']
CEuXe 1 ['C0.5Xe0.3Eu0.2', '-0.298', '-0.20819542125']
CFH 1 ['H0.555556C0.111111F0.333333', '-1.287', '-0.20410387456']
CFHg 1 ['C0.1F0.8Hg0.1', '-1.758', '-0.239437390277']
CFK 4 ['C0.1F0.7K0.2', '-2.403', '-0.215818528772']
CFLi 1 ['Li0.1C0.1F0.8', '-1.893', '-0.241050977686']
CFLu 6 ['C0.3F0.3Lu0.4', '-1.978', '-0.204638129296']
CFNa 2 ['C0.111111F0.777778Na0.111111', '-2.068', '-0.271585657336']
CFNi 1 ['C0.1F0.8Ni0.1', '-1.975', '-0.22687471636']
CFO 5 ['C0.1O0.2F0.7', '-1.223', '-0.2010263296']
CFPa 3 ['C0.125F0.75Pa0.125', '-3.115', '-0.358211970575']
CFPm 8 ['C0.222222F0.333333Pm0.444444', '-2.071', '-0.217994793347']
CFPu 1 ['C0.1F0.8Pu0.1', '-2.969', '-0.38586002436']

CFRb 4 ['C0.125F0.75Rb0.125', '-2.224', '-0.232187498358']
CFSc 6 ['C0.1F0.8Sc0.1', '-2.946', '-0.21609288536']
CFTi 1 ['C0.1F0.8Ti0.1', '-2.779', '-0.313675863484']
CFTm 40 ['C0.375F0.375Tm0.25', '-1.617', '-0.208061518215']
CFY 2 ['C0.1F0.8Y0.1', '-2.902', '-0.200780487692']
CFYb 16 ['C0.1F0.4Yb0.5', '-2.432', '-0.251773268278']
CFeLa 5 ['C0.3Fe0.3La0.4', '-0.313', '-0.22785911925']
CFeNd 11 ['C0.555556Fe0.111111Nd0.333333', '-0.37', '-0.22590570125']
CFeNp 1 ['C0.555556Fe0.111111Np0.333333', '-0.223', '-0.223']
CFePm 12 ['C0.444444Fe0.222222Pm0.333333', '-0.209', '-0.209']
CFePr 4 ['C0.428571Fe0.142857Pr0.428571', '-0.317', '-0.2116612875']
CFeTh 3 ['C0.444444Fe0.222222Th0.333333', '-0.416', '-0.202734092222']
CFeTi 8 ['C0.166667Ti0.333333Fe0.5', '-0.619', '-0.200897424155']
CGaHf 2 ['C0.333333Ga0.333333Hf0.333333', '-0.841', '-0.209226419167']
CGaMo 1 ['C0.375Ga0.125Mo0.5', '-0.332', '-0.229858684063']
CGaNp 12 ['C0.375Ga0.25Np0.375', '-0.277', '-0.213050958021']
CGaPa 21 ['C0.3Ga0.4Pa0.3', '-0.478', '-0.20508452275']
CGaRe 1 ['C0.4Ga0.1Re0.5', '-0.233', '-0.210660676875']
CGaW 7 ['C0.5Ga0.166667W0.333333', '-0.284', '-0.2002275675']
CGdH 22 ['H0.571429C0.285714Gd0.142857', '-0.541', '-0.200432384183']
CGdI 23 ['C0.25I0.375Gd0.375', '-1.181', '-0.210111699713']
CGdN 11 ['C0.285714N0.285714Gd0.428571', '-1.284', '-0.21272332026']
CGdNa 2 ['C0.571429Na0.142857Gd0.285714', '-0.382', '-0.201983802143']
CGdNi 3 ['C0.25Ni0.25Gd0.5', '-0.53', '-0.208900940833']
CGdO 3 ['C0.1O0.6Gd0.3', '-3.408', '-0.216971242721']
CGdP 1 ['C0.1P0.5Gd0.4', '-1.642', '-0.230466791645']
CGdPt 5 ['C0.111111Gd0.555556Pt0.333333', '-1.116', '-0.201118010463']
CGdRe 4 ['C0.6Gd0.2Re0.2', '-0.396', '-0.203837834375']
CGdRu 11 ['C0.111111Ru0.333333Gd0.555556', '-0.415', '-0.205360968056']
CGdS 2 ['C0.2S0.3Gd0.5', '-1.629', '-0.244675287544']
CGdSe 3 ['C0.25Se0.25Gd0.5', '-1.25', '-0.247246664375']
CGdSi 1 ['C0.444444Si0.222222Gd0.333333', '-0.66', '-0.223322785278']
CGdTe 1 ['C0.1Te0.4Gd0.5', '-1.341', '-0.228145200164']
CGeHf 19 ['C0.222222Ge0.333333Hf0.444444', '-0.927', '-0.204307904861']
CGeLu 1 ['C0.333333Ge0.166667Lu0.5', '-0.796', '-0.271645240317']
CGeNb 2 ['C0.4Ge0.4Nb0.2', '-0.413', '-0.207644653333']
CGeNp 20 ['C0.333333Ge0.444444Np0.222222', '-0.36', '-0.230660943148']
CGePa 18 ['C0.5Ge0.3Pa0.2', '-0.384', '-0.2020563485']
CGePm 10 ['C0.25Ge0.25Pm0.5', '-0.509', '-0.202666545625']
CGePu 5 ['C0.428571Ge0.285714Pu0.285714', '-0.488', '-0.234387658571']
CGeSc 5 ['C0.1Sc0.5Ge0.4', '-1.0', '-0.247363557']
CGeTa 3 ['C0.4Ge0.4Ta0.2', '-0.434', '-0.2006926205']
CGeU 2 ['C0.375Ge0.375U0.25', '-0.418', '-0.21475211375']
CGeV 1 ['C0.4V0.4Ge0.2', '-0.588', '-0.2026849375']

CGeW 30 ['C0.444444Ge0.111111W0.444444', '-0.348', '-0.236303423333']
CGeZr 2 ['C0.333333Ge0.222222Zr0.444444', '-0.943', '-0.202172335833']
CHHf 2 ['H0.3C0.3Hf0.4', '-0.956', '-0.241549703313']
CHHo 4 ['H0.6C0.3Ho0.1', '-0.447', '-0.204132054845']
CHK 6 ['H0.555556C0.111111K0.333333', '-0.332', '-0.200085380939']
CHLa 19 ['H0.625C0.125La0.25', '-0.746', '-0.212112480509']
CHLu 8 ['H0.555556C0.222222Lu0.222222', '-0.659', '-0.204668977918']
CHMg 9 ['H0.5C0.3Mg0.2', '-0.312', '-0.201301292293']
CHNd 23 ['H0.333333C0.444444Nd0.222222', '-0.54', '-0.20182598698']
CHNp 40 ['H0.2C0.6Np0.2', '-0.256', '-0.200000802313']
CHPa 10 ['H0.571429C0.285714Pa0.142857', '-0.408', '-0.20087743835']
CHPm 48 ['H0.222222C0.555556Pm0.222222', '-0.315', '-0.204844793404']
CHPr 39 ['H0.5C0.375Pr0.125', '-0.45', '-0.200174684493']
CHPu 23 ['H0.222222C0.444444Pu0.333333', '-0.333', '-0.202776932293']
CHRb 12 ['H0.4C0.1Rb0.5', '-0.332', '-0.200560214189']
CHSm 28 ['H0.625C0.25Sm0.125', '-0.492', '-0.205952576785']
CHSr 9 ['H0.555556C0.333333Sr0.111111', '-0.381', '-0.20092351007']
CHTa 4 ['H0.625C0.25Ta0.125', '-0.35', '-0.204182887812']
CHTb 3 ['H0.4C0.3Tb0.3', '-0.486', '-0.200233686789']
CHTh 4 ['H0.3C0.4Th0.3', '-0.543', '-0.200069833631']
CHTl 1 ['H0.625C0.25Tl0.125', '-0.222', '-0.222']
CHTm 8 ['H0.4C0.3Tm0.3', '-0.689', '-0.204444097126']
CHU 25 ['H0.111111C0.555556U0.333333', '-0.341', '-0.201679515822']
CHYb 15 ['H0.428571C0.285714Yb0.285714', '-0.62', '-0.20016684451']
CHZr 6 ['H0.333333C0.444444Zr0.222222', '-0.572', '-0.202738777189']
CHfHg 2 ['C0.375Hf0.5Hg0.125', '-0.973', '-0.243741200002']
CHfIn 6 ['C0.333333In0.111111Hf0.555556', '-0.869', '-0.202368268611']
CHfKr 9 ['C0.142857Kr0.428571Hf0.428571', '-0.477', '-0.206239893929']
CHfN 11 ['C0.333333N0.333333Hf0.333333', '-1.44', '-0.203614016553']
CHfOs 15 ['C0.1Hf0.6Os0.3', '-0.821', '-0.20619477025']
CHfP 13 ['C0.333333P0.444444Hf0.222222', '-0.838', '-0.204015464676']
CHfPb 1 ['C0.333333Hf0.5Pb0.166667', '-0.892', '-0.260226419167']
CHfPt 2 ['C0.375Hf0.5Pt0.125', '-1.224', '-0.225698625313']
CHfRe 23 ['C0.4Hf0.4Re0.2', '-0.959', '-0.200871703']
CHfRu 11 ['C0.428571Ru0.142857Hf0.428571', '-1.016', '-0.203719681786']
CHfSb 19 ['C0.444444Sb0.333333Hf0.222222', '-0.625', '-0.203817612778']
CHfSe 18 ['C0.3Se0.3Hf0.4', '-1.291', '-0.211310206125']
CHfSi 6 ['C0.25Si0.375Hf0.375', '-0.902', '-0.202795320208']
CHfSn 7 ['C0.428571Sn0.142857Hf0.428571', '-1.02', '-0.207719681786']
CHfTc 25 ['C0.1Tc0.3Hf0.6', '-0.681', '-0.20185317275']
CHfTe 4 ['C0.333333Te0.166667Hf0.5', '-1.063', '-0.200938375278']
CHfTl 2 ['C0.333333Hf0.444444Tl0.222222', '-0.925', '-0.222778613611']
CHgLu 3 ['C0.285714Lu0.571429Hg0.142857', '-0.501', '-0.228652661944']
CHgNp 2 ['C0.555556Hg0.111111Np0.333333', '-0.213', '-0.213']

CHgPa 1 ['C0.5Hg0.1Pa0.4', '-0.576', '-0.212112697']
CHoI 27 ['C0.4I0.3Ho0.3', '-0.755', '-0.211873607834']
CHoN 14 ['C0.2N0.3Ho0.5', '-1.462', '-0.219634961396']
CHoNa 1 ['C0.555556Na0.222222Ho0.222222', '-0.403', '-0.221696843843']
CHoNi 7 ['C0.125Ni0.375Ho0.5', '-0.646', '-0.20585337']
CHoNp 3 ['C0.555556Ho0.222222Np0.222222', '-0.391', '-0.209696843843']
CHoO 1 ['C0.1O0.6Ho0.3', '-3.453', '-0.200840074286']
CHoOs 6 ['C0.222222Ho0.444444Os0.333333', '-0.408', '-0.203538772407']
CHoP 12 ['C0.125P0.375Ho0.5', '-1.613', '-0.224214171638']
CHoRe 1 ['C0.5Ho0.1Re0.4', '-0.323', '-0.200513328479']
CHoRu 6 ['C0.166667Ru0.333333Ho0.5', '-0.571', '-0.229943340555']
CHoS 10 ['C0.111111S0.444444Ho0.444444', '-2.094', '-0.210668490071']
CHoSe 5 ['C0.2Se0.3Ho0.5', '-1.391', '-0.224568946998']
CHoTc 4 ['C0.444444Tc0.333333Ho0.222222', '-0.383', '-0.201696843843']
CILu 46 ['C0.4I0.2Lu0.4', '-0.604', '-0.201086050139']
CINd 15 ['C0.4I0.2Nd0.4', '-0.747', '-0.205610308013']
CIPa 53 ['C0.166667I0.333333Pa0.5', '-0.445', '-0.200927812941']
CIPm 51 ['C0.428571I0.285714Pm0.285714', '-0.715', '-0.203786572878']
CIPr 9 ['C0.4I0.2Pr0.4', '-0.732', '-0.202912946346']
CIPt 3 ['C0.166667I0.5Pt0.333333', '-0.407', '-0.207582538786']
CIRh 3 ['C0.1Rh0.3I0.6', '-0.216', '-0.216']
CISc 8 ['C0.285714Sc0.428571I0.285714', '-1.014', '-0.200968724322']
CISm 19 ['C0.142857I0.714286Sm0.142857', '-0.759', '-0.213483683602']
CISr 10 ['C0.2Sr0.4I0.4', '-1.315', '-0.219427780528']
CITb 50 ['C0.428571I0.142857Tb0.428571', '-0.335', '-0.200354066403']
CITm 8 ['C0.222222I0.444444Tm0.333333', '-1.117', '-0.20494520114']
CIXe 6 ['C0.1I0.6Xe0.3', '-0.205', '-0.205']
CIYb 1 ['C0.222222I0.444444Yb0.333333', '-1.454', '-0.230986387948']
CInMo 3 ['C0.333333Mo0.555556In0.111111', '-0.299', '-0.202395626944']
CInNp 17 ['C0.3In0.5Np0.2', '-0.204', '-0.201062120208']
CInPa 7 ['C0.4In0.2Pa0.4', '-0.569', '-0.205112697']
CInPu 1 ['C0.375In0.25Pu0.375', '-0.342', '-0.231046757188']
CIrLa 4 ['C0.285714La0.428571Ir0.285714', '-0.649', '-0.217894301857']
CIrLu 4 ['C0.166667Lu0.5Ir0.333333', '-0.992', '-0.216883047262']
CIrNd 1 ['C0.25Nd0.5Ir0.25', '-0.548', '-0.200965686875']
CIrNp 1 ['C0.571429Ir0.142857Np0.285714', '-0.262', '-0.202376504821']
CIrPm 5 ['C0.25Pm0.5Ir0.25', '-0.506', '-0.203653854375']
CIrPr 7 ['C0.166667Pr0.5Ir0.333333', '-0.576', '-0.201237498055']
CIrSc 17 ['C0.125Sc0.625Ir0.25', '-0.93', '-0.209700231146']
CIrSm 8 ['C0.375Sm0.375Ir0.25', '-0.605', '-0.2181104675']
CIrSr 3 ['C0.5Sr0.3Ir0.2', '-0.281', '-0.201408652']
CIrTi 1 ['C0.222222Ti0.555556Ir0.222222', '-1.047', '-0.245426047341']
CIrY 5 ['C0.166667Y0.5Ir0.333333', '-0.85', '-0.20901630825']
CKNp 2 ['C0.571429K0.142857Np0.285714', '-0.265', '-0.2421422275']

CKPm 1 ['C0.6K0.2Pm0.2', '-0.245', '-0.220999338875']
CKrLa 1 ['C0.4Kr0.3La0.3', '-0.327', '-0.213478825667']
CKrLu 1 ['C0.333333Kr0.333333Lu0.333333', '-0.384', '-0.211924327262']
CKrNp 2 ['C0.5Kr0.2Np0.3', '-0.216', '-0.216']
CKrPa 2 ['C0.5Kr0.166667Pa0.333333', '-0.511', '-0.207760580833']
CKrPm 12 ['C0.4Kr0.2Pm0.4', '-0.216', '-0.216']
CKrPr 6 ['C0.5Kr0.166667Pr0.333333', '-0.325', '-0.202104835417']
CKrSc 7 ['C0.3Sc0.5Kr0.2', '-0.567', '-0.20261577425']
CKrTh 13 ['C0.222222Kr0.333333Th0.444444', '-0.326', '-0.201454545']
CKrTm 3 ['C0.428571Kr0.285714Tm0.285714', '-0.401', '-0.202020457143']
CLaLi 5 ['Li0.285714C0.571429La0.142857', '-0.272', '-0.201775730714']
CLaN 40 ['C0.5N0.2La0.3', '-0.83', '-0.210265232432']
CLaNa 3 ['C0.555556Na0.222222La0.222222', '-0.319', '-0.209762247778']
CLaNi 8 ['C0.333333Ni0.222222La0.444444', '-0.449', '-0.204609833611']
CLaNp 1 ['C0.625La0.25Np0.125', '-0.35', '-0.22710752875']
CLaO 3 ['C0.25O0.25La0.5', '-1.83', '-0.212158375092']
CLaPd 6 ['C0.222222Pd0.333333La0.444444', '-0.813', '-0.204418042222']
CLaPt 5 ['C0.222222La0.555556Pt0.222222', '-0.805', '-0.214205746481']
CLaRe 5 ['C0.6La0.3Re0.1', '-0.351', '-0.2035290345']
CLaRu 18 ['C0.2Ru0.4La0.4', '-0.355', '-0.2062802785']
CLaS 8 ['C0.111111S0.333333La0.555556', '-1.783', '-0.211537512456']
CLaSe 20 ['C0.111111Se0.444444La0.444444', '-1.979', '-0.201314542222']
CLaSi 1 ['C0.444444Si0.222222La0.333333', '-0.613', '-0.224281748333']
CLaTc 7 ['C0.5Tc0.166667La0.333333', '-0.349', '-0.207098532083']
CLaXe 7 ['C0.444444Xe0.333333La0.222222', '-0.315', '-0.205762247778']
CLaZn 2 ['C0.2Zn0.4La0.4', '-0.493', '-0.204192094833']
CLiLu 1 ['Li0.333333C0.555556Lu0.111111', '-0.302', '-0.201622524236']
CLiMo 6 ['Li0.166667C0.333333Mo0.5', '-0.297', '-0.20987230125']
CLiNd 1 ['Li0.333333C0.555556Nd0.111111', '-0.299', '-0.250968567083']
CLiPu 1 ['Li0.4C0.4Pu0.2', '-0.216', '-0.2007700115']
CLiRe 11 ['Li0.2C0.4Re0.4', '-0.221', '-0.2031285415']
CLiRh 11 ['Li0.2C0.3Rh0.5', '-0.281', '-0.203326812']
CLiSm 1 ['Li0.222222C0.555556Sm0.222222', '-0.376', '-0.258259745556']
CLiTb 1 ['Li0.222222C0.555556Tb0.222222', '-0.239', '-0.228387050213']
CLiTm 1 ['Li0.222222C0.555556Tm0.222222', '-0.36', '-0.205238133333']
CLiV 3 ['Li0.3C0.5V0.2', '-0.395', '-0.20234246875']
CLiXe 1 ['Li0.333333C0.5Xe0.166667', '-0.235', '-0.235']
CLiY 7 ['Li0.375C0.5Y0.125', '-0.334', '-0.236694909297']
CLuN 19 ['C0.1N0.5Lu0.4', '-1.855', '-0.217868463017']
CLuNi 10 ['C0.285714Ni0.142857Lu0.571429', '-0.516', '-0.217274930867']
CLuO 2 ['C0.333333O0.166667Lu0.5', '-1.489', '-0.216470336559']
CLuOs 19 ['C0.125Lu0.5Os0.375', '-0.505', '-0.218617313973']
CLuP 13 ['C0.3P0.3Lu0.4', '-1.318', '-0.200910165625']
CLuPd 12 ['C0.125Pd0.375Lu0.5', '-1.008', '-0.214170349598']

CLuPt 11 ['C0.3Lu0.5Pt0.2', '-0.932', '-0.201953120497']
CLuRh 6 ['C0.125Rh0.375Lu0.5', '-1.043', '-0.208952071964']
CLuRu 28 ['C0.4Ru0.2Lu0.4', '-0.574', '-0.205173045429']
CLuS 18 ['C0.375S0.25Lu0.375', '-1.34', '-0.216143593814']
CLuSe 18 ['C0.111111Se0.333333Lu0.555556', '-1.315', '-0.200082184087']
CLuSn 2 ['C0.2Sn0.3Lu0.5', '-0.796', '-0.20142531882']
CLuTc 16 ['C0.285714Tc0.428571Lu0.285714', '-0.362', '-0.214506566224']
CLuTe 4 ['C0.111111Te0.333333Lu0.555556', '-1.004', '-0.215828754652']
CLuV 7 ['C0.5V0.166667Lu0.333333', '-0.555', '-0.208036745159']
CLuW 6 ['C0.444444Lu0.333333W0.222222', '-0.432', '-0.202565769683']
CLuXe 4 ['C0.4Xe0.3Lu0.3', '-0.423', '-0.216509192714']
CLuZn 3 ['C0.2Zn0.3Lu0.5', '-0.558', '-0.241308146857']
CMgMo 9 ['C0.3Mg0.3Mo0.4', '-0.287', '-0.217114078']
CMgNp 3 ['C0.555556Mg0.111111Np0.333333', '-0.238', '-0.238']
CMgRe 9 ['C0.4Mg0.2Re0.4', '-0.229', '-0.2111285415']
CMgV 2 ['C0.4Mg0.2V0.4', '-0.603', '-0.2176849375']
CMgW 3 ['C0.555556Mg0.111111W0.333333', '-0.302', '-0.2182275675']
CMnMo 15 ['C0.375Mn0.125Mo0.5', '-0.314', '-0.211637753955']
CMnNb 14 ['C0.222222Mn0.444444Nb0.333333', '-0.512', '-0.212102518605']
CMnRe 2 ['C0.444444Mn0.111111Re0.444444', '-0.253', '-0.219645391269']
CMnTa 3 ['C0.285714Mn0.428571Ta0.285714', '-0.54', '-0.206703743571']
CMnV 1 ['C0.222222V0.333333Mn0.444444', '-0.499', '-0.20419555408']
CMnY 1 ['C0.5Mn0.2Y0.3', '-0.49', '-0.201622789078']
CMoN 2 ['C0.285714N0.142857Mo0.571429', '-0.411', '-0.201789906379']
CMoNb 6 ['C0.25Nb0.375Mo0.375', '-0.525', '-0.20101033625']
CMoPa 5 ['C0.6Mo0.1Pa0.3', '-0.494', '-0.20347522']
CMoPm 7 ['C0.444444Mo0.222222Pm0.333333', '-0.25', '-0.210868216111']
CMoSc 16 ['C0.428571Sc0.285714Mo0.285714', '-0.496', '-0.200589064107']
CMoTc 2 ['C0.375Mo0.5Tc0.125', '-0.298', '-0.2106425975']
CMoTh 1 ['C0.5Mo0.125Th0.375', '-0.447', '-0.200695649219']
CMoTi 22 ['C0.25Ti0.5Mo0.25', '-0.733', '-0.202942402898']
CMoZn 1 ['C0.333333Zn0.166667Mo0.5', '-0.298', '-0.208597178958']
CNNd 26 ['C0.166667N0.333333Nd0.5', '-1.224', '-0.200035240303']
CNNp 19 ['C0.375N0.375Np0.25', '-0.809', '-0.202771393975']
CNPa 43 ['C0.428571N0.142857Pa0.428571', '-0.819', '-0.200655720129']
CNPm 5 ['C0.4N0.2Pm0.4', '-0.818', '-0.201383964432']
CNPu 26 ['C0.111111N0.444444Pu0.444444', '-1.419', '-0.20200265096']
CNRe 3 ['C0.375N0.125Re0.5', '-0.274', '-0.224831210426']
CNSc 12 ['C0.3N0.4Sc0.3', '-1.413', '-0.200643398898']
CNSm 12 ['C0.142857N0.428571Sm0.428571', '-1.562', '-0.202168535926']
CNTb 12 ['C0.333333N0.5Tb0.166667', '-0.689', '-0.210244629997']
CNTi 8 ['C0.222222N0.444444Ti0.333333', '-1.126', '-0.261308785328']
CNTm 10 ['C0.142857N0.428571Tm0.428571', '-1.856', '-0.202289537711']
CNW 9 ['C0.333333N0.222222W0.444444', '-0.341', '-0.206113097146']

CNXe 7 ['C0.222222N0.222222Xe0.555556', '-0.203', '-0.203']
CNY 22 ['C0.333333N0.5Y0.166667', '-0.806', '-0.207592418277']
CNYb 19 ['C0.375N0.375Yb0.25', '-0.736', '-0.221344700563']
CNZn 4 ['C0.125N0.375Zn0.5', '-0.324', '-0.238178579914']
CNaNd 3 ['C0.6Na0.2Nd0.2', '-0.291', '-0.20454342075']
CNaPm 14 ['C0.5Na0.125Pm0.375', '-0.23', '-0.23']
CNaPr 5 ['C0.555556Na0.222222Pr0.222222', '-0.287', '-0.205069890278']
CNaRh 1 ['C0.5Na0.375Rh0.125', '-0.219', '-0.219']
CNaSm 2 ['C0.571429Na0.142857Sm0.285714', '-0.368', '-0.216619672857']
CNbSb 3 ['C0.333333Nb0.444444Sb0.222222', '-0.647', '-0.203133882389']
CNbTc 8 ['C0.3Nb0.4Tc0.3', '-0.599', '-0.20609004995']
CNbTe 2 ['C0.285714Nb0.428571Te0.285714', '-0.697', '-0.216943554047']
CNdNi 11 ['C0.3Ni0.2Nd0.5', '-0.422', '-0.202617448375']
CNdO 4 ['C0.2O0.6Nd0.2', '-2.718', '-0.214670116221']
CNdP 2 ['C0.166667P0.333333Nd0.5', '-1.365', '-0.205902915763']
CNdPt 2 ['C0.2Nd0.5Pt0.3', '-0.951', '-0.20404407175']
CNdRe 8 ['C0.555556Nd0.111111Re0.333333', '-0.284', '-0.200040290069']
CNdRh 6 ['C0.333333Rh0.166667Nd0.5', '-0.541', '-0.2198602075']
CNdRu 1 ['C0.3Ru0.2Nd0.5', '-0.325', '-0.20198809025']
CNdS 6 ['C0.4S0.2Nd0.4', '-1.165', '-0.209457074946']
CNdSe 4 ['C0.3Se0.3Nd0.4', '-1.352', '-0.203470194875']
CNdSi 4 ['C0.375Si0.25Nd0.375', '-0.65', '-0.212882544219']
CNdTc 1 ['C0.5Tc0.25Nd0.25', '-0.312', '-0.203929275937']
CNdXe 10 ['C0.555556Xe0.111111Nd0.333333', '-0.354', '-0.20990570125']
CNiNp 3 ['C0.6Ni0.1Np0.3', '-0.213', '-0.213']
CNiPa 1 ['C0.428571Ni0.142857Pa0.428571', '-0.64', '-0.212248505476']
CNiPm 16 ['C0.5Ni0.166667Pm0.333333', '-0.298', '-0.206875673125']
CNiPr 3 ['C0.3Ni0.2Pr0.5', '-0.399', '-0.202801416375']
CNiSc 7 ['C0.25Sc0.5Ni0.25', '-0.704', '-0.200447119375']
CNiSr 7 ['C0.571429Ni0.142857Sr0.285714', '-0.231', '-0.205325453333']
CNiTb 1 ['C0.285714Ni0.285714Tb0.428571', '-0.368', '-0.226429052928']
CNiTi 4 ['C0.25Ti0.5Ni0.25', '-0.851', '-0.207489700294']
CNiTm 8 ['C0.333333Ni0.222222Tm0.444444', '-0.581', '-0.200157980556']
CNiY 7 ['C0.2Ni0.3Y0.5', '-0.601', '-0.205271931917']
CNiZr 1 ['C0.4Ni0.1Zr0.5', '-0.978', '-0.2372331075']
CNpOs 2 ['C0.555556Os0.111111Np0.333333', '-0.223', '-0.223']
CNpP 5 ['C0.1P0.5Np0.4', '-1.008', '-0.21108593602']
CNpPb 15 ['C0.5Pb0.1Np0.4', '-0.203', '-0.203']
CNpPd 5 ['C0.5Pd0.125Np0.375', '-0.271', '-0.202223406719']
CNpPm 3 ['C0.625Pm0.125Np0.25', '-0.225', '-0.225']
CNpPr 3 ['C0.571429Pr0.142857Np0.285714', '-0.264', '-0.21133064375']
CNpPt 1 ['C0.4Pt0.2Np0.4', '-0.394', '-0.21214400925']
CNpRe 13 ['C0.444444Re0.444444Np0.111111', '-0.222', '-0.202142823889']
CNpRh 7 ['C0.5Rh0.125Np0.375', '-0.27', '-0.210932485469']

CNpRu 4 ['C0.5Ru0.166667Np0.333333', '-0.228', '-0.206721453403']
CNpSb 21 ['C0.444444Sb0.111111Np0.444444', '-0.249', '-0.206342532917']
CNpSc 11 ['C0.555556Sc0.333333Np0.111111', '-0.489', '-0.202592176458']
CNpSe 5 ['C0.111111Se0.555556Np0.333333', '-1.136', '-0.220459179307']
CNpSi 11 ['C0.428571Si0.428571Np0.142857', '-0.406', '-0.226938444643']
CNpSn 12 ['C0.333333Sn0.333333Np0.333333', '-0.329', '-0.236911535633']
CNpSr 5 ['C0.571429Sr0.142857Np0.285714', '-0.216', '-0.216']
CNpTb 5 ['C0.6Tb0.1Np0.3', '-0.214', '-0.209224172596']
CNpTc 3 ['C0.5Tc0.25Np0.25', '-0.214', '-0.214']
CNpTl 5 ['C0.4Tl0.3Np0.3', '-0.222', '-0.222']
CNpXe 14 ['C0.428571Xe0.428571Np0.142857', '-0.204', '-0.204']
CNpY 1 ['C0.6Y0.2Np0.2', '-0.379', '-0.223311854875']
CNpZn 17 ['C0.285714Zn0.285714Np0.428571', '-0.2', '-0.2']
COPa 2 ['C0.222222O0.666667Pa0.111111', '-2.291', '-0.231228044413']
COPb 1 ['C0.2O0.5Pb0.3', '-1.589', '-0.220616393308']
COPm 8 ['C0.222222O0.333333Pm0.444444', '-2.261', '-0.218423928456']
COPr 1 ['C0.25O0.625Pr0.125', '-2.379', '-0.250684268121']
COSc 5 ['C0.25O0.25Sc0.5', '-2.083', '-0.210179792071']
COSm 2 ['C0.1O0.6Sm0.3', '-3.384', '-0.261668934033']
COTb 1 ['C0.25O0.625Tb0.125', '-2.39', '-0.266622186915']
COTh 3 ['C0.2O0.3Th0.5', '-2.25', '-0.2312979813']
COY 1 ['C0.25O0.625Y0.125', '-2.417', '-0.216145052262']
COYb 1 ['C0.2O0.6Yb0.2', '-2.542', '-0.214521566472']
COZn 1 ['C0.25O0.625Zn0.125', '-1.839', '-0.227922942886']
COsPa 12 ['C0.571429Os0.142857Pa0.285714', '-0.464', '-0.204080497857']
COsPm 24 ['C0.333333Pm0.333333Os0.333333', '-0.2', '-0.2']
COsPr 20 ['C0.3Pr0.5Os0.2', '-0.292', '-0.20051875475']
COsPu 11 ['C0.5Os0.166667Pu0.333333', '-0.244', '-0.218616685833']
COsSc 28 ['C0.4Sc0.4Os0.2', '-0.651', '-0.210634706118']
COsSe 1 ['C0.111111Se0.555556Os0.333333', '-0.369', '-0.216190136806']
COsSm 2 ['C0.25Sm0.5Os0.25', '-0.368', '-0.208620107292']
COsSr 3 ['C0.6Sr0.2Os0.2', '-0.201', '-0.201']
COsTh 9 ['C0.428571Os0.142857Th0.428571', '-0.482', '-0.205317055']
COsTi 1 ['C0.222222Ti0.555556Os0.222222', '-0.973', '-0.23503356586']
COsTm 2 ['C0.5Tm0.25Os0.25', '-0.378', '-0.2038929']
COsY 9 ['C0.1Y0.6Os0.3', '-0.445', '-0.219083775375']
CPPa 3 ['C0.5P0.125Pa0.375', '-0.818', '-0.226623008255']
CPPb 7 ['C0.222222P0.444444Pb0.333333', '-0.205', '-0.205']
CPPm 48 ['C0.333333P0.444444Pm0.222222', '-0.735', '-0.205325219676']
CPPr 21 ['C0.125P0.375Pr0.5', '-1.474', '-0.209477512682']
CPPu 8 ['C0.111111P0.555556Pu0.333333', '-1.062', '-0.201936767013']
CPSm 2 ['C0.222222P0.444444Sm0.333333', '-1.367', '-0.203515198518']
CPSr 42 ['C0.5P0.1Sr0.4', '-0.451', '-0.21572858827']
CPTa 19 ['C0.2P0.5Ta0.3', '-0.609', '-0.208375749312']

CPTb 3 ['C0.2P0.5Tb0.3', '-1.046', '-0.200385705914']
CPTm 11 ['C0.333333P0.222222Tm0.444444', '-1.127', '-0.203158984676']
CPV 4 ['C0.142857P0.428571V0.428571', '-0.998', '-0.202363445446']
CPW 12 ['C0.4P0.3W0.3', '-0.5', '-0.203064960062']
CPXe 10 ['C0.375P0.25Xe0.375', '-0.221', '-0.221']
CPY 10 ['C0.2P0.5Y0.3', '-1.305', '-0.20313440377']
CPZr 1 ['C0.1P0.5Zr0.4', '-1.496', '-0.25309761077']
CPaPb 6 ['C0.444444Pb0.222222Pa0.333333', '-0.507', '-0.203760580833']
CPaPd 1 ['C0.5Pd0.1Pa0.4', '-0.659', '-0.236810461583']
CPaPt 7 ['C0.333333Pt0.222222Pa0.444444', '-0.818', '-0.2111393625']
CPaRe 5 ['C0.555556Re0.111111Pa0.333333', '-0.525', '-0.216796286806']
CPaRu 4 ['C0.428571Ru0.142857Pa0.428571', '-0.62', '-0.202845494286']
CPaS 5 ['C0.125S0.625Pa0.25', '-1.279', '-0.201891229053']
CPaSb 11 ['C0.555556Sb0.111111Pa0.333333', '-0.548', '-0.207214521806']
CPaSe 41 ['C0.444444Se0.222222Pa0.333333', '-0.842', '-0.205797813611']
CPaSi 12 ['C0.333333Si0.222222Pa0.444444', '-0.581', '-0.202375968426']
CPaSn 14 ['C0.428571Sn0.142857Pa0.428571', '-0.625', '-0.206579315212']
CPaTc 1 ['C0.5Tc0.1Pa0.4', '-0.595', '-0.231112697']
CPaTe 31 ['C0.333333Te0.166667Pa0.5', '-0.716', '-0.202491846944']
CPaXe 6 ['C0.5Xe0.125Pa0.375', '-0.553', '-0.211855653438']
CPaZn 4 ['C0.5Zn0.166667Pa0.333333', '-0.532', '-0.228760580833']
CPbPm 2 ['C0.4Pm0.4Pb0.2', '-0.425', '-0.2501285255']
CPbPu 13 ['C0.3Pb0.4Pu0.3', '-0.313', '-0.210606293583']
CPbTh 2 ['C0.555556Pb0.111111Th0.333333', '-0.459', '-0.203834549722']
CPdSc 22 ['C0.111111Sc0.444444Pd0.444444', '-1.014', '-0.206749566667']
CPdSr 2 ['C0.285714Sr0.428571Pd0.285714', '-0.588', '-0.239340427143']
CPmPt 10 ['C0.4Pm0.5Pt0.1', '-0.421', '-0.20518176875']
CPmRe 28 ['C0.4Pm0.1Re0.5', '-0.234', '-0.211660676875']
CPmRh 3 ['C0.3Rh0.2Pm0.5', '-0.477', '-0.2043025335']
CPmRu 20 ['C0.375Ru0.125Pm0.5', '-0.244', '-0.216523585938']
CPmS 26 ['C0.2S0.2Pm0.6', '-0.952', '-0.203860334696']
CPmSe 41 ['C0.333333Se0.444444Pm0.222222', '-0.924', '-0.203564355']
CPmSi 10 ['C0.555556Si0.111111Pm0.333333', '-0.305', '-0.207524526944']
CPmTc 22 ['C0.6Tc0.1Pm0.3', '-0.2', '-0.2']
CPmTe 3 ['C0.1Te0.5Pm0.4', '-1.248', '-0.203954161664']
CPmW 15 ['C0.444444Pm0.333333W0.222222', '-0.278', '-0.222151711667']
CPmXe 12 ['C0.571429Xe0.142857Pm0.285714', '-0.2', '-0.2']
CPrPt 15 ['C0.285714Pr0.428571Pt0.285714', '-0.892', '-0.20026991875']
CPrRe 14 ['C0.5Pr0.4Re0.1', '-0.391', '-0.206100442']
CPrRh 2 ['C0.25Rh0.25Pr0.5', '-0.608', '-0.223899995833']
CPrRu 20 ['C0.4Ru0.3Pr0.3', '-0.327', '-0.22051848325']
CPrS 16 ['C0.222222S0.222222Pr0.555556', '-1.222', '-0.203759719847']
CPrSe 8 ['C0.3Se0.3Pr0.4', '-1.364', '-0.218045320375']
CPrSi 10 ['C0.428571Si0.285714Pr0.285714', '-0.631', '-0.205140225']

CPrTc 10 ['C0.4Tc0.3Pr0.3', '-0.31', '-0.211683868333']
CPrTe 2 ['C0.428571Te0.142857Pr0.428571', '-0.718', '-0.200500731666']
CPrXe 10 ['C0.5Xe0.3Pr0.2', '-0.276', '-0.20226290125']
CPtSc 18 ['C0.25Sc0.625Pt0.125', '-0.865', '-0.201782342188']
CPtSr 18 ['C0.333333Sr0.5Pt0.166667', '-0.443', '-0.202393716667']
CPtTh 2 ['C0.111111Pt0.666667Th0.222222', '-0.787', '-0.244186841111']
CPtTi 9 ['C0.3Ti0.6Pt0.1', '-0.972', '-0.207508693978']
CPtY 6 ['C0.285714Y0.571429Pt0.142857', '-0.786', '-0.206536944405']
CPuRe 3 ['C0.375Re0.25Pu0.375', '-0.227', '-0.203138681354']
CPuSb 12 ['C0.428571Sb0.285714Pu0.285714', '-0.584', '-0.219917658571']
CPuTc 11 ['C0.333333Tc0.222222Pu0.444444', '-0.218', '-0.201077790556']
CPuTl 1 ['C0.375Tl0.25Pu0.375', '-0.228', '-0.2011946825']
CRbS 7 ['C0.125S0.375Rb0.5', '-1.148', '-0.20320866808']
CReSc 2 ['C0.5Sc0.1Re0.4', '-0.358', '-0.2059772788']
CReSi 23 ['C0.4Si0.3Re0.3', '-0.402', '-0.203770410268']
CReSm 6 ['C0.5Sm0.166667Re0.333333', '-0.344', '-0.201389259271']
CReSr 6 ['C0.555556Sr0.333333Re0.111111', '-0.216', '-0.211035705972']
CReTb 9 ['C0.5Tb0.2Re0.3', '-0.28', '-0.204911727316']
CReTc 1 ['C0.4Tc0.1Re0.5', '-0.258', '-0.235660676875']
CReTh 16 ['C0.555556Re0.222222Th0.222222', '-0.381', '-0.200442226389']
CReTm 1 ['C0.6Tm0.2Re0.2', '-0.46', '-0.228735838625']
CReZn 3 ['C0.333333Zn0.166667Re0.5', '-0.229', '-0.206660676875']
CReZr 2 ['C0.111111Zr0.555556Re0.333333', '-0.564', '-0.2030725475']
CRhSc 18 ['C0.111111Sc0.333333Rh0.555556', '-0.943', '-0.203566294167']
CRhSm 15 ['C0.125Rh0.5Sm0.375', '-0.824', '-0.205871147813']
CRhSr 27 ['C0.285714Sr0.428571Rh0.285714', '-0.312', '-0.204562961429']
CRhTi 1 ['C0.285714Ti0.571429Rh0.142857', '-0.986', '-0.238465133789']
CRhY 10 ['C0.222222Y0.555556Rh0.222222', '-0.75', '-0.204914073704']
CRuSc 29 ['C0.1Sc0.4Ru0.5', '-0.67', '-0.213942548625']
CRuSm 10 ['C0.555556Ru0.111111Sm0.333333', '-0.389', '-0.210912489583']
CRuSr 6 ['C0.625Sr0.25Ru0.125', '-0.214', '-0.214']
CRuTb 2 ['C0.285714Ru0.285714Tb0.428571', '-0.23', '-0.219083823076']
CRuTh 9 ['C0.166667Ru0.333333Th0.5', '-0.655', '-0.205227780417']
CRuTm 12 ['C0.2Ru0.4Tm0.4', '-0.538', '-0.21375696']
CRuY 9 ['C0.3Y0.4Ru0.3', '-0.528', '-0.210471337292']
CRuZr 2 ['C0.142857Zr0.428571Ru0.428571', '-0.806', '-0.206552964643']
CSSc 6 ['C0.25S0.25Sc0.5', '-1.43', '-0.206384575089']
CSSm 17 ['C0.1S0.4Sm0.5', '-1.972', '-0.200095143059']
CSTh 4 ['C0.25S0.375Th0.375', '-1.713', '-0.217971842243']
CSV 12 ['C0.142857S0.428571V0.428571', '-1.181', '-0.201207318679']
CSXe 7 ['C0.2S0.4Xe0.4', '-0.307', '-0.218698599895']
CSY 4 ['C0.166667S0.333333Y0.5', '-1.752', '-0.217631408799']
CSZr 2 ['C0.1S0.4Zr0.5', '-1.674', '-0.213765228142']
CSbSc 10 ['C0.333333Sc0.444444Sb0.222222', '-0.819', '-0.209230059861']

CSbTa 1 ['C0.333333Sb0.166667Ta0.5', '-0.698', '-0.200783834167']
CSbTh 4 ['C0.555556Sb0.111111Th0.333333', '-0.586', '-0.21202400375']
CSbTm 2 ['C0.5Sb0.2Tm0.3', '-0.698', '-0.200962481']
CSbU 24 ['C0.333333Sb0.222222U0.444444', '-0.398', '-0.217072007639']
CSbV 7 ['C0.3V0.4Sb0.3', '-0.561', '-0.202879059483']
CSbW 4 ['C0.5Sb0.1W0.4', '-0.321', '-0.220473081']
CScSe 23 ['C0.2Sc0.3Se0.5', '-1.159', '-0.2025929485']
CScTc 12 ['C0.3Sc0.5Tc0.2', '-0.566', '-0.20161577425']
CScV 6 ['C0.333333Sc0.333333V0.333333', '-0.644', '-0.20583972125']
CScW 2 ['C0.5Sc0.1W0.4', '-0.403', '-0.216550733938']
CScZn 11 ['C0.2Sc0.4Zn0.4', '-0.6', '-0.2066970265']
CSeSr 4 ['C0.222222Se0.333333Sr0.444444', '-1.592', '-0.210276603333']
CSeTb 3 ['C0.3Se0.3Tb0.4', '-1.09', '-0.218144845198']
CSeTc 12 ['C0.285714Se0.428571Tc0.285714', '-0.225', '-0.225']
CSeTh 1 ['C0.142857Se0.428571Th0.428571', '-1.54', '-0.212152655714']
CSeTm 8 ['C0.25Se0.25Tm0.5', '-1.172', '-0.2277417175']
CSeU 1 ['C0.3Se0.3U0.4', '-0.858', '-0.2053600045']
CSeXe 10 ['C0.1Se0.4Xe0.5', '-0.203', '-0.203']
CSeY 12 ['C0.285714Se0.428571Y0.285714', '-1.234', '-0.224913584286']
CSeZr 15 ['C0.25Se0.25Zr0.5', '-1.178', '-0.204652223055']
CSiTc 13 ['C0.333333Si0.333333Tc0.333333', '-0.493', '-0.204581566667']
CSiTh 16 ['C0.4Si0.2Th0.4', '-0.649', '-0.216881182']
CSiTm 1 ['C0.5Si0.125Tm0.375', '-0.587', '-0.208709845']
CSiU 2 ['C0.166667Si0.5U0.333333', '-0.507', '-0.201950577484']
CSiW 32 ['C0.428571Si0.428571W0.142857', '-0.413', '-0.200528749246']
CSiZr 1 ['C0.428571Si0.142857Zr0.428571', '-0.956', '-0.212560999405']
CSmXe 4 ['C0.428571Xe0.285714Sm0.285714', '-0.356', '-0.204619672857']
CSnZr 1 ['C0.428571Zr0.428571Sn0.142857', '-0.904', '-0.207992171071']
CSrTc 2 ['C0.6Sr0.3Tc0.1', '-0.208', '-0.208']
CSrW 3 ['C0.6Sr0.1W0.3', '-0.295', '-0.21960481075']
CSrXe 6 ['C0.444444Sr0.222222Xe0.333333', '-0.222', '-0.222']
CTaTc 5 ['C0.2Tc0.4Ta0.4', '-0.643', '-0.2092655455']
CTcTh 34 ['C0.333333Tc0.444444Th0.222222', '-0.355', '-0.204735749167']
CTcTm 3 ['C0.444444Tc0.222222Tm0.333333', '-0.45', '-0.243650844444']
CTcZr 2 ['C0.142857Zr0.428571Tc0.428571', '-0.681', '-0.212162582143']
CTeTh 1 ['C0.2Te0.4Th0.4', '-1.126', '-0.230430267967']
CTeY 10 ['C0.111111Y0.555556Te0.333333', '-1.174', '-0.201940898609']
CThW 1 ['C0.444444W0.444444Th0.111111', '-0.365', '-0.21895484']
CThXe 4 ['C0.428571Xe0.285714Th0.285714', '-0.415', '-0.221803106071']
CTmXe 2 ['C0.428571Xe0.285714Tm0.285714', '-0.403', '-0.204020457143']
CUXe 4 ['C0.5Xe0.3U0.2', '-0.29', '-0.221228022']
CZnZr 2 ['C0.428571Zn0.142857Zr0.428571', '-0.915', '-0.218992171071']
CaCdF 6 ['F0.7Ca0.2Cd0.1', '-3.47', '-0.201625238522']
CaCdO 3 ['O0.6Ca0.3Cd0.1', '-2.391', '-0.20067920047']

CaCeF 2 ['F0.8Ca0.1Ce0.1', '-3.238', '-0.223397360018']
CaCeI 4 ['Ca0.2I0.7Ce0.1', '-1.558', '-0.214689318915']
CaCeO 4 ['O0.6Ca0.1Ce0.3', '-3.504', '-0.218188996283']
CaClEr 11 ['Cl0.666667Ca0.222222Er0.111111', '-2.424', '-0.361248390211']
CaClEu 1 ['Cl0.6Ca0.1Eu0.3', '-2.764', '-0.224612510878']
CaClF 12 ['F0.333333Cl0.222222Ca0.444444', '-3.214', '-0.220076459597']
CaClHo 11 ['Cl0.6Ca0.1Ho0.3', '-1.883', '-0.274940529188']
CaClIn 2 ['Cl0.714286Ca0.142857In0.142857', '-1.829', '-0.209084337379']
CaClLu 11 ['Cl0.666667Ca0.222222Lu0.111111', '-2.418', '-0.373817031878']
CaClNa 5 ['Na0.333333Cl0.444444Ca0.222222', '-2.012', '-0.203151138327']
CaClO 19 ['O0.222222Cl0.444444Ca0.333333', '-2.705', '-0.212078755083']
CaClP 11 ['P0.3Cl0.4Ca0.3', '-2.121', '-0.201119020064']
CaClPm 1 ['Cl0.7Ca0.1Pm0.2', '-2.473', '-0.254447960566']
CaClPu 3 ['Cl0.75Ca0.125Pu0.125', '-2.308', '-0.225543805865']
CaClRh 7 ['Cl0.75Ca0.125Rh0.125', '-1.223', '-0.228252268595']
CaClS 2 ['S0.4Cl0.1Ca0.5', '-2.502', '-0.25328498183']
CaClSb 3 ['Cl0.3Ca0.4Sb0.3', '-1.855', '-0.201141426147']
CaClSe 2 ['Cl0.375Ca0.375Se0.25', '-2.461', '-0.202517666643']
CaClSm 2 ['Cl0.7Ca0.1Sm0.2', '-2.407', '-0.219410051317']
CaCoF 3 ['F0.75Ca0.125Co0.125', '-2.866', '-0.310170817628']
CaCoH 5 ['H0.4Ca0.5Co0.1', '-0.551', '-0.208424153626']
CaCoN 29 ['N0.555556Ca0.333333Co0.111111', '-0.75', '-0.203954713663']
CaCoO 2 ['O0.625Ca0.25Co0.125', '-2.267', '-0.250608275247']
CaCoP 3 ['P0.8Ca0.1Co0.1', '-0.827', '-0.224217796874']
CaCrF 1 ['F0.8Ca0.1Cr0.1', '-2.992', '-0.27027480136']
CaCrH 2 ['H0.444444Ca0.444444Cr0.111111', '-0.601', '-0.220360170696']
CaCrN 18 ['N0.5Ca0.3Cr0.2', '-1.036', '-0.203548075827']
CaCrO 2 ['O0.625Ca0.25Cr0.125', '-2.837', '-0.248223913918']
CaCsF 16 ['F0.5Ca0.166667Cs0.333333', '-3.298', '-0.2000877621']
CaCsO 1 ['O0.666667Ca0.166667Cs0.166667', '-1.862', '-0.211133039831']
CaCuF 6 ['F0.7Ca0.2Cu0.1', '-3.253', '-0.202253036023']
CaCuH 2 ['H0.4Ca0.5Cu0.1', '-0.593', '-0.214531058626']
CaCuN 10 ['N0.5Ca0.25Cu0.25', '-0.632', '-0.209472498266']
CaCuO 4 ['O0.6Ca0.3Cu0.1', '-2.317', '-0.203961651302']
CaDyF 3 ['F0.75Ca0.125Dy0.125', '-3.98', '-0.333652879713']
CaDyI 6 ['Ca0.111111I0.666667Dy0.222222', '-1.354', '-0.238797153837']
CaDyO 1 ['O0.6Ca0.2Dy0.2', '-3.448', '-0.231930730765']
CaErF 3 ['F0.75Ca0.125Er0.125', '-4.038', '-0.234859275858']
CaErI 7 ['Ca0.1I0.8Er0.1', '-0.958', '-0.200110956963']
CaErO 2 ['O0.625Ca0.125Er0.25', '-3.471', '-0.220556618198']
CaEuF 2 ['F0.777778Ca0.111111Eu0.111111', '-3.177', '-0.248405158163']
CaEuN 4 ['N0.4Ca0.2Eu0.4', '-0.845', '-0.240082299529']
CaEuO 1 ['O0.625Ca0.25Eu0.125', '-2.688', '-0.208796360072']
CaEuP 11 ['P0.5Ca0.2Eu0.3', '-1.364', '-0.20113508994']

CaFFe 2 ['F0.777778Ca0.111111Fe0.111111', '-2.911', '-0.441124767149']
CaFGa 3 ['F0.75Ca0.125Ga0.125', '-3.324', '-0.305609442417']
CaFGd 3 ['F0.714286Ca0.142857Gd0.142857', '-4.426', '-0.243211592409']
CaFGe 2 ['F0.8Ca0.1Ge0.1', '-2.852', '-0.296854438024']
CaFH 30 ['H0.5F0.1Ca0.4', '-1.262', '-0.20086919162']
CaFHf 2 ['F0.777778Ca0.111111Hf0.111111', '-4.049', '-0.367060897804']
CaFHg 5 ['F0.571429Ca0.142857Hg0.285714', '-2.798', '-0.214291071934']
CaFHo 2 ['F0.777778Ca0.111111Ho0.111111', '-3.624', '-0.243166347891']
CaFIn 4 ['F0.75Ca0.125In0.125', '-3.21', '-0.238949923668']
CaFK 17 ['F0.666667K0.111111Ca0.222222', '-3.67', '-0.200698717982']
CaFKr 4 ['F0.714286Ca0.142857Kr0.142857', '-2.143', '-0.201026208354']
CaFLa 2 ['F0.8Ca0.1La0.1', '-3.396', '-0.338484574102']
CaFLi 8 ['Li0.1F0.7Ca0.2', '-3.381', '-0.219380646434']
CaFLu 3 ['F0.75Ca0.125Lu0.125', '-3.966', '-0.360605292418']
CaFMg 11 ['F0.555556Mg0.333333Ca0.111111', '-3.558', '-0.239944258953']
CaFMn 2 ['F0.777778Ca0.111111Mn0.111111', '-3.024', '-0.230922574188']
CaFMo 3 ['F0.75Ca0.125Mo0.125', '-3.426', '-0.239356419193']
CaFN 3 ['N0.125F0.75Ca0.125', '-1.91', '-0.327722498968']
CaFNa 30 ['F0.428571Na0.428571Ca0.142857', '-2.872', '-0.213969658823']
CaFNb 2 ['F0.777778Ca0.111111Nb0.111111', '-3.847', '-0.27045380734']
CaFNd 2 ['F0.777778Ca0.111111Nd0.111111', '-3.711', '-0.352539141962']
CaFNi 3 ['F0.75Ca0.125Ni0.125', '-2.708', '-0.218032982418']
CaFNp 2 ['F0.8Ca0.1Np0.1', '-3.752', '-0.599909147479']
CaFO 8 ['O0.222222F0.444444Ca0.333333', '-3.756', '-0.218581386857']
CaFP 11 ['F0.111111P0.333333Ca0.555556', '-1.728', '-0.20446015989']
CaFPa 3 ['F0.75Ca0.125Pa0.125', '-4.405', '-1.02403478554']
CaFPb 2 ['F0.8Ca0.1Pb0.1', '-2.611', '-0.281710096524']
CaFPd 2 ['F0.8Ca0.1Pd0.1', '-2.285', '-0.24710414752']
CaFPm 3 ['F0.777778Ca0.111111Pm0.111111', '-3.53', '-0.27052590354']
CaFPr 3 ['F0.777778Ca0.111111Pr0.111111', '-3.581', '-0.226235880207']
CaFPt 2 ['F0.777778Ca0.111111Pt0.111111', '-2.549', '-0.218148186092']
CaFPu 4 ['F0.666667Ca0.111111Pu0.222222', '-3.924', '-0.204692139467']
CaFRb 13 ['F0.5Ca0.2Rb0.3', '-3.321', '-0.200512759434']
CaFRh 3 ['F0.75Ca0.125Rh0.125', '-2.743', '-0.263533599292']
CaFRu 3 ['F0.75Ca0.125Ru0.125', '-2.997', '-0.222920900655']
CaFS 7 ['F0.285714S0.285714Ca0.428571', '-3.332', '-0.201671480529']
CaFSb 27 ['F0.3Ca0.5Sb0.2', '-2.658', '-0.225317960261']
CaFSc 3 ['F0.75Ca0.125Sc0.125', '-3.961', '-0.243805693667']
CaFSe 11 ['F0.8Ca0.1Se0.1', '-2.452', '-0.202971706524']
CaFSi 6 ['F0.666667Si0.111111Ca0.222222', '-3.944', '-0.201892220859']
CaFSm 2 ['F0.8Ca0.1Sm0.1', '-3.246', '-0.211391405186']
CaFSn 3 ['F0.777778Ca0.111111Sn0.111111', '-3.202', '-0.203856879822']
CaFSr 3 ['F0.777778Ca0.111111Sr0.111111', '-3.012', '-0.20353522483']
CaFTa 3 ['F0.777778Ca0.111111Ta0.111111', '-3.949', '-0.22962709512']

CaFTc 2 ['F0.777778Ca0.111111Tc0.111111', '-3.118', '-0.470023077943']
CaFTe 1 ['F0.666667Ca0.222222Te0.111111', '-3.675', '-0.204835168728']
CaFTh 2 ['F0.8Ca0.1Th0.1', '-3.846', '-0.349991382689']
CaFTi 2 ['F0.777778Ca0.111111Ti0.111111', '-3.71', '-0.241293736827']
CaFTl 4 ['F0.6Ca0.2Tl0.2', '-3.526', '-0.22545930352']
CaFTm 11 ['F0.666667Ca0.222222Tm0.111111', '-4.024', '-0.726808127149']
CaFV 2 ['F0.777778Ca0.111111V0.111111', '-3.498', '-0.426253590027']
CaFW 1 ['F0.8Ca0.1W0.1', '-3.52', '-0.207582255198']
CaFXe 1 ['F0.8Ca0.1Xe0.1', '-1.871', '-0.228290721522']
CaFY 3 ['F0.75Ca0.125Y0.125', '-3.979', '-0.297665196583']
CaFYb 12 ['F0.75Ca0.125Yb0.125', '-3.412', '-0.234715254292']
CaFZn 5 ['F0.714286Ca0.142857Zn0.142857', '-3.114', '-0.205491161927']
CaFZr 2 ['F0.8Ca0.1Zr0.1', '-3.585', '-0.338697203399']
CaFeH 6 ['H0.5Ca0.333333Fe0.166667', '-0.661', '-0.232780192033']
CaFeO 3 ['O0.625Ca0.25Fe0.125', '-2.416', '-0.229349467147']
CaFeSi 4 ['Si0.444444Ca0.111111Fe0.444444', '-0.697', '-0.210610967361']
CaGaIr 6 ['Ca0.166667Ga0.5Ir0.333333', '-0.679', '-0.209109337222']
CaGaO 3 ['O0.6Ca0.3Ga0.1', '-2.821', '-0.327591225386']
CaGaPt 1 ['Ca0.111111Ga0.555556Pt0.333333', '-0.944', '-0.244633057222']
CaGaRh 3 ['Ca0.1Ga0.4Rh0.5', '-0.825', '-0.202466148333']
CaGdI 8 ['Ca0.1I0.8Gd0.1', '-1.043', '-0.2616767929']
CaGdO 3 ['O0.625Ca0.125Gd0.25', '-3.494', '-0.308566776948']
CaGeH 15 ['H0.25Ca0.5Ge0.25', '-0.821', '-0.202930590391']
CaGeIr 3 ['Ca0.1Ge0.5Ir0.4', '-0.718', '-0.225825199904']
CaGeO 4 ['O0.666667Ca0.166667Ge0.166667', '-2.247', '-0.215849605245']
CaGeP 2 ['P0.285714Ca0.428571Ge0.285714', '-1.118', '-0.202915718394']
CaGePa 2 ['Ca0.1Ge0.7Pa0.2', '-0.499', '-0.222926481']
CaGeS 13 ['S0.428571Ca0.285714Ge0.285714', '-1.649', '-0.213540190778']
CaGeSe 18 ['Ca0.444444Ge0.222222Se0.333333', '-1.731', '-0.203718957778']
CaHI 5 ['H0.4Ca0.5I0.1', '-0.833', '-0.226908691884']
CaHIr 20 ['H0.666667Ca0.111111Ir0.222222', '-0.397', '-0.206680085348']
CaHLi 4 ['H0.5Li0.1Ca0.4', '-0.643', '-0.211283792283']
CaHMg 1 ['H0.5Mg0.1Ca0.4', '-0.664', '-0.216864568866']
CaHMn 8 ['H0.666667Ca0.166667Mn0.166667', '-0.493', '-0.207520128022']
CaHN 16 ['H0.3N0.3Ca0.4', '-1.003', '-0.203025373117']
CaHNa 6 ['H0.5Na0.2Ca0.3', '-0.635', '-0.206780192033']
CaHNi 4 ['H0.5Ca0.4Ni0.1', '-0.676', '-0.220331098533']
CaHO 1 ['H0.166667O0.333333Ca0.5', '-2.531', '-0.258193422051']
CaHOs 4 ['H0.444444Ca0.444444Os0.111111', '-0.614', '-0.209204582733']
CaHP 20 ['H0.25P0.25Ca0.5', '-1.043', '-0.213661049611']
CaHPb 4 ['H0.4Ca0.4Pb0.2', '-0.757', '-0.202203571626']
CaHPd 11 ['H0.444444Ca0.444444Pd0.111111', '-0.776', '-0.205687082316']
CaHPm 8 ['H0.666667Ca0.166667Pm0.166667', '-0.664', '-0.213287318127']
CaHPt 11 ['H0.666667Ca0.166667Pt0.166667', '-0.589', '-0.208674574844']

CaHRh 6 ['H0.375Ca0.5Rh0.125', '-0.607', '-0.20661332715']
CaHRu 4 ['H0.428571Ca0.428571Ru0.142857', '-0.619', '-0.22810595835']
CaHS 7 ['H0.222222S0.333333Ca0.444444', '-1.937', '-0.204333480675']
CaHSb 12 ['H0.6Ca0.3Sb0.1', '-0.735', '-0.208199438845']
CaHSe 3 ['H0.428571Ca0.428571Se0.142857', '-1.177', '-0.226060556028']
CaHSi 22 ['H0.285714Si0.285714Ca0.428571', '-0.766', '-0.206863079733']
CaHSn 11 ['H0.6Ca0.3Sn0.1', '-0.723', '-0.20432757815']
CaHTc 2 ['H0.5Ca0.375Tc0.125', '-0.638', '-0.209780192033']
CaHfN 7 ['N0.5Ca0.125Hf0.375', '-1.841', '-0.227571075664']
CaHfS 5 ['S0.5Ca0.125Hf0.375', '-1.895', '-0.230182100494']
CaHgO 1 ['O0.4Ca0.2Hg0.4', '-1.777', '-0.216752654656']
CaHoI 6 ['Ca0.111111I0.777778Ho0.111111', '-1.125', '-0.282998989519']
CaHoO 2 ['O0.625Ca0.125Ho0.25', '-3.505', '-0.268624136585']
CaILu 9 ['Ca0.222222I0.666667Lu0.111111', '-1.53', '-0.235356562541']
CaINd 2 ['Ca0.2I0.7Nd0.1', '-1.689', '-0.208823900043']
CaIPa 7 ['Ca0.111111I0.666667Pa0.222222', '-0.852', '-0.204775100033']
CaIPm 8 ['Ca0.1I0.8Pm0.1', '-0.979', '-0.273044377022']
CaIPt 1 ['Ca0.111111I0.666667Pt0.222222', '-0.969', '-0.206150119493']
CaIPu 1 ['Ca0.125I0.75Pu0.125', '-1.471', '-0.208244737234']
CaIRh 2 ['Ca0.125Rh0.25I0.625', '-0.914', '-0.255211345644']
CaIS 1 ['S0.4Ca0.5I0.1', '-2.344', '-0.22966861265']
CaISm 4 ['Ca0.2I0.7Sm0.1', '-1.641', '-0.205076731552']
CaITb 8 ['Ca0.166667I0.666667Tb0.166667', '-1.307', '-0.287447629676']
CaInIr 1 ['Ca0.166667In0.5Ir0.333333', '-0.557', '-0.203903279896']
CaInN 25 ['N0.5Ca0.3In0.2', '-0.708', '-0.20156271851']
CaInO 3 ['O0.625Ca0.25In0.125', '-2.433', '-0.223835743821']
CaInP 1 ['P0.8Ca0.1In0.1', '-0.669', '-0.241400424624']
CaInS 2 ['S0.444444Ca0.444444In0.111111', '-2.265', '-0.208537860436']
CaInSe 2 ['Ca0.444444Se0.333333In0.222222', '-1.718', '-0.205056157222']
CaIrN 7 ['N0.571429Ca0.285714Ir0.142857', '-0.692', '-0.207816389402']
CaIrO 1 ['O0.6Ca0.3Ir0.1', '-2.511', '-0.24915576697']
CaIrP 5 ['P0.5Ca0.25Ir0.25', '-1.256', '-0.209605417327']
CaIrPa 1 ['Ca0.125Ir0.625Pa0.25', '-0.992', '-0.267446515']
CaIrSi 6 ['Si0.4Ca0.2Ir0.4', '-1.036', '-0.207038943']
CaIrTa 1 ['Ca0.125Ta0.25Ir0.625', '-0.814', '-0.2071728995']
CaKSe 4 ['K0.285714Ca0.285714Se0.428571', '-1.849', '-0.205196674286']
CaKrP 3 ['P0.75Ca0.125Kr0.125', '-0.619', '-0.20864423789']
CaLaN 1 ['N0.444444Ca0.111111La0.444444', '-1.516', '-0.248049379849']
CaLaO 2 ['O0.625Ca0.125La0.25', '-3.388', '-0.252622725854']
CaLiP 8 ['Li0.2P0.3Ca0.5', '-0.981', '-0.203093657758']
CaLiSe 4 ['Li0.166667Ca0.5Se0.333333', '-1.594', '-0.225753580417']
CaLuO 4 ['O0.625Ca0.25Lu0.125', '-3.071', '-0.21104035851']
CaMgO 2 ['O0.625Mg0.125Ca0.25', '-2.571', '-0.204432273927']
CaMgP 1 ['Mg0.222222P0.333333Ca0.444444', '-1.072', '-0.209659886645']

CaMnN 19 ['N0.333333Ca0.333333Mn0.333333', '-0.943', '-0.204897058353']
CaMnO 3 ['O0.6Ca0.2Mn0.2', '-2.643', '-0.200112688033']
CaMoN 16 ['N0.625Ca0.125Mo0.25', '-0.687', '-0.220819827949']
CaMoO 2 ['O0.625Ca0.125Mo0.25', '-2.698', '-0.205493345078']
CaNNb 13 ['N0.571429Ca0.285714Nb0.142857', '-1.029', '-0.217889074892']
CaNNi 2 ['N0.428571Ca0.142857Ni0.428571', '-0.538', '-0.20513056646']
CaNNp 25 ['N0.555556Ca0.333333Np0.111111', '-1.024', '-0.225844493628']
CaNO 8 ['N0.166667O0.333333Ca0.5', '-2.592', '-0.206235430847']
CaNOs 8 ['N0.5Ca0.4Os0.1', '-0.839', '-0.20973273036']
CaNP 31 ['N0.333333P0.444444Ca0.222222', '-1.133', '-0.212302290307']
CaNPa 24 ['N0.666667Ca0.111111Pa0.222222', '-0.884', '-0.210924449538']
CaNPu 12 ['N0.5Ca0.1Pu0.4', '-1.453', '-0.204283629548']
CaNRe 26 ['N0.375Ca0.375Re0.25', '-0.796', '-0.20461722827']
CaNRh 5 ['N0.5Ca0.25Rh0.25', '-0.628', '-0.205472498266']
CaNRu 5 ['N0.571429Ca0.285714Ru0.142857', '-0.705', '-0.222111426589']
CaNSb 1 ['N0.571429Ca0.142857Sb0.285714', '-0.527', '-0.217658031946']
CaNTa 14 ['N0.5Ca0.1Ta0.4', '-1.412', '-0.208460840996']
CaNTc 23 ['N0.625Ca0.125Tc0.25', '-0.525', '-0.20933806252']
CaNTi 13 ['N0.428571Ca0.285714Ti0.285714', '-1.599', '-0.239714663054']
CaNU 14 ['N0.428571Ca0.428571U0.142857', '-1.289', '-0.207157919851']
CaNV 1 ['N0.5Ca0.25V0.25', '-1.517', '-0.23752026764']
CaNW 25 ['N0.666667Ca0.166667W0.166667', '-0.595', '-0.22804896399']
CaNZn 1 ['N0.4Ca0.1Zn0.5', '-0.427', '-0.204205747029']
CaNZr 4 ['N0.5Ca0.333333Zr0.166667', '-1.349', '-0.225677424777']
CaNaO 4 ['O0.4Na0.3Ca0.3', '-2.567', '-0.221345786477']
CaNaP 14 ['Na0.222222P0.444444Ca0.333333', '-1.197', '-0.204827890692']
CaNaS 4 ['Na0.166667S0.333333Ca0.5', '-1.77', '-0.227653395327']
CaNaSe 16 ['Na0.285714Ca0.285714Se0.428571', '-1.868', '-0.208639609759']
CaNbO 1 ['O0.6Ca0.2Nb0.2', '-3.217', '-0.21179410147']
CaNdO 1 ['O0.625Ca0.125Nd0.25', '-3.374', '-0.233344862577']
CaNiO 3 ['O0.625Ca0.25Ni0.125', '-2.16', '-0.219539863018']
CaNiP 2 ['P0.333333Ca0.444444Ni0.222222', '-1.103', '-0.221696619238']
CaNpO 3 ['O0.625Ca0.25Np0.125', '-3.236', '-0.230780824044']
CaNpS 2 ['S0.5Ca0.125Np0.375', '-1.911', '-0.201241695963']
CaOOs 1 ['O0.6Ca0.3Os0.1', '-2.792', '-0.330400086974']
CaOP 18 ['O0.333333P0.222222Ca0.444444', '-2.661', '-0.201852277515']
CaOPa 8 ['O0.6Ca0.2Pa0.2', '-3.392', '-0.20410898347']
CaOPb 2 ['O0.6Ca0.2Pb0.2', '-2.266', '-0.21097158947']
CaOPd 2 ['O0.625Ca0.25Pd0.125', '-2.116', '-0.229980412885']
CaOPr 1 ['O0.625Ca0.25Pr0.125', '-3.001', '-0.261529576244']
CaOPt 3 ['O0.6Ca0.2Pt0.2', '-2.103', '-0.259742391139']
CaOPu 3 ['O0.625Ca0.25Pu0.125', '-3.195', '-0.290297964454']
CaORb 1 ['O0.428571Ca0.285714Rb0.285714', '-2.489', '-0.214249380337']
CaORe 3 ['O0.625Ca0.125Re0.25', '-2.33', '-0.215725250441']

CaORh 2 ['O0.625Ca0.125Rh0.25', '-1.811', '-0.208078926325']
CaORu 1 ['O0.6Ca0.3Ru0.1', '-2.58', '-0.247536667472']
CaOSc 4 ['O0.625Ca0.125Sc0.25', '-3.398', '-0.203340485697']
CaOSe 14 ['O0.222222Ca0.444444Se0.333333', '-2.537', '-0.208676180916']
CaOSi 2 ['O0.6Si0.1Ca0.3', '-3.191', '-0.205175479723']
CaOSm 2 ['O0.625Ca0.25Sm0.125', '-2.984', '-0.205478993432']
CaOSn 3 ['O0.4Ca0.5Sn0.1', '-2.902', '-0.205303161463']
CaOTb 1 ['O0.625Ca0.25Tb0.125', '-2.946', '-0.211467495038']
CaOTc 7 ['O0.666667Ca0.166667Tc0.166667', '-2.347', '-0.216959481773']
CaOTh 1 ['O0.571429Ca0.142857Th0.285714', '-3.918', '-0.281384140303']
CaOTl 4 ['O0.625Ca0.125Tl0.25', '-1.622', '-0.221175661949']
CaOTm 4 ['O0.6Ca0.3Tm0.1', '-3.157', '-0.237459684845']
CaOU 1 ['O0.666667Ca0.111111U0.222222', '-3.715', '-0.261755935518']
CaOV 1 ['O0.6Ca0.2V0.2', '-3.105', '-0.20439852492']
CaOY 3 ['O0.6Ca0.2Y0.2', '-3.41', '-0.21485007697']
CaOZn 3 ['O0.6Ca0.2Zn0.2', '-2.182', '-0.21837278447']
CaOZr 3 ['O0.6Ca0.3Zr0.1', '-3.261', '-0.21142490572']
CaOsP 10 ['P0.555556Ca0.222222Os0.222222', '-1.102', '-0.210573961875']
CaOsSi 3 ['Si0.444444Ca0.111111Os0.444444', '-0.594', '-0.220746975972']
CaPPm 10 ['P0.4Ca0.2Pm0.4', '-1.181', '-0.212109460584']
CaPPu 16 ['P0.428571Ca0.142857Pu0.428571', '-1.31', '-0.204204414731']
CaPRh 29 ['P0.625Ca0.125Rh0.25', '-1.122', '-0.202280124817']
CaPRu 1 ['P0.555556Ca0.111111Ru0.333333', '-1.102', '-0.203063406444']
CaPS 3 ['P0.222222S0.444444Ca0.333333', '-1.849', '-0.201455907859']
CaPSb 1 ['P0.4Ca0.3Sb0.3', '-1.002', '-0.210108964194']
CaPSe 1 ['P0.111111Ca0.555556Se0.333333', '-1.848', '-0.212145781598']
CaPSi 4 ['Si0.333333P0.5Ca0.166667', '-0.795', '-0.20097684941']
CaPSn 7 ['P0.625Ca0.125Sn0.25', '-0.709', '-0.201607674076']
CaPTa 7 ['P0.375Ca0.125Ta0.5', '-1.161', '-0.236357307682']
CaPTc 10 ['P0.5Ca0.125Tc0.375', '-0.965', '-0.200002073259']
CaPTe 3 ['P0.5Ca0.125Te0.375', '-0.622', '-0.206835899583']
CaPTi 1 ['P0.5Ca0.2Ti0.3', '-1.495', '-0.209031916554']
CaPXe 3 ['P0.75Ca0.125Xe0.125', '-0.626', '-0.21564423789']
CaPaPt 2 ['Ca0.125Pt0.625Pa0.25', '-1.13', '-0.218208363958']
CaPaS 3 ['S0.666667Ca0.166667Pa0.166667', '-1.673', '-0.220546433743']
CaPaSe 5 ['Ca0.1Se0.7Pa0.2', '-1.111', '-0.220664132']
CaPaSi 2 ['Si0.666667Ca0.111111Pa0.222222', '-0.509', '-0.242188710556']
CaPdPm 1 ['Ca0.1Pd0.7Pm0.2', '-0.753', '-0.2180275105']
CaPdSn 1 ['Ca0.111111Pd0.333333Sn0.555556', '-0.803', '-0.203315203832']
CaPmS 6 ['S0.428571Ca0.142857Pm0.428571', '-1.945', '-0.215223361849']
CaPmSe 6 ['Ca0.2Se0.6Pm0.2', '-1.678', '-0.2121564675']
CaPmTe 1 ['Ca0.1Te0.6Pm0.3', '-1.345', '-0.229834340914']
CaPrPt 2 ['Ca0.1Pr0.5Pt0.4', '-1.109', '-0.2030184264']
CaPtSi 4 ['Si0.428571Ca0.142857Pt0.428571', '-1.008', '-0.201664853572']

CaPtSn 4 ['Ca0.111111Sn0.555556Pt0.333333', '-0.818', '-0.207559136194']
CaPtTh 3 ['Ca0.111111Pt0.777778Th0.111111', '-0.846', '-0.218492870556']
CaPuS 11 ['S0.444444Ca0.111111Pu0.444444', '-2.038', '-0.244699567102']
CaRbSe 2 ['Ca0.4Se0.4Rb0.2', '-1.835', '-0.200097096']
CaRhS 3 ['S0.444444Ca0.111111Rh0.444444', '-1.101', '-0.208816144604']
CaRhSi 15 ['Si0.6Ca0.1Rh0.3', '-0.855', '-0.23650471775']
CaRhSn 5 ['Ca0.142857Rh0.285714Sn0.571429', '-0.807', '-0.201060676085']
CaRhTa 2 ['Ca0.1Rh0.7Ta0.2', '-0.763', '-0.2092823485']
CaRuSi 1 ['Si0.5Ca0.125Ru0.375', '-0.828', '-0.211122635625']
CaSSb 7 ['S0.3Ca0.5Sb0.2', '-1.965', '-0.210155847877']
CaSSe 4 ['S0.4Ca0.2Se0.4', '-1.127', '-0.201592037196']
CaSSi 10 ['Si0.142857S0.428571Ca0.428571', '-2.198', '-0.214982936849']
CaSSn 1 ['S0.428571Ca0.285714Sn0.285714', '-1.703', '-0.200033067299']
CaSTc 4 ['S0.5Ca0.125Tc0.375', '-1.233', '-0.200617414243']
CaSTe 2 ['S0.2Ca0.4Te0.4', '-1.8', '-0.210329476528']
CaSTh 1 ['S0.5Ca0.1Th0.4', '-2.274', '-0.21659931699']
CaSZr 4 ['S0.555556Ca0.111111Zr0.333333', '-1.987', '-0.243345548878']
CaSbSe 23 ['Ca0.444444Se0.444444Sb0.111111', '-2.031', '-0.214441217778']
CaScSe 5 ['Ca0.222222Sc0.222222Se0.555556', '-1.849', '-0.232270941111']
CaSeSi 13 ['Si0.25Ca0.375Se0.375', '-1.748', '-0.2152785275']
CaSeSn 3 ['Ca0.2Se0.4Sn0.4', '-1.215', '-0.20452531363']
CaSeTc 2 ['Ca0.1Se0.6Tc0.3', '-0.626', '-0.217274274']
CaSeTm 1 ['Ca0.1Se0.6Tm0.3', '-1.851', '-0.203517471002']
CaSeY 7 ['Ca0.111111Se0.666667Y0.222222', '-1.454', '-0.215015314444']
CdCeCl 1 ['Cl0.4Cd0.2Ce0.4', '-1.733', '-0.223612513252']
CdCeI 20 ['Cd0.222222I0.666667Ce0.111111', '-0.991', '-0.201863167435']
CdCeO 5 ['O0.625Cd0.25Ce0.125', '-1.962', '-0.243793136724']
CdClCu 6 ['Cl0.5Cu0.4Cd0.1', '-0.953', '-0.209911119191']
CdClEr 38 ['Cl0.3Cd0.3Er0.4', '-1.16', '-0.257146470314']
CdClEu 4 ['Cl0.6Cd0.1Eu0.3', '-2.74', '-0.200612510878']
CdClF 6 ['F0.8Cl0.1Cd0.1', '-1.322', '-0.202619356873']
CdClGd 3 ['Cl0.5Cd0.166667Gd0.333333', '-2.005', '-0.209878113023']
CdClHf 1 ['Cl0.555556Cd0.222222Hf0.222222', '-1.733', '-0.249465925347']
CdClHo 38 ['Cl0.333333Cd0.333333Ho0.333333', '-1.177', '-0.235768456062']
CdClIr 5 ['Cl0.75Cd0.125Ir0.125', '-0.699', '-0.200063133596']
CdClLa 1 ['Cl0.666667Cd0.111111La0.222222', '-2.762', '-0.375916216258']
CdClLu 50 ['Cl0.25Cd0.375Lu0.375', '-0.945', '-0.206787707345']
CdClNd 1 ['Cl0.444444Cd0.222222Nd0.333333', '-1.864', '-0.20760540769']
CdClNp 10 ['Cl0.666667Cd0.222222Np0.111111', '-1.758', '-0.205377355284']
CdClPm 31 ['Cl0.571429Cd0.285714Pm0.142857', '-1.718', '-0.224768593575']
CdClPr 4 ['Cl0.444444Cd0.222222Pr0.333333', '-1.869', '-0.205907245002']
CdClPt 2 ['Cl0.5Cd0.333333Pt0.166667', '-1.264', '-0.212162247193']
CdClPu 30 ['Cl0.8Cd0.1Pu0.1', '-1.471', '-0.201683736693']
CdClRh 12 ['Cl0.6Rh0.2Cd0.2', '-1.005', '-0.206701013754']

CdClSi 14 ['Si0.166667Cl0.5Cd0.333333', '-1.202', '-0.204126267192']
CdClSm 21 ['Cl0.6Cd0.1Sm0.3', '-2.08', '-0.207255384631']
CdClTa 7 ['Cl0.625Cd0.25Ta0.125', '-1.458', '-0.212815297427']
CdClTb 1 ['Cl0.7Cd0.1Tb0.2', '-2.354', '-0.253886820135']
CdClU 1 ['Cl0.666667Cd0.166667U0.166667', '-1.853', '-0.205019164583']
CdClXe 2 ['Cl0.6Cd0.1Xe0.3', '-0.619', '-0.219850506877']
CdClY 2 ['Cl0.444444Y0.333333Cd0.222222', '-1.866', '-0.207431420653']
CdClZr 2 ['Cl0.625Zr0.25Cd0.125', '-1.907', '-0.201007015865']
CdCoF 5 ['F0.666667Co0.222222Cd0.111111', '-2.18', '-0.207571237124']
CdCoN 22 ['N0.5Co0.2Cd0.3', '-0.209', '-0.205146988966']
CdCoO 4 ['O0.6Co0.3Cd0.1', '-1.391', '-0.203608297478']
CdCrN 15 ['N0.5Cr0.25Cd0.25', '-0.494', '-0.210368068664']
CdCrO 5 ['O0.625Cr0.25Cd0.125', '-2.162', '-0.212437141199']
CdCsF 9 ['F0.625Cd0.125Cs0.25', '-2.656', '-0.25655002205']
CdCsO 2 ['O0.333333Cd0.166667Cs0.5', '-1.244', '-0.201863115198']
CdCsSe 7 ['Se0.375Cd0.25Cs0.375', '-1.008', '-0.20458543338']
CdCuF 4 ['F0.714286Cu0.142857Cd0.142857', '-2.04', '-0.245951825499']
CdCuO 2 ['O0.625Cu0.125Cd0.25', '-1.127', '-0.307458865709']
CdDyF 4 ['F0.7Cd0.1Dy0.2', '-3.896', '-0.225123229279']
CdDyI 16 ['Cd0.25I0.5Dy0.25', '-0.953', '-0.208440351258']
CdDyO 1 ['O0.6Cd0.1Dy0.3', '-3.454', '-0.319239903707']
CdErF 3 ['F0.777778Cd0.111111Er0.111111', '-2.998', '-0.205309624096']
CdErI 20 ['Cd0.2I0.5Er0.3', '-1.052', '-0.22449164621']
CdErN 1 ['N0.333333Cd0.111111Er0.555556', '-1.544', '-0.214645521553']
CdErO 1 ['O0.6Cd0.1Er0.3', '-3.4', '-0.230281760515']
CdErP 1 ['P0.5Cd0.125Er0.375', '-1.567', '-0.207977773176']
CdEuF 2 ['F0.777778Cd0.111111Eu0.111111', '-2.551', '-0.21028431483']
CdEuI 1 ['Cd0.166667I0.5Eu0.333333', '-1.773', '-0.263300701282']
CdEuN 19 ['N0.222222Cd0.222222Eu0.555556', '-0.676', '-0.209495783257']
CdEuO 6 ['O0.666667Cd0.222222Eu0.111111', '-1.517', '-0.212813305818']
CdEuP 3 ['P0.333333Cd0.111111Eu0.555556', '-1.051', '-0.201077290071']
CdEuSe 2 ['Se0.4Cd0.1Eu0.5', '-2.079', '-0.219243718']
CdFFe 3 ['F0.75Fe0.125Cd0.125', '-2.392', '-0.274754414292']
CdFGa 1 ['F0.8Ga0.1Cd0.1', '-2.183', '-0.297378794934']
CdFGd 3 ['F0.8Cd0.1Gd0.1', '-2.732', '-0.333139355686']
CdFGe 1 ['F0.714286Ge0.142857Cd0.142857', '-2.699', '-0.229941949729']
CdFH 19 ['H0.1F0.7Cd0.2', '-2.002', '-0.203669642716']
CdFHf 1 ['F0.8Cd0.1Hf0.1', '-3.006', '-0.221346049024']
CdFHg 4 ['F0.714286Cd0.142857Hg0.142857', '-1.981', '-0.219114715501']
CdFHo 1 ['F0.777778Cd0.111111Ho0.111111', '-3.016', '-0.223045504558']
CdFIn 3 ['F0.75Cd0.125In0.125', '-2.516', '-0.206313974918']
CdFK 9 ['F0.666667K0.166667Cd0.166667', '-2.483', '-0.243049139183']
CdFKr 4 ['F0.75Kr0.125Cd0.125', '-1.28', '-0.24213698356']
CdFLa 3 ['F0.777778Cd0.111111La0.111111', '-3.077', '-0.267639794558']

CdFLi 5 ['Li0.2F0.7Cd0.1', '-2.204', '-0.207318536346']
CdFLu 6 ['F0.666667Cd0.111111Lu0.222222', '-3.857', '-0.260347188356']
CdFMg 3 ['F0.75Mg0.125Cd0.125', '-2.56', '-0.205396411788']
CdFMn 3 ['F0.777778Mn0.111111Cd0.111111', '-2.415', '-0.209801730854']
CdFMo 1 ['F0.75Mo0.125Cd0.125', '-2.738', '-0.212720470443']
CdFN 10 ['N0.1F0.4Cd0.5', '-1.697', '-0.223538480348']
CdFNa 8 ['F0.666667Na0.222222Cd0.111111', '-2.382', '-0.241262240752']
CdFNb 1 ['F0.8Nb0.1Cd0.1', '-2.893', '-0.203199667606']
CdFNd 1 ['F0.75Cd0.125Nd0.125', '-3.417', '-0.300095585958']
CdFNi 4 ['F0.666667Ni0.166667Cd0.166667', '-2.457', '-0.26656118058']
CdFNp 2 ['F0.777778Cd0.111111Np0.111111', '-3.328', '-0.413555987199']
CdFO 13 ['O0.2F0.3Cd0.5', '-1.811', '-0.200881079834']
CdFP 2 ['F0.666667P0.111111Cd0.222222', '-2.645', '-0.234673198093']
CdFPa 8 ['F0.666667Cd0.222222Pa0.111111', '-3.069', '-0.242281877246']
CdFPb 1 ['F0.8Cd0.1Pb0.1', '-2.077', '-0.276801337524']
CdFPd 2 ['F0.777778Pd0.111111Cd0.111111', '-1.891', '-0.218251577244']
CdFPm 2 ['F0.777778Cd0.111111Pm0.111111', '-2.878', '-0.206405060207']
CdFPr 3 ['F0.75Cd0.125Pr0.125', '-3.336', '-0.223254416482']
CdFPt 1 ['F0.8Cd0.1Pt0.1', '-1.858', '-0.205539647942']
CdFPu 6 ['F0.7Cd0.1Pu0.2', '-3.719', '-0.228302009607']
CdFRb 10 ['F0.7Rb0.1Cd0.2', '-2.301', '-0.224166666434']
CdFRh 1 ['F0.8Rh0.1Cd0.1', '-1.891', '-0.27888992652']
CdFRu 2 ['F0.777778Ru0.111111Cd0.111111', '-2.134', '-0.25603106836']
CdFSb 6 ['F0.571429Cd0.285714Sb0.142857', '-2.33', '-0.225054971926']
CdFSc 3 ['F0.777778Sc0.111111Cd0.111111', '-2.993', '-0.276706439927']
CdFSe 1 ['F0.8Se0.1Cd0.1', '-1.966', '-0.246062947524']
CdFSi 2 ['F0.6Si0.1Cd0.3', '-2.61', '-0.200763241024']
CdFSn 1 ['F0.8Cd0.1Sn0.1', '-2.272', '-0.205523270834']
CdFSr 5 ['F0.625Sr0.25Cd0.125', '-3.816', '-0.201052533041']
CdFTc 2 ['F0.777778Tc0.111111Cd0.111111', '-2.459', '-0.39890223461']
CdFTe 1 ['F0.666667Cd0.222222Te0.111111', '-2.546', '-0.251593482062']
CdFTh 2 ['F0.8Cd0.1Th0.1', '-3.294', '-0.327082623689']
CdFTi 1 ['F0.8Ti0.1Cd0.1', '-2.783', '-0.240606855144']
CdFTl 7 ['F0.666667Cd0.111111Tl0.222222', '-2.363', '-0.204291643911']
CdFTm 44 ['F0.375Cd0.125Tm0.5', '-2.018', '-0.210190232828']
CdFV 3 ['F0.7V0.2Cd0.1', '-3.065', '-0.215562298607']
CdFY 3 ['F0.75Y0.125Cd0.125', '-3.326', '-0.306029247833']
CdFYb 37 ['F0.3Cd0.3Yb0.4', '-1.877', '-0.209178655476']
CdFZn 3 ['F0.75Zn0.125Cd0.125', '-2.117', '-0.233418817936']
CdFZr 1 ['F0.8Zr0.1Cd0.1', '-2.936', '-0.218788444399']
CdFeO 5 ['O0.625Fe0.25Cd0.125', '-1.607', '-0.215492191207']
CdGaO 4 ['O0.6Ga0.1Cd0.3', '-1.482', '-0.204722055603']
CdGdI 21 ['Cd0.3I0.6Gd0.1', '-0.938', '-0.205209083926']
CdGdO 2 ['O0.625Cd0.125Gd0.25', '-2.89', '-0.210563581434']

CdGeO 2 ['O0.6Ge0.2Cd0.2', '-1.989', '-0.28770370539']
CdHN 5 ['H0.333333N0.333333Cd0.333333', '-0.391', '-0.224659259789']
CdHPm 5 ['H0.625Cd0.125Pm0.25', '-0.561', '-0.223860194677']
CdHfO 2 ['O0.625Cd0.25Hf0.125', '-2.328', '-0.270230867684']
CdHfS 11 ['S0.333333Cd0.111111Hf0.555556', '-1.276', '-0.202256291999']
CdHfSe 6 ['Se0.5Cd0.3Hf0.2', '-1.136', '-0.201262728']
CdHoI 17 ['Cd0.25I0.625Ho0.125', '-0.963', '-0.210852747649']
CdHoO 2 ['O0.625Cd0.125Ho0.25', '-3.065', '-0.334620941071']
CdHoOs 2 ['Cd0.111111Ho0.555556Os0.333333', '-0.316', '-0.20261001963']
CdHoS 1 ['S0.5Cd0.125Ho0.375', '-2.154', '-0.218759298201']
CdILu 25 ['Cd0.333333I0.5Lu0.166667', '-0.836', '-0.238468295483']
CdINd 3 ['Cd0.142857I0.714286Nd0.142857', '-1.173', '-0.263569413452']
CdINi 3 ['Ni0.333333Cd0.166667I0.5', '-0.607', '-0.201512606101']
CdIPa 14 ['Cd0.111111I0.555556Pa0.333333', '-0.566', '-0.239672902972']
CdIPm 31 ['Cd0.2I0.4Pm0.4', '-0.917', '-0.201301202029']
CdIPt 3 ['Cd0.111111I0.666667Pt0.222222', '-0.622', '-0.210865414924']
CdIPu 5 ['Cd0.125I0.625Pu0.25', '-1.213', '-0.204249383483']
CdIRh 7 ['Rh0.2Cd0.2I0.6', '-0.627', '-0.206025684806']
CdISm 3 ['Cd0.142857I0.714286Sm0.142857', '-1.052', '-0.205787744178']
CdITb 22 ['Cd0.25I0.5Tb0.25', '-0.729', '-0.202782106008']
CdITm 2 ['Cd0.111111I0.666667Tm0.222222', '-1.336', '-0.267451514921']
CdIXe 2 ['Cd0.111111I0.444444Xe0.444444', '-0.456', '-0.222125380448']
CdIYb 1 ['Cd0.125I0.625Yb0.25', '-1.736', '-0.270689220287']
CdIrN 2 ['N0.444444Cd0.333333Ir0.222222', '-0.228', '-0.228']
CdIrO 4 ['O0.666667Cd0.166667Ir0.166667', '-1.178', '-0.233780522892']
CdKO 1 ['O0.666667K0.166667Cd0.166667', '-1.2', '-0.200594765247']
CdKTe 1 ['K0.4Cd0.2Te0.4', '-1.056', '-0.208426699667']
CdKrO 1 ['O0.6Kr0.2Cd0.2', '-0.751', '-0.256529386824']
CdLaN 2 ['N0.375Cd0.125La0.5', '-1.363', '-0.209899501747']
CdLaO 2 ['O0.625Cd0.25La0.125', '-2.069', '-0.290743565317']
CdLuN 1 ['N0.4Cd0.1Lu0.5', '-1.854', '-0.205910744364']
CdLuO 1 ['O0.625Cd0.125Lu0.25', '-3.025', '-0.239934387684']
CdMnN 20 ['N0.285714Mn0.428571Cd0.285714', '-0.46', '-0.220565592919']
CdMnO 3 ['O0.625Mn0.125Cd0.25', '-1.553', '-0.234865510531']
CdMoN 12 ['N0.428571Mo0.285714Cd0.285714', '-0.47', '-0.200941582045']
CdMoO 2 ['O0.6Mo0.1Cd0.3', '-1.875', '-0.203699833472']
CdNNa 1 ['N0.3Na0.5Cd0.2', '-0.416', '-0.251937419141']
CdNNi 1 ['N0.375Ni0.25Cd0.375', '-0.286', '-0.238070311221']
CdNNp 22 ['N0.375Cd0.25Np0.375', '-1.114', '-0.204657090963']
CdNO 8 ['N0.25O0.625Cd0.125', '-0.94', '-0.25101319249']
CdNPa 29 ['N0.571429Cd0.285714Pa0.142857', '-0.502', '-0.219730231157']
CdNPu 16 ['N0.555556Cd0.222222Pu0.222222', '-0.826', '-0.21750132548']
CdNRe 1 ['N0.444444Cd0.333333Re0.222222', '-0.262', '-0.236172468863']
CdNTc 13 ['N0.444444Tc0.333333Cd0.222222', '-0.289', '-0.201776565776']

CdNTi 11 ['N0.4Ti0.5Cd0.1', '-1.498', '-0.200963177992']
CdNU 1 ['N0.5Cd0.1U0.4', '-1.526', '-0.203311457327']
CdNV 1 ['N0.5V0.25Cd0.25', '-0.833', '-0.206547063665']
CdNW 8 ['N0.555556Cd0.222222W0.222222', '-0.283', '-0.201726279157']
CdNYb 3 ['N0.375Cd0.125Yb0.5', '-1.114', '-0.241177698217']
CdNaP 1 ['Na0.222222P0.333333Cd0.444444', '-0.509', '-0.207360229741']
CdNbO 1 ['O0.625Nb0.125Cd0.25', '-2.112', '-0.226836887942']
CdNbRh 1 ['Nb0.166667Rh0.666667Cd0.166667', '-0.625', '-0.254809975556']
CdNbSe 1 ['Se0.5Nb0.25Cd0.25', '-0.932', '-0.21029886375']
CdNdO 3 ['O0.625Cd0.125Nd0.25', '-2.855', '-0.204075720077']
CdNiO 1 ['O0.6Ni0.2Cd0.2', '-1.319', '-0.32792416836']
CdNpO 1 ['O0.666667Cd0.111111Np0.222222', '-3.044', '-0.295688330458']
CdNpP 1 ['P0.444444Cd0.222222Np0.333333', '-0.919', '-0.210779256573']
CdNpS 4 ['S0.428571Cd0.285714Np0.285714', '-1.381', '-0.205839338279']
CdOOs 2 ['O0.6Cd0.2Os0.2', '-1.459', '-0.217274036471']
CdOPa 8 ['O0.666667Cd0.222222Pa0.111111', '-1.887', '-0.297428689608']
CdOPd 1 ['O0.625Pd0.125Cd0.25', '-1.079', '-0.212988748311']
CdORe 3 ['O0.625Cd0.25Re0.125', '-1.808', '-0.23952691445']
CdORh 1 ['O0.6Rh0.2Cd0.2', '-1.341', '-0.223848764474']
CdORu 2 ['O0.6Ru0.1Cd0.3', '-1.461', '-0.288762741723']
CdOSb 2 ['O0.6Cd0.3Sb0.1', '-1.625', '-0.239403696517']
CdOSi 4 ['O0.6Si0.2Cd0.2', '-2.609', '-0.22931938847']
CdOSn 3 ['O0.6Cd0.3Sn0.1', '-1.582', '-0.230174420878']
CdOSr 1 ['O0.625Sr0.125Cd0.25', '-1.69', '-0.262155505809']
CdOTa 1 ['O0.6Cd0.3Ta0.1', '-2.11', '-0.229782370269']
CdOTc 3 ['O0.666667Tc0.222222Cd0.111111', '-1.784', '-0.218124645618']
CdOTe 4 ['O0.666667Cd0.111111Te0.222222', '-1.488', '-0.216215033579']
CdOTi 2 ['O0.25Ti0.625Cd0.125', '-1.732', '-0.212165238977']
CdOV 1 ['O0.6V0.2Cd0.2', '-2.237', '-0.20351268442']
CdOW 1 ['O0.6Cd0.2W0.2', '-2.199', '-0.242382996472']
CdOZr 2 ['O0.625Zr0.25Cd0.125', '-3.328', '-0.294219043822']
CdOsSc 3 ['Sc0.666667Cd0.111111Os0.222222', '-0.417', '-0.207852771667']
CdPPm 13 ['P0.333333Cd0.222222Pm0.444444', '-1.07', '-0.202612398124']
CdPPu 8 ['P0.555556Cd0.222222Pu0.222222', '-0.922', '-0.200608288772']
CdPSc 2 ['P0.333333Sc0.5Cd0.166667', '-1.388', '-0.207801094513']
CdPTa 14 ['P0.428571Cd0.428571Ta0.142857', '-0.539', '-0.204117427499']
CdPTi 1 ['P0.25Ti0.625Cd0.125', '-1.135', '-0.220462126683']
CdPYb 3 ['P0.444444Cd0.333333Yb0.222222', '-0.747', '-0.200348218518']
CdPaRh 2 ['Rh0.6Cd0.1Pa0.3', '-0.953', '-0.233240498']
CdPaS 6 ['S0.6Cd0.2Pa0.2', '-1.339', '-0.205446788492']
CdPaSe 6 ['Se0.555556Cd0.111111Pa0.333333', '-1.168', '-0.224289251111']
CdPdPm 9 ['Pd0.777778Cd0.111111Pm0.111111', '-0.467', '-0.200761399722']
CdPmS 10 ['S0.444444Cd0.222222Pm0.333333', '-1.871', '-0.216469341085']
CdPmSe 18 ['Se0.4Cd0.2Pm0.4', '-1.501', '-0.204215839']

CdPrPt 3 ['Cd0.222222Pr0.444444Pt0.333333', '-1.083', '-0.218607203333']
CdPrSe 1 ['Se0.5Cd0.166667Pr0.333333', '-1.782', '-0.258189172498']
CdPtS 10 ['S0.4Cd0.2Pt0.4', '-0.76', '-0.202688432894']
CdPtTh 10 ['Cd0.111111Pt0.666667Th0.222222', '-0.825', '-0.210234814444']
CdPuS 8 ['S0.428571Cd0.142857Pu0.428571', '-1.846', '-0.201333702563']
CdPuSe 7 ['Se0.375Cd0.25Pu0.375', '-1.36', '-0.20052424375']
CdPuSn 2 ['Cd0.285714Sn0.285714Pu0.428571', '-0.454', '-0.227176753042']
CdRhS 1 ['S0.4Rh0.4Cd0.2', '-0.771', '-0.228142112894']
CdRhTa 1 ['Rh0.666667Cd0.111111Ta0.222222', '-0.746', '-0.201177557778']
CdRhTh 2 ['Rh0.666667Cd0.111111Th0.222222', '-0.86', '-0.213661426667']
CdSTa 1 ['S0.5Cd0.25Ta0.25', '-1.142', '-0.210703357992']
CdSTc 2 ['S0.5Tc0.375Cd0.125', '-0.864', '-0.212486831743']
CdSTh 3 ['S0.5Cd0.125Th0.375', '-2.048', '-0.216777291741']
CdSXe 3 ['S0.5Cd0.166667Xe0.333333', '-0.466', '-0.202652587664']
CdSZr 14 ['S0.4Zr0.3Cd0.3', '-1.313', '-0.206414975392']
CdSeTc 6 ['Se0.5Tc0.375Cd0.125', '-0.37', '-0.21134442375']
CdSeV 1 ['V0.333333Se0.5Cd0.166667', '-0.835', '-0.202985711667']
CdSeY 1 ['Se0.5Y0.375Cd0.125', '-1.773', '-0.220079745626']
CdSeZr 7 ['Se0.444444Zr0.444444Cd0.111111', '-1.209', '-0.200389848765']
CeClCu 1 ['Cl0.6Cu0.3Ce0.1', '-1.606', '-0.227475466378']
CeClEr 6 ['Cl0.666667Ce0.111111Er0.222222', '-2.049', '-0.310838552156']
CeClGa 13 ['Cl0.428571Ga0.285714Ce0.285714', '-1.982', '-0.208741064734']
CeClGe 10 ['Cl0.222222Ge0.222222Ce0.555556', '-1.364', '-0.206608654677']
CeClH 6 ['H0.5Cl0.1Ce0.4', '-0.997', '-0.206500776721']
CeClHo 6 ['Cl0.666667Ce0.111111Ho0.222222', '-2.113', '-0.361785922524']
CeClI 19 ['Cl0.166667I0.666667Ce0.166667', '-1.129', '-0.232842881019']
CeClIn 30 ['Cl0.5In0.25Ce0.25', '-2.088', '-0.202964961982']
CeClIr 5 ['Cl0.4Ce0.4Ir0.2', '-1.829', '-0.206587000691']
CeClLu 6 ['Cl0.666667Ce0.111111Lu0.222222', '-2.177', '-0.475975835489']
CeClMo 1 ['Cl0.8Mo0.1Ce0.1', '-1.904', '-0.217078127756']
CeClNi 1 ['Cl0.75Ni0.125Ce0.125', '-1.826', '-0.219562474925']
CeClNp 1 ['Cl0.8Ce0.1Np0.1', '-2.254', '-0.283268398439']
CeClO 15 ['O0.6Cl0.2Ce0.2', '-2.171', '-0.208912613198']
CeClOs 6 ['Cl0.25Ce0.5Os0.25', '-1.09', '-0.204484793664']
CeClP 3 ['P0.333333Cl0.111111Ce0.555556', '-1.666', '-0.2039380575']
CeClPd 4 ['Cl0.285714Pd0.285714Ce0.428571', '-1.64', '-0.215462855537']
CeClPm 1 ['Cl0.777778Ce0.111111Pm0.111111', '-2.316', '-0.226808462364']
CeClPt 24 ['Cl0.3Ce0.3Pt0.4', '-1.838', '-0.205341527564']
CeClPu 2 ['Cl0.777778Ce0.111111Pu0.111111', '-2.322', '-0.205608982089']
CeClRe 1 ['Cl0.8Ce0.1Re0.1', '-1.685', '-0.221539562817']
CeClRh 10 ['Cl0.625Rh0.25Ce0.125', '-1.494', '-0.20076856758']
CeClRu 3 ['Cl0.428571Ru0.142857Ce0.428571', '-1.736', '-0.205291522847']
CeClS 1 ['S0.1Cl0.6Ce0.3', '-3.033', '-0.532518930976']
CeClSb 2 ['Cl0.4Sb0.3Ce0.3', '-2.02', '-0.22081499025']

CeClSe 24 ['Cl0.428571Se0.285714Ce0.285714', '-2.383', '-0.200113154376']
CeClSi 25 ['Si0.125Cl0.75Ce0.125', '-2.21', '-0.200963247502']
CeClSm 2 ['Cl0.8Ce0.1Sm0.1', '-2.065', '-0.262871295628']
CeClTc 2 ['Cl0.714286Tc0.142857Ce0.142857', '-2.07', '-0.219230193724']
CeClTe 9 ['Cl0.444444Te0.333333Ce0.222222', '-2.037', '-0.205513912133']
CeClTl 8 ['Cl0.285714Ce0.428571Tl0.285714', '-1.42', '-0.201654982561']
CeCoF 1 ['F0.8Co0.1Ce0.1', '-2.962', '-0.434378015772']
CeCoH 24 ['H0.75Co0.125Ce0.125', '-0.451', '-0.207490859493']
CeCoI 29 ['Co0.111111I0.444444Ce0.444444', '-1.093', '-0.208456202123']
CeCoN 9 ['N0.4Co0.4Ce0.2', '-0.738', '-0.205739971363']
CeCoO 3 ['O0.6Co0.2Ce0.2', '-2.626', '-0.20323828609']
CeCrI 11 ['Cr0.25I0.625Ce0.125', '-0.841', '-0.209757437544']
CeCrN 4 ['N0.5Cr0.4Ce0.1', '-0.94', '-0.223911906578']
CeCrO 3 ['O0.625Cr0.125Ce0.25', '-3.253', '-0.226514584206']
CeCsF 7 ['F0.666667Cs0.222222Ce0.111111', '-3.467', '-0.247078077338']
CeCsI 13 ['I0.8Cs0.1Ce0.1', '-0.958', '-0.260134481914']
CeCsO 2 ['O0.6Cs0.2Ce0.2', '-2.53', '-0.206077317847']
CeCsSe 2 ['Se0.5Cs0.25Ce0.25', '-1.719', '-0.208956366029']
CeCsTe 2 ['Te0.6Cs0.1Ce0.3', '-1.493', '-0.212005570122']
CeCuF 1 ['F0.8Cu0.1Ce0.1', '-2.54', '-0.272116398519']
CeCuI 22 ['Cu0.111111I0.555556Ce0.333333', '-1.208', '-0.21650236226']
CeCuO 3 ['O0.625Cu0.25Ce0.125', '-1.841', '-0.291887235392']
CeCuP 1 ['P0.375Cu0.375Ce0.25', '-1.116', '-0.220480958515']
CeDyF 2 ['F0.777778Ce0.111111Dy0.111111', '-4.045', '-0.267181850489']
CeDyI 10 ['I0.625Ce0.125Dy0.25', '-1.171', '-0.213624409779']
CeDyO 3 ['O0.625Ce0.125Dy0.25', '-3.732', '-0.225610745106']
CeErF 1 ['F0.8Ce0.1Er0.1', '-3.862', '-0.336528782356']
CeErI 11 ['I0.625Ce0.125Er0.25', '-1.149', '-0.210293658475']
CeErO 3 ['O0.6Ce0.2Er0.2', '-4.001', '-0.292488289845']
CeEuF 1 ['F0.8Ce0.1Eu0.1', '-3.342', '-0.223306004017']
CeEuI 4 ['I0.666667Ce0.222222Eu0.111111', '-1.485', '-0.219444026981']
CeEuN 1 ['N0.4Ce0.1Eu0.5', '-0.853', '-0.243688602113']
CeEuO 3 ['O0.6Ce0.2Eu0.2', '-3.337', '-0.218215421934']
CeEuP 2 ['P0.5Ce0.2Eu0.3', '-1.526', '-0.207988971772']
CeFFe 2 ['F0.777778Fe0.111111Ce0.111111', '-3.275', '-0.268504057893']
CeFGa 2 ['F0.777778Ga0.111111Ce0.111111', '-3.425', '-0.205365461782']
CeFGd 2 ['F0.777778Ce0.111111Gd0.111111', '-4.052', '-0.262099418173']
CeFGe 4 ['F0.166667Ge0.333333Ce0.5', '-1.76', '-0.222709816164']
CeFH 15 ['H0.444444F0.444444Ce0.111111', '-2.508', '-0.203945025791']
CeFHf 2 ['F0.75Ce0.125Hf0.125', '-4.325', '-0.219096209352']
CeFHo 1 ['F0.777778Ce0.111111Ho0.111111', '-4.224', '-0.306545638636']
CeFI 10 ['F0.2I0.5Ce0.3', '-2.063', '-0.222232294294']
CeFIn 7 ['F0.333333In0.333333Ce0.333333', '-2.423', '-0.206324222049']
CeFK 9 ['F0.571429K0.285714Ce0.142857', '-3.595', '-0.2203141105']

CeFKr 1 ['F0.8Kr0.1Ce0.1', '-2.059', '-0.216659707518']
CeFLi 2 ['Li0.125F0.75Ce0.125', '-3.184', '-0.210555011163']
CeFLu 2 ['F0.777778Ce0.111111Lu0.111111', '-4.078', '-0.336583995116']
CeFMg 2 ['F0.777778Mg0.111111Ce0.111111', '-3.517', '-0.299519166778']
CeFN 1 ['N0.1F0.8Ce0.1', '-1.992', '-0.243219360844']
CeFNa 7 ['F0.6Na0.2Ce0.2', '-3.724', '-0.202694005129']
CeFNd 1 ['F0.8Ce0.1Nd0.1', '-3.786', '-0.280426589436']
CeFNi 2 ['F0.777778Ni0.111111Ce0.111111', '-2.997', '-0.247075275116']
CeFNp 1 ['F0.8Ce0.1Np0.1', '-4.147', '-0.511950509149']
CeFO 3 ['O0.111111F0.777778Ce0.111111', '-2.187', '-0.243910400938']
CeFP 1 ['F0.714286P0.142857Ce0.142857', '-3.72', '-0.243598190083']
CeFPa 3 ['F0.75Ce0.125Pa0.125', '-4.227', '-0.24233648763']
CeFPd 1 ['F0.8Pd0.1Ce0.1', '-2.667', '-0.208114635194']
CeFPm 1 ['F0.8Ce0.1Pm0.1', '-3.654', '-0.237514674856']
CeFPr 1 ['F0.777778Ce0.111111Pr0.111111', '-4.151', '-0.259615170951']
CeFPu 2 ['F0.777778Ce0.111111Pu0.111111', '-4.242', '-0.564281172893']
CeFRb 7 ['F0.6Rb0.3Ce0.1', '-3.667', '-0.203790368102']
CeFRe 1 ['F0.8Ce0.1Re0.1', '-3.239', '-0.200618635564']
CeFRh 2 ['F0.777778Rh0.111111Ce0.111111', '-2.958', '-0.217409156782']
CeFRu 1 ['F0.8Ru0.1Ce0.1', '-3.082', '-0.379778082194']
CeFS 1 ['F0.5S0.2Ce0.3', '-3.896', '-0.211282709718']
CeFSb 1 ['F0.8Sb0.1Ce0.1', '-3.301', '-0.374773473896']
CeFSc 2 ['F0.777778Sc0.111111Ce0.111111', '-4.067', '-0.226206574004']
CeFSe 2 ['F0.4Se0.3Ce0.3', '-3.365', '-0.211765990847']
CeFSi 19 ['F0.25Si0.375Ce0.375', '-2.114', '-0.208433545287']
CeFSn 1 ['F0.8Sn0.1Ce0.1', '-3.342', '-0.263473391504']
CeFTc 1 ['F0.8Tc0.1Ce0.1', '-3.341', '-0.474862131819']
CeFTh 1 ['F0.8Ce0.1Th0.1', '-4.304', '-0.325032744359']
CeFTi 2 ['F0.777778Ti0.111111Ce0.111111', '-3.979', '-0.432187660809']
CeFTm 6 ['F0.666667Ce0.111111Tm0.222222', '-3.553', '-0.641402214462']
CeFU 1 ['F0.777778Ce0.111111U0.111111', '-4.246', '-0.209981228483']
CeFV 1 ['F0.8V0.1Ce0.1', '-3.602', '-0.354469592694']
CeFW 1 ['F0.75Ce0.125W0.125', '-3.667', '-0.20190186107']
CeFY 2 ['F0.777778Y0.111111Ce0.111111', '-4.095', '-0.286081687707']
CeFYb 6 ['F0.8Ce0.1Yb0.1', '-3.256', '-0.231213565104']
CeFZn 2 ['F0.777778Zn0.111111Ce0.111111', '-3.009', '-0.210205750021']
CeFeI 19 ['Fe0.3I0.5Ce0.2', '-0.845', '-0.202441515284']
CeFeO 5 ['O0.666667Fe0.111111Ce0.222222', '-2.669', '-0.208369234944']
CeFeSi 5 ['Si0.6Fe0.3Ce0.1', '-0.753', '-0.235311132']
CeGaI 41 ['Ga0.2I0.7Ce0.1', '-0.95', '-0.200290296633']
CeGaIr 4 ['Ga0.571429Ce0.142857Ir0.285714', '-0.745', '-0.205242764087']
CeGaO 4 ['O0.25Ga0.25Ce0.5', '-1.968', '-0.215972304883']
CeGaPd 1 ['Ga0.5Pd0.4Ce0.1', '-0.924', '-0.21561214425']
CeGaPt 4 ['Ga0.571429Ce0.142857Pt0.285714', '-0.964', '-0.217300786786']

CeGaRh 5 ['Ga0.444444Rh0.444444Ce0.111111', '-0.92', '-0.201741513333']
CeGaSe 1 ['Ga0.166667Se0.333333Ce0.5', '-1.627', '-0.203800967917']
CeGdI 10 ['I0.625Ce0.125Gd0.25', '-1.274', '-0.276708248319']
CeGdO 2 ['O0.625Ce0.25Gd0.125', '-3.677', '-0.291566663722']
CeGeH 16 ['H0.777778Ge0.111111Ce0.111111', '-0.423', '-0.206547430661']
CeGeI 28 ['Ge0.3I0.5Ce0.2', '-0.924', '-0.206729660088']
CeGeIr 4 ['Ge0.5Ce0.1Ir0.4', '-0.76', '-0.214072851389']
CeGeLi 1 ['Li0.4Ge0.5Ce0.1', '-0.647', '-0.206646652875']
CeGeO 2 ['O0.6Ge0.2Ce0.2', '-2.967', '-0.272818541409']
CeGePa 2 ['Ge0.7Ce0.1Pa0.2', '-0.525', '-0.20751682125']
CeGePt 2 ['Ge0.625Ce0.125Pt0.25', '-0.788', '-0.205678946563']
CeGeRh 1 ['Ge0.6Rh0.3Ce0.1', '-0.786', '-0.20538784825']
CeGeS 3 ['S0.428571Ge0.142857Ce0.428571', '-2.067', '-0.212115947564']
CeGeSe 12 ['Ge0.25Se0.25Ce0.5', '-1.54', '-0.206495074375']
CeHI 52 ['H0.25I0.625Ce0.125', '-0.6', '-0.205122897453']
CeHIn 1 ['H0.571429In0.142857Ce0.285714', '-0.749', '-0.203042219466']
CeHIr 11 ['H0.666667Ce0.111111Ir0.222222', '-0.428', '-0.211547430661']
CeHMn 12 ['H0.666667Mn0.111111Ce0.222222', '-0.635', '-0.202094861322']
CeHN 25 ['H0.571429N0.285714Ce0.142857', '-0.812', '-0.202341301154']
CeHNi 3 ['H0.5Ni0.1Ce0.4', '-0.701', '-0.200597411533']
CeHO 8 ['H0.5O0.25Ce0.25', '-1.833', '-0.203835777254']
CeHOs 3 ['H0.375Ce0.375Os0.25', '-0.547', '-0.2022594559']
CeHPd 21 ['H0.7Pd0.2Ce0.1', '-0.46', '-0.201435568032']
CeHPm 7 ['H0.555556Ce0.111111Pm0.333333', '-0.566', '-0.202706075453']
CeHPt 29 ['H0.6Ce0.1Pt0.3', '-0.541', '-0.200965642583']
CeHRh 29 ['H0.5Rh0.1Ce0.4', '-0.783', '-0.201028324241']
CeHRu 12 ['H0.6Ru0.2Ce0.2', '-0.596', '-0.20638537519']
CeHS 26 ['H0.555556S0.111111Ce0.333333', '-1.112', '-0.20199659084']
CeHSb 2 ['H0.5Sb0.2Ce0.3', '-0.881', '-0.206773861095']
CeHSi 9 ['H0.4Si0.3Ce0.3', '-0.765', '-0.204735645276']
CeHSn 3 ['H0.625Sn0.125Ce0.25', '-0.703', '-0.201041440083']
CeHTc 1 ['H0.555556Tc0.111111Ce0.333333', '-0.715', '-0.21984721337']
CeHfI 4 ['I0.714286Ce0.142857Hf0.142857', '-1.291', '-0.216531939873']
CeHfO 3 ['O0.6Ce0.3Hf0.1', '-3.775', '-0.270906848157']
CeHfS 10 ['S0.5Ce0.25Hf0.25', '-2.017', '-0.200747597992']
CeHfSe 1 ['Se0.555556Ce0.222222Hf0.222222', '-1.815', '-0.248528617013']
CeHgI 28 ['I0.5Ce0.1Hg0.4', '-0.707', '-0.206417805047']
CeHgO 4 ['O0.6Ce0.2Hg0.2', '-2.143', '-0.201045500274']
CeHoI 11 ['I0.625Ce0.25Ho0.125', '-1.234', '-0.222415432278']
CeHoO 3 ['O0.6Ce0.2Ho0.2', '-3.928', '-0.230742304555']
CeIIn 37 ['In0.4I0.4Ce0.2', '-0.957', '-0.216686894194']
CeIIr 34 ['I0.8Ce0.1Ir0.1', '-0.495', '-0.205751165885']
CeIK 13 ['K0.1I0.6Ce0.3', '-1.385', '-0.200490527913']
CeIKr 10 ['Kr0.1I0.6Ce0.3', '-1.082', '-0.214253497655']

CeILa 5 ['I0.714286La0.142857Ce0.142857', '-1.623', '-0.254321015939']
CeILi 15 ['Li0.1I0.6Ce0.3', '-1.336', '-0.203866064913']
CeILu 15 ['I0.571429Ce0.142857Lu0.285714', '-0.959', '-0.228309531999']
CeIMg 9 ['Mg0.1I0.8Ce0.1', '-0.843', '-0.201406296482']
CeIMn 7 ['Mn0.2I0.7Ce0.1', '-0.828', '-0.200336652155']
CeIMo 6 ['Mo0.111111I0.666667Ce0.222222', '-1.05', '-0.240512662445']
CeIN 27 ['N0.333333I0.444444Ce0.222222', '-0.846', '-0.203224813078']
CeINa 13 ['Na0.1I0.6Ce0.3', '-1.357', '-0.206030406327']
CeINb 6 ['Nb0.111111I0.666667Ce0.222222', '-1.117', '-0.214836786713']
CeINd 5 ['I0.714286Ce0.142857Nd0.142857', '-1.454', '-0.307475388971']
CeINi 33 ['Ni0.444444I0.444444Ce0.111111', '-0.615', '-0.202486632253']
CeINp 4 ['I0.7Ce0.2Np0.1', '-1.232', '-0.250359226166']
CeIO 12 ['O0.3I0.2Ce0.5', '-2.498', '-0.207601040539']
CeIOs 19 ['I0.4Ce0.4Os0.2', '-0.999', '-0.204507892029']
CeIP 46 ['P0.166667I0.333333Ce0.5', '-1.404', '-0.202482113947']
CeIPa 11 ['I0.6Ce0.1Pa0.3', '-0.656', '-0.283543936157']
CeIPb 14 ['I0.375Ce0.375Pb0.25', '-1.105', '-0.214901394059']
CeIPd 46 ['Pd0.333333I0.222222Ce0.444444', '-1.113', '-0.202120153904']
CeIPm 12 ['I0.625Ce0.25Pm0.125', '-1.162', '-0.215222040346']
CeIPr 5 ['I0.7Ce0.2Pr0.1', '-1.435', '-0.319822851163']
CeIPt 69 ['I0.111111Ce0.444444Pt0.444444', '-1.278', '-0.202870473804']
CeIPu 6 ['I0.7Ce0.1Pu0.2', '-1.407', '-0.23193347355']
CeIRb 15 ['Rb0.333333I0.555556Ce0.111111', '-1.603', '-0.201309351847']
CeIRe 10 ['I0.625Ce0.25Re0.125', '-0.986', '-0.200038536744']
CeIRh 45 ['Rh0.142857I0.428571Ce0.428571', '-1.207', '-0.206504642886']
CeIRu 28 ['Ru0.3I0.5Ce0.2', '-0.783', '-0.20450233177']
CeIS 8 ['S0.166667I0.5Ce0.333333', '-1.724', '-0.268442866028']
CeISb 54 ['Sb0.1I0.4Ce0.5', '-1.246', '-0.204136775444']
CeISc 9 ['Sc0.222222I0.666667Ce0.111111', '-1.357', '-0.214809077679']
CeISe 32 ['Se0.444444I0.111111Ce0.444444', '-1.934', '-0.200267487507']
CeISi 22 ['Si0.142857I0.428571Ce0.428571', '-1.256', '-0.203791633603']
CeISm 6 ['I0.7Ce0.1Sm0.2', '-1.373', '-0.201382199052']
CeISn 25 ['Sn0.285714I0.428571Ce0.285714', '-1.104', '-0.208531291121']
CeISr 1 ['Sr0.1I0.7Ce0.2', '-1.567', '-0.304582327161']
CeITa 3 ['I0.75Ce0.125Ta0.125', '-0.973', '-0.243005288329']
CeITb 12 ['I0.625Ce0.25Tb0.125', '-1.057', '-0.228002291326']
CeITc 10 ['Tc0.125I0.625Ce0.25', '-0.925', '-0.201877914713']
CeITe 57 ['Te0.571429I0.142857Ce0.285714', '-1.335', '-0.206046851203']
CeITh 3 ['I0.75Ce0.125Th0.125', '-1.434', '-0.214178457706']
CeITi 6 ['Ti0.111111I0.666667Ce0.222222', '-1.263', '-0.20905937907']
CeITl 32 ['I0.5Ce0.166667Tl0.333333', '-1.086', '-0.210244188276']
CeITm 7 ['I0.7Ce0.1Tm0.2', '-1.331', '-0.290544686911']
CeIU 3 ['I0.75Ce0.125U0.125', '-1.186', '-0.218774136767']
CeIV 8 ['V0.166667I0.666667Ce0.166667', '-1.01', '-0.243885775665']

CeIW 7 ['I0.666667Ce0.166667W0.166667', '-0.699', '-0.216918609808']
CeIXe 24 ['I0.4Xe0.5Ce0.1', '-0.501', '-0.211751165885']
CeIY 5 ['Y0.142857I0.714286Ce0.142857', '-1.566', '-0.259260900224']
CeIYb 6 ['I0.666667Ce0.222222Yb0.111111', '-1.438', '-0.202444232538']
CeIZn 22 ['Zn0.333333I0.444444Ce0.222222', '-0.977', '-0.208436506892']
CeIZr 5 ['Zr0.142857I0.714286Ce0.142857', '-1.266', '-0.23044273915']
CeInIr 5 ['In0.5Ce0.1Ir0.4', '-0.516', '-0.2027029625']
CeInN 14 ['N0.4In0.5Ce0.1', '-0.507', '-0.233609003113']
CeInO 4 ['O0.25In0.25Ce0.5', '-1.941', '-0.218716688425']
CeInRh 1 ['Rh0.333333In0.555556Ce0.111111', '-0.696', '-0.207807591759']
CeInS 1 ['S0.333333In0.222222Ce0.444444', '-1.813', '-0.21266803366']
CeIrN 4 ['N0.3Ce0.6Ir0.1', '-1.166', '-0.220103868648']
CeIrO 2 ['O0.666667Ce0.222222Ir0.111111', '-2.512', '-0.201459850481']
CeIrSe 6 ['Se0.428571Ce0.428571Ir0.142857', '-1.8', '-0.200824256428']
CeIrSi 6 ['Si0.444444Ce0.111111Ir0.444444', '-1.083', '-0.210332443056']
CeKO 3 ['O0.6K0.1Ce0.3', '-3.131', '-0.201918130783']
CeKSe 3 ['K0.166667Se0.5Ce0.333333', '-1.882', '-0.203181829999']
CeLaPd 1 ['Pd0.666667La0.222222Ce0.111111', '-1.019', '-0.212681264444']
CeLiO 2 ['Li0.285714O0.428571Ce0.285714', '-2.726', '-0.201233996885']
CeLiS 1 ['Li0.3S0.4Ce0.3', '-1.934', '-0.206175149518']
CeLiSe 6 ['Li0.142857Se0.428571Ce0.428571', '-1.828', '-0.234267870894']
CeLuO 6 ['O0.666667Ce0.166667Lu0.166667', '-3.328', '-0.210494218204']
CeLuTe 1 ['Te0.6Ce0.1Lu0.3', '-1.348', '-0.205360247584']
CeMgO 5 ['O0.666667Mg0.111111Ce0.222222', '-2.832', '-0.211837205021']
CeMgS 14 ['Mg0.125S0.375Ce0.5', '-1.852', '-0.203877066097']
CeMnN 11 ['N0.4Mn0.3Ce0.3', '-1.114', '-0.220212109105']
CeMnO 2 ['O0.6Mn0.2Ce0.2', '-3.173', '-0.32480488803']
CeMnSi 4 ['Si0.5Mn0.333333Ce0.166667', '-0.798', '-0.249000315402']
CeMoO 7 ['O0.6Mo0.1Ce0.3', '-3.39', '-0.249443561042']
CeMoSe 4 ['Se0.571429Mo0.285714Ce0.142857', '-1.213', '-0.20475030369']
CeNNp 15 ['N0.428571Ce0.285714Np0.285714', '-1.314', '-0.218220787174']
CeNO 2 ['N0.125O0.625Ce0.25', '-2.643', '-0.25279146925']
CeNP 19 ['N0.444444P0.111111Ce0.444444', '-1.471', '-0.204535130034']
CeNPa 13 ['N0.5Ce0.333333Pa0.166667', '-1.425', '-0.22142859207']
CeNPt 22 ['N0.222222Ce0.555556Pt0.222222', '-1.287', '-0.208784963119']
CeNPu 3 ['N0.555556Ce0.111111Pu0.333333', '-1.485', '-0.280833095682']
CeNRe 1 ['N0.6Ce0.2Re0.2', '-0.781', '-0.233201215409']
CeNSb 1 ['N0.5Sb0.3Ce0.2', '-0.728', '-0.203445993432']
CeNSi 2 ['N0.1Si0.5Ce0.4', '-1.053', '-0.240706961216']
CeNTa 1 ['N0.5Ce0.25Ta0.25', '-1.56', '-0.228211420034']
CeNTi 8 ['N0.555556Ti0.222222Ce0.222222', '-1.361', '-0.201701405143']
CeNW 7 ['N0.571429Ce0.285714W0.142857', '-1.011', '-0.209389741504']
CeNZn 1 ['N0.4Zn0.5Ce0.1', '-0.513', '-0.25002688355']
CeNaO 1 ['O0.625Na0.125Ce0.25', '-2.854', '-0.262166981062']

CeNaSe 8 ['Na0.1Se0.5Ce0.4', '-1.967', '-0.214425865915']
CeNbO 3 ['O0.666667Nb0.111111Ce0.222222', '-3.412', '-0.227184558279']
CeNbSe 1 ['Se0.6Nb0.2Ce0.2', '-1.581', '-0.233645950148']
CeNiO 5 ['O0.666667Ni0.166667Ce0.166667', '-2.125', '-0.213396615575']
CeNpO 3 ['O0.6Ce0.2Np0.2', '-3.701', '-0.2004029137']
CeOOs 1 ['O0.625Ce0.25Os0.125', '-3.006', '-0.370227546166']
CeOP 8 ['O0.3P0.3Ce0.4', '-2.681', '-0.209548289016']
CeOPa 8 ['O0.7Ce0.2Pa0.1', '-2.911', '-0.221170191434']
CeOPb 2 ['O0.666667Ce0.166667Pb0.166667', '-2.268', '-0.243893083133']
CeOPd 4 ['O0.6Pd0.2Ce0.2', '-2.345', '-0.23834949697']
CeOPr 1 ['O0.625Ce0.25Pr0.125', '-3.75', '-0.432241284581']
CeOPt 5 ['O0.6Ce0.3Pt0.1', '-3.024', '-0.209754176033']
CeOPu 3 ['O0.666667Ce0.222222Pu0.111111', '-3.33', '-0.287288009378']
CeORe 3 ['O0.666667Ce0.166667Re0.166667', '-2.765', '-0.237580426184']
CeORh 5 ['O0.666667Rh0.166667Ce0.166667', '-2.19', '-0.204274685633']
CeORu 2 ['O0.625Ru0.125Ce0.25', '-2.966', '-0.290874645542']
CeOS 14 ['O0.5S0.166667Ce0.333333', '-3.193', '-0.20309541332']
CeOSb 5 ['O0.285714Sb0.142857Ce0.571429', '-2.255', '-0.208697292843']
CeOSc 2 ['O0.6Sc0.2Ce0.2', '-3.904', '-0.20256373297']
CeOSe 14 ['O0.2Se0.2Ce0.6', '-2.117', '-0.203701903907']
CeOSi 6 ['O0.25Si0.25Ce0.5', '-2.071', '-0.2366564178']
CeOSn 3 ['O0.6Sn0.2Ce0.2', '-2.857', '-0.203349095476']
CeOTa 4 ['O0.666667Ce0.111111Ta0.222222', '-3.555', '-0.272034685357']
CeOTb 1 ['O0.625Ce0.25Tb0.125', '-3.609', '-0.296179203375']
CeOTc 6 ['O0.6Tc0.1Ce0.3', '-3.343', '-0.320104537595']
CeOTe 4 ['O0.333333Te0.222222Ce0.444444', '-2.874', '-0.211993921048']
CeOTi 3 ['O0.625Ti0.125Ce0.25', '-3.726', '-0.309772471944']
CeOTl 2 ['O0.6Ce0.2Tl0.2', '-2.441', '-0.212183524846']
CeOTm 4 ['O0.666667Ce0.166667Tm0.166667', '-3.31', '-0.204906535912']
CeOV 3 ['O0.6V0.1Ce0.3', '-3.629', '-0.28931076607']
CeOW 5 ['O0.666667Ce0.166667W0.166667', '-3.2', '-0.268184950481']
CeOZn 7 ['O0.625Zn0.25Ce0.125', '-2.135', '-0.210429259224']
CeOZr 2 ['O0.625Zr0.125Ce0.25', '-3.825', '-0.220346993433']
CeOsP 1 ['P0.5Ce0.25Os0.25', '-1.298', '-0.230421474895']
CeOsSi 1 ['Si0.5Ce0.125Os0.375', '-0.787', '-0.201821661719']
CePPm 1 ['P0.5Ce0.1Pm0.4', '-1.48', '-0.20292071777']
CePRh 4 ['P0.428571Rh0.142857Ce0.428571', '-1.638', '-0.204685533482']
CePaS 2 ['S0.666667Ce0.166667Pa0.166667', '-1.855', '-0.207672878489']
CePaSe 7 ['Se0.625Ce0.25Pa0.125', '-1.74', '-0.205404546247']
CePdSe 13 ['Se0.555556Pd0.111111Ce0.333333', '-1.78', '-0.212082417312']
CePmS 9 ['S0.375Ce0.125Pm0.5', '-1.676', '-0.20168694693']
CePmSe 8 ['Se0.625Ce0.25Pm0.125', '-1.84', '-0.201165834685']
CePtS 5 ['S0.333333Ce0.222222Pt0.444444', '-1.477', '-0.217811756525']
CePtSb 1 ['Sb0.375Ce0.25Pt0.375', '-1.161', '-0.209086943266']

CePtSe 18 ['Se0.6Ce0.2Pt0.2', '-1.329', '-0.203401743052']
CePtSi 3 ['Si0.5Ce0.125Pt0.375', '-1.062', '-0.209561971875']
CeRbTe 1 ['Rb0.142857Te0.571429Ce0.285714', '-1.601', '-0.237999799523']
CeRhS 2 ['S0.5Rh0.3Ce0.2', '-1.547', '-0.207039085492']
CeRhSe 14 ['Se0.428571Rh0.142857Ce0.428571', '-1.8', '-0.205879209999']
CeRhSi 7 ['Si0.555556Rh0.333333Ce0.111111', '-1.008', '-0.201102723611']
CeRuS 1 ['S0.5Ru0.25Ce0.25', '-1.762', '-0.232171044867']
CeRuSe 1 ['Se0.4Ru0.1Ce0.5', '-1.736', '-0.237868086964']
CeSSe 2 ['S0.2Se0.5Ce0.3', '-1.78', '-0.212843537511']
CeSSi 15 ['Si0.285714S0.428571Ce0.285714', '-1.815', '-0.224828682564']
CeSTc 11 ['S0.444444Tc0.333333Ce0.222222', '-1.573', '-0.201680794882']
CeSZr 7 ['S0.444444Zr0.444444Ce0.111111', '-1.718', '-0.200299595712']
CeSbSe 5 ['Se0.4Sb0.2Ce0.4', '-1.778', '-0.220430942499']
CeScSe 2 ['Sc0.25Se0.625Ce0.125', '-1.645', '-0.231199566249']
CeScSi 2 ['Si0.375Sc0.375Ce0.25', '-0.904', '-0.216035393636']
CeSeSi 6 ['Si0.2Se0.4Ce0.4', '-1.692', '-0.201897828999']
CeSeTa 1 ['Se0.6Ce0.2Ta0.2', '-1.492', '-0.206126174388']
CeSeTc 4 ['Se0.555556Tc0.333333Ce0.111111', '-0.76', '-0.21173817111']
CeSeTe 1 ['Se0.2Te0.4Ce0.4', '-1.8', '-0.243315630166']
CeSeTl 4 ['Se0.444444Ce0.444444Tl0.111111', '-1.872', '-0.227914978889']
CeSeTm 3 ['Se0.625Ce0.125Tm0.25', '-1.843', '-0.213236801564']
CeSeY 9 ['Se0.625Y0.125Ce0.25', '-1.888', '-0.212935578122']
CeSeZr 4 ['Se0.6Zr0.2Ce0.2', '-1.79', '-0.230616990481']
CeTeY 1 ['Y0.3Te0.6Ce0.1', '-1.496', '-0.222095555584']
ClCoEr 30 ['Cl0.5Co0.333333Er0.166667', '-0.977', '-0.21719010219']
ClCoF 17 ['F0.4Cl0.4Co0.2', '-1.398', '-0.20108333378']
ClCoH 1 ['H0.444444Cl0.444444Co0.111111', '-0.802', '-0.20390896542']
ClCoHo 27 ['Cl0.444444Co0.111111Ho0.444444', '-1.434', '-0.230649947129']
ClCoLu 24 ['Cl0.428571Co0.142857Lu0.428571', '-1.287', '-0.223421566877']
ClCoMg 2 ['Mg0.25Cl0.625Co0.125', '-1.92', '-0.223821789823']
ClCoO 7 ['O0.1Cl0.6Co0.3', '-0.967', '-0.232168042145']
ClCoPm 13 ['Cl0.75Co0.125Pm0.125', '-1.559', '-0.204775566098']
ClCoPu 1 ['Cl0.8Co0.1Pu0.1', '-1.357', '-0.24914092063']
ClCoRh 7 ['Cl0.75Co0.125Rh0.125', '-0.574', '-0.276884613518']
ClCoSe 4 ['Cl0.6Co0.3Se0.1', '-0.797', '-0.201370004128']
ClCoSm 8 ['Cl0.75Co0.125Sm0.125', '-1.473', '-0.216455165473']
ClCoTe 1 ['Cl0.6Co0.3Te0.1', '-0.837', '-0.200534552545']
ClCrEr 16 ['Cl0.555556Cr0.222222Er0.222222', '-1.449', '-0.246389440213']
ClCrGa 1 ['Cl0.625Cr0.25Ga0.125', '-1.398', '-0.222619241852']
ClCrGe 1 ['Cl0.6Cr0.3Ge0.1', '-1.347', '-0.224226942965']
ClCrHo 15 ['Cl0.625Cr0.25Ho0.125', '-1.517', '-0.257575553157']
ClCrIn 2 ['Cl0.777778Cr0.111111In0.111111', '-1.21', '-0.22029763845']
ClCrK 1 ['Cl0.666667K0.222222Cr0.111111', '-1.78', '-0.214412723547']
ClCrLi 1 ['Li0.2Cl0.7Cr0.1', '-1.571', '-0.21678236779']

ClCrLu 17 ['Cl0.5Cr0.125Lu0.375', '-1.365', '-0.204123309899']
ClCrNb 1 ['Cl0.75Cr0.125Nb0.125', '-1.663', '-0.205811721411']
ClCrNp 1 ['Cl0.8Cr0.1Np0.1', '-1.798', '-0.309264197691']
ClCrO 3 ['O0.6Cl0.1Cr0.3', '-2.137', '-0.212005527934']
ClCrPm 8 ['Cl0.7Cr0.1Pm0.2', '-2.107', '-0.231429075233']
ClCrPr 2 ['Cl0.7Cr0.2Pr0.1', '-1.974', '-0.206578653485']
ClCrPu 2 ['Cl0.777778Cr0.111111Pu0.111111', '-1.816', '-0.235159870147']
ClCrRb 5 ['Cl0.666667Cr0.111111Rb0.222222', '-1.801', '-0.23949636688']
ClCrRh 7 ['Cl0.666667Cr0.166667Rh0.166667', '-1.128', '-0.207017755527']
ClCrSb 8 ['Cl0.5Cr0.25Sb0.25', '-1.122', '-0.201017755527']
ClCrSc 1 ['Cl0.8Sc0.1Cr0.1', '-1.723', '-0.215414369132']
ClCrSe 2 ['Cl0.571429Cr0.285714Se0.142857', '-1.409', '-0.231534425602']
ClCrSi 1 ['Si0.1Cl0.7Cr0.2', '-1.553', '-0.209678065029']
ClCrSm 9 ['Cl0.666667Cr0.111111Sm0.222222', '-2.053', '-0.256456324405']
ClCrTa 5 ['Cl0.777778Cr0.111111Ta0.111111', '-1.737', '-0.243683944518']
ClCrTb 1 ['Cl0.777778Cr0.111111Tb0.111111', '-1.875', '-0.205157151849']
ClCrTe 11 ['Cl0.5Cr0.3Te0.2', '-1.191', '-0.20026553861']
ClCrTh 1 ['Cl0.8Cr0.1Th0.1', '-2.033', '-0.255915208566']
ClCrTi 1 ['Cl0.7Ti0.1Cr0.2', '-1.721', '-0.236334561857']
ClCrTl 2 ['Cl0.6Cr0.2Tl0.2', '-1.427', '-0.208569864297']
ClCsCu 1 ['Cl0.5Cu0.375Cs0.125', '-1.221', '-0.222004065943']
ClCsEr 19 ['Cl0.555556Cs0.111111Er0.333333', '-1.774', '-0.412428867502']
ClCsEu 5 ['Cl0.5Cs0.375Eu0.125', '-2.373', '-0.209798619691']
ClCsF 36 ['F0.1Cl0.5Cs0.4', '-2.06', '-0.201043957027']
ClCsGa 1 ['Cl0.444444Ga0.111111Cs0.444444', '-2.129', '-0.214449850836']
ClCsGd 1 ['Cl0.714286Cs0.142857Gd0.142857', '-2.352', '-0.291329064289']
ClCsHo 19 ['Cl0.555556Cs0.111111Ho0.333333', '-1.878', '-0.496849923056']
ClCsI 4 ['Cl0.625I0.125Cs0.25', '-1.41', '-0.200284178838']
ClCsIn 8 ['Cl0.6In0.3Cs0.1', '-1.637', '-0.206175020963']
ClCsK 1 ['Cl0.555556K0.222222Cs0.222222', '-2.129', '-0.220125811947']
ClCsLa 3 ['Cl0.714286Cs0.142857La0.142857', '-2.359', '-0.209697876791']
ClCsLi 1 ['Li0.333333Cl0.555556Cs0.111111', '-2.029', '-0.215410691391']
ClCsLu 8 ['Cl0.777778Cs0.111111Lu0.111111', '-1.646', '-0.279319353658']
ClCsMn 3 ['Cl0.625Mn0.125Cs0.25', '-1.829', '-0.201107148802']
ClCsMo 1 ['Cl0.777778Mo0.111111Cs0.111111', '-1.421', '-0.211596449622']
ClCsNp 11 ['Cl0.571429Cs0.285714Np0.142857', '-2.193', '-0.201611913099']
ClCsO 2 ['O0.666667Cl0.111111Cs0.222222', '-1.226', '-0.231248903791']
ClCsP 16 ['P0.142857Cl0.714286Cs0.142857', '-1.503', '-0.206131494885']
ClCsPa 8 ['Cl0.7Cs0.1Pa0.2', '-2.175', '-0.207396863064']
ClCsPm 15 ['Cl0.571429Cs0.142857Pm0.285714', '-2.094', '-0.213590864646']
ClCsPr 1 ['Cl0.75Cs0.125Pr0.125', '-2.027', '-0.200238331878']
ClCsPt 1 ['Cl0.625Cs0.125Pt0.25', '-1.319', '-0.247491140657']
ClCsPu 13 ['Cl0.5Cs0.375Pu0.125', '-2.183', '-0.205028824066']
ClCsRb 4 ['Cl0.5Rb0.2Cs0.3', '-2.35', '-0.20491472619']

ClCsRe 1 ['Cl0.777778Cs0.111111Re0.111111', '-1.25', '-0.221335213822']
ClCsRh 19 ['Cl0.8Rh0.1Cs0.1', '-0.634', '-0.203226216438']
ClCsRu 1 ['Cl0.666667Ru0.111111Cs0.222222', '-1.573', '-0.209586234932']
ClCsS 6 ['S0.142857Cl0.714286Cs0.142857', '-0.998', '-0.204210129518']
ClCsSe 11 ['Cl0.333333Se0.222222Cs0.444444', '-1.87', '-0.200718370904']
ClCsSi 18 ['Si0.1Cl0.7Cs0.2', '-1.838', '-0.212926758126']
ClCsSm 10 ['Cl0.666667Cs0.222222Sm0.111111', '-2.11', '-0.299898749378']
ClCsTa 2 ['Cl0.7Cs0.1Ta0.2', '-1.858', '-0.206915083065']
ClCsTb 4 ['Cl0.714286Cs0.142857Tb0.142857', '-2.173', '-0.200081428266']
ClCsTc 2 ['Cl0.7Tc0.1Cs0.2', '-1.566', '-0.228634999753']
ClCsTe 29 ['Cl0.5Te0.166667Cs0.333333', '-1.864', '-0.200724602468']
ClCsXe 9 ['Cl0.6Xe0.2Cs0.2', '-1.062', '-0.200452432876']
ClCsY 1 ['Cl0.555556Y0.111111Cs0.333333', '-2.465', '-0.235890460398']
ClCsZn 2 ['Cl0.555556Zn0.222222Cs0.222222', '-1.923', '-0.226986564243']
ClCsZr 1 ['Cl0.555556Zr0.111111Cs0.333333', '-2.324', '-0.245431300583']
ClCuEr 41 ['Cl0.333333Cu0.333333Er0.333333', '-1.173', '-0.290066404793']
ClCuEu 1 ['Cl0.555556Cu0.333333Eu0.111111', '-1.559', '-0.236331239933']
ClCuGd 3 ['Cl0.555556Cu0.333333Gd0.111111', '-1.61', '-0.231123559378']
ClCuHg 7 ['Cl0.6Cu0.3Hg0.1', '-0.909', '-0.221186882132']
ClCuHo 37 ['Cl0.4Cu0.3Ho0.3', '-1.128', '-0.20109225175']
ClCuIr 4 ['Cl0.75Cu0.125Ir0.125', '-0.467', '-0.200296012345']
ClCuK 2 ['Cl0.5K0.1Cu0.4', '-1.128', '-0.237547099439']
ClCuLa 2 ['Cl0.6Cu0.3La0.1', '-1.651', '-0.23332290963']
ClCuLu 43 ['Cl0.444444Cu0.444444Lu0.111111', '-0.861', '-0.203102568058']
ClCuNd 3 ['Cl0.555556Cu0.333333Nd0.111111', '-1.64', '-0.244853651324']
ClCuNp 3 ['Cl0.7Cu0.1Np0.2', '-1.95', '-0.215217403815']
ClCuPa 1 ['Cl0.75Cu0.125Pa0.125', '-1.987', '-0.242828012032']
ClCuPb 2 ['Cl0.571429Cu0.285714Pb0.142857', '-1.133', '-0.208244441076']
ClCuPm 30 ['Cl0.5Cu0.333333Pm0.166667', '-1.619', '-0.209521270107']
ClCuPr 6 ['Cl0.444444Cu0.222222Pr0.333333', '-1.821', '-0.226132561322']
ClCuPu 14 ['Cl0.625Cu0.125Pu0.25', '-2.016', '-0.203152562117']
ClCuRh 12 ['Cl0.777778Cu0.111111Rh0.111111', '-0.449', '-0.211929788751']
ClCuSm 14 ['Cl0.625Cu0.125Sm0.25', '-1.951', '-0.211260295551']
ClCuSr 1 ['Cl0.6Cu0.3Sr0.1', '-1.508', '-0.245088783629']
ClCuTa 1 ['Cl0.7Cu0.1Ta0.2', '-1.604', '-0.219058569313']
ClCuTb 1 ['Cl0.6Cu0.3Tb0.1', '-1.505', '-0.210791395662']
ClCuTe 6 ['Cl0.571429Cu0.285714Te0.142857', '-0.904', '-0.247648728277']
ClCuTh 1 ['Cl0.666667Cu0.222222Th0.111111', '-1.841', '-0.225679589029']
ClCuTl 3 ['Cl0.5Cu0.375Tl0.125', '-0.954', '-0.223048052815']
ClCuU 1 ['Cl0.7Cu0.1U0.2', '-1.969', '-0.203251324566']
ClCuY 8 ['Cl0.25Cu0.25Y0.5', '-1.193', '-0.208733013179']
ClCuYb 1 ['Cl0.555556Cu0.333333Yb0.111111', '-1.54', '-0.24813192299']
ClDyEr 6 ['Cl0.666667Dy0.111111Er0.222222', '-2.052', '-0.341732015582']
ClDyF 5 ['F0.444444Cl0.222222Dy0.333333', '-3.401', '-0.206931540993']

ClDyFe 1 ['Cl0.777778Fe0.111111Dy0.111111', '-1.812', '-0.260456678292']
ClDyH 3 ['H0.333333Cl0.333333Dy0.333333', '-1.671', '-0.203505008881']
ClDyHo 6 ['Cl0.666667Dy0.111111Ho0.222222', '-2.131', '-0.407679385951']
ClDyI 34 ['Cl0.714286I0.142857Dy0.142857', '-1.763', '-0.202719657455']
ClDyIn 10 ['Cl0.4In0.3Dy0.3', '-1.767', '-0.21528436742']
ClDyIr 4 ['Cl0.714286Dy0.142857Ir0.142857', '-1.662', '-0.219884244497']
ClDyLa 1 ['Cl0.777778La0.111111Dy0.111111', '-2.516', '-0.201312520516']
ClDyLu 6 ['Cl0.666667Dy0.111111Lu0.222222', '-2.099', '-0.425869298916']
ClDyN 2 ['N0.333333Cl0.166667Dy0.5', '-1.983', '-0.21646820552']
ClDyNa 1 ['Na0.142857Cl0.714286Dy0.142857', '-2.254', '-0.218395895479']
ClDyNp 1 ['Cl0.8Dy0.1Np0.1', '-2.261', '-0.315372515523']
ClDyO 5 ['O0.6Cl0.1Dy0.3', '-3.173', '-0.219760480297']
ClDyPt 3 ['Cl0.75Dy0.125Pt0.125', '-1.72', '-0.231438519197']
ClDyPu 2 ['Cl0.777778Dy0.111111Pu0.111111', '-2.3', '-0.211502445516']
ClDyRb 1 ['Cl0.714286Rb0.142857Dy0.142857', '-2.306', '-0.204564211906']
ClDyRe 1 ['Cl0.8Dy0.1Re0.1', '-1.645', '-0.206643679901']
ClDyRh 8 ['Cl0.6Rh0.3Dy0.1', '-1.22', '-0.210518971148']
ClDyRu 3 ['Cl0.8Ru0.1Dy0.1', '-1.54', '-0.209990270462']
ClDySe 2 ['Cl0.25Se0.5Dy0.25', '-1.833', '-0.20856762151']
ClDySm 2 ['Cl0.8Sm0.1Dy0.1', '-2.058', '-0.280975412712']
ClDyTa 1 ['Cl0.8Dy0.1Ta0.1', '-2.022', '-0.221123867898']
ClDyTc 2 ['Cl0.7Tc0.2Dy0.1', '-1.8', '-0.268611534234']
ClDyTe 4 ['Cl0.333333Te0.444444Dy0.222222', '-1.748', '-0.218722977757']
ClDyTl 3 ['Cl0.5Dy0.375Tl0.125', '-1.985', '-0.202209107816']
ClDyV 1 ['Cl0.8V0.1Dy0.1', '-1.947', '-0.236446259898']
ClErEu 11 ['Cl0.6Eu0.1Er0.3', '-1.941', '-0.29989726794']
ClErF 39 ['F0.222222Cl0.333333Er0.444444', '-2.403', '-0.203999309617']
ClErFe 25 ['Cl0.555556Fe0.333333Er0.111111', '-1.144', '-0.211977127989']
ClErGa 53 ['Cl0.142857Ga0.571429Er0.285714', '-0.971', '-0.204367003127']
ClErGd 6 ['Cl0.666667Gd0.111111Er0.222222', '-2.082', '-0.369271079378']
ClErGe 62 ['Cl0.428571Ge0.428571Er0.142857', '-1.106', '-0.203211787619']
ClErH 41 ['H0.5Cl0.4Er0.1', '-1.082', '-0.218953502878']
ClErHf 6 ['Cl0.714286Er0.142857Hf0.142857', '-2.1', '-0.209991160983']
ClErHg 43 ['Cl0.3Er0.3Hg0.4', '-1.002', '-0.207359764314']
ClErHo 19 ['Cl0.555556Ho0.333333Er0.111111', '-1.586', '-0.389176261944']
ClErI 80 ['Cl0.142857I0.428571Er0.428571', '-1.257', '-0.203547085263']
ClErIn 48 ['Cl0.375In0.125Er0.5', '-1.32', '-0.215116770393']
ClErIr 48 ['Cl0.5Er0.1Ir0.4', '-0.48', '-0.215119921438']
ClErK 19 ['Cl0.555556K0.111111Er0.333333', '-1.786', '-0.427266848058']
ClErKr 34 ['Cl0.444444Kr0.222222Er0.333333', '-1.127', '-0.244066404793']
ClErLa 6 ['Cl0.666667La0.111111Er0.222222', '-2.123', '-0.341335711324']
ClErLi 21 ['Li0.125Cl0.5Er0.375', '-1.717', '-0.22373856094']
ClErLu 24 ['Cl0.5Er0.333333Lu0.166667', '-1.509', '-0.21245256969']
ClErMg 15 ['Mg0.2Cl0.5Er0.3', '-1.728', '-0.201680283358']

ClErMn 34 ['Cl0.444444Mn0.333333Er0.222222', '-1.236', '-0.205810529302']
ClErMo 11 ['Cl0.6Mo0.1Er0.3', '-1.608', '-0.361725684852']
ClErN 65 ['N0.3Cl0.2Er0.5', '-1.892', '-0.230421219274']
ClErNa 7 ['Na0.2Cl0.6Er0.2', '-2.391', '-0.203016895123']
ClErNb 10 ['Cl0.666667Nb0.166667Er0.166667', '-1.749', '-0.285597742084']
ClErNd 6 ['Cl0.666667Nd0.111111Er0.222222', '-2.122', '-0.393001171324']
ClErNi 46 ['Cl0.333333Ni0.444444Er0.222222', '-0.985', '-0.210189052369']
ClErNp 7 ['Cl0.7Er0.1Np0.2', '-2.104', '-0.241847640318']
ClErO 42 ['O0.125Cl0.75Er0.125', '-1.149', '-0.226832485905']
ClErOs 32 ['Cl0.7Er0.1Os0.2', '-0.966', '-0.201529316567']
ClErP 67 ['P0.375Cl0.5Er0.125', '-1.315', '-0.201211527493']
ClErPa 10 ['Cl0.555556Er0.333333Pa0.111111', '-1.679', '-0.226870347988']
ClErPb 32 ['Cl0.333333Er0.444444Pb0.222222', '-1.257', '-0.243639588329']
ClErPd 45 ['Cl0.333333Pd0.444444Er0.222222', '-1.144', '-0.206935481831']
ClErPm 12 ['Cl0.6Pm0.3Er0.1', '-1.946', '-0.214800150878']
ClErPr 6 ['Cl0.666667Pr0.111111Er0.222222', '-2.159', '-0.425226991044']
ClErPt 55 ['Cl0.222222Er0.333333Pt0.444444', '-1.343', '-0.207280506582']
ClErPu 19 ['Cl0.5Er0.3Pu0.2', '-1.651', '-0.200722049693']
ClErRb 19 ['Cl0.555556Rb0.111111Er0.333333', '-1.742', '-0.385308669724']
ClErRe 13 ['Cl0.5Er0.375Re0.125', '-1.45', '-0.299950335037']
ClErRh 51 ['Cl0.3Rh0.5Er0.2', '-0.731', '-0.201239842876']
ClErRu 30 ['Cl0.4Ru0.1Er0.5', '-1.338', '-0.203423025752']
ClErS 44 ['S0.333333Cl0.111111Er0.555556', '-1.934', '-0.202286929147']
ClErSb 39 ['Cl0.3Sb0.2Er0.5', '-1.442', '-0.208829705314']
ClErSc 6 ['Cl0.666667Sc0.111111Er0.222222', '-2.106', '-0.456270620769']
ClErSe 68 ['Cl0.4Se0.5Er0.1', '-0.985', '-0.200132223752']
ClErSi 42 ['Si0.333333Cl0.166667Er0.5', '-1.195', '-0.202098079063']
ClErSm 11 ['Cl0.6Sm0.1Er0.3', '-1.825', '-0.262816205878']
ClErSn 36 ['Cl0.5Sn0.333333Er0.166667', '-1.358', '-0.210748531738']
ClErSr 11 ['Cl0.6Sr0.1Er0.3', '-2.033', '-0.418104663191']
ClErTa 9 ['Cl0.7Er0.1Ta0.2', '-1.679', '-0.227027266563']
ClErTb 6 ['Cl0.666667Tb0.111111Er0.222222', '-2.086', '-0.441522918027']
ClErTc 23 ['Cl0.444444Tc0.111111Er0.444444', '-1.379', '-0.201755206391']
ClErTe 75 ['Cl0.2Te0.6Er0.2', '-1.131', '-0.204718529251']
ClErTh 4 ['Cl0.7Er0.2Th0.1', '-2.034', '-0.279744398126']
ClErTi 12 ['Cl0.666667Ti0.166667Er0.166667', '-1.942', '-0.254067131457']
ClErTl 42 ['Cl0.333333Er0.444444Tl0.222222', '-1.22', '-0.247106957016']
ClErTm 6 ['Cl0.666667Er0.222222Tm0.111111', '-2.074', '-0.366224874658']
ClErU 6 ['Cl0.714286Er0.142857U0.142857', '-2.069', '-0.278044885983']
ClErV 14 ['Cl0.555556V0.111111Er0.333333', '-1.553', '-0.21581364021']
ClErW 12 ['Cl0.571429Er0.285714W0.142857', '-1.412', '-0.258518226073']
ClErXe 38 ['Cl0.375Xe0.5Er0.125', '-0.552', '-0.220899901797']
ClErY 6 ['Cl0.666667Y0.111111Er0.222222', '-2.034', '-0.310584207436']
ClErYb 11 ['Cl0.625Er0.25Yb0.125', '-2.065', '-0.379372451566']

ClErZn 34 ['Cl0.375Zn0.375Er0.25', '-1.229', '-0.23216338767']
ClErZr 9 ['Cl0.666667Zr0.166667Er0.166667', '-1.99', '-0.215232553757']
ClEuF 12 ['F0.8Cl0.1Eu0.1', '-1.967', '-0.214436759872']
ClEuFe 1 ['Cl0.625Fe0.125Eu0.25', '-2.567', '-0.271487350863']
ClEuGa 2 ['Cl0.444444Ga0.111111Eu0.444444', '-2.24', '-0.221663933058']
ClEuGe 9 ['Cl0.333333Ge0.111111Eu0.555556', '-1.858', '-0.203239526877']
ClEuHg 6 ['Cl0.444444Eu0.444444Hg0.111111', '-2.24', '-0.209403177506']
ClEuHo 11 ['Cl0.6Eu0.1Ho0.3', '-1.998', '-0.339276217938']
ClEuI 7 ['Cl0.5I0.1Eu0.4', '-2.603', '-0.206592405322']
ClEuIn 12 ['Cl0.666667In0.222222Eu0.111111', '-1.892', '-0.200057718005']
ClEuIr 12 ['Cl0.333333Eu0.5Ir0.166667', '-1.837', '-0.201410949615']
ClEuK 5 ['Cl0.6K0.1Eu0.3', '-2.747', '-0.202624158003']
ClEuLi 2 ['Li0.1Cl0.6Eu0.3', '-2.746', '-0.206612510878']
ClEuLu 11 ['Cl0.6Eu0.1Lu0.3', '-2.05', '-0.45903260044']
ClEuMg 2 ['Mg0.142857Cl0.571429Eu0.285714', '-2.627', '-0.208535724646']
ClEuN 30 ['N0.1Cl0.3Eu0.6', '-1.596', '-0.210628123905']
ClEuNa 12 ['Na0.1Cl0.4Eu0.5', '-1.897', '-0.204075007252']
ClEuNp 2 ['Cl0.8Eu0.1Np0.1', '-2.034', '-0.251391048001']
ClEuO 6 ['O0.6Cl0.1Eu0.3', '-2.373', '-0.200658477131']
ClEuP 27 ['P0.111111Cl0.555556Eu0.333333', '-2.736', '-0.202421605855']
ClEuPd 3 ['Cl0.5Pd0.166667Eu0.333333', '-2.555', '-0.217190560315']
ClEuPm 6 ['Cl0.8Pm0.1Eu0.1', '-1.896', '-0.20385026569']
ClEuPt 23 ['Cl0.7Eu0.1Pt0.2', '-1.41', '-0.200801192045']
ClEuPu 5 ['Cl0.7Eu0.2Pu0.1', '-2.809', '-0.245908237068']
ClEuRb 3 ['Cl0.571429Rb0.142857Eu0.285714', '-2.651', '-0.228034705539']
ClEuRh 14 ['Cl0.6Rh0.2Eu0.2', '-1.895', '-0.202075007252']
ClEuS 20 ['S0.285714Cl0.142857Eu0.571429', '-2.223', '-0.206764104299']
ClEuSb 20 ['Cl0.375Sb0.125Eu0.5', '-2.157', '-0.202520637215']
ClEuSe 39 ['Cl0.285714Se0.142857Eu0.571429', '-2.042', '-0.202229463751']
ClEuSi 25 ['Si0.142857Cl0.714286Eu0.142857', '-2.246', '-0.218704639376']
ClEuSm 7 ['Cl0.7Sm0.2Eu0.1', '-2.489', '-0.250745740067']
ClEuSn 1 ['Cl0.444444Sn0.111111Eu0.444444', '-2.31', '-0.241722592296']
ClEuSr 1 ['Cl0.6Sr0.1Eu0.3', '-2.762', '-0.222612510878']
ClEuTa 1 ['Cl0.8Eu0.1Ta0.1', '-1.844', '-0.206142400376']
ClEuTc 2 ['Cl0.777778Tc0.111111Eu0.111111', '-1.674', '-0.204800078337']
ClEuTe 18 ['Cl0.444444Te0.222222Eu0.333333', '-2.5', '-0.212927403242']
ClEuTh 1 ['Cl0.777778Eu0.111111Th0.111111', '-2.514', '-0.212935620973']
ClEuTl 11 ['Cl0.333333Eu0.444444Tl0.222222', '-1.863', '-0.202338744932']
ClEuXe 2 ['Cl0.666667Xe0.166667Eu0.166667', '-1.622', '-0.21122917271']
ClFFe 14 ['F0.5Cl0.333333Fe0.166667', '-1.797', '-0.201890485433']
ClFGa 1 ['F0.8Cl0.1Ga0.1', '-1.807', '-0.275459671459']
ClFGd 7 ['F0.7Cl0.1Gd0.2', '-3.669', '-0.208879322299']
ClFGe 5 ['F0.571429Cl0.142857Ge0.285714', '-2.378', '-0.215214279067']
ClFH 3 ['H0.125F0.75Cl0.125', '-1.092', '-0.207601598834']

ClFHg 2 ['F0.8Cl0.1Hg0.1', '-1.093', '-0.213761177376']
ClFHo 41 ['F0.5Cl0.2Ho0.3', '-3.524', '-0.201447840685']
ClFI 10 ['F0.5Cl0.3I0.2', '-1.347', '-0.22190437305']
ClFIn 17 ['F0.111111Cl0.444444In0.444444', '-1.506', '-0.201170947602']
ClFIr 36 ['F0.555556Cl0.333333Ir0.111111', '-1.445', '-0.205195736431']
ClFK 23 ['F0.25Cl0.375K0.375', '-2.194', '-0.202215747637']
ClFKr 2 ['F0.777778Cl0.111111Kr0.111111', '-0.743', '-0.213878292636']
ClFLa 2 ['F0.7Cl0.1La0.2', '-3.931', '-0.211752241131']
ClFLi 4 ['Li0.125F0.75Cl0.125', '-1.571', '-0.305218455981']
ClFLu 59 ['F0.666667Cl0.166667Lu0.166667', '-3.139', '-0.215075543392']
ClFMg 3 ['F0.777778Mg0.111111Cl0.111111', '-1.95', '-0.250442228839']
ClFMn 1 ['F0.571429Cl0.142857Mn0.285714', '-2.611', '-0.220630737419']
ClFN 3 ['N0.125F0.75Cl0.125', '-0.741', '-0.262687645874']
ClFNa 10 ['F0.5Na0.25Cl0.25', '-2.032', '-0.205349633193']
ClFNb 2 ['F0.6Cl0.2Nb0.2', '-2.975', '-0.204282258727']
ClFNd 3 ['F0.8Cl0.1Nd0.1', '-2.341', '-0.201557345291']
ClFNi 22 ['F0.5Cl0.166667Ni0.333333', '-1.853', '-0.200364608665']
ClFNp 8 ['F0.166667Cl0.666667Np0.166667', '-2.158', '-0.201871575596']
ClFO 7 ['O0.142857F0.714286Cl0.142857', '-0.752', '-0.20535730957']
ClFOs 15 ['F0.444444Cl0.444444Os0.111111', '-1.345', '-0.208193925662']
ClFPa 24 ['F0.444444Cl0.444444Pa0.111111', '-2.126', '-0.233080147084']
ClFPb 10 ['F0.3Cl0.5Pb0.2', '-1.62', '-0.201075420887']
ClFPd 20 ['F0.5Cl0.375Pd0.125', '-1.135', '-0.203424114931']
ClFPm 21 ['F0.142857Cl0.571429Pm0.285714', '-2.634', '-0.210057613818']
ClFPt 47 ['F0.3Cl0.4Pt0.3', '-1.127', '-0.203143935056']
ClFPu 21 ['F0.555556Cl0.333333Pu0.111111', '-2.237', '-0.200457641456']
ClFRb 11 ['F0.285714Cl0.285714Rb0.428571', '-2.442', '-0.200093395157']
ClFRe 1 ['F0.75Cl0.125Re0.125', '-2.319', '-0.308014038643']
ClFRh 43 ['F0.75Cl0.125Rh0.125', '-1.576', '-0.200498746198']
ClFRu 38 ['F0.166667Cl0.666667Ru0.166667', '-1.0', '-0.202071924706']
ClFS 12 ['F0.222222S0.222222Cl0.555556', '-0.982', '-0.223898882684']
ClFSb 2 ['F0.714286Cl0.142857Sb0.142857', '-2.284', '-0.243130737601']
ClFSc 1 ['F0.8Cl0.1Sc0.1', '-2.362', '-0.271416672459']
ClFSe 1 ['F0.2Cl0.5Se0.3', '-1.106', '-0.222412381491']
ClFSm 2 ['F0.111111Cl0.666667Sm0.222222', '-2.313', '-0.236516894012']
ClFSn 2 ['F0.8Cl0.1Sn0.1', '-1.914', '-0.201604147359']
ClFSr 1 ['F0.8Cl0.1Sr0.1', '-1.91', '-0.265553819872']
ClFTb 1 ['F0.428571Cl0.285714Tb0.285714', '-3.988', '-0.636938532061']
ClFTc 17 ['F0.4Cl0.4Tc0.2', '-1.815', '-0.221825337852']
ClFTe 1 ['F0.714286Cl0.142857Te0.142857', '-2.228', '-0.238531460384']
ClFTh 1 ['F0.5Cl0.333333Th0.166667', '-3.578', '-0.280060294081']
ClFTi 10 ['F0.666667Cl0.222222Ti0.111111', '-2.257', '-0.214259143495']
ClFTl 19 ['F0.428571Cl0.285714Tl0.285714', '-1.83', '-0.200767496624']
ClFTm 60 ['F0.3Cl0.2Tm0.5', '-2.351', '-0.215669397806']

ClFV 3 ['F0.75Cl0.125V0.125', '-2.398', '-0.200493544796']
ClFXe 16 ['F0.5Cl0.125Xe0.375', '-0.815', '-0.202113580357']
ClFY 8 ['F0.5Cl0.25Y0.25', '-3.852', '-0.202161883333']
ClFYb 16 ['F0.4Cl0.1Yb0.5', '-3.0', '-0.2321006965']
ClFZn 1 ['F0.8Cl0.1Zn0.1', '-1.429', '-0.276215930874']
ClFeGd 2 ['Cl0.75Fe0.125Gd0.125', '-1.954', '-0.205745209848']
ClFeGe 1 ['Cl0.666667Fe0.166667Ge0.166667', '-1.233', '-0.222114194417']
ClFeH 3 ['H0.5Cl0.333333Fe0.166667', '-0.679', '-0.200716244794']
ClFeHg 1 ['Cl0.7Fe0.2Hg0.1', '-1.14', '-0.22096478357']
ClFeHo 27 ['Cl0.4Fe0.2Ho0.4', '-1.33', '-0.246984952416']
ClFeI 2 ['Cl0.5Fe0.3I0.2', '-0.952', '-0.202543958947']
ClFeLi 4 ['Li0.25Cl0.625Fe0.125', '-1.701', '-0.217442760238']
ClFeLu 27 ['Cl0.375Fe0.125Lu0.5', '-1.167', '-0.20446591758']
ClFeNp 2 ['Cl0.8Fe0.1Np0.1', '-1.577', '-0.253945583689']
ClFeO 3 ['O0.428571Cl0.142857Fe0.428571', '-1.689', '-0.204092229497']
ClFePm 15 ['Cl0.5Fe0.125Pm0.375', '-1.705', '-0.212551742503']
ClFePr 1 ['Cl0.777778Fe0.111111Pr0.111111', '-1.785', '-0.209951653754']
ClFePt 5 ['Cl0.777778Fe0.111111Pt0.111111', '-0.846', '-0.214582092804']
ClFePu 4 ['Cl0.714286Fe0.142857Pu0.142857', '-1.856', '-0.20294710956']
ClFeRh 10 ['Cl0.6Fe0.1Rh0.3', '-0.589', '-0.202092039314']
ClFeRu 3 ['Cl0.714286Fe0.142857Ru0.142857', '-1.071', '-0.203144351701']
ClFeS 1 ['S0.1Cl0.8Fe0.1', '-0.717', '-0.205212913539']
ClFeSc 1 ['Cl0.777778Sc0.111111Fe0.111111', '-1.702', '-0.210995283478']
ClFeSe 6 ['Cl0.571429Fe0.285714Se0.142857', '-1.04', '-0.205418276789']
ClFeSm 10 ['Cl0.625Fe0.25Sm0.125', '-1.522', '-0.203620518989']
ClFeTb 2 ['Cl0.75Fe0.125Tb0.125', '-1.885', '-0.213528528328']
ClFeTc 2 ['Cl0.7Fe0.2Tc0.1', '-1.166', '-0.200628916067']
ClFeTe 8 ['Cl0.3Fe0.2Te0.5', '-0.665', '-0.207579038231']
ClFeTh 1 ['Cl0.8Fe0.1Th0.1', '-1.89', '-0.278596594564']
ClFeXe 4 ['Cl0.6Fe0.1Xe0.3', '-0.587', '-0.200092039314']
ClFeY 2 ['Cl0.8Fe0.1Y0.1', '-1.647', '-0.23877798313']
ClGaGe 1 ['Cl0.6Ga0.3Ge0.1', '-1.354', '-0.218619743881']
ClGaHg 1 ['Cl0.555556Ga0.111111Hg0.333333', '-1.18', '-0.26234766911']
ClGaHo 37 ['Cl0.6Ga0.3Ho0.1', '-1.444', '-0.203189057869']
ClGaIr 1 ['Cl0.75Ga0.125Ir0.125', '-0.945', '-0.260193570395']
ClGaLu 52 ['Cl0.555556Ga0.333333Lu0.111111', '-1.373', '-0.208987714381']
ClGaN 3 ['N0.166667Cl0.5Ga0.333333', '-1.309', '-0.202834641303']
ClGaNd 1 ['Cl0.4Ga0.2Nd0.4', '-1.847', '-0.242708462755']
ClGaNp 4 ['Cl0.777778Ga0.111111Np0.111111', '-1.862', '-0.213120445212']
ClGaO 5 ['O0.2Cl0.5Ga0.3', '-1.826', '-0.212641445017']
ClGaPa 3 ['Cl0.571429Ga0.142857Pa0.285714', '-1.745', '-0.207834654643']
ClGaPb 3 ['Cl0.625Ga0.25Pb0.125', '-1.452', '-0.219842382272']
ClGaPm 45 ['Cl0.25Ga0.5Pm0.25', '-1.097', '-0.203129192137']
ClGaPt 3 ['Cl0.625Ga0.25Pt0.125', '-1.43', '-0.21308425045']

ClGaPu 19 ['Cl0.7Ga0.1Pu0.2', '-2.131', '-0.201436416946']
ClGaRb 2 ['Cl0.5Ga0.166667Rb0.333333', '-1.972', '-0.206097199274']
ClGaRh 10 ['Cl0.625Ga0.25Rh0.125', '-1.408', '-0.209135245867']
ClGaRu 4 ['Cl0.7Ga0.1Ru0.2', '-1.18', '-0.204783255402']
ClGaSb 1 ['Cl0.6Ga0.3Sb0.1', '-1.415', '-0.279619743881']
ClGaSe 17 ['Cl0.666667Ga0.222222Se0.111111', '-1.447', '-0.229566347369']
ClGaSi 1 ['Si0.111111Cl0.555556Ga0.333333', '-1.274', '-0.215382536182']
ClGaSm 16 ['Cl0.666667Ga0.222222Sm0.111111', '-1.736', '-0.201368471257']
ClGaTa 2 ['Cl0.714286Ga0.142857Ta0.142857', '-1.609', '-0.203534546234']
ClGaTb 6 ['Cl0.5Ga0.2Tb0.3', '-1.923', '-0.20822758678']
ClGaTc 1 ['Cl0.8Ga0.1Tc0.1', '-1.244', '-0.220337423194']
ClGaTe 5 ['Cl0.444444Ga0.333333Te0.222222', '-1.191', '-0.213035730929']
ClGaTm 2 ['Cl0.333333Ga0.222222Tm0.444444', '-1.65', '-0.248771543129']
ClGaXe 2 ['Cl0.666667Ga0.111111Xe0.222222', '-0.811', '-0.202283173684']
ClGaY 22 ['Cl0.375Ga0.125Y0.5', '-1.676', '-0.207363367164']
ClGdGe 1 ['Cl0.8Ge0.1Gd0.1', '-1.82', '-0.205204808564']
ClGdH 1 ['H0.333333Cl0.333333Gd0.333333', '-1.686', '-0.209868120732']
ClGdHo 6 ['Cl0.666667Gd0.111111Ho0.222222', '-2.111', '-0.385218449747']
ClGdI 48 ['Cl0.5I0.333333Gd0.166667', '-1.888', '-0.201840214273']
ClGdIn 6 ['Cl0.444444In0.111111Gd0.444444', '-1.853', '-0.211090371207']
ClGdIr 8 ['Cl0.428571Gd0.428571Ir0.142857', '-1.89', '-0.203244783664']
ClGdK 1 ['Cl0.714286K0.142857Gd0.142857', '-2.263', '-0.205977896431']
ClGdKr 1 ['Cl0.7Kr0.1Gd0.2', '-2.23', '-0.206608257128']
ClGdLu 6 ['Cl0.666667Gd0.111111Lu0.222222', '-2.126', '-0.450408362711']
ClGdMn 1 ['Cl0.777778Mn0.111111Gd0.111111', '-1.796', '-0.230326402289']
ClGdMo 1 ['Cl0.777778Mo0.111111Gd0.111111', '-1.997', '-0.259576361866']
ClGdN 9 ['N0.3Cl0.3Gd0.4', '-2.293', '-0.20095019971']
ClGdNi 3 ['Cl0.4Ni0.2Gd0.4', '-1.765', '-0.231920794753']
ClGdNp 2 ['Cl0.777778Gd0.111111Np0.111111', '-2.386', '-0.22173074771']
ClGdO 14 ['O0.25Cl0.25Gd0.5', '-2.711', '-0.202898270247']
ClGdOs 1 ['Cl0.3Gd0.5Os0.2', '-1.313', '-0.201231417065']
ClGdP 1 ['P0.1Cl0.8Gd0.1', '-1.801', '-0.242805643358']
ClGdPb 1 ['Cl0.777778Gd0.111111Pb0.111111', '-1.824', '-0.204451614241']
ClGdPm 1 ['Cl0.777778Pm0.111111Gd0.111111', '-2.321', '-0.257240989587']
ClGdPr 1 ['Cl0.777778Pr0.111111Gd0.111111', '-2.502', '-0.232742864031']
ClGdPt 6 ['Cl0.666667Gd0.111111Pt0.222222', '-1.629', '-0.202613216531']
ClGdPu 3 ['Cl0.714286Gd0.142857Pu0.142857', '-2.518', '-0.243989926345']
ClGdRb 1 ['Cl0.714286Rb0.142857Gd0.142857', '-2.35', '-0.295603095717']
ClGdRe 2 ['Cl0.777778Gd0.111111Re0.111111', '-1.811', '-0.210365374796']
ClGdRh 9 ['Cl0.6Rh0.3Gd0.1', '-1.276', '-0.264304128564']
ClGdRu 4 ['Cl0.714286Ru0.142857Gd0.142857', '-1.981', '-0.230454754438']
ClGdS 10 ['S0.3Cl0.2Gd0.5', '-2.189', '-0.200650676087']
ClGdSe 27 ['Cl0.6Se0.2Gd0.2', '-2.227', '-0.203608257128']
ClGdSi 24 ['Si0.111111Cl0.444444Gd0.444444', '-1.926', '-0.207995513846']

ClGdSm 3 ['Cl0.75Sm0.125Gd0.125', '-2.506', '-0.28195071266']
ClGdTa 1 ['Cl0.8Gd0.1Ta0.1', '-2.118', '-0.314909025314']
ClGdTb 1 ['Cl0.777778Gd0.111111Tb0.111111', '-2.431', '-0.251038791013']
ClGdTc 3 ['Cl0.7Tc0.2Gd0.1', '-1.747', '-0.21339669165']
ClGdTe 22 ['Cl0.166667Te0.333333Gd0.5', '-1.668', '-0.202264007673']
ClGdTh 1 ['Cl0.8Gd0.1Th0.1', '-2.492', '-0.255808683814']
ClGdTl 7 ['Cl0.428571Gd0.285714Tl0.285714', '-1.772', '-0.20359467402']
ClGdV 1 ['Cl0.8V0.1Gd0.1', '-1.923', '-0.210231417314']
ClGdXe 2 ['Cl0.666667Xe0.222222Gd0.111111', '-1.331', '-0.206893476182']
ClGdY 1 ['Cl0.8Y0.1Gd0.1', '-2.235', '-0.20199007238']
ClGeHg 1 ['Cl0.666667Ge0.222222Hg0.111111', '-1.21', '-0.204834466667']
ClGeHo 51 ['Cl0.4Ge0.4Ho0.2', '-1.171', '-0.200771446']
ClGeK 2 ['Cl0.625K0.125Ge0.25', '-1.505', '-0.215851348672']
ClGeLa 8 ['Cl0.25Ge0.25La0.5', '-1.587', '-0.209622961097']
ClGeLu 54 ['Cl0.5Ge0.375Lu0.125', '-1.167', '-0.20000155625']
ClGeNa 1 ['Na0.3Cl0.6Ge0.1', '-1.89', '-0.207610627056']
ClGeNi 9 ['Cl0.7Ni0.1Ge0.2', '-1.288', '-0.201627705238']
ClGeNp 10 ['Cl0.555556Ge0.111111Np0.333333', '-1.725', '-0.213709419935']
ClGeO 2 ['O0.6Cl0.1Ge0.3', '-1.962', '-0.239093370235']
ClGePa 9 ['Cl0.555556Ge0.111111Pa0.333333', '-1.694', '-0.200066658542']
ClGePd 1 ['Cl0.666667Ge0.222222Pd0.111111', '-1.316', '-0.220434813056']
ClGePm 45 ['Cl0.333333Ge0.111111Pm0.555556', '-1.367', '-0.202698482016']
ClGePr 1 ['Cl0.714286Ge0.142857Pr0.142857', '-2.115', '-0.211878270091']
ClGePt 17 ['Cl0.6Ge0.1Pt0.3', '-0.985', '-0.200532524209']
ClGePu 26 ['Cl0.5Ge0.3Pu0.2', '-1.701', '-0.201164834193']
ClGeRb 1 ['Cl0.714286Ge0.142857Rb0.142857', '-1.71', '-0.239312454911']
ClGeRh 7 ['Cl0.625Ge0.125Rh0.25', '-0.959', '-0.20512585']
ClGeRu 3 ['Cl0.75Ge0.125Ru0.125', '-1.258', '-0.237018599429']
ClGeSb 1 ['Cl0.625Ge0.25Sb0.125', '-1.19', '-0.24543232758']
ClGeSc 11 ['Cl0.5Sc0.25Ge0.25', '-1.935', '-0.200976863443']
ClGeSm 28 ['Cl0.375Ge0.375Sm0.25', '-1.497', '-0.202649866905']
ClGeTb 18 ['Cl0.714286Ge0.142857Tb0.142857', '-1.994', '-0.205687319069']
ClGeTe 16 ['Cl0.375Ge0.25Te0.375', '-0.794', '-0.201157004948']
ClGeTm 1 ['Cl0.4Ge0.2Tm0.4', '-1.964', '-0.239405632588']
ClGeU 3 ['Cl0.625Ge0.125U0.25', '-1.878', '-0.20591117045']
ClGeY 17 ['Cl0.8Ge0.1Y0.1', '-1.83', '-0.205586623816']
ClGeYb 10 ['Cl0.5Ge0.166667Yb0.333333', '-2.378', '-0.20145491048']
ClHHg 1 ['H0.5Cl0.3Hg0.2', '-0.615', '-0.202387563102']
ClHHo 64 ['H0.6Cl0.1Ho0.3', '-0.968', '-0.211510347794']
ClHIn 1 ['H0.142857Cl0.571429In0.285714', '-1.26', '-0.21698035638']
ClHIr 4 ['H0.2Cl0.6Ir0.2', '-0.48', '-0.210859034439']
ClHLa 16 ['H0.428571Cl0.142857La0.428571', '-1.124', '-0.206445022548']
ClHLu 43 ['H0.571429Cl0.142857Lu0.285714', '-1.142', '-0.207629853263']
ClHMo 2 ['H0.333333Cl0.5Mo0.166667', '-1.013', '-0.203244601968']

ClHNd 1 ['H0.5Cl0.166667Nd0.333333', '-1.244', '-0.202574339222']
ClHNi 12 ['H0.571429Cl0.142857Ni0.285714', '-0.427', '-0.206242866833']
ClHNp 3 ['H0.3Cl0.5Np0.2', '-1.569', '-0.20994016689']
ClHP 7 ['H0.5P0.25Cl0.25', '-0.556', '-0.216358454307']
ClHPa 8 ['H0.4Cl0.3Pa0.3', '-1.174', '-0.212270932439']
ClHPm 54 ['H0.5Cl0.3Pm0.2', '-1.208', '-0.213603233159']
ClHPr 15 ['H0.285714Cl0.428571Pr0.285714', '-1.94', '-0.204772838396']
ClHPt 9 ['H0.4Cl0.3Pt0.3', '-0.607', '-0.203288551659']
ClHPu 16 ['H0.1Cl0.7Pu0.2', '-2.079', '-0.204095976852']
ClHRh 10 ['H0.142857Cl0.714286Rh0.142857', '-0.416', '-0.223756453171']
ClHRu 12 ['H0.333333Cl0.5Ru0.166667', '-0.836', '-0.209360223684']
ClHSc 1 ['H0.5Cl0.1Sc0.4', '-1.048', '-0.200735686847']
ClHSm 29 ['H0.428571Cl0.285714Sm0.285714', '-1.421', '-0.202305509007']
ClHTb 4 ['H0.2Cl0.5Tb0.3', '-1.934', '-0.209722729343']
ClHTe 10 ['H0.4Cl0.3Te0.3', '-0.62', '-0.216288551659']
ClHTm 5 ['H0.5Cl0.1Tm0.4', '-1.065', '-0.210612852847']
ClHW 1 ['H0.333333Cl0.5W0.166667', '-0.904', '-0.209807288437']
ClHZr 1 ['H0.2Cl0.6Zr0.2', '-1.839', '-0.208995112657']
ClHfHo 5 ['Cl0.666667Ho0.222222Hf0.111111', '-1.988', '-0.210629297176']
ClHfLu 5 ['Cl0.666667Lu0.222222Hf0.111111', '-1.934', '-0.20681921014']
ClHfO 1 ['O0.3Cl0.4Hf0.3', '-3.003', '-0.217256852234']
ClHfPm 1 ['Cl0.7Pm0.2Hf0.1', '-2.21', '-0.254093996941']
ClHfPu 1 ['Cl0.8Hf0.1Pu0.1', '-2.158', '-0.229707121066']
ClHfRh 5 ['Cl0.8Rh0.1Hf0.1', '-1.262', '-0.20387389125']
ClHfRu 1 ['Cl0.777778Ru0.111111Hf0.111111', '-1.854', '-0.322161322849']
ClHfTe 10 ['Cl0.5Te0.333333Hf0.166667', '-1.647', '-0.211320278785']
ClHgHo 42 ['Cl0.333333Ho0.444444Hg0.222222', '-1.235', '-0.221813834428']
ClHgIn 5 ['Cl0.6In0.1Hg0.3', '-1.087', '-0.204431059439']
ClHgIr 5 ['Cl0.625Ir0.125Hg0.25', '-0.815', '-0.202107493449']
ClHgLu 45 ['Cl0.3Lu0.3Hg0.4', '-0.955', '-0.210495096814']
ClHgMo 4 ['Cl0.666667Mo0.222222Hg0.111111', '-1.261', '-0.207974983668']
ClHgNd 2 ['Cl0.5Nd0.375Hg0.125', '-2.029', '-0.206633030948']
ClHgNp 3 ['Cl0.625Hg0.125Np0.25', '-1.865', '-0.201174707167']
ClHgP 9 ['P0.1Cl0.5Hg0.4', '-0.916', '-0.211146620908']
ClHgPd 3 ['Cl0.555556Pd0.222222Hg0.222222', '-0.876', '-0.212325314108']
ClHgPm 29 ['Cl0.7Pm0.1Hg0.2', '-1.537', '-0.200998756823']
ClHgPr 12 ['Cl0.571429Pr0.142857Hg0.285714', '-1.886', '-0.215062102865']
ClHgPu 37 ['Cl0.25Hg0.375Pu0.375', '-0.981', '-0.201258671318']
ClHgRh 17 ['Cl0.6Rh0.1Hg0.3', '-0.97', '-0.234528992139']
ClHgRu 1 ['Cl0.6Ru0.1Hg0.3', '-0.965', '-0.227408368139']
ClHgSb 1 ['Cl0.666667Sb0.111111Hg0.222222', '-1.297', '-0.365575047094']
ClHgSm 14 ['Cl0.555556Sm0.111111Hg0.333333', '-1.398', '-0.236238319385']
ClHgSn 4 ['Cl0.625Sn0.125Hg0.25', '-1.173', '-0.203239989232']
ClHgTb 1 ['Cl0.5Tb0.333333Hg0.166667', '-1.847', '-0.222585875641']

ClHgTc 1 ['Cl0.6Tc0.1Hg0.3', '-1.014', '-0.205506491139']
ClHgTe 4 ['Cl0.5Te0.125Hg0.375', '-0.887', '-0.212626245849']
ClHgXe 5 ['Cl0.6Xe0.2Hg0.2', '-0.701', '-0.210685994759']
ClHgY 5 ['Cl0.428571Y0.428571Hg0.142857', '-1.826', '-0.210127289026']
ClHoI 78 ['Cl0.375I0.125Ho0.5', '-1.417', '-0.209365071267']
ClHoIn 52 ['Cl0.444444In0.444444Ho0.111111', '-1.206', '-0.20075406365']
ClHoIr 40 ['Cl0.5Ho0.1Ir0.4', '-0.508', '-0.237246238104']
ClHoK 19 ['Cl0.555556K0.222222Ho0.222222', '-2.007', '-0.453725860093']
ClHoKr 36 ['Cl0.375Kr0.375Ho0.25', '-0.915', '-0.23811559526']
ClHoLa 6 ['Cl0.666667La0.111111Ho0.222222', '-2.159', '-0.364283081693']
ClHoLi 19 ['Li0.333333Cl0.555556Ho0.111111', '-2.011', '-0.376932768242']
ClHoLu 19 ['Cl0.555556Ho0.333333Lu0.111111', '-1.61', '-0.431744903611']
ClHoMg 14 ['Mg0.166667Cl0.5Ho0.333333', '-1.704', '-0.201544031773']
ClHoMn 27 ['Cl0.6Mn0.3Ho0.1', '-1.474', '-0.209720321844']
ClHoMo 14 ['Cl0.6Mo0.2Ho0.2', '-1.451', '-0.20692696413']
ClHoN 67 ['N0.5Cl0.375Ho0.125', '-0.683', '-0.220884066415']
ClHoNa 15 ['Na0.2Cl0.7Ho0.1', '-1.852', '-0.257308416395']
ClHoNb 10 ['Cl0.6Nb0.1Ho0.3', '-1.735', '-0.309577438125']
ClHoNd 6 ['Cl0.666667Nd0.111111Ho0.222222', '-2.079', '-0.336948541693']
ClHoNi 35 ['Cl0.375Ni0.375Ho0.25', '-1.099', '-0.232528630467']
ClHoNp 8 ['Cl0.666667Ho0.166667Np0.166667', '-1.985', '-0.202683495907']
ClHoO 32 ['O0.25Cl0.5Ho0.25', '-2.709', '-0.20097646254']
ClHoOs 34 ['Cl0.3Ho0.4Os0.3', '-1.038', '-0.213396974979']
ClHoP 59 ['P0.3Cl0.2Ho0.5', '-1.803', '-0.217107212518']
ClHoPa 6 ['Cl0.714286Ho0.142857Pa0.142857', '-2.063', '-0.212561908363']
ClHoPb 32 ['Cl0.3Ho0.4Pb0.3', '-1.161', '-0.213242980618']
ClHoPd 47 ['Cl0.222222Pd0.333333Ho0.444444', '-1.321', '-0.205480161296']
ClHoPm 12 ['Cl0.6Pm0.3Ho0.1', '-1.943', '-0.209667010544']
ClHoPr 6 ['Cl0.666667Pr0.111111Ho0.222222', '-2.177', '-0.430174361413']
ClHoPt 51 ['Cl0.2Ho0.5Pt0.3', '-1.523', '-0.200465737206']
ClHoPu 21 ['Cl0.555556Ho0.333333Pu0.111111', '-1.782', '-0.213473006693']
ClHoRb 19 ['Cl0.555556Rb0.333333Ho0.111111', '-2.115', '-0.392889281576']
ClHoRe 13 ['Cl0.5Ho0.4Re0.1', '-1.438', '-0.229585456132']
ClHoRh 49 ['Cl0.222222Rh0.333333Ho0.444444', '-1.277', '-0.221277980602']
ClHoRu 30 ['Cl0.333333Ru0.222222Ho0.444444', '-1.184', '-0.204826017754']
ClHoS 75 ['S0.3Cl0.1Ho0.6', '-1.758', '-0.215997468902']
ClHoSb 47 ['Cl0.571429Sb0.285714Ho0.142857', '-1.353', '-0.206122258544']
ClHoSc 6 ['Cl0.666667Sc0.111111Ho0.222222', '-2.131', '-0.468217991138']
ClHoSe 74 ['Cl0.444444Se0.444444Ho0.111111', '-1.096', '-0.222667556856']
ClHoSi 36 ['Si0.3Cl0.5Ho0.2', '-1.477', '-0.207553397671']
ClHoSm 10 ['Cl0.625Sm0.125Ho0.25', '-1.873', '-0.236686147215']
ClHoSn 44 ['Cl0.3Sn0.2Ho0.5', '-1.298', '-0.204830439132']
ClHoSr 11 ['Cl0.6Sr0.1Ho0.3', '-1.972', '-0.339483613189']
ClHoTa 9 ['Cl0.625Ho0.25Ta0.125', '-1.621', '-0.202182685963']

ClHoTb 6 ['Cl0.666667Tb0.111111Ho0.222222', '-2.035', '-0.377470288396']
ClHoTc 25 ['Cl0.444444Tc0.111111Ho0.444444', '-1.42', '-0.216649947129']
ClHoTe 68 ['Cl0.375Te0.125Ho0.5', '-1.539', '-0.212712077057']
ClHoTh 4 ['Cl0.7Ho0.2Th0.1', '-2.188', '-0.421997031458']
ClHoTi 11 ['Cl0.666667Ti0.166667Ho0.166667', '-1.94', '-0.242277659233']
ClHoTl 48 ['Cl0.333333Ho0.5Tl0.166667', '-1.213', '-0.201881355624']
ClHoTm 6 ['Cl0.666667Ho0.222222Tm0.111111', '-2.052', '-0.331172245027']
ClHoU 6 ['Cl0.666667Ho0.222222U0.111111', '-1.949', '-0.248671083287']
ClHoV 11 ['Cl0.666667V0.222222Ho0.111111', '-1.737', '-0.321042335328']
ClHoW 12 ['Cl0.571429Ho0.285714W0.142857', '-1.427', '-0.25673627369']
ClHoXe 37 ['Cl0.4Xe0.5Ho0.1', '-0.507', '-0.236246238104']
ClHoY 6 ['Cl0.666667Y0.111111Ho0.222222', '-2.134', '-0.397531577804']
ClHoYb 11 ['Cl0.6Ho0.3Yb0.1', '-1.97', '-0.338996832689']
ClHoZn 30 ['Cl0.444444Zn0.333333Ho0.222222', '-1.358', '-0.244666774976']
ClHoZr 6 ['Cl0.714286Zr0.142857Ho0.142857', '-2.091', '-0.231548443363']
ClIIn 24 ['Cl0.4In0.4I0.2', '-1.179', '-0.200802317724']
ClIIr 20 ['Cl0.5I0.25Ir0.25', '-0.417', '-0.210211972675']
ClIKr 3 ['Cl0.666667Kr0.222222I0.111111', '-0.294', '-0.202094210078']
ClILa 5 ['Cl0.777778I0.111111La0.111111', '-1.493', '-0.208052318207']
ClILu 82 ['Cl0.1I0.8Lu0.1', '-0.53', '-0.281831698938']
ClINa 13 ['Na0.4Cl0.2I0.4', '-1.597', '-0.210510562048']
ClINd 18 ['Cl0.285714I0.285714Nd0.428571', '-1.81', '-0.223799839843']
ClINi 12 ['Cl0.428571Ni0.285714I0.285714', '-0.793', '-0.209210585817']
ClINp 17 ['Cl0.444444I0.333333Np0.222222', '-1.689', '-0.200900487523']
ClIO 13 ['O0.142857Cl0.571429I0.285714', '-0.514', '-0.20668609461']
ClIOs 1 ['Cl0.333333I0.5Os0.166667', '-0.481', '-0.20344966396']
ClIPa 41 ['Cl0.5I0.2Pa0.3', '-1.562', '-0.225837385702']
ClIPm 78 ['Cl0.375I0.125Pm0.5', '-1.582', '-0.201671645089']
ClIPr 7 ['Cl0.25I0.5Pr0.25', '-1.955', '-0.201671239876']
ClIPt 20 ['Cl0.625I0.125Pt0.25', '-0.759', '-0.202185596861']
ClIPu 13 ['Cl0.5I0.1Pu0.4', '-1.815', '-0.203664287451']
ClIRb 7 ['Cl0.666667Rb0.111111I0.222222', '-0.861', '-0.203430685087']
ClIRe 1 ['Cl0.8I0.1Re0.1', '-0.725', '-0.213409497823']
ClIRh 47 ['Cl0.2Rh0.2I0.6', '-0.372', '-0.20656957814']
ClIRu 8 ['Cl0.5Ru0.2I0.3', '-0.739', '-0.204785498857']
ClISb 4 ['Cl0.428571Sb0.142857I0.428571', '-0.869', '-0.220279317234']
ClISc 10 ['Cl0.2Sc0.3I0.5', '-1.711', '-0.212492315241']
ClISe 13 ['Cl0.222222Se0.333333I0.444444', '-0.385', '-0.201188420156']
ClISi 1 ['Si0.2Cl0.4I0.4', '-1.236', '-0.216957450778']
ClISm 61 ['Cl0.1I0.6Sm0.3', '-1.455', '-0.204186798228']
ClITa 4 ['Cl0.714286I0.142857Ta0.142857', '-1.485', '-0.236270979743']
ClITb 54 ['Cl0.5I0.333333Tb0.166667', '-1.792', '-0.208217972247']
ClITe 7 ['Cl0.444444Te0.111111I0.444444', '-0.701', '-0.200603255289']
ClITh 1 ['Cl0.8I0.1Th0.1', '-1.51', '-0.20278934432']

ClITm 15 ['Cl0.285714I0.428571Tm0.285714', '-1.881', '-0.206291490802']
ClIXe 46 ['Cl0.25I0.375Xe0.375', '-0.407', '-0.200211972676']
ClIY 1 ['Cl0.428571Y0.428571I0.142857', '-2.003', '-0.226946517722']
ClIZr 18 ['Cl0.571429Zr0.285714I0.142857', '-1.977', '-0.200788561324']
ClInIr 7 ['Cl0.666667In0.166667Ir0.166667', '-0.785', '-0.221428702149']
ClInK 3 ['Cl0.666667K0.166667In0.166667', '-1.622', '-0.202661491227']
ClInLa 11 ['Cl0.444444In0.222222La0.333333', '-2.023', '-0.215678153153']
ClInLi 1 ['Li0.1Cl0.7In0.2', '-1.398', '-0.257290162692']
ClInLu 49 ['Cl0.25In0.25Lu0.5', '-1.022', '-0.204034171095']
ClInMg 1 ['Mg0.142857Cl0.714286In0.142857', '-1.598', '-0.213910574642']
ClInN 7 ['N0.125Cl0.5In0.375', '-1.19', '-0.205497534481']
ClInNa 3 ['Na0.1Cl0.5In0.4', '-1.425', '-0.204229277976']
ClInNd 11 ['Cl0.333333In0.222222Nd0.444444', '-1.586', '-0.21362749424']
ClInNp 8 ['Cl0.5In0.25Np0.25', '-1.54', '-0.207470825838']
ClInO 7 ['O0.111111Cl0.444444In0.444444', '-1.428', '-0.200659902877']
ClInPb 1 ['Cl0.7In0.1Pb0.2', '-1.406', '-0.215068263268']
ClInPm 48 ['Cl0.4In0.1Pm0.5', '-1.538', '-0.202782256502']
ClInPr 6 ['Cl0.8In0.1Pr0.1', '-1.577', '-0.208221670353']
ClInPt 23 ['Cl0.4In0.4Pt0.2', '-1.149', '-0.211361599754']
ClInPu 19 ['Cl0.571429In0.285714Pu0.142857', '-1.74', '-0.207351366031']
ClInRb 9 ['Cl0.7Rb0.2In0.1', '-1.416', '-0.225093298165']
ClInRe 1 ['Cl0.8In0.1Re0.1', '-0.982', '-0.214981930042']
ClInRh 17 ['Cl0.5Rh0.1In0.4', '-1.251', '-0.214782254358']
ClInRu 2 ['Cl0.75Ru0.125In0.125', '-1.038', '-0.214660650755']
ClInS 4 ['S0.125Cl0.75In0.125', '-0.804', '-0.225222619393']
ClInSc 2 ['Cl0.75Sc0.125In0.125', '-1.834', '-0.217576171382']
ClInSe 21 ['Cl0.285714Se0.428571In0.285714', '-0.898', '-0.206565040142']
ClInSi 1 ['Si0.111111Cl0.777778In0.111111', '-1.448', '-0.223923940599']
ClInSm 38 ['Cl0.25In0.25Sm0.5', '-1.248', '-0.209325935159']
ClInSr 13 ['Cl0.444444Sr0.111111In0.444444', '-1.564', '-0.202189279654']
ClInTa 2 ['Cl0.777778In0.111111Ta0.111111', '-1.563', '-0.307957908932']
ClInTb 17 ['Cl0.5In0.1Tb0.4', '-1.834', '-0.214475931424']
ClInTc 1 ['Cl0.8Tc0.1In0.1', '-1.064', '-0.250039788167']
ClInTe 1 ['Cl0.571429In0.285714Te0.142857', '-1.243', '-0.213240691208']
ClInTh 2 ['Cl0.777778In0.111111Th0.111111', '-1.999', '-0.262735307266']
ClInTi 1 ['Cl0.777778Ti0.111111In0.111111', '-1.667', '-0.267252072127']
ClInTl 3 ['Cl0.625In0.25Tl0.125', '-1.422', '-0.236624256115']
ClInTm 6 ['Cl0.428571In0.142857Tm0.428571', '-1.79', '-0.202003147594']
ClInU 1 ['Cl0.8In0.1U0.1', '-1.802', '-0.205881025085']
ClInXe 11 ['Cl0.5In0.1Xe0.4', '-0.547', '-0.208857221289']
ClInY 23 ['Cl0.444444Y0.222222In0.333333', '-1.845', '-0.204787314172']
ClInYb 2 ['Cl0.714286In0.142857Yb0.142857', '-1.869', '-0.216307628095']
ClInZr 2 ['Cl0.8Zr0.1In0.1', '-1.641', '-0.271994893539']
ClIrK 11 ['Cl0.6K0.1Ir0.3', '-0.631', '-0.202780398938']

ClIrKr 9 ['Cl0.571429Kr0.285714Ir0.142857', '-0.205', '-0.205']
ClIrLa 6 ['Cl0.666667La0.111111Ir0.222222', '-1.431', '-0.237958108129']
ClIrLi 13 ['Li0.285714Cl0.571429Ir0.142857', '-1.383', '-0.240231669823']
ClIrLu 42 ['Cl0.5Lu0.125Ir0.375', '-0.516', '-0.2040551925']
ClIrMg 3 ['Mg0.1Cl0.8Ir0.1', '-0.858', '-0.22728018096']
ClIrN 20 ['N0.444444Cl0.222222Ir0.333333', '-0.202', '-0.202']
ClIrNa 11 ['Na0.2Cl0.6Ir0.2', '-1.027', '-0.206956744704']
ClIrNd 22 ['Cl0.666667Nd0.166667Ir0.166667', '-1.913', '-0.202435352193']
ClIrNp 3 ['Cl0.75Ir0.125Np0.125', '-1.392', '-0.221816930469']
ClIrO 17 ['O0.166667Cl0.5Ir0.333333', '-0.493', '-0.226919683603']
ClIrP 1 ['P0.1Cl0.8Ir0.1', '-0.759', '-0.212501514794']
ClIrPa 3 ['Cl0.555556Ir0.111111Pa0.333333', '-1.763', '-0.20145956132']
ClIrPb 3 ['Cl0.75Ir0.125Pb0.125', '-0.782', '-0.224627905316']
ClIrPm 29 ['Cl0.625Pm0.125Ir0.25', '-1.275', '-0.21789095258']
ClIrPr 9 ['Cl0.5Pr0.4Ir0.1', '-2.075', '-0.201160535166']
ClIrPt 9 ['Cl0.666667Ir0.111111Pt0.222222', '-0.648', '-0.244959653799']
ClIrPu 10 ['Cl0.6Ir0.1Pu0.3', '-2.046', '-0.203606251918']
ClIrRb 14 ['Cl0.8Rb0.1Ir0.1', '-0.673', '-0.246618038438']
ClIrRh 12 ['Cl0.6Rh0.2Ir0.2', '-0.209', '-0.201359194']
ClIrSc 3 ['Cl0.8Sc0.1Ir0.1', '-1.212', '-0.257003715816']
ClIrSe 1 ['Cl0.8Se0.1Ir0.1', '-0.523', '-0.209412422252']
ClIrSi 2 ['Si0.111111Cl0.777778Ir0.111111', '-1.072', '-0.223638139167']
ClIrSm 30 ['Cl0.5Sm0.375Ir0.125', '-1.925', '-0.204049167505']
ClIrSr 8 ['Cl0.7Sr0.2Ir0.1', '-1.85', '-0.209489797754']
ClIrTa 2 ['Cl0.8Ta0.1Ir0.1', '-1.043', '-0.25160489675']
ClIrTb 9 ['Cl0.444444Tb0.444444Ir0.111111', '-1.713', '-0.204275369256']
ClIrTe 8 ['Cl0.428571Te0.428571Ir0.142857', '-0.814', '-0.205202458526']
ClIrTh 3 ['Cl0.75Ir0.125Th0.125', '-1.749', '-0.218380694063']
ClIrTl 8 ['Cl0.7Ir0.1Tl0.2', '-0.839', '-0.201391321754']
ClIrTm 3 ['Cl0.75Tm0.125Ir0.125', '-1.49', '-0.230953180395']
ClIrXe 16 ['Cl0.777778Xe0.111111Ir0.111111', '-0.211', '-0.211']
ClIrY 3 ['Cl0.714286Y0.142857Ir0.142857', '-1.685', '-0.225979919737']
ClIrYb 5 ['Cl0.7Yb0.1Ir0.2', '-1.019', '-0.200258118377']
ClIrZn 1 ['Cl0.714286Zn0.142857Ir0.142857', '-0.801', '-0.208213119467']
ClIrZr 2 ['Cl0.777778Zr0.111111Ir0.111111', '-1.37', '-0.224597413611']
ClKLa 1 ['Cl0.666667K0.111111La0.222222', '-2.698', '-0.217114737924']
ClKLu 19 ['Cl0.555556K0.222222Lu0.222222', '-2.084', '-0.580915773058']
ClKNp 3 ['Cl0.7K0.2Np0.1', '-2.003', '-0.210414342251']
ClKP 7 ['P0.1Cl0.6K0.3', '-1.9', '-0.207771341982']
ClKPb 1 ['Cl0.666667K0.166667Pb0.166667', '-1.673', '-0.23222447386']
ClKPm 12 ['Cl0.777778K0.111111Pm0.111111', '-1.65', '-0.234547956669']
ClKPr 3 ['Cl0.666667K0.111111Pr0.222222', '-2.605', '-0.201990561624']
ClKPt 1 ['Cl0.7K0.1Pt0.2', '-1.125', '-0.201239513131']
ClKPu 4 ['Cl0.6K0.1Pu0.3', '-2.118', '-0.217923472881']

ClKRh 15 ['Cl0.777778K0.111111Rh0.111111', '-0.707', '-0.231200443264']
ClKS 9 ['S0.142857Cl0.714286K0.142857', '-0.991', '-0.200858961661']
ClKSi 15 ['Si0.142857Cl0.571429K0.285714', '-1.971', '-0.202139943573']
ClKSm 10 ['Cl0.6K0.3Sm0.1', '-2.313', '-0.260797638378']
ClKTa 3 ['Cl0.7K0.2Ta0.1', '-1.882', '-0.219694210633']
ClKTc 1 ['Cl0.7K0.1Tc0.2', '-1.438', '-0.207942585152']
ClKTl 1 ['Cl0.555556K0.222222Tl0.222222', '-1.812', '-0.235454546323']
ClKV 3 ['Cl0.666667K0.166667V0.166667', '-1.917', '-0.21199958459']
ClKXe 3 ['Cl0.6K0.1Xe0.3', '-0.638', '-0.209780398938']
ClKrLu 36 ['Cl0.444444Kr0.444444Lu0.111111', '-0.536', '-0.260257443264']
ClKrNp 1 ['Cl0.75Kr0.125Np0.125', '-1.381', '-0.210816930469']
ClKrPm 15 ['Cl0.5Kr0.125Pm0.375', '-1.695', '-0.202551742503']
ClKrPt 4 ['Cl0.666667Kr0.111111Pt0.222222', '-0.613', '-0.209959653799']
ClKrPu 4 ['Cl0.7Kr0.1Pu0.2', '-1.958', '-0.217666459632']
ClKrRh 16 ['Cl0.555556Kr0.222222Rh0.222222', '-0.218', '-0.218']
ClKrS 7 ['S0.1Cl0.8Kr0.1', '-0.334', '-0.209120874225']
ClKrSm 6 ['Cl0.666667Kr0.222222Sm0.111111', '-1.054', '-0.20117382396']
ClKrTh 1 ['Cl0.75Kr0.125Th0.125', '-1.731', '-0.200380694063']
ClLaLu 6 ['Cl0.666667La0.111111Lu0.222222', '-2.158', '-0.413472994658']
ClLaN 20 ['N0.2Cl0.3La0.5', '-1.99', '-0.242325898746']
ClLaNa 9 ['Na0.375Cl0.5La0.125', '-2.189', '-0.204028186868']
ClLaNp 1 ['Cl0.8La0.1Np0.1', '-2.266', '-0.256115841691']
ClLaO 8 ['O0.7Cl0.2La0.1', '-1.316', '-0.207488957986']
ClLaP 1 ['P0.3Cl0.2La0.5', '-1.937', '-0.202032736189']
ClLaPd 5 ['Cl0.333333Pd0.222222La0.444444', '-1.763', '-0.200649387018']
ClLaPm 2 ['Cl0.8La0.1Pm0.1', '-2.165', '-0.24557505938']
ClLaPr 1 ['Cl0.777778La0.111111Pr0.111111', '-2.555', '-0.216807495978']
ClLaPt 13 ['Cl0.666667La0.166667Pt0.166667', '-2.145', '-0.204297032368']
ClLaPu 2 ['Cl0.777778La0.111111Pu0.111111', '-2.384', '-0.224106141258']
ClLaRh 10 ['Cl0.666667Rh0.166667La0.166667', '-2.008', '-0.218437162193']
ClLaRu 2 ['Cl0.777778Ru0.111111La0.111111', '-1.77', '-0.220815107367']
ClLaS 2 ['S0.3Cl0.2La0.5', '-2.343', '-0.241238919671']
ClLaSb 14 ['Cl0.333333Sb0.166667La0.5', '-1.853', '-0.205869033129']
ClLaSe 35 ['Cl0.333333Se0.111111La0.555556', '-1.839', '-0.201536743684']
ClLaSi 8 ['Si0.1Cl0.4La0.5', '-1.793', '-0.200442385755']
ClLaSm 3 ['Cl0.75La0.125Sm0.125', '-2.57', '-0.2683984236']
ClLaSn 1 ['Cl0.3Sn0.2La0.5', '-1.705', '-0.201933530612']
ClLaTb 1 ['Cl0.777778La0.111111Tb0.111111', '-2.519', '-0.27010342296']
ClLaTc 1 ['Cl0.8Tc0.1La0.1', '-1.781', '-0.231444864194']
ClLaTe 18 ['Cl0.555556Te0.222222La0.222222', '-2.357', '-0.212801582838']
ClLaTl 2 ['Cl0.428571La0.428571Tl0.142857', '-1.873', '-0.210323834023']
ClLaXe 1 ['Cl0.714286Xe0.142857La0.142857', '-1.739', '-0.205088996166']
ClLiLu 22 ['Li0.166667Cl0.5Lu0.333333', '-1.737', '-0.243157470523']
ClLiNa 8 ['Li0.285714Na0.285714Cl0.428571', '-1.963', '-0.220125470203']

ClLiNb 3 ['Li0.1Cl0.7Nb0.2', '-1.843', '-0.216708532063']
ClLiNd 1 ['Li0.142857Cl0.714286Nd0.142857', '-2.27', '-0.232417565363']
ClLiNp 3 ['Li0.2Cl0.7Np0.1', '-2.025', '-0.288915713251']
ClLiP 3 ['Li0.375P0.125Cl0.5', '-1.879', '-0.209295793796']
ClLiPm 19 ['Li0.285714Cl0.571429Pm0.142857', '-2.178', '-0.210850789289']
ClLiPt 6 ['Li0.166667Cl0.666667Pt0.166667', '-1.175', '-0.206104881079']
ClLiPu 8 ['Li0.111111Cl0.666667Pu0.222222', '-2.281', '-0.225170149035']
ClLiRb 1 ['Li0.2Cl0.6Rb0.2', '-1.906', '-0.250572713752']
ClLiRh 17 ['Li0.333333Cl0.555556Rh0.111111', '-1.571', '-0.23777028146']
ClLiS 1 ['Li0.111111S0.111111Cl0.777778', '-0.788', '-0.204835509625']
ClLiSi 3 ['Li0.166667Si0.166667Cl0.666667', '-1.834', '-0.212978047293']
ClLiSm 8 ['Li0.25Cl0.625Sm0.125', '-2.175', '-0.21564826305']
ClLiTa 1 ['Li0.125Cl0.75Ta0.125', '-1.707', '-0.217794976485']
ClLiTl 1 ['Li0.166667Cl0.666667Tl0.166667', '-1.403', '-0.205044575525']
ClLiU 1 ['Li0.222222Cl0.666667U0.111111', '-2.205', '-0.217526297362']
ClLiXe 1 ['Li0.1Cl0.6Xe0.3', '-0.603', '-0.203031084438']
ClLuMg 18 ['Mg0.222222Cl0.555556Lu0.222222', '-1.897', '-0.219657845398']
ClLuMn 26 ['Cl0.571429Mn0.285714Lu0.142857', '-1.409', '-0.202880213117']
ClLuMo 13 ['Cl0.5Mo0.1Lu0.4', '-1.399', '-0.230685417732']
ClLuN 58 ['N0.428571Cl0.285714Lu0.285714', '-1.348', '-0.21454024276']
ClLuNa 2 ['Na0.111111Cl0.666667Lu0.222222', '-2.435', '-0.565926341784']
ClLuNb 7 ['Cl0.6Nb0.1Lu0.3', '-1.691', '-0.261409865255']
ClLuNd 6 ['Cl0.666667Nd0.111111Lu0.222222', '-2.064', '-0.372138454658']
ClLuNi 40 ['Cl0.3Ni0.3Lu0.4', '-1.118', '-0.200671238981']
ClLuNp 9 ['Cl0.7Lu0.1Np0.2', '-2.055', '-0.209559417818']
ClLuO 41 ['O0.375Cl0.125Lu0.5', '-2.996', '-0.209768144591']
ClLuOs 38 ['Cl0.5Lu0.25Os0.25', '-1.033', '-0.204416495315']
ClLuP 57 ['P0.142857Cl0.285714Lu0.571429', '-1.414', '-0.216019089257']
ClLuPa 5 ['Cl0.714286Lu0.142857Pa0.142857', '-2.035', '-0.205441245983']
ClLuPb 38 ['Cl0.3Lu0.4Pb0.3', '-1.136', '-0.240890304088']
ClLuPd 44 ['Cl0.3Pd0.2Lu0.5', '-1.334', '-0.200534417814']
ClLuPm 16 ['Cl0.6Pm0.2Lu0.2', '-1.913', '-0.221625524128']
ClLuPr 6 ['Cl0.666667Pr0.111111Lu0.222222', '-2.148', '-0.451364274378']
ClLuPt 56 ['Cl0.5Lu0.125Pt0.375', '-1.007', '-0.206992601565']
ClLuPu 18 ['Cl0.6Lu0.2Pu0.2', '-1.969', '-0.228666459632']
ClLuRb 19 ['Cl0.555556Rb0.111111Lu0.333333', '-1.64', '-0.339014594724']
ClLuRe 12 ['Cl0.571429Lu0.285714Re0.142857', '-1.309', '-0.241663436154']
ClLuRh 58 ['Cl0.333333Rh0.166667Lu0.5', '-1.368', '-0.202852042293']
ClLuRu 36 ['Cl0.222222Ru0.222222Lu0.555556', '-0.959', '-0.207125994307']
ClLuS 66 ['S0.375Cl0.125Lu0.5', '-2.029', '-0.20778009654']
ClLuSb 48 ['Cl0.333333Sb0.111111Lu0.555556', '-1.272', '-0.201633946553']
ClLuSc 6 ['Cl0.666667Sc0.111111Lu0.222222', '-2.069', '-0.456407904102']
ClLuSe 67 ['Cl0.142857Se0.428571Lu0.428571', '-1.887', '-0.205527909911']
ClLuSi 34 ['Si0.1Cl0.4Lu0.5', '-1.357', '-0.205072467252']

ClLuSm 11 ['Cl0.666667Sm0.222222Lu0.111111', '-2.081', '-0.258822150421']
ClLuSn 39 ['Cl0.625Sn0.25Lu0.125', '-1.462', '-0.203422749772']
ClLuSr 11 ['Cl0.6Sr0.1Lu0.3', '-1.987', '-0.422239995691']
ClLuTa 8 ['Cl0.625Lu0.25Ta0.125', '-1.594', '-0.231646338048']
ClLuTb 6 ['Cl0.666667Tb0.111111Lu0.222222', '-2.006', '-0.39866020136']
ClLuTc 30 ['Cl0.428571Tc0.285714Lu0.285714', '-1.096', '-0.200552198068']
ClLuTe 70 ['Cl0.444444Te0.444444Lu0.111111', '-1.037', '-0.201609718892']
ClLuTh 5 ['Cl0.714286Lu0.142857Th0.142857', '-2.305', '-0.20119464884']
ClLuTi 18 ['Cl0.5Ti0.1Lu0.4', '-1.454', '-0.209783755312']
ClLuTl 47 ['Cl0.428571Lu0.142857Tl0.428571', '-1.281', '-0.206372659199']
ClLuTm 6 ['Cl0.666667Tm0.111111Lu0.222222', '-2.059', '-0.388362157991']
ClLuU 6 ['Cl0.666667Lu0.222222U0.111111', '-1.911', '-0.260860996251']
ClLuV 14 ['Cl0.5V0.1Lu0.4', '-1.415', '-0.21791305169']
ClLuW 11 ['Cl0.7Lu0.1W0.2', '-1.333', '-0.251800445063']
ClLuXe 36 ['Cl0.375Xe0.375Lu0.25', '-0.825', '-0.204579247345']
ClLuY 6 ['Cl0.666667Y0.111111Lu0.222222', '-2.154', '-0.467721490769']
ClLuYb 11 ['Cl0.6Yb0.1Lu0.3', '-1.988', '-0.424753215191']
ClLuZn 28 ['Cl0.5Zn0.3Lu0.2', '-1.373', '-0.21370873037']
ClLuZr 9 ['Cl0.666667Zr0.166667Lu0.166667', '-1.962', '-0.215085516257']
ClMgNp 5 ['Mg0.25Cl0.625Np0.125', '-2.11', '-0.200435393833']
ClMgO 18 ['O0.2Mg0.4Cl0.4', '-2.65', '-0.210183000912']
ClMgPm 23 ['Mg0.25Cl0.625Pm0.125', '-2.163', '-0.200642958885']
ClMgPt 13 ['Mg0.3Cl0.5Pt0.2', '-1.896', '-0.200163833942']
ClMgPu 22 ['Mg0.333333Cl0.555556Pu0.111111', '-1.984', '-0.232000502667']
ClMgRh 10 ['Mg0.2Cl0.7Rh0.1', '-1.519', '-0.25756036192']
ClMgS 2 ['Mg0.333333S0.166667Cl0.5', '-2.064', '-0.223926795468']
ClMgSe 2 ['Mg0.3Cl0.5Se0.2', '-1.919', '-0.215420689192']
ClMgSi 1 ['Mg0.3Si0.1Cl0.6', '-2.107', '-0.21484054288']
ClMgSm 6 ['Mg0.3Cl0.5Sm0.2', '-1.799', '-0.200951087005']
ClMgTa 2 ['Mg0.1Cl0.8Ta0.1', '-1.648', '-0.22588507771']
ClMgTc 1 ['Mg0.222222Cl0.666667Tc0.111111', '-1.898', '-0.20645182607']
ClMgTl 1 ['Mg0.333333Cl0.555556Tl0.111111', '-1.957', '-0.200866624657']
ClMgZr 1 ['Mg0.166667Cl0.666667Zr0.166667', '-2.53', '-0.53744363806']
ClMnNp 2 ['Cl0.777778Mn0.111111Np0.111111', '-1.689', '-0.207270197634']
ClMnO 1 ['O0.142857Cl0.571429Mn0.285714', '-1.477', '-0.215916950088']
ClMnPm 11 ['Cl0.5Mn0.1Pm0.4', '-1.715', '-0.21259539919']
ClMnPu 2 ['Cl0.777778Mn0.111111Pu0.111111', '-1.641', '-0.232580959236']
ClMnRh 8 ['Cl0.777778Mn0.111111Rh0.111111', '-0.659', '-0.217432926107']
ClMnSc 1 ['Cl0.555556Sc0.333333Mn0.111111', '-2.031', '-0.219101525349']
ClMnSm 4 ['Cl0.7Mn0.1Sm0.2', '-1.995', '-0.205797869937']
ClMnTa 1 ['Cl0.8Mn0.1Ta0.1', '-1.392', '-0.203194530246']
ClMnTh 1 ['Cl0.8Mn0.1Th0.1', '-1.86', '-0.238094188746']
ClMoN 6 ['N0.5Cl0.166667Mo0.333333', '-0.754', '-0.225838254256']
ClMoNi 1 ['Cl0.625Ni0.25Mo0.125', '-1.14', '-0.231087415969']

ClMoNp 1 ['Cl0.777778Mo0.111111Np0.111111', '-1.797', '-0.255021627681']
ClMoO 4 ['O0.333333Cl0.444444Mo0.222222', '-1.83', '-0.20722874999']
ClMoPm 5 ['Cl0.7Mo0.1Pm0.2', '-2.093', '-0.225984146108']
ClMoPr 1 ['Cl0.75Mo0.125Pr0.125', '-2.115', '-0.262162962003']
ClMoPt 9 ['Cl0.8Mo0.1Pt0.1', '-1.044', '-0.210295117901']
ClMoPu 6 ['Cl0.7Mo0.2Pu0.1', '-1.782', '-0.209267717738']
ClMoRb 1 ['Cl0.714286Rb0.142857Mo0.142857', '-1.655', '-0.226789266878']
ClMoRh 5 ['Cl0.666667Mo0.111111Rh0.222222', '-0.961', '-0.236181415213']
ClMoSc 2 ['Cl0.8Sc0.1Mo0.1', '-1.825', '-0.217666989508']
ClMoSe 1 ['Cl0.666667Se0.111111Mo0.222222', '-1.234', '-0.202097583525']
ClMoSm 5 ['Cl0.7Mo0.2Sm0.1', '-1.746', '-0.275890929486']
ClMoTc 2 ['Cl0.75Mo0.125Tc0.125', '-1.275', '-0.221169178565']
ClMoTe 2 ['Cl0.666667Mo0.166667Te0.166667', '-1.184', '-0.227312417159']
ClMoZr 2 ['Cl0.777778Zr0.111111Mo0.111111', '-1.848', '-0.200781769764']
ClNNb 2 ['N0.4Cl0.3Nb0.3', '-1.352', '-0.206956015455']
ClNNd 1 ['N0.428571Cl0.142857Nd0.428571', '-1.87', '-0.233458198591']
ClNNp 34 ['N0.571429Cl0.142857Np0.285714', '-1.16', '-0.202336498757']
ClNOs 7 ['N0.4Cl0.4Os0.2', '-0.567', '-0.233939596752']
ClNPa 46 ['N0.111111Cl0.666667Pa0.222222', '-2.087', '-0.203777306748']
ClNPm 33 ['N0.2Cl0.3Pm0.5', '-1.743', '-0.201045979996']
ClNPu 38 ['N0.333333Cl0.444444Pu0.222222', '-1.712', '-0.220031152665']
ClNRe 6 ['N0.5Cl0.25Re0.25', '-0.539', '-0.206130610937']
ClNRh 18 ['N0.333333Cl0.333333Rh0.333333', '-0.2', '-0.2']
ClNRu 7 ['N0.5Cl0.25Ru0.25', '-0.469', '-0.201892749429']
ClNSc 2 ['N0.3Cl0.2Sc0.5', '-2.108', '-0.215486258272']
ClNSm 23 ['N0.375Cl0.125Sm0.5', '-1.788', '-0.207992340733']
ClNTb 19 ['N0.375Cl0.125Tb0.5', '-1.681', '-0.207854910554']
ClNTc 2 ['N0.25Cl0.5Tc0.25', '-0.877', '-0.202809845301']
ClNTh 1 ['N0.25Cl0.375Th0.375', '-2.325', '-0.212893635778']
ClNTi 14 ['N0.2Cl0.5Ti0.3', '-2.001', '-0.239902711488']
ClNTm 11 ['N0.2Cl0.3Tm0.5', '-1.98', '-0.201030995248']
ClNU 6 ['N0.5Cl0.2U0.3', '-1.82', '-0.204212097953']
ClNW 4 ['N0.375Cl0.375W0.25', '-0.886', '-0.209578401126']
ClNY 6 ['N0.375Cl0.125Y0.5', '-1.986', '-0.206494381671']
ClNYb 1 ['N0.3Cl0.1Yb0.6', '-1.423', '-0.228088987584']
ClNaNd 16 ['Na0.125Cl0.5Nd0.375', '-1.996', '-0.200549479585']
ClNaNp 4 ['Na0.1Cl0.8Np0.1', '-1.549', '-0.202831916727']
ClNaO 3 ['O0.2Na0.5Cl0.3', '-1.969', '-0.205765617708']
ClNaPm 24 ['Na0.4Cl0.5Pm0.1', '-2.151', '-0.202467494596']
ClNaPr 1 ['Na0.111111Cl0.666667Pr0.222222', '-2.608', '-0.243836060139']
ClNaPt 9 ['Na0.4Cl0.5Pt0.1', '-1.944', '-0.213229411513']
ClNaPu 9 ['Na0.1Cl0.8Pu0.1', '-1.493', '-0.212811602168']
ClNaRb 2 ['Na0.333333Cl0.555556Rb0.111111', '-2.046', '-0.205503506104']
ClNaRh 17 ['Na0.111111Cl0.777778Rh0.111111', '-0.658', '-0.202420413724']

ClNaS 5 ['Na0.142857S0.142857Cl0.714286', '-1.013', '-0.248856066538']
ClNaSc 1 ['Na0.166667Cl0.5Sc0.333333', '-1.972', '-0.204302144881']
ClNaSe 1 ['Na0.2Cl0.6Se0.2', '-1.334', '-0.200369166957']
ClNaSi 3 ['Na0.142857Si0.285714Cl0.571429', '-1.607', '-0.203191594699']
ClNaSm 12 ['Na0.285714Cl0.571429Sm0.142857', '-2.282', '-0.218726485116']
ClNaTb 1 ['Na0.111111Cl0.666667Tb0.222222', '-2.495', '-0.27966260511']
ClNaTc 2 ['Na0.2Cl0.7Tc0.1', '-1.499', '-0.203139311581']
ClNaTe 20 ['Na0.2Cl0.4Te0.4', '-1.244', '-0.201226951038']
ClNaXe 3 ['Na0.125Cl0.625Xe0.25', '-0.722', '-0.20947296544']
ClNaY 4 ['Na0.125Cl0.5Y0.375', '-2.009', '-0.203038058334']
ClNaYb 1 ['Na0.111111Cl0.555556Yb0.333333', '-2.542', '-0.266994010118']
ClNbNp 2 ['Cl0.8Nb0.1Np0.1', '-1.933', '-0.2198225763']
ClNbO 3 ['O0.571429Cl0.142857Nb0.285714', '-2.783', '-0.205642059783']
ClNbPm 3 ['Cl0.75Nb0.125Pm0.125', '-2.078', '-0.254439357346']
ClNbPt 4 ['Cl0.75Nb0.125Pt0.125', '-1.474', '-0.217468298706']
ClNbPu 4 ['Cl0.714286Nb0.142857Pu0.142857', '-2.058', '-0.202583022725']
ClNbRh 3 ['Cl0.75Nb0.125Rh0.125', '-1.392', '-0.248823396075']
ClNbRu 1 ['Cl0.75Nb0.125Ru0.125', '-1.537', '-0.260269770789']
ClNbSm 4 ['Cl0.714286Nb0.142857Sm0.142857', '-1.968', '-0.200272169556']
ClNbTc 1 ['Cl0.777778Nb0.111111Tc0.111111', '-1.516', '-0.20989444267']
ClNbTe 7 ['Cl0.5Nb0.166667Te0.333333', '-1.246', '-0.209201499578']
ClNbU 1 ['Cl0.777778Nb0.111111U0.111111', '-2.068', '-0.287754637107']
ClNdNi 6 ['Cl0.333333Ni0.166667Nd0.5', '-1.46', '-0.20502036424']
ClNdO 6 ['O0.571429Cl0.142857Nd0.285714', '-2.992', '-0.213287791992']
ClNdOs 6 ['Cl0.4Nd0.4Os0.2', '-1.609', '-0.207655625755']
ClNdP 1 ['P0.25Cl0.25Nd0.5', '-1.906', '-0.217418437607']
ClNdPd 22 ['Cl0.5Pd0.1Nd0.4', '-2.081', '-0.207324115693']
ClNdPm 2 ['Cl0.8Nd0.1Pm0.1', '-2.086', '-0.21397397338']
ClNdPt 36 ['Cl0.444444Nd0.222222Pt0.333333', '-2.022', '-0.202273116579']
ClNdPu 2 ['Cl0.777778Nd0.111111Pu0.111111', '-2.35', '-0.242771601258']
ClNdRb 1 ['Cl0.714286Rb0.142857Nd0.142857', '-2.289', '-0.213684642506']
ClNdRe 1 ['Cl0.8Nd0.1Re0.1', '-1.664', '-0.208785920069']
ClNdRh 11 ['Cl0.4Rh0.2Nd0.4', '-1.859', '-0.220455969755']
ClNdRu 2 ['Cl0.428571Ru0.142857Nd0.428571', '-1.708', '-0.215690780094']
ClNdS 4 ['S0.2Cl0.4Nd0.4', '-2.438', '-0.200461935951']
ClNdSb 2 ['Cl0.25Sb0.25Nd0.5', '-1.71', '-0.20359825818']
ClNdSe 42 ['Cl0.142857Se0.428571Nd0.428571', '-2.187', '-0.200999853007']
ClNdSi 14 ['Si0.111111Cl0.666667Nd0.222222', '-2.483', '-0.202247136258']
ClNdSm 3 ['Cl0.75Nd0.125Sm0.125', '-2.509', '-0.2666470661']
ClNdTc 2 ['Cl0.777778Tc0.111111Nd0.111111', '-1.873', '-0.203937531326']
ClNdTe 11 ['Cl0.666667Te0.166667Nd0.166667', '-2.111', '-0.214827190805']
ClNdTl 5 ['Cl0.444444Nd0.222222Tl0.333333', '-1.819', '-0.209085809357']
ClNdV 1 ['Cl0.8V0.1Nd0.1', '-1.938', '-0.210588500066']
ClNdXe 2 ['Cl0.666667Xe0.222222Nd0.111111', '-1.389', '-0.248623568129']

ClNiNp 2 ['Cl0.777778Ni0.111111Np0.111111', '-1.573', '-0.254431855835']
ClNiO 9 ['O0.25Cl0.375Ni0.375', '-1.165', '-0.210348997979']
ClNiPa 1 ['Cl0.666667Ni0.111111Pa0.222222', '-1.973', '-0.202262373103']
ClNiPm 37 ['Cl0.333333Ni0.166667Pm0.5', '-1.321', '-0.20189443521']
ClNiPu 9 ['Cl0.444444Ni0.222222Pu0.333333', '-1.562', '-0.220094774589']
ClNiRh 6 ['Cl0.777778Ni0.111111Rh0.111111', '-0.48', '-0.201594584307']
ClNiS 2 ['S0.111111Cl0.777778Ni0.111111', '-0.619', '-0.201840000112']
ClNiSm 10 ['Cl0.625Ni0.25Sm0.125', '-1.474', '-0.2013644593']
ClNiTa 2 ['Cl0.8Ni0.1Ta0.1', '-1.256', '-0.214040022626']
ClNiTb 2 ['Cl0.5Ni0.125Tb0.375', '-1.831', '-0.206508947267']
ClNiTe 7 ['Cl0.625Ni0.25Te0.125', '-1.001', '-0.220486987572']
ClNiTh 2 ['Cl0.777778Ni0.111111Th0.111111', '-1.892', '-0.25304409014']
ClNiU 3 ['Cl0.75Ni0.125U0.125', '-1.791', '-0.202523208908']
ClNiXe 6 ['Cl0.625Ni0.25Xe0.125', '-0.845', '-0.218587814691']
ClNiY 1 ['Cl0.444444Ni0.111111Y0.444444', '-1.848', '-0.214412512689']
ClNpO 31 ['O0.285714Cl0.285714Np0.428571', '-2.627', '-0.208863360029']
ClNpP 3 ['P0.1Cl0.7Np0.2', '-1.935', '-0.200217403815']
ClNpPb 6 ['Cl0.666667Pb0.222222Np0.111111', '-1.803', '-0.218899900284']
ClNpPd 5 ['Cl0.777778Pd0.111111Np0.111111', '-1.477', '-0.200344483983']
ClNpPm 7 ['Cl0.7Pm0.1Np0.2', '-2.112', '-0.201464574651']
ClNpPr 1 ['Cl0.8Pr0.1Np0.1', '-2.279', '-0.312217993439']
ClNpPt 18 ['Cl0.5Pt0.2Np0.3', '-1.654', '-0.201702438567']
ClNpRb 4 ['Cl0.8Rb0.1Np0.1', '-1.565', '-0.202471582813']
ClNpRh 6 ['Cl0.666667Rh0.222222Np0.111111', '-1.322', '-0.281837271528']
ClNpRu 1 ['Cl0.8Ru0.1Np0.1', '-1.516', '-0.259324843689']
ClNpS 12 ['S0.111111Cl0.666667Np0.222222', '-1.999', '-0.200116246388']
ClNpSc 2 ['Cl0.777778Sc0.111111Np0.111111', '-2.351', '-0.249730289101']
ClNpSe 40 ['Cl0.125Se0.625Np0.25', '-1.111', '-0.2060219285']
ClNpSi 16 ['Si0.1Cl0.8Np0.1', '-1.915', '-0.215327869625']
ClNpSm 3 ['Cl0.777778Sm0.111111Np0.111111', '-2.184', '-0.291011095488']
ClNpSr 1 ['Cl0.777778Sr0.111111Np0.111111', '-2.237', '-0.285442714724']
ClNpTa 1 ['Cl0.777778Ta0.111111Np0.111111', '-1.97', '-0.203298617989']
ClNpTb 1 ['Cl0.8Tb0.1Np0.1', '-2.212', '-0.325584327723']
ClNpTc 3 ['Cl0.75Tc0.125Np0.125', '-1.742', '-0.245624782397']
ClNpTe 8 ['Cl0.5Te0.25Np0.25', '-1.692', '-0.205708310282']
ClNpTl 25 ['Cl0.7Tl0.1Np0.2', '-2.053', '-0.214905548818']
ClNpTm 1 ['Cl0.8Tm0.1Np0.1', '-2.179', '-0.235616088691']
ClNpV 5 ['Cl0.714286V0.142857Np0.142857', '-1.956', '-0.231051959021']
ClNpXe 5 ['Cl0.666667Xe0.222222Np0.111111', '-1.283', '-0.242837271528']
ClNpY 1 ['Cl0.8Y0.1Np0.1', '-2.196', '-0.238539488191']
ClNpYb 1 ['Cl0.777778Yb0.111111Np0.111111', '-2.156', '-0.206124069724']
ClNpZn 1 ['Cl0.777778Zn0.111111Np0.111111', '-1.761', '-0.259780808891']
ClOP 10 ['O0.75P0.125Cl0.125', '-1.307', '-0.201680884402']
ClOPa 12 ['O0.6Cl0.2Pa0.2', '-2.216', '-0.206944195702']

ClOPb 7 ['O0.428571Cl0.285714Pb0.285714', '-1.309', '-0.203676391949']
ClOPm 47 ['O0.3Cl0.5Pm0.2', '-2.052', '-0.21368153561']
ClOPr 8 ['O0.25Cl0.375Pr0.375', '-3.098', '-0.201663680693']
ClOPt 9 ['O0.166667Cl0.666667Pt0.166667', '-0.568', '-0.200528894213']
ClOPu 37 ['O0.7Cl0.2Pu0.1', '-1.335', '-0.200290932605']
ClORb 2 ['O0.666667Cl0.111111Rb0.222222', '-1.229', '-0.244618541844']
ClORe 1 ['O0.7Cl0.1Re0.2', '-1.852', '-0.275412849635']
ClORh 17 ['O0.3Cl0.4Rh0.3', '-0.676', '-0.208989533238']
ClORu 1 ['O0.111111Cl0.666667Ru0.222222', '-1.031', '-0.234600674003']
ClOSb 10 ['O0.4Cl0.3Sb0.3', '-1.653', '-0.20757261021']
ClOSc 4 ['O0.3Cl0.3Sc0.4', '-3.081', '-0.202042065217']
ClOSe 9 ['O0.571429Cl0.285714Se0.142857', '-0.76', '-0.209516827535']
ClOSm 6 ['O0.4Cl0.2Sm0.4', '-3.469', '-0.243729348605']
ClOSn 1 ['O0.428571Cl0.142857Sn0.428571', '-1.839', '-0.271103769636']
ClOTb 22 ['O0.285714Cl0.428571Tb0.285714', '-2.851', '-0.205330838307']
ClOTc 1 ['O0.333333Cl0.444444Tc0.222222', '-1.474', '-0.239737915033']
ClOTe 3 ['O0.7Cl0.1Te0.2', '-1.154', '-0.219605230329']
ClOTh 4 ['O0.2Cl0.6Th0.2', '-2.734', '-0.225770327525']
ClOTi 1 ['O0.3Cl0.3Ti0.4', '-2.611', '-0.217463241538']
ClOTl 7 ['O0.3Cl0.3Tl0.4', '-1.236', '-0.207746770551']
ClOTm 8 ['O0.2Cl0.3Tm0.5', '-2.554', '-0.236147609639']
ClOU 12 ['O0.375Cl0.25U0.375', '-3.0', '-0.204177616594']
ClOXe 17 ['O0.142857Cl0.571429Xe0.285714', '-0.263', '-0.207122131884']
ClOY 2 ['O0.142857Cl0.428571Y0.428571', '-2.623', '-0.201777624848']
ClOZr 12 ['O0.222222Cl0.444444Zr0.333333', '-2.672', '-0.205863638417']
ClOsPm 10 ['Cl0.777778Pm0.111111Os0.111111', '-1.516', '-0.206280398685']
ClOsPr 1 ['Cl0.428571Pr0.428571Os0.142857', '-1.692', '-0.206989108306']
ClOsRh 4 ['Cl0.75Rh0.125Os0.125', '-0.679', '-0.262674495941']
ClOsSc 2 ['Cl0.1Sc0.6Os0.3', '-0.762', '-0.220147095813']
ClOsSm 4 ['Cl0.75Sm0.125Os0.125', '-1.48', '-0.20832642391']
ClOsTb 2 ['Cl0.5Tb0.375Os0.125', '-1.787', '-0.203217972247']
ClOsTe 1 ['Cl0.375Te0.5Os0.125', '-0.666', '-0.221602578231']
ClOsTm 1 ['Cl0.3Tm0.5Os0.2', '-1.264', '-0.203006442316']
ClOsXe 1 ['Cl0.7Xe0.2Os0.1', '-0.537', '-0.203939596753']
ClOsY 4 ['Cl0.375Y0.5Os0.125', '-1.58', '-0.229652515862']
ClPPm 67 ['P0.333333Cl0.5Pm0.166667', '-1.614', '-0.204521270107']
ClPPr 3 ['P0.25Cl0.25Pr0.5', '-1.886', '-0.204604576772']
ClPPt 8 ['P0.222222Cl0.444444Pt0.333333', '-0.972', '-0.239431000419']
ClPPu 22 ['P0.375Cl0.25Pu0.375', '-1.686', '-0.208305695983']
ClPRb 11 ['P0.111111Cl0.777778Rb0.111111', '-1.29', '-0.209021725814']
ClPRh 7 ['P0.125Cl0.75Rh0.125', '-0.914', '-0.230876893493']
ClPRu 3 ['P0.5Cl0.166667Ru0.333333', '-0.994', '-0.208143030555']
ClPSm 46 ['P0.5Cl0.1Sm0.4', '-1.744', '-0.201151790083']
ClPSr 12 ['P0.333333Cl0.222222Sr0.444444', '-1.9', '-0.204367404097']

ClPTa 4 ['P0.111111Cl0.777778Ta0.111111', '-1.533', '-0.200816713242']
ClPTb 28 ['P0.444444Cl0.111111Tb0.444444', '-1.714', '-0.213683347543']
ClPTe 3 ['P0.2Cl0.6Te0.2', '-1.029', '-0.213860290336']
ClPTm 2 ['P0.5Cl0.125Tm0.375', '-1.819', '-0.20780447961']
ClPY 18 ['P0.1Cl0.8Y0.1', '-1.768', '-0.20018745861']
ClPYb 2 ['P0.375Cl0.25Yb0.375', '-1.806', '-0.227603822268']
ClPaPm 1 ['Cl0.7Pm0.2Pa0.1', '-2.206', '-0.258487298566']
ClPaPt 3 ['Cl0.5Pt0.125Pa0.375', '-1.693', '-0.211436932188']
ClPaRb 1 ['Cl0.714286Rb0.142857Pa0.142857', '-2.324', '-0.251235908841']
ClPaRh 4 ['Cl0.75Rh0.125Pa0.125', '-1.841', '-0.240136090235']
ClPaS 19 ['S0.142857Cl0.428571Pa0.428571', '-1.797', '-0.201413157015']
ClPaSb 1 ['Cl0.375Sb0.25Pa0.375', '-1.639', '-0.444308646328']
ClPaSe 29 ['Cl0.3Se0.5Pa0.2', '-1.308', '-0.202458223913']
ClPaSi 8 ['Si0.142857Cl0.571429Pa0.285714', '-1.758', '-0.206313426072']
ClPaSm 1 ['Cl0.8Sm0.1Pa0.1', '-2.285', '-0.236765313752']
ClPaTe 30 ['Cl0.571429Te0.142857Pa0.285714', '-1.845', '-0.201122653453']
ClPaZn 2 ['Cl0.666667Zn0.166667Pa0.166667', '-1.921', '-0.201496280584']
ClPbPm 24 ['Cl0.5Pm0.25Pb0.25', '-1.751', '-0.221888718232']
ClPbPt 13 ['Cl0.6Pt0.2Pb0.2', '-1.226', '-0.208559911963']
ClPbPu 23 ['Cl0.5Pb0.3Pu0.2', '-1.683', '-0.21029653736']
ClPbRb 1 ['Cl0.666667Rb0.166667Pb0.166667', '-1.791', '-0.28385010542']
ClPbRh 13 ['Cl0.6Rh0.2Pb0.2', '-1.086', '-0.22206635813']
ClPbSi 4 ['Si0.2Cl0.6Pb0.2', '-1.433', '-0.215165230379']
ClPbSm 14 ['Cl0.555556Sm0.222222Pb0.222222', '-1.83', '-0.210539562992']
ClPbTa 2 ['Cl0.777778Ta0.111111Pb0.111111', '-1.595', '-0.235708973128']
ClPbTe 25 ['Cl0.222222Te0.444444Pb0.333333', '-0.847', '-0.201077385789']
ClPbY 1 ['Cl0.428571Y0.428571Pb0.142857', '-1.899', '-0.208197572594']
ClPbZr 1 ['Cl0.777778Zr0.111111Pb0.111111', '-1.847', '-0.221634279239']
ClPdPm 41 ['Cl0.333333Pd0.444444Pm0.222222', '-1.323', '-0.21020861646']
ClPdPr 8 ['Cl0.428571Pd0.142857Pr0.428571', '-1.92', '-0.221225373306']
ClPdPu 22 ['Cl0.625Pd0.125Pu0.25', '-2.089', '-0.219802010867']
ClPdRh 8 ['Cl0.6Rh0.3Pd0.1', '-0.424', '-0.21115649121']
ClPdSe 11 ['Cl0.25Se0.5Pd0.25', '-0.608', '-0.203493369637']
ClPdSm 22 ['Cl0.5Pd0.2Sm0.3', '-1.928', '-0.202191635317']
ClPdTa 4 ['Cl0.666667Pd0.166667Ta0.166667', '-1.536', '-0.21700816125']
ClPdTb 2 ['Cl0.4Pd0.2Tb0.4', '-1.709', '-0.206890210864']
ClPdTe 19 ['Cl0.4Pd0.2Te0.4', '-0.827', '-0.207771881169']
ClPdTm 6 ['Cl0.2Pd0.3Tm0.5', '-1.442', '-0.201246215877']
ClPdXe 4 ['Cl0.625Pd0.125Xe0.25', '-0.473', '-0.206945614012']
ClPdZr 2 ['Cl0.625Zr0.25Pd0.125', '-2.011', '-0.208073772429']
ClPmPr 1 ['Cl0.8Pr0.1Pm0.1', '-2.104', '-0.227677211128']
ClPmPt 42 ['Cl0.3Pm0.3Pt0.4', '-1.618', '-0.201635489564']
ClPmPu 5 ['Cl0.7Pm0.2Pu0.1', '-2.232', '-0.207699997068']
ClPmRb 7 ['Cl0.625Rb0.125Pm0.25', '-2.192', '-0.216356007113']

ClPmRe 3 ['Cl0.555556Pm0.333333Re0.111111', '-1.845', '-0.219911969378']
ClPmRh 32 ['Cl0.375Rh0.25Pm0.375', '-1.632', '-0.234019119455']
ClPmRu 23 ['Cl0.375Ru0.125Pm0.5', '-1.388', '-0.203851105392']
ClPmS 64 ['S0.5Cl0.166667Pm0.333333', '-2.206', '-0.207824836279']
ClPmSb 60 ['Cl0.666667Sb0.166667Pm0.166667', '-1.87', '-0.208241004587']
ClPmSc 2 ['Cl0.8Sc0.1Pm0.1', '-2.07', '-0.26931647788']
ClPmSe 78 ['Cl0.333333Se0.111111Pm0.555556', '-1.596', '-0.20762886146']
ClPmSi 46 ['Si0.125Cl0.75Pm0.125', '-1.973', '-0.200085632502']
ClPmSm 3 ['Cl0.75Pm0.125Sm0.125', '-2.335', '-0.318461504535']
ClPmSn 34 ['Cl0.375Sn0.125Pm0.5', '-1.523', '-0.206310495786']
ClPmSr 4 ['Cl0.7Sr0.1Pm0.2', '-2.499', '-0.255991044567']
ClPmTa 2 ['Cl0.75Pm0.125Ta0.125', '-2.009', '-0.209958043283']
ClPmTb 1 ['Cl0.8Pm0.1Tb0.1', '-2.006', '-0.210043545412']
ClPmTc 18 ['Cl0.444444Tc0.111111Pm0.444444', '-1.577', '-0.206128911947']
ClPmTe 80 ['Cl0.111111Te0.333333Pm0.555556', '-1.42', '-0.207244029373']
ClPmTh 1 ['Cl0.8Pm0.1Th0.1', '-2.387', '-0.316817317314']
ClPmTi 8 ['Cl0.666667Ti0.166667Pm0.166667', '-2.028', '-0.203032579793']
ClPmTl 36 ['Cl0.375Pm0.5Tl0.125', '-1.481', '-0.226139937267']
ClPmTm 1 ['Cl0.8Pm0.1Tm0.1', '-2.078', '-0.22507530638']
ClPmU 4 ['Cl0.75Pm0.125U0.125', '-2.251', '-0.22269073485']
ClPmV 10 ['Cl0.666667V0.166667Pm0.166667', '-1.954', '-0.203831696669']
ClPmW 5 ['Cl0.714286Pm0.142857W0.142857', '-1.846', '-0.241193824913']
ClPmXe 13 ['Cl0.6Xe0.3Pm0.1', '-1.059', '-0.213312762064']
ClPmY 1 ['Cl0.777778Y0.111111Pm0.111111', '-2.328', '-0.253554117644']
ClPmYb 1 ['Cl0.666667Pm0.222222Yb0.111111', '-2.433', '-0.240916539588']
ClPmZn 17 ['Cl0.428571Zn0.142857Pm0.428571', '-1.563', '-0.200297213187']
ClPmZr 4 ['Cl0.666667Zr0.111111Pm0.222222', '-2.152', '-0.242058632364']
ClPrPt 42 ['Cl0.5Pr0.4Pt0.1', '-2.143', '-0.201602828023']
ClPrPu 5 ['Cl0.714286Pr0.142857Pu0.142857', '-2.509', '-0.207933241345']
ClPrRb 2 ['Cl0.7Rb0.1Pr0.2', '-2.752', '-0.24302802044']
ClPrRh 14 ['Cl0.428571Rh0.142857Pr0.428571', '-1.864', '-0.207290686163']
ClPrRu 2 ['Cl0.8Ru0.1Pr0.1', '-1.56', '-0.208835748378']
ClPrS 2 ['S0.1Cl0.8Pr0.1', '-1.365', '-0.209485323289']
ClPrSb 13 ['Cl0.222222Sb0.333333Pr0.444444', '-1.775', '-0.201289264307']
ClPrSe 17 ['Cl0.3Se0.3Pr0.4', '-2.34', '-0.200278318814']
ClPrSi 8 ['Si0.111111Cl0.777778Pr0.111111', '-2.206', '-0.212487527016']
ClPrSm 3 ['Cl0.777778Pr0.111111Sm0.111111', '-2.274', '-0.276023211809']
ClPrTa 1 ['Cl0.8Pr0.1Ta0.1', '-2.061', '-0.238969345814']
ClPrTc 3 ['Cl0.777778Tc0.111111Pr0.111111', '-1.896', '-0.222163351046']
ClPrTe 31 ['Cl0.1Te0.5Pr0.4', '-1.792', '-0.200258739942']
ClPrTh 1 ['Cl0.8Pr0.1Th0.1', '-2.492', '-0.236869004314']
ClPrTl 14 ['Cl0.5Pr0.125Tl0.375', '-1.791', '-0.201677848752']
ClPrV 2 ['Cl0.8V0.1Pr0.1', '-1.954', '-0.222291737814']
ClPrXe 3 ['Cl0.7Xe0.1Pr0.2', '-2.262', '-0.200728898128']

ClPrZn 1 ['Cl0.7Zn0.1Pr0.2', '-2.471', '-0.202253489942']
ClPtPu 32 ['Cl0.6Pt0.3Pu0.1', '-1.343', '-0.20078099613']
ClPtRe 4 ['Cl0.75Re0.125Pt0.125', '-0.993', '-0.230195691202']
ClPtRh 10 ['Cl0.625Rh0.25Pt0.125', '-0.497', '-0.270289805262']
ClPtRu 3 ['Cl0.714286Ru0.142857Pt0.142857', '-0.94', '-0.223004490748']
ClPtS 6 ['S0.333333Cl0.444444Pt0.222222', '-0.804', '-0.215355984776']
ClPtSb 7 ['Cl0.6Sb0.2Pt0.2', '-1.121', '-0.212791044128']
ClPtSc 3 ['Cl0.75Sc0.125Pt0.125', '-1.667', '-0.246544450032']
ClPtSe 14 ['Cl0.4Se0.4Pt0.2', '-0.709', '-0.200979536286']
ClPtSi 12 ['Si0.142857Cl0.714286Pt0.142857', '-1.432', '-0.211700353364']
ClPtSm 46 ['Cl0.25Sm0.5Pt0.25', '-1.587', '-0.207797953596']
ClPtSr 7 ['Cl0.166667Sr0.5Pt0.333333', '-1.388', '-0.223241515731']
ClPtTa 8 ['Cl0.666667Ta0.111111Pt0.222222', '-1.345', '-0.264151934399']
ClPtTb 7 ['Cl0.285714Tb0.428571Pt0.285714', '-1.649', '-0.205472142808']
ClPtTc 2 ['Cl0.8Tc0.1Pt0.1', '-0.863', '-0.205814411087']
ClPtTe 29 ['Cl0.6Te0.3Pt0.1', '-0.976', '-0.211814667004']
ClPtTh 8 ['Cl0.1Pt0.7Th0.2', '-0.937', '-0.203410776188']
ClPtTi 2 ['Cl0.714286Ti0.142857Pt0.142857', '-1.669', '-0.222836522471']
ClPtTl 5 ['Cl0.7Pt0.2Tl0.1', '-0.932', '-0.200172244131']
ClPtTm 3 ['Cl0.8Tm0.1Pt0.1', '-1.394', '-0.205394388525']
ClPtU 5 ['Cl0.75Pt0.125U0.125', '-1.789', '-0.216529754745']
ClPtV 5 ['Cl0.666667V0.111111Pt0.222222', '-1.194', '-0.213510147733']
ClPtW 2 ['Cl0.7W0.1Pt0.2', '-1.106', '-0.209365352065']
ClPtXe 14 ['Cl0.5Xe0.375Pt0.125', '-0.443', '-0.216289805262']
ClPtY 17 ['Cl0.5Y0.3Pt0.2', '-2.292', '-0.203863158527']
ClPtZr 1 ['Cl0.714286Zr0.142857Pt0.142857', '-1.876', '-0.273790849078']
ClPuRb 2 ['Cl0.777778Rb0.111111Pu0.111111', '-1.823', '-0.283096116634']
ClPuRe 1 ['Cl0.8Re0.1Pu0.1', '-1.514', '-0.214957938569']
ClPuRh 18 ['Cl0.625Rh0.125Pu0.25', '-2.101', '-0.220228887117']
ClPuRu 6 ['Cl0.7Ru0.1Pu0.2', '-2.052', '-0.204823559403']
ClPuS 42 ['S0.111111Cl0.666667Pu0.222222', '-2.135', '-0.201296066258']
ClPuSb 22 ['Cl0.7Sb0.1Pu0.2', '-2.094', '-0.20229830032']
ClPuSc 2 ['Cl0.777778Sc0.111111Pu0.111111', '-2.328', '-0.300041050702']
ClPuSe 48 ['Cl0.25Se0.25Pu0.5', '-1.702', '-0.203877187347']
ClPuSi 16 ['Si0.111111Cl0.555556Pu0.333333', '-1.921', '-0.202603846326']
ClPuSm 5 ['Cl0.7Sm0.2Pu0.1', '-2.321', '-0.20233681957']
ClPuSn 15 ['Cl0.8Sn0.1Pu0.1', '-1.69', '-0.203647870381']
ClPuSr 5 ['Cl0.7Sr0.1Pu0.2', '-2.475', '-0.20446694857']
ClPuTa 1 ['Cl0.8Ta0.1Pu0.1', '-1.91', '-0.248438126566']
ClPuTb 2 ['Cl0.8Tb0.1Pu0.1', '-2.121', '-0.300564013164']
ClPuTc 11 ['Cl0.5Tc0.25Pu0.25', '-1.651', '-0.200722049693']
ClPuTe 47 ['Cl0.5Te0.1Pu0.4', '-1.889', '-0.217769361359']
ClPuTh 1 ['Cl0.8Th0.1Pu0.1', '-2.382', '-0.287337785066']
ClPuTi 2 ['Cl0.6Ti0.1Pu0.3', '-1.963', '-0.222666459632']

ClPuTl 40 ['Cl0.666667Tl0.166667Pu0.166667', '-2.068', '-0.216351766257']
ClPuTm 2 ['Cl0.777778Tm0.111111Pu0.111111', '-2.288', '-0.201995304591']
ClPuV 3 ['Cl0.714286V0.142857Pu0.142857', '-2.065', '-0.237865345273']
ClPuW 3 ['Cl0.714286W0.142857Pu0.142857', '-1.841', '-0.201223064559']
ClPuXe 9 ['Cl0.7Xe0.1Pu0.2', '-1.999', '-0.258666459632']
ClPuY 2 ['Cl0.777778Y0.111111Pu0.111111', '-2.365', '-0.263354637369']
ClPuYb 2 ['Cl0.666667Yb0.111111Pu0.222222', '-2.449', '-0.250150842369']
ClPuZn 17 ['Cl0.7Zn0.1Pu0.2', '-2.156', '-0.208191051446']
ClPuZr 10 ['Cl0.666667Zr0.111111Pu0.222222', '-2.138', '-0.202868352368']
ClRbRe 5 ['Cl0.8Rb0.1Re0.1', '-1.063', '-0.207742747191']
ClRbRh 15 ['Cl0.555556Rb0.222222Rh0.222222', '-1.177', '-0.229484529862']
ClRbRu 2 ['Cl0.666667Rb0.222222Ru0.111111', '-1.526', '-0.2223415291']
ClRbS 16 ['S0.4Cl0.3Rb0.3', '-1.484', '-0.204854115314']
ClRbSb 1 ['Cl0.7Rb0.2Sb0.1', '-1.656', '-0.236409053566']
ClRbSc 1 ['Cl0.75Sc0.125Rb0.125', '-1.935', '-0.208277192818']
ClRbSe 1 ['Cl0.111111Se0.333333Rb0.555556', '-1.45', '-0.202753411597']
ClRbSi 12 ['Si0.222222Cl0.555556Rb0.222222', '-1.8', '-0.216213134237']
ClRbSm 11 ['Cl0.8Rb0.1Sm0.1', '-1.418', '-0.224074480002']
ClRbTa 3 ['Cl0.7Rb0.2Ta0.1', '-1.877', '-0.232840973626']
ClRbTc 5 ['Cl0.666667Rb0.166667Tc0.166667', '-1.591', '-0.227979101254']
ClRbTe 6 ['Cl0.285714Rb0.428571Te0.285714', '-1.688', '-0.20465707137']
ClRbTh 1 ['Cl0.777778Rb0.111111Th0.111111', '-2.041', '-0.206691770764']
ClRbTl 4 ['Cl0.5Rb0.2Tl0.3', '-1.777', '-0.20176956669']
ClRbV 1 ['Cl0.75V0.125Rb0.125', '-1.632', '-0.222681658985']
ClRbXe 4 ['Cl0.5Rb0.1Xe0.4', '-0.64', '-0.213618038438']
ClReRh 5 ['Cl0.75Rh0.125Re0.125', '-0.809', '-0.272905885941']
ClReS 10 ['S0.3Cl0.5Re0.2', '-0.978', '-0.214238470436']
ClReSe 2 ['Cl0.25Se0.5Re0.25', '-0.722', '-0.211447791373']
ClReSm 2 ['Cl0.777778Sm0.111111Re0.111111', '-1.59', '-0.260645722574']
ClReTb 1 ['Cl0.8Tb0.1Re0.1', '-1.592', '-0.212855492101']
ClReXe 1 ['Cl0.7Xe0.2Re0.1', '-0.714', '-0.285124708753']
ClRhRu 6 ['Cl0.8Ru0.1Rh0.1', '-0.556', '-0.235471299314']
ClRhS 24 ['S0.3Cl0.4Rh0.3', '-0.569', '-0.205821252298']
ClRhSb 9 ['Cl0.625Rh0.25Sb0.125', '-0.911', '-0.202466220863']
ClRhSc 9 ['Cl0.666667Sc0.166667Rh0.166667', '-1.836', '-0.24433952636']
ClRhSe 22 ['Cl0.444444Se0.222222Rh0.333333', '-0.624', '-0.207514209308']
ClRhSi 8 ['Si0.125Cl0.625Rh0.25', '-1.287', '-0.332592906563']
ClRhSm 23 ['Cl0.5Rh0.2Sm0.3', '-1.876', '-0.208852162984']
ClRhSn 1 ['Cl0.7Rh0.2Sn0.1', '-0.883', '-0.266814640565']
ClRhSr 13 ['Cl0.777778Sr0.111111Rh0.111111', '-1.167', '-0.255605443197']
ClRhTa 6 ['Cl0.666667Rh0.222222Ta0.111111', '-1.187', '-0.3076721075']
ClRhTb 10 ['Cl0.6Rh0.3Tb0.1', '-1.161', '-0.210730783348']
ClRhTc 6 ['Cl0.714286Tc0.142857Rh0.142857', '-0.92', '-0.240260809825']
ClRhTe 25 ['Cl0.666667Rh0.111111Te0.222222', '-1.023', '-0.218528396995']

ClRhTh 6 ['Cl0.666667Rh0.222222Th0.111111', '-1.58', '-0.219449505833']
ClRhTi 6 ['Cl0.8Ti0.1Rh0.1', '-1.123', '-0.201369643625']
ClRhTl 16 ['Cl0.555556Rh0.222222Tl0.222222', '-0.922', '-0.21354591306']
ClRhTm 6 ['Cl0.666667Rh0.222222Tm0.111111', '-1.455', '-0.335847271462']
ClRhU 3 ['Cl0.7Rh0.2U0.1', '-1.515', '-0.257023803796']
ClRhV 5 ['Cl0.666667V0.111111Rh0.222222', '-1.004', '-0.225030320833']
ClRhXe 29 ['Cl0.4Rh0.2Xe0.4', '-0.211', '-0.211']
ClRhY 9 ['Cl0.666667Y0.166667Rh0.166667', '-1.917', '-0.21480990636']
ClRhYb 7 ['Cl0.75Rh0.125Yb0.125', '-1.287', '-0.263572647971']
ClRhZn 8 ['Cl0.6Zn0.1Rh0.3', '-0.632', '-0.217049183627']
ClRhZr 6 ['Cl0.666667Zr0.111111Rh0.222222', '-1.493', '-0.347597413611']
ClRuSi 4 ['Si0.125Cl0.75Ru0.125', '-1.422', '-0.200485655991']
ClRuSm 8 ['Cl0.7Ru0.2Sm0.1', '-1.5', '-0.30508484065']
ClRuTa 3 ['Cl0.777778Ru0.111111Ta0.111111', '-1.458', '-0.222529106738']
ClRuTb 4 ['Cl0.5Ru0.1Tb0.4', '-1.792', '-0.208217972247']
ClRuTe 24 ['Cl0.6Ru0.1Te0.3', '-0.953', '-0.206065165015']
ClRuTh 1 ['Cl0.8Ru0.1Th0.1', '-1.904', '-0.358975854564']
ClRuTi 1 ['Cl0.75Ti0.125Ru0.125', '-1.744', '-0.32485480396']
ClRuTm 2 ['Cl0.777778Ru0.111111Tm0.111111', '-1.725', '-0.2497042707']
ClRuXe 4 ['Cl0.625Ru0.125Xe0.25', '-0.61', '-0.209339124143']
ClRuY 5 ['Cl0.428571Y0.428571Ru0.142857', '-1.764', '-0.209030942593']
ClRuZr 2 ['Cl0.8Zr0.1Ru0.1', '-1.565', '-0.213608971564']
ClSSc 3 ['S0.428571Cl0.285714Sc0.285714', '-2.107', '-0.20142355201']
ClSSm 8 ['S0.428571Cl0.142857Sm0.428571', '-2.477', '-0.217456240779']
ClSSn 1 ['S0.428571Cl0.285714Sn0.285714', '-1.027', '-0.202004122215']
ClSTb 8 ['S0.3Cl0.3Tb0.4', '-2.246', '-0.200359905744']
ClSTc 17 ['S0.555556Cl0.222222Tc0.222222', '-0.922', '-0.200311431915']
ClSTe 7 ['S0.2Cl0.5Te0.3', '-0.82', '-0.206966940879']
ClSTh 6 ['S0.222222Cl0.333333Th0.444444', '-2.109', '-0.202644517371']
ClSTm 6 ['S0.222222Cl0.333333Tm0.444444', '-2.252', '-0.212318192791']
ClSU 6 ['S0.4Cl0.3U0.3', '-1.945', '-0.203000149084']
ClSXe 26 ['S0.2Cl0.6Xe0.2', '-0.453', '-0.20324174845']
ClSY 2 ['S0.3Cl0.3Y0.4', '-2.507', '-0.20109138911']
ClSbSc 18 ['Cl0.222222Sc0.444444Sb0.333333', '-1.554', '-0.200875489308']
ClSbSm 23 ['Cl0.3Sb0.3Sm0.4', '-1.729', '-0.200986495815']
ClSbTb 1 ['Cl0.4Sb0.2Tb0.4', '-1.801', '-0.205075735864']
ClSbTe 1 ['Cl0.6Sb0.2Te0.2', '-1.141', '-0.232791044128']
ClSbU 7 ['Cl0.5Sb0.25U0.25', '-1.568', '-0.20714426761']
ClSbY 19 ['Cl0.166667Y0.5Sb0.333333', '-1.587', '-0.201308587953']
ClSbYb 2 ['Cl0.25Sb0.25Yb0.5', '-1.853', '-0.209636846931']
ClScSe 38 ['Cl0.125Sc0.5Se0.375', '-1.807', '-0.204868007236']
ClScSi 16 ['Si0.222222Cl0.666667Sc0.111111', '-1.908', '-0.210621621948']
ClScSm 2 ['Cl0.777778Sc0.111111Sm0.111111', '-2.166', '-0.252066841533']
ClScTa 1 ['Cl0.8Sc0.1Ta0.1', '-1.979', '-0.232608612566']

ClScTc 1 ['Cl0.8Sc0.1Tc0.1', '-1.677', '-0.246186282694']
ClScTe 4 ['Cl0.3Sc0.4Te0.3', '-1.862', '-0.204094746314']
ClScTh 1 ['Cl0.8Sc0.1Th0.1', '-2.415', '-0.235508271066']
ClScTl 21 ['Cl0.5Sc0.2Tl0.3', '-1.809', '-0.200091323693']
ClScV 1 ['Cl0.8Sc0.1V0.1', '-1.875', '-0.218931004566']
ClSeSi 1 ['Si0.2Cl0.6Se0.2', '-1.428', '-0.214790553625']
ClSeSm 46 ['Cl0.111111Se0.555556Sm0.333333', '-1.929', '-0.204461572663']
ClSeTb 38 ['Cl0.7Se0.1Tb0.2', '-2.18', '-0.201064672259']
ClSeTc 16 ['Cl0.333333Se0.333333Tc0.333333', '-0.639', '-0.204077135905']
ClSeTe 1 ['Cl0.75Se0.125Te0.125', '-0.963', '-0.210183279744']
ClSeTh 13 ['Cl0.25Se0.375Th0.375', '-2.068', '-0.201976559531']
ClSeTi 6 ['Cl0.5Ti0.25Se0.25', '-1.775', '-0.203017892807']
ClSeTm 12 ['Cl0.111111Se0.555556Tm0.333333', '-1.881', '-0.208889117157']
ClSeU 42 ['Cl0.285714Se0.285714U0.428571', '-1.515', '-0.208530899349']
ClSeY 24 ['Cl0.333333Se0.222222Y0.444444', '-2.136', '-0.201435092572']
ClSeYb 1 ['Cl0.555556Se0.111111Yb0.333333', '-2.745', '-0.236699287158']
ClSeZr 28 ['Cl0.111111Se0.555556Zr0.333333', '-1.628', '-0.202151345487']
ClSiSm 22 ['Si0.25Cl0.5Sm0.25', '-1.765', '-0.204379487193']
ClSiSr 2 ['Si0.166667Cl0.666667Sr0.166667', '-2.212', '-0.20863676917']
ClSiTb 12 ['Si0.142857Cl0.571429Tb0.285714', '-2.12', '-0.214539818314']
ClSiTc 1 ['Si0.111111Cl0.777778Tc0.111111', '-1.488', '-0.204715275072']
ClSiTe 8 ['Si0.166667Cl0.666667Te0.166667', '-1.483', '-0.21045720875']
ClSiTl 1 ['Si0.142857Cl0.571429Tl0.285714', '-1.477', '-0.243561175001']
ClSiTm 3 ['Si0.285714Cl0.285714Tm0.428571', '-1.658', '-0.21472187661']
ClSiU 1 ['Si0.111111Cl0.777778U0.111111', '-1.952', '-0.217074714097']
ClSiXe 1 ['Si0.111111Cl0.666667Xe0.222222', '-1.096', '-0.247638139167']
ClSiY 5 ['Si0.222222Cl0.333333Y0.444444', '-1.774', '-0.224042767851']
ClSiYb 7 ['Si0.2Cl0.6Yb0.2', '-2.22', '-0.200753399379']
ClSiZr 1 ['Si0.1Cl0.8Zr0.1', '-2.039', '-0.2446119975']
ClSmSn 6 ['Cl0.625Sn0.125Sm0.25', '-2.022', '-0.224287768717']
ClSmSr 5 ['Cl0.8Sr0.1Sm0.1', '-1.797', '-0.209201340441']
ClSmTa 3 ['Cl0.777778Sm0.111111Ta0.111111', '-1.938', '-0.20584593146']
ClSmTb 3 ['Cl0.75Sm0.125Tb0.125', '-2.373', '-0.22573403114']
ClSmTc 8 ['Cl0.666667Tc0.166667Sm0.166667', '-1.74', '-0.243299303892']
ClSmTe 65 ['Cl0.3Te0.2Sm0.5', '-1.695', '-0.204681674148']
ClSmTh 2 ['Cl0.777778Sm0.111111Th0.111111', '-2.444', '-0.230623329793']
ClSmTi 2 ['Cl0.777778Ti0.111111Sm0.111111', '-2.097', '-0.220140094654']
ClSmTl 31 ['Cl0.5Sm0.375Tl0.125', '-1.867', '-0.21077046313']
ClSmTm 2 ['Cl0.8Sm0.1Tm0.1', '-1.985', '-0.21021898588']
ClSmU 2 ['Cl0.777778Sm0.111111U0.111111', '-2.235', '-0.268614221183']
ClSmV 5 ['Cl0.7V0.2Sm0.1', '-1.829', '-0.201143658568']
ClSmW 4 ['Cl0.7Sm0.2W0.1', '-1.882', '-0.208074340815']
ClSmXe 9 ['Cl0.6Xe0.3Sm0.1', '-0.997', '-0.229456441564']
ClSmY 2 ['Cl0.8Y0.1Sm0.1', '-2.104', '-0.31514238538']

ClSmYb 1 ['Cl0.75Sm0.125Yb0.125', '-2.26', '-0.277143199926']
ClSmZn 7 ['Cl0.7Zn0.2Sm0.1', '-1.818', '-0.220554808818']
ClSmZr 2 ['Cl0.777778Zr0.111111Sm0.111111', '-2.243', '-0.244771237571']
ClSnTb 1 ['Cl0.5Sn0.125Tb0.375', '-1.906', '-0.20611505521']
ClSnTe 35 ['Cl0.6Sn0.3Te0.1', '-1.339', '-0.20095975132']
ClSnTm 3 ['Cl0.333333Sn0.222222Tm0.444444', '-1.724', '-0.205065017321']
ClSnY 5 ['Cl0.4Y0.4Sn0.2', '-1.927', '-0.20118355148']
ClSrTe 4 ['Cl0.555556Sr0.222222Te0.222222', '-2.168', '-0.221472112135']
ClSrZr 1 ['Cl0.7Sr0.2Zr0.1', '-2.735', '-0.29450940857']
ClTaTb 1 ['Cl0.8Tb0.1Ta0.1', '-1.972', '-0.230335680098']
ClTaTe 7 ['Cl0.666667Te0.166667Ta0.166667', '-1.535', '-0.21600816125']
ClTaTl 3 ['Cl0.8Ta0.1Tl0.1', '-1.315', '-0.204800557627']
ClTaTm 1 ['Cl0.8Tm0.1Ta0.1', '-2.138', '-0.339367441066']
ClTaU 1 ['Cl0.8Ta0.1U0.1', '-1.993', '-0.2128163955']
ClTaXe 5 ['Cl0.777778Xe0.111111Ta0.111111', '-1.104', '-0.2246721075']
ClTaY 1 ['Cl0.8Y0.1Ta0.1', '-2.086', '-0.273290840566']
ClTbTc 3 ['Cl0.75Tc0.125Tb0.125', '-1.916', '-0.238875257078']
ClTbTe 31 ['Cl0.625Te0.125Tb0.25', '-2.299', '-0.200073069419']
ClTbTh 1 ['Cl0.8Tb0.1Th0.1', '-2.422', '-0.247235338598']
ClTbTl 11 ['Cl0.666667Tb0.222222Tl0.111111', '-2.314', '-0.202290629662']
ClTbV 2 ['Cl0.7V0.2Tb0.1', '-1.971', '-0.203075807098']
ClTbXe 1 ['Cl0.714286Xe0.142857Tb0.142857', '-1.565', '-0.20747254764']
ClTbY 1 ['Cl0.777778Y0.111111Tb0.111111', '-2.418', '-0.227351919071']
ClTcTe 5 ['Cl0.7Tc0.2Te0.1', '-1.109', '-0.214773947796']
ClTcTh 1 ['Cl0.8Tc0.1Th0.1', '-1.931', '-0.230687122128']
ClTcTi 1 ['Cl0.8Ti0.1Tc0.1', '-1.618', '-0.220552210503']
ClTcTm 1 ['Cl0.8Tc0.1Tm0.1', '-1.741', '-0.257945111194']
ClTcU 1 ['Cl0.8Tc0.1U0.1', '-1.763', '-0.226070426502']
ClTcV 1 ['Cl0.714286V0.142857Tc0.142857', '-1.431', '-0.20852233337']
ClTcXe 4 ['Cl0.714286Tc0.142857Xe0.142857', '-0.901', '-0.221260809825']
ClTcY 2 ['Cl0.777778Y0.111111Tc0.111111', '-1.883', '-0.219520567437']
ClTeTh 42 ['Cl0.625Te0.25Th0.125', '-1.873', '-0.203174573022']
ClTeTi 5 ['Cl0.6Ti0.2Te0.2', '-1.696', '-0.200240714872']
ClTeTl 1 ['Cl0.5Te0.2Tl0.3', '-1.162', '-0.203200361024']
ClTeTm 13 ['Cl0.375Te0.25Tm0.375', '-2.051', '-0.21011330956']
ClTeU 30 ['Cl0.444444Te0.222222U0.333333', '-1.586', '-0.20265774232']
ClTeV 7 ['Cl0.714286V0.142857Te0.142857', '-1.379', '-0.211127940215']
ClTeW 1 ['Cl0.4Te0.4W0.2', '-0.809', '-0.223329395583']
ClTeY 57 ['Cl0.1Y0.5Te0.4', '-1.598', '-0.205714489436']
ClTeYb 20 ['Cl0.6Te0.1Yb0.3', '-2.659', '-0.202774355131']
ClTeZr 23 ['Cl0.555556Zr0.333333Te0.111111', '-1.92', '-0.203558269287']
ClThTl 10 ['Cl0.5Tl0.333333Th0.166667', '-1.761', '-0.203352609271']
ClThV 2 ['Cl0.777778V0.111111Th0.111111', '-2.238', '-0.216582118962']
ClThY 1 ['Cl0.8Y0.1Th0.1', '-2.45', '-0.204190499066']

ClTlTm 3 ['Cl0.444444Tm0.333333Tl0.222222', '-1.805', '-0.204470761579']
ClTlU 3 ['Cl0.6Tl0.3U0.1', '-1.702', '-0.20257331563']
ClTlXe 7 ['Cl0.5Xe0.4Tl0.1', '-0.528', '-0.209195660877']
ClTlY 23 ['Cl0.6Y0.3Tl0.1', '-2.323', '-0.205350532632']
ClTmV 1 ['Cl0.8V0.1Tm0.1', '-1.946', '-0.237689833066']
ClUV 1 ['Cl0.777778V0.111111U0.111111', '-1.987', '-0.227478722851']
ClUW 2 ['Cl0.8W0.1U0.1', '-1.783', '-0.22098537725']
ClVY 1 ['Cl0.8V0.1Y0.1', '-1.993', '-0.270613232566']
CoCrF 1 ['F0.8Cr0.1Co0.1', '-2.52', '-0.285255457114']
CoCrI 2 ['Cr0.111111Co0.222222I0.666667', '-0.361', '-0.202854425048']
CoCrN 3 ['N0.333333Cr0.555556Co0.111111', '-0.658', '-0.230060484145']
CoCsF 11 ['F0.6Co0.1Cs0.3', '-2.68', '-0.201342683186']
CoCsH 2 ['H0.555556Co0.222222Cs0.222222', '-0.271', '-0.20141917507']
CoCsN 33 ['N0.375Co0.375Cs0.25', '-0.353', '-0.202042632996']
CoCsO 11 ['O0.5Co0.166667Cs0.333333', '-1.466', '-0.219619114514']
CoCsS 2 ['S0.5Co0.375Cs0.125', '-0.952', '-0.209908065285']
CoCuF 11 ['F0.666667Co0.111111Cu0.222222', '-1.943', '-0.212449123823']
CoCuN 1 ['N0.4Co0.5Cu0.1', '-0.234', '-0.224367472414']
CoDyF 3 ['F0.75Co0.125Dy0.125', '-3.359', '-0.321378699405']
CoDyH 17 ['H0.7Co0.1Dy0.2', '-0.688', '-0.203690145023']
CoDyI 35 ['Co0.1I0.5Dy0.4', '-1.059', '-0.230388912486']
CoDyN 6 ['N0.4Co0.5Dy0.1', '-0.582', '-0.210654772212']
CoDyO 3 ['O0.6Co0.2Dy0.2', '-2.785', '-0.233212542907']
CoDyP 2 ['P0.5Co0.375Dy0.125', '-1.176', '-0.20435612875']
CoErF 4 ['F0.714286Co0.142857Er0.142857', '-3.525', '-0.245116940187']
CoErH 12 ['H0.7Co0.1Er0.2', '-0.695', '-0.20882145069']
CoErI 34 ['Co0.25I0.375Er0.375', '-0.97', '-0.213034835093']
CoErN 6 ['N0.444444Co0.444444Er0.111111', '-0.631', '-0.203245670441']
CoErO 6 ['O0.625Co0.25Er0.125', '-2.206', '-0.204835114681']
CoErP 2 ['P0.444444Co0.444444Er0.111111', '-1.149', '-0.206934336018']
CoErSe 4 ['Co0.333333Se0.222222Er0.444444', '-1.09', '-0.203035167444']
CoErSn 1 ['Co0.5Sn0.4Er0.1', '-0.469', '-0.213218078759']
CoEuF 2 ['F0.777778Co0.111111Eu0.111111', '-2.649', '-0.261494775668']
CoEuH 1 ['H0.6Co0.2Eu0.2', '-0.626', '-0.240533737126']
CoEuN 46 ['N0.6Co0.2Eu0.2', '-0.443', '-0.207790725897']
CoEuO 2 ['O0.6Co0.1Eu0.3', '-2.631', '-0.203173192369']
CoEuP 8 ['P0.375Co0.125Eu0.5', '-1.107', '-0.217041929143']
CoEuSe 1 ['Co0.125Se0.375Eu0.5', '-1.906', '-0.25083670375']
CoFFe 4 ['F0.7Fe0.1Co0.2', '-2.221', '-0.225479164497']
CoFGa 5 ['F0.75Co0.125Ga0.125', '-2.675', '-0.26533526211']
CoFGd 5 ['F0.7Co0.2Gd0.1', '-2.907', '-0.206414988749']
CoFGe 11 ['F0.666667Co0.166667Ge0.166667', '-2.363', '-0.214127398083']
CoFH 24 ['H0.333333F0.444444Co0.222222', '-1.576', '-0.204644117126']
CoFHf 2 ['F0.777778Co0.111111Hf0.111111', '-3.399', '-0.258150515309']

CoFHg 4 ['F0.625Co0.25Hg0.125', '-1.895', '-0.257697186034']
CoFHo 3 ['F0.75Co0.125Ho0.125', '-3.44', '-0.24528796107']
CoFI 19 ['F0.111111Co0.333333I0.555556', '-0.515', '-0.200084635661']
CoFIn 4 ['F0.7Co0.2In0.1', '-2.427', '-0.277526812997']
CoFK 7 ['F0.666667K0.166667Co0.166667', '-2.508', '-0.238924590857']
CoFKr 4 ['F0.714286Co0.142857Kr0.142857', '-1.482', '-0.235712859431']
CoFLa 2 ['F0.777778Co0.111111La0.111111', '-3.195', '-0.338850255396']
CoFLi 5 ['Li0.142857F0.714286Co0.142857', '-2.317', '-0.252745982637']
CoFLu 6 ['F0.7Co0.1Lu0.2', '-3.773', '-0.276398687829']
CoFMg 3 ['F0.75Mg0.125Co0.125', '-2.787', '-0.37975818023']
CoFMn 5 ['F0.75Mn0.125Co0.125', '-2.528', '-0.20424133372']
CoFMo 3 ['F0.75Co0.125Mo0.125', '-2.57', '-0.211358337525']
CoFN 8 ['N0.142857F0.571429Co0.285714', '-1.689', '-0.203657908894']
CoFNa 6 ['F0.666667Na0.222222Co0.111111', '-2.467', '-0.279844643258']
CoFNb 2 ['F0.777778Co0.111111Nb0.111111', '-3.035', '-0.288003182279']
CoFNd 2 ['F0.777778Co0.111111Nd0.111111', '-3.055', '-0.237628759467']
CoFNi 7 ['F0.7Co0.1Ni0.2', '-2.134', '-0.200093591276']
CoFNp 2 ['F0.777778Co0.111111Np0.111111', '-3.296', '-0.334766448037']
CoFO 27 ['O0.25F0.375Co0.375', '-1.942', '-0.206034265943']
CoFP 6 ['F0.666667P0.166667Co0.166667', '-2.548', '-0.203892842749']
CoFPa 7 ['F0.7Co0.1Pa0.2', '-3.338', '-0.200485876829']
CoFPb 3 ['F0.714286Co0.142857Pb0.142857', '-2.46', '-0.208344991574']
CoFPd 2 ['F0.777778Co0.111111Pd0.111111', '-1.861', '-0.206615476976']
CoFPm 3 ['F0.714286Co0.142857Pm0.142857', '-3.38', '-0.25581107233']
CoFPr 2 ['F0.777778Co0.111111Pr0.111111', '-3.189', '-0.375325497711']
CoFPt 2 ['F0.777778Co0.111111Pt0.111111', '-1.927', '-0.230332204198']
CoFPu 4 ['F0.714286Co0.142857Pu0.142857', '-3.212', '-0.240008759112']
CoFRb 8 ['F0.666667Co0.166667Rb0.166667', '-2.557', '-0.209798617804']
CoFRe 2 ['F0.777778Co0.111111Re0.111111', '-2.273', '-0.258034596463']
CoFRh 2 ['F0.777778Co0.111111Rh0.111111', '-1.942', '-0.279119483542']
CoFRu 2 ['F0.75Co0.125Ru0.125', '-2.149', '-0.26062910836']
CoFS 12 ['F0.571429S0.142857Co0.285714', '-1.83', '-0.20647734053']
CoFSb 3 ['F0.7Co0.2Sb0.1', '-2.296', '-0.209905877135']
CoFSc 3 ['F0.75Sc0.125Co0.125', '-3.434', '-0.32553151336']
CoFSe 10 ['F0.5Co0.166667Se0.333333', '-1.558', '-0.200165655892']
CoFSi 3 ['F0.777778Si0.111111Co0.111111', '-2.987', '-0.263280728642']
CoFSm 2 ['F0.777778Co0.111111Sm0.111111', '-3.213', '-0.382302289933']
CoFSn 4 ['F0.714286Co0.142857Sn0.142857', '-2.488', '-0.201948288135']
CoFSr 4 ['F0.666667Co0.222222Sr0.111111', '-2.756', '-0.2001650849']
CoFTa 1 ['F0.8Co0.1Ta0.1', '-3.165', '-0.304545041362']
CoFTb 1 ['F0.8Co0.1Tb0.1', '-2.868', '-0.376928438472']
CoFTc 1 ['F0.8Co0.1Tc0.1', '-2.325', '-0.428801425903']
CoFTe 14 ['F0.7Co0.1Te0.2', '-2.294', '-0.216117895943']
CoFTh 3 ['F0.75Co0.125Th0.125', '-3.667', '-0.230232275167']

CoFTi 5 ['F0.714286Ti0.142857Co0.142857', '-3.087', '-0.283317100718']
CoFTl 18 ['F0.666667Co0.222222Tl0.111111', '-2.073', '-0.20353638237']
CoFTm 41 ['F0.3Co0.2Tm0.5', '-1.761', '-0.231634540096']
CoFU 2 ['F0.8Co0.1U0.1', '-3.361', '-0.323699837864']
CoFV 2 ['F0.75V0.125Co0.125', '-2.751', '-0.226778117735']
CoFW 1 ['F0.8Co0.1W0.1', '-2.768', '-0.202176692643']
CoFXe 3 ['F0.777778Co0.111111Xe0.111111', '-1.499', '-0.214857085862']
CoFY 4 ['F0.714286Co0.142857Y0.142857', '-3.408', '-0.26732370673']
CoFYb 25 ['F0.4Co0.1Yb0.5', '-2.389', '-0.208773268278']
CoFZn 3 ['F0.75Co0.125Zn0.125', '-2.211', '-0.274780586379']
CoFZr 1 ['F0.8Co0.1Zr0.1', '-3.116', '-0.356677859153']
CoFeN 3 ['N0.333333Fe0.166667Co0.5', '-0.302', '-0.219283929052']
CoGaHf 3 ['Co0.428571Ga0.428571Hf0.142857', '-0.62', '-0.209669394167']
CoGaN 5 ['N0.375Co0.5Ga0.125', '-0.365', '-0.210549882996']
CoGaP 17 ['P0.333333Co0.5Ga0.166667', '-0.787', '-0.203868115763']
CoGaPm 2 ['Co0.111111Ga0.666667Pm0.222222', '-0.627', '-0.256114410278']
CoGdH 6 ['H0.555556Co0.222222Gd0.222222', '-0.698', '-0.205968426379']
CoGdI 29 ['Co0.1I0.8Gd0.1', '-0.597', '-0.207329544275']
CoGdN 3 ['N0.375Co0.125Gd0.5', '-1.565', '-0.237923241748']
CoGdO 1 ['O0.625Co0.125Gd0.25', '-3.112', '-0.34987588303']
CoGdP 3 ['P0.444444Co0.444444Gd0.111111', '-1.157', '-0.222606428981']
CoGdSn 1 ['Co0.333333Sn0.333333Gd0.333333', '-0.763', '-0.250277943653']
CoGeP 1 ['P0.8Co0.1Ge0.1', '-0.577', '-0.237353441374']
CoGePa 4 ['Co0.1Ge0.7Pa0.2', '-0.443', '-0.26801952825']
CoGeSc 1 ['Sc0.555556Co0.111111Ge0.333333', '-1.004', '-0.227310933889']
CoHK 5 ['H0.5K0.2Co0.3', '-0.284', '-0.204851228563']
CoHLa 2 ['H0.444444Co0.222222La0.333333', '-0.665', '-0.216723748474']
CoHLu 1 ['H0.6Co0.2Lu0.2', '-0.638', '-0.229102080126']
CoHN 3 ['H0.166667N0.333333Co0.5', '-0.295', '-0.202197102309']
CoHNd 18 ['H0.5Co0.1Nd0.4', '-0.693', '-0.203587715783']
CoHPm 38 ['H0.375Co0.375Pm0.25', '-0.395', '-0.202416582306']
CoHSc 1 ['H0.5Sc0.4Co0.1', '-0.781', '-0.200898013908']
CoHSe 4 ['H0.222222Co0.333333Se0.444444', '-0.445', '-0.215414258889']
CoHSm 8 ['H0.5Co0.166667Sm0.333333', '-0.717', '-0.200699298908']
CoHTb 15 ['H0.666667Co0.222222Tb0.111111', '-0.403', '-0.208747087457']
CoHTm 13 ['H0.4Co0.2Tm0.4', '-0.709', '-0.203370263126']
CoHY 7 ['H0.6Co0.2Y0.2', '-0.688', '-0.206394399023']
CoHYb 1 ['H0.6Co0.2Yb0.2', '-0.577', '-0.207627192126']
CoHfN 9 ['N0.5Co0.1Hf0.4', '-1.795', '-0.222013581331']
CoHfP 1 ['P0.4Co0.2Hf0.4', '-1.366', '-0.215955429616']
CoHfS 4 ['S0.375Co0.125Hf0.5', '-1.409', '-0.220865344747']
CoHfSi 2 ['Si0.375Co0.375Hf0.25', '-0.99', '-0.206242104629']
CoHgO 3 ['O0.625Co0.25Hg0.125', '-1.112', '-0.214933371199']
CoHoI 26 ['Co0.333333I0.444444Ho0.222222', '-0.733', '-0.214886687625']

CoHoN 8 ['N0.333333Co0.222222Ho0.444444', '-1.52', '-0.218082092847']
CoHoO 3 ['O0.6Co0.3Ho0.1', '-2.128', '-0.219309633788']
CoHoOs 3 ['Co0.125Ho0.625Os0.25', '-0.323', '-0.201809867917']
CoHoP 3 ['P0.5Co0.125Ho0.375', '-1.689', '-0.204716012705']
CoIK 1 ['K0.1Co0.3I0.6', '-0.567', '-0.226426984044']
CoILa 2 ['Co0.25I0.375La0.375', '-1.115', '-0.204620008127']
CoILi 1 ['Li0.125Co0.25I0.625', '-0.558', '-0.203705662858']
CoILu 42 ['Co0.3I0.2Lu0.5', '-0.584', '-0.201451517014']
CoINa 2 ['Na0.1Co0.3I0.6', '-0.528', '-0.220966862458']
CoINd 17 ['Co0.222222I0.444444Nd0.333333', '-1.178', '-0.208778137286']
CoINp 8 ['Co0.1I0.7Np0.2', '-1.023', '-0.211951779549']
CoIO 2 ['O0.6Co0.3I0.1', '-1.386', '-0.253313441861']
CoIPa 31 ['Co0.3I0.4Pa0.3', '-0.423', '-0.233632444514']
CoIPm 39 ['Co0.285714I0.285714Pm0.428571', '-0.725', '-0.206133422521']
CoIPr 16 ['Co0.2I0.6Pr0.2', '-1.28', '-0.201879029544']
CoISc 13 ['Sc0.111111Co0.333333I0.555556', '-0.627', '-0.200724971427']
CoISe 7 ['Co0.285714Se0.285714I0.428571', '-0.381', '-0.215451347143']
CoISi 2 ['Si0.111111Co0.222222I0.666667', '-0.501', '-0.206510118936']
CoISm 33 ['Co0.3I0.5Sm0.2', '-0.958', '-0.205943044535']
CoITa 4 ['Co0.2I0.7Ta0.1', '-0.513', '-0.20396703705']
CoITb 44 ['Co0.3I0.3Tb0.4', '-0.588', '-0.218499141277']
CoITm 17 ['Co0.3I0.5Tm0.2', '-0.974', '-0.218031511783']
CoIV 2 ['V0.111111Co0.222222I0.666667', '-0.415', '-0.204480291714']
CoIW 2 ['Co0.222222I0.666667W0.111111', '-0.245', '-0.217384842222']
CoIXe 14 ['Co0.222222I0.444444Xe0.333333', '-0.223', '-0.201835514476']
CoIY 6 ['Co0.222222Y0.333333I0.444444', '-1.226', '-0.218429226884']
CoIZr 7 ['Co0.125Zr0.125I0.75', '-0.761', '-0.204543416293']
CoInN 36 ['N0.5Co0.125In0.375', '-0.232', '-0.206707880496']
CoKN 31 ['N0.625K0.25Co0.125', '-0.473', '-0.226680378952']
CoKO 6 ['O0.428571K0.428571Co0.142857', '-1.575', '-0.201453235399']
CoLaN 16 ['N0.3Co0.1La0.6', '-1.107', '-0.208831005648']
CoLaO 1 ['O0.625Co0.125La0.25', '-2.958', '-0.231956783719']
CoLiN 31 ['Li0.3N0.3Co0.4', '-0.415', '-0.205277655897']
CoLiO 1 ['Li0.222222O0.444444Co0.333333', '-1.669', '-0.205352468071']
CoLiP 12 ['Li0.166667P0.5Co0.333333', '-0.932', '-0.204823349553']
CoLiS 2 ['Li0.1S0.5Co0.4', '-0.969', '-0.232360683889']
CoLuN 10 ['N0.444444Co0.444444Lu0.111111', '-0.685', '-0.235647847664']
CoLuOs 1 ['Co0.1Lu0.6Os0.3', '-0.464', '-0.20046185925']
CoLuP 3 ['P0.5Co0.4Lu0.1', '-1.146', '-0.23158226002']
CoLuSi 4 ['Si0.333333Co0.555556Lu0.111111', '-0.752', '-0.221297151566']
CoMgN 29 ['N0.6Mg0.1Co0.3', '-0.355', '-0.205517499301']
CoMgO 1 ['O0.6Mg0.1Co0.3', '-1.826', '-0.287886843475']
CoMgS 1 ['Mg0.111111S0.555556Co0.333333', '-1.033', '-0.213974075085']
CoMnS 1 ['S0.5Mn0.2Co0.3', '-0.886', '-0.243087708568']

CoMoN 27 ['N0.5Co0.25Mo0.25', '-0.444', '-0.203757620496']
CoNNa 20 ['N0.6Na0.1Co0.3', '-0.344', '-0.22342017076']
CoNNb 4 ['N0.4Co0.5Nb0.1', '-0.46', '-0.201901132972']
CoNNd 1 ['N0.4Co0.5Nd0.1', '-0.513', '-0.21058747438']
CoNNp 21 ['N0.428571Co0.428571Np0.142857', '-0.561', '-0.206327201484']
CoNO 9 ['N0.142857O0.428571Co0.428571', '-1.217', '-0.207625462731']
CoNOs 1 ['N0.4Co0.5Os0.1', '-0.219', '-0.209367472414']
CoNP 20 ['N0.4P0.1Co0.5', '-0.392', '-0.200905289552']
CoNPa 37 ['N0.625Co0.25Pa0.125', '-0.452', '-0.20019768847']
CoNPm 3 ['N0.375Co0.5Pm0.125', '-0.599', '-0.203982450184']
CoNPr 5 ['N0.444444Co0.444444Pr0.111111', '-0.542', '-0.223871702941']
CoNPu 17 ['N0.6Co0.2Pu0.2', '-0.757', '-0.205498181898']
CoNRb 27 ['N0.428571Co0.285714Rb0.285714', '-0.383', '-0.216440214853']
CoNRe 28 ['N0.25Co0.375Re0.375', '-0.25', '-0.201599777414']
CoNSb 33 ['N0.6Co0.3Sb0.1', '-0.24', '-0.201357538966']
CoNSc 14 ['N0.5Sc0.125Co0.375', '-0.714', '-0.201627020518']
CoNSm 2 ['N0.333333Co0.555556Sm0.111111', '-0.563', '-0.201888855164']
CoNSn 40 ['N0.333333Co0.222222Sn0.444444', '-0.296', '-0.203592476538']
CoNSr 35 ['N0.428571Co0.142857Sr0.428571', '-0.747', '-0.209257522353']
CoNTa 6 ['N0.4Co0.5Ta0.1', '-0.525', '-0.201168181363']
CoNTb 9 ['N0.333333Co0.5Tb0.166667', '-0.695', '-0.209822944939']
CoNTc 20 ['N0.571429Co0.142857Tc0.285714', '-0.28', '-0.202484905641']
CoNTe 17 ['N0.222222Co0.444444Te0.333333', '-0.31', '-0.203090044675']
CoNTh 14 ['N0.5Co0.125Th0.375', '-1.737', '-0.222675836078']
CoNTi 38 ['N0.555556Ti0.111111Co0.333333', '-0.499', '-0.204347910052']
CoNTl 7 ['N0.25Co0.5Tl0.25', '-0.221', '-0.211367472414']
CoNTm 4 ['N0.375Co0.5Tm0.125', '-0.709', '-0.217035254247']
CoNU 1 ['N0.5Co0.125U0.375', '-1.51', '-0.206481660867']
CoNW 47 ['N0.222222Co0.444444W0.333333', '-0.323', '-0.200786619183']
CoNXe 4 ['N0.333333Co0.444444Xe0.222222', '-0.221', '-0.212437753257']
CoNY 6 ['N0.3Co0.2Y0.5', '-1.372', '-0.213886123898']
CoNYb 40 ['N0.555556Co0.333333Yb0.111111', '-0.409', '-0.208617803435']
CoNZn 16 ['N0.4Co0.1Zn0.5', '-0.235', '-0.20415517122']
CoNZr 1 ['N0.375Co0.5Zr0.125', '-0.675', '-0.201531084559']
CoNdSi 2 ['Si0.5Co0.333333Nd0.166667', '-0.926', '-0.213861925417']
CoNiP 1 ['P0.6Co0.2Ni0.2', '-0.871', '-0.210709100424']
CoNiS 1 ['S0.5Co0.375Ni0.125', '-0.812', '-0.201344880106']
CoNpP 1 ['P0.777778Co0.111111Np0.111111', '-0.749', '-0.21047387111']
CoOP 1 ['O0.5P0.1Co0.4', '-1.837', '-0.239316408977']
CoOPa 3 ['O0.7Co0.1Pa0.2', '-2.486', '-0.313334427337']
CoOPb 1 ['O0.6Co0.3Pb0.1', '-1.53', '-0.255474331391']
CoOPr 2 ['O0.666667Co0.111111Pr0.222222', '-2.563', '-0.227755090111']
CoORb 4 ['O0.444444Co0.222222Rb0.333333', '-1.535', '-0.207372211127']
CoOS 3 ['O0.571429S0.142857Co0.285714', '-1.654', '-0.20207806273']

CoOSr 1 ['O0.5Co0.166667Sr0.333333', '-2.626', '-0.271528566401']
CoOTb 1 ['O0.625Co0.125Tb0.25', '-2.872', '-0.255100962336']
CoOTc 1 ['O0.625Co0.125Tc0.25', '-1.788', '-0.218831692703']
CoOTe 5 ['O0.666667Co0.111111Te0.222222', '-1.472', '-0.208760245307']
CoOTh 1 ['O0.666667Co0.111111Th0.222222', '-3.433', '-0.260184876765']
CoOsTm 3 ['Co0.111111Tm0.555556Os0.333333', '-0.355', '-0.215008344444']
CoPPm 23 ['P0.5Co0.375Pm0.125', '-1.035', '-0.20065071302']
CoPSc 16 ['P0.2Sc0.5Co0.3', '-1.08', '-0.20549149846']
CoPSn 2 ['P0.8Co0.1Sn0.1', '-0.595', '-0.232687693689']
CoPTa 40 ['P0.25Co0.125Ta0.625', '-0.885', '-0.202939665746']
CoPTi 7 ['P0.5Ti0.125Co0.375', '-1.073', '-0.205849090985']
CoPTm 16 ['P0.5Co0.1Tm0.4', '-1.73', '-0.20261560627']
CoPV 5 ['P0.333333V0.333333Co0.333333', '-0.94', '-0.201127439097']
CoPXe 1 ['P0.777778Co0.111111Xe0.111111', '-0.509', '-0.204002673958']
CoPY 15 ['P0.375Co0.125Y0.5', '-1.568', '-0.20192150664']
CoPYb 14 ['P0.444444Co0.111111Yb0.444444', '-1.166', '-0.209557734104']
CoPZn 12 ['P0.5Co0.3Zn0.2', '-0.771', '-0.20080501802']
CoPaSe 5 ['Co0.111111Se0.555556Pa0.333333', '-1.107', '-0.203490752222']
CoPdPm 1 ['Co0.1Pd0.7Pm0.2', '-0.544', '-0.2323499855']
CoPmS 7 ['S0.4Co0.2Pm0.4', '-1.739', '-0.242720669392']
CoPmSe 18 ['Co0.2Se0.4Pm0.4', '-1.497', '-0.200215839']
CoPmSi 1 ['Si0.375Co0.5Pm0.125', '-0.697', '-0.210600025938']
CoPmSn 2 ['Co0.166667Sn0.666667Pm0.166667', '-0.499', '-0.201146274391']
CoSTa 16 ['S0.5Co0.166667Ta0.333333', '-1.201', '-0.200682097749']
CoSTc 2 ['S0.5Co0.166667Tc0.333333', '-0.829', '-0.200064492375']
CoScSe 11 ['Sc0.25Co0.25Se0.5', '-1.143', '-0.2011390525']
CoSeTc 4 ['Co0.111111Se0.555556Tc0.333333', '-0.351', '-0.250174322222']
CoSeTh 1 ['Co0.2Se0.4Th0.4', '-1.47', '-0.230675812']
CoSeY 8 ['Co0.285714Se0.428571Y0.285714', '-1.297', '-0.200649803036']
CoSiTh 1 ['Si0.4Co0.5Th0.1', '-0.884', '-0.21444415']
CoSiTi 1 ['Si0.333333Ti0.222222Co0.444444', '-0.938', '-0.218443624899']
CoSiY 1 ['Si0.375Co0.125Y0.5', '-0.967', '-0.223476471875']
CrCsF 6 ['F0.625Cr0.125Cs0.25', '-3.155', '-0.203946368455']
CrCsN 16 ['N0.666667Cr0.111111Cs0.222222', '-0.565', '-0.215300820141']
CrCsO 1 ['O0.666667Cr0.111111Cs0.222222', '-1.795', '-0.218002807748']
CrCuN 8 ['N0.333333Cr0.444444Cu0.222222', '-0.604', '-0.200942287848']
CrCuO 1 ['O0.6Cr0.2Cu0.2', '-1.808', '-0.210695339947']
CrDyF 2 ['F0.8Cr0.1Dy0.1', '-3.397', '-0.289841106782']
CrDyI 11 ['Cr0.25I0.625Dy0.125', '-0.778', '-0.210411206399']
CrDyO 3 ['O0.625Cr0.25Dy0.125', '-3.039', '-0.241924256148']
CrErF 1 ['F0.777778Cr0.111111Er0.111111', '-3.649', '-0.222518297763']
CrErI 12 ['Cr0.2I0.6Er0.2', '-0.886', '-0.208538545045']
CrErN 4 ['N0.5Cr0.3Er0.2', '-1.307', '-0.212095933329']
CrErO 4 ['O0.6Cr0.3Er0.1', '-3.074', '-0.224596223945']

CrEuN 32 ['N0.285714Cr0.142857Eu0.571429', '-0.55', '-0.205085149664']
CrEuO 1 ['O0.6Cr0.2Eu0.2', '-3.064', '-0.26417299912']
CrEuP 5 ['P0.428571Cr0.142857Eu0.428571', '-1.165', '-0.206883324377']
CrFGd 2 ['F0.8Cr0.1Gd0.1', '-3.334', '-0.215966917698']
CrFGe 3 ['F0.714286Cr0.142857Ge0.142857', '-2.845', '-0.206160800261']
CrFH 13 ['H0.4F0.5Cr0.1', '-2.012', '-0.208971074496']
CrFHf 1 ['F0.8Cr0.1Hf0.1', '-3.619', '-0.263933855325']
CrFI 1 ['F0.777778Cr0.111111I0.111111', '-2.491', '-0.306372647349']
CrFIn 1 ['F0.8Cr0.1In0.1', '-2.769', '-0.202078741946']
CrFIr 1 ['F0.8Cr0.1Ir0.1', '-2.38', '-0.22322406082']
CrFK 1 ['F0.666667K0.166667Cr0.166667', '-3.109', '-0.215841415577']
CrFKr 1 ['F0.8Cr0.1Kr0.1', '-1.81', '-0.26053714886']
CrFLa 1 ['F0.777778Cr0.111111La0.111111', '-3.766', '-0.322848468226']
CrFLi 3 ['Li0.25F0.625Cr0.125', '-3.246', '-0.210613663108']
CrFLu 3 ['F0.75Cr0.125Lu0.125', '-3.803', '-0.33826873065']
CrFMg 2 ['F0.777778Mg0.111111Cr0.111111', '-3.131', '-0.238938546047']
CrFMo 1 ['F0.8Cr0.1Mo0.1', '-2.971', '-0.230358462027']
CrFN 1 ['N0.1F0.8Cr0.1', '-1.833', '-0.377096802186']
CrFNa 2 ['F0.777778Na0.111111Cr0.111111', '-2.577', '-0.203021493822']
CrFNi 3 ['F0.75Cr0.125Ni0.125', '-2.564', '-0.208155643311']
CrFNp 1 ['F0.8Cr0.1Np0.1', '-3.395', '-0.20158819478']
CrFO 5 ['O0.285714F0.428571Cr0.285714', '-2.81', '-0.205437759983']
CrFP 4 ['F0.714286P0.142857Cr0.142857', '-2.86', '-0.212945689431']
CrFPa 3 ['F0.75Cr0.125Pa0.125', '-3.532', '-0.291698223775']
CrFPb 2 ['F0.666667Cr0.111111Pb0.222222', '-2.851', '-0.213191078173']
CrFPm 1 ['F0.8Cr0.1Pm0.1', '-3.333', '-0.209392116198']
CrFPr 1 ['F0.8Cr0.1Pr0.1', '-3.42', '-0.210631095198']
CrFPu 3 ['F0.714286Cr0.142857Pu0.142857', '-3.554', '-0.22538980181']
CrFRb 1 ['F0.8Cr0.1Rb0.1', '-2.417', '-0.25892454252']
CrFRe 1 ['F0.8Cr0.1Re0.1', '-2.688', '-0.24510802448']
CrFRu 1 ['F0.8Cr0.1Ru0.1', '-2.512', '-0.251415767825']
CrFSb 2 ['F0.8Cr0.1Sb0.1', '-2.801', '-0.201610225193']
CrFSc 2 ['F0.8Sc0.1Cr0.1', '-3.395', '-0.231163357946']
CrFSi 3 ['F0.75Si0.125Cr0.125', '-3.261', '-0.208344134933']
CrFSm 1 ['F0.8Cr0.1Sm0.1', '-3.442', '-0.217310208198']
CrFSr 1 ['F0.8Cr0.1Sr0.1', '-2.942', '-0.224300505359']
CrFTc 1 ['F0.8Cr0.1Tc0.1', '-2.726', '-0.30149981745']
CrFTe 6 ['F0.714286Cr0.142857Te0.142857', '-2.78', '-0.287434861811']
CrFTi 2 ['F0.8Ti0.1Cr0.1', '-3.232', '-0.33274633607']
CrFTl 2 ['F0.6Cr0.1Tl0.3', '-2.524', '-0.217363707518']
CrFTm 18 ['F0.5Cr0.166667Tm0.333333', '-2.371', '-0.204335450343']
CrFU 1 ['F0.777778Cr0.111111U0.111111', '-3.676', '-0.253592412248']
CrFV 2 ['F0.75V0.125Cr0.125', '-3.195', '-0.202715736275']
CrFY 2 ['F0.777778Cr0.111111Y0.111111', '-3.679', '-0.360790227297']

CrFYb 19 ['F0.428571Cr0.142857Yb0.428571', '-2.451', '-0.251219465612']
CrFeN 5 ['N0.375Cr0.5Fe0.125', '-0.673', '-0.219560073829']
CrFeP 1 ['P0.333333Cr0.444444Fe0.222222', '-0.812', '-0.217897506041']
CrGdI 15 ['Cr0.142857I0.571429Gd0.285714', '-1.015', '-0.200391346467']
CrGdO 1 ['O0.6Cr0.2Gd0.2', '-3.481', '-0.29573531512']
CrGeO 2 ['O0.625Cr0.25Ge0.125', '-2.47', '-0.210597882136']
CrGePa 3 ['Cr0.1Ge0.7Pa0.2', '-0.361', '-0.215707877333']
CrHI 8 ['H0.428571Cr0.142857I0.428571', '-0.396', '-0.200843707933']
CrHN 3 ['H0.166667N0.333333Cr0.5', '-0.677', '-0.228919432114']
CrHPm 6 ['H0.5Cr0.1Pm0.4', '-0.462', '-0.214150785158']
CrHSe 1 ['H0.333333Cr0.166667Se0.5', '-0.429', '-0.20139528']
CrHfN 2 ['N0.5Cr0.125Hf0.375', '-1.744', '-0.211249802954']
CrHfO 1 ['O0.6Cr0.2Hf0.2', '-3.344', '-0.213267809569']
CrHfS 1 ['S0.4Cr0.1Hf0.5', '-1.418', '-0.210523382897']
CrHgI 1 ['Cr0.222222I0.555556Hg0.222222', '-0.545', '-0.203947865321']
CrHgN 2 ['N0.333333Cr0.555556Hg0.111111', '-0.65', '-0.222060484145']
CrHgO 4 ['O0.6Cr0.3Hg0.1', '-2.186', '-0.204562215699']
CrHoI 10 ['Cr0.222222I0.666667Ho0.111111', '-0.804', '-0.273626725645']
CrHoN 15 ['N0.5Cr0.166667Ho0.333333', '-1.64', '-0.218602889549']
CrHoO 4 ['O0.6Cr0.3Ho0.1', '-3.106', '-0.25987511611']
CrILu 18 ['Cr0.3I0.6Lu0.1', '-0.635', '-0.215675302043']
CrINd 7 ['Cr0.2I0.7Nd0.1', '-0.905', '-0.232319786041']
CrINi 8 ['Cr0.2Ni0.3I0.5', '-0.488', '-0.208628760286']
CrINp 2 ['Cr0.111111I0.777778Np0.111111', '-0.794', '-0.209082126567']
CrIO 2 ['O0.625Cr0.25I0.125', '-1.996', '-0.237981249769']
CrIPa 15 ['Cr0.111111I0.555556Pa0.333333', '-0.43', '-0.200566433095']
CrIPd 6 ['Cr0.285714Pd0.142857I0.571429', '-0.553', '-0.200762912898']
CrIPm 18 ['Cr0.3I0.6Pm0.1', '-0.703', '-0.215867849293']
CrIPt 15 ['Cr0.1I0.7Pt0.2', '-0.519', '-0.2079415053']
CrIRh 10 ['Cr0.142857Rh0.142857I0.714286', '-0.384', '-0.207881456449']
CrISe 21 ['Cr0.285714Se0.428571I0.285714', '-0.588', '-0.202404250271']
CrISm 4 ['Cr0.111111I0.777778Sm0.111111', '-0.822', '-0.260728442262']
CrITb 22 ['Cr0.2I0.5Tb0.3', '-0.604', '-0.226615523386']
CrITm 6 ['Cr0.166667I0.666667Tm0.166667', '-1.036', '-0.204522966712']
CrIXe 8 ['Cr0.166667I0.5Xe0.333333', '-0.422', '-0.216528365857']
CrInN 4 ['N0.5Cr0.25In0.25', '-0.499', '-0.206106407328']
CrIrN 3 ['N0.4Cr0.5Ir0.1', '-0.714', '-0.215300698307']
CrKN 19 ['N0.4K0.2Cr0.4', '-0.673', '-0.219188909862']
CrKO 2 ['O0.444444K0.333333Cr0.222222', '-2.147', '-0.218720132']
CrLaN 22 ['N0.5Cr0.1La0.4', '-1.486', '-0.213475895496']
CrLaO 3 ['O0.625Cr0.125La0.25', '-3.451', '-0.258728342721']
CrLiN 32 ['Li0.333333N0.5Cr0.166667', '-0.633', '-0.210887218995']
CrLiO 1 ['Li0.333333O0.444444Cr0.222222', '-2.462', '-0.207776714195']
CrLuN 5 ['N0.5Cr0.4Lu0.1', '-1.083', '-0.232477994828']

CrLuO 4 ['O0.625Cr0.25Lu0.125', '-3.034', '-0.222215098072']
CrMgN 17 ['N0.555556Mg0.111111Cr0.333333', '-0.764', '-0.226154109166']
CrMnN 1 ['N0.444444Cr0.444444Mn0.111111', '-0.731', '-0.217933382157']
CrMnO 1 ['O0.6Cr0.3Mn0.1', '-2.632', '-0.26637983935']
CrMoN 16 ['N0.333333Cr0.111111Mo0.555556', '-0.539', '-0.201945207061']
CrMoO 7 ['O0.7Cr0.1Mo0.2', '-2.45', '-0.208564019337']
CrMoP 3 ['P0.4Cr0.2Mo0.4', '-0.836', '-0.203121102083']
CrNNa 12 ['N0.5Na0.333333Cr0.166667', '-0.518', '-0.201356142789']
CrNNb 21 ['N0.4Cr0.4Nb0.2', '-0.971', '-0.228251969197']
CrNNd 7 ['N0.4Cr0.5Nd0.1', '-0.889', '-0.211074437696']
CrNNi 12 ['N0.375Cr0.375Ni0.25', '-0.629', '-0.203552102996']
CrNNp 17 ['N0.5Cr0.25Np0.25', '-1.116', '-0.226139462639']
CrNO 2 ['N0.1O0.6Cr0.3', '-2.287', '-0.311720886295']
CrNOs 2 ['N0.375Cr0.5Os0.125', '-0.669', '-0.215560073829']
CrNP 1 ['N0.4P0.1Cr0.5', '-0.814', '-0.228336534216']
CrNPa 29 ['N0.375Cr0.5Pa0.125', '-0.844', '-0.219456650426']
CrNPd 3 ['N0.333333Cr0.555556Pd0.111111', '-0.648', '-0.220060484145']
CrNPm 3 ['N0.444444Cr0.444444Pm0.111111', '-0.948', '-0.202377823644']
CrNPr 5 ['N0.444444Cr0.444444Pr0.111111', '-0.941', '-0.228376237533']
CrNPt 11 ['N0.285714Cr0.571429Pt0.142857', '-0.63', '-0.204009808632']
CrNPu 3 ['N0.555556Cr0.222222Pu0.222222', '-1.099', '-0.238384053181']
CrNRb 15 ['N0.5Cr0.2Rb0.3', '-0.56', '-0.216502605328']
CrNRe 15 ['N0.375Cr0.5Re0.125', '-0.675', '-0.221560073829']
CrNRh 20 ['N0.3Cr0.5Rh0.2', '-0.607', '-0.203731266619']
CrNRu 1 ['N0.375Cr0.5Ru0.125', '-0.661', '-0.207560073829']
CrNSc 19 ['N0.428571Sc0.285714Cr0.285714', '-1.553', '-0.204308385806']
CrNSm 8 ['N0.444444Cr0.333333Sm0.222222', '-1.199', '-0.216903127811']
CrNSr 16 ['N0.6Cr0.3Sr0.1', '-0.754', '-0.206313343481']
CrNTa 1 ['N0.333333Cr0.111111Ta0.555556', '-1.151', '-0.203597957695']
CrNTb 10 ['N0.428571Cr0.428571Tb0.142857', '-0.979', '-0.212496585137']
CrNTc 10 ['N0.5Cr0.25Tc0.25', '-0.568', '-0.218950492996']
CrNTh 7 ['N0.5Cr0.25Th0.25', '-1.443', '-0.210694921077']
CrNTi 16 ['N0.555556Ti0.222222Cr0.222222', '-1.038', '-0.20942191792']
CrNTl 1 ['N0.4Cr0.5Tl0.1', '-0.71', '-0.233795286529']
CrNTm 3 ['N0.5Cr0.375Tm0.125', '-1.112', '-0.204219884829']
CrNU 1 ['N0.5Cr0.4U0.1', '-1.018', '-0.206690598495']
CrNW 23 ['N0.5Cr0.333333W0.166667', '-0.655', '-0.203249232328']
CrNXe 9 ['N0.3Cr0.5Xe0.2', '-0.592', '-0.20685443573']
CrNY 8 ['N0.5Cr0.1Y0.4', '-1.766', '-0.21636903133']
CrNYb 29 ['N0.375Cr0.125Yb0.5', '-1.175', '-0.245575033272']
CrNZn 27 ['N0.5Cr0.375Zn0.125', '-0.64', '-0.214552102996']
CrNbO 8 ['O0.666667Cr0.166667Nb0.166667', '-2.895', '-0.206901163287']
CrNbRu 1 ['Cr0.111111Nb0.666667Ru0.222222', '-0.373', '-0.286132288889']
CrNdO 3 ['O0.625Cr0.25Nd0.125', '-3.002', '-0.246834820886']

CrOOs 1 ['O0.625Cr0.25Os0.125', '-2.247', '-0.26038448026']
CrOPa 9 ['O0.6Cr0.3Pa0.1', '-2.83', '-0.245874336171']
CrOPr 1 ['O0.625Cr0.25Pr0.125', '-2.964', '-0.218677869947']
CrORb 3 ['O0.5Cr0.2Rb0.3', '-2.099', '-0.217685014236']
CrORe 3 ['O0.666667Cr0.111111Re0.222222', '-2.232', '-0.26622794547']
CrOS 7 ['O0.3S0.3Cr0.4', '-1.873', '-0.205820153431']
CrOSc 1 ['O0.625Sc0.25Cr0.125', '-3.385', '-0.214117600791']
CrOSi 2 ['O0.625Si0.125Cr0.25', '-2.906', '-0.202336260262']
CrOSn 1 ['O0.6Cr0.3Sn0.1', '-2.616', '-0.307973113761']
CrOSr 2 ['O0.5Cr0.125Sr0.375', '-2.928', '-0.202100006591']
CrOTa 5 ['O0.666667Cr0.111111Ta0.222222', '-3.21', '-0.25291601839']
CrOTb 1 ['O0.6Cr0.2Tb0.2', '-3.298', '-0.23387831169']
CrOTc 1 ['O0.666667Cr0.111111Tc0.222222', '-2.114', '-0.221596682642']
CrOTe 1 ['O0.625Cr0.25Te0.125', '-2.246', '-0.207543620782']
CrOTh 2 ['O0.7Cr0.1Th0.2', '-3.4', '-0.224840796298']
CrOTi 3 ['O0.6Ti0.1Cr0.3', '-2.86', '-0.216692013815']
CrOW 2 ['O0.625Cr0.25W0.125', '-2.589', '-0.210041351509']
CrOsSc 1 ['Sc0.6Cr0.1Os0.3', '-0.427', '-0.2353858975']
CrOsTa 3 ['Cr0.1Ta0.6Os0.3', '-0.422', '-0.200026143833']
CrPTa 15 ['P0.25Cr0.25Ta0.5', '-0.84', '-0.222971830885']
CrPTc 8 ['P0.4Cr0.2Tc0.4', '-0.748', '-0.200642795702']
CrPaPd 1 ['Cr0.1Pd0.8Pa0.1', '-0.469', '-0.203121468']
CrPaS 3 ['S0.666667Cr0.166667Pa0.166667', '-1.285', '-0.245773854342']
CrPaSe 3 ['Cr0.111111Se0.666667Pa0.222222', '-0.9', '-0.21314114']
CrPaSi 3 ['Si0.444444Cr0.444444Pa0.111111', '-0.561', '-0.207989152222']
CrPdPm 6 ['Cr0.166667Pd0.666667Pm0.166667', '-0.464', '-0.204291654583']
CrPmS 2 ['S0.375Cr0.125Pm0.5', '-1.624', '-0.221238127555']
CrPmSe 15 ['Cr0.1Se0.7Pm0.2', '-0.992', '-0.2070450875']
CrPtTh 3 ['Cr0.125Pt0.75Th0.125', '-0.638', '-0.205299285625']
CrSTa 1 ['S0.6Cr0.3Ta0.1', '-1.239', '-0.258235642925']
CrSTc 3 ['S0.555556Cr0.111111Tc0.333333', '-0.908', '-0.200340457909']
CrSXe 1 ['S0.5Cr0.1Xe0.4', '-0.416', '-0.201232470958']
CrSZr 2 ['S0.5Cr0.125Zr0.375', '-1.606', '-0.22276871924']
CrScSe 1 ['Sc0.333333Cr0.111111Se0.555556', '-1.415', '-0.200589018333']
CrSeTc 5 ['Cr0.111111Se0.666667Tc0.222222', '-0.359', '-0.20726352']
CrSeY 10 ['Cr0.1Se0.5Y0.4', '-1.712', '-0.2113977995']
CsCuF 5 ['F0.7Cu0.1Cs0.2', '-2.171', '-0.202695875525']
CsCuSe 1 ['Cu0.2Se0.4Cs0.4', '-0.944', '-0.2005032066']
CsDyF 13 ['F0.571429Cs0.285714Dy0.142857', '-3.449', '-0.257503174147']
CsDyI 9 ['I0.666667Cs0.222222Dy0.111111', '-1.411', '-0.221159731252']
CsDyO 5 ['O0.625Cs0.125Dy0.25', '-3.023', '-0.215272864194']
CsDyS 1 ['S0.555556Cs0.333333Dy0.111111', '-1.651', '-0.218712524253']
CsDyTe 6 ['Te0.666667Cs0.111111Dy0.222222', '-1.22', '-0.208169085415']
CsErF 9 ['F0.625Cs0.25Er0.125', '-3.902', '-0.264623467105']

CsErI 13 ['I0.6Cs0.3Er0.1', '-1.554', '-0.281990629233']
CsErO 1 ['O0.625Cs0.25Er0.125', '-2.184', '-0.212509220701']
CsErS 1 ['S0.5Cs0.125Er0.375', '-2.245', '-0.20918433424']
CsEuF 15 ['F0.5Cs0.166667Eu0.333333', '-3.631', '-0.206216607932']
CsEuN 4 ['N0.333333Cs0.166667Eu0.5', '-0.635', '-0.249406228219']
CsEuO 1 ['O0.666667Cs0.166667Eu0.166667', '-1.929', '-0.211004008164']
CsEuP 6 ['P0.333333Cs0.166667Eu0.5', '-0.965', '-0.219798141182']
CsEuSe 2 ['Se0.375Cs0.125Eu0.5', '-1.86', '-0.20483670375']
CsFFe 6 ['F0.714286Fe0.142857Cs0.142857', '-2.678', '-0.379904734786']
CsFGa 17 ['F0.444444Ga0.111111Cs0.444444', '-2.727', '-0.208754115938']
CsFGd 14 ['F0.7Cs0.1Gd0.2', '-4.118', '-0.22713490711']
CsFGe 5 ['F0.7Ge0.2Cs0.1', '-2.892', '-0.201534947358']
CsFH 33 ['H0.111111F0.555556Cs0.333333', '-2.571', '-0.202332834129']
CsFHf 9 ['F0.5Cs0.375Hf0.125', '-3.019', '-0.200679148088']
CsFHg 10 ['F0.6Cs0.3Hg0.1', '-2.523', '-0.256134262229']
CsFHo 6 ['F0.666667Cs0.222222Ho0.111111', '-3.538', '-0.286802914098']
CsFI 39 ['F0.5I0.125Cs0.375', '-2.612', '-0.201019036281']
CsFIn 11 ['F0.5In0.1Cs0.4', '-2.838', '-0.201239350931']
CsFIr 3 ['F0.7Cs0.2Ir0.1', '-2.458', '-0.204357331232']
CsFK 19 ['F0.555556K0.111111Cs0.333333', '-2.815', '-0.261268538258']
CsFKr 16 ['F0.555556Kr0.111111Cs0.333333', '-2.215', '-0.222360416591']
CsFLa 7 ['F0.5Cs0.375La0.125', '-3.201', '-0.253126563767']
CsFLi 19 ['Li0.111111F0.555556Cs0.333333', '-2.875', '-0.268633592147']
CsFLu 11 ['F0.5Cs0.375Lu0.125', '-3.182', '-0.210336886472']
CsFMg 15 ['F0.5Mg0.2Cs0.3', '-3.103', '-0.208301124307']
CsFMn 4 ['F0.75Mn0.125Cs0.125', '-2.635', '-0.208677948586']
CsFMo 7 ['F0.571429Mo0.142857Cs0.285714', '-2.772', '-0.218096569065']
CsFN 11 ['N0.222222F0.666667Cs0.111111', '-0.859', '-0.2205838382']
CsFNa 21 ['F0.5Na0.333333Cs0.166667', '-3.156', '-0.228993666817']
CsFNb 3 ['F0.6Nb0.1Cs0.3', '-3.265', '-0.299270992518']
CsFNd 8 ['F0.571429Cs0.285714Nd0.142857', '-3.535', '-0.215859863826']
CsFNi 3 ['F0.75Ni0.125Cs0.125', '-2.187', '-0.371358818926']
CsFNp 13 ['F0.625Cs0.25Np0.125', '-3.474', '-0.200264391945']
CsFO 23 ['O0.1F0.5Cs0.4', '-2.489', '-0.206640260932']
CsFOs 5 ['F0.666667Cs0.222222Os0.111111', '-2.477', '-0.204426688538']
CsFP 24 ['F0.666667P0.222222Cs0.111111', '-2.732', '-0.200492340691']
CsFPa 15 ['F0.5Cs0.4Pa0.1', '-2.948', '-0.201928647431']
CsFPb 8 ['F0.666667Cs0.222222Pb0.111111', '-2.675', '-0.234246055024']
CsFPd 4 ['F0.7Pd0.1Cs0.2', '-2.33', '-0.208336191524']
CsFPm 9 ['F0.571429Cs0.285714Pm0.142857', '-3.417', '-0.209837468829']
CsFPr 13 ['F0.5Cs0.375Pr0.125', '-3.144', '-0.243911211372']
CsFPt 7 ['F0.666667Cs0.222222Pt0.111111', '-2.607', '-0.20200247502']
CsFPu 22 ['F0.5Cs0.3Pu0.2', '-2.943', '-0.202406491431']
CsFRb 19 ['F0.555556Rb0.111111Cs0.333333', '-2.774', '-0.232632504369']

CsFRe 2 ['F0.625Cs0.25Re0.125', '-2.789', '-0.229750498918']
CsFRh 12 ['F0.666667Rh0.222222Cs0.111111', '-2.163', '-0.204269788725']
CsFRu 4 ['F0.714286Ru0.142857Cs0.142857', '-2.567', '-0.222824241762']
CsFS 8 ['F0.666667S0.111111Cs0.222222', '-2.513', '-0.200190103986']
CsFSc 12 ['F0.666667Sc0.222222Cs0.111111', '-4.007', '-0.211592346133']
CsFSe 14 ['F0.75Se0.125Cs0.125', '-2.153', '-0.205773952162']
CsFSi 14 ['F0.444444Si0.111111Cs0.444444', '-2.755', '-0.236754115938']
CsFSm 5 ['F0.666667Cs0.222222Sm0.111111', '-3.471', '-0.228849238636']
CsFSn 13 ['F0.571429Sn0.285714Cs0.142857', '-2.645', '-0.200804819383']
CsFSr 18 ['F0.555556Sr0.111111Cs0.333333', '-3.511', '-0.22031970159']
CsFTa 11 ['F0.6Cs0.3Ta0.1', '-3.177', '-0.228215860118']
CsFTb 4 ['F0.75Cs0.125Tb0.125', '-3.131', '-0.265285822459']
CsFTc 4 ['F0.75Tc0.125Cs0.125', '-2.534', '-0.419085281694']
CsFTe 6 ['F0.75Te0.125Cs0.125', '-2.723', '-0.202540712889']
CsFTh 11 ['F0.6Cs0.3Th0.1', '-3.661', '-0.266410175768']
CsFTi 11 ['F0.555556Ti0.111111Cs0.333333', '-3.217', '-0.218126554644']
CsFTl 8 ['F0.625Cs0.125Tl0.25', '-2.45', '-0.201515478874']
CsFTm 27 ['F0.5Cs0.166667Tm0.333333', '-2.678', '-0.280895513763']
CsFU 12 ['F0.7Cs0.2U0.1', '-3.615', '-0.223330535108']
CsFV 4 ['F0.75V0.125Cs0.125', '-3.066', '-0.350248350234']
CsFW 1 ['F0.8Cs0.1W0.1', '-2.86', '-0.238829710404']
CsFXe 3 ['F0.555556Xe0.222222Cs0.222222', '-1.822', '-0.204012883258']
CsFY 11 ['F0.666667Y0.111111Cs0.222222', '-3.417', '-0.230279662271']
CsFYb 10 ['F0.714286Cs0.142857Yb0.142857', '-2.971', '-0.201391627514']
CsFZn 11 ['F0.6Zn0.1Cs0.3', '-2.753', '-0.223518025925']
CsFZr 6 ['F0.75Zr0.125Cs0.125', '-3.489', '-0.208386574216']
CsFeN 2 ['N0.666667Fe0.111111Cs0.222222', '-0.482', '-0.207496532363']
CsFeO 1 ['O0.666667Fe0.111111Cs0.222222', '-1.398', '-0.200132932806']
CsGaO 7 ['O0.333333Ga0.333333Cs0.333333', '-1.468', '-0.205501372328']
CsGaS 2 ['S0.4Ga0.2Cs0.4', '-1.291', '-0.210359295393']
CsGaSe 14 ['Ga0.4Se0.4Cs0.2', '-0.984', '-0.2099655909']
CsGdI 13 ['I0.6Cs0.2Gd0.2', '-1.42', '-0.222064216293']
CsGdO 6 ['O0.7Cs0.2Gd0.1', '-1.854', '-0.21801021951']
CsGdS 2 ['S0.5Cs0.166667Gd0.333333', '-2.134', '-0.200084093825']
CsGdTe 1 ['Te0.555556Cs0.111111Gd0.333333', '-1.555', '-0.204598832453']
CsGeN 1 ['N0.5Ge0.333333Cs0.166667', '-0.417', '-0.210441536303']
CsGeO 10 ['O0.3Ge0.1Cs0.6', '-1.245', '-0.231887573333']
CsGePa 2 ['Ge0.7Cs0.1Pa0.2', '-0.395', '-0.2250152865']
CsGeS 3 ['S0.4Ge0.3Cs0.3', '-1.039', '-0.211312738614']
CsGeTe 6 ['Ge0.2Te0.7Cs0.1', '-0.447', '-0.21194993275']
CsHMn 1 ['H0.571429Mn0.285714Cs0.142857', '-0.253', '-0.208269469688']
CsHO 2 ['H0.571429O0.285714Cs0.142857', '-1.315', '-0.214270019526']
CsHP 9 ['H0.4P0.2Cs0.4', '-0.513', '-0.220326655959']
CsHPm 8 ['H0.666667Cs0.166667Pm0.166667', '-0.516', '-0.215965166461']

CsHPt 4 ['H0.555556Cs0.111111Pt0.333333', '-0.407', '-0.212406738161']
CsHRh 2 ['H0.666667Rh0.222222Cs0.111111', '-0.244', '-0.209209587535']
CsHS 5 ['H0.571429S0.285714Cs0.142857', '-0.681', '-0.202401708274']
CsHSb 6 ['H0.5Sb0.25Cs0.25', '-0.436', '-0.202888833437']
CsHSe 17 ['H0.375Se0.25Cs0.375', '-0.823', '-0.220993318437']
CsHSi 10 ['H0.5Si0.125Cs0.375', '-0.319', '-0.201582357931']
CsHfO 3 ['O0.5Cs0.333333Hf0.166667', '-2.676', '-0.20132848289']
CsHfS 18 ['S0.6Cs0.1Hf0.3', '-1.839', '-0.204377063217']
CsHfSe 4 ['Se0.5Cs0.166667Hf0.333333', '-1.413', '-0.232660255']
CsHgO 10 ['O0.25Cs0.375Hg0.375', '-0.926', '-0.206467546654']
CsHgS 1 ['S0.25Cs0.375Hg0.375', '-0.884', '-0.203087448684']
CsHgSe 29 ['Se0.166667Cs0.333333Hg0.5', '-0.705', '-0.20288095']
CsHoI 13 ['I0.6Cs0.3Ho0.1', '-1.485', '-0.220203189337']
CsILu 16 ['I0.571429Cs0.142857Lu0.285714', '-1.082', '-0.251772921827']
CsINd 9 ['I0.8Cs0.1Nd0.1', '-1.039', '-0.204269063042']
CsINp 5 ['I0.8Cs0.1Np0.1', '-1.037', '-0.225240210424']
CsIO 16 ['O0.5I0.1Cs0.4', '-1.456', '-0.204954122652']
CsIP 2 ['P0.142857I0.714286Cs0.142857', '-0.871', '-0.2089459527']
CsIPa 12 ['I0.625Cs0.125Pa0.25', '-0.755', '-0.201382932858']
CsIPm 17 ['I0.555556Cs0.333333Pm0.111111', '-1.542', '-0.210108143098']
CsIPr 4 ['I0.666667Cs0.111111Pr0.222222', '-1.776', '-0.216238092828']
CsIPu 3 ['I0.714286Cs0.142857Pu0.142857', '-1.457', '-0.254003217195']
CsIRh 2 ['Rh0.222222I0.666667Cs0.111111', '-0.662', '-0.207981462254']
CsISc 7 ['Sc0.142857I0.714286Cs0.142857', '-1.295', '-0.213880478264']
CsISe 12 ['Se0.1I0.4Cs0.5', '-1.72', '-0.217959063782']
CsISi 11 ['Si0.1I0.6Cs0.3', '-1.396', '-0.205766363046']
CsISm 10 ['I0.8Cs0.1Sm0.1', '-0.998', '-0.20752189455']
CsITb 15 ['I0.571429Cs0.285714Tb0.142857', '-1.333', '-0.227211835448']
CsITe 29 ['Te0.222222I0.222222Cs0.555556', '-1.471', '-0.204254904832']
CsITh 3 ['I0.7Cs0.1Th0.2', '-1.578', '-0.207369924052']
CsITm 1 ['I0.7Cs0.1Tm0.2', '-1.625', '-0.371401990789']
CsIU 1 ['I0.75Cs0.125U0.125', '-1.298', '-0.243679602867']
CsIV 1 ['V0.142857I0.714286Cs0.142857', '-1.006', '-0.209083046123']
CsIZr 1 ['Zr0.142857I0.714286Cs0.142857', '-1.36', '-0.224906128978']
CsInO 3 ['O0.6In0.2Cs0.2', '-1.701', '-0.202954438306']
CsInSe 27 ['Se0.333333In0.166667Cs0.5', '-1.053', '-0.212025988125']
CsIrN 8 ['N0.625Cs0.25Ir0.125', '-0.461', '-0.209404388327']
CsIrO 4 ['O0.666667Cs0.222222Ir0.111111', '-1.343', '-0.251789115246']
CsIrP 1 ['P0.555556Cs0.222222Ir0.222222', '-0.923', '-0.217227802615']
CsIrSe 4 ['Se0.555556Cs0.222222Ir0.222222', '-0.781', '-0.201256934445']
CsKO 17 ['O0.7K0.1Cs0.2', '-1.204', '-0.206814855634']
CsKSe 7 ['K0.25Se0.375Cs0.375', '-1.22', '-0.201479723437']
CsKrO 2 ['O0.6Kr0.2Cs0.2', '-0.936', '-0.232999632973']
CsKrS 5 ['S0.5Kr0.4Cs0.1', '-0.507', '-0.200028636796']

CsLaO 2 ['O0.666667Cs0.222222La0.111111', '-1.977', '-0.215186222679']
CsLaTe 1 ['Te0.571429Cs0.142857La0.285714', '-1.573', '-0.202282103005']
CsLuSe 5 ['Se0.5Cs0.2Lu0.3', '-1.77', '-0.2225986305']
CsLuTe 3 ['Te0.571429Cs0.142857Lu0.285714', '-1.288', '-0.257905224881']
CsMgS 1 ['Mg0.5S0.4Cs0.1', '-1.477', '-0.208546242062']
CsMnN 15 ['N0.3Mn0.5Cs0.2', '-0.549', '-0.261480148459']
CsMnO 2 ['O0.625Mn0.125Cs0.25', '-1.7', '-0.243691831282']
CsMoN 8 ['N0.625Mo0.125Cs0.25', '-0.535', '-0.216010452806']
CsMoO 12 ['O0.6Mo0.2Cs0.2', '-2.323', '-0.20884647147']
CsMoSe 10 ['Se0.444444Mo0.222222Cs0.333333', '-1.019', '-0.200681049131']
CsNNi 2 ['N0.714286Ni0.142857Cs0.142857', '-0.41', '-0.23353848436']
CsNNp 14 ['N0.444444Cs0.111111Np0.444444', '-1.282', '-0.204260255956']
CsNOs 11 ['N0.6Cs0.1Os0.3', '-0.354', '-0.233234106397']
CsNP 19 ['N0.555556P0.333333Cs0.111111', '-0.816', '-0.200611087907']
CsNPa 15 ['N0.5Cs0.3Pa0.2', '-0.761', '-0.218219564772']
CsNPt 9 ['N0.5Cs0.166667Pt0.333333', '-0.41', '-0.208723510661']
CsNPu 1 ['N0.555556Cs0.111111Pu0.333333', '-1.376', '-0.373795770736']
CsNRe 10 ['N0.666667Cs0.166667Re0.166667', '-0.457', '-0.236352862309']
CsNRh 16 ['N0.6Rh0.3Cs0.1', '-0.324', '-0.203234106397']
CsNRu 9 ['N0.7Ru0.1Cs0.2', '-0.458', '-0.216468212794']
CsNTc 9 ['N0.5Tc0.2Cs0.3', '-0.435', '-0.214952883217']
CsNTi 1 ['N0.5Ti0.4Cs0.1', '-1.387', '-0.255441514035']
CsNU 3 ['N0.5Cs0.2U0.3', '-1.412', '-0.207089256826']
CsNW 17 ['N0.5Cs0.1W0.4', '-0.403', '-0.211099353328']
CsNaP 1 ['Na0.4P0.4Cs0.2', '-0.751', '-0.210998698297']
CsNaS 13 ['Na0.285714S0.428571Cs0.285714', '-1.369', '-0.20805565375']
CsNaSe 13 ['Na0.3Se0.5Cs0.2', '-1.143', '-0.201097541746']
CsNbO 3 ['O0.5Nb0.166667Cs0.333333', '-2.435', '-0.205182941225']
CsNbS 7 ['S0.555556Nb0.111111Cs0.333333', '-1.342', '-0.208420386657']
CsNbSe 2 ['Se0.444444Nb0.111111Cs0.444444', '-1.331', '-0.218846314446']
CsNdO 4 ['O0.666667Cs0.166667Nd0.166667', '-2.334', '-0.23747126525']
CsNdS 1 ['S0.5Cs0.333333Nd0.166667', '-1.802', '-0.232120217992']
CsNdSe 3 ['Se0.555556Cs0.111111Nd0.333333', '-1.862', '-0.200885669167']
CsNdTe 4 ['Te0.555556Cs0.222222Nd0.222222', '-1.481', '-0.212901806955']
CsNpO 2 ['O0.625Cs0.25Np0.125', '-2.37', '-0.239135934669']
CsNpS 13 ['S0.5Cs0.375Np0.125', '-1.473', '-0.20718360346']
CsNpSe 9 ['Se0.6Cs0.2Np0.2', '-1.175', '-0.205611276584']
CsNpTe 2 ['Te0.571429Cs0.142857Np0.285714', '-0.929', '-0.206378233452']
CsOOs 6 ['O0.6Cs0.2Os0.2', '-1.765', '-0.34040827147']
CsOP 18 ['O0.3P0.2Cs0.5', '-1.758', '-0.20138607005']
CsOPa 12 ['O0.6Cs0.3Pa0.1', '-2.242', '-0.242686592722']
CsOPb 5 ['O0.666667Cs0.166667Pb0.166667', '-1.334', '-0.24144257525']
CsOPd 4 ['O0.6Pd0.2Cs0.2', '-1.196', '-0.293908416474']
CsOPr 2 ['O0.625Cs0.25Pr0.125', '-2.096', '-0.224688920936']

CsOPt 11 ['O0.375Cs0.5Pt0.125', '-1.227', '-0.204257865294']
CsOPu 11 ['O0.6Cs0.3Pu0.1', '-2.017', '-0.207072742477']
CsORb 17 ['O0.333333Rb0.5Cs0.166667', '-1.275', '-0.211737863872']
CsORe 1 ['O0.625Cs0.25Re0.125', '-2.171', '-0.262794962572']
CsORh 1 ['O0.625Rh0.25Cs0.125', '-1.356', '-0.24173743945']
CsORu 7 ['O0.666667Ru0.222222Cs0.111111', '-1.417', '-0.215904178303']
CsOS 1 ['O0.3S0.1Cs0.6', '-1.496', '-0.20944116402']
CsOSb 4 ['O0.666667Sb0.111111Cs0.222222', '-1.605', '-0.206843667957']
CsOSe 24 ['O0.1Se0.3Cs0.6', '-1.318', '-0.2002604056']
CsOSm 2 ['O0.625Cs0.25Sm0.125', '-2.145', '-0.234638338123']
CsOSn 6 ['O0.444444Sn0.222222Cs0.333333', '-1.874', '-0.218048397915']
CsOTa 1 ['O0.666667Cs0.222222Ta0.111111', '-2.225', '-0.253922569203']
CsOTb 2 ['O0.666667Cs0.222222Tb0.111111', '-1.926', '-0.206483613967']
CsOTc 2 ['O0.625Tc0.25Cs0.125', '-2.077', '-0.275477949293']
CsOTe 11 ['O0.4Te0.1Cs0.5', '-1.606', '-0.204382431733']
CsOTh 2 ['O0.625Cs0.25Th0.125', '-2.61', '-0.316491249921']
CsOTi 10 ['O0.375Ti0.125Cs0.5', '-1.87', '-0.225456089737']
CsOTl 13 ['O0.375Cs0.5Tl0.125', '-1.264', '-0.200998887793']
CsOTm 2 ['O0.625Cs0.25Tm0.125', '-2.217', '-0.234508941483']
CsOU 2 ['O0.666667Cs0.111111U0.222222', '-3.356', '-0.212663716419']
CsOV 4 ['O0.375V0.125Cs0.5', '-1.698', '-0.20707487901']
CsOW 3 ['O0.6Cs0.3W0.1', '-1.949', '-0.212273355725']
CsOZn 1 ['O0.333333Zn0.111111Cs0.555556', '-1.383', '-0.211352725087']
CsOZr 1 ['O0.625Zr0.125Cs0.25', '-2.493', '-0.397544191742']
CsOsP 1 ['P0.555556Cs0.111111Os0.333333', '-0.779', '-0.202704634466']
CsPPb 1 ['P0.5Cs0.1Pb0.4', '-0.437', '-0.228063530409']
CsPPm 6 ['P0.5Cs0.125Pm0.375', '-1.259', '-0.234438586874']
CsPRh 3 ['P0.8Rh0.1Cs0.1', '-0.718', '-0.200551702971']
CsPRu 14 ['P0.4Ru0.5Cs0.1', '-0.763', '-0.208069197666']
CsPS 6 ['P0.142857S0.428571Cs0.428571', '-1.209', '-0.21194372246']
CsPSe 1 ['P0.111111Se0.333333Cs0.555556', '-1.18', '-0.233067093522']
CsPTe 8 ['P0.1Te0.5Cs0.4', '-0.957', '-0.20237327958']
CsPZn 1 ['P0.333333Zn0.333333Cs0.333333', '-0.609', '-0.254031990208']
CsPaS 14 ['S0.7Cs0.2Pa0.1', '-1.219', '-0.211236139065']
CsPaSe 13 ['Se0.5Cs0.375Pa0.125', '-1.192', '-0.214741479062']
CsPaSi 1 ['Si0.7Cs0.1Pa0.2', '-0.394', '-0.259212287062']
CsPaTe 2 ['Te0.7Cs0.2Pa0.1', '-0.739', '-0.2130790705']
CsPbS 4 ['S0.4Cs0.2Pb0.4', '-0.939', '-0.202714717145']
CsPbSe 4 ['Se0.4Cs0.3Pb0.3', '-0.944', '-0.2076192775']
CsPdS 3 ['S0.5Pd0.2Cs0.3', '-1.063', '-0.204981874491']
CsPmS 15 ['S0.555556Cs0.222222Pm0.222222', '-1.918', '-0.207940322768']
CsPmSe 20 ['Se0.5Cs0.333333Pm0.166667', '-1.366', '-0.224560215417']
CsPmTe 5 ['Te0.666667Cs0.166667Pm0.166667', '-0.97', '-0.201847826388']
CsPrS 3 ['S0.5Cs0.3Pr0.2', '-1.894', '-0.231987720742']

CsPrSe 5 ['Se0.555556Cs0.111111Pr0.333333', '-1.882', '-0.206709176224']
CsPtSe 3 ['Se0.444444Cs0.444444Pt0.111111', '-1.079', '-0.216235093519']
CsPuS 19 ['S0.5Cs0.4Pu0.1', '-1.513', '-0.233700766616']
CsPuSe 3 ['Se0.444444Cs0.111111Pu0.444444', '-1.576', '-0.201806511111']
CsRbSe 9 ['Se0.4Rb0.4Cs0.2', '-1.203', '-0.202855657498']
CsReS 19 ['S0.5Cs0.166667Re0.333333', '-1.02', '-0.202789105684']
CsRuS 1 ['S0.5Ru0.166667Cs0.333333', '-1.147', '-0.231357118825']
CsSSe 35 ['S0.375Se0.375Cs0.25', '-0.85', '-0.201931375432']
CsSSm 1 ['S0.5Cs0.1Sm0.4', '-2.256', '-0.229584524741']
CsSTa 6 ['S0.666667Cs0.111111Ta0.222222', '-1.332', '-0.203842200588']
CsSTb 2 ['S0.5Cs0.2Tb0.3', '-1.919', '-0.233023508096']
CsSTc 7 ['S0.5Tc0.125Cs0.375', '-1.191', '-0.207419042681']
CsSTe 25 ['S0.3Te0.4Cs0.3', '-0.912', '-0.20511810545']
CsSTh 8 ['S0.6Cs0.1Th0.3', '-2.208', '-0.202716094464']
CsSTl 10 ['S0.4Cs0.3Tl0.3', '-1.007', '-0.205856782102']
CsSTm 1 ['S0.444444Cs0.111111Tm0.444444', '-2.043', '-0.201941842658']
CsSU 7 ['S0.5Cs0.125U0.375', '-1.695', '-0.216463451429']
CsSXe 5 ['S0.555556Xe0.333333Cs0.111111', '-0.547', '-0.205920707551']
CsSY 2 ['S0.5Y0.3Cs0.2', '-2.108', '-0.220963960246']
CsSZr 12 ['S0.444444Zr0.444444Cs0.111111', '-1.565', '-0.203026052241']
CsScSe 9 ['Sc0.25Se0.5Cs0.25', '-1.479', '-0.231159335625']
CsSeSi 9 ['Si0.222222Se0.333333Cs0.444444', '-1.012', '-0.207463307708']
CsSeSm 1 ['Se0.5Cs0.2Sm0.3', '-1.83', '-0.21555375225']
CsSeTa 18 ['Se0.571429Cs0.142857Ta0.285714', '-1.034', '-0.202646239167']
CsSeTb 5 ['Se0.555556Cs0.111111Tb0.333333', '-1.719', '-0.203049222608']
CsSeTc 15 ['Se0.5Tc0.2Cs0.3', '-0.839', '-0.2089036535']
CsSeTe 38 ['Se0.25Te0.5Cs0.25', '-0.749', '-0.215256184166']
CsSeTh 2 ['Se0.571429Cs0.142857Th0.285714', '-1.755', '-0.200317076426']
CsSeTl 9 ['Se0.3Cs0.4Tl0.3', '-0.898', '-0.207140072']
CsSeTm 6 ['Se0.5Cs0.1Tm0.4', '-1.737', '-0.200032137001']
CsSeU 1 ['Se0.5Cs0.3U0.2', '-1.301', '-0.2123171336']
CsSeV 4 ['V0.1Se0.5Cs0.4', '-1.087', '-0.20899225685']
CsSeY 18 ['Se0.571429Y0.142857Cs0.285714', '-1.311', '-0.206365033571']
CsSeZn 2 ['Zn0.1Se0.4Cs0.5', '-1.14', '-0.21338428375']
CsSeZr 3 ['Se0.555556Zr0.333333Cs0.111111', '-1.547', '-0.257647648564']
CsTbTe 1 ['Te0.555556Cs0.111111Tb0.333333', '-1.302', '-0.328441747148']
CsTeTm 2 ['Te0.6Cs0.1Tm0.3', '-1.314', '-0.239256748751']
CsTeU 4 ['Te0.625Cs0.25U0.125', '-0.93', '-0.208418360417']
CsTeY 3 ['Y0.285714Te0.571429Cs0.142857', '-1.388', '-0.233870439167']
CuDyF 3 ['F0.75Cu0.125Dy0.125', '-2.961', '-0.248051677839']
CuDyI 23 ['Cu0.333333I0.5Dy0.166667', '-0.74', '-0.219783443508']
CuDyN 2 ['N0.4Cu0.1Dy0.5', '-1.779', '-0.281427042525']
CuErF 2 ['F0.777778Cu0.111111Er0.111111', '-2.795', '-0.244118287986']
CuErI 23 ['Cu0.2I0.5Er0.3', '-0.966', '-0.211922212289']

CuErO 2 ['O0.6Cu0.1Er0.3', '-3.42', '-0.297872195325']
CuErOs 1 ['Cu0.1Er0.6Os0.3', '-0.47', '-0.216690531']
CuEuF 3 ['F0.75Cu0.125Eu0.125', '-2.596', '-0.23472960106']
CuEuN 45 ['N0.142857Cu0.428571Eu0.428571', '-0.548', '-0.200450435397']
CuEuO 2 ['O0.625Cu0.125Eu0.25', '-2.25', '-0.249984135654']
CuEuP 5 ['P0.333333Cu0.111111Eu0.555556', '-1.065', '-0.201591930905']
CuEuS 1 ['S0.375Cu0.125Eu0.5', '-2.159', '-0.23787929318']
CuEuSe 7 ['Cu0.142857Se0.428571Eu0.428571', '-2.101', '-0.209384804286']
CuFFe 6 ['F0.666667Fe0.111111Cu0.222222', '-2.129', '-0.200422053081']
CuFGa 3 ['F0.8Cu0.1Ga0.1', '-1.979', '-0.311006592435']
CuFGd 3 ['F0.75Cu0.125Gd0.125', '-3.056', '-0.329458941484']
CuFGe 10 ['F0.6Cu0.2Ge0.2', '-2.186', '-0.202780249273']
CuFH 12 ['H0.375F0.5Cu0.125', '-1.753', '-0.210302507427']
CuFHf 1 ['F0.8Cu0.1Hf0.1', '-2.84', '-0.272973846525']
CuFHg 5 ['F0.714286Cu0.142857Hg0.142857', '-1.666', '-0.215011569074']
CuFHo 2 ['F0.777778Cu0.111111Ho0.111111', '-2.772', '-0.220854168448']
CuFI 1 ['F0.666667Cu0.111111I0.222222', '-1.759', '-0.206642139663']
CuFIn 3 ['F0.75Cu0.125In0.125', '-2.258', '-0.220348721794']
CuFIr 5 ['F0.777778Cu0.111111Ir0.111111', '-1.894', '-0.202235943039']
CuFK 12 ['F0.5K0.4Cu0.1', '-2.826', '-0.205352514994']
CuFKr 5 ['F0.7Cu0.2Kr0.1', '-1.356', '-0.224234422024']
CuFLa 3 ['F0.75Cu0.125La0.125', '-3.143', '-0.254504515754']
CuFLi 9 ['Li0.25F0.625Cu0.125', '-2.425', '-0.201182917309']
CuFLu 7 ['F0.571429Cu0.142857Lu0.285714', '-3.366', '-0.217711916686']
CuFMg 2 ['F0.777778Mg0.111111Cu0.111111', '-2.182', '-0.33082769659']
CuFMn 2 ['F0.777778Mn0.111111Cu0.111111', '-2.179', '-0.215610394744']
CuFN 5 ['N0.1F0.7Cu0.2', '-1.284', '-0.24579407535']
CuFNa 10 ['F0.666667Na0.111111Cu0.222222', '-2.074', '-0.233661193722']
CuFNb 1 ['F0.8Cu0.1Nb0.1', '-2.764', '-0.291827465107']
CuFNd 1 ['F0.8Cu0.1Nd0.1', '-2.523', '-0.247104266267']
CuFNi 3 ['F0.75Ni0.125Cu0.125', '-1.895', '-0.338431780544']
CuFNp 2 ['F0.777778Cu0.111111Np0.111111', '-3.141', '-0.468364651089']
CuFO 9 ['O0.222222F0.666667Cu0.111111', '-0.799', '-0.22221893075']
CuFP 1 ['F0.8P0.1Cu0.1', '-2.475', '-0.244145468211']
CuFPa 10 ['F0.6Cu0.3Pa0.1', '-2.514', '-0.296395385772']
CuFPb 2 ['F0.777778Cu0.111111Pb0.111111', '-1.979', '-0.220587927806']
CuFPd 2 ['F0.777778Cu0.111111Pd0.111111', '-1.738', '-0.372213680028']
CuFPm 2 ['F0.8Cu0.1Pm0.1', '-2.416', '-0.229192351687']
CuFPr 1 ['F0.777778Cu0.111111Pr0.111111', '-2.778', '-0.252923700763']
CuFPt 1 ['F0.777778Cu0.111111Pt0.111111', '-1.775', '-0.243375370815']
CuFPu 3 ['F0.75Cu0.125Pu0.125', '-3.256', '-0.655663415544']
CuFRb 10 ['F0.666667Cu0.222222Rb0.111111', '-2.089', '-0.237091002706']
CuFRh 4 ['F0.666667Cu0.222222Rh0.111111', '-1.886', '-0.200770365396']
CuFRu 6 ['F0.7Cu0.2Ru0.1', '-1.974', '-0.20393870711']

CuFS 2 ['F0.6S0.1Cu0.3', '-1.781', '-0.205219034838']
CuFSb 2 ['F0.777778Cu0.111111Sb0.111111', '-2.214', '-0.32894572303']
CuFSc 3 ['F0.75Sc0.125Cu0.125', '-3.024', '-0.240204491794']
CuFSe 5 ['F0.555556Cu0.333333Se0.111111', '-1.658', '-0.200254389653']
CuFSi 2 ['F0.8Si0.1Cu0.1', '-2.404', '-0.212391038525']
CuFSm 1 ['F0.8Cu0.1Sm0.1', '-2.502', '-0.214110443687']
CuFSn 5 ['F0.666667Cu0.166667Sn0.166667', '-2.454', '-0.21707819444']
CuFSr 7 ['F0.6Cu0.2Sr0.2', '-3.254', '-0.211304444021']
CuFTc 2 ['F0.777778Cu0.111111Tc0.111111', '-2.149', '-0.3307108985']
CuFTe 9 ['F0.6Cu0.2Te0.2', '-2.004', '-0.223179149856']
CuFTh 3 ['F0.8Cu0.1Th0.1', '-2.969', '-0.21971042119']
CuFTi 5 ['F0.6Ti0.1Cu0.3', '-2.473', '-0.250995090396']
CuFTl 4 ['F0.5Cu0.125Tl0.375', '-1.995', '-0.211585801056']
CuFTm 37 ['F0.4Cu0.1Tm0.5', '-2.084', '-0.223350371146']
CuFV 2 ['F0.777778V0.111111Cu0.111111', '-2.57', '-0.327941410583']
CuFXe 2 ['F0.7Cu0.2Xe0.1', '-1.553', '-0.218691184111']
CuFY 6 ['F0.666667Cu0.166667Y0.166667', '-3.435', '-0.203671128216']
CuFYb 37 ['F0.333333Cu0.166667Yb0.5', '-2.18', '-0.254306737792']
CuFZn 3 ['F0.75Cu0.125Zn0.125', '-1.824', '-0.212453564813']
CuFZr 1 ['F0.8Cu0.1Zr0.1', '-2.706', '-0.2064162419']
CuGaLu 2 ['Cu0.3Ga0.1Lu0.6', '-0.516', '-0.2036913']
CuGaPm 1 ['Cu0.1Ga0.7Pm0.2', '-0.49', '-0.2394378135']
CuGaTe 1 ['Cu0.333333Ga0.111111Te0.555556', '-0.377', '-0.221605545741']
CuGdI 22 ['Cu0.333333I0.5Gd0.166667', '-0.754', '-0.207172669201']
CuGdN 1 ['N0.4Cu0.1Gd0.5', '-1.659', '-0.240616396114']
CuGdO 2 ['O0.6Cu0.1Gd0.3', '-3.304', '-0.254453531199']
CuGdP 1 ['P0.444444Cu0.333333Gd0.222222', '-1.089', '-0.225340634073']
CuGdSi 1 ['Si0.375Cu0.125Gd0.5', '-0.888', '-0.207336972344']
CuGeHf 1 ['Cu0.285714Ge0.428571Hf0.285714', '-0.635', '-0.207227095591']
CuGeNd 1 ['Cu0.25Ge0.375Nd0.375', '-0.87', '-0.209422532014']
CuGeO 7 ['O0.444444Cu0.444444Ge0.111111', '-1.292', '-0.206097658304']
CuGePa 2 ['Cu0.1Ge0.6Pa0.3', '-0.402', '-0.2091913515']
CuGePr 2 ['Cu0.25Ge0.375Pr0.375', '-0.857', '-0.21993643558']
CuGeTe 2 ['Cu0.333333Ge0.111111Te0.555556', '-0.298', '-0.217276375741']
CuGeZr 4 ['Cu0.125Ge0.375Zr0.5', '-0.929', '-0.215965002969']
CuHHf 1 ['H0.428571Cu0.142857Hf0.428571', '-0.566', '-0.201704606564']
CuHPm 15 ['H0.428571Cu0.285714Pm0.285714', '-0.463', '-0.203558471207']
CuHPu 1 ['H0.4Cu0.2Pu0.4', '-0.436', '-0.232058455127']
CuHfMo 1 ['Cu0.1Mo0.3Hf0.6', '-0.328', '-0.20316905625']
CuHfN 5 ['N0.375Cu0.375Hf0.25', '-1.223', '-0.221274480304']
CuHfS 2 ['S0.375Cu0.125Hf0.5', '-1.404', '-0.256800202872']
CuHoI 18 ['Cu0.333333I0.555556Ho0.111111', '-0.633', '-0.21257976036']
CuHoN 11 ['N0.444444Cu0.111111Ho0.444444', '-1.844', '-0.200921125031']
CuHoO 2 ['O0.6Cu0.1Ho0.3', '-3.315', '-0.208536882544']

CuHoOs 4 ['Cu0.111111Ho0.555556Os0.333333', '-0.32', '-0.219442116296']
CuILu 30 ['Cu0.375I0.5Lu0.125', '-0.534', '-0.210593154411']
CuINd 9 ['Cu0.111111I0.777778Nd0.111111', '-0.744', '-0.215870004189']
CuINp 4 ['Cu0.125I0.75Np0.125', '-0.774', '-0.208567688941']
CuIPa 24 ['Cu0.3I0.5Pa0.2', '-0.405', '-0.201920283786']
CuIPm 26 ['Cu0.1I0.5Pm0.4', '-0.965', '-0.200098458786']
CuIPr 2 ['Cu0.166667I0.666667Pr0.166667', '-1.204', '-0.227529626918']
CuISc 1 ['Sc0.2Cu0.1I0.7', '-1.017', '-0.229074260783']
CuISi 2 ['Si0.2Cu0.2I0.6', '-0.604', '-0.20355585198']
CuISm 7 ['Cu0.25I0.625Sm0.125', '-0.818', '-0.217666365045']
CuITb 30 ['Cu0.375I0.5Tb0.125', '-0.492', '-0.201614089453']
CuITm 5 ['Cu0.222222I0.666667Tm0.111111', '-0.733', '-0.206323637808']
CuIXe 4 ['Cu0.142857I0.428571Xe0.428571', '-0.271', '-0.200710366796']
CuInN 1 ['N0.4Cu0.3In0.3', '-0.224', '-0.210741282319']
CuKN 4 ['N0.333333K0.333333Cu0.333333', '-0.339', '-0.207629535441']
CuKO 1 ['O0.4K0.5Cu0.1', '-1.446', '-0.233331542478']
CuKP 1 ['P0.444444K0.222222Cu0.333333', '-0.587', '-0.210503641232']
CuKTe 1 ['K0.1Cu0.4Te0.5', '-0.44', '-0.202019811167']
CuLaN 14 ['N0.333333Cu0.444444La0.222222', '-0.837', '-0.203024689924']
CuLaO 2 ['O0.6Cu0.2La0.2', '-2.532', '-0.26823938224']
CuLaP 1 ['P0.4Cu0.3La0.3', '-1.33', '-0.231832961845']
CuLuN 19 ['N0.4Cu0.3Lu0.3', '-1.398', '-0.207867254898']
CuLuO 1 ['O0.6Cu0.2Lu0.2', '-2.639', '-0.31258511822']
CuLuOs 4 ['Cu0.142857Lu0.571429Os0.285714', '-0.458', '-0.201479735']
CuMgO 1 ['O0.6Mg0.2Cu0.2', '-1.726', '-0.207761890305']
CuMnN 2 ['N0.375Mn0.375Cu0.25', '-0.43', '-0.202572985324']
CuMoN 22 ['N0.3Cu0.2Mo0.5', '-0.487', '-0.204488661147']
CuMoO 1 ['O0.6Cu0.2Mo0.2', '-1.959', '-0.225358928423']
CuNNb 7 ['N0.444444Cu0.222222Nb0.333333', '-1.037', '-0.208778868526']
CuNNp 18 ['N0.444444Cu0.222222Np0.333333', '-1.025', '-0.216695191967']
CuNO 2 ['N0.111111O0.222222Cu0.666667', '-0.671', '-0.214717087944']
CuNPa 34 ['N0.4Cu0.5Pa0.1', '-0.431', '-0.23341116181']
CuNPm 1 ['N0.375Cu0.375Pm0.25', '-0.983', '-0.21222995554']
CuNPu 15 ['N0.5Cu0.3Pu0.2', '-0.757', '-0.209351192932']
CuNRe 2 ['N0.444444Cu0.333333Re0.222222', '-0.227', '-0.201172468863']
CuNSn 4 ['N0.375Cu0.375Sn0.25', '-0.254', '-0.217664615787']
CuNTb 2 ['N0.4Cu0.3Tb0.3', '-1.103', '-0.241240333994']
CuNTh 8 ['N0.5Cu0.125Th0.375', '-1.737', '-0.222675836078']
CuNTi 15 ['N0.375Ti0.25Cu0.375', '-0.881', '-0.232481588996']
CuNTm 1 ['N0.4Cu0.1Tm0.5', '-1.911', '-0.309342548864']
CuNU 5 ['N0.444444Cu0.333333U0.222222', '-1.199', '-0.20220564318']
CuNW 12 ['N0.6Cu0.1W0.3', '-0.322', '-0.212280476862']
CuNY 9 ['N0.375Cu0.25Y0.375', '-1.551', '-0.204582941123']
CuNYb 20 ['N0.285714Cu0.571429Yb0.142857', '-0.453', '-0.20362219949']

CuNZn 4 ['N0.375Cu0.125Zn0.5', '-0.242', '-0.2072607475']
CuNZr 3 ['N0.428571Cu0.285714Zr0.285714', '-1.28', '-0.216133007727']
CuNaP 16 ['Na0.2P0.4Cu0.4', '-0.535', '-0.211244535371']
CuNbO 2 ['O0.6Cu0.2Nb0.2', '-2.41', '-0.219818293134']
CuNdO 2 ['O0.6Cu0.2Nd0.2', '-2.548', '-0.289002889134']
CuNdP 2 ['P0.375Cu0.375Nd0.25', '-1.156', '-0.227312569244']
CuNpS 1 ['S0.428571Cu0.142857Np0.428571', '-1.555', '-0.261996197389']
CuOP 13 ['O0.3P0.1Cu0.6', '-1.14', '-0.215671722477']
CuOPa 3 ['O0.625Cu0.25Pa0.125', '-1.964', '-0.246929464142']
CuOPr 3 ['O0.5Cu0.333333Pr0.166667', '-2.144', '-0.206329403584']
CuORe 2 ['O0.6Cu0.2Re0.2', '-1.728', '-0.207384289472']
CuORh 2 ['O0.333333Cu0.555556Rh0.111111', '-0.906', '-0.245185417289']
CuOSn 2 ['O0.375Cu0.5Sn0.125', '-1.195', '-0.205783934641']
CuOTc 3 ['O0.625Cu0.125Tc0.25', '-1.701', '-0.219256828716']
CuOTe 6 ['O0.125Cu0.5Te0.375', '-0.499', '-0.203374618575']
CuOTi 3 ['O0.285714Ti0.571429Cu0.142857', '-1.92', '-0.21739489044']
CuOU 1 ['O0.6Cu0.3U0.1', '-1.982', '-0.238611559382']
CuOY 1 ['O0.5Cu0.4Y0.1', '-1.766', '-0.205753623886']
CuOZn 2 ['O0.4Cu0.4Zn0.2', '-1.305', '-0.234783663974']
CuOsSc 1 ['Sc0.6Cu0.1Os0.3', '-0.495', '-0.203332471']
CuOsTm 4 ['Cu0.222222Tm0.555556Os0.222222', '-0.416', '-0.226950213333']
CuPPm 17 ['P0.285714Cu0.285714Pm0.428571', '-0.939', '-0.210990223511']
CuPPr 11 ['P0.333333Cu0.333333Pr0.333333', '-1.319', '-0.222290047847']
CuPPu 1 ['P0.777778Cu0.111111Pu0.111111', '-0.643', '-0.20783340302']
CuPTa 1 ['P0.444444Cu0.111111Ta0.444444', '-0.869', '-0.226773518518']
CuPTb 3 ['P0.444444Cu0.333333Tb0.222222', '-0.923', '-0.202862043593']
CuPTe 1 ['P0.142857Cu0.428571Te0.428571', '-0.322', '-0.21667185378']
CuPTm 3 ['P0.4Cu0.3Tm0.3', '-1.306', '-0.225552500291']
CuPYb 35 ['P0.555556Cu0.222222Yb0.222222', '-0.789', '-0.200390203495']
CuPaS 5 ['S0.7Cu0.1Pa0.2', '-1.123', '-0.217418334492']
CuPaSe 23 ['Cu0.222222Se0.666667Pa0.111111', '-0.531', '-0.200267061111']
CuPdPm 4 ['Cu0.125Pd0.75Pm0.125', '-0.445', '-0.213292989687']
CuPmS 6 ['S0.333333Cu0.166667Pm0.5', '-1.502', '-0.200267989077']
CuPmSe 27 ['Cu0.333333Se0.555556Pm0.111111', '-0.668', '-0.215067240833']
CuPtTh 5 ['Cu0.142857Pt0.714286Th0.142857', '-0.638', '-0.216928388214']
CuPuS 1 ['S0.5Cu0.125Pu0.375', '-1.842', '-0.215727902366']
CuRbS 1 ['S0.375Cu0.25Rb0.375', '-1.029', '-0.204109423496']
CuSSe 15 ['S0.333333Cu0.222222Se0.444444', '-0.378', '-0.203827600699']
CuSZr 5 ['S0.5Cu0.166667Zr0.333333', '-1.562', '-0.203843860909']
CuSeTc 5 ['Cu0.222222Se0.555556Tc0.222222', '-0.265', '-0.201828251111']
CuSeTm 6 ['Cu0.166667Se0.5Tm0.333333', '-1.516', '-0.207640370002']
CuSeY 11 ['Cu0.333333Se0.444444Y0.222222', '-1.08', '-0.215204877222']
CuSeZr 2 ['Cu0.1Se0.5Zr0.4', '-1.307', '-0.222492148728']
CuSiTe 3 ['Si0.125Cu0.25Te0.625', '-0.24', '-0.208460946667']

CuSiY 2 ['Si0.333333Cu0.166667Y0.5', '-0.874', '-0.200339620952']
CuSnSr 1 ['Cu0.166667Sr0.166667Sn0.666667', '-0.554', '-0.217468702377']
CuSnTe 1 ['Cu0.333333Sn0.111111Te0.555556', '-0.466', '-0.349289577757']
CuTeZr 2 ['Cu0.375Zr0.125Te0.5', '-0.6', '-0.201399021459']
DyErF 2 ['F0.777778Dy0.111111Er0.111111', '-4.085', '-0.276170806787']
DyErI 10 ['I0.625Dy0.125Er0.25', '-1.096', '-0.22094742733']
DyEuF 3 ['F0.75Eu0.125Dy0.125', '-4.083', '-0.306538684711']
DyEuI 8 ['I0.777778Eu0.111111Dy0.111111', '-1.126', '-0.238412748313']
DyEuO 5 ['O0.666667Eu0.166667Dy0.166667', '-2.944', '-0.207657208628']
DyFFe 3 ['F0.75Fe0.125Dy0.125', '-3.474', '-0.21359824482']
DyFGa 4 ['F0.714286Ga0.142857Dy0.142857', '-3.781', '-0.327881915861']
DyFGd 2 ['F0.777778Gd0.111111Dy0.111111', '-3.995', '-0.313460466787']
DyFGe 9 ['F0.571429Ge0.142857Dy0.285714', '-3.528', '-0.205084485104']
DyFH 25 ['H0.3F0.3Dy0.4', '-2.187', '-0.224479841441']
DyFHf 1 ['F0.8Dy0.1Hf0.1', '-4.207', '-0.507821113446']
DyFHg 3 ['F0.75Dy0.125Hg0.125', '-2.926', '-0.241194206591']
DyFHo 2 ['F0.777778Dy0.111111Ho0.111111', '-4.121', '-0.311906687249']
DyFI 36 ['F0.375I0.25Dy0.375', '-2.669', '-0.20772068236']
DyFIn 2 ['F0.8In0.1Dy0.1', '-3.221', '-0.458726244356']
DyFIr 1 ['F0.777778Dy0.111111Ir0.111111', '-2.965', '-0.222610941051']
DyFK 13 ['F0.6K0.3Dy0.1', '-3.624', '-0.224932603354']
DyFKr 2 ['F0.777778Kr0.111111Dy0.111111', '-2.204', '-0.265316279189']
DyFLa 2 ['F0.777778La0.111111Dy0.111111', '-4.322', '-0.496500977249']
DyFLi 9 ['Li0.142857F0.571429Dy0.285714', '-3.465', '-0.206098503831']
DyFLu 3 ['F0.75Dy0.125Lu0.125', '-4.293', '-0.205813174195']
DyFMg 5 ['F0.7Mg0.1Dy0.2', '-4.172', '-0.272955063583']
DyFMn 2 ['F0.777778Mn0.111111Dy0.111111', '-3.473', '-0.251662913546']
DyFMo 3 ['F0.75Mo0.125Dy0.125', '-3.546', '-0.25470209232']
DyFN 2 ['N0.111111F0.777778Dy0.111111', '-2.107', '-0.272271449551']
DyFNa 8 ['F0.625Na0.125Dy0.25', '-3.704', '-0.208408126829']
DyFNb 3 ['F0.777778Nb0.111111Dy0.111111', '-3.819', '-0.22093123475']
DyFNd 2 ['F0.777778Nd0.111111Dy0.111111', '-4.076', '-0.28927948132']
DyFNi 3 ['F0.75Ni0.125Dy0.125', '-3.373', '-0.401240864195']
DyFNp 1 ['F0.8Dy0.1Np0.1', '-4.168', '-0.630475452901']
DyFO 11 ['O0.25F0.5Dy0.25', '-3.81', '-0.255816954964']
DyFOs 2 ['F0.8Dy0.1Os0.1', '-2.999', '-0.207612638986']
DyFP 7 ['F0.666667P0.166667Dy0.166667', '-3.557', '-0.234323317839']
DyFPa 3 ['F0.75Dy0.125Pa0.125', '-4.171', '-0.30824266732']
DyFPb 3 ['F0.777778Dy0.111111Pb0.111111', '-3.385', '-0.368640446607']
DyFPd 1 ['F0.8Pd0.1Dy0.1', '-2.613', '-0.251639578946']
DyFPm 2 ['F0.777778Pm0.111111Dy0.111111', '-4.012', '-0.324266242898']
DyFPr 2 ['F0.777778Pr0.111111Dy0.111111', '-4.142', '-0.358976219564']
DyFPu 7 ['F0.625Dy0.125Pu0.25', '-3.583', '-0.217958459712']
DyFRb 12 ['F0.7Rb0.1Dy0.2', '-4.106', '-0.232012278278']

DyFRe 2 ['F0.8Dy0.1Re0.1', '-3.243', '-0.302143579316']
DyFRh 3 ['F0.75Rh0.125Dy0.125', '-3.201', '-0.23974148107']
DyFRu 3 ['F0.777778Ru0.111111Dy0.111111', '-3.144', '-0.249892251051']
DyFS 8 ['F0.285714S0.428571Dy0.285714', '-2.87', '-0.226692400296']
DyFSb 4 ['F0.5Sb0.2Dy0.3', '-3.262', '-0.207194094883']
DyFSc 2 ['F0.777778Sc0.111111Dy0.111111', '-4.068', '-0.335567622618']
DyFSe 11 ['F0.444444Se0.222222Dy0.333333', '-3.392', '-0.210719442982']
DyFSi 7 ['F0.4Si0.2Dy0.4', '-2.755', '-0.2043908896']
DyFSm 2 ['F0.777778Sm0.111111Dy0.111111', '-4.018', '-0.217953011787']
DyFSn 1 ['F0.8Sn0.1Dy0.1', '-3.319', '-0.337998335256']
DyFSr 3 ['F0.777778Sr0.111111Dy0.111111', '-3.534', '-0.297275564188']
DyFTa 3 ['F0.75Dy0.125Ta0.125', '-3.919', '-0.29372017057']
DyFTc 3 ['F0.75Tc0.125Dy0.125', '-3.387', '-0.275407978534']
DyFTe 4 ['F0.714286Te0.142857Dy0.142857', '-3.433', '-0.229057083005']
DyFTh 1 ['F0.8Dy0.1Th0.1', '-4.209', '-0.327557688111']
DyFTi 2 ['F0.777778Ti0.111111Dy0.111111', '-4.112', '-0.673548709422']
DyFTl 16 ['F0.6Dy0.1Tl0.3', '-3.011', '-0.206399262354']
DyFTm 6 ['F0.666667Dy0.111111Tm0.222222', '-3.52', '-0.716763263076']
DyFU 3 ['F0.777778Dy0.111111U0.111111', '-4.155', '-0.227342277097']
DyFV 3 ['F0.777778V0.111111Dy0.111111', '-3.742', '-0.241993929384']
DyFY 2 ['F0.777778Y0.111111Dy0.111111', '-4.19', '-0.48944273632']
DyFYb 11 ['F0.571429Dy0.142857Yb0.285714', '-3.437', '-0.239771221082']
DyFZn 3 ['F0.75Zn0.125Dy0.125', '-3.238', '-0.211262648464']
DyFZr 1 ['F0.8Zr0.1Dy0.1', '-4.111', '-0.479263508821']
DyFeI 26 ['Fe0.125I0.5Dy0.375', '-1.007', '-0.200773064341']
DyFeN 1 ['N0.4Fe0.1Dy0.5', '-1.692', '-0.233392970609']
DyFeO 1 ['O0.625Fe0.125Dy0.25', '-3.347', '-0.404804789056']
DyFeSi 3 ['Si0.5Fe0.375Dy0.125', '-0.917', '-0.288055275104']
DyGaI 21 ['Ga0.285714I0.571429Dy0.142857', '-0.891', '-0.202399649565']
DyGaIr 4 ['Ga0.5Dy0.125Ir0.375', '-0.77', '-0.208524567361']
DyGaNa 1 ['Na0.166667Ga0.666667Dy0.166667', '-0.607', '-0.217247912151']
DyGaO 2 ['O0.625Ga0.25Dy0.125', '-2.76', '-0.235405457836']
DyGaPd 3 ['Ga0.444444Pd0.444444Dy0.111111', '-0.941', '-0.201783209537']
DyGaPt 1 ['Ga0.555556Dy0.111111Pt0.333333', '-1.046', '-0.339209923912']
DyGaRh 2 ['Ga0.444444Rh0.444444Dy0.111111', '-0.915', '-0.202933600741']
DyGaS 1 ['S0.4Ga0.2Dy0.4', '-1.953', '-0.200124765227']
DyGdI 9 ['I0.666667Gd0.111111Dy0.222222', '-1.237', '-0.424840087026']
DyGdO 1 ['O0.625Gd0.25Dy0.125', '-3.806', '-0.232472385625']
DyGeH 15 ['H0.5Ge0.125Dy0.375', '-0.942', '-0.205235387658']
DyGeI 25 ['Ge0.285714I0.428571Dy0.285714', '-0.948', '-0.200721249569']
DyGeIr 2 ['Ge0.5Dy0.125Ir0.375', '-0.817', '-0.223076449449']
DyGeOs 2 ['Ge0.5Dy0.1Os0.4', '-0.433', '-0.202966955333']
DyGePa 2 ['Ge0.7Dy0.1Pa0.2', '-0.545', '-0.228456597167']
DyGePt 7 ['Ge0.5Dy0.166667Pt0.333333', '-0.967', '-0.220162894722']

DyGeRh 1 ['Ge0.571429Rh0.285714Dy0.142857', '-0.894', '-0.251219068869']
DyGeS 5 ['S0.428571Ge0.142857Dy0.428571', '-2.074', '-0.204814174708']
DyHI 53 ['H0.666667I0.166667Dy0.166667', '-0.629', '-0.203197747584']
DyHIr 4 ['H0.6Dy0.2Ir0.2', '-0.688', '-0.201191276845']
DyHMn 3 ['H0.6Mn0.2Dy0.2', '-0.711', '-0.226690145023']
DyHN 19 ['H0.5N0.2Dy0.3', '-1.177', '-0.201370040949']
DyHO 15 ['H0.6O0.2Dy0.2', '-1.65', '-0.205219030694']
DyHOs 1 ['H0.666667Dy0.222222Os0.111111', '-0.765', '-0.226877938915']
DyHPm 2 ['H0.7Pm0.2Dy0.1', '-0.672', '-0.216641143054']
DyHPt 12 ['H0.625Dy0.25Pt0.125', '-0.879', '-0.204517509121']
DyHRh 21 ['H0.428571Rh0.428571Dy0.142857', '-0.546', '-0.200064389302']
DyHRu 3 ['H0.6Ru0.2Dy0.2', '-0.695', '-0.210690145023']
DyHS 20 ['H0.1S0.4Dy0.5', '-2.015', '-0.200823810676']
DyHSc 5 ['H0.555556Sc0.333333Dy0.111111', '-0.769', '-0.202805506101']
DyHSi 28 ['H0.2Si0.3Dy0.5', '-0.946', '-0.215088083105']
DyHfI 5 ['I0.7Dy0.2Hf0.1', '-1.22', '-0.219682959718']
DyHfS 9 ['S0.555556Dy0.222222Hf0.222222', '-2.116', '-0.221701885733']
DyHgI 28 ['I0.428571Dy0.285714Hg0.285714', '-0.885', '-0.204069088483']
DyHoI 10 ['I0.625Dy0.125Ho0.25', '-1.082', '-0.207167761343']
DyIIn 35 ['In0.333333I0.333333Dy0.333333', '-0.937', '-0.22193530919']
DyIIr 34 ['I0.4Dy0.4Ir0.2', '-1.13', '-0.222125247029']
DyIK 7 ['K0.111111I0.777778Dy0.111111', '-0.981', '-0.220890707448']
DyIKr 18 ['Kr0.333333I0.555556Dy0.111111', '-0.477', '-0.21219353441']
DyILa 5 ['I0.7La0.1Dy0.2', '-1.401', '-0.25552190721']
DyILi 18 ['Li0.222222I0.555556Dy0.222222', '-1.355', '-0.237859440504']
DyILu 15 ['I0.571429Dy0.142857Lu0.285714', '-0.878', '-0.220056696405']
DyIMg 9 ['Mg0.1I0.8Dy0.1', '-0.833', '-0.242329311566']
DyIMn 11 ['Mn0.1I0.8Dy0.1', '-0.653', '-0.245466924104']
DyIMo 6 ['Mo0.111111I0.666667Dy0.222222', '-0.976', '-0.279674918188']
DyIN 33 ['N0.4I0.1Dy0.5', '-1.863', '-0.257265319838']
DyINa 12 ['Na0.3I0.6Dy0.1', '-1.299', '-0.211004906985']
DyINb 6 ['Nb0.111111I0.666667Dy0.222222', '-1.016', '-0.226999042455']
DyINd 8 ['I0.666667Nd0.166667Dy0.166667', '-1.312', '-0.20459987997']
DyINi 40 ['Ni0.25I0.375Dy0.375', '-0.978', '-0.212454555339']
DyINp 5 ['I0.714286Dy0.142857Np0.142857', '-1.173', '-0.25661582195']
DyIO 54 ['O0.285714I0.142857Dy0.571429', '-2.267', '-0.206673956666']
DyIOs 27 ['I0.571429Dy0.142857Os0.285714', '-0.558', '-0.217534544241']
DyIP 38 ['P0.222222I0.666667Dy0.111111', '-0.701', '-0.205344193126']
DyIPa 11 ['I0.6Dy0.1Pa0.3', '-0.574', '-0.252466951241']
DyIPb 35 ['I0.8Dy0.1Pb0.1', '-0.678', '-0.205414706733']
DyIPd 47 ['Pd0.5I0.25Dy0.25', '-1.031', '-0.203447110601']
DyIPm 10 ['I0.625Pm0.25Dy0.125', '-0.955', '-0.209780977479']
DyIPr 5 ['I0.714286Pr0.142857Dy0.142857', '-1.356', '-0.248849571946']
DyIPt 51 ['I0.25Dy0.5Pt0.25', '-1.279', '-0.224582805538']

DyIPu 9 ['I0.625Dy0.25Pu0.125', '-1.207', '-0.205823693205']
DyIRb 12 ['Rb0.3I0.6Dy0.1', '-1.487', '-0.252505884753']
DyIRe 14 ['I0.625Dy0.125Re0.25', '-0.622', '-0.214123814767']
DyIRh 49 ['Rh0.5I0.333333Dy0.166667', '-0.603', '-0.205790301615']
DyIRu 29 ['Ru0.2I0.4Dy0.4', '-0.926', '-0.206694492362']
DyIS 18 ['S0.285714I0.285714Dy0.428571', '-1.899', '-0.207124356257']
DyISb 44 ['Sb0.4I0.3Dy0.3', '-1.077', '-0.201513568274']
DyISc 9 ['Sc0.222222I0.666667Dy0.111111', '-1.302', '-0.21639020555']
DyISe 47 ['Se0.333333I0.222222Dy0.444444', '-1.678', '-0.201212474276']
DyISi 26 ['Si0.3I0.5Dy0.2', '-0.885', '-0.228155814149']
DyISm 6 ['I0.7Sm0.2Dy0.1', '-1.306', '-0.309784281758']
DyISn 21 ['Sn0.3I0.6Dy0.1', '-0.895', '-0.20005503823']
DyISr 5 ['Sr0.2I0.7Dy0.1', '-1.556', '-0.222101961497']
DyITa 5 ['I0.714286Dy0.142857Ta0.142857', '-0.992', '-0.283100875215']
DyITb 12 ['I0.625Tb0.125Dy0.25', '-0.934', '-0.232309829036']
DyITc 17 ['Tc0.285714I0.571429Dy0.142857', '-0.596', '-0.255534544241']
DyITe 56 ['Te0.1I0.5Dy0.4', '-1.256', '-0.226179109368']
DyITh 3 ['I0.75Dy0.125Th0.125', '-1.452', '-0.295832226561']
DyITi 8 ['Ti0.222222I0.666667Dy0.111111', '-1.092', '-0.210445383398']
DyITl 32 ['I0.5Dy0.4Tl0.1', '-1.056', '-0.208403731419']
DyITm 7 ['I0.666667Dy0.222222Tm0.111111', '-1.18', '-0.233050136057']
DyIU 3 ['I0.75Dy0.125U0.125', '-1.157', '-0.253427905622']
DyIV 8 ['V0.2I0.7Dy0.1', '-0.877', '-0.297834779997']
DyIW 7 ['I0.666667Dy0.166667W0.166667', '-0.63', '-0.232790301615']
DyIXe 28 ['I0.375Xe0.5Dy0.125', '-0.508', '-0.210092726211']
DyIY 5 ['Y0.1I0.7Dy0.2', '-1.321', '-0.21887982621']
DyIYb 7 ['I0.666667Dy0.222222Yb0.111111', '-1.331', '-0.20860648828']
DyIZn 25 ['Zn0.285714I0.428571Dy0.285714', '-0.929', '-0.21143626439']
DyIZr 6 ['Zr0.111111I0.666667Dy0.222222', '-1.217', '-0.203341237176']
DyInN 22 ['N0.285714In0.142857Dy0.571429', '-1.465', '-0.200417974661']
DyInP 1 ['P0.444444In0.333333Dy0.222222', '-1.171', '-0.241247585649']
DyInS 6 ['S0.333333In0.111111Dy0.555556', '-1.747', '-0.201755610514']
DyIrN 4 ['N0.4Dy0.5Ir0.1', '-1.82', '-0.209294141525']
DyIrP 1 ['P0.444444Dy0.222222Ir0.333333', '-1.303', '-0.208583356426']
DyIrS 4 ['S0.375Dy0.375Ir0.25', '-1.914', '-0.202911150061']
DyIrSe 10 ['Se0.428571Dy0.285714Ir0.285714', '-1.543', '-0.20750514095']
DyIrSi 5 ['Si0.444444Dy0.111111Ir0.444444', '-1.112', '-0.20299091315']
DyKO 2 ['O0.6K0.2Dy0.2', '-2.704', '-0.201388999765']
DyKTe 1 ['K0.142857Te0.571429Dy0.285714', '-1.467', '-0.216682857736']
DyLaO 3 ['O0.625La0.25Dy0.125', '-3.87', '-0.346528334531']
DyLiN 2 ['Li0.125N0.375Dy0.5', '-1.643', '-0.286577374243']
DyLiS 1 ['Li0.142857S0.428571Dy0.428571', '-2.055', '-0.218529536614']
DyLiSe 7 ['Li0.285714Se0.428571Dy0.285714', '-1.752', '-0.207207315241']
DyMgN 4 ['N0.4Mg0.2Dy0.4', '-1.655', '-0.208149199192']

DyMgO 1 ['O0.6Mg0.2Dy0.2', '-3.337', '-0.224838032435']
DyMgS 21 ['Mg0.1S0.5Dy0.4', '-2.23', '-0.20365024341']
DyMgSe 7 ['Mg0.25Se0.5Dy0.25', '-1.719', '-0.210444249795']
DyMnN 2 ['N0.428571Mn0.428571Dy0.142857', '-0.957', '-0.200833164059']
DyMnO 2 ['O0.6Mn0.2Dy0.2', '-3.2', '-0.2155396387']
DyMnSi 3 ['Si0.5Mn0.4Dy0.1', '-0.805', '-0.259899592691']
DyMoN 1 ['N0.4Mo0.1Dy0.5', '-1.651', '-0.204149199192']
DyNNa 1 ['N0.375Na0.125Dy0.5', '-1.559', '-0.202577374242']
DyNNb 3 ['N0.428571Nb0.142857Dy0.428571', '-1.8', '-0.24980271342']
DyNNi 1 ['N0.375Ni0.125Dy0.5', '-1.734', '-0.262203191534']
DyNNp 18 ['N0.5Dy0.4Np0.1', '-1.895', '-0.205657756782']
DyNO 8 ['N0.285714O0.285714Dy0.428571', '-2.897', '-0.202430212405']
DyNOs 12 ['N0.222222Dy0.555556Os0.222222', '-1.134', '-0.214737600662']
DyNP 28 ['N0.285714P0.285714Dy0.428571', '-1.852', '-0.258420458859']
DyNPa 16 ['N0.555556Dy0.333333Pa0.111111', '-1.669', '-0.243747845782']
DyNPt 5 ['N0.4Dy0.5Pt0.1', '-1.907', '-0.202643825776']
DyNPu 4 ['N0.555556Dy0.111111Pu0.333333', '-1.592', '-0.277348987996']
DyNRe 1 ['N0.4Dy0.5Re0.1', '-1.653', '-0.206149199192']
DyNRh 2 ['N0.375Rh0.125Dy0.5', '-1.799', '-0.224123368409']
DyNRu 1 ['N0.375Ru0.125Dy0.5', '-1.714', '-0.279043008409']
DyNSc 1 ['N0.444444Sc0.111111Dy0.444444', '-1.928', '-0.273270035956']
DyNTa 1 ['N0.444444Dy0.444444Ta0.111111', '-1.86', '-0.252387999102']
DyNTc 13 ['N0.5Tc0.375Dy0.125', '-0.767', '-0.216732761246']
DyNTe 3 ['N0.4Te0.1Dy0.5', '-1.925', '-0.222589317192']
DyNTi 12 ['N0.555556Ti0.222222Dy0.222222', '-1.595', '-0.21473318977']
DyNU 4 ['N0.5Dy0.166667U0.333333', '-1.755', '-0.20825339955']
DyNV 2 ['N0.555556V0.222222Dy0.222222', '-1.591', '-0.230346945031']
DyNW 7 ['N0.555556Dy0.222222W0.222222', '-1.089', '-0.203920278708']
DyNZn 6 ['N0.4Zn0.5Dy0.1', '-0.574', '-0.212287299798']
DyNaS 3 ['Na0.125S0.375Dy0.5', '-1.826', '-0.222479967245']
DyNaSe 1 ['Na0.222222Se0.444444Dy0.333333', '-1.759', '-0.210456508703']
DyNaTe 1 ['Na0.1Te0.6Dy0.3', '-1.428', '-0.207831810455']
DyNbSe 2 ['Se0.666667Nb0.222222Dy0.111111', '-1.294', '-0.202463207082']
DyNdO 1 ['O0.625Nd0.25Dy0.125', '-3.723', '-0.217540938853']
DyNiSe 2 ['Ni0.428571Se0.285714Dy0.285714', '-1.306', '-0.230808180475']
DyNiSi 1 ['Si0.125Ni0.625Dy0.25', '-0.778', '-0.204187441657']
DyNpPt 1 ['Dy0.1Pt0.8Np0.1', '-0.713', '-0.214088663957']
DyOP 2 ['O0.625P0.125Dy0.25', '-3.745', '-0.276358552471']
DyOPa 5 ['O0.714286Dy0.142857Pa0.142857', '-2.936', '-0.232930975841']
DyOPt 1 ['O0.625Dy0.25Pt0.125', '-2.903', '-0.218903909193']
DyOS 12 ['O0.5S0.25Dy0.25', '-2.906', '-0.214101809386']
DyOSb 1 ['O0.125Sb0.25Dy0.625', '-1.742', '-0.32141425536']
DyOSe 15 ['O0.5Se0.125Dy0.375', '-3.606', '-0.201839575605']
DyOTc 1 ['O0.666667Tc0.166667Dy0.166667', '-2.846', '-0.287514380629']

DyOTe 7 ['O0.1Te0.3Dy0.6', '-1.625', '-0.20018885508']
DyOV 2 ['O0.625V0.125Dy0.25', '-3.611', '-0.209607835795']
DyOW 3 ['O0.7Dy0.1W0.2', '-3.032', '-0.216373008971']
DyOZr 1 ['O0.625Zr0.25Dy0.125', '-3.806', '-0.212900143007']
DyOsS 4 ['S0.333333Dy0.444444Os0.222222', '-1.699', '-0.235163393477']
DyOsSi 1 ['Si0.444444Dy0.111111Os0.444444', '-0.772', '-0.202224551019']
DyPPd 5 ['P0.4Pd0.3Dy0.3', '-1.463', '-0.200387610668']
DyPPm 1 ['P0.555556Pm0.333333Dy0.111111', '-1.41', '-0.201398739004']
DyPRh 5 ['P0.375Rh0.375Dy0.25', '-1.434', '-0.205298346693']
DyPTa 3 ['P0.5Dy0.125Ta0.375', '-1.178', '-0.234071857031']
DyPaS 4 ['S0.666667Dy0.222222Pa0.111111', '-1.938', '-0.234682841514']
DyPaSe 8 ['Se0.6Dy0.2Pa0.2', '-1.664', '-0.242552032664']
DyPdPm 1 ['Pd0.7Pm0.2Dy0.1', '-0.884', '-0.214910910833']
DyPdS 1 ['S0.5Pd0.166667Dy0.333333', '-2.112', '-0.238304526192']
DyPdSb 1 ['Pd0.571429Sb0.285714Dy0.142857', '-0.929', '-0.208037799851']
DyPdSe 15 ['Se0.222222Pd0.555556Dy0.222222', '-1.173', '-0.211600491017']
DyPmS 4 ['S0.428571Pm0.428571Dy0.142857', '-1.901', '-0.221364275183']
DyPmSe 8 ['Se0.666667Pm0.111111Dy0.222222', '-1.616', '-0.211517927127']
DyPrTe 1 ['Te0.7Pr0.1Dy0.2', '-1.435', '-0.319061964751']
DyPtS 18 ['S0.333333Dy0.444444Pt0.222222', '-2.046', '-0.217919935938']
DyPtSe 21 ['Se0.444444Dy0.222222Pt0.333333', '-1.34', '-0.206418998588']
DyPtSi 2 ['Si0.5Dy0.1Pt0.4', '-1.015', '-0.200745742835']
DyPtTh 3 ['Dy0.1Pt0.7Th0.2', '-1.11', '-0.213484816332']
DyPuS 10 ['S0.5Dy0.3Pu0.2', '-2.259', '-0.208673034992']
DyRbSe 7 ['Se0.444444Rb0.333333Dy0.222222', '-1.646', '-0.205007379628']
DyReS 1 ['S0.555556Dy0.222222Re0.222222', '-1.702', '-0.203279347819']
DyReSi 2 ['Si0.375Dy0.125Re0.5', '-0.576', '-0.207660322515']
DyRhS 30 ['S0.333333Rh0.166667Dy0.5', '-1.92', '-0.203376851996']
DyRhSb 1 ['Rh0.375Sb0.375Dy0.25', '-0.997', '-0.218866297605']
DyRhSe 22 ['Se0.5Rh0.166667Dy0.333333', '-1.762', '-0.203922664442']
DyRhSi 8 ['Si0.6Rh0.3Dy0.1', '-0.948', '-0.207689337458']
DyRhSn 3 ['Rh0.4Sn0.5Dy0.1', '-0.842', '-0.201992293282']
DyRuS 11 ['S0.5Ru0.3Dy0.2', '-1.551', '-0.205296231912']
DyRuSe 8 ['Se0.428571Ru0.142857Dy0.428571', '-1.703', '-0.20358811286']
DyRuSi 1 ['Si0.555556Ru0.333333Dy0.111111', '-0.911', '-0.203437826759']
DySSb 2 ['S0.428571Sb0.142857Dy0.428571', '-2.131', '-0.20067123507']
DySSi 6 ['Si0.222222S0.444444Dy0.333333', '-1.94', '-0.200980723811']
DySSn 3 ['S0.428571Sn0.142857Dy0.428571', '-2.065', '-0.200034955872']
DySTc 9 ['S0.5Tc0.25Dy0.25', '-1.828', '-0.271394191642']
DySTh 3 ['S0.428571Dy0.285714Th0.285714', '-2.013', '-0.221735914946']
DySTl 7 ['S0.444444Dy0.444444Tl0.111111', '-2.11', '-0.209531813031']
DySZr 6 ['S0.444444Zr0.333333Dy0.222222', '-1.864', '-0.206278788399']
DySbSe 13 ['Se0.5Sb0.125Dy0.375', '-1.86', '-0.207306528435']
DyScSe 6 ['Sc0.2Se0.6Dy0.2', '-1.799', '-0.221557473664']

DySeSi 1 ['Si0.25Se0.375Dy0.375', '-1.654', '-0.25583129906']
DySeTc 4 ['Se0.625Tc0.25Dy0.125', '-0.852', '-0.264601359165']
DySeTe 13 ['Se0.285714Te0.285714Dy0.428571', '-1.818', '-0.200455387856']
DySeTl 1 ['Se0.5Dy0.4Tl0.1', '-1.875', '-0.240428051333']
DySeY 9 ['Se0.555556Y0.333333Dy0.111111', '-1.929', '-0.229600389813']
DySeZn 2 ['Zn0.222222Se0.444444Dy0.333333', '-1.659', '-0.203073518821']
DySeZr 3 ['Se0.555556Zr0.333333Dy0.111111', '-1.58', '-0.207846923626']
DySiTc 1 ['Si0.5Tc0.375Dy0.125', '-0.744', '-0.211593911979']
ErEuF 2 ['F0.777778Eu0.111111Er0.111111', '-3.722', '-0.225773405206']
ErEuI 7 ['I0.777778Eu0.111111Er0.111111', '-1.108', '-0.228710192178']
ErEuN 5 ['N0.444444Eu0.444444Er0.111111', '-1.018', '-0.213214145403']
ErEuO 5 ['O0.666667Eu0.166667Er0.166667', '-2.964', '-0.208236017966']
ErEuS 1 ['S0.555556Eu0.111111Er0.333333', '-2.501', '-0.230276817768']
ErFFe 2 ['F0.777778Fe0.111111Er0.111111', '-3.469', '-0.431493014191']
ErFGa 2 ['F0.777778Ga0.111111Er0.111111', '-3.594', '-0.34335441808']
ErFGd 2 ['F0.777778Gd0.111111Er0.111111', '-4.202', '-0.381088374471']
ErFGe 2 ['F0.8Ge0.1Er0.1', '-3.312', '-0.245985860362']
ErFH 8 ['H0.5F0.3Er0.2', '-2.22', '-0.200220146857']
ErFHf 1 ['F0.8Er0.1Hf0.1', '-4.253', '-0.428386230362']
ErFHg 3 ['F0.777778Er0.111111Hg0.111111', '-2.762', '-0.236133869099']
ErFHo 1 ['F0.8Ho0.1Er0.1', '-3.883', '-0.32938113544']
ErFI 21 ['F0.3I0.5Er0.2', '-2.24', '-0.23245130196']
ErFIn 2 ['F0.777778In0.111111Er0.111111', '-3.452', '-0.243434845858']
ErFK 6 ['F0.714286K0.142857Er0.142857', '-3.679', '-0.259483725497']
ErFKr 1 ['F0.8Kr0.1Er0.1', '-2.141', '-0.270749768186']
ErFLa 2 ['F0.777778La0.111111Er0.111111', '-4.336', '-0.371128884933']
ErFLi 6 ['Li0.222222F0.666667Er0.111111', '-3.595', '-0.22095413076']
ErFLu 2 ['F0.777778Er0.111111Lu0.111111', '-4.09', '-0.317572951413']
ErFMg 2 ['F0.777778Mg0.111111Er0.111111', '-3.511', '-0.262508123076']
ErFMn 1 ['F0.8Mn0.1Er0.1', '-3.262', '-0.237361739107']
ErFMo 2 ['F0.777778Mo0.111111Er0.111111', '-3.665', '-0.264796175213']
ErFN 8 ['N0.333333F0.166667Er0.5', '-2.445', '-0.200373430171']
ErFNa 13 ['F0.714286Na0.142857Er0.142857', '-3.605', '-0.21715740502']
ErFNb 2 ['F0.8Nb0.1Er0.1', '-3.939', '-0.209239848944']
ErFNd 2 ['F0.777778Nd0.111111Er0.111111', '-4.164', '-0.237907389004']
ErFNi 2 ['F0.777778Ni0.111111Er0.111111', '-3.161', '-0.380064231413']
ErFNp 2 ['F0.777778Er0.111111Np0.111111', '-4.343', '-0.273045077574']
ErFO 4 ['O0.125F0.75Er0.125', '-2.44', '-0.21913677689']
ErFOs 1 ['F0.8Er0.1Os0.1', '-3.122', '-0.205177755902']
ErFP 2 ['F0.714286P0.142857Er0.142857', '-3.746', '-0.229726848181']
ErFPa 2 ['F0.777778Er0.111111Pa0.111111', '-4.262', '-0.689065834191']
ErFPb 2 ['F0.777778Er0.111111Pb0.111111', '-3.406', '-0.250268354291']
ErFPd 1 ['F0.8Pd0.1Er0.1', '-2.694', '-0.207204695862']
ErFPm 2 ['F0.8Pm0.1Er0.1', '-3.711', '-0.266604735524']

ErFPr 1 ['F0.8Pr0.1Er0.1', '-3.904', '-0.373843714524']
ErFPu 3 ['F0.7Er0.1Pu0.2', '-4.075', '-0.216754347859']
ErFRb 9 ['F0.571429Rb0.285714Er0.142857', '-3.567', '-0.212475843711']
ErFRe 1 ['F0.8Er0.1Re0.1', '-3.348', '-0.281708696232']
ErFRh 2 ['F0.777778Rh0.111111Er0.111111', '-3.034', '-0.26239811308']
ErFRu 2 ['F0.777778Ru0.111111Er0.111111', '-3.26', '-0.226520158736']
ErFS 1 ['F0.3S0.4Er0.3', '-3.125', '-0.212857436908']
ErFSb 1 ['F0.8Sb0.1Er0.1', '-3.437', '-0.482863534564']
ErFSc 2 ['F0.777778Sc0.111111Er0.111111', '-4.098', '-0.226195530302']
ErFSe 1 ['F0.111111Se0.555556Er0.333333', '-2.256', '-0.201580679079']
ErFSi 2 ['F0.75Si0.125Er0.125', '-4.009', '-0.220162402687']
ErFSn 2 ['F0.777778Sn0.111111Er0.111111', '-3.657', '-0.205403835747']
ErFSr 2 ['F0.8Sr0.1Er0.1', '-3.287', '-0.248513124685']
ErFTa 2 ['F0.8Er0.1Ta0.1', '-4.096', '-0.237695807946']
ErFTc 1 ['F0.8Tc0.1Er0.1', '-3.359', '-0.464952192487']
ErFTh 1 ['F0.8Er0.1Th0.1', '-4.293', '-0.286122805027']
ErFTi 2 ['F0.777778Ti0.111111Er0.111111', '-4.105', '-0.527176617107']
ErFTl 3 ['F0.714286Er0.142857Tl0.142857', '-3.609', '-0.209365773121']
ErFTm 6 ['F0.666667Er0.111111Tm0.222222', '-3.545', '-0.60239117076']
ErFV 1 ['F0.8V0.1Er0.1', '-3.7', '-0.424559653362']
ErFY 1 ['F0.8Y0.1Er0.1', '-3.873', '-0.417063579604']
ErFYb 8 ['F0.666667Er0.166667Yb0.166667', '-4.035', '-0.219840057189']
ErFZn 2 ['F0.75Zn0.125Er0.125', '-3.39', '-0.206469044609']
ErFZr 1 ['F0.8Zr0.1Er0.1', '-4.055', '-0.297828625737']
ErFeGe 1 ['Fe0.125Ge0.375Er0.5', '-0.936', '-0.213262031875']
ErFeI 23 ['Fe0.2I0.5Er0.3', '-0.952', '-0.219387631039']
ErFeO 1 ['O0.625Fe0.125Er0.25', '-3.266', '-0.301670166266']
ErFeSi 3 ['Si0.5Fe0.4Er0.1', '-0.819', '-0.211554130875']
ErGaI 24 ['Ga0.333333I0.5Er0.166667', '-0.896', '-0.2115830472']
ErGaIr 6 ['Ga0.4Er0.1Ir0.5', '-0.739', '-0.201402973833']
ErGaN 27 ['N0.444444Ga0.111111Er0.444444', '-1.883', '-0.206231668738']
ErGaNa 1 ['Na0.142857Ga0.714286Er0.142857', '-0.582', '-0.229506054939']
ErGaNi 2 ['Ni0.222222Ga0.444444Er0.333333', '-0.846', '-0.211827677381']
ErGaPd 1 ['Ga0.5Pd0.375Er0.125', '-0.938', '-0.20866828375']
ErGaPt 3 ['Ga0.6Er0.1Pt0.3', '-0.862', '-0.200613683625']
ErGaRh 3 ['Ga0.4Rh0.5Er0.1', '-0.966', '-0.226608262']
ErGaS 3 ['S0.333333Ga0.222222Er0.444444', '-1.822', '-0.220813380882']
ErGaSe 10 ['Ga0.2Se0.4Er0.4', '-1.695', '-0.2235595295']
ErGdI 9 ['I0.666667Gd0.111111Er0.222222', '-1.262', '-0.466434974756']
ErGeH 18 ['H0.25Ge0.375Er0.375', '-0.926', '-0.203117427163']
ErGeI 27 ['Ge0.3I0.5Er0.2', '-0.865', '-0.202021263876']
ErGeIr 4 ['Ge0.5Er0.1Ir0.4', '-0.763', '-0.207509294399']
ErGeN 3 ['N0.5Ge0.3Er0.2', '-1.049', '-0.210825386829']
ErGeOs 2 ['Ge0.5Er0.1Os0.4', '-0.437', '-0.209473881833']

ErGePa 2 ['Ge0.7Er0.1Pa0.2', '-0.531', '-0.223674982333']
ErGePt 9 ['Ge0.5Er0.2Pt0.3', '-0.991', '-0.203845019748']
ErGeRh 1 ['Ge0.375Rh0.125Er0.5', '-1.123', '-0.239648496563']
ErGeS 12 ['S0.444444Ge0.222222Er0.333333', '-1.934', '-0.204129023188']
ErGeSe 5 ['Ge0.25Se0.375Er0.375', '-1.614', '-0.211265430417']
ErHI 54 ['H0.666667I0.166667Er0.166667', '-0.628', '-0.200640502306']
ErHIr 10 ['H0.666667Er0.111111Ir0.222222', '-0.484', '-0.207512123889']
ErHMn 4 ['H0.571429Mn0.285714Er0.142857', '-0.548', '-0.200729607635']
ErHN 33 ['H0.25N0.25Er0.5', '-1.41', '-0.206511240619']
ErHO 4 ['H0.5O0.2Er0.3', '-1.913', '-0.208969905398']
ErHOs 3 ['H0.666667Er0.222222Os0.111111', '-0.768', '-0.227801611877']
ErHPm 3 ['H0.666667Pm0.222222Er0.111111', '-0.654', '-0.202305966322']
ErHPt 11 ['H0.625Er0.25Pt0.125', '-0.883', '-0.200864765302']
ErHRh 32 ['H0.375Rh0.5Er0.125', '-0.504', '-0.200138406681']
ErHRu 9 ['H0.6Ru0.1Er0.3', '-0.847', '-0.22226422469']
ErHS 35 ['H0.666667S0.111111Er0.222222', '-0.978', '-0.202221550003']
ErHSc 2 ['H0.6Sc0.3Er0.1', '-0.812', '-0.20100087169']
ErHSe 5 ['H0.2Se0.5Er0.3', '-1.624', '-0.2101594045']
ErHSi 29 ['H0.222222Si0.444444Er0.333333', '-0.839', '-0.203195006367']
ErHfI 5 ['I0.714286Er0.142857Hf0.142857', '-1.265', '-0.230528250501']
ErHfS 13 ['S0.5Er0.3Hf0.2', '-2.091', '-0.203089796493']
ErHgI 26 ['I0.444444Er0.333333Hg0.222222', '-1.015', '-0.22870044587']
ErHgO 1 ['O0.6Er0.3Hg0.1', '-3.257', '-0.243634694685']
ErHoI 9 ['I0.666667Ho0.111111Er0.222222', '-1.197', '-0.427570861053']
ErIIn 20 ['In0.25I0.5Er0.25', '-1.014', '-0.215387023641']
ErIIr 33 ['I0.444444Er0.222222Ir0.333333', '-0.747', '-0.204013674971']
ErIK 14 ['K0.1I0.6Er0.3', '-1.213', '-0.203662671601']
ErIKr 18 ['Kr0.333333I0.555556Er0.111111', '-0.477', '-0.220490978275']
ErILa 5 ['I0.714286La0.142857Er0.142857', '-1.597', '-0.311736322456']
ErILi 18 ['Li0.222222I0.555556Er0.222222', '-1.33', '-0.229454328234']
ErILu 14 ['I0.571429Er0.142857Lu0.285714', '-0.857', '-0.209724838517']
ErIMg 10 ['Mg0.125I0.625Er0.25', '-1.262', '-0.244423614365']
ErIMn 15 ['Mn0.1I0.8Er0.1', '-0.643', '-0.242934623583']
ErIMo 7 ['Mo0.2I0.7Er0.1', '-0.758', '-0.227060009309']
ErIN 32 ['N0.333333I0.111111Er0.555556', '-1.668', '-0.205216534173']
ErINa 16 ['Na0.1I0.6Er0.3', '-1.195', '-0.219202550015']
ErINb 8 ['Nb0.2I0.7Er0.1', '-0.885', '-0.206697534968']
ErINd 8 ['I0.666667Nd0.166667Er0.166667', '-1.338', '-0.243046045768']
ErINi 30 ['Ni0.444444I0.444444Er0.111111', '-0.573', '-0.207853908921']
ErINp 5 ['I0.714286Er0.142857Np0.142857', '-1.196', '-0.290283964062']
ErIO 27 ['O0.3I0.2Er0.5', '-2.545', '-0.227305053451']
ErIOs 21 ['I0.7Er0.1Os0.2', '-0.433', '-0.202141880447']
ErIP 47 ['P0.333333I0.5Er0.166667', '-0.953', '-0.210491312466']
ErIPa 12 ['I0.6Er0.1Pa0.3', '-0.589', '-0.274934650719']

ErIPb 26 ['I0.25Er0.375Pb0.375', '-0.82', '-0.20050249827']
ErIPd 34 ['Pd0.428571I0.285714Er0.285714', '-1.081', '-0.201729629049']
ErIPm 12 ['I0.6Pm0.3Er0.1', '-1.009', '-0.241367781969']
ErIPr 6 ['I0.666667Pr0.111111Er0.222222', '-1.343', '-0.233671422543']
ErIPt 49 ['I0.222222Er0.333333Pt0.444444', '-1.275', '-0.225649830596']
ErIPu 6 ['I0.666667Er0.222222Pu0.111111', '-1.31', '-0.260122749074']
ErIRb 14 ['Rb0.285714I0.571429Er0.142857', '-1.524', '-0.254005099598']
ErIRe 11 ['I0.6Er0.3Re0.1', '-0.954', '-0.204870201171']
ErIRh 43 ['Rh0.428571I0.285714Er0.285714', '-0.904', '-0.245033086451']
ErIRu 23 ['Ru0.3I0.5Er0.2', '-0.682', '-0.220283760895']
ErIS 29 ['S0.444444I0.111111Er0.444444', '-2.156', '-0.200471182612']
ErISb 36 ['Sb0.4I0.3Er0.3', '-1.078', '-0.225757033272']
ErISc 8 ['Sc0.166667I0.666667Er0.166667', '-1.275', '-0.274633970767']
ErISe 18 ['Se0.333333I0.555556Er0.111111', '-0.781', '-0.200457736397']
ErISi 30 ['Si0.333333I0.444444Er0.222222', '-0.861', '-0.200353493922']
ErISm 7 ['I0.7Sm0.2Er0.1', '-1.206', '-0.214335509364']
ErISn 18 ['Sn0.222222I0.444444Er0.333333', '-1.057', '-0.2023169933']
ErISr 7 ['Sr0.111111I0.777778Er0.111111', '-1.105', '-0.239839745235']
ErITa 6 ['I0.714286Er0.142857Ta0.142857', '-0.933', '-0.208452326682']
ErITb 14 ['I0.571429Tb0.285714Er0.142857', '-0.776', '-0.20420126147']
ErITc 16 ['Tc0.142857I0.571429Er0.285714', '-0.884', '-0.224405372707']
ErITe 55 ['Te0.444444I0.444444Er0.111111', '-0.746', '-0.215991483967']
ErITh 3 ['I0.75Er0.125Th0.125', '-1.442', '-0.295166850909']
ErITi 8 ['Ti0.2I0.7Er0.1', '-1.141', '-0.200881506784']
ErITl 27 ['I0.5Er0.1Tl0.4', '-1.015', '-0.210015739009']
ErITm 7 ['I0.666667Er0.222222Tm0.111111', '-1.279', '-0.348645023787']
ErIU 3 ['I0.75Er0.125U0.125', '-1.236', '-0.34176252997']
ErIV 9 ['V0.222222I0.666667Er0.111111', '-0.837', '-0.201780532751']
ErIW 9 ['I0.625Er0.25W0.125', '-0.803', '-0.225854701119']
ErIXe 32 ['I0.333333Xe0.555556Er0.111111', '-0.473', '-0.216490978275']
ErIY 5 ['Y0.142857I0.714286Er0.142857', '-1.435', '-0.211676206742']
ErIYb 6 ['I0.8Er0.1Yb0.1', '-1.002', '-0.237639357962']
ErIZn 23 ['Zn0.333333I0.5Er0.166667', '-0.873', '-0.21025732929']
ErIZr 5 ['Zr0.142857I0.714286Er0.142857', '-1.313', '-0.360858045668']
ErInN 22 ['N0.444444In0.111111Er0.444444', '-1.893', '-0.216231668738']
ErInS 5 ['S0.5In0.1Er0.4', '-2.178', '-0.200273873991']
ErIrN 4 ['N0.285714Er0.571429Ir0.142857', '-1.62', '-0.21636177176']
ErIrOs 1 ['Er0.625Os0.25Ir0.125', '-0.644', '-0.207782595833']
ErIrP 4 ['P0.285714Er0.428571Ir0.285714', '-1.549', '-0.200360431012']
ErIrPa 2 ['Er0.125Ir0.625Pa0.25', '-1.031', '-0.2282594825']
ErIrS 4 ['S0.555556Er0.111111Ir0.333333', '-1.169', '-0.201556876752']
ErIrSe 12 ['Se0.333333Er0.333333Ir0.333333', '-1.529', '-0.205222793889']
ErIrSi 3 ['Si0.5Er0.1Ir0.4', '-1.071', '-0.223392187167']
ErKSe 1 ['K0.2Se0.5Er0.3', '-1.821', '-0.206650521']

ErKrP 2 ['P0.8Kr0.1Er0.1', '-0.568', '-0.220395695104']
ErLaO 2 ['O0.625La0.25Er0.125', '-3.913', '-0.374962441534']
ErLiN 3 ['Li0.166667N0.333333Er0.5', '-1.464', '-0.206423751553']
ErLiO 1 ['Li0.2O0.4Er0.4', '-2.806', '-0.20823739298']
ErLiOs 3 ['Li0.142857Er0.571429Os0.285714', '-0.446', '-0.220356165714']
ErLiPt 1 ['Li0.222222Er0.111111Pt0.666667', '-0.99', '-0.230861152407']
ErLiS 7 ['Li0.111111S0.444444Er0.444444', '-2.087', '-0.214929407658']
ErLiSe 10 ['Li0.125Se0.375Er0.5', '-1.502', '-0.205424773751']
ErMgN 10 ['N0.375Mg0.375Er0.25', '-1.422', '-0.209374718388']
ErMgS 16 ['Mg0.3S0.5Er0.2', '-1.992', '-0.205159084242']
ErMnN 16 ['N0.428571Mn0.142857Er0.428571', '-1.821', '-0.204116251997']
ErMnSi 3 ['Si0.5Mn0.4Er0.1', '-0.758', '-0.211919971357']
ErMoO 1 ['O0.625Mo0.125Er0.25', '-3.577', '-0.3335524432']
ErNNa 2 ['N0.375Na0.125Er0.5', '-1.617', '-0.202226720498']
ErNNb 3 ['N0.5Nb0.166667Er0.333333', '-1.855', '-0.204877718997']
ErNNi 18 ['N0.428571Ni0.428571Er0.142857', '-0.753', '-0.202222319188']
ErNNp 21 ['N0.571429Er0.285714Np0.142857', '-1.648', '-0.223661155031']
ErNO 8 ['N0.3O0.1Er0.6', '-2.001', '-0.219740724643']
ErNOs 2 ['N0.375Er0.5Os0.125', '-1.665', '-0.200867131748']
ErNP 30 ['N0.333333P0.111111Er0.555556', '-1.846', '-0.202196746113']
ErNPa 18 ['N0.571429Er0.285714Pa0.142857', '-1.61', '-0.249807732489']
ErNPd 6 ['N0.333333Pd0.222222Er0.444444', '-1.772', '-0.201081630664']
ErNPt 16 ['N0.4Er0.3Pt0.3', '-1.38', '-0.203776060932']
ErNPu 12 ['N0.555556Er0.222222Pu0.222222', '-1.67', '-0.223117159849']
ErNRe 8 ['N0.5Er0.3Re0.2', '-1.361', '-0.205936598375']
ErNRh 7 ['N0.428571Rh0.142857Er0.428571', '-1.821', '-0.204116251997']
ErNRu 3 ['N0.333333Ru0.166667Er0.5', '-1.585', '-0.20232931822']
ErNSc 2 ['N0.444444Sc0.222222Er0.333333', '-1.938', '-0.201573907627']
ErNSe 4 ['N0.285714Se0.142857Er0.571429', '-1.784', '-0.222394349903']
ErNSi 32 ['N0.222222Si0.444444Er0.333333', '-1.253', '-0.207442966342']
ErNTa 1 ['N0.5Er0.3Ta0.2', '-1.863', '-0.21847688533']
ErNTc 7 ['N0.555556Tc0.222222Er0.222222', '-1.099', '-0.20246687822']
ErNTe 9 ['N0.4Te0.3Er0.3', '-1.339', '-0.207181376398']
ErNTi 10 ['N0.555556Ti0.222222Er0.222222', '-1.619', '-0.204155024587']
ErNTl 1 ['N0.4Er0.5Tl0.1', '-1.808', '-0.236913530364']
ErNU 7 ['N0.555556Er0.222222U0.222222', '-1.901', '-0.222223298141']
ErNW 9 ['N0.5Er0.4W0.1', '-1.748', '-0.20334112533']
ErNZn 7 ['N0.4Zn0.5Er0.1', '-0.589', '-0.211727125466']
ErNaO 4 ['O0.6Na0.1Er0.3', '-3.446', '-0.226182795935']
ErNaS 2 ['Na0.1S0.4Er0.5', '-1.914', '-0.235888498392']
ErNaSe 4 ['Na0.1Se0.4Er0.5', '-1.575', '-0.203731583916']
ErNbO 1 ['O0.625Nb0.125Er0.25', '-3.743', '-0.24140018202']
ErNbS 13 ['S0.666667Nb0.222222Er0.111111', '-1.664', '-0.220518871259']
ErNiO 1 ['O0.625Ni0.125Er0.25', '-2.99', '-0.244219294379']

ErNiOs 2 ['Ni0.142857Er0.571429Os0.285714', '-0.546', '-0.235811622143']
ErNiSe 1 ['Ni0.428571Se0.428571Er0.142857', '-1.033', '-0.208392395714']
ErNpPt 1 ['Er0.1Pt0.8Np0.1', '-0.723', '-0.210393814625']
ErNpS 2 ['S0.5Er0.333333Np0.166667', '-2.117', '-0.215738936533']
ErNpSe 1 ['Se0.666667Er0.111111Np0.222222', '-1.353', '-0.218994787871']
ErOP 1 ['O0.6P0.1Er0.3', '-3.769', '-0.2157731902']
ErOPa 4 ['O0.666667Er0.222222Pa0.111111', '-3.447', '-0.24926443451']
ErOPd 1 ['O0.6Pd0.1Er0.3', '-3.448', '-0.395636457515']
ErOPr 1 ['O0.625Pr0.125Er0.25', '-3.763', '-0.210076034113']
ErOPt 2 ['O0.6Er0.3Pt0.1', '-3.306', '-0.209555875471']
ErORh 1 ['O0.6Rh0.1Er0.3', '-3.391', '-0.235011833721']
ErOS 13 ['O0.714286S0.142857Er0.142857', '-2.416', '-0.220818567788']
ErOSb 1 ['O0.666667Sb0.222222Er0.111111', '-2.445', '-0.209046680799']
ErOSc 2 ['O0.625Sc0.25Er0.125', '-3.893', '-0.295680201378']
ErOSe 19 ['O0.444444Se0.222222Er0.333333', '-3.279', '-0.218048881644']
ErOSr 1 ['O0.625Sr0.125Er0.25', '-3.498', '-0.252841328198']
ErOTa 2 ['O0.666667Er0.111111Ta0.222222', '-3.668', '-0.321466348411']
ErOTc 1 ['O0.666667Tc0.166667Er0.166667', '-2.806', '-0.228093189967']
ErOTi 3 ['O0.6Ti0.2Er0.2', '-3.761', '-0.251733655215']
ErOZr 2 ['O0.6Zr0.2Er0.2', '-3.793', '-0.20052425837']
ErOsP 4 ['P0.555556Er0.222222Os0.222222', '-1.321', '-0.203990214466']
ErOsPt 2 ['Er0.625Os0.25Pt0.125', '-0.729', '-0.202987517812']
ErOsSe 3 ['Se0.375Er0.5Os0.125', '-1.528', '-0.20897213875']
ErOsTa 1 ['Er0.2Ta0.2Os0.6', '-0.403', '-0.224929122']
ErPPm 3 ['P0.555556Pm0.333333Er0.111111', '-1.382', '-0.201260824073']
ErPPt 1 ['P0.125Er0.25Pt0.625', '-1.318', '-0.211724228076']
ErPRh 1 ['P0.8Rh0.1Er0.1', '-0.934', '-0.277883867666']
ErPS 1 ['P0.142857S0.571429Er0.285714', '-1.929', '-0.200437952749']
ErPSn 1 ['P0.8Sn0.1Er0.1', '-0.652', '-0.216580982231']
ErPTc 1 ['P0.5Tc0.125Er0.375', '-1.668', '-0.212022722127']
ErPTe 1 ['P0.555556Te0.333333Er0.111111', '-0.611', '-0.219206716667']
ErPXe 3 ['P0.5Xe0.125Er0.375', '-1.53', '-0.22648385664']
ErPaS 6 ['S0.5Er0.25Pa0.25', '-2.125', '-0.20494002799']
ErPaSe 8 ['Se0.666667Er0.222222Pa0.111111', '-1.601', '-0.28614948']
ErPbS 2 ['S0.4Er0.4Pb0.2', '-1.884', '-0.205888498392']
ErPdSe 28 ['Se0.222222Pd0.333333Er0.444444', '-1.498', '-0.200288004167']
ErPmS 11 ['S0.444444Pm0.222222Er0.333333', '-2.016', '-0.201940601269']
ErPmSe 7 ['Se0.666667Pm0.222222Er0.111111', '-1.452', '-0.20791969']
ErPtS 13 ['S0.333333Er0.222222Pt0.444444', '-1.477', '-0.202576014239']
ErPtSe 18 ['Se0.375Er0.25Pt0.375', '-1.424', '-0.203919480721']
ErPtTh 3 ['Er0.111111Pt0.777778Th0.111111', '-0.977', '-0.237062098333']
ErPuS 13 ['S0.555556Er0.111111Pu0.333333', '-2.187', '-0.201150665176']
ErPuTe 1 ['Te0.5Er0.1Pu0.4', '-1.372', '-0.24653982933']
ErReS 7 ['S0.333333Er0.5Re0.166667', '-1.6', '-0.20157374866']

ErRhS 17 ['S0.5Rh0.375Er0.125', '-1.261', '-0.205158001796']
ErRhSe 25 ['Se0.555556Rh0.333333Er0.111111', '-0.964', '-0.202162314375']
ErRhSi 2 ['Si0.5Rh0.4Er0.1', '-1.097', '-0.2030745195']
ErRuSe 8 ['Se0.5Ru0.1Er0.4', '-1.838', '-0.201946416625']
ErSSb 5 ['S0.5Sb0.1Er0.4', '-2.277', '-0.26041545305']
ErSSi 21 ['Si0.166667S0.333333Er0.5', '-1.875', '-0.200856186993']
ErSSn 4 ['S0.4Sn0.1Er0.5', '-2.02', '-0.203741907493']
ErSTa 7 ['S0.5Er0.3Ta0.2', '-1.997', '-0.203730410057']
ErSTc 10 ['S0.428571Tc0.428571Er0.142857', '-1.265', '-0.203966568876']
ErSTh 2 ['S0.444444Er0.222222Th0.333333', '-2.03', '-0.211773220436']
ErSTl 8 ['S0.5Er0.333333Tl0.166667', '-2.072', '-0.201592139383']
ErSV 1 ['S0.5V0.2Er0.3', '-2.046', '-0.239824539994']
ErSZr 13 ['S0.571429Zr0.285714Er0.142857', '-2.01', '-0.205206484729']
ErSbSe 3 ['Se0.3Sb0.3Er0.4', '-1.605', '-0.223909544']
ErScSe 21 ['Sc0.375Se0.5Er0.125', '-1.821', '-0.202268428125']
ErSeSi 1 ['Si0.25Se0.375Er0.375', '-1.648', '-0.250068265625']
ErSeTc 5 ['Se0.666667Tc0.222222Er0.111111', '-0.736', '-0.212355335']
ErSeTe 1 ['Se0.166667Te0.5Er0.333333', '-1.569', '-0.267356211389']
ErSeTi 1 ['Ti0.125Se0.5Er0.375', '-1.808', '-0.20657674625']
ErSeTl 1 ['Se0.5Er0.4Tl0.1', '-1.844', '-0.2239489335']
ErSeTm 1 ['Se0.6Er0.1Tm0.3', '-1.86', '-0.211196134502']
ErSeY 7 ['Se0.555556Y0.333333Er0.111111', '-1.92', '-0.21908785']
ErSeZn 13 ['Zn0.2Se0.5Er0.3', '-1.693', '-0.207289934']
ErSeZr 3 ['Se0.6Zr0.3Er0.1', '-1.618', '-0.205799801458']
ErSiTc 2 ['Si0.5Tc0.4Er0.1', '-0.725', '-0.213467343']
EuFFe 2 ['F0.777778Fe0.111111Eu0.111111', '-2.849', '-0.263467704926']
EuFGa 3 ['F0.75Ga0.125Eu0.125', '-3.372', '-0.223495247416']
EuFGd 4 ['F0.7Eu0.1Gd0.2', '-4.369', '-0.228870169026']
EuFGe 2 ['F0.777778Ge0.111111Eu0.111111', '-3.186', '-0.231292313359']
EuFH 3 ['H0.111111F0.777778Eu0.111111', '-2.222', '-0.338908672823']
EuFHf 1 ['F0.8Eu0.1Hf0.1', '-3.793', '-0.375163452023']
EuFHg 2 ['F0.777778Eu0.111111Hg0.111111', '-2.336', '-0.262108559833']
EuFHo 2 ['F0.8Eu0.1Ho0.1', '-3.436', '-0.289158357101']
EuFI 1 ['F0.285714I0.285714Eu0.428571', '-2.966', '-0.208262765266']
EuFIn 3 ['F0.75In0.125Eu0.125', '-3.311', '-0.209835728666']
EuFK 10 ['F0.6K0.3Eu0.1', '-3.344', '-0.226274941931']
EuFKr 3 ['F0.75Kr0.125Eu0.125', '-2.088', '-0.258658737309']
EuFLa 2 ['F0.777778La0.111111Eu0.111111', '-3.726', '-0.213103575668']
EuFLi 7 ['Li0.2F0.7Eu0.1', '-2.834', '-0.204135939345']
EuFLu 3 ['F0.75Eu0.125Lu0.125', '-4.027', '-0.291491097416']
EuFMg 8 ['F0.6Mg0.3Eu0.1', '-3.879', '-0.215182421685']
EuFN 21 ['N0.333333F0.222222Eu0.444444', '-2.132', '-0.224280276189']
EuFNa 13 ['F0.5Na0.2Eu0.3', '-3.625', '-0.200216607933']
EuFNb 1 ['F0.8Nb0.1Eu0.1', '-3.665', '-0.342017070605']

EuFNd 2 ['F0.777778Nd0.111111Eu0.111111', '-3.753', '-0.278882079739']
EuFNi 3 ['F0.75Ni0.125Eu0.125', '-2.826', '-0.205918787416']
EuFNp 2 ['F0.777778Eu0.111111Np0.111111', '-4.071', '-0.453019768309']
EuFO 4 ['O0.2F0.7Eu0.1', '-1.602', '-0.232086643173']
EuFOs 1 ['F0.777778Eu0.111111Os0.111111', '-3.012', '-0.223061086181']
EuFP 17 ['F0.125P0.25Eu0.625', '-1.619', '-0.20390275787']
EuFPa 3 ['F0.75Eu0.125Pa0.125', '-4.094', '-0.582920590541']
EuFPb 2 ['F0.777778Eu0.111111Pb0.111111', '-2.948', '-0.244243045026']
EuFPd 1 ['F0.8Pd0.1Eu0.1', '-2.331', '-0.250981917523']
EuFPm 1 ['F0.8Pm0.1Eu0.1', '-3.239', '-0.201381957185']
EuFPr 1 ['F0.8Pr0.1Eu0.1', '-3.352', '-0.228620936185']
EuFPu 5 ['F0.7Eu0.1Pu0.2', '-4.194', '-0.222142801106']
EuFRb 8 ['F0.714286Rb0.142857Eu0.142857', '-3.057', '-0.28358616037']
EuFRe 1 ['F0.8Eu0.1Re0.1', '-3.164', '-0.246565772837']
EuFRh 2 ['F0.777778Rh0.111111Eu0.111111', '-2.604', '-0.284372803814']
EuFRu 2 ['F0.777778Ru0.111111Eu0.111111', '-2.856', '-0.27449484947']
EuFSb 12 ['F0.222222Sb0.333333Eu0.444444', '-2.382', '-0.214472296303']
EuFSc 3 ['F0.75Sc0.125Eu0.125', '-4.084', '-0.236691498666']
EuFSe 13 ['F0.333333Se0.166667Eu0.5', '-3.222', '-0.203182940288']
EuFSi 3 ['F0.666667Si0.111111Eu0.222222', '-4.227', '-0.253578096412']
EuFSm 1 ['F0.777778Sm0.111111Eu0.111111', '-3.698', '-0.210555610206']
EuFSr 1 ['F0.8Sr0.1Eu0.1', '-2.918', '-0.286290346346']
EuFTa 1 ['F0.8Eu0.1Ta0.1', '-3.669', '-0.217473029607']
EuFTc 2 ['F0.777778Tc0.111111Eu0.111111', '-3.214', '-0.45036601572']
EuFTh 2 ['F0.777778Eu0.111111Th0.111111', '-4.266', '-0.265888918542']
EuFTi 3 ['F0.75Ti0.125Eu0.125', '-3.858', '-0.341420221321']
EuFTl 1 ['F0.777778Eu0.111111Tl0.111111', '-2.653', '-0.217920255481']
EuFTm 11 ['F0.6Eu0.1Tm0.3', '-3.27', '-0.592600591431']
EuFV 2 ['F0.777778V0.111111Eu0.111111', '-3.519', '-0.331596527803']
EuFY 2 ['F0.777778Y0.111111Eu0.111111', '-3.848', '-0.460045334739']
EuFYb 9 ['F0.8Eu0.1Yb0.1', '-2.846', '-0.200080847433']
EuFZn 1 ['F0.8Zn0.1Eu0.1', '-2.444', '-0.303952457348']
EuFZr 1 ['F0.8Zr0.1Eu0.1', '-3.677', '-0.326605847398']
EuFeN 15 ['N0.4Fe0.3Eu0.3', '-0.661', '-0.263229743362']
EuFeO 2 ['O0.6Fe0.2Eu0.2', '-2.636', '-0.21933657929']
EuFeP 9 ['P0.4Fe0.3Eu0.3', '-1.222', '-0.20656843367']
EuFeSi 1 ['Si0.5Fe0.375Eu0.125', '-0.8', '-0.2575966075']
EuGaI 1 ['Ga0.142857I0.571429Eu0.285714', '-1.83', '-0.228563692894']
EuGaN 38 ['N0.25Ga0.5Eu0.25', '-0.827', '-0.200485582831']
EuGaO 5 ['O0.6Ga0.1Eu0.3', '-2.796', '-0.215835824356']
EuGaP 1 ['P0.4Ga0.1Eu0.5', '-1.295', '-0.206066638416']
EuGaPd 6 ['Ga0.571429Pd0.285714Eu0.142857', '-0.869', '-0.203529710714']
EuGaPt 7 ['Ga0.6Eu0.1Pt0.3', '-0.862', '-0.203888161666']
EuGaRh 5 ['Ga0.428571Rh0.428571Eu0.142857', '-0.871', '-0.213647593857']

EuGaS 1 ['S0.444444Ga0.222222Eu0.333333', '-2.058', '-0.235779021825']
EuGaSe 1 ['Ga0.1Se0.4Eu0.5', '-2.113', '-0.234003895']
EuGdI 8 ['I0.777778Eu0.111111Gd0.111111', '-1.17', '-0.264672232109']
EuGdN 1 ['N0.4Eu0.5Gd0.1', '-0.932', '-0.241521602113']
EuGdO 4 ['O0.625Eu0.25Gd0.125', '-3.127', '-0.243560328364']
EuGdP 1 ['P0.444444Eu0.444444Gd0.111111', '-1.363', '-0.232846957131']
EuGeH 7 ['H0.5Ge0.125Eu0.375', '-0.89', '-0.202618288283']
EuGeI 18 ['Ge0.4I0.2Eu0.4', '-1.301', '-0.207355815513']
EuGeIr 4 ['Ge0.5Eu0.125Ir0.375', '-0.806', '-0.209249706226']
EuGeLi 1 ['Li0.428571Ge0.428571Eu0.142857', '-0.714', '-0.200410502321']
EuGeN 55 ['N0.428571Ge0.428571Eu0.142857', '-0.545', '-0.201362299675']
EuGeO 2 ['O0.6Ge0.2Eu0.2', '-2.942', '-0.23845288347']
EuGeP 7 ['P0.5Ge0.2Eu0.3', '-1.144', '-0.204685943322']
EuGePa 4 ['Ge0.6Eu0.1Pa0.3', '-0.628', '-0.2339764835']
EuGePd 8 ['Ge0.444444Pd0.444444Eu0.111111', '-0.814', '-0.200066890833']
EuGePm 1 ['Ge0.4Pm0.5Eu0.1', '-0.74', '-0.20133782325']
EuGeS 12 ['S0.333333Ge0.111111Eu0.555556', '-2.095', '-0.20412055616']
EuGeSe 24 ['Ge0.125Se0.5Eu0.375', '-1.919', '-0.208115055937']
EuHIr 10 ['H0.5Eu0.333333Ir0.166667', '-0.858', '-0.227601129741']
EuHN 42 ['H0.625N0.25Eu0.125', '-0.647', '-0.201888169799']
EuHP 2 ['H0.5P0.166667Eu0.333333', '-0.993', '-0.218871713431']
EuHPm 4 ['H0.7Pm0.2Eu0.1', '-0.647', '-0.206417653721']
EuHPt 4 ['H0.555556Eu0.222222Pt0.222222', '-0.812', '-0.218403310625']
EuHRh 2 ['H0.6Rh0.2Eu0.2', '-0.597', '-0.211533737126']
EuHRu 1 ['H0.6Ru0.1Eu0.3', '-0.823', '-0.21731816669']
EuHSb 16 ['H0.5Sb0.1Eu0.4', '-0.961', '-0.210344730783']
EuHSe 6 ['H0.2Se0.3Eu0.5', '-1.726', '-0.209136231563']
EuHSi 11 ['H0.5Si0.3Eu0.2', '-0.59', '-0.204533737126']
EuHSn 6 ['H0.625Sn0.125Eu0.25', '-0.711', '-0.207549471567']
EuHfN 13 ['N0.5Eu0.125Hf0.375', '-1.747', '-0.211468104205']
EuHfP 5 ['P0.5Eu0.333333Hf0.166667', '-1.427', '-0.206309739689']
EuHfS 5 ['S0.555556Eu0.111111Hf0.333333', '-2.013', '-0.230417158326']
EuHfSe 5 ['Se0.555556Eu0.111111Hf0.333333', '-1.67', '-0.203959418333']
EuHgI 7 ['I0.444444Eu0.333333Hg0.222222', '-1.631', '-0.201981718925']
EuHgN 12 ['N0.3Eu0.4Hg0.3', '-0.746', '-0.209138114174']
EuHgO 4 ['O0.6Eu0.3Hg0.1', '-2.351', '-0.222225392818']
EuHgP 18 ['P0.4Eu0.3Hg0.3', '-1.114', '-0.219757769419']
EuHgSe 1 ['Se0.333333Eu0.555556Hg0.111111', '-1.88', '-0.259174683893']
EuHoI 9 ['I0.8Eu0.1Ho0.1', '-1.016', '-0.224727306566']
EuHoN 17 ['N0.5Eu0.3Ho0.2', '-1.3', '-0.213580111661']
EuHoO 4 ['O0.625Eu0.25Ho0.125', '-3.117', '-0.208089008183']
EuHoOs 1 ['Eu0.111111Ho0.555556Os0.333333', '-0.262', '-0.220860868889']
EuHoP 3 ['P0.5Eu0.333333Ho0.166667', '-1.529', '-0.203584105799']
EuHoSe 1 ['Se0.444444Eu0.111111Ho0.444444', '-1.862', '-0.223643184442']

EuIIr 5 ['I0.3Eu0.5Ir0.2', '-1.297', '-0.201714507341']
EuILi 10 ['Li0.222222I0.444444Eu0.333333', '-1.453', '-0.207438427807']
EuILu 10 ['I0.625Eu0.125Lu0.25', '-1.232', '-0.253578498784']
EuIMg 4 ['Mg0.1I0.6Eu0.3', '-1.889', '-0.207491877539']
EuIN 26 ['N0.25I0.375Eu0.375', '-1.476', '-0.208160926835']
EuINa 6 ['Na0.3I0.5Eu0.2', '-1.63', '-0.219828018529']
EuINb 1 ['Nb0.222222I0.666667Eu0.111111', '-1.17', '-0.201368512083']
EuINi 1 ['Ni0.1I0.5Eu0.4', '-1.673', '-0.21530089647']
EuIO 1 ['O0.1I0.6Eu0.3', '-2.047', '-0.227566146938']
EuIP 17 ['P0.333333I0.111111Eu0.555556', '-1.436', '-0.200516112855']
EuIPa 7 ['I0.666667Eu0.222222Pa0.111111', '-1.507', '-0.230620935315']
EuIPb 7 ['I0.555556Eu0.333333Pb0.111111', '-1.852', '-0.206353072258']
EuIPd 18 ['Pd0.2I0.4Eu0.4', '-1.668', '-0.200990815026']
EuIPm 10 ['I0.8Pm0.1Eu0.1', '-0.944', '-0.20457259302']
EuIPt 24 ['I0.666667Eu0.111111Pt0.222222', '-1.01', '-0.209959248379']
EuIPu 4 ['I0.75Eu0.125Pu0.125', '-1.519', '-0.214405007231']
EuIRh 18 ['Rh0.3I0.4Eu0.3', '-1.487', '-0.200609630526']
EuIS 1 ['S0.333333I0.166667Eu0.5', '-2.427', '-0.313024612421']
EuISb 31 ['Sb0.125I0.625Eu0.25', '-1.689', '-0.206723282021']
EuISe 30 ['Se0.125I0.5Eu0.375', '-2.167', '-0.214022132532']
EuISi 4 ['Si0.1I0.5Eu0.4', '-1.767', '-0.225468859532']
EuISm 2 ['I0.714286Sm0.142857Eu0.142857', '-1.548', '-0.201765530049']
EuISn 2 ['Sn0.1I0.5Eu0.4', '-1.781', '-0.211218557097']
EuITb 10 ['I0.625Eu0.125Tb0.25', '-1.137', '-0.224620368868']
EuITe 11 ['Te0.25I0.375Eu0.375', '-1.937', '-0.200856777836']
EuITl 8 ['I0.555556Eu0.333333Tl0.111111', '-1.841', '-0.212051239203']
EuITm 2 ['I0.75Eu0.125Tm0.125', '-1.371', '-0.200867566283']
EuIZn 3 ['Zn0.1I0.6Eu0.3', '-1.882', '-0.200491877539']
EuInN 47 ['N0.222222In0.333333Eu0.444444', '-0.799', '-0.215499507146']
EuInO 4 ['O0.625In0.125Eu0.25', '-2.552', '-0.264177992116']
EuInP 2 ['P0.333333In0.111111Eu0.555556', '-1.14', '-0.208479547293']
EuInPd 1 ['Pd0.375In0.5Eu0.125', '-0.821', '-0.200735638125']
EuInS 3 ['S0.4In0.3Eu0.3', '-1.833', '-0.220214215393']
EuInSe 5 ['Se0.4In0.1Eu0.5', '-2.087', '-0.206242958']
EuIrN 16 ['N0.375Eu0.375Ir0.25', '-0.801', '-0.288955608664']
EuIrO 1 ['O0.6Eu0.2Ir0.2', '-2.301', '-0.20357279297']
EuIrP 22 ['P0.625Eu0.125Ir0.25', '-1.213', '-0.210560199484']
EuIrPa 1 ['Eu0.125Ir0.625Pa0.25', '-0.948', '-0.204979937969']
EuIrSe 2 ['Se0.375Eu0.5Ir0.125', '-1.997', '-0.211349127143']
EuIrSi 3 ['Si0.6Eu0.1Ir0.3', '-0.909', '-0.226464030667']
EuIrSn 1 ['Sn0.5Eu0.166667Ir0.333333', '-0.812', '-0.233244389907']
EuKN 4 ['N0.333333K0.166667Eu0.5', '-0.623', '-0.237406228219']
EuKP 3 ['P0.428571K0.142857Eu0.428571', '-1.17', '-0.211883324377']
EuKSe 3 ['K0.142857Se0.428571Eu0.428571', '-2.092', '-0.200384804286']

EuKrN 7 ['N0.333333Kr0.222222Eu0.444444', '-0.635', '-0.249406228219']
EuKrO 1 ['O0.6Kr0.2Eu0.2', '-1.569', '-0.210405176824']
EuKrP 3 ['P0.444444Kr0.111111Eu0.444444', '-1.22', '-0.226397521577']
EuLaN 7 ['N0.444444La0.444444Eu0.111111', '-1.484', '-0.216049379849']
EuLaO 5 ['O0.666667La0.222222Eu0.111111', '-3.024', '-0.206703910302']
EuLaP 1 ['P0.5La0.1Eu0.4', '-1.435', '-0.201043726523']
EuLiN 23 ['Li0.5N0.3Eu0.2', '-0.641', '-0.202963155397']
EuLiO 1 ['Li0.125O0.625Eu0.25', '-2.372', '-0.214273926325']
EuLiP 21 ['Li0.1P0.5Eu0.4', '-1.351', '-0.205871114857']
EuLiSb 2 ['Li0.25Sb0.375Eu0.375', '-1.108', '-0.203800224167']
EuLiSe 19 ['Li0.555556Se0.333333Eu0.111111', '-1.54', '-0.201516063336']
EuLiSn 3 ['Li0.4Sn0.3Eu0.3', '-0.72', '-0.201631526873']
EuLuN 2 ['N0.4Eu0.5Lu0.1', '-0.951', '-0.207254690363']
EuLuO 4 ['O0.6Eu0.1Lu0.3', '-3.925', '-0.259092719519']
EuLuP 1 ['P0.5Eu0.2Lu0.3', '-1.682', '-0.208128778521']
EuLuPt 1 ['Eu0.1Lu0.5Pt0.4', '-1.359', '-0.233127551347']
EuMgN 19 ['N0.444444Mg0.222222Eu0.333333', '-0.933', '-0.270910015866']
EuMgO 5 ['O0.666667Mg0.166667Eu0.166667', '-2.33', '-0.201243046368']
EuMgP 15 ['Mg0.111111P0.333333Eu0.555556', '-0.982', '-0.214742700164']
EuMgS 6 ['Mg0.125S0.375Eu0.5', '-2.099', '-0.221436606097']
EuMgSe 12 ['Mg0.333333Se0.444444Eu0.222222', '-1.745', '-0.200696876853']
EuMnN 34 ['N0.5Mn0.25Eu0.25', '-0.642', '-0.20118666138']
EuMnP 4 ['P0.4Mn0.2Eu0.4', '-1.102', '-0.207757769419']
EuMoN 18 ['N0.444444Mo0.333333Eu0.222222', '-0.668', '-0.201669827069']
EuNNa 18 ['N0.4Na0.2Eu0.4', '-0.677', '-0.214287473862']
EuNNb 15 ['N0.5Nb0.333333Eu0.166667', '-1.178', '-0.200111048996']
EuNNi 45 ['N0.3Ni0.6Eu0.1', '-0.342', '-0.207815447655']
EuNNp 29 ['N0.5Eu0.4Np0.1', '-0.95', '-0.244796031452']
EuNO 19 ['N0.3O0.2Eu0.5', '-1.914', '-0.208370782221']
EuNOs 19 ['N0.5Eu0.25Os0.25', '-0.544', '-0.254804671164']
EuNP 53 ['N0.555556P0.333333Eu0.111111', '-0.794', '-0.201474942761']
EuNPa 26 ['N0.571429Eu0.285714Pa0.142857', '-0.823', '-0.210221283916']
EuNPb 14 ['N0.3Eu0.4Pb0.3', '-0.744', '-0.212388803397']
EuNPd 26 ['N0.111111Pd0.444444Eu0.444444', '-1.014', '-0.205482511073']
EuNPm 1 ['N0.4Pm0.1Eu0.5', '-0.87', '-0.214657587613']
EuNPr 1 ['N0.4Pr0.1Eu0.5', '-0.882', '-0.256356160113']
EuNPt 26 ['N0.1Eu0.6Pt0.3', '-1.133', '-0.203222326509']
EuNPu 26 ['N0.444444Eu0.111111Pu0.444444', '-1.427', '-0.21000265096']
EuNRb 5 ['N0.3Rb0.1Eu0.6', '-0.555', '-0.207965605397']
EuNRe 23 ['N0.4Eu0.3Re0.3', '-0.598', '-0.216098438362']
EuNRh 11 ['N0.25Rh0.125Eu0.625', '-0.727', '-0.224986826789']
EuNRu 12 ['N0.333333Ru0.222222Eu0.444444', '-0.657', '-0.271406228219']
EuNS 7 ['N0.111111S0.333333Eu0.555556', '-1.986', '-0.210578944733']
EuNSb 15 ['N0.2Sb0.2Eu0.6', '-1.048', '-0.228864245598']

EuNSc 11 ['N0.5Sc0.375Eu0.125', '-1.876', '-0.215956584204']
EuNSe 19 ['N0.333333Se0.222222Eu0.444444', '-1.439', '-0.20109997659']
EuNSi 15 ['N0.428571Si0.285714Eu0.285714', '-1.432', '-0.232417893887']
EuNSm 2 ['N0.444444Sm0.111111Eu0.444444', '-0.952', '-0.213857330125']
EuNSn 7 ['N0.4Sn0.2Eu0.4', '-0.781', '-0.212594388561']
EuNSr 7 ['N0.333333Sr0.166667Eu0.5', '-0.663', '-0.218915673219']
EuNTa 15 ['N0.444444Eu0.222222Ta0.333333', '-1.193', '-0.209961257626']
EuNTc 29 ['N0.625Tc0.25Eu0.125', '-0.44', '-0.229984759914']
EuNTe 26 ['N0.3Te0.3Eu0.4', '-1.414', '-0.202000835464']
EuNTh 7 ['N0.5Eu0.2Th0.3', '-1.549', '-0.221862537328']
EuNTi 19 ['N0.571429Ti0.285714Eu0.142857', '-1.107', '-0.200581628089']
EuNTl 19 ['N0.333333Eu0.333333Tl0.333333', '-0.611', '-0.202212727443']
EuNTm 6 ['N0.428571Eu0.285714Tm0.285714', '-1.47', '-0.202271884854']
EuNU 16 ['N0.444444Eu0.222222U0.333333', '-1.395', '-0.239976763734']
EuNV 7 ['N0.333333V0.111111Eu0.555556', '-0.765', '-0.229513958219']
EuNW 25 ['N0.428571Eu0.285714W0.285714', '-0.634', '-0.252680514853']
EuNXe 9 ['N0.3Xe0.3Eu0.4', '-0.555', '-0.207965605397']
EuNY 2 ['N0.428571Y0.142857Eu0.428571', '-1.054', '-0.210570268425']
EuNYb 3 ['N0.333333Eu0.555556Yb0.111111', '-0.751', '-0.257133221552']
EuNZn 29 ['N0.222222Zn0.333333Eu0.444444', '-0.681', '-0.206819602701']
EuNZr 9 ['N0.5Zr0.25Eu0.25', '-1.41', '-0.228220387329']
EuNaP 20 ['Na0.25P0.25Eu0.5', '-0.764', '-0.205098605887']
EuNaSe 6 ['Na0.111111Se0.444444Eu0.444444', '-2.214', '-0.252324982222']
EuNbO 2 ['O0.625Nb0.25Eu0.125', '-3.282', '-0.230523575336']
EuNbSe 4 ['Se0.555556Nb0.222222Eu0.222222', '-1.665', '-0.202888901806']
EuNdP 5 ['P0.444444Nd0.111111Eu0.444444', '-1.331', '-0.215442924076']
EuNiO 3 ['O0.6Ni0.1Eu0.3', '-2.579', '-0.272949995919']
EuNiP 5 ['P0.428571Ni0.142857Eu0.428571', '-1.254', '-0.215251417503']
EuNiSe 7 ['Ni0.375Se0.375Eu0.25', '-1.4', '-0.20827019875']
EuNiSi 2 ['Si0.5Ni0.333333Eu0.166667', '-0.824', '-0.209760529583']
EuNpO 1 ['O0.666667Eu0.111111Np0.222222', '-3.713', '-0.483903676289']
EuNpP 7 ['P0.5Eu0.25Np0.25', '-1.277', '-0.202357139897']
EuNpPt 2 ['Eu0.111111Pt0.777778Np0.111111', '-0.709', '-0.228520128471']
EuNpS 5 ['S0.5Eu0.166667Np0.333333', '-2.044', '-0.214885476743']
EuNpSe 3 ['Se0.5Eu0.1Np0.4', '-1.492', '-0.200526853496']
EuOOs 4 ['O0.666667Eu0.111111Os0.222222', '-1.967', '-0.200374514413']
EuOP 23 ['O0.625P0.125Eu0.25', '-3.202', '-0.20878990208']
EuOPa 8 ['O0.6Eu0.3Pa0.1', '-3.289', '-0.259975056059']
EuOPb 1 ['O0.625Eu0.25Pb0.125', '-2.47', '-0.259995569871']
EuOPd 2 ['O0.625Pd0.125Eu0.25', '-2.281', '-0.254179768309']
EuOPt 1 ['O0.625Eu0.25Pt0.125', '-2.339', '-0.231360441383']
EuOPu 3 ['O0.6Eu0.2Pu0.2', '-3.741', '-0.23932110998']
EuORe 4 ['O0.625Eu0.25Re0.125', '-2.888', '-0.23937165195']
EuORh 6 ['O0.625Rh0.125Eu0.25', '-2.391', '-0.223811156121']

EuOS 1 ['O0.625S0.125Eu0.25', '-3.084', '-0.235845916765']
EuOSb 2 ['O0.666667Sb0.222222Eu0.111111', '-2.221', '-0.222852327883']
EuOSc 4 ['O0.625Sc0.25Eu0.125', '-3.513', '-0.27682733769']
EuOSe 17 ['O0.2Se0.3Eu0.5', '-2.888', '-0.205274539824']
EuOSi 6 ['O0.333333Si0.111111Eu0.555556', '-2.642', '-0.205328649151']
EuOSm 2 ['O0.6Sm0.3Eu0.1', '-3.678', '-0.222929537327']
EuOTa 2 ['O0.625Eu0.125Ta0.25', '-3.563', '-0.29508986614']
EuOTb 1 ['O0.625Eu0.25Tb0.125', '-3.049', '-0.238172868017']
EuOTc 5 ['O0.666667Tc0.222222Eu0.111111', '-2.379', '-0.26857839293']
EuOTe 3 ['O0.666667Te0.166667Eu0.166667', '-2.178', '-0.225471827758']
EuOTl 2 ['O0.625Eu0.125Tl0.25', '-1.706', '-0.250588076431']
EuOTm 4 ['O0.6Eu0.2Tm0.2', '-3.581', '-0.256482774809']
EuOU 1 ['O0.666667Eu0.111111U0.222222', '-3.647', '-0.209054446212']
EuOV 2 ['O0.6V0.1Eu0.3', '-3.159', '-0.223506262532']
EuOW 1 ['O0.6Eu0.2W0.2', '-3.341', '-0.428069261805']
EuOY 3 ['O0.6Y0.3Eu0.1', '-3.739', '-0.203556818015']
EuOZn 8 ['O0.666667Zn0.111111Eu0.222222', '-2.102', '-0.226027021373']
EuOsP 17 ['P0.4Eu0.3Os0.3', '-1.101', '-0.206757769419']
EuOsSc 1 ['Sc0.6Eu0.1Os0.3', '-0.406', '-0.2143858975']
EuOsSi 4 ['Si0.5Eu0.1Os0.4', '-0.661', '-0.234398222667']
EuPPa 10 ['P0.5Eu0.166667Pa0.333333', '-1.551', '-0.207872597605']
EuPPb 2 ['P0.3Eu0.6Pb0.1', '-1.074', '-0.204096659814']
EuPPd 2 ['P0.444444Pd0.111111Eu0.444444', '-1.395', '-0.202283429354']
EuPPm 22 ['P0.333333Pm0.5Eu0.166667', '-1.0', '-0.205487829513']
EuPPr 1 ['P0.444444Pr0.111111Eu0.444444', '-1.32', '-0.209228157131']
EuPPt 7 ['P0.333333Eu0.555556Pt0.111111', '-1.275', '-0.201555424793']
EuPPu 42 ['P0.6Eu0.2Pu0.2', '-1.389', '-0.216704805025']
EuPRb 3 ['P0.444444Rb0.111111Eu0.444444', '-1.258', '-0.264397521577']
EuPRh 16 ['P0.5Rh0.166667Eu0.333333', '-1.438', '-0.207525695524']
EuPRu 6 ['P0.5Ru0.125Eu0.375', '-1.405', '-0.215155765212']
EuPS 7 ['P0.285714S0.428571Eu0.285714', '-1.79', '-0.203403321433']
EuPSb 1 ['P0.3Sb0.1Eu0.6', '-1.18', '-0.215428581398']
EuPSc 23 ['P0.5Sc0.375Eu0.125', '-1.72', '-0.226243853958']
EuPSe 12 ['P0.142857Se0.285714Eu0.571429', '-1.783', '-0.20255097765']
EuPSi 1 ['Si0.111111P0.333333Eu0.555556', '-1.136', '-0.218451495627']
EuPSm 8 ['P0.5Sm0.1Eu0.4', '-1.439', '-0.204777394773']
EuPSn 10 ['P0.3Sn0.2Eu0.5', '-1.219', '-0.211268978694']
EuPSr 12 ['P0.428571Sr0.285714Eu0.285714', '-1.231', '-0.239423741278']
EuPTa 1 ['P0.5Eu0.125Ta0.375', '-1.132', '-0.230898197364']
EuPTc 11 ['P0.5Tc0.2Eu0.3', '-1.244', '-0.207931542916']
EuPTe 2 ['P0.125Te0.5Eu0.375', '-1.591', '-0.201538229205']
EuPTh 4 ['P0.5Eu0.3Th0.2', '-1.545', '-0.202461147272']
EuPTi 18 ['P0.333333Ti0.444444Eu0.222222', '-1.238', '-0.200509429275']
EuPTl 5 ['P0.444444Eu0.444444Tl0.111111', '-1.336', '-0.217452307688']

EuPTm 4 ['P0.5Eu0.3Tm0.2', '-1.59', '-0.227147093272']
EuPU 9 ['P0.428571Eu0.428571U0.142857', '-1.172', '-0.213883324377']
EuPW 1 ['P0.444444Eu0.444444W0.111111', '-1.198', '-0.204397521577']
EuPXe 5 ['P0.428571Xe0.142857Eu0.428571', '-1.169', '-0.210883324377']
EuPY 6 ['P0.5Y0.1Eu0.4', '-1.447', '-0.201966876023']
EuPYb 21 ['P0.5Eu0.125Yb0.375', '-1.248', '-0.203437620938']
EuPZn 9 ['P0.285714Zn0.142857Eu0.571429', '-0.948', '-0.201200505299']
EuPZr 14 ['P0.5Zr0.25Eu0.25', '-1.515', '-0.203021514897']
EuPaPt 1 ['Eu0.1Pt0.8Pa0.1', '-0.936', '-0.208675469999']
EuPaS 3 ['S0.666667Eu0.166667Pa0.166667', '-1.708', '-0.203274837076']
EuPaSe 9 ['Se0.666667Eu0.222222Pa0.111111', '-1.465', '-0.216601301111']
EuPaSi 2 ['Si0.666667Eu0.111111Pa0.222222', '-0.529', '-0.218352393333']
EuPbS 7 ['S0.4Eu0.3Pb0.3', '-1.82', '-0.200840353393']
EuPbSe 2 ['Se0.3Eu0.6Pb0.1', '-1.749', '-0.22564769575']
EuPdSe 28 ['Se0.444444Pd0.111111Eu0.444444', '-2.178', '-0.216324982222']
EuPmS 6 ['S0.4Pm0.5Eu0.1', '-1.895', '-0.278723562642']
EuPmSe 8 ['Se0.666667Pm0.166667Eu0.166667', '-1.477', '-0.201045134583']
EuPmTe 5 ['Te0.6Pm0.2Eu0.2', '-1.465', '-0.212096392164']
EuPtPu 1 ['Eu0.125Pt0.375Pu0.5', '-0.999', '-0.30733343389']
EuPtSe 24 ['Se0.375Eu0.25Pt0.375', '-1.391', '-0.200852770469']
EuPtSi 1 ['Si0.5Eu0.125Pt0.375', '-1.033', '-0.207049184688']
EuPtTh 6 ['Eu0.111111Pt0.666667Th0.222222', '-1.129', '-0.206738075554']
EuPuS 17 ['S0.555556Eu0.111111Pu0.333333', '-2.204', '-0.209461536101']
EuPuSe 7 ['Se0.555556Eu0.222222Pu0.222222', '-1.964', '-0.209312699999']
EuRbSe 10 ['Se0.444444Rb0.111111Eu0.444444', '-2.164', '-0.202324982222']
EuRhSi 6 ['Si0.555556Rh0.333333Eu0.111111', '-0.991', '-0.215890228809']
EuRuSi 2 ['Si0.5Ru0.375Eu0.125', '-0.917', '-0.211861774167']
EuSSb 8 ['S0.2Sb0.3Eu0.5', '-1.769', '-0.200004544696']
EuSSe 27 ['S0.2Se0.2Eu0.6', '-2.071', '-0.200112363196']
EuSSi 13 ['Si0.3S0.4Eu0.3', '-1.842', '-0.211319609145']
EuSTc 7 ['S0.6Tc0.3Eu0.1', '-1.3', '-0.200596248592']
EuSTl 1 ['S0.444444Eu0.333333Tl0.222222', '-2.002', '-0.207112918831']
EuSZn 1 ['S0.428571Zn0.285714Eu0.285714', '-1.886', '-0.201236619707']
EuSZr 7 ['S0.6Zr0.3Eu0.1', '-2.016', '-0.202406552588']
EuSbSe 20 ['Se0.444444Sb0.111111Eu0.444444', '-2.165', '-0.203324982222']
EuSeSi 19 ['Si0.25Se0.375Eu0.375', '-1.87', '-0.21483670375']
EuSeSn 1 ['Se0.333333Sn0.111111Eu0.555556', '-1.952', '-0.293494098683']
EuSeTa 3 ['Se0.6Eu0.2Ta0.2', '-1.58', '-0.206716452']
EuSeTc 4 ['Se0.625Tc0.25Eu0.125', '-0.789', '-0.23727890125']
EuSeTe 5 ['Se0.333333Te0.111111Eu0.555556', '-2.108', '-0.230698909629']
EuSeTl 3 ['Se0.375Eu0.5Tl0.125', '-2.013', '-0.217273338125']
EuSeXe 1 ['Se0.4Xe0.1Eu0.5', '-1.996', '-0.230492484']
EuSeY 1 ['Se0.6Y0.3Eu0.1', '-1.957', '-0.204329185002']
EuSeZn 12 ['Zn0.111111Se0.444444Eu0.444444', '-2.162', '-0.200324982222']

EuSeZr 10 ['Se0.5Zr0.2Eu0.3', '-2.004', '-0.225994794944']
EuTeY 1 ['Y0.3Te0.6Eu0.1', '-1.561', '-0.2349908765']
FFeGa 2 ['F0.777778Fe0.111111Ga0.111111', '-2.542', '-0.2020487178']
FFeGd 3 ['F0.75Fe0.125Gd0.125', '-3.475', '-0.201005508465']
FFeGe 3 ['F0.75Fe0.125Ge0.125', '-2.678', '-0.242155519871']
FFeH 23 ['H0.6F0.3Fe0.1', '-1.185', '-0.202394143542']
FFeHf 1 ['F0.8Fe0.1Hf0.1', '-3.303', '-0.29801110011']
FFeHg 3 ['F0.75Fe0.125Hg0.125', '-2.044', '-0.226931689921']
FFeHo 2 ['F0.777778Fe0.111111Ho0.111111', '-3.341', '-0.303228894653']
FFeI 10 ['F0.8Fe0.1I0.1', '-2.13', '-0.205701890952']
FFeIn 2 ['F0.777778Fe0.111111In0.111111', '-2.705', '-0.407129145578']
FFeK 6 ['F0.666667K0.222222Fe0.111111', '-2.812', '-0.36791192326']
FFeKr 3 ['F0.75Fe0.125Kr0.125', '-1.547', '-0.233718297417']
FFeLa 2 ['F0.777778Fe0.111111La0.111111', '-3.349', '-0.294823184653']
FFeLi 5 ['Li0.142857F0.714286Fe0.142857', '-2.523', '-0.25579848478']
FFeLu 5 ['F0.75Fe0.125Lu0.125', '-3.546', '-0.326550657525']
FFeMg 3 ['F0.75Mg0.125Fe0.125', '-3.047', '-0.416977725645']
FFeMn 1 ['F0.8Mn0.1Fe0.1', '-2.477', '-0.271986608855']
FFeMo 3 ['F0.75Fe0.125Mo0.125', '-2.639', '-0.212462646692']
FFeN 4 ['N0.142857F0.714286Fe0.142857', '-1.572', '-0.204763273229']
FFeNa 7 ['F0.6Na0.2Fe0.2', '-2.748', '-0.204610130021']
FFeNb 1 ['F0.8Fe0.1Nb0.1', '-3.222', '-0.311864718692']
FFeNd 2 ['F0.777778Fe0.111111Nd0.111111', '-3.347', '-0.331601688724']
FFeNi 2 ['F0.777778Fe0.111111Ni0.111111', '-2.273', '-0.402758531133']
FFeNp 2 ['F0.75Fe0.125Np0.125', '-3.387', '-0.206992661849']
FFeO 35 ['O0.555556F0.111111Fe0.333333', '-1.811', '-0.200432879404']
FFeP 9 ['F0.8P0.1Fe0.1', '-2.916', '-0.247182721796']
FFePa 6 ['F0.7Fe0.1Pa0.2', '-3.455', '-0.248231149107']
FFePb 2 ['F0.777778Fe0.111111Pb0.111111', '-2.549', '-0.303962654011']
FFePd 1 ['F0.8Fe0.1Pd0.1', '-1.969', '-0.30182956561']
FFePm 2 ['F0.777778Fe0.111111Pm0.111111', '-3.218', '-0.301588450302']
FFePr 2 ['F0.8Fe0.1Pr0.1', '-3.115', '-0.404468584272']
FFePt 1 ['F0.8Fe0.1Pt0.1', '-2.011', '-0.221989659569']
FFePu 5 ['F0.7Fe0.2Pu0.1', '-3.156', '-0.279359657108']
FFeRb 6 ['F0.666667Fe0.111111Rb0.222222', '-2.773', '-0.29907406048']
FFeRh 1 ['F0.8Fe0.1Rh0.1', '-2.041', '-0.36618317152']
FFeRu 1 ['F0.8Fe0.1Ru0.1', '-2.241', '-0.33049301261']
FFeS 2 ['F0.714286S0.142857Fe0.142857', '-2.254', '-0.209393088525']
FFeSb 2 ['F0.777778Fe0.111111Sb0.111111', '-2.583', '-0.211320449236']
FFeSc 2 ['F0.777778Sc0.111111Fe0.111111', '-3.323', '-0.361889830022']
FFeSe 9 ['F0.555556Fe0.222222Se0.222222', '-2.022', '-0.209793784556']
FFeSi 2 ['F0.777778Si0.111111Fe0.111111', '-3.15', '-0.2282536579']
FFeSm 2 ['F0.777778Fe0.111111Sm0.111111', '-3.358', '-0.329275219191']
FFeSn 1 ['F0.8Fe0.1Sn0.1', '-2.549', '-0.26218832192']

FFeSr 4 ['F0.75Fe0.125Sr0.125', '-3.038', '-0.240492070385']
FFeTa 1 ['F0.8Fe0.1Ta0.1', '-3.264', '-0.225320677694']
FFeTb 1 ['F0.8Fe0.1Tb0.1', '-3.06', '-0.390704074804']
FFeTc 1 ['F0.8Fe0.1Tc0.1', '-2.474', '-0.399577062235']
FFeTe 15 ['F0.555556Fe0.333333Te0.111111', '-2.065', '-0.207990967613']
FFeTh 1 ['F0.8Fe0.1Th0.1', '-3.488', '-0.300747674775']
FFeTi 3 ['F0.7Ti0.1Fe0.2', '-2.962', '-0.203175496232']
FFeTl 7 ['F0.7Fe0.1Tl0.2', '-2.376', '-0.212827530606']
FFeTm 33 ['F0.375Fe0.125Tm0.5', '-2.008', '-0.222768033036']
FFeU 2 ['F0.8Fe0.1U0.1', '-3.429', '-0.213475474196']
FFeV 1 ['F0.8V0.1Fe0.1', '-2.953', '-0.49718452311']
FFeXe 2 ['F0.777778Fe0.111111Xe0.111111', '-1.721', '-0.238830015119']
FFeY 3 ['F0.75Fe0.125Y0.125', '-3.574', '-0.27861056169']
FFeYb 28 ['F0.75Fe0.125Yb0.125', '-3.02', '-0.2286606194']
FFeZn 2 ['F0.777778Fe0.111111Zn0.111111', '-2.275', '-0.355889006039']
FFeZr 1 ['F0.8Fe0.1Zr0.1', '-3.359', '-0.421453495485']
FGaGd 4 ['F0.714286Ga0.142857Gd0.142857', '-3.679', '-0.210347360027']
FGaH 3 ['H0.125F0.75Ga0.125', '-2.086', '-0.24380089641']
FGaHf 3 ['F0.75Ga0.125Hf0.125', '-3.761', '-0.243687306696']
FGaHg 2 ['F0.777778Ga0.111111Hg0.111111', '-2.095', '-0.266689572708']
FGaHo 2 ['F0.777778Ga0.111111Ho0.111111', '-3.582', '-0.331090298542']
FGaI 2 ['F0.3Ga0.3I0.4', '-1.716', '-0.216193122122']
FGaIn 2 ['F0.777778Ga0.111111In0.111111', '-2.803', '-0.291990549467']
FGaIr 1 ['F0.777778Ga0.111111Ir0.111111', '-2.39', '-0.205794552344']
FGaK 14 ['F0.625K0.25Ga0.125', '-3.094', '-0.201377192415']
FGaKr 2 ['F0.777778Ga0.111111Kr0.111111', '-1.612', '-0.231499890482']
FGaLa 5 ['F0.75Ga0.125La0.125', '-3.907', '-0.23127016211']
FGaLi 5 ['Li0.142857F0.714286Ga0.142857', '-2.759', '-0.200310062557']
FGaLu 10 ['F0.285714Ga0.428571Lu0.285714', '-2.06', '-0.201748742867']
FGaMg 3 ['F0.75Mg0.125Ga0.125', '-3.083', '-0.21319680502']
FGaMo 2 ['F0.8Ga0.1Mo0.1', '-2.978', '-0.24390645786']
FGaN 15 ['N0.166667F0.333333Ga0.5', '-1.708', '-0.23836494162']
FGaNa 14 ['F0.6Na0.3Ga0.1', '-3.145', '-0.211711840766']
FGaNb 1 ['F0.714286Ga0.142857Nb0.142857', '-3.106', '-0.252933926945']
FGaNd 2 ['F0.777778Ga0.111111Nd0.111111', '-3.644', '-0.415463092613']
FGaNi 3 ['F0.714286Ni0.142857Ga0.142857', '-2.727', '-0.260681461334']
FGaNp 3 ['F0.75Ga0.125Np0.125', '-3.583', '-0.267755231015']
FGaO 16 ['O0.4F0.2Ga0.4', '-2.404', '-0.237506405487']
FGaP 3 ['F0.777778P0.111111Ga0.111111', '-3.034', '-0.23589811021']
FGaPa 10 ['F0.666667Ga0.222222Pa0.111111', '-3.128', '-0.2526215378']
FGaPb 4 ['F0.666667Ga0.166667Pb0.166667', '-2.814', '-0.201580481136']
FGaPd 1 ['F0.8Ga0.1Pd0.1', '-2.138', '-0.27900482911']
FGaPm 2 ['F0.777778Ga0.111111Pm0.111111', '-3.466', '-0.336449854191']
FGaPr 3 ['F0.75Ga0.125Pr0.125', '-3.886', '-0.258054809715']

FGaPt 1 ['F0.8Ga0.1Pt0.1', '-2.234', '-0.253164923069']
FGaPu 7 ['F0.666667Ga0.166667Pu0.166667', '-3.513', '-0.272783688356']
FGaRb 14 ['F0.7Ga0.2Rb0.1', '-3.07', '-0.200742778606']
FGaRe 2 ['F0.777778Ga0.111111Re0.111111', '-2.675', '-0.25214330504']
FGaRh 3 ['F0.75Ga0.125Rh0.125', '-2.549', '-0.215698043775']
FGaRu 3 ['F0.75Ga0.125Ru0.125', '-2.565', '-0.21406773315']
FGaSb 2 ['F0.777778Ga0.111111Sb0.111111', '-2.875', '-0.290181853124']
FGaSc 3 ['F0.777778Sc0.111111Ga0.111111', '-3.461', '-0.286751233911']
FGaSe 13 ['F0.2Ga0.3Se0.5', '-1.331', '-0.22153202284']
FGaSi 1 ['F0.8Si0.1Ga0.1', '-3.156', '-0.33460355561']
FGaSm 2 ['F0.777778Ga0.111111Sm0.111111', '-3.596', '-0.35413662308']
FGaSr 3 ['F0.8Ga0.1Sr0.1', '-2.675', '-0.264313257933']
FGaTa 3 ['F0.8Ga0.1Ta0.1', '-3.433', '-0.202495941194']
FGaTb 2 ['F0.777778Ga0.111111Tb0.111111', '-3.411', '-0.23197704256']
FGaTc 2 ['F0.777778Ga0.111111Tc0.111111', '-2.743', '-0.224947028594']
FGaTh 2 ['F0.75Ga0.125Th0.125', '-3.947', '-0.201858025027']
FGaTi 2 ['F0.777778Ti0.111111Ga0.111111', '-3.483', '-0.602732320716']
FGaTl 12 ['F0.75Ga0.125Tl0.125', '-2.664', '-0.2008139269']
FGaTm 48 ['F0.222222Ga0.333333Tm0.444444', '-1.571', '-0.213321413609']
FGaU 3 ['F0.777778Ga0.111111U0.111111', '-3.746', '-0.327667231693']
FGaV 2 ['F0.8V0.1Ga0.1', '-2.865', '-0.21735978661']
FGaW 1 ['F0.8Ga0.1W0.1', '-3.086', '-0.231613101447']
FGaY 4 ['F0.714286Ga0.142857Y0.142857', '-3.818', '-0.324895992284']
FGaYb 49 ['F0.3Ga0.5Yb0.2', '-1.729', '-0.20922162076']
FGaZn 1 ['F0.8Zn0.1Ga0.1', '-2.237', '-0.317975368935']
FGaZr 1 ['F0.8Ga0.1Zr0.1', '-3.431', '-0.301628758985']
FGdGe 16 ['F0.25Ge0.375Gd0.375', '-2.128', '-0.202798942823']
FGdH 28 ['H0.285714F0.428571Gd0.285714', '-2.867', '-0.200484329036']
FGdHf 1 ['F0.8Gd0.1Hf0.1', '-4.172', '-0.461946924362']
FGdHg 3 ['F0.75Gd0.125Hg0.125', '-2.907', '-0.208601470236']
FGdHo 2 ['F0.777778Gd0.111111Ho0.111111', '-4.142', '-0.320824254933']
FGdI 43 ['F0.5I0.2Gd0.3', '-3.311', '-0.2017271477']
FGdIn 4 ['F0.714286In0.142857Gd0.142857', '-3.653', '-0.220416298122']
FGdIr 1 ['F0.8Gd0.1Ir0.1', '-2.983', '-0.317385426572']
FGdK 7 ['F0.666667K0.222222Gd0.111111', '-3.515', '-0.204451657707']
FGdKr 2 ['F0.777778Kr0.111111Gd0.111111', '-2.188', '-0.237233846873']
FGdLa 2 ['F0.777778La0.111111Gd0.111111', '-4.208', '-0.370418544933']
FGdLi 7 ['Li0.222222F0.666667Gd0.111111', '-3.569', '-0.32224379076']
FGdLu 2 ['F0.777778Gd0.111111Lu0.111111', '-4.066', '-0.420862611413']
FGdMg 3 ['F0.75Mg0.125Gd0.125', '-3.793', '-0.28164750596']
FGdMn 1 ['F0.8Mn0.1Gd0.1', '-3.213', '-0.302922433107']
FGdMo 3 ['F0.75Mo0.125Gd0.125', '-3.524', '-0.219109355965']
FGdN 16 ['N0.3F0.2Gd0.5', '-2.345', '-0.206581400489']
FGdNa 18 ['F0.666667Na0.111111Gd0.222222', '-3.949', '-0.210093653173']

FGdNb 3 ['F0.75Nb0.125Gd0.125', '-3.817', '-0.213159126799']
FGdNd 2 ['F0.777778Nd0.111111Gd0.111111', '-4.084', '-0.285197049004']
FGdNi 4 ['F0.714286Ni0.142857Gd0.142857', '-3.4', '-0.200482262409']
FGdNp 1 ['F0.8Gd0.1Np0.1', '-4.249', '-0.700601263817']
FGdO 21 ['O0.25F0.375Gd0.375', '-3.859', '-0.201076120836']
FGdOs 1 ['F0.8Gd0.1Os0.1', '-3.008', '-0.205738449902']
FGdP 8 ['F0.7P0.2Gd0.1', '-3.235', '-0.203468859941']
FGdPa 2 ['F0.777778Gd0.111111Pa0.111111', '-4.274', '-0.828355494191']
FGdPb 3 ['F0.75Gd0.125Pb0.125', '-3.517', '-0.242812090965']
FGdPd 1 ['F0.8Pd0.1Gd0.1', '-2.662', '-0.289765389862']
FGdPm 2 ['F0.777778Pm0.111111Gd0.111111', '-3.916', '-0.216183810582']
FGdPr 2 ['F0.777778Pr0.111111Gd0.111111', '-4.114', '-0.318893787249']
FGdPu 6 ['F0.714286Gd0.142857Pu0.142857', '-4.094', '-0.232703683836']
FGdRb 13 ['F0.625Rb0.125Gd0.25', '-3.69', '-0.205438112211']
FGdRe 1 ['F0.8Gd0.1Re0.1', '-3.255', '-0.303269390232']
FGdRh 3 ['F0.75Rh0.125Gd0.125', '-3.189', '-0.214148744715']
FGdRu 3 ['F0.75Ru0.125Gd0.125', '-3.249', '-0.25651843409']
FGdS 5 ['F0.428571S0.285714Gd0.285714', '-3.49', '-0.213031984869']
FGdSb 8 ['F0.444444Sb0.222222Gd0.333333', '-3.095', '-0.201027802147']
FGdSc 2 ['F0.777778Sc0.111111Gd0.111111', '-4.078', '-0.333485190302']
FGdSe 26 ['F0.25Se0.25Gd0.5', '-2.488', '-0.205147876052']
FGdSi 17 ['F0.285714Si0.428571Gd0.285714', '-2.115', '-0.20714115144']
FGdSm 2 ['F0.777778Sm0.111111Gd0.111111', '-4.154', '-0.341870579471']
FGdSn 6 ['F0.8Sn0.1Gd0.1', '-3.363', '-0.200370047919']
FGdSr 3 ['F0.75Sr0.125Gd0.125', '-4.01', '-0.355092273356']
FGdTa 3 ['F0.8Gd0.1Ta0.1', '-3.959', '-0.215256501946']
FGdTb 2 ['F0.777778Gd0.111111Tb0.111111', '-3.955', '-0.205710998951']
FGdTc 3 ['F0.777778Tc0.111111Gd0.111111', '-3.349', '-0.260680984986']
FGdTe 3 ['F0.714286Te0.142857Gd0.142857', '-3.425', '-0.205522527171']
FGdTh 1 ['F0.8Gd0.1Th0.1', '-4.301', '-0.408683499027']
FGdTi 2 ['F0.777778Ti0.111111Gd0.111111', '-3.999', '-0.548466277107']
FGdTl 18 ['F0.333333Gd0.444444Tl0.222222', '-2.224', '-0.202506140014']
FGdTm 6 ['F0.666667Gd0.111111Tm0.222222', '-3.584', '-0.76868083076']
FGdU 2 ['F0.8Gd0.1U0.1', '-4.126', '-0.205411298448']
FGdV 3 ['F0.75V0.125Gd0.125', '-3.997', '-0.368667443465']
FGdY 2 ['F0.777778Y0.111111Gd0.111111', '-4.135', '-0.422360304004']
FGdYb 9 ['F0.777778Gd0.111111Yb0.111111', '-3.526', '-0.261404799747']
FGdZn 3 ['F0.75Zn0.125Gd0.125', '-3.382', '-0.341669912109']
FGdZr 1 ['F0.8Zr0.1Gd0.1', '-3.944', '-0.301389319737']
FGeH 23 ['H0.285714F0.571429Ge0.142857', '-2.12', '-0.200523050227']
FGeHf 2 ['F0.8Ge0.1Hf0.1', '-3.576', '-0.2387532477']
FGeHg 1 ['F0.8Ge0.1Hg0.1', '-2.045', '-0.259087499527']
FGeHo 1 ['F0.8Ge0.1Ho0.1', '-3.415', '-0.348748152778']
FGeI 7 ['F0.7Ge0.2I0.1', '-2.511', '-0.202276430182']

FGeIn 2 ['F0.8Ge0.1In0.1', '-2.692', '-0.29165837861']
FGeK 5 ['F0.75K0.125Ge0.125', '-2.69', '-0.208188889921']
FGeKr 2 ['F0.777778Ge0.111111Kr0.111111', '-1.74', '-0.203463095027']
FGeLa 21 ['F0.4Ge0.3La0.3', '-2.932', '-0.211560048571']
FGeLi 3 ['Li0.125F0.75Ge0.125', '-2.678', '-0.201934825126']
FGeLu 6 ['F0.5Ge0.166667Lu0.333333', '-3.163', '-0.2119341071']
FGeMg 3 ['F0.75Mg0.125Ge0.125', '-3.269', '-0.223655410133']
FGeMn 3 ['F0.714286Mn0.142857Ge0.142857', '-2.744', '-0.200592427759']
FGeMo 3 ['F0.75Ge0.125Mo0.125', '-2.706', '-0.234263683254']
FGeN 2 ['N0.111111F0.777778Ge0.111111', '-1.641', '-0.208418265389']
FGeNa 7 ['F0.666667Na0.222222Ge0.111111', '-3.169', '-0.20394024613']
FGeNb 1 ['F0.8Ge0.1Nb0.1', '-3.24', '-0.295320552069']
FGeNd 1 ['F0.8Ge0.1Nd0.1', '-3.391', '-0.344883667442']
FGeNi 18 ['F0.666667Ni0.111111Ge0.222222', '-2.412', '-0.200390007897']
FGeNp 3 ['F0.75Ge0.125Np0.125', '-3.437', '-0.21179369841']
FGeO 17 ['O0.1F0.6Ge0.3', '-2.516', '-0.200217096933']
FGeP 12 ['F0.555556P0.222222Ge0.222222', '-2.167', '-0.211705463244']
FGePa 11 ['F0.625Ge0.25Pa0.125', '-2.885', '-0.218942049604']
FGePb 1 ['F0.714286Ge0.142857Pb0.142857', '-2.804', '-0.248389948301']
FGePm 29 ['F0.25Ge0.25Pm0.5', '-1.897', '-0.200912640635']
FGePr 3 ['F0.714286Ge0.142857Pr0.142857', '-3.73', '-0.233768719193']
FGePu 8 ['F0.666667Ge0.111111Pu0.222222', '-3.701', '-0.231741543911']
FGeRb 3 ['F0.75Ge0.125Rb0.125', '-2.768', '-0.281777213671']
FGeRe 2 ['F0.777778Ge0.111111Re0.111111', '-2.513', '-0.220684448536']
FGeRh 1 ['F0.8Ge0.1Rh0.1', '-2.29', '-0.28292531911']
FGeRu 3 ['F0.75Ge0.125Ru0.125', '-2.441', '-0.219466075029']
FGeS 1 ['F0.7S0.1Ge0.2', '-2.509', '-0.213727418818']
FGeSb 1 ['F0.8Ge0.1Sb0.1', '-2.69', '-0.223230551902']
FGeSc 3 ['F0.111111Sc0.555556Ge0.333333', '-1.555', '-0.204449979726']
FGeSe 22 ['F0.375Ge0.375Se0.25', '-1.606', '-0.200695499856']
FGeSm 4 ['F0.3Ge0.4Sm0.3', '-2.376', '-0.210782210846']
FGeSn 1 ['F0.666667Ge0.111111Sn0.222222', '-2.67', '-0.203698600443']
FGeSr 10 ['F0.5Ge0.166667Sr0.333333', '-3.496', '-0.217533800433']
FGeTa 2 ['F0.8Ge0.1Ta0.1', '-3.314', '-0.24077651107']
FGeTb 1 ['F0.8Ge0.1Tb0.1', '-3.26', '-0.258446222394']
FGeTc 2 ['F0.8Ge0.1Tc0.1', '-2.641', '-0.234319209825']
FGeTe 6 ['F0.666667Ge0.166667Te0.166667', '-2.353', '-0.204127398083']
FGeTh 2 ['F0.8Ge0.1Th0.1', '-3.731', '-0.211489822365']
FGeTi 3 ['F0.75Ti0.125Ge0.125', '-3.312', '-0.268299573151']
FGeTl 13 ['F0.777778Ge0.111111Tl0.111111', '-2.554', '-0.2084644729']
FGeTm 65 ['F0.111111Ge0.444444Tm0.444444', '-1.316', '-0.213354947839']
FGeU 1 ['F0.8Ge0.1U0.1', '-3.569', '-0.309442494633']
FGeV 3 ['F0.777778V0.111111Ge0.111111', '-3.032', '-0.221044753456']
FGeY 5 ['F0.6Ge0.1Y0.3', '-3.741', '-0.203418822684']

FGeYb 61 ['F0.2Ge0.5Yb0.3', '-1.425', '-0.210395028822']
FGeZn 1 ['F0.8Zn0.1Ge0.1', '-2.314', '-0.254542253025']
FGeZr 2 ['F0.777778Ge0.111111Zr0.111111', '-3.506', '-0.203677032068']
FHHf 2 ['H0.1F0.8Hf0.1', '-2.603', '-0.230207971218']
FHHg 21 ['H0.285714F0.571429Hg0.142857', '-1.838', '-0.20039055059']
FHHo 7 ['H0.5F0.25Ho0.25', '-2.108', '-0.222446519681']
FHI 15 ['H0.142857F0.714286I0.142857', '-1.828', '-0.202653993174']
FHIn 5 ['H0.2F0.7In0.1', '-1.962', '-0.201244264496']
FHIr 8 ['H0.1F0.8Ir0.1', '-1.718', '-0.203056242138']
FHK 20 ['H0.111111F0.555556K0.333333', '-2.597', '-0.206333901809']
FHKr 8 ['H0.222222F0.666667Kr0.111111', '-1.089', '-0.263114079344']
FHLa 8 ['H0.5F0.333333La0.166667', '-2.329', '-0.210902820112']
FHLi 18 ['H0.333333Li0.111111F0.555556', '-2.068', '-0.230758512991']
FHLu 49 ['H0.222222F0.333333Lu0.444444', '-2.229', '-0.203508083137']
FHMg 10 ['H0.111111F0.666667Mg0.222222', '-3.112', '-0.202252156533']
FHMn 11 ['H0.333333F0.444444Mn0.222222', '-1.984', '-0.231165721767']
FHMo 10 ['H0.555556F0.333333Mo0.111111', '-1.298', '-0.207130410289']
FHN 10 ['H0.1N0.2F0.7', '-0.565', '-0.240131162368']
FHNa 19 ['H0.333333F0.555556Na0.111111', '-2.044', '-0.224252138453']
FHNb 10 ['H0.285714F0.571429Nb0.142857', '-2.468', '-0.200848295256']
FHNd 3 ['H0.125F0.75Nd0.125', '-2.865', '-0.2629229887']
FHNi 25 ['H0.555556F0.333333Ni0.111111', '-1.296', '-0.202015257651']
FHNp 9 ['H0.428571F0.285714Np0.285714', '-1.738', '-0.202975206275']
FHO 10 ['H0.1O0.2F0.7', '-0.595', '-0.270131162368']
FHOs 3 ['H0.166667F0.666667Os0.166667', '-1.89', '-0.20842027167']
FHP 2 ['H0.111111F0.777778P0.111111', '-2.472', '-0.20908843656']
FHPa 28 ['H0.4F0.4Pa0.2', '-2.233', '-0.21774257691']
FHPb 6 ['H0.444444F0.444444Pb0.111111', '-1.836', '-0.228161293233']
FHPd 4 ['H0.2F0.7Pd0.1', '-1.628', '-0.268157599086']
FHPm 35 ['H0.125F0.75Pm0.125', '-2.694', '-0.203283095475']
FHPr 3 ['H0.125F0.75Pr0.125', '-2.862', '-0.264081819225']
FHPt 6 ['H0.5F0.4Pt0.1', '-1.537', '-0.237524649472']
FHPu 39 ['H0.5F0.166667Pu0.333333', '-1.323', '-0.200758454886']
FHRb 23 ['H0.111111F0.555556Rb0.333333', '-2.566', '-0.205045131253']
FHRe 6 ['H0.285714F0.571429Re0.142857', '-1.876', '-0.210888620891']
FHRh 7 ['H0.5F0.4Rh0.1', '-1.51', '-0.210524649472']
FHRu 22 ['H0.428571F0.428571Ru0.142857', '-1.599', '-0.206704981577']
FHSb 12 ['H0.285714F0.428571Sb0.285714', '-1.704', '-0.216285876086']
FHSc 7 ['H0.5F0.2Sc0.3', '-1.81', '-0.201498188988']
FHSe 2 ['H0.111111F0.777778Se0.111111', '-1.854', '-0.400583188576']
FHSi 1 ['H0.1F0.8Si0.1', '-2.207', '-0.209625163218']
FHSm 5 ['H0.444444F0.222222Sm0.333333', '-1.947', '-0.20110536552']
FHSn 7 ['H0.375F0.5Sn0.125', '-1.975', '-0.20897406963']
FHSr 29 ['H0.444444F0.333333Sr0.222222', '-2.398', '-0.20496792699']

FHTa 4 ['H0.555556F0.333333Ta0.111111', '-1.607', '-0.206178536984']
FHTb 1 ['H0.1F0.8Tb0.1', '-2.293', '-0.255900945912']
FHTc 19 ['H0.555556F0.333333Tc0.111111', '-1.288', '-0.20510387456']
FHTe 24 ['H0.285714F0.571429Te0.142857', '-1.975', '-0.201797111776']
FHTh 2 ['H0.111111F0.777778Th0.111111', '-3.129', '-0.290049495426']
FHTi 16 ['H0.333333F0.5Ti0.166667', '-2.613', '-0.207415889807']
FHTl 25 ['H0.4F0.5Tl0.1', '-1.884', '-0.200076302058']
FHTm 73 ['H0.428571F0.142857Tm0.428571', '-1.367', '-0.216077394903']
FHU 23 ['H0.571429F0.285714U0.142857', '-1.637', '-0.20720464685']
FHV 16 ['H0.5F0.4V0.1', '-1.78', '-0.214595001628']
FHW 9 ['H0.3F0.5W0.2', '-1.917', '-0.211503546687']
FHY 11 ['H0.222222F0.444444Y0.333333', '-2.94', '-0.217968364651']
FHYb 63 ['H0.142857F0.714286Yb0.142857', '-2.488', '-0.20103623804']
FHZn 5 ['H0.2F0.7Zn0.1', '-1.657', '-0.237128138911']
FHZr 13 ['H0.1F0.8Zr0.1', '-2.513', '-0.207650366593']
FHfHg 1 ['F0.8Hf0.1Hg0.1', '-2.812', '-0.267487869527']
FHfHo 1 ['F0.8Ho0.1Hf0.1', '-4.234', '-0.409148522778']
FHfI 3 ['F0.75I0.125Hf0.125', '-3.368', '-0.259334066076']
FHfIn 2 ['F0.8In0.1Hf0.1', '-3.395', '-0.23605874861']
FHfK 5 ['F0.714286K0.142857Hf0.142857', '-4.128', '-0.292602120264']
FHfLi 7 ['Li0.111111F0.666667Hf0.222222', '-3.745', '-0.200689621276']
FHfLu 1 ['F0.8Lu0.1Hf0.1', '-4.176', '-0.50958304361']
FHfMg 2 ['F0.777778Mg0.111111Hf0.111111', '-3.753', '-0.203138553451']
FHfMo 1 ['F0.8Mo0.1Hf0.1', '-3.611', '-0.27958394503']
FHfN 10 ['N0.375F0.125Hf0.5', '-2.237', '-0.206089771388']
FHfNa 10 ['F0.6Na0.2Hf0.2', '-3.456', '-0.218478332854']
FHfNd 1 ['F0.8Nd0.1Hf0.1', '-4.194', '-0.389284037442']
FHfNi 1 ['F0.8Ni0.1Hf0.1', '-3.141', '-0.36692519561']
FHfNp 2 ['F0.8Hf0.1Np0.1', '-4.145', '-0.210807957155']
FHfO 1 ['O0.111111F0.777778Hf0.111111', '-2.609', '-0.333529787611']
FHfP 2 ['F0.75P0.125Hf0.125', '-3.684', '-0.26822022633']
FHfPa 3 ['F0.777778Hf0.111111Pa0.111111', '-4.235', '-0.360696264567']
FHfPb 1 ['F0.8Hf0.1Pb0.1', '-3.312', '-0.2006089062']
FHfPm 1 ['F0.8Pm0.1Hf0.1', '-4.167', '-0.451372122862']
FHfPr 2 ['F0.777778Pr0.111111Hf0.111111', '-4.46', '-0.236234557624']
FHfPu 3 ['F0.75Hf0.125Pu0.125', '-4.179', '-0.307614126747']
FHfRb 9 ['F0.6Rb0.3Hf0.1', '-3.515', '-0.204760210579']
FHfRe 1 ['F0.8Hf0.1Re0.1', '-3.301', '-0.221396228626']
FHfRh 1 ['F0.8Rh0.1Hf0.1', '-3.003', '-0.23732568911']
FHfRu 1 ['F0.8Ru0.1Hf0.1', '-3.231', '-0.2296355302']
FHfSc 1 ['F0.8Sc0.1Hf0.1', '-4.059', '-0.30314336461']
FHfSe 6 ['F0.222222Se0.444444Hf0.333333', '-2.29', '-0.215669085194']
FHfSi 2 ['F0.777778Si0.111111Hf0.111111', '-3.906', '-0.236774788319']
FHfSm 1 ['F0.8Sm0.1Hf0.1', '-4.129', '-0.312290214862']

FHfSr 1 ['F0.8Sr0.1Hf0.1', '-3.631', '-0.321280512023']
FHfTa 2 ['F0.8Hf0.1Ta0.1', '-3.96', '-0.246785917997']
FHfTh 1 ['F0.777778Hf0.111111Th0.111111', '-4.386', '-0.201412211281']
FHfTi 1 ['F0.8Ti0.1Hf0.1', '-3.958', '-0.466726342734']
FHfTl 3 ['F0.75Hf0.125Tl0.125', '-3.568', '-0.254279285654']
FHfTm 6 ['F0.714286Tm0.142857Hf0.142857', '-3.76', '-0.211783035623']
FHfU 2 ['F0.8Hf0.1U0.1', '-4.239', '-0.307440553641']
FHfY 1 ['F0.8Y0.1Hf0.1', '-4.149', '-0.421830966942']
FHfYb 10 ['F0.571429Yb0.285714Hf0.142857', '-3.276', '-0.225200810976']
FHfZn 1 ['F0.8Zn0.1Hf0.1', '-3.026', '-0.207942623025']
FHgHo 1 ['F0.8Ho0.1Hg0.1', '-2.505', '-0.231482774605']
FHgI 1 ['F0.777778I0.111111Hg0.111111', '-1.849', '-0.222531844854']
FHgIn 2 ['F0.777778In0.111111Hg0.111111', '-2.045', '-0.258770000486']
FHgIr 3 ['F0.714286Ir0.142857Hg0.142857', '-1.833', '-0.236054472767']
FHgK 11 ['F0.666667K0.111111Hg0.222222', '-2.0', '-0.2491313566']
FHgKr 3 ['F0.75Kr0.125Hg0.125', '-0.976', '-0.238314259189']
FHgLa 2 ['F0.777778La0.111111Hg0.111111', '-2.801', '-0.258464039561']
FHgLi 8 ['Li0.2F0.7Hg0.1', '-1.962', '-0.205460356849']
FHgLu 6 ['F0.571429Lu0.285714Hg0.142857', '-3.407', '-0.240918796689']
FHgMg 3 ['F0.75Mg0.125Hg0.125', '-2.357', '-0.302573687416']
FHgMn 1 ['F0.8Mn0.1Hg0.1', '-2.034', '-0.289463378272']
FHgN 3 ['N0.125F0.75Hg0.125', '-0.907', '-0.286263825846']
FHgNa 11 ['F0.625Na0.25Hg0.125', '-2.325', '-0.217265730851']
FHgNd 2 ['F0.8Nd0.1Hg0.1', '-2.468', '-0.214618289269']
FHgNi 4 ['F0.7Ni0.2Hg0.1', '-2.067', '-0.266726915611']
FHgNp 2 ['F0.777778Hg0.111111Np0.111111', '-3.184', '-0.536380232202']
FHgO 25 ['O0.3F0.1Hg0.6', '-0.76', '-0.215965758337']
FHgP 1 ['F0.8P0.1Hg0.1', '-2.481', '-0.272659491213']
FHgPa 10 ['F0.666667Hg0.166667Pa0.166667', '-3.16', '-0.309437841417']
FHgPb 4 ['F0.6Hg0.3Pb0.1', '-2.039', '-0.202319651031']
FHgPd 3 ['F0.75Pd0.125Hg0.125', '-1.779', '-0.270632918784']
FHgPm 1 ['F0.8Pm0.1Hg0.1', '-2.39', '-0.225706374689']
FHgPr 1 ['F0.8Pr0.1Hg0.1', '-2.486', '-0.235945353689']
FHgPt 2 ['F0.8Pt0.1Hg0.1', '-1.619', '-0.206681468445']
FHgPu 10 ['F0.666667Hg0.111111Pu0.222222', '-3.677', '-0.207741543911']
FHgRb 11 ['F0.5Rb0.1Hg0.4', '-1.861', '-0.203975171446']
FHgRe 1 ['F0.75Re0.125Hg0.125', '-2.208', '-0.256765187888']
FHgRh 2 ['F0.777778Rh0.111111Hg0.111111', '-1.744', '-0.221809297804']
FHgRu 3 ['F0.75Ru0.125Hg0.125', '-2.045', '-0.232462227534']
FHgSb 5 ['F0.6Sb0.1Hg0.3', '-2.067', '-0.211086302027']
FHgSc 2 ['F0.8Sc0.1Hg0.1', '-2.577', '-0.372477616437']
FHgSe 6 ['F0.555556Se0.111111Hg0.333333', '-1.657', '-0.201256623939']
FHgSi 1 ['F0.8Si0.1Hg0.1', '-2.371', '-0.201905061527']
FHgSm 2 ['F0.777778Sm0.111111Hg0.111111', '-2.724', '-0.206916074099']

FHgSn 6 ['F0.571429Sn0.142857Hg0.285714', '-2.132', '-0.200006058094']
FHgSr 3 ['F0.6Sr0.1Hg0.3', '-2.531', '-0.22984051503']
FHgTc 2 ['F0.777778Tc0.111111Hg0.111111', '-2.201', '-0.407726479613']
FHgTe 1 ['F0.555556Te0.111111Hg0.333333', '-1.853', '-0.237004647339']
FHgTh 2 ['F0.777778Hg0.111111Th0.111111', '-3.312', '-0.282249382436']
FHgTi 5 ['F0.714286Ti0.142857Hg0.142857', '-2.978', '-0.20665799223']
FHgTl 4 ['F0.6Hg0.3Tl0.1', '-1.86', '-0.21098664953']
FHgTm 49 ['F0.3Tm0.3Hg0.4', '-1.527', '-0.219513948258']
FHgU 1 ['F0.777778Hg0.111111U0.111111', '-3.315', '-0.248900301237']
FHgV 2 ['F0.777778V0.111111Hg0.111111', '-2.501', '-0.283956991697']
FHgY 7 ['F0.6Y0.1Hg0.3', '-2.699', '-0.205592784862']
FHgYb 43 ['F0.5Yb0.125Hg0.375', '-2.144', '-0.209758849813']
FHgZn 4 ['F0.714286Zn0.142857Hg0.142857', '-2.025', '-0.215395535503']
FHgZr 1 ['F0.8Zr0.1Hg0.1', '-2.699', '-0.221930264902']
FHoI 23 ['F0.166667I0.666667Ho0.166667', '-1.454', '-0.210406523352']
FHoIn 2 ['F0.777778In0.111111Ho0.111111', '-3.546', '-0.33717072632']
FHoK 7 ['F0.666667K0.222222Ho0.111111', '-3.516', '-0.214286344138']
FHoKr 1 ['F0.8Kr0.1Ho0.1', '-2.181', '-0.310512060602']
FHoLa 2 ['F0.8La0.1Ho0.1', '-3.927', '-0.358378288856']
FHoLi 5 ['Li0.142857F0.714286Ho0.142857', '-3.653', '-0.214566231449']
FHoLu 2 ['F0.777778Ho0.111111Lu0.111111', '-3.994', '-0.221308831876']
FHoMg 3 ['F0.75Mg0.125Ho0.125', '-3.875', '-0.22014950398']
FHoMo 2 ['F0.8Mo0.1Ho0.1', '-3.575', '-0.212868617028']
FHoN 12 ['N0.111111F0.777778Ho0.111111', '-2.193', '-0.218635237698']
FHoNa 7 ['F0.7Na0.2Ho0.1', '-3.173', '-0.206473237932']
FHoNb 3 ['F0.8Nb0.1Ho0.1', '-3.933', '-0.20300214136']
FHoNd 2 ['F0.8Nd0.1Ho0.1', '-3.829', '-0.29527894252']
FHoNi 3 ['F0.75Ni0.125Ho0.125', '-3.356', '-0.22715012586']
FHoNp 1 ['F0.8Ho0.1Np0.1', '-3.987', '-0.323802862233']
FHoO 5 ['O0.125F0.75Ho0.125', '-2.436', '-0.21483964241']
FHoP 3 ['F0.714286P0.142857Ho0.142857', '-3.8', '-0.283387265918']
FHoPa 2 ['F0.777778Ho0.111111Pa0.111111', '-4.252', '-0.678801714653']
FHoPb 2 ['F0.777778Ho0.111111Pb0.111111', '-3.373', '-0.217004234753']
FHoPm 1 ['F0.8Pm0.1Ho0.1', '-3.788', '-0.34336702794']
FHoPr 2 ['F0.777778Pr0.111111Ho0.111111', '-4.125', '-0.202340007711']
FHoPu 2 ['F0.777778Ho0.111111Pu0.111111', '-4.147', '-0.438006009653']
FHoRb 8 ['F0.714286Rb0.142857Ho0.142857', '-3.576', '-0.221136261449']
FHoRh 2 ['F0.8Rh0.1Ho0.1', '-2.824', '-0.329320594188']
FHoRu 1 ['F0.8Ru0.1Ho0.1', '-3.192', '-0.461630435278']
FHoSb 1 ['F0.8Sb0.1Ho0.1', '-3.263', '-0.30862582698']
FHoSc 2 ['F0.777778Sc0.111111Ho0.111111', '-4.083', '-0.210931410764']
FHoSe 12 ['F0.166667Se0.5Ho0.333333', '-2.508', '-0.208563115842']
FHoSi 1 ['F0.8Si0.1Ho0.1', '-3.815', '-0.365565714778']
FHoSm 1 ['F0.8Sm0.1Ho0.1', '-3.93', '-0.38428511994']

FHoSn 1 ['F0.8Sn0.1Ho0.1', '-3.331', '-0.224325744588']
FHoSr 2 ['F0.777778Sr0.111111Ho0.111111', '-3.647', '-0.270639352334']
FHoTc 1 ['F0.8Tc0.1Ho0.1', '-3.448', '-0.553714484903']
FHoTe 4 ['F0.2Te0.4Ho0.4', '-2.29', '-0.216449264842']
FHoTi 2 ['F0.777778Ti0.111111Ho0.111111', '-3.94', '-0.361912497569']
FHoTl 3 ['F0.777778Ho0.111111Tl0.111111', '-3.106', '-0.218681445209']
FHoTm 6 ['F0.666667Ho0.111111Tm0.222222', '-3.568', '-0.625127051222']
FHoV 1 ['F0.8V0.1Ho0.1', '-3.753', '-0.477321945778']
FHoY 1 ['F0.8Y0.1Ho0.1', '-3.827', '-0.37082587202']
FHoYb 7 ['F0.8Ho0.1Yb0.1', '-3.273', '-0.220065918188']
FHoZn 2 ['F0.777778Zn0.111111Ho0.111111', '-3.077', '-0.246930586781']
FIIn 9 ['F0.375In0.375I0.25', '-1.877', '-0.205643894093']
FIIr 11 ['F0.5I0.333333Ir0.166667', '-1.301', '-0.203476110027']
FIK 8 ['F0.3K0.3I0.4', '-1.948', '-0.200188298758']
FILa 1 ['F0.444444I0.111111La0.444444', '-3.121', '-0.208292318884']
FILu 51 ['F0.222222I0.333333Lu0.444444', '-1.792', '-0.222724906976']
FIMo 4 ['F0.714286Mo0.142857I0.142857', '-2.484', '-0.201950574888']
FINa 2 ['F0.5Na0.4I0.1', '-2.886', '-0.287298270013']
FINb 2 ['F0.8Nb0.1I0.1', '-2.819', '-0.207416093448']
FINd 3 ['F0.222222I0.555556Nd0.222222', '-2.154', '-0.221391646384']
FINi 5 ['F0.666667Ni0.166667I0.166667', '-1.901', '-0.206763186974']
FINp 4 ['F0.714286I0.142857Np0.142857', '-3.272', '-0.263751957586']
FIO 5 ['O0.166667F0.666667I0.166667', '-1.646', '-0.251071569614']
FIOs 13 ['F0.428571I0.428571Os0.142857', '-1.193', '-0.21574428662']
FIPa 51 ['F0.25I0.625Pa0.125', '-1.43', '-0.219318298032']
FIPd 2 ['F0.8Pd0.1I0.1', '-1.784', '-0.214718757452']
FIPm 37 ['F0.285714I0.285714Pm0.428571', '-2.309', '-0.209496395746']
FIPr 1 ['F0.375I0.375Pr0.25', '-3.104', '-0.241318515507']
FIPt 3 ['F0.8I0.1Pt0.1', '-1.83', '-0.268966961704']
FIPu 13 ['F0.555556I0.222222Pu0.222222', '-3.279', '-0.208998217434']
FIRb 34 ['F0.333333Rb0.166667I0.5', '-1.542', '-0.20246099674']
FIRe 7 ['F0.571429I0.285714Re0.142857', '-1.732', '-0.212002893418']
FIRh 3 ['F0.777778Rh0.111111I0.111111', '-1.951', '-0.250306125682']
FIRu 22 ['F0.375Ru0.25I0.375', '-1.121', '-0.2061807897']
FISb 8 ['F0.375Sb0.25I0.375', '-1.748', '-0.20319029379']
FISc 2 ['F0.111111Sc0.222222I0.666667', '-1.538', '-0.221429283639']
FISm 3 ['F0.111111I0.666667Sm0.222222', '-1.585', '-0.222743073563']
FISn 1 ['F0.4Sn0.2I0.4', '-1.855', '-0.239492946497']
FISr 4 ['F0.4Sr0.4I0.2', '-3.294', '-0.207311543859']
FITa 1 ['F0.75I0.125Ta0.125', '-3.09', '-0.213602010549']
FITb 34 ['F0.3I0.3Tb0.4', '-2.184', '-0.217668287416']
FITc 9 ['F0.6Tc0.2I0.2', '-1.888', '-0.211964156463']
FITe 21 ['F0.4Te0.2I0.4', '-1.508', '-0.200597597585']
FITi 5 ['F0.666667Ti0.166667I0.166667', '-3.043', '-0.271574593149']

FITl 32 ['F0.625I0.25Tl0.125', '-1.786', '-0.200686617641']
FITm 79 ['F0.2I0.7Tm0.1', '-1.021', '-0.206387204524']
FIU 2 ['F0.75I0.125U0.125', '-3.381', '-0.270871744404']
FIV 4 ['F0.714286V0.142857I0.142857', '-2.679', '-0.224350648365']
FIXe 3 ['F0.125I0.5Xe0.375', '-0.483', '-0.208619027507']
FIY 1 ['F0.7Y0.2I0.1', '-3.838', '-0.260003538189']
FIYb 42 ['F0.222222I0.333333Yb0.444444', '-2.305', '-0.204592056011']
FInIr 1 ['F0.8In0.1Ir0.1', '-2.319', '-0.20449725082']
FInK 10 ['F0.555556K0.333333In0.111111', '-3.01', '-0.219550119462']
FInKr 3 ['F0.75Kr0.125In0.125', '-1.735', '-0.229277858042']
FInLa 14 ['F0.4In0.2La0.4', '-2.818', '-0.215296620571']
FInLi 8 ['Li0.142857F0.714286In0.142857', '-2.749', '-0.237355551571']
FInLu 7 ['F0.666667In0.222222Lu0.111111', '-3.24', '-0.2072090828']
FInMg 3 ['F0.75Mg0.125In0.125', '-3.058', '-0.23553728627']
FInMo 2 ['F0.777778Mo0.111111In0.111111', '-2.922', '-0.2614323066']
FInN 19 ['N0.1F0.5In0.4', '-2.071', '-0.215598568399']
FInNa 7 ['F0.571429Na0.285714In0.142857', '-2.958', '-0.200461167396']
FInNb 4 ['F0.7Nb0.1In0.2', '-2.916', '-0.211561785191']
FInNd 2 ['F0.777778In0.111111Nd0.111111', '-3.485', '-0.298543520391']
FInNi 4 ['F0.714286Ni0.142857In0.142857', '-2.618', '-0.205784868477']
FInNp 5 ['F0.7In0.2Np0.1', '-3.209', '-0.211713088065']
FInO 4 ['O0.142857F0.714286In0.142857', '-1.865', '-0.277831342514']
FInOs 1 ['F0.8In0.1Os0.1', '-2.509', '-0.25785027415']
FInP 3 ['F0.714286P0.142857In0.142857', '-2.77', '-0.201033339219']
FInPa 5 ['F0.7In0.2Pa0.1', '-3.154', '-0.233692415607']
FInPb 2 ['F0.777778In0.111111Pb0.111111', '-2.71', '-0.293904485678']
FInPd 1 ['F0.8Pd0.1In0.1', '-2.062', '-0.24087721411']
FInPm 4 ['F0.666667In0.111111Pm0.222222', '-3.908', '-0.201989586693']
FInPr 2 ['F0.777778In0.111111Pr0.111111', '-3.56', '-0.377240258636']
FInPt 1 ['F0.8In0.1Pt0.1', '-2.3', '-0.357037308069']
FInPu 4 ['F0.714286In0.142857Pu0.142857', '-3.537', '-0.248649982762']
FInRb 9 ['F0.666667Rb0.166667In0.166667', '-3.048', '-0.202169297523']
FInRe 1 ['F0.8In0.1Re0.1', '-2.685', '-0.28438121448']
FInRh 2 ['F0.777778Rh0.111111In0.111111', '-2.33', '-0.298034244467']
FInRu 2 ['F0.777778Ru0.111111In0.111111', '-2.648', '-0.354156290122']
FInSb 2 ['F0.777778In0.111111Sb0.111111', '-2.811', '-0.268262280902']
FInSc 4 ['F0.714286Sc0.142857In0.142857', '-3.71', '-0.211982641333']
FInSe 2 ['F0.333333Se0.333333In0.333333', '-1.709', '-0.203809805289']
FInSi 1 ['F0.8Si0.1In0.1', '-3.064', '-0.28047594061']
FInSm 2 ['F0.777778In0.111111Sm0.111111', '-3.409', '-0.209217050858']
FInSn 2 ['F0.777778In0.111111Sn0.111111', '-2.958', '-0.246039967133']
FInSr 3 ['F0.75Sr0.125In0.125', '-3.192', '-0.214315546325']
FInTa 1 ['F0.777778In0.111111Ta0.111111', '-3.458', '-0.322119644008']
FInTb 1 ['F0.8In0.1Tb0.1', '-3.089', '-0.265751723304']

FInTc 1 ['F0.8Tc0.1In0.1', '-2.693', '-0.464624710735']
FInTh 2 ['F0.75In0.125Th0.125', '-3.936', '-0.22241834586']
FInTi 4 ['F0.714286Ti0.142857In0.142857', '-3.326', '-0.205958324368']
FInTl 11 ['F0.7In0.2Tl0.1', '-2.814', '-0.207515532106']
FInTm 53 ['F0.222222In0.444444Tm0.333333', '-1.388', '-0.200834270807']
FInU 2 ['F0.8In0.1U0.1', '-3.719', '-0.349523122696']
FInV 3 ['F0.75V0.125In0.125', '-3.179', '-0.239557223775']
FInY 7 ['F0.7Y0.1In0.2', '-3.361', '-0.200396744439']
FInYb 50 ['F0.5In0.4Yb0.1', '-2.217', '-0.200315497433']
FInZn 3 ['F0.777778Zn0.111111In0.111111', '-2.334', '-0.243830837706']
FInZr 1 ['F0.8Zr0.1In0.1', '-3.492', '-0.396669540108']
FIrK 3 ['F0.75K0.125Ir0.125', '-2.315', '-0.216123334556']
FIrLu 2 ['F0.8Lu0.1Ir0.1', '-2.947', '-0.32502154582']
FIrMg 1 ['F0.777778Mg0.111111Ir0.111111', '-2.645', '-0.255625778129']
FIrNa 3 ['F0.666667Na0.222222Ir0.111111', '-2.459', '-0.22956340706']
FIrNp 1 ['F0.8Ir0.1Np0.1', '-2.941', '-0.237836690655']
FIrO 2 ['O0.1F0.7Ir0.2', '-1.826', '-0.254419892397']
FIrP 2 ['F0.8P0.1Ir0.1', '-2.647', '-0.313975500796']
FIrPa 4 ['F0.714286Ir0.142857Pa0.142857', '-2.864', '-0.216668394429']
FIrPb 2 ['F0.8Ir0.1Pb0.1', '-2.226', '-0.238607918944']
FIrPd 2 ['F0.777778Pd0.111111Ir0.111111', '-1.779', '-0.202396333456']
FIrPm 1 ['F0.8Pm0.1Ir0.1', '-2.882', '-0.210810625072']
FIrPt 1 ['F0.777778Ir0.111111Pt0.111111', '-1.76', '-0.228873098875']
FIrPu 2 ['F0.777778Ir0.111111Pu0.111111', '-2.949', '-0.306710263456']
FIrRb 3 ['F0.777778Rb0.111111Ir0.111111', '-2.189', '-0.231720832062']
FIrS 9 ['F0.555556S0.222222Ir0.222222', '-1.634', '-0.202233362063']
FIrSc 2 ['F0.8Sc0.1Ir0.1', '-2.953', '-0.24158186682']
FIrTe 2 ['F0.8Te0.1Ir0.1', '-2.271', '-0.267191895862']
FIrTi 1 ['F0.8Ti0.1Ir0.1', '-2.654', '-0.207164844944']
FIrTl 4 ['F0.714286Ir0.142857Tl0.142857', '-1.987', '-0.221513762286']
FIrTm 45 ['F0.2Tm0.5Ir0.3', '-1.651', '-0.226489963172']
FIrY 2 ['F0.777778Y0.111111Ir0.111111', '-3.018', '-0.244510778269']
FIrYb 23 ['F0.666667Yb0.166667Ir0.166667', '-2.684', '-0.212172046133']
FKKr 16 ['F0.571429K0.142857Kr0.285714', '-1.25', '-0.217223742281']
FKLa 9 ['F0.571429K0.285714La0.142857', '-3.601', '-0.209146677877']
FKLi 18 ['Li0.333333F0.555556K0.111111', '-2.96', '-0.21274449316']
FKLu 21 ['F0.5K0.3Lu0.2', '-3.036', '-0.209192455265']
FKMg 20 ['F0.5Mg0.333333K0.166667', '-3.171', '-0.224788193075']
FKMn 2 ['F0.777778K0.111111Mn0.111111', '-2.387', '-0.231948430687']
FKMo 4 ['F0.75K0.125Mo0.125', '-2.95', '-0.208282515858']
FKN 13 ['N0.142857F0.571429K0.285714', '-1.893', '-0.228417427389']
FKNa 19 ['F0.6Na0.3K0.1', '-2.635', '-0.267998385592']
FKNb 2 ['F0.777778K0.111111Nb0.111111', '-3.229', '-0.247595660027']
FKNd 8 ['F0.6K0.2Nd0.2', '-3.722', '-0.208414457184']

FKNi 6 ['F0.7K0.1Ni0.2', '-2.297', '-0.210933632605']
FKNp 8 ['F0.714286K0.142857Np0.142857', '-3.735', '-0.208038925559']
FKO 19 ['O0.3F0.5K0.2', '-1.384', '-0.218792199172']
FKOs 2 ['F0.777778K0.111111Os0.111111', '-2.385', '-0.217486778727']
FKP 11 ['F0.6P0.2K0.2', '-2.875', '-0.210429030162']
FKPa 11 ['F0.666667K0.166667Pa0.166667', '-3.596', '-0.226743214743']
FKPb 7 ['F0.666667K0.222222Pb0.111111', '-2.692', '-0.215693662802']
FKPd 4 ['F0.7K0.2Pd0.1', '-2.374', '-0.280035502522']
FKPm 15 ['F0.625K0.125Pm0.25', '-3.713', '-0.205237314502']
FKPr 7 ['F0.6K0.2Pr0.2', '-3.756', '-0.200781765147']
FKPt 4 ['F0.75K0.125Pt0.125', '-2.153', '-0.244313904557']
FKPu 23 ['F0.5K0.333333Pu0.166667', '-3.014', '-0.204672384598']
FKRb 20 ['F0.5K0.125Rb0.375', '-3.121', '-0.249708883555']
FKRe 3 ['F0.777778K0.111111Re0.111111', '-2.685', '-0.225248981416']
FKRh 5 ['F0.777778K0.111111Rh0.111111', '-2.073', '-0.230916602249']
FKRu 2 ['F0.714286K0.142857Ru0.142857', '-2.528', '-0.204421151909']
FKS 9 ['F0.428571S0.142857K0.428571', '-2.698', '-0.201126141083']
FKSb 6 ['F0.555556K0.333333Sb0.111111', '-2.994', '-0.212748389183']
FKSc 11 ['F0.625K0.125Sc0.25', '-3.819', '-0.203304957419']
FKSe 2 ['F0.777778K0.111111Se0.111111', '-2.096', '-0.356210896596']
FKSi 16 ['F0.7Si0.1K0.2', '-3.261', '-0.225798122523']
FKSm 6 ['F0.6K0.3Sm0.1', '-3.769', '-0.226717159522']
FKSn 7 ['F0.666667K0.166667Sn0.166667', '-2.975', '-0.202312441103']
FKSr 12 ['F0.7K0.1Sr0.2', '-3.39', '-0.283803505932']
FKTa 4 ['F0.714286K0.142857Ta0.142857', '-3.566', '-0.2030910573']
FKTb 2 ['F0.777778K0.111111Tb0.111111', '-2.849', '-0.261431266631']
FKTc 4 ['F0.7K0.2Tc0.1', '-2.787', '-0.504434970147']
FKTe 3 ['F0.777778K0.111111Te0.111111', '-2.621', '-0.215816048639']
FKTh 6 ['F0.666667K0.166667Th0.166667', '-4.035', '-0.207349817438']
FKTi 7 ['F0.571429K0.285714Ti0.142857', '-3.289', '-0.202620306326']
FKTl 15 ['F0.555556K0.222222Tl0.222222', '-2.599', '-0.207100523812']
FKTm 28 ['F0.5K0.4Tm0.1', '-3.026', '-0.25975571443']
FKU 4 ['F0.777778K0.111111U0.111111', '-3.665', '-0.253103883454']
FKV 4 ['F0.7K0.2V0.1', '-3.107', '-0.443042431022']
FKW 2 ['F0.75K0.125W0.125', '-3.1', '-0.239874557841']
FKXe 14 ['F0.6K0.1Xe0.3', '-1.339', '-0.21809983752']
FKY 12 ['F0.5K0.375Y0.125', '-3.169', '-0.202500085184']
FKYb 26 ['F0.714286K0.142857Yb0.142857', '-2.99', '-0.249962105736']
FKZn 10 ['F0.7K0.1Zn0.2', '-2.408', '-0.243348110935']
FKZr 5 ['F0.625K0.25Zr0.125', '-3.656', '-0.240507632418']
FKrLa 2 ['F0.777778Kr0.111111La0.111111', '-2.309', '-0.214274357335']
FKrLi 13 ['Li0.3F0.6Kr0.1', '-2.192', '-0.208514290932']
FKrLu 2 ['F0.777778Kr0.111111Lu0.111111', '-2.242', '-0.339718423815']
FKrMg 3 ['F0.75Mg0.125Kr0.125', '-1.869', '-0.318360294912']

FKrMn 1 ['F0.8Mn0.1Kr0.1', '-1.605', '-0.263492664269']
FKrN 6 ['N0.111111F0.666667Kr0.222222', '-0.458', '-0.250089659275']
FKrNa 19 ['F0.555556Na0.333333Kr0.111111', '-2.306', '-0.219380702978']
FKrNd 1 ['F0.8Kr0.1Nd0.1', '-2.117', '-0.266647575266']
FKrNi 3 ['F0.75Ni0.125Kr0.125', '-1.253', '-0.228360916792']
FKrNp 2 ['F0.777778Kr0.111111Np0.111111', '-2.544', '-0.344190549977']
FKrO 13 ['O0.222222F0.555556Kr0.222222', '-0.458', '-0.250089659275']
FKrP 1 ['F0.8P0.1Kr0.1', '-2.023', '-0.21768877721']
FKrPa 6 ['F0.666667Kr0.222222Pa0.111111', '-2.094', '-0.339233721412']
FKrPb 1 ['F0.8Kr0.1Pb0.1', '-1.422', '-0.264972444024']
FKrPd 2 ['F0.777778Kr0.111111Pd0.111111', '-1.173', '-0.280039578915']
FKrPm 1 ['F0.8Kr0.1Pm0.1', '-2.078', '-0.316735660686']
FKrPr 1 ['F0.8Kr0.1Pr0.1', '-2.119', '-0.271974639686']
FKrPu 5 ['F0.7Kr0.2Pu0.1', '-1.988', '-0.239714388108']
FKrRb 16 ['F0.571429Kr0.285714Rb0.142857', '-1.222', '-0.205120270138']
FKrRh 3 ['F0.75Kr0.125Rh0.125', '-1.258', '-0.243861533667']
FKrRu 2 ['F0.777778Kr0.111111Ru0.111111', '-1.492', '-0.328665631138']
FKrSb 3 ['F0.75Kr0.125Sb0.125', '-1.927', '-0.209078796485']
FKrSc 2 ['F0.777778Sc0.111111Kr0.111111', '-2.267', '-0.265341002704']
FKrSe 1 ['F0.777778Se0.111111Kr0.111111', '-1.42', '-0.22359339336']
FKrSm 1 ['F0.8Kr0.1Sm0.1', '-2.094', '-0.231653752686']
FKrSn 1 ['F0.8Kr0.1Sn0.1', '-1.711', '-0.287694377334']
FKrSr 3 ['F0.75Kr0.125Sr0.125', '-1.976', '-0.281805062309']
FKrTc 3 ['F0.75Kr0.125Tc0.125', '-1.714', '-0.200353897061']
FKrTi 5 ['F0.7Ti0.1Kr0.2', '-1.835', '-0.204530227232']
FKrTl 3 ['F0.75Kr0.125Tl0.125', '-1.348', '-0.203977416792']
FKrTm 37 ['F0.444444Kr0.111111Tm0.444444', '-2.167', '-0.229983627049']
FKrV 2 ['F0.777778V0.111111Kr0.111111', '-2.021', '-0.251767309471']
FKrY 2 ['F0.777778Kr0.111111Y0.111111', '-2.28', '-0.310216116407']
FKrYb 10 ['F0.444444Kr0.111111Yb0.444444', '-2.487', '-0.209642723562']
FKrZn 3 ['F0.75Zn0.125Kr0.125', '-1.284', '-0.204382701061']
FLaLi 5 ['Li0.2F0.7La0.1', '-3.317', '-0.2653558711']
FLaLu 2 ['F0.8La0.1Lu0.1', '-3.962', '-0.551812809688']
FLaMg 4 ['F0.571429Mg0.285714La0.142857', '-3.582', '-0.202329749835']
FLaMn 1 ['F0.8Mn0.1La0.1', '-3.271', '-0.231358892523']
FLaMo 1 ['F0.777778Mo0.111111La0.111111', '-3.643', '-0.226126345676']
FLaN 17 ['N0.2F0.4La0.4', '-3.207', '-0.247497654169']
FLaNa 17 ['F0.625Na0.25La0.125', '-3.929', '-0.202385123665']
FLaNd 1 ['F0.8La0.1Nd0.1', '-3.952', '-0.40351380352']
FLaNi 4 ['F0.714286Ni0.142857La0.142857', '-3.587', '-0.202391490146']
FLaNp 1 ['F0.8La0.1Np0.1', '-4.126', '-0.448037723233']
FLaO 15 ['O0.444444F0.111111La0.444444', '-3.655', '-0.20640067651']
FLaP 2 ['F0.714286P0.142857La0.142857', '-3.767', '-0.229294210203']
FLaPa 2 ['F0.777778La0.111111Pa0.111111', '-4.246', '-0.656396004653']

FLaPb 2 ['F0.75La0.125Pb0.125', '-3.646', '-0.209857665235']
FLaPm 3 ['F0.8La0.1Pm0.1', '-3.803', '-0.34360188894']
FLaPr 2 ['F0.777778La0.111111Pr0.111111', '-4.207', '-0.267934297711']
FLaPu 4 ['F0.7La0.1Pu0.2', '-4.078', '-0.204751501275']
FLaRb 6 ['F0.666667Rb0.222222La0.111111', '-3.495', '-0.234282125667']
FLaRe 2 ['F0.777778La0.111111Re0.111111', '-3.338', '-0.200917771893']
FLaRh 2 ['F0.8Rh0.1La0.1', '-2.858', '-0.348555455188']
FLaRu 2 ['F0.777778Ru0.111111La0.111111', '-3.322', '-0.271850329198']
FLaS 1 ['F0.142857S0.428571La0.428571', '-2.957', '-0.204051363404']
FLaSb 25 ['F0.333333Sb0.222222La0.444444', '-2.79', '-0.204812392326']
FLaSc 2 ['F0.777778Sc0.111111La0.111111', '-4.216', '-0.327525700764']
FLaSe 2 ['F0.5Se0.25La0.25', '-3.654', '-0.226279355504']
FLaSi 41 ['F0.333333Si0.111111La0.555556', '-2.384', '-0.21444358992']
FLaSm 2 ['F0.8La0.1Sm0.1', '-3.769', '-0.20851998094']
FLaSn 2 ['F0.166667Sn0.333333La0.5', '-1.81', '-0.203506904898']
FLaSr 2 ['F0.777778Sr0.111111La0.111111', '-3.668', '-0.275233642334']
FLaTc 2 ['F0.777778Tc0.111111La0.111111', '-3.514', '-0.281721495448']
FLaTe 1 ['F0.8Te0.1La0.1', '-3.435', '-0.26077140053']
FLaTh 1 ['F0.8La0.1Th0.1', '-4.357', '-0.335119958443']
FLaTi 2 ['F0.777778Ti0.111111La0.111111', '-3.996', '-0.401506787569']
FLaTl 3 ['F0.714286La0.142857Tl0.142857', '-3.729', '-0.307933135144']
FLaTm 6 ['F0.666667La0.111111Tm0.222222', '-3.642', '-0.682721341222']
FLaU 1 ['F0.8La0.1U0.1', '-4.264', '-0.213847757864']
FLaV 2 ['F0.777778V0.111111La0.111111', '-3.989', '-0.332952007531']
FLaY 2 ['F0.777778Y0.111111La0.111111', '-4.428', '-0.571400814467']
FLaYb 8 ['F0.666667La0.166667Yb0.166667', '-4.053', '-0.212835312882']
FLaZn 2 ['F0.777778Zn0.111111La0.111111', '-3.086', '-0.239524876781']
FLaZr 1 ['F0.8Zr0.1La0.1', '-4.085', '-0.312825779153']
FLiLu 7 ['Li0.142857F0.714286Lu0.142857', '-3.655', '-0.200453215497']
FLiMg 11 ['Li0.142857F0.571429Mg0.285714', '-3.574', '-0.216280688529']
FLiMn 1 ['Li0.1F0.8Mn0.1', '-2.167', '-0.251385148701']
FLiN 6 ['Li0.222222N0.111111F0.666667', '-1.723', '-0.323054773524']
FLiNa 13 ['Li0.1F0.6Na0.3', '-2.637', '-0.222626934092']
FLiNb 5 ['Li0.2F0.6Nb0.2', '-3.129', '-0.208391497053']
FLiNd 7 ['Li0.222222F0.666667Nd0.111111', '-3.554', '-0.202062805293']
FLiNi 7 ['Li0.25F0.625Ni0.125', '-2.696', '-0.213372103665']
FLiNp 4 ['Li0.2F0.7Np0.1', '-3.501', '-0.354780444477']
FLiO 15 ['Li0.25O0.25F0.5', '-1.796', '-0.221061620215']
FLiOs 1 ['Li0.1F0.8Os0.1', '-2.28', '-0.281866649354']
FLiP 14 ['Li0.333333F0.444444P0.222222', '-2.695', '-0.203516622184']
FLiPa 7 ['Li0.166667F0.666667Pa0.166667', '-3.672', '-0.223790795577']
FLiPb 4 ['Li0.125F0.75Pb0.125', '-2.317', '-0.200195931795']
FLiPd 4 ['Li0.2F0.7Pd0.1', '-2.282', '-0.201820235018']
FLiPm 4 ['Li0.111111F0.777778Pm0.111111', '-2.84', '-0.287022180109']

FLiPr 6 ['Li0.111111F0.777778Pr0.111111', '-2.856', '-0.207732156776']
FLiPt 3 ['Li0.3F0.6Pt0.1', '-2.787', '-0.24473567002']
FLiPu 8 ['Li0.285714F0.571429Pu0.142857', '-3.492', '-0.205245370493']
FLiRb 19 ['Li0.333333F0.555556Rb0.111111', '-2.95', '-0.215108459271']
FLiRh 4 ['Li0.1F0.8Rh0.1', '-1.837', '-0.20866563752']
FLiRu 2 ['Li0.1F0.8Ru0.1', '-2.095', '-0.25010695652']
FLiSc 6 ['Li0.2F0.7Sc0.1', '-3.184', '-0.216115851932']
FLiSe 2 ['Li0.111111F0.777778Se0.111111', '-2.127', '-0.334575950484']
FLiSi 17 ['Li0.4F0.5Si0.1', '-3.15', '-0.211972092556']
FLiSm 3 ['Li0.125F0.75Sm0.125', '-3.199', '-0.200547567622']
FLiSn 3 ['Li0.111111F0.777778Sn0.111111', '-2.411', '-0.233531865273']
FLiSr 8 ['Li0.25F0.625Sr0.125', '-3.39', '-0.237816249181']
FLiTb 1 ['Li0.111111F0.777778Tb0.111111', '-2.818', '-0.215549368478']
FLiTc 4 ['Li0.2F0.7Tc0.1', '-2.765', '-0.387692067147']
FLiTe 5 ['Li0.333333F0.555556Te0.111111', '-2.978', '-0.220855108628']
FLiTh 5 ['Li0.2F0.7Th0.1', '-3.695', '-0.204862679687']
FLiTi 8 ['Li0.333333F0.555556Ti0.111111', '-3.37', '-0.200933666867']
FLiTl 8 ['Li0.111111F0.555556Tl0.333333', '-2.45', '-0.225638414368']
FLiTm 33 ['Li0.111111F0.444444Tm0.444444', '-2.5', '-0.216971012608']
FLiV 4 ['Li0.2F0.7V0.1', '-3.038', '-0.279299528022']
FLiW 2 ['Li0.1F0.8W0.1', '-2.893', '-0.21642890411']
FLiXe 2 ['Li0.125F0.75Xe0.125', '-1.5', '-0.241421713042']
FLiY 5 ['Li0.25F0.5Y0.25', '-3.307', '-0.253083727098']
FLiYb 20 ['Li0.1F0.7Yb0.2', '-3.444', '-0.208358514106']
FLiZn 5 ['Li0.25F0.625Zn0.125', '-2.748', '-0.210393887934']
FLiZr 7 ['Li0.1F0.7Zr0.2', '-3.835', '-0.211744522988']
FLuMg 11 ['F0.625Mg0.25Lu0.125', '-3.744', '-0.202247320957']
FLuMn 4 ['F0.7Mn0.1Lu0.2', '-3.861', '-0.229624756918']
FLuMo 3 ['F0.8Mo0.1Lu0.1', '-3.579', '-0.37530313786']
FLuN 10 ['N0.25F0.375Lu0.375', '-3.223', '-0.208105505865']
FLuNa 20 ['F0.666667Na0.166667Lu0.166667', '-3.918', '-0.229178327937']
FLuNb 2 ['F0.75Nb0.125Lu0.125', '-3.9', '-0.350704275859']
FLuNd 2 ['F0.8Nd0.1Lu0.1', '-3.841', '-0.465713463352']
FLuNi 8 ['F0.571429Ni0.142857Lu0.285714', '-3.437', '-0.229631929662']
FLuNp 3 ['F0.75Lu0.125Np0.125', '-4.165', '-0.28466049518']
FLuO 9 ['O0.4F0.2Lu0.4', '-4.058', '-0.213444383855']
FLuOs 2 ['F0.777778Lu0.111111Os0.111111', '-3.045', '-0.233223224747']
FLuP 8 ['F0.666667P0.111111Lu0.222222', '-3.811', '-0.214347188356']
FLuPa 3 ['F0.75Lu0.125Pa0.125', '-4.188', '-0.366195080025']
FLuPb 7 ['F0.666667Lu0.166667Pb0.166667', '-3.587', '-0.225079423912']
FLuPd 4 ['F0.7Pd0.1Lu0.2', '-3.694', '-0.255438463358']
FLuPm 2 ['F0.777778Pm0.111111Lu0.111111', '-4.074', '-0.422668387524']
FLuPr 2 ['F0.8Pr0.1Lu0.1', '-3.834', '-0.462040527772']
FLuPt 5 ['F0.714286Lu0.142857Pt0.142857', '-3.077', '-0.23046628455']

FLuPu 4 ['F0.7Lu0.1Pu0.2', '-3.936', '-0.235951161107']
FLuRb 10 ['F0.571429Rb0.285714Lu0.142857', '-3.626', '-0.236351062041']
FLuRe 2 ['F0.777778Lu0.111111Re0.111111', '-3.305', '-0.360361838373']
FLuRh 4 ['F0.75Rh0.125Lu0.125', '-3.146', '-0.225693893775']
FLuRu 4 ['F0.714286Ru0.142857Lu0.142857', '-3.228', '-0.218860937048']
FLuS 7 ['F0.777778S0.111111Lu0.111111', '-3.075', '-0.222986640195']
FLuSb 14 ['F0.5Sb0.25Lu0.25', '-3.102', '-0.2343915621']
FLuSc 2 ['F0.777778Sc0.111111Lu0.111111', '-4.025', '-0.328969767244']
FLuSe 27 ['F0.714286Se0.142857Lu0.142857', '-3.216', '-0.201575840621']
FLuSi 19 ['F0.3Si0.3Lu0.4', '-2.305', '-0.20874324926']
FLuSm 2 ['F0.777778Sm0.111111Lu0.111111', '-4.069', '-0.305355156413']
FLuSn 10 ['F0.5Sn0.125Lu0.375', '-3.137', '-0.204851932702']
FLuSr 4 ['F0.7Sr0.1Lu0.2', '-4.209', '-0.24971409444']
FLuTa 2 ['F0.8Lu0.1Ta0.1', '-3.988', '-0.287892621194']
FLuTb 1 ['F0.8Tb0.1Lu0.1', '-3.713', '-0.382276018304']
FLuTc 3 ['F0.75Tc0.125Lu0.125', '-3.336', '-0.265360391239']
FLuTe 5 ['F0.7Te0.1Lu0.2', '-3.749', '-0.216260296274']
FLuTh 1 ['F0.8Lu0.1Th0.1', '-4.271', '-0.422319618275']
FLuTi 2 ['F0.8Ti0.1Lu0.1', '-3.714', '-0.652155768644']
FLuTl 39 ['F0.5Lu0.166667Tl0.333333', '-2.901', '-0.203510391267']
FLuTm 7 ['F0.6Tm0.3Lu0.1', '-3.138', '-0.212020183018']
FLuU 3 ['F0.777778Lu0.111111U0.111111', '-4.119', '-0.227744421723']
FLuV 3 ['F0.777778V0.111111Lu0.111111', '-3.748', '-0.284396074011']
FLuW 1 ['F0.8Lu0.1W0.1', '-3.581', '-0.257009781447']
FLuXe 1 ['F0.8Xe0.1Lu0.1', '-2.232', '-0.236618957108']
FLuY 2 ['F0.777778Y0.111111Lu0.111111', '-4.21', '-0.545844880947']
FLuYb 15 ['F0.571429Yb0.285714Lu0.142857', '-3.378', '-0.222373672084']
FLuZn 4 ['F0.7Zn0.1Lu0.2', '-3.897', '-0.274945376608']
FLuZr 3 ['F0.75Zr0.125Lu0.125', '-4.084', '-0.204182047411']
FMgMn 2 ['F0.8Mg0.1Mn0.1', '-2.599', '-0.204100206851']
FMgN 3 ['N0.125F0.75Mg0.125', '-1.74', '-0.30630986157']
FMgNa 22 ['F0.4Na0.3Mg0.3', '-2.574', '-0.209003310385']
FMgNd 3 ['F0.8Mg0.1Nd0.1', '-3.256', '-0.352255117848']
FMgNi 4 ['F0.714286Mg0.142857Ni0.142857', '-2.946', '-0.270137537166']
FMgNp 2 ['F0.777778Mg0.111111Np0.111111', '-3.928', '-0.557754486179']
FMgO 10 ['O0.111111F0.666667Mg0.222222', '-2.77', '-0.22121753168']
FMgP 3 ['F0.8Mg0.1P0.1', '-3.125', '-0.266296319792']
FMgPa 4 ['F0.714286Mg0.142857Pa0.142857', '-3.905', '-0.210853883594']
FMgPb 3 ['F0.7Mg0.1Pb0.2', '-3.142', '-0.24051028569']
FMgPd 2 ['F0.777778Mg0.111111Pd0.111111', '-2.325', '-0.215202564009']
FMgPm 6 ['F0.7Mg0.1Pm0.2', '-4.137', '-0.210540079276']
FMgPr 2 ['F0.777778Mg0.111111Pr0.111111', '-3.589', '-0.366313535853']
FMgPt 2 ['F0.777778Mg0.111111Pt0.111111', '-2.408', '-0.209225841739']
FMgPu 8 ['F0.625Mg0.25Pu0.125', '-3.741', '-0.223133762623']

FMgRb 18 ['F0.5Mg0.1Rb0.4', '-3.112', '-0.208598564514']
FMgRh 2 ['F0.777778Mg0.111111Rh0.111111', '-2.458', '-0.225200139564']
FMgRu 3 ['F0.75Mg0.125Ru0.125', '-2.858', '-0.232508263257']
FMgS 1 ['F0.5Mg0.4S0.1', '-3.423', '-0.239691334822']
FMgSb 3 ['F0.75Mg0.125Sb0.125', '-3.129', '-0.223502502885']
FMgSc 3 ['F0.625Mg0.25Sc0.125', '-3.786', '-0.206486226999']
FMgSe 5 ['F0.714286Mg0.142857Se0.142857', '-2.894', '-0.202061671098']
FMgSi 3 ['F0.4Mg0.4Si0.2', '-2.552', '-0.209580553179']
FMgSm 2 ['F0.8Mg0.1Sm0.1', '-3.131', '-0.215261295268']
FMgSn 3 ['F0.777778Mg0.111111Sn0.111111', '-2.983', '-0.231113244351']
FMgSr 6 ['F0.714286Mg0.142857Sr0.142857', '-3.663', '-0.221930846327']
FMgTa 1 ['F0.8Mg0.1Ta0.1', '-3.515', '-0.28643427569']
FMgTb 2 ['F0.8Mg0.1Tb0.1', '-3.082', '-0.2228176728']
FMgTc 1 ['F0.777778Mg0.111111Tc0.111111', '-3.008', '-0.49210073359']
FMgTh 2 ['F0.777778Mg0.111111Th0.111111', '-4.034', '-0.281623636412']
FMgTi 3 ['F0.75Mg0.125Ti0.125', '-3.72', '-0.482121778925']
FMgTl 3 ['F0.666667Mg0.111111Tl0.222222', '-2.856', '-0.241490142891']
FMgTm 17 ['F0.5Mg0.166667Tm0.333333', '-2.938', '-0.202311438072']
FMgV 3 ['F0.75Mg0.125V0.125', '-3.522', '-0.214872651383']
FMgXe 1 ['F0.8Mg0.1Xe0.1', '-1.807', '-0.283160611603']
FMgY 7 ['F0.75Mg0.125Y0.125', '-3.745', '-0.212252559185']
FMgYb 32 ['F0.714286Mg0.142857Yb0.142857', '-3.663', '-0.201631562166']
FMgZn 4 ['F0.714286Mg0.142857Zn0.142857', '-2.951', '-0.212305290616']
FMgZr 2 ['F0.777778Mg0.111111Zr0.111111', '-3.689', '-0.214074548312']
FMnMo 2 ['F0.8Mn0.1Mo0.1', '-2.89', '-0.214311244815']
FMnN 2 ['N0.111111F0.777778Mn0.111111', '-1.706', '-0.319391463994']
FMnNa 2 ['F0.777778Na0.111111Mn0.111111', '-2.396', '-0.348503421774']
FMnNb 1 ['F0.8Mn0.1Nb0.1', '-3.258', '-0.22476483948']
FMnNd 1 ['F0.8Mn0.1Nd0.1', '-3.237', '-0.232259546187']
FMnNi 3 ['F0.714286Mn0.142857Ni0.142857', '-2.572', '-0.203859828889']
FMnNp 2 ['F0.777778Mn0.111111Np0.111111', '-3.568', '-0.271961733726']
FMnO 9 ['O0.333333F0.333333Mn0.333333', '-2.597', '-0.20395402093']
FMnP 4 ['F0.8P0.1Mn0.1', '-2.993', '-0.201082842584']
FMnPa 3 ['F0.75Mn0.125Pa0.125', '-3.631', '-0.482105301635']
FMnPb 1 ['F0.8Mn0.1Pb0.1', '-2.518', '-0.206584414945']
FMnPm 1 ['F0.8Mn0.1Pm0.1', '-3.165', '-0.249347631607']
FMnPr 1 ['F0.8Mn0.1Pr0.1', '-3.292', '-0.290586610607']
FMnPu 4 ['F0.75Mn0.125Pu0.125', '-3.613', '-0.31133513351']
FMnRb 2 ['F0.777778Mn0.111111Rb0.111111', '-2.474', '-0.319341574297']
FMnRe 1 ['F0.8Mn0.1Re0.1', '-2.639', '-0.269233686768']
FMnRh 1 ['F0.8Mn0.1Rh0.1', '-2.231', '-0.265301197855']
FMnS 1 ['F0.625S0.125Mn0.25', '-3.534', '-1.19782373355']
FMnSb 3 ['F0.8Mn0.1Sb0.1', '-2.754', '-0.201689711128']
FMnSc 2 ['F0.7Sc0.1Mn0.2', '-3.505', '-0.21951570535']

FMnSi 1 ['F0.8Si0.1Mn0.1', '-3.187', '-0.266546318445']
FMnSn 1 ['F0.777778Mn0.111111Sn0.111111', '-2.971', '-0.293320491898']
FMnTc 3 ['F0.75Mn0.125Tc0.125', '-2.62', '-0.222270612849']
FMnTe 11 ['F0.714286Mn0.142857Te0.142857', '-2.624', '-0.22966865008']
FMnTh 1 ['F0.8Mn0.1Th0.1', '-3.764', '-0.28586570111']
FMnTi 3 ['F0.7Ti0.1Mn0.2', '-3.345', '-0.324098683474']
FMnTl 2 ['F0.555556Mn0.111111Tl0.333333', '-2.362', '-0.204088369503']
FMnTm 30 ['F0.6Mn0.3Tm0.1', '-2.641', '-0.207958181845']
FMnV 2 ['F0.8V0.1Mn0.1', '-2.948', '-0.201302549445']
FMnY 2 ['F0.777778Mn0.111111Y0.111111', '-3.477', '-0.224562750763']
FMnYb 29 ['F0.444444Mn0.333333Yb0.222222', '-2.226', '-0.201580142419']
FMnZr 1 ['F0.8Mn0.1Zr0.1', '-3.429', '-0.20057152182']
FMoN 11 ['N0.4F0.2Mo0.4', '-1.231', '-0.256737253631']
FMoNa 1 ['F0.8Na0.1Mo0.1', '-2.741', '-0.259287432022']
FMoNb 1 ['F0.8Nb0.1Mo0.1', '-3.146', '-0.211147796692']
FMoNi 3 ['F0.75Ni0.125Mo0.125', '-2.569', '-0.210358337525']
FMoNp 2 ['F0.777778Mo0.111111Np0.111111', '-3.433', '-0.246276130628']
FMoO 15 ['O0.3F0.4Mo0.3', '-2.489', '-0.210302357084']
FMoP 3 ['F0.75P0.125Mo0.125', '-2.885', '-0.310984711091']
FMoPa 3 ['F0.75Mo0.125Pa0.125', '-3.468', '-0.44208399815']
FMoPb 1 ['F0.8Mo0.1Pb0.1', '-2.942', '-0.257769509194']
FMoPm 1 ['F0.8Mo0.1Pm0.1', '-3.606', '-0.353092217112']
FMoPr 2 ['F0.8Mo0.1Pr0.1', '-3.675', '-0.336331196112']
FMoPu 3 ['F0.75Mo0.125Pu0.125', '-3.444', '-0.265313830025']
FMoRb 5 ['F0.777778Rb0.111111Mo0.111111', '-2.972', '-0.24045593234']
FMoS 1 ['F0.777778S0.111111Mo0.111111', '-2.567', '-0.20700789486']
FMoSc 2 ['F0.8Sc0.1Mo0.1', '-3.562', '-0.26886345886']
FMoSe 1 ['F0.8Se0.1Mo0.1', '-2.593', '-0.214483523695']
FMoSi 1 ['F0.777778Si0.111111Mo0.111111', '-3.157', '-0.207790411233']
FMoSm 1 ['F0.8Mo0.1Sm0.1', '-3.587', '-0.233010309112']
FMoSr 1 ['F0.777778Sr0.111111Mo0.111111', '-3.364', '-0.200667340303']
FMoTc 1 ['F0.8Mo0.1Tc0.1', '-2.666', '-0.220408055507']
FMoTe 4 ['F0.714286Mo0.142857Te0.142857', '-2.516', '-0.250320466056']
FMoTh 1 ['F0.8Mo0.1Th0.1', '-3.76', '-0.246320519695']
FMoTl 2 ['F0.75Mo0.125Tl0.125', '-2.671', '-0.208935694608']
FMoTm 17 ['F0.666667Mo0.222222Tm0.111111', '-2.524', '-0.218408857986']
FMoU 1 ['F0.8Mo0.1U0.1', '-3.556', '-0.30427146561']
FMoV 3 ['F0.75V0.125Mo0.125', '-3.008', '-0.23010151065']
FMoY 3 ['F0.75Y0.125Mo0.125', '-3.689', '-0.36271440919']
FMoYb 15 ['F0.6Mo0.2Yb0.2', '-2.76', '-0.20798840852']
FMoZr 1 ['F0.8Zr0.1Mo0.1', '-3.5', '-0.236026340405']
FNNa 10 ['N0.1F0.6Na0.3', '-2.024', '-0.239602286006']
FNNb 4 ['N0.444444F0.222222Nb0.333333', '-1.788', '-0.202177431581']
FNNd 2 ['N0.111111F0.777778Nd0.111111', '-2.21', '-0.258008031769']

FNNp 17 ['N0.428571F0.142857Np0.428571', '-1.835', '-0.20370982005']
FNO 46 ['N0.111111O0.444444F0.444444', '-0.391', '-0.222136589211']
FNOs 1 ['N0.333333F0.444444Os0.222222', '-1.286', '-0.272549630569']
FNP 1 ['N0.1F0.8P0.1', '-1.916', '-0.204248430536']
FNPa 46 ['N0.333333F0.166667Pa0.5', '-1.93', '-0.2524106243']
FNPb 1 ['N0.1F0.8Pb0.1', '-1.374', '-0.31053209735']
FNPd 2 ['N0.111111F0.777778Pd0.111111', '-1.163', '-0.373994749278']
FNPm 3 ['N0.25F0.25Pm0.5', '-2.384', '-0.22347605055']
FNPr 1 ['N0.1F0.8Pr0.1', '-2.136', '-0.382534293012']
FNPt 1 ['N0.1F0.8Pt0.1', '-1.165', '-0.249270407768']
FNPu 9 ['N0.5F0.1Pu0.4', '-1.764', '-0.239588418629']
FNRb 13 ['N0.2F0.6Rb0.2', '-1.388', '-0.245047338172']
FNRe 15 ['N0.5F0.125Re0.375', '-0.567', '-0.203091286496']
FNRh 3 ['N0.125F0.75Rh0.125', '-1.147', '-0.249811100325']
FNRu 13 ['N0.375F0.25Ru0.375', '-0.822', '-0.212120526467']
FNSb 3 ['N0.125F0.75Sb0.125', '-1.708', '-0.236192641315']
FNSc 2 ['N0.111111F0.777778Sc0.111111', '-2.22', '-0.322296173067']
FNSi 1 ['N0.1F0.8Si0.1', '-1.889', '-0.21649400085']
FNSm 1 ['N0.1F0.8Sm0.1', '-2.022', '-0.253213406012']
FNSn 1 ['N0.1F0.8Sn0.1', '-1.645', '-0.31525403066']
FNSr 3 ['N0.125F0.75Sr0.125', '-1.907', '-0.329754628966']
FNTa 1 ['N0.1F0.8Ta0.1', '-2.295', '-0.213386386434']
FNTb 1 ['N0.1F0.8Tb0.1', '-1.967', '-0.254769783544']
FNTc 5 ['N0.5F0.166667Tc0.333333', '-0.745', '-0.20311398296']
FNTh 6 ['N0.5F0.125Th0.375', '-2.367', '-0.21256977745']
FNTi 18 ['N0.4F0.3Ti0.3', '-2.189', '-0.226834805081']
FNTl 2 ['N0.111111F0.777778Tl0.111111', '-1.276', '-0.363046207511']
FNTm 71 ['N0.222222F0.222222Tm0.555556', '-2.176', '-0.219718220119']
FNV 8 ['N0.125F0.75V0.125', '-2.077', '-0.203562789813']
FNW 17 ['N0.222222F0.444444W0.333333', '-1.797', '-0.201964538089']
FNXe 2 ['N0.111111F0.777778Xe0.111111', '-0.633', '-0.214236358164']
FNY 14 ['N0.333333F0.166667Y0.5', '-2.352', '-0.222270479938']
FNYb 38 ['N0.25F0.125Yb0.625', '-1.61', '-0.233248300334']
FNZn 3 ['N0.125F0.75Zn0.125', '-1.257', '-0.294332267719']
FNZr 1 ['N0.1F0.8Zr0.1', '-2.188', '-0.207519204225']
FNaNb 8 ['F0.5Na0.375Nb0.125', '-2.965', '-0.21266366449']
FNaNd 10 ['F0.7Na0.2Nd0.1', '-3.158', '-0.211608752596']
FNaNi 7 ['F0.666667Na0.111111Ni0.222222', '-2.352', '-0.217386470856']
FNaNp 4 ['F0.7Na0.2Np0.1', '-3.286', '-0.210132672309']
FNaO 16 ['O0.1F0.6Na0.3', '-1.986', '-0.201602286006']
FNaOs 1 ['F0.8Na0.1Os0.1', '-2.218', '-0.25504276327']
FNaP 10 ['F0.5Na0.3P0.2', '-2.745', '-0.22862265262']
FNaPa 7 ['F0.7Na0.1Pa0.2', '-3.764', '-0.23145529719']
FNaPb 8 ['F0.714286Na0.142857Pb0.142857', '-2.586', '-0.217046941931']

FNaPd 5 ['F0.714286Na0.142857Pd0.142857', '-2.069', '-0.204851480503']
FNaPm 10 ['F0.5Na0.3Pm0.2', '-3.123', '-0.226799162014']
FNaPr 15 ['F0.5Na0.3Pr0.2', '-3.182', '-0.228625148014']
FNaPt 16 ['F0.7Na0.1Pt0.2', '-1.921', '-0.203961255277']
FNaPu 25 ['F0.625Na0.25Pu0.125', '-3.645', '-0.206544023455']
FNaRb 23 ['F0.444444Na0.222222Rb0.333333', '-2.814', '-0.222276513529']
FNaRe 6 ['F0.6Na0.3Re0.1', '-2.767', '-0.208841850838']
FNaRh 6 ['F0.666667Na0.222222Rh0.111111', '-2.399', '-0.279722671404']
FNaRu 5 ['F0.7Na0.2Ru0.1', '-2.389', '-0.245960245354']
FNaS 13 ['F0.3Na0.5S0.2', '-2.409', '-0.210658753034']
FNaSb 11 ['F0.6Na0.3Sb0.1', '-3.095', '-0.212357884514']
FNaSc 23 ['F0.625Na0.25Sc0.125', '-3.847', '-0.202268193875']
FNaSe 5 ['F0.428571Na0.428571Se0.142857', '-2.775', '-0.22586040858']
FNaSi 15 ['F0.75Na0.125Si0.125', '-3.135', '-0.204817818357']
FNaSm 11 ['F0.555556Na0.222222Sm0.222222', '-3.498', '-0.210905477796']
FNaSn 6 ['F0.777778Na0.111111Sn0.111111', '-2.434', '-0.203987462961']
FNaSr 9 ['F0.714286Na0.142857Sr0.142857', '-2.853', '-0.200720664536']
FNaTa 6 ['F0.666667Na0.222222Ta0.111111', '-3.491', '-0.218446023501']
FNaTb 4 ['F0.5Na0.375Tb0.125', '-3.179', '-0.235073600651']
FNaTc 4 ['F0.7Na0.2Tc0.1', '-2.64', '-0.333044294979']
FNaTe 1 ['F0.8Na0.1Te0.1', '-2.46', '-0.283139106191']
FNaTh 6 ['F0.571429Na0.285714Th0.142857', '-3.512', '-0.202048632399']
FNaTi 12 ['F0.555556Na0.333333Ti0.111111', '-3.268', '-0.208274268438']
FNaTl 15 ['F0.666667Na0.222222Tl0.111111', '-2.454', '-0.219270123071']
FNaTm 33 ['F0.428571Na0.142857Tm0.428571', '-2.52', '-0.25754101311']
FNaV 4 ['F0.7Na0.2V0.1', '-3.016', '-0.327651755854']
FNaXe 10 ['F0.5Na0.4Xe0.1', '-2.699', '-0.212143795595']
FNaY 19 ['F0.5Na0.2Y0.3', '-3.089', '-0.220155682096']
FNaYb 40 ['F0.375Na0.375Yb0.25', '-2.435', '-0.204502857508']
FNaZn 9 ['F0.5Na0.4Zn0.1', '-2.984', '-0.211195057719']
FNaZr 8 ['F0.777778Na0.111111Zr0.111111', '-3.112', '-0.204453824972']
FNbNd 1 ['F0.777778Nb0.111111Nd0.111111', '-3.955', '-0.239667816968']
FNbNi 1 ['F0.8Ni0.1Nb0.1', '-2.948', '-0.268778814192']
FNbNp 1 ['F0.8Nb0.1Np0.1', '-3.743', '-0.269724954984']
FNbO 8 ['O0.4F0.1Nb0.5', '-2.35', '-0.210869842532']
FNbP 3 ['F0.75P0.125Nb0.125', '-3.209', '-0.339725141924']
FNbPa 3 ['F0.75Nb0.125Pa0.125', '-3.561', '-0.236133768984']
FNbPm 2 ['F0.8Nb0.1Pm0.1', '-3.835', '-0.214225741444']
FNbPu 3 ['F0.75Nb0.125Pu0.125', '-3.874', '-0.396363600859']
FNbRb 4 ['F0.555556Rb0.333333Nb0.111111', '-3.065', '-0.202751902704']
FNbSe 8 ['F0.375Se0.375Nb0.25', '-2.115', '-0.202142849657']
FNbSr 4 ['F0.714286Sr0.142857Nb0.142857', '-3.759', '-0.212229841571']
FNbTe 1 ['F0.8Nb0.1Te0.1', '-3.144', '-0.303673907693']
FNbTh 1 ['F0.8Nb0.1Th0.1', '-4.102', '-0.284807190194']

FNbTl 1 ['F0.777778Nb0.111111Tl0.111111', '-3.051', '-0.210863385672']
FNbTm 9 ['F0.666667Nb0.166667Tm0.166667', '-3.068', '-0.280325625393']
FNbU 1 ['F0.8Nb0.1U0.1', '-3.77', '-0.246934709193']
FNbY 3 ['F0.75Y0.125Nb0.125', '-3.872', '-0.246764180024']
FNbYb 14 ['F0.8Nb0.1Yb0.1', '-3.434', '-0.204924631692']
FNdNi 2 ['F0.777778Ni0.111111Nd0.111111', '-2.976', '-0.217172905947']
FNdNp 1 ['F0.8Nd0.1Np0.1', '-4.405', '-0.761938376897']
FNdO 6 ['O0.125F0.75Nd0.125', '-2.408', '-0.21200903574']
FNdP 2 ['F0.75P0.125Nd0.125', '-3.704', '-0.224195358642']
FNdPa 2 ['F0.777778Nd0.111111Pa0.111111', '-4.314', '-0.763174508724']
FNdPb 3 ['F0.75Nd0.125Pb0.125', '-3.692', '-0.299483482315']
FNdPm 1 ['F0.8Nd0.1Pm0.1', '-3.687', '-0.262502542604']
FNdPr 2 ['F0.8Pr0.1Nd0.1', '-3.853', '-0.342741521604']
FNdPu 4 ['F0.7Nd0.1Pu0.2', '-4.051', '-0.212652154939']
FNdRb 8 ['F0.555556Rb0.333333Nd0.111111', '-3.438', '-0.216060629738']
FNdRh 1 ['F0.8Rh0.1Nd0.1', '-2.78', '-0.305456108852']
FNdRu 1 ['F0.8Ru0.1Nd0.1', '-3.195', '-0.484765949942']
FNdS 1 ['F0.5S0.25Nd0.25', '-3.658', '-0.245820033858']
FNdSb 2 ['F0.3Sb0.3Nd0.4', '-2.677', '-0.203793407592']
FNdSc 2 ['F0.777778Sc0.111111Nd0.111111', '-4.059', '-0.209304204836']
FNdSe 6 ['F0.428571Se0.285714Nd0.285714', '-3.413', '-0.202728541561']
FNdSi 25 ['F0.714286Si0.142857Nd0.142857', '-3.909', '-0.204648898596']
FNdSm 2 ['F0.777778Nd0.111111Sm0.111111', '-4.144', '-0.226689594004']
FNdSn 2 ['F0.8Sn0.1Nd0.1', '-3.307', '-0.220461259252']
FNdSr 1 ['F0.8Sr0.1Nd0.1', '-3.246', '-0.227410931765']
FNdTc 1 ['F0.8Tc0.1Nd0.1', '-3.352', '-0.477849999567']
FNdTe 1 ['F0.7Te0.1Nd0.2', '-4.145', '-0.335662283938']
FNdTh 1 ['F0.8Nd0.1Th0.1', '-4.279', '-0.292020612107']
FNdTi 2 ['F0.777778Ti0.111111Nd0.111111', '-3.97', '-0.41428529164']
FNdTl 2 ['F0.777778Nd0.111111Tl0.111111', '-3.163', '-0.29805423928']
FNdTm 6 ['F0.666667Nd0.111111Tm0.222222', '-3.537', '-0.616499845293']
FNdV 1 ['F0.8V0.1Nd0.1', '-3.652', '-0.396457460442']
FNdY 2 ['F0.777778Y0.111111Nd0.111111', '-4.152', '-0.334179318538']
FNdYb 9 ['F0.666667Nd0.166667Yb0.166667', '-4.047', '-0.265003068989']
FNdZn 3 ['F0.75Zn0.125Nd0.125', '-3.402', '-0.243341303459']
FNdZr 1 ['F0.8Zr0.1Nd0.1', '-4.05', '-0.312726432817']
FNiNp 2 ['F0.777778Ni0.111111Np0.111111', '-3.43', '-0.527310594517']
FNiO 24 ['O0.222222F0.555556Ni0.222222', '-1.67', '-0.205387212297']
FNiP 6 ['F0.666667P0.111111Ni0.222222', '-2.479', '-0.256202405137']
FNiPa 8 ['F0.666667Ni0.166667Pa0.166667', '-3.087', '-0.207472605578']
FNiPb 4 ['F0.7Ni0.2Pb0.1', '-2.323', '-0.210714493608']
FNiPd 2 ['F0.777778Ni0.111111Pd0.111111', '-1.808', '-0.212159623456']
FNiPm 3 ['F0.75Ni0.125Pm0.125', '-3.244', '-0.251679625965']
FNiPr 3 ['F0.75Ni0.125Pr0.125', '-3.301', '-0.201478349715']

FNiPt 2 ['F0.777778Ni0.111111Pt0.111111', '-1.894', '-0.252276468729']
FNiPu 6 ['F0.7Ni0.1Pu0.2', '-3.641', '-0.229901123607']
FNiRb 4 ['F0.714286Ni0.142857Rb0.142857', '-2.247', '-0.201516439488']
FNiRh 2 ['F0.777778Ni0.111111Rh0.111111', '-1.955', '-0.325405391689']
FNiRu 2 ['F0.777778Ni0.111111Ru0.111111', '-2.083', '-0.216785675678']
FNiS 11 ['F0.5S0.2Ni0.3', '-1.725', '-0.202867934533']
FNiSb 2 ['F0.777778Ni0.111111Sb0.111111', '-2.53', '-0.414891666458']
FNiSc 2 ['F0.777778Sc0.111111Ni0.111111', '-3.059', '-0.354461047244']
FNiSe 9 ['F0.375Ni0.25Se0.375', '-1.339', '-0.210504386733']
FNiSi 4 ['F0.714286Si0.142857Ni0.142857', '-3.009', '-0.207182478481']
FNiSm 2 ['F0.8Ni0.1Sm0.1', '-2.798', '-0.303061792772']
FNiSn 3 ['F0.75Ni0.125Sn0.125', '-2.608', '-0.223901873542']
FNiSr 3 ['F0.7Ni0.2Sr0.1', '-2.786', '-0.217173939107']
FNiTa 1 ['F0.8Ni0.1Ta0.1', '-3.098', '-0.290234773194']
FNiTc 2 ['F0.777778Ni0.111111Tc0.111111', '-2.319', '-0.270656841928']
FNiTe 6 ['F0.714286Ni0.142857Te0.142857', '-2.322', '-0.215479712697']
FNiTh 2 ['F0.777778Ni0.111111Th0.111111', '-3.648', '-0.36317974475']
FNiTi 5 ['F0.714286Ti0.142857Ni0.142857', '-3.1', '-0.213024288654']
FNiTl 2 ['F0.777778Ni0.111111Tl0.111111', '-1.977', '-0.257211081689']
FNiTm 51 ['F0.285714Ni0.428571Tm0.285714', '-1.531', '-0.239107572974']
FNiU 2 ['F0.777778Ni0.111111U0.111111', '-3.371', '-0.219898501233']
FNiV 3 ['F0.75V0.125Ni0.125', '-2.841', '-0.24564712503']
FNiXe 1 ['F0.8Ni0.1Xe0.1', '-1.325', '-0.221961109108']
FNiY 4 ['F0.75Ni0.125Y0.125', '-3.255', '-0.248253181065']
FNiYb 33 ['F0.555556Ni0.333333Yb0.111111', '-2.261', '-0.201512969371']
FNiZn 4 ['F0.7Ni0.2Zn0.1', '-2.302', '-0.228181669109']
FNiZr 1 ['F0.8Ni0.1Zr0.1', '-3.036', '-0.329367590985']
FNpO 21 ['O0.428571F0.285714Np0.285714', '-3.937', '-0.204402395932']
FNpOs 1 ['F0.8Os0.1Np0.1', '-3.057', '-0.258625815817']
FNpP 4 ['F0.714286P0.142857Np0.142857', '-3.421', '-0.237258334875']
FNpPa 2 ['F0.777778Pa0.111111Np0.111111', '-4.043', '-0.348312197294']
FNpPb 3 ['F0.7Pb0.2Np0.1', '-3.286', '-0.203755407066']
FNpPd 2 ['F0.75Pd0.125Np0.125', '-3.112', '-0.250228919975']
FNpPm 1 ['F0.8Pm0.1Np0.1', '-4.017', '-0.463026462317']
FNpPr 1 ['F0.8Pr0.1Np0.1', '-4.141', '-0.501265441317']
FNpPt 2 ['F0.777778Pt0.111111Np0.111111', '-3.014', '-0.294679327714']
FNpPu 5 ['F0.714286Np0.142857Pu0.142857', '-3.927', '-0.281735908118']
FNpRb 5 ['F0.714286Rb0.142857Np0.142857', '-3.777', '-0.265935453416']
FNpRe 1 ['F0.8Re0.1Np0.1', '-3.13', '-0.212050568081']
FNpRh 3 ['F0.777778Rh0.111111Np0.111111', '-3.124', '-0.230644476183']
FNpRu 2 ['F0.777778Ru0.111111Np0.111111', '-3.121', '-0.211973088961']
FNpS 1 ['F0.7S0.1Np0.2', '-3.621', '-0.212641662127']
FNpSb 1 ['F0.8Sb0.1Np0.1', '-3.289', '-0.225285261357']
FNpSc 2 ['F0.777778Sc0.111111Np0.111111', '-4.461', '-0.467441893406']

FNpSe 18 ['F0.25Se0.5Np0.25', '-1.897', '-0.201081390807']
FNpSi 3 ['F0.8Si0.1Np0.1', '-3.771', '-0.212225149155']
FNpSm 2 ['F0.777778Sm0.111111Np0.111111', '-4.275', '-0.213827282574']
FNpSn 3 ['F0.8Sn0.1Np0.1', '-3.437', '-0.220985178965']
FNpSr 3 ['F0.75Sr0.125Np0.125', '-4.293', '-0.357918564348']
FNpTa 1 ['F0.8Ta0.1Np0.1', '-3.969', '-0.401608547818']
FNpTc 2 ['F0.777778Tc0.111111Np0.111111', '-3.251', '-0.224014696151']
FNpTe 4 ['F0.8Te0.1Np0.1', '-3.272', '-0.20272245532']
FNpTh 1 ['F0.75Th0.125Np0.125', '-4.679', '-0.653118530547']
FNpTi 2 ['F0.777778Ti0.111111Np0.111111', '-3.939', '-0.23942298021']
FNpTl 8 ['F0.625Tl0.25Np0.125', '-3.081', '-0.242603501114']
FNpTm 9 ['F0.666667Tm0.166667Np0.166667', '-3.508', '-0.259807454582']
FNpU 2 ['F0.777778U0.111111Np0.111111', '-3.952', '-0.208206562417']
FNpV 4 ['F0.7V0.1Np0.2', '-3.607', '-0.221932269188']
FNpXe 3 ['F0.777778Xe0.111111Np0.111111', '-2.687', '-0.262142507851']
FNpY 2 ['F0.777778Y0.111111Np0.111111', '-4.303', '-0.341317007108']
FNpYb 14 ['F0.5Yb0.4Np0.1', '-2.922', '-0.225840404643']
FNpZn 2 ['F0.8Zn0.1Np0.1', '-3.187', '-0.53059696248']
FOOs 3 ['O0.2F0.7Os0.1', '-1.584', '-0.215842001268']
FOP 3 ['O0.2F0.7P0.1', '-1.948', '-0.236248430536']
FOPa 41 ['O0.444444F0.222222Pa0.333333', '-3.335', '-0.20324419869']
FOPb 4 ['O0.142857F0.714286Pb0.142857', '-1.736', '-0.216760139071']
FOPd 5 ['O0.2F0.7Pd0.1', '-0.956', '-0.24589527435']
FOPm 8 ['O0.2F0.7Pm0.1', '-1.883', '-0.215295314012']
FOPr 8 ['O0.2F0.7Pr0.1', '-1.96', '-0.206534293012']
FOPt 1 ['O0.1F0.8Pt0.1', '-1.241', '-0.278718892432']
FOPu 25 ['O0.2F0.4Pu0.4', '-3.677', '-0.204101317181']
FORb 16 ['O0.285714F0.571429Rb0.142857', '-1.049', '-0.232605241551']
FORe 2 ['O0.333333F0.444444Re0.222222', '-2.277', '-0.243360720043']
FORh 4 ['O0.1F0.7Rh0.2', '-1.817', '-0.252850447881']
FORu 8 ['O0.142857F0.714286Ru0.142857', '-1.788', '-0.200068201571']
FOS 1 ['O0.1F0.8S0.1', '-1.627', '-0.204522612123']
FOSb 7 ['O0.125F0.75Sb0.125', '-1.974', '-0.21400567275']
FOSc 12 ['O0.3F0.4Sc0.3', '-4.08', '-0.207521138505']
FOSe 5 ['O0.142857F0.714286Se0.142857', '-1.617', '-0.212419581929']
FOSi 4 ['O0.2F0.7Si0.1', '-1.887', '-0.21449400085']
FOSm 7 ['O0.428571F0.142857Sm0.428571', '-3.726', '-0.205038268792']
FOSn 8 ['O0.222222F0.555556Sn0.222222', '-2.543', '-0.207759313254']
FOSr 5 ['O0.142857F0.714286Sr0.142857', '-2.093', '-0.290433861676']
FOTb 4 ['O0.111111F0.777778Tb0.111111', '-2.145', '-0.242521981716']
FOTc 4 ['O0.2F0.7Tc0.1', '-1.605', '-0.287446565928']
FOTe 6 ['O0.666667F0.111111Te0.222222', '-1.402', '-0.208337105302']
FOTh 10 ['O0.333333F0.333333Th0.333333', '-4.182', '-0.205054364929']
FOTi 42 ['O0.333333F0.555556Ti0.111111', '-1.822', '-0.218277259871']

FOTl 13 ['O0.1F0.5Tl0.4', '-2.043', '-0.221104732283']
FOTm 42 ['O0.4F0.4Tm0.2', '-2.724', '-0.200075098998']
FOU 1 ['O0.1F0.7U0.2', '-4.09', '-0.263579452236']
FOV 9 ['O0.222222F0.666667V0.111111', '-1.878', '-0.212722479833']
FOW 5 ['O0.375F0.375W0.25', '-2.711', '-0.207441342496']
FOXe 3 ['O0.125F0.75Xe0.125', '-0.674', '-0.202890902934']
FOY 17 ['O0.111111F0.666667Y0.222222', '-3.938', '-0.206342573538']
FOYb 30 ['O0.25F0.625Yb0.125', '-1.802', '-0.206992755325']
FOZn 8 ['O0.1F0.7Zn0.2', '-1.744', '-0.20373162835']
FOZr 1 ['O0.1F0.8Zr0.1', '-2.239', '-0.258519204225']
FOsP 1 ['F0.8P0.1Os0.1', '-2.706', '-0.31016943117']
FOsPa 2 ['F0.777778Os0.111111Pa0.111111', '-2.867', '-0.254716107524']
FOsPb 1 ['F0.8Os0.1Pb0.1', '-2.46', '-0.294524840442']
FOsPm 1 ['F0.8Pm0.1Os0.1', '-3.038', '-0.230163648402']
FOsPu 2 ['F0.777778Os0.111111Pu0.111111', '-2.958', '-0.209920402524']
FOsRb 6 ['F0.714286Rb0.142857Os0.142857', '-2.349', '-0.229597623711']
FOsS 9 ['F0.7S0.2Os0.1', '-1.95', '-0.210238735223']
FOsSc 1 ['F0.8Sc0.1Os0.1', '-3.106', '-0.25793489015']
FOsSe 2 ['F0.75Se0.125Os0.125', '-2.038', '-0.238926301382']
FOsTe 1 ['F0.8Te0.1Os0.1', '-2.346', '-0.250885974527']
FOsTl 3 ['F0.777778Os0.111111Tl0.111111', '-2.153', '-0.205659615393']
FOsTm 32 ['F0.2Tm0.5Os0.3', '-1.261', '-0.218321845175']
FOsXe 1 ['F0.8Xe0.1Os0.1', '-1.787', '-0.203523496442']
FOsY 1 ['F0.8Y0.1Os0.1', '-3.249', '-0.429622492482']
FOsYb 15 ['F0.714286Yb0.142857Os0.142857', '-2.7', '-0.225630768594']
FPPa 9 ['F0.666667P0.222222Pa0.111111', '-2.942', '-0.20199876398']
FPPb 2 ['F0.7P0.1Pb0.2', '-2.849', '-0.22731473667']
FPPm 22 ['F0.714286P0.142857Pm0.142857', '-3.58', '-0.219420980323']
FPPr 3 ['F0.714286P0.142857Pr0.142857', '-3.74', '-0.256905236038']
FPPu 6 ['F0.666667P0.166667Pu0.166667', '-3.414', '-0.241472301445']
FPRb 16 ['F0.5P0.1Rb0.4', '-2.842', '-0.213744362451']
FPRe 2 ['F0.777778P0.111111Re0.111111', '-2.708', '-0.232897933804']
FPRu 3 ['F0.75P0.125Ru0.125', '-2.689', '-0.244370801403']
FPSc 3 ['F0.75P0.125Sc0.125', '-3.669', '-0.250269517602']
FPSn 2 ['F0.75P0.125Sn0.125', '-2.892', '-0.225931084885']
FPSr 10 ['F0.7P0.2Sr0.1', '-3.187', '-0.213452133709']
FPTa 3 ['F0.8P0.1Ta0.1', '-3.351', '-0.242335444756']
FPTc 3 ['F0.75P0.125Tc0.125', '-2.732', '-0.2431364041']
FPTe 7 ['F0.6P0.2Te0.2', '-2.262', '-0.207898116643']
FPTh 1 ['F0.777778P0.111111Th0.111111', '-3.823', '-0.203847157596']
FPTi 4 ['F0.8P0.1Ti0.1', '-3.441', '-0.28589796442']
FPTl 3 ['F0.571429P0.142857Tl0.285714', '-2.439', '-0.276446422193']
FPTm 43 ['F0.3P0.4Tm0.3', '-2.277', '-0.202597521825']
FPU 2 ['F0.8P0.1U0.1', '-3.646', '-0.328683114265']

FPV 5 ['F0.7P0.1V0.2', '-3.052', '-0.21877635871']
FPW 3 ['F0.75P0.125W0.125', '-2.95', '-0.324700362541']
FPY 5 ['F0.8P0.1Y0.1', '-3.627', '-0.236002588628']
FPYb 51 ['F0.625P0.25Yb0.125', '-2.703', '-0.210750419194']
FPZn 1 ['F0.777778P0.111111Zn0.111111', '-2.978', '-0.22034916079']
FPZr 3 ['F0.777778P0.111111Zr0.111111', '-3.549', '-0.207298069496']
FPaPb 7 ['F0.7Pb0.2Pa0.1', '-3.242', '-0.208415507608']
FPaPd 10 ['F0.625Pd0.25Pa0.125', '-2.528', '-0.225377271169']
FPaPm 2 ['F0.777778Pm0.111111Pa0.111111', '-4.122', '-0.670161270302']
FPaPr 2 ['F0.777778Pr0.111111Pa0.111111', '-4.262', '-0.714871246969']
FPaPt 7 ['F0.7Pt0.2Pa0.1', '-2.459', '-0.27189015811']
FPaPu 5 ['F0.7Pa0.1Pu0.2', '-3.843', '-0.241017332607']
FPaRb 7 ['F0.6Rb0.3Pa0.1', '-3.407', '-0.226021465318']
FPaRe 2 ['F0.777778Re0.111111Pa0.111111', '-3.183', '-0.437854721151']
FPaRh 8 ['F0.666667Rh0.222222Pa0.111111', '-2.654', '-0.2576652328']
FPaRu 3 ['F0.75Ru0.125Pa0.125', '-3.086', '-0.372493076275']
FPaS 37 ['F0.5S0.2Pa0.3', '-3.145', '-0.201725504297']
FPaSb 6 ['F0.714286Sb0.142857Pa0.142857', '-3.318', '-0.270547483']
FPaSc 2 ['F0.777778Sc0.111111Pa0.111111', '-4.442', '-0.945462650022']
FPaSe 47 ['F0.5Se0.2Pa0.3', '-2.92', '-0.200676287433']
FPaSi 15 ['F0.555556Si0.222222Pa0.222222', '-2.894', '-0.206452387704']
FPaSm 2 ['F0.777778Sm0.111111Pa0.111111', '-4.265', '-0.700848039191']
FPaSn 9 ['F0.666667Sn0.166667Pa0.166667', '-3.263', '-0.229010102923']
FPaSr 3 ['F0.75Sr0.125Pa0.125', '-4.083', '-0.707066915541']
FPaTa 3 ['F0.75Ta0.125Pa0.125', '-3.606', '-0.246102076401']
FPaTb 2 ['F0.777778Tb0.111111Pa0.111111', '-4.155', '-0.653688458671']
FPaTc 3 ['F0.75Tc0.125Pa0.125', '-3.223', '-0.376789884364']
FPaTe 15 ['F0.666667Te0.222222Pa0.111111', '-2.796', '-0.211325899468']
FPaTh 1 ['F0.8Th0.1Pa0.1', '-4.432', '-0.762863212775']
FPaTi 4 ['F0.75Ti0.125Pa0.125', '-3.899', '-0.29612420393']
FPaTl 9 ['F0.6Tl0.3Pa0.1', '-2.834', '-0.241704787018']
FPaTm 7 ['F0.6Tm0.3Pa0.1', '-2.963', '-0.216563777518']
FPaU 2 ['F0.777778Pa0.111111U0.111111', '-3.896', '-0.204237304501']
FPaV 3 ['F0.75V0.125Pa0.125', '-3.799', '-0.44964208565']
FPaW 2 ['F0.777778W0.111111Pa0.111111', '-3.418', '-0.303169629566']
FPaXe 6 ['F0.666667Xe0.222222Pa0.111111', '-2.276', '-0.318302302245']
FPaY 3 ['F0.75Y0.125Pa0.125', '-4.098', '-0.20025498419']
FPaYb 12 ['F0.571429Yb0.285714Pa0.142857', '-3.205', '-0.277365538116']
FPaZn 7 ['F0.7Zn0.2Pa0.1', '-3.194', '-0.21478145761']
FPaZr 1 ['F0.8Zr0.1Pa0.1', '-4.082', '-0.662569033485']
FPbPd 2 ['F0.8Pd0.1Pb0.1', '-1.999', '-0.2254273717']
FPbPm 1 ['F0.8Pm0.1Pb0.1', '-2.937', '-0.205827411362']
FPbPr 2 ['F0.8Pr0.1Pb0.1', '-3.124', '-0.307066390362']
FPbPt 1 ['F0.8Pt0.1Pb0.1', '-2.027', '-0.2153724262']

FPbPu 9 ['F0.625Pb0.25Pu0.125', '-3.158', '-0.209895667417']
FPbRb 9 ['F0.7Rb0.1Pb0.2', '-2.689', '-0.203082783606']
FPbRe 1 ['F0.8Re0.1Pb0.1', '-2.555', '-0.210161968838']
FPbRh 1 ['F0.8Rh0.1Pb0.1', '-2.085', '-0.30378097761']
FPbRu 2 ['F0.777778Ru0.111111Pb0.111111', '-2.35', '-0.227042531789']
FPbSb 3 ['F0.6Sb0.1Pb0.3', '-2.61', '-0.218048517522']
FPbSc 3 ['F0.75Sc0.125Pb0.125', '-3.545', '-0.213557641275']
FPbSi 1 ['F0.7Si0.1Pb0.2', '-3.114', '-0.245518259611']
FPbSr 2 ['F0.8Sr0.1Pb0.1', '-2.548', '-0.222735800523']
FPbTc 2 ['F0.777778Tc0.111111Pb0.111111', '-2.587', '-0.281913698039']
FPbTh 2 ['F0.8Pb0.1Th0.1', '-3.52', '-0.226345480865']
FPbTi 3 ['F0.75Ti0.125Pb0.125', '-3.39', '-0.38928636393']
FPbTl 2 ['F0.777778Tl0.111111Pb0.111111', '-2.305', '-0.210415204567']
FPbTm 43 ['F0.375Tm0.25Pb0.375', '-1.924', '-0.228359405326']
FPbU 1 ['F0.8Pb0.1U0.1', '-3.535', '-0.249580234697']
FPbV 2 ['F0.777778V0.111111Pb0.111111', '-2.967', '-0.238144210122']
FPbY 2 ['F0.777778Y0.111111Pb0.111111', '-3.293', '-0.245540283824']
FPbYb 44 ['F0.777778Yb0.111111Pb0.111111', '-2.816', '-0.216584779567']
FPbZn 1 ['F0.8Zn0.1Pb0.1', '-2.083', '-0.21950712252']
FPdPm 1 ['F0.8Pd0.1Pm0.1', '-2.7', '-0.322190588362']
FPdPr 1 ['F0.8Pd0.1Pr0.1', '-2.773', '-0.309429567362']
FPdPt 2 ['F0.8Pd0.1Pt0.1', '-1.671', '-0.2127356032']
FPdPu 5 ['F0.666667Pd0.166667Pu0.166667', '-3.16', '-0.222099480996']
FPdRb 3 ['F0.75Rb0.125Pd0.125', '-2.028', '-0.269250242418']
FPdRh 1 ['F0.8Rh0.1Pd0.1', '-1.709', '-0.28114415461']
FPdRu 2 ['F0.75Ru0.125Pd0.125', '-1.896', '-0.200263386281']
FPdSb 1 ['F0.8Pd0.1Sb0.1', '-2.168', '-0.280449387402']
FPdSc 1 ['F0.8Sc0.1Pd0.1', '-2.672', '-0.25396183011']
FPdSe 1 ['F0.444444Se0.333333Pd0.222222', '-1.451', '-0.212520625944']
FPdSm 1 ['F0.8Pd0.1Sm0.1', '-2.725', '-0.246108680362']
FPdSr 2 ['F0.777778Sr0.111111Pd0.111111', '-2.415', '-0.223998863914']
FPdTc 1 ['F0.8Tc0.1Pd0.1', '-2.066', '-0.238538045325']
FPdTe 3 ['F0.8Pd0.1Te0.1', '-2.214', '-0.228790331864']
FPdTi 3 ['F0.75Ti0.125Pd0.125', '-2.798', '-0.241250864555']
FPdTl 7 ['F0.571429Pd0.142857Tl0.285714', '-1.942', '-0.218990341923']
FPdTm 49 ['F0.375Pd0.125Tm0.5', '-2.117', '-0.230587128658']
FPdXe 2 ['F0.777778Pd0.111111Xe0.111111', '-1.32', '-0.20199153679']
FPdY 2 ['F0.777778Y0.111111Pd0.111111', '-2.863', '-0.208166036047']
FPdYb 29 ['F0.5Pd0.25Yb0.25', '-2.561', '-0.2018074771']
FPdZn 2 ['F0.777778Zn0.111111Pd0.111111', '-1.915', '-0.216538269467']
FPmPr 1 ['F0.8Pr0.1Pm0.1', '-3.77', '-0.348829607024']
FPmPt 1 ['F0.8Pm0.1Pt0.1', '-2.738', '-0.238350682321']
FPmPu 5 ['F0.714286Pm0.142857Pu0.142857', '-4.085', '-0.215739681693']
FPmRb 11 ['F0.555556Rb0.222222Pm0.222222', '-3.341', '-0.218047391316']

FPmRh 2 ['F0.777778Rh0.111111Pm0.111111', '-2.954', '-0.303493549191']
FPmRu 2 ['F0.777778Ru0.111111Pm0.111111', '-3.13', '-0.217615594847']
FPmS 20 ['F0.375S0.125Pm0.5', '-2.765', '-0.2127818517']
FPmSb 35 ['F0.375Sb0.25Pm0.375', '-2.765', '-0.21651428314']
FPmSc 2 ['F0.777778Sc0.111111Pm0.111111', '-4.032', '-0.281290966413']
FPmSe 41 ['F0.111111Se0.333333Pm0.555556', '-1.903', '-0.204678130282']
FPmSi 30 ['F0.166667Si0.333333Pm0.5', '-1.424', '-0.205070977507']
FPmSm 1 ['F0.8Pm0.1Sm0.1', '-3.658', '-0.221508720024']
FPmSn 17 ['F0.428571Sn0.142857Pm0.428571', '-2.771', '-0.205688135824']
FPmSr 2 ['F0.8Sr0.1Pm0.1', '-3.243', '-0.313499017185']
FPmTa 2 ['F0.8Pm0.1Ta0.1', '-4.008', '-0.258681700446']
FPmTc 2 ['F0.777778Tc0.111111Pm0.111111', '-3.295', '-0.200486761097']
FPmTe 20 ['F0.4Te0.2Pm0.4', '-2.954', '-0.208370832848']
FPmTh 1 ['F0.8Pm0.1Th0.1', '-4.14', '-0.242108697527']
FPmTi 2 ['F0.777778Ti0.111111Pm0.111111', '-3.891', '-0.434272053218']
FPmTl 7 ['F0.625Pm0.25Tl0.125', '-3.717', '-0.209886043462']
FPmTm 6 ['F0.666667Pm0.111111Tm0.222222', '-3.521', '-0.699486606871']
FPmV 2 ['F0.777778V0.111111Pm0.111111', '-3.844', '-0.32571727318']
FPmY 2 ['F0.777778Y0.111111Pm0.111111', '-4.051', '-0.332166080116']
FPmYb 11 ['F0.571429Pm0.142857Yb0.285714', '-3.431', '-0.212883613991']
FPmZn 1 ['F0.8Zn0.1Pm0.1', '-2.744', '-0.306161128187']
FPrPt 2 ['F0.125Pr0.5Pt0.375', '-1.809', '-0.239622087192']
FPrPu 4 ['F0.7Pr0.1Pu0.2', '-4.075', '-0.239979219359']
FPrRb 6 ['F0.666667Rb0.222222Pr0.111111', '-3.537', '-0.318757367982']
FPrRe 2 ['F0.777778Pr0.111111Re0.111111', '-3.318', '-0.223393014209']
FPrRh 2 ['F0.777778Rh0.111111Pr0.111111', '-3.093', '-0.347203525858']
FPrRu 2 ['F0.777778Ru0.111111Pr0.111111', '-3.244', '-0.236325571513']
FPrSb 3 ['F0.3Sb0.4Pr0.3', '-2.433', '-0.200523856512']
FPrSc 2 ['F0.8Sc0.1Pr0.1', '-3.85', '-0.388600848772']
FPrSe 6 ['F0.375Se0.375Pr0.25', '-3.011', '-0.203893989702']
FPrSi 18 ['F0.166667Si0.333333Pr0.5', '-1.682', '-0.200063876118']
FPrSm 1 ['F0.8Pr0.1Sm0.1', '-3.843', '-0.320747699024']
FPrSn 1 ['F0.8Sn0.1Pr0.1', '-3.46', '-0.376788323672']
FPrSr 2 ['F0.777778Sr0.111111Pr0.111111', '-3.565', '-0.21470888465']
FPrTa 2 ['F0.8Pr0.1Ta0.1', '-4.066', '-0.230920679446']
FPrTc 1 ['F0.8Tc0.1Pr0.1', '-3.322', '-0.451177063987']
FPrTe 1 ['F0.8Te0.1Pr0.1', '-3.403', '-0.266999118614']
FPrTi 2 ['F0.777778Ti0.111111Pr0.111111', '-4.099', '-0.546982029884']
FPrTl 4 ['F0.777778Pr0.111111Tl0.111111', '-3.068', '-0.206750977524']
FPrTm 6 ['F0.666667Pr0.111111Tm0.222222', '-3.568', '-0.651196583538']
FPrV 2 ['F0.777778V0.111111Pr0.111111', '-3.913', '-0.299427249847']
FPrY 2 ['F0.8Y0.1Pr0.1', '-3.845', '-0.412288451104']
FPrYb 8 ['F0.8Pr0.1Yb0.1', '-3.308', '-0.278528497272']
FPrZn 3 ['F0.777778Zn0.111111Pr0.111111', '-3.099', '-0.295000119097']

FPrZr 1 ['F0.8Zr0.1Pr0.1', '-4.008', '-0.25754243861']
FPtPu 3 ['F0.75Pt0.125Pu0.125', '-2.966', '-0.313142426747']
FPtRb 4 ['F0.7Rb0.2Pt0.1', '-2.391', '-0.246918601518']
FPtRh 1 ['F0.8Rh0.1Pt0.1', '-1.765', '-0.215304248569']
FPtSc 2 ['F0.777778Sc0.111111Pt0.111111', '-2.95', '-0.221007649567']
FPtSr 3 ['F0.7Sr0.2Pt0.1', '-3.287', '-0.202287652984']
FPtTe 5 ['F0.714286Te0.142857Pt0.142857', '-2.181', '-0.20594975086']
FPtTi 3 ['F0.75Ti0.125Pt0.125', '-2.715', '-0.209412225652']
FPtTl 3 ['F0.75Pt0.125Tl0.125', '-1.929', '-0.200527291747']
FPtTm 55 ['F0.166667Tm0.5Pt0.333333', '-1.825', '-0.246543653477']
FPtV 2 ['F0.75V0.125Pt0.125', '-2.568', '-0.226962995344']
FPtXe 1 ['F0.777778Xe0.111111Pt0.111111', '-1.403', '-0.239374292759']
FPtY 2 ['F0.777778Y0.111111Pt0.111111', '-2.903', '-0.205882763269']
FPtYb 38 ['F0.375Yb0.25Pt0.375', '-2.211', '-0.205910314074']
FPtZn 1 ['F0.8Zn0.1Pt0.1', '-1.891', '-0.205136221943']
FPtZr 1 ['F0.777778Zr0.111111Pt0.111111', '-3.041', '-0.216999389847']
FPuRb 20 ['F0.625Rb0.25Pu0.125', '-3.599', '-0.218851291165']
FPuRe 2 ['F0.777778Re0.111111Pu0.111111', '-3.169', '-0.288059016151']
FPuRh 3 ['F0.75Rh0.125Pu0.125', '-3.073', '-0.224353218775']
FPuRu 3 ['F0.75Ru0.125Pu0.125', '-3.146', '-0.27972290815']
FPuS 44 ['F0.428571S0.142857Pu0.428571', '-2.987', '-0.20854032194']
FPuSb 15 ['F0.571429Sb0.142857Pu0.285714', '-3.298', '-0.200273559543']
FPuSc 4 ['F0.625Sc0.125Pu0.25', '-3.638', '-0.202111273667']
FPuSe 34 ['F0.6Se0.1Pu0.3', '-3.639', '-0.20747385452']
FPuSi 4 ['F0.714286Si0.142857Pu0.142857', '-3.628', '-0.203115278836']
FPuSm 3 ['F0.75Sm0.125Pu0.125', '-4.47', '-0.307558875965']
FPuSn 5 ['F0.666667Sn0.111111Pu0.222222', '-3.674', '-0.204741543911']
FPuSr 4 ['F0.7Sr0.1Pu0.2', '-4.224', '-0.360259861106']
FPuTa 3 ['F0.8Ta0.1Pu0.1', '-3.912', '-0.269220081194']
FPuTb 1 ['F0.8Tb0.1Pu0.1', '-4.016', '-0.742603478304']
FPuTc 3 ['F0.75Tc0.125Pu0.125', '-3.213', '-0.214019716239']
FPuTe 13 ['F0.571429Te0.142857Pu0.285714', '-3.414', '-0.20314994859']
FPuTh 3 ['F0.777778Th0.111111Pu0.111111', '-4.419', '-0.206385642528']
FPuTi 6 ['F0.666667Ti0.111111Pu0.222222', '-3.736', '-0.266741543911']
FPuTl 12 ['F0.571429Tl0.285714Pu0.142857', '-2.995', '-0.215550496209']
FPuTm 10 ['F0.625Tm0.25Pu0.125', '-3.299', '-0.257970408665']
FPuU 2 ['F0.8U0.1Pu0.1', '-4.205', '-0.385374877696']
FPuV 6 ['F0.7V0.1Pu0.2', '-3.798', '-0.26215533594']
FPuW 1 ['F0.8W0.1Pu0.1', '-3.549', '-0.282337241447']
FPuXe 6 ['F0.666667Xe0.222222Pu0.111111', '-2.327', '-0.233506597245']
FPuY 3 ['F0.714286Y0.142857Pu0.142857', '-4.164', '-0.278252316093']
FPuYb 22 ['F0.5Yb0.25Pu0.25', '-2.929', '-0.231524140851']
FPuZn 3 ['F0.75Zn0.125Pu0.125', '-3.541', '-0.626874386169']
FPuZr 3 ['F0.75Zr0.125Pu0.125', '-4.068', '-0.259841372411']

FRbRe 8 ['F0.8Rb0.1Re0.1', '-2.625', '-0.248082653694']
FRbRh 5 ['F0.714286Rb0.142857Rh0.142857', '-2.336', '-0.255545494844']
FRbRu 2 ['F0.777778Rb0.111111Ru0.111111', '-2.343', '-0.237544977244']
FRbS 16 ['F0.5S0.166667Rb0.333333', '-2.54', '-0.207709000681']
FRbSb 2 ['F0.5Rb0.375Sb0.125', '-2.814', '-0.214953755953']
FRbSc 11 ['F0.555556Sc0.111111Rb0.333333', '-3.398', '-0.227943012331']
FRbSe 13 ['F0.142857Se0.285714Rb0.571429', '-1.745', '-0.202977938693']
FRbSi 11 ['F0.571429Si0.142857Rb0.285714', '-3.213', '-0.223880954069']
FRbSm 6 ['F0.714286Rb0.142857Sm0.142857', '-3.584', '-0.24076725014']
FRbSn 7 ['F0.625Rb0.25Sn0.125', '-2.987', '-0.20704837906']
FRbSr 14 ['F0.666667Rb0.111111Sr0.222222', '-3.655', '-0.216034528258']
FRbTa 7 ['F0.666667Rb0.222222Ta0.111111', '-3.385', '-0.26472938591']
FRbTb 1 ['F0.8Rb0.1Tb0.1', '-2.621', '-0.30741348555']
FRbTc 6 ['F0.666667Rb0.222222Tc0.111111', '-2.736', '-0.224544565719']
FRbTe 1 ['F0.222222Rb0.444444Te0.333333', '-1.875', '-0.202735625747']
FRbTh 5 ['F0.625Rb0.25Th0.125', '-3.794', '-0.236026966403']
FRbTi 6 ['F0.571429Ti0.142857Rb0.285714', '-3.245', '-0.20237391737']
FRbTl 6 ['F0.625Rb0.125Tl0.25', '-2.452', '-0.214463509291']
FRbTm 27 ['F0.5Rb0.2Tm0.3', '-2.66', '-0.20956128643']
FRbU 3 ['F0.75Rb0.125U0.125', '-3.819', '-0.271304150026']
FRbV 5 ['F0.666667V0.111111Rb0.222222', '-3.22', '-0.284775077802']
FRbW 1 ['F0.8Rb0.1W0.1', '-3.006', '-0.38792792511']
FRbXe 8 ['F0.6Rb0.2Xe0.2', '-1.784', '-0.210410328519']
FRbY 13 ['F0.5Rb0.375Y0.125', '-3.06', '-0.206007864807']
FRbYb 12 ['F0.7Rb0.1Yb0.2', '-3.444', '-0.23009107052']
FRbZn 11 ['F0.666667Zn0.166667Rb0.166667', '-2.536', '-0.229980190158']
FRbZr 5 ['F0.666667Rb0.166667Zr0.166667', '-3.769', '-0.24303889386']
FReS 14 ['F0.4S0.4Re0.2', '-1.585', '-0.203997502335']
FReSc 2 ['F0.777778Sc0.111111Re0.111111', '-3.293', '-0.248984417262']
FReSe 4 ['F0.666667Se0.222222Re0.111111', '-1.957', '-0.201799234748']
FReSi 1 ['F0.8Si0.1Re0.1', '-2.906', '-0.201813420626']
FReSn 2 ['F0.777778Sn0.111111Re0.111111', '-2.551', '-0.203567538724']
FReTe 2 ['F0.777778Te0.111111Re0.111111', '-2.39', '-0.21581207983']
FReTi 2 ['F0.777778Ti0.111111Re0.111111', '-2.967', '-0.216965504067']
FReTl 3 ['F0.75Re0.125Tl0.125', '-2.299', '-0.206438659133']
FReTm 17 ['F0.5Tm0.3Re0.2', '-2.066', '-0.242673658146']
FReV 1 ['F0.777778V0.111111Re0.111111', '-2.727', '-0.20198866298']
FReY 2 ['F0.777778Y0.111111Re0.111111', '-3.268', '-0.255859530964']
FReYb 12 ['F0.571429Yb0.285714Re0.142857', '-2.759', '-0.204126881516']
FRhRu 1 ['F0.8Ru0.1Rh0.1', '-1.99', '-0.31880760161']
FRhS 1 ['F0.7S0.1Rh0.2', '-1.971', '-0.232638694783']
FRhSb 2 ['F0.777778Rh0.111111Sb0.111111', '-2.348', '-0.242225548124']
FRhSc 2 ['F0.777778Sc0.111111Rh0.111111', '-3.077', '-0.381794928911']
FRhSe 13 ['F0.3Se0.4Rh0.3', '-1.167', '-0.20425034476']

FRhSi 1 ['F0.8Si0.1Rh0.1', '-2.601', '-0.21074288111']
FRhSm 2 ['F0.777778Rh0.111111Sm0.111111', '-3.054', '-0.29118031808']
FRhSr 2 ['F0.777778Sr0.111111Rh0.111111', '-2.637', '-0.234476171689']
FRhTc 1 ['F0.8Tc0.1Rh0.1', '-2.159', '-0.323891651235']
FRhTe 5 ['F0.666667Rh0.222222Te0.111111', '-2.071', '-0.22487840058']
FRhTh 1 ['F0.8Rh0.1Th0.1', '-3.356', '-0.408062263775']
FRhTi 3 ['F0.75Ti0.125Rh0.125', '-2.924', '-0.22262301768']
FRhTl 3 ['F0.555556Rh0.111111Tl0.333333', '-2.068', '-0.254838012516']
FRhTm 52 ['F0.333333Rh0.444444Tm0.222222', '-1.57', '-0.275325055368']
FRhU 1 ['F0.777778Rh0.111111U0.111111', '-3.249', '-0.20793381827']
FRhV 2 ['F0.8V0.1Rh0.1', '-2.51', '-0.29349911211']
FRhXe 1 ['F0.8Rh0.1Xe0.1', '-1.372', '-0.277361602608']
FRhY 3 ['F0.75Y0.125Rh0.125', '-3.228', '-0.23175379794']
FRhYb 28 ['F0.5Rh0.25Yb0.25', '-2.477', '-0.210819800433']
FRhZn 1 ['F0.8Zn0.1Rh0.1', '-1.893', '-0.25700867552']
FRhZr 1 ['F0.8Zr0.1Rh0.1', '-2.936', '-0.237768084485']
FRuS 19 ['F0.3S0.4Ru0.3', '-1.379', '-0.204171664155']
FRuSb 1 ['F0.8Ru0.1Sb0.1', '-2.363', '-0.232112834402']
FRuSc 2 ['F0.777778Sc0.111111Ru0.111111', '-3.211', '-0.253916974567']
FRuSe 3 ['F0.714286Se0.142857Ru0.142857', '-1.983', '-0.20054052296']
FRuSm 2 ['F0.777778Ru0.111111Sm0.111111', '-3.249', '-0.224302363736']
FRuSn 1 ['F0.8Ru0.1Sn0.1', '-2.515', '-0.23181275201']
FRuSr 3 ['F0.75Sr0.125Ru0.125', '-2.985', '-0.215953030654']
FRuTb 1 ['F0.8Ru0.1Tb0.1', '-2.878', '-0.212328504894']
FRuTe 3 ['F0.75Ru0.125Te0.125', '-2.34', '-0.251359660235']
FRuTi 3 ['F0.75Ti0.125Ru0.125', '-2.932', '-0.212992707055']
FRuTl 5 ['F0.666667Ru0.111111Tl0.222222', '-2.134', '-0.22029114058']
FRuTm 39 ['F0.3Ru0.2Tm0.5', '-1.735', '-0.211086540763']
FRuXe 3 ['F0.75Ru0.125Xe0.125', '-1.683', '-0.222050270655']
FRuY 2 ['F0.777778Y0.111111Ru0.111111', '-3.255', '-0.329792088269']
FRuYb 17 ['F0.666667Ru0.166667Yb0.166667', '-2.737', '-0.203737358078']
FRuZn 2 ['F0.777778Zn0.111111Ru0.111111', '-2.197', '-0.281916150583']
FSSc 1 ['F0.428571S0.285714Sc0.285714', '-3.498', '-0.311052946654']
FSSn 1 ['F0.5S0.25Sn0.25', '-2.18', '-0.228649811387']
FSSr 9 ['F0.25S0.375Sr0.375', '-2.951', '-0.204596174023']
FSTc 17 ['F0.5S0.25Tc0.25', '-1.907', '-0.207635057715']
FSTe 35 ['F0.333333S0.222222Te0.444444', '-1.193', '-0.207159422513']
FSTh 2 ['F0.3S0.4Th0.3', '-3.246', '-0.221815847591']
FSTi 1 ['F0.777778S0.111111Ti0.111111', '-2.946', '-0.288590305888']
FSTl 3 ['F0.555556S0.111111Tl0.333333', '-2.034', '-0.225662031629']
FSTm 62 ['F0.3S0.5Tm0.2', '-2.073', '-0.234530191982']
FSV 20 ['F0.3S0.4V0.3', '-2.067', '-0.210725584656']
FSY 2 ['F0.444444S0.222222Y0.333333', '-3.62', '-0.20501181653']
FSYb 18 ['F0.625S0.125Yb0.25', '-3.306', '-0.210167442246']

FSZr 1 ['F0.1S0.4Zr0.5', '-1.923', '-0.205623045323']
FSbSc 5 ['F0.4Sc0.4Sb0.2', '-2.894', '-0.208356171347']
FSbSi 1 ['F0.8Si0.1Sb0.1', '-3.058', '-0.208048113902']
FSbSm 1 ['F0.8Sb0.1Sm0.1', '-3.255', '-0.308767519064']
FSbSr 11 ['F0.75Sr0.125Sb0.125', '-3.269', '-0.219947270281']
FSbTc 1 ['F0.8Tc0.1Sb0.1', '-2.565', '-0.270196884027']
FSbTi 2 ['F0.777778Ti0.111111Sb0.111111', '-3.153', '-0.241004052151']
FSbTl 14 ['F0.428571Sb0.285714Tl0.285714', '-1.817', '-0.201614590846']
FSbTm 62 ['F0.333333Sb0.111111Tm0.555556', '-2.021', '-0.200502137512']
FSbV 1 ['F0.8V0.1Sb0.1', '-2.925', '-0.248804344902']
FSbY 3 ['F0.777778Y0.111111Sb0.111111', '-3.419', '-0.244898079049']
FSbYb 68 ['F0.1Sb0.4Yb0.5', '-1.492', '-0.223462958659']
FSbZn 2 ['F0.777778Zn0.111111Sb0.111111', '-2.6', '-0.436022141363']
FScSe 26 ['F0.571429Sc0.285714Se0.142857', '-3.776', '-0.219172153352']
FScSi 14 ['F0.222222Si0.333333Sc0.444444', '-2.047', '-0.202420503619']
FScSm 2 ['F0.777778Sc0.111111Sm0.111111', '-4.066', '-0.202977735302']
FScSn 2 ['F0.777778Sc0.111111Sn0.111111', '-3.578', '-0.202800651578']
FScSr 3 ['F0.75Sc0.125Sr0.125', '-3.968', '-0.255837823666']
FScTa 1 ['F0.777778Sc0.111111Ta0.111111', '-4.076', '-0.327972961008']
FScTc 2 ['F0.777778Sc0.111111Tc0.111111', '-3.358', '-0.218788140817']
FScTh 1 ['F0.8Sc0.1Th0.1', '-4.27', '-0.331879939275']
FScTi 2 ['F0.777778Sc0.111111Ti0.111111', '-3.979', '-0.477573432938']
FScTl 8 ['F0.333333Sc0.444444Tl0.222222', '-2.245', '-0.214612598067']
FScTm 6 ['F0.666667Sc0.111111Tm0.222222', '-3.657', '-0.790787986591']
FScU 2 ['F0.8Sc0.1U0.1', '-4.207', '-0.240607738696']
FScV 1 ['F0.8Sc0.1V0.1', '-3.653', '-0.44631678761']
FScY 3 ['F0.75Sc0.125Y0.125', '-4.498', '-0.264025892315']
FScYb 11 ['F0.571429Sc0.142857Yb0.285714', '-3.528', '-0.258799476529']
FScZn 3 ['F0.777778Sc0.111111Zn0.111111', '-3.041', '-0.28759152215']
FSeSr 7 ['F0.5Se0.125Sr0.375', '-3.9', '-0.227362984182']
FSeTc 17 ['F0.333333Se0.444444Tc0.222222', '-1.138', '-0.206868975812']
FSeTe 16 ['F0.444444Se0.333333Te0.222222', '-1.522', '-0.207545896683']
FSeTh 2 ['F0.5Se0.25Th0.25', '-3.641', '-0.200994389393']
FSeTi 1 ['F0.8Ti0.1Se0.1', '-2.717', '-0.290443241234']
FSeTl 37 ['F0.625Se0.125Tl0.25', '-2.085', '-0.202123232106']
FSeTm 77 ['F0.125Se0.375Tm0.5', '-1.992', '-0.204933821357']
FSeU 27 ['F0.3Se0.3U0.4', '-2.219', '-0.211440427093']
FSeV 2 ['F0.75V0.125Se0.125', '-2.718', '-0.230058856906']
FSeY 9 ['F0.7Se0.1Y0.2', '-3.819', '-0.214706743021']
FSeYb 51 ['F0.2Se0.5Yb0.3', '-2.036', '-0.202455094507']
FSeZn 1 ['F0.8Zn0.1Se0.1', '-1.954', '-0.200659521525']
FSeZr 14 ['F0.375Se0.375Zr0.25', '-2.697', '-0.201627403023']
FSiSm 10 ['F0.375Si0.375Sm0.25', '-2.611', '-0.20066295439']
FSiSn 1 ['F0.666667Si0.111111Sn0.222222', '-3.02', '-0.314007184264']

FSiSr 17 ['F0.6Si0.2Sr0.2', '-3.56', '-0.200154406771']
FSiTa 2 ['F0.777778Si0.111111Ta0.111111', '-3.475', '-0.200597119852']
FSiTb 1 ['F0.8Si0.1Tb0.1', '-3.642', '-0.257263784394']
FSiTh 3 ['F0.375Si0.25Th0.375', '-2.656', '-0.200908627827']
FSiTi 2 ['F0.777778Si0.111111Ti0.111111', '-3.705', '-0.242937260816']
FSiTl 2 ['F0.5Si0.1Tl0.4', '-2.37', '-0.203227156687']
FSiTm 57 ['F0.166667Si0.5Tm0.333333', '-1.445', '-0.203634353688']
FSiU 2 ['F0.8Si0.1U0.1', '-3.793', '-0.210466463634']
FSiV 1 ['F0.8Si0.1V0.1', '-3.477', '-0.3057442327']
FSiY 9 ['F0.333333Si0.222222Y0.444444', '-2.496', '-0.21500745038']
FSiYb 53 ['F0.285714Si0.142857Yb0.571429', '-2.024', '-0.212009265964']
FSiZn 2 ['F0.777778Si0.111111Zn0.111111', '-2.955', '-0.240955350028']
FSiZr 1 ['F0.8Si0.1Zr0.1', '-3.888', '-0.235013205075']
FSmSr 1 ['F0.8Sr0.1Sm0.1', '-3.268', '-0.237417109185']
FSmTa 1 ['F0.8Sm0.1Ta0.1', '-4.107', '-0.256599792446']
FSmTc 2 ['F0.777778Tc0.111111Sm0.111111', '-3.42', '-0.213173529986']
FSmTh 1 ['F0.8Sm0.1Th0.1', '-4.235', '-0.236026789527']
FSmTi 2 ['F0.777778Ti0.111111Sm0.111111', '-4.048', '-0.478958822107']
FSmTl 1 ['F0.777778Sm0.111111Tl0.111111', '-3.089', '-0.210727769747']
FSmTm 6 ['F0.666667Sm0.111111Tm0.222222', '-3.48', '-0.54617337576']
FSmV 1 ['F0.8V0.1Sm0.1', '-3.638', '-0.370463637862']
FSmY 1 ['F0.8Y0.1Sm0.1', '-3.73', '-0.281967564104']
FSmYb 6 ['F0.8Sm0.1Yb0.1', '-3.248', '-0.203207610272']
FSmZn 1 ['F0.8Zn0.1Sm0.1', '-2.804', '-0.265079220187']
FSnSr 3 ['F0.8Sr0.1Sn0.1', '-2.828', '-0.236457733833']
FSnTe 3 ['F0.777778Sn0.111111Te0.111111', '-2.722', '-0.206425191212']
FSnTi 3 ['F0.777778Ti0.111111Sn0.111111', '-3.318', '-0.236781738382']
FSnTl 1 ['F0.555556Sn0.111111Tl0.333333', '-2.362', '-0.23285269194']
FSnTm 58 ['F0.5Sn0.4Tm0.1', '-2.27', '-0.205862353335']
FSnV 1 ['F0.777778V0.111111Sn0.111111', '-3.071', '-0.204601774362']
FSnY 4 ['F0.375Y0.375Sn0.25', '-2.676', '-0.203521246974']
FSnYb 58 ['F0.777778Sn0.111111Yb0.111111', '-3.11', '-0.214720261022']
FSnZn 1 ['F0.75Zn0.125Sn0.125', '-2.837', '-0.212149806044']
FSnZr 1 ['F0.8Zr0.1Sn0.1', '-3.52', '-0.209773234885']
FSrTa 1 ['F0.75Sr0.125Ta0.125', '-3.993', '-0.255934169897']
FSrTc 2 ['F0.8Sr0.1Tc0.1', '-2.94', '-0.560846474148']
FSrTe 1 ['F0.625Sr0.25Te0.125', '-3.965', '-0.440819041375']
FSrTh 1 ['F0.8Sr0.1Th0.1', '-3.829', '-0.337017086688']
FSrTi 3 ['F0.75Ti0.125Sr0.125', '-3.852', '-0.470566546321']
FSrTl 7 ['F0.625Sr0.125Tl0.25', '-3.032', '-0.22037746929']
FSrTm 11 ['F0.666667Sr0.222222Tm0.111111', '-3.909', '-0.620754136036']
FSrV 2 ['F0.777778V0.111111Sr0.111111', '-3.567', '-0.49972659447']
FSrXe 1 ['F0.8Sr0.1Xe0.1', '-1.856', '-0.217316425521']
FSrY 4 ['F0.7Sr0.1Y0.2', '-4.292', '-0.231460633326']

FSrYb 9 ['F0.6Sr0.2Yb0.2', '-3.774', '-0.225596631949']
FSrZn 4 ['F0.75Zn0.125Sr0.125', '-2.746', '-0.206086896685']
FSrZr 1 ['F0.8Sr0.1Zr0.1', '-3.58', '-0.337722907398']
FTaTe 2 ['F0.8Te0.1Ta0.1', '-3.176', '-0.207129866695']
FTaTh 2 ['F0.8Ta0.1Th0.1', '-4.12', '-0.224522492662']
FTaTl 3 ['F0.714286Ta0.142857Tl0.142857', '-3.222', '-0.248266266334']
FTaTm 8 ['F0.625Tm0.25Ta0.125', '-2.937', '-0.204752476877']
FTaU 1 ['F0.777778Ta0.111111U0.111111', '-3.798', '-0.287002673452']
FTaV 1 ['F0.777778V0.111111Ta0.111111', '-3.465', '-0.231157555473']
FTaY 3 ['F0.75Y0.125Ta0.125', '-3.864', '-0.20373248744']
FTaYb 11 ['F0.8Yb0.1Ta0.1', '-3.598', '-0.240380590694']
FTaZn 1 ['F0.75Zn0.125Ta0.125', '-3.332', '-0.248649116902']
FTaZr 1 ['F0.8Zr0.1Ta0.1', '-3.939', '-0.293228313372']
FTbTc 1 ['F0.8Tc0.1Tb0.1', '-3.17', '-0.340412554519']
FTbTi 2 ['F0.777778Ti0.111111Tb0.111111', '-3.846', '-0.339799241587']
FTbTm 6 ['F0.666667Tb0.111111Tm0.222222', '-3.574', '-0.70301379524']
FTbV 1 ['F0.8V0.1Tb0.1', '-3.513', '-0.302020015394']
FTbY 1 ['F0.8Y0.1Tb0.1', '-3.671', '-0.279523941636']
FTbYb 5 ['F0.714286Tb0.142857Yb0.142857', '-3.955', '-0.231520274392']
FTcTe 1 ['F0.8Tc0.1Te0.1', '-2.505', '-0.20463407799']
FTcTi 1 ['F0.8Ti0.1Tc0.1', '-3.029', '-0.468292304859']
FTcTl 4 ['F0.75Tc0.125Tl0.125', '-2.474', '-0.32348673831']
FTcTm 29 ['F0.571429Tc0.285714Tm0.142857', '-2.064', '-0.244219134739']
FTcXe 3 ['F0.75Tc0.125Xe0.125', '-1.953', '-0.287155332686']
FTcY 3 ['F0.75Y0.125Tc0.125', '-3.37', '-0.223420295404']
FTcYb 15 ['F0.444444Tc0.111111Yb0.444444', '-2.535', '-0.257642723562']
FTcZn 2 ['F0.777778Zn0.111111Tc0.111111', '-2.472', '-0.374787316833']
FTeTi 3 ['F0.777778Ti0.111111Te0.111111', '-3.146', '-0.227823156554']
FTeTl 8 ['F0.5Te0.166667Tl0.333333', '-2.013', '-0.205248700917']
FTeTm 56 ['F0.166667Te0.333333Tm0.5', '-1.704', '-0.201832365697']
FTeU 5 ['F0.428571Te0.285714U0.285714', '-2.519', '-0.202159536562']
FTeV 5 ['F0.666667V0.166667Te0.166667', '-2.778', '-0.217519443356']
FTeY 13 ['F0.2Y0.4Te0.4', '-2.254', '-0.203347616727']
FTeYb 51 ['F0.8Te0.1Yb0.1', '-2.861', '-0.202459029862']
FTeZr 1 ['F0.8Zr0.1Te0.1', '-3.394', '-0.23051051124']
FThTi 1 ['F0.8Ti0.1Th0.1', '-4.279', '-0.605462917399']
FThTl 5 ['F0.714286Tl0.142857Th0.142857', '-3.954', '-0.21880719443']
FThTm 4 ['F0.7Tm0.2Th0.1', '-3.825', '-0.723156015687']
FThV 1 ['F0.8V0.1Th0.1', '-4.03', '-0.301063615365']
FThY 1 ['F0.8Y0.1Th0.1', '-4.376', '-0.466567541607']
FThYb 5 ['F0.8Yb0.1Th0.1', '-3.858', '-0.351807587775']
FThZn 1 ['F0.8Zn0.1Th0.1', '-3.331', '-0.33067919769']
FTiTl 9 ['F0.571429Ti0.142857Tl0.285714', '-2.86', '-0.209050858528']
FTiTm 9 ['F0.625Ti0.125Tm0.25', '-3.262', '-0.36824020757']

FTiU 6 ['F0.714286Ti0.142857U0.142857', '-3.657', '-0.249382917998']
FTiV 2 ['F0.777778Ti0.111111V0.111111', '-3.504', '-0.234999739704']
FTiXe 3 ['F0.75Ti0.125Xe0.125', '-2.433', '-0.258683303306']
FTiY 2 ['F0.777778Ti0.111111Y0.111111', '-4.16', '-0.69044854664']
FTiYb 19 ['F0.5Ti0.166667Yb0.333333', '-2.824', '-0.203768875087']
FTiZn 3 ['F0.75Ti0.125Zn0.125', '-3.189', '-0.422144185074']
FTiZr 1 ['F0.8Ti0.1Zr0.1', '-3.807', '-0.383168738109']
FTlTm 56 ['F0.3Tm0.2Tl0.5', '-1.583', '-0.28297138576']
FTlU 2 ['F0.777778Tl0.111111U0.111111', '-3.394', '-0.202276720121']
FTlV 5 ['F0.714286V0.142857Tl0.142857', '-2.894', '-0.203716977766']
FTlY 31 ['F0.625Y0.25Tl0.125', '-3.738', '-0.201069513942']
FTlYb 52 ['F0.333333Yb0.166667Tl0.5', '-1.704', '-0.207772585011']
FTlZn 15 ['F0.7Zn0.1Tl0.2', '-2.4', '-0.205154020608']
FTlZr 2 ['F0.75Zr0.125Tl0.125', '-3.444', '-0.210302926901']
FTmU 5 ['F0.714286Tm0.142857U0.142857', '-3.734', '-0.347933389403']
FTmV 13 ['F0.666667V0.222222Tm0.111111', '-3.084', '-0.267290774035']
FTmW 14 ['F0.666667Tm0.166667W0.166667', '-2.648', '-0.203602800305']
FTmXe 39 ['F0.428571Xe0.142857Tm0.428571', '-2.082', '-0.214162783226']
FTmY 6 ['F0.666667Y0.111111Tm0.222222', '-3.554', '-0.719663100293']
FTmYb 28 ['F0.5Tm0.4Yb0.1', '-2.598', '-0.257964936674']
FTmZn 44 ['F0.333333Zn0.166667Tm0.5', '-1.883', '-0.201407295375']
FTmZr 4 ['F0.7Zr0.1Tm0.2', '-3.67', '-0.817861836397']
FUV 2 ['F0.8V0.1U0.1', '-3.745', '-0.246005022196']
FUY 1 ['F0.8Y0.1U0.1', '-4.175', '-0.237295341028']
FUYb 10 ['F0.555556Yb0.333333U0.111111', '-3.088', '-0.23450761252']
FUZr 1 ['F0.8Zr0.1U0.1', '-4.077', '-0.212882949016']
FVY 3 ['F0.75V0.125Y0.125', '-3.89', '-0.24027249669']
FVYb 18 ['F0.444444V0.111111Yb0.444444', '-2.528', '-0.203662206067']
FVZn 2 ['F0.777778V0.111111Zn0.111111', '-2.892', '-0.371017828917']
FVZr 1 ['F0.8V0.1Zr0.1', '-3.778', '-0.298769436075']
FWYb 12 ['F0.5Yb0.375W0.125', '-2.668', '-0.269229542667']
FWZr 1 ['F0.8Zr0.1W0.1', '-3.618', '-0.273122041574']
FXeY 1 ['F0.8Y0.1Xe0.1', '-2.275', '-0.218866880439']
FXeYb 8 ['F0.5Xe0.2Yb0.3', '-2.502', '-0.201183429863']
FYYb 11 ['F0.7Y0.2Yb0.1', '-4.095', '-0.224102928985']
FYZn 5 ['F0.75Zn0.125Y0.125', '-3.321', '-0.259274965334']
FYZr 1 ['F0.8Y0.1Zr0.1', '-3.936', '-0.276273362317']
FYbZn 40 ['F0.5Zn0.375Yb0.125', '-2.326', '-0.22299798706']
FYbZr 10 ['F0.8Zr0.1Yb0.1', '-3.481', '-0.224513408485']
FZnZr 1 ['F0.8Zn0.1Zr0.1', '-3.008', '-0.2573850184']
FeGaN 14 ['N0.375Fe0.375Ga0.25', '-0.554', '-0.200944993621']
FeGdH 1 ['H0.625Fe0.125Gd0.25', '-0.754', '-0.200464479676']
FeGdI 37 ['Fe0.166667I0.333333Gd0.5', '-0.832', '-0.203108904606']
FeGdN 2 ['N0.4Fe0.1Gd0.5', '-1.616', '-0.237231123614']

FeGdSi 1 ['Si0.5Fe0.375Gd0.125', '-0.884', '-0.261544990469']
FeGeHo 1 ['Fe0.1Ge0.4Ho0.5', '-0.99', '-0.220220035333']
FeGePa 2 ['Fe0.444444Ge0.444444Pa0.111111', '-0.377', '-0.209394853518']
FeGeSc 1 ['Sc0.5Fe0.125Ge0.375', '-0.976', '-0.24072170375']
FeGeYb 1 ['Fe0.375Ge0.5Yb0.125', '-0.516', '-0.230654461833']
FeHI 4 ['H0.5Fe0.2I0.3', '-0.304', '-0.207775475537']
FeHLu 1 ['H0.666667Fe0.111111Lu0.222222', '-0.663', '-0.208372756286']
FeHNd 1 ['H0.666667Fe0.166667Nd0.166667', '-0.569', '-0.202381710349']
FeHPm 17 ['H0.555556Fe0.222222Pm0.222222', '-0.476', '-0.200611983509']
FeHPr 2 ['H0.6Fe0.2Pr0.2', '-0.601', '-0.20127949519']
FeHRb 1 ['H0.625Fe0.125Rb0.25', '-0.294', '-0.214315355118']
FeHS 12 ['H0.666667S0.222222Fe0.111111', '-0.445', '-0.211139497441']
FeHTm 2 ['H0.571429Fe0.142857Tm0.285714', '-0.805', '-0.212267053038']
FeHY 2 ['H0.444444Fe0.222222Y0.333333', '-0.7', '-0.210486631807']
FeHfN 1 ['N0.428571Fe0.142857Hf0.428571', '-1.817', '-0.227360878426']
FeHfO 2 ['O0.666667Fe0.111111Hf0.222222', '-3.225', '-0.207299810054']
FeHfS 5 ['S0.444444Fe0.222222Hf0.333333', '-1.509', '-0.214288400438']
FeHgO 2 ['O0.625Fe0.125Hg0.25', '-0.95', '-0.207243086959']
FeHoI 10 ['Fe0.25I0.625Ho0.125', '-0.8', '-0.212433804138']
FeHoN 2 ['N0.5Fe0.3Ho0.2', '-1.08', '-0.203284611661']
FeHoO 2 ['O0.6Fe0.1Ho0.3', '-3.571', '-0.239506245755']
FeHoOs 1 ['Fe0.1Ho0.6Os0.3', '-0.29', '-0.238137289083']
FeHoP 12 ['P0.375Fe0.375Ho0.25', '-1.334', '-0.211452787368']
FeHoSi 2 ['Si0.5Fe0.4Ho0.1', '-0.812', '-0.204958752042']
FeILa 1 ['Fe0.333333I0.333333La0.333333', '-1.064', '-0.254168179189']
FeILu 29 ['Fe0.285714I0.428571Lu0.285714', '-0.564', '-0.200764426102']
FeINd 9 ['Fe0.1I0.8Nd0.1', '-0.699', '-0.208824930527']
FeINi 7 ['Fe0.1Ni0.3I0.6', '-0.405', '-0.200420704293']
FeINp 8 ['Fe0.1I0.7Np0.2', '-1.045', '-0.206683380549']
FeIO 3 ['O0.4Fe0.4I0.2', '-1.451', '-0.230121844796']
FeIP 5 ['P0.25Fe0.25I0.5', '-0.585', '-0.204276033546']
FeIPa 30 ['Fe0.1I0.5Pa0.4', '-0.381', '-0.232873949857']
FeIPm 29 ['Fe0.375I0.5Pm0.125', '-0.571', '-0.227230094723']
FeIPt 5 ['Fe0.166667I0.666667Pt0.166667', '-0.453', '-0.207535934381']
FeIRh 7 ['Fe0.142857Rh0.142857I0.714286', '-0.294', '-0.202484547878']
FeISc 1 ['Sc0.142857Fe0.142857I0.714286', '-0.826', '-0.206825265039']
FeISe 28 ['Fe0.222222Se0.555556I0.222222', '-0.44', '-0.200826137778']
FeISm 13 ['Fe0.125I0.625Sm0.25', '-1.172', '-0.206473154729']
FeITb 33 ['Fe0.4I0.4Tb0.2', '-0.471', '-0.237538186096']
FeITm 12 ['Fe0.3I0.6Tm0.1', '-0.709', '-0.205275127542']
FeIXe 15 ['Fe0.25I0.5Xe0.25', '-0.367', '-0.206847958786']
FeInN 23 ['N0.5Fe0.375In0.125', '-0.381', '-0.204706801078']
FeKN 1 ['N0.3K0.1Fe0.6', '-0.347', '-0.202231259647']
FeKO 2 ['O0.625K0.25Fe0.125', '-1.634', '-0.324924037356']

FeLaO 1 ['O0.625Fe0.125La0.25', '-3.223', '-0.328130458922']
FeLaSi 6 ['Si0.6Fe0.3La0.1', '-0.75', '-0.21119392775']
FeLuN 4 ['N0.375Fe0.125Lu0.5', '-1.821', '-0.301431115185']
FeLuO 3 ['O0.666667Fe0.166667Lu0.166667', '-2.558', '-0.217415148992']
FeMgN 28 ['N0.3Mg0.2Fe0.5', '-0.575', '-0.203034261647']
FeMgO 3 ['O0.666667Mg0.166667Fe0.166667', '-1.951', '-0.239454189549']
FeMnN 2 ['N0.333333Mn0.5Fe0.166667', '-0.531', '-0.223394258406']
FeMnP 18 ['P0.333333Mn0.555556Fe0.111111', '-0.79', '-0.200797800184']
FeMoN 28 ['N0.3Fe0.1Mo0.6', '-0.489', '-0.205542597272']
FeNNa 14 ['N0.5Na0.25Fe0.25', '-0.419', '-0.208891493924']
FeNNp 14 ['N0.571429Fe0.285714Np0.142857', '-0.71', '-0.232793277887']
FeNO 1 ['N0.1O0.6Fe0.3', '-1.566', '-0.214694840925']
FeNOs 8 ['N0.2Fe0.7Os0.1', '-0.309', '-0.207528275931']
FeNPa 35 ['N0.375Fe0.5Pa0.125', '-0.59', '-0.20433291185']
FeNPu 15 ['N0.5Fe0.1Pu0.4', '-1.352', '-0.21092575433']
FeNRe 4 ['N0.285714Fe0.571429Re0.142857', '-0.348', '-0.208055680854']
FeNSc 2 ['N0.5Sc0.4Fe0.1', '-1.881', '-0.21874790033']
FeNSi 5 ['N0.444444Si0.222222Fe0.333333', '-0.915', '-0.212844021979']
FeNTc 2 ['N0.25Fe0.625Tc0.125', '-0.339', '-0.204795604497']
FeNTe 2 ['N0.4Fe0.5Te0.1', '-0.424', '-0.212688084154']
FeNTi 12 ['N0.428571Ti0.142857Fe0.428571', '-0.737', '-0.21146774074']
FeNTl 5 ['N0.4Fe0.5Tl0.1', '-0.39', '-0.204413858612']
FeNTm 1 ['N0.4Fe0.1Tm0.5', '-1.77', '-0.205540070864']
FeNW 14 ['N0.222222Fe0.555556W0.222222', '-0.366', '-0.217898944646']
FeNXe 1 ['N0.3Fe0.5Xe0.2', '-0.351', '-0.208710874897']
FeNY 1 ['N0.4Fe0.1Y0.5', '-1.653', '-0.207374804114']
FeNYb 2 ['N0.5Fe0.2Yb0.3', '-0.855', '-0.239753355861']
FeNZn 8 ['N0.444444Fe0.333333Zn0.222222', '-0.363', '-0.210411228219']
FeNdO 1 ['O0.6Fe0.2Nd0.2', '-2.935', '-0.22904979329']
FeNdSi 9 ['Si0.5Fe0.3Nd0.2', '-0.87', '-0.2426934455']
FeNiO 1 ['O0.6Fe0.1Ni0.3', '-1.369', '-0.20549796858']
FeNiP 5 ['P0.5Fe0.333333Ni0.166667', '-0.896', '-0.210356836437']
FeNpP 1 ['P0.4Fe0.2Np0.4', '-1.014', '-0.223341596916']
FeOPa 8 ['O0.6Fe0.3Pa0.1', '-2.284', '-0.203523715451']
FeOPr 1 ['O0.625Fe0.125Pr0.25', '-3.003', '-0.204102955562']
FeOS 9 ['O0.444444S0.111111Fe0.444444', '-1.643', '-0.211181301725']
FeOSe 10 ['O0.5Fe0.25Se0.25', '-1.417', '-0.20719031199']
FeOSm 1 ['O0.625Fe0.125Sm0.25', '-3.155', '-0.280943535953']
FeOTa 2 ['O0.666667Fe0.111111Ta0.222222', '-3.077', '-0.23294792348']
FeOTc 3 ['O0.666667Fe0.166667Tc0.166667', '-1.86', '-0.218293510025']
FeOTe 5 ['O0.666667Fe0.166667Te0.166667', '-1.686', '-0.211458655995']
FeOTh 1 ['O0.666667Fe0.111111Th0.222222', '-3.53', '-0.247123409781']
FeOTm 2 ['O0.666667Fe0.111111Tm0.222222', '-2.868', '-0.207684250431']
FeOY 1 ['O0.625Fe0.125Y0.25', '-3.143', '-0.229300809391']

FeOsSc 2 ['Sc0.6Fe0.1Os0.3', '-0.438', '-0.2049859975']
FePPm 27 ['P0.5Fe0.25Pm0.25', '-1.161', '-0.200512134895']
FePPu 2 ['P0.4Fe0.1Pu0.5', '-1.241', '-0.208924120416']
FePRu 19 ['P0.333333Fe0.111111Ru0.555556', '-0.7', '-0.20364242243']
FePSn 3 ['P0.75Fe0.125Sn0.125', '-0.643', '-0.206705894333']
FePSr 11 ['P0.555556Fe0.111111Sr0.333333', '-1.223', '-0.201247330802']
FePTa 5 ['P0.4Fe0.1Ta0.5', '-1.012', '-0.20187608038']
FePTc 2 ['P0.555556Fe0.111111Tc0.333333', '-0.897', '-0.214933548395']
FePTh 1 ['P0.444444Fe0.333333Th0.222222', '-1.315', '-0.209592521388']
FePTi 38 ['P0.142857Ti0.428571Fe0.428571', '-0.849', '-0.201548046026']
FePTm 3 ['P0.5Fe0.1Tm0.4', '-1.74', '-0.20981603752']
FePU 2 ['P0.2Fe0.5U0.3', '-0.691', '-0.200804562094']
FePXe 2 ['P0.8Fe0.1Xe0.1', '-0.488', '-0.202279384666']
FePY 8 ['P0.4Fe0.1Y0.5', '-1.615', '-0.202389426666']
FePYb 23 ['P0.5Fe0.2Yb0.3', '-1.103', '-0.20655170552']
FePaSe 5 ['Fe0.166667Se0.666667Pa0.166667', '-0.783', '-0.202277818334']
FePdPm 3 ['Fe0.125Pd0.75Pm0.125', '-0.451', '-0.202290345938']
FePmS 9 ['S0.5Fe0.1Pm0.4', '-2.085', '-0.236449111991']
FePmSe 19 ['Fe0.125Se0.375Pm0.5', '-1.422', '-0.206264849062']
FePmSi 2 ['Si0.4Fe0.1Pm0.5', '-0.586', '-0.21998757225']
FePrSe 1 ['Fe0.111111Se0.555556Pr0.333333', '-1.778', '-0.21546162384']
FePrSi 4 ['Si0.571429Fe0.285714Pr0.142857', '-0.77', '-0.21304079']
FePtTh 4 ['Fe0.1Pt0.7Th0.2', '-0.786', '-0.217234707']
FeSSe 1 ['S0.333333Fe0.166667Se0.5', '-0.555', '-0.204209246162']
FeSTc 1 ['S0.5Fe0.1Tc0.4', '-0.853', '-0.237315414993']
FeSTi 1 ['S0.5Ti0.375Fe0.125', '-1.578', '-0.216209394698']
FeSZr 8 ['S0.5Fe0.166667Zr0.333333', '-1.561', '-0.213160259657']
FeScSe 1 ['Sc0.4Fe0.1Se0.5', '-1.544', '-0.2123119965']
FeSeTc 4 ['Fe0.142857Se0.571429Tc0.285714', '-0.409', '-0.255245374286']
FeSeY 3 ['Fe0.125Se0.625Y0.25', '-1.257', '-0.23951408875']
FeSeZr 2 ['Fe0.1Se0.5Zr0.4', '-1.297', '-0.200638898444']
FeSiSm 3 ['Si0.555556Fe0.333333Sm0.111111', '-0.783', '-0.229087180278']
FeSiTb 4 ['Si0.6Fe0.3Tb0.1', '-0.691', '-0.20336192995']
FeSiTh 1 ['Si0.5Fe0.4Th0.1', '-0.819', '-0.22716990225']
FeSiTm 2 ['Si0.5Fe0.4Tm0.1', '-0.793', '-0.21686174325']
FeSiY 2 ['Si0.5Fe0.375Y0.125', '-0.829', '-0.230292885']
FeSiYb 2 ['Si0.555556Fe0.333333Yb0.111111', '-0.718', '-0.2157599425']
FeSnU 2 ['Fe0.5Sn0.125U0.375', '-0.36', '-0.218525953519']
GaGdI 23 ['Ga0.333333I0.444444Gd0.222222', '-0.949', '-0.207913113836']
GaGdIr 3 ['Ga0.5Gd0.125Ir0.375', '-0.771', '-0.211976163507']
GaGdN 1 ['N0.333333Ga0.111111Gd0.555556', '-1.558', '-0.26983089572']
GaGdO 1 ['O0.625Ga0.125Gd0.25', '-3.347', '-0.294965855336']
GaGdPa 1 ['Ga0.75Gd0.125Pa0.125', '-0.518', '-0.203134455938']
GaGdPd 3 ['Ga0.5Pd0.375Gd0.125', '-0.898', '-0.215306803563']

GaGdPt 4 ['Ga0.6Gd0.1Pt0.3', '-0.868', '-0.203778603']
GaGdRh 2 ['Ga0.444444Rh0.444444Gd0.111111', '-0.909', '-0.204187548056']
GaGdS 7 ['S0.333333Ga0.222222Gd0.444444', '-1.892', '-0.224503611993']
GaGdSe 2 ['Ga0.25Se0.375Gd0.375', '-1.649', '-0.21653114625']
GaGdTc 1 ['Ga0.666667Tc0.166667Gd0.166667', '-0.543', '-0.202374555417']
GaHK 8 ['H0.428571K0.285714Ga0.285714', '-0.383', '-0.202042675076']
GaHLi 2 ['H0.444444Li0.444444Ga0.111111', '-0.564', '-0.200039585696']
GaHMn 1 ['H0.5Mn0.4Ga0.1', '-0.225', '-0.206608641548']
GaHPm 9 ['H0.4Ga0.3Pm0.3', '-0.592', '-0.20458918521']
GaHPu 23 ['H0.666667Ga0.111111Pu0.222222', '-0.527', '-0.20024799317']
GaHRb 1 ['H0.571429Ga0.142857Rb0.285714', '-0.398', '-0.209791587859']
GaHSe 7 ['H0.25Ga0.25Se0.5', '-0.579', '-0.2109203425']
GaHY 3 ['H0.375Ga0.25Y0.375', '-0.897', '-0.211043910431']
GaHYb 1 ['H0.5Ga0.2Yb0.3', '-0.766', '-0.201911091658']
GaHfIr 2 ['Ga0.375Hf0.125Ir0.5', '-0.831', '-0.204744342917']
GaHfMn 1 ['Mn0.166667Ga0.666667Hf0.166667', '-0.507', '-0.209240222961']
GaHfN 2 ['N0.428571Ga0.428571Hf0.142857', '-1.074', '-0.213108659853']
GaHfO 1 ['O0.6Ga0.3Hf0.1', '-2.773', '-0.220688676303']
GaHfPm 1 ['Ga0.7Pm0.2Hf0.1', '-0.591', '-0.2111659905']
GaHfPt 3 ['Ga0.375Hf0.25Pt0.375', '-1.065', '-0.244987175312']
GaHfRh 9 ['Ga0.285714Rh0.571429Hf0.142857', '-0.987', '-0.207162629762']
GaHfS 8 ['S0.333333Ga0.222222Hf0.444444', '-1.37', '-0.233509500884']
GaHfV 2 ['V0.1Ga0.7Hf0.2', '-0.56', '-0.20207049725']
GaHfY 1 ['Ga0.7Y0.1Hf0.2', '-0.695', '-0.2075268285']
GaHgO 1 ['O0.6Ga0.2Hg0.2', '-1.442', '-0.209810281899']
GaHoI 12 ['Ga0.222222I0.555556Ho0.222222', '-0.977', '-0.231916567133']
GaHoIr 2 ['Ga0.444444Ho0.111111Ir0.444444', '-0.783', '-0.212793127037']
GaHoO 2 ['O0.625Ga0.125Ho0.25', '-3.302', '-0.210954293878']
GaHoPd 3 ['Ga0.5Pd0.4Ho0.1', '-0.921', '-0.204867181917']
GaHoPt 3 ['Ga0.555556Ho0.111111Pt0.333333', '-0.939', '-0.231151097407']
GaHoRh 5 ['Ga0.4Rh0.5Ho0.1', '-0.935', '-0.201795236667']
GaHoS 4 ['S0.5Ga0.125Ho0.375', '-2.163', '-0.201881175874']
GaIK 1 ['K0.222222Ga0.222222I0.555556', '-1.248', '-0.233699250874']
GaILu 31 ['Ga0.285714I0.571429Lu0.142857', '-0.814', '-0.200616452005']
GaINa 3 ['Na0.25Ga0.25I0.5', '-1.129', '-0.201619501281']
GaINd 7 ['Ga0.166667I0.666667Nd0.166667', '-1.205', '-0.202379217823']
GaINi 3 ['Ni0.222222Ga0.222222I0.555556', '-0.711', '-0.206763711429']
GaIO 4 ['O0.166667Ga0.333333I0.5', '-1.218', '-0.203951409472']
GaIPa 14 ['Ga0.222222I0.555556Pa0.222222', '-0.711', '-0.231777021059']
GaIPd 7 ['Ga0.3Pd0.1I0.6', '-0.841', '-0.212217473793']
GaIPm 36 ['Ga0.4I0.3Pm0.3', '-0.808', '-0.211803836411']
GaIPr 2 ['Ga0.125I0.5Pr0.375', '-1.442', '-0.219834517845']
GaIPt 21 ['Ga0.222222I0.444444Pt0.333333', '-0.718', '-0.20254864139']
GaIRh 5 ['Ga0.1Rh0.3I0.6', '-0.462', '-0.213512159272']

GaISe 14 ['Ga0.444444Se0.111111I0.444444', '-0.722', '-0.200725441791']
GaISm 9 ['Ga0.285714I0.571429Sm0.142857', '-0.989', '-0.204405631498']
GaITb 37 ['Ga0.222222I0.333333Tb0.444444', '-0.652', '-0.224120131561']
GaITe 23 ['Ga0.333333Te0.5I0.166667', '-0.575', '-0.208739657929']
GaITm 5 ['Ga0.111111I0.777778Tm0.111111', '-0.905', '-0.211565466427']
GaInTe 3 ['Ga0.285714In0.142857Te0.571429', '-0.542', '-0.210693630295']
GaIrLu 5 ['Ga0.5Lu0.1Ir0.4', '-0.775', '-0.220013496083']
GaIrNd 5 ['Ga0.5Nd0.125Ir0.375', '-0.76', '-0.221160690069']
GaIrNp 6 ['Ga0.5Ir0.375Np0.125', '-0.589', '-0.203990695052']
GaIrP 1 ['P0.2Ga0.4Ir0.4', '-0.755', '-0.200938662041']
GaIrPm 4 ['Ga0.666667Pm0.222222Ir0.111111', '-0.636', '-0.206878790185']
GaIrPr 2 ['Ga0.5Pr0.125Ir0.375', '-0.736', '-0.20520939684']
GaIrSc 4 ['Sc0.1Ga0.5Ir0.4', '-0.794', '-0.209812979833']
GaIrSe 6 ['Ga0.3Se0.6Ir0.1', '-0.721', '-0.202046336072']
GaIrSm 5 ['Ga0.444444Sm0.111111Ir0.444444', '-0.675', '-0.211642007315']
GaIrTb 6 ['Ga0.444444Tb0.111111Ir0.444444', '-0.701', '-0.209708544111']
GaIrTm 2 ['Ga0.444444Tm0.111111Ir0.444444', '-0.778', '-0.204358859629']
GaIrU 1 ['Ga0.444444Ir0.444444U0.111111', '-0.715', '-0.200098723963']
GaIrY 6 ['Ga0.5Y0.125Ir0.375', '-0.764', '-0.203636228819']
GaIrYb 6 ['Ga0.4Yb0.1Ir0.5', '-0.676', '-0.202535216833']
GaIrZr 1 ['Ga0.428571Zr0.142857Ir0.428571', '-0.788', '-0.210952510338']
GaKN 2 ['N0.428571K0.142857Ga0.428571', '-0.722', '-0.225482550567']
GaKO 1 ['O0.5K0.2Ga0.3', '-2.123', '-0.24129974719']
GaKS 1 ['S0.375K0.375Ga0.25', '-1.298', '-0.206657506929']
GaKSe 4 ['K0.333333Ga0.333333Se0.333333', '-1.057', '-0.211363254441']
GaKrP 7 ['P0.4Ga0.1Kr0.5', '-0.315', '-0.208166208604']
GaKrPm 2 ['Ga0.666667Kr0.166667Pm0.166667', '-0.393', '-0.203868557083']
GaLaN 2 ['N0.4Ga0.5La0.1', '-0.869', '-0.207114012843']
GaLaO 3 ['O0.625Ga0.25La0.125', '-2.719', '-0.228305164191']
GaLaPt 5 ['Ga0.5La0.166667Pt0.333333', '-0.954', '-0.203876934375']
GaLaRh 3 ['Ga0.444444Rh0.444444La0.111111', '-0.9', '-0.201009173333']
GaLaSe 3 ['Ga0.111111Se0.444444La0.444444', '-1.998', '-0.205784636667']
GaLiP 1 ['Li0.285714P0.285714Ga0.428571', '-0.707', '-0.212517684583']
GaLiPa 6 ['Li0.111111Ga0.777778Pa0.111111', '-0.334', '-0.204686482222']
GaLiPm 5 ['Li0.111111Ga0.666667Pm0.222222', '-0.532', '-0.207692157778']
GaLiPt 3 ['Li0.1Ga0.6Pt0.3', '-0.811', '-0.210905583333']
GaLiRh 2 ['Li0.111111Ga0.555556Rh0.333333', '-0.794', '-0.201960786111']
GaLuN 8 ['N0.222222Ga0.222222Lu0.555556', '-1.364', '-0.200722999647']
GaLuNa 1 ['Na0.166667Ga0.666667Lu0.166667', '-0.6', '-0.243333005798']
GaLuO 1 ['O0.625Ga0.125Lu0.25', '-3.338', '-0.205462912628']
GaLuPd 3 ['Ga0.428571Pd0.428571Lu0.142857', '-0.97', '-0.204094762679']
GaLuRh 9 ['Ga0.25Rh0.375Lu0.375', '-1.051', '-0.209717677589']
GaLuSc 3 ['Sc0.25Ga0.625Lu0.125', '-0.782', '-0.20856922625']
GaLuTe 2 ['Ga0.111111Te0.555556Lu0.333333', '-1.155', '-0.208628708705']

GaMgPa 2 ['Mg0.111111Ga0.666667Pa0.222222', '-0.395', '-0.233680409815']
GaMnN 7 ['N0.222222Mn0.444444Ga0.333333', '-0.554', '-0.20399648946']
GaMnP 7 ['P0.2Mn0.6Ga0.2', '-0.596', '-0.200956635787']
GaMnPa 6 ['Mn0.125Ga0.75Pa0.125', '-0.337', '-0.215198455463']
GaMnPm 3 ['Mn0.1Ga0.8Pm0.1', '-0.361', '-0.201542738121']
GaMnV 1 ['V0.25Mn0.625Ga0.125', '-0.387', '-0.203641993786']
GaMoO 5 ['O0.6Ga0.3Mo0.1', '-2.291', '-0.229048984338']
GaMoP 13 ['P0.166667Ga0.166667Mo0.666667', '-0.575', '-0.210118225173']
GaMoPa 4 ['Ga0.777778Mo0.111111Pa0.111111', '-0.281', '-0.200603237222']
GaMoPm 3 ['Ga0.8Mo0.1Pm0.1', '-0.337', '-0.20262688725']
GaMoU 2 ['Ga0.777778Mo0.111111U0.111111', '-0.325', '-0.201528801111']
GaNNp 26 ['N0.5Ga0.4Np0.1', '-0.939', '-0.233092271452']
GaNP 7 ['N0.375P0.125Ga0.5', '-0.768', '-0.200004992501']
GaNPa 35 ['N0.625Ga0.125Pa0.25', '-0.859', '-0.220210315107']
GaNPm 4 ['N0.375Ga0.5Pm0.125', '-0.916', '-0.240979798934']
GaNPt 13 ['N0.142857Ga0.571429Pt0.285714', '-0.797', '-0.202298371379']
GaNPu 19 ['N0.5Ga0.166667Pu0.333333', '-1.319', '-0.213161868996']
GaNRh 12 ['N0.142857Ga0.428571Rh0.428571', '-0.812', '-0.220358465']
GaNSc 16 ['N0.5Sc0.1Ga0.4', '-1.068', '-0.200464846828']
GaNSr 4 ['N0.444444Ga0.444444Sr0.111111', '-0.813', '-0.210092056375']
GaNTb 4 ['N0.4Ga0.5Tb0.1', '-0.838', '-0.203184563395']
GaNTc 2 ['N0.444444Ga0.222222Tc0.333333', '-0.548', '-0.203323073477']
GaNTh 3 ['N0.444444Ga0.444444Th0.111111', '-0.999', '-0.207040035773']
GaNTi 8 ['N0.4Ti0.1Ga0.5', '-0.846', '-0.20041239715']
GaNU 2 ['N0.5Ga0.166667U0.333333', '-1.466', '-0.205411195658']
GaNY 5 ['N0.375Ga0.5Y0.125', '-0.94', '-0.201559134871']
GaNYb 5 ['N0.4Ga0.2Yb0.4', '-1.082', '-0.207241174611']
GaNaNd 1 ['Na0.166667Ga0.666667Nd0.166667', '-0.626', '-0.211012557429']
GaNaNp 5 ['Na0.142857Ga0.714286Np0.142857', '-0.372', '-0.203379786546']
GaNaPa 10 ['Na0.25Ga0.625Pa0.125', '-0.38', '-0.212433203019']
GaNaPd 1 ['Na0.142857Ga0.571429Pd0.285714', '-0.651', '-0.215415730654']
GaNaPm 9 ['Na0.2Ga0.7Pm0.1', '-0.439', '-0.201635237123']
GaNaPt 6 ['Na0.1Ga0.6Pt0.3', '-0.749', '-0.2225426']
GaNaRh 3 ['Na0.1Ga0.5Rh0.4', '-0.795', '-0.2090836415']
GaNaTe 1 ['Na0.444444Ga0.111111Te0.444444', '-0.979', '-0.200817336104']
GaNaTh 2 ['Na0.142857Ga0.714286Th0.142857', '-0.568', '-0.206708411337']
GaNaU 6 ['Na0.1Ga0.7U0.2', '-0.444', '-0.242892686479']
GaNbO 6 ['O0.6Ga0.3Nb0.1', '-2.457', '-0.231555826636']
GaNbP 1 ['P0.3Ga0.2Nb0.5', '-1.027', '-0.203522658145']
GaNdO 6 ['O0.625Ga0.125Nd0.25', '-3.203', '-0.2245096332']
GaNdPt 8 ['Ga0.428571Nd0.142857Pt0.428571', '-0.965', '-0.201125703392']
GaNdRh 9 ['Ga0.555556Rh0.333333Nd0.111111', '-0.891', '-0.200059732222']
GaNdS 4 ['S0.4Ga0.3Nd0.3', '-1.775', '-0.212695005893']
GaNdSe 2 ['Ga0.333333Se0.333333Nd0.333333', '-1.461', '-0.202842088333']

GaNiPm 2 ['Ni0.111111Ga0.666667Pm0.222222', '-0.601', '-0.226506759815']
GaNiSe 9 ['Ni0.285714Ga0.285714Se0.428571', '-0.669', '-0.203656383647']
GaNiY 1 ['Ni0.166667Ga0.5Y0.333333', '-0.869', '-0.208113235']
GaNpO 2 ['O0.333333Ga0.111111Np0.555556', '-2.189', '-0.226792279776']
GaNpP 30 ['P0.222222Ga0.333333Np0.444444', '-0.733', '-0.208479942314']
GaNpPa 1 ['Ga0.7Pa0.2Np0.1', '-0.384', '-0.204335470625']
GaNpRh 2 ['Ga0.5Rh0.4Np0.1', '-0.808', '-0.2220836415']
GaNpSe 5 ['Ga0.111111Se0.444444Np0.444444', '-1.171', '-0.207696991108']
GaOPa 9 ['O0.6Ga0.3Pa0.1', '-2.576', '-0.245793315304']
GaOPu 1 ['O0.3Ga0.2Pu0.5', '-2.182', '-0.209308089254']
GaORb 7 ['O0.375Ga0.25Rb0.375', '-1.716', '-0.208851011914']
GaORe 6 ['O0.625Ga0.125Re0.25', '-1.982', '-0.204596532655']
GaOS 1 ['O0.625S0.125Ga0.25', '-2.128', '-0.250298046748']
GaOSb 1 ['O0.625Ga0.25Sb0.125', '-2.155', '-0.22239196351']
GaOSe 1 ['O0.375Ga0.375Se0.25', '-1.703', '-0.205928954669']
GaOSi 2 ['O0.625Si0.125Ga0.25', '-2.674', '-0.2083853032']
GaOTa 4 ['O0.625Ga0.125Ta0.25', '-2.941', '-0.202044785699']
GaOTc 3 ['O0.625Ga0.25Tc0.125', '-2.169', '-0.218284066635']
GaOTh 2 ['O0.625Ga0.25Th0.125', '-3.111', '-0.209126192419']
GaOTi 4 ['O0.2Ti0.6Ga0.2', '-1.599', '-0.205751357551']
GaOU 5 ['O0.666667Ga0.111111U0.222222', '-3.433', '-0.23474564642']
GaOV 3 ['O0.625V0.125Ga0.25', '-2.473', '-0.221181368478']
GaOW 5 ['O0.6Ga0.3W0.1', '-2.337', '-0.271588013095']
GaOZr 1 ['O0.6Ga0.3Zr0.1', '-2.737', '-0.246538639803']
GaOsP 1 ['P0.333333Ga0.333333Os0.333333', '-0.683', '-0.221737377291']
GaOsPm 2 ['Ga0.666667Pm0.222222Os0.111111', '-0.572', '-0.203117894722']
GaOsSc 9 ['Sc0.571429Ga0.142857Os0.285714', '-0.631', '-0.202728222857']
GaPPd 15 ['P0.2Ga0.5Pd0.3', '-0.812', '-0.202911882958']
GaPPm 9 ['P0.4Ga0.2Pm0.4', '-1.165', '-0.211585395416']
GaPPu 2 ['P0.333333Ga0.333333Pu0.333333', '-1.083', '-0.217563012013']
GaPRe 7 ['P0.4Ga0.2Re0.4', '-0.634', '-0.202842155416']
GaPRh 14 ['P0.111111Ga0.444444Rh0.444444', '-0.902', '-0.209324985671']
GaPRu 18 ['P0.333333Ga0.222222Ru0.444444', '-0.778', '-0.205826169236']
GaPSc 21 ['P0.166667Sc0.666667Ga0.166667', '-1.009', '-0.200131639272']
GaPSm 1 ['P0.333333Ga0.166667Sm0.5', '-1.648', '-0.313072460763']
GaPSr 1 ['P0.444444Ga0.444444Sr0.111111', '-0.861', '-0.225899396165']
GaPTa 9 ['P0.5Ga0.1Ta0.4', '-0.887', '-0.24600054102']
GaPTb 8 ['P0.375Ga0.25Tb0.375', '-1.264', '-0.206982132392']
GaPTc 17 ['P0.3Ga0.2Tc0.5', '-0.613', '-0.201082369145']
GaPTe 1 ['P0.571429Ga0.142857Te0.285714', '-0.364', '-0.211380298006']
GaPTi 9 ['P0.222222Ti0.666667Ga0.111111', '-1.125', '-0.20017463163']
GaPU 8 ['P0.375Ga0.375U0.25', '-0.897', '-0.206140495182']
GaPXe 1 ['P0.428571Ga0.428571Xe0.142857', '-0.662', '-0.204140894017']
GaPY 2 ['P0.5Ga0.1Y0.4', '-1.712', '-0.20200263502']

GaPZn 1 ['P0.428571Zn0.285714Ga0.285714', '-0.647', '-0.238955700892']
GaPaPd 1 ['Ga0.7Pd0.1Pa0.2', '-0.473', '-0.219903005']
GaPaPm 5 ['Ga0.7Pm0.2Pa0.1', '-0.506', '-0.227579429']
GaPaRe 3 ['Ga0.666667Re0.166667Pa0.166667', '-0.356', '-0.270228600833']
GaPaS 7 ['S0.6Ga0.2Pa0.2', '-1.439', '-0.204408794591']
GaPaSe 20 ['Ga0.333333Se0.333333Pa0.333333', '-1.006', '-0.20331643']
GaPaTc 10 ['Ga0.777778Tc0.111111Pa0.111111', '-0.272', '-0.210434988333']
GaPaU 1 ['Ga0.75Pa0.125U0.125', '-0.403', '-0.225884160625']
GaPaV 1 ['V0.1Ga0.7Pa0.2', '-0.423', '-0.215053132429']
GaPbSe 2 ['Ga0.375Se0.375Pb0.25', '-0.672', '-0.228634988437']
GaPdPm 7 ['Ga0.1Pd0.7Pm0.2', '-0.714', '-0.202211055']
GaPdPu 1 ['Ga0.1Pd0.4Pu0.5', '-0.686', '-0.27238048125']
GaPdSc 1 ['Sc0.4Ga0.2Pd0.4', '-1.046', '-0.213158436833']
GaPdSm 3 ['Ga0.555556Pd0.333333Sm0.111111', '-0.878', '-0.210819897778']
GaPdTb 2 ['Ga0.571429Pd0.285714Tb0.142857', '-0.802', '-0.21737666117']
GaPdTc 1 ['Ga0.6Tc0.3Pd0.1', '-0.375', '-0.215116219125']
GaPdTm 5 ['Ga0.428571Pd0.428571Tm0.142857', '-0.956', '-0.200513768571']
GaPdY 8 ['Ga0.444444Y0.222222Pd0.333333', '-1.013', '-0.20077857375']
GaPmPt 7 ['Ga0.625Pm0.125Pt0.25', '-0.795', '-0.214436917812']
GaPmRe 1 ['Ga0.7Pm0.2Re0.1', '-0.429', '-0.2020422685']
GaPmRh 10 ['Ga0.666667Rh0.111111Pm0.222222', '-0.657', '-0.200727764608']
GaPmRu 2 ['Ga0.7Ru0.1Pm0.2', '-0.581', '-0.20227939']
GaPmS 16 ['S0.3Ga0.3Pm0.4', '-1.541', '-0.213631045795']
GaPmSe 33 ['Ga0.3Se0.5Pm0.2', '-1.224', '-0.22091591025']
GaPmTc 5 ['Ga0.666667Tc0.111111Pm0.222222', '-0.48', '-0.223440663889']
GaPmTe 4 ['Ga0.285714Te0.571429Pm0.142857', '-0.803', '-0.203883434595']
GaPrPt 3 ['Ga0.571429Pr0.142857Pt0.285714', '-0.91', '-0.225863091785']
GaPrRh 3 ['Ga0.5Rh0.375Pr0.125', '-0.946', '-0.259006443229']
GaPtS 18 ['S0.555556Ga0.111111Pt0.333333', '-0.834', '-0.204376549437']
GaPtSc 1 ['Sc0.125Ga0.5Pt0.375', '-0.967', '-0.204983105625']
GaPtSm 6 ['Ga0.5Sm0.125Pt0.375', '-0.95', '-0.211839605']
GaPtSr 3 ['Ga0.6Sr0.1Pt0.3', '-0.801', '-0.205520034']
GaPtTb 6 ['Ga0.6Tb0.1Pt0.3', '-0.854', '-0.21747377114']
GaPtTh 10 ['Ga0.5Pt0.4Th0.1', '-0.942', '-0.205291126167']
GaPtTi 3 ['Ti0.166667Ga0.5Pt0.333333', '-0.919', '-0.207822827702']
GaPtTm 3 ['Ga0.555556Tm0.111111Pt0.333333', '-0.929', '-0.227561988333']
GaPtY 6 ['Ga0.5Y0.166667Pt0.333333', '-0.977', '-0.210134215972']
GaPtYb 4 ['Ga0.6Yb0.1Pt0.3', '-0.89', '-0.2094344875']
GaPtZr 2 ['Ga0.5Zr0.166667Pt0.333333', '-1.003', '-0.220872269444']
GaPuS 7 ['S0.375Ga0.25Pu0.375', '-1.66', '-0.220916989742']
GaPuTe 1 ['Ga0.1Te0.5Pu0.4', '-1.211', '-0.254561416997']
GaRbS 1 ['S0.5Ga0.1Rb0.4', '-1.331', '-0.215973382744']
GaRbSe 18 ['Ga0.333333Se0.444444Rb0.222222', '-0.952', '-0.20263303861']
GaRhSc 4 ['Sc0.1Ga0.4Rh0.5', '-1.024', '-0.217139093167']

GaRhSe 1 ['Ga0.375Se0.5Rh0.125', '-0.796', '-0.208329142679']
GaRhSm 2 ['Ga0.5Rh0.4Sm0.1', '-0.897', '-0.20641832375']
GaRhTb 6 ['Ga0.4Rh0.5Tb0.1', '-0.859', '-0.204427794533']
GaRhTi 3 ['Ti0.111111Ga0.444444Rh0.444444', '-0.944', '-0.202097419394']
GaRhTm 3 ['Ga0.444444Rh0.444444Tm0.111111', '-0.936', '-0.214275557674']
GaRhU 2 ['Ga0.5Rh0.4U0.1', '-0.817', '-0.20336506875']
GaRhV 1 ['V0.125Ga0.5Rh0.375', '-0.815', '-0.24018060125']
GaRhY 6 ['Ga0.2Y0.4Rh0.4', '-1.006', '-0.213065482555']
GaRhYb 6 ['Ga0.428571Rh0.428571Yb0.142857', '-0.852', '-0.201337534524']
GaRhZr 3 ['Ga0.444444Zr0.111111Rh0.444444', '-0.952', '-0.216008175926']
GaRuTb 1 ['Ga0.555556Ru0.333333Tb0.111111', '-0.55', '-0.205452022259']
GaRuTh 1 ['Ga0.5Ru0.4Th0.1', '-0.586', '-0.204152829333']
GaRuY 1 ['Ga0.666667Y0.222222Ru0.111111', '-0.762', '-0.203855675556']
GaSSc 4 ['S0.444444Sc0.333333Ga0.222222', '-1.825', '-0.2023913434']
GaSSm 8 ['S0.444444Ga0.222222Sm0.333333', '-1.941', '-0.200563527381']
GaSTb 1 ['S0.333333Ga0.333333Tb0.333333', '-1.49', '-0.22128974072']
GaSTc 4 ['S0.5Ga0.166667Tc0.333333', '-0.869', '-0.202445355077']
GaSTh 4 ['S0.333333Ga0.333333Th0.333333', '-1.613', '-0.203541801577']
GaSTm 2 ['S0.333333Ga0.222222Tm0.444444', '-1.784', '-0.204387541993']
GaSU 1 ['S0.5Ga0.1U0.4', '-1.637', '-0.204419652825']
GaSY 10 ['S0.333333Ga0.222222Y0.444444', '-1.853', '-0.205340501993']
GaSZr 8 ['S0.5Ga0.166667Zr0.333333', '-1.639', '-0.22084981674']
GaSbSe 4 ['Ga0.428571Se0.285714Sb0.285714', '-0.604', '-0.218458665']
GaScSe 24 ['Sc0.285714Ga0.285714Se0.428571', '-1.286', '-0.206235184643']
GaSeSi 1 ['Si0.1Ga0.4Se0.5', '-0.765', '-0.22415638675']
GaSeSm 1 ['Ga0.375Se0.25Sm0.375', '-1.425', '-0.219812818125']
GaSeTb 14 ['Ga0.125Se0.5Tb0.375', '-1.626', '-0.216424253872']
GaSeTc 4 ['Ga0.125Se0.625Tc0.25', '-0.405', '-0.22096017125']
GaSeTh 17 ['Ga0.4Se0.4Th0.2', '-1.249', '-0.205364255998']
GaSeTi 5 ['Ti0.333333Ga0.222222Se0.444444', '-1.178', '-0.203492072245']
GaSeTm 5 ['Ga0.125Se0.5Tm0.375', '-1.602', '-0.206636836251']
GaSeY 18 ['Ga0.222222Se0.555556Y0.222222', '-1.344', '-0.231973092222']
GaSeZr 9 ['Ga0.142857Se0.571429Zr0.285714', '-1.354', '-0.202318813143']
GaTcU 1 ['Ga0.75Tc0.125U0.125', '-0.318', '-0.20028062125']
GdGeH 22 ['H0.5Ge0.125Gd0.375', '-0.932', '-0.201172409845']
GdGeI 29 ['Ge0.25I0.375Gd0.375', '-1.099', '-0.211915959702']
GdGeIr 2 ['Ge0.5Gd0.125Ir0.375', '-0.786', '-0.209818334375']
GdGePa 1 ['Ge0.666667Gd0.111111Pa0.222222', '-0.61', '-0.251320158796']
GdGePt 1 ['Ge0.5Gd0.125Pt0.375', '-0.869', '-0.22685929375']
GdGeRu 2 ['Ge0.555556Ru0.333333Gd0.111111', '-0.706', '-0.201341558987']
GdGeS 6 ['S0.2Ge0.3Gd0.5', '-1.607', '-0.212722192446']
GdGeSe 5 ['Ge0.111111Se0.444444Gd0.444444', '-1.845', '-0.200063395833']
GdHI 69 ['H0.1I0.5Gd0.4', '-1.15', '-0.200143512023']
GdHIn 2 ['H0.571429In0.142857Gd0.285714', '-0.813', '-0.209016643117']

GdHIr 4 ['H0.714286Gd0.142857Ir0.142857', '-0.561', '-0.220432384183']
GdHMn 4 ['H0.625Mn0.25Gd0.125', '-0.507', '-0.20900333616']
GdHN 7 ['H0.555556N0.222222Gd0.222222', '-1.014', '-0.209439257538']
GdHNa 1 ['H0.555556Na0.111111Gd0.333333', '-0.772', '-0.204135874481']
GdHO 14 ['H0.2O0.4Gd0.4', '-2.941', '-0.208150477126']
GdHOs 3 ['H0.666667Gd0.222222Os0.111111', '-0.736', '-0.206228153173']
GdHPm 3 ['H0.6Pm0.3Gd0.1', '-0.619', '-0.21628954294']
GdHPt 9 ['H0.5Gd0.2Pt0.3', '-0.833', '-0.203506179178']
GdHRh 21 ['H0.5Rh0.3Gd0.2', '-0.676', '-0.204158876908']
GdHRu 9 ['H0.6Ru0.1Gd0.3', '-0.826', '-0.212706744439']
GdHS 15 ['H0.5S0.166667Gd0.333333', '-1.321', '-0.201272696293']
GdHSc 2 ['H0.6Sc0.3Gd0.1', '-0.811', '-0.20381504494']
GdHSi 27 ['H0.555556Si0.333333Gd0.111111', '-0.465', '-0.200114076587']
GdHSn 5 ['H0.555556Sn0.111111Gd0.333333', '-0.877', '-0.200855200519']
GdHTl 1 ['H0.5Gd0.3Tl0.2', '-0.821', '-0.260314779408']
GdHfI 5 ['I0.714286Gd0.142857Hf0.142857', '-1.258', '-0.233469869159']
GdHfS 10 ['S0.428571Gd0.142857Hf0.428571', '-1.743', '-0.212006411318']
GdHgI 30 ['I0.5Gd0.4Hg0.1', '-1.133', '-0.225533781119']
GdHoI 9 ['I0.666667Gd0.111111Ho0.222222', '-1.254', '-0.458630827211']
GdHoO 1 ['O0.625Gd0.125Ho0.25', '-3.871', '-0.264468931641']
GdHoOs 1 ['Gd0.125Ho0.5Os0.375', '-0.329', '-0.213922416771']
GdIIn 40 ['In0.333333I0.222222Gd0.444444', '-0.888', '-0.204922699644']
GdIIr 52 ['I0.3Gd0.3Ir0.4', '-0.964', '-0.21764037352']
GdIK 13 ['K0.3I0.6Gd0.1', '-1.415', '-0.210418807159']
GdIKr 21 ['Kr0.111111I0.555556Gd0.333333', '-1.056', '-0.208359054617']
GdILa 5 ['I0.714286La0.142857Gd0.142857', '-1.671', '-0.352258945224']
GdILi 17 ['Li0.333333I0.555556Gd0.111111', '-1.416', '-0.252161575732']
GdILu 14 ['I0.6Gd0.2Lu0.2', '-0.949', '-0.218180939284']
GdIMg 9 ['Mg0.222222I0.666667Gd0.111111', '-1.326', '-0.260464419532']
GdIMn 19 ['Mn0.125I0.5Gd0.375', '-1.119', '-0.222709471647']
GdIMo 8 ['Mo0.2I0.7Gd0.1', '-0.758', '-0.203625845247']
GdIN 37 ['N0.166667I0.333333Gd0.5', '-1.359', '-0.221499364271']
GdINa 21 ['Na0.25I0.5Gd0.25', '-1.335', '-0.203121798988']
GdINb 8 ['Nb0.125I0.625Gd0.25', '-1.059', '-0.228728271189']
GdINd 8 ['I0.666667Nd0.166667Gd0.166667', '-1.48', '-0.345989105663']
GdINi 45 ['Ni0.2I0.3Gd0.5', '-0.919', '-0.23639946052']
GdINp 6 ['I0.666667Gd0.222222Np0.111111', '-1.294', '-0.280969252407']
GdIO 46 ['O0.5I0.125Gd0.375', '-3.472', '-0.205521834719']
GdIOs 28 ['I0.625Gd0.125Os0.25', '-0.537', '-0.219134645481']
GdIP 40 ['P0.2I0.3Gd0.5', '-1.405', '-0.203503301478']
GdIPa 11 ['I0.6Gd0.1Pa0.3', '-0.638', '-0.300500486657']
GdIPb 31 ['I0.444444Gd0.444444Pb0.111111', '-1.103', '-0.215348006233']
GdIPd 43 ['Pd0.1I0.8Gd0.1', '-0.562', '-0.201570799649']
GdIPm 12 ['I0.6Pm0.3Gd0.1', '-1.039', '-0.247933617907']

GdIPr 6 ['I0.666667Pr0.111111Gd0.222222', '-1.439', '-0.277595502404']
GdIPt 57 ['I0.142857Gd0.428571Pt0.428571', '-1.37', '-0.217961939058']
GdIPu 15 ['I0.555556Gd0.333333Pu0.111111', '-1.145', '-0.207882520037']
GdIRb 13 ['Rb0.285714I0.571429Gd0.142857', '-1.508', '-0.213061846292']
GdIRe 14 ['I0.625Gd0.125Re0.25', '-0.652', '-0.224165734037']
GdIRh 59 ['Rh0.5I0.2Gd0.3', '-0.917', '-0.205382431985']
GdIRu 35 ['Ru0.3I0.2Gd0.5', '-0.693', '-0.207272272597']
GdIS 20 ['S0.222222I0.444444Gd0.333333', '-1.799', '-0.201306063999']
GdISb 40 ['Sb0.125I0.375Gd0.5', '-1.158', '-0.203361403566']
GdISc 10 ['Sc0.125I0.625Gd0.25', '-1.322', '-0.224567418479']
GdISe 44 ['Se0.375I0.375Gd0.25', '-1.385', '-0.203068140938']
GdISi 13 ['Si0.111111I0.555556Gd0.333333', '-1.386', '-0.232717798166']
GdISm 8 ['I0.666667Sm0.166667Gd0.166667', '-1.304', '-0.253465397754']
GdISn 22 ['Sn0.285714I0.571429Gd0.142857', '-0.969', '-0.231008127526']
GdISr 8 ['Sr0.1I0.8Gd0.1', '-1.045', '-0.242921606649']
GdITa 6 ['I0.714286Gd0.142857Ta0.142857', '-1.044', '-0.312291640095']
GdITb 12 ['I0.6Gd0.1Tb0.3', '-0.765', '-0.256606220257']
GdITc 16 ['Tc0.3I0.6Gd0.1', '-0.47', '-0.215707716385']
GdITe 57 ['Te0.555556I0.111111Gd0.333333', '-1.421', '-0.203804268052']
GdITh 4 ['I0.666667Gd0.222222Th0.111111', '-1.346', '-0.203015302686']
GdITi 9 ['Ti0.222222I0.666667Gd0.111111', '-1.107', '-0.207704867194']
GdITl 31 ['I0.4Gd0.3Tl0.3', '-0.964', '-0.210776012278']
GdITm 8 ['I0.666667Gd0.166667Tm0.166667', '-1.346', '-0.296174128163']
GdIU 3 ['I0.75Gd0.125U0.125', '-1.192', '-0.268469824892']
GdIV 10 ['V0.125I0.625Gd0.25', '-1.051', '-0.202244665355']
GdIW 10 ['I0.666667Gd0.111111W0.222222', '-0.494', '-0.211453018206']
GdIXe 31 ['I0.428571Xe0.285714Gd0.285714', '-0.932', '-0.205450618243']
GdIY 5 ['Y0.142857I0.714286Gd0.142857', '-1.541', '-0.28419882951']
GdIYb 7 ['I0.7Gd0.1Yb0.2', '-1.604', '-0.282702671413']
GdIZn 20 ['Zn0.3I0.6Gd0.1', '-0.878', '-0.214684168157']
GdIZr 7 ['Zr0.166667I0.666667Gd0.166667', '-1.245', '-0.207118725045']
GdInN 31 ['N0.4In0.1Gd0.5', '-1.677', '-0.201202979114']
GdInO 1 ['O0.625In0.125Gd0.25', '-3.171', '-0.224342225362']
GdIrN 3 ['N0.375Gd0.5Ir0.125', '-1.713', '-0.239234492998']
GdIrNa 1 ['Na0.1Gd0.5Ir0.4', '-0.8', '-0.205278417']
GdIrO 1 ['O0.666667Gd0.222222Ir0.111111', '-2.757', '-0.295210880064']
GdIrP 18 ['P0.6Gd0.2Ir0.2', '-1.431', '-0.207481068124']
GdIrSe 14 ['Se0.571429Gd0.285714Ir0.142857', '-1.628', '-0.204574641071']
GdIrSi 5 ['Si0.6Gd0.1Ir0.3', '-0.909', '-0.211731736333']
GdIrSn 1 ['Sn0.375Gd0.25Ir0.375', '-0.843', '-0.200567169602']
GdKO 3 ['O0.6K0.1Gd0.3', '-3.354', '-0.22073202072']
GdLaO 3 ['O0.6La0.1Gd0.3', '-4.001', '-0.228394723033']
GdLiSe 3 ['Li0.111111Se0.444444Gd0.444444', '-1.825', '-0.216493213751']
GdLuO 1 ['O0.625Gd0.125Lu0.25', '-3.906', '-0.244782378253']

GdLuTe 1 ['Te0.6Gd0.1Lu0.3', '-1.357', '-0.217604632875']
GdMgN 2 ['N0.4Mg0.1Gd0.5', '-1.604', '-0.211678769197']
GdMgO 1 ['O0.6Mg0.1Gd0.3', '-3.645', '-0.206956537055']
GdMgS 8 ['Mg0.222222S0.333333Gd0.444444', '-1.679', '-0.203242839123']
GdMnN 9 ['N0.375Mn0.375Gd0.25', '-1.201', '-0.208693890486']
GdMnO 3 ['O0.625Mn0.125Gd0.25', '-3.278', '-0.210604797172']
GdMnSi 2 ['Si0.5Mn0.4Gd0.1', '-0.738', '-0.201597453482']
GdMoSe 2 ['Se0.555556Mo0.222222Gd0.222222', '-1.483', '-0.208540413056']
GdNNa 1 ['N0.4Na0.1Gd0.5', '-1.687', '-0.313223986864']
GdNNp 4 ['N0.555556Gd0.222222Np0.222222', '-1.545', '-0.242921231791']
GdNO 12 ['N0.25O0.625Gd0.125', '-1.719', '-0.201354017344']
GdNOs 4 ['N0.333333Gd0.5Os0.166667', '-1.417', '-0.205753049678']
GdNP 25 ['N0.3P0.1Gd0.6', '-1.593', '-0.216211924502']
GdNPa 15 ['N0.571429Gd0.285714Pa0.142857', '-1.47', '-0.206461650346']
GdNPd 3 ['N0.375Pd0.125Gd0.5', '-1.717', '-0.203838166747']
GdNPt 2 ['N0.285714Gd0.428571Pt0.285714', '-1.657', '-0.205892583833']
GdNPu 5 ['N0.444444Gd0.222222Pu0.333333', '-1.575', '-0.203292429293']
GdNRh 1 ['N0.375Rh0.125Gd0.5', '-1.757', '-0.267793044247']
GdNRu 1 ['N0.4Ru0.1Gd0.5', '-1.684', '-0.261333537114']
GdNSe 1 ['N0.333333Se0.111111Gd0.555556', '-1.758', '-0.214189372664']
GdNSi 5 ['N0.285714Si0.285714Gd0.428571', '-1.448', '-0.20317972776']
GdNTa 1 ['N0.444444Gd0.444444Ta0.111111', '-1.751', '-0.224582207627']
GdNTc 5 ['N0.444444Tc0.111111Gd0.444444', '-1.727', '-0.200582207627']
GdNTe 3 ['N0.375Te0.125Gd0.5', '-1.823', '-0.20044404383']
GdNTi 9 ['N0.5Ti0.2Gd0.3', '-1.775', '-0.225853261345']
GdNV 1 ['N0.4V0.1Gd0.5', '-1.607', '-0.233223986864']
GdNW 2 ['N0.6Gd0.1W0.3', '-0.671', '-0.217836473578']
GdNZn 1 ['N0.444444Zn0.111111Gd0.444444', '-1.812', '-0.285582207627']
GdNaO 1 ['O0.625Na0.125Gd0.25', '-3.015', '-0.252428031092']
GdNaSe 2 ['Na0.1Se0.5Gd0.4', '-1.939', '-0.216884181541']
GdNbO 1 ['O0.6Nb0.2Gd0.2', '-3.431', '-0.238067137374']
GdNbSe 4 ['Se0.625Nb0.125Gd0.25', '-1.704', '-0.204443116875']
GdNiO 3 ['O0.625Ni0.125Gd0.25', '-2.971', '-0.290229453129']
GdNiSi 3 ['Si0.4Ni0.2Gd0.4', '-1.012', '-0.2179542955']
GdNpPt 2 ['Gd0.111111Pt0.777778Np0.111111', '-0.748', '-0.21241723875']
GdOP 4 ['O0.333333P0.333333Gd0.333333', '-2.761', '-0.211902460608']
GdOPa 5 ['O0.666667Gd0.166667Pa0.166667', '-3.356', '-0.226338386986']
GdOPd 3 ['O0.666667Pd0.166667Gd0.166667', '-2.063', '-0.204124341055']
GdOPt 1 ['O0.625Gd0.25Pt0.125', '-2.875', '-0.226782281949']
GdOPu 1 ['O0.666667Gd0.166667Pu0.166667', '-3.483', '-0.272320081996']
GdORh 1 ['O0.6Rh0.2Gd0.2', '-2.703', '-0.29517707097']
GdOS 11 ['O0.375S0.25Gd0.375', '-3.408', '-0.20042835785']
GdOSb 3 ['O0.333333Sb0.166667Gd0.5', '-2.734', '-0.201774118733']
GdOSc 2 ['O0.6Sc0.2Gd0.2', '-4.019', '-0.21899131047']

GdOSe 17 ['O0.444444Se0.222222Gd0.333333', '-3.222', '-0.205597763219']
GdOSi 2 ['O0.666667Si0.222222Gd0.111111', '-3.354', '-0.213930079188']
GdOSr 1 ['O0.625Sr0.125Gd0.25', '-3.446', '-0.265851486948']
GdOTc 6 ['O0.666667Tc0.222222Gd0.111111', '-2.474', '-0.217458004503']
GdOTi 3 ['O0.625Ti0.25Gd0.125', '-3.627', '-0.2048122796']
GdOTm 1 ['O0.625Gd0.25Tm0.125', '-3.872', '-0.272906213409']
GdOV 3 ['O0.625V0.125Gd0.25', '-3.628', '-0.251402636136']
GdOW 2 ['O0.6Gd0.3W0.1', '-3.6', '-0.214319952721']
GdOY 1 ['O0.625Y0.125Gd0.25', '-3.848', '-0.287546977003']
GdOZn 4 ['O0.666667Zn0.111111Gd0.222222', '-2.75', '-0.276561460163']
GdOZr 1 ['O0.625Zr0.25Gd0.125', '-3.811', '-0.235839329385']
GdOsP 1 ['P0.444444Gd0.444444Os0.111111', '-1.764', '-0.224195263796']
GdOsSi 1 ['Si0.444444Gd0.111111Os0.444444', '-0.81', '-0.240183157593']
GdPPd 1 ['P0.4Pd0.3Gd0.3', '-1.492', '-0.219151909858']
GdPPm 2 ['P0.5Pm0.4Gd0.1', '-1.527', '-0.22712932977']
GdPPt 1 ['P0.166667Gd0.166667Pt0.666667', '-1.099', '-0.219480160642']
GdPPu 2 ['P0.444444Gd0.333333Pu0.222222', '-1.642', '-0.200458703518']
GdPRh 24 ['P0.5Rh0.25Gd0.25', '-1.488', '-0.206326865311']
GdPRu 1 ['P0.444444Ru0.111111Gd0.444444', '-1.757', '-0.217195263796']
GdPTa 1 ['P0.5Gd0.1Ta0.4', '-1.1', '-0.20850350752']
GdPXe 2 ['P0.4Xe0.1Gd0.5', '-1.588', '-0.202175737416']
GdPaS 3 ['S0.666667Gd0.166667Pa0.166667', '-1.852', '-0.204385584324']
GdPaSe 6 ['Se0.625Gd0.25Pa0.125', '-1.745', '-0.249314079373']
GdPaSi 2 ['Si0.666667Gd0.111111Pa0.222222', '-0.533', '-0.206537386389']
GdPdPm 1 ['Pd0.8Pm0.1Gd0.1', '-0.714', '-0.210802727']
GdPdSe 20 ['Se0.5Pd0.25Gd0.25', '-1.485', '-0.211282388507']
GdPmS 5 ['S0.555556Pm0.333333Gd0.111111', '-2.327', '-0.207088770546']
GdPmSe 8 ['Se0.625Pm0.125Gd0.25', '-1.849', '-0.24907536781']
GdPtS 2 ['S0.428571Gd0.285714Pt0.285714', '-1.813', '-0.20098258935']
GdPtSe 15 ['Se0.285714Gd0.285714Pt0.428571', '-1.487', '-0.207452392729']
GdPtTh 2 ['Gd0.111111Pt0.777778Th0.111111', '-0.953', '-0.247041765278']
GdRbTe 1 ['Rb0.142857Te0.571429Gd0.285714', '-1.495', '-0.241251929167']
GdReSi 1 ['Si0.444444Gd0.111111Re0.444444', '-0.6', '-0.20279210627']
GdRhS 11 ['S0.444444Rh0.333333Gd0.222222', '-1.581', '-0.201185907104']
GdRhSe 24 ['Se0.555556Rh0.222222Gd0.222222', '-1.388', '-0.201283605556']
GdRhSi 4 ['Si0.444444Rh0.444444Gd0.111111', '-1.197', '-0.2058927075']
GdRuS 2 ['S0.555556Ru0.222222Gd0.222222', '-1.734', '-0.234112587492']
GdRuSe 3 ['Se0.5Ru0.1Gd0.4', '-1.867', '-0.20325940325']
GdSSb 1 ['S0.375Sb0.25Gd0.375', '-1.95', '-0.248183416619']
GdSSe 2 ['S0.2Se0.5Gd0.3', '-1.768', '-0.219272153778']
GdSSi 14 ['Si0.333333S0.222222Gd0.444444', '-1.547', '-0.210954480496']
GdSSn 1 ['S0.333333Sn0.222222Gd0.444444', '-1.863', '-0.201641814903']
GdSTa 1 ['S0.5Gd0.2Ta0.3', '-1.775', '-0.206977750992']
GdSTc 5 ['S0.6Tc0.3Gd0.1', '-1.328', '-0.218990775467']

GdSTe 1 ['S0.333333Te0.111111Gd0.555556', '-1.943', '-0.200744546067']
GdSTl 5 ['S0.375Gd0.5Tl0.125', '-1.92', '-0.203152685367']
GdSZr 3 ['S0.5Zr0.333333Gd0.166667', '-1.94', '-0.220806854865']
GdSbSe 5 ['Se0.333333Sb0.222222Gd0.444444', '-1.782', '-0.213524838056']
GdScSe 8 ['Sc0.1Se0.6Gd0.3', '-1.965', '-0.222780329499']
GdSeSi 11 ['Si0.166667Se0.333333Gd0.5', '-1.688', '-0.216004964167']
GdSeTc 1 ['Se0.6Tc0.3Gd0.1', '-0.683', '-0.205128167249']
GdSeTe 1 ['Se0.3Te0.3Gd0.4', '-1.857', '-0.203163615']
GdSeTl 2 ['Se0.444444Gd0.333333Tl0.222222', '-1.65', '-0.20039662']
GdSeTm 1 ['Se0.6Gd0.1Tm0.3', '-1.878', '-0.227703589376']
GdSeY 8 ['Se0.555556Y0.333333Gd0.111111', '-1.928', '-0.219763811943']
GdSeZn 3 ['Zn0.111111Se0.444444Gd0.444444', '-1.819', '-0.20959782402']
GdSeZr 4 ['Se0.571429Zr0.285714Gd0.142857', '-1.65', '-0.202357103333']
GeHHo 21 ['H0.6Ge0.2Ho0.2', '-0.694', '-0.20826410969']
GeHK 9 ['H0.6K0.3Ge0.1', '-0.355', '-0.233244450013']
GeHLa 19 ['H0.5Ge0.125La0.375', '-0.901', '-0.213989726408']
GeHLi 11 ['H0.625Li0.25Ge0.125', '-0.414', '-0.209272266954']
GeHLu 5 ['H0.666667Ge0.111111Lu0.222222', '-0.666', '-0.211668977918']
GeHNd 14 ['H0.333333Ge0.333333Nd0.333333', '-0.869', '-0.200295441864']
GeHPa 1 ['H0.1Ge0.6Pa0.3', '-0.492', '-0.273407723615']
GeHPm 26 ['H0.125Ge0.375Pm0.5', '-0.745', '-0.223537514727']
GeHPr 14 ['H0.625Ge0.125Pr0.25', '-0.706', '-0.211786453427']
GeHPu 5 ['H0.6Ge0.2Pu0.2', '-0.496', '-0.20315958546']
GeHRb 2 ['H0.5Ge0.166667Rb0.333333', '-0.328', '-0.222198139272']
GeHSc 17 ['H0.571429Sc0.285714Ge0.142857', '-0.779', '-0.203637328752']
GeHSe 5 ['H0.111111Ge0.333333Se0.555556', '-0.428', '-0.205927435278']
GeHSm 8 ['H0.625Ge0.125Sm0.25', '-0.77', '-0.210664462315']
GeHSr 5 ['H0.5Ge0.166667Sr0.333333', '-0.737', '-0.207852440158']
GeHTb 21 ['H0.4Ge0.2Tb0.4', '-0.703', '-0.20268780326']
GeHTm 23 ['H0.428571Ge0.285714Tm0.285714', '-0.801', '-0.200800268588']
GeHY 16 ['H0.4Ge0.2Y0.4', '-0.991', '-0.211152981126']
GeHYb 18 ['H0.4Ge0.3Yb0.3', '-0.732', '-0.205396006793']
GeHfMn 1 ['Mn0.375Ge0.375Hf0.25', '-0.651', '-0.248790023116']
GeHfO 2 ['O0.666667Ge0.166667Hf0.166667', '-3.094', '-0.21746523941']
GeHfOs 1 ['Ge0.428571Hf0.142857Os0.428571', '-0.5', '-0.210909726429']
GeHfPa 1 ['Ge0.7Hf0.1Pa0.2', '-0.493', '-0.223333561']
GeHfRu 1 ['Ge0.333333Ru0.444444Hf0.222222', '-0.873', '-0.250867783426']
GeHfS 2 ['S0.375Ge0.125Hf0.5', '-1.552', '-0.278042776308']
GeHfTc 3 ['Ge0.444444Tc0.444444Hf0.111111', '-0.387', '-0.201456906667']
GeHgO 4 ['O0.625Ge0.25Hg0.125', '-1.796', '-0.256086713518']
GeHoI 17 ['Ge0.166667I0.5Ho0.333333', '-1.019', '-0.206717504875']
GeHoIr 7 ['Ge0.444444Ho0.111111Ir0.444444', '-0.781', '-0.219782488248']
GeHoN 2 ['N0.111111Ge0.333333Ho0.555556', '-1.27', '-0.20343995911']
GeHoO 1 ['O0.625Ge0.125Ho0.25', '-3.381', '-0.251343215961']

GeHoPt 4 ['Ge0.1Ho0.1Pt0.8', '-0.75', '-0.205999871668']
GeHoRh 4 ['Ge0.625Rh0.25Ho0.125', '-0.763', '-0.207074404114']
GeHoS 12 ['S0.375Ge0.125Ho0.5', '-2.013', '-0.207794563706']
GeHoSe 5 ['Ge0.25Se0.375Ho0.375', '-1.621', '-0.208723406044']
GeILu 35 ['Ge0.3I0.6Lu0.1', '-0.718', '-0.210580929376']
GeINd 12 ['Ge0.222222I0.555556Nd0.222222', '-1.223', '-0.200190329852']
GeINi 1 ['Ni0.2Ge0.2I0.6', '-0.58', '-0.201302870093']
GeIPa 19 ['Ge0.25I0.5Pa0.25', '-0.557', '-0.215152211598']
GeIPd 2 ['Ge0.166667Pd0.166667I0.666667', '-0.622', '-0.203716140152']
GeIPm 52 ['Ge0.3I0.6Pm0.1', '-0.728', '-0.200190262793']
GeIPr 1 ['Ge0.222222I0.444444Pr0.333333', '-1.429', '-0.201527766866']
GeIPt 22 ['Ge0.142857I0.714286Pt0.142857', '-0.572', '-0.203554985115']
GeIRh 8 ['Ge0.142857Rh0.142857I0.714286', '-0.512', '-0.212529498255']
GeISc 4 ['Sc0.5Ge0.25I0.25', '-1.193', '-0.205191347516']
GeISm 11 ['Ge0.285714I0.571429Sm0.142857', '-0.957', '-0.233005816706']
GeISr 1 ['Ge0.222222Sr0.444444I0.333333', '-1.435', '-0.224585055301']
GeITb 40 ['Ge0.25I0.25Tb0.5', '-0.693', '-0.20709661456']
GeITm 10 ['Ge0.1I0.8Tm0.1', '-0.803', '-0.217767409292']
GeIXe 5 ['Ge0.111111I0.444444Xe0.444444', '-0.437', '-0.204078498643']
GeIYb 1 ['Ge0.111111I0.555556Yb0.333333', '-1.798', '-0.229588291675']
GeInO 2 ['O0.6Ge0.1In0.3', '-2.028', '-0.211710851331']
GeInPa 1 ['Ge0.6In0.1Pa0.3', '-0.413', '-0.2214533615']
GeIrLa 1 ['Ge0.555556La0.111111Ir0.333333', '-0.797', '-0.228967537222']
GeIrNa 8 ['Na0.111111Ge0.555556Ir0.333333', '-0.531', '-0.22361213381']
GeIrNd 5 ['Ge0.571429Nd0.142857Ir0.285714', '-0.818', '-0.239461647347']
GeIrP 1 ['P0.8Ge0.1Ir0.1', '-0.61', '-0.211366814374']
GeIrPa 1 ['Ge0.666667Ir0.111111Pa0.222222', '-0.454', '-0.209833162407']
GeIrPm 1 ['Ge0.5Pm0.125Ir0.375', '-0.68', '-0.271126403229']
GeIrPr 1 ['Ge0.5Pr0.125Ir0.375', '-0.827', '-0.228807324375']
GeIrSc 3 ['Sc0.125Ge0.5Ir0.375', '-0.69', '-0.209500619167']
GeIrSm 4 ['Ge0.555556Sm0.111111Ir0.333333', '-0.781', '-0.234079501325']
GeIrSr 5 ['Ge0.6Sr0.1Ir0.3', '-0.662', '-0.230626371404']
GeIrTb 1 ['Ge0.555556Tb0.111111Ir0.333333', '-0.666', '-0.202134778306']
GeIrTh 2 ['Ge0.5Ir0.4Th0.1', '-0.769', '-0.200599738404']
GeIrTm 4 ['Ge0.375Tm0.25Ir0.375', '-0.844', '-0.206303188638']
GeIrY 6 ['Ge0.375Y0.375Ir0.25', '-1.051', '-0.208586424792']
GeIrYb 5 ['Ge0.571429Yb0.142857Ir0.285714', '-0.686', '-0.207937965119']
GeKN 2 ['N0.444444K0.222222Ge0.333333', '-0.421', '-0.212789731602']
GeKPa 2 ['K0.111111Ge0.666667Pa0.222222', '-0.402', '-0.216047402464']
GeKTe 1 ['K0.125Ge0.25Te0.625', '-0.482', '-0.207530026511']
GeKrP 9 ['P0.555556Ge0.222222Kr0.222222', '-0.323', '-0.20235376817']
GeKrPa 3 ['Ge0.666667Kr0.111111Pa0.222222', '-0.393', '-0.251113601111']
GeLaN 2 ['N0.375Ge0.125La0.5', '-1.496', '-0.213116251748']
GeLaNi 1 ['Ni0.222222Ge0.444444La0.333333', '-1.004', '-0.225042766296']

GeLaPa 2 ['Ge0.7La0.1Pa0.2', '-0.563', '-0.229275785']
GeLaPt 4 ['Ge0.571429La0.142857Pt0.285714', '-0.985', '-0.285043858929']
GeLaRh 1 ['Ge0.375Rh0.25La0.375', '-1.103', '-0.210486800789']
GeLaRu 1 ['Ge0.5Ru0.375La0.125', '-0.751', '-0.200783690687']
GeLaS 9 ['S0.333333Ge0.222222La0.444444', '-1.972', '-0.203152887549']
GeLaSe 22 ['Ge0.1Se0.3La0.6', '-1.593', '-0.200024068']
GeLiN 4 ['Li0.375N0.375Ge0.25', '-0.488', '-0.200568944392']
GeLiPa 2 ['Li0.125Ge0.625Pa0.25', '-0.433', '-0.201816003281']
GeLiPm 6 ['Li0.2Ge0.3Pm0.5', '-0.585', '-0.203218388321']
GeLiSe 3 ['Li0.2Ge0.4Se0.4', '-0.72', '-0.204638713251']
GeLiSr 1 ['Li0.333333Ge0.444444Sr0.222222', '-0.721', '-0.219423314889']
GeLiTe 1 ['Li0.125Ge0.25Te0.625', '-0.484', '-0.244450140833']
GeLuN 5 ['N0.375Ge0.125Lu0.5', '-1.89', '-0.212151855497']
GeLuOs 2 ['Ge0.5Lu0.1Os0.4', '-0.412', '-0.20443190425']
GeLuPa 2 ['Ge0.666667Lu0.111111Pa0.222222', '-0.549', '-0.217669784444']
GeMgPa 3 ['Mg0.125Ge0.625Pa0.25', '-0.419', '-0.207233577917']
GeMgPm 1 ['Mg0.125Ge0.375Pm0.5', '-0.701', '-0.213688786979']
GeMgPt 1 ['Mg0.222222Ge0.111111Pt0.666667', '-0.841', '-0.240933994629']
GeMgRh 4 ['Mg0.2Ge0.4Rh0.4', '-0.725', '-0.203599521167']
GeMnNb 2 ['Mn0.428571Ge0.428571Nb0.142857', '-0.376', '-0.2063364802']
GeMnO 1 ['O0.6Mn0.3Ge0.1', '-2.361', '-0.33957956806']
GeMnP 2 ['P0.3Mn0.5Ge0.2', '-0.714', '-0.207317859915']
GeMnSc 1 ['Sc0.5Mn0.1Ge0.4', '-0.96', '-0.202968786787']
GeMnTa 1 ['Mn0.4Ge0.4Ta0.2', '-0.352', '-0.20114438776']
GeMoO 7 ['O0.666667Ge0.222222Mo0.111111', '-2.195', '-0.217498818303']
GeMoP 10 ['P0.285714Ge0.285714Mo0.428571', '-0.725', '-0.208070299226']
GeMoPa 4 ['Ge0.7Mo0.1Pa0.2', '-0.381', '-0.20394409825']
GeNNp 14 ['N0.444444Ge0.333333Np0.222222', '-0.805', '-0.204182821235']
GeNP 1 ['N0.25P0.375Ge0.375', '-0.46', '-0.201765982679']
GeNPa 29 ['N0.5Ge0.375Pa0.125', '-0.572', '-0.208862752119']
GeNPu 2 ['N0.571429Ge0.142857Pu0.285714', '-1.054', '-0.218546869837']
GeNRb 7 ['N0.5Ge0.2Rb0.3', '-0.405', '-0.210680250661']
GeNSc 9 ['N0.555556Sc0.333333Ge0.111111', '-1.601', '-0.212638905391']
GeNSr 2 ['N0.3Ge0.5Sr0.2', '-0.612', '-0.209705062147']
GeNTb 1 ['N0.5Ge0.3Tb0.2', '-0.898', '-0.239864691893']
GeNTi 14 ['N0.4Ti0.5Ge0.1', '-1.518', '-0.202378985969']
GeNW 5 ['N0.5Ge0.1W0.4', '-0.374', '-0.203854205828']
GeNYb 34 ['N0.333333Ge0.5Yb0.166667', '-0.558', '-0.20379517126']
GeNaPa 8 ['Na0.142857Ge0.714286Pa0.142857', '-0.354', '-0.200951413003']
GeNaPm 3 ['Na0.111111Ge0.333333Pm0.555556', '-0.613', '-0.202657267405']
GeNaRh 3 ['Na0.1Ge0.4Rh0.5', '-0.706', '-0.2308025655']
GeNbO 1 ['O0.625Ge0.25Nb0.125', '-2.328', '-0.202063679293']
GeNbP 1 ['P0.333333Ge0.166667Nb0.5', '-1.111', '-0.225178183759']
GeNbPa 3 ['Ge0.7Nb0.1Pa0.2', '-0.434', '-0.203624567667']

GeNbRu 2 ['Ge0.375Nb0.25Ru0.375', '-0.596', '-0.208497788438']
GeNdPt 4 ['Ge0.571429Nd0.142857Pt0.285714', '-0.819', '-0.213375884048']
GeNdRu 1 ['Ge0.5Ru0.375Nd0.125', '-0.742', '-0.205988376313']
GeNdS 2 ['S0.4Ge0.2Nd0.4', '-1.972', '-0.215708220107']
GeNiPa 1 ['Ni0.111111Ge0.666667Pa0.222222', '-0.427', '-0.215287505278']
GeNiSe 35 ['Ni0.333333Ge0.5Se0.166667', '-0.486', '-0.202226182083']
GeNiY 1 ['Ni0.25Ge0.375Y0.375', '-0.98', '-0.225172121419']
GeNiZr 4 ['Ni0.375Ge0.375Zr0.25', '-0.754', '-0.201502981875']
GeNpP 10 ['P0.428571Ge0.285714Np0.285714', '-0.813', '-0.217220681041']
GeNpPa 2 ['Ge0.666667Pa0.222222Np0.111111', '-0.456', '-0.217109308472']
GeNpS 6 ['S0.444444Ge0.111111Np0.444444', '-1.543', '-0.200324328865']
GeNpSe 2 ['Ge0.4Se0.5Np0.1', '-0.64', '-0.201887586875']
GeOOs 3 ['O0.666667Ge0.222222Os0.111111', '-1.852', '-0.20561868719']
GeOP 2 ['O0.5P0.3Ge0.2', '-1.907', '-0.206362997347']
GeOPa 4 ['O0.714286Ge0.142857Pa0.142857', '-2.435', '-0.297432085211']
GeOPr 1 ['O0.625Ge0.125Pr0.25', '-3.187', '-0.243635098042']
GeORe 5 ['O0.7Ge0.1Re0.2', '-2.04', '-0.200628779635']
GeOS 2 ['O0.666667S0.166667Ge0.166667', '-1.817', '-0.233937488617']
GeOSc 3 ['O0.1Sc0.6Ge0.3', '-1.521', '-0.210539707745']
GeOSr 2 ['O0.5Ge0.166667Sr0.333333', '-2.795', '-0.206121179143']
GeOTa 4 ['O0.625Ge0.25Ta0.125', '-2.456', '-0.201120069918']
GeOTc 7 ['O0.625Ge0.125Tc0.25', '-1.88', '-0.215151066479']
GeOTh 1 ['O0.666667Ge0.111111Th0.222222', '-3.712', '-0.223100237473']
GeOTi 8 ['O0.166667Ti0.666667Ge0.166667', '-1.534', '-0.203161415524']
GeOV 3 ['O0.625V0.25Ge0.125', '-2.571', '-0.21863126688']
GeOW 7 ['O0.6Ge0.3W0.1', '-2.068', '-0.239705268222']
GeOsPa 1 ['Ge0.666667Os0.111111Pa0.222222', '-0.371', '-0.201655333889']
GeOsSc 2 ['Sc0.142857Ge0.428571Os0.428571', '-0.516', '-0.211117376428']
GeOsSe 12 ['Ge0.3Se0.6Os0.1', '-0.433', '-0.200068015']
GeOsZr 1 ['Ge0.4Zr0.2Os0.4', '-0.598', '-0.203124207834']
GePPm 19 ['P0.375Ge0.25Pm0.375', '-1.101', '-0.207173808203']
GePPu 12 ['P0.5Ge0.4Pu0.1', '-0.549', '-0.204115743187']
GePRh 1 ['P0.8Ge0.1Rh0.1', '-0.58', '-0.206339207374']
GePS 10 ['P0.333333S0.5Ge0.166667', '-0.643', '-0.206145941796']
GePSc 2 ['P0.25Sc0.5Ge0.25', '-1.442', '-0.21777426026']
GePSe 15 ['P0.3Ge0.2Se0.5', '-0.435', '-0.210204838547']
GePSi 1 ['Si0.2P0.6Ge0.2', '-0.421', '-0.203673551957']
GePSm 2 ['P0.4Ge0.2Sm0.4', '-1.563', '-0.203078501416']
GePSn 1 ['P0.8Ge0.1Sn0.1', '-0.403', '-0.250036321939']
GePSr 1 ['P0.8Ge0.1Sr0.1', '-0.663', '-0.216172489131']
GePTa 9 ['P0.5Ge0.125Ta0.375', '-0.743', '-0.212365367759']
GePTc 2 ['P0.285714Ge0.285714Tc0.428571', '-0.587', '-0.210149808409']
GePTe 8 ['P0.444444Ge0.222222Te0.333333', '-0.315', '-0.205475662808']
GePTi 7 ['P0.375Ti0.5Ge0.125', '-1.36', '-0.207631741803']

GePTm 2 ['P0.444444Ge0.222222Tm0.333333', '-1.393', '-0.214118260879']
GePU 1 ['P0.8Ge0.1U0.1', '-0.591', '-0.23512491427']
GePV 3 ['P0.3V0.5Ge0.2', '-0.914', '-0.212334785312']
GePXe 3 ['P0.7Ge0.1Xe0.2', '-0.274', '-0.208851034812']
GePY 8 ['P0.555556Ge0.333333Y0.111111', '-0.704', '-0.2042480828']
GePYb 2 ['P0.5Ge0.1Yb0.4', '-1.075', '-0.227848069645']
GePZr 2 ['P0.333333Ge0.222222Zr0.444444', '-1.429', '-0.225408970347']
GePaPr 2 ['Ge0.7Pr0.1Pa0.2', '-0.574', '-0.25949099125']
GePaPu 2 ['Ge0.7Pa0.2Pu0.1', '-0.502', '-0.2256305945']
GePaRb 3 ['Ge0.75Rb0.125Pa0.125', '-0.285', '-0.205188900625']
GePaRe 2 ['Ge0.666667Re0.111111Pa0.222222', '-0.356', '-0.214017523704']
GePaRh 1 ['Ge0.666667Rh0.111111Pa0.222222', '-0.49', '-0.224695176944']
GePaRu 3 ['Ge0.444444Ru0.444444Pa0.111111', '-0.551', '-0.200023215429']
GePaS 9 ['S0.625Ge0.125Pa0.25', '-1.418', '-0.201297858271']
GePaSc 3 ['Sc0.166667Ge0.666667Pa0.166667', '-0.605', '-0.213859875']
GePaSe 16 ['Ge0.1Se0.7Pa0.2', '-0.768', '-0.203880054375']
GePaSm 3 ['Ge0.625Sm0.125Pa0.25', '-0.614', '-0.206608304271']
GePaSr 2 ['Ge0.666667Sr0.111111Pa0.222222', '-0.546', '-0.238812991111']
GePaTa 2 ['Ge0.666667Ta0.111111Pa0.222222', '-0.437', '-0.221743188148']
GePaTc 14 ['Ge0.5Tc0.4Pa0.1', '-0.321', '-0.2071109125']
GePaV 2 ['V0.1Ge0.7Pa0.2', '-0.423', '-0.247128742333']
GePaW 4 ['Ge0.6W0.1Pa0.3', '-0.436', '-0.2444533615']
GePaY 2 ['Ge0.7Y0.1Pa0.2', '-0.561', '-0.2414869605']
GePaYb 4 ['Ge0.714286Yb0.142857Pa0.142857', '-0.516', '-0.200171335953']
GePaZn 3 ['Zn0.111111Ge0.666667Pa0.222222', '-0.365', '-0.223113601111']
GePbSc 1 ['Sc0.6Ge0.3Pb0.1', '-0.959', '-0.203003928917']
GePbTe 2 ['Ge0.125Te0.625Pb0.25', '-0.43', '-0.203932110625']
GePdPm 3 ['Ge0.333333Pd0.222222Pm0.444444', '-0.859', '-0.204819829815']
GePdZr 1 ['Ge0.333333Zr0.222222Pd0.444444', '-0.904', '-0.209112880556']
GePmPt 4 ['Ge0.6Pm0.1Pt0.3', '-0.683', '-0.21528439075']
GePmS 16 ['S0.25Ge0.25Pm0.5', '-1.444', '-0.202491963995']
GePmSe 28 ['Ge0.333333Se0.5Pm0.166667', '-0.892', '-0.203082205417']
GePmTe 6 ['Ge0.25Te0.625Pm0.125', '-0.571', '-0.200835863437']
GePrRu 1 ['Ge0.555556Ru0.333333Pr0.111111', '-0.709', '-0.211638522579']
GePrS 1 ['S0.375Ge0.25Pr0.375', '-1.897', '-0.266883140056']
GePrSe 6 ['Ge0.333333Se0.222222Pr0.444444', '-1.417', '-0.201843481111']
GePtS 23 ['S0.571429Ge0.285714Pt0.142857', '-0.732', '-0.20953519229']
GePtSm 5 ['Ge0.1Sm0.1Pt0.8', '-0.698', '-0.20508628425']
GePtTb 9 ['Ge0.4Tb0.2Pt0.4', '-0.943', '-0.204844177567']
GePtTh 6 ['Ge0.5Pt0.4Th0.1', '-0.881', '-0.20848372']
GePtTm 4 ['Ge0.5Tm0.166667Pt0.333333', '-0.88', '-0.228085155417']
GePtY 5 ['Ge0.6Y0.1Pt0.3', '-0.758', '-0.201895986917']
GePtYb 1 ['Ge0.5Yb0.125Pt0.375', '-0.91', '-0.260723409375']
GePuRu 4 ['Ge0.5Ru0.4Pu0.1', '-0.555', '-0.2326323885']

GePuS 13 ['S0.6Ge0.1Pu0.3', '-1.701', '-0.207487036778']
GePuSc 1 ['Sc0.5Ge0.4Pu0.1', '-0.991', '-0.226340342']
GePuSe 2 ['Ge0.375Se0.5Pu0.125', '-0.801', '-0.205467608124']
GePuTe 1 ['Ge0.1Te0.4Pu0.5', '-1.173', '-0.200424927164']
GeRhSr 1 ['Ge0.5Sr0.1Rh0.4', '-0.829', '-0.2105674965']
GeRhZr 1 ['Ge0.3Zr0.3Rh0.4', '-0.96', '-0.202407317375']
GeRuSe 3 ['Ge0.444444Se0.333333Ru0.222222', '-0.52', '-0.205123779528']
GeRuSm 2 ['Ge0.5Ru0.375Sm0.125', '-0.75', '-0.211979468125']
GeRuYb 2 ['Ge0.5Ru0.333333Yb0.166667', '-0.707', '-0.201046751094']
GeRuZr 1 ['Ge0.333333Zr0.166667Ru0.5', '-0.71', '-0.216919007639']
GeSSc 1 ['S0.142857Sc0.571429Ge0.285714', '-1.404', '-0.226272504426']
GeSSm 16 ['S0.3Ge0.4Sm0.3', '-1.523', '-0.202236420462']
GeSTc 5 ['S0.5Ge0.2Tc0.3', '-0.821', '-0.209408161993']
GeSTm 4 ['S0.333333Ge0.222222Tm0.444444', '-1.831', '-0.205460926578']
GeSU 8 ['S0.5Ge0.2U0.3', '-1.514', '-0.215964576496']
GeSXe 1 ['S0.5Ge0.1Xe0.4', '-0.372', '-0.216174603322']
GeSY 6 ['S0.3Ge0.2Y0.5', '-1.862', '-0.219226458294']
GeSZr 11 ['S0.3Ge0.2Zr0.5', '-1.416', '-0.200515388794']
GeScSe 36 ['Sc0.6Ge0.3Se0.1', '-1.206', '-0.211302311']
GeSeSm 1 ['Ge0.375Se0.25Sm0.375', '-1.436', '-0.232214816702']
GeSeSr 1 ['Ge0.3Se0.3Sr0.4', '-1.61', '-0.217678394']
GeSeTb 2 ['Ge0.25Se0.375Tb0.375', '-1.394', '-0.224169068665']
GeSeTc 2 ['Ge0.142857Se0.571429Tc0.285714', '-0.325', '-0.207128851964']
GeSeTh 15 ['Ge0.111111Se0.555556Th0.333333', '-1.761', '-0.207238075275']
GeSeTm 8 ['Ge0.3Se0.3Tm0.4', '-1.382', '-0.2106410455']
GeSeY 36 ['Ge0.125Se0.375Y0.5', '-1.757', '-0.2087122125']
GeSeYb 11 ['Ge0.142857Se0.428571Yb0.428571', '-2.011', '-0.205720535714']
GeSeZn 3 ['Zn0.333333Ge0.111111Se0.555556', '-0.768', '-0.202599091111']
GeSeZr 6 ['Ge0.142857Se0.428571Zr0.428571', '-1.349', '-0.205124913214']
GeTeY 1 ['Ge0.1Y0.4Te0.5', '-1.368', '-0.222613424533']
HHfN 2 ['H0.1N0.4Hf0.5', '-1.763', '-0.206409782896']
HHfNa 3 ['H0.5Na0.125Hf0.375', '-0.593', '-0.228364815158']
HHfPm 4 ['H0.7Pm0.2Hf0.1', '-0.609', '-0.215296711221']
HHfRh 3 ['H0.142857Rh0.285714Hf0.571429', '-0.798', '-0.200391794331']
HHfS 20 ['H0.444444S0.222222Hf0.333333', '-1.129', '-0.215075325359']
HHfSe 1 ['H0.125Se0.5Hf0.375', '-1.386', '-0.241170919102']
HHfSi 1 ['H0.444444Si0.333333Hf0.222222', '-0.598', '-0.226678769444']
HHgO 1 ['H0.375O0.375Hg0.25', '-1.044', '-0.20771234214']
HHgPm 7 ['H0.555556Pm0.222222Hg0.222222', '-0.522', '-0.201803671753']
HHgS 2 ['H0.6S0.3Hg0.1', '-0.421', '-0.206689436236']
HHgY 1 ['H0.5Y0.25Hg0.25', '-0.734', '-0.206914960158']
HHgYb 3 ['H0.5Yb0.166667Hg0.333333', '-0.51', '-0.202189326772']
HHoI 48 ['H0.5I0.2Ho0.3', '-0.921', '-0.208526776811']
HHoIr 1 ['H0.666667Ho0.166667Ir0.166667', '-0.611', '-0.20284738876']

HHoN 31 ['H0.666667N0.111111Ho0.222222', '-0.899', '-0.205664861932']
HHoO 15 ['H0.444444O0.222222Ho0.333333', '-2.053', '-0.20697579084']
HHoOs 1 ['H0.666667Ho0.222222Os0.111111', '-0.743', '-0.203293455211']
HHoPm 5 ['H0.571429Pm0.285714Ho0.142857', '-0.648', '-0.209041594704']
HHoRh 12 ['H0.6Rh0.3Ho0.1', '-0.446', '-0.203132054845']
HHoS 31 ['H0.1S0.5Ho0.4', '-2.205', '-0.201037132941']
HHoSi 20 ['H0.625Si0.125Ho0.25', '-0.788', '-0.206597005475']
HHoTe 3 ['H0.125Te0.5Ho0.375', '-1.294', '-0.21432059174']
HIIr 1 ['H0.5I0.375Ir0.125', '-0.276', '-0.226025910145']
HILu 58 ['H0.666667I0.111111Lu0.222222', '-0.673', '-0.203861840183']
HIMo 9 ['H0.4Mo0.2I0.4', '-0.508', '-0.207918128862']
HINd 3 ['H0.125I0.625Nd0.25', '-1.297', '-0.215056337581']
HINi 16 ['H0.25Ni0.375I0.375', '-0.347', '-0.20526816368']
HINp 8 ['H0.222222I0.555556Np0.222222', '-0.979', '-0.201328027944']
HIOs 1 ['H0.555556I0.333333Os0.111111', '-0.248', '-0.203578586796']
HIP 2 ['H0.5P0.166667I0.333333', '-0.384', '-0.216893532994']
HIPa 63 ['H0.7I0.2Pa0.1', '-0.382', '-0.210361358922']
HIPd 2 ['H0.5Pd0.25I0.25', '-0.377', '-0.227544687557']
HIPm 73 ['H0.714286I0.142857Pm0.142857', '-0.461', '-0.205393286439']
HIPr 6 ['H0.5I0.2Pr0.3', '-1.044', '-0.200302724573']
HIPt 15 ['H0.375I0.375Pt0.25', '-0.356', '-0.206436904089']
HIPu 2 ['H0.4I0.3Pu0.3', '-0.889', '-0.201885168399']
HIRe 12 ['H0.166667I0.666667Re0.166667', '-0.349', '-0.201110537461']
HIRh 17 ['H0.5Rh0.1I0.4', '-0.258', '-0.204694304155']
HIRu 24 ['H0.4Ru0.3I0.3', '-0.25', '-0.210020728116']
HIS 2 ['H0.555556S0.222222I0.222222', '-0.39', '-0.216444296514']
HISb 5 ['H0.3Sb0.2I0.5', '-0.534', '-0.209920202953']
HISc 2 ['H0.5Sc0.375I0.125', '-0.949', '-0.214706726416']
HISi 3 ['H0.111111Si0.222222I0.666667', '-0.658', '-0.21588139727']
HISm 41 ['H0.5I0.2Sm0.3', '-1.004', '-0.208909473254']
HITb 61 ['H0.3I0.3Tb0.4', '-0.642', '-0.213070882583']
HITc 8 ['H0.6Tc0.1I0.3', '-0.26', '-0.220020728116']
HITe 22 ['H0.333333Te0.333333I0.333333', '-0.315', '-0.203122780579']
HITm 38 ['H0.1I0.6Tm0.3', '-1.327', '-0.200190281539']
HIV 4 ['H0.5V0.125I0.375', '-0.431', '-0.201317344441']
HIW 6 ['H0.444444I0.444444W0.111111', '-0.264', '-0.204771449061']
HIXe 34 ['H0.25I0.5Xe0.25', '-0.235', '-0.201683940097']
HInK 5 ['H0.555556K0.333333In0.111111', '-0.347', '-0.20633908961']
HInLi 4 ['H0.428571Li0.428571In0.142857', '-0.566', '-0.215038171921']
HInO 5 ['H0.2O0.5In0.3', '-1.824', '-0.233532084911']
HInPm 14 ['H0.6In0.2Pm0.2', '-0.504', '-0.20658094219']
HInPu 1 ['H0.375In0.25Pu0.375', '-0.525', '-0.241831586994']
HInRb 7 ['H0.5Rb0.375In0.125', '-0.335', '-0.205448736437']
HInTm 4 ['H0.5In0.25Tm0.25', '-0.73', '-0.203315404186']

HInY 8 ['H0.375Y0.375In0.25', '-0.807', '-0.200364652618']
HInYb 8 ['H0.5In0.125Yb0.375', '-0.821', '-0.229511371408']
HIrK 2 ['H0.666667K0.166667Ir0.166667', '-0.314', '-0.248042690469']
HIrMg 1 ['H0.666667Mg0.166667Ir0.166667', '-0.323', '-0.205006549983']
HIrNd 3 ['H0.7Nd0.2Ir0.1', '-0.65', '-0.210591251356']
HIrPm 18 ['H0.625Pm0.125Ir0.25', '-0.395', '-0.209113088868']
HIrPr 5 ['H0.625Pr0.25Ir0.125', '-0.702', '-0.202087984084']
HIrRb 2 ['H0.714286Rb0.142857Ir0.142857', '-0.262', '-0.216656345402']
HIrSc 1 ['H0.625Sc0.25Ir0.125', '-0.734', '-0.213353854507']
HIrSm 4 ['H0.625Sm0.25Ir0.125', '-0.737', '-0.205881259052']
HIrSr 11 ['H0.666667Sr0.111111Ir0.222222', '-0.41', '-0.22992351007']
HIrTb 5 ['H0.625Tb0.25Ir0.125', '-0.645', '-0.20726613948']
HIrTm 5 ['H0.625Tm0.25Ir0.125', '-0.816', '-0.204861638459']
HIrY 6 ['H0.7Y0.2Ir0.1', '-0.691', '-0.209394399023']
HIrYb 14 ['H0.7Yb0.1Ir0.2', '-0.388', '-0.2032931545']
HKMn 3 ['H0.571429K0.142857Mn0.285714', '-0.29', '-0.233465163259']
HKO 2 ['H0.4O0.2K0.4', '-1.183', '-0.223011272949']
HKP 13 ['H0.5P0.166667K0.333333', '-0.456', '-0.203314064966']
HKPb 4 ['H0.571429K0.285714Pb0.142857', '-0.331', '-0.204530362635']
HKPd 2 ['H0.6K0.1Pd0.3', '-0.352', '-0.215606584017']
HKPm 7 ['H0.6K0.2Pm0.2', '-0.503', '-0.20558094219']
HKPt 3 ['H0.571429K0.142857Pt0.285714', '-0.458', '-0.202038420492']
HKRh 5 ['H0.625K0.25Rh0.125', '-0.305', '-0.206064035704']
HKS 1 ['H0.285714S0.285714K0.428571', '-1.077', '-0.203667763543']
HKSb 4 ['H0.5K0.333333Sb0.166667', '-0.434', '-0.218092757227']
HKSe 11 ['H0.333333K0.444444Se0.222222', '-0.949', '-0.208531386667']
HKSi 13 ['H0.555556Si0.111111K0.333333', '-0.362', '-0.201413014897']
HKSn 7 ['H0.428571K0.428571Sn0.142857', '-0.423', '-0.210670546433']
HKrO 1 ['H0.333333O0.444444Kr0.222222', '-0.799', '-0.20710381371']
HKrPm 14 ['H0.4Kr0.2Pm0.4', '-0.401', '-0.202720628126']
HLaMn 10 ['H0.428571Mn0.285714La0.285714', '-0.611', '-0.204748690493']
HLaN 20 ['H0.666667N0.111111La0.222222', '-0.825', '-0.201355679617']
HLaO 10 ['H0.5O0.166667La0.333333', '-1.708', '-0.209298765847']
HLaOs 1 ['H0.666667La0.222222Os0.111111', '-0.747', '-0.237693169655']
HLaPm 3 ['H0.7La0.1Pm0.2', '-0.651', '-0.213403645409']
HLaPt 9 ['H0.625La0.125Pt0.25', '-0.608', '-0.202513563751']
HLaPu 7 ['H0.555556La0.333333Pu0.111111', '-0.731', '-0.20437793212']
HLaRh 4 ['H0.571429Rh0.285714La0.142857', '-0.536', '-0.208588466207']
HLaS 20 ['H0.222222S0.444444La0.333333', '-2.043', '-0.201931107231']
HLaSe 4 ['H0.1Se0.4La0.5', '-1.895', '-0.200291115782']
HLaSi 22 ['H0.375Si0.375La0.25', '-0.737', '-0.200805436681']
HLaSn 2 ['H0.571429Sn0.142857La0.285714', '-0.83', '-0.201178396773']
HLiO 2 ['H0.5Li0.4O0.1', '-0.96', '-0.202341447976']
HLiP 1 ['H0.444444Li0.444444P0.111111', '-0.637', '-0.206522287095']

HLiPb 1 ['H0.444444Li0.444444Pb0.111111', '-0.575', '-0.211039585696']
HLiPm 13 ['H0.5Li0.3Pm0.2', '-0.547', '-0.202187034408']
HLiPu 22 ['H0.4Li0.5Pu0.1', '-0.533', '-0.205435627126']
HLiSb 13 ['H0.5Li0.3Sb0.2', '-0.463', '-0.20059377425']
HLiSe 7 ['H0.333333Li0.333333Se0.333333', '-0.838', '-0.201951113335']
HLiSi 22 ['H0.333333Li0.333333Si0.333333', '-0.483', '-0.210029689272']
HLiSn 6 ['H0.5Li0.375Sn0.125', '-0.527', '-0.216394497309']
HLiTe 10 ['H0.5Li0.375Te0.125', '-0.701', '-0.207336039309']
HLiTl 1 ['H0.5Li0.4Tl0.1', '-0.568', '-0.240435627126']
HLiYb 1 ['H0.444444Li0.444444Yb0.111111', '-0.598', '-0.210812677363']
HLuN 25 ['H0.4N0.4Lu0.2', '-1.198', '-0.204969281679']
HLuPd 1 ['H0.6Pd0.2Lu0.2', '-0.746', '-0.224051414417']
HLuPm 3 ['H0.6Pm0.3Lu0.1', '-0.616', '-0.21327166819']
HLuRh 31 ['H0.333333Rh0.333333Lu0.333333', '-0.928', '-0.200905669272']
HLuRu 3 ['H0.625Ru0.125Lu0.25', '-0.714', '-0.202877600158']
HLuS 20 ['H0.625S0.125Lu0.25', '-1.009', '-0.200427413874']
HLuSi 15 ['H0.1Si0.4Lu0.5', '-0.967', '-0.206513974282']
HMgO 7 ['H0.444444O0.333333Mg0.222222', '-1.917', '-0.200516075518']
HMgPm 12 ['H0.5Mg0.1Pm0.4', '-0.476', '-0.205901959991']
HMgRh 31 ['H0.375Mg0.25Rh0.375', '-0.448', '-0.200054254583']
HMgU 3 ['H0.571429Mg0.142857U0.285714', '-0.411', '-0.208581875656']
HMnNa 6 ['H0.625Na0.125Mn0.25', '-0.266', '-0.20383641212']
HMnNd 8 ['H0.625Mn0.25Nd0.125', '-0.478', '-0.203369532097']
HMnPm 20 ['H0.555556Mn0.333333Pm0.111111', '-0.367', '-0.201767190105']
HMnPr 6 ['H0.6Mn0.2Pr0.2', '-0.601', '-0.20127949519']
HMnRb 1 ['H0.625Mn0.25Rb0.125', '-0.249', '-0.209324302227']
HMnRh 2 ['H0.4Mn0.2Rh0.4', '-0.319', '-0.232448145241']
HMnS 3 ['H0.571429S0.142857Mn0.285714', '-0.389', '-0.206416800313']
HMnSb 3 ['H0.4Mn0.3Sb0.3', '-0.226', '-0.203612039862']
HMnSe 8 ['H0.333333Mn0.333333Se0.333333', '-0.443', '-0.200822792069']
HMnSm 6 ['H0.571429Mn0.142857Sm0.285714', '-0.765', '-0.204836988038']
HMnSn 17 ['H0.25Mn0.5Sn0.25', '-0.23', '-0.203575748563']
HMnTl 19 ['H0.571429Mn0.142857Tl0.285714', '-0.213', '-0.213']
HMnY 1 ['H0.625Mn0.25Y0.125', '-0.503', '-0.20199649939']
HMoN 15 ['H0.3N0.4Mo0.3', '-0.638', '-0.205781994957']
HMoO 2 ['H0.25O0.5Mo0.25', '-1.883', '-0.227010191204']
HMoPm 3 ['H0.666667Mo0.111111Pm0.222222', '-0.531', '-0.200534380211']
HMoS 7 ['H0.428571S0.285714Mo0.285714', '-0.656', '-0.203792220997']
HNNb 15 ['H0.2N0.5Nb0.3', '-1.049', '-0.203796537547']
HNNd 6 ['H0.555556N0.222222Nd0.222222', '-0.969', '-0.247423113186']
HNNp 38 ['H0.375N0.25Np0.375', '-0.915', '-0.203772898312']
HNPa 40 ['H0.285714N0.571429Pa0.142857', '-0.625', '-0.200152453834']
HNPm 44 ['H0.666667N0.222222Pm0.111111', '-0.711', '-0.202094795585']
HNPr 13 ['H0.571429N0.142857Pr0.285714', '-0.917', '-0.216577223941']

HNPu 19 ['H0.333333N0.444444Pu0.222222', '-0.982', '-0.207160585269']
HNRe 20 ['H0.4N0.3Re0.3', '-0.442', '-0.207523944713']
HNSm 25 ['H0.666667N0.222222Sm0.111111', '-0.76', '-0.207082252542']
HNTa 17 ['H0.142857N0.428571Ta0.428571', '-1.299', '-0.20034751914']
HNTb 12 ['H0.4N0.3Tb0.3', '-1.094', '-0.207386290343']
HNTc 9 ['H0.222222N0.444444Tc0.333333', '-0.4', '-0.201882738969']
HNTh 13 ['H0.333333N0.444444Th0.222222', '-1.269', '-0.205281977465']
HNTi 15 ['H0.1N0.4Ti0.5', '-1.575', '-0.228060955928']
HNTm 15 ['H0.4N0.3Tm0.3', '-1.373', '-0.211105337635']
HNU 25 ['H0.555556N0.222222U0.222222', '-0.841', '-0.202020149715']
HNW 25 ['H0.2N0.3W0.5', '-0.394', '-0.211205010627']
HNY 11 ['H0.6N0.2Y0.2', '-0.954', '-0.204445984288']
HNYb 39 ['H0.444444N0.444444Yb0.111111', '-0.623', '-0.207251834878']
HNaPd 2 ['H0.571429Na0.142857Pd0.285714', '-0.375', '-0.206843135215']
HNaPm 13 ['H0.5Na0.25Pm0.25', '-0.471', '-0.22274821682']
HNaPu 2 ['H0.555556Na0.111111Pu0.333333', '-0.497', '-0.213747854342']
HNaSc 2 ['H0.555556Na0.111111Sc0.333333', '-0.794', '-0.234619625176']
HNaSi 4 ['H0.714286Na0.142857Si0.142857', '-0.279', '-0.207955899566']
HNbO 1 ['H0.125O0.5Nb0.375', '-2.336', '-0.224995028793']
HNbPm 7 ['H0.571429Nb0.142857Pm0.285714', '-0.49', '-0.206743754466']
HNbS 8 ['H0.555556S0.222222Nb0.222222', '-0.734', '-0.201510709699']
HNdO 5 ['H0.4O0.2Nd0.4', '-1.785', '-0.201200808116']
HNdPd 4 ['H0.444444Pd0.222222Nd0.333333', '-0.887', '-0.203250522362']
HNdPm 3 ['H0.6Nd0.1Pm0.3', '-0.589', '-0.202225650189']
HNdPt 17 ['H0.5Nd0.166667Pt0.333333', '-0.723', '-0.201010201665']
HNdRh 29 ['H0.428571Rh0.285714Nd0.285714', '-0.797', '-0.201292738111']
HNdRu 5 ['H0.666667Ru0.166667Nd0.166667', '-0.569', '-0.202826042797']
HNdS 8 ['H0.285714S0.285714Nd0.428571', '-1.73', '-0.219169537513']
HNdSe 2 ['H0.166667Se0.5Nd0.333333', '-1.703', '-0.209258685833']
HNdSi 6 ['H0.555556Si0.222222Nd0.222222', '-0.676', '-0.202705247367']
HNiPm 25 ['H0.428571Ni0.142857Pm0.428571', '-0.492', '-0.201451249957']
HNiS 20 ['H0.25S0.375Ni0.375', '-0.606', '-0.208189677012']
HNiSe 28 ['H0.166667Ni0.333333Se0.5', '-0.43', '-0.200808659375']
HNpO 12 ['H0.1O0.4Np0.5', '-2.549', '-0.20045729261']
HNpPm 6 ['H0.555556Pm0.333333Np0.111111', '-0.491', '-0.215611983509']
HNpPt 2 ['H0.571429Pt0.285714Np0.142857', '-0.33', '-0.20010286375']
HNpS 16 ['H0.571429S0.285714Np0.142857', '-0.852', '-0.204337607872']
HNpSe 24 ['H0.25Se0.5Np0.25', '-0.888', '-0.20134438448']
HNpSi 20 ['H0.666667Si0.166667Np0.166667', '-0.35', '-0.202680200869']
HOPa 21 ['H0.444444O0.444444Pa0.111111', '-2.026', '-0.203871691973']
HOPm 18 ['H0.5O0.25Pm0.25', '-1.872', '-0.216143338921']
HOPr 4 ['H0.2O0.4Pr0.4', '-2.779', '-0.210930624626']
HOPu 16 ['H0.2O0.4Pu0.4', '-2.734', '-0.202555724899']
HORb 4 ['H0.3O0.2Rb0.5', '-1.097', '-0.209059341168']

HOSb 9 ['H0.5O0.3Sb0.2', '-1.229', '-0.209519615438']
HOSm 14 ['H0.375O0.25Sm0.375', '-2.111', '-0.201296883908']
HOSr 5 ['H0.375O0.25Sr0.375', '-1.876', '-0.201359247359']
HOTa 7 ['H0.4O0.3Ta0.3', '-1.577', '-0.209331328549']
HOTb 12 ['H0.2O0.4Tb0.4', '-2.731', '-0.21699307067']
HOTc 1 ['H0.428571O0.428571Tc0.142857', '-1.509', '-0.201403717527']
HOTi 2 ['H0.25O0.25Ti0.5', '-1.826', '-0.210439816091']
HOTm 7 ['H0.5O0.166667Tm0.333333', '-1.78', '-0.20727938177']
HOU 8 ['H0.222222O0.444444U0.333333', '-2.828', '-0.20308264458']
HOW 8 ['H0.625O0.25W0.125', '-1.097', '-0.209155720565']
HOY 2 ['H0.4O0.2Y0.4', '-1.917', '-0.22593384795']
HOZr 5 ['H0.5O0.25Zr0.25', '-1.773', '-0.201353099233']
HOsPm 18 ['H0.4Pm0.5Os0.1', '-0.401', '-0.202720628126']
HOsPu 2 ['H0.428571Os0.142857Pu0.428571', '-0.431', '-0.201475214136']
HOsS 11 ['H0.375S0.375Os0.25', '-0.514', '-0.203105679901']
HOsSc 7 ['H0.142857Sc0.571429Os0.285714', '-0.527', '-0.200669710759']
HOsSe 15 ['H0.5Se0.375Os0.125', '-0.272', '-0.203235561563']
HOsSr 1 ['H0.625Sr0.25Os0.125', '-0.632', '-0.226827897658']
HOsTb 1 ['H0.666667Tb0.222222Os0.111111', '-0.613', '-0.224494174915']
HOsY 12 ['H0.4Y0.4Os0.2', '-0.727', '-0.214271844876']
HPPb 11 ['H0.3P0.5Pb0.2', '-0.2', '-0.2']
HPPm 23 ['H0.5P0.166667Pm0.333333', '-0.848', '-0.202894699915']
HPRb 20 ['H0.25P0.25Rb0.5', '-0.528', '-0.202702153881']
HPSe 1 ['H0.3P0.3Se0.4', '-0.325', '-0.202616223187']
HPSr 10 ['H0.555556P0.222222Sr0.222222', '-0.731', '-0.2081746406']
HPTa 19 ['H0.2P0.5Ta0.3', '-0.602', '-0.201375749312']
HPTe 16 ['H0.222222P0.333333Te0.444444', '-0.202', '-0.202']
HPYb 4 ['H0.571429P0.142857Yb0.285714', '-0.78', '-0.20086890474']
HPaPm 7 ['H0.666667Pm0.166667Pa0.166667', '-0.531', '-0.202603122294']
HPaPu 1 ['H0.555556Pa0.222222Pu0.222222', '-0.498', '-0.217699841565']
HPaS 21 ['H0.142857S0.428571Pa0.428571', '-1.698', '-0.204445228277']
HPaSe 35 ['H0.555556Se0.222222Pa0.222222', '-0.737', '-0.20187762']
HPaSi 9 ['H0.375Si0.375Pa0.25', '-0.465', '-0.206733134181']
HPbPm 9 ['H0.5Pm0.333333Pb0.166667', '-0.611', '-0.217424556408']
HPbPu 13 ['H0.555556Pb0.111111Pu0.333333', '-0.517', '-0.208262532884']
HPbRb 11 ['H0.4Rb0.4Pb0.2', '-0.363', '-0.208867898438']
HPbS 2 ['H0.571429S0.285714Pb0.142857', '-0.5', '-0.202291881444']
HPbSe 10 ['H0.333333Se0.5Pb0.166667', '-0.396', '-0.206350948333']
HPdPm 43 ['H0.4Pd0.5Pm0.1', '-0.411', '-0.201465659876']
HPdPr 3 ['H0.6Pd0.3Pr0.1', '-0.505', '-0.20057485825']
HPdPu 3 ['H0.5Pd0.3Pu0.2', '-0.504', '-0.200927927908']
HPdYb 5 ['H0.666667Pd0.222222Yb0.111111', '-0.476', '-0.200916564468']
HPmPr 2 ['H0.666667Pr0.111111Pm0.222222', '-0.65', '-0.225219073266']
HPmPt 26 ['H0.714286Pm0.142857Pt0.142857', '-0.514', '-0.2033913814']

HPmPu 9 ['H0.5Pm0.375Pu0.125', '-0.459', '-0.207611927033']
HPmRb 6 ['H0.7Rb0.1Pm0.2', '-0.537', '-0.207840383971']
HPmRe 5 ['H0.7Pm0.2Re0.1', '-0.508', '-0.21058094219']
HPmRh 46 ['H0.3Rh0.5Pm0.2', '-0.488', '-0.202941737845']
HPmRu 21 ['H0.428571Ru0.142857Pm0.428571', '-0.446', '-0.202156199778']
HPmS 49 ['H0.666667S0.166667Pm0.166667', '-0.896', '-0.202044026883']
HPmSb 9 ['H0.5Sb0.25Pm0.25', '-0.665', '-0.201145140625']
HPmSc 10 ['H0.666667Sc0.222222Pm0.111111', '-0.772', '-0.214340493544']
HPmSe 54 ['H0.285714Se0.571429Pm0.142857', '-0.671', '-0.207862799643']
HPmSi 39 ['H0.1Si0.4Pm0.5', '-0.601', '-0.200518454032']
HPmSm 1 ['H0.666667Pm0.222222Sm0.111111', '-0.641', '-0.202848415488']
HPmSn 17 ['H0.5Sn0.25Pm0.25', '-0.556', '-0.201472769379']
HPmSr 5 ['H0.666667Sr0.166667Pm0.166667', '-0.694', '-0.258652455211']
HPmTa 3 ['H0.7Pm0.2Ta0.1', '-0.537', '-0.214341938305']
HPmTb 2 ['H0.6Pm0.3Tb0.1', '-0.58', '-0.241225385223']
HPmTc 12 ['H0.6Tc0.2Pm0.2', '-0.505', '-0.20758094219']
HPmTe 9 ['H0.571429Te0.142857Pm0.285714', '-0.794', '-0.208684302158']
HPmTh 5 ['H0.7Pm0.2Th0.1', '-0.623', '-0.229844262721']
HPmTi 8 ['H0.7Ti0.1Pm0.2', '-0.597', '-0.201166798342']
HPmTl 14 ['H0.5Pm0.4Tl0.1', '-0.529', '-0.202600719408']
HPmTm 2 ['H0.666667Pm0.222222Tm0.111111', '-0.669', '-0.218182329655']
HPmU 5 ['H0.666667Pm0.166667U0.166667', '-0.512', '-0.205112165558']
HPmV 5 ['H0.7V0.1Pm0.2', '-0.568', '-0.236959697305']
HPmW 3 ['H0.6Pm0.3W0.1', '-0.508', '-0.21058094219']
HPmXe 12 ['H0.666667Xe0.111111Pm0.222222', '-0.535', '-0.204534380211']
HPmY 1 ['H0.666667Y0.111111Pm0.222222', '-0.684', '-0.229429569099']
HPmZn 4 ['H0.571429Zn0.142857Pm0.285714', '-0.567', '-0.207279800181']
HPmZr 6 ['H0.7Zr0.1Pm0.2', '-0.62', '-0.204727993721']
HPrPt 16 ['H0.333333Pr0.333333Pt0.333333', '-1.023', '-0.204107009274']
HPrRh 21 ['H0.5Rh0.2Pr0.3', '-0.786', '-0.212128443658']
HPrRu 8 ['H0.571429Ru0.142857Pr0.285714', '-0.731', '-0.205218679466']
HPrS 9 ['H0.6S0.3Pr0.1', '-0.866', '-0.200203599015']
HPrSe 1 ['H0.166667Se0.5Pr0.333333', '-1.758', '-0.250082192891']
HPrSi 11 ['H0.444444Si0.222222Pr0.333333', '-0.817', '-0.208101863196']
HPrTe 2 ['H0.166667Te0.5Pr0.333333', '-1.376', '-0.210878428335']
HPtPu 4 ['H0.428571Pt0.285714Pu0.285714', '-0.773', '-0.202268366921']
HPtRb 1 ['H0.666667Rb0.111111Pt0.222222', '-0.396', '-0.202808579828']
HPtSm 16 ['H0.5Sm0.3Pt0.2', '-0.964', '-0.208489248214']
HPtSr 2 ['H0.666667Sr0.222222Pt0.111111', '-0.622', '-0.208910182883']
HPtTb 7 ['H0.625Tb0.25Pt0.125', '-0.709', '-0.205198861288']
HPtTh 29 ['H0.4Pt0.5Th0.1', '-0.445', '-0.2007340785']
HPtY 3 ['H0.5Y0.25Pt0.25', '-0.931', '-0.202617523769']
HPtYb 7 ['H0.666667Yb0.166667Pt0.166667', '-0.632', '-0.204706369219']
HPuRh 8 ['H0.625Rh0.125Pu0.25', '-0.574', '-0.203175587522']

HPuS 20 ['H0.111111S0.444444Pu0.444444', '-1.908', '-0.202420135991']
HPuSb 10 ['H0.375Sb0.375Pu0.25', '-0.565', '-0.209839203556']
HPuSe 1 ['H0.1Se0.4Pu0.5', '-1.584', '-0.296240473782']
HPuSi 2 ['H0.5Si0.166667Pu0.333333', '-0.593', '-0.201201135575']
HPuTa 2 ['H0.7Ta0.1Pu0.2', '-0.538', '-0.222172020805']
HPuTc 1 ['H0.375Tc0.25Pu0.375', '-0.396', '-0.204804801681']
HPuTl 2 ['H0.444444Tl0.222222Pu0.333333', '-0.48', '-0.24649354403']
HPuTm 4 ['H0.666667Tm0.222222Pu0.111111', '-0.78', '-0.205684627433']
HPuZr 11 ['H0.666667Zr0.166667Pu0.166667', '-0.674', '-0.225010726878']
HRbRh 2 ['H0.666667Rb0.111111Rh0.222222', '-0.267', '-0.231732713091']
HRbS 8 ['H0.333333S0.166667Rb0.5', '-0.808', '-0.203253820636']
HRbSb 18 ['H0.428571Rb0.428571Sb0.142857', '-0.419', '-0.204457797769']
HRbSe 6 ['H0.375Se0.25Rb0.375', '-0.852', '-0.217076109999']
HRbSi 20 ['H0.7Si0.1Rb0.2', '-0.264', '-0.200518883563']
HRbSn 6 ['H0.5Rb0.3Sn0.2', '-0.375', '-0.209809503286']
HRbTe 14 ['H0.5Rb0.375Te0.125', '-0.62', '-0.201936244311']
HReS 4 ['H0.625S0.25Re0.125', '-0.497', '-0.216210871653']
HRhSc 12 ['H0.666667Sc0.222222Rh0.111111', '-0.666', '-0.201923279932']
HRhSm 22 ['H0.444444Rh0.333333Sm0.222222', '-0.705', '-0.208162573659']
HRhSr 7 ['H0.555556Sr0.222222Rh0.222222', '-0.563', '-0.202847020141']
HRhTb 23 ['H0.666667Rh0.222222Tb0.111111', '-0.396', '-0.201747087457']
HRhTm 10 ['H0.5Rh0.333333Tm0.166667', '-0.606', '-0.201658139186']
HRhY 46 ['H0.4Y0.2Rh0.4', '-0.675', '-0.203416968904']
HRhYb 21 ['H0.5Rh0.375Yb0.125', '-0.441', '-0.210141995079']
HRuS 7 ['H0.333333S0.333333Ru0.333333', '-0.57', '-0.200855860329']
HRuSm 8 ['H0.714286Ru0.142857Sm0.142857', '-0.532', '-0.205088659183']
HRuTb 3 ['H0.5Ru0.166667Tb0.333333', '-0.553', '-0.201761892741']
HRuTm 3 ['H0.7Ru0.1Tm0.2', '-0.691', '-0.205789767023']
HRuY 20 ['H0.625Y0.125Ru0.25', '-0.503', '-0.20199649939']
HSSc 2 ['H0.2S0.4Sc0.4', '-1.893', '-0.242240255956']
HSSm 29 ['H0.625S0.125Sm0.25', '-1.051', '-0.201875109899']
HSTa 42 ['H0.625S0.25Ta0.125', '-0.673', '-0.207351678996']
HSTc 39 ['H0.25S0.5Tc0.25', '-0.814', '-0.208663187993']
HSTe 11 ['H0.5S0.2Te0.3', '-0.343', '-0.200126290824']
HSTh 12 ['H0.142857S0.428571Th0.428571', '-1.944', '-0.200086525585']
HSTi 4 ['H0.375S0.25Ti0.375', '-1.18', '-0.200697317031']
HSTl 2 ['H0.571429S0.285714Tl0.142857', '-0.456', '-0.207779915278']
HSTm 19 ['H0.4S0.3Tm0.3', '-1.573', '-0.203163769432']
HSU 18 ['H0.285714S0.428571U0.285714', '-1.377', '-0.200154307921']
HSW 14 ['H0.666667S0.166667W0.166667', '-0.458', '-0.20680328433']
HSY 6 ['H0.571429S0.142857Y0.285714', '-1.195', '-0.200293214448']
HSZr 18 ['H0.166667S0.5Zr0.333333', '-1.649', '-0.232424347626']
HSbSr 3 ['H0.5Sr0.3Sb0.2', '-0.743', '-0.20001333595']
HSbU 7 ['H0.444444Sb0.333333U0.222222', '-0.417', '-0.200966251066']

HSbYb 14 ['H0.625Sb0.125Yb0.25', '-0.68', '-0.211839688243']
HScSe 26 ['H0.333333Sc0.166667Se0.5', '-0.737', '-0.205662749167']
HScSi 31 ['H0.3Si0.2Sc0.5', '-0.915', '-0.202622980262']
HScTc 1 ['H0.444444Sc0.444444Tc0.111111', '-0.671', '-0.210741750141']
HScTl 7 ['H0.428571Sc0.428571Tl0.142857', '-0.719', '-0.202181412636']
HSeTc 37 ['H0.5Se0.166667Tc0.333333', '-0.2', '-0.2']
HSeTe 10 ['H0.5Se0.3Te0.2', '-0.203', '-0.203']
HSeTh 2 ['H0.25Se0.375Th0.375', '-1.451', '-0.206091767037']
HSeTm 12 ['H0.2Se0.4Tm0.4', '-1.558', '-0.210369897675']
HSeY 7 ['H0.125Se0.5Y0.375', '-1.704', '-0.279586199104']
HSeZr 6 ['H0.111111Se0.444444Zr0.444444', '-1.274', '-0.202117104665']
HSiSm 5 ['H0.25Si0.375Sm0.375', '-0.856', '-0.200142843898']
HSiSr 6 ['H0.4Si0.3Sr0.3', '-0.65', '-0.215926475627']
HSiTb 21 ['H0.555556Si0.333333Tb0.111111', '-0.403', '-0.208747087457']
HSiTm 20 ['H0.666667Si0.166667Tm0.166667', '-0.605', '-0.200658139186']
HSiU 20 ['H0.444444Si0.222222U0.333333', '-0.492', '-0.203860321598']
HSiY 13 ['H0.625Si0.125Y0.25', '-0.78', '-0.207294273774']
HSiYb 18 ['H0.444444Si0.333333Yb0.222222', '-0.615', '-0.204585769029']
HSnSr 2 ['H0.555556Sr0.333333Sn0.111111', '-0.75', '-0.204502511741']
HSnTm 1 ['H0.625Sn0.125Tm0.25', '-0.851', '-0.27094380362']
HSnYb 10 ['H0.666667Sn0.166667Yb0.166667', '-0.522', '-0.202329792262']
HTbTe 4 ['H0.111111Te0.555556Tb0.333333', '-1.087', '-0.207931825548']
HTeY 1 ['H0.111111Y0.333333Te0.555556', '-1.295', '-0.203078678524']
HTlY 3 ['H0.625Y0.25Tl0.125', '-0.767', '-0.202453979468']
HTlYb 3 ['H0.5Yb0.333333Tl0.166667', '-0.756', '-0.209471909741']
HfHgO 1 ['O0.6Hf0.2Hg0.2', '-2.793', '-0.30774586981']
HfHgS 2 ['S0.375Hf0.5Hg0.125', '-1.385', '-0.237800202872']
HfHoI 4 ['I0.714286Ho0.142857Hf0.142857', '-1.222', '-0.231073151541']
HfHoO 1 ['O0.625Ho0.25Hf0.125', '-4.08', '-0.21898420846']
HfHoS 6 ['S0.5Ho0.166667Hf0.333333', '-1.91', '-0.208164325494']
HfILu 5 ['I0.714286Lu0.142857Hf0.142857', '-1.11', '-0.203167644339']
HfINd 2 ['I0.8Nd0.1Hf0.1', '-1.154', '-0.204220344793']
HfIPa 2 ['I0.777778Hf0.111111Pa0.111111', '-0.952', '-0.339332060597']
HfIPm 4 ['I0.7Pm0.2Hf0.1', '-1.157', '-0.275485198794']
HfIPt 10 ['I0.5Hf0.166667Pt0.333333', '-1.07', '-0.201108986495']
HfIPu 1 ['I0.714286Hf0.142857Pu0.142857', '-1.409', '-0.335807482076']
HfIRh 4 ['Rh0.142857I0.714286Hf0.142857', '-0.963', '-0.214906568257']
HfIS 1 ['S0.4I0.1Hf0.5', '-1.546', '-0.219759407217']
HfISe 2 ['Se0.5I0.125Hf0.375', '-1.442', '-0.20273214165']
HfISm 1 ['I0.777778Sm0.111111Hf0.111111', '-1.225', '-0.21885908478']
HfITb 4 ['I0.7Tb0.2Hf0.1', '-1.036', '-0.342933600361']
HfITm 1 ['I0.777778Tm0.111111Hf0.111111', '-1.274', '-0.274812620326']
HfIYb 1 ['I0.7Yb0.2Hf0.1', '-1.718', '-0.2489027413']
HfInN 3 ['N0.444444In0.111111Hf0.444444', '-1.849', '-0.200485355404']

HfInO 2 ['O0.6In0.2Hf0.2', '-3.151', '-0.232827426053']
HfInS 11 ['S0.375In0.25Hf0.375', '-1.292', '-0.201280107995']
HfIrLi 1 ['Li0.2Hf0.2Ir0.6', '-0.859', '-0.2144144775']
HfIrN 4 ['N0.333333Hf0.555556Ir0.111111', '-1.682', '-0.207595625998']
HfIrS 5 ['S0.444444Hf0.444444Ir0.111111', '-1.659', '-0.223369070438']
HfIrSe 17 ['Se0.375Hf0.375Ir0.25', '-1.338', '-0.200667983438']
HfIrSi 6 ['Si0.428571Hf0.142857Ir0.428571', '-0.943', '-0.204933687679']
HfIrZn 2 ['Zn0.142857Hf0.428571Ir0.428571', '-0.997', '-0.224558796071']
HfKN 4 ['N0.5K0.125Hf0.375', '-1.747', '-0.244411720456']
HfKO 1 ['O0.6K0.2Hf0.2', '-3.365', '-0.48185750614']
HfKS 21 ['S0.75K0.125Hf0.125', '-1.26', '-0.207693582709']
HfKSe 1 ['K0.111111Se0.555556Hf0.333333', '-1.494', '-0.240577805']
HfKrRe 3 ['Kr0.142857Hf0.571429Re0.285714', '-0.378', '-0.203274971429']
HfKrS 2 ['S0.375Kr0.125Hf0.5', '-1.41', '-0.262800202872']
HfLaN 1 ['N0.5La0.25Hf0.25', '-1.986', '-0.34548828858']
HfLaO 4 ['O0.666667La0.166667Hf0.166667', '-3.756', '-0.209233011574']
HfLaS 7 ['S0.5La0.166667Hf0.333333', '-1.967', '-0.214346398411']
HfLaSe 1 ['Se0.571429La0.142857Hf0.285714', '-1.723', '-0.212640459524']
HfLiN 8 ['Li0.1N0.5Hf0.4', '-1.82', '-0.220028126997']
HfLiPt 2 ['Li0.2Hf0.1Pt0.7', '-0.933', '-0.218406500833']
HfLiRh 7 ['Li0.1Rh0.7Hf0.2', '-0.853', '-0.207592907']
HfLiS 16 ['Li0.111111S0.555556Hf0.333333', '-1.839', '-0.230858636382']
HfLiSe 2 ['Li0.25Se0.5Hf0.25', '-1.497', '-0.218734553751']
HfLuS 4 ['S0.5Lu0.1Hf0.4', '-1.819', '-0.219297338869']
HfLuSe 2 ['Se0.555556Lu0.222222Hf0.222222', '-1.777', '-0.218093998889']
HfLuTe 1 ['Te0.6Lu0.3Hf0.1', '-1.204', '-0.215323971499']
HfMgN 3 ['N0.375Mg0.125Hf0.5', '-1.591', '-0.200065768623']
HfMgO 1 ['O0.625Mg0.25Hf0.125', '-3.131', '-0.207387332785']
HfMgS 23 ['Mg0.166667S0.333333Hf0.5', '-1.317', '-0.207910645818']
HfMgSe 1 ['Mg0.222222Se0.555556Hf0.222222', '-1.56', '-0.284331024631']
HfMnN 11 ['N0.375Mn0.375Hf0.25', '-1.294', '-0.233014411111']
HfMnO 1 ['O0.6Mn0.2Hf0.2', '-3.402', '-0.283849253026']
HfMnS 8 ['S0.666667Mn0.166667Hf0.166667', '-1.41', '-0.202770267525']
HfMoN 7 ['N0.333333Mo0.111111Hf0.555556', '-1.466', '-0.20126955072']
HfMoS 18 ['S0.4Mo0.3Hf0.3', '-1.324', '-0.211331842894']
HfNNa 11 ['N0.333333Na0.111111Hf0.555556', '-1.437', '-0.200614016553']
HfNNb 3 ['N0.5Nb0.166667Hf0.333333', '-1.835', '-0.206067983997']
HfNNp 5 ['N0.555556Hf0.111111Np0.333333', '-1.466', '-0.21248384988']
HfNO 2 ['N0.4O0.2Hf0.4', '-2.634', '-0.280184683688']
HfNP 11 ['N0.5P0.2Hf0.3', '-1.493', '-0.207180458993']
HfNPa 14 ['N0.555556Hf0.333333Pa0.111111', '-1.769', '-0.213822820194']
HfNPt 7 ['N0.4Hf0.3Pt0.3', '-1.453', '-0.212382754743']
HfNPu 11 ['N0.571429Hf0.285714Pu0.142857', '-1.776', '-0.239993115299']
HfNRe 7 ['N0.555556Hf0.111111Re0.333333', '-0.687', '-0.203047361208']

HfNRu 1 ['N0.375Ru0.125Hf0.5', '-1.809', '-0.214815101747']
HfNS 1 ['N0.1S0.4Hf0.5', '-1.736', '-0.206217087362']
HfNTb 1 ['N0.444444Tb0.111111Hf0.444444', '-1.857', '-0.208485355404']
HfNTc 9 ['N0.5Tc0.25Hf0.25', '-1.278', '-0.210856904636']
HfNTi 9 ['N0.5Ti0.3Hf0.2', '-1.781', '-0.201397491038']
HfNTl 1 ['N0.4Hf0.5Tl0.1', '-1.769', '-0.285336819864']
HfNU 4 ['N0.5Hf0.25U0.25', '-1.866', '-0.23079143733']
HfNW 3 ['N0.6Hf0.1W0.3', '-0.73', '-0.219590268984']
HfNYb 5 ['N0.444444Yb0.333333Hf0.222222', '-1.617', '-0.21086114318']
HfNZn 19 ['N0.555556Zn0.111111Hf0.333333', '-1.536', '-0.200365973739']
HfNaO 1 ['O0.6Na0.2Hf0.2', '-3.091', '-0.254341115298']
HfNaS 23 ['Na0.125S0.625Hf0.25', '-1.798', '-0.212225143635']
HfNaSe 3 ['Na0.1Se0.6Hf0.3', '-1.522', '-0.221645265915']
HfNbS 18 ['S0.4Nb0.3Hf0.3', '-1.326', '-0.215742381895']
HfNbSe 1 ['Se0.555556Nb0.222222Hf0.222222', '-1.274', '-0.2023090125']
HfNdO 2 ['O0.625Nd0.125Hf0.25', '-3.886', '-0.23517482601']
HfNdS 10 ['S0.428571Nd0.142857Hf0.428571', '-1.725', '-0.200154792389']
HfNdSe 1 ['Se0.6Nd0.2Hf0.2', '-1.782', '-0.217345494666']
HfNiS 3 ['S0.375Ni0.125Hf0.5', '-1.447', '-0.229645328567']
HfNiSe 1 ['Ni0.4Se0.4Hf0.2', '-1.022', '-0.214187189']
HfNiTc 1 ['Ni0.111111Tc0.333333Hf0.555556', '-0.638', '-0.200865665556']
HfOPa 3 ['O0.7Hf0.2Pa0.1', '-3.436', '-0.20287056097']
HfOPu 2 ['O0.714286Hf0.142857Pu0.142857', '-3.271', '-0.228148958076']
HfOS 19 ['O0.1S0.5Hf0.4', '-2.164', '-0.212458005905']
HfOSe 11 ['O0.285714Se0.142857Hf0.571429', '-2.186', '-0.201969554033']
HfOSi 1 ['O0.666667Si0.166667Hf0.166667', '-3.685', '-0.21013436525']
HfOTe 1 ['O0.7Te0.2Hf0.1', '-2.235', '-0.237860732214']
HfOU 1 ['O0.714286Hf0.142857U0.142857', '-3.785', '-0.350477803473']
HfOsRe 5 ['Hf0.6Re0.1Os0.3', '-0.708', '-0.2181285225']
HfOsS 3 ['S0.444444Hf0.444444Os0.111111', '-1.604', '-0.212871848217']
HfOsSe 3 ['Se0.25Hf0.375Os0.375', '-1.061', '-0.201722696875']
HfOsSi 2 ['Si0.428571Hf0.142857Os0.428571', '-0.795', '-0.217474444643']
HfOsTc 3 ['Tc0.111111Hf0.555556Os0.333333', '-0.8', '-0.205915997222']
HfPPu 3 ['P0.375Hf0.25Pu0.375', '-1.279', '-0.21803606185']
HfPRe 5 ['P0.2Hf0.6Re0.2', '-0.995', '-0.20555474854']
HfPRh 1 ['P0.444444Rh0.222222Hf0.333333', '-1.441', '-0.208026059351']
HfPS 2 ['P0.125S0.375Hf0.5', '-1.654', '-0.205374701283']
HfPSi 1 ['Si0.333333P0.222222Hf0.444444', '-1.212', '-0.20669423412']
HfPTa 1 ['P0.444444Hf0.111111Ta0.444444', '-1.14', '-0.252014286295']
HfPaS 16 ['S0.7Hf0.2Pa0.1', '-1.723', '-0.20150437244']
HfPaSe 13 ['Se0.5Hf0.333333Pa0.166667', '-1.422', '-0.230402816667']
HfPbS 4 ['S0.5Hf0.125Pb0.375', '-1.206', '-0.200072854868']
HfPdPm 1 ['Pd0.8Pm0.1Hf0.1', '-0.717', '-0.20422378125']
HfPdS 3 ['S0.4Pd0.1Hf0.5', '-1.531', '-0.22208813448']

HfPdSe 1 ['Se0.5Pd0.125Hf0.375', '-1.42', '-0.204646939687']
HfPmS 22 ['S0.6Pm0.1Hf0.3', '-1.982', '-0.200983130592']
HfPmSe 14 ['Se0.571429Pm0.142857Hf0.285714', '-1.589', '-0.210172763929']
HfPrS 10 ['S0.444444Pr0.222222Hf0.333333', '-1.869', '-0.202600410439']
HfPrSe 3 ['Se0.555556Pr0.111111Hf0.333333', '-1.603', '-0.204734569721']
HfPtS 8 ['S0.4Hf0.3Pt0.3', '-1.705', '-0.204115051894']
HfPtSc 1 ['Sc0.1Hf0.1Pt0.8', '-1.106', '-0.248081377']
HfPtTh 2 ['Hf0.1Pt0.8Th0.1', '-0.959', '-0.275497249']
HfPtTm 1 ['Tm0.1Hf0.1Pt0.8', '-1.074', '-0.2057815475']
HfPuS 7 ['S0.5Hf0.2Pu0.3', '-1.937', '-0.206300032992']
HfPuSe 5 ['Se0.6Hf0.3Pu0.1', '-1.557', '-0.2029170345']
HfRbS 18 ['S0.555556Rb0.111111Hf0.333333', '-1.763', '-0.21148351916']
HfRbSe 3 ['Se0.571429Rb0.142857Hf0.285714', '-1.481', '-0.217761362856']
HfReS 10 ['S0.7Hf0.2Re0.1', '-1.521', '-0.200667678315']
HfReSc 1 ['Sc0.111111Hf0.555556Re0.333333', '-0.407', '-0.200042536889']
HfReTc 1 ['Tc0.1Hf0.6Re0.3', '-0.497', '-0.217000469']
HfRhS 30 ['S0.555556Rh0.333333Hf0.111111', '-1.169', '-0.201127169159']
HfRhSe 21 ['Se0.5Rh0.333333Hf0.166667', '-0.99', '-0.201066114531']
HfRhTc 2 ['Tc0.3Rh0.1Hf0.6', '-0.714', '-0.2002199575']
HfRuS 26 ['S0.333333Ru0.166667Hf0.5', '-1.449', '-0.21227651283']
HfRuSe 2 ['Se0.25Ru0.375Hf0.375', '-1.157', '-0.245615659375']
HfRuSi 3 ['Si0.333333Ru0.5Hf0.166667', '-0.949', '-0.206433759167']
HfSSb 2 ['S0.333333Sb0.111111Hf0.555556', '-1.439', '-0.297817672553']
HfSSc 13 ['S0.555556Sc0.111111Hf0.333333', '-1.89', '-0.208020621103']
HfSSe 16 ['S0.1Se0.5Hf0.4', '-1.547', '-0.201575123098']
HfSSi 4 ['Si0.142857S0.428571Hf0.428571', '-1.626', '-0.202385738994']
HfSSm 9 ['S0.444444Sm0.111111Hf0.444444', '-1.734', '-0.224577568079']
HfSSn 6 ['S0.333333Sn0.166667Hf0.5', '-1.321', '-0.211977386688']
HfSSr 5 ['S0.444444Sr0.111111Hf0.444444', '-1.752', '-0.222249806551']
HfSTa 16 ['S0.5Hf0.25Ta0.25', '-1.534', '-0.206939851743']
HfSTb 8 ['S0.571429Tb0.142857Hf0.285714', '-1.916', '-0.204986810078']
HfSTc 28 ['S0.625Tc0.125Hf0.25', '-1.682', '-0.203605648741']
HfSTe 6 ['S0.4Te0.2Hf0.4', '-1.565', '-0.206674578728']
HfSTh 20 ['S0.6Hf0.1Th0.3', '-2.223', '-0.211257353839']
HfSTi 17 ['S0.571429Ti0.142857Hf0.285714', '-1.834', '-0.207900116451']
HfSTl 14 ['S0.3Hf0.5Tl0.2', '-1.173', '-0.206630662799']
HfSTm 7 ['S0.444444Tm0.111111Hf0.444444', '-1.743', '-0.23097582801']
HfSU 3 ['S0.5Hf0.375U0.125', '-1.674', '-0.207419047369']
HfSV 11 ['S0.555556V0.111111Hf0.333333', '-1.709', '-0.205813264807']
HfSW 6 ['S0.5Hf0.375W0.125', '-1.611', '-0.221386883618']
HfSXe 4 ['S0.428571Xe0.142857Hf0.428571', '-1.457', '-0.21535665971']
HfSY 3 ['S0.444444Y0.111111Hf0.444444', '-1.72', '-0.20852423822']
HfSYb 6 ['S0.555556Yb0.111111Hf0.333333', '-1.992', '-0.223089907214']
HfSZn 7 ['S0.333333Zn0.333333Hf0.333333', '-1.205', '-0.204163334773']

HfSZr 22 ['S0.4Zr0.4Hf0.2', '-1.487', '-0.214054134393']
HfScSe 3 ['Sc0.25Se0.5Hf0.25', '-1.605', '-0.215302575']
HfSeSi 6 ['Si0.166667Se0.5Hf0.333333', '-1.355', '-0.20343083']
HfSeSm 1 ['Se0.555556Sm0.111111Hf0.333333', '-1.631', '-0.220837372778']
HfSeSr 3 ['Se0.555556Sr0.111111Hf0.333333', '-1.683', '-0.246803707222']
HfSeTb 1 ['Se0.571429Tb0.142857Hf0.285714', '-1.592', '-0.217512199689']
HfSeTc 3 ['Se0.625Tc0.25Hf0.125', '-0.708', '-0.2022370075']
HfSeTi 1 ['Ti0.111111Se0.555556Hf0.333333', '-1.442', '-0.207370842358']
HfSeTl 6 ['Se0.444444Hf0.444444Tl0.111111', '-1.268', '-0.214326135556']
HfSeTm 8 ['Se0.625Tm0.125Hf0.25', '-1.632', '-0.208140545']
HfSeY 8 ['Se0.666667Y0.111111Hf0.222222', '-1.524', '-0.233225505556']
HfSeZn 2 ['Zn0.166667Se0.5Hf0.333333', '-1.274', '-0.200158665833']
HfSeZr 2 ['Se0.555556Zr0.111111Hf0.333333', '-1.437', '-0.201919361667']
HfSiTc 7 ['Si0.4Tc0.5Hf0.1', '-0.652', '-0.209359629']
HgHoI 24 ['I0.5Ho0.333333Hg0.166667', '-0.971', '-0.201766713508']
HgHoO 1 ['O0.6Ho0.3Hg0.1', '-3.339', '-0.342328044206']
HgILu 31 ['I0.5Lu0.125Hg0.375', '-0.647', '-0.238067929736']
HgINb 10 ['Nb0.111111I0.666667Hg0.222222', '-0.671', '-0.202890597277']
HgINd 11 ['I0.6Nd0.1Hg0.3', '-0.884', '-0.217249864927']
HgINp 12 ['I0.777778Hg0.111111Np0.111111', '-0.793', '-0.211869925321']
HgIPa 27 ['I0.5Hg0.2Pa0.3', '-0.47', '-0.202516333543']
HgIPm 38 ['I0.444444Pm0.111111Hg0.444444', '-0.601', '-0.200247493519']
HgIPr 3 ['I0.666667Pr0.111111Hg0.222222', '-1.033', '-0.20335455207']
HgIPu 8 ['I0.714286Hg0.142857Pu0.142857', '-1.065', '-0.20350393095']
HgIRh 8 ['Rh0.166667I0.666667Hg0.166667', '-0.402', '-0.202210063988']
HgISi 2 ['Si0.111111I0.666667Hg0.222222', '-0.633', '-0.211657452139']
HgISm 18 ['I0.5Sm0.333333Hg0.166667', '-1.214', '-0.202033279413']
HgITa 5 ['I0.571429Ta0.142857Hg0.285714', '-0.63', '-0.208932949684']
HgITb 37 ['I0.428571Tb0.428571Hg0.142857', '-0.583', '-0.211957925102']
HgITh 5 ['I0.666667Hg0.222222Th0.111111', '-1.099', '-0.201921866446']
HgITm 8 ['I0.6Tm0.2Hg0.2', '-1.084', '-0.212031600547']
HgIU 4 ['I0.666667Hg0.166667U0.166667', '-1.028', '-0.220446905881']
HgIXe 5 ['I0.5Xe0.4Hg0.1', '-0.322', '-0.202126038393']
HgInPr 1 ['In0.142857Pr0.285714Hg0.571429', '-0.514', '-0.209205827151']
HgIrO 3 ['O0.666667Ir0.222222Hg0.111111', '-1.028', '-0.217472075809']
HgKO 8 ['O0.428571K0.142857Hg0.428571', '-0.918', '-0.220200283328']
HgKSe 3 ['K0.333333Se0.333333Hg0.333333', '-0.857', '-0.221097987778']
HgKTe 4 ['K0.333333Te0.333333Hg0.333333', '-0.789', '-0.225608273333']
HgKrP 21 ['P0.333333Kr0.444444Hg0.222222', '-0.201', '-0.201']
HgLaO 2 ['O0.625La0.25Hg0.125', '-2.646', '-0.212060698053']
HgMnO 4 ['O0.6Mn0.1Hg0.3', '-0.999', '-0.201100390849']
HgMoO 4 ['O0.666667Mo0.166667Hg0.166667', '-1.922', '-0.27836406025']
HgNNp 9 ['N0.428571Hg0.285714Np0.285714', '-0.9', '-0.2071673074']
HgNPa 19 ['N0.4Hg0.4Pa0.2', '-0.605', '-0.20982232362']

HgNPu 5 ['N0.5Hg0.166667Pu0.333333', '-1.155', '-0.24225198822']
HgNTi 1 ['N0.444444Ti0.444444Hg0.111111', '-1.383', '-0.230078380437']
HgNYb 1 ['N0.333333Yb0.555556Hg0.111111', '-1.154', '-0.203416527109']
HgNaO 16 ['O0.428571Na0.285714Hg0.285714', '-1.105', '-0.213497326049']
HgNbO 4 ['O0.571429Nb0.142857Hg0.285714', '-1.835', '-0.218837898198']
HgNdO 1 ['O0.625Nd0.25Hg0.125', '-2.708', '-0.25251688779']
HgNpO 2 ['O0.625Hg0.25Np0.125', '-1.841', '-0.222390821455']
HgNpP 19 ['P0.5Hg0.166667Np0.333333', '-0.871', '-0.201691895937']
HgNpPt 1 ['Pt0.714286Hg0.142857Np0.142857', '-0.35', '-0.212220542326']
HgNpS 1 ['S0.428571Hg0.142857Np0.428571', '-1.555', '-0.261996197389']
HgNpSe 10 ['Se0.571429Hg0.142857Np0.285714', '-1.044', '-0.206969512982']
HgOPa 9 ['O0.571429Hg0.285714Pa0.142857', '-1.912', '-0.229128599032']
HgOPb 1 ['O0.6Hg0.2Pb0.2', '-1.124', '-0.273496709814']
HgOPd 2 ['O0.6Pd0.2Hg0.2', '-0.792', '-0.266617088479']
HgOPt 2 ['O0.625Pt0.125Hg0.25', '-0.718', '-0.212968636236']
HgOPu 1 ['O0.625Hg0.125Pu0.25', '-2.787', '-0.210433722594']
HgORb 3 ['O0.428571Rb0.142857Hg0.428571', '-0.922', '-0.239693061899']
HgORe 1 ['O0.6Re0.2Hg0.2', '-1.792', '-0.271384289472']
HgORh 2 ['O0.625Rh0.125Hg0.25', '-0.82', '-0.203618679987']
HgORu 1 ['O0.6Ru0.2Hg0.2', '-1.151', '-0.209414197812']
HgOSb 2 ['O0.625Sb0.25Hg0.125', '-1.658', '-0.24362754716']
HgOSc 2 ['O0.6Sc0.2Hg0.2', '-2.356', '-0.270540393899']
HgOSi 4 ['O0.5Si0.1Hg0.4', '-1.441', '-0.239612301409']
HgOSn 1 ['O0.6Sn0.2Hg0.2', '-1.693', '-0.33914417144']
HgOSr 2 ['O0.4Sr0.2Hg0.4', '-1.712', '-0.201724472656']
HgOTa 3 ['O0.6Ta0.1Hg0.3', '-1.635', '-0.266770797026']
HgOTc 3 ['O0.666667Tc0.222222Hg0.111111', '-1.62', '-0.208847903671']
HgOTe 2 ['O0.666667Te0.222222Hg0.111111', '-1.292', '-0.222109636315']
HgOTi 1 ['O0.6Ti0.2Hg0.2', '-2.418', '-0.290650720054']
HgOU 7 ['O0.7Hg0.1U0.2', '-2.819', '-0.223275937798']
HgOV 1 ['O0.6V0.2Hg0.2', '-2.136', '-0.40991551342']
HgOW 2 ['O0.6W0.1Hg0.3', '-1.527', '-0.272568080818']
HgOXe 2 ['O0.6Xe0.2Hg0.2', '-0.412', '-0.230235255164']
HgOY 1 ['O0.625Y0.125Hg0.25', '-1.625', '-0.20773333129']
HgOZn 1 ['O0.6Zn0.2Hg0.2', '-1.05', '-0.208673539988']
HgOZr 3 ['O0.625Zr0.25Hg0.125', '-3.053', '-0.214660211535']
HgOsSc 2 ['Sc0.625Os0.25Hg0.125', '-0.504', '-0.244376142504']
HgPPa 1 ['P0.8Hg0.1Pa0.1', '-0.586', '-0.223987509709']
HgPPm 7 ['P0.428571Pm0.428571Hg0.142857', '-1.238', '-0.216484352231']
HgPPu 20 ['P0.285714Hg0.285714Pu0.428571', '-0.972', '-0.204703288878']
HgPRh 2 ['P0.777778Rh0.111111Hg0.111111', '-0.551', '-0.208209080624']
HgPS 12 ['P0.285714S0.285714Hg0.428571', '-0.458', '-0.214876110342']
HgPSn 1 ['P0.8Sn0.1Hg0.1', '-0.383', '-0.295185287127']
HgPTa 17 ['P0.375Ta0.25Hg0.375', '-0.543', '-0.20914645776']

HgPTe 9 ['P0.5Te0.333333Hg0.166667', '-0.248', '-0.202235115283']
HgPU 1 ['P0.8Hg0.1U0.1', '-0.52', '-0.229273879458']
HgPXe 12 ['P0.375Xe0.5Hg0.125', '-0.207', '-0.207']
HgPaS 9 ['S0.571429Hg0.285714Pa0.142857', '-0.927', '-0.200923499788']
HgPaSe 13 ['Se0.571429Hg0.285714Pa0.142857', '-0.655', '-0.206430331438']
HgPdPm 11 ['Pd0.555556Pm0.333333Hg0.111111', '-0.756', '-0.204863208615']
HgPmS 7 ['S0.333333Pm0.444444Hg0.222222', '-1.625', '-0.202701306446']
HgPmSe 26 ['Se0.375Pm0.25Hg0.375', '-1.082', '-0.225763838754']
HgPrSe 1 ['Se0.555556Pr0.333333Hg0.111111', '-1.791', '-0.22846162384']
HgPtTh 15 ['Pt0.571429Hg0.142857Th0.285714', '-0.91', '-0.204215045719']
HgPuS 4 ['S0.5Hg0.1Pu0.4', '-1.889', '-0.204226852491']
HgPuSe 3 ['Se0.5Hg0.166667Pu0.333333', '-1.374', '-0.213225313332']
HgPuTe 1 ['Te0.5Hg0.1Pu0.4', '-1.144', '-0.203974736331']
HgRbSe 3 ['Se0.333333Rb0.333333Hg0.333333', '-0.846', '-0.218663813333']
HgSTc 4 ['S0.5Tc0.4Hg0.1', '-0.816', '-0.210663187993']
HgSXe 1 ['S0.5Xe0.4Hg0.1', '-0.27', '-0.220257304102']
HgSZr 5 ['S0.4Zr0.4Hg0.2', '-1.397', '-0.205475278392']
HgScSe 10 ['Sc0.222222Se0.444444Hg0.333333', '-1.004', '-0.21422400223']
HgSeTc 4 ['Se0.625Tc0.25Hg0.125', '-0.251', '-0.205253939379']
HgSeTm 2 ['Se0.5Tm0.333333Hg0.166667', '-1.514', '-0.205640370002']
HgSeY 18 ['Se0.428571Y0.142857Hg0.428571', '-0.814', '-0.204894367867']
HgSeZr 2 ['Se0.5Zr0.375Hg0.125', '-1.278', '-0.21022839493']
HoIIn 25 ['In0.25I0.375Ho0.375', '-0.937', '-0.20135335409']
HoIIr 30 ['I0.5Ho0.375Ir0.125', '-1.079', '-0.203521801206']
HoIK 13 ['K0.2I0.6Ho0.2', '-1.302', '-0.206934088621']
HoIKr 18 ['Kr0.333333I0.555556Ho0.111111', '-0.458', '-0.201588904503']
HoILa 5 ['I0.714286La0.142857Ho0.142857', '-1.539', '-0.253862227606']
HoILi 15 ['Li0.333333I0.555556Ho0.111111', '-1.387', '-0.249297462029']
HoILu 14 ['I0.571429Ho0.142857Lu0.285714', '-0.848', '-0.200850743667']
HoIMg 9 ['Mg0.222222I0.666667Ho0.111111', '-1.28', '-0.240600305829']
HoIMn 13 ['Mn0.25I0.625Ho0.125', '-0.861', '-0.202396643173']
HoIMo 6 ['Mo0.111111I0.666667Ho0.222222', '-0.914', '-0.234465658373']
HoIN 29 ['N0.222222I0.333333Ho0.444444', '-1.622', '-0.287638371521']
HoINa 15 ['Na0.333333I0.555556Ho0.111111', '-1.421', '-0.220511933409']
HoINb 5 ['Nb0.142857I0.714286Ho0.142857', '-0.914', '-0.202910187432']
HoINd 8 ['I0.666667Nd0.166667Ho0.166667', '-1.35', '-0.255192935109']
HoINi 32 ['Ni0.111111I0.555556Ho0.333333', '-1.001', '-0.210358811767']
HoINp 5 ['I0.714286Ho0.142857Np0.142857', '-1.182', '-0.276409869212']
HoIO 29 ['O0.222222I0.333333Ho0.444444', '-2.196', '-0.248312667854']
HoIOs 16 ['I0.625Ho0.125Os0.25', '-0.491', '-0.202537517566']
HoIP 34 ['P0.4I0.2Ho0.4', '-1.611', '-0.21848631508']
HoIPa 11 ['I0.6Ho0.1Pa0.3', '-0.564', '-0.250022784324']
HoIPb 24 ['I0.375Ho0.375Pb0.25', '-0.944', '-0.222243984495']
HoIPd 34 ['Pd0.333333I0.333333Ho0.333333', '-1.129', '-0.214295216413']

HoIPm 12 ['I0.6Pm0.3Ho0.1', '-0.996', '-0.228455915574']
HoIPr 5 ['I0.7Pr0.1Ho0.2', '-1.327', '-0.328780547498']
HoIPt 49 ['I0.333333Ho0.166667Pt0.5', '-0.945', '-0.208229037446']
HoIPu 8 ['I0.666667Ho0.166667Pu0.166667', '-1.263', '-0.201300093289']
HoIRb 14 ['Rb0.285714I0.571429Ho0.142857', '-1.5', '-0.238665128674']
HoIRe 9 ['I0.625Ho0.25Re0.125', '-0.865', '-0.225235657163']
HoIRh 47 ['Rh0.333333I0.222222Ho0.444444', '-1.097', '-0.201533992053']
HoIRu 20 ['Ru0.25I0.5Ho0.25', '-0.803', '-0.226075035131']
HoIS 33 ['S0.25I0.25Ho0.5', '-1.648', '-0.204009382419']
HoISb 43 ['Sb0.3I0.1Ho0.6', '-1.083', '-0.201594181535']
HoISc 8 ['Sc0.166667I0.666667Ho0.166667', '-1.238', '-0.237780860109']
HoISe 43 ['Se0.2I0.7Ho0.1', '-0.675', '-0.202588378918']
HoISi 15 ['Si0.222222I0.555556Ho0.222222', '-0.897', '-0.202381292787']
HoISm 8 ['I0.666667Sm0.166667Ho0.166667', '-1.259', '-0.2476692272']
HoISn 20 ['Sn0.166667I0.5Ho0.333333', '-1.042', '-0.210449381609']
HoISr 8 ['Sr0.166667I0.666667Ho0.166667', '-1.57', '-0.272406507194']
HoITa 5 ['I0.714286Ho0.142857Ta0.142857', '-0.94', '-0.241894922477']
HoITb 14 ['I0.571429Tb0.285714Ho0.142857', '-0.774', '-0.20232716662']
HoITc 12 ['Tc0.2I0.6Ho0.2', '-0.679', '-0.217460028105']
HoITe 54 ['Te0.571429I0.142857Ho0.285714', '-1.196', '-0.208128374011']
HoITh 3 ['I0.75Ho0.125Th0.125', '-1.417', '-0.270277017916']
HoITi 7 ['Ti0.166667I0.666667Ho0.166667', '-1.136', '-0.237426564244']
HoITl 30 ['I0.444444Ho0.222222Tl0.333333', '-0.939', '-0.200710523958']
HoITm 7 ['I0.666667Ho0.222222Tm0.111111', '-1.245', '-0.314840876242']
HoIU 3 ['I0.75Ho0.125U0.125', '-1.202', '-0.307872696976']
HoIV 8 ['V0.166667I0.666667Ho0.166667', '-0.933', '-0.264350522611']
HoIW 7 ['I0.666667Ho0.166667W0.166667', '-0.603', '-0.218383356754']
HoIXe 30 ['I0.333333Xe0.555556Ho0.111111', '-0.479', '-0.222588904503']
HoIY 4 ['Y0.1I0.7Ho0.2', '-1.289', '-0.201991492377']
HoIYb 6 ['I0.8Ho0.1Yb0.1', '-0.98', '-0.215727491567']
HoIZn 23 ['Zn0.2I0.5Ho0.3', '-1.021', '-0.26144836711']
HoIZr 5 ['Zr0.142857I0.714286Ho0.142857', '-1.21', '-0.257983950818']
HoInN 17 ['N0.5In0.166667Ho0.333333', '-1.444', '-0.205516402883']
HoInP 1 ['P0.375In0.125Ho0.5', '-1.658', '-0.237382776221']
HoInS 10 ['S0.444444In0.222222Ho0.333333', '-1.938', '-0.212215532906']
HoIrN 5 ['N0.4Ho0.5Ir0.1', '-1.852', '-0.202503825195']
HoIrP 5 ['P0.428571Ho0.428571Ir0.142857', '-1.711', '-0.201056213443']
HoIrS 1 ['S0.444444Ho0.444444Ir0.111111', '-2.142', '-0.203052161829']
HoIrSe 11 ['Se0.5Ho0.3Ir0.2', '-1.646', '-0.203339004753']
HoIrSi 6 ['Si0.4Ho0.1Ir0.5', '-1.032', '-0.20875813529']
HoKO 2 ['O0.625K0.125Ho0.25', '-3.046', '-0.21901535096']
HoKTe 2 ['K0.1Te0.6Ho0.3', '-1.367', '-0.241754462998']
HoKrOs 3 ['Kr0.142857Ho0.571429Os0.285714', '-0.238', '-0.202737887619']
HoLaO 5 ['O0.666667La0.166667Ho0.166667', '-3.377', '-0.215885825254']

HoLaOs 2 ['La0.111111Ho0.555556Os0.333333', '-0.289', '-0.211255847778']
HoLiN 3 ['Li0.111111N0.444444Ho0.444444', '-1.867', '-0.223921125031']
HoLiO 1 ['Li0.1O0.6Ho0.3', '-3.555', '-0.30849825066']
HoLiOs 6 ['Li0.2Ho0.5Os0.3', '-0.246', '-0.208974782']
HoLiP 1 ['Li0.142857P0.428571Ho0.428571', '-1.74', '-0.248021051871']
HoLiS 7 ['Li0.1S0.4Ho0.5', '-1.907', '-0.20736046673']
HoLiSe 11 ['Li0.125Se0.5Ho0.375', '-1.935', '-0.2221021025']
HoLuOs 1 ['Ho0.5Lu0.125Os0.375', '-0.373', '-0.209669953333']
HoLuTe 1 ['Te0.6Ho0.1Lu0.3', '-1.321', '-0.242070168665']
HoMgN 4 ['N0.428571Mg0.285714Ho0.285714', '-1.571', '-0.206800042917']
HoMgO 1 ['O0.625Mg0.125Ho0.25', '-3.407', '-0.238219123568']
HoMgOs 3 ['Mg0.142857Ho0.571429Os0.285714', '-0.291', '-0.230525789643']
HoMgS 22 ['Mg0.375S0.5Ho0.125', '-1.918', '-0.20358155664']
HoMgSe 1 ['Mg0.125Se0.5Ho0.375', '-1.829', '-0.217494174376']
HoMnN 19 ['N0.444444Mn0.333333Ho0.222222', '-1.21', '-0.202233801453']
HoMnO 2 ['O0.6Mn0.2Ho0.2', '-3.202', '-0.2059170497']
HoMnSi 3 ['Si0.5Mn0.375Ho0.125', '-0.774', '-0.207773744411']
HoMoO 2 ['O0.666667Mo0.166667Ho0.166667', '-3.188', '-0.23407811136']
HoMoS 1 ['S0.5Mo0.3Ho0.2', '-1.667', '-0.226011582993']
HoMoSe 1 ['Se0.555556Mo0.222222Ho0.222222', '-1.478', '-0.204342907594']
HoNNa 2 ['N0.375Na0.125Ho0.5', '-1.597', '-0.210652199245']
HoNNi 3 ['N0.3Ni0.2Ho0.5', '-1.501', '-0.201605822729']
HoNNp 20 ['N0.5Ho0.375Np0.125', '-1.894', '-0.204537896232']
HoNO 7 ['N0.3O0.2Ho0.5', '-2.606', '-0.205543132359']
HoNOs 7 ['N0.3Ho0.5Os0.2', '-1.356', '-0.222238280729']
HoNP 45 ['N0.333333P0.166667Ho0.5', '-2.018', '-0.20547680839']
HoNPa 15 ['N0.5Ho0.375Pa0.125', '-1.91', '-0.276666151507']
HoNPd 9 ['N0.375Pd0.25Ho0.375', '-1.595', '-0.208652199245']
HoNPt 22 ['N0.142857Ho0.571429Pt0.285714', '-1.53', '-0.230666236619']
HoNPu 12 ['N0.428571Ho0.285714Pu0.285714', '-1.654', '-0.206557289614']
HoNRe 4 ['N0.5Ho0.3Re0.2', '-1.341', '-0.208676981373']
HoNRh 4 ['N0.4Rh0.1Ho0.5', '-1.883', '-0.223223015195']
HoNRu 2 ['N0.333333Ru0.111111Ho0.555556', '-1.522', '-0.213029401181']
HoNSb 5 ['N0.428571Sb0.142857Ho0.428571', '-1.791', '-0.206602513423']
HoNSe 4 ['N0.4Se0.2Ho0.4', '-1.825', '-0.202470929741']
HoNSi 6 ['N0.4Si0.1Ho0.5', '-1.854', '-0.209081767945']
HoNTc 5 ['N0.444444Tc0.222222Ho0.333333', '-1.494', '-0.203541887624']
HoNTe 5 ['N0.222222Te0.444444Ho0.333333', '-1.405', '-0.207547666959']
HoNTi 13 ['N0.571429Ti0.285714Ho0.142857', '-1.48', '-0.210703606184']
HoNW 4 ['N0.555556Ho0.111111W0.333333', '-0.745', '-0.212319699994']
HoNZn 9 ['N0.375Zn0.125Ho0.5', '-1.682', '-0.208651102578']
HoNZr 1 ['N0.444444Zr0.111111Ho0.444444', '-1.846', '-0.202921125031']
HoNaO 3 ['O0.6Na0.2Ho0.2', '-2.772', '-0.255070649511']
HoNaOs 2 ['Na0.1Ho0.6Os0.3', '-0.29', '-0.252974782']

HoNaS 5 ['Na0.166667S0.5Ho0.333333', '-2.274', '-0.216036175077']
HoNaSe 5 ['Na0.166667Se0.5Ho0.333333', '-1.859', '-0.210927583332']
HoNaTe 1 ['Na0.111111Te0.555556Ho0.333333', '-1.41', '-0.26297767972']
HoNbO 2 ['O0.666667Nb0.222222Ho0.111111', '-3.4', '-0.230816329187']
HoNbS 2 ['S0.666667Nb0.166667Ho0.166667', '-1.778', '-0.214709630448']
HoNbSe 1 ['Se0.555556Nb0.111111Ho0.333333', '-1.878', '-0.216233997642']
HoNdO 2 ['O0.625Nd0.25Ho0.125', '-3.803', '-0.29000880505']
HoNdOs 3 ['Nd0.111111Ho0.555556Os0.333333', '-0.271', '-0.201384630556']
HoNiO 1 ['O0.625Ni0.125Ho0.25', '-2.968', '-0.236286812766']
HoNiOs 2 ['Ni0.142857Ho0.571429Os0.285714', '-0.403', '-0.225196217024']
HoNiP 2 ['P0.375Ni0.25Ho0.375', '-1.586', '-0.239636223781']
HoNiS 1 ['S0.375Ni0.125Ho0.5', '-1.932', '-0.223991578081']
HoNiSe 2 ['Ni0.111111Se0.444444Ho0.444444', '-1.8', '-0.238521591665']
HoNiSi 3 ['Si0.333333Ni0.333333Ho0.333333', '-0.974', '-0.206194738611']
HoNpOs 7 ['Ho0.125Os0.625Np0.25', '-0.217', '-0.201572825833']
HoNpPt 2 ['Ho0.111111Pt0.777778Np0.111111', '-0.814', '-0.251260931437']
HoNpSe 5 ['Se0.666667Ho0.166667Np0.166667', '-1.453', '-0.207876887849']
HoOP 14 ['O0.5P0.125Ho0.375', '-3.613', '-0.219557172087']
HoOPa 5 ['O0.714286Ho0.142857Pa0.142857', '-2.943', '-0.231322822924']
HoOPb 1 ['O0.6Ho0.3Pb0.1', '-3.435', '-0.220930828287']
HoOPd 2 ['O0.6Pd0.1Ho0.3', '-3.268', '-0.23251747958']
HoORh 1 ['O0.6Rh0.1Ho0.3', '-3.47', '-0.330892855786']
HoORu 1 ['O0.6Ru0.2Ho0.2', '-2.721', '-0.214066266431']
HoOS 14 ['O0.444444S0.222222Ho0.333333', '-3.351', '-0.209084428601']
HoOSc 3 ['O0.666667Sc0.111111Ho0.222222', '-3.436', '-0.221834310114']
HoOSe 24 ['O0.125Se0.375Ho0.5', '-2.312', '-0.203246092806']
HoOSi 2 ['O0.666667Si0.222222Ho0.111111', '-3.367', '-0.215616909095']
HoOTa 1 ['O0.666667Ho0.222222Ta0.111111', '-3.681', '-0.230667319559']
HoOTc 4 ['O0.7Tc0.2Ho0.1', '-2.384', '-0.207864477987']
HoOTe 5 ['O0.7Te0.2Ho0.1', '-2.042', '-0.200670676653']
HoOTi 1 ['O0.6Ti0.2Ho0.2', '-3.721', '-0.222987669925']
HoOU 1 ['O0.7Ho0.1U0.2', '-3.857', '-0.420296839545']
HoOV 1 ['O0.625V0.125Ho0.25', '-3.805', '-0.394098268971']
HoOW 4 ['O0.625Ho0.25W0.125', '-3.501', '-0.215517272889']
HoOZn 2 ['O0.6Zn0.1Ho0.3', '-3.463', '-0.22761723158']
HoOZr 3 ['O0.666667Zr0.111111Ho0.222222', '-3.569', '-0.205708144742']
HoOsP 3 ['P0.444444Ho0.222222Os0.333333', '-1.219', '-0.215677436387']
HoOsPd 6 ['Pd0.2Ho0.6Os0.2', '-0.613', '-0.211814044833']
HoOsPt 1 ['Ho0.6Os0.3Pt0.1', '-0.525', '-0.214861605779']
HoOsRh 1 ['Rh0.1Ho0.6Os0.3', '-0.473', '-0.239438749778']
HoOsRu 3 ['Ru0.142857Ho0.571429Os0.285714', '-0.362', '-0.228173175714']
HoOsS 2 ['S0.4Ho0.5Os0.1', '-1.923', '-0.215659901731']
HoOsSi 2 ['Si0.428571Ho0.142857Os0.428571', '-0.834', '-0.210962855655']
HoOsSm 1 ['Sm0.125Ho0.5Os0.375', '-0.305', '-0.212883240312']

HoOsTa 4 ['Ho0.25Ta0.125Os0.625', '-0.296', '-0.203210941667']
HoOsTc 2 ['Tc0.125Ho0.625Os0.25', '-0.24', '-0.209145651667']
HoOsXe 3 ['Xe0.142857Ho0.571429Os0.285714', '-0.245', '-0.209737887619']
HoOsYb 1 ['Ho0.555556Yb0.111111Os0.333333', '-0.283', '-0.241860868889']
HoOsZn 2 ['Zn0.142857Ho0.571429Os0.285714', '-0.341', '-0.206308062857']
HoPPd 14 ['P0.4Pd0.4Ho0.2', '-1.23', '-0.200286750448']
HoPPm 4 ['P0.5Pm0.375Ho0.125', '-1.533', '-0.204013281665']
HoPPt 1 ['P0.25Ho0.125Pt0.625', '-1.068', '-0.207040007423']
HoPRe 2 ['P0.5Ho0.166667Re0.333333', '-1.107', '-0.215660297963']
HoPRh 23 ['P0.444444Rh0.222222Ho0.333333', '-1.644', '-0.201574791571']
HoPSi 1 ['Si0.2P0.4Ho0.4', '-1.621', '-0.22848631508']
HoPTa 3 ['P0.5Ho0.4Ta0.1', '-1.738', '-0.210115030372']
HoPTc 4 ['P0.5Tc0.3Ho0.2', '-1.268', '-0.205836434709']
HoPXe 1 ['P0.8Xe0.1Ho0.1', '-0.654', '-0.210146371687']
HoPaPt 1 ['Ho0.1Pt0.8Pa0.1', '-1.009', '-0.207642192668']
HoPaS 7 ['S0.625Ho0.125Pa0.25', '-1.933', '-0.208302746803']
HoPaSe 8 ['Se0.625Ho0.25Pa0.125', '-1.741', '-0.258964608544']
HoPaSi 3 ['Si0.666667Ho0.166667Pa0.166667', '-0.614', '-0.209574732361']
HoPdS 7 ['S0.5Pd0.25Ho0.25', '-1.743', '-0.202112427298']
HoPdSe 16 ['Se0.375Pd0.375Ho0.25', '-1.388', '-0.202606547085']
HoPdSi 3 ['Si0.3Pd0.4Ho0.3', '-1.132', '-0.20087976925']
HoPdTe 1 ['Pd0.125Te0.5Ho0.375', '-1.337', '-0.207809596666']
HoPdZn 1 ['Zn0.125Pd0.375Ho0.5', '-1.0', '-0.208965992396']
HoPmS 3 ['S0.5Pm0.375Ho0.125', '-2.151', '-0.218551140388']
HoPmSe 8 ['Se0.666667Pm0.166667Ho0.166667', '-1.528', '-0.200320564446']
HoPtS 19 ['S0.5Ho0.333333Pt0.166667', '-2.084', '-0.202302888517']
HoPtSe 31 ['Se0.333333Ho0.5Pt0.166667', '-1.792', '-0.210158194997']
HoPtSi 1 ['Si0.5Ho0.125Pt0.375', '-1.078', '-0.234220261769']
HoPtTh 4 ['Ho0.125Pt0.75Th0.125', '-1.045', '-0.22024614021']
HoPtU 1 ['Ho0.1Pt0.8U0.1', '-0.932', '-0.217795995668']
HoPtZr 1 ['Zr0.1Ho0.1Pt0.8', '-1.026', '-0.202451897668']
HoPuS 9 ['S0.5Ho0.333333Pu0.166667', '-2.259', '-0.20690891855']
HoPuSe 1 ['Se0.6Ho0.2Pu0.2', '-1.852', '-0.210711945834']
HoRbS 2 ['S0.5Rb0.1Ho0.4', '-2.285', '-0.213647463662']
HoRbTe 3 ['Rb0.142857Te0.571429Ho0.285714', '-1.33', '-0.211111202141']
HoReS 4 ['S0.571429Ho0.285714Re0.142857', '-1.969', '-0.20220196708']
HoReSi 4 ['Si0.375Ho0.125Re0.5', '-0.582', '-0.213911617306']
HoRhS 38 ['S0.2Rh0.3Ho0.5', '-1.601', '-0.210482828532']
HoRhSe 24 ['Se0.5Rh0.25Ho0.25', '-1.462', '-0.20440890495']
HoRhSi 3 ['Si0.5Rh0.4Ho0.1', '-1.114', '-0.218243738165']
HoRuS 6 ['S0.3Ru0.2Ho0.5', '-1.619', '-0.209760634131']
HoRuSe 9 ['Se0.5Ru0.125Ho0.375', '-1.816', '-0.204494174376']
HoSSb 7 ['S0.375Sb0.25Ho0.375', '-1.916', '-0.231287316826']
HoSSe 9 ['S0.166667Se0.5Ho0.333333', '-1.885', '-0.210505857758']

HoSSi 18 ['Si0.333333S0.333333Ho0.333333', '-1.654', '-0.203477296902']
HoSSn 6 ['S0.5Sn0.1Ho0.4', '-2.217', '-0.223212910474']
HoSTa 1 ['S0.5Ho0.2Ta0.3', '-1.818', '-0.253946966743']
HoSTc 17 ['S0.5Tc0.166667Ho0.333333', '-2.082', '-0.207928380492']
HoSTe 1 ['S0.1Te0.5Ho0.4', '-1.577', '-0.223701244987']
HoSTh 7 ['S0.555556Ho0.333333Th0.111111', '-2.367', '-0.203814736843']
HoSTl 6 ['S0.5Ho0.4Tl0.1', '-2.175', '-0.203057848827']
HoSXe 2 ['S0.5Xe0.375Ho0.125', '-0.926', '-0.223223142684']
HoSZn 3 ['S0.5Zn0.25Ho0.25', '-1.853', '-0.208445676117']
HoSZr 3 ['S0.5Zr0.375Ho0.125', '-1.886', '-0.22673442549']
HoSbSe 3 ['Se0.333333Sb0.222222Ho0.444444', '-1.769', '-0.222913786852']
HoScSe 7 ['Sc0.111111Se0.444444Ho0.444444', '-1.745', '-0.214415918516']
HoSeTc 7 ['Se0.555556Tc0.222222Ho0.222222', '-1.257', '-0.207196397594']
HoSeTe 24 ['Se0.4Te0.1Ho0.5', '-1.827', '-0.200705273998']
HoSeTl 5 ['Se0.5Ho0.375Tl0.125', '-1.817', '-0.205494174376']
HoSeY 6 ['Se0.571429Y0.285714Ho0.142857', '-1.9', '-0.216039839882']
HoSeZn 13 ['Zn0.1Se0.5Ho0.4', '-1.854', '-0.202195562835']
HoSeZr 2 ['Se0.5Zr0.125Ho0.375', '-1.845', '-0.21823871776']
HoSiTc 3 ['Si0.444444Tc0.444444Ho0.111111', '-0.688', '-0.21497351713']
IInK 5 ['K0.166667In0.333333I0.5', '-1.108', '-0.203283676287']
IInLa 7 ['In0.25I0.5La0.25', '-1.48', '-0.201520567953']
IInLu 32 ['In0.3I0.6Lu0.1', '-0.814', '-0.20532726571']
IInNa 1 ['Na0.5In0.166667I0.333333', '-1.223', '-0.209654472931']
IInNd 7 ['In0.2I0.7Nd0.1', '-1.071', '-0.203504999674']
IInNp 1 ['In0.222222I0.666667Np0.111111', '-1.029', '-0.243697524769']
IInPa 16 ['In0.222222I0.555556Pa0.222222', '-0.721', '-0.230576947401']
IInPm 42 ['In0.285714I0.285714Pm0.428571', '-0.886', '-0.204493711806']
IInPr 3 ['In0.142857I0.571429Pr0.285714', '-1.528', '-0.204257178042']
IInPt 17 ['In0.375I0.5Pt0.125', '-0.81', '-0.210363959098']
IInPu 1 ['In0.111111I0.666667Pu0.222222', '-1.282', '-0.208281585049']
IInRh 5 ['Rh0.3In0.1I0.6', '-0.475', '-0.224214962522']
IInSm 18 ['In0.125I0.5Sm0.375', '-1.254', '-0.203358689095']
IInTb 42 ['In0.25I0.25Tb0.5', '-0.51', '-0.208893651171']
IInTm 7 ['In0.111111I0.777778Tm0.111111', '-0.91', '-0.214013025594']
IInY 1 ['Y0.4In0.2I0.4', '-1.306', '-0.258437094863']
IIrLa 7 ['I0.5La0.4Ir0.1', '-1.504', '-0.203604361984']
IIrLu 36 ['I0.3Lu0.4Ir0.3', '-0.9', '-0.204663747757']
IIrNd 23 ['I0.5Nd0.25Ir0.25', '-1.267', '-0.201714367532']
IIrP 5 ['P0.2I0.5Ir0.3', '-0.486', '-0.203659928041']
IIrPa 32 ['I0.375Ir0.375Pa0.25', '-0.671', '-0.211322740947']
IIrPm 47 ['I0.428571Pm0.142857Ir0.428571', '-0.512', '-0.256393286439']
IIrPr 1 ['I0.222222Pr0.444444Ir0.333333', '-1.02', '-0.207568195153']
IIrRh 1 ['Rh0.2I0.7Ir0.1', '-0.204', '-0.200179597']
IIrSm 31 ['I0.666667Sm0.111111Ir0.222222', '-0.631', '-0.206709531691']

IIrTb 40 ['I0.375Tb0.25Ir0.375', '-0.624', '-0.241265976494']
IIrTm 20 ['I0.555556Tm0.333333Ir0.111111', '-1.391', '-0.208765429758']
IIrXe 14 ['I0.555556Xe0.222222Ir0.222222', '-0.23', '-0.23']
IKLu 16 ['K0.142857I0.571429Lu0.285714', '-0.978', '-0.208003623961']
IKMg 2 ['Mg0.3K0.1I0.6', '-1.439', '-0.241374856751']
IKNd 7 ['K0.25I0.625Nd0.125', '-1.533', '-0.208449759411']
IKNp 5 ['K0.142857I0.714286Np0.142857', '-1.233', '-0.204562749506']
IKO 11 ['O0.5K0.375I0.125', '-1.581', '-0.205812713167']
IKPa 12 ['K0.125I0.625Pa0.25', '-0.711', '-0.245706929715']
IKPm 17 ['K0.222222I0.555556Pm0.222222', '-1.302', '-0.200471846145']
IKPt 1 ['K0.125I0.625Pt0.25', '-0.799', '-0.203628826608']
IKPu 3 ['K0.125I0.75Pu0.125', '-1.238', '-0.238079679412']
IKRh 5 ['K0.1Rh0.2I0.7', '-0.537', '-0.220237030258']
IKSc 8 ['K0.222222Sc0.111111I0.666667', '-1.321', '-0.206680625032']
IKSi 3 ['Si0.166667K0.166667I0.666667', '-1.066', '-0.206472765049']
IKSm 7 ['K0.1I0.8Sm0.1', '-0.933', '-0.23437560878']
IKTb 14 ['K0.3I0.6Tb0.1', '-1.277', '-0.242010592065']
IKTe 18 ['K0.4Te0.5I0.1', '-1.062', '-0.202835078758']
IKTm 6 ['K0.111111I0.777778Tm0.111111', '-0.983', '-0.213704211968']
IKU 2 ['K0.111111I0.777778U0.111111', '-1.106', '-0.215672415318']
IKW 3 ['K0.1I0.8W0.1', '-0.566', '-0.249237030258']
IKrLu 26 ['Kr0.1I0.5Lu0.4', '-0.657', '-0.212531013029']
IKrNd 5 ['Kr0.125I0.75Nd0.125', '-0.738', '-0.205357183766']
IKrPa 25 ['Kr0.375I0.5Pa0.125', '-0.287', '-0.252330320946']
IKrPm 25 ['Kr0.333333I0.555556Pm0.111111', '-0.412', '-0.213194778341']
IKrRh 1 ['Kr0.111111Rh0.222222I0.666667', '-0.213', '-0.213']
IKrSm 3 ['Kr0.166667I0.666667Sm0.166667', '-0.837', '-0.200564297536']
IKrTb 29 ['Kr0.333333I0.444444Tb0.222222', '-0.394', '-0.205776669535']
IKrTm 4 ['Kr0.166667I0.666667Tm0.166667', '-0.837', '-0.210994600855']
ILaLu 6 ['I0.666667La0.111111Lu0.222222', '-1.313', '-0.322932279763']
ILaN 29 ['N0.142857I0.571429La0.285714', '-1.756', '-0.210341143819']
ILaNa 1 ['Na0.142857I0.571429La0.285714', '-1.666', '-0.268221462776']
ILaNd 2 ['I0.777778La0.111111Nd0.111111', '-1.426', '-0.20939921365']
ILaNi 1 ['Ni0.166667I0.666667La0.166667', '-1.405', '-0.235613777549']
ILaO 23 ['O0.333333I0.111111La0.555556', '-2.577', '-0.200623767678']
ILaOs 3 ['I0.428571La0.428571Os0.142857', '-1.229', '-0.225191148246']
ILaP 4 ['P0.4I0.1La0.5', '-1.832', '-0.211586152674']
ILaPa 5 ['I0.714286La0.142857Pa0.142857', '-1.196', '-0.200911145756']
ILaPd 34 ['Pd0.166667I0.333333La0.5', '-1.253', '-0.200977305027']
ILaPm 6 ['I0.666667La0.111111Pm0.222222', '-1.359', '-0.218249051429']
ILaPt 50 ['I0.428571La0.142857Pt0.428571', '-1.157', '-0.201533636103']
ILaPu 2 ['I0.8La0.1Pu0.1', '-1.37', '-0.218000258544']
ILaRh 40 ['Rh0.125I0.625La0.25', '-1.684', '-0.203000943067']
ILaRu 1 ['Ru0.2I0.5La0.3', '-1.455', '-0.226027428121']

ILaS 1 ['S0.444444I0.111111La0.444444', '-2.445', '-0.218311564981']
ILaSb 30 ['Sb0.166667I0.5La0.333333', '-1.742', '-0.20202114462']
ILaSc 1 ['Sc0.125I0.75La0.125', '-1.528', '-0.230265059106']
ILaSe 3 ['Se0.3I0.2La0.5', '-1.887', '-0.222037623516']
ILaSi 5 ['Si0.3I0.3La0.4', '-1.328', '-0.202027559104']
ILaSm 2 ['I0.8La0.1Sm0.1', '-1.309', '-0.258312123794']
ILaTb 6 ['I0.666667La0.111111Tb0.222222', '-1.196', '-0.264636164281']
ILaTe 12 ['Te0.3I0.4La0.3', '-1.772', '-0.202297106904']
ILaTl 2 ['I0.625La0.25Tl0.125', '-1.661', '-0.209843340358']
ILaW 1 ['I0.8La0.1W0.1', '-0.898', '-0.229173545272']
ILiLu 19 ['Li0.1I0.8Lu0.1', '-0.614', '-0.238495320515']
ILiMg 1 ['Li0.222222Mg0.222222I0.555556', '-1.379', '-0.204230922679']
ILiNa 3 ['Li0.166667Na0.333333I0.5', '-1.591', '-0.20627730767']
ILiNd 14 ['Li0.375I0.5Nd0.125', '-1.463', '-0.205225719101']
ILiNp 3 ['Li0.111111I0.777778Np0.111111', '-0.954', '-0.212299401838']
ILiO 10 ['Li0.4O0.5I0.1', '-1.746', '-0.20480548257']
ILiPa 15 ['Li0.285714I0.571429Pa0.142857', '-1.004', '-0.208984844676']
ILiPm 19 ['Li0.111111I0.555556Pm0.333333', '-1.201', '-0.310820520866']
ILiPr 15 ['Li0.142857I0.571429Pr0.285714', '-1.501', '-0.200223120396']
ILiPu 3 ['Li0.166667I0.666667Pu0.166667', '-1.447', '-0.20106546755']
ILiRe 2 ['Li0.111111I0.777778Re0.111111', '-0.584', '-0.206450348551']
ILiRh 4 ['Li0.1Rh0.2I0.7', '-0.469', '-0.204612567258']
ILiSc 5 ['Li0.222222Sc0.111111I0.666667', '-1.216', '-0.218070707254']
ILiSe 1 ['Li0.444444Se0.222222I0.333333', '-1.329', '-0.235692261972']
ILiSm 8 ['Li0.3I0.6Sm0.1', '-1.405', '-0.229976280296']
ILiTb 19 ['Li0.1I0.8Tb0.1', '-0.668', '-0.318912068549']
ILiTh 2 ['Li0.1I0.7Th0.2', '-1.517', '-0.212409246552']
ILiTm 9 ['Li0.125I0.75Tm0.125', '-1.002', '-0.202011659714']
ILiU 1 ['Li0.2I0.7U0.1', '-1.24', '-0.224502105544']
ILiV 1 ['Li0.166667V0.166667I0.666667', '-0.936', '-0.21132144462']
ILiW 4 ['Li0.142857I0.714286W0.142857', '-0.61', '-0.232303667511']
ILiXe 4 ['Li0.125I0.5Xe0.375', '-0.546', '-0.215515709073']
ILiYb 1 ['Li0.1I0.6Yb0.3', '-1.823', '-0.222492432542']
ILuMg 11 ['Mg0.1I0.6Lu0.3', '-0.969', '-0.283303390369']
ILuMn 22 ['Mn0.3I0.5Lu0.2', '-0.686', '-0.209954621217']
ILuMo 9 ['Mo0.125I0.625Lu0.25', '-0.668', '-0.202655713682']
ILuN 26 ['N0.3I0.1Lu0.6', '-1.504', '-0.202750008155']
ILuNa 16 ['Na0.142857I0.571429Lu0.285714', '-0.926', '-0.203917735981']
ILuNb 9 ['Nb0.125I0.625Lu0.25', '-0.773', '-0.203395353483']
ILuNd 10 ['I0.6Nd0.1Lu0.3', '-0.988', '-0.228534006785']
ILuNi 36 ['Ni0.444444I0.444444Lu0.111111', '-0.521', '-0.20227015892']
ILuNp 6 ['I0.666667Lu0.222222Np0.111111', '-1.054', '-0.359136001012']
ILuO 15 ['O0.142857I0.428571Lu0.428571', '-1.798', '-0.220673744546']
ILuOs 30 ['I0.5Lu0.2Os0.3', '-0.456', '-0.222957929629']

ILuP 24 ['P0.2I0.6Lu0.2', '-0.909', '-0.224499929208']
ILuPa 17 ['I0.555556Lu0.333333Pa0.111111', '-0.651', '-0.249791685032']
ILuPb 27 ['I0.428571Lu0.285714Pb0.285714', '-0.854', '-0.21297702594']
ILuPd 46 ['Pd0.222222I0.333333Lu0.444444', '-0.891', '-0.203459503172']
ILuPm 19 ['I0.555556Pm0.222222Lu0.222222', '-0.883', '-0.238462341699']
ILuPr 6 ['I0.714286Pr0.142857Lu0.142857', '-1.256', '-0.330576103787']
ILuPt 58 ['I0.1Lu0.6Pt0.3', '-1.186', '-0.209957035753']
ILuPu 11 ['I0.555556Lu0.333333Pu0.111111', '-0.994', '-0.21021357754']
ILuRb 16 ['Rb0.333333I0.555556Lu0.111111', '-1.429', '-0.211958033931']
ILuRe 16 ['I0.555556Lu0.222222Re0.222222', '-0.542', '-0.211286947725']
ILuRh 47 ['Rh0.5I0.375Lu0.125', '-0.492', '-0.202240451875']
ILuRu 26 ['Ru0.333333I0.555556Lu0.111111', '-0.331', '-0.200664991111']
ILuS 32 ['S0.444444I0.111111Lu0.444444', '-2.021', '-0.202298347111']
ILuSb 34 ['Sb0.111111I0.444444Lu0.444444', '-0.838', '-0.212611146976']
ILuSc 10 ['Sc0.125I0.625Lu0.25', '-0.963', '-0.223505010659']
ILuSe 52 ['Se0.428571I0.142857Lu0.428571', '-1.69', '-0.204315130367']
ILuSi 19 ['Si0.25I0.5Lu0.25', '-0.853', '-0.247484386036']
ILuSm 8 ['I0.666667Sm0.166667Lu0.166667', '-1.076', '-0.254368886298']
ILuSn 25 ['Sn0.25I0.5Lu0.25', '-0.828', '-0.201576936126']
ILuSr 9 ['Sr0.222222I0.666667Lu0.111111', '-1.543', '-0.202233926428']
ILuTa 6 ['I0.666667Lu0.222222Ta0.111111', '-0.827', '-0.252576190325']
ILuTb 17 ['I0.555556Tb0.333333Lu0.111111', '-0.651', '-0.24520139681']
ILuTc 23 ['Tc0.1I0.5Lu0.4', '-0.646', '-0.201531013029']
ILuTe 63 ['Te0.3I0.6Lu0.1', '-0.658', '-0.209659463106']
ILuTh 4 ['I0.7Lu0.2Th0.1', '-1.237', '-0.328157106794']
ILuTi 10 ['Ti0.1I0.6Lu0.3', '-0.914', '-0.210599369164']
ILuTl 36 ['I0.3Lu0.4Tl0.3', '-0.713', '-0.209567166272']
ILuTm 7 ['I0.666667Tm0.111111Lu0.222222', '-1.032', '-0.367735852253']
ILuU 4 ['I0.7Lu0.2U0.1', '-1.068', '-0.361233650043']
ILuV 10 ['V0.1I0.6Lu0.3', '-0.817', '-0.313228559286']
ILuW 10 ['I0.7Lu0.1W0.2', '-0.361', '-0.249882753257']
ILuXe 36 ['I0.5Xe0.1Lu0.4', '-0.649', '-0.204531013029']
ILuY 6 ['Y0.111111I0.666667Lu0.222222', '-1.171', '-0.229107745318']
ILuYb 9 ['I0.625Yb0.125Lu0.25', '-1.158', '-0.213328730036']
ILuZn 30 ['Zn0.333333I0.555556Lu0.111111', '-0.768', '-0.213429010318']
ILuZr 6 ['Zr0.111111I0.666667Lu0.222222', '-0.999', '-0.268026953372']
IMgNd 4 ['Mg0.1I0.7Nd0.2', '-1.448', '-0.243426624623']
IMgNp 2 ['Mg0.125I0.75Np0.125', '-1.183', '-0.238640031241']
IMgPa 9 ['Mg0.222222I0.666667Pa0.111111', '-1.016', '-0.202193908835']
IMgPm 13 ['Mg0.111111I0.555556Pm0.333333', '-1.19', '-0.202090035687']
IMgPt 5 ['Mg0.285714I0.571429Pt0.142857', '-1.221', '-0.214300373134']
IMgPu 1 ['Mg0.222222I0.666667Pu0.111111', '-1.344', '-0.20310526301']
IMgRh 7 ['Mg0.142857Rh0.285714I0.571429', '-0.71', '-0.206650186567']
IMgSe 1 ['Mg0.285714Se0.142857I0.571429', '-1.27', '-0.263300373134']

IMgSi 1 ['Mg0.2Si0.1I0.7', '-1.105', '-0.201356889966']
IMgSm 4 ['Mg0.2I0.7Sm0.1', '-1.31', '-0.223448839716']
IMgSr 1 ['Mg0.25Sr0.125I0.625', '-1.646', '-0.300620732699']
IMgTb 12 ['Mg0.25I0.625Tb0.125', '-1.228', '-0.241262203106']
IMgTh 3 ['Mg0.25I0.625Th0.125', '-1.318', '-0.215359718637']
IMgTm 4 ['Mg0.125I0.75Tm0.125', '-1.123', '-0.213064863888']
IMgYb 2 ['Mg0.3I0.6Yb0.1', '-1.468', '-0.229807738708']
IMnNd 2 ['Mn0.142857I0.714286Nd0.142857', '-1.058', '-0.207540700211']
IMnO 1 ['O0.6Mn0.3I0.1', '-1.862', '-0.200192715233']
IMnPa 10 ['Mn0.125I0.625Pa0.25', '-0.507', '-0.226151570812']
IMnPm 10 ['Mn0.2I0.7Pm0.1', '-0.758', '-0.240660786777']
IMnPt 15 ['Mn0.1I0.7Pt0.2', '-0.566', '-0.209017228921']
IMnRh 4 ['Mn0.1Rh0.3I0.6', '-0.393', '-0.223792743135']
IMnSm 1 ['Mn0.1I0.7Sm0.2', '-1.193', '-0.27173578767']
IMnTb 20 ['Mn0.166667I0.5Tb0.333333', '-0.625', '-0.201658956915']
IMnTm 3 ['Mn0.125I0.75Tm0.125', '-0.909', '-0.22798687956']
IMnXe 9 ['Mn0.166667I0.5Xe0.333333', '-0.489', '-0.206987905225']
IMoNd 2 ['Mo0.111111I0.777778Nd0.111111', '-0.88', '-0.239827568271']
IMoO 2 ['O0.666667Mo0.222222I0.111111', '-2.171', '-0.248354323307']
IMoPa 7 ['Mo0.2I0.7Pa0.1', '-0.537', '-0.209182385619']
IMoPm 9 ['Mo0.1I0.6Pm0.3', '-0.917', '-0.230184965953']
IMoPt 4 ['Mo0.1I0.6Pt0.3', '-0.556', '-0.233382795154']
IMoRh 6 ['Mo0.1Rh0.3I0.6', '-0.354', '-0.203959064431']
IMoSe 9 ['Se0.222222Mo0.111111I0.666667', '-0.433', '-0.20914651']
IMoSm 2 ['Mo0.111111I0.777778Sm0.111111', '-0.816', '-0.224997381058']
IMoTb 10 ['Mo0.2I0.7Tb0.1', '-0.599', '-0.214217630152']
IMoTm 2 ['Mo0.1I0.8Tm0.1', '-0.755', '-0.229355824944']
IMoXe 4 ['Mo0.125I0.5Xe0.375', '-0.401', '-0.213448830539']
INNd 9 ['N0.2I0.5Nd0.3', '-1.423', '-0.211324368516']
INNp 31 ['N0.1I0.7Np0.2', '-1.034', '-0.227713788792']
INPa 55 ['N0.25I0.25Pa0.5', '-1.005', '-0.229372177734']
INPm 36 ['N0.166667I0.333333Pm0.5', '-1.314', '-0.203737638717']
INPr 27 ['N0.4I0.2Pr0.4', '-1.545', '-0.224106190606']
INPu 3 ['N0.555556I0.111111Pu0.333333', '-1.216', '-0.225715364815']
INSc 12 ['N0.222222Sc0.444444I0.333333', '-1.725', '-0.211355576613']
INSm 19 ['N0.1I0.7Sm0.2', '-0.966', '-0.202277157043']
INTb 35 ['N0.375I0.125Tb0.5', '-1.401', '-0.217924794106']
INTi 4 ['N0.5Ti0.375I0.125', '-1.321', '-0.203963701302']
INTm 31 ['N0.428571I0.142857Tm0.428571', '-1.893', '-0.239289537711']
INU 2 ['N0.222222I0.555556U0.222222', '-1.26', '-0.213241204427']
INXe 5 ['N0.1I0.5Xe0.4', '-0.227', '-0.227']
INY 12 ['N0.333333Y0.444444I0.222222', '-1.865', '-0.204874810088']
INaNd 3 ['Na0.166667I0.666667Nd0.166667', '-1.391', '-0.208771092808']
INaNp 2 ['Na0.111111I0.777778Np0.111111', '-0.998', '-0.235370892298']

INaO 26 ['O0.5Na0.3I0.2', '-1.465', '-0.200107133894']
INaPa 12 ['Na0.125I0.625Pa0.25', '-0.637', '-0.213631777733']
INaPb 4 ['Na0.4I0.5Pb0.1', '-1.463', '-0.21297789757']
INaPm 16 ['Na0.142857I0.571429Pm0.285714', '-1.151', '-0.235182156695']
INaPr 2 ['Na0.1I0.7Pr0.2', '-1.561', '-0.204417947459']
INaPu 3 ['Na0.125I0.75Pu0.125', '-1.185', '-0.22700452743']
INaRe 2 ['Na0.125I0.75Re0.125', '-0.656', '-0.207712068888']
INaRh 3 ['Na0.142857Rh0.285714I0.571429', '-0.628', '-0.223395583817']
INaS 2 ['Na0.5S0.125I0.375', '-1.522', '-0.201198699412']
INaSb 10 ['Na0.333333Sb0.222222I0.444444', '-1.229', '-0.212905296229']
INaSc 1 ['Na0.1Sc0.2I0.7', '-1.232', '-0.210053912698']
INaSi 1 ['Na0.25Si0.125I0.625', '-1.175', '-0.218250557644']
INaSm 4 ['Na0.111111I0.666667Sm0.222222', '-1.36', '-0.209688836896']
INaTb 19 ['Na0.333333I0.555556Tb0.111111', '-1.3', '-0.261811363674']
INaTe 8 ['Na0.428571Te0.428571I0.142857', '-1.041', '-0.203012440241']
INaTm 2 ['Na0.1I0.7Tm0.2', '-1.31', '-0.275570429698']
INaW 1 ['Na0.111111I0.777778W0.111111', '-0.533', '-0.218307676302']
INaXe 4 ['Na0.166667I0.5Xe0.333333', '-0.675', '-0.202961514453']
INbNd 3 ['Nb0.111111I0.777778Nd0.111111', '-1.064', '-0.20982788311']
INbNi 2 ['Ni0.3Nb0.1I0.6', '-0.582', '-0.206554069043']
INbO 9 ['O0.375Nb0.375I0.25', '-2.001', '-0.201148771355']
INbPa 5 ['Nb0.142857I0.714286Pa0.142857', '-0.712', '-0.22251335398']
INbPd 4 ['Nb0.142857Pd0.142857I0.714286', '-0.719', '-0.202050012706']
INbPm 9 ['Nb0.125I0.625Pm0.25', '-0.943', '-0.203876721607']
INbPt 11 ['Nb0.25I0.625Pt0.125', '-0.817', '-0.206678204732']
INbRh 9 ['Nb0.125Rh0.25I0.625', '-0.639', '-0.210699184733']
INbSe 3 ['Se0.6Nb0.2I0.2', '-0.783', '-0.200491922385']
INbSm 1 ['Nb0.1I0.8Sm0.1', '-0.986', '-0.261497926308']
INbTb 12 ['Nb0.142857I0.571429Tb0.285714', '-0.678', '-0.213665981089']
INbTe 1 ['Nb0.2Te0.3I0.5', '-0.73', '-0.204667446119']
INbTm 2 ['Nb0.1I0.8Tm0.1', '-0.952', '-0.233756108299']
INdNi 19 ['Ni0.3I0.5Nd0.2', '-1.114', '-0.228966215283']
INdNp 3 ['I0.75Nd0.125Np0.125', '-1.397', '-0.360428301761']
INdO 23 ['O0.6I0.2Nd0.2', '-2.334', '-0.202750582382']
INdOs 5 ['I0.75Nd0.125Os0.125', '-0.739', '-0.206357183766']
INdP 15 ['P0.2I0.5Nd0.3', '-1.476', '-0.201297845928']
INdPa 6 ['I0.7Nd0.2Pa0.1', '-1.175', '-0.295035750783']
INdPb 3 ['I0.571429Nd0.285714Pb0.142857', '-1.419', '-0.201530705751']
INdPd 40 ['Pd0.111111I0.555556Nd0.333333', '-1.517', '-0.200913422814']
INdPm 10 ['I0.625Nd0.125Pm0.25', '-1.199', '-0.219045435034']
INdPr 2 ['I0.777778Pr0.111111Nd0.111111', '-1.352', '-0.282229184896']
INdPt 61 ['I0.1Nd0.5Pt0.4', '-1.336', '-0.203851928507']
INdPu 5 ['I0.7Nd0.1Pu0.2', '-1.438', '-0.2065969358']
INdRb 5 ['Rb0.111111I0.777778Nd0.111111', '-1.139', '-0.256762586427']

INdRe 7 ['I0.666667Nd0.222222Re0.111111', '-1.213', '-0.210222212946']
INdRh 35 ['Rh0.111111I0.555556Nd0.333333', '-1.514', '-0.220743347814']
INdRu 19 ['Ru0.142857I0.428571Nd0.428571', '-1.143', '-0.203787078956']
INdS 15 ['S0.3I0.2Nd0.5', '-1.95', '-0.220256228307']
INdSb 33 ['Sb0.222222I0.666667Nd0.111111', '-0.969', '-0.207468788195']
INdSc 6 ['Sc0.2I0.7Nd0.1', '-1.41', '-0.245162751039']
INdSe 42 ['Se0.5I0.1Nd0.4', '-1.964', '-0.203651921256']
INdSi 11 ['Si0.222222I0.555556Nd0.222222', '-1.248', '-0.227393004018']
INdSm 4 ['I0.714286Nd0.142857Sm0.142857', '-1.418', '-0.263749036478']
INdSn 2 ['Sn0.142857I0.714286Nd0.142857', '-1.137', '-0.205379265082']
INdTa 1 ['I0.8Nd0.1Ta0.1', '-0.989', '-0.268138811792']
INdTb 9 ['I0.666667Nd0.222222Tb0.111111', '-1.253', '-0.211967772574']
INdTc 7 ['Tc0.222222I0.666667Nd0.111111', '-0.689', '-0.215539718903']
INdTe 44 ['Te0.428571I0.428571Nd0.142857', '-0.895', '-0.204441415834']
INdTi 3 ['Ti0.125I0.75Nd0.125', '-1.29', '-0.220541941454']
INdTl 21 ['I0.5Nd0.2Tl0.3', '-1.205', '-0.200314396283']
INdTm 4 ['I0.714286Nd0.142857Tm0.142857', '-1.372', '-0.226689296466']
INdU 2 ['I0.777778Nd0.111111U0.111111', '-1.268', '-0.256170989491']
INdV 5 ['V0.142857I0.714286Nd0.142857', '-1.108', '-0.255808637896']
INdW 5 ['I0.714286Nd0.142857W0.142857', '-0.834', '-0.225265352876']
INdXe 15 ['I0.444444Xe0.444444Nd0.111111', '-0.706', '-0.232539718903']
INdY 2 ['Y0.111111I0.777778Nd0.111111', '-1.395', '-0.226574679206']
INdYb 2 ['I0.75Nd0.125Yb0.125', '-1.413', '-0.213479030659']
INdZn 7 ['Zn0.222222I0.666667Nd0.111111', '-1.08', '-0.211642324491']
INdZr 2 ['Zr0.111111I0.777778Nd0.111111', '-1.222', '-0.264493887259']
INiNp 15 ['Ni0.142857I0.714286Np0.142857', '-0.87', '-0.200351909872']
INiO 5 ['O0.5Ni0.333333I0.166667', '-1.173', '-0.207365642085']
INiP 3 ['P0.166667Ni0.333333I0.5', '-0.533', '-0.205958716438']
INiPa 32 ['Ni0.444444I0.444444Pa0.111111', '-0.495', '-0.215487873603']
INiPm 42 ['Ni0.3I0.3Pm0.4', '-0.856', '-0.238377689272']
INiPr 10 ['Ni0.2I0.7Pr0.1', '-0.868', '-0.200099404422']
INiPt 7 ['Ni0.285714I0.571429Pt0.142857', '-0.418', '-0.204510919326']
INiPu 4 ['Ni0.222222I0.666667Pu0.111111', '-0.85', '-0.203789863381']
INiRe 2 ['Ni0.1I0.7Re0.2', '-0.396', '-0.202140559315']
INiRh 2 ['Ni0.1Rh0.2I0.7', '-0.282', '-0.216389442514']
INiSc 6 ['Sc0.166667Ni0.166667I0.666667', '-0.93', '-0.205046574212']
INiSe 34 ['Ni0.444444Se0.444444I0.111111', '-0.482', '-0.20102973973']
INiSi 12 ['Si0.111111Ni0.333333I0.555556', '-0.566', '-0.211435297539']
INiSm 22 ['Ni0.3I0.5Sm0.2', '-0.978', '-0.204803653283']
INiTa 2 ['Ni0.333333I0.555556Ta0.111111', '-0.575', '-0.211053095595']
INiTb 45 ['Ni0.4I0.3Tb0.3', '-0.529', '-0.215559236599']
INiTe 1 ['Ni0.375Te0.125I0.5', '-0.415', '-0.200195185556']
INiTm 18 ['Ni0.4I0.5Tm0.1', '-0.72', '-0.245980924284']
INiU 4 ['Ni0.3I0.6U0.1', '-0.772', '-0.221857586043']

INiV 1 ['V0.142857Ni0.285714I0.571429', '-0.54', '-0.202813917183']
INiW 5 ['Ni0.333333I0.555556W0.111111', '-0.395', '-0.205280453789']
INiXe 20 ['Ni0.3I0.6Xe0.1', '-0.398', '-0.201168327543']
INiYb 2 ['Ni0.2I0.6Yb0.2', '-1.354', '-0.221384397542']
INiZr 7 ['Ni0.333333Zr0.111111I0.555556', '-0.758', '-0.201053548927']
INpO 9 ['O0.125I0.625Np0.25', '-1.732', '-0.200456083426']
INpP 1 ['P0.8I0.1Np0.1', '-0.478', '-0.20350168309']
INpPa 6 ['I0.666667Pa0.222222Np0.111111', '-0.714', '-0.204428231012']
INpPb 1 ['I0.7Pb0.1Np0.2', '-1.135', '-0.211584051674']
INpPd 10 ['Pd0.285714I0.571429Np0.142857', '-0.852', '-0.200269194326']
INpPm 5 ['I0.714286Pm0.142857Np0.142857', '-1.191', '-0.359474564147']
INpPr 3 ['I0.75Pr0.125Np0.125', '-1.416', '-0.241221767237']
INpPt 31 ['I0.6Pt0.1Np0.3', '-1.115', '-0.217785793417']
INpPu 2 ['I0.777778Np0.111111Pu0.111111', '-1.205', '-0.22020400852']
INpRb 5 ['Rb0.142857I0.714286Np0.142857', '-1.268', '-0.200647174515']
INpRe 2 ['I0.777778Re0.111111Np0.111111', '-0.747', '-0.215277378704']
INpRh 20 ['Rh0.25I0.5Np0.25', '-1.01', '-0.219959794931']
INpRu 6 ['Ru0.2I0.7Np0.1', '-0.611', '-0.207856894396']
INpSb 1 ['Sb0.1I0.8Np0.1', '-0.831', '-0.233409016168']
INpSc 3 ['Sc0.125I0.75Np0.125', '-1.282', '-0.316369245511']
INpSe 45 ['Se0.375I0.25Np0.375', '-1.321', '-0.209840101945']
INpSi 1 ['Si0.1I0.8Np0.1', '-0.872', '-0.213340019924']
INpSm 3 ['I0.75Sm0.125Np0.125', '-1.327', '-0.345744341147']
INpTb 7 ['I0.7Tb0.1Np0.2', '-1.097', '-0.206013290083']
INpTi 3 ['Ti0.1I0.7Np0.2', '-1.133', '-0.20336415859']
INpTl 6 ['I0.8Tl0.1Np0.1', '-0.8', '-0.210964605168']
INpTm 3 ['I0.75Tm0.125Np0.125', '-1.298', '-0.324567068636']
INpV 1 ['V0.1I0.8Np0.1', '-0.827', '-0.25343719391']
INpW 3 ['I0.75W0.125Np0.125', '-0.729', '-0.225071117995']
INpXe 11 ['I0.777778Xe0.111111Np0.111111', '-0.666', '-0.218063215996']
INpY 2 ['Y0.1I0.8Np0.1', '-1.23', '-0.201388358668']
INpYb 2 ['I0.75Yb0.125Np0.125', '-1.394', '-0.223192964888']
IOP 8 ['O0.428571P0.285714I0.285714', '-1.696', '-0.214859768656']
IOPa 34 ['O0.5I0.25Pa0.25', '-2.539', '-0.21489986581']
IOPb 3 ['O0.5I0.166667Pb0.333333', '-1.347', '-0.230779834491']
IOPm 21 ['O0.222222I0.333333Pm0.444444', '-2.332', '-0.205590505941']
IOPr 15 ['O0.333333I0.166667Pr0.5', '-2.626', '-0.201936924372']
IOPu 17 ['O0.3I0.2Pu0.5', '-2.562', '-0.211945663018']
IORb 9 ['O0.5Rb0.3I0.2', '-1.483', '-0.206574584077']
IORe 1 ['O0.1I0.7Re0.2', '-0.593', '-0.213885292309']
IOSb 22 ['O0.555556Sb0.333333I0.111111', '-1.673', '-0.206987687202']
IOSc 21 ['O0.375Sc0.375I0.25', '-3.059', '-0.209286122601']
IOSi 1 ['O0.666667Si0.222222I0.111111', '-2.438', '-0.205017376756']
IOSm 15 ['O0.222222I0.444444Sm0.333333', '-2.269', '-0.204059192705']

IOSr 8 ['O0.222222Sr0.333333I0.444444', '-2.186', '-0.204102606503']
IOTa 3 ['O0.666667I0.166667Ta0.166667', '-2.234', '-0.20109136642']
IOTb 54 ['O0.1I0.4Tb0.5', '-1.146', '-0.213820083556']
IOTc 2 ['O0.7Tc0.1I0.2', '-1.192', '-0.204832334694']
IOTe 7 ['O0.5Te0.2I0.3', '-1.097', '-0.234422812197']
IOTh 15 ['O0.5I0.1Th0.4', '-3.567', '-0.202637253945']
IOTl 1 ['O0.333333I0.222222Tl0.444444', '-1.114', '-0.254510093612']
IOTm 19 ['O0.111111I0.666667Tm0.222222', '-1.598', '-0.200235704647']
IOU 7 ['O0.222222I0.555556U0.222222', '-2.012', '-0.200531672611']
IOV 2 ['O0.6V0.3I0.1', '-2.494', '-0.240910204141']
IOXe 13 ['O0.222222I0.444444Xe0.333333', '-0.387', '-0.203054674922']
IOY 7 ['O0.4Y0.4I0.2', '-3.21', '-0.202226760163']
IOZr 13 ['O0.2Zr0.3I0.5', '-2.038', '-0.211232308107']
IOsP 11 ['P0.25I0.625Os0.125', '-0.459', '-0.20058316901']
IOsPa 32 ['I0.4Os0.3Pa0.3', '-0.336', '-0.222500103']
IOsPm 28 ['I0.333333Pm0.444444Os0.222222', '-0.848', '-0.251584335024']
IOsPt 3 ['I0.666667Os0.166667Pt0.166667', '-0.391', '-0.210985783786']
IOsRh 1 ['Rh0.2I0.7Os0.1', '-0.238', '-0.238']
IOsSc 1 ['Sc0.625I0.125Os0.25', '-0.626', '-0.235470645008']
IOsSm 5 ['I0.5Sm0.375Os0.125', '-1.166', '-0.204973814095']
IOsTb 26 ['I0.444444Tb0.444444Os0.111111', '-0.601', '-0.224553339069']
IOsTm 4 ['I0.8Tm0.1Os0.1', '-0.578', '-0.202396760513']
IOsXe 11 ['I0.4Xe0.5Os0.1', '-0.209', '-0.209']
IPPa 19 ['P0.5I0.1Pa0.4', '-1.468', '-0.206981037942']
IPPm 77 ['P0.111111I0.333333Pm0.555556', '-1.076', '-0.214746944862']
IPPr 10 ['P0.375I0.125Pr0.5', '-1.705', '-0.201969477586']
IPPt 16 ['P0.333333I0.444444Pt0.222222', '-0.711', '-0.2083016672']
IPPu 6 ['P0.25I0.375Pu0.375', '-1.455', '-0.20598596685']
IPRe 7 ['P0.2I0.6Re0.2', '-0.483', '-0.210635683941']
IPRh 7 ['P0.111111Rh0.333333I0.555556', '-0.486', '-0.204002862338']
IPRu 13 ['P0.25Ru0.25I0.5', '-0.542', '-0.20097954776']
IPSe 17 ['P0.142857Se0.142857I0.714286', '-0.341', '-0.201141560537']
IPSm 30 ['P0.111111I0.555556Sm0.333333', '-1.443', '-0.210692643346']
IPSn 4 ['P0.714286Sn0.142857I0.142857', '-0.409', '-0.209991481873']
IPSr 2 ['P0.4Sr0.5I0.1', '-1.431', '-0.216021298212']
IPTa 2 ['P0.444444I0.111111Ta0.444444', '-0.841', '-0.202703280927']
IPTb 46 ['P0.2I0.3Tb0.5', '-1.018', '-0.200155641148']
IPTc 1 ['P0.222222Tc0.222222I0.555556', '-0.474', '-0.202957983088']
IPTe 11 ['P0.142857Te0.285714I0.571429', '-0.405', '-0.200511432094']
IPTm 31 ['P0.5I0.25Tm0.25', '-1.199', '-0.210345482257']
IPU 2 ['P0.8I0.1U0.1', '-0.554', '-0.214822445476']
IPW 2 ['P0.111111I0.777778W0.111111', '-0.331', '-0.221024059282']
IPXe 12 ['P0.7I0.1Xe0.2', '-0.244', '-0.205992167059']
IPY 2 ['P0.375Y0.25I0.375', '-1.485', '-0.202809568543']

IPaPb 14 ['I0.6Pb0.2Pa0.2', '-0.737', '-0.213009565043']
IPaPd 34 ['Pd0.3I0.4Pa0.3', '-0.577', '-0.232716278243']
IPaPm 16 ['I0.555556Pm0.333333Pa0.111111', '-0.832', '-0.204766842532']
IPaPr 6 ['I0.666667Pr0.111111Pa0.222222', '-0.865', '-0.207054481009']
IPaPt 64 ['I0.111111Pt0.666667Pa0.222222', '-1.074', '-0.216620797778']
IPaPu 5 ['I0.7Pa0.2Pu0.1', '-0.919', '-0.380355226786']
IPaRb 11 ['Rb0.3I0.6Pa0.1', '-1.261', '-0.225148187045']
IPaRe 14 ['I0.7Re0.2Pa0.1', '-0.386', '-0.207449749633']
IPaRh 36 ['Rh0.428571I0.428571Pa0.142857', '-0.745', '-0.23088607']
IPaRu 29 ['Ru0.333333I0.444444Pa0.222222', '-0.4', '-0.201754398889']
IPaS 54 ['S0.142857I0.714286Pa0.142857', '-0.703', '-0.205148409426']
IPaSb 34 ['Sb0.2I0.4Pa0.4', '-0.537', '-0.215290026945']
IPaSc 10 ['Sc0.166667I0.666667Pa0.166667', '-0.881', '-0.219171264617']
IPaSe 73 ['Se0.4I0.2Pa0.4', '-1.167', '-0.203779716']
IPaSi 16 ['Si0.2I0.5Pa0.3', '-0.623', '-0.257383132286']
IPaSm 7 ['I0.7Sm0.2Pa0.1', '-1.011', '-0.2195414138']
IPaSn 15 ['Sn0.142857I0.571429Pa0.285714', '-0.574', '-0.200795270848']
IPaSr 6 ['Sr0.2I0.7Pa0.1', '-1.325', '-0.201692037285']
IPaTa 4 ['I0.7Ta0.1Pa0.2', '-0.592', '-0.241781578293']
IPaTb 17 ['I0.571429Tb0.142857Pa0.285714', '-0.429', '-0.228754306864']
IPaTc 22 ['Tc0.1I0.5Pa0.4', '-0.369', '-0.254669735772']
IPaTe 64 ['Te0.4I0.5Pa0.1', '-0.492', '-0.204595566452']
IPaTh 2 ['I0.777778Th0.111111Pa0.111111', '-1.168', '-0.374284285597']
IPaTi 5 ['Ti0.142857I0.714286Pa0.142857', '-0.889', '-0.235874375582']
IPaTl 21 ['I0.5Tl0.25Pa0.25', '-0.651', '-0.200517897536']
IPaTm 7 ['I0.7Tm0.2Pa0.1', '-0.989', '-0.210057777783']
IPaU 2 ['I0.777778Pa0.111111U0.111111', '-0.83', '-0.260813778095']
IPaV 10 ['V0.1I0.6Pa0.3', '-0.457', '-0.203373069786']
IPaW 8 ['I0.6W0.1Pa0.3', '-0.328', '-0.244792770272']
IPaXe 37 ['I0.333333Xe0.555556Pa0.111111', '-0.236', '-0.205182507508']
IPaY 3 ['Y0.125I0.75Pa0.125', '-1.166', '-0.349494651286']
IPaYb 6 ['I0.7Yb0.2Pa0.1', '-1.363', '-0.268259211785']
IPaZn 12 ['Zn0.2I0.6Pa0.2', '-0.622', '-0.211120858543']
IPaZr 5 ['Zr0.142857I0.714286Pa0.142857', '-0.887', '-0.225032868967']
IPbPm 33 ['I0.444444Pm0.444444Pb0.111111', '-1.027', '-0.205822256262']
IPbPr 3 ['I0.4Pr0.4Pb0.2', '-1.284', '-0.202440872296']
IPbRh 2 ['Rh0.3I0.6Pb0.1', '-0.435', '-0.200740525764']
IPbSm 22 ['I0.6Sm0.2Pb0.2', '-1.179', '-0.20434945779']
IPbTb 27 ['I0.428571Tb0.428571Pb0.142857', '-0.657', '-0.201342895793']
IPbTm 12 ['I0.3Tm0.4Pb0.3', '-0.971', '-0.203881812769']
IPbY 3 ['Y0.5I0.2Pb0.3', '-1.052', '-0.204037229781']
IPbZr 3 ['Zr0.166667I0.666667Pb0.166667', '-1.142', '-0.220715024004']
IPdPm 71 ['Pd0.444444I0.111111Pm0.444444', '-0.943', '-0.202579001489']
IPdPr 18 ['Pd0.3I0.6Pr0.1', '-0.936', '-0.213580915106']

IPdSc 3 ['Sc0.222222Pd0.222222I0.555556', '-1.113', '-0.200509688128']
IPdSe 13 ['Se0.333333Pd0.166667I0.5', '-0.38', '-0.203105138774']
IPdSi 4 ['Si0.125Pd0.25I0.625', '-0.681', '-0.218369039654']
IPdSm 29 ['Pd0.444444I0.444444Sm0.111111', '-0.798', '-0.213477482671']
IPdSn 1 ['Pd0.166667Sn0.166667I0.666667', '-0.713', '-0.218109784601']
IPdTa 5 ['Pd0.142857I0.714286Ta0.142857', '-0.718', '-0.205194925122']
IPdTb 46 ['Pd0.285714I0.285714Tb0.428571', '-0.744', '-0.212994950878']
IPdTi 1 ['Ti0.166667Pd0.166667I0.666667', '-0.939', '-0.20903126775']
IPdTm 19 ['Pd0.1I0.6Tm0.3', '-1.361', '-0.208956020727']
IPdU 4 ['Pd0.166667I0.666667U0.166667', '-1.019', '-0.211446905881']
IPdXe 8 ['Pd0.111111I0.444444Xe0.444444', '-0.318', '-0.200070092516']
IPdY 1 ['Y0.375Pd0.375I0.25', '-1.359', '-0.237929832518']
IPdZr 11 ['Zr0.2Pd0.2I0.6', '-1.101', '-0.207190779985']
IPmPr 6 ['I0.666667Pr0.111111Pm0.222222', '-1.294', '-0.300079022675']
IPmPt 66 ['I0.222222Pm0.555556Pt0.222222', '-1.082', '-0.204793487238']
IPmPu 6 ['I0.666667Pm0.222222Pu0.111111', '-1.248', '-0.313530349207']
IPmRb 16 ['Rb0.333333I0.555556Pm0.111111', '-1.58', '-0.287616419764']
IPmRe 16 ['I0.555556Pm0.222222Re0.222222', '-0.719', '-0.237603719392']
IPmRh 59 ['Rh0.5I0.125Pm0.375', '-0.765', '-0.200472292509']
IPmRu 31 ['Ru0.2I0.4Pm0.4', '-0.939', '-0.223301202029']
IPmS 51 ['S0.571429I0.142857Pm0.285714', '-1.779', '-0.206983967564']
IPmSb 65 ['Sb0.142857I0.285714Pm0.571429', '-0.982', '-0.205726653235']
IPmSc 11 ['Sc0.1I0.6Pm0.3', '-1.14', '-0.233864403535']
IPmSe 76 ['Se0.2I0.3Pm0.5', '-1.39', '-0.204833821022']
IPmSi 46 ['Si0.285714I0.142857Pm0.571429', '-0.716', '-0.20974207001']
IPmSm 8 ['I0.666667Pm0.166667Sm0.166667', '-1.194', '-0.259356465048']
IPmSn 37 ['Sn0.3I0.4Pm0.3', '-0.908', '-0.201681419112']
IPmSr 9 ['Sr0.222222I0.666667Pm0.111111', '-1.645', '-0.228892312261']
IPmTa 6 ['I0.714286Pm0.142857Ta0.142857', '-0.898', '-0.221326236122']
IPmTb 17 ['I0.555556Pm0.111111Tb0.333333', '-0.705', '-0.223859782643']
IPmTc 23 ['Tc0.285714I0.571429Pm0.142857', '-0.517', '-0.261393286439']
IPmTe 78 ['Te0.166667I0.333333Pm0.5', '-1.263', '-0.231565235717']
IPmTh 4 ['I0.7Pm0.2Th0.1', '-1.387', '-0.342542201294']
IPmTi 11 ['Ti0.2I0.7Pm0.1', '-1.13', '-0.210977519293']
IPmTl 40 ['I0.428571Pm0.142857Tl0.428571', '-0.938', '-0.209599818602']
IPmTm 8 ['I0.666667Pm0.166667Tm0.166667', '-1.247', '-0.322786768367']
IPmU 4 ['I0.7Pm0.2U0.1', '-1.138', '-0.295618744543']
IPmV 11 ['V0.222222I0.666667Pm0.111111', '-0.799', '-0.221484332817']
IPmW 14 ['I0.666667Pm0.111111W0.222222', '-0.414', '-0.215194778341']
IPmXe 32 ['I0.4Xe0.4Pm0.2', '-0.582', '-0.224150601014']
IPmY 5 ['Y0.142857I0.714286Pm0.142857', '-1.424', '-0.274866806827']
IPmYb 8 ['I0.666667Pm0.166667Yb0.166667', '-1.409', '-0.221621296702']
IPmZn 27 ['Zn0.333333I0.444444Pm0.222222', '-0.846', '-0.213969203921']
IPmZr 6 ['Zr0.111111I0.666667Pm0.222222', '-1.13', '-0.248343725038']

IPrPt 39 ['I0.333333Pr0.222222Pt0.444444', '-1.292', '-0.200501069636']
IPrPu 3 ['I0.75Pr0.125Pu0.125', '-1.485', '-0.210184040832']
IPrRb 3 ['Rb0.166667I0.666667Pr0.166667', '-1.686', '-0.238107367758']
IPrRe 1 ['I0.8Pr0.1Re0.1', '-0.89', '-0.277913265832']
IPrRh 22 ['Rh0.1I0.6Pr0.3', '-1.595', '-0.205161916094']
IPrS 1 ['S0.375I0.375Pr0.25', '-1.64', '-0.215639103582']
IPrSb 32 ['Sb0.111111I0.777778Pr0.111111', '-1.018', '-0.205636267961']
IPrSc 2 ['Sc0.125I0.75Pr0.125', '-1.376', '-0.243448776758']
IPrSe 20 ['Se0.375I0.5Pr0.125', '-0.88', '-0.209150649242']
IPrSi 2 ['Si0.125I0.5Pr0.375', '-1.491', '-0.2263989401']
IPrSm 2 ['I0.777778Pr0.111111Sm0.111111', '-1.259', '-0.238398997683']
IPrTb 7 ['I0.666667Pr0.166667Tb0.166667', '-1.241', '-0.20536670114']
IPrTe 43 ['Te0.2I0.4Pr0.4', '-1.639', '-0.200433377858']
IPrTl 19 ['I0.5Pr0.375Tl0.125', '-1.378', '-0.200224423974']
IPrTm 3 ['I0.8Pr0.1Tm0.1', '-1.129', '-0.216717279907']
IPrV 1 ['V0.111111I0.777778Pr0.111111', '-0.989', '-0.203334243231']
IPrW 1 ['I0.8Pr0.1W0.1', '-0.79', '-0.253320519394']
IPrXe 4 ['I0.5Xe0.4Pr0.1', '-0.742', '-0.205320519394']
IPrZn 1 ['Zn0.166667I0.666667Pr0.166667', '-1.318', '-0.201404414632']
IPtPu 21 ['I0.444444Pt0.333333Pu0.222222', '-1.151', '-0.210691284415']
IPtRb 1 ['Rb0.222222I0.555556Pt0.222222', '-1.057', '-0.207925295318']
IPtRe 14 ['I0.555556Re0.111111Pt0.333333', '-0.44', '-0.218425043095']
IPtRu 1 ['Ru0.166667I0.666667Pt0.166667', '-0.385', '-0.204985783786']
IPtSb 20 ['Sb0.25I0.625Pt0.125', '-0.648', '-0.200568862076']
IPtSc 34 ['Sc0.375I0.5Pt0.125', '-1.442', '-0.211009278157']
IPtSi 16 ['Si0.25I0.625Pt0.125', '-0.773', '-0.222167290748']
IPtSm 54 ['I0.125Sm0.5Pt0.375', '-1.339', '-0.209290106258']
IPtSn 10 ['Sn0.2I0.7Pt0.1', '-0.722', '-0.220832115955']
IPtSr 6 ['Sr0.111111I0.666667Pt0.222222', '-0.986', '-0.200088801436']
IPtTa 10 ['I0.5Ta0.166667Pt0.333333', '-0.763', '-0.20857938792']
IPtTb 53 ['I0.444444Tb0.111111Pt0.444444', '-0.71', '-0.203567419884']
IPtTc 5 ['Tc0.111111I0.666667Pt0.222222', '-0.447', '-0.206981045048']
IPtTe 18 ['Te0.1I0.7Pt0.2', '-0.498', '-0.222478933819']
IPtTh 42 ['I0.3Pt0.4Th0.3', '-1.266', '-0.201445376835']
IPtTi 25 ['Ti0.3I0.4Pt0.3', '-1.137', '-0.204157107285']
IPtTl 4 ['I0.625Pt0.25Tl0.125', '-0.593', '-0.203011166607']
IPtTm 36 ['I0.8Tm0.1Pt0.1', '-0.7', '-0.216388230785']
IPtU 22 ['I0.444444Pt0.333333U0.222222', '-1.081', '-0.210779228365']
IPtV 18 ['V0.2I0.7Pt0.1', '-0.658', '-0.2091520693']
IPtW 13 ['I0.666667W0.222222Pt0.111111', '-0.321', '-0.200990522524']
IPtXe 27 ['I0.7Xe0.1Pt0.2', '-0.417', '-0.200982940543']
IPtY 42 ['Y0.3I0.6Pt0.1', '-1.704', '-0.200989614294']
IPtYb 7 ['I0.5Yb0.333333Pt0.166667', '-1.811', '-0.203855399618']
IPtZn 1 ['Zn0.125I0.625Pt0.25', '-0.619', '-0.242244367388']

IPtZr 27 ['Zr0.333333I0.555556Pt0.111111', '-1.274', '-0.213910722538']
IPuRb 4 ['Rb0.142857I0.714286Pu0.142857', '-1.397', '-0.215318344338']
IPuRe 2 ['I0.777778Re0.111111Pu0.111111', '-0.858', '-0.237354955233']
IPuRh 9 ['Rh0.333333I0.555556Pu0.111111', '-0.761', '-0.224140792524']
IPuS 22 ['S0.166667I0.5Pu0.333333', '-1.649', '-0.204118739783']
IPuSb 1 ['Sb0.1I0.8Pu0.1', '-0.878', '-0.200378835044']
IPuSc 9 ['Sc0.25I0.625Pu0.125', '-1.349', '-0.224274052229']
IPuSe 10 ['Se0.4I0.2Pu0.4', '-1.607', '-0.202187530847']
IPuSi 2 ['Si0.125I0.75Pu0.125', '-1.054', '-0.201341677555']
IPuSm 6 ['I0.7Sm0.1Pu0.2', '-1.392', '-0.2165156548']
IPuSr 1 ['Sr0.142857I0.714286Pu0.142857', '-1.749', '-0.276200862194']
IPuTb 9 ['I0.625Tb0.25Pu0.125', '-1.042', '-0.226282144816']
IPuTe 4 ['Te0.444444I0.111111Pu0.444444', '-1.368', '-0.268049122691']
IPuTh 1 ['I0.75Th0.125Pu0.125', '-1.545', '-0.284095094743']
IPuTi 5 ['Ti0.125I0.75Pu0.125', '-1.275', '-0.208472278331']
IPuTl 34 ['I0.7Tl0.2Pu0.1', '-1.024', '-0.202477326301']
IPuTm 3 ['I0.8Tm0.1Pu0.1', '-1.227', '-0.368223473785']
IPuV 1 ['V0.1I0.8Pu0.1', '-0.869', '-0.215407012786']
IPuW 2 ['I0.777778W0.111111Pu0.111111', '-0.746', '-0.209140792524']
IPuXe 6 ['I0.75Xe0.125Pu0.125', '-0.816', '-0.21203339159']
IPuY 2 ['Y0.142857I0.714286Pu0.142857', '-1.564', '-0.21030848541']
IPuYb 2 ['I0.777778Yb0.111111Pu0.111111', '-1.389', '-0.259360211984']
IPuZn 1 ['Zn0.125I0.625Pu0.25', '-1.243', '-0.208458563003']
IPuZr 8 ['Zr0.2I0.6Pu0.2', '-1.175', '-0.208653426544']
IRbRe 2 ['Rb0.111111I0.777778Re0.111111', '-0.729', '-0.236437030233']
IRbRh 3 ['Rb0.1Rh0.2I0.7', '-0.575', '-0.207100580772']
IRbSb 1 ['Rb0.6Sb0.3I0.1', '-0.861', '-0.221589948008']
IRbSc 3 ['Sc0.166667Rb0.166667I0.666667', '-1.421', '-0.232057716296']
IRbSi 3 ['Si0.142857Rb0.142857I0.714286', '-1.028', '-0.205678286124']
IRbSm 10 ['Rb0.2I0.7Sm0.1', '-1.296', '-0.213901174549']
IRbTb 14 ['Rb0.2I0.6Tb0.2', '-1.088', '-0.230591258111']
IRbTe 12 ['Rb0.5Te0.333333I0.166667', '-1.3', '-0.202043618485']
IRbTh 1 ['Rb0.125I0.75Th0.125', '-1.522', '-0.233734660056']
IRbTi 1 ['Ti0.222222Rb0.111111I0.666667', '-1.248', '-0.207048359718']
IRbTm 7 ['Rb0.111111I0.777778Tm0.111111', '-1.06', '-0.233885934761']
IRbU 3 ['Rb0.142857I0.714286U0.142857', '-1.35', '-0.200514026124']
IRbW 2 ['Rb0.1I0.8W0.1', '-0.599', '-0.231100580772']
IReRh 10 ['Rh0.111111I0.666667Re0.222222', '-0.391', '-0.223428325418']
IReRu 2 ['Ru0.142857I0.714286Re0.142857', '-0.31', '-0.202275352054']
IReSc 1 ['Sc0.1I0.8Re0.1', '-0.65', '-0.205231248451']
IReSe 22 ['Se0.4I0.4Re0.2', '-0.437', '-0.2005358385']
IReSm 5 ['I0.714286Sm0.142857Re0.142857', '-0.847', '-0.210424589216']
IReTb 18 ['I0.5Tb0.375Re0.125', '-0.551', '-0.201953440856']
IReTe 4 ['Te0.25I0.5Re0.25', '-0.364', '-0.204399965765']

IReTm 3 ['I0.75Tm0.125Re0.125', '-0.807', '-0.243236883689']
IReXe 10 ['I0.75Xe0.125Re0.125', '-0.304', '-0.209740933047']
IRhRu 3 ['Ru0.111111Rh0.222222I0.666667', '-0.229', '-0.229']
IRhSb 11 ['Rh0.333333Sb0.111111I0.555556', '-0.417', '-0.200946801968']
IRhSc 27 ['Sc0.333333Rh0.222222I0.444444', '-1.281', '-0.209257563918']
IRhSe 19 ['Se0.5Rh0.375I0.125', '-0.507', '-0.200751830625']
IRhSi 12 ['Si0.111111Rh0.333333I0.555556', '-0.486', '-0.202092361698']
IRhSm 47 ['Rh0.4I0.2Sm0.4', '-1.072', '-0.207301384263']
IRhSn 2 ['Rh0.222222Sn0.111111I0.666667', '-0.505', '-0.214729655104']
IRhSr 2 ['Sr0.125Rh0.25I0.625', '-0.927', '-0.24226736283']
IRhTa 11 ['Rh0.285714I0.571429Ta0.142857', '-0.628', '-0.206932949684']
IRhTb 58 ['Rh0.5I0.166667Tb0.333333', '-0.665', '-0.202114938659']
IRhTc 7 ['Tc0.1Rh0.3I0.6', '-0.22', '-0.2137966105']
IRhTe 6 ['Rh0.333333Te0.111111I0.555556', '-0.389', '-0.250784767301']
IRhTh 5 ['Rh0.3I0.6Th0.1', '-0.926', '-0.23939160028']
IRhTi 5 ['Ti0.166667Rh0.166667I0.666667', '-0.921', '-0.205246343583']
IRhTl 6 ['Rh0.125I0.625Tl0.25', '-0.673', '-0.229166271608']
IRhTm 35 ['Rh0.4I0.2Tm0.4', '-1.194', '-0.200439377513']
IRhU 17 ['Rh0.222222I0.555556U0.222222', '-1.083', '-0.201811444762']
IRhV 8 ['V0.111111Rh0.333333I0.555556', '-0.406', '-0.216644777238']
IRhW 11 ['Rh0.111111I0.666667W0.222222', '-0.244', '-0.202576109074']
IRhXe 33 ['Rh0.222222I0.333333Xe0.444444', '-0.203', '-0.203']
IRhY 25 ['Y0.444444Rh0.333333I0.222222', '-1.226', '-0.200979125201']
IRhYb 5 ['Rh0.166667I0.666667Yb0.166667', '-1.105', '-0.21582912919']
IRhZn 5 ['Zn0.142857Rh0.285714I0.571429', '-0.454', '-0.200137389306']
IRhZr 20 ['Zr0.111111Rh0.111111I0.777778', '-0.692', '-0.207954168356']
IRuSc 3 ['Sc0.222222Ru0.111111I0.666667', '-1.027', '-0.20619667114']
IRuSm 23 ['Ru0.2I0.4Sm0.4', '-1.018', '-0.218466736526']
IRuTa 3 ['Ru0.2I0.7Ta0.1', '-0.501', '-0.206253064779']
IRuTb 32 ['Ru0.333333I0.444444Tb0.222222', '-0.396', '-0.207776669535']
IRuTc 1 ['Tc0.166667Ru0.166667I0.666667', '-0.207', '-0.207']
IRuTm 15 ['Ru0.222222I0.555556Tm0.222222', '-1.048', '-0.213326134473']
IRuV 5 ['V0.222222Ru0.111111I0.666667', '-0.586', '-0.207289554476']
IRuW 2 ['Ru0.166667I0.666667W0.166667', '-0.216', '-0.209795441111']
IRuXe 8 ['Ru0.125I0.5Xe0.375', '-0.205', '-0.205']
IRuZr 2 ['Zr0.2Ru0.1I0.7', '-1.104', '-0.23271750304']
ISSc 3 ['S0.142857Sc0.285714I0.571429', '-1.411', '-0.20942620088']
ISSe 27 ['S0.166667Se0.166667I0.666667', '-0.201', '-0.201']
ISSm 1 ['S0.142857I0.571429Sm0.285714', '-1.634', '-0.245812717324']
ISTb 42 ['S0.166667I0.666667Tb0.166667', '-0.813', '-0.204460623553']
ISTc 23 ['S0.142857Tc0.285714I0.571429', '-0.376', '-0.203046625141']
ISTe 9 ['S0.285714Te0.285714I0.428571', '-0.34', '-0.200852600685']
ISTm 29 ['S0.4I0.1Tm0.5', '-2.061', '-0.216246038648']
ISU 1 ['S0.333333I0.444444U0.222222', '-1.326', '-0.204132014249']

ISV 1 ['S0.222222V0.222222I0.555556', '-0.763', '-0.213797719948']
ISXe 49 ['S0.2I0.2Xe0.6', '-0.207', '-0.207']
ISY 1 ['S0.285714Y0.285714I0.428571', '-1.911', '-0.213421343456']
ISZr 1 ['S0.428571Zr0.428571I0.142857', '-1.701', '-0.257365630906']
ISbSc 7 ['Sc0.5Sb0.25I0.25', '-1.192', '-0.200142216891']
ISbSe 3 ['Se0.25Sb0.125I0.625', '-0.451', '-0.207940152214']
ISbSm 47 ['Sb0.1I0.4Sm0.5', '-1.203', '-0.203996038276']
ISbSr 9 ['Sr0.555556Sb0.333333I0.111111', '-1.357', '-0.223229165784']
ISbTb 40 ['Sb0.428571I0.142857Tb0.428571', '-0.969', '-0.223253247296']
ISbTm 30 ['Sb0.25I0.625Tm0.125', '-0.917', '-0.204436102856']
ISbXe 3 ['Sb0.1I0.5Xe0.4', '-0.397', '-0.202552121772']
ISbY 1 ['Y0.375Sb0.25I0.375', '-1.571', '-0.20514337784']
ISbYb 20 ['Sb0.25I0.5Yb0.25', '-1.541', '-0.207243693785']
IScSe 52 ['Sc0.142857Se0.142857I0.714286', '-0.733', '-0.205340717161']
IScSi 1 ['Si0.111111Sc0.111111I0.777778', '-0.915', '-0.220690697268']
IScSm 5 ['Sc0.1I0.7Sm0.2', '-1.337', '-0.215581546548']
IScTb 11 ['Sc0.222222I0.666667Tb0.111111', '-1.14', '-0.225085005907']
IScTe 1 ['Sc0.285714Te0.142857I0.571429', '-1.335', '-0.277782517215']
IScTl 24 ['Sc0.166667I0.5Tl0.333333', '-1.07', '-0.20030234045']
IScTm 4 ['Sc0.1I0.8Tm0.1', '-0.953', '-0.208035262526']
IScV 1 ['Sc0.125V0.125I0.75', '-0.895', '-0.220273501909']
IScW 3 ['Sc0.125I0.75W0.125', '-0.667', '-0.205298127516']
IScXe 15 ['Sc0.25I0.625Xe0.125', '-1.127', '-0.203596255032']
IScZr 1 ['Sc0.111111Zr0.111111I0.777778', '-1.113', '-0.218552503926']
ISeSi 6 ['Si0.2Se0.2I0.6', '-0.654', '-0.202803754043']
ISeSm 24 ['Se0.111111I0.555556Sm0.333333', '-1.518', '-0.202430425407']
ISeSr 2 ['Se0.3Sr0.5I0.2', '-2.003', '-0.211662833264']
ISeTa 4 ['Se0.285714I0.571429Ta0.142857', '-0.631', '-0.205179055648']
ISeTb 63 ['Se0.2I0.3Tb0.5', '-1.038', '-0.20584561894']
ISeTc 49 ['Se0.2Tc0.3I0.5', '-0.213', '-0.213']
ISeTe 1 ['Se0.222222Te0.111111I0.666667', '-0.36', '-0.221784767301']
ISeTm 45 ['Se0.444444I0.444444Tm0.111111', '-0.709', '-0.204843305557']
ISeU 19 ['Se0.4I0.4U0.2', '-1.061', '-0.205837312029']
ISeV 8 ['V0.111111Se0.222222I0.666667', '-0.434', '-0.206967751111']
ISeW 5 ['Se0.166667I0.666667W0.166667', '-0.345', '-0.20624185375']
ISeXe 45 ['Se0.375I0.125Xe0.5', '-0.205', '-0.205']
ISeY 16 ['Se0.428571Y0.285714I0.285714', '-1.498', '-0.229591403116']
ISeYb 3 ['Se0.222222I0.333333Yb0.444444', '-2.028', '-0.202758295857']
ISeZn 3 ['Zn0.111111Se0.333333I0.555556', '-0.4', '-0.202551302794']
ISeZr 42 ['Se0.1Zr0.3I0.6', '-1.299', '-0.200780219012']
ISiSm 20 ['Si0.2I0.7Sm0.1', '-0.902', '-0.200215513966']
ISiTb 33 ['Si0.285714I0.285714Tb0.428571', '-0.695', '-0.219150661735']
ISiTl 1 ['Si0.125I0.625Tl0.25', '-0.858', '-0.228165541607']
ISiTm 17 ['Si0.142857I0.571429Tm0.285714', '-1.285', '-0.21184788718']

ISiXe 10 ['Si0.166667I0.5Xe0.333333', '-0.54', '-0.208411047953']
ISmSn 12 ['Sn0.1I0.6Sm0.3', '-1.321', '-0.200812402458']
ISmSr 1 ['Sr0.111111I0.777778Sm0.111111', '-1.236', '-0.203058298651']
ISmTa 2 ['I0.777778Sm0.111111Ta0.111111', '-0.969', '-0.217212937']
ISmTb 9 ['I0.666667Sm0.222222Tb0.111111', '-1.173', '-0.243269495361']
ISmTc 3 ['Tc0.1I0.7Sm0.2', '-0.97', '-0.206277157043']
ISmTe 62 ['Te0.555556I0.333333Sm0.111111', '-0.741', '-0.200516298589']
ISmTh 1 ['I0.8Sm0.1Th0.1', '-1.339', '-0.270530178802']
ISmTi 3 ['Ti0.125I0.75Sm0.125', '-1.225', '-0.225440340204']
ISmTl 32 ['I0.4Sm0.3Tl0.3', '-1.054', '-0.202617072776']
ISmTm 5 ['I0.7Sm0.2Tm0.1', '-1.344', '-0.216339805048']
ISmU 2 ['I0.777778Sm0.111111U0.111111', '-1.185', '-0.222340802278']
ISmV 5 ['V0.142857I0.714286Sm0.142857', '-0.994', '-0.205026968623']
ISmW 3 ['I0.75Sm0.125W0.125', '-0.742', '-0.264673223152']
ISmXe 18 ['I0.5Xe0.333333Sm0.166667', '-0.851', '-0.214564297536']
ISmY 1 ['Y0.111111I0.777778Sm0.111111', '-1.353', '-0.233744491993']
ISmYb 1 ['I0.714286Sm0.142857Yb0.142857', '-1.553', '-0.245337222908']
ISmZn 13 ['Zn0.285714I0.571429Sm0.142857', '-1.026', '-0.235942900566']
ISmZr 3 ['Zr0.125I0.75Sm0.125', '-1.258', '-0.236121662552']
ISnTb 37 ['Sn0.2I0.3Tb0.5', '-0.621', '-0.207128788811']
ISnTm 7 ['Sn0.1I0.6Tm0.3', '-1.328', '-0.201190281539']
ISnXe 1 ['Sn0.1I0.6Xe0.3', '-0.474', '-0.212756689593']
ISrTb 9 ['Sr0.222222I0.666667Tb0.111111', '-1.546', '-0.234585868687']
ISrTe 3 ['Sr0.333333Te0.444444I0.222222', '-1.624', '-0.239035881773']
ISrTm 1 ['Sr0.2I0.7Tm0.1', '-1.679', '-0.207824541041']
ITaTb 7 ['I0.666667Tb0.166667Ta0.166667', '-0.751', '-0.241398833124']
ITaTm 2 ['I0.777778Tm0.111111Ta0.111111', '-0.963', '-0.218166472546']
ITaXe 3 ['I0.6Xe0.3Ta0.1', '-0.507', '-0.212253064779']
ITbTc 23 ['Tc0.1I0.5Tb0.4', '-0.56', '-0.221198005162']
ITbTe 61 ['Te0.3I0.6Tb0.1', '-0.63', '-0.202316861389']
ITbTh 4 ['I0.7Tb0.2Th0.1', '-1.095', '-0.238990602861']
ITbTi 8 ['Ti0.125I0.625Tb0.25', '-0.895', '-0.220687639967']
ITbTl 34 ['I0.444444Tb0.444444Tl0.111111', '-0.655', '-0.203268229032']
ITbTm 11 ['I0.625Tb0.25Tm0.125', '-0.927', '-0.245744703868']
ITbU 4 ['I0.7Tb0.2U0.1', '-0.967', '-0.31306714611']
ITbV 13 ['V0.2I0.6Tb0.2', '-0.717', '-0.20675960161']
ITbW 14 ['I0.6Tb0.2W0.2', '-0.384', '-0.214599002581']
ITbXe 39 ['I0.375Xe0.375Tb0.25', '-0.415', '-0.203248753227']
ITbY 5 ['Y0.142857I0.714286Tb0.142857', '-1.312', '-0.297472807947']
ITbYb 7 ['I0.666667Tb0.166667Yb0.166667', '-1.236', '-0.205661631341']
ITbZn 28 ['Zn0.333333I0.555556Tb0.111111', '-0.739', '-0.223782718609']
ITbZr 7 ['Zr0.2I0.7Tb0.1', '-1.181', '-0.225017004331']
ITcTe 18 ['Tc0.375Te0.25I0.375', '-0.331', '-0.2092460256']
ITcTm 5 ['Tc0.111111I0.666667Tm0.222222', '-1.043', '-0.208326134473']

ITcXe 13 ['Tc0.2I0.5Xe0.3', '-0.201', '-0.201']
ITeTh 36 ['Te0.111111I0.666667Th0.222222', '-1.54', '-0.205115952857']
ITeTm 40 ['Te0.428571I0.428571Tm0.142857', '-0.797', '-0.202289870389']
ITeU 2 ['Te0.2I0.6U0.2', '-1.054', '-0.222520883793']
ITeXe 6 ['Te0.142857I0.571429Xe0.285714', '-0.392', '-0.214294700815']
ITeY 25 ['Y0.428571Te0.285714I0.285714', '-1.55', '-0.20619458359']
ITeYb 5 ['Te0.111111I0.666667Yb0.222222', '-1.463', '-0.206505490371']
ITeZr 12 ['Zr0.25Te0.375I0.375', '-1.126', '-0.2337940819']
IThTl 10 ['I0.571429Tl0.142857Th0.285714', '-1.237', '-0.205640244208']
IThTm 2 ['I0.777778Tm0.111111Th0.111111', '-1.46', '-0.279764845326']
ITiTm 2 ['Ti0.1I0.8Tm0.1', '-1.017', '-0.211944566663']
ITlTm 14 ['I0.7Tm0.1Tl0.2', '-0.948', '-0.217329777799']
ITlV 2 ['V0.125I0.625Tl0.25', '-0.82', '-0.204935635357']
ITlW 1 ['I0.75W0.125Tl0.125', '-0.436', '-0.203634638465']
ITmU 2 ['I0.777778Tm0.111111U0.111111', '-1.235', '-0.279294337824']
ITmV 3 ['V0.1I0.8Tm0.1', '-0.851', '-0.304977060027']
ITmW 3 ['I0.75Tm0.125W0.125', '-0.804', '-0.334495950641']
ITmXe 15 ['I0.444444Xe0.444444Tm0.111111', '-0.622', '-0.204663067237']
ITmYb 1 ['I0.714286Tm0.142857Yb0.142857', '-1.52', '-0.221277482896']
ITmZn 9 ['Zn0.125I0.75Tm0.125', '-0.897', '-0.205366166284']
ITmZr 3 ['Zr0.1I0.8Tm0.1', '-1.055', '-0.243755512033']
IVXe 8 ['V0.111111I0.444444Xe0.444444', '-0.398', '-0.208644777238']
IWXe 9 ['I0.666667Xe0.166667W0.166667', '-0.224', '-0.224']
IWZr 1 ['Zr0.1I0.8W0.1', '-0.64', '-0.20435875152']
IXeZn 3 ['Zn0.111111I0.444444Xe0.444444', '-0.419', '-0.221551302794']
IXeZr 4 ['Zr0.142857I0.714286Xe0.142857', '-0.832', '-0.209655359314']
InIrLa 1 ['In0.5La0.166667Ir0.333333', '-0.662', '-0.205054670179']
InIrN 16 ['N0.285714In0.428571Ir0.285714', '-0.313', '-0.201335444821']
InIrNa 5 ['Na0.125In0.625Ir0.25', '-0.391', '-0.208773268643']
InIrNd 2 ['In0.5Nd0.166667Ir0.333333', '-0.636', '-0.210257016167']
InIrP 1 ['P0.8In0.1Ir0.1', '-0.646', '-0.213200813874']
InIrSe 6 ['Se0.222222In0.555556Ir0.222222', '-0.525', '-0.204028625416']
InIrSr 4 ['Sr0.1In0.5Ir0.4', '-0.458', '-0.211909625875']
InKN 11 ['N0.375K0.25In0.375', '-0.434', '-0.211708944365']
InKO 5 ['O0.625K0.125In0.25', '-1.773', '-0.214819298041']
InKSe 2 ['K0.428571Se0.285714In0.285714', '-1.013', '-0.202544146786']
InLaN 39 ['N0.2In0.3La0.5', '-1.141', '-0.229333971289']
InLaO 4 ['O0.6In0.3La0.1', '-2.529', '-0.217828291095']
InLaS 1 ['S0.375In0.25La0.375', '-1.955', '-0.222580068493']
InLaSe 9 ['Se0.285714In0.285714La0.428571', '-1.576', '-0.205730455179']
InLiN 21 ['Li0.375N0.25In0.375', '-0.467', '-0.203410419081']
InLiS 1 ['Li0.3S0.4In0.3', '-1.221', '-0.244989676395']
InLuN 19 ['N0.428571In0.428571Lu0.142857', '-0.784', '-0.206685365568']
InMgPt 1 ['Mg0.5In0.1Pt0.4', '-1.027', '-0.277037684917']

InMnN 12 ['N0.444444Mn0.444444In0.111111', '-0.471', '-0.201456871495']
InMnO 1 ['O0.6Mn0.2In0.2', '-2.147', '-0.206351390606']
InMoN 18 ['N0.571429Mo0.142857In0.285714', '-0.346', '-0.20088603521']
InMoO 4 ['O0.625Mo0.25In0.125', '-2.27', '-0.269732781692']
InMoP 26 ['P0.5Mo0.125In0.375', '-0.664', '-0.20544484052']
InNNa 13 ['N0.333333Na0.5In0.166667', '-0.423', '-0.200123372771']
InNNb 5 ['N0.5Nb0.1In0.4', '-0.464', '-0.201455935327']
InNNd 10 ['N0.444444In0.444444Nd0.111111', '-0.543', '-0.205340009292']
InNNp 34 ['N0.333333In0.166667Np0.5', '-1.016', '-0.206715898703']
InNO 12 ['N0.222222O0.666667In0.111111', '-1.05', '-0.200037187928']
InNOs 1 ['N0.5In0.375Os0.125', '-0.22', '-0.206107507996']
InNPa 41 ['N0.333333In0.5Pa0.166667', '-0.554', '-0.216452681829']
InNPm 18 ['N0.428571In0.142857Pm0.428571', '-1.522', '-0.200679923783']
InNPr 14 ['N0.428571In0.285714Pr0.285714', '-1.007', '-0.205680635568']
InNPt 15 ['N0.444444In0.444444Pt0.111111', '-0.373', '-0.201179505479']
InNPu 20 ['N0.4In0.2Pu0.4', '-1.302', '-0.206702385864']
InNRb 1 ['N0.5Rb0.166667In0.333333', '-0.413', '-0.218680250661']
InNRe 7 ['N0.5In0.2Re0.3', '-0.254', '-0.211723503897']
InNRh 14 ['N0.4Rh0.4In0.2', '-0.384', '-0.210806116']
InNSc 11 ['N0.5Sc0.1In0.4', '-0.675', '-0.256062474829']
InNSm 9 ['N0.444444In0.444444Sm0.111111', '-0.568', '-0.203102220125']
InNSr 14 ['N0.571429Sr0.142857In0.285714', '-0.52', '-0.205777152304']
InNTb 26 ['N0.555556In0.222222Tb0.222222', '-0.858', '-0.211426918808']
InNTc 10 ['N0.375Tc0.375In0.25', '-0.31', '-0.204927390496']
InNTh 17 ['N0.5In0.1Th0.4', '-1.76', '-0.209462126078']
InNTi 23 ['N0.555556Ti0.222222In0.222222', '-0.796', '-0.211306602364']
InNTm 12 ['N0.4In0.3Tm0.3', '-1.364', '-0.202698011864']
InNU 19 ['N0.6In0.1U0.3', '-1.547', '-0.201327618293']
InNV 4 ['N0.5V0.166667In0.333333', '-0.636', '-0.206015827329']
InNW 6 ['N0.5In0.25W0.25', '-0.301', '-0.202819897328']
InNY 35 ['N0.5Y0.4In0.1', '-1.653', '-0.21311713933']
InNYb 23 ['N0.333333In0.333333Yb0.333333', '-0.789', '-0.203002171551']
InNZr 8 ['N0.5Zr0.1In0.4', '-0.634', '-0.248062782745']
InNaPm 7 ['Na0.111111In0.666667Pm0.222222', '-0.483', '-0.211412597683']
InNaPt 4 ['Na0.1In0.6Pt0.3', '-0.663', '-0.214999566915']
InNaRh 4 ['Na0.125Rh0.25In0.625', '-0.546', '-0.230085299581']
InNbO 4 ['O0.625Nb0.125In0.25', '-2.422', '-0.202416617317']
InNbP 2 ['P0.571429Nb0.142857In0.285714', '-0.741', '-0.200595582737']
InNdO 2 ['O0.6In0.3Nd0.1', '-2.498', '-0.209855898658']
InNdS 2 ['S0.444444In0.222222Nd0.333333', '-1.936', '-0.20619248803']
InNiP 1 ['P0.8Ni0.1In0.1', '-0.496', '-0.202061683124']
InNiTi 2 ['Ti0.25Ni0.5In0.25', '-0.605', '-0.221000790303']
InNpO 1 ['O0.4In0.1Np0.5', '-2.527', '-0.205869315495']
InNpP 9 ['P0.5In0.166667Np0.333333', '-0.977', '-0.201119217603']

InNpSe 2 ['Se0.4In0.2Np0.4', '-1.051', '-0.200903732496']
InOOs 2 ['O0.625In0.25Os0.125', '-1.825', '-0.236933761166']
InOPa 8 ['O0.6In0.3Pa0.1', '-2.454', '-0.291199891345']
InORb 6 ['O0.444444Rb0.333333In0.222222', '-1.663', '-0.206408618718']
InORe 3 ['O0.666667In0.111111Re0.222222', '-1.995', '-0.206360959206']
InOS 18 ['O0.444444S0.111111In0.444444', '-1.717', '-0.205942951569']
InOSe 1 ['O0.5Se0.125In0.375', '-1.809', '-0.221530962892']
InOTa 4 ['O0.6In0.3Ta0.1', '-2.412', '-0.216477371053']
InOTc 3 ['O0.666667Tc0.166667In0.166667', '-1.933', '-0.210340833613']
InOTi 2 ['O0.6Ti0.2In0.2', '-2.907', '-0.346732276297']
InOU 8 ['O0.571429In0.285714U0.142857', '-2.735', '-0.220038818897']
InOW 4 ['O0.625In0.125W0.25', '-2.249', '-0.209823494449']
InOZr 2 ['O0.625Zr0.25In0.125', '-3.36', '-0.251086184187']
InOsSc 8 ['Sc0.571429In0.285714Os0.142857', '-0.603', '-0.2093065575']
InPPa 1 ['P0.8In0.1Pa0.1', '-0.669', '-0.207672544021']
InPPd 3 ['P0.4Pd0.3In0.3', '-0.728', '-0.214087329702']
InPPm 18 ['P0.5In0.2Pm0.3', '-1.07', '-0.21454051077']
InPPu 2 ['P0.777778In0.111111Pu0.111111', '-0.613', '-0.215962293796']
InPRh 3 ['P0.75Rh0.125In0.125', '-0.735', '-0.225216508593']
InPSc 5 ['P0.3Sc0.5In0.2', '-1.349', '-0.201399169812']
InPSr 1 ['P0.8Sr0.1In0.1', '-0.691', '-0.210006488631']
InPTa 20 ['P0.428571In0.285714Ta0.285714', '-0.757', '-0.203843137946']
InPTb 1 ['P0.8In0.1Tb0.1', '-0.594', '-0.21281360295']
InPTi 8 ['P0.3Ti0.5In0.2', '-1.201', '-0.201493526168']
InPTm 1 ['P0.4In0.2Tm0.4', '-1.596', '-0.211657532416']
InPV 1 ['P0.5V0.2In0.3', '-0.783', '-0.20110513752']
InPXe 3 ['P0.75In0.125Xe0.125', '-0.365', '-0.24085629289']
InPY 3 ['P0.3Y0.5In0.2', '-1.458', '-0.203799318812']
InPZr 2 ['P0.375Zr0.5In0.125', '-1.422', '-0.216323708749']
InPaS 1 ['S0.666667In0.111111Pa0.222222', '-1.324', '-0.212751603409']
InPaSe 8 ['Se0.666667In0.166667Pa0.166667', '-0.864', '-0.2596139525']
InPdPm 4 ['Pd0.75In0.125Pm0.125', '-0.635', '-0.207064793438']
InPdPu 1 ['Pd0.4In0.1Pu0.5', '-0.626', '-0.210800806']
InPmRu 1 ['Ru0.3In0.6Pm0.1', '-0.433', '-0.22453812825']
InPmS 10 ['S0.333333In0.111111Pm0.555556', '-1.603', '-0.236144720604']
InPmSe 21 ['Se0.444444In0.222222Pm0.333333', '-1.401', '-0.20328595']
InPrS 1 ['S0.428571In0.142857Pr0.428571', '-2.065', '-0.202206029111']
InPtS 22 ['S0.5In0.333333Pt0.166667', '-0.88', '-0.211316879452']
InPtTh 8 ['In0.2Pt0.7Th0.1', '-0.775', '-0.2343045065']
InPuSe 1 ['Se0.5In0.1Pu0.4', '-1.553', '-0.21087133575']
InRhSr 1 ['Sr0.1Rh0.3In0.6', '-0.652', '-0.231766359125']
InRhTm 1 ['Rh0.5In0.166667Tm0.333333', '-0.991', '-0.20196391']
InSSc 2 ['S0.5Sc0.4In0.1', '-2.057', '-0.204093367991']
InSSm 3 ['S0.5In0.125Sm0.375', '-2.15', '-0.201433568929']

InSTc 2 ['S0.5Tc0.375In0.125', '-0.828', '-0.210766182993']
InSTm 8 ['S0.444444In0.111111Tm0.444444', '-2.057', '-0.20433882789']
InSY 15 ['S0.25Y0.5In0.25', '-1.53', '-0.207219536495']
InSZr 12 ['S0.333333Zr0.444444In0.222222', '-1.306', '-0.21630089616']
InScSe 10 ['Sc0.5Se0.4In0.1', '-1.571', '-0.2077123625']
InSeSr 9 ['Se0.333333Sr0.444444In0.222222', '-1.739', '-0.200104999444']
InSeTb 2 ['Se0.375In0.125Tb0.5', '-1.336', '-0.207473289725']
InSeTc 3 ['Se0.571429Tc0.285714In0.142857', '-0.429', '-0.254962060714']
InSeTm 6 ['Se0.571429In0.142857Tm0.285714', '-1.501', '-0.204597071431']
InSeY 22 ['Se0.428571Y0.428571In0.142857', '-1.728', '-0.214370376429']
InSeZr 5 ['Se0.5Zr0.375In0.125', '-1.289', '-0.200257269809']
IrKN 3 ['N0.625K0.25Ir0.125', '-0.456', '-0.209680378952']
IrKO 3 ['O0.666667K0.166667Ir0.166667', '-1.343', '-0.231196601495']
IrLaN 8 ['N0.428571La0.428571Ir0.142857', '-1.436', '-0.213333330569']
IrLaP 6 ['P0.625La0.125Ir0.25', '-1.288', '-0.20009894565']
IrLaSe 21 ['Se0.333333La0.333333Ir0.333333', '-1.569', '-0.20751814']
IrLaSi 3 ['Si0.5La0.1Ir0.4', '-1.065', '-0.223845223536']
IrLiN 1 ['Li0.5N0.375Ir0.125', '-0.567', '-0.232792909663']
IrLiP 32 ['Li0.166667P0.5Ir0.333333', '-0.972', '-0.203228265983']
IrLiPa 2 ['Li0.1Ir0.6Pa0.3', '-0.898', '-0.2009480035']
IrLiSc 1 ['Li0.111111Sc0.555556Ir0.333333', '-0.987', '-0.200042540556']
IrLiSe 3 ['Li0.5Se0.333333Ir0.166667', '-1.212', '-0.215549783336']
IrLiSi 4 ['Li0.1Si0.5Ir0.4', '-0.845', '-0.203308503315']
IrLiTa 1 ['Li0.25Ta0.125Ir0.625', '-0.67', '-0.2014952025']
IrLiTm 5 ['Li0.142857Tm0.571429Ir0.285714', '-0.733', '-0.206473648571']
IrLuN 5 ['N0.333333Lu0.5Ir0.166667', '-1.857', '-0.210064169053']
IrLuP 5 ['P0.428571Lu0.285714Ir0.285714', '-1.407', '-0.205391032303']
IrLuS 1 ['S0.375Lu0.5Ir0.125', '-1.993', '-0.238565887243']
IrLuSe 1 ['Se0.333333Lu0.444444Ir0.222222', '-1.674', '-0.209176112222']
IrLuSi 12 ['Si0.4Lu0.1Ir0.5', '-0.982', '-0.207116728374']
IrMgN 5 ['N0.428571Mg0.285714Ir0.285714', '-0.612', '-0.201420045292']
IrMgP 8 ['Mg0.333333P0.222222Ir0.444444', '-0.732', '-0.214162172136']
IrMgPu 1 ['Mg0.25Ir0.375Pu0.375', '-0.655', '-0.221156996796']
IrMgSc 1 ['Mg0.125Sc0.5Ir0.375', '-1.012', '-0.201891295625']
IrMgSi 7 ['Mg0.142857Si0.428571Ir0.428571', '-0.822', '-0.205669586706']
IrNNa 20 ['N0.4Na0.5Ir0.1', '-0.369', '-0.215932916415']
IrNNp 13 ['N0.428571Ir0.285714Np0.285714', '-0.92', '-0.2271673074']
IrNPa 17 ['N0.555556Ir0.222222Pa0.222222', '-0.748', '-0.20341945157']
IrNPu 7 ['N0.571429Ir0.142857Pu0.285714', '-0.996', '-0.213644561331']
IrNRb 10 ['N0.666667Rb0.222222Ir0.111111', '-0.485', '-0.225907000882']
IrNSc 3 ['N0.555556Sc0.333333Ir0.111111', '-1.627', '-0.279937109887']
IrNSr 2 ['N0.444444Sr0.444444Ir0.111111', '-0.774', '-0.206236218458']
IrNTh 1 ['N0.5Ir0.2Th0.3', '-1.42', '-0.208540668862']
IrNTi 6 ['N0.5Ti0.3Ir0.2', '-1.004', '-0.20596244281']

IrNTl 1 ['N0.428571Ir0.285714Tl0.285714', '-0.217', '-0.217']
IrNXe 3 ['N0.3Xe0.6Ir0.1', '-0.201', '-0.201']
IrNY 3 ['N0.25Y0.5Ir0.25', '-1.528', '-0.237826871165']
IrNYb 10 ['N0.285714Yb0.571429Ir0.142857', '-1.108', '-0.208266816329']
IrNaO 4 ['O0.666667Na0.222222Ir0.111111', '-1.347', '-0.214950222557']
IrNaP 9 ['Na0.25P0.375Ir0.375', '-0.773', '-0.203390836496']
IrNaSi 3 ['Na0.1Si0.6Ir0.3', '-0.717', '-0.238305782968']
IrNaSn 6 ['Na0.111111Sn0.444444Ir0.444444', '-0.475', '-0.207852574305']
IrNdP 1 ['P0.444444Nd0.333333Ir0.222222', '-1.528', '-0.21179736074']
IrNdS 3 ['S0.333333Nd0.5Ir0.166667', '-1.853', '-0.22119798116']
IrNdSe 24 ['Se0.2Nd0.5Ir0.3', '-1.267', '-0.2001477265']
IrNdSi 3 ['Si0.428571Nd0.142857Ir0.428571', '-1.162', '-0.212106945357']
IrNiSc 1 ['Sc0.6Ni0.1Ir0.3', '-1.006', '-0.210826226']
IrNpP 1 ['P0.428571Ir0.285714Np0.285714', '-1.058', '-0.266886606211']
IrNpS 1 ['S0.5Ir0.1Np0.4', '-1.674', '-0.233746048492']
IrNpSe 21 ['Se0.5Ir0.125Np0.375', '-1.23', '-0.20001657672']
IrOPa 2 ['O0.714286Ir0.142857Pa0.142857', '-2.01', '-0.22580509521']
IrOPr 1 ['O0.666667Pr0.111111Ir0.222222', '-1.95', '-0.219704155462']
IrOS 1 ['O0.666667S0.222222Ir0.111111', '-1.47', '-0.217523072894']
IrOSr 3 ['O0.666667Sr0.222222Ir0.111111', '-2.007', '-0.212659617743']
IrOYb 2 ['O0.625Yb0.125Ir0.25', '-1.929', '-0.213466214135']
IrOsSc 4 ['Sc0.666667Os0.111111Ir0.222222', '-0.856', '-0.218038369445']
IrOsTm 3 ['Tm0.6Os0.2Ir0.2', '-0.637', '-0.214675452']
IrPPm 26 ['P0.777778Pm0.111111Ir0.111111', '-0.837', '-0.201624587129']
IrPPr 1 ['P0.4Pr0.3Ir0.3', '-1.404', '-0.200288465416']
IrPPu 14 ['P0.4Ir0.4Pu0.2', '-1.049', '-0.201579918016']
IrPSc 1 ['P0.3Sc0.4Ir0.3', '-1.495', '-0.20274006231']
IrPSm 9 ['P0.5Sm0.166667Ir0.333333', '-1.225', '-0.201996482048']
IrPSn 2 ['P0.8Sn0.1Ir0.1', '-0.626', '-0.204701066689']
IrPSr 7 ['P0.444444Sr0.333333Ir0.222222', '-1.28', '-0.200720476017']
IrPTa 4 ['P0.444444Ta0.444444Ir0.111111', '-1.046', '-0.202748960462']
IrPTb 2 ['P0.428571Tb0.285714Ir0.285714', '-1.233', '-0.215289268685']
IrPTi 1 ['P0.444444Ti0.444444Ir0.111111', '-1.474', '-0.248121716004']
IrPTm 4 ['P0.444444Tm0.444444Ir0.111111', '-1.761', '-0.206587430573']
IrPU 3 ['P0.333333Ir0.333333U0.333333', '-1.107', '-0.208227159363']
IrPY 11 ['P0.444444Y0.333333Ir0.222222', '-1.534', '-0.20243351824']
IrPYb 14 ['P0.5Yb0.166667Ir0.333333', '-1.071', '-0.21566686752']
IrPZn 2 ['P0.2Zn0.4Ir0.4', '-0.624', '-0.208652328875']
IrPaS 2 ['S0.5Ir0.166667Pa0.333333', '-1.612', '-0.221256519964']
IrPaSe 19 ['Se0.444444Ir0.222222Pa0.333333', '-1.132', '-0.20135998254']
IrPaYb 2 ['Yb0.125Ir0.625Pa0.25', '-0.929', '-0.200939326625']
IrPmS 16 ['S0.3Pm0.4Ir0.3', '-1.561', '-0.218711860795']
IrPmSe 38 ['Se0.666667Pm0.111111Ir0.222222', '-0.808', '-0.213858966389']
IrPmSi 6 ['Si0.428571Pm0.142857Ir0.428571', '-0.849', '-0.206854040893']

IrPrS 1 ['S0.5Pr0.25Ir0.25', '-1.775', '-0.267834183409']
IrPrSe 22 ['Se0.2Pr0.5Ir0.3', '-1.241', '-0.201191544125']
IrPrSi 6 ['Si0.4Pr0.2Ir0.4', '-0.916', '-0.215566214833']
IrPrSn 1 ['Sn0.375Pr0.25Ir0.375', '-0.818', '-0.201159565016']
IrPuS 1 ['S0.5Ir0.166667Pu0.333333', '-1.735', '-0.206229652158']
IrPuSi 2 ['Si0.5Ir0.375Pu0.125', '-0.947', '-0.208815277344']
IrSSe 13 ['S0.5Se0.3Ir0.2', '-0.553', '-0.200536701894']
IrSSm 10 ['S0.375Sm0.375Ir0.25', '-1.841', '-0.21295364318']
IrSTa 1 ['S0.555556Ta0.222222Ir0.222222', '-1.159', '-0.200336857527']
IrSTh 3 ['S0.5Ir0.125Th0.375', '-2.101', '-0.204950934398']
IrSTi 5 ['S0.571429Ti0.142857Ir0.285714', '-1.094', '-0.205774033862']
IrSXe 4 ['S0.5Xe0.333333Ir0.166667', '-0.498', '-0.204280584912']
IrSZr 1 ['S0.5Zr0.4Ir0.1', '-1.702', '-0.21100727674']
IrScSe 29 ['Sc0.428571Se0.285714Ir0.285714', '-1.432', '-0.200183798571']
IrScSi 7 ['Si0.5Sc0.125Ir0.375', '-0.975', '-0.206638625937']
IrScTl 1 ['Sc0.5Ir0.375Tl0.125', '-1.043', '-0.205233923125']
IrSeSm 10 ['Se0.428571Sm0.285714Ir0.285714', '-1.558', '-0.202281105714']
IrSeTb 2 ['Se0.4Tb0.4Ir0.2', '-1.49', '-0.215935588132']
IrSeTc 5 ['Se0.571429Tc0.285714Ir0.142857', '-0.376', '-0.223925118095']
IrSeTm 17 ['Se0.4Tm0.3Ir0.3', '-1.359', '-0.201511921002']
IrSeY 28 ['Se0.333333Y0.333333Ir0.333333', '-1.39', '-0.212732515']
IrSeZr 1 ['Se0.444444Zr0.444444Ir0.111111', '-1.353', '-0.213351667438']
IrSiSm 2 ['Si0.5Sm0.125Ir0.375', '-1.181', '-0.317526680417']
IrSiSr 10 ['Si0.4Sr0.1Ir0.5', '-0.8', '-0.20548608375']
IrSiTb 7 ['Si0.428571Tb0.142857Ir0.428571', '-1.079', '-0.200309678262']
IrSiTh 1 ['Si0.444444Ir0.444444Th0.111111', '-1.141', '-0.225273248333']
IrSiTi 3 ['Si0.4Ti0.2Ir0.4', '-0.95', '-0.203715443687']
IrSiTm 9 ['Si0.375Tm0.25Ir0.375', '-1.008', '-0.228292686875']
IrSiV 1 ['Si0.375V0.25Ir0.375', '-0.833', '-0.201839137768']
IrSiY 3 ['Si0.555556Y0.111111Ir0.333333', '-0.986', '-0.21173407287']
IrSiYb 8 ['Si0.6Yb0.1Ir0.3', '-0.782', '-0.204233902687']
IrSiZr 1 ['Si0.428571Zr0.142857Ir0.428571', '-0.996', '-0.246836706071']
IrSnY 1 ['Y0.333333Sn0.333333Ir0.333333', '-0.978', '-0.24267562855']
KLaN 2 ['N0.4K0.1La0.5', '-1.346', '-0.204844441864']
KLaO 1 ['O0.6K0.2La0.2', '-2.667', '-0.23056152722']
KLaTe 5 ['K0.1Te0.6La0.3', '-1.64', '-0.200011690998']
KLuO 1 ['O0.6K0.2Lu0.2', '-2.765', '-0.20658834297']
KLuS 1 ['S0.5K0.3Lu0.2', '-1.91', '-0.221722318077']
KLuTe 3 ['K0.142857Te0.571429Lu0.285714', '-1.249', '-0.215859094169']
KMgO 3 ['O0.444444Mg0.333333K0.222222', '-2.579', '-0.22350369063']
KMgS 4 ['Mg0.428571S0.428571K0.142857', '-1.602', '-0.224508553579']
KMnN 26 ['N0.5K0.333333Mn0.166667', '-0.457', '-0.203656185099']
KMnO 2 ['O0.625K0.25Mn0.125', '-1.692', '-0.243164460503']
KMnTe 1 ['K0.444444Mn0.111111Te0.444444', '-1.002', '-0.200607229208']

KMoN 22 ['N0.428571K0.142857Mo0.428571', '-0.61', '-0.206412373067']
KMoO 2 ['O0.428571K0.428571Mo0.142857', '-1.95', '-0.200586951052']
KMoSe 7 ['K0.222222Se0.444444Mo0.333333', '-0.985', '-0.200020220952']
KNNb 1 ['N0.5K0.25Nb0.25', '-0.937', '-0.237011872659']
KNNi 12 ['N0.555556K0.222222Ni0.222222', '-0.419', '-0.200049225735']
KNNp 12 ['N0.428571K0.285714Np0.285714', '-0.954', '-0.204865679732']
KNOs 7 ['N0.571429K0.142857Os0.285714', '-0.382', '-0.213095116995']
KNP 28 ['N0.571429P0.285714K0.142857', '-0.803', '-0.208502677898']
KNPa 12 ['N0.5K0.3Pa0.2', '-0.741', '-0.201314812605']
KNPt 11 ['N0.6K0.2Pt0.2', '-0.437', '-0.200533163794']
KNPu 9 ['N0.6K0.1Pu0.3', '-1.154', '-0.214293371295']
KNRe 15 ['N0.428571K0.285714Re0.285714', '-0.372', '-0.203095116995']
KNRh 9 ['N0.444444K0.222222Rh0.333333', '-0.384', '-0.208839380588']
KNRu 13 ['N0.571429K0.285714Ru0.142857', '-0.431', '-0.205793489327']
KNSn 1 ['N0.5K0.166667Sn0.333333', '-0.409', '-0.200176844923']
KNTc 16 ['N0.5K0.125Tc0.375', '-0.466', '-0.232694178578']
KNTi 9 ['N0.444444K0.111111Ti0.444444', '-1.412', '-0.2006915073']
KNV 2 ['N0.555556K0.222222V0.222222', '-0.908', '-0.219782480921']
KNW 29 ['N0.428571K0.428571W0.142857', '-0.372', '-0.203095116995']
KNZr 1 ['N0.5K0.1Zr0.4', '-1.698', '-0.223905944579']
KNaP 1 ['Na0.4P0.4K0.2', '-0.779', '-0.247606125159']
KNaS 3 ['Na0.166667S0.5K0.333333', '-1.279', '-0.202721478824']
KNaSe 2 ['Na0.3K0.3Se0.4', '-1.295', '-0.225875168662']
KNbO 2 ['O0.5K0.375Nb0.125', '-2.384', '-0.262041321434']
KNdSe 2 ['K0.111111Se0.444444Nd0.444444', '-1.845', '-0.207516273333']
KNdTe 1 ['K0.222222Te0.555556Nd0.222222', '-1.482', '-0.216167121701']
KNpO 3 ['O0.375K0.125Np0.5', '-2.376', '-0.200490835737']
KNpS 3 ['S0.555556K0.222222Np0.222222', '-1.614', '-0.209271352803']
KOOs 10 ['O0.625K0.125Os0.25', '-1.628', '-0.215804323412']
KOP 3 ['O0.4P0.3K0.3', '-2.223', '-0.222596836235']
KOPa 5 ['O0.666667K0.166667Pa0.166667', '-2.685', '-0.229253332892']
KOPb 5 ['O0.666667K0.166667Pb0.166667', '-1.33', '-0.23185158108']
KOPd 11 ['O0.4K0.5Pd0.1', '-1.414', '-0.21863676865']
KOPt 10 ['O0.666667K0.166667Pt0.166667', '-1.158', '-0.231402860663']
KOPu 4 ['O0.666667K0.111111Pu0.222222', '-2.805', '-0.242139825398']
KORb 2 ['O0.7K0.1Rb0.2', '-1.196', '-0.202411472382']
KORe 5 ['O0.5K0.4Re0.1', '-1.873', '-0.227462592631']
KORh 6 ['O0.375K0.5Rh0.125', '-1.299', '-0.203835578867']
KORu 2 ['O0.625K0.25Ru0.125', '-1.714', '-0.332250084291']
KOSb 3 ['O0.444444K0.444444Sb0.111111', '-1.858', '-0.222416940445']
KOSe 24 ['O0.285714K0.285714Se0.428571', '-1.317', '-0.20345439814']
KOSi 1 ['O0.6Si0.2K0.2', '-2.682', '-0.206254943713']
KOSn 2 ['O0.625K0.25Sn0.125', '-1.738', '-0.248679191195']
KOTa 2 ['O0.625K0.25Ta0.125', '-2.449', '-0.218091855073']

KOTc 8 ['O0.4K0.5Tc0.1', '-1.525', '-0.207369076047']
KOTe 11 ['O0.5K0.3Te0.2', '-1.868', '-0.207411297352']
KOTh 2 ['O0.625K0.25Th0.125', '-2.516', '-0.205164316794']
KOTi 5 ['O0.5K0.25Ti0.25', '-2.839', '-0.201304129243']
KOTl 11 ['O0.444444K0.111111Tl0.444444', '-1.204', '-0.210222858776']
KOTm 1 ['O0.6K0.2Tm0.2', '-2.809', '-0.26548312422']
KOZr 1 ['O0.6K0.2Zr0.2', '-3.024', '-0.264847497805']
KOsP 1 ['P0.5K0.2Os0.3', '-0.747', '-0.21369527352']
KPPm 6 ['P0.4K0.2Pm0.4', '-1.172', '-0.218585395416']
KPPt 4 ['P0.444444K0.333333Pt0.222222', '-0.771', '-0.200031868795']
KPRh 3 ['P0.571429K0.142857Rh0.285714', '-1.004', '-0.222033332607']
KPRu 3 ['P0.428571K0.142857Ru0.428571', '-0.787', '-0.202393510446']
KPS 4 ['P0.111111S0.444444K0.444444', '-1.224', '-0.203357915767']
KPTe 5 ['P0.1K0.5Te0.4', '-1.1', '-0.25487456498']
KPaS 16 ['S0.5K0.4Pa0.1', '-1.437', '-0.203849105244']
KPaSe 13 ['K0.333333Se0.555556Pa0.111111', '-1.144', '-0.214577520926']
KPaSi 2 ['Si0.666667K0.111111Pa0.222222', '-0.414', '-0.277049556667']
KPaTe 1 ['K0.2Te0.6Pa0.2', '-0.83', '-0.216076218333']
KPbS 7 ['S0.375K0.5Pb0.125', '-1.247', '-0.203686255998']
KPbSe 3 ['K0.111111Se0.444444Pb0.444444', '-0.83', '-0.202368392778']
KPdPm 1 ['K0.1Pd0.7Pm0.2', '-0.524', '-0.2123499855']
KPmS 17 ['S0.6K0.2Pm0.2', '-1.785', '-0.200379570591']
KPmSe 18 ['K0.1Se0.4Pm0.5', '-1.503', '-0.201708421125']
KPmTe 5 ['K0.2Te0.6Pm0.2', '-1.122', '-0.238375779832']
KPrSe 3 ['K0.2Se0.5Pr0.3', '-1.856', '-0.214821609748']
KPrTe 1 ['K0.111111Te0.555556Pr0.333333', '-1.611', '-0.202617362317']
KPtS 1 ['S0.625K0.25Pt0.125', '-1.022', '-0.204149443624']
KPtTh 3 ['K0.1Pt0.8Th0.1', '-0.461', '-0.2167340785']
KPuS 15 ['S0.428571K0.142857Pu0.428571', '-1.847', '-0.202333702563']
KPuTe 2 ['K0.125Te0.5Pu0.375', '-1.302', '-0.244472105207']
KRhS 7 ['S0.444444K0.222222Rh0.333333', '-0.966', '-0.200324920162']
KSSe 13 ['S0.428571K0.285714Se0.285714', '-0.912', '-0.204696863208']
KSSi 9 ['Si0.125S0.375K0.5', '-1.254', '-0.220435680686']
KSTb 2 ['S0.5K0.2Tb0.3', '-1.937', '-0.227586222347']
KSTc 4 ['S0.5K0.375Tc0.125', '-1.214', '-0.204457450287']
KSTe 7 ['S0.125K0.5Te0.375', '-1.141', '-0.236299774395']
KSTh 2 ['S0.555556K0.222222Th0.222222', '-2.074', '-0.23147378388']
KSU 5 ['S0.5K0.333333U0.166667', '-1.571', '-0.208721498275']
KSXe 2 ['S0.666667K0.111111Xe0.222222', '-0.508', '-0.200257584302']
KSY 1 ['S0.5K0.166667Y0.333333', '-2.162', '-0.213107074663']
KSZn 1 ['S0.4K0.4Zn0.2', '-1.286', '-0.205906858397']
KSZr 15 ['S0.555556K0.111111Zr0.333333', '-1.748', '-0.20635687999']
KScSe 11 ['K0.3Sc0.2Se0.5', '-1.41', '-0.200083488']
KSeSi 18 ['Si0.1K0.5Se0.4', '-1.135', '-0.200091408625']

KSeSm 1 ['K0.166667Se0.5Sm0.333333', '-1.906', '-0.230535595417']
KSeTc 14 ['K0.1Se0.7Tc0.2', '-0.406', '-0.2025620165']
KSeTe 29 ['K0.625Se0.125Te0.25', '-1.236', '-0.220572018335']
KSeTh 1 ['K0.142857Se0.571429Th0.285714', '-1.782', '-0.204503186426']
KSeTi 1 ['K0.125Ti0.375Se0.5', '-1.403', '-0.218175005848']
KSeTl 4 ['K0.111111Se0.333333Tl0.555556', '-0.644', '-0.213557657778']
KSeTm 5 ['K0.142857Se0.571429Tm0.285714', '-1.665', '-0.218065211431']
KSeY 14 ['K0.2Se0.6Y0.2', '-1.352', '-0.23974924725']
KSeZn 1 ['K0.444444Zn0.222222Se0.333333', '-1.107', '-0.206070710576']
KSeZr 2 ['K0.125Se0.5Zr0.375', '-1.418', '-0.250349992413']
KTbTe 3 ['K0.142857Te0.571429Tb0.285714', '-1.215', '-0.24038839962']
KTeTm 2 ['K0.111111Te0.555556Tm0.333333', '-1.365', '-0.267854153427']
KTeY 2 ['K0.1Y0.3Te0.6', '-1.48', '-0.216214139']
KrLaSe 1 ['Se0.4Kr0.1La0.5', '-1.817', '-0.217083088']
KrLiS 5 ['Li0.111111S0.444444Kr0.444444', '-0.443', '-0.202426700888']
KrNNp 1 ['N0.555556Kr0.111111Np0.333333', '-1.214', '-0.405695191967']
KrNPa 15 ['N0.5Kr0.25Pa0.25', '-0.7', '-0.206027904525']
KrNTi 3 ['N0.5Ti0.375Kr0.125', '-1.181', '-0.208222383494']
KrNaO 2 ['O0.625Na0.25Kr0.125', '-1.038', '-0.205327638614']
KrNpP 4 ['P0.555556Kr0.333333Np0.111111', '-0.44', '-0.206471197152']
KrOP 1 ['O0.7P0.2Kr0.1', '-1.828', '-0.215947445769']
KrOPa 1 ['O0.666667Kr0.111111Pa0.222222', '-2.507', '-0.441133214053']
KrOSe 6 ['O0.5Se0.125Kr0.375', '-0.559', '-0.201584551968']
KrOsSc 6 ['Sc0.555556Kr0.222222Os0.222222', '-0.342', '-0.200063627778']
KrOsV 2 ['V0.666667Kr0.111111Os0.222222', '-0.301', '-0.200256968889']
KrPPa 2 ['P0.777778Kr0.111111Pa0.111111', '-0.629', '-0.226763899677']
KrPPb 1 ['P0.428571Kr0.285714Pb0.285714', '-0.203', '-0.203']
KrPPm 32 ['P0.5Kr0.25Pm0.25', '-0.796', '-0.200115872135']
KrPPu 1 ['P0.777778Kr0.111111Pu0.111111', '-0.493', '-0.206312255671']
KrPRh 1 ['P0.777778Kr0.111111Rh0.111111', '-0.581', '-0.238209080624']
KrPSn 2 ['P0.8Kr0.1Sn0.1', '-0.289', '-0.201185287127']
KrPTa 27 ['P0.444444Kr0.333333Ta0.222222', '-0.503', '-0.206241295787']
KrPTe 33 ['P0.4Kr0.5Te0.1', '-0.202', '-0.202']
KrPU 4 ['P0.7Kr0.2U0.1', '-0.497', '-0.206273879458']
KrPXe 17 ['P0.5Kr0.166667Xe0.333333', '-0.201', '-0.201']
KrPY 1 ['P0.8Kr0.1Y0.1', '-0.655', '-0.20522327452']
KrPZn 7 ['P0.5Zn0.3Kr0.2', '-0.516', '-0.200069116801']
KrPaS 2 ['S0.666667Kr0.111111Pa0.222222', '-1.119', '-0.210626314772']
KrPaSe 18 ['Se0.444444Kr0.333333Pa0.222222', '-0.748', '-0.21287762']
KrPdPm 1 ['Kr0.1Pd0.7Pm0.2', '-0.529', '-0.2173499855']
KrPmS 11 ['S0.5Kr0.1Pm0.4', '-2.063', '-0.214449111991']
KrPmSe 24 ['Se0.555556Kr0.333333Pm0.111111', '-0.566', '-0.2057821775']
KrPtTh 2 ['Kr0.111111Pt0.777778Th0.111111', '-0.494', '-0.222593420556']
KrSTc 1 ['S0.5Kr0.125Tc0.375', '-0.831', '-0.225663187993']

KrSXe 4 ['S0.5Kr0.125Xe0.375', '-0.208', '-0.208']
KrScSe 14 ['Sc0.333333Se0.444444Kr0.222222', '-1.268', '-0.205325498333']
KrSeTc 13 ['Se0.444444Kr0.222222Tc0.333333', '-0.206', '-0.206']
KrSeTm 1 ['Se0.555556Kr0.111111Tm0.333333', '-1.618', '-0.241603552224']
KrSeY 15 ['Se0.5Kr0.375Y0.125', '-0.648', '-0.206524693125']
LaLiN 15 ['Li0.444444N0.333333La0.222222', '-1.036', '-0.212825132664']
LaLiS 7 ['Li0.2S0.5La0.3', '-2.286', '-0.205564928992']
LaLiSe 8 ['Li0.333333Se0.444444La0.222222', '-1.847', '-0.202276127779']
LaLiSn 1 ['Li0.333333Sn0.333333La0.333333', '-0.836', '-0.206186547439']
LaLuO 5 ['O0.666667La0.222222Lu0.111111', '-3.367', '-0.204024821364']
LaLuTe 1 ['Te0.6La0.1Lu0.3', '-1.376', '-0.201511107334']
LaMgN 13 ['N0.5Mg0.125La0.375', '-1.466', '-0.216537934063']
LaMgO 3 ['O0.6Mg0.1La0.3', '-3.584', '-0.206023675743']
LaMgS 22 ['Mg0.1S0.4La0.5', '-2.078', '-0.207007105475']
LaMgSe 14 ['Mg0.333333Se0.444444La0.222222', '-1.664', '-0.211691656853']
LaMgSi 5 ['Mg0.142857Si0.428571La0.428571', '-0.859', '-0.213718251163']
LaMnN 25 ['N0.4Mn0.5La0.1', '-0.796', '-0.236150144466']
LaMnSi 5 ['Si0.571429Mn0.285714La0.142857', '-0.771', '-0.22033157963']
LaNNa 4 ['N0.333333Na0.166667La0.5', '-1.185', '-0.234037034887']
LaNNi 14 ['N0.444444Ni0.333333La0.222222', '-0.848', '-0.204834132108']
LaNNp 23 ['N0.4La0.2Np0.4', '-1.267', '-0.211439336112']
LaNO 24 ['N0.1O0.5La0.4', '-3.484', '-0.200025625077']
LaNP 47 ['N0.555556P0.333333La0.111111', '-0.947', '-0.200222404948']
LaNPa 18 ['N0.444444La0.333333Pa0.222222', '-1.487', '-0.202613279477']
LaNPd 8 ['N0.3Pd0.2La0.5', '-1.39', '-0.201755482398']
LaNPt 21 ['N0.25La0.5Pt0.25', '-1.526', '-0.219084974915']
LaNPu 4 ['N0.555556La0.222222Pu0.222222', '-1.473', '-0.230526015404']
LaNRh 8 ['N0.4Rh0.4La0.2', '-0.771', '-0.200422220932']
LaNRu 1 ['N0.375Ru0.125La0.5', '-1.332', '-0.221508160498']
LaNS 4 ['N0.142857S0.285714La0.571429', '-1.933', '-0.205505448089']
LaNSe 2 ['N0.142857Se0.285714La0.571429', '-1.764', '-0.213646649237']
LaNSi 22 ['N0.111111Si0.333333La0.555556', '-1.044', '-0.208258906629']
LaNSr 2 ['N0.428571Sr0.142857La0.428571', '-1.428', '-0.205333330569']
LaNTa 4 ['N0.5La0.3Ta0.2', '-1.63', '-0.208659855998']
LaNTc 2 ['N0.333333Tc0.111111La0.555556', '-1.173', '-0.222037034887']
LaNTe 3 ['N0.4Te0.2La0.4', '-1.616', '-0.211114439243']
LaNTi 15 ['N0.5Ti0.125La0.375', '-1.61', '-0.215907458745']
LaNTl 5 ['N0.428571La0.428571Tl0.142857', '-1.441', '-0.218333330569']
LaNU 8 ['N0.555556La0.222222U0.222222', '-1.683', '-0.208632153697']
LaNV 6 ['N0.5V0.166667La0.333333', '-1.586', '-0.214340599692']
LaNW 12 ['N0.571429La0.285714W0.142857', '-1.077', '-0.209641495075']
LaNXe 1 ['N0.375Xe0.125La0.5', '-1.27', '-0.200166664247']
LaNYb 1 ['N0.444444La0.444444Yb0.111111', '-1.495', '-0.227049379849']
LaNZn 23 ['N0.333333Zn0.444444La0.222222', '-0.844', '-0.210024689924']

LaNZr 2 ['N0.428571Zr0.142857La0.428571', '-1.546', '-0.220840724854']
LaNaO 5 ['O0.4Na0.1La0.5', '-2.688', '-0.21297457448']
LaNaPd 1 ['Na0.1Pd0.6La0.3', '-0.941', '-0.20239376375']
LaNaS 6 ['Na0.2S0.5La0.3', '-2.226', '-0.204578311822']
LaNaSe 12 ['Na0.222222Se0.444444La0.333333', '-1.92', '-0.204401660923']
LaNbO 2 ['O0.666667Nb0.222222La0.111111', '-3.359', '-0.200544762333']
LaNbS 1 ['S0.555556Nb0.222222La0.222222', '-2.017', '-0.203333834232']
LaNbSe 4 ['Se0.555556Nb0.222222La0.222222', '-1.648', '-0.201775983315']
LaNiO 2 ['O0.625Ni0.125La0.25', '-2.858', '-0.213138230656']
LaNiSe 1 ['Ni0.1Se0.4La0.5', '-1.881', '-0.2136779535']
LaNiSi 1 ['Si0.444444Ni0.222222La0.333333', '-1.044', '-0.269243451667']
LaNpO 1 ['O0.666667La0.222222Np0.111111', '-3.559', '-0.2596759254']
LaNpSe 2 ['Se0.5La0.4Np0.1', '-2.02', '-0.204385026']
LaOP 16 ['O0.375P0.125La0.5', '-2.947', '-0.202021109955']
LaOPa 7 ['O0.625La0.25Pa0.125', '-3.828', '-0.34561359648']
LaOPb 1 ['O0.625La0.25Pb0.125', '-2.984', '-0.245702072731']
LaOPd 4 ['O0.666667Pd0.111111La0.222222', '-2.552', '-0.297004667744']
LaOPt 3 ['O0.625La0.25Pt0.125', '-2.813', '-0.214838230855']
LaORh 1 ['O0.6Rh0.2La0.2', '-2.611', '-0.22529276197']
LaOS 8 ['O0.2S0.3La0.5', '-2.831', '-0.202007593621']
LaOSb 2 ['O0.3Sb0.3La0.4', '-2.59', '-0.212367174234']
LaOSc 1 ['O0.625Sc0.125La0.25', '-3.784', '-0.255773014581']
LaOSe 33 ['O0.5Se0.2La0.3', '-3.057', '-0.201113306684']
LaOSi 1 ['O0.333333Si0.166667La0.5', '-2.533', '-0.2022999054']
LaOSm 1 ['O0.625La0.25Sm0.125', '-3.7', '-0.223091558956']
LaOTa 2 ['O0.6La0.2Ta0.2', '-3.561', '-0.257711757675']
LaOTb 2 ['O0.666667La0.222222Tb0.111111', '-3.344', '-0.292515609389']
LaOTc 8 ['O0.625Tc0.25La0.125', '-2.512', '-0.235883577416']
LaOTe 4 ['O0.375Te0.25La0.375', '-3.198', '-0.202404981544']
LaOTh 1 ['O0.666667La0.166667Th0.166667', '-3.916', '-0.25065232105']
LaOTl 1 ['O0.333333La0.111111Tl0.555556', '-1.767', '-0.37492001172']
LaOTm 5 ['O0.666667La0.166667Tm0.166667', '-3.449', '-0.263840440704']
LaOW 4 ['O0.666667La0.166667W0.166667', '-3.25', '-0.220295124219']
LaOY 2 ['O0.625Y0.25La0.125', '-3.795', '-0.254710356456']
LaOYb 1 ['O0.625La0.25Yb0.125', '-3.416', '-0.21858633409']
LaOZn 5 ['O0.625Zn0.25La0.125', '-2.192', '-0.207379687818']
LaOZr 3 ['O0.625Zr0.25La0.125', '-3.808', '-0.211483326888']
LaOsP 1 ['P0.5La0.2Os0.3', '-1.223', '-0.23347171702']
LaOsSe 1 ['Se0.375La0.5Os0.125', '-1.753', '-0.245219476875']
LaPPd 1 ['P0.5Pd0.25La0.25', '-1.425', '-0.24164063018']
LaPPm 2 ['P0.5La0.1Pm0.4', '-1.526', '-0.23287135252']
LaPPu 2 ['P0.444444La0.222222Pu0.333333', '-1.596', '-0.267704416018']
LaPRh 9 ['P0.444444Rh0.222222La0.333333', '-1.645', '-0.201671158798']
LaPS 2 ['P0.3S0.5La0.2', '-1.6', '-0.210302895506']

LaPSe 2 ['P0.166667Se0.5La0.333333', '-1.881', '-0.209906942657']
LaPZn 1 ['P0.428571Zn0.285714La0.285714', '-1.274', '-0.200000411745']
LaPaS 2 ['S0.666667La0.111111Pa0.222222', '-1.796', '-0.200795421379']
LaPaSe 7 ['Se0.6La0.1Pa0.3', '-1.466', '-0.213706863749']
LaPaSi 1 ['Si0.666667La0.111111Pa0.222222', '-0.561', '-0.202085582222']
LaPdSe 29 ['Se0.5Pd0.1La0.4', '-2.055', '-0.201465436625']
LaPmS 3 ['S0.5La0.1Pm0.4', '-2.162', '-0.20374202099']
LaPmSe 7 ['Se0.555556La0.111111Pm0.333333', '-1.87', '-0.200593284443']
LaPmTe 1 ['Te0.6La0.1Pm0.3', '-1.422', '-0.218318408331']
LaPtS 8 ['S0.5La0.4Pt0.1', '-2.375', '-0.203679374992']
LaPtSe 25 ['Se0.25La0.375Pt0.375', '-1.697', '-0.203998004377']
LaPtSi 7 ['Si0.428571La0.142857Pt0.428571', '-1.279', '-0.2830183125']
LaPuSe 1 ['Se0.4La0.5Pu0.1', '-1.813', '-0.213083088']
LaRbS 1 ['S0.555556Rb0.333333La0.111111', '-1.654', '-0.21031668888']
LaRbTe 2 ['Rb0.1Te0.6La0.3', '-1.587', '-0.208419740187']
LaRhS 14 ['S0.428571Rh0.428571La0.142857', '-1.294', '-0.204224305243']
LaRhSe 23 ['Se0.375Rh0.375La0.25', '-1.424', '-0.203020116839']
LaRhSi 10 ['Si0.375Rh0.5La0.125', '-1.18', '-0.20294461281']
LaRhSn 1 ['Rh0.4Sn0.4La0.2', '-0.967', '-0.204752535426']
LaRuSe 7 ['Se0.5Ru0.125La0.375', '-1.983', '-0.2134553175']
LaRuSi 1 ['Si0.5Ru0.375La0.125', '-0.992', '-0.224853256458']
LaSSb 5 ['S0.222222Sb0.333333La0.444444', '-1.858', '-0.223875616144']
LaSSe 1 ['S0.142857Se0.285714La0.571429', '-2.012', '-0.209232707997']
LaSSi 18 ['Si0.111111S0.333333La0.555556', '-1.925', '-0.206285234216']
LaSTc 6 ['S0.6Tc0.3La0.1', '-1.359', '-0.221484627092']
LaSTe 1 ['S0.25Te0.375La0.375', '-2.073', '-0.237728404673']
LaSZr 3 ['S0.5Zr0.4La0.1', '-1.865', '-0.20921560374']
LaSbSe 18 ['Se0.125Sb0.5La0.375', '-1.362', '-0.201973914844']
LaSbTe 1 ['Sb0.25Te0.375La0.375', '-1.575', '-0.201221014374']
LaScSe 3 ['Sc0.333333Se0.555556La0.111111', '-1.855', '-0.203572250277']
LaSeSi 21 ['Si0.25Se0.25La0.5', '-1.577', '-0.2011901325']
LaSeTa 3 ['Se0.6La0.2Ta0.2', '-1.564', '-0.208670209222']
LaSeTc 3 ['Se0.625Tc0.25La0.125', '-0.883', '-0.220652595936']
LaSeTe 14 ['Se0.3Te0.3La0.4', '-1.965', '-0.20185717']
LaSeTl 7 ['Se0.428571La0.428571Tl0.142857', '-1.921', '-0.206803308571']
LaSeTm 3 ['Se0.6La0.2Tm0.2', '-1.986', '-0.216404967335']
LaSeXe 1 ['Se0.375Xe0.125La0.5', '-1.721', '-0.221077895']
LaSeY 7 ['Se0.625Y0.125La0.25', '-1.973', '-0.206829884997']
LaSeZn 20 ['Zn0.166667Se0.333333La0.5', '-1.651', '-0.212410713333']
LaSeZr 5 ['Se0.5Zr0.333333La0.166667', '-1.649', '-0.202414434336']
LaTbTe 1 ['Te0.6La0.1Tb0.3', '-1.336', '-0.223368541267']
LiLuN 2 ['Li0.125N0.375Lu0.5', '-1.844', '-0.356334068623']
LiLuOs 1 ['Li0.1Lu0.6Os0.3', '-0.388', '-0.210516553']
LiLuPt 2 ['Li0.166667Lu0.5Pt0.333333', '-1.162', '-0.21775241958']

LiLuSe 1 ['Li0.125Se0.5Lu0.375', '-1.813', '-0.200773372501']
LiMnN 11 ['Li0.222222N0.444444Mn0.333333', '-0.546', '-0.201432329694']
LiMnP 3 ['Li0.1P0.4Mn0.5', '-0.889', '-0.201996469186']
LiMoN 3 ['Li0.125N0.375Mo0.5', '-0.665', '-0.204365916746']
LiNNb 19 ['Li0.125N0.5Nb0.375', '-1.305', '-0.239526663061']
LiNNd 1 ['Li0.1N0.4Nd0.5', '-1.384', '-0.212880007864']
LiNNp 16 ['Li0.555556N0.333333Np0.111111', '-0.816', '-0.223928256968']
LiNOs 1 ['Li0.5N0.4Os0.1', '-0.53', '-0.200184131112']
LiNP 9 ['Li0.5N0.333333P0.166667', '-1.067', '-0.206698742246']
LiNPa 25 ['Li0.1N0.4Pa0.5', '-1.276', '-0.224722151444']
LiNPm 4 ['Li0.2N0.4Pm0.4', '-1.441', '-0.207767928864']
LiNPt 4 ['Li0.3N0.5Pt0.2', '-0.584', '-0.226912933397']
LiNPu 25 ['Li0.555556N0.333333Pu0.111111', '-0.85', '-0.223113855719']
LiNRe 6 ['Li0.2N0.5Re0.3', '-0.554', '-0.227130273715']
LiNRu 10 ['Li0.125N0.5Ru0.375', '-0.352', '-0.215312261746']
LiNSb 5 ['Li0.4N0.4Sb0.2', '-0.599', '-0.219949767418']
LiNSn 5 ['Li0.6N0.3Sn0.1', '-0.606', '-0.219494909312']
LiNTc 18 ['Li0.444444N0.444444Tc0.111111', '-0.53', '-0.201182070264']
LiNTh 14 ['Li0.1N0.5Th0.4', '-1.778', '-0.213612431996']
LiNTi 20 ['Li0.111111N0.555556Ti0.333333', '-1.2', '-0.213808573546']
LiNTm 2 ['Li0.125N0.375Tm0.5', '-1.714', '-0.267003345498']
LiNU 6 ['Li0.5N0.375U0.125', '-1.072', '-0.201556342997']
LiNV 3 ['Li0.166667N0.5V0.333333', '-1.229', '-0.209199037273']
LiNW 16 ['Li0.333333N0.444444W0.222222', '-0.795', '-0.204670909847']
LiNY 9 ['Li0.1N0.4Y0.5', '-1.643', '-0.206821803864']
LiNZr 6 ['Li0.111111N0.444444Zr0.444444', '-1.788', '-0.201183495404']
LiNaP 1 ['Li0.571429Na0.142857P0.285714', '-0.921', '-0.211299571664']
LiNdP 1 ['Li0.125P0.375Nd0.5', '-1.487', '-0.237051142265']
LiNdS 1 ['Li0.25S0.375Nd0.375', '-1.853', '-0.223463101617']
LiNdSe 12 ['Li0.1Se0.4Nd0.5', '-1.684', '-0.20366689925']
LiNpO 4 ['Li0.125O0.375Np0.5', '-2.405', '-0.220902967956']
LiNpP 3 ['Li0.166667P0.5Np0.333333', '-1.078', '-0.201074054478']
LiNpPt 2 ['Li0.222222Pt0.666667Np0.111111', '-0.628', '-0.205672709028']
LiNpS 4 ['Li0.125S0.5Np0.375', '-1.767', '-0.227970030806']
LiNpSe 1 ['Li0.1Se0.6Np0.3', '-1.221', '-0.206198595377']
LiOOs 3 ['Li0.2O0.6Os0.2', '-1.778', '-0.20306638247']
LiOP 1 ['Li0.3O0.4P0.3', '-2.347', '-0.222006304209']
LiOPa 1 ['Li0.111111O0.666667Pa0.222222', '-2.916', '-0.237012382288']
LiOPu 7 ['Li0.125O0.375Pu0.5', '-2.527', '-0.208567001572']
LiORe 2 ['Li0.2O0.6Re0.2', '-2.201', '-0.213006799472']
LiORh 2 ['Li0.125O0.625Rh0.25', '-1.405', '-0.201218484868']
LiOSe 17 ['Li0.333333O0.222222Se0.444444', '-1.308', '-0.202198419898']
LiOSi 1 ['Li0.2O0.6Si0.2', '-2.834', '-0.256075252874']
LiOTc 1 ['Li0.2O0.6Tc0.2', '-2.18', '-0.33300009947']

LiOTe 3 ['Li0.166667O0.666667Te0.166667', '-1.625', '-0.212057770597']
LiOTi 2 ['Li0.125O0.5Ti0.375', '-3.008', '-0.218051428765']
LiOU 4 ['Li0.2O0.6U0.2', '-3.359', '-0.21265277297']
LiOV 1 ['Li0.1O0.5V0.4', '-2.594', '-0.228412852948']
LiOZr 1 ['Li0.333333O0.333333Zr0.333333', '-2.193', '-0.223182317126']
LiOsP 12 ['Li0.222222P0.555556Os0.222222', '-0.905', '-0.20316718148']
LiOsSc 3 ['Li0.142857Sc0.571429Os0.285714', '-0.513', '-0.229973005714']
LiOsTm 6 ['Li0.2Tm0.5Os0.3', '-0.296', '-0.215365847']
LiOsV 1 ['Li0.1V0.6Os0.3', '-0.338', '-0.201996908']
LiPPd 13 ['Li0.222222P0.444444Pd0.333333', '-0.836', '-0.201229184759']
LiPPm 6 ['Li0.125P0.5Pm0.375', '-1.264', '-0.206640850989']
LiPPr 2 ['Li0.125P0.375Pr0.5', '-1.446', '-0.212201303827']
LiPPt 29 ['Li0.5P0.333333Pt0.166667', '-0.971', '-0.201337847489']
LiPPu 5 ['Li0.25P0.5Pu0.25', '-1.176', '-0.203886660833']
LiPRh 46 ['Li0.142857P0.571429Rh0.285714', '-1.011', '-0.203398694959']
LiPRu 28 ['Li0.142857P0.285714Ru0.571429', '-0.694', '-0.201585644464']
LiPSm 2 ['Li0.125P0.375Sm0.5', '-1.489', '-0.214073595078']
LiPSn 2 ['Li0.3P0.3Sn0.4', '-0.617', '-0.222769229816']
LiPTa 10 ['Li0.166667P0.333333Ta0.5', '-1.009', '-0.210152991649']
LiPTb 2 ['Li0.142857P0.428571Tb0.428571', '-1.488', '-0.26870938129']
LiPTc 10 ['Li0.3P0.5Tc0.2', '-0.911', '-0.205379205687']
LiPXe 4 ['Li0.4P0.4Xe0.2', '-0.724', '-0.200694536916']
LiPY 6 ['Li0.111111P0.333333Y0.555556', '-1.372', '-0.202697022013']
LiPYb 1 ['Li0.1P0.4Yb0.5', '-1.044', '-0.202996122583']
LiPZr 9 ['Li0.25P0.25Zr0.5', '-1.083', '-0.210961581093']
LiPaPt 12 ['Li0.1Pt0.8Pa0.1', '-0.769', '-0.204071333667']
LiPaS 4 ['Li0.1S0.7Pa0.2', '-1.235', '-0.200947714094']
LiPaSe 9 ['Li0.166667Se0.666667Pa0.166667', '-0.93', '-0.210633771668']
LiPaSi 2 ['Li0.1Si0.7Pa0.2', '-0.387', '-0.223247794375']
LiPbSe 2 ['Li0.25Se0.375Pb0.375', '-0.984', '-0.222489757501']
LiPdPm 8 ['Li0.125Pd0.625Pm0.25', '-0.706', '-0.204010460625']
LiPmPt 1 ['Li0.222222Pm0.111111Pt0.666667', '-0.898', '-0.213104651019']
LiPmS 17 ['Li0.333333S0.333333Pm0.333333', '-1.549', '-0.203830381578']
LiPmSb 1 ['Li0.1Sb0.4Pm0.5', '-0.971', '-0.205024852417']
LiPmSe 24 ['Li0.111111Se0.666667Pm0.222222', '-1.135', '-0.202548059445']
LiPmSi 2 ['Li0.111111Si0.333333Pm0.555556', '-0.543', '-0.209241070833']
LiPrSe 1 ['Li0.111111Se0.555556Pr0.333333', '-1.921', '-0.201065897336']
LiPtPu 1 ['Li0.1Pt0.4Pu0.5', '-0.856', '-0.209762866']
LiPtS 7 ['Li0.375S0.375Pt0.25', '-1.232', '-0.207142883537']
LiPtSc 1 ['Li0.2Sc0.1Pt0.7', '-0.898', '-0.203961536833']
LiPtSe 1 ['Li0.5Se0.333333Pt0.166667', '-1.227', '-0.210976170576']
LiPtSm 2 ['Li0.222222Sm0.111111Pt0.666667', '-0.908', '-0.212447192685']
LiPtTa 2 ['Li0.111111Ta0.222222Pt0.666667', '-0.918', '-0.208656160833']
LiPtTh 14 ['Li0.1Pt0.6Th0.3', '-1.089', '-0.204714003833']

LiPtTm 2 ['Li0.125Tm0.125Pt0.75', '-0.934', '-0.208412681667']
LiPtU 3 ['Li0.222222Pt0.666667U0.111111', '-0.844', '-0.221540432407']
LiPtZr 1 ['Li0.222222Zr0.111111Pt0.666667', '-0.944', '-0.200046990185']
LiPuS 5 ['Li0.166667S0.5Pu0.333333', '-1.987', '-0.222162428408']
LiPuSe 4 ['Li0.166667Se0.5Pu0.333333', '-1.624', '-0.210265655']
LiRhS 19 ['Li0.142857S0.428571Rh0.428571', '-0.919', '-0.20461827203']
LiRhTa 2 ['Li0.166667Rh0.666667Ta0.166667', '-0.694', '-0.220655511667']
LiRhY 2 ['Li0.125Y0.5Rh0.375', '-0.871', '-0.210997043125']
LiRhZr 1 ['Li0.166667Zr0.166667Rh0.666667', '-0.722', '-0.201359073333']
LiSSe 1 ['Li0.375S0.25Se0.375', '-1.018', '-0.206065115498']
LiSSi 1 ['Li0.333333Si0.222222S0.444444', '-1.297', '-0.216504300856']
LiSSm 4 ['Li0.142857S0.428571Sm0.428571', '-2.076', '-0.215375592206']
LiSTa 6 ['Li0.166667S0.5Ta0.333333', '-1.394', '-0.215127388358']
LiSTc 5 ['Li0.111111S0.444444Tc0.444444', '-0.917', '-0.205609180439']
LiSTh 11 ['Li0.111111S0.444444Th0.444444', '-1.996', '-0.205027129881']
LiSTm 3 ['Li0.2S0.4Tm0.4', '-1.88', '-0.204253805393']
LiSXe 1 ['Li0.1S0.5Xe0.4', '-0.461', '-0.2444840308']
LiSY 7 ['Li0.3S0.4Y0.3', '-1.924', '-0.203956630143']
LiSZn 1 ['Li0.25S0.375Zn0.375', '-1.257', '-0.237708858495']
LiSZr 20 ['Li0.3S0.5Zr0.2', '-1.778', '-0.213277506491']
LiScSe 9 ['Li0.125Sc0.5Se0.375', '-1.437', '-0.202224322188']
LiScSi 1 ['Li0.1Si0.4Sc0.5', '-0.935', '-0.20834494075']
LiSeSi 3 ['Li0.375Si0.25Se0.375', '-1.046', '-0.203093250783']
LiSeSn 1 ['Li0.285714Se0.571429Sn0.142857', '-0.971', '-0.265191510451']
LiSeTb 3 ['Li0.25Se0.375Tb0.375', '-1.406', '-0.206397228836']
LiSeTc 9 ['Li0.1Se0.5Tc0.4', '-0.4', '-0.209185334']
LiSeTh 4 ['Li0.1Se0.6Th0.3', '-1.853', '-0.202725130498']
LiSeTi 1 ['Li0.222222Ti0.333333Se0.444444', '-1.407', '-0.209234590545']
LiSeTm 8 ['Li0.142857Se0.571429Tm0.285714', '-1.693', '-0.211480600003']
LiSeY 10 ['Li0.1Se0.5Y0.4', '-1.947', '-0.203198645']
LiSeZr 4 ['Li0.111111Se0.444444Zr0.444444', '-1.379', '-0.209322530988']
LuMgN 4 ['N0.444444Mg0.111111Lu0.444444', '-1.977', '-0.213840377627']
LuMgS 3 ['Mg0.222222S0.444444Lu0.333333', '-1.908', '-0.209159766038']
LuMnN 3 ['N0.4Mn0.4Lu0.2', '-1.209', '-0.201664407846']
LuMnO 1 ['O0.625Mn0.125Lu0.25', '-3.408', '-0.221600661049']
LuMnOs 1 ['Mn0.1Lu0.6Os0.3', '-0.39', '-0.209026110751']
LuMnSi 1 ['Si0.5Mn0.4Lu0.1', '-0.735', '-0.204345036982']
LuMoN 1 ['N0.4Mo0.1Lu0.5', '-1.842', '-0.255156339864']
LuMoO 1 ['O0.7Mo0.2Lu0.1', '-2.874', '-0.262587531512']
LuNNa 4 ['N0.333333Na0.166667Lu0.5', '-1.531', '-0.20863028322']
LuNNb 3 ['N0.375Nb0.125Lu0.5', '-1.721', '-0.233334068623']
LuNNi 4 ['N0.375Ni0.125Lu0.5', '-1.85', '-0.230006387685']
LuNNp 18 ['N0.4Lu0.3Np0.3', '-1.635', '-0.202375812488']
LuNO 2 ['N0.375O0.125Lu0.5', '-2.576', '-0.262993575595']

LuNOs 11 ['N0.125Lu0.625Os0.25', '-0.848', '-0.204208483707']
LuNP 30 ['N0.4P0.4Lu0.2', '-1.242', '-0.223103891529']
LuNPa 13 ['N0.5Lu0.333333Pa0.166667', '-1.896', '-0.244315552903']
LuNPb 1 ['N0.333333Lu0.555556Pb0.111111', '-1.665', '-0.205262010627']
LuNPd 13 ['N0.4Pd0.1Lu0.5', '-1.991', '-0.209676000364']
LuNPt 16 ['N0.111111Lu0.555556Pt0.333333', '-1.572', '-0.203743508571']
LuNPu 8 ['N0.428571Lu0.285714Pu0.285714', '-1.745', '-0.220362523426']
LuNRe 3 ['N0.4Lu0.5Re0.1', '-1.88', '-0.293156339864']
LuNRh 5 ['N0.333333Rh0.222222Lu0.444444', '-1.814', '-0.234066240442']
LuNRu 7 ['N0.111111Ru0.333333Lu0.555556', '-0.959', '-0.217626756073']
LuNS 3 ['N0.333333S0.111111Lu0.555556', '-1.973', '-0.202923756662']
LuNSb 1 ['N0.333333Sb0.111111Lu0.555556', '-1.841', '-0.275491899979']
LuNSc 8 ['N0.375Sc0.125Lu0.5', '-1.702', '-0.205074128622']
LuNSe 6 ['N0.3Se0.2Lu0.5', '-2.03', '-0.205331699898']
LuNSi 12 ['N0.444444Si0.222222Lu0.333333', '-1.773', '-0.21447520846']
LuNSn 1 ['N0.375Sn0.125Lu0.5', '-1.846', '-0.208102350683']
LuNTa 3 ['N0.428571Lu0.428571Ta0.142857', '-1.918', '-0.21781036414']
LuNTc 11 ['N0.444444Tc0.111111Lu0.444444', '-1.966', '-0.202840377627']
LuNTe 9 ['N0.4Te0.1Lu0.5', '-2.009', '-0.20504806003']
LuNTi 18 ['N0.375Ti0.125Lu0.5', '-1.726', '-0.222670287837']
LuNTl 7 ['N0.4Lu0.4Tl0.2', '-1.81', '-0.223156339864']
LuNU 9 ['N0.571429Lu0.142857U0.285714', '-1.854', '-0.206765431944']
LuNW 7 ['N0.5Lu0.4W0.1', '-1.834', '-0.21158896333']
LuNXe 1 ['N0.4Xe0.1Lu0.5', '-1.81', '-0.223156339864']
LuNZn 13 ['N0.333333Zn0.166667Lu0.5', '-1.645', '-0.204048922387']
LuNaS 4 ['Na0.111111S0.444444Lu0.444444', '-2.023', '-0.227839050898']
LuNaSe 5 ['Na0.1Se0.4Lu0.5', '-1.488', '-0.205512368166']
LuNaTe 1 ['Na0.111111Te0.555556Lu0.333333', '-1.301', '-0.259962201665']
LuNbS 4 ['S0.571429Nb0.285714Lu0.142857', '-1.807', '-0.209719559605']
LuNbSe 5 ['Se0.571429Nb0.142857Lu0.285714', '-1.753', '-0.206367905929']
LuNdO 5 ['O0.625Nd0.25Lu0.125', '-3.745', '-0.204665528356']
LuNiRu 2 ['Ni0.142857Ru0.285714Lu0.571429', '-0.622', '-0.213125503214']
LuNiSe 1 ['Ni0.333333Se0.444444Lu0.222222', '-1.335', '-0.224453061111']
LuNiSi 8 ['Si0.4Ni0.3Lu0.3', '-1.014', '-0.2025946665']
LuNpO 4 ['O0.666667Lu0.111111Np0.222222', '-3.789', '-0.214940235964']
LuNpP 1 ['P0.5Lu0.166667Np0.333333', '-1.438', '-0.20870127177']
LuNpSe 1 ['Se0.666667Lu0.111111Np0.222222', '-1.341', '-0.214604918427']
LuOPa 5 ['O0.7Lu0.2Pa0.1', '-3.12', '-0.209542763059']
LuOPr 1 ['O0.625Pr0.125Lu0.25', '-3.85', '-0.256456999112']
LuOS 8 ['O0.714286S0.142857Lu0.142857', '-2.471', '-0.252607690645']
LuOSc 1 ['O0.625Sc0.125Lu0.25', '-3.898', '-0.232169232628']
LuOSe 7 ['O0.7Se0.2Lu0.1', '-1.782', '-0.219726691516']
LuOSr 1 ['O0.6Sr0.1Lu0.3', '-3.798', '-0.20846079372']
LuOTa 1 ['O0.625Lu0.25Ta0.125', '-3.978', '-0.352926697697']

LuOTc 1 ['O0.666667Tc0.166667Lu0.166667', '-2.81', '-0.2050138333']
LuOTe 1 ['O0.7Te0.2Lu0.1', '-2.064', '-0.20424120872']
LuOTl 1 ['O0.6Lu0.3Tl0.1', '-3.431', '-0.225460864971']
LuOU 1 ['O0.666667Lu0.222222U0.111111', '-3.897', '-0.275363505519']
LuOsPd 3 ['Pd0.142857Lu0.571429Os0.285714', '-0.652', '-0.205139089286']
LuOsRh 3 ['Rh0.125Lu0.625Os0.25', '-0.617', '-0.201347505833']
LuOsRu 1 ['Ru0.125Lu0.625Os0.25', '-0.469', '-0.208378375625']
LuOsS 1 ['S0.375Lu0.5Os0.125', '-1.789', '-0.204039036618']
LuOsSe 4 ['Se0.444444Lu0.444444Os0.111111', '-1.665', '-0.201931473611']
LuPPd 1 ['P0.5Pd0.333333Lu0.166667', '-1.165', '-0.209876696187']
LuPPm 5 ['P0.4Pm0.5Lu0.1', '-1.258', '-0.200689011166']
LuPPt 1 ['P0.2Lu0.5Pt0.3', '-1.832', '-0.342985792208']
LuPRh 3 ['P0.375Rh0.375Lu0.25', '-1.426', '-0.205503314556']
LuPRu 2 ['P0.4Ru0.5Lu0.1', '-0.982', '-0.211929264416']
LuPS 9 ['P0.333333S0.333333Lu0.333333', '-1.876', '-0.200043525397']
LuPTa 1 ['P0.4Lu0.1Ta0.5', '-1.232', '-0.261888689291']
LuPTi 2 ['P0.5Ti0.125Lu0.375', '-1.825', '-0.210818681298']
LuPaS 4 ['S0.666667Lu0.222222Pa0.111111', '-1.948', '-0.246281638547']
LuPaSe 10 ['Se0.714286Lu0.142857Pa0.142857', '-1.224', '-0.216519782857']
LuPaTc 1 ['Tc0.5Lu0.25Pa0.25', '-0.28', '-0.20219241375']
LuPbS 1 ['S0.375Lu0.5Pb0.125', '-1.818', '-0.214266888868']
LuPdRu 2 ['Ru0.285714Pd0.142857Lu0.571429', '-0.736', '-0.200528082143']
LuPdSe 22 ['Se0.2Pd0.5Lu0.3', '-1.468', '-0.225383627833']
LuPdTm 1 ['Pd0.4Tm0.4Lu0.2', '-0.978', '-0.209531223']
LuPmS 19 ['S0.5Pm0.166667Lu0.333333', '-2.168', '-0.20143069924']
LuPmSe 14 ['Se0.5Pm0.166667Lu0.333333', '-1.804', '-0.206114007917']
LuPmTe 1 ['Te0.6Pm0.3Lu0.1', '-1.303', '-0.213573955582']
LuPtS 9 ['S0.428571Lu0.285714Pt0.285714', '-1.837', '-0.205813496811']
LuPtSe 8 ['Se0.222222Lu0.444444Pt0.333333', '-1.728', '-0.214110595278']
LuPtTh 2 ['Lu0.111111Pt0.777778Th0.111111', '-0.992', '-0.235056251111']
LuPtZr 1 ['Zr0.1Lu0.1Pt0.8', '-1.065', '-0.2200056115']
LuPuS 5 ['S0.375Lu0.375Pu0.25', '-1.723', '-0.211990472868']
LuPuTe 1 ['Te0.6Lu0.3Pu0.1', '-1.281', '-0.2301557005']
LuRbSe 1 ['Se0.571429Rb0.142857Lu0.285714', '-1.844', '-0.203277849286']
LuRbTe 3 ['Rb0.142857Te0.571429Lu0.285714', '-1.285', '-0.257147340596']
LuRhS 2 ['S0.5Rh0.3Lu0.2', '-1.551', '-0.201380640742']
LuRhSe 22 ['Se0.333333Rh0.166667Lu0.5', '-1.597', '-0.201520454167']
LuRhSi 8 ['Si0.444444Rh0.444444Lu0.111111', '-1.188', '-0.211803221111']
LuRhSn 1 ['Rh0.6Sn0.3Lu0.1', '-0.763', '-0.200470525444']
LuRuS 8 ['S0.375Ru0.125Lu0.5', '-1.832', '-0.208271720993']
LuRuSe 5 ['Se0.375Ru0.125Lu0.5', '-1.516', '-0.2135270825']
LuSSe 1 ['S0.2Se0.5Lu0.3', '-1.727', '-0.20442928703']
LuSSi 2 ['Si0.25S0.375Lu0.375', '-1.803', '-0.200459130681']
LuSSn 1 ['S0.4Sn0.2Lu0.4', '-1.883', '-0.21313282119']

LuSTa 2 ['S0.555556Lu0.222222Ta0.222222', '-1.914', '-0.215285859853']
LuSTc 5 ['S0.5Tc0.375Lu0.125', '-1.289', '-0.208928013149']
LuSTh 1 ['S0.4Lu0.3Th0.3', '-1.81', '-0.202518202892']
LuSTl 11 ['S0.4Lu0.3Tl0.3', '-1.732', '-0.206285759143']
LuSXe 1 ['S0.5Xe0.4Lu0.1', '-0.784', '-0.222610816523']
LuSZr 8 ['S0.444444Zr0.444444Lu0.111111', '-1.72', '-0.211604494046']
LuSbSe 2 ['Se0.3Sb0.2Lu0.5', '-1.577', '-0.2169114775']
LuScSe 4 ['Sc0.2Se0.6Lu0.2', '-1.799', '-0.232533137']
LuSeSi 2 ['Si0.125Se0.375Lu0.5', '-1.611', '-0.205888512917']
LuSeTc 3 ['Se0.571429Tc0.285714Lu0.142857', '-0.883', '-0.219527027143']
LuSeTe 3 ['Se0.1Te0.3Lu0.6', '-1.181', '-0.203438171713']
LuSeTh 1 ['Se0.6Lu0.2Th0.2', '-1.961', '-0.200488918999']
LuSeTl 13 ['Se0.5Lu0.333333Tl0.166667', '-1.759', '-0.210896396667']
LuSeTm 3 ['Se0.625Tm0.25Lu0.125', '-1.857', '-0.218650006252']
LuSeY 5 ['Se0.571429Y0.285714Lu0.142857', '-1.879', '-0.206440611429']
LuSeZn 3 ['Zn0.2Se0.5Lu0.3', '-1.694', '-0.2288372865']
LuSeZr 4 ['Se0.6Zr0.3Lu0.1', '-1.625', '-0.219648918958']
LuSrTe 2 ['Sr0.1Te0.6Lu0.3', '-1.389', '-0.2097675905']
LuTeTl 1 ['Te0.555556Lu0.333333Tl0.111111', '-1.17', '-0.247898209816']
LuTeTm 1 ['Te0.6Tm0.1Lu0.3', '-1.294', '-0.2312115455']
LuTeU 1 ['Te0.6Lu0.3U0.1', '-1.152', '-0.209954232']
LuTeY 1 ['Y0.3Te0.6Lu0.1', '-1.4', '-0.220560369']
LuTeZr 1 ['Zr0.111111Te0.555556Lu0.333333', '-1.241', '-0.218098449258']
MgMnN 20 ['N0.333333Mg0.222222Mn0.444444', '-0.723', '-0.205591736999']
MgMoN 1 ['N0.375Mg0.375Mo0.25', '-1.024', '-0.250204239598']
MgNNb 4 ['N0.5Mg0.166667Nb0.333333', '-1.305', '-0.244462072673']
MgNNi 1 ['N0.428571Mg0.142857Ni0.428571', '-0.447', '-0.229893591168']
MgNNp 27 ['N0.444444Mg0.444444Np0.111111', '-1.137', '-0.228885134443']
MgNO 8 ['N0.2O0.2Mg0.6', '-1.821', '-0.211513686548']
MgNP 18 ['N0.3Mg0.5P0.2', '-1.127', '-0.20508574781']
MgNPa 22 ['N0.428571Mg0.428571Pa0.142857', '-1.122', '-0.223860299095']
MgNPm 1 ['N0.444444Mg0.111111Pm0.444444', '-1.633', '-0.262742143182']
MgNPt 14 ['N0.25Mg0.375Pt0.375', '-0.879', '-0.219515246563']
MgNPu 18 ['N0.4Mg0.3Pu0.3', '-1.247', '-0.209972313176']
MgNRe 19 ['N0.4Mg0.2Re0.4', '-0.551', '-0.217104475659']
MgNRh 12 ['N0.5Mg0.125Rh0.375', '-0.381', '-0.201371269815']
MgNRu 10 ['N0.5Mg0.1Ru0.4', '-0.38', '-0.236297015852']
MgNSc 3 ['N0.5Mg0.125Sc0.375', '-1.906', '-0.210925518438']
MgNSm 1 ['N0.428571Mg0.285714Sm0.285714', '-1.497', '-0.282510724586']
MgNSr 1 ['N0.4Mg0.2Sr0.4', '-0.961', '-0.251219337779']
MgNTb 1 ['N0.444444Mg0.222222Tb0.333333', '-1.446', '-0.248984286414']
MgNTc 18 ['N0.6Mg0.1Tc0.3', '-0.432', '-0.209795925051']
MgNTh 5 ['N0.5Mg0.25Th0.25', '-1.61', '-0.241193097015']
MgNTi 13 ['N0.444444Mg0.444444Ti0.111111', '-1.133', '-0.206089665563']

MgNTm 4 ['N0.444444Mg0.111111Tm0.444444', '-1.928', '-0.213041002071']
MgNU 16 ['N0.428571Mg0.428571U0.142857', '-1.225', '-0.204604882175']
MgNV 12 ['N0.5Mg0.1V0.4', '-1.357', '-0.210972317716']
MgNW 18 ['N0.444444Mg0.111111W0.444444', '-0.51', '-0.218598993412']
MgNY 1 ['N0.428571Mg0.285714Y0.285714', '-1.573', '-0.2392234653']
MgNYb 9 ['N0.285714Mg0.142857Yb0.571429', '-0.974', '-0.203944820912']
MgNZr 4 ['N0.5Mg0.25Zr0.25', '-1.499', '-0.208858921391']
MgNaO 2 ['O0.333333Na0.166667Mg0.5', '-2.167', '-0.203037731653']
MgNaS 3 ['Na0.3Mg0.3S0.4', '-1.554', '-0.203151195849']
MgNdO 2 ['O0.625Mg0.125Nd0.25', '-3.319', '-0.240634002682']
MgNdS 17 ['Mg0.142857S0.428571Nd0.428571', '-2.065', '-0.202672116134']
MgNdSi 6 ['Mg0.285714Si0.428571Nd0.285714', '-0.729', '-0.204973350119']
MgNpO 4 ['O0.625Mg0.25Np0.125', '-3.092', '-0.216664951131']
MgNpS 10 ['Mg0.111111S0.555556Np0.333333', '-1.816', '-0.215731401334']
MgOOs 2 ['O0.666667Mg0.166667Os0.166667', '-2.046', '-0.246380487471']
MgOP 24 ['O0.5Mg0.4P0.1', '-2.981', '-0.201388638749']
MgOPa 6 ['O0.666667Mg0.222222Pa0.111111', '-2.639', '-0.277273805713']
MgOPu 5 ['O0.285714Mg0.285714Pu0.428571', '-1.979', '-0.203870417629']
MgORe 8 ['O0.666667Mg0.166667Re0.166667', '-2.456', '-0.206839107053']
MgOS 9 ['O0.25Mg0.5S0.25', '-2.467', '-0.204207327945']
MgOSi 6 ['O0.666667Mg0.222222Si0.111111', '-2.598', '-0.203480912853']
MgOSn 2 ['O0.625Mg0.25Sn0.125', '-2.417', '-0.200511271304']
MgOTc 1 ['O0.666667Mg0.111111Tc0.222222', '-2.196', '-0.268939003023']
MgOTe 1 ['O0.666667Mg0.111111Te0.222222', '-1.901', '-0.242550515894']
MgOTh 1 ['O0.666667Mg0.111111Th0.222222', '-3.765', '-0.275994548677']
MgOTi 1 ['O0.6Mg0.2Ti0.2', '-3.443', '-0.25480094688']
MgOU 1 ['O0.666667Mg0.111111U0.222222', '-3.615', '-0.27674691867']
MgOsSc 2 ['Mg0.125Sc0.625Os0.25', '-0.455', '-0.202197690729']
MgOsSi 2 ['Mg0.111111Si0.444444Os0.444444', '-0.563', '-0.228372205741']
MgPPd 12 ['Mg0.428571P0.142857Pd0.428571', '-0.928', '-0.202517501711']
MgPPm 12 ['Mg0.2P0.3Pm0.5', '-0.961', '-0.201441396229']
MgPPt 12 ['Mg0.375P0.375Pt0.25', '-1.038', '-0.205958569452']
MgPRh 3 ['Mg0.5P0.125Rh0.375', '-0.805', '-0.216097840547']
MgPRu 10 ['Mg0.25P0.375Ru0.375', '-0.824', '-0.20036080289']
MgPSm 2 ['Mg0.142857P0.428571Sm0.428571', '-1.663', '-0.205941251517']
MgPTa 10 ['Mg0.2P0.5Ta0.3', '-0.919', '-0.208150026299']
MgPTc 1 ['Mg0.166667P0.5Tc0.333333', '-0.88', '-0.200735854905']
MgPY 1 ['Mg0.1P0.4Y0.5', '-1.723', '-0.297911003999']
MgPYb 9 ['Mg0.1P0.4Yb0.5', '-1.024', '-0.202301016124']
MgPZr 7 ['Mg0.111111P0.444444Zr0.444444', '-1.563', '-0.224196282684']
MgPaRu 3 ['Mg0.125Ru0.625Pa0.25', '-0.535', '-0.21357480625']
MgPaS 7 ['Mg0.25S0.625Pa0.125', '-1.517', '-0.216218831264']
MgPaSe 8 ['Mg0.285714Se0.571429Pa0.142857', '-1.269', '-0.200536965954']
MgPdPm 1 ['Mg0.1Pd0.7Pm0.2', '-0.737', '-0.225034987583']

MgPmS 34 ['Mg0.5S0.333333Pm0.166667', '-1.381', '-0.200716990814']
MgPmSe 23 ['Mg0.333333Se0.444444Pm0.222222', '-1.486', '-0.202098740742']
MgPmSi 1 ['Mg0.125Si0.375Pm0.5', '-0.57', '-0.203182841042']
MgPrS 18 ['Mg0.142857S0.428571Pr0.428571', '-2.068', '-0.209589600777']
MgPrSe 7 ['Mg0.222222Se0.444444Pr0.333333', '-1.716', '-0.201949270371']
MgPrSi 7 ['Mg0.333333Si0.333333Pr0.333333', '-0.751', '-0.254163595833']
MgPtS 15 ['Mg0.333333S0.333333Pt0.333333', '-1.283', '-0.201855138523']
MgPtTh 4 ['Mg0.1Pt0.7Th0.2', '-0.947', '-0.220394920083']
MgPuS 23 ['Mg0.444444S0.444444Pu0.111111', '-1.697', '-0.217510406271']
MgPuSn 2 ['Mg0.3Sn0.3Pu0.4', '-0.448', '-0.205251185945']
MgReS 2 ['Mg0.222222S0.555556Re0.222222', '-1.308', '-0.231551410386']
MgRhS 17 ['Mg0.5S0.25Rh0.25', '-1.23', '-0.201949541914']
MgRhSi 6 ['Mg0.111111Si0.555556Rh0.333333', '-0.793', '-0.20869096287']
MgRhTa 2 ['Mg0.1Rh0.7Ta0.2', '-0.79', '-0.204368007083']
MgRhV 1 ['Mg0.111111V0.222222Rh0.666667', '-0.601', '-0.202508159537']
MgRuS 1 ['Mg0.142857S0.571429Ru0.285714', '-1.141', '-0.215059837112']
MgSSc 1 ['Mg0.222222S0.444444Sc0.333333', '-1.907', '-0.210889047751']
MgSSe 4 ['Mg0.2S0.4Se0.4', '-0.853', '-0.221143223364']
MgSSi 5 ['Mg0.3Si0.2S0.5', '-1.427', '-0.220896257743']
MgSSm 18 ['Mg0.2S0.5Sm0.3', '-2.155', '-0.220706137908']
MgSTb 8 ['Mg0.142857S0.428571Tb0.428571', '-1.768', '-0.203184460566']
MgSTc 9 ['Mg0.142857S0.571429Tc0.285714', '-1.183', '-0.212813606397']
MgSTh 20 ['Mg0.125S0.5Th0.375', '-2.091', '-0.201061356845']
MgSTi 1 ['Mg0.222222S0.666667Ti0.111111', '-1.535', '-0.309563408644']
MgSTm 10 ['Mg0.444444S0.444444Tm0.111111', '-1.715', '-0.201640832938']
MgSW 1 ['Mg0.142857S0.571429W0.285714', '-1.376', '-0.278739319251']
MgSY 13 ['Mg0.444444S0.444444Y0.111111', '-1.736', '-0.207129611271']
MgSZr 27 ['Mg0.375S0.5Zr0.125', '-1.761', '-0.203917068305']
MgSeSm 2 ['Mg0.222222Se0.444444Sm0.333333', '-1.698', '-0.202361887038']
MgSeTc 5 ['Mg0.111111Se0.666667Tc0.222222', '-0.492', '-0.210267192871']
MgSeY 8 ['Mg0.333333Se0.5Y0.166667', '-1.636', '-0.202167836113']
MgSeZr 2 ['Mg0.111111Se0.555556Zr0.333333', '-1.431', '-0.200136877254']
MnMoN 18 ['N0.444444Mn0.333333Mo0.222222', '-0.627', '-0.202293599694']
MnMoP 1 ['P0.375Mn0.25Mo0.375', '-0.825', '-0.205367396317']
MnNNa 26 ['N0.555556Na0.222222Mn0.222222', '-0.489', '-0.200084483747']
MnNNb 2 ['N0.4Mn0.4Nb0.2', '-0.906', '-0.221030998846']
MnNNd 13 ['N0.375Mn0.5Nd0.125', '-0.847', '-0.21363279985']
MnNNp 13 ['N0.6Mn0.2Np0.2', '-0.813', '-0.206722707353']
MnNP 11 ['N0.375P0.25Mn0.375', '-0.708', '-0.205715799093']
MnNPa 28 ['N0.4Mn0.2Pa0.4', '-1.112', '-0.209908590602']
MnNPm 3 ['N0.444444Mn0.333333Pm0.222222', '-1.082', '-0.210644310528']
MnNPr 13 ['N0.375Mn0.5Pr0.125', '-0.819', '-0.203345990787']
MnNPt 4 ['N0.3Mn0.4Pt0.3', '-0.538', '-0.21248058788']
MnNPu 8 ['N0.5Mn0.2Pu0.3', '-1.217', '-0.274232381571']

MnNRb 18 ['N0.5Mn0.25Rb0.25', '-0.516', '-0.236564086692']
MnNRh 1 ['N0.4Mn0.3Rh0.3', '-0.387', '-0.205058388259']
MnNSc 18 ['N0.4Sc0.1Mn0.5', '-0.892', '-0.213320166966']
MnNSm 10 ['N0.5Mn0.3Sm0.2', '-1.027', '-0.210470371691']
MnNSn 9 ['N0.5Mn0.4Sn0.1', '-0.443', '-0.200411184346']
MnNSr 20 ['N0.571429Mn0.285714Sr0.142857', '-0.655', '-0.206908730125']
MnNTb 12 ['N0.444444Mn0.333333Tb0.222222', '-1.036', '-0.211432745599']
MnNTc 10 ['N0.5Mn0.2Tc0.3', '-0.401', '-0.201204501371']
MnNTh 7 ['N0.5Mn0.25Th0.25', '-1.372', '-0.208807508883']
MnNTi 17 ['N0.375Ti0.375Mn0.25', '-1.309', '-0.20252628219']
MnNTm 8 ['N0.4Mn0.4Tm0.2', '-1.186', '-0.200354688846']
MnNXe 1 ['N0.375Mn0.375Xe0.25', '-0.443', '-0.215572985324']
MnNY 12 ['N0.5Mn0.25Y0.25', '-1.28', '-0.230770617631']
MnNYb 21 ['N0.4Mn0.5Yb0.1', '-0.661', '-0.207095398049']
MnNZn 29 ['N0.5Mn0.166667Zn0.333333', '-0.302', '-0.200921326811']
MnNZr 2 ['N0.428571Mn0.428571Zr0.142857', '-0.967', '-0.202459490489']
MnNaP 3 ['Na0.166667P0.333333Mn0.5', '-0.798', '-0.208684772475']
MnNbO 1 ['O0.6Mn0.2Nb0.2', '-2.735', '-0.226679036278']
MnNbP 2 ['P0.222222Mn0.444444Nb0.333333', '-0.856', '-0.207371756323']
MnNdO 1 ['O0.6Mn0.2Nd0.2', '-3.098', '-0.21193810053']
MnNdSi 5 ['Si0.5Mn0.3Nd0.2', '-0.787', '-0.201983849112']
MnOPa 4 ['O0.625Mn0.25Pa0.125', '-2.72', '-0.297140309836']
MnOPr 1 ['O0.625Mn0.125Pr0.25', '-3.152', '-0.211284543301']
MnOSn 1 ['O0.6Mn0.2Sn0.2', '-2.255', '-0.22867472466']
MnOTa 2 ['O0.6Mn0.2Ta0.2', '-2.906', '-0.235950749504']
MnOTc 1 ['O0.625Mn0.125Tc0.25', '-1.989', '-0.20446721542']
MnOTe 1 ['O0.666667Mn0.166667Te0.166667', '-1.767', '-0.22196477203']
MnOTi 2 ['O0.625Ti0.25Mn0.125', '-3.19', '-0.200585115725']
MnOW 1 ['O0.666667Mn0.111111W0.222222', '-2.563', '-0.222773831943']
MnOsSc 3 ['Sc0.625Mn0.125Os0.25', '-0.389', '-0.203697217963']
MnOsTm 1 ['Mn0.1Tm0.6Os0.3', '-0.293', '-0.210918002287']
MnPPm 5 ['P0.375Mn0.125Pm0.5', '-1.102', '-0.208173808203']
MnPRh 1 ['P0.8Mn0.1Rh0.1', '-0.88', '-0.291000825099']
MnPRu 2 ['P0.333333Mn0.166667Ru0.5', '-0.731', '-0.217459081173']
MnPTa 10 ['P0.4Mn0.1Ta0.5', '-1.006', '-0.213218717358']
MnPTc 13 ['P0.375Mn0.375Tc0.25', '-0.815', '-0.221776999176']
MnPTi 18 ['P0.4Ti0.2Mn0.4', '-1.094', '-0.206549455143']
MnPV 3 ['P0.25V0.375Mn0.375', '-0.819', '-0.20146215915']
MnPaS 3 ['S0.7Mn0.1Pa0.2', '-1.227', '-0.232976353314']
MnPaSe 11 ['Mn0.222222Se0.666667Pa0.111111', '-0.689', '-0.214493034157']
MnPdPm 3 ['Mn0.142857Pd0.714286Pm0.142857', '-0.555', '-0.20379825553']
MnPmS 5 ['S0.5Mn0.1Pm0.4', '-2.065', '-0.216449111991']
MnPmSe 15 ['Mn0.333333Se0.5Pm0.166667', '-0.983', '-0.200496058319']
MnPrSi 6 ['Si0.6Mn0.3Pr0.1', '-0.691', '-0.232488125362']

MnPtS 18 ['S0.5Mn0.166667Pt0.333333', '-0.799', '-0.20118371875']
MnPtSe 1 ['Mn0.375Se0.375Pt0.25', '-0.574', '-0.208986566077']
MnPtTh 2 ['Mn0.1Pt0.8Th0.1', '-0.61', '-0.222156278121']
MnReSi 3 ['Si0.428571Mn0.142857Re0.428571', '-0.517', '-0.201037685427']
MnSSe 1 ['S0.333333Mn0.166667Se0.5', '-0.497', '-0.202854450031']
MnSTc 7 ['S0.5Mn0.2Tc0.3', '-0.841', '-0.222181433235']
MnSTi 8 ['S0.444444Ti0.333333Mn0.222222', '-1.404', '-0.202893912088']
MnSXe 6 ['S0.555556Mn0.111111Xe0.333333', '-0.403', '-0.206902966688']
MnSZr 2 ['S0.5Mn0.1Zr0.4', '-1.613', '-0.20853379499']
MnScSe 1 ['Sc0.375Mn0.125Se0.5', '-1.659', '-0.270524041015']
MnSeTc 4 ['Mn0.142857Se0.571429Tc0.285714', '-0.367', '-0.233963429815']
MnSeTe 11 ['Mn0.3Se0.2Te0.5', '-0.393', '-0.210248580945']
MnSeTm 11 ['Mn0.1Se0.6Tm0.3', '-1.563', '-0.201776925003']
MnSeY 10 ['Mn0.2Se0.6Y0.2', '-1.104', '-0.211388310742']
MnSeZr 3 ['Mn0.125Se0.5Zr0.375', '-1.283', '-0.214928259804']
MnSiSm 6 ['Si0.571429Mn0.285714Sm0.142857', '-0.718', '-0.22488091463']
MnSiTh 1 ['Si0.5Mn0.4Th0.1', '-0.795', '-0.269855934733']
MnSiTm 4 ['Si0.428571Mn0.428571Tm0.142857', '-0.737', '-0.203766267481']
MnSiYb 1 ['Si0.555556Mn0.333333Yb0.111111', '-0.653', '-0.217362207277']
MnSiZr 1 ['Si0.444444Mn0.333333Zr0.222222', '-0.884', '-0.239378764569']
MoNNa 7 ['N0.5Na0.333333Mo0.166667', '-0.689', '-0.215356941033']
MoNNb 2 ['N0.375Nb0.125Mo0.5', '-0.767', '-0.237164359872']
MoNNi 14 ['N0.3Ni0.2Mo0.5', '-0.565', '-0.201201650397']
MoNNp 10 ['N0.6Mo0.2Np0.2', '-0.897', '-0.223676222611']
MoNO 13 ['N0.333333O0.333333Mo0.333333', '-1.447', '-0.212655772568']
MoNPa 16 ['N0.444444Mo0.333333Pa0.222222', '-0.925', '-0.206890148365']
MoNPt 6 ['N0.444444Mo0.333333Pt0.222222', '-0.544', '-0.213175839368']
MoNPu 10 ['N0.6Mo0.2Pu0.2', '-0.945', '-0.209010300363']
MoNRb 8 ['N0.555556Rb0.111111Mo0.333333', '-0.635', '-0.234734179346']
MoNRh 1 ['N0.5Mo0.166667Rh0.333333', '-0.376', '-0.211819193175']
MoNSc 13 ['N0.428571Sc0.142857Mo0.428571', '-1.047', '-0.200628914853']
MoNSi 3 ['N0.4Si0.1Mo0.5', '-0.798', '-0.263492720197']
MoNSn 1 ['N0.375Mo0.5Sn0.125', '-0.573', '-0.21108457089']
MoNTa 4 ['N0.428571Mo0.428571Ta0.142857', '-0.857', '-0.221724088425']
MoNTb 7 ['N0.428571Mo0.285714Tb0.285714', '-1.165', '-0.209747299588']
MoNTc 4 ['N0.333333Mo0.555556Tc0.111111', '-0.522', '-0.208098512385']
MoNTi 22 ['N0.333333Ti0.444444Mo0.222222', '-1.388', '-0.200200437369']
MoNV 9 ['N0.3V0.1Mo0.6', '-0.642', '-0.203077932897']
MoNW 9 ['N0.444444Mo0.333333W0.222222', '-0.558', '-0.204579205125']
MoNYb 11 ['N0.571429Mo0.285714Yb0.142857', '-0.751', '-0.232563781535']
MoNZn 38 ['N0.3Zn0.1Mo0.6', '-0.489', '-0.20228058548']
MoNaP 1 ['Na0.1P0.5Mo0.4', '-0.95', '-0.211479241185']
MoNaS 2 ['Na0.111111S0.555556Mo0.333333', '-1.211', '-0.211848300317']
MoNaSe 1 ['Na0.25Se0.5Mo0.25', '-1.038', '-0.21871941461']

MoNdO 3 ['O0.666667Mo0.222222Nd0.111111', '-2.863', '-0.21708088997']
MoNdSe 2 ['Se0.571429Mo0.285714Nd0.142857', '-1.209', '-0.205207194523']
MoNiSe 1 ['Ni0.4Se0.4Mo0.2', '-0.64', '-0.237063718']
MoOPa 3 ['O0.666667Mo0.111111Pa0.222222', '-2.996', '-0.211870891637']
MoOPm 1 ['O0.666667Mo0.222222Pm0.111111', '-2.841', '-0.261657061429']
MoOPr 3 ['O0.666667Mo0.166667Pr0.166667', '-2.991', '-0.241282433686']
MoOS 11 ['O0.571429S0.142857Mo0.285714', '-2.156', '-0.232169539642']
MoOSe 2 ['O0.7Se0.1Mo0.2', '-2.091', '-0.217652711763']
MoOSm 1 ['O0.666667Mo0.222222Sm0.111111', '-2.961', '-0.328066667332']
MoOSn 1 ['O0.666667Mo0.222222Sn0.111111', '-2.337', '-0.249313641987']
MoOY 1 ['O0.7Y0.1Mo0.2', '-2.855', '-0.23288560301']
MoOYb 2 ['O0.666667Mo0.222222Yb0.111111', '-2.76', '-0.266255331359']
MoPPm 2 ['P0.5Mo0.1Pm0.4', '-1.313', '-0.20333907152']
MoPSb 7 ['P0.444444Mo0.444444Sb0.111111', '-0.895', '-0.200571893796']
MoPSi 6 ['Si0.222222P0.333333Mo0.444444', '-0.897', '-0.210628375902']
MoPSn 18 ['P0.6Mo0.2Sn0.2', '-0.7', '-0.208354222129']
MoPTa 16 ['P0.375Mo0.125Ta0.5', '-1.024', '-0.211663926015']
MoPTe 14 ['P0.444444Mo0.222222Te0.333333', '-0.662', '-0.205288653795']
MoPTi 18 ['P0.444444Ti0.333333Mo0.222222', '-1.265', '-0.204873020313']
MoPTl 1 ['P0.428571Mo0.428571Tl0.142857', '-0.883', '-0.213372897589']
MoPY 1 ['P0.555556Y0.111111Mo0.333333', '-1.166', '-0.200662614467']
MoPZn 4 ['P0.428571Zn0.142857Mo0.428571', '-0.872', '-0.202372897589']
MoPZr 4 ['P0.555556Zr0.333333Mo0.111111', '-1.434', '-0.201541538911']
MoPaSe 6 ['Se0.7Mo0.1Pa0.2', '-0.886', '-0.202921717']
MoPaSi 3 ['Si0.444444Mo0.444444Pa0.111111', '-0.606', '-0.213745666825']
MoPdPm 2 ['Mo0.1Pd0.8Pm0.1', '-0.356', '-0.20017499275']
MoPmS 8 ['S0.4Mo0.2Pm0.4', '-1.697', '-0.200720669392']
MoPmSe 20 ['Se0.666667Mo0.166667Pm0.166667', '-1.105', '-0.22889303125']
MoPrSe 5 ['Se0.6Mo0.2Pr0.2', '-1.408', '-0.200241422165']
MoPtTh 1 ['Mo0.1Pt0.8Th0.1', '-0.591', '-0.2373332265']
MoPuS 10 ['S0.555556Mo0.111111Pu0.333333', '-1.835', '-0.204097940547']
MoPuSe 11 ['Se0.5Mo0.2Pu0.3', '-1.33', '-0.200951254']
MoRbSe 10 ['Se0.625Rb0.125Mo0.25', '-0.941', '-0.2020785775']
MoSSm 1 ['S0.5Mo0.333333Sm0.166667', '-1.572', '-0.238872670687']
MoSTh 1 ['S0.6Mo0.2Th0.2', '-1.836', '-0.21002704359']
MoSTi 2 ['S0.5Ti0.333333Mo0.166667', '-1.545', '-0.222115196315']
MoSZr 5 ['S0.444444Zr0.333333Mo0.222222', '-1.436', '-0.206461083769']
MoScSe 11 ['Sc0.444444Se0.444444Mo0.111111', '-1.628', '-0.211100664444']
MoSeSm 1 ['Se0.555556Mo0.222222Sm0.222222', '-1.492', '-0.213698481111']
MoSeSr 2 ['Se0.555556Sr0.111111Mo0.333333', '-1.116', '-0.207718554444']
MoSeTe 2 ['Se0.25Mo0.5Te0.25', '-0.578', '-0.216999695417']
MoSeTm 2 ['Se0.555556Mo0.111111Tm0.333333', '-1.612', '-0.235603552224']
MoSeY 7 ['Se0.555556Y0.333333Mo0.111111', '-1.602', '-0.200879025']
MoSeZn 4 ['Zn0.222222Se0.444444Mo0.333333', '-0.749', '-0.205726641111']

MoSiY 1 ['Si0.4Y0.2Mo0.4', '-0.881', '-0.274895038']
NNaNi 20 ['N0.5Na0.333333Ni0.166667', '-0.393', '-0.201666145519']
NNaNp 13 ['N0.428571Na0.285714Np0.285714', '-0.966', '-0.218500491834']
NNaOs 20 ['N0.5Na0.2Os0.3', '-0.422', '-0.230666145519']
NNaP 3 ['N0.333333Na0.444444P0.222222', '-0.87', '-0.206616528999']
NNaPa 25 ['N0.4Na0.2Pa0.4', '-1.072', '-0.230622286045']
NNaPm 1 ['N0.4Na0.1Pm0.5', '-1.443', '-0.209767928864']
NNaPr 1 ['N0.4Na0.1Pr0.5', '-1.33', '-0.215562218864']
NNaPt 22 ['N0.625Na0.25Pt0.125', '-0.493', '-0.201183001469']
NNaPu 10 ['N0.444444Na0.333333Pu0.222222', '-0.916', '-0.222464056822']
NNaRe 19 ['N0.428571Na0.285714Re0.285714', '-0.523', '-0.245001912364']
NNaRh 16 ['N0.571429Na0.142857Rh0.285714', '-0.378', '-0.213999553302']
NNaRu 26 ['N0.6Na0.1Ru0.3', '-0.318', '-0.203199687312']
NNaSb 2 ['N0.555556Na0.222222Sb0.222222', '-0.444', '-0.202618298694']
NNaSc 1 ['N0.5Na0.125Sc0.375', '-1.766', '-0.202720785002']
NNaSi 1 ['N0.4Na0.4Si0.2', '-0.957', '-0.227134498918']
NNaSm 1 ['N0.375Na0.125Sm0.5', '-1.448', '-0.258147468935']
NNaTa 1 ['N0.428571Na0.142857Ta0.428571', '-1.313', '-0.21434751914']
NNaTc 28 ['N0.5Na0.125Tc0.375', '-0.434', '-0.204985560347']
NNaTh 11 ['N0.444444Na0.111111Th0.444444', '-1.707', '-0.217723806516']
NNaTi 20 ['N0.375Na0.5Ti0.125', '-0.639', '-0.203129379384']
NNaTm 2 ['N0.375Na0.125Tm0.5', '-1.66', '-0.213003345497']
NNaU 11 ['N0.5Na0.333333U0.166667', '-1.013', '-0.201626280891']
NNaV 1 ['N0.5Na0.25V0.25', '-0.959', '-0.236880136425']
NNaW 17 ['N0.5Na0.1W0.4', '-0.478', '-0.206742391909']
NNaXe 1 ['N0.625Na0.25Xe0.125', '-0.442', '-0.202832681899']
NNaY 2 ['N0.375Na0.125Y0.5', '-1.555', '-0.208582941123']
NNaYb 1 ['N0.333333Na0.111111Yb0.555556', '-1.078', '-0.205177698217']
NNaZn 4 ['N0.3Na0.3Zn0.4', '-0.375', '-0.218181006641']
NNaZr 1 ['N0.5Na0.125Zr0.375', '-1.628', '-0.231674572641']
NNbNp 5 ['N0.6Nb0.2Np0.2', '-1.246', '-0.264084436296']
NNbO 4 ['N0.4O0.2Nb0.4', '-1.835', '-0.242911482827']
NNbP 21 ['N0.5P0.3Nb0.2', '-0.982', '-0.204228822534']
NNbPa 13 ['N0.571429Nb0.285714Pa0.142857', '-1.228', '-0.235826404179']
NNbPu 11 ['N0.5Nb0.25Pu0.25', '-1.487', '-0.21361994233']
NNbSc 7 ['N0.5Sc0.333333Nb0.166667', '-1.943', '-0.20339107733']
NNbSi 3 ['N0.375Si0.125Nb0.5', '-1.29', '-0.239033424178']
NNbTb 4 ['N0.5Nb0.125Tb0.375', '-1.572', '-0.200390893075']
NNbTi 5 ['N0.5Ti0.3Nb0.2', '-1.478', '-0.202845227911']
NNbU 1 ['N0.571429Nb0.142857U0.285714', '-1.657', '-0.240027282659']
NNbYb 14 ['N0.571429Nb0.142857Yb0.285714', '-1.062', '-0.208292485492']
NNbZn 13 ['N0.4Zn0.4Nb0.2', '-0.697', '-0.200067321116']
NNdNp 10 ['N0.5Nd0.3Np0.2', '-1.571', '-0.207677121078']
NNdO 2 ['N0.222222O0.666667Nd0.111111', '-1.582', '-0.203437557672']

NNdP 20 ['N0.5P0.333333Nd0.166667', '-1.098', '-0.227296433092']
NNdPa 14 ['N0.5Nd0.375Pa0.125', '-1.545', '-0.200088959635']
NNdPu 3 ['N0.571429Nd0.142857Pu0.285714', '-1.476', '-0.275387421283']
NNdSi 7 ['N0.5Si0.25Nd0.25', '-1.545', '-0.255632370009']
NNdTe 5 ['N0.25Te0.375Nd0.375', '-1.543', '-0.210327920327']
NNdTi 5 ['N0.5Ti0.25Nd0.25', '-1.733', '-0.352531593911']
NNdW 3 ['N0.555556Nd0.111111W0.333333', '-0.655', '-0.207778309809']
NNdZn 3 ['N0.444444Zn0.333333Nd0.222222', '-0.854', '-0.203377782147']
NNiNp 26 ['N0.375Ni0.375Np0.25', '-0.826', '-0.209432016432']
NNiP 7 ['N0.5P0.25Ni0.25', '-0.612', '-0.207704082039']
NNiPa 39 ['N0.375Ni0.125Pa0.5', '-1.193', '-0.201609349869']
NNiPu 27 ['N0.444444Ni0.444444Pu0.111111', '-0.537', '-0.220496585652']
NNiRb 1 ['N0.4Ni0.4Rb0.2', '-0.377', '-0.221544200529']
NNiSc 9 ['N0.4Sc0.3Ni0.3', '-1.421', '-0.200371896864']
NNiSr 3 ['N0.428571Ni0.428571Sr0.142857', '-0.505', '-0.202825778781']
NNiTe 1 ['N0.142857Ni0.428571Te0.428571', '-0.377', '-0.2029025575']
NNiTi 25 ['N0.5Ti0.2Ni0.3', '-0.739', '-0.211913769162']
NNiTm 5 ['N0.428571Ni0.285714Tm0.285714', '-1.312', '-0.201648737489']
NNiU 5 ['N0.5Ni0.1U0.4', '-1.541', '-0.204634428126']
NNiYb 22 ['N0.5Ni0.333333Yb0.166667', '-0.511', '-0.210868674923']
NNiZn 8 ['N0.333333Ni0.333333Zn0.333333', '-0.38', '-0.205284626667']
NNiZr 1 ['N0.444444Ni0.333333Zr0.222222', '-1.05', '-0.213357337083']
NNpO 25 ['N0.25O0.25Np0.5', '-2.265', '-0.208431951133']
NNpOs 14 ['N0.666667Os0.166667Np0.166667', '-0.611', '-0.206847595983']
NNpP 39 ['N0.2P0.4Np0.4', '-1.162', '-0.233035318972']
NNpPa 23 ['N0.5Pa0.125Np0.375', '-1.384', '-0.227671043225']
NNpPb 14 ['N0.5Pb0.333333Np0.166667', '-0.609', '-0.204847595983']
NNpPd 15 ['N0.5Pd0.3Np0.2', '-0.711', '-0.22601711518']
NNpPm 3 ['N0.571429Pm0.142857Np0.285714', '-1.416', '-0.282727281994']
NNpPr 9 ['N0.5Pr0.125Np0.375', '-1.46', '-0.202395284358']
NNpPt 44 ['N0.428571Pt0.428571Np0.142857', '-0.556', '-0.2095836537']
NNpPu 18 ['N0.428571Np0.285714Pu0.285714', '-1.339', '-0.210228215031']
NNpRb 8 ['N0.6Rb0.1Np0.3', '-1.157', '-0.312933823167']
NNpRe 18 ['N0.625Re0.25Np0.125', '-0.535', '-0.202829724459']
NNpRh 28 ['N0.428571Rh0.142857Np0.428571', '-1.243', '-0.2037509611']
NNpRu 26 ['N0.333333Ru0.222222Np0.444444', '-1.038', '-0.201323796504']
NNpS 15 ['N0.285714S0.285714Np0.428571', '-1.583', '-0.222198981686']
NNpSb 8 ['N0.5Sb0.2Np0.3', '-0.999', '-0.27152567277']
NNpSc 21 ['N0.428571Sc0.142857Np0.428571', '-1.484', '-0.213854640209']
NNpSe 13 ['N0.571429Se0.142857Np0.285714', '-0.936', '-0.208698589045']
NNpSi 24 ['N0.625Si0.25Np0.125', '-1.216', '-0.204420472708']
NNpSm 5 ['N0.555556Sm0.222222Np0.222222', '-1.517', '-0.273032331791']
NNpSn 12 ['N0.555556Sn0.222222Np0.222222', '-0.767', '-0.228130127978']
NNpSr 26 ['N0.444444Sr0.111111Np0.444444', '-1.287', '-0.209260255956']

NNpTa 4 ['N0.6Ta0.2Np0.2', '-1.331', '-0.216334196065']
NNpTb 19 ['N0.5Tb0.125Np0.375', '-1.493', '-0.22459056346']
NNpTc 26 ['N0.666667Tc0.111111Np0.222222', '-0.775', '-0.207055649903']
NNpTe 24 ['N0.3Te0.4Np0.3', '-0.936', '-0.20852567277']
NNpTh 18 ['N0.444444Th0.111111Np0.444444', '-1.394', '-0.213376143596']
NNpTi 17 ['N0.5Ti0.166667Np0.333333', '-1.466', '-0.225349584631']
NNpTl 28 ['N0.222222Tl0.333333Np0.444444', '-0.743', '-0.204130127978']
NNpTm 14 ['N0.428571Tm0.285714Np0.285714', '-1.7', '-0.251110012174']
NNpU 2 ['N0.6U0.1Np0.3', '-1.46', '-0.283968212201']
NNpV 14 ['N0.625V0.25Np0.125', '-1.151', '-0.221432760653']
NNpW 20 ['N0.5W0.333333Np0.166667', '-0.729', '-0.206289674202']
NNpXe 9 ['N0.428571Xe0.285714Np0.285714', '-0.9', '-0.2071673074']
NNpY 13 ['N0.444444Y0.111111Np0.444444', '-1.441', '-0.233756804151']
NNpYb 27 ['N0.5Yb0.1Np0.4', '-1.362', '-0.217469770003']
NNpZn 38 ['N0.285714Zn0.428571Np0.285714', '-0.893', '-0.2001673074']
NNpZr 4 ['N0.571429Zr0.142857Np0.285714', '-1.435', '-0.210233811263']
NOOs 4 ['N0.428571O0.285714Os0.285714', '-0.705', '-0.20724541689']
NOP 1 ['N0.2O0.7P0.1', '-1.31', '-0.200019583464']
NOPa 32 ['N0.125O0.625Pa0.25', '-2.63', '-0.210914197241']
NOPm 4 ['N0.222222O0.333333Pm0.444444', '-2.937', '-0.209295000047']
NOPr 8 ['N0.1O0.4Pr0.5', '-2.871', '-0.208344641529']
NOPu 26 ['N0.285714O0.285714Pu0.428571', '-2.585', '-0.203086241348']
NORe 3 ['N0.125O0.625Re0.25', '-1.794', '-0.203107832889']
NOSm 2 ['N0.1O0.4Sm0.5', '-2.986', '-0.201352168529']
NOSn 2 ['N0.2O0.4Sn0.4', '-1.459', '-0.229337568912']
NOSr 4 ['N0.111111O0.333333Sr0.555556', '-2.389', '-0.219741734113']
NOTb 7 ['N0.2O0.3Tb0.5', '-2.567', '-0.212359791176']
NOTh 16 ['N0.25O0.25Th0.5', '-2.632', '-0.205439550165']
NOTi 41 ['N0.142857O0.285714Ti0.571429', '-2.37', '-0.200174702539']
NOTl 3 ['N0.142857O0.428571Tl0.428571', '-0.997', '-0.255858901452']
NOTm 13 ['N0.125O0.375Tm0.5', '-3.151', '-0.211264779314']
NOU 20 ['N0.4O0.1U0.5', '-1.906', '-0.200434660775']
NOV 4 ['N0.375O0.25V0.375', '-1.837', '-0.218924636538']
NOW 8 ['N0.5O0.2W0.3', '-0.944', '-0.206456925272']
NOY 6 ['N0.222222O0.666667Y0.111111', '-1.609', '-0.213441411608']
NOYb 4 ['N0.2O0.2Yb0.6', '-2.181', '-0.253982891754']
NOsPa 17 ['N0.666667Os0.166667Pa0.166667', '-0.539', '-0.209685269683']
NOsPu 7 ['N0.5Os0.1Pu0.4', '-1.329', '-0.233702385864']
NOsRb 10 ['N0.625Rb0.125Os0.25', '-0.361', '-0.215260187996']
NOsSc 7 ['N0.2Sc0.6Os0.2', '-1.154', '-0.218019530932']
NOsSr 12 ['N0.444444Sr0.444444Os0.111111', '-0.764', '-0.206341134292']
NOsTi 6 ['N0.375Ti0.5Os0.125', '-1.51', '-0.212963177992']
NOsTm 1 ['N0.4Tm0.5Os0.1', '-1.966', '-0.395658850864']
NOsY 3 ['N0.2Y0.6Os0.2', '-1.012', '-0.202850778682']

NOsYb 12 ['N0.555556Yb0.222222Os0.222222', '-0.61', '-0.222078976985']
NOsZn 2 ['N0.444444Zn0.444444Os0.111111', '-0.202', '-0.202']
NPPa 40 ['N0.25P0.25Pa0.5', '-1.456', '-0.217700040768']
NPPm 50 ['N0.111111P0.555556Pm0.333333', '-1.097', '-0.209433476878']
NPPr 16 ['N0.5P0.125Pr0.375', '-1.398', '-0.206605790428']
NPPt 1 ['N0.5P0.3Pt0.2', '-0.641', '-0.205074102786']
NPPu 18 ['N0.625P0.125Pu0.25', '-1.084', '-0.224962079973']
NPRb 13 ['N0.444444P0.333333Rb0.222222', '-0.673', '-0.200912795042']
NPRu 3 ['N0.5P0.3Ru0.2', '-0.625', '-0.200130682447']
NPSc 28 ['N0.222222P0.444444Sc0.333333', '-1.495', '-0.229987122955']
NPSm 27 ['N0.4P0.1Sm0.5', '-1.814', '-0.204843592218']
NPSr 51 ['N0.333333P0.111111Sr0.555556', '-0.947', '-0.200239223519']
NPTa 5 ['N0.1P0.5Ta0.4', '-0.881', '-0.224023503778']
NPTb 21 ['N0.333333P0.222222Tb0.444444', '-1.563', '-0.237877164916']
NPTc 4 ['N0.555556P0.333333Tc0.111111', '-0.696', '-0.211548333196']
NPTh 36 ['N0.222222P0.444444Th0.333333', '-1.569', '-0.204050822506']
NPTi 26 ['N0.285714P0.285714Ti0.428571', '-1.479', '-0.242723140414']
NPTm 19 ['N0.571429P0.285714Tm0.142857', '-1.141', '-0.222987683405']
NPU 15 ['N0.555556P0.333333U0.111111', '-0.991', '-0.202334720588']
NPV 5 ['N0.5P0.3V0.2', '-0.986', '-0.203708841217']
NPW 4 ['N0.6P0.2W0.2', '-0.574', '-0.221690593335']
NPY 38 ['N0.111111P0.444444Y0.444444', '-1.824', '-0.203331369105']
NPYb 53 ['N0.125P0.25Yb0.625', '-1.034', '-0.202720549591']
NPZr 11 ['N0.5P0.3Zr0.2', '-1.211', '-0.215258859025']
NPaPb 18 ['N0.444444Pb0.333333Pa0.222222', '-0.67', '-0.230913692911']
NPaPd 19 ['N0.375Pd0.125Pa0.5', '-1.224', '-0.207601769962']
NPaPm 14 ['N0.625Pm0.125Pa0.25', '-1.081', '-0.201642882295']
NPaPr 12 ['N0.5Pr0.333333Pa0.166667', '-1.472', '-0.213987118737']
NPaPt 27 ['N0.333333Pt0.222222Pa0.444444', '-1.246', '-0.228396420778']
NPaPu 25 ['N0.428571Pa0.142857Pu0.428571', '-1.409', '-0.213982219377']
NPaRb 9 ['N0.444444Rb0.111111Pa0.444444', '-1.287', '-0.351251163796']
NPaRe 27 ['N0.4Re0.2Pa0.4', '-1.026', '-0.212399869217']
NPaRh 23 ['N0.555556Rh0.333333Pa0.111111', '-0.611', '-0.21113361']
NPaRu 30 ['N0.333333Ru0.333333Pa0.333333', '-0.861', '-0.202370539367']
NPaS 37 ['N0.1S0.7Pa0.2', '-1.019', '-0.201463683295']
NPaSb 33 ['N0.666667Sb0.166667Pa0.166667', '-0.55', '-0.220685269683']
NPaSc 21 ['N0.666667Sc0.111111Pa0.222222', '-1.09', '-0.20189272954']
NPaSe 53 ['N0.111111Se0.444444Pa0.444444', '-1.272', '-0.20175524']
NPaSi 29 ['N0.2Si0.3Pa0.5', '-0.95', '-0.230156815653']
NPaSm 13 ['N0.5Sm0.333333Pa0.166667', '-1.614', '-0.227038575403']
NPaSn 24 ['N0.571429Sn0.285714Pa0.142857', '-0.56', '-0.277730231157']
NPaSr 16 ['N0.5Sr0.4Pa0.1', '-0.945', '-0.224161267578']
NPaTa 11 ['N0.444444Ta0.111111Pa0.444444', '-1.467', '-0.271181250454']
NPaTb 17 ['N0.5Tb0.4Pa0.1', '-1.647', '-0.300398273802']

NPaTc 25 ['N0.666667Tc0.111111Pa0.222222', '-0.679', '-0.210839214836']
NPaTe 44 ['N0.142857Te0.428571Pa0.428571', '-0.898', '-0.204249831736']
NPaTh 11 ['N0.5Th0.2Pa0.3', '-1.657', '-0.256593931338']
NPaTi 26 ['N0.428571Ti0.142857Pa0.428571', '-1.456', '-0.238608744326']
NPaTl 19 ['N0.5Tl0.333333Pa0.166667', '-0.557', '-0.227685269683']
NPaTm 15 ['N0.5Tm0.4Pa0.1', '-1.95', '-0.208948063674']
NPaU 10 ['N0.6Pa0.1U0.3', '-1.688', '-0.238579872458']
NPaV 16 ['N0.5V0.3Pa0.2', '-1.372', '-0.225078800018']
NPaW 20 ['N0.5W0.3Pa0.2', '-0.725', '-0.221108598327']
NPaXe 21 ['N0.4Xe0.1Pa0.5', '-1.189', '-0.20105580905']
NPaY 14 ['N0.5Y0.375Pa0.125', '-1.794', '-0.200596893385']
NPaYb 19 ['N0.444444Yb0.333333Pa0.222222', '-1.253', '-0.232032158389']
NPaZn 37 ['N0.666667Zn0.111111Pa0.222222', '-0.654', '-0.214913692911']
NPaZr 10 ['N0.5Zr0.2Pa0.3', '-1.538', '-0.200526590839']
NPbPu 7 ['N0.5Pb0.125Pu0.375', '-1.291', '-0.264158486747']
NPbSc 3 ['N0.5Sc0.333333Pb0.166667', '-1.57', '-0.222937109887']
NPbTi 4 ['N0.5Ti0.3Pb0.2', '-1.02', '-0.241777906795']
NPdPu 8 ['N0.5Pd0.125Pu0.375', '-1.228', '-0.201158486747']
NPdSc 3 ['N0.5Sc0.4Pd0.1', '-1.846', '-0.229524531864']
NPdTi 9 ['N0.4Ti0.3Pd0.3', '-0.985', '-0.206361683318']
NPdZr 2 ['N0.4Zr0.3Pd0.3', '-1.319', '-0.201939658113']
NPmPt 15 ['N0.428571Pm0.285714Pt0.285714', '-1.126', '-0.200785641126']
NPmPu 1 ['N0.555556Pm0.111111Pu0.333333', '-1.488', '-0.232687524016']
NPmRh 1 ['N0.4Rh0.1Pm0.5', '-1.611', '-0.241419195614']
NPmRu 1 ['N0.4Ru0.1Pm0.5', '-1.458', '-0.202786797614']
NPmS 12 ['N0.375S0.125Pm0.5', '-1.83', '-0.206257642495']
NPmSe 21 ['N0.2Se0.6Pm0.2', '-0.858', '-0.2096079195']
NPmSi 1 ['N0.444444Si0.111111Pm0.444444', '-1.58', '-0.209742143182']
NPmTe 17 ['N0.4Te0.2Pm0.4', '-1.441', '-0.207767928864']
NPmTi 9 ['N0.444444Ti0.111111Pm0.444444', '-1.636', '-0.202706061789']
NPmZn 7 ['N0.444444Zn0.444444Pm0.111111', '-0.579', '-0.236435535796']
NPrPt 12 ['N0.111111Pr0.555556Pt0.333333', '-1.279', '-0.223863103851']
NPrPu 5 ['N0.5Pr0.125Pu0.375', '-1.576', '-0.200896680142']
NPrTi 5 ['N0.555556Ti0.333333Pr0.111111', '-1.393', '-0.218742735012']
NPrW 2 ['N0.625Pr0.125W0.25', '-0.682', '-0.242305257447']
NPrZn 2 ['N0.428571Zn0.428571Pr0.142857', '-0.6', '-0.201986506737']
NPtPu 26 ['N0.428571Pt0.142857Pu0.428571', '-1.385', '-0.200319328721']
NPtRb 7 ['N0.6Rb0.2Pt0.2', '-0.44', '-0.206816300794']
NPtSc 21 ['N0.111111Sc0.555556Pt0.333333', '-1.527', '-0.203880424129']
NPtSm 4 ['N0.285714Sm0.428571Pt0.285714', '-1.57', '-0.20399631169']
NPtSr 7 ['N0.2Sr0.5Pt0.3', '-0.931', '-0.203430459307']
NPtTa 1 ['N0.25Ta0.625Pt0.125', '-1.102', '-0.21679850356']
NPtTb 4 ['N0.3Tb0.5Pt0.2', '-1.446', '-0.207283645061']
NPtTh 17 ['N0.1Pt0.7Th0.2', '-0.811', '-0.202802765341']

NPtTi 28 ['N0.285714Ti0.285714Pt0.428571', '-1.064', '-0.203754167252']
NPtTm 3 ['N0.428571Tm0.428571Pt0.142857', '-1.877', '-0.202758181299']
NPtW 3 ['N0.571429W0.142857Pt0.285714', '-0.26', '-0.20775260803']
NPtXe 3 ['N0.3Xe0.6Pt0.1', '-0.224', '-0.224']
NPtY 2 ['N0.375Y0.375Pt0.25', '-1.617', '-0.242742256567']
NPtYb 10 ['N0.3Yb0.6Pt0.1', '-1.25', '-0.202944167145']
NPtZr 11 ['N0.333333Zr0.333333Pt0.333333', '-1.483', '-0.202202410454']
NPuRb 6 ['N0.5Rb0.125Pu0.375', '-1.281', '-0.205578549413']
NPuRe 17 ['N0.555556Re0.333333Pu0.111111', '-0.559', '-0.216009366035']
NPuRh 13 ['N0.444444Rh0.333333Pu0.222222', '-0.809', '-0.20050132548']
NPuRu 6 ['N0.5Ru0.166667Pu0.333333', '-1.134', '-0.22125198822']
NPuS 10 ['N0.444444S0.111111Pu0.444444', '-1.554', '-0.200108798502']
NPuSb 11 ['N0.5Sb0.25Pu0.25', '-0.911', '-0.226438991165']
NPuSc 20 ['N0.5Sc0.1Pu0.4', '-1.705', '-0.20558351883']
NPuSi 2 ['N0.571429Si0.142857Pu0.285714', '-1.396', '-0.208807290315']
NPuSm 3 ['N0.6Sm0.1Pu0.3', '-1.415', '-0.276232781114']
NPuSn 2 ['N0.555556Sn0.111111Pu0.333333', '-1.116', '-0.20325198822']
NPuSr 17 ['N0.444444Sr0.444444Pu0.111111', '-1.01', '-0.220402565959']
NPuTa 6 ['N0.6Ta0.2Pu0.2', '-1.382', '-0.204668273817']
NPuTb 19 ['N0.4Tb0.4Pu0.2', '-1.351', '-0.201987111992']
NPuTc 27 ['N0.375Tc0.25Pu0.375', '-1.26', '-0.233158486747']
NPuTe 19 ['N0.5Te0.25Pu0.25', '-0.894', '-0.205260497249']
NPuTh 7 ['N0.625Th0.125Pu0.25', '-1.391', '-0.201664269857']
NPuTi 18 ['N0.625Ti0.125Pu0.25', '-1.235', '-0.226179785663']
NPuTl 9 ['N0.555556Tl0.111111Pu0.333333', '-1.133', '-0.22025198822']
NPuTm 5 ['N0.5Tm0.166667Pu0.333333', '-1.798', '-0.242142363997']
NPuU 5 ['N0.5U0.166667Pu0.333333', '-1.591', '-0.206305938163']
NPuV 11 ['N0.5V0.25Pu0.25', '-1.537', '-0.22598605483']
NPuW 20 ['N0.444444W0.222222Pu0.333333', '-1.165', '-0.21273268096']
NPuXe 1 ['N0.555556Xe0.111111Pu0.333333', '-1.184', '-0.27125198822']
NPuY 3 ['N0.6Y0.1Pu0.3', '-1.384', '-0.203482240364']
NPuYb 8 ['N0.5Yb0.166667Pu0.333333', '-1.42', '-0.216311220959']
NPuZn 22 ['N0.5Zn0.4Pu0.1', '-0.485', '-0.211175596466']
NPuZr 3 ['N0.571429Zr0.142857Pu0.285714', '-1.578', '-0.263711065195']
NRbRe 15 ['N0.6Rb0.3Re0.1', '-0.436', '-0.202816300794']
NRbRh 13 ['N0.666667Rb0.222222Rh0.111111', '-0.465', '-0.205907000882']
NRbRu 8 ['N0.5Rb0.166667Ru0.333333', '-0.408', '-0.213680250661']
NRbTc 11 ['N0.625Rb0.25Tc0.125', '-0.457', '-0.201788942841']
NRbTi 5 ['N0.5Ti0.25Rb0.25', '-1.006', '-0.227935089437']
NRbW 23 ['N0.666667Rb0.111111W0.222222', '-0.423', '-0.212179779598']
NReSc 13 ['N0.6Sc0.1Re0.3', '-0.666', '-0.227013965932']
NReSm 6 ['N0.5Sm0.375Re0.125', '-1.415', '-0.210619482671']
NReSr 21 ['N0.428571Sr0.285714Re0.285714', '-0.655', '-0.204975378781']
NReTb 6 ['N0.5Tb0.333333Re0.166667', '-1.19', '-0.213118611641']

NReTi 25 ['N0.555556Ti0.111111Re0.333333', '-0.552', '-0.225028298404']
NReYb 19 ['N0.5Yb0.166667Re0.333333', '-0.556', '-0.226317936034']
NReZn 11 ['N0.4Zn0.5Re0.1', '-0.233', '-0.221377610989']
NRhSc 5 ['N0.5Sc0.3Rh0.2', '-1.419', '-0.206643398898']
NRhSr 3 ['N0.5Sr0.166667Rh0.333333', '-0.547', '-0.205750013787']
NRhTi 12 ['N0.444444Ti0.333333Rh0.222222', '-1.088', '-0.223308785328']
NRhXe 7 ['N0.3Rh0.2Xe0.5', '-0.205', '-0.205']
NRhY 1 ['N0.4Y0.5Rh0.1', '-1.806', '-0.201308549364']
NRhYb 14 ['N0.333333Rh0.166667Yb0.5', '-1.074', '-0.201177698217']
NRhZn 7 ['N0.333333Zn0.444444Rh0.222222', '-0.463', '-0.204711268889']
NRuSc 5 ['N0.571429Sc0.285714Ru0.142857', '-1.359', '-0.204374665617']
NRuTi 19 ['N0.4Ti0.3Ru0.3', '-0.996', '-0.217777906795']
NRuY 7 ['N0.25Y0.5Ru0.25', '-1.235', '-0.203062591165']
NRuYb 7 ['N0.444444Ru0.111111Yb0.444444', '-0.979', '-0.20315795397']
NSTa 1 ['N0.3S0.1Ta0.6', '-1.262', '-0.203955070829']
NSTi 11 ['N0.285714S0.285714Ti0.428571', '-1.614', '-0.245815197165']
NSXe 4 ['N0.142857S0.428571Xe0.428571', '-0.203', '-0.203']
NSbSc 9 ['N0.125Sc0.625Sb0.25', '-1.304', '-0.209724909958']
NSbTh 8 ['N0.6Sb0.2Th0.2', '-1.014', '-0.20427627043']
NSbTi 4 ['N0.5Ti0.375Sb0.125', '-1.18', '-0.207222383494']
NSbY 1 ['N0.285714Y0.571429Sb0.142857', '-1.611', '-0.217489220617']
NSbYb 16 ['N0.5Sb0.1Yb0.4', '-0.909', '-0.200525745252']
NScSe 17 ['N0.166667Sc0.5Se0.333333', '-1.938', '-0.201794053277']
NScSi 32 ['N0.222222Si0.222222Sc0.555556', '-1.536', '-0.20293809298']
NScSn 5 ['N0.555556Sc0.333333Sn0.111111', '-1.605', '-0.257937109887']
NScTa 3 ['N0.555556Sc0.333333Ta0.111111', '-1.925', '-0.228113265934']
NScTc 16 ['N0.6Sc0.2Tc0.2', '-1.072', '-0.211428205398']
NScTi 8 ['N0.555556Sc0.333333Ti0.111111', '-1.841', '-0.205706704996']
NScTl 4 ['N0.5Sc0.333333Tl0.166667', '-1.55', '-0.202937109887']
NScU 14 ['N0.6Sc0.1U0.3', '-1.821', '-0.211970389792']
NScV 10 ['N0.5Sc0.4V0.1', '-2.069', '-0.20194335733']
NScW 14 ['N0.5Sc0.25W0.25', '-1.301', '-0.201784391079']
NScYb 3 ['N0.444444Sc0.222222Yb0.333333', '-1.684', '-0.204076538736']
NScZn 8 ['N0.4Sc0.1Zn0.5', '-0.63', '-0.225881132966']
NSeSr 4 ['N0.125Se0.25Sr0.625', '-1.482', '-0.213373955582']
NSeTi 7 ['N0.333333Ti0.444444Se0.222222', '-1.481', '-0.243025086018']
NSeTm 1 ['N0.222222Se0.333333Tm0.444444', '-1.93', '-0.200280747703']
NSeXe 6 ['N0.2Se0.3Xe0.5', '-0.201', '-0.201']
NSeY 14 ['N0.142857Se0.571429Y0.285714', '-1.228', '-0.210536007809']
NSeYb 9 ['N0.1Se0.3Yb0.6', '-1.739', '-0.213457684465']
NSiSr 1 ['N0.2Si0.3Sr0.5', '-0.942', '-0.207830496932']
NSiTb 11 ['N0.4Si0.2Tb0.4', '-1.398', '-0.219865851708']
NSiTc 4 ['N0.142857Si0.428571Tc0.428571', '-0.782', '-0.200254271737']
NSiTh 22 ['N0.5Si0.3Th0.2', '-1.537', '-0.205941719052']

NSiTi 15 ['N0.375Si0.25Ti0.375', '-1.489', '-0.208409545397']
NSiTm 17 ['N0.444444Si0.222222Tm0.333333', '-1.736', '-0.213625676794']
NSiU 20 ['N0.625Si0.125U0.25', '-1.627', '-0.21002441916']
NSiV 4 ['N0.428571Si0.142857V0.428571', '-1.331', '-0.200517108452']
NSiYb 25 ['N0.5Si0.3Yb0.2', '-1.344', '-0.20220830908']
NSiZn 1 ['N0.5Si0.25Zn0.25', '-0.912', '-0.203534775721']
NSmTe 9 ['N0.142857Te0.428571Sm0.428571', '-1.62', '-0.208618743877']
NSmTi 11 ['N0.444444Ti0.222222Sm0.333333', '-1.732', '-0.20571667873']
NSmW 11 ['N0.5Sm0.4W0.1', '-1.516', '-0.21125659033']
NSmZn 3 ['N0.444444Zn0.333333Sm0.222222', '-0.906', '-0.200902203813']
NSnTb 2 ['N0.5Sn0.2Tb0.3', '-1.072', '-0.210240333994']
NSnTc 1 ['N0.444444Tc0.333333Sn0.222222', '-0.296', '-0.208776565776']
NSnTi 6 ['N0.428571Ti0.428571Sn0.142857', '-1.312', '-0.200254152564']
NSrTc 20 ['N0.555556Sr0.333333Tc0.111111', '-0.779', '-0.216768826476']
NSrTh 1 ['N0.5Sr0.25Th0.25', '-1.474', '-0.205000522119']
NSrTi 6 ['N0.5Ti0.3Sr0.2', '-1.626', '-0.210077892702']
NSrU 11 ['N0.5Sr0.125U0.375', '-1.518', '-0.218598859201']
NSrV 3 ['N0.5V0.25Sr0.25', '-1.428', '-0.203845339515']
NSrW 22 ['N0.555556Sr0.333333W0.111111', '-0.779', '-0.218376416754']
NTaTc 1 ['N0.428571Tc0.142857Ta0.428571', '-1.324', '-0.22534751914']
NTaTh 1 ['N0.555556Ta0.222222Th0.222222', '-1.79', '-0.271345858558']
NTaTi 6 ['N0.571429Ti0.285714Ta0.142857', '-1.414', '-0.223062588056']
NTaYb 17 ['N0.5Yb0.166667Ta0.333333', '-1.398', '-0.202703688718']
NTbTc 5 ['N0.5Tc0.25Tb0.25', '-0.984', '-0.200449369327']
NTbTe 15 ['N0.4Te0.3Tb0.3', '-1.09', '-0.228240333994']
NTbTi 11 ['N0.5Ti0.1Tb0.4', '-1.64', '-0.23157974759']
NTbU 5 ['N0.5Tb0.25U0.25', '-1.63', '-0.20394786991']
NTbW 11 ['N0.5Tb0.3W0.2', '-1.15', '-0.217105580925']
NTbZn 4 ['N0.444444Zn0.333333Tb0.222222', '-0.87', '-0.231659506662']
NTcTh 11 ['N0.5Tc0.1Th0.4', '-1.75', '-0.203453525328']
NTcTi 37 ['N0.222222Ti0.333333Tc0.444444', '-1.066', '-0.201308785328']
NTcTm 7 ['N0.333333Tc0.111111Tm0.555556', '-1.489', '-0.202780751553']
NTcU 2 ['N0.571429Tc0.285714U0.142857', '-0.925', '-0.209440684138']
NTcW 19 ['N0.6Tc0.2W0.2', '-0.335', '-0.209519590707']
NTcY 8 ['N0.5Y0.166667Tc0.333333', '-0.886', '-0.200368984053']
NTcYb 30 ['N0.4Tc0.4Yb0.2', '-0.663', '-0.209202958218']
NTcZn 22 ['N0.571429Zn0.142857Tc0.285714', '-0.275', '-0.200237056379']
NTeTh 10 ['N0.3Te0.3Th0.4', '-1.932', '-0.202695981301']
NTeTi 9 ['N0.375Ti0.375Te0.25', '-1.175', '-0.202222383494']
NTeTm 1 ['N0.333333Te0.166667Tm0.5', '-1.886', '-0.21188750433']
NTeW 4 ['N0.6Te0.1W0.3', '-0.329', '-0.219280476862']
NTeY 21 ['N0.142857Y0.571429Te0.285714', '-1.466', '-0.204958118997']
NThTi 9 ['N0.5Ti0.3Th0.2', '-1.802', '-0.216138352703']
NThW 2 ['N0.6W0.3Th0.1', '-0.758', '-0.244460699816']

NThYb 6 ['N0.5Yb0.3Th0.2', '-1.543', '-0.211667064838']
NThZn 12 ['N0.5Zn0.125Th0.375', '-1.76', '-0.245675836077']
NTiTl 5 ['N0.428571Ti0.428571Tl0.142857', '-1.384', '-0.272254152564']
NTiTm 8 ['N0.4Ti0.1Tm0.5', '-1.765', '-0.203583306985']
NTiU 14 ['N0.5Ti0.166667U0.333333', '-1.697', '-0.215196724474']
NTiV 4 ['N0.555556Ti0.222222V0.222222', '-1.335', '-0.201692135699']
NTiW 21 ['N0.444444Ti0.222222W0.333333', '-0.882', '-0.200154370857']
NTiXe 3 ['N0.5Ti0.375Xe0.125', '-1.221', '-0.248222383494']
NTiY 12 ['N0.428571Ti0.142857Y0.428571', '-1.792', '-0.208431636099']
NTiYb 16 ['N0.571429Ti0.285714Yb0.142857', '-1.204', '-0.2134583012']
NTiZn 32 ['N0.333333Ti0.111111Zn0.555556', '-0.497', '-0.208769595109']
NTiZr 7 ['N0.5Ti0.2Zr0.3', '-1.848', '-0.227444663071']
NTlTm 1 ['N0.4Tm0.5Tl0.1', '-1.871', '-0.269635907364']
NTlW 2 ['N0.571429W0.285714Tl0.142857', '-0.342', '-0.237505216059']
NTlY 2 ['N0.333333Y0.555556Tl0.111111', '-1.539', '-0.237944183035']
NTlYb 1 ['N0.375Yb0.5Tl0.125', '-1.102', '-0.229177698217']
NTmU 1 ['N0.555556Tm0.111111U0.333333', '-1.832', '-0.248237014252']
NTmV 1 ['N0.4V0.1Tm0.5', '-1.764', '-0.220536901864']
NTmW 7 ['N0.5Tm0.333333W0.166667', '-1.55', '-0.204501790663']
NTmYb 1 ['N0.444444Tm0.444444Yb0.111111', '-1.954', '-0.239041002071']
NTmZn 3 ['N0.4Zn0.5Tm0.1', '-0.597', '-0.211134225466']
NUW 9 ['N0.625W0.125U0.25', '-1.38', '-0.214147127909']
NUYb 6 ['N0.5Yb0.333333U0.166667', '-1.465', '-0.213723608159']
NUZn 9 ['N0.444444Zn0.444444U0.111111', '-0.711', '-0.21260282159']
NVYb 13 ['N0.4V0.2Yb0.4', '-1.274', '-0.204253764305']
NVZn 8 ['N0.5V0.125Zn0.375', '-0.52', '-0.206773531832']
NWY 5 ['N0.555556Y0.111111W0.333333', '-0.74', '-0.21915103092']
NWYb 30 ['N0.4Yb0.3W0.3', '-0.802', '-0.207171865861']
NWZn 32 ['N0.625Zn0.125W0.25', '-0.293', '-0.201567064052']
NXeZn 8 ['N0.222222Zn0.222222Xe0.555556', '-0.203', '-0.203']
NYZn 9 ['N0.444444Zn0.444444Y0.111111', '-0.627', '-0.228061612184']
NYZr 1 ['N0.444444Y0.333333Zr0.222222', '-1.81', '-0.200794736514']
NYbZn 19 ['N0.3Zn0.1Yb0.6', '-1.055', '-0.201812860395']
NYbZr 2 ['N0.5Zr0.25Yb0.25', '-1.579', '-0.211705230869']
NZnZr 9 ['N0.4Zn0.4Zr0.2', '-0.959', '-0.214293105409']
NaNbPd 2 ['Na0.1Nb0.2Pd0.7', '-0.572', '-0.203693485581']
NaNbS 2 ['Na0.111111S0.666667Nb0.222222', '-1.316', '-0.237513811628']
NaNdP 4 ['Na0.142857P0.428571Nd0.428571', '-1.637', '-0.208487019731']
NaNdS 1 ['Na0.3S0.5Nd0.2', '-1.904', '-0.200132983893']
NaNiO 1 ['O0.666667Na0.222222Ni0.111111', '-1.218', '-0.200816234621']
NaNpO 1 ['O0.6Na0.2Np0.2', '-3.167', '-0.31639445965']
NaNpS 1 ['Na0.166667S0.5Np0.333333', '-1.723', '-0.222972672227']
NaNpSe 4 ['Na0.2Se0.6Np0.2', '-1.124', '-0.21077909258']
NaOOs 3 ['O0.666667Na0.222222Os0.111111', '-1.602', '-0.217574765061']

NaOP 5 ['O0.375Na0.5P0.125', '-2.02', '-0.205748475273']
NaOPa 4 ['O0.625Na0.25Pa0.125', '-2.415', '-0.201336186738']
NaOPb 3 ['O0.625Na0.25Pb0.125', '-1.408', '-0.229753961923']
NaOPd 5 ['O0.625Na0.25Pd0.125', '-1.269', '-0.231855304238']
NaOPt 11 ['O0.333333Na0.555556Pt0.111111', '-1.512', '-0.202446028863']
NaOPu 4 ['O0.6Na0.1Pu0.3', '-3.345', '-0.206485767407']
NaORe 5 ['O0.625Na0.125Re0.25', '-2.094', '-0.221834191689']
NaORh 3 ['O0.6Na0.2Rh0.2', '-1.467', '-0.2346556813']
NaORu 3 ['O0.666667Na0.222222Ru0.111111', '-1.418', '-0.202131851723']
NaOSb 2 ['O0.625Na0.25Sb0.125', '-1.846', '-0.237708119987']
NaOSc 1 ['O0.6Na0.2Sc0.2', '-2.715', '-0.22599825997']
NaOSe 10 ['O0.333333Na0.5Se0.166667', '-1.7', '-0.206857321365']
NaOSi 1 ['O0.6Na0.2Si0.2', '-2.763', '-0.253678832302']
NaOSr 5 ['O0.5Na0.166667Sr0.333333', '-2.634', '-0.226952481917']
NaOTb 1 ['O0.666667Na0.222222Tb0.111111', '-1.938', '-0.229801355568']
NaOTc 9 ['O0.5Na0.4Tc0.1', '-1.818', '-0.201112213217']
NaOTe 2 ['O0.6Na0.2Te0.2', '-1.842', '-0.270656605507']
NaOTi 7 ['O0.444444Na0.111111Ti0.444444', '-2.668', '-0.20691613672']
NaOTl 6 ['O0.4Na0.2Tl0.4', '-1.284', '-0.205452628312']
NaOTm 2 ['O0.6Na0.2Tm0.2', '-2.769', '-0.223216188051']
NaOU 2 ['O0.666667Na0.166667U0.166667', '-2.878', '-0.22362911131']
NaOW 1 ['O0.6Na0.2W0.2', '-2.581', '-0.2011396423']
NaOY 7 ['O0.4Na0.1Y0.5', '-2.741', '-0.202203759647']
NaOZn 1 ['O0.666667Na0.222222Zn0.111111', '-1.368', '-0.261423614781']
NaOsP 18 ['Na0.333333P0.5Os0.166667', '-0.811', '-0.208644154194']
NaOsSc 2 ['Na0.125Sc0.625Os0.25', '-0.361', '-0.20132158125']
NaOsSe 1 ['Na0.25Se0.5Os0.25', '-0.742', '-0.208727315883']
NaOsTm 2 ['Na0.111111Tm0.555556Os0.333333', '-0.306', '-0.216406496667']
NaPPd 6 ['Na0.5P0.375Pd0.125', '-0.739', '-0.201122309443']
NaPPm 15 ['Na0.222222P0.333333Pm0.444444', '-1.0', '-0.205487829513']
NaPPr 3 ['Na0.125P0.375Pr0.5', '-1.435', '-0.201201303828']
NaPPt 12 ['Na0.5P0.25Pt0.25', '-0.731', '-0.201363340363']
NaPPu 2 ['Na0.166667P0.5Pu0.333333', '-1.259', '-0.209710994961']
NaPRe 3 ['Na0.25P0.5Re0.25', '-0.738', '-0.216200937495']
NaPRh 19 ['Na0.222222P0.555556Rh0.222222', '-0.916', '-0.200039639694']
NaPRu 20 ['Na0.142857P0.285714Ru0.571429', '-0.674', '-0.233830514104']
NaPS 2 ['Na0.5P0.125S0.375', '-1.317', '-0.204640818023']
NaPSc 1 ['Na0.142857P0.428571Sc0.428571', '-1.624', '-0.236222344017']
NaPSi 10 ['Na0.25Si0.25P0.5', '-0.63', '-0.207882414197']
NaPSn 2 ['Na0.166667P0.5Sn0.333333', '-0.555', '-0.204112646092']
NaPSr 3 ['Na0.1P0.4Sr0.5', '-1.237', '-0.253849557495']
NaPTa 15 ['Na0.111111P0.333333Ta0.555556', '-1.007', '-0.235479788377']
NaPTc 3 ['Na0.2P0.5Tc0.3', '-0.841', '-0.208394744112']
NaPY 18 ['Na0.111111P0.555556Y0.333333', '-1.543', '-0.205149518608']

NaPYb 10 ['Na0.1P0.3Yb0.6', '-0.818', '-0.213234695312']
NaPZr 2 ['Na0.1P0.5Zr0.4', '-1.523', '-0.204541191185']
NaPaPd 1 ['Na0.166667Pd0.666667Pa0.166667', '-0.708', '-0.220566976525']
NaPaS 17 ['Na0.375S0.5Pa0.125', '-1.465', '-0.202848788612']
NaPaSe 18 ['Na0.3Se0.6Pa0.1', '-0.998', '-0.211350306495']
NaPaSi 3 ['Na0.142857Si0.714286Pa0.142857', '-0.329', '-0.220229449037']
NaPaTe 3 ['Na0.142857Te0.571429Pa0.285714', '-0.788', '-0.210847741545']
NaPdPm 14 ['Na0.1Pd0.8Pm0.1', '-0.384', '-0.201593710665']
NaPdU 1 ['Na0.142857Pd0.714286U0.142857', '-0.547', '-0.206023825235']
NaPmS 17 ['Na0.333333S0.444444Pm0.222222', '-1.893', '-0.242663025154']
NaPmSb 1 ['Na0.1Sb0.4Pm0.5', '-0.968', '-0.217992591665']
NaPmSe 24 ['Na0.166667Se0.333333Pm0.5', '-1.305', '-0.207759215067']
NaPmSi 5 ['Na0.142857Si0.285714Pm0.571429', '-0.471', '-0.220348783571']
NaPmSn 3 ['Na0.1Sn0.3Pm0.6', '-0.592', '-0.207959143194']
NaPmTe 9 ['Na0.2Te0.5Pm0.3', '-1.305', '-0.218597420745']
NaPrPt 1 ['Na0.1Pr0.5Pt0.4', '-1.103', '-0.208314985']
NaPrS 4 ['Na0.333333S0.555556Pr0.111111', '-1.538', '-0.202181725806']
NaPrTe 4 ['Na0.166667Te0.5Pr0.333333', '-1.633', '-0.24517239736']
NaPtSr 6 ['Na0.1Sr0.6Pt0.3', '-0.646', '-0.205804422915']
NaPtTh 4 ['Na0.111111Pt0.666667Th0.222222', '-0.886', '-0.207068774905']
NaPuS 21 ['Na0.4S0.5Pu0.1', '-1.484', '-0.217444318404']
NaRbS 4 ['Na0.333333S0.5Rb0.166667', '-1.263', '-0.204454431044']
NaRbSe 1 ['Na0.5Se0.375Rb0.125', '-1.296', '-0.200496877077']
NaReS 2 ['Na0.285714S0.571429Re0.142857', '-1.132', '-0.219750234786']
NaRhSb 3 ['Na0.111111Rh0.555556Sb0.333333', '-0.608', '-0.201965564072']
NaRhSi 4 ['Na0.125Si0.375Rh0.5', '-0.896', '-0.207066444219']
NaSSc 2 ['Na0.2S0.5Sc0.3', '-2.031', '-0.207598635322']
NaSSe 2 ['Na0.555556S0.333333Se0.111111', '-1.288', '-0.215000561524']
NaSSi 1 ['Na0.5Si0.1S0.4', '-1.416', '-0.248307973408']
NaSSm 5 ['Na0.125S0.375Sm0.5', '-1.829', '-0.20095364318']
NaSSr 3 ['Na0.333333S0.5Sr0.166667', '-1.692', '-0.237068551043']
NaSTb 7 ['Na0.222222S0.444444Tb0.333333', '-1.836', '-0.20334507425']
NaSTc 4 ['Na0.1S0.6Tc0.3', '-1.054', '-0.201120229007']
NaSTh 10 ['Na0.1S0.5Th0.4', '-2.126', '-0.209816200406']
NaSTl 1 ['Na0.375S0.375Tl0.25', '-1.105', '-0.212265153871']
NaSU 1 ['Na0.333333S0.555556U0.111111', '-1.527', '-0.217894399012']
NaSXe 3 ['Na0.166667S0.5Xe0.333333', '-0.633', '-0.204632567769']
NaSY 4 ['Na0.1S0.5Y0.4', '-2.301', '-0.205570345906']
NaSZr 27 ['Na0.3S0.4Zr0.3', '-1.532', '-0.205781192888']
NaScSe 14 ['Na0.222222Sc0.333333Se0.444444', '-1.651', '-0.20599125259']
NaScSi 1 ['Na0.125Si0.375Sc0.5', '-0.901', '-0.202141849062']
NaSeSm 4 ['Na0.1Se0.5Sm0.4', '-1.946', '-0.206933201165']
NaSeSr 5 ['Na0.111111Se0.444444Sr0.444444', '-2.053', '-0.210702137778']
NaSeTa 5 ['Na0.222222Se0.555556Ta0.222222', '-1.131', '-0.203632654257']

NaSeTb 3 ['Na0.166667Se0.5Tb0.333333', '-1.647', '-0.204249697887']
NaSeTc 11 ['Na0.2Se0.6Tc0.2', '-0.575', '-0.20689278358']
NaSeTe 3 ['Na0.555556Se0.222222Te0.222222', '-1.137', '-0.203793619345']
NaSeTh 7 ['Na0.1Se0.6Th0.3', '-1.841', '-0.209489385913']
NaSeTi 4 ['Na0.142857Ti0.428571Se0.428571', '-1.325', '-0.205692962451']
NaSeTl 10 ['Na0.142857Se0.428571Tl0.428571', '-0.687', '-0.2041837587']
NaSeTm 8 ['Na0.1Se0.6Tm0.3', '-1.718', '-0.245959650418']
NaSeU 4 ['Na0.222222Se0.555556U0.222222', '-1.364', '-0.224528892257']
NaSeY 22 ['Na0.285714Se0.428571Y0.285714', '-1.721', '-0.220340982616']
NaSeYb 1 ['Na0.125Se0.375Yb0.5', '-1.795', '-0.21538046875']
NaSeZr 14 ['Na0.2Se0.4Zr0.4', '-1.323', '-0.204953867026']
NaSiY 1 ['Na0.125Si0.375Y0.5', '-0.9', '-0.219841008438']
NaTbTe 1 ['Na0.111111Te0.555556Tb0.333333', '-1.233', '-0.260820195943']
NaTeTm 2 ['Na0.1Te0.5Tm0.4', '-1.339', '-0.211649965997']
NaTeY 2 ['Na0.125Y0.375Te0.5', '-1.43', '-0.203785426663']
NbNdO 2 ['O0.6Nb0.2Nd0.2', '-3.331', '-0.205900930769']
NbNdSe 3 ['Se0.6Nb0.2Nd0.2', '-1.551', '-0.218443680334']
NbNiP 1 ['P0.444444Ni0.222222Nb0.333333', '-1.153', '-0.212749746018']
NbOPa 4 ['O0.7Nb0.2Pa0.1', '-3.142', '-0.227505549883']
NbORb 3 ['O0.5Rb0.333333Nb0.166667', '-2.437', '-0.208863695808']
NbOS 26 ['O0.4S0.3Nb0.3', '-2.302', '-0.201557466693']
NbOSb 1 ['O0.444444Nb0.444444Sb0.111111', '-2.185', '-0.248607519955']
NbOSi 2 ['O0.666667Si0.222222Nb0.111111', '-3.166', '-0.210621555667']
NbOTi 1 ['O0.666667Ti0.111111Nb0.222222', '-3.115', '-0.218105572007']
NbOW 1 ['O0.714286Nb0.142857W0.142857', '-2.814', '-0.231003142227']
NbOsV 1 ['V0.6Nb0.1Os0.3', '-0.365', '-0.2106587645']
NbPPm 4 ['P0.5Nb0.1Pm0.4', '-1.377', '-0.20025868802']
NbPPu 11 ['P0.25Nb0.375Pu0.375', '-0.878', '-0.202238658073']
NbPRh 2 ['P0.285714Nb0.428571Rh0.285714', '-1.065', '-0.203639617361']
NbPRu 18 ['P0.444444Nb0.444444Ru0.111111', '-1.194', '-0.201436856018']
NbPS 9 ['P0.222222S0.555556Nb0.222222', '-1.126', '-0.20559818872']
NbPSn 1 ['P0.1Nb0.6Sn0.3', '-0.62', '-0.210303721438']
NbPTa 2 ['P0.5Nb0.1Ta0.4', '-0.967', '-0.20950762502']
NbPTe 7 ['P0.428571Nb0.142857Te0.428571', '-0.523', '-0.203961846577']
NbPaS 11 ['S0.444444Nb0.222222Pa0.333333', '-1.628', '-0.213889682705']
NbPaSe 10 ['Se0.666667Nb0.222222Pa0.111111', '-1.048', '-0.200750157315']
NbPaSn 1 ['Nb0.5Sn0.4Pa0.1', '-0.495', '-0.244422116843']
NbPdPm 1 ['Nb0.111111Pd0.777778Pm0.111111', '-0.6', '-0.237013751759']
NbPmS 8 ['S0.5Nb0.1Pm0.4', '-2.054', '-0.205449111991']
NbPmSe 21 ['Se0.444444Nb0.222222Pm0.333333', '-1.458', '-0.203225335']
NbPrS 1 ['S0.5Nb0.25Pr0.25', '-1.887', '-0.206383520544']
NbPrSe 2 ['Se0.555556Nb0.111111Pr0.333333', '-1.839', '-0.214835757664']
NbPtTh 1 ['Nb0.1Pt0.8Th0.1', '-0.728', '-0.222085417']
NbPuS 9 ['S0.5Nb0.25Pu0.25', '-1.723', '-0.205017495263']

NbPuSe 8 ['Se0.555556Nb0.111111Pu0.333333', '-1.517', '-0.201781577592']
NbRbS 1 ['S0.5Rb0.166667Nb0.333333', '-1.481', '-0.243215942737']
NbRbSe 8 ['Se0.625Rb0.125Nb0.25', '-1.084', '-0.213498881875']
NbRuSi 1 ['Si0.333333Nb0.333333Ru0.333333', '-0.779', '-0.212558237727']
NbSSc 6 ['S0.555556Sc0.333333Nb0.111111', '-2.079', '-0.209673386681']
NbSSi 1 ['Si0.222222S0.555556Nb0.222222', '-1.209', '-0.200761058592']
NbSTh 14 ['S0.625Nb0.125Th0.25', '-2.122', '-0.205918695499']
NbSTi 6 ['S0.571429Ti0.142857Nb0.285714', '-1.578', '-0.218797034785']
NbSTm 1 ['S0.666667Nb0.166667Tm0.166667', '-1.833', '-0.267386178469']
NbSU 2 ['S0.555556Nb0.111111U0.333333', '-1.704', '-0.200987849806']
NbSV 1 ['S0.555556V0.222222Nb0.222222', '-1.449', '-0.200553906319']
NbSZr 9 ['S0.444444Zr0.333333Nb0.222222', '-1.446', '-0.200606126038']
NbScSe 9 ['Sc0.166667Se0.666667Nb0.166667', '-1.185', '-0.207629802222']
NbSeSm 1 ['Se0.555556Nb0.222222Sm0.222222', '-1.623', '-0.226817962556']
NbSeTc 6 ['Se0.555556Nb0.111111Tc0.333333', '-0.528', '-0.212907421945']
NbSeTe 19 ['Se0.5Nb0.166667Te0.333333', '-0.647', '-0.200967053055']
NbSeTm 12 ['Se0.5Nb0.3Tm0.2', '-1.326', '-0.20210958245']
NbSeY 5 ['Se0.6Y0.2Nb0.2', '-1.415', '-0.2004394705']
NbSeZr 5 ['Se0.444444Zr0.444444Nb0.111111', '-1.209', '-0.200389848765']
NbSiTc 6 ['Si0.444444Nb0.111111Tc0.444444', '-0.667', '-0.209772712014']
NbSnU 4 ['Nb0.6Sn0.3U0.1', '-0.397', '-0.214346625445']
NdNiO 3 ['O0.6Ni0.2Nd0.2', '-2.634', '-0.263890664186']
NdNiSb 1 ['Ni0.3Sb0.4Nd0.3', '-1.032', '-0.2254177675']
NdNiSe 4 ['Ni0.2Se0.5Nd0.3', '-1.589', '-0.202357962001']
NdNiSi 1 ['Si0.571429Ni0.285714Nd0.142857', '-0.871', '-0.251381745']
NdNpSe 1 ['Se0.666667Nd0.111111Np0.222222', '-1.365', '-0.209753620094']
NdOP 15 ['O0.333333P0.222222Nd0.444444', '-2.954', '-0.201940839103']
NdOPa 7 ['O0.7Nd0.2Pa0.1', '-3.068', '-0.264855828974']
NdOPd 3 ['O0.625Pd0.125Nd0.25', '-2.795', '-0.255956937891']
NdOPu 1 ['O0.666667Nd0.222222Pu0.111111', '-3.37', '-0.2013831622']
NdORe 1 ['O0.625Nd0.25Re0.125', '-3.168', '-0.253181271896']
NdORh 1 ['O0.6Rh0.2Nd0.2', '-2.601', '-0.26486759447']
NdORu 1 ['O0.6Ru0.2Nd0.2', '-2.71', '-0.329920352971']
NdOS 7 ['O0.666667S0.111111Nd0.222222', '-3.112', '-0.214075214893']
NdOSc 1 ['O0.625Sc0.125Nd0.25', '-3.742', '-0.2360941732']
NdOSe 9 ['O0.25Se0.375Nd0.375', '-2.768', '-0.20664917072']
NdOSi 2 ['O0.666667Si0.111111Nd0.222222', '-3.427', '-0.279899626453']
NdOSm 1 ['O0.625Nd0.25Sm0.125', '-3.752', '-0.293104163278']
NdOTa 1 ['O0.6Nd0.3Ta0.1', '-3.733', '-0.287173417519']
NdOTb 1 ['O0.625Nd0.25Tb0.125', '-3.678', '-0.263092664884']
NdOTc 5 ['O0.666667Tc0.222222Nd0.111111', '-2.466', '-0.257291323531']
NdOTe 1 ['O0.6Te0.1Nd0.3', '-3.311', '-0.256512241931']
NdOTi 1 ['O0.6Ti0.2Nd0.2', '-3.659', '-0.240384272218']
NdOTm 3 ['O0.6Nd0.2Tm0.2', '-4.017', '-0.24086374397']

NdOV 1 ['O0.6V0.2Nd0.2', '-3.558', '-0.36203213292']
NdOW 2 ['O0.666667Nd0.166667W0.166667', '-3.202', '-0.203314563163']
NdOZn 5 ['O0.666667Zn0.166667Nd0.166667', '-2.339', '-0.228111806228']
NdOZr 1 ['O0.6Zr0.2Nd0.2', '-3.795', '-0.307533575199']
NdOsSe 3 ['Se0.555556Nd0.222222Os0.222222', '-1.332', '-0.211666361808']
NdPPm 3 ['P0.5Nd0.125Pm0.375', '-1.539', '-0.228524188957']
NdPPt 6 ['P0.2Nd0.2Pt0.6', '-1.129', '-0.209372648456']
NdPPu 12 ['P0.5Nd0.25Pu0.25', '-1.686', '-0.20765333677']
NdPRh 7 ['P0.4Rh0.3Nd0.3', '-1.547', '-0.241839774293']
NdPSe 2 ['P0.125Se0.5Nd0.375', '-1.86', '-0.200874674629']
NdPTe 2 ['P0.5Te0.375Nd0.125', '-0.755', '-0.20835368977']
NdPaS 3 ['S0.666667Nd0.166667Pa0.166667', '-1.877', '-0.227335708489']
NdPaSe 10 ['Se0.6Nd0.3Pa0.1', '-1.832', '-0.204552891001']
NdPaSi 2 ['Si0.7Nd0.1Pa0.2', '-0.525', '-0.2223706405']
NdPdS 1 ['S0.428571Pd0.285714Nd0.285714', '-1.827', '-0.227279351493']
NdPdSe 26 ['Se0.2Pd0.6Nd0.2', '-1.008', '-0.200124880499']
NdPmS 5 ['S0.5Nd0.166667Pm0.333333', '-2.179', '-0.207861936323']
NdPmSe 9 ['Se0.555556Nd0.111111Pm0.333333', '-1.827', '-0.201460699723']
NdPtS 1 ['S0.375Nd0.25Pt0.375', '-1.619', '-0.20223869984']
NdPtSe 24 ['Se0.571429Nd0.142857Pt0.285714', '-1.101', '-0.213369136668']
NdPtSi 2 ['Si0.5Nd0.1Pt0.4', '-1.027', '-0.203286962']
NdPtTh 2 ['Nd0.111111Pt0.777778Th0.111111', '-0.948', '-0.207587106667']
NdPuS 1 ['S0.428571Nd0.285714Pu0.285714', '-2.006', '-0.216225978277']
NdPuTe 1 ['Te0.5Nd0.1Pu0.4', '-1.415', '-0.24613320483']
NdRbTe 2 ['Rb0.222222Te0.555556Nd0.222222', '-1.477', '-0.215664916717']
NdReS 3 ['S0.5Nd0.3Re0.2', '-1.95', '-0.214135493398']
NdReSi 3 ['Si0.428571Nd0.142857Re0.428571', '-0.728', '-0.248742806429']
NdRhS 4 ['S0.4Rh0.2Nd0.4', '-1.955', '-0.203361664143']
NdRhSe 21 ['Se0.333333Rh0.222222Nd0.444444', '-1.664', '-0.216675306111']
NdRhSi 6 ['Si0.6Rh0.3Nd0.1', '-0.917', '-0.20378896625']
NdRuS 2 ['S0.666667Ru0.111111Nd0.222222', '-1.742', '-0.204725203433']
NdRuSe 6 ['Se0.5Ru0.2Nd0.3', '-1.594', '-0.207357962001']
NdRuSi 3 ['Si0.555556Ru0.333333Nd0.111111', '-0.926', '-0.208898320278']
NdSSb 5 ['S0.4Sb0.2Nd0.4', '-2.023', '-0.208508877227']
NdSSe 6 ['S0.166667Se0.5Nd0.333333', '-1.92', '-0.208114748916']
NdSSi 20 ['Si0.166667S0.5Nd0.333333', '-2.09', '-0.223659243408']
NdSTc 11 ['S0.5Tc0.25Nd0.25', '-1.752', '-0.200910229555']
NdSTl 2 ['S0.4Nd0.4Tl0.2', '-1.939', '-0.200827308392']
NdSTm 1 ['S0.555556Nd0.333333Tm0.111111', '-2.792', '-0.577995411309']
NdSZr 7 ['S0.555556Zr0.222222Nd0.222222', '-2.136', '-0.229814650268']
NdSbSe 4 ['Se0.375Sb0.25Nd0.375', '-1.654', '-0.205172971796']
NdScSe 6 ['Sc0.333333Se0.555556Nd0.111111', '-1.821', '-0.213439665557']
NdSeSi 12 ['Si0.222222Se0.444444Nd0.333333', '-1.649', '-0.202230747221']
NdSeTc 4 ['Se0.571429Tc0.285714Nd0.142857', '-0.938', '-0.237432500716']

NdSeTe 8 ['Se0.5Te0.2Nd0.3', '-1.656', '-0.216662033334']
NdSeTm 4 ['Se0.571429Nd0.142857Tm0.285714', '-1.884', '-0.200822195477']
NdSeY 8 ['Se0.571429Y0.285714Nd0.142857', '-1.945', '-0.235346085001']
NdTeY 1 ['Y0.3Te0.6Nd0.1', '-1.488', '-0.204572945375']
NiNpO 2 ['O0.6Ni0.3Np0.1', '-2.078', '-0.219969574304']
NiNpP 3 ['P0.75Ni0.125Np0.125', '-0.709', '-0.203000907811']
NiNpS 1 ['S0.4Ni0.2Np0.4', '-1.442', '-0.235196450896']
NiOPa 4 ['O0.625Ni0.25Pa0.125', '-2.027', '-0.244193409825']
NiOPu 2 ['O0.6Ni0.3Pu0.1', '-1.977', '-0.243967185886']
NiOS 1 ['O0.6S0.1Ni0.3', '-1.68', '-0.208922271638']
NiOSe 1 ['O0.6Ni0.3Se0.1', '-1.303', '-0.231540979381']
NiOSr 1 ['O0.5Ni0.166667Sr0.333333', '-2.579', '-0.202422715987']
NiOTe 1 ['O0.666667Ni0.111111Te0.222222', '-1.565', '-0.303358365642']
NiOTh 1 ['O0.666667Ni0.111111Th0.222222', '-3.314', '-0.213499161298']
NiOsSc 3 ['Sc0.625Ni0.125Os0.25', '-0.546', '-0.214034192812']
NiOsTa 2 ['Ni0.125Ta0.625Os0.25', '-0.413', '-0.20194852625']
NiOsTm 3 ['Ni0.111111Tm0.555556Os0.333333', '-0.414', '-0.206593686944']
NiPPm 18 ['P0.285714Ni0.142857Pm0.571429', '-0.962', '-0.202883002261']
NiPSe 2 ['P0.285714Ni0.428571Se0.285714', '-0.638', '-0.20378971125']
NiPSn 1 ['P0.8Ni0.1Sn0.1', '-0.507', '-0.224561935939']
NiPSr 1 ['P0.8Ni0.1Sr0.1', '-0.789', '-0.212698103131']
NiPTa 24 ['P0.25Ni0.625Ta0.125', '-0.763', '-0.200800854947']
NiPTe 6 ['P0.222222Ni0.444444Te0.333333', '-0.609', '-0.200101648704']
NiPTi 5 ['P0.3Ti0.5Ni0.2', '-1.277', '-0.211987264833']
NiPXe 3 ['P0.75Ni0.125Xe0.125', '-0.456', '-0.212720811015']
NiPYb 4 ['P0.4Ni0.1Yb0.5', '-1.071', '-0.201140790417']
NiPZn 10 ['P0.222222Ni0.444444Zn0.333333', '-0.614', '-0.203982598379']
NiPaS 4 ['S0.4Ni0.3Pa0.3', '-1.375', '-0.206105957893']
NiPaSe 28 ['Ni0.3Se0.6Pa0.1', '-0.652', '-0.200684738875']
NiPdPm 4 ['Ni0.142857Pd0.714286Pm0.142857', '-0.434', '-0.211392846786']
NiPmS 14 ['S0.3Ni0.3Pm0.4', '-1.425', '-0.202307647295']
NiPmSe 44 ['Ni0.25Se0.625Pm0.125', '-0.791', '-0.203266485312']
NiPmSn 7 ['Ni0.111111Sn0.777778Pm0.111111', '-0.427', '-0.209939835353']
NiPrSe 5 ['Ni0.2Se0.5Pr0.3', '-1.607', '-0.200715461456']
NiPrSi 8 ['Si0.375Ni0.125Pr0.5', '-0.84', '-0.201523102266']
NiPtTh 4 ['Ni0.125Pt0.75Th0.125', '-0.548', '-0.207194139375']
NiPuS 4 ['S0.428571Ni0.142857Pu0.428571', '-1.847', '-0.202333702563']
NiRbS 6 ['S0.444444Ni0.333333Rb0.222222', '-0.947', '-0.200847422291']
NiSSc 2 ['S0.4Sc0.4Ni0.2', '-1.831', '-0.209422753321']
NiSSe 13 ['S0.2Ni0.2Se0.6', '-0.429', '-0.206733701822']
NiSSm 1 ['S0.5Ni0.1Sm0.4', '-2.2', '-0.208695166091']
NiSTi 1 ['S0.444444Ti0.333333Ni0.222222', '-1.494', '-0.26937583904']
NiSXe 5 ['S0.444444Ni0.111111Xe0.444444', '-0.356', '-0.210231587164']
NiSZr 8 ['S0.375Ni0.125Zr0.5', '-1.434', '-0.202996091618']

NiSbU 2 ['Ni0.3Sb0.4U0.3', '-0.471', '-0.201931865816']
NiScSe 15 ['Sc0.2Ni0.4Se0.4', '-0.993', '-0.214135133']
NiSeTb 1 ['Ni0.375Se0.375Tb0.25', '-1.236', '-0.224566679456']
NiSeTc 6 ['Ni0.25Se0.5Tc0.25', '-0.39', '-0.207511535625']
NiSeTi 7 ['Ti0.2Ni0.4Se0.4', '-0.877', '-0.202910507075']
NiSeTm 21 ['Ni0.2Se0.4Tm0.4', '-1.53', '-0.204900988']
NiSeU 4 ['Ni0.3Se0.5U0.2', '-0.955', '-0.2029467412']
NiSeY 25 ['Ni0.111111Se0.666667Y0.222222', '-1.1', '-0.234049025833']
NiSeZn 14 ['Ni0.5Zn0.166667Se0.333333', '-0.565', '-0.207718293333']
NiSeZr 7 ['Ni0.125Se0.5Zr0.375', '-1.329', '-0.205996717245']
NiSiSm 4 ['Si0.333333Ni0.333333Sm0.333333', '-0.974', '-0.238728899028']
NiSiTm 2 ['Si0.375Ni0.25Tm0.375', '-0.998', '-0.215531548558']
NiSiY 6 ['Si0.333333Ni0.222222Y0.444444', '-0.971', '-0.202419285666']
NiSnTm 2 ['Ni0.333333Sn0.333333Tm0.333333', '-0.764', '-0.2161908397']
NpOP 24 ['O0.444444P0.222222Np0.333333', '-2.902', '-0.201816158148']
NpOPa 3 ['O0.7Pa0.1Np0.2', '-3.36', '-0.204114750324']
NpORb 3 ['O0.625Rb0.125Np0.25', '-3.406', '-0.22876550395']
NpORh 2 ['O0.333333Rh0.111111Np0.555556', '-2.231', '-0.24470961885']
NpOS 17 ['O0.714286S0.142857Np0.142857', '-2.596', '-0.202301967263']
NpOSc 2 ['O0.666667Sc0.111111Np0.222222', '-3.764', '-0.232785970408']
NpOSe 6 ['O0.333333Se0.111111Np0.555556', '-2.371', '-0.201076224127']
NpOSi 4 ['O0.333333Si0.166667Np0.5', '-2.211', '-0.212990312982']
NpOSr 3 ['O0.6Sr0.1Np0.3', '-3.677', '-0.22758317']
NpOTa 1 ['O0.666667Ta0.222222Np0.111111', '-3.385', '-0.200495627608']
NpOTi 2 ['O0.428571Ti0.142857Np0.428571', '-2.714', '-0.208741791636']
NpOTl 2 ['O0.3Tl0.1Np0.6', '-1.946', '-0.20559266859']
NpOTm 1 ['O0.666667Tm0.222222Np0.111111', '-3.754', '-0.332838888872']
NpOV 1 ['O0.625V0.25Np0.125', '-3.393', '-0.357549877725']
NpOZr 1 ['O0.7Zr0.1Np0.2', '-3.648', '-0.331860074823']
NpOsTm 4 ['Tm0.3Os0.6Np0.1', '-0.295', '-0.214365847']
NpPPb 4 ['P0.5Pb0.375Np0.125', '-0.471', '-0.208280096796']
NpPPm 5 ['P0.571429Pm0.285714Np0.142857', '-1.194', '-0.22167490863']
NpPRh 17 ['P0.333333Rh0.444444Np0.222222', '-1.01', '-0.200794923735']
NpPRu 1 ['P0.3Ru0.6Np0.1', '-0.698', '-0.208922879937']
NpPS 1 ['P0.4S0.3Np0.3', '-1.242', '-0.209211614232']
NpPSe 13 ['P0.2Se0.4Np0.4', '-1.23', '-0.202092999709']
NpPSn 9 ['P0.714286Sn0.142857Np0.142857', '-0.618', '-0.201235750657']
NpPTa 6 ['P0.5Ta0.333333Np0.166667', '-0.98', '-0.205420942395']
NpPTc 2 ['P0.5Tc0.3Np0.2', '-0.966', '-0.204764075627']
NpPTe 14 ['P0.6Te0.2Np0.2', '-0.621', '-0.200648154874']
NpPTl 21 ['P0.571429Tl0.142857Np0.285714', '-0.856', '-0.211075553237']
NpPXe 12 ['P0.6Xe0.2Np0.2', '-0.622', '-0.201648154874']
NpPaPt 1 ['Pt0.8Pa0.1Np0.1', '-0.695', '-0.218251363625']
NpPaS 1 ['S0.666667Pa0.222222Np0.111111', '-1.586', '-0.200346992965']

NpPaSe 13 ['Se0.555556Pa0.333333Np0.111111', '-1.315', '-0.207136156436']
NpPbPt 2 ['Pt0.777778Pb0.111111Np0.111111', '-0.345', '-0.203542370694']
NpPbSe 2 ['Se0.625Pb0.125Np0.25', '-1.032', '-0.20310759573']
NpPdPt 1 ['Pd0.125Pt0.75Np0.125', '-0.371', '-0.226450077239']
NpPdSe 1 ['Se0.6Pd0.1Np0.3', '-1.115', '-0.206959698876']
NpPmS 5 ['S0.5Pm0.4Np0.1', '-2.05', '-0.201449111991']
NpPmSe 18 ['Se0.7Pm0.1Np0.2', '-1.078', '-0.204479467334']
NpPmTe 1 ['Te0.6Pm0.3Np0.1', '-1.226', '-0.256687874706']
NpPtS 16 ['S0.25Pt0.375Np0.375', '-1.089', '-0.214056290298']
NpPtSc 1 ['Sc0.1Pt0.8Np0.1', '-0.736', '-0.226390211125']
NpPtSe 22 ['Se0.555556Pt0.222222Np0.222222', '-0.976', '-0.201662471729']
NpPtTb 1 ['Tb0.1Pt0.8Np0.1', '-0.674', '-0.248087378158']
NpPtTh 5 ['Pt0.714286Th0.142857Np0.142857', '-0.716', '-0.237151547321']
NpPtTm 2 ['Tm0.111111Pt0.777778Np0.111111', '-0.804', '-0.22632264625']
NpPtY 1 ['Y0.1Pt0.8Np0.1', '-0.709', '-0.225618634125']
NpPtYb 3 ['Yb0.125Pt0.75Np0.125', '-0.75', '-0.22625756453']
NpPtZr 1 ['Zr0.1Pt0.8Np0.1', '-0.759', '-0.260061068625']
NpRbS 4 ['S0.5Rb0.1Np0.4', '-1.739', '-0.25703753187']
NpRbSe 21 ['Se0.5Rb0.166667Np0.333333', '-1.338', '-0.207792448749']
NpReSe 3 ['Se0.375Re0.125Np0.5', '-1.003', '-0.206034749215']
NpRhS 13 ['S0.444444Rh0.333333Np0.222222', '-1.247', '-0.206963378493']
NpRhSe 40 ['Se0.6Rh0.3Np0.1', '-0.761', '-0.200506129042']
NpRhSi 2 ['Si0.5Rh0.375Np0.125', '-0.899', '-0.232747784766']
NpRuSe 24 ['Se0.333333Ru0.166667Np0.5', '-0.934', '-0.204307897149']
NpSSc 10 ['S0.5Sc0.333333Np0.166667', '-2.05', '-0.2020857782']
NpSSe 4 ['S0.3Se0.3Np0.4', '-1.592', '-0.214550557014']
NpSSi 4 ['Si0.2S0.4Np0.4', '-1.447', '-0.230404553061']
NpSSr 2 ['S0.5Sr0.166667Np0.333333', '-1.995', '-0.224304815077']
NpSTc 1 ['S0.5Tc0.125Np0.375', '-1.617', '-0.211608311899']
NpSTl 10 ['S0.285714Tl0.285714Np0.428571', '-1.09', '-0.227997464926']
NpScSe 16 ['Sc0.2Se0.5Np0.3', '-1.479', '-0.203823098372']
NpSeSr 3 ['Se0.625Sr0.125Np0.25', '-1.409', '-0.20419811073']
NpSeTa 2 ['Se0.666667Ta0.166667Np0.166667', '-1.093', '-0.205539384931']
NpSeTc 14 ['Se0.7Tc0.2Np0.1', '-0.475', '-0.200337753792']
NpSeTe 7 ['Se0.625Te0.125Np0.25', '-0.889', '-0.20234438448']
NpSeTh 7 ['Se0.555556Th0.222222Np0.222222', '-1.597', '-0.200669941386']
NpSeTl 25 ['Se0.375Tl0.125Np0.5', '-1.016', '-0.219034749215']
NpSeTm 1 ['Se0.6Tm0.3Np0.1', '-1.679', '-0.20640304146']
NpSeXe 3 ['Se0.5Xe0.3Np0.2', '-0.76', '-0.210675507584']
NpSeY 15 ['Se0.7Y0.2Np0.1', '-1.202', '-0.220977262792']
NpSeZn 19 ['Zn0.142857Se0.428571Np0.428571', '-1.113', '-0.202182570531']
NpSeZr 10 ['Se0.5Zr0.4Np0.1', '-1.336', '-0.211390428338']
NpSiTc 8 ['Si0.4Tc0.4Np0.2', '-0.633', '-0.21361818325']
OOsP 2 ['O0.7P0.1Os0.2', '-1.806', '-0.232039897112']

OOsRb 2 ['O0.625Rb0.25Os0.125', '-1.648', '-0.253126835073']
OOsS 20 ['O0.4S0.3Os0.3', '-1.146', '-0.211198487411']
OOsSe 1 ['O0.666667Se0.111111Os0.222222', '-1.368', '-0.276012472467']
OOsSr 3 ['O0.666667Sr0.222222Os0.111111', '-2.235', '-0.223464881082']
OOsYb 1 ['O0.666667Yb0.111111Os0.222222', '-1.964', '-0.203254578857']
OPPa 8 ['O0.75P0.125Pa0.125', '-2.374', '-0.204417086511']
OPPb 4 ['O0.5P0.3Pb0.2', '-1.995', '-0.206447060629']
OPPm 47 ['O0.7P0.2Pm0.1', '-2.732', '-0.200788213574']
OPPr 20 ['O0.7P0.2Pr0.1', '-3.051', '-0.207830886764']
OPPu 33 ['O0.222222P0.333333Pu0.444444', '-2.311', '-0.20098198716']
OPRb 7 ['O0.4P0.3Rb0.3', '-2.199', '-0.255451847489']
OPRu 1 ['O0.7P0.2Ru0.1', '-2.198', '-0.206036917093']
OPS 8 ['O0.625P0.125S0.25', '-1.863', '-0.23367836215']
OPSc 2 ['O0.6P0.1Sc0.3', '-3.655', '-0.213727808033']
OPSe 6 ['O0.75P0.125Se0.125', '-1.578', '-0.213051705574']
OPSm 20 ['O0.222222P0.444444Sm0.333333', '-2.283', '-0.203690327629']
OPSr 16 ['O0.444444P0.111111Sr0.444444', '-2.915', '-0.200896419855']
OPTa 1 ['O0.666667P0.111111Ta0.222222', '-3.169', '-0.319410615014']
OPTb 1 ['O0.375P0.25Tb0.375', '-2.812', '-0.212268070293']
OPTc 1 ['O0.7P0.2Tc0.1', '-2.331', '-0.208799672342']
OPTe 1 ['O0.6P0.3Te0.1', '-2.17', '-0.235536934923']
OPTh 1 ['O0.375P0.125Th0.5', '-3.031', '-0.22782537613']
OPTi 2 ['O0.666667P0.222222Ti0.111111', '-2.797', '-0.219836202426']
OPTl 7 ['O0.333333P0.333333Tl0.333333', '-1.461', '-0.20632827789']
OPTm 1 ['O0.25P0.375Tm0.375', '-2.606', '-0.208825105586']
OPU 2 ['O0.714286P0.142857U0.142857', '-3.269', '-0.24912555032']
OPY 24 ['O0.444444P0.111111Y0.444444', '-3.42', '-0.209347629167']
OPYb 8 ['O0.666667P0.222222Yb0.111111', '-2.804', '-0.2026158684']
OPZn 2 ['O0.5P0.125Zn0.375', '-2.021', '-0.218440734374']
OPaPb 5 ['O0.666667Pb0.222222Pa0.111111', '-1.985', '-0.20902377886']
OPaPd 3 ['O0.666667Pd0.166667Pa0.166667', '-2.047', '-0.240814945392']
OPaPm 3 ['O0.666667Pm0.111111Pa0.222222', '-3.331', '-0.243845178281']
OPaPr 4 ['O0.666667Pr0.222222Pa0.111111', '-3.379', '-0.359361679371']
OPaPt 2 ['O0.7Pt0.1Pa0.2', '-2.297', '-0.215459546472']
OPaPu 3 ['O0.7Pa0.1Pu0.2', '-3.105', '-0.218857872984']
OPaRb 1 ['O0.666667Rb0.111111Pa0.222222', '-3.105', '-0.44589439701']
OPaRh 1 ['O0.7Rh0.1Pa0.2', '-2.407', '-0.236379581473']
OPaRu 1 ['O0.7Ru0.1Pa0.2', '-2.526', '-0.260061422259']
OPaS 30 ['O0.125S0.625Pa0.25', '-1.61', '-0.217075542273']
OPaSb 4 ['O0.7Sb0.2Pa0.1', '-2.289', '-0.274288838882']
OPaSc 4 ['O0.666667Sc0.222222Pa0.111111', '-3.555', '-0.40685009451']
OPaSe 19 ['O0.25Se0.375Pa0.375', '-1.966', '-0.201937255405']
OPaSi 5 ['O0.666667Si0.111111Pa0.222222', '-3.318', '-0.22761456497']
OPaSm 4 ['O0.666667Sm0.222222Pa0.111111', '-3.441', '-0.351938421038']

OPaSn 4 ['O0.714286Sn0.142857Pa0.142857', '-2.42', '-0.254734863517']
OPaSr 7 ['O0.6Sr0.3Pa0.1', '-3.076', '-0.24915353047']
OPaTa 5 ['O0.666667Ta0.166667Pa0.166667', '-3.211', '-0.200823796246']
OPaTb 3 ['O0.666667Tb0.111111Pa0.222222', '-3.389', '-0.334170011376']
OPaTc 1 ['O0.7Tc0.1Pa0.2', '-2.777', '-0.2827014493']
OPaTe 7 ['O0.7Te0.2Pa0.1', '-1.983', '-0.207965371215']
OPaTh 2 ['O0.714286Th0.142857Pa0.142857', '-3.533', '-0.389122676463']
OPaTi 6 ['O0.666667Ti0.222222Pa0.111111', '-3.44', '-0.24530601246']
OPaTl 1 ['O0.666667Tl0.111111Pa0.222222', '-2.905', '-0.448170108954']
OPaTm 4 ['O0.666667Tm0.222222Pa0.111111', '-3.531', '-0.313708382566']
OPaU 2 ['O0.7Pa0.2U0.1', '-3.375', '-0.263299047756']
OPaV 5 ['O0.7V0.2Pa0.1', '-2.748', '-0.233961910831']
OPaW 4 ['O0.666667W0.111111Pa0.222222', '-3.023', '-0.23154132219']
OPaXe 2 ['O0.625Xe0.125Pa0.25', '-2.655', '-0.33089986581']
OPaY 4 ['O0.666667Y0.111111Pa0.222222', '-3.467', '-0.343301447239']
OPaYb 5 ['O0.6Yb0.3Pa0.1', '-3.327', '-0.269883108559']
OPaZn 5 ['O0.625Zn0.25Pa0.125', '-2.295', '-0.308497788935']
OPaZr 2 ['O0.714286Zr0.142857Pa0.142857', '-3.118', '-0.23295031021']
OPbPr 1 ['O0.428571Pr0.428571Pb0.142857', '-2.908', '-0.214219632419']
OPbS 5 ['O0.4S0.2Pb0.4', '-1.483', '-0.201734567723']
OPbSe 7 ['O0.428571Se0.142857Pb0.428571', '-1.395', '-0.218270896052']
OPbSr 6 ['O0.222222Sr0.555556Pb0.222222', '-1.838', '-0.204040198136']
OPbTc 3 ['O0.666667Tc0.222222Pb0.111111', '-1.809', '-0.242941026819']
OPbTe 1 ['O0.666667Te0.222222Pb0.111111', '-1.573', '-0.267324276033']
OPbU 1 ['O0.666667Pb0.111111U0.222222', '-3.244', '-0.22997360135']
OPdTl 2 ['O0.333333Pd0.111111Tl0.555556', '-0.916', '-0.219346671837']
OPdYb 1 ['O0.625Pd0.25Yb0.125', '-1.55', '-0.204040172059']
OPmS 24 ['O0.25S0.375Pm0.375', '-2.893', '-0.20576590816']
OPmSb 4 ['O0.2Sb0.3Pm0.5', '-1.984', '-0.201828525823']
OPmSe 28 ['O0.25Se0.25Pm0.5', '-2.567', '-0.224577845717']
OPmTe 16 ['O0.333333Te0.166667Pm0.5', '-2.682', '-0.204404829149']
OPmTi 2 ['O0.4Ti0.2Pm0.4', '-2.707', '-0.242466952848']
OPrPt 1 ['O0.666667Pr0.222222Pt0.111111', '-2.505', '-0.271339132149']
OPrRb 1 ['O0.5Rb0.25Pr0.25', '-2.885', '-0.257100145652']
OPrRh 1 ['O0.666667Rh0.222222Pr0.111111', '-1.905', '-0.219780178006']
OPrS 16 ['O0.428571S0.142857Pr0.428571', '-3.396', '-0.20580653804']
OPrSb 2 ['O0.4Sb0.1Pr0.5', '-2.845', '-0.20066584673']
OPrSc 1 ['O0.444444Sc0.111111Pr0.444444', '-3.026', '-0.296505069376']
OPrSe 15 ['O0.428571Se0.285714Pr0.285714', '-2.759', '-0.204665093014']
OPrSi 1 ['O0.666667Si0.166667Pr0.166667', '-3.249', '-0.222193330633']
OPrTa 1 ['O0.666667Pr0.111111Ta0.222222', '-3.557', '-0.252396662362']
OPrTc 3 ['O0.625Tc0.125Pr0.25', '-3.182', '-0.309272239604']
OPrTe 3 ['O0.3Te0.3Pr0.4', '-2.837', '-0.214598554486']
OPrTi 2 ['O0.625Ti0.25Pr0.125', '-3.611', '-0.281410311319']

OPtPu 1 ['O0.166667Pt0.333333Pu0.5', '-1.758', '-0.201707188197']
OPtRb 3 ['O0.444444Rb0.444444Pt0.111111', '-1.286', '-0.217419417387']
OPtSr 1 ['O0.111111Sr0.555556Pt0.333333', '-1.35', '-0.214540899347']
OPuRb 2 ['O0.444444Rb0.444444Pu0.111111', '-2.074', '-0.20367429406']
OPuS 31 ['O0.25S0.25Pu0.5', '-2.786', '-0.217218910041']
OPuSe 27 ['O0.111111Se0.333333Pu0.555556', '-1.923', '-0.202026712411']
OPuSr 1 ['O0.666667Sr0.111111Pu0.222222', '-3.158', '-0.221288464522']
OPuTa 2 ['O0.7Ta0.2Pu0.1', '-3.45', '-0.280584791889']
OPuTe 10 ['O0.333333Te0.166667Pu0.5', '-2.615', '-0.20738608917']
ORbRe 1 ['O0.5Rb0.375Re0.125', '-1.821', '-0.208564058641']
ORbRh 2 ['O0.6Rb0.1Rh0.3', '-1.279', '-0.219907583973']
ORbRu 4 ['O0.666667Rb0.111111Ru0.222222', '-1.485', '-0.225471214412']
ORbS 9 ['O0.142857S0.428571Rb0.428571', '-1.517', '-0.201375915536']
ORbSb 6 ['O0.4Rb0.4Sb0.2', '-1.626', '-0.217212504315']
ORbSe 23 ['O0.333333Se0.111111Rb0.555556', '-1.43', '-0.205861272795']
ORbSn 2 ['O0.5Rb0.333333Sn0.166667', '-1.945', '-0.20975788342']
ORbTb 1 ['O0.5Rb0.25Tb0.25', '-2.829', '-0.21097598324']
ORbTc 3 ['O0.5Rb0.333333Tc0.166667', '-1.734', '-0.202341798558']
ORbTe 5 ['O0.285714Rb0.571429Te0.142857', '-1.425', '-0.205499098083']
ORbTi 1 ['O0.444444Ti0.222222Rb0.333333', '-2.581', '-0.252718054602']
ORbTl 31 ['O0.375Rb0.5Tl0.125', '-1.338', '-0.207983309174']
ORbV 4 ['O0.428571V0.142857Rb0.428571', '-1.947', '-0.255204293157']
ORbZr 3 ['O0.5Rb0.3Zr0.2', '-2.783', '-0.20239248206']
OReS 9 ['O0.625S0.125Re0.25', '-1.906', '-0.218929346623']
OReYb 1 ['O0.666667Yb0.111111Re0.222222', '-2.432', '-0.238249336359']
ORhTb 1 ['O0.666667Rh0.222222Tb0.111111', '-1.892', '-0.211169439156']
ORhYb 1 ['O0.625Rh0.25Yb0.125', '-1.955', '-0.244200490075']
ORuS 4 ['O0.625S0.125Ru0.25', '-1.454', '-0.20218992676']
OSSb 3 ['O0.666667S0.166667Sb0.166667', '-1.679', '-0.214264206532']
OSSc 7 ['O0.6S0.2Sc0.2', '-2.891', '-0.227026530081']
OSSe 3 ['O0.666667S0.166667Se0.166667', '-1.376', '-0.200279659785']
OSSi 3 ['O0.666667Si0.222222S0.111111', '-2.683', '-0.208207123885']
OSSm 12 ['O0.625S0.25Sm0.125', '-2.22', '-0.217531597973']
OSSn 9 ['O0.25S0.375Sn0.375', '-1.316', '-0.200603264455']
OSTa 7 ['O0.666667S0.222222Ta0.111111', '-2.199', '-0.229778751503']
OSTb 12 ['O0.5S0.25Tb0.25', '-2.715', '-0.204205261448']
OSTc 6 ['O0.666667S0.111111Tc0.222222', '-1.846', '-0.280840628375']
OSTe 22 ['O0.6S0.2Te0.2', '-1.481', '-0.200080797061']
OSTh 19 ['O0.428571S0.285714Th0.285714', '-3.482', '-0.205314048678']
OSTi 7 ['O0.166667S0.166667Ti0.666667', '-1.807', '-0.20051729888']
OSTm 4 ['O0.714286S0.142857Tm0.142857', '-2.427', '-0.219246820109']
OSU 9 ['O0.625S0.125U0.25', '-3.37', '-0.220141401078']
OSV 3 ['O0.5S0.2V0.3', '-2.321', '-0.20178210134']
OSW 7 ['O0.625S0.125W0.25', '-2.375', '-0.204022106015']

OSY 13 ['O0.555556S0.222222Y0.222222', '-2.792', '-0.223238654391']
OSYb 4 ['O0.714286S0.142857Yb0.142857', '-2.008', '-0.202103016423']
OSZr 11 ['O0.25S0.375Zr0.375', '-2.589', '-0.200167505772']
OSbSc 3 ['O0.333333Sc0.555556Sb0.111111', '-2.599', '-0.221949484706']
OSbSr 1 ['O0.4Sr0.4Sb0.2', '-2.56', '-0.204712477648']
OSbTb 1 ['O0.7Sb0.2Tb0.1', '-2.199', '-0.223862010148']
OSbTi 26 ['O0.333333Ti0.555556Sb0.111111', '-2.213', '-0.208777529364']
OSbTm 3 ['O0.142857Sb0.285714Tm0.571429', '-1.798', '-0.206424944279']
OSbYb 2 ['O0.666667Sb0.222222Yb0.111111', '-2.194', '-0.2087411433']
OScSe 42 ['O0.625Sc0.25Se0.125', '-2.997', '-0.205129028617']
OScSi 4 ['O0.4Si0.1Sc0.5', '-2.953', '-0.209584376313']
OScSn 1 ['O0.25Sc0.5Sn0.25', '-2.192', '-0.205740902441']
OScTe 5 ['O0.1Sc0.5Te0.4', '-1.777', '-0.201207870965']
OScTl 2 ['O0.25Sc0.5Tl0.25', '-1.955', '-0.225954427696']
OScU 1 ['O0.666667Sc0.222222U0.111111', '-3.727', '-0.220204770937']
OSeSm 22 ['O0.555556Se0.222222Sm0.222222', '-2.583', '-0.209169193538']
OSeSr 4 ['O0.375Se0.25Sr0.375', '-2.528', '-0.206356603627']
OSeTb 13 ['O0.625Se0.25Tb0.125', '-1.946', '-0.207939362931']
OSeTc 5 ['O0.111111Se0.555556Tc0.333333', '-0.505', '-0.221584570318']
OSeTe 1 ['O0.2Se0.4Te0.4', '-0.59', '-0.200099104241']
OSeTh 3 ['O0.285714Se0.285714Th0.428571', '-2.95', '-0.24377573368']
OSeTi 9 ['O0.2Ti0.6Se0.2', '-1.868', '-0.20445153855']
OSeTl 35 ['O0.25Se0.5Tl0.25', '-0.735', '-0.200076075561']
OSeTm 13 ['O0.666667Se0.166667Tm0.166667', '-2.316', '-0.201177400945']
OSeU 5 ['O0.625Se0.125U0.25', '-3.285', '-0.241818180536']
OSeY 37 ['O0.6Se0.1Y0.3', '-3.28', '-0.209404960783']
OSeZr 14 ['O0.2Se0.2Zr0.6', '-1.873', '-0.203543613456']
OSiSr 5 ['O0.333333Si0.166667Sr0.5', '-2.337', '-0.217478561373']
OSiTe 1 ['O0.7Si0.1Te0.2', '-1.998', '-0.230538640716']
OSiTh 1 ['O0.7Si0.2Th0.1', '-3.524', '-0.391303718351']
OSiTm 1 ['O0.666667Si0.166667Tm0.166667', '-3.409', '-0.204168912614']
OSiU 2 ['O0.7Si0.2U0.1', '-3.302', '-0.205445586758']
OSiYb 1 ['O0.5Si0.2Yb0.3', '-3.359', '-0.316155699559']
OSnSr 3 ['O0.5Sr0.4Sn0.1', '-2.908', '-0.215932659844']
OSnTi 13 ['O0.222222Ti0.666667Sn0.111111', '-1.66', '-0.20033452874']
OSnU 1 ['O0.7Sn0.1U0.2', '-3.357', '-0.356100871317']
OSrTc 3 ['O0.666667Sr0.166667Tc0.166667', '-2.346', '-0.279403503023']
OSrTi 3 ['O0.5Ti0.125Sr0.375', '-3.12', '-0.201962789085']
OSrTl 2 ['O0.4Sr0.2Tl0.4', '-1.821', '-0.210372965648']
OSrY 1 ['O0.6Sr0.1Y0.3', '-3.903', '-0.42851180572']
OTaTh 1 ['O0.7Ta0.1Th0.2', '-3.881', '-0.23581799318']
OTbTc 1 ['O0.666667Tc0.166667Tb0.166667', '-2.894', '-0.456250015337']
OTbTe 11 ['O0.333333Te0.222222Tb0.444444', '-2.707', '-0.202141563822']
OTcTl 2 ['O0.625Tc0.125Tl0.25', '-1.573', '-0.275270648577']

OTcYb 2 ['O0.625Tc0.25Yb0.125', '-2.415', '-0.214334734448']
OTeTh 2 ['O0.4Te0.2Th0.4', '-3.192', '-0.205208421108']
OTeTm 1 ['O0.7Te0.2Tm0.1', '-2.16', '-0.30623732252']
OTeU 3 ['O0.7Te0.2U0.1', '-2.3', '-0.213275050967']
OTeY 10 ['O0.285714Y0.428571Te0.285714', '-2.681', '-0.202463288794']
OTeYb 3 ['O0.666667Te0.166667Yb0.166667', '-2.148', '-0.203947897009']
OTeZn 3 ['O0.625Zn0.25Te0.125', '-1.663', '-0.204181147227']
OThU 1 ['O0.714286Th0.142857U0.142857', '-3.82', '-0.21500726044']
OThV 2 ['O0.7V0.1Th0.2', '-3.542', '-0.207652836653']
OTiU 1 ['O0.7Ti0.2U0.1', '-3.409', '-0.210994619998']
OUV 1 ['O0.7V0.1U0.2', '-3.382', '-0.200066782848']
OUZn 1 ['O0.666667Zn0.111111U0.222222', '-3.28', '-0.230406172879']
OVY 1 ['O0.625V0.125Y0.25', '-3.714', '-0.340470583321']
OsPPm 34 ['P0.3Pm0.4Os0.3', '-0.916', '-0.200939046562']
OsPPr 1 ['P0.5Pr0.166667Os0.333333', '-1.11', '-0.21708924927']
OsPPu 9 ['P0.5Os0.375Pu0.125', '-0.912', '-0.201851041145']
OsPSc 8 ['P0.111111Sc0.666667Os0.222222', '-0.732', '-0.205547005256']
OsPSm 1 ['P0.428571Sm0.285714Os0.285714', '-1.328', '-0.208960740446']
OsPSn 2 ['P0.777778Sn0.111111Os0.111111', '-0.536', '-0.208724247039']
OsPSr 7 ['P0.6Sr0.3Os0.1', '-1.186', '-0.206576272626']
OsPTa 3 ['P0.5Ta0.4Os0.1', '-0.931', '-0.21037243377']
OsPTe 7 ['P0.5Te0.333333Os0.166667', '-0.545', '-0.200444225347']
OsPTh 2 ['P0.5Os0.3Th0.2', '-1.224', '-0.24204262302']
OsPTm 2 ['P0.5Tm0.4Os0.1', '-1.689', '-0.20129080002']
OsPXe 1 ['P0.7Xe0.2Os0.1', '-0.422', '-0.215266535208']
OsPY 6 ['P0.571429Y0.142857Os0.285714', '-1.155', '-0.210869870594']
OsPYb 16 ['P0.428571Yb0.428571Os0.142857', '-1.066', '-0.202049564731']
OsPaS 1 ['S0.6Os0.1Pa0.3', '-1.623', '-0.272337796903']
OsPaSe 15 ['Se0.555556Os0.222222Pa0.222222', '-0.848', '-0.221191702083']
OsPdTm 1 ['Pd0.1Tm0.6Os0.3', '-0.481', '-0.210611798']
OsPdV 1 ['V0.7Pd0.1Os0.2', '-0.358', '-0.2158101685']
OsPmS 7 ['S0.428571Pm0.428571Os0.142857', '-1.864', '-0.260843574349']
OsPmSe 23 ['Se0.375Pm0.5Os0.125', '-1.421', '-0.205264849063']
OsPmSi 3 ['Si0.444444Pm0.111111Os0.444444', '-0.552', '-0.20355368125']
OsReTa 1 ['Ta0.666667Re0.222222Os0.111111', '-0.447', '-0.201367750926']
OsReZr 1 ['Zr0.571429Re0.285714Os0.142857', '-0.511', '-0.202839155714']
OsRhSc 3 ['Sc0.6Rh0.1Os0.3', '-0.611', '-0.213817974']
OsRhTa 1 ['Rh0.125Ta0.625Os0.25', '-0.453', '-0.226976372083']
OsRhY 1 ['Y0.6Rh0.1Os0.3', '-0.529', '-0.223896560625']
OsRuSc 2 ['Sc0.666667Ru0.111111Os0.222222', '-0.468', '-0.207432735']
OsRuTm 3 ['Ru0.142857Tm0.571429Os0.285714', '-0.395', '-0.202404482857']
OsRuV 12 ['V0.714286Ru0.142857Os0.142857', '-0.331', '-0.201895724286']
OsSSc 4 ['S0.125Sc0.625Os0.25', '-0.873', '-0.208916802622']
OsSSe 6 ['S0.428571Se0.428571Os0.142857', '-0.437', '-0.200128137068']

OsSXe 6 ['S0.5Xe0.333333Os0.166667', '-0.507', '-0.230649493245']
OsScSe 19 ['Sc0.333333Se0.5Os0.166667', '-1.321', '-0.212482539375']
OsScSi 11 ['Si0.333333Sc0.166667Os0.5', '-0.641', '-0.201090263932']
OsScSn 3 ['Sc0.6Sn0.1Os0.3', '-0.598', '-0.214402799148']
OsScTc 8 ['Sc0.6Tc0.3Os0.1', '-0.3', '-0.2016929675']
OsScTl 3 ['Sc0.625Os0.25Tl0.125', '-0.505', '-0.2408258175']
OsScXe 3 ['Sc0.571429Xe0.142857Os0.285714', '-0.405', '-0.222510378571']
OsScZn 3 ['Sc0.6Zn0.1Os0.3', '-0.538', '-0.224472898']
OsSeTb 2 ['Se0.555556Tb0.222222Os0.222222', '-1.184', '-0.223824214239']
OsSeTc 6 ['Se0.625Tc0.125Os0.25', '-0.382', '-0.244471123125']
OsSeXe 7 ['Se0.428571Xe0.428571Os0.142857', '-0.3', '-0.221412070357']
OsSeY 19 ['Se0.5Y0.2Os0.3', '-0.996', '-0.207122182875']
OsSiSr 1 ['Si0.555556Sr0.111111Os0.333333', '-0.64', '-0.200850981296']
OsSiTb 5 ['Si0.444444Tb0.111111Os0.444444', '-0.706', '-0.208944777908']
OsSiV 2 ['Si0.428571V0.142857Os0.428571', '-0.624', '-0.217352747624']
OsTaTm 2 ['Tm0.25Ta0.125Os0.625', '-0.342', '-0.2128701625']
OsTcV 1 ['V0.7Tc0.1Os0.2', '-0.395', '-0.2060172205']
OsTmXe 2 ['Xe0.111111Tm0.555556Os0.333333', '-0.3', '-0.210406496667']
PPaPm 1 ['P0.5Pm0.4Pa0.1', '-1.502', '-0.25742745352']
PPaPu 4 ['P0.4Pa0.2Pu0.4', '-1.305', '-0.206646176416']
PPaRh 1 ['P0.8Rh0.1Pa0.1', '-0.928', '-0.257475682271']
PPaRu 2 ['P0.333333Ru0.555556Pa0.111111', '-0.849', '-0.20107767118']
PPaS 12 ['P0.25S0.375Pa0.375', '-1.677', '-0.202563991255']
PPaSb 1 ['P0.777778Sb0.111111Pa0.111111', '-0.622', '-0.219763899677']
PPaSe 37 ['P0.111111Se0.444444Pa0.444444', '-1.346', '-0.207230531504']
PPaSn 1 ['P0.8Sn0.1Pa0.1', '-0.705', '-0.255172796836']
PPaTa 1 ['P0.444444Ta0.444444Pa0.111111', '-1.171', '-0.220294416019']
PPaXe 4 ['P0.7Xe0.2Pa0.1', '-0.58', '-0.217987509709']
PPaZr 1 ['P0.5Zr0.333333Pa0.166667', '-1.696', '-0.20663397552']
PPbPm 18 ['P0.3Pm0.6Pb0.1', '-1.005', '-0.202503309312']
PPbPu 26 ['P0.6Pb0.2Pu0.2', '-0.722', '-0.205962060208']
PPbRh 2 ['P0.777778Rh0.111111Pb0.111111', '-0.557', '-0.214209080624']
PPbTa 8 ['P0.5Ta0.3Pb0.2', '-0.613', '-0.212375749312']
PPbTe 11 ['P0.5Te0.25Pb0.25', '-0.416', '-0.211882016667']
PPbXe 1 ['P0.428571Xe0.142857Pb0.428571', '-0.22', '-0.22']
PPbY 4 ['P0.4Y0.5Pb0.1', '-1.715', '-0.204540736458']
PPdPm 40 ['P0.111111Pd0.777778Pm0.111111', '-0.651', '-0.204604771227']
PPdSc 9 ['P0.428571Sc0.428571Pd0.142857', '-1.649', '-0.205034939553']
PPdSr 7 ['P0.142857Sr0.428571Pd0.428571', '-0.999', '-0.212869678957']
PPdTa 8 ['P0.333333Pd0.222222Ta0.444444', '-0.943', '-0.209276956846']
PPdTb 5 ['P0.444444Pd0.111111Tb0.444444', '-1.506', '-0.201341589946']
PPdTe 2 ['P0.6Pd0.1Te0.3', '-0.391', '-0.211492283208']
PPdTm 15 ['P0.428571Pd0.142857Tm0.428571', '-1.762', '-0.221070645981']
PPdY 2 ['P0.444444Y0.222222Pd0.333333', '-1.302', '-0.208975595796']

PPdYb 37 ['P0.1Pd0.6Yb0.3', '-0.96', '-0.200372693104']
PPdZn 14 ['P0.375Zn0.375Pd0.25', '-0.706', '-0.205354426484']
PPmPr 5 ['P0.4Pr0.1Pm0.5', '-1.25', '-0.205926060916']
PPmPt 12 ['P0.222222Pm0.111111Pt0.666667', '-0.917', '-0.201016624815']
PPmPu 22 ['P0.428571Pm0.285714Pu0.285714', '-1.291', '-0.213297727231']
PPmRb 6 ['P0.5Rb0.1Pm0.4', '-1.339', '-0.287206815187']
PPmRe 8 ['P0.428571Pm0.428571Re0.142857', '-1.266', '-0.244484352231']
PPmRh 33 ['P0.625Rh0.25Pm0.125', '-1.111', '-0.200952584087']
PPmRu 38 ['P0.25Ru0.25Pm0.5', '-0.857', '-0.20616304401']
PPmS 30 ['P0.222222S0.333333Pm0.444444', '-1.956', '-0.203646083337']
PPmSb 8 ['P0.555556Sb0.111111Pm0.333333', '-1.014', '-0.219487829513']
PPmSc 5 ['P0.428571Sc0.142857Pm0.428571', '-1.347', '-0.20339701616']
PPmSe 45 ['P0.428571Se0.285714Pm0.285714', '-1.127', '-0.200725599286']
PPmSi 19 ['Si0.111111P0.555556Pm0.333333', '-1.1', '-0.2088801603']
PPmSm 1 ['P0.5Pm0.4Sm0.1', '-1.54', '-0.24660502077']
PPmSn 11 ['P0.75Sn0.125Pm0.125', '-0.618', '-0.210289544976']
PPmSr 11 ['P0.555556Sr0.111111Pm0.333333', '-1.339', '-0.224914391634']
PPmTa 13 ['P0.375Pm0.5Ta0.125', '-1.11', '-0.204804743255']
PPmTb 3 ['P0.5Pm0.375Tb0.125', '-1.448', '-0.201834519']
PPmTc 20 ['P0.5Tc0.3Pm0.2', '-1.045', '-0.202385974877']
PPmTe 46 ['P0.6Te0.3Pm0.1', '-0.467', '-0.205988540416']
PPmTh 1 ['P0.444444Pm0.444444Th0.111111', '-1.509', '-0.34123384074']
PPmTi 7 ['P0.4Ti0.2Pm0.4', '-1.281', '-0.213807187258']
PPmTl 20 ['P0.333333Pm0.444444Tl0.222222', '-1.085', '-0.20320997868']
PPmTm 4 ['P0.428571Pm0.428571Tm0.142857', '-1.391', '-0.215581544017']
PPmV 2 ['P0.4V0.1Pm0.5', '-1.181', '-0.211382931708']
PPmW 6 ['P0.5Pm0.333333W0.166667', '-1.216', '-0.256523918437']
PPmXe 15 ['P0.75Xe0.125Pm0.125', '-0.498', '-0.200057936067']
PPmY 2 ['P0.444444Y0.111111Pm0.444444', '-1.399', '-0.214720170184']
PPmYb 11 ['P0.5Pm0.3Yb0.2', '-1.32', '-0.20176217677']
PPmZn 17 ['P0.5Zn0.166667Pm0.333333', '-1.118', '-0.211675868489']
PPmZr 2 ['P0.444444Zr0.111111Pm0.444444', '-1.34', '-0.210786900184']
PPrPu 9 ['P0.428571Pr0.142857Pu0.428571', '-1.437', '-0.229784392231']
PPrSi 1 ['Si0.125P0.375Pr0.5', '-1.634', '-0.213887652265']
PPtSm 1 ['P0.142857Sm0.142857Pt0.714286', '-0.956', '-0.209807444077']
PPtSr 3 ['P0.5Sr0.25Pt0.25', '-1.23', '-0.205439352692']
PPtTh 19 ['P0.25Pt0.5Th0.25', '-1.159', '-0.206796661588']
PPtTm 4 ['P0.142857Tm0.142857Pt0.714286', '-1.06', '-0.22872542064']
PPtY 9 ['P0.3Y0.2Pt0.5', '-1.284', '-0.201983907062']
PPtYb 7 ['P0.555556Yb0.222222Pt0.222222', '-1.203', '-0.205416149604']
PPtZn 1 ['P0.333333Zn0.333333Pt0.333333', '-0.882', '-0.22862913993']
PPuRh 2 ['P0.777778Rh0.111111Pu0.111111', '-0.852', '-0.222521336296']
PPuRu 19 ['P0.8Ru0.1Pu0.1', '-0.781', '-0.201312753187']
PPuS 2 ['P0.4S0.2Pu0.4', '-1.513', '-0.213441274474']

PPuSb 2 ['P0.555556Sb0.111111Pu0.333333', '-1.07', '-0.209936767013']
PPuSe 5 ['P0.2Se0.4Pu0.4', '-1.497', '-0.206419506206']
PPuSn 4 ['P0.714286Sn0.142857Pu0.142857', '-0.711', '-0.216951881758']
PPuSr 20 ['P0.555556Sr0.333333Pu0.111111', '-1.357', '-0.211767519469']
PPuTa 4 ['P0.4Ta0.5Pu0.1', '-1.096', '-0.210817077916']
PPuTb 2 ['P0.444444Tb0.222222Pu0.333333', '-1.464', '-0.26424355276']
PPuTc 8 ['P0.5Tc0.4Pu0.1', '-0.953', '-0.207105399663']
PPuTe 6 ['P0.1Te0.5Pu0.4', '-1.238', '-0.204693944437']
PPuTi 9 ['P0.428571Ti0.142857Pu0.428571', '-1.372', '-0.201790253547']
PPuTl 16 ['P0.571429Tl0.285714Pu0.142857', '-0.7', '-0.211148804737']
PPuXe 4 ['P0.7Xe0.2Pu0.1', '-0.467', '-0.208981030104']
PPuYb 7 ['P0.5Yb0.4Pu0.1', '-1.265', '-0.20062729052']
PPuZn 1 ['P0.5Zn0.25Pu0.25', '-1.085', '-0.272234633723']
PPuZr 7 ['P0.4Zr0.3Pu0.3', '-1.376', '-0.214288520916']
PRbRh 1 ['P0.8Rb0.1Rh0.1', '-0.705', '-0.248920302218']
PRbSe 1 ['P0.111111Se0.333333Rb0.555556', '-1.138', '-0.206076475838']
PRbTa 3 ['P0.5Rb0.1Ta0.4', '-0.917', '-0.245402245145']
PRbTe 7 ['P0.142857Rb0.428571Te0.428571', '-0.946', '-0.218712120863']
PReSr 4 ['P0.5Sr0.2Re0.3', '-0.964', '-0.213444076552']
PReTa 2 ['P0.444444Ta0.444444Re0.111111', '-0.928', '-0.203629770859']
PReTe 12 ['P0.5Te0.4Re0.1', '-0.389', '-0.201442168135']
PRhS 3 ['P0.3S0.5Rh0.2', '-0.791', '-0.200828133034']
PRhSb 3 ['P0.75Rh0.125Sb0.125', '-0.586', '-0.200360215703']
PRhSc 29 ['P0.4Sc0.5Rh0.1', '-1.701', '-0.200172930916']
PRhSm 17 ['P0.444444Rh0.333333Sm0.222222', '-1.427', '-0.204809598425']
PRhSn 2 ['P0.777778Rh0.111111Sn0.111111', '-0.725', '-0.284637177432']
PRhSr 18 ['P0.5Sr0.166667Rh0.333333', '-1.184', '-0.201569279503']
PRhTa 2 ['P0.428571Rh0.142857Ta0.428571', '-1.061', '-0.238507762886']
PRhTb 11 ['P0.333333Rh0.222222Tb0.444444', '-1.318', '-0.204072423275']
PRhTi 2 ['P0.333333Ti0.444444Rh0.222222', '-1.341', '-0.200801683716']
PRhTl 3 ['P0.75Rh0.125Tl0.125', '-0.691', '-0.200139132217']
PRhTm 12 ['P0.4Rh0.3Tm0.3', '-1.53', '-0.203234605916']
PRhU 4 ['P0.333333Rh0.333333U0.333333', '-1.136', '-0.207215376013']
PRhXe 5 ['P0.714286Rh0.142857Xe0.142857', '-0.654', '-0.213268817946']
PRhY 49 ['P0.75Y0.125Rh0.125', '-1.087', '-0.201005453905']
PRhYb 26 ['P0.555556Rh0.222222Yb0.222222', '-1.109', '-0.204297556874']
PRhZn 10 ['P0.166667Zn0.333333Rh0.5', '-0.817', '-0.200287745173']
PRuS 1 ['P0.333333S0.555556Ru0.111111', '-0.753', '-0.206328381609']
PRuSc 1 ['P0.3Sc0.5Ru0.2', '-1.416', '-0.231020033812']
PRuSn 7 ['P0.333333Ru0.5Sn0.166667', '-0.734', '-0.206144669205']
PRuTa 11 ['P0.375Ru0.125Ta0.5', '-1.007', '-0.219461604765']
PRuTe 1 ['P0.555556Ru0.111111Te0.333333', '-0.57', '-0.212590803425']
PRuTi 1 ['P0.222222Ti0.333333Ru0.444444', '-1.134', '-0.220663419708']
PRuTm 2 ['P0.25Ru0.375Tm0.375', '-1.179', '-0.21246000776']

PRuU 12 ['P0.375Ru0.375U0.25', '-0.957', '-0.212560788515']
PRuY 2 ['P0.5Y0.2Ru0.3', '-1.346', '-0.200446198022']
PRuYb 2 ['P0.555556Ru0.333333Yb0.111111', '-1.14', '-0.216144921136']
PRuZn 13 ['P0.25Zn0.375Ru0.375', '-0.612', '-0.200926488385']
PSSc 1 ['P0.222222S0.444444Sc0.333333', '-1.974', '-0.304647877327']
PSTa 6 ['P0.285714S0.571429Ta0.142857', '-0.975', '-0.204868038226']
PSTm 1 ['P0.285714S0.428571Tm0.285714', '-1.848', '-0.231370438652']
PSXe 3 ['P0.111111S0.555556Xe0.333333', '-0.401', '-0.203331305222']
PSY 8 ['P0.5S0.1Y0.4', '-1.793', '-0.207228576077']
PSZr 3 ['P0.2S0.6Zr0.2', '-1.407', '-0.210254441247']
PSbTa 13 ['P0.444444Sb0.222222Ta0.333333', '-0.657', '-0.21186194368']
PSbU 3 ['P0.75Sb0.125U0.125', '-0.564', '-0.200592349323']
PSbV 1 ['P0.333333V0.5Sb0.166667', '-1.094', '-0.35457187993']
PSbW 6 ['P0.444444Sb0.222222W0.333333', '-0.604', '-0.201654172129']
PScSe 20 ['P0.375Sc0.25Se0.375', '-1.108', '-0.202742098203']
PScSi 3 ['Si0.2P0.3Sc0.5', '-1.522', '-0.215418563812']
PScTa 1 ['P0.5Sc0.1Ta0.4', '-1.078', '-0.22001954602']
PScTe 1 ['P0.166667Sc0.666667Te0.166667', '-1.234', '-0.246644003439']
PScTl 6 ['P0.285714Sc0.571429Tl0.142857', '-1.288', '-0.225322102086']
PSeSr 9 ['P0.166667Se0.333333Sr0.5', '-1.98', '-0.206157583783']
PSeTc 8 ['P0.2Se0.6Tc0.2', '-0.445', '-0.201062184779']
PSeTm 1 ['P0.1Se0.5Tm0.4', '-1.804', '-0.219157580106']
PSeY 27 ['P0.375Se0.25Y0.375', '-1.584', '-0.200694624453']
PSiSr 4 ['Si0.1P0.4Sr0.5', '-1.242', '-0.20684525108']
PSiTa 12 ['Si0.1P0.3Ta0.6', '-0.963', '-0.203554534612']
PSiTb 1 ['Si0.2P0.4Tb0.4', '-1.337', '-0.209514274552']
PSiTe 9 ['Si0.2P0.5Te0.3', '-0.374', '-0.200106195416']
PSiW 1 ['Si0.2P0.4W0.4', '-0.706', '-0.240429794916']
PSiXe 6 ['Si0.333333P0.555556Xe0.111111', '-0.456', '-0.201494510578']
PSiY 4 ['Si0.222222P0.444444Y0.333333', '-1.437', '-0.206406871018']
PSmTe 1 ['P0.5Te0.4Sm0.1', '-0.665', '-0.21623883577']
PSnSr 7 ['P0.5Sr0.125Sn0.375', '-0.682', '-0.204677976644']
PSnTa 10 ['P0.285714Sn0.142857Ta0.571429', '-0.908', '-0.202824949583']
PSnTb 2 ['P0.777778Sn0.111111Tb0.111111', '-0.643', '-0.232237617516']
PSnTc 1 ['P0.8Tc0.1Sn0.1', '-0.631', '-0.236968163793']
PSnTe 1 ['P0.571429Sn0.142857Te0.285714', '-0.377', '-0.251550410181']
PSnTi 11 ['P0.285714Ti0.428571Sn0.285714', '-1.109', '-0.204567178554']
PSnTm 2 ['P0.375Sn0.125Tm0.5', '-1.675', '-0.208768524891']
PSnU 3 ['P0.75Sn0.125U0.125', '-0.69', '-0.216823958231']
PSnXe 6 ['P0.5Sn0.333333Xe0.166667', '-0.379', '-0.208784508792']
PSnYb 2 ['P0.5Sn0.1Yb0.4', '-1.071', '-0.208766962335']
PSnZr 3 ['P0.375Zr0.5Sn0.125', '-1.522', '-0.250589072705']
PSrTa 5 ['P0.4Sr0.1Ta0.5', '-1.098', '-0.215895241716']
PSrTe 2 ['P0.6Sr0.1Te0.3', '-0.591', '-0.209321454319']

PSrTl 2 ['P0.333333Sr0.555556Tl0.111111', '-1.08', '-0.200011639233']
PSrZn 4 ['P0.3Zn0.4Sr0.3', '-0.955', '-0.204969990322']
PTaTb 2 ['P0.444444Tb0.111111Ta0.444444', '-1.1', '-0.215816074667']
PTaTc 12 ['P0.333333Tc0.333333Ta0.333333', '-0.862', '-0.200926877847']
PTaTe 22 ['P0.4Te0.1Ta0.5', '-0.878', '-0.226087046478']
PTaTh 2 ['P0.5Ta0.4Th0.1', '-1.116', '-0.220747628438']
PTaTi 3 ['P0.5Ti0.125Ta0.375', '-1.076', '-0.225838660984']
PTaTl 17 ['P0.5Ta0.25Tl0.25', '-0.621', '-0.207264831957']
PTaTm 1 ['P0.5Tm0.1Ta0.4', '-1.114', '-0.22881841877']
PTaV 7 ['P0.5V0.2Ta0.3', '-1.031', '-0.220593760895']
PTaW 1 ['P0.5Ta0.4W0.1', '-0.917', '-0.23895134677']
PTaXe 14 ['P0.4Xe0.3Ta0.3', '-0.603', '-0.202375749312']
PTaY 4 ['P0.428571Y0.142857Ta0.428571', '-1.266', '-0.218312823035']
PTaYb 7 ['P0.428571Yb0.428571Ta0.142857', '-1.102', '-0.205747541339']
PTaZn 20 ['P0.5Zn0.333333Ta0.166667', '-0.679', '-0.201921124348']
PTaZr 1 ['P0.5Zr0.1Ta0.4', '-1.062', '-0.20889601852']
PTbTe 5 ['P0.5Te0.375Tb0.125', '-0.572', '-0.214401812333']
PTbXe 3 ['P0.777778Xe0.111111Tb0.111111', '-0.528', '-0.214809520709']
PTcTe 30 ['P0.25Tc0.5Te0.25', '-0.557', '-0.202434433742']
PTcTi 30 ['P0.166667Ti0.333333Tc0.5', '-0.82', '-0.201010059231']
PTcV 1 ['P0.5V0.25Tc0.25', '-1.007', '-0.237956407484']
PTcY 1 ['P0.5Y0.25Tc0.25', '-1.387', '-0.205100497484']
PTcYb 3 ['P0.428571Tc0.285714Yb0.285714', '-0.979', '-0.20042414815']
PTcZn 5 ['P0.555556Zn0.222222Tc0.222222', '-0.745', '-0.211418047592']
PTcZr 1 ['P0.5Zr0.3Tc0.2', '-1.364', '-0.216369675591']
PTeTh 3 ['P0.5Te0.333333Th0.166667', '-0.877', '-0.210367082237']
PTeTi 25 ['P0.5Ti0.375Te0.125', '-1.242', '-0.205792144727']
PTeU 10 ['P0.5Te0.3U0.2', '-0.79', '-0.208547758916']
PTeV 2 ['P0.5V0.166667Te0.333333', '-0.618', '-0.200151901353']
PTeW 14 ['P0.428571Te0.428571W0.142857', '-0.44', '-0.20549349744']
PTeXe 28 ['P0.4Te0.4Xe0.2', '-0.203', '-0.203']
PTeY 3 ['P0.5Y0.2Te0.3', '-1.004', '-0.204860076854']
PTeYb 6 ['P0.5Te0.3Yb0.2', '-0.901', '-0.200248391686']
PTeZr 18 ['P0.375Zr0.25Te0.375', '-1.032', '-0.207010953567']
PTiTl 5 ['P0.222222Ti0.555556Tl0.222222', '-1.014', '-0.20107744594']
PTiZn 1 ['P0.5Ti0.2Zn0.3', '-0.957', '-0.226548572608']
PTlTm 2 ['P0.5Tm0.375Tl0.125', '-1.563', '-0.230105242145']
PTlXe 2 ['P0.777778Xe0.111111Tl0.111111', '-0.361', '-0.205116913355']
PTlY 4 ['P0.333333Y0.555556Tl0.111111', '-1.479', '-0.205456368495']
PTlYb 11 ['P0.5Yb0.2Tl0.3', '-0.727', '-0.201428557895']
PTlZr 2 ['P0.5Zr0.333333Tl0.166667', '-1.268', '-0.200598805937']
PTmZn 1 ['P0.428571Zn0.285714Tm0.285714', '-1.312', '-0.220379056606']
PUXe 5 ['P0.666667Xe0.222222U0.111111', '-0.533', '-0.209970977176']
PWZn 18 ['P0.555556Zn0.333333W0.111111', '-0.607', '-0.200982131515']

PXeY 1 ['P0.8Y0.1Xe0.1', '-0.662', '-0.21222327452']
PXeZn 21 ['P0.375Zn0.125Xe0.5', '-0.369', '-0.201749733046']
PYZn 1 ['P0.4Zn0.1Y0.5', '-1.692', '-0.216168583916']
PYZr 1 ['P0.571429Y0.285714Zr0.142857', '-1.862', '-0.375154761666']
PYbZn 1 ['P0.375Zn0.25Yb0.375', '-1.065', '-0.226176097265']
PaPbS 2 ['S0.666667Pb0.166667Pa0.166667', '-1.111', '-0.201455022077']
PaPbSe 8 ['Se0.666667Pb0.166667Pa0.166667', '-0.793', '-0.202009163333']
PaPdPm 3 ['Pd0.75Pm0.125Pa0.125', '-0.738', '-0.210870575938']
PaPdS 11 ['S0.666667Pd0.111111Pa0.222222', '-1.296', '-0.23427949138']
PaPdSe 42 ['Se0.555556Pd0.333333Pa0.111111', '-0.711', '-0.204018025']
PaPmS 12 ['S0.666667Pm0.222222Pa0.111111', '-1.884', '-0.207134021047']
PaPmSe 16 ['Se0.555556Pm0.111111Pa0.333333', '-1.427', '-0.2640986075']
PaPmSi 2 ['Si0.666667Pm0.111111Pa0.222222', '-0.462', '-0.227574083611']
PaPmTe 2 ['Te0.666667Pm0.111111Pa0.222222', '-0.775', '-0.204628955277']
PaPrS 3 ['S0.7Pr0.1Pa0.2', '-1.619', '-0.220697698741']
PaPrSe 8 ['Se0.666667Pr0.222222Pa0.111111', '-1.583', '-0.221618585']
PaPtS 46 ['S0.1Pt0.7Pa0.2', '-1.084', '-0.200843286099']
PaPtSe 35 ['Se0.222222Pt0.555556Pa0.222222', '-1.064', '-0.207125158095']
PaPtSi 1 ['Si0.111111Pt0.777778Pa0.111111', '-0.871', '-0.201429687222']
PaPtSm 1 ['Sm0.1Pt0.8Pa0.1', '-0.983', '-0.23272860525']
PaPtTh 3 ['Pt0.75Th0.125Pa0.125', '-1.0', '-0.212391796875']
PaPtTm 1 ['Tm0.1Pt0.8Pa0.1', '-1.024', '-0.209197736']
PaPtY 1 ['Y0.1Pt0.8Pa0.1', '-0.991', '-0.2127259885']
PaPtYb 4 ['Yb0.111111Pt0.777778Pa0.111111', '-1.002', '-0.208792673332']
PaPuS 3 ['S0.7Pa0.2Pu0.1', '-1.497', '-0.202166556491']
PaPuSe 11 ['Se0.555556Pa0.333333Pu0.111111', '-1.437', '-0.20401501111']
PaPuSi 5 ['Si0.75Pa0.125Pu0.125', '-0.463', '-0.2082609525']
PaPuTe 1 ['Te0.5Pa0.1Pu0.4', '-1.242', '-0.232028006331']
PaRbS 14 ['S0.5Rb0.333333Pa0.166667', '-1.52', '-0.208858848133']
PaRbSe 13 ['Se0.714286Rb0.142857Pa0.142857', '-0.846', '-0.206242035714']
PaRbSi 2 ['Si0.666667Rb0.111111Pa0.222222', '-0.364', '-0.227049556667']
PaReS 2 ['S0.7Re0.1Pa0.2', '-1.26', '-0.217832380617']
PaReSe 5 ['Se0.7Re0.1Pa0.2', '-0.832', '-0.23215777725']
PaReSi 2 ['Si0.444444Re0.444444Pa0.111111', '-0.535', '-0.249836752063']
PaRhS 19 ['S0.4Rh0.3Pa0.3', '-1.469', '-0.200207497394']
PaRhSe 35 ['Se0.111111Rh0.555556Pa0.333333', '-1.139', '-0.204994826667']
PaRuS 15 ['S0.6Ru0.3Pa0.1', '-1.116', '-0.208887253092']
PaRuSe 34 ['Se0.6Ru0.2Pa0.2', '-0.946', '-0.2085942475']
PaSSb 14 ['S0.625Sb0.25Pa0.125', '-0.988', '-0.208531329617']
PaSSc 7 ['S0.5Sc0.333333Pa0.166667', '-2.132', '-0.206093734657']
PaSSe 40 ['S0.444444Se0.333333Pa0.222222', '-1.109', '-0.200626314772']
PaSSi 4 ['Si0.111111S0.555556Pa0.333333', '-1.654', '-0.205325647213']
PaSSm 3 ['S0.666667Sm0.166667Pa0.166667', '-1.884', '-0.26726308445']
PaSSn 2 ['S0.666667Sn0.166667Pa0.166667', '-1.211', '-0.230381475266']

PaSSr 11 ['S0.6Sr0.3Pa0.1', '-2.024', '-0.212241695715']
PaSTa 7 ['S0.5Ta0.166667Pa0.333333', '-1.707', '-0.206959322679']
PaSTb 4 ['S0.7Tb0.1Pa0.2', '-1.541', '-0.211623118276']
PaSTc 5 ['S0.5Tc0.333333Pa0.166667', '-1.189', '-0.204615269659']
PaSTe 3 ['S0.6Te0.1Pa0.3', '-1.427', '-0.200695524943']
PaSTh 4 ['S0.5Th0.25Pa0.25', '-2.071', '-0.20307427799']
PaSTi 11 ['S0.714286Ti0.142857Pa0.142857', '-1.459', '-0.202136694376']
PaSTl 8 ['S0.571429Tl0.142857Pa0.285714', '-1.486', '-0.203357065726']
PaSTm 5 ['S0.625Tm0.125Pa0.25', '-1.933', '-0.206560157819']
PaSU 3 ['S0.666667Pa0.222222U0.111111', '-1.694', '-0.202127058434']
PaSV 2 ['S0.7V0.1Pa0.2', '-1.405', '-0.20439744719']
PaSW 6 ['S0.666667W0.166667Pa0.166667', '-1.388', '-0.204326304739']
PaSXe 16 ['S0.666667Xe0.222222Pa0.111111', '-0.67', '-0.215813157386']
PaSY 3 ['S0.666667Y0.166667Pa0.166667', '-1.881', '-0.261658862992']
PaSYb 1 ['S0.666667Yb0.111111Pa0.222222', '-1.711', '-0.265113728879']
PaSZn 2 ['S0.625Zn0.125Pa0.25', '-1.471', '-0.210078994867']
PaSZr 17 ['S0.666667Zr0.222222Pa0.111111', '-1.82', '-0.200500400377']
PaSbSe 16 ['Se0.428571Sb0.285714Pa0.285714', '-0.959', '-0.20798625381']
PaSbTe 1 ['Sb0.2Te0.4Pa0.4', '-0.791', '-0.246003864917']
PaScSe 16 ['Sc0.2Se0.7Pa0.1', '-1.088', '-0.209590228']
PaScTc 7 ['Sc0.333333Tc0.444444Pa0.222222', '-0.302', '-0.207329801111']
PaSeSi 28 ['Si0.1Se0.7Pa0.2', '-0.819', '-0.2015479895']
PaSeSm 8 ['Se0.6Sm0.2Pa0.2', '-1.671', '-0.215667354301']
PaSeSn 5 ['Se0.571429Sn0.142857Pa0.285714', '-1.061', '-0.212361781878']
PaSeSr 7 ['Se0.6Sr0.2Pa0.2', '-1.579', '-0.26835582']
PaSeTa 8 ['Se0.714286Ta0.142857Pa0.142857', '-1.009', '-0.287723131786']
PaSeTb 7 ['Se0.666667Tb0.222222Pa0.111111', '-1.369', '-0.202386969517']
PaSeTc 16 ['Se0.714286Tc0.142857Pa0.142857', '-0.603', '-0.258992755714']
PaSeTe 57 ['Se0.7Te0.2Pa0.1', '-0.452', '-0.211194929']
PaSeTh 8 ['Se0.6Th0.2Pa0.2', '-1.736', '-0.210754113998']
PaSeTi 7 ['Ti0.2Se0.6Pa0.2', '-1.362', '-0.208479199242']
PaSeTl 17 ['Se0.5Tl0.166667Pa0.333333', '-1.101', '-0.206533358333']
PaSeTm 11 ['Se0.555556Tm0.333333Pa0.111111', '-1.753', '-0.245115997779']
PaSeU 3 ['Se0.625Pa0.25U0.125', '-1.309', '-0.243698783125']
PaSeV 5 ['V0.1Se0.7Pa0.2', '-0.996', '-0.2919795']
PaSeW 5 ['Se0.7W0.1Pa0.2', '-0.942', '-0.2938800825']
PaSeXe 19 ['Se0.5Xe0.125Pa0.375', '-1.121', '-0.21798098375']
PaSeY 16 ['Se0.555556Y0.111111Pa0.333333', '-1.452', '-0.256893935']
PaSeYb 5 ['Se0.666667Yb0.166667Pa0.166667', '-1.324', '-0.22060509']
PaSeZn 15 ['Zn0.166667Se0.666667Pa0.166667', '-0.845', '-0.204093313333']
PaSeZr 15 ['Se0.7Zr0.2Pa0.1', '-1.284', '-0.200473154']
PaSiSr 3 ['Si0.714286Sr0.142857Pa0.142857', '-0.45', '-0.204909225714']
PaSiTc 11 ['Si0.333333Tc0.555556Pa0.111111', '-0.53', '-0.207000417222']
PaSiY 2 ['Si0.7Y0.1Pa0.2', '-0.538', '-0.2442648605']

PaSiYb 2 ['Si0.666667Yb0.111111Pa0.222222', '-0.498', '-0.234912292708']
PbPdPm 2 ['Pd0.666667Pm0.222222Pb0.111111', '-0.684', '-0.204543828333']
PbPdPu 1 ['Pd0.4Pb0.1Pu0.5', '-0.61', '-0.24054407275']
PbPmS 6 ['S0.333333Pm0.5Pb0.166667', '-1.625', '-0.232374329077']
PbPmSe 11 ['Se0.5Pm0.3Pb0.2', '-1.423', '-0.22283301725']
PbPtS 39 ['S0.5Pt0.2Pb0.3', '-0.834', '-0.200092650993']
PbPtTh 8 ['Pt0.666667Pb0.166667Th0.166667', '-0.691', '-0.223250345833']
PbPuS 4 ['S0.5Pb0.1Pu0.4', '-1.887', '-0.202226852491']
PbPuSe 11 ['Se0.4Pb0.3Pu0.3', '-1.254', '-0.212629964']
PbPuTl 2 ['Tl0.3Pb0.3Pu0.4', '-0.292', '-0.2060806665']
PbRbS 1 ['S0.428571Rb0.142857Pb0.428571', '-0.966', '-0.240356220424']
PbRhTm 1 ['Rh0.4Tm0.3Pb0.3', '-0.821', '-0.203042617']
PbSSe 2 ['S0.285714Se0.428571Pb0.285714', '-0.598', '-0.20668906171']
PbSTe 1 ['S0.3Te0.3Pb0.4', '-0.725', '-0.232476321462']
PbSTm 3 ['S0.4Tm0.4Pb0.2', '-1.888', '-0.231047658392']
PbSZr 6 ['S0.4Zr0.4Pb0.2', '-1.394', '-0.202475278392']
PbScSe 1 ['Sc0.4Se0.5Pb0.1', '-1.659', '-0.223771491376']
PbSeSr 1 ['Se0.333333Sr0.555556Pb0.111111', '-1.798', '-0.233347243056']
PbSeTc 4 ['Se0.625Tc0.25Pb0.125', '-0.371', '-0.22876321125']
PbSeTm 7 ['Se0.444444Tm0.333333Pb0.222222', '-1.456', '-0.215677187779']
PbSeY 12 ['Se0.444444Y0.444444Pb0.111111', '-1.781', '-0.21131002']
PbSeZr 5 ['Se0.428571Zr0.428571Pb0.142857', '-1.173', '-0.200411639881']
PdPmPt 2 ['Pd0.7Pm0.2Pt0.1', '-0.643', '-0.2650053075']
PdPmS 11 ['S0.3Pd0.2Pm0.5', '-1.642', '-0.208140487544']
PdPmSb 8 ['Pd0.5Sb0.3Pm0.2', '-0.822', '-0.201048432445']
PdPmSe 67 ['Se0.111111Pd0.444444Pm0.444444', '-1.081', '-0.201365486667']
PdPmSi 3 ['Si0.1Pd0.7Pm0.2', '-0.755', '-0.20094787475']
PdPmSm 1 ['Pd0.777778Pm0.111111Sm0.111111', '-0.745', '-0.204776987778']
PdPmSn 11 ['Pd0.3Sn0.3Pm0.4', '-0.889', '-0.205601174694']
PdPmTa 2 ['Pd0.8Pm0.1Ta0.1', '-0.561', '-0.21122030275']
PdPmTe 2 ['Pd0.666667Te0.111111Pm0.222222', '-0.731', '-0.252496052303']
PdPmTh 1 ['Pd0.8Pm0.1Th0.1', '-0.743', '-0.20743076825']
PdPmTl 4 ['Pd0.75Pm0.125Tl0.125', '-0.518', '-0.213131722188']
PdPmU 1 ['Pd0.777778Pm0.111111U0.111111', '-0.613', '-0.204192169444']
PdPmXe 2 ['Pd0.666667Xe0.111111Pm0.222222', '-0.562', '-0.215722206111']
PdPmYb 1 ['Pd0.7Pm0.2Yb0.1', '-0.806', '-0.2153305495']
PdPmZn 11 ['Zn0.3Pd0.6Pm0.1', '-0.7', '-0.20112548475']
PdPmZr 3 ['Zr0.125Pd0.75Pm0.125', '-0.808', '-0.204317490625']
PdPrSe 20 ['Se0.5Pd0.25Pr0.25', '-1.499', '-0.249545979001']
PdPtSr 1 ['Sr0.6Pd0.1Pt0.3', '-0.761', '-0.2058778395']
PdPtTh 7 ['Pd0.125Pt0.625Th0.25', '-0.873', '-0.205885903333']
PdPuS 3 ['S0.5Pd0.166667Pu0.333333', '-1.746', '-0.217229652158']
PdPuSe 5 ['Se0.375Pd0.375Pu0.25', '-1.081', '-0.207871962968']
PdPuSi 4 ['Si0.428571Pd0.428571Pu0.142857', '-0.942', '-0.259957936786']

PdPuTe 1 ['Pd0.1Te0.5Pu0.4', '-1.256', '-0.296103989281']
PdPuZn 1 ['Zn0.1Pd0.4Pu0.5', '-0.575', '-0.20191118375']
PdSSb 8 ['S0.375Pd0.375Sb0.25', '-0.668', '-0.201790356057']
PdSSe 23 ['S0.285714Se0.285714Pd0.428571', '-0.608', '-0.202131488853']
PdSSm 2 ['S0.444444Pd0.222222Sm0.333333', '-1.987', '-0.2016045809']
PdSTc 1 ['S0.555556Tc0.333333Pd0.111111', '-0.89', '-0.217403542215']
PdSTh 2 ['S0.5Pd0.125Th0.375', '-2.194', '-0.258599921117']
PdSZn 23 ['S0.125Zn0.375Pd0.5', '-0.76', '-0.206154117936']
PdSZr 12 ['S0.333333Zr0.5Pd0.166667', '-1.438', '-0.21779990352']
PdSbSr 1 ['Sr0.333333Pd0.333333Sb0.333333', '-1.058', '-0.2371686615']
PdScSe 14 ['Sc0.222222Se0.333333Pd0.444444', '-1.061', '-0.205646635833']
PdSeSm 23 ['Se0.222222Pd0.555556Sm0.222222', '-1.149', '-0.201311903889']
PdSeTb 9 ['Se0.4Pd0.2Tb0.4', '-1.513', '-0.2041406838']
PdSeTc 1 ['Se0.6Tc0.3Pd0.1', '-0.336', '-0.2519464375']
PdSeTh 14 ['Se0.5Pd0.166667Th0.333333', '-1.744', '-0.201307355831']
PdSeTm 31 ['Se0.111111Pd0.666667Tm0.222222', '-1.092', '-0.206553547778']
PdSeU 6 ['Se0.375Pd0.375U0.25', '-0.985', '-0.207980477642']
PdSeY 22 ['Se0.222222Y0.222222Pd0.555556', '-1.072', '-0.209017983611']
PdSeZn 1 ['Zn0.333333Se0.111111Pd0.555556', '-0.703', '-0.240246544506']
PdSeZr 15 ['Se0.4Zr0.3Pd0.3', '-1.329', '-0.20119356775']
PdSnYb 3 ['Pd0.375Sn0.5Yb0.125', '-0.883', '-0.210351041574']
PmPrS 7 ['S0.5Pr0.166667Pm0.333333', '-2.19', '-0.220385402573']
PmPrSe 6 ['Se0.571429Pr0.142857Pm0.285714', '-1.838', '-0.208555454643']
PmPrSn 1 ['Sn0.666667Pr0.166667Pm0.166667', '-0.822', '-0.227099290433']
PmPtS 29 ['S0.5Pm0.333333Pt0.166667', '-2.036', '-0.201981295492']
PmPtSe 51 ['Se0.7Pm0.2Pt0.1', '-0.969', '-0.2052309575']
PmPtSi 8 ['Si0.571429Pm0.142857Pt0.285714', '-0.761', '-0.252438323929']
PmPtTh 3 ['Pm0.125Pt0.75Th0.125', '-0.951', '-0.202093796562']
PmPuS 22 ['S0.428571Pm0.142857Pu0.428571', '-1.846', '-0.201333702563']
PmPuSe 9 ['Se0.555556Pm0.222222Pu0.222222', '-1.715', '-0.220714563888']
PmPuTe 1 ['Te0.6Pm0.3Pu0.1', '-1.309', '-0.248798422581']
PmRbS 9 ['S0.5Rb0.2Pm0.3', '-2.08', '-0.263829591242']
PmRbSe 21 ['Se0.7Rb0.1Pm0.2', '-1.084', '-0.2285824155']
PmRbTe 5 ['Rb0.2Te0.6Pm0.2', '-1.133', '-0.239824826999']
PmReS 9 ['S0.333333Pm0.5Re0.166667', '-1.462', '-0.215100557827']
PmReSe 13 ['Se0.625Pm0.125Re0.25', '-0.917', '-0.216174747813']
PmRhS 24 ['S0.5Rh0.2Pm0.3', '-1.91', '-0.202672917867']
PmRhSb 3 ['Rh0.333333Sb0.333333Pm0.333333', '-0.906', '-0.200047672083']
PmRhSe 46 ['Se0.625Rh0.25Pm0.125', '-0.922', '-0.210506780313']
PmRhSi 9 ['Si0.625Rh0.25Pm0.125', '-0.731', '-0.203322992187']
PmRhSn 1 ['Rh0.3Sn0.6Pm0.1', '-0.723', '-0.211727774389']
PmRuS 19 ['S0.571429Ru0.285714Pm0.142857', '-1.385', '-0.203480405563']
PmRuSe 34 ['Se0.666667Ru0.166667Pm0.166667', '-0.959', '-0.2055102575']
PmRuSi 1 ['Si0.4Ru0.1Pm0.5', '-0.601', '-0.21000024575']

PmSSb 6 ['S0.428571Sb0.142857Pm0.428571', '-2.042', '-0.204924047921']
PmSSc 14 ['S0.428571Sc0.285714Pm0.285714', '-1.889', '-0.201689316491']
PmSSe 35 ['S0.375Se0.25Pm0.375', '-1.983', '-0.202240921306']
PmSSi 21 ['Si0.2S0.5Pm0.3', '-1.917', '-0.201803521617']
PmSSm 4 ['S0.5Pm0.4Sm0.1', '-2.134', '-0.20357497424']
PmSSn 12 ['S0.3Sn0.2Pm0.5', '-1.598', '-0.219763264174']
PmSSr 9 ['S0.375Sr0.125Pm0.5', '-1.726', '-0.217056247867']
PmSTa 8 ['S0.444444Pm0.333333Ta0.222222', '-1.839', '-0.20068771627']
PmSTb 5 ['S0.428571Pm0.428571Tb0.142857', '-1.805', '-0.201843574349']
PmSTc 33 ['S0.625Tc0.25Pm0.125', '-1.422', '-0.204573275304']
PmSTe 29 ['S0.111111Te0.555556Pm0.333333', '-1.317', '-0.232743911188']
PmSTh 11 ['S0.428571Pm0.285714Th0.285714', '-1.88', '-0.206545452206']
PmSTi 12 ['S0.333333Ti0.166667Pm0.5', '-1.463', '-0.216100557827']
PmSTl 12 ['S0.5Pm0.375Tl0.125', '-2.102', '-0.235252101429']
PmSTm 6 ['S0.5Pm0.166667Tm0.333333', '-2.209', '-0.204756660907']
PmSU 1 ['S0.4Pm0.5U0.1', '-1.729', '-0.232720669392']
PmSV 4 ['S0.428571V0.142857Pm0.428571', '-1.839', '-0.235843574349']
PmSW 3 ['S0.428571Pm0.428571W0.142857', '-1.838', '-0.234843574349']
PmSXe 10 ['S0.5Xe0.125Pm0.375', '-2.047', '-0.203898680804']
PmSY 5 ['S0.5Y0.2Pm0.3', '-2.191', '-0.21239413224']
PmSYb 9 ['S0.444444Pm0.333333Yb0.222222', '-2.113', '-0.211152670991']
PmSZn 7 ['S0.333333Zn0.111111Pm0.555556', '-1.559', '-0.239633396438']
PmSZr 17 ['S0.428571Zr0.285714Pm0.285714', '-1.778', '-0.205983967564']
PmSbSe 30 ['Se0.5Sb0.25Pm0.25', '-1.123', '-0.202261198542']
PmSbTe 1 ['Sb0.1Te0.5Pm0.4', '-1.272', '-0.206546088331']
PmScSe 22 ['Sc0.1Se0.5Pm0.4', '-1.825', '-0.2094134885']
PmSeSi 26 ['Si0.166667Se0.333333Pm0.5', '-1.43', '-0.203133322917']
PmSeSm 7 ['Se0.6Pm0.2Sm0.2', '-1.837', '-0.214885415801']
PmSeSn 29 ['Se0.375Sn0.375Pm0.25', '-1.15', '-0.218870377893']
PmSeSr 12 ['Se0.625Sr0.25Pm0.125', '-1.642', '-0.200462402187']
PmSeTa 18 ['Se0.7Pm0.1Ta0.2', '-1.059', '-0.20662648625']
PmSeTb 5 ['Se0.555556Pm0.333333Tb0.111111', '-1.743', '-0.212820612258']
PmSeTc 25 ['Se0.444444Tc0.111111Pm0.444444', '-1.658', '-0.21712871']
PmSeTe 70 ['Se0.666667Te0.222222Pm0.111111', '-0.563', '-0.2027821775']
PmSeTh 5 ['Se0.666667Pm0.166667Th0.166667', '-1.649', '-0.205455063125']
PmSeTi 14 ['Ti0.111111Se0.444444Pm0.444444', '-1.672', '-0.23112871']
PmSeTl 31 ['Se0.666667Pm0.222222Tl0.111111', '-0.982', '-0.200375640556']
PmSeTm 12 ['Se0.555556Pm0.111111Tm0.333333', '-1.824', '-0.223459365279']
PmSeU 7 ['Se0.555556Pm0.333333U0.111111', '-1.685', '-0.2257781015']
PmSeV 13 ['V0.111111Se0.666667Pm0.222222', '-1.184', '-0.220459809222']
PmSeW 11 ['Se0.666667Pm0.166667W0.166667', '-1.021', '-0.20315697375']
PmSeXe 26 ['Se0.5Xe0.4Pm0.1', '-0.529', '-0.20480395975']
PmSeY 17 ['Se0.5Y0.125Pm0.375', '-1.859', '-0.201789542188']
PmSeYb 4 ['Se0.625Pm0.25Yb0.125', '-1.648', '-0.310970055625']

PmSeZn 24 ['Zn0.333333Se0.333333Pm0.333333', '-1.286', '-0.204724013611']
PmSeZr 22 ['Se0.5Zr0.375Pm0.125', '-1.468', '-0.211740134583']
PmSnTe 1 ['Sn0.1Te0.6Pm0.3', '-1.063', '-0.21357925023']
PmSrTe 1 ['Sr0.1Te0.6Pm0.3', '-1.362', '-0.229624822914']
PmTeTh 1 ['Te0.625Pm0.25Th0.125', '-1.298', '-0.228008165684']
PmTeTl 4 ['Te0.571429Pm0.142857Tl0.285714', '-0.715', '-0.206275341309']
PmTeYb 3 ['Te0.571429Pm0.285714Yb0.142857', '-1.429', '-0.203295243569']
PrPtS 3 ['S0.4Pr0.2Pt0.4', '-1.444', '-0.20064120474']
PrPtSe 18 ['Se0.5Pr0.4Pt0.1', '-1.936', '-0.204945066873']
PrPtTh 2 ['Pr0.111111Pt0.777778Th0.111111', '-0.944', '-0.202685558611']
PrPuS 3 ['S0.375Pr0.375Pu0.25', '-1.829', '-0.20289090068']
PrPuTe 1 ['Te0.6Pr0.3Pu0.1', '-1.507', '-0.204000244749']
PrRbTe 1 ['Rb0.111111Te0.555556Pr0.333333', '-1.592', '-0.25870595815']
PrReSi 3 ['Si0.4Pr0.1Re0.5', '-0.569', '-0.227463886393']
PrRhS 5 ['S0.375Rh0.25Pr0.375', '-1.856', '-0.221912400056']
PrRhSb 4 ['Rh0.4Sb0.3Pr0.3', '-1.018', '-0.229933220917']
PrRhSe 27 ['Se0.5Rh0.125Pr0.375', '-1.859', '-0.224040607185']
PrRhSi 3 ['Si0.444444Rh0.444444Pr0.111111', '-1.201', '-0.236147798611']
PrRuSe 3 ['Se0.333333Ru0.222222Pr0.444444', '-1.466', '-0.206464127222']
PrRuSi 3 ['Si0.555556Ru0.333333Pr0.111111', '-0.939', '-0.226256419444']
PrSSc 1 ['S0.571429Sc0.285714Pr0.142857', '-2.335', '-0.213436521276']
PrSSi 1 ['Si0.25S0.375Pr0.375', '-1.858', '-0.23189090068']
PrSTc 8 ['S0.5Tc0.25Pr0.25', '-1.78', '-0.232062999242']
PrSTe 1 ['S0.2Te0.4Pr0.4', '-1.883', '-0.206047917198']
PrSZr 4 ['S0.5Zr0.375Pr0.125', '-1.883', '-0.223909207052']
PrSbSe 7 ['Se0.5Sb0.125Pr0.375', '-1.835', '-0.200040607185']
PrScSe 9 ['Sc0.1Se0.6Pr0.3', '-1.949', '-0.223913110956']
PrSeSi 2 ['Si0.3Se0.3Pr0.4', '-1.509', '-0.215378767999']
PrSeTa 5 ['Se0.571429Pr0.285714Ta0.142857', '-1.695', '-0.211734179125']
PrSeTc 1 ['Se0.6Tc0.3Pr0.1', '-0.699', '-0.20678089875']
PrSeTe 12 ['Se0.25Te0.5Pr0.25', '-1.357', '-0.20005756021']
PrSeTi 1 ['Ti0.222222Se0.555556Pr0.222222', '-1.763', '-0.21542809513']
PrSeTl 5 ['Se0.5Pr0.375Tl0.125', '-1.838', '-0.203040607185']
PrSeTm 4 ['Se0.6Pr0.2Tm0.2', '-1.906', '-0.2081837885']
PrSeV 1 ['V0.1Se0.6Pr0.3', '-1.763', '-0.221298394887']
PrSeY 11 ['Se0.666667Y0.166667Pr0.166667', '-1.633', '-0.22400108875']
PrSeZn 13 ['Zn0.1Se0.5Pr0.4', '-1.891', '-0.20436166341']
PrSeZr 10 ['Se0.5Zr0.25Pr0.25', '-1.733', '-0.220188388031']
PrTeY 1 ['Y0.3Te0.6Pr0.1', '-1.497', '-0.227214238085']
PtPuS 27 ['S0.3Pt0.3Pu0.4', '-1.628', '-0.202974921461']
PtPuSe 9 ['Se0.285714Pt0.285714Pu0.428571', '-1.468', '-0.221421820714']
PtPuSi 1 ['Si0.5Pt0.375Pu0.125', '-0.971', '-0.241488193437']
PtPuSr 1 ['Sr0.111111Pt0.444444Pu0.444444', '-0.931', '-0.215515871111']
PtRbTh 1 ['Rb0.1Pt0.8Th0.1', '-0.451', '-0.2067340785']

PtReTh 1 ['Re0.1Pt0.8Th0.1', '-0.473', '-0.2287340785']
PtSSb 12 ['S0.428571Sb0.142857Pt0.428571', '-0.738', '-0.201702159351']
PtSSc 4 ['S0.3Sc0.4Pt0.3', '-1.837', '-0.207746737794']
PtSSe 13 ['S0.222222Se0.555556Pt0.222222', '-0.505', '-0.200480972442']
PtSSi 10 ['Si0.125S0.5Pt0.375', '-0.859', '-0.200592908931']
PtSSm 15 ['S0.428571Sm0.428571Pt0.142857', '-2.164', '-0.202143119529']
PtSSn 22 ['S0.285714Sn0.428571Pt0.285714', '-0.822', '-0.201825626988']
PtSTb 2 ['S0.25Tb0.375Pt0.375', '-1.58', '-0.229656308707']
PtSTc 6 ['S0.5Tc0.25Pt0.25', '-0.814', '-0.208663187993']
PtSTe 4 ['S0.428571Te0.142857Pt0.428571', '-0.729', '-0.212129336375']
PtSTh 28 ['S0.5Pt0.25Th0.25', '-1.841', '-0.204215611115']
PtSTi 10 ['S0.5Ti0.166667Pt0.333333', '-1.169', '-0.209322908482']
PtSTm 4 ['S0.444444Tm0.222222Pt0.333333', '-1.616', '-0.223496818632']
PtSU 8 ['S0.333333Pt0.444444U0.222222', '-1.177', '-0.200663013848']
PtSV 24 ['S0.333333V0.222222Pt0.444444', '-0.907', '-0.210018592293']
PtSW 2 ['S0.6W0.2Pt0.2', '-1.054', '-0.228097018589']
PtSY 9 ['S0.222222Y0.444444Pt0.333333', '-1.822', '-0.210610677007']
PtSZn 35 ['S0.3Zn0.2Pt0.5', '-0.772', '-0.209920872296']
PtSZr 21 ['S0.5Zr0.166667Pt0.333333', '-1.29', '-0.212696795158']
PtSbTh 5 ['Sb0.1Pt0.8Th0.1', '-0.549', '-0.21207138475']
PtSbU 3 ['Sb0.333333Pt0.333333U0.333333', '-0.748', '-0.209608516667']
PtSbY 2 ['Y0.4Sb0.2Pt0.4', '-1.441', '-0.203591485502']
PtScSe 27 ['Sc0.285714Se0.285714Pt0.428571', '-1.306', '-0.202195826327']
PtScTh 2 ['Sc0.111111Pt0.777778Th0.111111', '-1.007', '-0.270391427778']
PtSeSm 19 ['Se0.285714Sm0.428571Pt0.285714', '-1.724', '-0.210739104167']
PtSeTb 5 ['Se0.3Tb0.3Pt0.4', '-1.375', '-0.22380054547']
PtSeTc 1 ['Se0.6Tc0.3Pt0.1', '-0.365', '-0.249623038']
PtSeTh 25 ['Se0.4Pt0.3Th0.3', '-1.513', '-0.225098334498']
PtSeTm 6 ['Se0.444444Tm0.222222Pt0.333333', '-1.287', '-0.203171977779']
PtSeU 16 ['Se0.428571Pt0.428571U0.142857', '-0.841', '-0.206381855054']
PtSeY 40 ['Se0.5Y0.166667Pt0.333333', '-1.035', '-0.2078991275']
PtSeYb 2 ['Se0.285714Yb0.285714Pt0.428571', '-1.407', '-0.203480357143']
PtSeZr 1 ['Se0.5Zr0.4Pt0.1', '-1.466', '-0.221169896972']
PtSiSr 4 ['Si0.6Sr0.1Pt0.3', '-0.822', '-0.25490148575']
PtSiTb 2 ['Si0.5Tb0.125Pt0.375', '-0.993', '-0.260154215979']
PtSiTh 6 ['Si0.1Pt0.7Th0.2', '-0.921', '-0.2156755165']
PtSiY 1 ['Si0.5Y0.1Pt0.4', '-1.084', '-0.267743463']
PtSiYb 11 ['Si0.111111Yb0.111111Pt0.777778', '-0.808', '-0.202601562777']
PtSiZr 1 ['Si0.5Zr0.125Pt0.375', '-1.032', '-0.260244525001']
PtSmTh 3 ['Sm0.1Pt0.7Th0.2', '-1.086', '-0.23301740325']
PtSnSr 1 ['Sr0.125Sn0.5Pt0.375', '-0.899', '-0.211130471574']
PtSnTh 6 ['Sn0.2Pt0.7Th0.1', '-0.773', '-0.20155035913']
PtTaTh 1 ['Ta0.1Pt0.8Th0.1', '-0.784', '-0.25174029075']
PtTbTh 2 ['Tb0.111111Pt0.777778Th0.111111', '-0.884', '-0.240388280037']

PtTcTh 2 ['Tc0.111111Pt0.777778Th0.111111', '-0.488', '-0.206566594444']
PtTeTh 5 ['Te0.1Pt0.7Th0.2', '-0.778', '-0.220682833167']
PtThTl 10 ['Pt0.6Tl0.2Th0.2', '-0.749', '-0.2056762705']
PtThTm 2 ['Tm0.111111Pt0.777778Th0.111111', '-1.018', '-0.269947172778']
PtThU 1 ['Pt0.8Th0.1U0.1', '-0.808', '-0.2650672405']
PtThW 2 ['W0.111111Pt0.777778Th0.111111', '-0.54', '-0.247206832778']
PtThXe 8 ['Xe0.166667Pt0.666667Th0.166667', '-0.64', '-0.232890130833']
PtThY 3 ['Y0.1Pt0.7Th0.2', '-1.109', '-0.2280147865']
PtThYb 6 ['Yb0.111111Pt0.666667Th0.222222', '-1.122', '-0.214669115554']
PtThZn 5 ['Zn0.142857Pt0.714286Th0.142857', '-0.77', '-0.234850886429']
PtThZr 2 ['Zr0.1Pt0.8Th0.1', '-0.95', '-0.2977231425']
PtUYb 1 ['Yb0.1Pt0.8U0.1', '-0.846', '-0.219267208999']
PuRbS 21 ['S0.5Rb0.4Pu0.1', '-1.469', '-0.207726446744']
PuRbSe 1 ['Se0.5Rb0.166667Pu0.333333', '-1.621', '-0.258841551665']
PuReS 2 ['S0.5Re0.125Pu0.375', '-1.828', '-0.201727902366']
PuRhS 2 ['S0.4Rh0.3Pu0.3', '-1.519', '-0.217982321893']
PuRhSi 6 ['Si0.428571Rh0.428571Pu0.142857', '-1.098', '-0.202168096071']
PuRuSe 10 ['Se0.3Ru0.2Pu0.5', '-1.192', '-0.200090721']
PuSSc 13 ['S0.4Sc0.2Pu0.4', '-1.789', '-0.214441415392']
PuSSe 1 ['S0.142857Se0.428571Pu0.428571', '-1.76', '-0.216828169424']
PuSSm 11 ['S0.5Sm0.3Pu0.2', '-2.276', '-0.20605197574']
PuSSr 13 ['S0.666667Sr0.222222Pu0.111111', '-1.789', '-0.2002000201']
PuSTa 18 ['S0.5Ta0.1Pu0.4', '-1.946', '-0.207945942803']
PuSTb 1 ['S0.444444Tb0.444444Pu0.111111', '-1.865', '-0.221526281104']
PuSTc 27 ['S0.444444Tc0.111111Pu0.444444', '-1.922', '-0.216420135991']
PuSTe 5 ['S0.4Te0.2Pu0.4', '-1.792', '-0.213815123226']
PuSTh 1 ['S0.428571Th0.285714Pu0.285714', '-1.893', '-0.205708828277']
PuSTl 4 ['S0.444444Tl0.222222Pu0.333333', '-1.647', '-0.201424802103']
PuSTm 4 ['S0.428571Tm0.285714Pu0.285714', '-1.951', '-0.219240513991']
PuSW 3 ['S0.5W0.166667Pu0.333333', '-1.735', '-0.204618386323']
PuSY 1 ['S0.4Y0.4Pu0.2', '-1.921', '-0.208207260392']
PuSYb 1 ['S0.444444Yb0.111111Pu0.444444', '-2.066', '-0.25152458488']
PuSZn 2 ['S0.5Zn0.125Pu0.375', '-1.889', '-0.210916380491']
PuSZr 8 ['S0.555556Zr0.222222Pu0.222222', '-1.952', '-0.2006502411']
PuSbTe 9 ['Sb0.3Te0.3Pu0.4', '-1.054', '-0.200749003499']
PuScSe 9 ['Sc0.375Se0.5Pu0.125', '-1.784', '-0.201999266875']
PuScTe 1 ['Sc0.1Te0.5Pu0.4', '-1.338', '-0.21988625683']
PuSeTa 9 ['Se0.571429Ta0.142857Pu0.285714', '-1.446', '-0.212211478571']
PuSeTc 10 ['Se0.6Tc0.1Pu0.3', '-1.368', '-0.206186168997']
PuSeTe 12 ['Se0.222222Te0.333333Pu0.444444', '-1.441', '-0.200436714444']
PuSeTh 1 ['Se0.444444Th0.333333Pu0.222222', '-1.612', '-0.235681471111']
PuSeTi 3 ['Ti0.333333Se0.5Pu0.166667', '-1.49', '-0.200944623321']
PuSeTl 26 ['Se0.5Tl0.3Pu0.2', '-1.072', '-0.200948917199']
PuSeTm 9 ['Se0.6Tm0.1Pu0.3', '-1.667', '-0.207600628']

PuSeU 1 ['Se0.571429U0.142857Pu0.285714', '-1.581', '-0.210859060143']
PuSeY 18 ['Se0.5Y0.2Pu0.3', '-1.837', '-0.203058904']
PuSeYb 1 ['Se0.444444Yb0.111111Pu0.444444', '-1.706', '-0.207319466667']
PuSeZr 16 ['Se0.6Zr0.3Pu0.1', '-1.559', '-0.222183748972']
PuSiTc 8 ['Si0.5Tc0.375Pu0.125', '-0.729', '-0.238862338125']
PuSiTe 1 ['Si0.1Te0.5Pu0.4', '-1.166', '-0.225974736331']
PuSnTe 4 ['Sn0.142857Te0.428571Pu0.428571', '-1.186', '-0.232087768662']
PuTbTe 3 ['Te0.6Tb0.3Pu0.1', '-1.19', '-0.200783612599']
PuTeTi 1 ['Ti0.1Te0.5Pu0.4', '-1.241', '-0.211224901327']
PuTeY 2 ['Y0.333333Te0.555556Pu0.111111', '-1.467', '-0.251443718147']
RbReS 18 ['S0.555556Rb0.111111Re0.333333', '-0.954', '-0.208473326972']
RbSSb 1 ['S0.3Rb0.6Sb0.1', '-1.224', '-0.2306785518']
RbSSc 2 ['S0.5Sc0.3Rb0.2', '-1.989', '-0.208510041493']
RbSSe 36 ['S0.1Se0.3Rb0.6', '-1.242', '-0.20195967393']
RbSSm 2 ['S0.5Rb0.125Sm0.375', '-2.293', '-0.21601081143']
RbSSn 1 ['S0.444444Rb0.444444Sn0.111111', '-1.196', '-0.215442271531']
RbSTc 7 ['S0.5Rb0.375Tc0.125', '-1.194', '-0.211993959662']
RbSTe 28 ['S0.166667Rb0.333333Te0.5', '-0.887', '-0.201631066276']
RbSTh 6 ['S0.5Rb0.2Th0.3', '-2.002', '-0.205914683993']
RbSTl 1 ['S0.333333Rb0.333333Tl0.333333', '-1.015', '-0.241158826257']
RbSU 10 ['S0.555556Rb0.111111U0.333333', '-1.764', '-0.207064942908']
RbSXe 1 ['S0.5Rb0.1Xe0.4', '-0.484', '-0.203066432872']
RbSY 5 ['S0.6Rb0.3Y0.1', '-1.49', '-0.203724584841']
RbSZr 13 ['S0.428571Rb0.142857Zr0.428571', '-1.532', '-0.202364930778']
RbScSe 14 ['Sc0.2Se0.5Rb0.3', '-1.416', '-0.213792731']
RbSeSi 8 ['Si0.3Se0.5Rb0.2', '-0.8', '-0.208578006999']
RbSeSm 2 ['Se0.5Rb0.166667Sm0.333333', '-1.97', '-0.202035932497']
RbSeTa 7 ['Se0.5Rb0.3Ta0.2', '-1.115', '-0.204080698415']
RbSeTb 1 ['Se0.5Rb0.1Tb0.4', '-1.71', '-0.210862876134']
RbSeTc 10 ['Se0.666667Rb0.222222Tc0.111111', '-0.671', '-0.210943324444']
RbSeTe 34 ['Se0.2Rb0.3Te0.5', '-0.79', '-0.203783905444']
RbSeTh 5 ['Se0.5Rb0.25Th0.25', '-1.644', '-0.204766031247']
RbSeTl 9 ['Se0.333333Rb0.166667Tl0.5', '-0.695', '-0.207758783333']
RbSeTm 10 ['Se0.444444Rb0.333333Tm0.222222', '-1.569', '-0.23189997111']
RbSeU 2 ['Se0.5Rb0.333333U0.166667', '-1.322', '-0.221250260165']
RbSeY 19 ['Se0.428571Rb0.428571Y0.142857', '-1.433', '-0.202829489284']
RbSeZn 3 ['Zn0.3Se0.4Rb0.3', '-1.04', '-0.201045263499']
RbSeZr 10 ['Se0.625Rb0.125Zr0.25', '-1.445', '-0.20889214']
RbSmTe 1 ['Rb0.142857Te0.571429Sm0.285714', '-1.442', '-0.220053332021']
RbTbTe 3 ['Rb0.1Te0.6Tb0.3', '-1.229', '-0.2643951021']
RbTeTm 3 ['Rb0.142857Te0.571429Tm0.285714', '-1.331', '-0.258152084168']
RbTeY 3 ['Rb0.1Y0.3Te0.6', '-1.546', '-0.287726286']
ReSSc 1 ['S0.5Sc0.4Re0.1', '-2.056', '-0.203093367991']
ReSSe 8 ['S0.428571Se0.428571Re0.142857', '-0.522', '-0.201098139032']

ReSTi 1 ['S0.555556Ti0.333333Re0.111111', '-1.532', '-0.20360183762']
ReSTl 4 ['S0.555556Re0.222222Tl0.222222', '-0.852', '-0.204822044221']
ReSXe 2 ['S0.5Xe0.375Re0.125', '-0.483', '-0.202210871653']
ReSZn 2 ['S0.5Zn0.25Re0.25', '-0.964', '-0.205209653149']
ReSZr 4 ['S0.428571Zr0.428571Re0.142857', '-1.522', '-0.228688591134']
ReScSe 4 ['Sc0.3Se0.6Re0.1', '-1.276', '-0.20136086775']
ReSeTc 2 ['Se0.625Tc0.25Re0.125', '-0.4', '-0.252209899063']
ReSeY 3 ['Se0.555556Y0.333333Re0.111111', '-1.51', '-0.201363536389']
ReSiTb 3 ['Si0.5Tb0.125Re0.375', '-0.57', '-0.20415006272']
ReSiTh 1 ['Si0.428571Re0.428571Th0.142857', '-0.726', '-0.227856186084']
RhSSb 7 ['S0.333333Rh0.333333Sb0.333333', '-0.768', '-0.219869444542']
RhSSc 27 ['S0.5Sc0.1Rh0.4', '-1.12', '-0.200098770368']
RhSSe 11 ['S0.25Se0.375Rh0.375', '-0.72', '-0.20676263259']
RhSSm 20 ['S0.444444Rh0.444444Sm0.111111', '-1.141', '-0.202305298493']
RhSTb 1 ['S0.444444Rh0.222222Tb0.333333', '-1.784', '-0.251149333886']
RhSTh 6 ['S0.5Rh0.375Th0.125', '-1.305', '-0.203056565179']
RhSTi 18 ['S0.6Ti0.3Rh0.1', '-1.538', '-0.211637735901']
RhSTm 4 ['S0.444444Rh0.333333Tm0.222222', '-1.582', '-0.203498210854']
RhSU 1 ['S0.5Rh0.375U0.125', '-1.132', '-0.238683866706']
RhSV 3 ['S0.5V0.166667Rh0.333333', '-0.981', '-0.20268086945']
RhSXe 2 ['S0.5Rh0.1Xe0.4', '-0.373', '-0.202469213697']
RhSY 1 ['S0.5Y0.166667Rh0.333333', '-1.442', '-0.220387886534']
RhSYb 1 ['S0.375Rh0.5Yb0.125', '-1.086', '-0.200246927869']
RhSZn 31 ['S0.571429Zn0.285714Rh0.142857', '-0.992', '-0.202097484135']
RhSZr 25 ['S0.333333Zr0.444444Rh0.222222', '-1.468', '-0.20804436666']
RhScSe 46 ['Sc0.3Se0.6Rh0.1', '-1.302', '-0.201312179833']
RhScSi 9 ['Si0.5Sc0.125Rh0.375', '-1.078', '-0.205731000313']
RhSeSm 26 ['Se0.555556Rh0.111111Sm0.333333', '-1.82', '-0.204300382292']
RhSeTb 16 ['Se0.5Rh0.2Tb0.3', '-1.451', '-0.20665519841']
RhSeTc 4 ['Se0.666667Tc0.222222Rh0.111111', '-0.373', '-0.212688034815']
RhSeTi 4 ['Ti0.25Se0.5Rh0.25', '-1.072', '-0.205924039209']
RhSeTm 29 ['Se0.571429Rh0.285714Tm0.142857', '-1.025', '-0.201799581787']
RhSeY 47 ['Se0.666667Y0.166667Rh0.166667', '-1.031', '-0.201898309722']
RhSeZn 4 ['Zn0.3Se0.4Rh0.3', '-0.7', '-0.207533543125']
RhSeZr 19 ['Se0.3Zr0.4Rh0.3', '-1.248', '-0.204647459687']
RhSiSm 4 ['Si0.555556Rh0.333333Sm0.111111', '-1.005', '-0.202015338889']
RhSiSr 8 ['Si0.5Sr0.1Rh0.4', '-0.97', '-0.216640879']
RhSiTb 6 ['Si0.4Rh0.5Tb0.1', '-1.117', '-0.211965129908']
RhSiTh 2 ['Si0.444444Rh0.444444Th0.111111', '-1.18', '-0.201718121667']
RhSiTm 2 ['Si0.4Rh0.5Tm0.1', '-1.198', '-0.232803066542']
RhSiU 2 ['Si0.5Rh0.375U0.125', '-1.012', '-0.264683777656']
RhSiY 6 ['Si0.4Y0.1Rh0.5', '-1.152', '-0.201816921625']
RhSiYb 4 ['Si0.444444Rh0.444444Yb0.111111', '-1.147', '-0.203750051667']
RhSiZr 14 ['Si0.5Zr0.125Rh0.375', '-1.084', '-0.232135060313']

RhSmSn 1 ['Rh0.375Sn0.5Sm0.125', '-0.845', '-0.200874944283']
RhSnTb 3 ['Rh0.428571Sn0.285714Tb0.285714', '-0.859', '-0.213946376888']
RhTcZr 1 ['Zr0.625Tc0.125Rh0.25', '-0.823', '-0.279520409375']
RuSSc 16 ['S0.333333Sc0.5Ru0.166667', '-1.732', '-0.20897425116']
RuSSm 6 ['S0.5Ru0.3Sm0.2', '-1.545', '-0.200965534242']
RuSTb 2 ['S0.571429Ru0.142857Tb0.285714', '-1.781', '-0.200732413877']
RuSTh 10 ['S0.428571Ru0.285714Th0.285714', '-1.731', '-0.207708583993']
RuSTi 12 ['S0.5Ti0.333333Ru0.166667', '-1.467', '-0.205927145065']
RuSTm 2 ['S0.5Ru0.25Tm0.25', '-1.762', '-0.214532055023']
RuSU 4 ['S0.625Ru0.25U0.125', '-1.205', '-0.202721732285']
RuSY 1 ['S0.555556Y0.222222Ru0.222222', '-1.72', '-0.223156076103']
RuSZr 16 ['S0.555556Zr0.333333Ru0.111111', '-1.697', '-0.206600628878']
RuScSe 10 ['Sc0.555556Se0.333333Ru0.111111', '-1.389', '-0.207694605556']
RuScSi 2 ['Si0.333333Sc0.222222Ru0.444444', '-0.958', '-0.202504075185']
RuScSn 1 ['Sc0.444444Ru0.444444Sn0.111111', '-0.755', '-0.20171232094']
RuSeTb 3 ['Se0.5Ru0.125Tb0.375', '-1.575', '-0.202283626374']
RuSeTc 6 ['Se0.6Tc0.2Ru0.2', '-0.472', '-0.2162043895']
RuSeTh 8 ['Se0.4Ru0.2Th0.4', '-1.465', '-0.207546378998']
RuSeTm 12 ['Se0.3Ru0.3Tm0.4', '-1.275', '-0.200114981']
RuSeU 8 ['Se0.3Ru0.4U0.3', '-0.921', '-0.202399605']
RuSeY 27 ['Se0.555556Y0.111111Ru0.333333', '-0.879', '-0.20236016']
RuSeZr 2 ['Se0.555556Zr0.333333Ru0.111111', '-1.401', '-0.217489372778']
RuSiTb 4 ['Si0.5Ru0.4Tb0.1', '-0.927', '-0.228607974284']
RuSiZr 2 ['Si0.333333Zr0.166667Ru0.5', '-0.887', '-0.200390666111']
SSbSc 4 ['S0.333333Sc0.5Sb0.166667', '-1.86', '-0.200797046993']
SSbSm 8 ['S0.5Sb0.1Sm0.4', '-2.241', '-0.212021034658']
SSbTc 11 ['S0.5Tc0.3Sb0.2', '-0.807', '-0.201663187994']
SSbTh 2 ['S0.4Sb0.2Th0.4', '-2.052', '-0.330706438518']
SSbTm 5 ['S0.285714Sb0.285714Tm0.428571', '-1.799', '-0.216643368733']
SSbXe 3 ['S0.5Sb0.1Xe0.4', '-0.316', '-0.208596611023']
SSbY 3 ['S0.428571Y0.428571Sb0.142857', '-2.157', '-0.215169387564']
SSbZr 1 ['S0.444444Zr0.444444Sb0.111111', '-1.6', '-0.210280450991']
SScSe 20 ['S0.4Sc0.3Se0.3', '-1.707', '-0.20625390156']
SScSn 4 ['S0.4Sc0.5Sn0.1', '-1.943', '-0.201010436207']
SScTa 8 ['S0.6Sc0.2Ta0.2', '-1.825', '-0.203590177403']
SScTc 7 ['S0.5Sc0.2Tc0.3', '-1.488', '-0.200006288992']
SScTh 8 ['S0.555556Sc0.222222Th0.222222', '-2.282', '-0.200498181101']
SScTl 6 ['S0.333333Sc0.444444Tl0.222222', '-1.613', '-0.210426581438']
SScXe 1 ['S0.5Sc0.1Xe0.4', '-0.728', '-0.205070506897']
SScZr 16 ['S0.6Sc0.1Zr0.3', '-1.95', '-0.214014740589']
SSeSr 16 ['S0.222222Se0.555556Sr0.222222', '-1.222', '-0.201965919107']
SSeTc 34 ['S0.375Se0.5Tc0.125', '-0.512', '-0.209331593997']
SSeTl 9 ['S0.166667Se0.5Tl0.333333', '-0.431', '-0.203174674081']
SSeTm 4 ['S0.25Se0.375Tm0.375', '-2.037', '-0.203374549518']

SSeV 9 ['S0.5V0.2Se0.3', '-0.854', '-0.200663190994']
SSeXe 30 ['S0.5Se0.1Xe0.4', '-0.204', '-0.204']
SSeY 13 ['S0.333333Se0.333333Y0.333333', '-1.85', '-0.206829674217']
SSeZn 2 ['S0.4Zn0.4Se0.2', '-0.965', '-0.200198050394']
SSeZr 26 ['S0.375Se0.375Zr0.25', '-1.435', '-0.20193017662']
SSiSm 20 ['Si0.3S0.5Sm0.2', '-1.586', '-0.202161078554']
SSiSn 4 ['Si0.1S0.5Sn0.4', '-0.847', '-0.208674752438']
SSiTb 4 ['Si0.3S0.3Tb0.4', '-1.401', '-0.205166416682']
SSiTh 13 ['Si0.222222S0.444444Th0.333333', '-1.879', '-0.216950482102']
SSiTm 10 ['Si0.3S0.4Tm0.3', '-1.765', '-0.20757636656']
SSiY 2 ['Si0.166667S0.5Y0.333333', '-2.078', '-0.201878253825']
SSiZr 3 ['Si0.1S0.5Zr0.4', '-1.733', '-0.22644905449']
SSmSn 3 ['S0.5Sn0.125Sm0.375', '-2.162', '-0.200335736823']
SSmTc 12 ['S0.5Tc0.2Sm0.3', '-1.962', '-0.217644345867']
SSmTe 3 ['S0.4Te0.2Sm0.4', '-2.144', '-0.200154110059']
SSmTl 8 ['S0.3Sm0.4Tl0.3', '-1.632', '-0.218571055294']
SSmZn 1 ['S0.4Zn0.4Sm0.2', '-1.582', '-0.268251530644']
SSmZr 4 ['S0.571429Zr0.285714Sm0.142857', '-2.011', '-0.205989866454']
SSnTc 6 ['S0.5Tc0.4Sn0.1', '-0.823', '-0.212061660308']
SSnTe 1 ['S0.444444Sn0.333333Te0.222222', '-0.809', '-0.327954737044']
SSnTh 4 ['S0.428571Sn0.142857Th0.428571', '-1.951', '-0.206431131228']
SSnTm 1 ['S0.428571Sn0.142857Tm0.428571', '-2.079', '-0.258246672031']
SSnY 3 ['S0.4Y0.4Sn0.2', '-1.969', '-0.206626417027']
SSnZr 15 ['S0.285714Zr0.428571Sn0.285714', '-1.283', '-0.229149815571']
SSrTc 3 ['S0.6Sr0.1Tc0.3', '-1.272', '-0.207647851591']
SSrTh 3 ['S0.6Sr0.1Th0.3', '-2.385', '-0.204966916839']
SSrZr 2 ['S0.5Sr0.1Zr0.4', '-1.869', '-0.21845994199']
STaTb 2 ['S0.5Tb0.25Ta0.25', '-1.729', '-0.209938626048']
STaTc 2 ['S0.625Tc0.25Ta0.125', '-1.129', '-0.209349069991']
STaTh 19 ['S0.5Ta0.125Th0.375', '-2.035', '-0.203777291741']
STaXe 2 ['S0.6Xe0.3Ta0.1', '-0.616', '-0.207640898628']
STaY 2 ['S0.571429Y0.142857Ta0.285714', '-1.707', '-0.201794111947']
STaZr 6 ['S0.5Zr0.333333Ta0.166667', '-1.583', '-0.227534095908']
STbTc 3 ['S0.5Tc0.4Tb0.1', '-1.136', '-0.214542197403']
STbTe 3 ['S0.142857Te0.428571Tb0.428571', '-1.432', '-0.21860277969']
STbTl 2 ['S0.444444Tb0.444444Tl0.111111', '-1.825', '-0.202228329476']
STbZr 2 ['S0.5Zr0.333333Tb0.166667', '-1.834', '-0.225613620757']
STcTh 13 ['S0.5Tc0.25Th0.25', '-1.842', '-0.205215611115']
STcTi 16 ['S0.428571Ti0.142857Tc0.428571', '-1.012', '-0.212025720597']
STcTl 7 ['S0.444444Tc0.444444Tl0.111111', '-0.745', '-0.20018383797']
STcTm 10 ['S0.571429Tc0.142857Tm0.285714', '-2.005', '-0.22171646217']
STcU 3 ['S0.555556Tc0.333333U0.111111', '-1.133', '-0.20384850092']
STcV 9 ['S0.5V0.333333Tc0.166667', '-1.182', '-0.205691016879']
STcW 4 ['S0.6Tc0.2W0.2', '-1.051', '-0.205993157589']

STcXe 18 ['S0.5Tc0.4Xe0.1', '-0.806', '-0.200663187994']
STcY 7 ['S0.5Y0.2Tc0.3', '-1.595', '-0.227192227492']
STcYb 1 ['S0.5Tc0.4Yb0.1', '-1.177', '-0.210969084993']
STcZn 6 ['S0.5Zn0.2Tc0.3', '-0.958', '-0.212396937993']
STcZr 14 ['S0.5Zr0.1Tc0.4', '-1.076', '-0.201975248992']
STeTh 9 ['S0.333333Te0.166667Th0.5', '-1.895', '-0.200223758103']
STeXe 6 ['S0.5Te0.166667Xe0.333333', '-0.207', '-0.207']
STeY 6 ['S0.1Y0.4Te0.5', '-1.623', '-0.227617015219']
STeZr 1 ['S0.444444Zr0.444444Te0.111111', '-1.699', '-0.269457561176']
SThTi 14 ['S0.4Ti0.2Th0.4', '-1.8', '-0.205303298392']
SThTl 6 ['S0.5Tl0.2Th0.3', '-1.939', '-0.217982253241']
SThW 1 ['S0.6W0.2Th0.2', '-1.825', '-0.214136430088']
SThZn 1 ['S0.444444Zn0.222222Th0.333333', '-1.865', '-0.216242451483']
SThZr 18 ['S0.6Zr0.1Th0.3', '-2.233', '-0.214101072839']
STiZr 13 ['S0.444444Ti0.333333Zr0.222222', '-1.632', '-0.20031177652']
STlTm 5 ['S0.5Tm0.4Tl0.1', '-2.158', '-0.202292715116']
STlZr 8 ['S0.555556Zr0.333333Tl0.111111', '-1.667', '-0.200859435544']
STmZr 4 ['S0.571429Zr0.285714Tm0.142857', '-2.011', '-0.202644772079']
SUZr 7 ['S0.5Zr0.375U0.125', '-1.705', '-0.207802543615']
SVW 2 ['S0.555556V0.222222W0.222222', '-1.209', '-0.212216661841']
SVZr 9 ['S0.428571V0.285714Zr0.285714', '-1.393', '-0.200485799809']
SWXe 1 ['S0.555556Xe0.333333W0.111111', '-0.549', '-0.214071045773']
SWZr 6 ['S0.555556Zr0.333333W0.111111', '-1.685', '-0.218859435544']
SXeZn 4 ['S0.5Zn0.166667Xe0.333333', '-0.521', '-0.202332520998']
SXeZr 1 ['S0.5Zr0.4Xe0.1', '-1.744', '-0.33953379499']
SYZr 6 ['S0.555556Y0.111111Zr0.333333', '-1.939', '-0.202387992489']
SYbZr 2 ['S0.5Zr0.375Yb0.125', '-1.943', '-0.22374374174']
SZnZr 11 ['S0.5Zn0.2Zr0.3', '-1.541', '-0.20755211674']
SbScSe 19 ['Sc0.4Se0.3Sb0.3', '-1.345', '-0.2001188225']
SbSeSm 2 ['Se0.25Sb0.375Sm0.375', '-1.522', '-0.236287920729']
SbSeTb 6 ['Se0.5Sb0.125Tb0.375', '-1.618', '-0.200285122778']
SbSeTc 11 ['Se0.571429Tc0.142857Sb0.285714', '-0.393', '-0.204002227143']
SbSeTh 2 ['Se0.5Sb0.125Th0.375', '-1.758', '-0.21810856781']
SbSeTm 11 ['Se0.3Sb0.3Tm0.4', '-1.419', '-0.204145113752']
SbSeU 4 ['Se0.3Sb0.3U0.4', '-0.928', '-0.200311712']
SbSeY 22 ['Se0.222222Y0.333333Sb0.444444', '-1.245', '-0.200590142222']
SbSeYb 1 ['Se0.333333Sb0.111111Yb0.555556', '-1.997', '-0.301754827778']
SbSeZr 5 ['Se0.428571Zr0.428571Sb0.142857', '-1.316', '-0.212069263571']
SbSrZn 5 ['Zn0.222222Sr0.333333Sb0.444444', '-0.927', '-0.2338136725']
ScSeSi 24 ['Si0.1Sc0.3Se0.6', '-1.293', '-0.20075108']
ScSeSm 4 ['Sc0.285714Se0.571429Sm0.142857', '-1.809', '-0.202620067358']
ScSeSn 16 ['Sc0.444444Se0.333333Sn0.222222', '-1.459', '-0.218930714866']
ScSeSr 4 ['Sc0.25Se0.625Sr0.125', '-1.621', '-0.30584785']
ScSeTa 12 ['Sc0.125Se0.625Ta0.25', '-1.213', '-0.201334824375']

ScSeTb 4 ['Sc0.285714Se0.571429Tb0.142857', '-1.705', '-0.216174243975']
ScSeTc 30 ['Sc0.333333Se0.444444Tc0.222222', '-1.266', '-0.203325498333']
ScSeTe 21 ['Sc0.3Se0.4Te0.3', '-1.165', '-0.2085929485']
ScSeTh 9 ['Sc0.111111Se0.555556Th0.333333', '-1.824', '-0.201464092777']
ScSeTi 9 ['Sc0.25Ti0.125Se0.625', '-1.42', '-0.203049962026']
ScSeTl 24 ['Sc0.625Se0.25Tl0.125', '-1.113', '-0.21149836']
ScSeTm 9 ['Sc0.2Se0.6Tm0.2', '-1.771', '-0.225913249002']
ScSeU 9 ['Sc0.333333Se0.5U0.166667', '-1.601', '-0.207863064167']
ScSeV 8 ['Sc0.285714V0.142857Se0.571429', '-1.415', '-0.212237535714']
ScSeW 5 ['Sc0.285714Se0.571429W0.142857', '-1.354', '-0.205265033571']
ScSeXe 10 ['Sc0.285714Se0.571429Xe0.142857', '-1.123', '-0.212136141429']
ScSeY 13 ['Sc0.333333Se0.555556Y0.111111', '-1.782', '-0.326903003333']
ScSeYb 3 ['Sc0.285714Se0.571429Yb0.142857', '-1.771', '-0.25837632']
ScSeZn 4 ['Sc0.2Zn0.3Se0.5', '-1.274', '-0.205178476']
ScSeZr 8 ['Sc0.222222Se0.555556Zr0.222222', '-1.619', '-0.213572554414']
ScTcTh 1 ['Sc0.222222Tc0.444444Th0.333333', '-0.232', '-0.201270847778']
SeSiSm 3 ['Si0.375Se0.25Sm0.375', '-1.356', '-0.232907639792']
SeSiTb 5 ['Si0.2Se0.4Tb0.4', '-1.428', '-0.215544781465']
SeSiTc 6 ['Si0.142857Se0.571429Tc0.285714', '-0.42', '-0.225940187857']
SeSiTh 10 ['Si0.222222Se0.444444Th0.333333', '-1.567', '-0.200274667776']
SeSiTm 3 ['Si0.1Se0.5Tm0.4', '-1.658', '-0.210434716001']
SeSiY 19 ['Si0.125Se0.625Y0.25', '-1.253', '-0.200247050625']
SeSiYb 12 ['Si0.25Se0.375Yb0.375', '-1.782', '-0.20238046875']
SeSmTc 1 ['Se0.6Tc0.3Sm0.1', '-0.73', '-0.24313874815']
SeSmTe 3 ['Se0.555556Te0.111111Sm0.333333', '-1.809', '-0.210161791598']
SeSmTm 1 ['Se0.6Sm0.1Tm0.3', '-1.857', '-0.204974720001']
SeSmY 5 ['Se0.571429Y0.285714Sm0.142857', '-1.915', '-0.210397510215']
SeSmZn 2 ['Zn0.285714Se0.428571Sm0.285714', '-1.557', '-0.201281105714']
SeSmZr 4 ['Se0.625Zr0.25Sm0.125', '-1.7', '-0.214394588733']
SeSnTc 9 ['Se0.5Tc0.25Sn0.25', '-0.486', '-0.204908473287']
SeSnTm 1 ['Se0.5Sn0.1Tm0.4', '-1.678', '-0.230434716001']
SeSnV 1 ['V0.2Se0.4Sn0.4', '-0.697', '-0.231939027753']
SeSnY 11 ['Se0.428571Y0.285714Sn0.285714', '-1.352', '-0.20503984545']
SeSnZr 7 ['Se0.5Zr0.333333Sn0.166667', '-1.308', '-0.212613016127']
SeSrTc 4 ['Se0.625Sr0.125Tc0.25', '-0.763', '-0.24485372625']
SeSrTm 2 ['Se0.571429Sr0.142857Tm0.285714', '-1.949', '-0.235381718573']
SeSrY 9 ['Se0.666667Sr0.111111Y0.222222', '-1.461', '-0.215580544444']
SeSrZr 2 ['Se0.555556Sr0.111111Zr0.333333', '-1.639', '-0.229295218827']
SeTaTc 3 ['Se0.666667Tc0.222222Ta0.111111', '-0.514', '-0.220568070278']
SeTaTe 8 ['Se0.666667Te0.111111Ta0.222222', '-0.789', '-0.202136140556']
SeTaTh 2 ['Se0.666667Ta0.222222Th0.111111', '-1.33', '-0.205169264443']
SeTaTm 4 ['Se0.6Tm0.2Ta0.2', '-1.38', '-0.218859649669']
SeTaY 9 ['Se0.571429Y0.285714Ta0.142857', '-1.58', '-0.220535162857']
SeTbTc 3 ['Se0.571429Tc0.285714Tb0.142857', '-0.806', '-0.228038102546']

SeTbTe 10 ['Se0.2Te0.4Tb0.4', '-1.391', '-0.208655363799']
SeTbTl 4 ['Se0.4Tb0.4Tl0.2', '-1.357', '-0.200894230136']
SeTbY 6 ['Se0.666667Y0.222222Tb0.111111', '-1.448', '-0.213629089758']
SeTbZn 6 ['Zn0.125Se0.5Tb0.375', '-1.595', '-0.206756305712']
SeTbZr 1 ['Se0.555556Zr0.333333Tb0.111111', '-1.508', '-0.205680115237']
SeTcTe 42 ['Se0.6Tc0.1Te0.3', '-0.218', '-0.218']
SeTcTi 4 ['Ti0.111111Se0.555556Tc0.333333', '-0.588', '-0.21471630069']
SeTcTl 5 ['Se0.666667Tc0.222222Tl0.111111', '-0.263', '-0.201811285556']
SeTcTm 2 ['Se0.571429Tc0.285714Tm0.142857', '-0.852', '-0.203798535716']
SeTcU 3 ['Se0.666667Tc0.222222U0.111111', '-0.625', '-0.213187965']
SeTcV 6 ['V0.2Se0.6Tc0.2', '-0.677', '-0.2538768848']
SeTcW 3 ['Se0.666667Tc0.222222W0.111111', '-0.394', '-0.208989138333']
SeTcXe 23 ['Se0.5Tc0.4Xe0.1', '-0.211', '-0.211']
SeTcY 22 ['Se0.5Y0.125Tc0.375', '-0.645', '-0.203524693125']
SeTcYb 4 ['Se0.625Tc0.25Yb0.125', '-0.787', '-0.26046015625']
SeTcZn 9 ['Zn0.2Se0.6Tc0.2', '-0.51', '-0.222522118']
SeTcZr 4 ['Se0.5Zr0.4Tc0.1', '-1.343', '-0.241679385819']
SeTeTh 15 ['Se0.5Te0.2Th0.3', '-1.673', '-0.201470045855']
SeTeTi 1 ['Ti0.3Se0.4Te0.3', '-1.122', '-0.237590503197']
SeTeTm 8 ['Se0.428571Te0.142857Tm0.428571', '-1.703', '-0.202271238454']
SeTeU 16 ['Se0.555556Te0.111111U0.333333', '-1.261', '-0.204361488241']
SeTeY 52 ['Se0.333333Y0.166667Te0.5', '-0.789', '-0.2003662575']
SeTeZr 24 ['Se0.571429Zr0.285714Te0.142857', '-1.348', '-0.204163668571']
SeThTl 1 ['Se0.5Tl0.3Th0.2', '-1.362', '-0.230047187998']
SeThTm 1 ['Se0.6Tm0.3Th0.1', '-1.84', '-0.201891597']
SeThY 5 ['Se0.6Y0.1Th0.3', '-1.93', '-0.223352963498']
SeThZn 13 ['Zn0.3Se0.5Th0.2', '-1.395', '-0.207625314998']
SeThZr 5 ['Se0.571429Zr0.142857Th0.285714', '-1.742', '-0.202175857795']
SeTiY 5 ['Ti0.2Se0.6Y0.2', '-1.622', '-0.243728850242']
SeTlTm 11 ['Se0.444444Tm0.444444Tl0.111111', '-1.68', '-0.207667764444']
SeTlY 36 ['Se0.375Y0.5Tl0.125', '-1.623', '-0.204797385625']
SeTlZr 11 ['Se0.375Zr0.375Tl0.25', '-1.074', '-0.222985184896']
SeTmU 1 ['Se0.6Tm0.3U0.1', '-1.689', '-0.216946264251']
SeTmV 3 ['V0.1Se0.6Tm0.3', '-1.571', '-0.204875879668']
SeTmY 13 ['Se0.555556Y0.333333Tm0.111111', '-1.931', '-0.249575820557']
SeTmZn 22 ['Zn0.142857Se0.571429Tm0.285714', '-1.528', '-0.201207544287']
SeTmZr 7 ['Se0.5Zr0.166667Tm0.333333', '-1.686', '-0.20352201662']
SeUY 4 ['Se0.666667Y0.166667U0.166667', '-1.442', '-0.235648205']
SeVY 4 ['V0.111111Se0.555556Y0.333333', '-1.653', '-0.248700266111']
SeVZn 5 ['V0.375Zn0.125Se0.5', '-0.857', '-0.20429361375']
SeVZr 3 ['V0.166667Se0.5Zr0.333333', '-1.261', '-0.215533333287']
SeWY 1 ['Se0.6Y0.3W0.1', '-1.469', '-0.242949488']
SeWZr 2 ['Se0.428571Zr0.428571W0.142857', '-1.251', '-0.278411639881']
SeXeY 18 ['Se0.5Y0.1Xe0.4', '-0.576', '-0.2228197545']

SeXeZr 2 ['Se0.5Zr0.4Xe0.1', '-1.287', '-0.205845431917']
SeYYb 4 ['Se0.555556Y0.333333Yb0.111111', '-1.903', '-0.257697098333']
SeYZn 11 ['Zn0.222222Se0.444444Y0.333333', '-1.545', '-0.20554852235']
SeYZr 8 ['Se0.5Y0.125Zr0.375', '-1.496', '-0.203509878021']
SeYbZn 1 ['Zn0.1Se0.4Yb0.5', '-2.009', '-0.256425432']
SeZnZr 7 ['Zn0.142857Se0.428571Zr0.428571', '-1.238', '-0.237197070622']
SiTaTc 2 ['Si0.333333Tc0.444444Ta0.222222', '-0.684', '-0.210032468472']
SiTbTc 3 ['Si0.5Tc0.375Tb0.125', '-0.681', '-0.231285815667']
SiTcTi 5 ['Si0.333333Ti0.222222Tc0.444444', '-0.785', '-0.220072948881']
SiTcTm 1 ['Si0.428571Tc0.428571Tm0.142857', '-0.718', '-0.237432137143']
SiTcU 4 ['Si0.5Tc0.4U0.1', '-0.69', '-0.216970949875']
SiTcV 7 ['Si0.333333V0.166667Tc0.5', '-0.691', '-0.206030519445']
SiTcY 2 ['Si0.444444Y0.111111Tc0.444444', '-0.711', '-0.233159632778']
TbTeY 1 ['Y0.1Te0.6Tb0.3', '-1.235', '-0.204893917099']
TeThU 1 ['Te0.7Th0.1U0.2', '-0.999', '-0.252465115239']
TeUY 2 ['Y0.3Te0.6U0.1', '-1.308', '-0.234395778']
